# Supplementary material for: Transcriptional dissection of symptomatic profiles across the brain of men and women with depression
Source: Nat Commun. 2023 Oct 26;14:6835. doi: 10.1038/s41467-023-42686-5 (PMC10603117; doi:10.1038/s41467-023-42686-5)
Supplement: Supplementary file 3 — Reporting Summary [file 41467_2023_42686_MOESM3_ESM.pdf]

nature portfolio

Corresponding author(s): Benoit Labonté

Last updated by author(s): Sep 5, 2023

Reporting Summary

Nature Portfolio wishes to improve the reproducibility of the work that we publish. This form provides structure for consistency and transparency in reporting. For further information on Nature Portfolio policies, see our Editorial Policies and the Editorial Policy Checklist.

Statistics

For all statistical analyses, confirm that the following items are present in the figure legend, table legend, main text, or Methods section.

n/a	Confirmed	
☐	☒	The exact sample size (*n*) for each experimental group/condition, given as a discrete number and unit of measurement
☐	☒	A statement on whether measurements were taken from distinct samples or whether the same sample was measured repeatedly
☐	☒	The statistical test(s) used AND whether they are one- or two-sided<br>*Only common tests should be described solely by name; describe more complex techniques in the Methods section.*
☐	☒	A description of all covariates tested
☐	☒	A description of any assumptions or corrections, such as tests of normality and adjustment for multiple comparisons
☐	☒	A full description of the statistical parameters including central tendency (e.g. means) or other basic estimates (e.g. regression coefficient) AND variation (e.g. standard deviation) or associated estimates of uncertainty (e.g. confidence intervals)
☐	☒	For null hypothesis testing, the test statistic (e.g. *F*, *t*, *r*) with confidence intervals, effect sizes, degrees of freedom and *P* value noted<br>*Give P values as exact values whenever suitable.*
☒	☐	For Bayesian analysis, information on the choice of priors and Markov chain Monte Carlo settings
☒	☐	For hierarchical and complex designs, identification of the appropriate level for tests and full reporting of outcomes
☐	☒	Estimates of effect sizes (e.g. Cohen's *d*, Pearson's *r*), indicating how they were calculated

Our web collection on statistics for biologists contains articles on many of the points above.

Software and code

Policy information about availability of computer code

Data collection

RNA from human postmortem brain samples was extracted using the RNeasy micro kit with Trizol, followed by DNase I treatment, as described by the manufacturer (Qiagen). RNA integrity (RIN) and concentration was quantified using a Bioanalyzer (Agilent). RNA libraries were synthesized using the ScriptSeq Complete Gold Kit (Epicentre, Illumina). Samples were barcoded and sequenced in multiplex on Illumina HiSeq2500.

Data analysis

Sequencing quality and trim reads were assessed using FASTQ and FASTX-toolkit. TopHat was used to align paired-end reads to the GENCODE 2019 (GRCh38.p12) human annotation. Reads for every sample were counted using HTSeq. Software R 3.6.0 was used to carry out all the analyzes that were necessary for this manuscript. We used package Limma and edgeR for normalization and Differential expression analysis. Package RUVSeq was used to identify the batch effect and unwanted variations among samples. We used a rank-rank hypergeometric overlap (RRHO) analysis using an R package named RRHO2 to measure transcriptional overlap. Gene ontology analysis was performed using package g:Profiler2 from Bioconductor. Network organization was represented through Cytoscape v3.9.1. Modular enrichment for DEGs was assessed using the GeneOverlap package from Bioconductor.

For manuscripts utilizing custom algorithms or software that are central to the research but not yet described in published literature, software must be made available to editors and reviewers. We strongly encourage code deposition in a community repository (e.g. GitHub). See the Nature Portfolio guidelines for submitting code & software for further information.

Data

Policy information about availability of data

All manuscripts must include a data availability statement. This statement should provide the following information, where applicable:

- Accession codes, unique identifiers, or web links for publicly available datasets
- A description of any restrictions on data availability
- For clinical datasets or third party data, please ensure that the statement adheres to our policy

Brain tissues were obtained from the Douglas Bell Canada Brain Bank (Douglas Mental Health Institute, Verdun, Québec and from the University of Texas Southwestern Medical Center Brain Bank. Postmortem tissue from all six brain regions was carefully dissected at 4ºC after having been flash-frozen in isopentane at -80°C. All dissections were performed by histopathologists using reference neuroanatomical maps. Psychiatric history and socio-demographic information was obtained via psychological autopsies carried out by trained clinicians using the same methods in case and control groups. Diagnosis and clinical information including symptomatic profiles were obtained using DSM-IV criteria by means of SCID-I interview adapted for psychological autopsies. Sequencing data from all 89 samples for all 6 brain regions were analyzed using the same criteria. Overall, every sample included in this study passed QC assessment. A gene was considered the union of all its exons in any known isoforms, based on GENCODE annotation. Any reads that fell in multiple genes were excluded from the analysis. Threshold for filtering out genes expressed at low levels was set to <5 reads in at least 20% of the samples per group.
Data Availability: Sequencing data and source files have been used in this study to generate all the results, including figures, and tables is composed of two cohorts. The first cohort is available on NCBI GEO website (accession codes GSE102556). The second cohort will be made available with no restriction on NCBI GEO website upon publication. Any additional data supporting the findings of this study are available from the corresponding author upon reasonable request. Source data are provided with this paper.

Research involving human participants, their data, or biological material

Policy information about studies with human participants or human data. See also policy information about sex, gender (identity/presentation), and sexual orientation and race, ethnicity and racism.

Reporting on sex and gender	This is a sex-specific study on depression to find the transcriptional dissection of symptomatic profiles across the brain of men and women with depression. Sex was determined based on their autopsy results. Post-mortem brain tissues were obtained from the Douglas Bell Canada Brain Bank (Douglas Mental Health Institute, Verdun, Québec and from the University of Texas Southwestern Medical Center Brain Bank. Analyses were performed on 89 samples including 25 male MDD, 25 female MDD, 17 male CTRL (healthy controls) and 22 female CTRL. Sociodemographic and clinical information including sex, phenotype (MDD, CTRL), age, pH, postmortem interval (PMI), treatment history, smoking history, history of early life adversity, cause of death, presence of drug and/or alcohol abuse and cohort (Montreal, Texas) is listed in Suppl. Table 1. In all analyzes and results the effect of sex on depression has been investigated to identify sex- and brain region-specific transcriptional organizations of genes.
Reporting on race, ethnicity, or other socially relevant groupings	Psychiatric history and socio-demographic information was obtained via psychological autopsies carried out by trained clinicians using the same methods in case and control groups. Diagnosis and clinical information including symptomatic profiles were obtained using DSM-IV criteria by means of SCID-I interview adapted for psychological autopsies. Nine main categories of symptoms were recorded, including depressed mood, loss of interest or pleasure, change in appetite/weight, insomnia/ hypersomnia, psychomotor agitation/retardation, fatigue or loss of energy, low self-esteem, difficulty in concentration/indecision and recurrent suicidal thoughts. Notably, since depressed mood, anhedonia, fatigue and recurrent suicidal thoughts were expressed by every MDD patient, we did not include those symptoms in our analysis (Suppl. Table 2). We adapted multiple preprocessing steps to ensure both statistical and biological relevance. Gene expression was first transformed and normalized using the voom function in the Limma package. Batch effect and potential unwanted sources of variance in gene expression across all samples was identified through RUVseq using spike-in controls. This method is designed to identify any sources of unwanted variation, including the probable heterogeneity between two merged datasets. As expected, the effect of batch (new and previous cohort) was found to be significant for every brain region. The top first factor was extracted and included as a covariate in the differential expression analysis. We then performed a principal component analysis (PCA) to reveal the effect of clinical and technical covariates on variations of gene expression. We identified significant effects for PMI, pH, cohort, drug abuse and RIN in the aINS; age, PMI, pH, childhood abuse, cohort, drug abuse and RIN in the OFC; age, pH, cohort, drug abuse and RIN in the vmPFC; age, PMI, pH, cohort, drug abuse and RIN in the dlPFC; age, PMI, childhood abuse, cohort, drug abuse and smoking in the NAc; and PMI, cohort, drug abuse and RIN in the vSub (Suppl. Table 3). The effects of these covariates were adjusted in our downstream differential expression and gene co-expression network analyses.
Population characteristics	Sociodemographic and clinical information including sex, phenotype (MDD, CTRL), age, pH, postmortem interval (PMI), treatment history, smoking history, history of early life adversity, cause of death, presence of drug and/or alcohol abuse and cohort (Montreal, Texas) reported in Supp Table 1 and their effects have been assessed and adjusted (if needed) in every step of analysis.
Recruitment	Postmortem brain tissues were obtained from the Douglas Bell Canada Brain Bank (Douglas Mental Health Institute, Verdun, Québec and from the University of Texas Southwestern Medical Center Brain Bank. To perform this study we tried to collect all samples, without considering their identity. Therefore the potential self-selection bias or other biases are not applicable here.
Ethics oversight	Any qualified researcher from the public sector can get tissue, under the condition that their research protocol is approved by their institution's Research Ethics Board. As for private sector research centers, the Douglas Institute Research Ethics Board must specifically approve requests before any tissue is sent.<br>For all requests for samples, researchers are asked to provide:<br>• A duly completed Human Brain Tissue Request Form

- A duly completed copy of the Statement Form
- A general summary of the research project
- A copy of the research protocol duly approved by the Research Ethics Board (REB) of their institution

Note that full information on the approval of the study protocol must also be provided in the manuscript.

Field-specific reporting

Please select the one below that is the best fit for your research. If you are not sure, read the appropriate sections before making your selection.

☒ Life sciences ☐ Behavioural & social sciences ☐ Ecological, evolutionary & environmental sciences

For a reference copy of the document with all sections, see nature.com/documents/nr-reporting-summary-flat.pdf

Life sciences study design

All studies must disclose on these points even when the disclosure is negative.

Sample size	Although sample size calculation was not performed, the sample size in this study is justified based on several previously published reports using similar or even smaller sample sizes and showing the power to detect significant statistical differences. In total, 89 samples including 25 male MDD, 25 female MDD, 17 male CTRL and 22 female CTRL, from six brain regions (total 534 samples) were included in this study (25,29,31).
Data exclusions	We removed samples where their RNA quality was not good enough. The outliers were also removed from the final dataset. Samples with count values larger than 99th percentile of the data considered outliers and removed from analysis. Using Euclidean distance based sample network, we calculated a Z value and removed samples with their Z value less than or equal to -3.5.
Replication	To ensure the reproducibility of our findings, we have implemented a range of strategic measures. Initially, we identified covariates that exhibited significant associations with our outcome variables. These associations were then effectively addressed using GLM statistical models to adjust their effects. Notably, with the exception of DEGs analysis, we've presented adjusted P-values for all our statistical tests. This practice not only mitigates Type I errors but also contributes to generating outcomes that are both valid and readily reproducible.<br>Within the context of our network analysis, we've incorporated a metric known as "module preservation." This approach enables us to gauge the consistent performance of the constructed modules across varying conditions. For module selection, we employed adjusted P-values to pinpoint the modules with the most profound significance. This process was undertaken to minimize potential sources of error.<br>Furthermore, to validate the reproducibility of our results, we conducted a comparative analysis with findings from our previous study using the RRHO method (Figure. 1d). It revealed highly significant overlaps between genes that exhibited differential expression in this current study and those identified in our prior study (31). This additional layer of validation provides robust support for the credibility and significance of our findings.<br>Moreover, the implementation of the RRHO method has yielded compelling results. It has revealed notably substantial overlaps between genes that exhibited differential expression in the current study and those identified in our previous study (Figure. 1d). This additional layer of validation further reinforces the robustness and significance of our findings.
Randomization	According to study design our study is considered as an observational explanatory study. So we have not had any randomization. We used post-mortem brain tissues to identify how the transcriptional organizations of genes are similar or different in males and females with and without MDD. Therefore we adjusted for the probable confounding effects of the related covariates in our statistical models.
Blinding	Blinding was not applicable in our study, due to the nature and type of the study.

Reporting for specific materials, systems and methods

We require information from authors about some types of materials, experimental systems and methods used in many studies. Here, indicate whether each material, system or method listed is relevant to your study. If you are not sure if a list item applies to your research, read the appropriate section before selecting a response.

Materials & experimental systems

n/a	Involved in the study	
☒	☐	Antibodies
☒	☐	Eukaryotic cell lines
☒	☐	Palaeontology and archaeology
☒	☐	Animals and other organisms
☒	☐	Clinical data
☒	☐	Dual use research of concern
☒	☐	Plants

Methods

n/a	Involved in the study	
☒	☐	ChIP-seq
☒	☐	Flow cytometry
☒	☐	MRI-based neuroimaging
