# Supplementary material for: Transcriptional dissection of symptomatic profiles across the brain of men and women with depression
Source: Nat Commun. 2023 Oct 26;14:6835. doi: 10.1038/s41467-023-42686-5 (PMC10603117; doi:10.1038/s41467-023-42686-5)
Supplement: Supplementary file 1 — Supplementary Information [file 41467_2023_42686_MOESM1_ESM.pdf]

Supplemental Figure 1

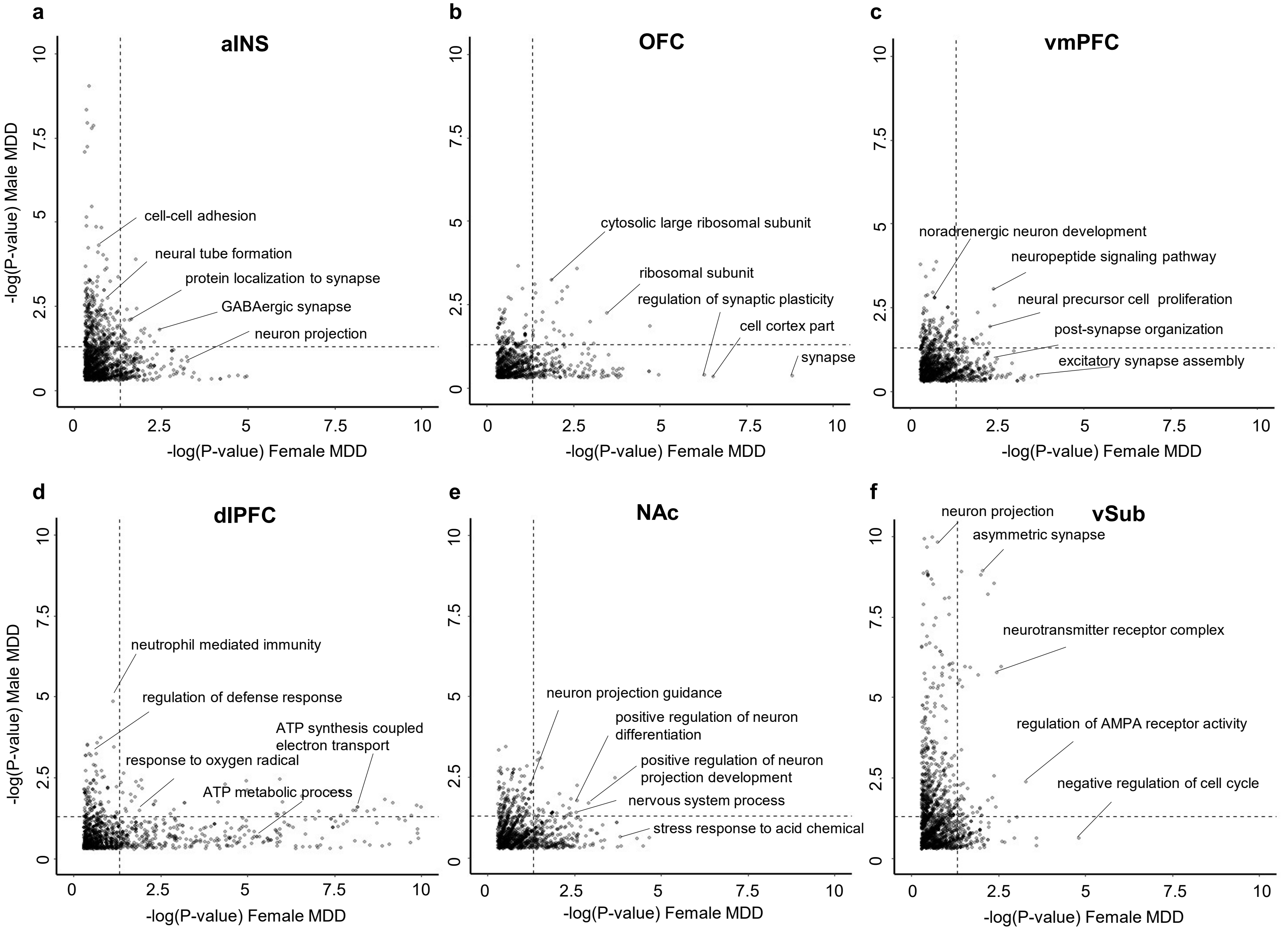

Supplemental Figure 1. Common functional terms enriched in DEGs in males and females. (a-f) Scatter plots showing the ontological terms (commonly/differently) enriched for DEGs in males and females with MDD across brain regions. Plots are shown within the same range to be comparable.

Supplemental Fig 2

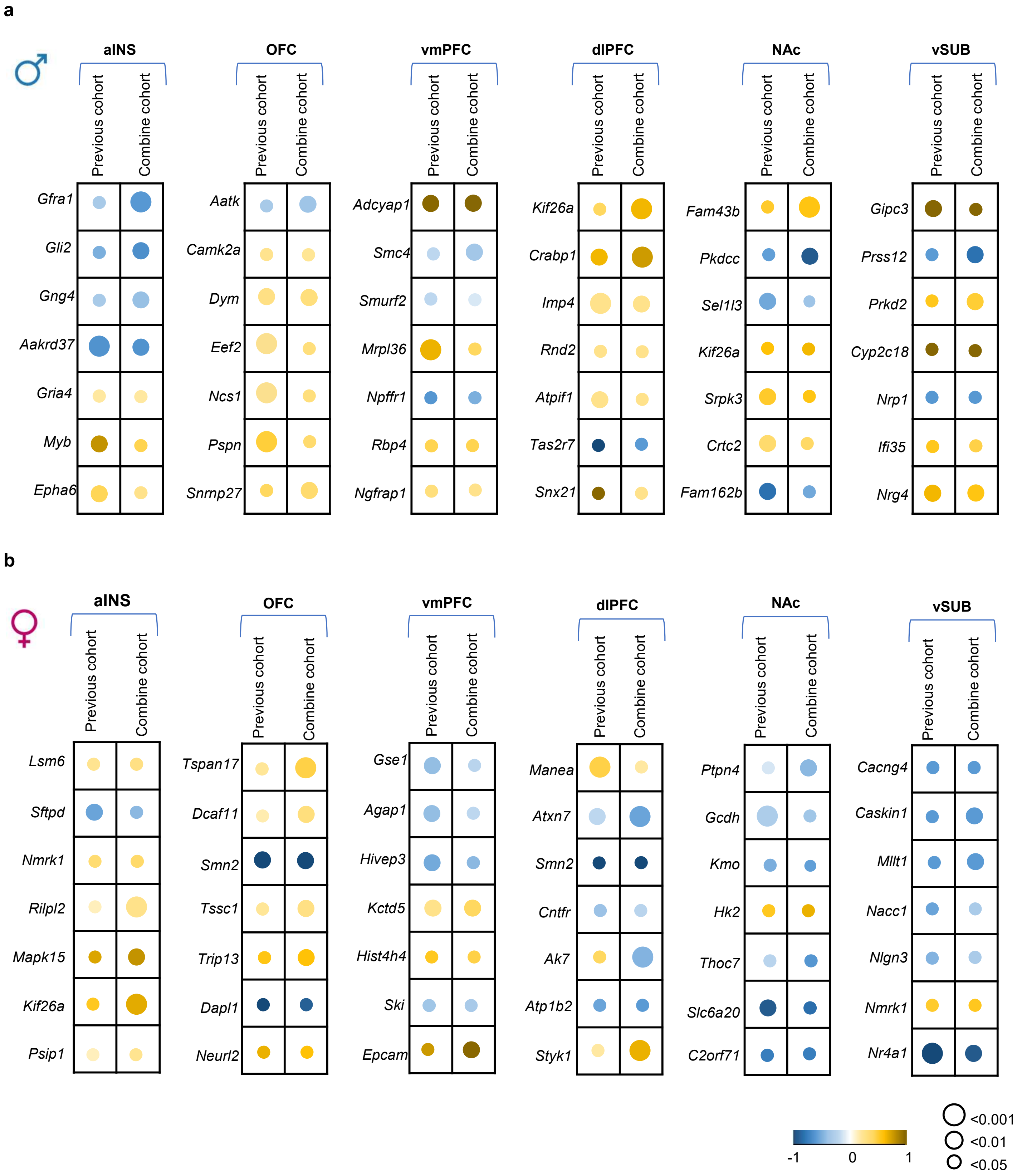

Supplemental Figure 2. Comparison of DEGs from distinct postmortem cohorts shows consistent transcriptional changes in males and females across brain regions. Representative list of genes differentially expressed in this study and our previously published study[27] in males (a) and females (b) across six brain regions. Colors represent log fold change values, with blue for genes downregulated and yellow for genes upregulated in MDD versus control conditions. The size of the circles corresponds to the level of significance.

Supplemental Figure 3

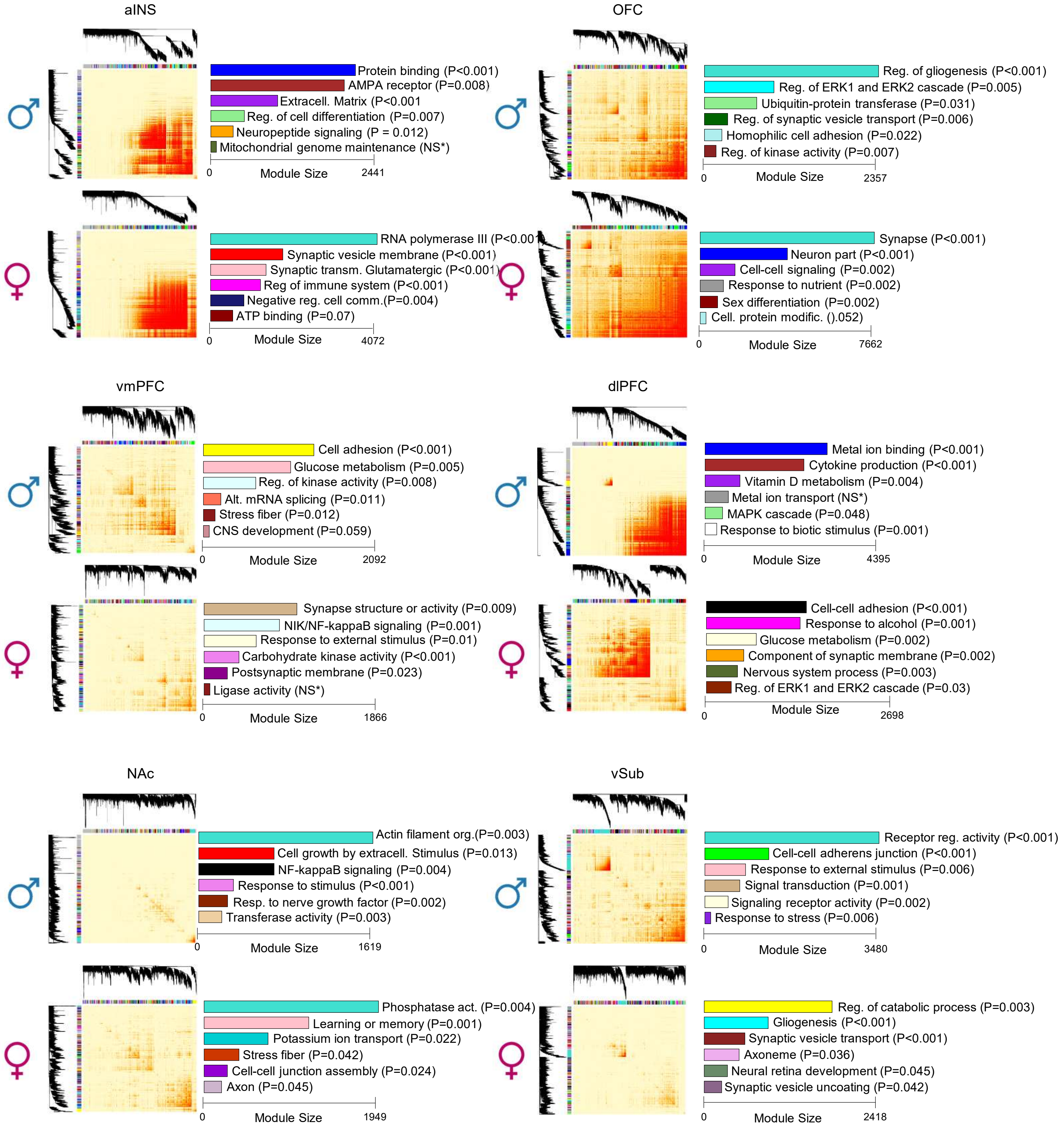

Supplemental Figure 3. Topological overlap matrix (TOM) plots for MDD modules in males and females across six brain regions. The color bars at the right side of each plot show module color and size with their enriched GO functions. The scale at the bottom right of each plot represents the module size. GO terms' p-values are shown in parentheses.

*NS (Not Significant GO term)

Supplemental Figure 4

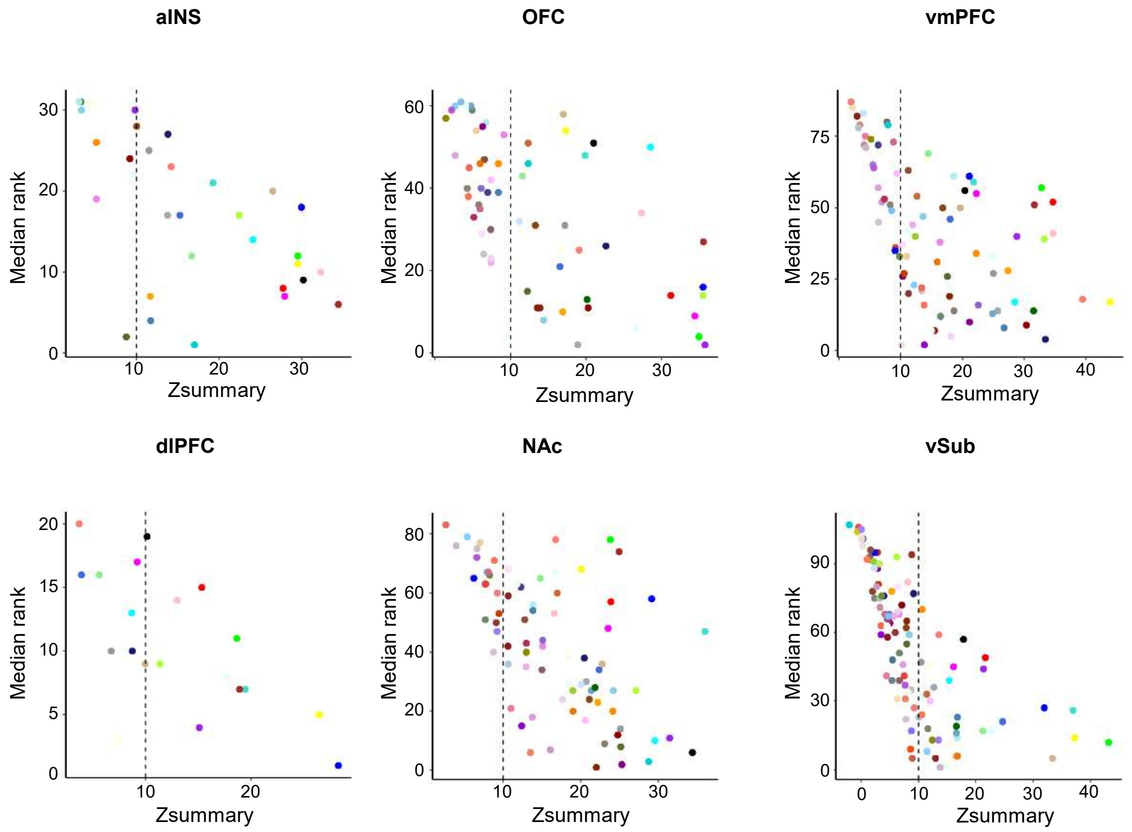

Supplemental Figure 4. Preservation of the transcriptional organization of gene networks between male MDD and female MDD. Modules with $Z_{summary}$ scores higher than 10 are considered as male MDD modules preserved in female MDD. Each individual dot corresponds to a distinct module, and its representation is underscored by the utilization of a consistent color. This color serves as an identifying marker for each module within the context of network analysis.

Supplemental Figure 5

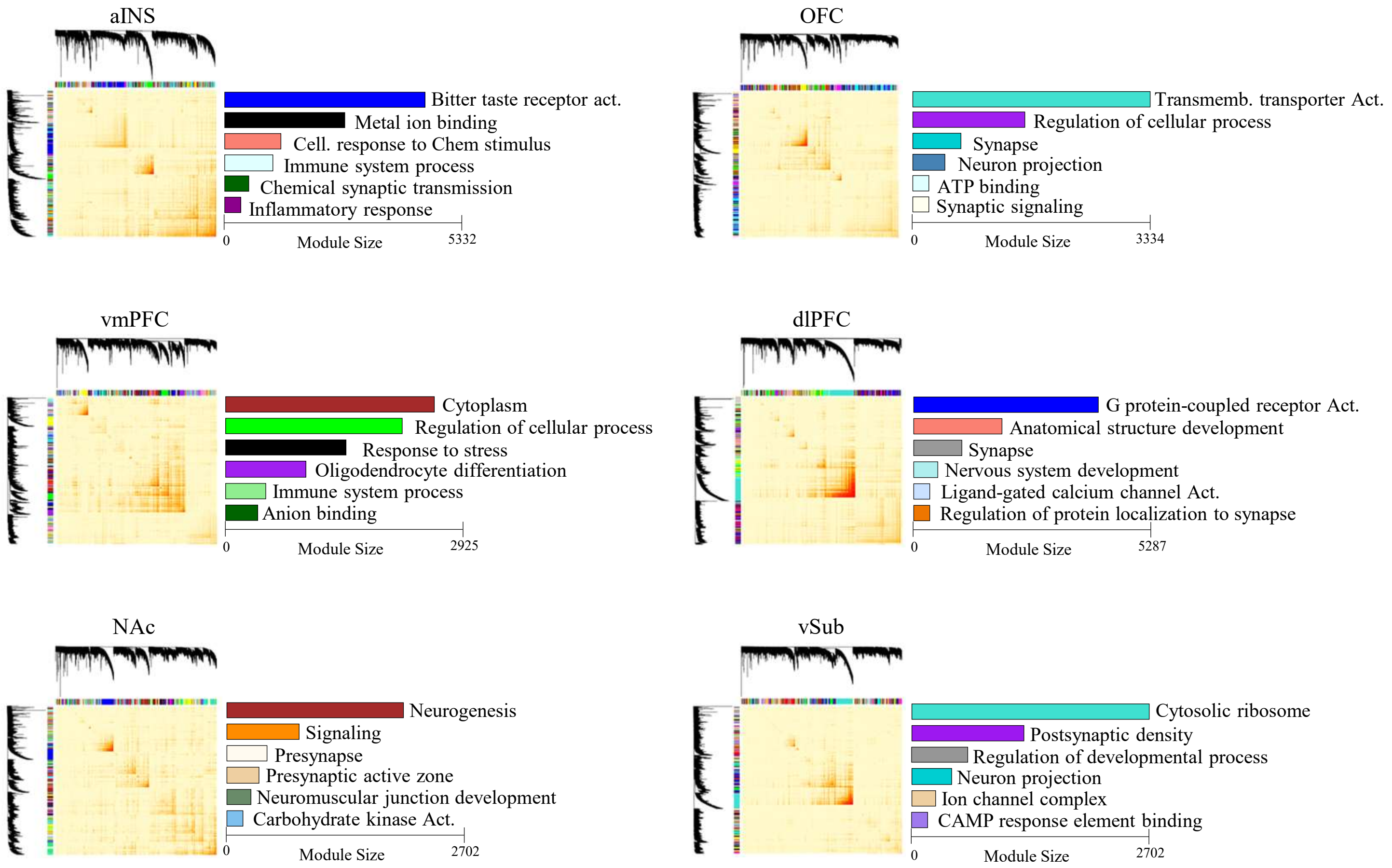

Supplemental Figure 5. Topological overlap matrix (TOM) plots for modules combining males and females across six brain regions. The color bars are employed to show randomly selected modules, each aligned with the same color that serves as its identifier in the network analysis. The length of each bar is indicative of the module's size (denoting the number of genes it encompasses), annotated with their enriched ontological terms.

Supplementary Figure 6

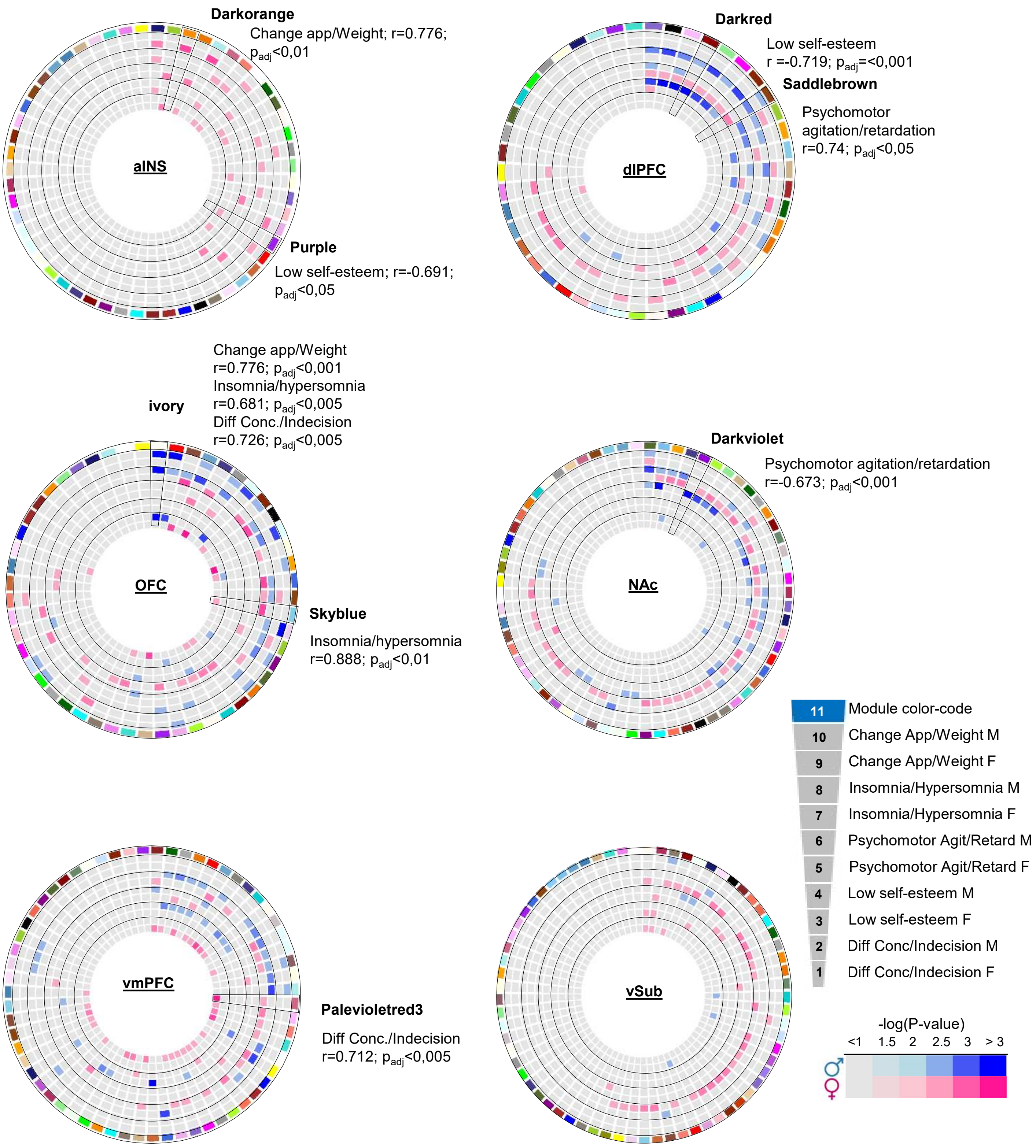

Supplementary Figure 6. Circos plots displaying the degree of associations between modules and symptomatic features of MDD across six brain regions. These associations are differentiated by color, with males represented in blue and females in pink. The intensity of these connections is visualized through varying levels of shading, reflecting the potency of the links between module eigengenes and the depression-related symptomatic features. To quantitatively represent this strength, the scale employs the negative logarithm of the P-value.. The order of the symptoms associations is, from top to bottom, change in appetite/weight, insomnia/hypersomnia, psychomotor agitation/retardation, low self-esteem and difficulty in concentration/indecision in males and females, respectively.

Supplementary Figure 7

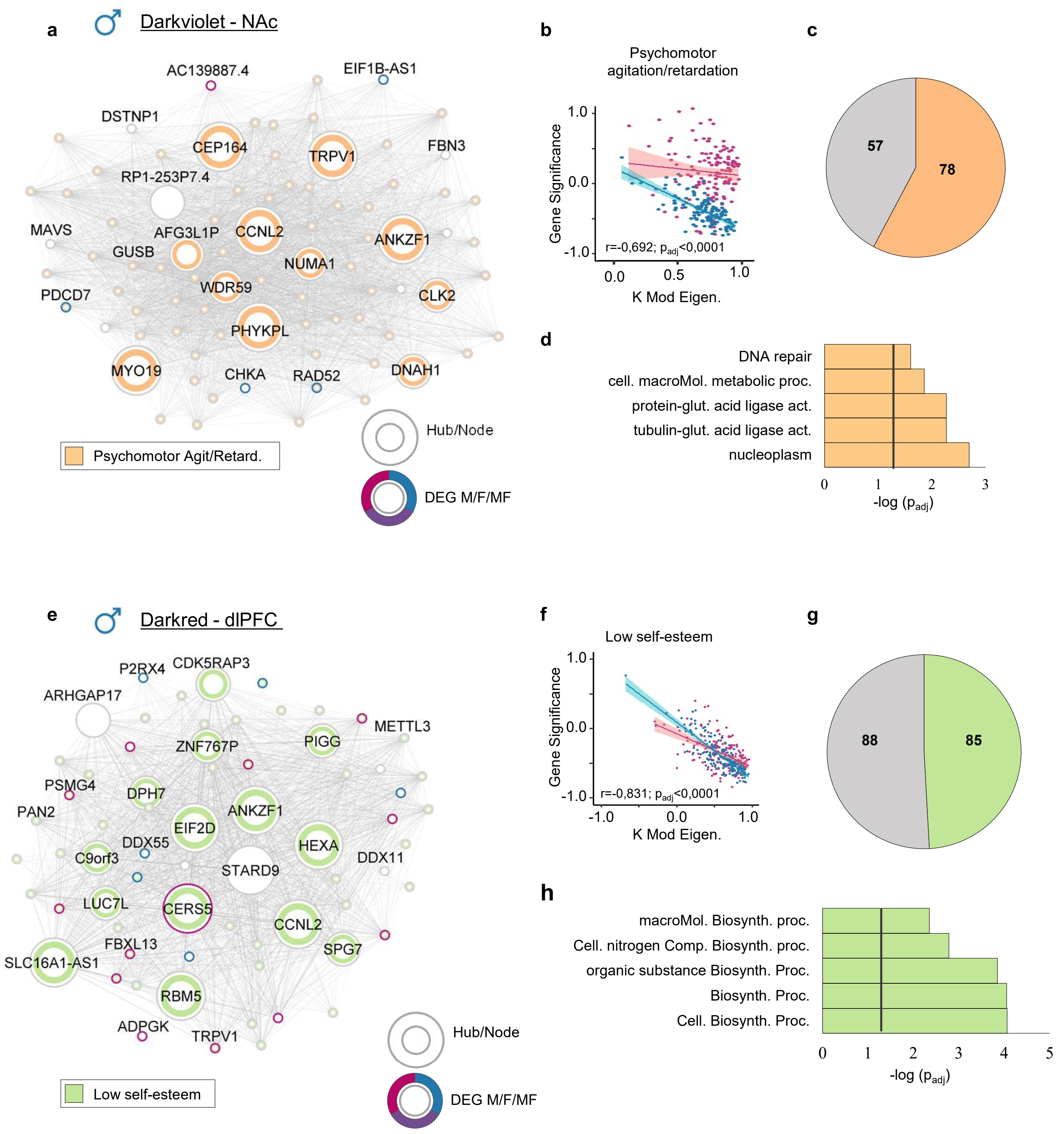

Supplementary Figure 7. Representative modules (Darkviolet and Darkred) associated with the expression of psychomotor agitation/retardation and low self-esteem in male MDD identified in the NAc and dlPFC, respectively. (a,e) Hubs and nodes representation of (a) module Ivory and (e) module Darkred and their associations with respective clinical symptoms. (a) The inner color in orange shows the genes significantly associated with the expression of the symptom: psychomotor agitation/retardation. (e) The inner color in bright green shows the genes significantly associated with the expression of low self-esteem. The surrounding circle shows if the gene is differentially expressed in males (blue), females (pink) or both (violet). Hubs and nodes are defined by the size of the circles. (b,f) Correlation between module membership (KME) and gene significance values of the association between each gene and relevant symptomatic feature for genes co-expressed in (b) module Darkviolet and (f) module Darkred, in males (blue) and females (pink) with the 95% confidence interval bands. (c,g) Pie charts showing the number of genes in (c) module Darkviolet associated with psychomotor agitation/retardation in orange and (g) module Darkred associated with low self-esteem in bright green. (d,h) GO enrichment for (d) module Darkviolet and (h) module Darkred for the first five most significantly enriched GO terms. The black vertical line indicates the significance threshold (p_{adj}<0,05).

Supplementary Fig. 8

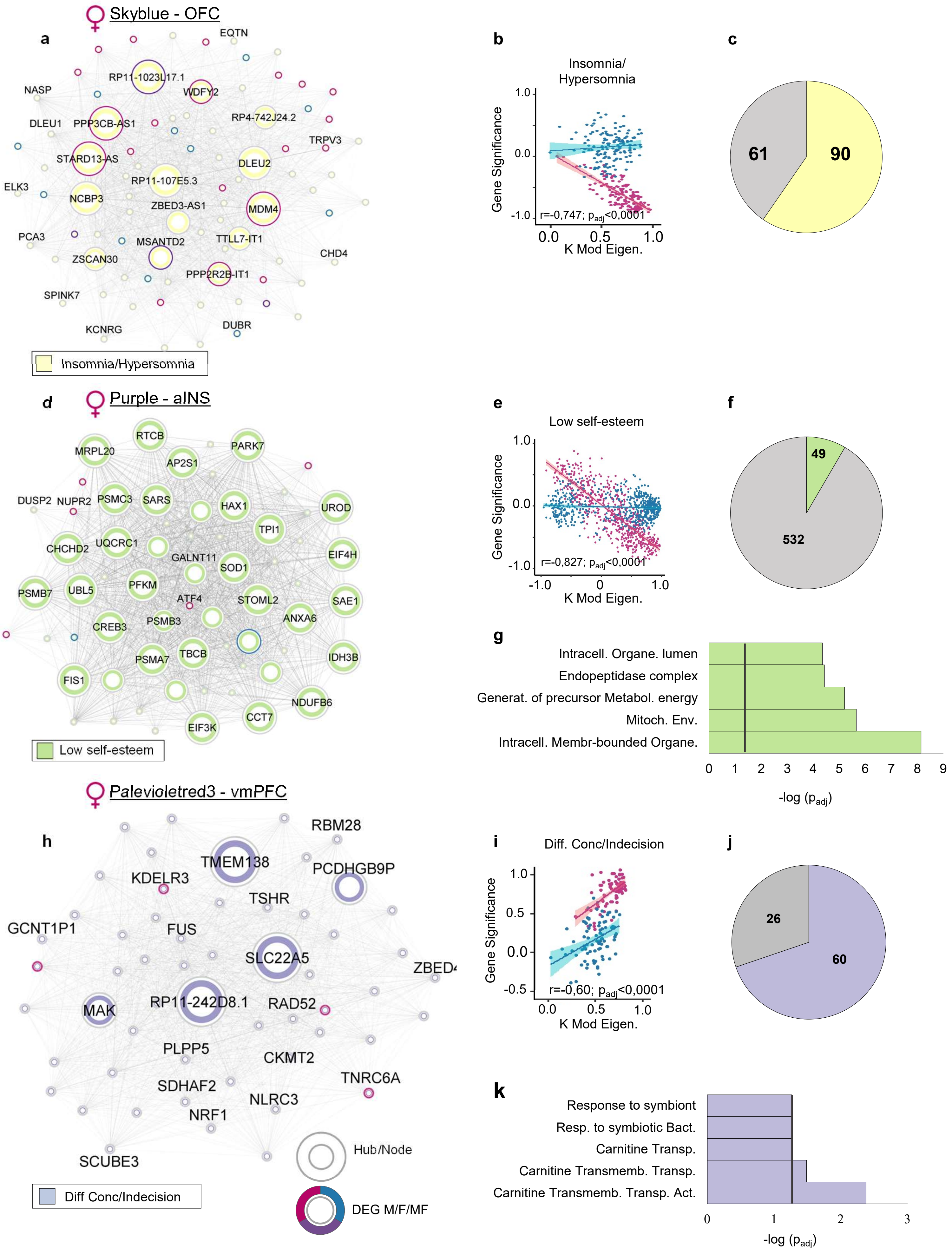

Supplementary Fig. 8. Representative modules (Skyblue, Purple, and Palevioletred3) associated with the expression of insomnia/hypersomnia, low self-esteem and difficulty in concentration/indecision in females detected in the OFC, aINS and vmPFC, respectively. (a,d,h) Hubs and nodes representation of (a) module Skyblue, (e) module Purple, (h) module Palevioletred3 and their associations with respective clinical symptoms of MDD. The inner color shows the genes significantly associated with the expression of insomnia/hypersomnia (a, yellow), low self-esteem (e, green) and difficulty in concentration/indecision (h, purple). The surrounding circle shows if the gene is differentially expressed in males (blue), females (pink) or both (violet). Hubs and nodes are defined by the size of the circles. (b,e,i) Correlation between module membership (KME) and gene significance values of the association between each gene and respective symptomatic feature for genes co-expressed in (b) module Skyblue, (e) module Purple and (i) module Palevioletred3 in males (blue) and females (pink) with the 95% confidence interval bands. (c,f,j) Pie charts showing the number of genes in (c) module Skyblue associated with insomnia/hypersomnia showing in yellow color, (f) module Purple associated with low self-esteem showing in bright green color and (j) module Palevioletred3 associated with difficulty in concentration/indecision showing in purple color. (g,k) GO enrichment for (g) module Purple and (k) module Palevioletred3 for the first five most significantly enriched GO terms. The black vertical line indicates the significance threshold (p_{adj} <0,05).

Supplementary Table 1. Postmortem samples demographics information

	Women CTRL	Women MDD	Men CTRL	Men MDD
Age	65.5±11.26	47.12±14.69	48.47±14.24	46.08±12.75
PMI	34.53±31.08	36.38±29.57	22.97±20.64	39.62±22.55
pH	6.38±0.41	6.64±0.3	6.49±0.33	6.65±0.3
RIN aINS	7.26±1.66	6.82±1.14	7.44±1.22	6.9±1.66
RIN OFC	7.46±1.52	7.26±0.84	7.5±1.09	7.18±1.45
RIN vmPFC	7.07±1.22	6.56±1.3	7.39±1.21	6.55±1.27
RIN dlPFC	7.31±1.14	7.1±0.84	7.59±1.21	7.25±1.19
RIN NAc	7.77±1.42	7.69±0.8	7.49±1.5	7.88±1.33
RIN vSub	6.27±1.45	6.88±1.04	6.88±1.79	7.33±1.51
Childhood abuse (NA/No/Yes)	4/15/3	7/5/13	2/9/6	6/9/10
Alcohol abuse (NA/No/Yes)	4/17/1	9/13/3	2/14/1	2/14/9
Drug abuse (NA/No/Yes)	3/16/3	9/11/5	2/13/2	2/20/3
Medication (NA/No/Yes)	4/15/3	4/4/17	1/15/1	5/9/11
Smoking (NA/No/Heavy/light/moderate)	0/11/7/0/4	3/7/11/1/3	2/5/9/0/1	5/5/13/2
Cohort (Montreal/Texas)	10/12	19/6	9/8	21/4
Cause of dead Natural/Accident/Suicide	18/4/0	2/1/22	11/6/0	3/0/22

Supplementary Table 2. Frequency of Clinical symptoms in men and women with MDD across brain region

Sex	Region	Response	Change in appetite/weight	Insomnia/ Hypersomnia	Psychomotor agitation/retardation	Low self-esteem	Difficulty in concentration/ indecision
Male MDD	aINS	No	7	5	8	7	9
		Yes	11	14	9	11	8
	OFC	No	7	5	8	7	9
		Yes	11	14	9	11	8
	vmPFC	No	7	5	7	6	8
		Yes	9	12	8	10	7
	dlPFC	No	7	5	8	7	9
		Yes	11	14	9	11	8
	NAc	No	7	5	8	7	9
		Yes	11	14	9	11	8
	vSub	No	4	3	6	5	7
		Yes	9	11	7	8	5
Female MDD	aINS	No	3	3	3	3	4
		Yes	9	7	7	6	7
	OFC	No	3	3	3	3	4
		Yes	9	7	7	6	7
	vmPFC	No	3	3	3	3	4
		Yes	9	7	7	6	7
	dlPFC	No	3	3	3	3	4
		Yes	9	7	7	6	7
	NAc	No	3	3	2	3	3
		Yes	7	6	7	5	6
	vSub	No	3	3	2	3	3
		Yes	8	6	6	6	6

Supplementary Table 3_PCA results by brain region in the 95 percent most variable genes

**AntIns**	PC1_55.375.	PC2_10.719.	PC3_5.923.	PC4_3.392.	PC5_1.683.
Gender	0.302	0.077	0.000	0.856	0.995
Cause Of Death	0.003	0.996	0.924	0.584	0.414
Age	0.102	0.170	0.013	0.676	0.473
PMI	0.012	0.014	0.793	0.701	0.048
pH	0.486	0.019	0.500	0.919	0.447
Childhood Abuse	0.080	0.097	0.473	0.489	0.847
Condition	0.024	0.434	0.549	0.389	0.177
Cohort	0.000	0.076	0.732	0.344	0.996
Alcohol	0.677	0.295	0.203	0.298	0.031
Drug Abuse	0.011	0.090	0.348	0.529	0.122
Medication	0.122	0.305	0.167	0.897	0.003
Smoking	0.216	0.239	0.333	0.942	0.441
RIN	0.001	0.000	0.533	0.022	0.000
Gender+Condition	0.056	0.247	0.000	0.848	0.430

PC6_1.459.	PC7_1.218.	PC8_0.966.	PC9_0.786.	PC10_0.727.
0.783	0.499	0.975	0.859	0.918
0.401	0.078	0.647	0.148	0.495
0.863	0.531	0.142	0.001	0.316
0.007	0.941	0.018	0.600	0.118
0.439	0.000	0.923	0.653	0.186
0.000	0.639	0.151	0.827	0.509
0.125	0.298	0.690	0.352	0.521
0.030	0.778	0.001	0.579	0.305
0.034	0.755	0.279	0.489	0.071
0.376	0.392	0.589	0.100	0.875
0.712	0.825	0.681	0.710	0.922
0.615	0.370	0.516	0.871	0.912
0.240	0.877	0.882	0.431	0.952
0.476	0.607	0.968	0.807	0.570

**OFC**	PC1_60.937.	PC2_8.034.	PC3_5.729.	PC4_3.034.	PC5_2.125.	PC6_1.447.
Gender	0.291	0.000	0.000	0.378	0.875	0.636
Cause Of Death	0.001	0.091	0.389	0.471	0.817	0.026
Age	0.069	0.000	0.571	0.021	0.790	0.199
PMI	0.011	0.435	0.962	0.082	0.332	0.516
pH	0.133	0.001	0.375	0.731	0.080	0.057
Childhood Abuse	0.060	0.927	0.611	0.142	0.871	0.416
Condition	0.012	0.073	0.466	0.192	0.746	0.732
Cohort	0.000	0.688	0.707	0.103	0.838	0.416
Alcohol	0.586	0.321	0.078	0.679	0.827	0.505
Drug Abuse	0.015	0.040	0.111	0.719	0.670	0.125
Medication	0.173	0.723	0.021	0.407	0.122	0.681
Smoking	0.303	0.372	0.323	0.376	0.780	0.768
RIN	0.660	0.011	0.364	0.000	0.000	0.168
Gender+Condition	0.034	0.000	0.000	0.504	0.847	0.954

PC7_0.933.	PC8_0.723.	PC9_0.679.	PC10_0.582.
0.794	0.835	0.585	0.926
0.000	0.678	0.410	0.932
0.007	0.049	0.519	0.290
0.000	0.009	0.611	0.184
0.008	0.491	0.789	0.574
0.307	0.236	0.076	0.020
0.010	0.385	0.115	0.821
0.000	0.001	0.593	0.627
0.646	0.018	0.070	0.200
0.576	0.972	0.446	0.094
0.085	0.189	0.914	0.173
0.135	0.645	0.427	0.859
0.056	0.354	0.913	0.511
0.076	0.361	0.266	0.388

**vmPFC**	PC1_54.736.	PC2_7.871.	PC3_5.641.	PC4_4.78.	PC5_2.272.
Gender	0.250	0.000	0.000	0.000	0.750
Cause Of Death	0.008	0.517	0.680	0.247	0.391
Age	0.101	0.014	0.444	0.376	0.672
PMI	0.121	0.571	0.159	0.554	0.014
pH	0.192	0.001	0.800	0.089	0.107
Childhood Abuse	0.133	0.817	0.859	0.664	0.060
Condition	0.018	0.567	0.457	0.441	0.161
Cohort	0.000	0.433	0.921	0.870	0.200
Alcohol	0.247	0.135	0.668	0.470	0.189
Drug Abuse	0.015	0.820	0.809	0.353	0.851
Medication	0.110	0.531	0.018	0.377	0.031
Smoking	0.193	0.536	0.056	0.077	0.879
RIN	0.039	0.000	0.515	0.083	0.000
Gender+Condition	0.047	0.000	0.000	0.000	0.553

PC6_1.69.	PC7_1.524.	PC8_1.152.	PC9_0.906.	PC10_0.78.
0.672	0.615	0.926	0.852	0.843
0.014	0.955	0.035	0.636	0.826
0.015	0.027	0.399	0.477	0.708
0.001	0.910	0.342	0.227	0.732
0.004	0.065	0.114	0.374	0.234
0.069	0.890	0.906	0.004	0.723
0.022	0.664	0.102	0.250	0.967
0.032	0.462	0.903	0.259	0.106
0.147	0.214	0.703	0.686	0.723
0.098	0.463	0.281	0.794	0.565
0.201	0.416	0.377	0.757	0.693
0.713	0.174	0.117	0.982	0.937
0.777	0.165	0.365	0.531	0.170
0.109	0.613	0.256	0.681	0.636

**dlPFC**	PC1_69.353.	PC2_6.837.	PC3_4.539.	PC4_1.549.	PC5_1.372.
Gender	0.308	0.000	0.000	0.572	0.667
Cause Of Death	0.001	0.235	0.201	0.977	0.120
Age	0.136	0.021	0.081	0.553	0.001
PMI	0.013	0.030	0.639	0.207	0.000
pH	0.137	0.030	0.887	0.363	0.736
Childhood Abuse	0.106	0.974	0.898	0.657	0.056
Condition	0.012	0.590	0.651	0.430	0.023
Cohort	0.000	0.067	0.403	0.074	0.019
Alcohol	0.576	0.516	0.055	0.115	0.915
Drug Abuse	0.011	0.615	0.416	0.465	0.506
Medication	0.144	0.184	0.114	0.307	0.009
Smoking	0.260	0.122	0.124	0.968	0.690
RIN	0.916	0.000	0.121	0.004	0.017
Gender+Condition	0.037	0.001	0.000	0.801	0.154

PC6_0.975.	PC7_0.719.	PC8_0.616.	PC9_0.517.	PC10_0.502.
0.710	0.924	0.958	0.691	0.853
0.003	0.603	0.032	0.551	0.734
0.003	0.986	0.314	0.084	0.406
0.241	0.982	0.417	0.147	0.730
0.000	0.199	0.003	0.297	0.351
0.745	0.704	0.347	0.024	0.888
0.131	0.892	0.127	0.253	0.303
0.128	0.966	0.562	0.395	0.238
0.127	0.121	0.829	0.261	0.269
0.362	0.216	0.829	0.241	0.013
0.804	0.388	0.277	0.107	0.073
0.548	0.586	0.779	0.308	0.366
0.972	0.030	0.797	0.119	0.630
0.482	0.926	0.402	0.689	0.233

**NAC**	PC1_58.084.	PC2_6.45.	PC3_5.403.	PC4_4.531.	PC5_2.516.
Gender	0.397	0.000	0.000	0.356	0.647
Cause Of Death	0.004	0.866	0.343	0.610	0.305
Age	0.275	0.036	0.105	0.934	0.316
PMI	0.028	0.059	0.347	0.549	0.063
pH	0.259	0.422	0.541	0.027	0.545
Childhood Abuse	0.117	0.058	0.225	0.674	0.313
Condition	0.029	0.714	0.574	0.691	0.187
Cohort	0.000	0.030	0.261	0.072	0.189
Alcohol	0.754	0.451	0.094	0.930	0.099
Drug Abuse	0.022	0.193	0.286	0.612	0.287
Medication	0.157	0.127	0.110	0.605	0.015
Smoking	0.162	0.054	0.678	0.832	0.043
RIN	0.721	0.521	0.958	0.000	0.003
Gender+Condition	0.073	0.002	0.000	0.510	0.455

PC6_1.687.	PC7_1.387.	PC8_1.114.	PC9_0.981.	PC10_0.881.
0.491	0.901	0.894	0.880	0.837
0.003	0.710	0.371	0.476	0.112
0.193	0.474	0.528	0.785	0.288
0.969	0.002	0.833	0.312	0.623
0.000	0.230	0.534	0.912	0.188
0.193	0.450	0.201	0.404	0.946
0.398	0.599	0.095	0.286	0.520
0.202	0.087	0.655	0.128	0.301
0.329	0.092	0.725	0.515	0.353
0.642	0.980	0.558	0.731	0.376
0.994	0.679	0.371	0.616	0.922
0.229	0.761	0.802	0.675	0.110
0.016	0.659	0.135	0.592	0.444
0.734	0.564	0.432	0.336	0.933

**vSUB**	PC1_63.015.	PC2_5.862.	PC3_5.191.	PC4_3.519.	PC5_1.936.	PC6_1.676.
Gender	0.121	0.000	0.173	0.067	0.816	0.989
Cause Of Death	0.008	0.749	0.642	0.615	0.708	0.375
Age	0.269	0.089	0.550	0.135	0.154	0.456
PMI	0.006	0.713	0.670	0.570	0.102	0.303
pH	0.323	0.296	0.006	0.008	0.538	0.445
Childhood Abuse	0.230	0.862	0.778	0.543	0.626	0.084
Condition	0.026	0.686	0.984	0.272	0.344	0.819
Cohort	0.000	0.574	0.224	0.818	0.501	0.909
Alcohol	0.717	0.118	0.696	0.976	0.248	0.434
Drug Abuse	0.015	0.952	0.636	0.064	0.782	0.016
Medication	0.132	0.115	0.332	0.003	0.991	0.975
Smoking	0.204	0.768	0.207	0.283	0.755	0.825
RIN	0.054	0.030	0.000	0.475	0.061	0.030
Gender+Condition	0.037	0.000	0.604	0.211	0.514	0.876

PC7_1.366.	PC8_1.164.	PC9_1.141.	PC10_0.872.
0.776	0.963	0.580	0.990
0.572	0.036	0.911	0.866
0.014	0.982	0.807	0.779
0.576	0.318	0.511	0.012
0.170	0.655	0.972	0.237
0.035	0.695	0.697	0.129
0.252	0.043	0.769	0.898
0.195	0.604	0.513	0.004
0.253	0.126	0.503	0.780
0.897	0.166	0.896	0.813
0.581	0.536	0.871	0.247
0.737	0.202	0.947	0.606
0.203	0.840	0.485	0.756
0.659	0.135	0.874	0.983

Supplementary Table 4. Cohort composition by brain region

Region	Women CTRL	Women MDD	Men CTRL	Men MDD
aINS	21	23	16	25
OFC	21	23	16	24
vmPFC	19	25	17	22
dlPFC	21	24	17	24
NAc	19	22	15	24
vSub	12	19	12	18

Supplementary Table 5_list of DEGs in men

AnIns

gene_id	gene_name	logFC	AveExpr	t	P.Value
ENSG00000208018	MIR645	1.207	-2.617	3.997	0.000
ENSG00000248810	LINC02432	-1.541	-2.240	-3.977	0.000
ENSG00000154734	ADAMTS1	-0.923	4.336	-3.737	0.000
ENSG00000151892	GFRA1	-0.451	5.484	-3.724	0.000
ENSG00000266187	RN7SL480P	1.824	-3.496	3.715	0.000
ENSG00000285728	#N/A	0.527	-0.033	3.691	0.000
ENSG00000200903	RNU1-42P	1.554	-3.582	3.576	0.001
ENSG00000140092	FBLN5	-0.433	2.869	-3.504	0.001
ENSG00000267191	RP11-15A1.2	0.581	-0.165	3.447	0.001
ENSG00000231769	RP1-8B1.4	0.611	-0.011	3.441	0.001
ENSG00000170946	DNAJC24	0.311	4.577	3.416	0.001
ENSG00000099994	SUSD2	-1.115	-1.234	-3.405	0.001
ENSG00000183508	FAM46C	-0.680	0.506	-3.401	0.001
ENSG00000243321	LINC01998	-1.022	-0.873	-3.371	0.001
ENSG00000238825	RNU1-13P	1.111	-0.311	3.365	0.001
ENSG00000159388	BTG2	-0.793	4.356	-3.336	0.001
ENSG00000181847	TIGIT	-0.913	-1.862	-3.325	0.001
ENSG00000241391	RN7SL234P	1.319	-2.723	3.300	0.001
ENSG00000212205	Y_RNA	0.948	-2.323	3.293	0.001
ENSG00000244389	RN7SL242P	1.684	-0.208	3.275	0.002
ENSG00000074047	GLI2	-0.665	0.982	-3.262	0.002
ENSG00000105825	TFPI2	-1.290	-1.913	-3.254	0.002
ENSG00000118523	CTGF	-0.746	3.219	-3.251	0.002
ENSG00000224016	RP11-728K20.1	-0.591	-0.683	-3.236	0.002
ENSG00000242170	RN7SL329P	1.505	1.133	3.218	0.002
ENSG00000206702	RNU1-11P	0.783	-0.632	3.210	0.002
ENSG00000272754	AL133245.2	1.129	-2.183	3.196	0.002
ENSG00000263905	RN7SL555P	1.733	-1.986	3.170	0.002
ENSG00000163820	FYCO1	-0.362	4.107	-3.148	0.002
ENSG00000143867	OSR1	-0.683	0.517	-3.129	0.002
ENSG00000244113	RP11-468H14.1	1.308	-2.775	3.112	0.003
ENSG00000259276	RP11-815J21.3	-0.895	-1.716	-3.106	0.003
ENSG00000197728	RPS26	0.748	2.026	3.100	0.003
ENSG00000254651	RP11-430H10.3	1.058	-2.060	3.092	0.003
ENSG00000212443	SNORA53	0.895	-1.605	3.068	0.003
ENSG00000006327	TNFRSF12A	-1.118	-1.795	-3.067	0.003
ENSG00000178764	ZHX2	-0.403	4.237	-3.056	0.003
ENSG00000184678	HIST2H2BE	-0.419	3.639	-3.041	0.003
ENSG00000181104	F2R	-0.398	1.610	-3.023	0.003
ENSG00000207349	RNVU1-17	1.493	-3.471	3.018	0.003
ENSG00000236936	RP3-329E20.2	-0.578	-1.298	-3.001	0.004
ENSG00000112715	VEGFA	-0.579	5.008	-2.996	0.004
ENSG00000259356	RP11-90E5.1	-0.985	-1.538	-2.996	0.004
ENSG00000248479	RP11-807H7.2	0.633	-0.296	2.979	0.004
ENSG00000200354	SNORA71D	1.190	-1.416	2.971	0.004

ENSG00000167419	LPO	0.499	0.081	2.965	0.004
ENSG00000168243	GNG4	-0.359	5.269	-2.960	0.004
ENSG00000279117	CTD-2562J17.6	-0.225	5.777	-2.957	0.004
ENSG00000285595	#N/A	-0.759	-1.804	-2.949	0.004
ENSG00000145975	FAM217A	0.625	-1.151	2.943	0.004
ENSG00000242894	RN7SL634P	1.409	-2.695	2.941	0.004
ENSG00000109667	SLC2A9	-0.444	0.301	-2.939	0.004
ENSG00000226977	HMGN1P24	0.532	-0.454	2.916	0.005
ENSG00000276031	RN7SL197P	1.192	-3.089	2.913	0.005
ENSG00000142871	CYR61	-0.717	2.428	-2.911	0.005
ENSG00000222764	RN7SKP106	0.838	-2.098	2.911	0.005
ENSG00000188783	PRELP	-0.556	3.668	-2.906	0.005
ENSG00000279039	CTC-260E6.3	1.132	-2.026	2.904	0.005
ENSG00000166199	ALKBH3	0.288	3.303	2.897	0.005
ENSG00000243491	RP11-521D12.5	-1.091	-2.354	-2.893	0.005
ENSG00000276588	RN7SL322P	1.207	-2.778	2.892	0.005
ENSG00000226051	ZNF503-AS1	0.782	-1.293	2.892	0.005
ENSG00000236999	ATP2B2-IT1	0.798	-1.933	2.887	0.005
ENSG00000265753	RN7SL444P	1.102	-1.929	2.880	0.005
ENSG00000255524	NPIPB8	0.904	0.030	2.877	0.005
ENSG00000185896	LAMP1	-0.181	5.936	-2.877	0.005
ENSG00000269794	AC010642.2	0.977	-2.005	2.875	0.005
ENSG00000121966	CXCR4	-0.797	1.593	-2.874	0.005
ENSG00000196562	SULF2	-0.273	5.517	-2.873	0.005
ENSG00000272829	XXbac-B135H6.18	-0.749	-2.069	-2.856	0.005
ENSG00000258824	CTD-2555O16.2	-0.396	0.356	-2.853	0.005
ENSG00000265813	RN7SL300P	1.460	-0.552	2.843	0.006
ENSG00000136715	SAP130	-0.202	4.790	-2.843	0.006
ENSG00000243951	RN7SL308P	0.773	-0.127	2.837	0.006
ENSG00000251401	RP11-11N6.1	1.039	-2.293	2.833	0.006
ENSG00000239942	RN7SL394P	1.339	-0.658	2.829	0.006
ENSG00000124243	BCAS4	0.268	5.121	2.828	0.006
ENSG00000166482	MFAP4	-0.866	0.851	-2.827	0.006
ENSG00000264169	RN7SL665P	0.887	1.418	2.827	0.006
ENSG00000113319	RASGRF2	0.307	8.245	2.826	0.006
ENSG00000230325	RP11-385F5.4	-0.768	-2.616	-2.824	0.006
ENSG00000153266	FEZF2	-0.358	3.765	-2.823	0.006
ENSG00000162772	ATF3	-0.864	2.105	-2.819	0.006
ENSG00000121101	TEX14	-0.537	0.172	-2.818	0.006
ENSG00000115461	IGFBP5	-0.427	6.440	-2.817	0.006
ENSG00000279684	RP11-755E23.2	1.152	-2.297	2.812	0.006
ENSG00000241162	RN7SL617P	1.577	-0.536	2.812	0.006
ENSG00000211584	SLC48A1	-0.391	5.495	-2.808	0.006
ENSG00000174021	GNG5	-0.864	-1.582	-2.807	0.006
ENSG00000060237	WNK1	-0.254	8.786	-2.798	0.006
ENSG00000185860	CCDC190	-0.791	0.591	-2.793	0.006
ENSG00000140836	ZFHX3	-0.233	5.284	-2.787	0.007

ENSG00000272197	RN7SL803P	0.971	-2.092	2.778	0.007
ENSG00000185053	SGCZ	0.321	2.932	2.774	0.007
ENSG00000143067	ZNF697	0.263	5.527	2.772	0.007
ENSG00000200726	SNORD115-8	0.913	-1.892	2.767	0.007
ENSG00000247157	LINC01252	0.341	3.130	2.762	0.007
ENSG00000173402	DAG1	-0.280	5.558	-2.760	0.007
ENSG00000282997	RP11-95C14.1	-0.685	-0.217	-2.758	0.007
ENSG00000145428	RNF175	0.346	4.335	2.755	0.007
ENSG00000095485	CWF19L1	0.261	3.822	2.749	0.007
ENSG00000092094	OSGEP	0.347	4.517	2.746	0.007
ENSG00000239868	RN7SL34P	1.132	-1.735	2.746	0.007
ENSG00000235271	LINC01422	0.702	-2.009	2.745	0.007
ENSG00000186352	ANKRD37	-0.564	1.411	-2.743	0.007
ENSG00000285257	#N/A	-0.768	-0.697	-2.738	0.008
ENSG00000019186	CYP24A1	1.045	-0.155	2.729	0.008
ENSG00000144285	SCN1A	0.408	7.872	2.726	0.008
ENSG00000011295	TTC19	0.226	6.986	2.723	0.008
ENSG00000153094	BCL2L11	-0.251	2.559	-2.715	0.008
ENSG00000131471	AOC3	-0.887	-0.504	-2.713	0.008
ENSG00000251552	COQ10BP2	-0.733	-1.753	-2.707	0.008
ENSG00000250643	RP11-93K22.6	-0.908	-2.163	-2.705	0.008
ENSG00000212232	SNORD17	0.596	3.298	2.704	0.008
ENSG00000271973	RP11-572O6.1	0.802	-2.292	2.699	0.008
ENSG00000120129	DUSP1	-0.662	4.177	-2.698	0.008
ENSG00000263551	RP11-78F17.1	0.706	-1.231	2.696	0.008
ENSG00000244264	RN7SL597P	1.097	-2.528	2.695	0.009
ENSG00000277450	CTB-96E2.10	0.474	-0.637	2.694	0.009
ENSG00000112902	SEMA5A	-0.250	6.231	-2.693	0.009
ENSG00000183486	MX2	-0.448	1.457	-2.690	0.009
ENSG00000146909	NOM1	0.174	4.551	2.682	0.009
ENSG00000171310	CHST11	-0.205	5.203	-2.681	0.009
ENSG00000049323	LTBP1	-0.368	4.242	-2.680	0.009
ENSG00000271601	LIX1L	-0.280	4.095	-2.680	0.009
ENSG00000169604	ANTXR1	-0.288	5.459	-2.680	0.009
ENSG00000274357	Metazoa_SRP	1.581	-2.329	2.678	0.009
ENSG00000198342	ZNF442	0.374	2.351	2.674	0.009
ENSG00000278099	RNVU1-2A	0.963	1.426	2.671	0.009
ENSG00000204388	HSPA1B	-0.830	5.029	-2.670	0.009
ENSG00000240750	RN7SL559P	0.701	-0.609	2.670	0.009
ENSG00000196814	MVB12B	-0.245	5.342	-2.668	0.009
ENSG00000153495	TEX29	0.563	0.761	2.666	0.009
ENSG00000255202	RP4-541C22.5	0.701	-2.173	2.662	0.009
ENSG00000153291	SLC25A27	0.227	6.343	2.661	0.009
ENSG00000266844	RP11-862L9.3	-0.964	-2.682	-2.659	0.009
ENSG00000081307	UBA5	0.263	5.994	2.656	0.009
ENSG00000275803	RN7SL736P	0.857	-0.758	2.655	0.009
ENSG00000199970	SNORD115-3	0.828	-1.744	2.652	0.010

ENSG00000226770	AC000124.1	0.882	-2.108	2.647	0.010
ENSG00000232287	SLC6A1-AS1	-0.425	1.340	-2.640	0.010
ENSG00000168032	ENTPD3	0.263	4.406	2.639	0.010
ENSG00000213891	RPL3P6	-0.474	0.794	-2.636	0.010
ENSG00000232277	AC104651.3	-0.898	-1.686	-2.636	0.010
ENSG00000137760	ALKBH8	0.280	4.308	2.635	0.010
ENSG00000254236	KB-1639H6.2	0.822	-1.654	2.634	0.010
ENSG00000274114	CTC-281F24.3	0.510	1.143	2.634	0.010
ENSG00000207750	MIR553	0.922	-2.115	2.633	0.010
ENSG00000238168	RP11-686G8.1	-0.507	-0.887	-2.632	0.010
ENSG00000081913	PHLPP1	-0.234	6.411	-2.629	0.010
ENSG00000099139	PCSK5	-0.236	4.419	-2.628	0.010
ENSG00000234817	RP3-400B16.1	-0.577	-0.757	-2.626	0.010
ENSG00000118515	SGK1	-0.416	5.285	-2.625	0.010
ENSG00000213551	DNAJC9	0.237	4.337	2.625	0.010
ENSG00000207445	SNORD15B	1.048	-2.681	2.620	0.010
ENSG00000170345	FOS	-0.996	4.591	-2.619	0.010
ENSG00000250446	LINC01332	0.860	-2.180	2.618	0.011
ENSG00000256870	SLC5A8	0.503	0.543	2.617	0.011
ENSG00000240207	RP11-379F4.4	0.249	3.882	2.615	0.011
ENSG00000251226	RP11-469N6.1	0.926	0.747	2.615	0.011
ENSG00000232445	RP11-132A1.4	0.763	-1.784	2.614	0.011
ENSG00000148926	ADM	-0.980	0.410	-2.614	0.011
ENSG00000254101	LINC02055	0.443	3.115	2.613	0.011
ENSG00000241834	RN7SL149P	0.963	-1.994	2.612	0.011
ENSG00000065534	MYLK	-0.284	6.518	-2.609	0.011
ENSG00000174448	STARD6	0.940	-2.093	2.608	0.011
ENSG00000280291	RP11-759A24.2	-0.763	-1.455	-2.604	0.011
ENSG00000198829	SUCNR1	1.201	-0.636	2.603	0.011
ENSG00000225938	RP4-575N6.4	-0.686	-1.328	-2.602	0.011
ENSG00000204389	HSPA1A	-0.955	5.523	-2.599	0.011
ENSG00000200593	SNORD115-33	1.013	-0.973	2.597	0.011
ENSG00000118503	TNFAIP3	-0.538	1.306	-2.592	0.011
ENSG00000111684	LPCAT3	-0.268	1.940	-2.592	0.011
ENSG00000075673	ATP12A	1.117	-2.121	2.589	0.011
ENSG00000168209	DDIT4	-0.621	2.806	-2.587	0.011
ENSG00000243902	ELFN2	0.426	0.392	2.585	0.011
ENSG00000249203	LINC02224	0.670	-1.462	2.584	0.012
ENSG00000234545	FAM133B	0.292	3.984	2.584	0.012
ENSG00000200959	SNORA74A	0.719	-0.639	2.583	0.012
ENSG00000115290	GRB14	0.465	3.103	2.580	0.012
ENSG00000258399	MEG8	0.396	2.710	2.579	0.012
ENSG00000260628	RP11-1166P10.1	-0.446	0.946	-2.576	0.012
ENSG00000122254	HS3ST2	-0.396	2.777	-2.575	0.012
ENSG00000196456	ZNF775	-0.587	-0.515	-2.571	0.012
ENSG00000179673	RPRML	0.562	0.249	2.571	0.012
ENSG00000146859	TMEM140	-0.333	2.136	-2.570	0.012

ENSG00000035862	TIMP2	-0.192	6.744	-2.570	0.012
ENSG00000223812	RP11-197K6.1	0.467	3.305	2.563	0.012
ENSG00000131969	ABHD12B	0.284	2.440	2.562	0.012
ENSG00000142173	COL6A2	-0.703	1.288	-2.561	0.012
ENSG00000276627	RP5-1016A21.1	0.721	-1.848	2.554	0.012
ENSG00000258498	DIO3OS	-0.877	-1.715	-2.553	0.013
ENSG00000275703	U47924.32	0.852	-2.345	2.551	0.013
ENSG00000186340	THBS2	-0.399	4.630	-2.548	0.013
ENSG00000264920	RP11-6N17.4	0.340	1.049	2.547	0.013
ENSG00000265386	RN7SL219P	1.207	-3.337	2.547	0.013
ENSG00000257319	RP11-478B9.1	-0.727	-2.175	-2.545	0.013
ENSG00000128016	ZFP36	-1.089	2.047	-2.544	0.013
ENSG00000189334	S100A14	0.955	-2.278	2.542	0.013
ENSG00000200795	RNU4-1	0.687	3.396	2.542	0.013
ENSG00000151164	RAD9B	-0.534	0.036	-2.541	0.013
ENSG00000118004	COLEC11	-1.004	-1.076	-2.538	0.013
ENSG00000228735	GS1-18A18.2	0.628	0.082	2.536	0.013
ENSG00000124762	CDKN1A	-0.839	2.124	-2.536	0.013
ENSG00000171714	ANO5	0.280	6.462	2.534	0.013
ENSG00000116044	NFE2L2	-0.255	5.511	-2.531	0.013
ENSG00000250846	EPHA5-AS1	0.466	-0.427	2.529	0.013
ENSG00000121797	CCRL2	-0.698	-1.241	-2.529	0.013
ENSG00000021300	PLEKHB1	-0.325	7.809	-2.523	0.014
ENSG00000203772	SPRN	-0.413	3.823	-2.522	0.014
ENSG00000150995	ITPR1	0.197	9.254	2.517	0.014
ENSG00000199476	Y_RNA	-0.562	-1.428	-2.517	0.014
ENSG00000254860	TMEM9B-AS1	0.460	0.075	2.515	0.014
ENSG00000123358	NR4A1	-0.769	3.253	-2.507	0.014
ENSG00000206634	SNORA22	0.617	1.462	2.507	0.014
ENSG00000069399	BCL3	-0.670	-0.697	-2.506	0.014
ENSG00000168824	NSG1	0.298	5.762	2.505	0.014
ENSG00000104953	TLE6	1.060	-2.453	2.504	0.014
ENSG00000279521	RP11-314P15.1	0.853	-0.230	2.503	0.014
ENSG00000199812	RNU6-428P	1.251	-3.997	2.501	0.014
ENSG00000139880	CDH24	-0.472	2.216	-2.500	0.014
ENSG00000254221	PCDHGB1	-0.344	2.120	-2.498	0.014
ENSG00000108405	P2RX1	-0.952	-1.970	-2.496	0.015
ENSG00000208308	SNORA40	0.630	-0.853	2.496	0.015
ENSG00000265918	RN7SL543P	1.001	-2.388	2.495	0.015
ENSG00000180447	GAS1	-0.353	1.705	-2.495	0.015
ENSG00000222069	RN7SKP285	0.786	-2.478	2.494	0.015
ENSG00000215478	CES5AP1	0.779	-1.943	2.493	0.015
ENSG00000163909	HEYL	-0.536	0.787	-2.492	0.015
ENSG00000161021	MAML1	-0.202	4.561	-2.488	0.015
ENSG00000240606	RN7SL564P	1.195	-1.987	2.488	0.015
ENSG00000227836	AC008850.3	0.784	-1.762	2.488	0.015
ENSG00000141568	FOXK2	-0.228	5.512	-2.487	0.015

ENSG00000236511	LINC01231	1.057	-2.296	2.487	0.015
ENSG00000125740	FOSB	-0.673	2.275	-2.486	0.015
ENSG00000242214	MTND3P6	-0.865	-1.264	-2.486	0.015
ENSG00000020256	ZFP64	-0.203	3.857	-2.485	0.015
ENSG00000250069	CTB-131B5.2	-0.605	-0.056	-2.484	0.015
ENSG00000229010	RP5-979D14.1	0.676	-1.456	2.484	0.015
ENSG00000257986	LINC02306	0.833	0.393	2.484	0.015
ENSG00000227359	AC017074.2	-1.013	-2.501	-2.482	0.015
ENSG00000103241	FOXF1	-0.766	0.058	-2.482	0.015
ENSG00000244194	RN7SL218P	0.992	-0.155	2.481	0.015
ENSG00000253317	RP11-142A23.1	0.759	-1.250	2.480	0.015
ENSG00000139433	GLTP	-0.375	4.344	-2.477	0.015
ENSG00000272787	KB-226F1.2	-0.739	-2.337	-2.476	0.015
ENSG00000187479	C11orf96	-0.816	-0.047	-2.476	0.015
ENSG00000240718	RN7SL851P	1.128	-2.599	2.472	0.015
ENSG00000078246	TULP3	-0.298	3.678	-2.469	0.016
ENSG00000183864	TOB2	-0.296	5.266	-2.469	0.016
ENSG00000179761	PIPOX	-0.346	2.975	-2.468	0.016
ENSG00000243304	CTC-428G20.1	-0.945	-2.726	-2.467	0.016
ENSG00000273783	CTD-2506P8.6	-0.644	-1.615	-2.461	0.016
ENSG00000275120	RP11-182J1.18	-0.437	-0.359	-2.460	0.016
ENSG00000164949	GEM	-0.504	2.499	-2.458	0.016
ENSG00000150556	LYPD6B	0.365	0.947	2.456	0.016
ENSG00000259408	RP11-3D4.3	-0.463	-0.511	-2.456	0.016
ENSG00000239468	RN7SL569P	0.854	-2.251	2.453	0.016
ENSG00000244139	RN7SL397P	1.020	-3.782	2.452	0.016
ENSG00000261357	RP11-626G11.1	-0.837	-2.026	-2.451	0.016
ENSG00000243446	RN7SL284P	0.807	-1.881	2.450	0.016
ENSG00000165521	EML5	0.227	5.535	2.449	0.016
ENSG00000221931	OR6X1	0.766	-1.553	2.448	0.016
ENSG00000235284	SNORD62A	1.038	-3.444	2.446	0.017
ENSG00000038427	VCAN	-0.324	7.887	-2.445	0.017
ENSG00000259286	RP11-696L21.2	0.796	-1.902	2.444	0.017
ENSG00000131788	PIAS3	-0.265	3.591	-2.444	0.017
ENSG00000261560	RP11-166B2.3	0.430	0.171	2.444	0.017
ENSG00000251022	THAP9-AS1	0.284	5.102	2.442	0.017
ENSG00000248049	UBA6-AS1	-0.236	4.230	-2.441	0.017
ENSG00000137331	IER3	-0.604	0.302	-2.441	0.017
ENSG00000228649	AC005682.5	-0.690	-1.572	-2.441	0.017
ENSG00000159307	SCUBE1	-0.629	1.250	-2.440	0.017
ENSG00000144893	MED12L	0.156	6.713	2.437	0.017
ENSG00000144802	NFKBIZ	-0.377	2.944	-2.437	0.017
ENSG00000183580	FBXL7	-0.334	3.330	-2.436	0.017
ENSG00000270091	RP11-78O7.2	0.299	1.374	2.434	0.017
ENSG00000130287	NCAN	-0.265	7.277	-2.431	0.017
ENSG00000151876	FBXO4	0.268	2.709	2.429	0.017
ENSG00000214595	EML6	0.201	6.452	2.427	0.017

ENSG00000241939	RN7SL517P	-0.542	-1.517	-2.427	0.017
ENSG00000188771	PLET1	0.681	-2.036	2.427	0.017
ENSG00000267397	RP11-873E20.1	0.506	-0.154	2.427	0.017
ENSG00000156269	NAA11	-0.835	-1.925	-2.427	0.017
ENSG00000163507	KIAA1524	0.311	3.716	2.426	0.017
ENSG00000198633	ZNF534	0.371	2.365	2.426	0.017
ENSG00000248319	LINC02275	0.935	-2.074	2.421	0.018
ENSG00000166387	PPFIBP2	-0.327	3.864	-2.419	0.018
ENSG00000047457	CP	-0.980	2.405	-2.418	0.018
ENSG00000167604	NFKBID	-0.563	0.508	-2.416	0.018
ENSG00000257497	RP11-585P4.5	0.545	-0.218	2.413	0.018
ENSG00000270802	AC005776.1	0.635	-1.261	2.413	0.018
ENSG00000130592	LSP1	-0.627	-0.475	-2.407	0.018
ENSG00000238077	NMNAT1P3	0.518	-0.153	2.406	0.018
ENSG00000177051	FBXO46	-0.388	1.931	-2.406	0.018
ENSG00000187266	EPOR	-0.305	1.316	-2.406	0.018
ENSG00000157851	DPYSL5	-0.303	4.949	-2.405	0.018
ENSG00000064218	DMRT3	0.732	-0.779	2.405	0.018
ENSG00000165633	VSTM4	-0.189	4.283	-2.404	0.018
ENSG00000020426	MNAT1	0.227	5.545	2.404	0.018
ENSG00000026025	VIM	-0.426	5.460	-2.404	0.018
ENSG00000059588	TARBP1	0.298	6.942	2.404	0.018
ENSG00000261654	RP11-96K19.4	0.448	1.330	2.401	0.019
ENSG00000258301	RP11-488C13.5	0.428	-0.799	2.397	0.019
ENSG00000172893	DHCR7	-0.346	3.522	-2.393	0.019
ENSG00000104177	MYEF2	0.212	7.236	2.393	0.019
ENSG00000087903	RFX2	-0.307	2.402	-2.391	0.019
ENSG00000196935	SRGAP1	-0.213	4.835	-2.391	0.019
ENSG00000198795	ZNF521	-0.202	4.564	-2.391	0.019
ENSG00000132394	EEFSEC	-0.331	1.395	-2.390	0.019
ENSG00000196743	GM2A	-0.238	5.235	-2.389	0.019
ENSG00000268713	CTC-444N24.8	-0.277	2.057	-2.388	0.019
ENSG00000163444	TMEM183A	0.316	6.038	2.387	0.019
ENSG00000144810	COL8A1	-0.833	1.009	-2.387	0.019
ENSG00000283390	RP11-134F2.7	-0.535	-0.268	-2.387	0.019
ENSG00000166484	MAPK7	-0.413	1.498	-2.387	0.019
ENSG00000144161	ZC3H8	0.210	5.858	2.386	0.019
ENSG00000199266	SNORA60	0.808	-0.136	2.385	0.019
ENSG00000135605	TEC	0.442	1.205	2.385	0.019
ENSG00000153048	CARHSP1	-0.230	3.109	-2.384	0.019
ENSG00000224609	HSD52	0.387	0.822	2.383	0.019
ENSG00000008516	MMP25	-0.641	-1.929	-2.383	0.019
ENSG00000121236	TRIM6	-0.577	-0.617	-2.382	0.020
ENSG00000166582	CENPV	0.202	3.564	2.381	0.020
ENSG00000183696	UPP1	0.255	4.211	2.380	0.020
ENSG00000204361	NXPE2	-0.490	-0.768	-2.378	0.020
ENSG00000128641	MYO1B	0.334	5.640	2.377	0.020

ENSG00000223341	RN7SKP3	0.956	-4.358	2.374	0.020
ENSG00000105877	DNAH11	-0.304	1.854	-2.373	0.020
ENSG00000254092	RP11-369E15.3	0.503	0.363	2.372	0.020
ENSG00000106538	RARRES2	-0.469	0.778	-2.372	0.020
ENSG00000121104	FAM117A	-0.289	1.680	-2.371	0.020
ENSG00000111364	DDX55	0.191	4.299	2.370	0.020
ENSG00000250015	CTC-339F2.2	-0.607	-1.503	-2.368	0.020
ENSG00000154856	APCDD1	-0.301	4.853	-2.367	0.020
ENSG00000197927	C2orf27A	0.289	2.768	2.367	0.020
ENSG00000201634	SNORD115-48	0.813	-3.155	2.367	0.020
ENSG00000225465	RFPL1S	0.307	6.553	2.366	0.020
ENSG00000257964	RP11-133N21.10	0.460	-0.117	2.364	0.020
ENSG00000277586	NEFL	0.327	9.323	2.363	0.020
ENSG00000144655	CSRNP1	-0.405	2.936	-2.361	0.021
ENSG00000201998	SNORA23	0.540	2.517	2.360	0.021
ENSG00000225447	RPS15AP10	-0.600	-0.381	-2.360	0.021
ENSG00000264151	RP11-739N10.1	-0.769	-2.063	-2.359	0.021
ENSG00000135776	ABCB10	0.245	3.978	2.357	0.021
ENSG00000103507	BCKDK	-0.211	3.738	-2.356	0.021
ENSG00000243620	RP11-649A16.1	0.937	-2.386	2.355	0.021
ENSG00000185650	ZFP36L1	-0.366	5.362	-2.355	0.021
ENSG00000177201	OR2T12	0.826	-1.058	2.354	0.021
ENSG00000232940	HCG25	0.550	-1.163	2.351	0.021
ENSG00000214491	SEC14L6	-0.368	1.424	-2.349	0.021
ENSG00000100243	CYB5R3	-0.417	4.727	-2.349	0.021
ENSG00000248540	RP11-247C2.2	-0.409	1.342	-2.349	0.021
ENSG00000235092	ID2-AS1	-0.310	1.501	-2.348	0.021
ENSG00000106333	PCOLCE	-0.738	-0.797	-2.348	0.021
ENSG00000104853	CLPTM1	-0.235	6.120	-2.348	0.021
ENSG00000135736	CCDC102A	-0.699	-1.118	-2.347	0.021
ENSG00000279932	RP11-349F21.2	-0.914	-2.086	-2.346	0.021
ENSG00000272335	RP11-53O19.3	0.262	5.161	2.345	0.021
ENSG00000147955	SIGMAR1	-0.209	3.844	-2.345	0.021
ENSG00000138036	DYNC2LI1	0.233	5.183	2.344	0.021
ENSG00000263499	RP11-118E18.4	-0.428	-0.071	-2.344	0.021
ENSG00000112062	MAPK14	0.135	5.872	2.344	0.022
ENSG00000085382	HACE1	0.219	6.616	2.343	0.022
ENSG00000102466	FGF14	0.233	6.676	2.342	0.022
ENSG00000164318	EGFLAM	-0.596	-0.780	-2.342	0.022
ENSG00000142621	FHAD1	0.308	2.098	2.342	0.022
ENSG00000150527	CTAGE5	0.240	5.013	2.341	0.022
ENSG00000253305	PCDHGB6	-0.255	3.257	-2.341	0.022
ENSG00000162512	SDC3	-0.250	7.030	-2.341	0.022
ENSG00000277062	RN7SL88P	0.681	-0.333	2.340	0.022
ENSG00000203737	GPR52	-0.284	1.962	-2.339	0.022
ENSG00000274180	NATD1	-0.324	2.037	-2.339	0.022
ENSG00000228868	MYLKP1	-0.686	-1.822	-2.338	0.022

ENSG00000139438	FAM222A	-0.373	2.804	-2.338	0.022
ENSG00000276578	LLNLR-285B5.1	0.466	-0.377	2.338	0.022
ENSG00000227745	AC098826.5	0.431	2.935	2.336	0.022
ENSG00000258711	RP11-218E20.3	0.770	-0.584	2.335	0.022
ENSG00000112769	LAMA4	-0.232	4.892	-2.334	0.022
ENSG00000198142	SOWAHC	-0.278	2.043	-2.329	0.022
ENSG00000136297	MMD2	-0.401	3.576	-2.329	0.022
ENSG00000233900	RPL17P35	0.781	-1.227	2.329	0.022
ENSG00000198270	TMEM116	-0.155	4.398	-2.326	0.022
ENSG00000172380	GNG12	-0.321	4.663	-2.326	0.022
ENSG00000109390	NDUFC1	0.234	3.685	2.324	0.023
ENSG00000285852	#N/A	-0.433	-0.486	-2.324	0.023
ENSG00000183055	FAM133CP	0.468	1.073	2.321	0.023
ENSG00000263624	RP11-45M22.3	-0.512	-0.116	-2.320	0.023
ENSG00000234056	LINC00463	-0.852	-1.803	-2.320	0.023
ENSG00000143412	ANXA9	-0.376	1.005	-2.319	0.023
ENSG00000144407	PTH2R	0.429	2.199	2.318	0.023
ENSG00000130270	ATP8B3	-0.789	-2.158	-2.315	0.023
ENSG00000258859	LINC02296	0.676	0.188	2.315	0.023
ENSG00000240498	CDKN2B-AS1	0.492	0.163	2.314	0.023
ENSG00000205279	CTXN3	0.600	3.302	2.313	0.023
ENSG00000128284	APOL3	0.442	2.072	2.312	0.023
ENSG00000273394	RP11-255E6.6	0.868	-1.747	2.312	0.023
ENSG00000276547	PCDHGB5	-0.214	3.087	-2.311	0.023
ENSG00000258842	RP11-355I22.5	0.600	-1.651	2.310	0.023
ENSG00000227502	LINC01268	0.549	1.317	2.310	0.023
ENSG00000164405	UQCRQ	0.330	4.961	2.308	0.024
ENSG00000248485	PCP4L1	0.368	4.160	2.308	0.024
ENSG00000205758	CRYZL1	0.204	5.283	2.306	0.024
ENSG00000261916	RP11-235E17.4	-0.498	-1.344	-2.305	0.024
ENSG00000171223	JUNB	-0.717	3.073	-2.301	0.024
ENSG00000106069	CHN2	-0.361	1.623	-2.301	0.024
ENSG00000011422	PLAUR	-0.511	0.644	-2.300	0.024
ENSG00000243870	RN7SL236P	0.795	-3.913	2.300	0.024
ENSG00000184557	SOCS3	-1.304	0.603	-2.299	0.024
ENSG00000135540	NHSL1	-0.282	4.950	-2.299	0.024
ENSG00000247287	RP11-902B17.1	0.654	-0.671	2.296	0.024
ENSG00000275835	TUBGCP5	0.215	5.341	2.294	0.024
ENSG00000138769	CDKL2	0.251	5.308	2.293	0.024
ENSG00000115232	ITGA4	0.362	2.642	2.293	0.024
ENSG00000204909	SPINK9	-0.431	0.300	-2.291	0.025
ENSG00000172000	ZNF556	0.516	-0.794	2.291	0.025
ENSG00000065548	ZC3H15	0.285	7.091	2.290	0.025
ENSG00000161681	SHANK1	-0.465	5.405	-2.290	0.025
ENSG00000204262	COL5A2	0.491	5.256	2.290	0.025
ENSG00000138160	KIF11	0.275	2.239	2.289	0.025
ENSG00000201027	RN7SKP107	0.990	-3.280	2.289	0.025

ENSG00000258794	DUX4L27	-0.847	-1.930	-2.287	0.025
ENSG00000274659	LINC02371	0.643	-1.321	2.285	0.025
ENSG00000105355	PLIN3	-0.498	0.892	-2.284	0.025
ENSG00000213064	SFT2D2	-0.240	5.483	-2.284	0.025
ENSG00000186166	CCDC84	0.245	4.894	2.283	0.025
ENSG00000255853	RP11-407A16.1	0.653	-0.160	2.282	0.025
ENSG00000106336	FBXO24	-0.626	-1.740	-2.281	0.025
ENSG00000140832	MARVELD3	0.533	-0.525	2.281	0.025
ENSG00000147419	CCDC25	0.247	6.378	2.278	0.025
ENSG00000223882	ABCC5-AS1	0.560	-0.725	2.277	0.025
ENSG00000170791	CHCHD7	0.288	5.421	2.275	0.025
ENSG00000124721	DNAH8	0.463	-0.173	2.275	0.025
ENSG00000097007	ABL1	-0.255	4.670	-2.275	0.026
ENSG00000102678	FGF9	0.348	4.480	2.275	0.026
ENSG00000285554	#N/A	-0.815	-2.386	-2.274	0.026
ENSG00000228022	HCG20	-0.856	-2.024	-2.274	0.026
ENSG00000115507	OTX1	-0.533	0.334	-2.274	0.026
ENSG00000157766	ACAN	-0.554	0.680	-2.274	0.026
ENSG00000182621	PLCB1	0.289	8.643	2.274	0.026
ENSG00000260157	CH17-53B9.2	0.712	-0.909	2.274	0.026
ENSG00000267890	CTD-2126E3.4	-0.530	0.240	-2.272	0.026
ENSG00000104327	CALB1	0.473	5.853	2.272	0.026
ENSG00000119699	TGFB3	-0.372	4.195	-2.270	0.026
ENSG00000077782	FGFR1	-0.217	5.561	-2.270	0.026
ENSG00000277918	RNVU1-28	0.802	2.596	2.269	0.026
ENSG00000259411	HNRNPA1P45	-0.813	-1.778	-2.267	0.026
ENSG00000136859	ANGPTL2	-0.250	2.830	-2.267	0.026
ENSG00000152578	GRIA4	0.246	7.916	2.267	0.026
ENSG00000188996	HUS1B	0.655	-1.207	2.266	0.026
ENSG00000170419	VSTM2A	0.334	7.081	2.266	0.026
ENSG00000275426	CH17-262A2.1	0.532	-0.260	2.264	0.026
ENSG00000284686	#N/A	-0.848	-2.588	-2.263	0.026
ENSG00000197763	TXNRD3	0.204	3.141	2.263	0.026
ENSG00000252080	RNU7-99P	0.756	-2.166	2.262	0.026
ENSG00000176984	AP000679.2	-0.642	-1.880	-2.262	0.026
ENSG00000248221	STX18-IT1	-0.542	-1.534	-2.259	0.027
ENSG00000048471	SNX29	-0.186	5.103	-2.259	0.027
ENSG00000260785	CASC17	-0.653	-2.050	-2.258	0.027
ENSG00000233583	RP4-635A23.3	-0.779	-1.852	-2.257	0.027
ENSG00000247828	TMEM161B-AS1	0.206	5.726	2.257	0.027
ENSG00000155970	MICU3	0.336	6.818	2.256	0.027
ENSG00000119705	SLIRP	0.307	4.501	2.256	0.027
ENSG00000278901	AC005609.17	0.616	-1.844	2.255	0.027
ENSG00000279071	RP11-83B20.3	0.868	-2.303	2.255	0.027
ENSG00000157833	GAREM2	-0.362	4.157	-2.255	0.027
ENSG00000186777	ZNF732	0.462	1.053	2.255	0.027
ENSG00000239205	LINC02023	0.882	-2.104	2.254	0.027

ENSG00000276045	ORAI1	-0.625	-1.235	-2.253	0.027
ENSG00000107317	PTGDS	-0.447	3.984	-2.252	0.027
ENSG00000227253	RP11-166N17.1	0.584	-1.328	2.252	0.027
ENSG00000249846	LINC02021	0.388	0.360	2.252	0.027
ENSG00000100483	VCPKMT	0.288	3.164	2.250	0.027
ENSG00000118939	UCHL3	0.320	1.076	2.250	0.027
ENSG00000172586	CHCHD1	0.274	2.623	2.249	0.027
ENSG00000231587	SNORD62B	0.683	-2.559	2.249	0.027
ENSG00000167863	ATP5H	0.358	5.431	2.249	0.027
ENSG00000265656	RP11-13N13.6	-0.893	-1.650	-2.248	0.027
ENSG00000077942	FBLN1	-0.441	2.558	-2.247	0.027
ENSG00000165626	BEND7	-0.231	3.180	-2.247	0.027
ENSG00000160606	TLCD1	-0.377	0.813	-2.245	0.027
ENSG00000183476	SH2D7	-0.659	-1.479	-2.244	0.027
ENSG00000069188	SDK2	-0.345	4.366	-2.243	0.028
ENSG00000151468	CCDC3	-0.209	5.306	-2.242	0.028
ENSG00000205790	DPP9-AS1	0.815	-2.395	2.242	0.028
ENSG00000242863	RN7SL677P	0.824	-2.623	2.241	0.028
ENSG00000165821	SALL2	-0.239	5.262	-2.241	0.028
ENSG00000213216	RP11-355O1.7	-0.476	-0.175	-2.240	0.028
ENSG00000177426	TGIF1	-0.409	1.515	-2.239	0.028
ENSG00000042445	RETSAT	-0.196	4.239	-2.238	0.028
ENSG00000239830	RPS4XP22	-0.917	0.287	-2.238	0.028
ENSG00000253661	ZFHX4-AS1	-0.418	1.078	-2.236	0.028
ENSG00000201025	SNORD74	-0.652	-2.035	-2.236	0.028
ENSG00000228408	RP1-111D6.3	-0.647	2.904	-2.235	0.028
ENSG00000107438	PDLIM1	-0.640	-0.029	-2.235	0.028
ENSG00000272195	RP11-156E8.1	0.646	-1.702	2.234	0.028
ENSG00000159450	TCHH	-0.473	0.590	-2.234	0.028
ENSG00000122477	LRRC39	0.259	3.489	2.233	0.028
ENSG00000149257	SERPINH1	-0.875	1.774	-2.233	0.028
ENSG00000201500	SNORD113	0.516	-0.520	2.233	0.028
ENSG00000270948	RP11-460N20.7	0.547	-0.624	2.232	0.028
ENSG00000237090	RP11-342M1.6	0.780	-1.279	2.230	0.028
ENSG00000214770	RP11-544I20.2	0.411	-0.380	2.230	0.028
ENSG00000249860	MTRNR2L5	-0.868	-2.475	-2.230	0.028
ENSG00000257748	LINC02281	0.753	-2.506	2.228	0.029
ENSG00000129250	KIF1C	-0.333	6.279	-2.228	0.029
ENSG00000162601	MYSM1	0.203	7.133	2.227	0.029
ENSG00000130038	CRACR2A	0.269	1.365	2.227	0.029
ENSG00000239821	RN7SL513P	0.849	-3.715	2.226	0.029
ENSG00000138814	PPP3CA	0.285	9.425	2.226	0.029
ENSG00000253710	ALG11	0.167	4.065	2.226	0.029
ENSG00000205927	OLIG2	-0.331	3.622	-2.225	0.029
ENSG00000184903	IMMP2L	0.198	2.867	2.225	0.029
ENSG00000241074	RN7SL813P	0.910	-3.308	2.225	0.029
ENSG00000136826	KLF4	-0.709	1.466	-2.225	0.029

ENSG00000254315	RP11-267M23.3	0.722	-2.513	2.223	0.029
ENSG00000230709	AC104024.1	0.874	-2.876	2.223	0.029
ENSG00000285676	#N/A	-0.356	-0.052	-2.222	0.029
ENSG00000255303	OR5BA1P	0.973	-2.786	2.222	0.029
ENSG00000239595	RN7SL79P	1.099	-1.844	2.221	0.029
ENSG00000214132	RP11-420O16.2	0.485	0.393	2.221	0.029
ENSG00000237989	LINC01679	-0.782	-1.145	-2.220	0.029
ENSG00000285622	#N/A	0.487	-1.105	2.220	0.029
ENSG00000152049	KCNE4	-0.625	1.602	-2.220	0.029
ENSG00000207245	SNORD116-29	0.481	0.128	2.220	0.029
ENSG00000284839	#N/A	0.756	-2.246	2.216	0.029
ENSG00000254363	CTB-131B5.5	0.601	0.657	2.214	0.030
ENSG00000261522	RP11-407P15.1	0.420	-0.101	2.213	0.030
ENSG00000066651	TRMT11	0.200	5.529	2.212	0.030
ENSG00000162385	MAGOH	0.230	2.806	2.211	0.030
ENSG00000110906	KCTD10	-0.143	5.145	-2.211	0.030
ENSG00000253873	PCDHGA11	-0.232	2.919	-2.211	0.030
ENSG00000063015	SEZ6	-0.343	4.419	-2.211	0.030
ENSG00000263753	LINC00667	0.203	5.167	2.210	0.030
ENSG00000158006	PAFAH2	-0.229	2.494	-2.209	0.030
ENSG00000120645	IQSEC3	-0.310	5.397	-2.209	0.030
ENSG00000237329	RP11-201O14.1	-0.657	-1.509	-2.209	0.030
ENSG00000119777	TMEM214	-0.263	3.590	-2.208	0.030
ENSG00000165209	STRBP	0.229	7.236	2.208	0.030
ENSG00000235050	MLIP-AS1	0.836	-2.531	2.208	0.030
ENSG00000152404	CWF19L2	0.176	5.698	2.206	0.030
ENSG00000140577	CRTC3	-0.213	4.783	-2.206	0.030
ENSG00000187848	P2RX2	1.164	-2.514	2.205	0.030
ENSG00000065970	FOXJ2	-0.148	4.345	-2.204	0.030
ENSG00000232087	HCFC2P1	0.895	-2.562	2.203	0.030
ENSG00000235837	AC073333.8	0.457	-0.805	2.203	0.030
ENSG00000054803	CBLN4	0.421	4.544	2.202	0.030
ENSG00000235665	LINC00298	0.385	0.094	2.202	0.030
ENSG00000251623	RP11-826N14.4	0.440	1.690	2.202	0.030
ENSG00000197183	NOL4L	-0.168	5.598	-2.202	0.030
ENSG00000141385	AFG3L2	0.219	5.901	2.199	0.031
ENSG00000144290	SLC4A10	0.266	8.544	2.199	0.031
ENSG00000145423	SFRP2	-0.440	1.767	-2.198	0.031
ENSG00000113240	CLK4	0.203	6.156	2.197	0.031
ENSG00000270640	RP11-373D23.2	-0.734	-1.283	-2.197	0.031
ENSG00000259649	RP11-351M8.1	-0.550	-1.509	-2.197	0.031
ENSG00000259072	RP11-96D24.1	0.486	-0.052	2.195	0.031
ENSG00000185022	MAFF	-0.601	1.603	-2.195	0.031
ENSG00000229124	VIM-AS1	-0.788	-2.634	-2.195	0.031
ENSG00000196503	ARL9	0.485	0.293	2.193	0.031
ENSG00000250366	TUNAR	0.313	5.154	2.192	0.031
ENSG00000180773	SLC36A4	0.237	5.550	2.192	0.031

ENSG00000177238	TRIM72	0.441	0.496	2.191	0.031
ENSG00000270919	RP11-379K22.3	0.454	-0.570	2.191	0.031
ENSG00000213462	ERV3-1	-0.268	4.125	-2.191	0.031
ENSG00000235618	FAM21EP	-0.270	1.012	-2.190	0.031
ENSG00000256235	SMIM3	-0.432	1.217	-2.190	0.031
ENSG00000166946	CCNDBP1	0.237	5.260	2.190	0.031
ENSG00000145384	FABP2	0.803	-2.054	2.189	0.031
ENSG00000260793	RP5-882C2.2	0.339	0.577	2.188	0.032
ENSG00000264304	RP11-20B24.7	0.711	-1.589	2.188	0.032
ENSG00000166340	TPP1	-0.313	4.928	-2.187	0.032
ENSG00000166398	KIAA0355	-0.146	4.856	-2.185	0.032
ENSG00000200204	RNU1-22P	0.779	-4.737	2.185	0.032
ENSG00000250909	RP11-843P14.2	-0.556	-1.863	-2.184	0.032
ENSG00000188786	MTF1	-0.175	4.241	-2.184	0.032
ENSG00000101335	MYL9	-0.770	1.664	-2.183	0.032
ENSG00000108679	LGALS3BP	-0.398	3.518	-2.182	0.032
ENSG00000184384	MAML2	-0.225	4.622	-2.182	0.032
ENSG00000103647	CORO2B	-0.151	6.559	-2.182	0.032
ENSG00000113739	STC2	-0.742	-0.773	-2.181	0.032
ENSG00000127241	MASP1	-0.320	2.781	-2.180	0.032
ENSG00000236404	VLDLR-AS1	0.240	3.074	2.180	0.032
ENSG00000161132	XXbac-B444P24.1	-0.453	-0.780	-2.179	0.032
ENSG00000196517	SLC6A9	-0.380	2.336	-2.178	0.032
ENSG00000282375	RP11-43A14.2	0.332	1.052	2.178	0.032
ENSG00000254862	RP11-159H22.2	0.441	-1.105	2.178	0.032
ENSG00000108175	ZMIZ1	-0.238	6.124	-2.177	0.032
ENSG00000248927	CTD-2334D19.1	0.396	1.130	2.177	0.032
ENSG00000228554	AC004837.5	0.452	-0.414	2.177	0.032
ENSG00000167306	MYO5B	0.250	4.489	2.176	0.032
ENSG00000176532	PRR15	-0.619	-1.599	-2.176	0.032
ENSG00000188536	HBA2	-0.879	0.944	-2.175	0.032
ENSG00000146535	GNA12	-0.261	4.890	-2.175	0.032
ENSG00000134830	C5AR2	-0.428	0.620	-2.174	0.033
ENSG00000267577	CTD-2587H24.5	0.520	0.123	2.173	0.033
ENSG00000143816	WNT9A	-0.399	0.523	-2.172	0.033
ENSG00000280162	RP11-314N13.9	-0.548	-0.329	-2.171	0.033
ENSG00000137364	TPMT	0.222	4.947	2.171	0.033
ENSG00000139726	DENR	0.272	6.179	2.171	0.033
ENSG00000240997	RN7SL183P	1.121	-2.360	2.171	0.033
ENSG00000260351	RP11-382A20.6	0.748	-2.364	2.170	0.033
ENSG00000186088	GSAP	0.223	4.287	2.170	0.033
ENSG00000113263	ITK	-0.663	-0.719	-2.170	0.033
ENSG00000070831	CDC42	0.289	5.338	2.169	0.033
ENSG00000153234	NR4A2	-0.609	4.580	-2.169	0.033
ENSG00000201300	SNORD115-27	0.507	-1.140	2.169	0.033
ENSG00000259709	CTD-2184D3.7	0.485	0.704	2.166	0.033
ENSG00000248213	CICP16	-0.620	-1.661	-2.165	0.033

ENSG00000260422	RP1-155D22.2	-0.816	-2.403	-2.165	0.033
ENSG00000255968	RP11-513G19.1	-0.393	0.456	-2.164	0.033
ENSG00000167191	GPRC5B	-0.291	7.293	-2.164	0.033
ENSG00000144362	PHOSPHO2	0.229	1.536	2.164	0.033
ENSG00000269427	CTC-429P9.1	0.267	0.807	2.163	0.033
ENSG00000264916	RN7SL230P	0.893	-1.348	2.163	0.033
ENSG00000175130	MARCKSL1	-0.320	5.206	-2.163	0.033
ENSG00000224751	SHMT1P1	0.463	-0.858	2.162	0.034
ENSG00000162599	NFIA	-0.241	7.515	-2.161	0.034
ENSG00000230552	AC092162.1	0.440	0.324	2.161	0.034
ENSG00000071967	CYBRD1	-0.401	5.752	-2.161	0.034
ENSG00000101144	BMP7	-0.275	4.543	-2.160	0.034
ENSG00000260464	RP4-561L24.3	0.223	2.516	2.159	0.034
ENSG00000249188	ENPP7P1	-0.494	-0.280	-2.156	0.034
ENSG00000150457	LATS2	-0.320	1.989	-2.154	0.034
ENSG00000260832	CTD-3253I12.1	0.459	0.380	2.153	0.034
ENSG00000104419	NDRG1	-0.344	6.146	-2.152	0.034
ENSG00000186951	PPARA	-0.271	5.113	-2.152	0.034
ENSG00000212283	SNORD89	0.478	1.365	2.151	0.034
ENSG00000168264	IRF2BP2	-0.156	6.016	-2.150	0.034
ENSG00000128059	PPAT	0.236	4.498	2.150	0.035
ENSG00000129009	ISLR	-0.532	3.011	-2.149	0.035
ENSG00000247416	RP11-629G13.1	-0.360	0.032	-2.149	0.035
ENSG00000256971	LINC00508	0.653	-0.304	2.149	0.035
ENSG00000011260	UTP18	0.275	4.572	2.148	0.035
ENSG00000243339	RN7SL738P	0.838	-3.220	2.148	0.035
ENSG00000230896	RP11-767N6.7	-0.345	0.389	-2.147	0.035
ENSG00000264653	MIR5194	0.696	-2.122	2.147	0.035
ENSG00000253525	CTD-2114J12.1	-1.108	-0.989	-2.146	0.035
ENSG00000230124	ACBD6	0.154	4.674	2.146	0.035
ENSG00000166479	TMX3	0.238	6.967	2.146	0.035
ENSG00000150401	DCUN1D2	0.161	4.329	2.145	0.035
ENSG00000118263	KLF7	-0.140	6.203	-2.145	0.035
ENSG00000085840	ORC1	-0.537	-1.376	-2.142	0.035
ENSG00000241461	RN7SL182P	0.752	-1.390	2.141	0.035
ENSG00000109066	TMEM104	-0.299	2.611	-2.141	0.035
ENSG00000166788	SAAL1	0.202	3.022	2.141	0.035
ENSG00000250584	LINC01511	0.588	0.620	2.141	0.035
ENSG00000285399	#N/A	0.254	2.424	2.138	0.036
ENSG00000153446	C16orf89	-0.380	2.513	-2.137	0.036
ENSG00000223893	GNL2P1	0.525	-0.592	2.136	0.036
ENSG00000104231	ZFAND1	0.244	5.137	2.136	0.036
ENSG00000233912	AC026202.3	-0.422	-1.110	-2.134	0.036
ENSG00000163673	DCLK3	0.376	3.091	2.134	0.036
ENSG00000145920	CPLX2	0.178	9.154	2.134	0.036
ENSG00000100314	CABP7	-0.365	1.713	-2.133	0.036
ENSG00000206145	P2RX6P	0.662	-1.125	2.133	0.036

ENSG00000241644	INMT	-0.948	-1.583	-2.133	0.036
ENSG00000235725	AC007389.3	-0.550	-1.618	-2.133	0.036
ENSG00000272536	RN7SL840P	0.798	-3.143	2.132	0.036
ENSG00000110696	C11orf58	0.223	7.206	2.132	0.036
ENSG00000272081	CTD-2376I4.2	-0.761	-2.108	-2.131	0.036
ENSG00000125611	CHCHD5	-0.446	0.348	-2.131	0.036
ENSG00000267506	RP11-13K12.1	-0.420	3.398	-2.131	0.036
ENSG00000118418	HMGN3	0.229	4.492	2.130	0.036
ENSG00000175352	NRIP3	0.290	6.015	2.129	0.036
ENSG00000078328	RBFOX1	0.219	8.062	2.128	0.036
ENSG00000261292	RP11-389G6.3	0.421	4.464	2.128	0.036
ENSG00000224715	CITF22-49D8.1	0.761	-1.800	2.128	0.036
ENSG00000228506	RP11-98I9.4	-0.270	1.150	-2.128	0.036
ENSG00000272812	RP5-855D21.3	0.378	0.397	2.128	0.036
ENSG00000135318	NT5E	-0.312	4.600	-2.127	0.036
ENSG00000101247	NDUFAF5	0.222	5.150	2.127	0.036
ENSG00000134042	MRO	-0.302	6.222	-2.127	0.036
ENSG00000243103	RN7SL452P	0.588	-2.365	2.126	0.036
ENSG00000024422	EHD2	-0.374	2.663	-2.126	0.036
ENSG00000196189	SEMA4A	-0.277	4.377	-2.124	0.037
ENSG00000241420	RN7SL505P	0.867	-1.151	2.124	0.037
ENSG00000187772	LIN28B	0.311	1.326	2.122	0.037
ENSG00000100181	TPTEP1	-0.398	2.179	-2.122	0.037
ENSG00000183783	KCTD8	0.311	3.642	2.121	0.037
ENSG00000007866	TEAD3	-0.540	0.210	-2.121	0.037
ENSG00000120251	GRIA2	0.211	10.224	2.120	0.037
ENSG00000197077	KIAA1671	-0.154	6.647	-2.119	0.037
ENSG00000236062	GSTM5P1	-0.570	-0.455	-2.119	0.037
ENSG00000277452	RN7SL473P	0.654	-1.089	2.119	0.037
ENSG00000104760	FGL1	0.551	0.566	2.118	0.037
ENSG00000265204	RP11-403A21.3	0.598	-1.206	2.117	0.037
ENSG00000261087	KB-1460A1.5	-0.220	5.019	-2.117	0.037
ENSG00000165323	FAT3	0.217	9.530	2.117	0.037
ENSG00000137821	LRRC49	0.196	5.735	2.116	0.037
ENSG00000231296	RP1-39G22.4	0.579	-1.922	2.116	0.037
ENSG00000240317	RN7SL764P	0.768	-2.843	2.116	0.037
ENSG00000250116	RP11-417F21.1	-0.422	0.848	-2.115	0.037
ENSG00000141505	ASGR1	0.466	0.511	2.114	0.037
ENSG00000188037	CLCN1	-0.766	-2.551	-2.114	0.037
ENSG00000231548	OR55B1P	-0.626	-1.316	-2.114	0.038
ENSG00000234459	AC002064.4	0.376	0.408	2.114	0.038
ENSG00000118513	MYB	0.439	0.630	2.114	0.038
ENSG00000275993	CH507-42P11.8	-0.649	1.121	-2.114	0.038
ENSG00000237356	AL163953.3	0.403	-0.621	2.114	0.038
ENSG00000133392	MYH11	-0.891	3.855	-2.113	0.038
ENSG00000267285	RP5-951N9.2	0.578	-1.318	2.113	0.038
ENSG00000196352	CD55	-0.293	4.249	-2.113	0.038

ENSG00000236682	AC068282.3	-0.519	0.613	-2.112	0.038
ENSG00000226254	PTP4A1P3	0.744	-0.597	2.112	0.038
ENSG00000084072	PPIE	0.182	4.064	2.111	0.038
ENSG00000185742	C11orf87	0.261	7.387	2.111	0.038
ENSG00000257754	RP11-650K20.2	-0.782	-2.009	-2.110	0.038
ENSG00000136936	XPA	0.178	4.496	2.110	0.038
ENSG00000196236	XPNPEP3	-0.137	4.644	-2.109	0.038
ENSG00000176720	BOK	-0.481	2.737	-2.109	0.038
ENSG00000204789	ZNF204P	0.288	6.356	2.107	0.038
ENSG00000248138	RP11-446J8.1	-0.460	-1.517	-2.107	0.038
ENSG00000135698	MPHOSPH6	0.210	4.791	2.107	0.038
ENSG00000196350	ZNF729	-0.937	-2.196	-2.106	0.038
ENSG00000136100	VPS36	0.197	6.567	2.105	0.038
ENSG00000116030	SUMO1	0.323	5.857	2.104	0.038
ENSG00000107338	SHB	-0.409	1.142	-2.104	0.038
ENSG00000235823	OLMALINC	-0.345	4.141	-2.104	0.038
ENSG00000182158	CREB3L2	-0.230	3.929	-2.102	0.039
ENSG00000266897	AC005546.2	0.856	-2.612	2.101	0.039
ENSG00000244091	RN7SL483P	0.949	-3.283	2.101	0.039
ENSG00000143384	MCL1	-0.263	6.302	-2.101	0.039
ENSG00000228280	RP11-367B6.2	0.550	-1.761	2.100	0.039
ENSG00000275839	CTC-421K24.1	0.497	-0.828	2.100	0.039
ENSG00000142798	HSPG2	-0.608	1.582	-2.100	0.039
ENSG00000077585	GPR137B	-0.249	4.620	-2.100	0.039
ENSG00000103489	XYLT1	-0.182	6.354	-2.100	0.039
ENSG00000197713	RPE	0.194	5.677	2.099	0.039
ENSG00000109536	FRG1	0.271	3.958	2.098	0.039
ENSG00000274996	CTB-58E17.2	0.461	-1.198	2.098	0.039
ENSG00000104164	BLOC1S6	0.266	6.992	2.097	0.039
ENSG00000267219	AC010504.2	0.354	-0.377	2.096	0.039
ENSG00000172020	GAP43	0.281	8.339	2.096	0.039
ENSG00000008324	SS18L2	0.270	2.369	2.096	0.039
ENSG00000244568	RN7SL654P	0.737	-3.733	2.096	0.039
ENSG00000279766	RP11-642A1.2	-0.477	-0.872	-2.095	0.039
ENSG00000199683	RN7SKP185	0.586	0.292	2.095	0.039
ENSG00000112561	TFEB	-0.435	1.774	-2.094	0.039
ENSG00000107819	SFXN3	-0.141	5.568	-2.094	0.039
ENSG00000122012	SV2C	0.417	4.611	2.094	0.039
ENSG00000099901	RANBP1	0.285	4.045	2.093	0.039
ENSG00000279375	CH17-140K24.6	0.466	0.742	2.093	0.039
ENSG00000197568	HHLA3	-0.372	1.216	-2.093	0.039
ENSG00000214429	CYCSP6	0.786	-2.433	2.093	0.039
ENSG00000159147	DONSON	0.202	3.019	2.092	0.039
ENSG00000085872	CHERP	-0.348	2.949	-2.092	0.040
ENSG00000236137	RP11-27K13.3	0.584	-1.473	2.090	0.040
ENSG00000285940	#N/A	0.273	1.923	2.089	0.040
ENSG00000166501	PRKCB	0.214	9.851	2.089	0.040

ENSG00000247925	RP3-510L9.1	-0.611	-2.064	-2.089	0.040
ENSG00000187678	SPRY4	-0.289	4.681	-2.089	0.040
ENSG00000252906	SCARNA3	0.788	-2.410	2.087	0.040
ENSG00000079308	TNS1	-0.323	5.568	-2.087	0.040
ENSG00000265136	RP13-638C3.6	0.608	-1.145	2.086	0.040
ENSG00000271670	RP11-95I16.4	0.481	-1.064	2.086	0.040
ENSG00000254730	RP11-801G16.2	0.744	-3.002	2.086	0.040
ENSG00000123119	NECAB1	0.353	8.395	2.086	0.040
ENSG00000091986	CCDC80	-0.265	3.535	-2.085	0.040
ENSG00000240694	PNMA2	0.202	9.124	2.084	0.040
ENSG00000231859	AC007875.2	0.607	-1.396	2.084	0.040
ENSG00000157680	DGKI	0.197	7.937	2.084	0.040
ENSG00000138642	HERC6	0.241	4.873	2.084	0.040
ENSG00000155254	MARVELD1	-0.356	1.417	-2.084	0.040
ENSG00000267534	S1PR2	-0.364	0.435	-2.083	0.040
ENSG00000272515	RP11-29P20.1	0.316	1.581	2.082	0.040
ENSG00000159792	PSKH1	-0.253	1.890	-2.082	0.040
ENSG00000184900	SUMO3	0.285	5.617	2.081	0.040
ENSG00000183067	IGSF5	-0.371	-0.127	-2.081	0.041
ENSG00000081818	PCDHB4	-0.286	3.749	-2.081	0.041
ENSG00000264322	RN7SL448P	1.396	-0.718	2.081	0.041
ENSG00000280183	RP11-400F19.12	0.423	-0.717	2.080	0.041
ENSG00000033327	GAB2	-0.278	6.520	-2.080	0.041
ENSG00000253910	PCDHGB2	-0.242	3.345	-2.079	0.041
ENSG00000008128	CDK11A	-0.392	-0.037	-2.079	0.041
ENSG00000135547	HEY2	-0.301	2.886	-2.077	0.041
ENSG00000099194	SCD	-0.316	9.385	-2.077	0.041
ENSG00000187079	TEAD1	-0.198	5.812	-2.077	0.041
ENSG00000273456	RP11-686O6.2	0.319	0.441	2.076	0.041
ENSG00000154240	CEP112	0.179	5.068	2.076	0.041
ENSG00000168081	PNOC	0.548	0.821	2.076	0.041
ENSG00000100647	SUSD6	-0.186	4.868	-2.076	0.041
ENSG00000168724	DNAJC21	0.186	6.461	2.075	0.041
ENSG00000106993	CDC37L1	0.256	5.732	2.075	0.041
ENSG00000177283	FZD8	-0.355	2.105	-2.075	0.041
ENSG00000198944	SOWAHA	0.287	5.783	2.075	0.041
ENSG00000116793	PHTF1	0.168	5.640	2.075	0.041
ENSG00000271947	RP11-439M11.1	0.525	-0.651	2.074	0.041
ENSG00000228358	LINC02263	0.585	0.679	2.074	0.041
ENSG00000177807	KCNJ10	-0.312	6.962	-2.074	0.041
ENSG00000261420	RP1-168L15.5	0.288	0.763	2.074	0.041
ENSG00000156687	UNC5D	0.213	6.633	2.073	0.041
ENSG00000155980	KIF5A	-0.208	10.332	-2.072	0.041
ENSG00000197885	NKIRAS1	0.251	5.852	2.072	0.041
ENSG00000086065	CHMP5	0.358	5.819	2.071	0.041
ENSG00000224271	RP11-191L9.4	0.450	1.229	2.070	0.042
ENSG00000163377	FAM19A4	0.361	0.876	2.069	0.042

ENSG00000100439	ABHD4	-0.304	2.864	-2.069	0.042
ENSG00000134874	DZIP1	0.137	6.928	2.069	0.042
ENSG00000134531	EMP1	-0.586	2.979	-2.069	0.042
ENSG00000273154	RP4-583P15.15	0.627	-2.176	2.069	0.042
ENSG00000082068	WDR70	0.140	4.753	2.067	0.042
ENSG00000231561	CEACAMP5	0.389	-0.852	2.067	0.042
ENSG00000103035	PSMD7	0.213	5.865	2.067	0.042
ENSG00000203307	RP11-375H19.2	0.544	-1.165	2.067	0.042
ENSG00000106078	COBL	-0.147	7.135	-2.067	0.042
ENSG00000213484	EIF4A1P8	-0.387	-0.588	-2.066	0.042
ENSG00000201229	SNORA63	0.814	-1.723	2.065	0.042
ENSG00000008277	ADAM22	0.226	8.392	2.065	0.042
ENSG00000174099	MSRB3	-0.235	4.468	-2.065	0.042
ENSG00000228793	RP1-223B1.1	-1.089	-1.456	-2.065	0.042
ENSG00000270728	RP4-657E11.10	-0.269	0.943	-2.064	0.042
ENSG00000067141	NEO1	-0.153	6.666	-2.064	0.042
ENSG00000162409	PRKAA2	0.235	6.928	2.064	0.042
ENSG00000283486	RP11-392E22.9	-0.447	0.825	-2.064	0.042
ENSG00000152779	SLC16A12	-0.842	-1.326	-2.063	0.042
ENSG00000174945	AMZ1	-0.292	3.551	-2.063	0.042
ENSG00000263989	RN7SL615P	0.562	-1.374	2.062	0.042
ENSG00000151136	BTBD11	0.354	4.104	2.062	0.042
ENSG00000119508	NR4A3	-0.433	4.214	-2.062	0.042
ENSG00000265327	RN7SL411P	0.813	-0.279	2.061	0.042
ENSG00000140497	SCAMP2	-0.313	2.930	-2.061	0.042
ENSG00000236892	RP11-482E14.2	0.531	-0.219	2.061	0.042
ENSG00000231858	AC067945.4	0.502	-0.475	2.061	0.042
ENSG00000260269	CTD-2323K18.1	0.547	-1.762	2.061	0.042
ENSG00000116754	SRSF11	0.149	7.512	2.061	0.042
ENSG00000070501	POLB	0.225	3.863	2.061	0.042
ENSG00000261220	RP11-629O1.2	0.469	-0.145	2.060	0.042
ENSG00000169075	RP3-509L4.3	-0.323	1.298	-2.060	0.043
ENSG00000156463	SH3RF2	0.361	1.576	2.059	0.043
ENSG00000234996	RP11-480I12.9	-0.482	-0.440	-2.059	0.043
ENSG00000125505	MBOAT7	-0.313	5.294	-2.058	0.043
ENSG00000185591	SP1	-0.195	5.536	-2.058	0.043
ENSG00000275025	RP5-875H18.10	0.449	-1.241	2.058	0.043
ENSG00000095932	SMIM24	0.632	0.561	2.058	0.043
ENSG00000199872	RNU6-942P	0.648	-1.965	2.058	0.043
ENSG00000152192	POU4F1	-0.635	-1.649	-2.058	0.043
ENSG00000110169	HPX	0.605	-1.045	2.057	0.043
ENSG00000080007	DDX43	0.771	-1.257	2.057	0.043
ENSG00000234509	AP000253.1	-0.523	-1.282	-2.056	0.043
ENSG00000156970	BUB1B	0.431	-0.171	2.056	0.043
ENSG00000126581	BECN1	0.218	5.551	2.056	0.043
ENSG00000115271	GCA	0.261	4.834	2.056	0.043
ENSG00000137955	RABGGTB	0.191	5.935	2.055	0.043

ENSG00000144230	GPR17	-0.424	2.793	-2.055	0.043
ENSG00000135930	EIF4E2	0.165	5.670	2.054	0.043
ENSG00000181126	HLA-V	0.552	-0.938	2.054	0.043
ENSG00000180730	SHISA2	-0.362	0.654	-2.053	0.043
ENSG00000149100	EIF3M	0.225	5.852	2.053	0.043
ENSG00000104140	RHOV	0.386	0.646	2.053	0.043
ENSG00000163964	PIGX	0.229	5.285	2.053	0.043
ENSG00000227006	RP5-956O18.2	-0.346	0.019	-2.052	0.043
ENSG00000272885	RP11-2H3.6	0.421	-0.232	2.052	0.043
ENSG00000269399	CTD-3222D19.12	0.264	0.516	2.052	0.043
ENSG00000256124	LINC01152	-0.294	0.973	-2.051	0.043
ENSG00000107957	SH3PXD2A	-0.198	6.987	-2.051	0.043
ENSG00000266904	CTC-559E9.1	-0.212	2.133	-2.051	0.043
ENSG00000240936	RN7SL554P	0.663	-3.501	2.050	0.044
ENSG00000249042	CTD-2015H6.3	0.215	3.653	2.050	0.044
ENSG00000142233	NTN5	-0.641	-1.732	-2.050	0.044
ENSG00000248766	RP11-61K20.1	0.920	-2.968	2.049	0.044
ENSG00000232542	DPYD-IT1	0.576	-1.548	2.049	0.044
ENSG00000130147	SH3BP4	-0.298	3.425	-2.049	0.044
ENSG00000222009	BTBD19	-0.391	1.024	-2.049	0.044
ENSG00000254636	ARMS2	0.690	-2.101	2.048	0.044
ENSG00000207322	RNU1-89P	0.912	-2.098	2.048	0.044
ENSG00000180573	HIST1H2AC	-0.248	4.398	-2.048	0.044
ENSG00000198408	MGEA5	0.147	8.855	2.048	0.044
ENSG00000164741	DLC1	-0.172	6.590	-2.047	0.044
ENSG00000267610	AC007787.2	0.718	-1.967	2.047	0.044
ENSG00000223668	EEF1A1P24	-0.521	-1.212	-2.047	0.044
ENSG00000252213	SNORA74	0.854	-2.373	2.046	0.044
ENSG00000165637	VDAC2	0.267	4.911	2.046	0.044
ENSG00000156639	ZFAND3	-0.172	4.903	-2.045	0.044
ENSG00000235286	AP000925.2	-0.429	-0.319	-2.045	0.044
ENSG00000165474	GJB2	-0.614	-0.287	-2.045	0.044
ENSG00000033867	SLC4A7	0.281	7.133	2.044	0.044
ENSG00000101265	RASSF2	-0.349	6.782	-2.044	0.044
ENSG00000124134	KCNS1	0.404	4.896	2.044	0.044
ENSG00000253159	PCDHGA12	-0.314	3.661	-2.043	0.044
ENSG00000246596	RP11-1277A3.2	-0.239	3.427	-2.043	0.044
ENSG00000276754	RP11-461A8.5	0.617	-2.137	2.042	0.044
ENSG00000239211	RN7SL563P	0.836	-3.953	2.042	0.044
ENSG00000116983	HPCAL4	0.220	8.824	2.041	0.044
ENSG00000173511	VEGFB	-0.273	4.158	-2.041	0.044
ENSG00000127252	HRASLS	0.204	2.769	2.041	0.044
ENSG00000092208	GEMIN2	0.243	2.788	2.041	0.044
ENSG00000143033	MTF2	0.166	5.531	2.039	0.045
ENSG00000162458	FBLIM1	-0.572	-0.258	-2.039	0.045
ENSG00000180340	FZD2	-0.836	-2.374	-2.037	0.045
ENSG00000109099	PMP22	-0.307	5.267	-2.037	0.045

ENSG00000156253	RWDD2B	0.312	4.005	2.037	0.045
ENSG00000083814	ZNF671	0.154	3.854	2.036	0.045
ENSG00000164167	LSM6	0.253	3.092	2.036	0.045
ENSG00000138867	GUCD1	-0.232	4.050	-2.036	0.045
ENSG00000285898	#N/A	0.540	-0.812	2.035	0.045
ENSG00000163046	ANKRD30BL	0.356	3.726	2.035	0.045
ENSG00000126878	AIF1L	-0.371	5.582	-2.035	0.045
ENSG00000241219	RP11-572M11.1	0.497	-1.126	2.035	0.045
ENSG00000228020	HNRNPA1P46	0.696	-0.809	2.034	0.045
ENSG00000254863	RP11-831A10.2	0.829	-2.347	2.034	0.045
ENSG00000149599	DUSP15	-0.341	-0.214	-2.033	0.045
ENSG00000185201	IFITM2	-0.622	0.844	-2.032	0.045
ENSG00000256609	RP11-64B16.4	-0.797	-1.036	-2.032	0.045
ENSG00000136267	DGKB	0.275	7.917	2.032	0.045
ENSG00000247121	CTD-2260A17.2	-0.181	2.810	-2.032	0.045
ENSG00000227719	AC006042.6	0.383	0.703	2.031	0.045
ENSG00000103512	NOMO1	-0.168	5.963	-2.031	0.046
ENSG00000135414	GDF11	-0.175	5.301	-2.030	0.046
ENSG00000168028	RPSA	0.425	5.321	2.030	0.046
ENSG00000242659	RP11-271C24.2	0.535	-0.760	2.029	0.046
ENSG00000174567	GOLT1A	0.504	-1.378	2.028	0.046
ENSG00000225364	ATP6V0E1P1	-0.422	-1.094	-2.028	0.046
ENSG00000188674	C2orf80	0.333	5.191	2.028	0.046
ENSG00000141756	FKBP10	-0.316	2.673	-2.028	0.046
ENSG00000200949	SNORD115-32	0.655	2.066	2.028	0.046
ENSG00000057757	PITHD1	0.226	6.415	2.028	0.046
ENSG00000137266	SLC22A23	-0.170	4.859	-2.027	0.046
ENSG00000228513	AC023271.1	-0.663	-2.046	-2.027	0.046
ENSG00000167178	ISLR2	-0.437	3.817	-2.027	0.046
ENSG00000233107	SUCLA2P2	0.637	-1.211	2.025	0.046
ENSG00000244699	RP11-803B1.2	-0.723	-2.131	-2.025	0.046
ENSG00000117586	TNFSF4	-0.220	2.116	-2.025	0.046
ENSG00000081277	PKP1	-0.482	0.077	-2.025	0.046
ENSG00000264978	RN7SL630P	0.675	-2.382	2.025	0.046
ENSG00000228133	AC099684.1	0.367	0.651	2.024	0.046
ENSG00000165478	HEPACAM	-0.255	5.740	-2.023	0.046
ENSG00000280850	RP4-655C5.9	-0.622	-1.069	-2.023	0.046
ENSG00000112096	SOD2	0.301	7.772	2.022	0.046
ENSG00000006210	CX3CL1	-0.254	5.735	-2.021	0.047
ENSG00000159658	EFCAB14	-0.164	6.967	-2.020	0.047
ENSG00000236296	GUSBP5	0.448	-0.050	2.020	0.047
ENSG00000125285	SOX21	-0.307	2.440	-2.020	0.047
ENSG00000204060	FOXO6	-0.592	-0.183	-2.020	0.047
ENSG00000107882	SUFU	-0.215	3.510	-2.019	0.047
ENSG00000283891	MIR628	0.542	-1.314	2.019	0.047
ENSG00000179954	SSC5D	-0.373	1.159	-2.019	0.047
ENSG00000078804	TP53INP2	-0.401	5.679	-2.019	0.047

ENSG00000267466	RP11-13K12.5	-0.353	2.575	-2.018	0.047
ENSG00000252034	RNY4P37	0.697	-1.615	2.017	0.047
ENSG00000206530	CFAP44	0.188	5.087	2.017	0.047
ENSG00000238741	SCARNA7	0.407	5.301	2.017	0.047
ENSG00000136682	CBWD2	0.183	4.465	2.017	0.047
ENSG00000164120	HPGD	0.643	0.019	2.016	0.047
ENSG00000198435	NRARP	-0.348	0.905	-2.015	0.047
ENSG00000252759	Y_RNA	-0.615	-1.161	-2.014	0.047
ENSG00000065320	NTN1	-0.304	1.908	-2.013	0.047
ENSG00000258856	CTD-2325K12.1	-0.568	-1.656	-2.013	0.047
ENSG00000201607	RNU4-16P	-0.580	-2.553	-2.012	0.047
ENSG00000006625	GGCT	0.351	4.104	2.012	0.048
ENSG00000254285	KRT8P3	-0.798	-2.253	-2.012	0.048
ENSG00000157212	PAXIP1	0.147	3.654	2.011	0.048
ENSG00000237930	AC007563.4	-0.546	-0.888	-2.011	0.048
ENSG00000201036	SNORD113	0.443	-0.054	2.011	0.048
ENSG00000136444	RSAD1	0.151	4.098	2.011	0.048
ENSG00000125637	PSD4	-0.247	3.032	-2.010	0.048
ENSG00000253485	PCDHGA5	-0.224	2.669	-2.010	0.048
ENSG00000167711	SERPINF2	-0.750	-1.927	-2.010	0.048
ENSG00000184232	OAF	-0.371	3.587	-2.010	0.048
ENSG00000127946	HIP1	-0.250	5.121	-2.009	0.048
ENSG00000244026	FAM86DP	-0.304	2.570	-2.009	0.048
ENSG00000259359	RP11-327J17.2	-0.646	-1.971	-2.009	0.048
ENSG00000143318	CASQ1	0.367	3.776	2.008	0.048
ENSG00000212588	SNORA26	0.488	3.158	2.008	0.048
ENSG00000224389	C4B	-0.844	1.590	-2.008	0.048
ENSG00000139112	GABARAPL1	0.259	8.333	2.007	0.048
ENSG00000198673	FAM19A2	0.332	6.361	2.007	0.048
ENSG00000268182	SMIM17	0.313	2.832	2.007	0.048
ENSG00000257609	RP11-275O18.1	-0.902	-2.863	-2.007	0.048
ENSG00000137936	BCAR3	-0.209	3.480	-2.007	0.048
ENSG00000186815	TPCN1	-0.235	4.895	-2.006	0.048
ENSG00000143061	IGSF3	0.213	4.668	2.006	0.048
ENSG00000107902	LHPP	-0.316	3.561	-2.006	0.048
ENSG00000082397	EPB41L3	0.196	8.355	2.006	0.048
ENSG00000259863	SH3RF3-AS1	-0.406	-0.260	-2.005	0.048
ENSG00000179403	VWA1	-0.500	2.627	-2.005	0.048
ENSG00000170275	CRTAP	-0.158	6.042	-2.004	0.048
ENSG00000133114	GPALPP1	0.194	6.296	2.004	0.048
ENSG00000249460	LINC02500	0.692	-2.089	2.004	0.048
ENSG00000157570	TSPAN18	-0.356	2.015	-2.004	0.048
ENSG00000252351	RNU6ATAC12P	-0.697	-2.352	-2.004	0.048
ENSG00000105793	GTPBP10	0.262	5.670	2.003	0.048
ENSG00000225746	SNHG23	0.241	6.118	2.003	0.048
ENSG00000135929	CYP27A1	-0.383	2.784	-2.003	0.049
ENSG00000229668	RP11-317F20.2	-0.466	-1.143	-2.002	0.049

ENSG00000227606	AC005392.13	0.444	-1.158	2.002	0.049
ENSG00000269952	RP11-324I22.3	-0.604	-1.363	-2.001	0.049
ENSG00000229806	RPS15P5	-0.646	-2.030	-2.000	0.049
ENSG00000155974	GRIP1	0.185	5.048	2.000	0.049
ENSG00000070404	FSTL3	-0.332	1.351	-2.000	0.049
ENSG00000173546	CSPG4	-0.408	3.148	-2.000	0.049
ENSG00000273123	RP11-9N20.3	-0.617	-2.055	-2.000	0.049
ENSG00000080224	EPHA6	0.249	6.071	1.999	0.049
ENSG00000239129	snoU13	0.632	-2.435	1.998	0.049
ENSG00000121410	A1BG	-0.455	-0.219	-1.998	0.049
ENSG00000136802	LRRC8A	-0.282	6.165	-1.997	0.049
ENSG00000225394	AC106883.1	0.818	-2.032	1.996	0.049
ENSG00000101346	POFUT1	-0.152	4.177	-1.996	0.049
ENSG00000240775	RP11-170N16.2	-0.603	-1.680	-1.996	0.049
ENSG00000171530	TBCA	0.319	4.339	1.995	0.049
ENSG00000148730	EIF4EBP2	-0.180	7.122	-1.995	0.049
ENSG00000260460	RP11-284F21.8	0.424	-0.398	1.995	0.049
ENSG00000248727	LINC01948	0.774	-3.199	1.995	0.049
ENSG00000254319	RP11-134O21.1	0.484	-0.752	1.994	0.049
ENSG00000280129	RP11-298I3.3	-0.521	-0.984	-1.994	0.049
ENSG00000253642	RP11-317N12.1	-0.645	-1.518	-1.994	0.049
ENSG00000263750	MIR548AV	0.704	-2.548	1.994	0.049
ENSG00000243508	RP11-775J23.2	0.415	0.735	1.994	0.049
ENSG00000280276	RP11-314C9.1	0.583	-0.445	1.994	0.049
ENSG00000178878	APOLD1	-0.607	5.112	-1.993	0.050
ENSG00000250670	AC004063.1	0.654	-2.377	1.993	0.050
ENSG00000116132	PRRX1	-0.238	4.158	-1.992	0.050
ENSG00000123240	OPTN	0.200	8.000	1.992	0.050
ENSG00000274124	RP11-474C8.8	0.741	-2.755	1.992	0.050
ENSG00000111752	PHC1	-0.203	3.981	-1.992	0.050
ENSG00000244514	RN7SL125P	0.834	-2.990	1.991	0.050

OFC

gene_id	gene_name	logFC	AveExpr	t	P.Value
ENSG00000251620	STPG2-AS1	-0.969	-2.373	-3.776	0.000
ENSG00000272645	GTF2IP20	-0.520	2.044	-3.776	0.000
ENSG00000137101	CD72	-0.777	-0.970	-3.673	0.000
ENSG00000238086	PPP1R26P1	-0.757	-0.664	-3.668	0.000
ENSG00000269352	PTOV1-AS2	-0.637	-0.535	-3.647	0.000
ENSG00000188493	C19orf54	-0.613	0.423	-3.626	0.000
ENSG00000274272	RP11-44M6.7	-0.900	-0.856	-3.614	0.001
ENSG00000128242	GAL3ST1	-0.689	0.660	-3.607	0.001
ENSG00000271828	CTD-2310F14.1	-0.491	0.459	-3.510	0.001
ENSG00000272138	LINC01607	0.971	-2.013	3.413	0.001
ENSG00000141627	DYM	0.201	5.989	3.400	0.001
ENSG00000254391	CTC-529G1.1	-1.011	-1.530	-3.393	0.001
ENSG00000179967	PPP1R14BP3	-0.456	0.850	-3.379	0.001
ENSG00000137634	NXPE4	-0.668	-0.890	-3.366	0.001
ENSG00000231799	RP13-93L13.2	-0.575	0.873	-3.349	0.001
ENSG00000132170	PPARG	0.328	2.856	3.341	0.001
ENSG00000223901	AP001469.5	-0.634	0.281	-3.241	0.002
ENSG00000106069	CHN2	-0.424	1.810	-3.234	0.002
ENSG00000259440	NPM1P43	-0.735	0.286	-3.200	0.002
ENSG00000125820	NKX2-2	-0.519	0.682	-3.198	0.002
ENSG00000224367	OACYLP	0.883	-1.743	3.151	0.002
ENSG00000260647	RP1-178F10.1	-0.771	-1.283	-3.135	0.002
ENSG00000241549	GUSBP2	-0.489	1.676	-3.132	0.002
ENSG00000260628	RP11-1166P10.1	-0.560	0.430	-3.104	0.003
ENSG00000188152	NUTM2G	-0.578	-0.447	-3.093	0.003
ENSG00000124380	SNRNP27	0.318	5.766	3.093	0.003
ENSG00000176463	SLCO3A1	-0.314	4.240	-3.089	0.003
ENSG00000235833	AC159540.14	-1.867	0.031	-3.083	0.003
ENSG00000228793	RP1-223B1.1	-1.353	-1.431	-3.079	0.003
ENSG00000275144	SNX18P5	-1.046	-0.873	-3.079	0.003
ENSG00000105327	BBC3	0.442	0.808	3.072	0.003
ENSG00000260988	RP11-285A1.1	0.726	-1.456	3.068	0.003
ENSG00000253525	CTD-2114J12.1	-1.363	-0.911	-3.028	0.003
ENSG00000235927	NEXN-AS1	-0.791	-1.093	-3.015	0.003
ENSG00000168418	KCNG4	-0.981	-1.524	-3.014	0.003
ENSG00000181409	AATK	-0.339	5.145	-2.990	0.004
ENSG00000254463	RP11-484D2.3	-0.456	1.098	-2.985	0.004
ENSG00000107816	LZTS2	-0.248	3.990	-2.977	0.004
ENSG00000184182	UBE2F	0.269	3.863	2.973	0.004
ENSG00000258948	KRT8P1	-0.788	-1.140	-2.945	0.004
ENSG00000226148	SLC25A39P1	-0.612	-0.120	-2.925	0.004
ENSG00000275494	RP11-266L9.8	-0.658	-1.591	-2.919	0.005
ENSG00000266651	RP11-138I1.3	-0.891	-2.179	-2.919	0.005
ENSG00000230988	RPL23AP11	0.991	-2.503	2.913	0.005
ENSG00000188266	HYKK	0.246	2.520	2.900	0.005

ENSG00000255141	HNRNPA1P76	-0.738	-0.590	-2.886	0.005
ENSG00000161911	TREML1	1.074	-1.784	2.878	0.005
ENSG00000117266	CDK18	-0.482	4.901	-2.874	0.005
ENSG00000278175	GLIDR	-0.465	1.732	-2.874	0.005
ENSG00000227267	RP11-305O4.2	-0.615	-0.877	-2.870	0.005
ENSG00000019186	CYP24A1	1.079	-0.538	2.867	0.005
ENSG00000120458	MSANTD2	-0.202	3.898	-2.853	0.005
ENSG00000250197	HMGN1P15	-0.578	-0.374	-2.844	0.006
ENSG00000270842	RP11-57B24.2	-0.532	-0.109	-2.840	0.006
ENSG00000095203	EPB41L4B	0.234	6.646	2.839	0.006
ENSG00000237317	RP4-809F4.1	-0.842	-1.713	-2.831	0.006
ENSG00000198944	SOWAHA	0.401	5.614	2.830	0.006
ENSG00000230006	ANKRD36BP2	0.249	5.901	2.829	0.006
ENSG00000011132	APBA3	-0.389	1.413	-2.826	0.006
ENSG00000255893	RP11-685N10.1	-0.804	-0.857	-2.820	0.006
ENSG00000266477	RN7SL616P	1.230	-3.369	2.812	0.006
ENSG00000234998	RP11-439A17.10	0.751	-1.759	2.808	0.006
ENSG00000225299	RP11-344A5.1	-0.586	1.688	-2.798	0.006
ENSG00000143552	NUP210L	-0.540	1.594	-2.793	0.007
ENSG00000250140	CTD-2517O10.5	-1.031	-1.960	-2.790	0.007
ENSG00000280197	C18orf12	-0.899	-2.470	-2.785	0.007
ENSG00000153234	NR4A2	-0.532	3.350	-2.779	0.007
ENSG00000231769	RP1-8B1.4	0.387	0.243	2.775	0.007
ENSG00000201291	RNU1-34P	1.279	-3.693	2.768	0.007
ENSG00000253653	CTC-248O19.1	0.959	-2.597	2.765	0.007
ENSG00000278881	FP325331.1	-0.858	-1.189	-2.765	0.007
ENSG00000221063	MIR1296	-0.678	-1.386	-2.757	0.007
ENSG00000249937	LINC02223	-0.482	2.531	-2.754	0.007
ENSG00000124243	BCAS4	0.235	5.237	2.752	0.007
ENSG00000154898	CCDC144CP	-0.330	6.000	-2.751	0.007
ENSG00000276170	AC124789.1	-0.799	-1.616	-2.749	0.007
ENSG00000207357	RNU6-2	0.886	3.298	2.747	0.007
ENSG00000207492	RNU6-713P	1.078	-3.612	2.746	0.007
ENSG00000266783	RP11-715F3.2	-0.688	-0.702	-2.741	0.008
ENSG00000067601	PMS2P4	0.276	2.255	2.738	0.008
ENSG00000103313	MEFV	0.546	0.328	2.730	0.008
ENSG00000237911	SRP68P3	-0.604	-0.355	-2.728	0.008
ENSG00000241945	PWP2	-1.084	1.104	-2.719	0.008
ENSG00000226695	ANKRD20A10P	0.698	-0.558	2.718	0.008
ENSG00000079689	SCGN	0.926	-1.178	2.718	0.008
ENSG00000103184	SEC14L5	-0.238	5.425	-2.714	0.008
ENSG00000277500	RP11-456J20.2	-0.568	-0.916	-2.712	0.008
ENSG00000268081	RP11-678G14.2	0.747	-0.934	2.712	0.008
ENSG00000039139	DNAH5	0.238	3.897	2.709	0.008
ENSG00000204963	PCDHA7	-0.448	1.232	-2.707	0.008
ENSG00000111906	HDDC2	0.245	5.858	2.703	0.008
ENSG00000276868	RP11-318K12.3	-0.745	-1.719	-2.694	0.009

ENSG00000243352	RN7SL8P	0.980	-2.662	2.690	0.009
ENSG00000154889	MPPE1	-0.228	3.261	-2.689	0.009
ENSG00000212205	Y_RNA	-0.679	-2.155	-2.689	0.009
ENSG00000280927	CTBP1-AS	-0.468	0.320	-2.685	0.009
ENSG00000035664	DAPK2	-0.336	2.954	-2.682	0.009
ENSG00000232457	SLC16A6P1	-0.725	-0.910	-2.681	0.009
ENSG00000269837	IPO5P1	0.225	3.254	2.680	0.009
ENSG00000274425	AC114271.2	0.867	-2.261	2.679	0.009
ENSG00000183458	RP11-958N24.1	-0.606	1.896	-2.676	0.009
ENSG00000229427	ANKRD26P4	-0.527	0.341	-2.674	0.009
ENSG00000129810	SGO1	-0.681	-1.065	-2.669	0.009
ENSG00000175206	NPPA	0.700	-0.728	2.661	0.009
ENSG00000283050	GTF2IP12	-0.304	1.903	-2.661	0.009
ENSG00000137975	CLCA2	-0.960	-1.705	-2.656	0.010
ENSG00000279227	AC009303.2	-0.562	-0.198	-2.652	0.010
ENSG00000219797	PPIAP9	0.688	-0.942	2.639	0.010
ENSG00000251276	MAGI2-AS1	-0.590	0.228	-2.637	0.010
ENSG00000243321	LINC01998	-0.690	-0.891	-2.637	0.010
ENSG00000236556	RP11-443O13.3	-0.425	0.783	-2.632	0.010
ENSG00000259505	RP11-74D7.1	-0.890	-0.535	-2.631	0.010
ENSG00000172123	SLFN12	0.695	0.551	2.627	0.010
ENSG00000066379	ZNRD1	-0.344	0.951	-2.627	0.010
ENSG00000078304	PPP2R5C	0.193	7.403	2.625	0.010
ENSG00000142279	WTIP	0.364	2.127	2.619	0.011
ENSG00000258033	RP11-350G24.2	-0.357	2.724	-2.614	0.011
ENSG00000256563	NANOGNBP2	-1.055	-2.240	-2.610	0.011
ENSG00000242020	RN7SL68P	-0.584	-0.611	-2.606	0.011
ENSG00000227000	HSPD1P14	-0.766	-1.493	-2.603	0.011
ENSG00000201820	Y_RNA	-0.716	-2.402	-2.600	0.011
ENSG00000223899	SEC13P1	-0.529	-0.586	-2.597	0.011
ENSG00000265298	RP13-104F24.3	-0.299	2.207	-2.597	0.011
ENSG00000213600	XXcos-LUCA16.1	0.751	-2.245	2.597	0.011
ENSG00000264343	NOTCH2NL	-0.475	2.719	-2.590	0.011
ENSG00000167360	OR51Q1	-1.032	-2.388	-2.590	0.011
ENSG00000237361	TUSC8	0.659	-1.619	2.590	0.011
ENSG00000254714	RP11-163O19.1	-0.741	-1.614	-2.587	0.011
ENSG00000115268	RPS15	0.307	4.798	2.586	0.011
ENSG00000198815	FOXJ3	0.134	6.688	2.581	0.012
ENSG00000224678	GAPDHP46	-0.652	-1.169	-2.574	0.012
ENSG00000232158	C11orf98P1	-0.601	0.290	-2.573	0.012
ENSG00000178821	TMEM52	1.071	-2.127	2.572	0.012
ENSG00000255964	RP11-69M1.3	-0.497	0.261	-2.571	0.012
ENSG00000276045	ORAI1	0.635	-1.119	2.571	0.012
ENSG00000108587	GOSR1	0.138	5.818	2.571	0.012
ENSG00000260942	CAPN10-AS1	-0.295	1.436	-2.563	0.012
ENSG00000279409	RP11-133K1.9	0.696	-0.957	2.563	0.012
ENSG00000261924	CTD-2561B21.5	-0.619	-1.084	-2.560	0.012

ENSG00000101353	MROH8	0.209	2.724	2.559	0.012
ENSG00000100129	EIF3L	0.272	5.632	2.555	0.012
ENSG00000265337	RP11-466A19.5	-0.931	-1.679	-2.551	0.013
ENSG00000258738	RP11-73E17.2	-0.888	-1.863	-2.549	0.013
ENSG00000107331	ABCA2	-0.327	7.262	-2.548	0.013
ENSG00000228510	RP11-339B21.8	-0.538	0.998	-2.548	0.013
ENSG00000137747	TMPRSS13	0.871	-2.538	2.547	0.013
ENSG00000246334	PRR7-AS1	-0.426	0.061	-2.543	0.013
ENSG00000259151	CAP2P1	-0.418	0.660	-2.539	0.013
ENSG00000251187	CTC-459M5.2	-0.597	-1.432	-2.537	0.013
ENSG00000223508	RPL23AP53	0.322	2.585	2.534	0.013
ENSG00000132671	SSTR4	0.778	-1.561	2.532	0.013
ENSG00000095397	WHRN	-0.484	2.622	-2.529	0.013
ENSG00000033867	SLC4A7	0.297	7.169	2.528	0.013
ENSG00000254783	RP11-320L11.2	-0.364	0.286	-2.527	0.013
ENSG00000226330	RP11-739N20.2	-0.511	-0.981	-2.527	0.013
ENSG00000278993	RP3-424M6.4	-0.643	-1.523	-2.521	0.014
ENSG00000101306	MYLK2	0.948	-1.501	2.520	0.014
ENSG00000200170	Y_RNA	0.731	-2.227	2.514	0.014
ENSG00000178125	PPP1R42	0.514	-0.700	2.514	0.014
ENSG00000259628	RP11-467H10.2	-0.776	-2.072	-2.512	0.014
ENSG00000267605	CTD-3220F14.1	-0.414	1.220	-2.510	0.014
ENSG00000133055	MYBPH	-0.491	0.058	-2.507	0.014
ENSG00000263958	RP11-676J15.1	0.409	0.669	2.505	0.014
ENSG00000272072	CTA-363E19.2	-0.291	1.896	-2.504	0.014
ENSG00000153237	CCDC148	0.268	4.055	2.503	0.014
ENSG00000284726	#N/A	-0.396	0.143	-2.499	0.014
ENSG00000160803	UBQLN4	0.179	5.256	2.495	0.015
ENSG00000183281	PLGLB1	-0.801	1.280	-2.494	0.015
ENSG00000161180	CCDC116	-0.850	-2.355	-2.492	0.015
ENSG00000285588	#N/A	-0.357	3.633	-2.491	0.015
ENSG00000227115	LINC01630	-0.514	2.997	-2.487	0.015
ENSG00000226673	LINC01108	0.955	-2.092	2.485	0.015
ENSG00000249767	ENPP7P10	-0.486	0.354	-2.485	0.015
ENSG00000261173	RP11-169E6.1	-0.603	-1.190	-2.484	0.015
ENSG00000215895	RP11-334L9.1	-0.709	0.587	-2.483	0.015
ENSG00000228536	RP11-392O17.1	-0.530	0.580	-2.480	0.015
ENSG00000154493	C10orf90	-0.490	4.165	-2.479	0.015
ENSG00000254206	NPIPB11	-0.725	2.154	-2.479	0.015
ENSG00000212135	SNORD67	0.735	-1.221	2.479	0.015
ENSG00000221539	SNORD99	0.970	-0.234	2.479	0.015
ENSG00000148584	A1CF	-0.608	-0.050	-2.475	0.015
ENSG00000267882	RP4-569M23.5	-0.486	0.740	-2.473	0.015
ENSG00000116983	HPCAL4	0.285	8.597	2.467	0.016
ENSG00000265281	MIR3935	-0.610	-1.181	-2.467	0.016
ENSG00000186523	FAM86B1	-0.566	2.704	-2.464	0.016
ENSG00000264706	RN7SL217P	0.855	-1.986	2.461	0.016

ENSG00000213891	RPL3P6	-0.355	0.963	-2.460	0.016
ENSG00000230424	RP1-43E13.2	-0.596	-2.230	-2.460	0.016
ENSG00000203326	ZNF525	0.248	4.093	2.459	0.016
ENSG00000207116	RNU6-31P	0.774	1.614	2.459	0.016
ENSG00000212526	RNU6-466P	-0.814	-2.243	-2.457	0.016
ENSG00000262619	LINC00621	0.469	0.207	2.456	0.016
ENSG00000125650	PSPN	0.303	1.385	2.456	0.016
ENSG00000115008	IL1A	-0.735	-1.375	-2.456	0.016
ENSG00000227950	RP11-312B8.2	-0.692	-1.235	-2.455	0.016
ENSG00000241416	KRT8P13	-0.783	-0.113	-2.454	0.016
ENSG00000173327	MAP3K11	-0.199	3.971	-2.452	0.016
ENSG00000279801	RP11-75C10.6	-0.665	0.011	-2.451	0.016
ENSG00000235027	AC068580.6	-0.493	-0.775	-2.448	0.016
ENSG00000225828	FAM229A	-0.518	-0.344	-2.447	0.017
ENSG00000199880	RNU6-888P	0.823	-2.128	2.447	0.017
ENSG00000158769	F11R	0.426	1.844	2.446	0.017
ENSG00000149633	KIAA1755	-0.413	3.570	-2.446	0.017
ENSG00000103569	AQP9	0.373	1.736	2.446	0.017
ENSG00000274996	CTB-58E17.2	0.598	-0.987	2.446	0.017
ENSG00000140835	CHST4	-0.848	-1.622	-2.445	0.017
ENSG00000230601	RP11-402G3.3	-0.767	-1.212	-2.444	0.017
ENSG00000276156	RP11-491H9.4	-0.779	-2.322	-2.444	0.017
ENSG00000197978	GOLGA6L9	-0.389	1.961	-2.444	0.017
ENSG00000173200	PARP15	0.664	-1.199	2.441	0.017
ENSG00000234719	RP11-166B2.1	-0.873	0.907	-2.438	0.017
ENSG00000187764	SEMA4D	-0.247	6.328	-2.436	0.017
ENSG00000188690	UROS	0.227	5.618	2.434	0.017
ENSG00000135722	FBXL8	0.424	-0.463	2.432	0.017
ENSG00000204179	PTPN20	0.414	3.333	2.429	0.017
ENSG00000169499	PLEKHA2	0.232	5.935	2.427	0.017
ENSG00000218631	RP3-395C13.1	0.759	-1.441	2.423	0.018
ENSG00000197728	RPS26	0.544	2.196	2.423	0.018
ENSG00000188383	AC018892.9	-0.531	-1.085	-2.420	0.018
ENSG00000137221	TJAP1	-0.310	3.783	-2.420	0.018
ENSG00000183876	ARSI	-0.549	-0.860	-2.419	0.018
ENSG00000237667	LINC01115	-1.614	0.102	-2.418	0.018
ENSG00000165185	KIAA1958	-0.183	5.328	-2.417	0.018
ENSG00000238777	RNU7-141P	-0.661	-1.659	-2.416	0.018
ENSG00000226851	AC004112.4	-0.425	-0.524	-2.416	0.018
ENSG00000226756	AC007365.3	-0.696	-2.272	-2.415	0.018
ENSG00000065518	NDUFB4	0.236	3.828	2.415	0.018
ENSG00000220091	LAP3P1	-0.812	-2.132	-2.413	0.018
ENSG00000276281	RN7SL126P	0.883	-3.297	2.408	0.018
ENSG00000207507	RNU6-9	0.742	3.274	2.406	0.018
ENSG00000277117	CH507-9B2.1	-0.898	-0.546	-2.405	0.018
ENSG00000099260	PALMD	0.398	3.701	2.404	0.018
ENSG00000176912	TYMSOS	-0.900	-2.315	-2.404	0.018

ENSG00000163378	EOGT	0.243	4.020	2.401	0.019
ENSG00000130956	HABP4	0.233	6.927	2.400	0.019
ENSG00000278206	RP1-20N2.8	-0.407	-0.094	-2.397	0.019
ENSG00000270392	PFN1P2	-0.453	1.171	-2.397	0.019
ENSG00000245748	RP11-367J11.2	-0.483	-0.489	-2.394	0.019
ENSG00000106018	VIPR2	-0.405	1.377	-2.393	0.019
ENSG00000112561	TFEB	-0.391	1.889	-2.393	0.019
ENSG00000186301	MST1P2	-0.406	0.136	-2.392	0.019
ENSG00000280359	CTD-2071N1.2	-1.048	-2.107	-2.392	0.019
ENSG00000165071	TMEM71	0.519	-0.640	2.391	0.019
ENSG00000259876	CTD-3037G24.4	-0.648	-1.753	-2.390	0.019
ENSG00000253384	CTD-2547L16.3	-0.637	-1.152	-2.390	0.019
ENSG00000186881	OR13F1	-1.049	-2.387	-2.389	0.019
ENSG00000266304	RP11-484N16.1	-0.991	-3.091	-2.386	0.019
ENSG00000151532	VTI1A	0.137	6.261	2.386	0.019
ENSG00000253372	RP11-44N11.1	-0.441	0.821	-2.382	0.020
ENSG00000164764	SBSPON	-0.306	1.644	-2.378	0.020
ENSG00000242507	RP3-477O4.5	-1.065	-2.400	-2.377	0.020
ENSG00000154133	ROBO4	0.487	1.152	2.376	0.020
ENSG00000158315	RHBDL2	-0.326	2.540	-2.375	0.020
ENSG00000240888	RP13-635I23.3	-0.637	-1.635	-2.374	0.020
ENSG00000204930	FAM221B	-0.515	-0.754	-2.373	0.020
ENSG00000259999	RP11-252K23.1	-0.853	-1.953	-2.371	0.020
ENSG00000105889	STEAP1B	-0.355	1.204	-2.370	0.020
ENSG00000277147	LINC00869	0.243	3.981	2.370	0.020
ENSG00000226620	LINC00343	-0.552	0.958	-2.370	0.020
ENSG00000256922	RP11-749H20.1	-0.898	-2.336	-2.368	0.020
ENSG00000139613	SMARCC2	0.164	7.805	2.367	0.020
ENSG00000126822	PLEKHG3	-0.446	4.697	-2.367	0.020
ENSG00000272159	RP11-350N15.6	-0.897	-2.981	-2.364	0.020
ENSG00000228599	RPL7P52	-0.456	0.459	-2.361	0.021
ENSG00000205578	POM121B	-0.701	0.552	-2.361	0.021
ENSG00000140479	PCSK6	-0.357	3.874	-2.360	0.021
ENSG00000183542	KLRC4	-0.748	-2.001	-2.359	0.021
ENSG00000272886	DCP1A	0.110	5.005	2.356	0.021
ENSG00000273447	AC004067.5	0.574	-0.897	2.355	0.021
ENSG00000225986	UBXN10-AS1	0.829	-2.762	2.354	0.021
ENSG00000188846	RPL14	0.219	6.935	2.353	0.021
ENSG00000173480	ZNF417	-0.160	4.581	-2.353	0.021
ENSG00000184144	CNTN2	-0.397	6.700	-2.353	0.021
ENSG00000271049	CTD-2553L13.7	-0.453	-0.395	-2.351	0.021
ENSG00000236675	MTX1P1	-0.405	0.304	-2.350	0.021
ENSG00000266456	RP11-806L2.2	-0.545	1.109	-2.348	0.021
ENSG00000124523	SIRT5	0.145	5.285	2.347	0.021
ENSG00000140848	CPNE2	-0.259	3.687	-2.347	0.021
ENSG00000137251	TINAG	0.403	-0.278	2.347	0.021
ENSG00000100568	VTI1B	0.191	6.350	2.347	0.021

ENSG00000120256	LRP11	-0.215	2.477	-2.344	0.022
ENSG00000267339	LINC00906	-0.720	-1.184	-2.344	0.022
ENSG00000196821	C6orf106	0.175	6.810	2.343	0.022
ENSG00000282508	LINC01002	-0.648	1.241	-2.343	0.022
ENSG00000165914	TTC7B	0.152	6.464	2.343	0.022
ENSG00000250174	MYLK-AS2	-0.674	-1.277	-2.342	0.022
ENSG00000160710	ADAR	0.138	8.405	2.341	0.022
ENSG00000233339	AC018878.3	-0.852	-1.704	-2.340	0.022
ENSG00000267152	CTD-2528L19.6	0.224	2.298	2.339	0.022
ENSG00000148926	ADM	-0.878	0.710	-2.338	0.022
ENSG00000235605	RP5-827C21.1	-0.545	0.798	-2.337	0.022
ENSG00000008382	MPND	0.289	2.023	2.336	0.022
ENSG00000010803	SCMH1	0.141	5.028	2.335	0.022
ENSG00000182318	ZSCAN22	-0.229	2.659	-2.334	0.022
ENSG00000112096	SOD2	0.325	7.704	2.332	0.022
ENSG00000137807	KIF23	0.329	0.825	2.331	0.022
ENSG00000108830	RND2	0.241	4.629	2.323	0.023
ENSG00000121440	PDZRN3	0.192	5.676	2.319	0.023
ENSG00000272320	RP11-506K6.4	-0.373	2.510	-2.319	0.023
ENSG00000138316	ADAMTS14	-0.870	-0.001	-2.317	0.023
ENSG00000278341	RP5-1142A6.10	-0.869	-2.231	-2.315	0.023
ENSG00000228166	MTND1P11	-0.828	-1.730	-2.314	0.023
ENSG00000168038	ULK4	0.228	4.338	2.312	0.023
ENSG00000205746	RP11-1212A22.1	-0.702	1.723	-2.312	0.023
ENSG00000092529	CAPN3	-0.499	1.985	-2.311	0.023
ENSG00000171804	WDR87	-0.935	-1.736	-2.311	0.023
ENSG00000199730	RN7SKP95	-0.566	-1.393	-2.311	0.023
ENSG00000134086	VHL	0.149	5.307	2.310	0.023
ENSG00000173915	USMG5	0.337	5.435	2.310	0.023
ENSG00000254314	RP11-26M5.3	-0.798	-1.233	-2.310	0.023
ENSG00000243789	JMJD7	-0.470	-0.723	-2.310	0.023
ENSG00000144713	RPL32	0.405	6.252	2.310	0.023
ENSG00000233524	RANP8	-0.622	-1.399	-2.310	0.023
ENSG00000107130	NCS1	0.204	8.839	2.310	0.023
ENSG00000186352	ANKRD37	-0.516	1.468	-2.309	0.023
ENSG00000188612	SUMO2	0.303	5.333	2.307	0.024
ENSG00000255874	LINC00346	0.565	-0.128	2.306	0.024
ENSG00000266126	RP11-209D14.4	-0.587	-0.846	-2.305	0.024
ENSG00000081913	PHLPP1	-0.198	6.463	-2.304	0.024
ENSG00000206593	RNU6-47P	0.908	-1.483	2.304	0.024
ENSG00000249411	RP11-391J13.1	-0.856	-1.585	-2.303	0.024
ENSG00000261123	RP11-304L19.3	-0.622	-0.453	-2.302	0.024
ENSG00000261411	RP11-268G13.1	1.068	-2.979	2.301	0.024
ENSG00000274883	Metazoa_SRP	-0.877	-1.880	-2.299	0.024
ENSG00000186399	GOLGA8R	-0.566	0.824	-2.297	0.024
ENSG00000133687	TMTC1	0.225	6.812	2.296	0.024
ENSG00000248641	HMGA1P2	-0.560	-0.082	-2.295	0.024

ENSG00000100399	CHADL	-0.416	1.491	-2.295	0.024
ENSG00000134874	DZIP1	0.154	6.914	2.295	0.024
ENSG00000277829	RP5-1054A22.4	-0.484	0.060	-2.295	0.024
ENSG00000135929	CYP27A1	-0.316	2.970	-2.294	0.024
ENSG00000123473	STIL	0.177	3.120	2.294	0.024
ENSG00000243701	DUBR	-0.189	4.318	-2.294	0.024
ENSG00000285571	#N/A	-0.643	-0.441	-2.294	0.024
ENSG00000237065	NANOGP4	-0.590	0.068	-2.294	0.024
ENSG00000100461	RBM23	0.170	5.752	2.289	0.025
ENSG00000175220	ARHGAP1	-0.186	4.688	-2.288	0.025
ENSG00000196912	ANKRD36B	-0.249	6.859	-2.287	0.025
ENSG00000251389	YTHDF1P1	-0.797	-2.168	-2.286	0.025
ENSG00000140105	WARS	0.345	6.023	2.284	0.025
ENSG00000250407	CTB-99A3.1	-0.529	-0.556	-2.282	0.025
ENSG00000254398	RP11-578F21.2	-0.526	-0.313	-2.282	0.025
ENSG00000233296	AC092159.2	-1.098	-2.047	-2.279	0.025
ENSG00000184923	NUTM2A	-0.596	0.978	-2.278	0.025
ENSG00000136048	DRAM1	0.349	1.295	2.278	0.025
ENSG00000138823	MTTP	0.331	0.509	2.278	0.025
ENSG00000148826	NKX6-2	-0.550	2.333	-2.277	0.025
ENSG00000211452	DIO1	-0.566	-0.912	-2.276	0.025
ENSG00000118680	MYL12B	0.272	5.904	2.276	0.025
ENSG00000232732	AC073043.1	-0.552	-0.144	-2.276	0.025
ENSG00000269899	RP11-589N15.2	-0.534	-1.153	-2.276	0.025
ENSG00000256571	RP11-274J7.2	-0.605	-1.520	-2.276	0.025
ENSG00000228696	ARL17B	-0.573	2.127	-2.275	0.026
ENSG00000122367	LDB3	-0.503	3.383	-2.275	0.026
ENSG00000235033	RP11-61I13.3	-0.468	1.987	-2.273	0.026
ENSG00000236498	AC107081.5	-0.733	-2.238	-2.273	0.026
ENSG00000227372	TP73-AS1	-0.171	3.806	-2.271	0.026
ENSG00000163155	LYSMD1	0.200	3.290	2.269	0.026
ENSG00000251226	RP11-469N6.1	0.794	1.129	2.269	0.026
ENSG00000171944	OR52A5	-0.929	-1.471	-2.267	0.026
ENSG00000112877	CEP72	-0.234	2.081	-2.266	0.026
ENSG00000251664	PCDHA12	-0.298	1.045	-2.265	0.026
ENSG00000284657	#N/A	-0.533	-1.145	-2.264	0.026
ENSG00000240265	RP11-62G11.1	-0.803	-1.566	-2.264	0.026
ENSG00000236438	FAM157A	-0.654	0.475	-2.263	0.026
ENSG00000131055	COX4I2	0.844	-2.708	2.263	0.026
ENSG00000174485	DENND4A	0.140	7.314	2.260	0.026
ENSG00000235823	OLMALINC	-0.298	4.146	-2.257	0.027
ENSG00000113971	NPHP3	-0.298	3.030	-2.255	0.027
ENSG00000199311	SNORD115-34	0.824	-1.192	2.255	0.027
ENSG00000010030	ETV7	0.719	-1.523	2.254	0.027
ENSG00000269570	CMB9-55A18.1	-0.327	0.630	-2.254	0.027
ENSG00000273890	RP11-603J24.21	-0.605	-1.823	-2.253	0.027
ENSG00000184979	USP18	0.451	0.140	2.250	0.027

ENSG00000252464	RN7SKP70	-0.455	-0.212	-2.250	0.027
ENSG00000221983	UBA52	0.191	6.256	2.249	0.027
ENSG00000106341	PPP1R17	-0.412	0.133	-2.248	0.027
ENSG00000231652	RP11-553A21.3	0.348	0.043	2.247	0.027
ENSG00000285636	#N/A	-0.511	-0.367	-2.247	0.027
ENSG00000112667	DNPH1	0.261	2.959	2.247	0.027
ENSG00000278041	RP5-984P4.6	-0.467	1.835	-2.246	0.027
ENSG00000245685	AF146191.4	-0.374	0.651	-2.246	0.027
ENSG00000184984	CHRM5	-0.581	1.357	-2.244	0.028
ENSG00000254827	SLC22A18AS	0.891	-2.363	2.244	0.028
ENSG00000196547	MAN2A2	-0.170	6.147	-2.241	0.028
ENSG00000220008	LINGO3	-0.468	0.403	-2.241	0.028
ENSG00000160305	DIP2A	-0.160	5.704	-2.239	0.028
ENSG00000139433	GLTP	-0.335	4.344	-2.237	0.028
ENSG00000188536	HBA2	-0.747	1.282	-2.237	0.028
ENSG00000258900	HNRNPCP1	-0.230	1.076	-2.236	0.028
ENSG00000112782	CLIC5	0.349	3.121	2.236	0.028
ENSG00000267248	CTD-2319I12.4	0.288	0.987	2.236	0.028
ENSG00000167658	EEF2	0.214	8.974	2.236	0.028
ENSG00000234144	COX6CP13	-0.475	-0.690	-2.235	0.028
ENSG00000111364	DDX55	0.149	4.422	2.235	0.028
ENSG00000127125	PPCS	0.265	3.972	2.235	0.028
ENSG00000169203	RP11-231C14.4	-0.690	2.749	-2.234	0.028
ENSG00000218052	ADAMTS7P4	-0.695	-1.557	-2.234	0.028
ENSG00000005513	SOX8	-0.304	4.393	-2.234	0.028
ENSG00000235370	DNM1P51	-0.440	1.529	-2.234	0.028
ENSG00000233008	LINC01725	-0.437	1.545	-2.233	0.028
ENSG00000269463	RP11-727F15.13	-0.651	-1.751	-2.232	0.028
ENSG00000200591	Y_RNA	-0.720	-2.087	-2.232	0.028
ENSG00000259660	DNM1P47	-0.679	1.888	-2.230	0.028
ENSG00000023191	RNH1	-0.184	4.391	-2.229	0.029
ENSG00000120156	TEK	0.253	4.521	2.229	0.029
ENSG00000254941	RP11-677M14.5	-0.628	-1.000	-2.227	0.029
ENSG00000231758	AC092652.1	0.542	-1.410	2.227	0.029
ENSG00000232259	RP11-4C20.3	-0.455	-0.279	-2.227	0.029
ENSG00000196091	MYBPC1	0.289	5.121	2.226	0.029
ENSG00000161970	RPL26	0.324	5.923	2.225	0.029
ENSG00000026036	RTEL1-TNFRSF6B	-0.492	0.190	-2.225	0.029
ENSG00000234281	LANCL1-AS1	-0.317	2.479	-2.224	0.029
ENSG00000148082	SHC3	0.189	9.137	2.224	0.029
ENSG00000178115	GOLGA8Q	-0.499	0.143	-2.224	0.029
ENSG00000234040	RP11-254B13.4	-0.584	-1.407	-2.223	0.029
ENSG00000144031	ANKRD53	-0.506	0.203	-2.223	0.029
ENSG00000251413	RP11-534L6.5	0.696	-2.170	2.222	0.029
ENSG00000113657	DPYSL3	0.192	6.847	2.220	0.029
ENSG00000235026	DPP10-AS1	0.190	3.416	2.220	0.029
ENSG00000131459	GFPT2	0.176	3.992	2.220	0.029

ENSG00000273901	CTD-2619J13.27	-0.687	-1.236	-2.220	0.029
ENSG00000006282	SPATA20	0.246	4.486	2.220	0.029
ENSG00000167711	SERPINF2	-0.734	-1.808	-2.219	0.029
ENSG00000230373	GOLGA6L5P	-0.480	1.048	-2.219	0.029
ENSG00000243423	RP5-837J1.1	-0.593	-0.965	-2.219	0.029
ENSG00000196275	GTF2IRD2	-0.377	2.173	-2.219	0.029
ENSG00000131899	LLGL1	-0.252	4.265	-2.218	0.029
ENSG00000199332	Y_RNA	-0.739	-2.365	-2.217	0.029
ENSG00000250215	CIR1P2	-0.683	-0.069	-2.215	0.030
ENSG00000176720	BOK	-0.375	2.618	-2.215	0.030
ENSG00000141753	IGFBP4	0.345	2.425	2.215	0.030
ENSG00000239569	KMT2E-AS1	0.561	-1.282	2.214	0.030
ENSG00000170165	CR848007.2	-0.590	1.640	-2.211	0.030
ENSG00000205629	LCMT1	0.168	4.502	2.210	0.030
ENSG00000131943	C19orf12	0.188	5.609	2.210	0.030
ENSG00000248394	FOSL1P1	-0.577	-0.985	-2.210	0.030
ENSG00000108465	CDK5RAP3	-0.259	2.723	-2.207	0.030
ENSG00000257194	RP11-567C2.1	-0.732	-1.807	-2.207	0.030
ENSG00000161944	ASGR2	0.547	-0.976	2.206	0.030
ENSG00000282987	RP11-901H12.1	0.911	-2.581	2.205	0.030
ENSG00000133056	PIK3C2B	-0.211	4.993	-2.205	0.030
ENSG00000163634	THOC7	0.259	5.369	2.205	0.030
ENSG00000222024	AC004945.1	-0.300	2.559	-2.204	0.030
ENSG00000204323	SMIM5	-0.428	0.612	-2.204	0.030
ENSG00000078081	LAMP3	0.715	-1.681	2.204	0.030
ENSG00000065361	ERBB3	-0.496	4.784	-2.203	0.030
ENSG00000250571	GLI4	-0.350	1.232	-2.202	0.030
ENSG00000266803	RP1-77H15.1	0.643	-1.462	2.202	0.030
ENSG00000213711	PHBP7	-0.454	-0.098	-2.201	0.031
ENSG00000275197	RP11-295I5.4	-0.778	-2.221	-2.200	0.031
ENSG00000227230	RP11-261C10.5	-0.526	-0.898	-2.200	0.031
ENSG00000101098	RIMS4	0.217	5.726	2.199	0.031
ENSG00000106327	TFR2	0.248	2.040	2.199	0.031
ENSG00000274918	RP11-54G14.1	-0.799	-2.054	-2.199	0.031
ENSG00000231443	AC024937.6	-0.403	0.606	-2.198	0.031
ENSG00000262052	RP11-763E3.1	0.516	-1.266	2.196	0.031
ENSG00000142606	MMEL1	0.968	-2.799	2.194	0.031
ENSG00000116525	TRIM62	-0.222	2.736	-2.194	0.031
ENSG00000066735	KIF26A	0.436	0.338	2.192	0.031
ENSG00000104671	DCTN6	0.227	5.054	2.192	0.031
ENSG00000149639	SOGA1	-0.202	6.372	-2.191	0.031
ENSG00000166387	PPFIBP2	-0.267	4.086	-2.191	0.031
ENSG00000214954	LRRC69	-0.401	0.435	-2.191	0.031
ENSG00000276550	HERC2P2	-0.408	6.363	-2.190	0.031
ENSG00000177042	TMEM80	0.224	2.506	2.189	0.031
ENSG00000105926	MPP6	0.195	7.639	2.189	0.031
ENSG00000232756	RP5-1185I7.1	0.859	-2.047	2.189	0.031

ENSG00000267834	RP11-167N5.5	-0.560	-1.275	-2.187	0.032
ENSG00000143549	TPM3	0.228	7.511	2.187	0.032
ENSG00000184363	PKP3	-0.779	-1.754	-2.186	0.032
ENSG00000281195	ZNF638-IT1	-0.256	4.014	-2.186	0.032
ENSG00000258012	RP11-603K19.1	-0.420	1.869	-2.185	0.032
ENSG00000200795	RNU4-1	0.890	4.339	2.183	0.032
ENSG00000015475	BID	0.199	4.450	2.182	0.032
ENSG00000054690	PLEKHH1	-0.458	6.535	-2.182	0.032
ENSG00000198829	SUCNR1	0.911	-0.810	2.181	0.032
ENSG00000162747	FCGR3B	0.774	-0.705	2.180	0.032
ENSG00000242370	KCNAB1-AS1	-0.527	-0.296	-2.179	0.032
ENSG00000171033	PKIA	0.279	7.020	2.179	0.032
ENSG00000102882	MAPK3	0.189	5.476	2.176	0.032
ENSG00000156587	UBE2L6	0.261	4.063	2.176	0.032
ENSG00000285269	#N/A	-0.662	-1.288	-2.176	0.032
ENSG00000275345	RP11-697E2.11	0.508	-1.559	2.176	0.032
ENSG00000175130	MARCKSL1	-0.237	5.244	-2.176	0.032
ENSG00000066923	STAG3	-0.227	4.488	-2.176	0.032
ENSG00000184900	SUMO3	0.288	5.465	2.175	0.032
ENSG00000220848	RPS18P9	-0.230	1.758	-2.175	0.033
ENSG00000258949	RP11-857B24.5	-0.574	-1.554	-2.175	0.033
ENSG00000232878	DPYD-AS1	-0.367	0.275	-2.173	0.033
ENSG00000234945	GTF3C2-AS1	-0.460	0.204	-2.172	0.033
ENSG00000237440	ZNF737	0.166	5.572	2.172	0.033
ENSG00000238287	RP11-656D10.3	-0.483	-0.403	-2.172	0.033
ENSG00000265491	RNF115	0.116	6.836	2.171	0.033
ENSG00000171496	OR1L8	-0.542	-0.652	-2.171	0.033
ENSG00000223839	FAM95B1	-0.808	0.693	-2.170	0.033
ENSG00000189423	USP32P3	-0.276	3.465	-2.169	0.033
ENSG00000232087	HCFC2P1	1.006	-2.512	2.168	0.033
ENSG00000173482	PTPRM	0.184	6.931	2.167	0.033
ENSG00000285632	#N/A	0.625	-1.032	2.167	0.033
ENSG00000252277	SNORD116-30	-0.475	0.149	-2.167	0.033
ENSG00000155313	USP25	0.150	7.192	2.167	0.033
ENSG00000153283	CD96	0.692	-1.906	2.167	0.033
ENSG00000048544	MRPS10	0.226	5.009	2.167	0.033
ENSG00000202430	RNA5SP88	-0.512	-1.267	-2.166	0.033
ENSG00000179455	MKRN3	-0.304	2.432	-2.166	0.033
ENSG00000272071	RP11-332J15.4	0.640	-1.211	2.165	0.033
ENSG00000168967	PMCHL1	-0.557	-0.326	-2.163	0.033
ENSG00000206052	DOK6	0.318	7.734	2.161	0.034
ENSG00000006047	YBX2	0.486	0.097	2.160	0.034
ENSG00000108785	HSD17B1P1	-0.500	0.021	-2.159	0.034
ENSG00000118971	CCND2	0.212	8.365	2.159	0.034
ENSG00000163171	CDC42EP3	0.249	4.620	2.158	0.034
ENSG00000265118	CTD-2370N5.3	-0.566	-2.084	-2.158	0.034
ENSG00000250366	TUNAR	0.361	4.615	2.157	0.034

ENSG00000179930	ZNF648	0.663	-1.108	2.156	0.034
ENSG00000006377	DLX6	0.320	1.444	2.156	0.034
ENSG00000280098	AC011155.1	-0.661	-2.029	-2.155	0.034
ENSG00000250746	RP11-39C10.1	-0.327	2.681	-2.155	0.034
ENSG00000215158	RP11-1023L17.1	-0.251	3.915	-2.154	0.034
ENSG00000260259	LINC02166	0.578	-1.835	2.154	0.034
ENSG00000159904	ZNF890P	-0.432	1.560	-2.153	0.034
ENSG00000240175	MAGI1-AS1	-0.495	-0.178	-2.153	0.034
ENSG00000245498	RP11-677M14.7	-0.240	2.175	-2.153	0.034
ENSG00000275832	ARHGAP23	-0.209	5.237	-2.152	0.034
ENSG00000263753	LINC00667	0.207	5.235	2.152	0.034
ENSG00000180747	SMG1P3	-0.315	5.672	-2.152	0.034
ENSG00000277050	RP11-102G14.1	-0.588	-1.606	-2.152	0.034
ENSG00000254701	RP11-1415C14.4	-0.382	1.001	-2.151	0.034
ENSG00000263494	AC004702.2	-0.623	-1.900	-2.151	0.034
ENSG00000109685	WHSC1	0.109	7.355	2.150	0.035
ENSG00000275632	RP5-967N21.11	0.656	-1.465	2.150	0.035
ENSG00000069764	PLA2G10	0.768	-2.161	2.149	0.035
ENSG00000235308	RP11-82K18.2	0.831	-2.277	2.149	0.035
ENSG00000127580	WDR24	-0.289	1.479	-2.149	0.035
ENSG00000123106	CCDC91	0.214	6.074	2.148	0.035
ENSG00000226545	RP4-633I8.3	-0.599	-1.181	-2.148	0.035
ENSG00000106636	YKT6	0.157	5.164	2.148	0.035
ENSG00000227331	AC005042.2	-0.318	0.566	-2.147	0.035
ENSG00000226994	AC012593.1	-0.511	0.709	-2.146	0.035
ENSG00000151689	INPP1	-0.184	4.524	-2.145	0.035
ENSG00000283196	RP4-614C10.2	-0.503	0.808	-2.145	0.035
ENSG00000104899	AMH	-0.782	-1.415	-2.144	0.035
ENSG00000270909	RP11-24B13.2	-0.581	-0.519	-2.143	0.035
ENSG00000285591	#N/A	-0.532	-0.302	-2.143	0.035
ENSG00000259462	CPEB1-AS1	-0.292	1.580	-2.143	0.035
ENSG00000185607	ACTBP7	-0.331	1.578	-2.143	0.035
ENSG00000261121	LINC02473	-0.799	-0.905	-2.143	0.035
ENSG00000277918	RNVU1-28	0.703	3.841	2.142	0.035
ENSG00000123240	OPTN	0.158	7.941	2.141	0.035
ENSG00000233766	AC098617.1	-0.313	3.212	-2.141	0.035
ENSG00000224965	LINC02586	0.708	-2.395	2.140	0.035
ENSG00000085491	SLC25A24	0.252	3.502	2.140	0.035
ENSG00000244274	DBNDD2	-0.404	1.856	-2.138	0.035
ENSG00000223598	RP1-292B18.4	-0.737	-2.406	-2.138	0.036
ENSG00000166394	CYB5R2	-0.377	2.091	-2.137	0.036
ENSG00000262766	RP11-196G11.4	-0.421	-0.403	-2.136	0.036
ENSG00000153802	TMPRSS11D	-0.387	1.863	-2.136	0.036
ENSG00000222007	AC064874.1	-0.424	-0.158	-2.135	0.036
ENSG00000239268	RP11-384F7.2	-0.154	4.481	-2.135	0.036
ENSG00000197756	RPL37A	0.300	6.876	2.135	0.036
ENSG00000103707	MTFMT	0.157	3.308	2.135	0.036

ENSG00000268379	CTC-360J11.4	-0.505	1.797	-2.134	0.036
ENSG00000241468	ATP5J2	0.250	4.016	2.134	0.036
ENSG00000127507	ADGRE2	-0.612	0.727	-2.134	0.036
ENSG00000253784	RP11-1145L24.1	-0.746	-1.502	-2.133	0.036
ENSG00000072134	EPN2	-0.137	5.511	-2.133	0.036
ENSG00000109917	ZPR1	0.193	5.892	2.132	0.036
ENSG00000100897	DCAF11	0.176	6.145	2.131	0.036
ENSG00000141933	TPGS1	-0.402	0.625	-2.131	0.036
ENSG00000206899	RNU6-36P	0.567	3.328	2.131	0.036
ENSG00000204909	SPINK9	-0.374	0.437	-2.129	0.036
ENSG00000153214	TMEM87B	0.186	4.454	2.128	0.036
ENSG00000229520	LINC00404	-0.394	-0.466	-2.128	0.036
ENSG00000251892	RNU7-84P	-0.662	-1.044	-2.128	0.036
ENSG00000280106	CTC-523E23.3	-0.293	0.629	-2.127	0.036
ENSG00000158158	CNNM4	-0.160	3.553	-2.125	0.037
ENSG00000235865	GSN-AS1	-0.460	3.017	-2.124	0.037
ENSG00000264340	RP11-231E4.3	-0.955	-2.776	-2.124	0.037
ENSG00000251307	RP11-506H20.1	-0.453	-1.318	-2.123	0.037
ENSG00000201025	SNORD74	-0.511	-1.778	-2.122	0.037
ENSG00000231649	SPATA31B1P	0.695	-1.571	2.122	0.037
ENSG00000197815	RP1-253P7.4	-0.287	2.599	-2.122	0.037
ENSG00000254791	FAR1-IT1	-0.721	-1.465	-2.122	0.037
ENSG00000252636	RNU6-826P	1.037	-3.426	2.121	0.037
ENSG00000271321	CTAGE6	-0.662	-1.421	-2.120	0.037
ENSG00000236202	GRM7-AS1	0.595	-0.509	2.119	0.037
ENSG00000205482	SPDYE18	-0.419	0.602	-2.119	0.037
ENSG00000102967	DHODH	-0.177	3.236	-2.119	0.037
ENSG00000180104	EXOC3	0.122	5.562	2.118	0.037
ENSG00000235426	RP11-342M3.5	-0.832	-2.239	-2.118	0.037
ENSG00000224805	LINC00853	0.971	-2.940	2.118	0.037
ENSG00000260372	AQP4-AS1	0.261	3.272	2.116	0.037
ENSG00000197124	ZNF682	-0.215	3.511	-2.116	0.037
ENSG00000280408	RP11-818O24.2	-0.803	-2.287	-2.116	0.037
ENSG00000262903	RP11-235E17.6	-0.599	-1.336	-2.115	0.037
ENSG00000256967	RP11-273B20.1	-0.460	-1.166	-2.114	0.038
ENSG00000063180	CA11	0.290	6.680	2.113	0.038
ENSG00000207442	SNORD116-6	0.481	-0.110	2.113	0.038
ENSG00000270571	RP11-355F16.1	-0.365	0.017	-2.112	0.038
ENSG00000160075	SSU72	0.150	5.584	2.112	0.038
ENSG00000269693	CTD-2192J16.20	-0.241	1.201	-2.111	0.038
ENSG00000234268	AP000936.1	-0.347	0.564	-2.111	0.038
ENSG00000233334	FAM53B-AS1	-0.360	0.869	-2.110	0.038
ENSG00000234247	RP11-100E13.3	-0.922	-2.166	-2.110	0.038
ENSG00000234492	RPL34-AS1	-0.310	0.882	-2.110	0.038
ENSG00000258970	RP11-100M12.1	-0.652	-1.371	-2.109	0.038
ENSG00000160285	LSS	-0.229	4.969	-2.108	0.038
ENSG00000241525	AC108004.3	0.923	-2.880	2.107	0.038

ENSG00000267319	CTD-2528L19.3	-0.560	-1.495	-2.106	0.038
ENSG00000111696	NT5DC3	0.184	6.897	2.106	0.038
ENSG00000207063	SNORD116-1	0.611	0.406	2.106	0.038
ENSG00000285665	#N/A	-0.612	1.665	-2.105	0.038
ENSG00000258839	MC1R	-0.452	-0.047	-2.104	0.038
ENSG00000264727	RP11-680C21.1	0.446	-0.020	2.104	0.038
ENSG00000170837	GPR27	0.179	3.762	2.103	0.039
ENSG00000254454	RCC2P6	-0.443	0.636	-2.102	0.039
ENSG00000144893	MED12L	0.105	6.770	2.101	0.039
ENSG00000130856	ZNF236	0.118	6.321	2.101	0.039
ENSG00000245648	RP11-277P12.20	-0.420	0.110	-2.101	0.039
ENSG00000256377	RP11-1060J15.4	-0.647	-2.175	-2.101	0.039
ENSG00000197019	SERTAD1	0.518	-0.174	2.099	0.039
ENSG00000279708	CTD-2335A18.2	0.941	-2.642	2.099	0.039
ENSG00000070808	CAMK2A	0.175	9.908	2.099	0.039
ENSG00000179029	TMEM107	0.173	3.134	2.097	0.039
ENSG00000229241	PNPT1P1	-0.370	2.813	-2.097	0.039
ENSG00000171476	HOPX	0.329	6.100	2.097	0.039
ENSG00000052126	PLEKHA5	0.145	7.400	2.097	0.039
ENSG00000162419	GMEB1	0.136	4.176	2.096	0.039
ENSG00000254245	PCDHGA3	-0.284	2.894	-2.095	0.039
ENSG00000156411	C14orf2	0.305	5.916	2.093	0.039
ENSG00000258204	RP11-90E9.1	-0.408	1.515	-2.092	0.040
ENSG00000173210	ABLIM3	0.170	5.436	2.092	0.040
ENSG00000273004	GS1-279B7.2	-0.596	-2.027	-2.092	0.040
ENSG00000230922	RP4-753M9.1	-0.443	-0.052	-2.091	0.040
ENSG00000250326	RP11-284M14.1	-0.446	0.298	-2.091	0.040
ENSG00000178734	LMO7DN	-0.768	-2.426	-2.091	0.040
ENSG00000262732	CTD-2135D7.2	-0.434	-0.083	-2.090	0.040
ENSG00000151247	EIF4E	0.216	7.091	2.090	0.040
ENSG00000248801	C8orf34-AS1	0.460	-0.391	2.089	0.040
ENSG00000250208	FZD10-AS1	-0.575	-0.742	-2.089	0.040
ENSG00000216809	RP11-57K17.1	-0.326	1.394	-2.088	0.040
ENSG00000107731	UNC5B	-0.246	3.887	-2.087	0.040
ENSG00000108100	CCNY	0.190	6.740	2.087	0.040
ENSG00000145545	SRD5A1	0.214	5.568	2.086	0.040
ENSG00000285190	#N/A	-0.444	-1.021	-2.086	0.040
ENSG00000235205	TATDN2P3	-0.877	-1.913	-2.086	0.040
ENSG00000270533	bP-21201H5.1	-0.635	-0.308	-2.086	0.040
ENSG00000163072	NOSTRIN	0.417	2.741	2.086	0.040
ENSG00000207360	RNU6-14P	0.510	1.318	2.085	0.040
ENSG00000138175	ARL3	0.164	5.679	2.085	0.040
ENSG00000275139	RP1-101D8.1	-0.418	0.059	-2.084	0.040
ENSG00000186777	ZNF732	0.380	1.164	2.084	0.040
ENSG00000261684	RP11-265N6.1	-0.593	-1.740	-2.084	0.040
ENSG00000271396	RP11-697E2.9	-0.434	-0.038	-2.084	0.040
ENSG00000226216	RPS12P5	0.662	-1.942	2.083	0.040

ENSG00000249236	CTD-2227I18.1	0.504	-2.002	2.082	0.040
ENSG00000172508	CARNS1	-0.539	4.388	-2.082	0.040
ENSG00000185864	NPIPB4	-0.495	4.318	-2.082	0.040
ENSG00000115677	HDLBP	0.153	8.032	2.081	0.041
ENSG00000117791	MTARC2	0.160	3.948	2.081	0.041
ENSG00000261126	RP11-795F19.1	-0.333	2.211	-2.080	0.041
ENSG00000204839	MROH6	-0.382	-0.689	-2.079	0.041
ENSG00000255176	AP002954.3	-0.513	1.128	-2.079	0.041
ENSG00000254080	RP11-730G20.1	-0.464	0.070	-2.077	0.041
ENSG00000245293	RP11-286E11.1	-0.307	1.051	-2.076	0.041
ENSG00000234136	AC055764.1	-0.589	-1.000	-2.076	0.041
ENSG00000239726	RN7SL688P	-0.751	-2.422	-2.076	0.041
ENSG00000225460	RP13-93L13.1	-0.558	-0.284	-2.075	0.041
ENSG00000171840	NINJ2	-0.521	0.204	-2.075	0.041
ENSG00000215458	AATBC	-0.382	0.866	-2.075	0.041
ENSG00000251805	SCARNA21	0.748	-2.361	2.074	0.041
ENSG00000259295	CSPG4P12	-0.656	1.880	-2.073	0.041
ENSG00000275014	RN7SL166P	0.409	-0.687	2.073	0.041
ENSG00000188452	CERKL	0.322	1.851	2.073	0.041
ENSG00000259212	CTD-3065B20.2	-0.434	0.278	-2.072	0.041
ENSG00000272979	RP11-647K16.1	-0.576	-1.287	-2.071	0.042
ENSG00000258794	DUX4L27	-0.766	-1.508	-2.069	0.042
ENSG00000223705	NSUN5P1	-0.318	3.371	-2.069	0.042
ENSG00000118816	CCNI	0.185	8.503	2.067	0.042
ENSG00000255035	SDHCP4	-0.529	-0.441	-2.067	0.042
ENSG00000252874	Y_RNA	-0.490	-1.207	-2.066	0.042
ENSG00000165669	FAM204A	0.174	6.294	2.065	0.042
ENSG00000272597	RP11-446H18.6	-0.470	0.072	-2.065	0.042
ENSG00000154734	ADAMTS1	-0.509	4.237	-2.063	0.042
ENSG00000264278	RP11-162A12.2	0.547	-1.686	2.063	0.042
ENSG00000008517	IL32	0.747	-0.535	2.062	0.042
ENSG00000162825	NBPF20	-0.456	4.390	-2.062	0.042
ENSG00000239830	RPS4XP22	-0.862	0.168	-2.062	0.042
ENSG00000111203	ITFG2	-0.173	3.687	-2.062	0.042
ENSG00000228509	AC006460.2	-0.442	-1.019	-2.061	0.042
ENSG00000165959	CLMN	-0.364	5.808	-2.061	0.043
ENSG00000217555	CKLF	-0.419	-0.410	-2.060	0.043
ENSG00000276588	RN7SL322P	0.823	-3.222	2.059	0.043
ENSG00000230630	DNM3OS	-0.247	2.767	-2.059	0.043
ENSG00000204103	MAFB	0.266	3.100	2.059	0.043
ENSG00000185787	MORF4L1	0.275	6.750	2.059	0.043
ENSG00000180479	ZNF571	-0.134	3.872	-2.059	0.043
ENSG00000163412	EIF4E3	0.168	6.298	2.058	0.043
ENSG00000207279	SNORD116-24	0.526	1.078	2.057	0.043
ENSG00000227236	RP11-365D9.1	-0.743	-1.792	-2.057	0.043
ENSG00000176102	CSTF3	0.134	4.743	2.057	0.043
ENSG00000224689	ZNF812P	0.505	-1.028	2.057	0.043

ENSG00000189043	NDUFA4	0.273	6.081	2.056	0.043
ENSG00000253516	HMGB1P41	-0.323	1.109	-2.056	0.043
ENSG00000154328	NEIL2	0.192	4.496	2.054	0.043
ENSG00000228539	RP11-526K17.2	0.478	-0.692	2.053	0.043
ENSG00000206562	METTL6	0.113	4.703	2.053	0.043
ENSG00000251497	RP11-197N18.7	-0.496	-0.167	-2.052	0.043
ENSG00000204525	HLA-C	0.364	4.452	2.052	0.043
ENSG00000230506	RP11-416N4.4	-0.580	-2.193	-2.050	0.044
ENSG00000270799	RP11-451F14.1	-0.511	-0.544	-2.050	0.044
ENSG00000277741	GOLGA6L17P	-0.480	-0.270	-2.050	0.044
ENSG00000240423	LINC00636	0.563	-1.502	2.049	0.044
ENSG00000119632	IFI27L2	0.247	2.552	2.049	0.044
ENSG00000065427	KARS	0.198	5.460	2.047	0.044
ENSG00000183889	AC138969.4	-0.803	2.064	-2.046	0.044
ENSG00000254285	KRT8P3	-0.710	-2.009	-2.046	0.044
ENSG00000172890	NADSYN1	-0.197	4.335	-2.046	0.044
ENSG00000285936	#N/A	-0.463	-0.808	-2.046	0.044
ENSG00000135778	NTPCR	0.208	6.036	2.045	0.044
ENSG00000244050	RP11-266K4.1	0.410	-0.495	2.045	0.044
ENSG00000225181	RP11-388K2.1	0.609	-1.916	2.045	0.044
ENSG00000050393	MCUR1	0.179	5.826	2.044	0.044
ENSG00000235389	RP11-134K1.3	-0.514	-0.444	-2.044	0.044
ENSG00000164683	HEY1	0.226	4.413	2.043	0.044
ENSG00000274038	RP11-66H6.4	-0.712	-2.320	-2.043	0.044
ENSG00000089009	RPL6	0.333	6.270	2.042	0.044
ENSG00000202354	RNY3	0.642	1.116	2.042	0.044
ENSG00000170142	UBE2E1	0.191	5.613	2.041	0.044
ENSG00000157895	C12orf43	0.144	4.680	2.041	0.045
ENSG00000139438	FAM222A	-0.319	2.814	-2.041	0.045
ENSG00000279817	RP11-386I8.4	0.674	-2.636	2.040	0.045
ENSG00000267506	RP11-13K12.1	-0.375	3.420	-2.040	0.045
ENSG00000280189	RP11-491F9.2	-0.582	-0.638	-2.039	0.045
ENSG00000118298	CA14	-0.409	1.892	-2.039	0.045
ENSG00000259423	RP11-265N7.2	-0.733	-2.910	-2.039	0.045
ENSG00000100300	TSPO	0.549	-0.702	2.039	0.045
ENSG00000267523	CTD-2537I9.12	-0.639	-1.143	-2.038	0.045
ENSG00000135678	CPM	-0.364	3.507	-2.038	0.045
ENSG00000056097	ZFR	0.165	7.693	2.037	0.045
ENSG00000224738	AC099850.1	0.391	-0.932	2.037	0.045
ENSG00000184321	OR51J1	-1.093	-2.633	-2.037	0.045
ENSG00000231840	AC073342.12	-0.640	-1.484	-2.036	0.045
ENSG00000108309	RUNDC3A	0.162	7.194	2.035	0.045
ENSG00000131471	AOC3	-0.644	-0.964	-2.035	0.045
ENSG00000221995	TIAF1	-0.266	3.643	-2.035	0.045
ENSG00000226609	RP11-276E15.4	0.746	-2.433	2.035	0.045
ENSG00000225767	RP5-850O15.3	-0.352	0.781	-2.034	0.045
ENSG00000149503	INCENP	0.153	4.258	2.033	0.045

ENSG00000285750	#N/A	-0.395	0.627	-2.033	0.045
ENSG00000177879	AP3S1	0.202	5.731	2.032	0.045
ENSG00000272849	RP11-347I19.8	-0.473	0.104	-2.031	0.045
ENSG00000170776	AKAP13	0.168	6.656	2.031	0.046
ENSG00000254806	SYS1-DBNDD2	-0.697	-2.397	-2.030	0.046
ENSG00000175820	CCDC168	-0.454	2.959	-2.030	0.046
ENSG00000234982	RP11-280O24.3	-0.520	-1.294	-2.030	0.046
ENSG00000250138	RP11-848G14.5	-0.541	-0.021	-2.029	0.046
ENSG00000260267	RP11-452L6.5	-0.207	1.885	-2.028	0.046
ENSG00000186340	THBS2	-0.326	4.449	-2.028	0.046
ENSG00000274403	CTD-2036P10.6	-0.476	-0.352	-2.027	0.046
ENSG00000081154	PCNP	0.177	6.618	2.027	0.046
ENSG00000213563	C8orf82	-0.194	2.003	-2.026	0.046
ENSG00000272150	NBPF25P	-0.305	2.449	-2.026	0.046
ENSG00000277575	RP11-292B8.2	-0.693	-1.258	-2.025	0.046
ENSG00000087903	RFX2	-0.221	2.512	-2.025	0.046
ENSG00000273597	RP11-392A14.9	-0.682	-1.475	-2.025	0.046
ENSG00000142871	CYR61	-0.481	2.359	-2.024	0.046
ENSG00000189233	NUGGC	0.710	-2.351	2.024	0.046
ENSG00000224635	RP4-564F22.5	-0.559	-0.472	-2.023	0.046
ENSG00000147419	CCDC25	0.217	6.415	2.023	0.046
ENSG00000207117	Y_RNA	-0.551	-1.700	-2.022	0.046
ENSG00000255423	EBLN2	-0.299	1.719	-2.022	0.046
ENSG00000115685	PPP1R7	0.206	5.744	2.021	0.047
ENSG00000225739	NPM1P18	-0.484	-0.402	-2.021	0.047
ENSG00000136267	DGKB	0.200	7.855	2.021	0.047
ENSG00000258837	CTD-2566J3.1	-0.732	-2.505	-2.021	0.047
ENSG00000259291	RP11-617F23.1	0.196	4.879	2.020	0.047
ENSG00000170537	TMC7	-0.323	2.910	-2.020	0.047
ENSG00000285816	#N/A	-0.623	-1.622	-2.019	0.047
ENSG00000239828	RP11-446H18.5	-0.315	2.680	-2.019	0.047
ENSG00000259008	RP11-932A10.1	-0.658	-0.836	-2.019	0.047
ENSG00000259322	RP11-762H8.1	-0.330	-0.017	-2.019	0.047
ENSG00000157870	FAM213B	0.216	4.573	2.018	0.047
ENSG00000259840	LA16c-380A1.1	-0.539	-1.283	-2.018	0.047
ENSG00000244215	LINC02016	0.618	-1.904	2.018	0.047
ENSG00000244346	RP11-531F16.3	-0.397	-0.153	-2.017	0.047
ENSG00000111087	GLI1	0.458	-0.256	2.017	0.047
ENSG00000202240	RNU6-737P	0.871	-2.396	2.016	0.047
ENSG00000178175	ZNF366	0.497	1.015	2.015	0.047
ENSG00000228838	RP4-784A16.2	-0.919	-2.528	-2.015	0.047
ENSG00000160584	SIK3	-0.141	7.333	-2.014	0.047
ENSG00000078369	GNB1	0.135	8.414	2.014	0.047
ENSG00000072694	FCGR2B	0.728	-1.383	2.014	0.047
ENSG00000119661	DNAL1	0.213	6.464	2.013	0.047
ENSG00000240445	FOXO3B	-0.447	1.903	-2.013	0.047
ENSG00000196814	MVB12B	-0.177	5.306	-2.013	0.047

ENSG00000230026	RP11-382D8.5	-0.517	-0.830	-2.012	0.047
ENSG00000111424	VDR	0.640	-1.915	2.011	0.048
ENSG00000258490	RP11-862P13.1	-0.359	0.314	-2.011	0.048
ENSG00000235436	DPY19L2P4	-0.298	2.166	-2.011	0.048
ENSG00000198755	RPL10A	0.351	5.429	2.010	0.048
ENSG00000127366	TAS2R5	-0.329	0.882	-2.009	0.048
ENSG00000125388	GRK4	0.152	4.782	2.009	0.048
ENSG00000243005	RN7SL16P	0.616	-2.011	2.009	0.048
ENSG00000143429	AC027612.6	-0.596	0.643	-2.006	0.048
ENSG00000251152	RP11-281P23.1	-0.677	-0.307	-2.004	0.048
ENSG00000254459	RP11-91P24.7	-0.586	-1.719	-2.004	0.048
ENSG00000200807	RNU1-32P	0.990	-3.286	2.004	0.048
ENSG00000135124	P2RX4	0.174	3.033	2.004	0.048
ENSG00000270171	RP11-338N10.1	-0.411	0.764	-2.002	0.049
ENSG00000155897	ADCY8	0.148	4.533	2.002	0.049
ENSG00000165702	GFI1B	0.541	-1.418	2.002	0.049
ENSG00000260528	FAM157C	-0.784	0.339	-2.001	0.049
ENSG00000115257	PCSK4	-0.347	0.289	-2.000	0.049
ENSG00000200462	RNU6-727P	0.925	-2.348	2.000	0.049
ENSG00000154240	CEP112	0.177	5.186	2.000	0.049
ENSG00000285218	#N/A	-0.869	-2.228	-1.999	0.049
ENSG00000140577	CRTC3	-0.154	4.844	-1.998	0.049
ENSG00000176771	NCKAP5	-0.274	4.820	-1.997	0.049
ENSG00000273151	RP11-449P15.2	-0.332	2.264	-1.997	0.049
ENSG00000166923	GREM1	-0.456	4.522	-1.997	0.049
ENSG00000269696	AC007228.11	-0.245	1.894	-1.995	0.049
ENSG00000246477	AF131216.6	-0.229	2.081	-1.994	0.049
ENSG00000237357	RP11-475I24.3	-0.560	-0.783	-1.994	0.049
ENSG00000225345	SNX18P3	-0.469	2.715	-1.993	0.050
ENSG00000225724	LL22NC03-80A10.1	-0.527	-1.462	-1.991	0.050
ENSG00000237732	AC010980.2	0.207	1.828	1.991	0.050
ENSG00000278338	VWA8-AS1	-0.486	-0.962	-1.990	0.050
ENSG00000285551	#N/A	-0.443	0.063	-1.990	0.050

vmPFC

gene_id	gene_name	logFC	AveExpr	t	P.Value
ENSG00000004059	ARF5	0.280	3.768	2.017	0.047
ENSG00000004799	PDK4	-0.517	5.725	-2.334	0.022
ENSG00000005175	RPAP3	0.173	5.480	2.038	0.045
ENSG00000005194	CIAPIN1	0.268	3.949	2.506	0.014
ENSG00000005471	ABCB4	0.362	1.077	2.086	0.040
ENSG00000006453	BAIAP2L1	-0.429	0.690	-2.734	0.008
ENSG00000007168	PAFAH1B1	0.240	8.658	2.211	0.030
ENSG00000007372	PAX6	-0.324	4.892	-2.198	0.031
ENSG00000007908	SELE	-1.311	-0.173	-2.538	0.013
ENSG00000008277	ADAM22	0.232	7.920	2.068	0.042
ENSG00000008283	CYB561	0.272	4.689	2.519	0.014
ENSG00000010379	SLC6A13	-0.533	2.369	-2.349	0.021
ENSG00000011405	PIK3C2A	-0.303	7.883	-2.477	0.015
ENSG00000013293	SLC7A14	0.350	7.388	2.204	0.031
ENSG00000014641	MDH1	0.287	7.158	1.994	0.050
ENSG00000016391	CHDH	-0.246	4.460	-2.112	0.038
ENSG00000019186	CYP24A1	1.277	-0.751	2.463	0.016
ENSG00000024526	DEPDC1	0.473	0.414	2.098	0.039
ENSG00000025423	HSD17B6	-0.413	3.775	-2.205	0.031
ENSG00000025770	NCAPH2	0.246	3.670	2.202	0.031
ENSG00000025800	KPNA6	0.180	6.690	2.147	0.035
ENSG00000026025	VIM	-0.569	5.755	-2.402	0.019
ENSG00000031081	ARHGAP31	-0.290	5.760	-2.637	0.010
ENSG00000033030	ZCCHC8	-0.169	4.515	-2.031	0.046
ENSG00000035141	FAM136A	0.319	3.769	2.315	0.023
ENSG00000035403	VCL	-0.216	5.677	-2.020	0.047
ENSG00000038274	MAT2B	0.230	6.031	2.155	0.034
ENSG00000040531	CTNS	-0.315	2.590	-2.282	0.025
ENSG00000041353	RAB27B	0.546	6.495	2.076	0.041
ENSG00000041515	MYO16	0.395	5.153	2.222	0.029
ENSG00000042286	AIFM2	-0.275	3.029	-2.795	0.007
ENSG00000043514	TRIT1	0.180	4.855	2.025	0.046
ENSG00000047365	ARAP2	-0.225	8.092	-2.106	0.039
ENSG00000048544	MRPS10	0.214	4.921	2.131	0.036
ENSG00000049323	LTBP1	-0.481	4.454	-2.060	0.043
ENSG00000050405	LIMA1	-0.238	4.877	-2.348	0.021
ENSG00000050438	SLC4A8	0.357	7.267	3.215	0.002
ENSG00000050628	PTGER3	1.011	4.156	2.750	0.007
ENSG00000054803	CBLN4	0.404	4.008	2.025	0.046
ENSG00000055609	KMT2C	-0.137	8.813	-2.132	0.036
ENSG00000057663	ATG5	0.228	5.120	2.017	0.047
ENSG00000057757	PITHD1	0.341	6.291	2.502	0.014
ENSG00000058091	CDK14	0.389	7.282	2.294	0.025
ENSG00000060656	PTPRU	0.299	4.272	2.330	0.022
ENSG00000060749	QSER1	-0.171	6.954	-2.254	0.027

ENSG00000064655	EYA2	-0.459	3.059	-2.529	0.014
ENSG00000065457	ADAT1	0.180	4.341	2.381	0.020
ENSG00000065526	SPEN	-0.207	8.109	-2.695	0.009
ENSG00000065559	MAP2K4	0.364	7.313	2.396	0.019
ENSG00000065665	SEC61A2	0.235	5.155	2.451	0.017
ENSG00000066027	PPP2R5A	-0.209	5.345	-2.337	0.022
ENSG00000066583	ISOC1	0.272	3.801	2.198	0.031
ENSG00000067221	STOML1	0.312	3.687	2.319	0.023
ENSG00000067900	ROCK1	-0.226	6.907	-2.660	0.010
ENSG00000068615	REEP1	0.357	6.415	3.045	0.003
ENSG00000070501	POLB	0.197	3.866	2.203	0.031
ENSG00000070718	AP3M2	0.273	6.314	2.466	0.016
ENSG00000070831	CDC42	0.443	5.074	2.327	0.023
ENSG00000071564	TCF3	-0.256	3.036	-2.151	0.035
ENSG00000071909	MYO3B	0.443	0.591	2.005	0.048
ENSG00000071967	CYBRD1	-0.456	6.065	-2.062	0.043
ENSG00000072736	NFATC3	-0.194	5.177	-2.369	0.020
ENSG00000073712	FERMT2	-0.257	6.462	-2.249	0.027
ENSG00000073969	NSF	0.371	7.839	2.049	0.044
ENSG00000074181	NOTCH3	-0.398	3.246	-2.226	0.029
ENSG00000075303	SLC25A40	0.265	4.672	2.799	0.006
ENSG00000075420	FNDC3B	-0.210	5.758	-2.170	0.033
ENSG00000075673	ATP12A	0.953	-2.266	2.154	0.034
ENSG00000075914	EXOSC7	0.157	3.963	2.417	0.018
ENSG00000076067	RBMS2	-0.278	4.478	-2.133	0.036
ENSG00000077152	UBE2T	0.537	2.176	2.661	0.010
ENSG00000077498	TYR	1.034	-3.329	2.109	0.038
ENSG00000078246	TULP3	-0.355	4.032	-2.291	0.025
ENSG00000078668	VDAC3	0.215	5.936	2.041	0.045
ENSG00000079308	TNS1	-0.438	6.098	-2.059	0.043
ENSG00000079335	CDC14A	-0.347	3.723	-2.282	0.025
ENSG00000080603	SRCAP	-0.227	2.307	-2.251	0.027
ENSG00000081138	CDH7	0.378	4.684	2.315	0.023
ENSG00000081377	CDC14B	-0.228	5.767	-2.085	0.040
ENSG00000081818	PCDHB4	-0.334	3.828	-2.361	0.021
ENSG00000082397	EPB41L3	0.259	8.016	2.051	0.044
ENSG00000083223	ZCCHC6	-0.151	6.117	-2.475	0.016
ENSG00000083535	PIBF1	-0.179	5.373	-2.505	0.014
ENSG00000083635	NUFIP1	0.196	3.775	2.153	0.034
ENSG00000084072	PPIE	0.173	4.107	2.134	0.036
ENSG00000084093	REST	-0.363	5.129	-2.025	0.046
ENSG00000085365	SCAMP1	0.237	7.095	2.036	0.045
ENSG00000086062	B4GALT1	-0.487	2.433	-2.499	0.015
ENSG00000086289	EPDR1	0.540	6.256	3.076	0.003
ENSG00000086300	SNX10	0.502	6.250	2.442	0.017
ENSG00000087448	KLHL42	0.236	6.729	2.387	0.019
ENSG00000089006	SNX5	-0.169	5.586	-2.023	0.047

ENSG00000089818	NECAP1	0.347	6.714	2.284	0.025
ENSG00000091436	AC013461.1	-0.387	5.138	-2.027	0.046
ENSG00000091592	NLRP1	0.282	6.842	2.236	0.028
ENSG00000091664	SLC17A6	1.660	3.100	3.833	0.000
ENSG00000091879	ANGPT2	-0.539	3.517	-2.149	0.035
ENSG00000091972	CD200	0.356	6.026	2.817	0.006
ENSG00000092094	OSGEP	0.279	4.802	2.319	0.023
ENSG00000095059	DHPS	-0.328	1.175	-2.120	0.037
ENSG00000095485	CWF19L1	0.302	3.807	3.215	0.002
ENSG00000095587	TLL2	0.353	2.983	2.887	0.005
ENSG00000095637	SORBS1	-0.231	8.974	-2.138	0.036
ENSG00000096092	TMEM14A	0.388	4.350	2.675	0.009
ENSG00000099246	RAB18	0.188	6.982	2.135	0.036
ENSG00000099875	MKNK2	-0.335	3.690	-2.327	0.023
ENSG00000099999	RNF215	0.207	3.340	2.333	0.022
ENSG00000100028	SNRPD3	0.204	5.229	2.143	0.035
ENSG00000100138	SNU13	0.324	5.818	2.762	0.007
ENSG00000100216	TOMM22	0.267	5.328	2.154	0.034
ENSG00000100227	POLDIP3	-0.179	5.051	-2.125	0.037
ENSG00000100300	TSPO	-0.545	-0.242	-2.316	0.023
ENSG00000100320	RBFOX2	0.236	8.150	2.100	0.039
ENSG00000100345	MYH9	-0.294	7.109	-2.438	0.017
ENSG00000100362	PVALB	1.132	-0.510	2.267	0.026
ENSG00000100387	RBX1	0.180	3.886	2.398	0.019
ENSG00000100483	VCPKMT	0.239	2.909	2.154	0.034
ENSG00000100554	ATP6V1D	0.302	5.975	2.002	0.049
ENSG00000100580	TMED8	0.268	6.838	3.116	0.003
ENSG00000100813	ACIN1	-0.143	6.839	-2.067	0.042
ENSG00000100814	CCNB1IP1	0.220	4.000	2.202	0.031
ENSG00000100867	DHRS2	0.937	-1.417	2.251	0.027
ENSG00000100941	PNN	-0.175	6.908	-2.141	0.036
ENSG00000101079	NDRG3	0.283	6.860	2.108	0.038
ENSG00000101084	C20orf24	0.353	0.570	2.188	0.032
ENSG00000101144	BMP7	-0.342	4.964	-2.134	0.036
ENSG00000101294	HM13	-0.144	4.599	-2.308	0.024
ENSG00000101447	FAM83D	-0.494	0.072	-2.000	0.049
ENSG00000101546	RBFA	0.178	3.647	2.039	0.045
ENSG00000102466	FGF14	0.291	6.363	2.271	0.026
ENSG00000102781	KATNAL1	0.210	6.832	2.202	0.031
ENSG00000102871	TRADD	0.386	0.460	2.029	0.046
ENSG00000102893	PHKB	0.122	6.875	2.040	0.045
ENSG00000102924	CBLN1	1.190	2.108	4.484	0.000
ENSG00000102984	ZNF821	0.432	2.666	3.778	0.000
ENSG00000103152	MPG	0.316	1.207	2.101	0.039
ENSG00000103160	HSDL1	0.238	5.777	2.260	0.027
ENSG00000103245	NARFL	0.205	3.081	2.013	0.048
ENSG00000103353	UBFD1	0.181	6.241	2.092	0.040

ENSG00000103591	AAGAB	0.261	5.756	2.337	0.022
ENSG00000103769	RAB11A	0.244	7.444	2.160	0.034
ENSG00000103852	TTC23	-0.241	3.638	-2.125	0.037
ENSG00000104067	TJP1	-0.231	8.453	-2.210	0.030
ENSG00000104164	BLOC1S6	0.230	6.761	2.060	0.043
ENSG00000104231	ZFAND1	0.313	5.005	2.854	0.006
ENSG00000104381	GDAP1	0.295	7.473	2.325	0.023
ENSG00000104388	RAB2A	0.265	7.006	2.405	0.019
ENSG00000104413	ESRP1	0.724	-1.016	2.104	0.039
ENSG00000104435	STMN2	0.556	8.734	2.563	0.012
ENSG00000104497	SNX16	0.367	4.117	3.095	0.003
ENSG00000104723	TUSC3	0.222	6.035	2.065	0.042
ENSG00000104863	LIN7B	0.366	2.439	2.343	0.022
ENSG00000104872	PIH1D1	0.290	4.168	2.297	0.024
ENSG00000105072	C19orf44	-0.264	2.959	-2.127	0.037
ENSG00000105173	CCNE1	0.322	1.385	2.400	0.019
ENSG00000105255	FSD1	0.375	3.501	2.445	0.017
ENSG00000105509	HAS1	0.520	-0.409	2.034	0.045
ENSG00000105722	ERF	-0.355	3.542	-2.387	0.019
ENSG00000105784	RUNDC3B	0.276	5.429	2.426	0.018
ENSG00000105793	GTPBP10	0.204	5.420	2.114	0.038
ENSG00000105989	WNT2	0.966	-0.045	2.795	0.007
ENSG00000106018	VIPR2	-0.440	0.975	-2.325	0.023
ENSG00000106236	NPTX2	0.595	4.370	2.229	0.029
ENSG00000106341	PPP1R17	0.633	0.823	2.456	0.016
ENSG00000106541	AGR2	1.052	-2.120	2.722	0.008
ENSG00000106723	SPIN1	0.258	7.832	2.456	0.016
ENSG00000106785	TRIM14	-0.207	3.759	-2.164	0.034
ENSG00000106789	CORO2A	0.363	3.230	2.616	0.011
ENSG00000106868	SUSD1	0.278	5.198	2.058	0.043
ENSG00000107821	KAZALD1	0.544	-0.547	2.089	0.040
ENSG00000107882	SUFU	-0.183	3.820	-2.274	0.026
ENSG00000108389	MTMR4	0.180	6.734	2.238	0.028
ENSG00000108395	TRIM37	0.313	7.649	2.009	0.048
ENSG00000108448	TRIM16L	0.240	2.620	2.145	0.035
ENSG00000108557	RAI1	-0.224	5.176	-2.003	0.049
ENSG00000108590	MED31	0.376	2.078	2.692	0.009
ENSG00000108666	C17orf75	0.233	4.694	2.063	0.043
ENSG00000108786	HSD17B1	-0.259	2.494	-1.997	0.049
ENSG00000108826	MRPL27	0.267	3.713	2.902	0.005
ENSG00000108852	MPP2	0.206	4.978	2.220	0.029
ENSG00000108854	SMURF2	-0.192	5.906	-2.213	0.030
ENSG00000109171	SLAIN2	-0.158	6.033	-2.081	0.041
ENSG00000109184	DCUN1D4	0.169	6.853	2.303	0.024
ENSG00000109255	NMU	0.874	-1.059	2.166	0.033
ENSG00000109320	NFKB1	-0.256	4.544	-3.056	0.003
ENSG00000109436	TBC1D9	0.369	6.488	2.607	0.011

ENSG00000109686	SH3D19	-0.249	5.792	-2.201	0.031
ENSG00000109787	KLF3	-0.222	5.813	-2.236	0.028
ENSG00000109911	ELP4	-0.208	5.192	-2.226	0.029
ENSG00000110077	MS4A6A	-0.892	1.890	-2.262	0.027
ENSG00000110092	CCND1	-0.335	4.753	-2.419	0.018
ENSG00000110218	PANX1	0.279	4.918	2.057	0.043
ENSG00000110619	CARS	0.188	4.874	2.169	0.033
ENSG00000110693	SOX6	-0.198	6.494	-2.002	0.049
ENSG00000110852	CLEC2B	-0.742	1.184	-2.521	0.014
ENSG00000110975	SYT10	0.450	4.427	2.113	0.038
ENSG00000111052	LIN7A	0.320	6.178	2.160	0.034
ENSG00000111058	ACSS3	-0.341	4.992	-2.543	0.013
ENSG00000111110	PPM1H	0.279	6.551	2.041	0.045
ENSG00000111666	CHPT1	-0.195	6.083	-2.250	0.027
ENSG00000111711	GOLT1B	0.253	5.093	2.475	0.016
ENSG00000111726	CMAS	0.391	5.978	2.657	0.010
ENSG00000111817	DSE	-0.309	4.587	-2.316	0.023
ENSG00000111850	SMIM8	0.164	4.874	2.221	0.029
ENSG00000112293	GPLD1	0.283	4.119	2.732	0.008
ENSG00000112333	NR2E1	-0.295	4.292	-2.397	0.019
ENSG00000112977	DAP	-0.229	3.785	-2.506	0.014
ENSG00000113209	PCDHB5	-0.313	3.715	-2.791	0.007
ENSG00000113532	ST8SIA4	0.370	4.739	2.401	0.019
ENSG00000113597	TRAPPC13	0.182	5.411	2.038	0.045
ENSG00000113810	SMC4	-0.328	4.115	-3.085	0.003
ENSG00000113966	ARL6	0.318	4.975	2.435	0.017
ENSG00000114098	ARMC8	0.141	6.091	2.176	0.033
ENSG00000114120	SLC25A36	0.234	6.637	2.591	0.011
ENSG00000114166	KAT2B	-0.260	6.623	-2.117	0.037
ENSG00000114279	FGF12	0.435	7.754	2.088	0.040
ENSG00000114405	C3orf14	0.278	5.133	2.000	0.049
ENSG00000114573	ATP6V1A	0.393	8.435	2.389	0.019
ENSG00000114738	MAPKAPK3	-0.338	3.089	-2.284	0.025
ENSG00000114784	EIF1B	0.206	5.100	2.046	0.044
ENSG00000114790	ARHGEF26	-0.290	5.599	-2.023	0.047
ENSG00000115170	ACVR1	-0.250	4.043	-2.077	0.041
ENSG00000115718	PROC	0.560	-0.665	2.580	0.012
ENSG00000115762	PLEKHB2	0.274	7.267	2.074	0.041
ENSG00000115828	QPCT	0.885	1.969	3.554	0.001
ENSG00000115840	SLC25A12	0.295	6.219	2.443	0.017
ENSG00000116005	PCYOX1	0.227	6.796	2.188	0.032
ENSG00000116044	NFE2L2	-0.348	5.719	-2.576	0.012
ENSG00000116120	FARSB	0.252	5.589	2.087	0.040
ENSG00000116127	ALMS1	-0.222	8.072	-2.933	0.004
ENSG00000116132	PRRX1	-0.307	5.139	-2.133	0.036
ENSG00000116147	TNR	-0.207	9.094	-2.075	0.041
ENSG00000116285	ERRFI1	-0.199	4.933	-2.096	0.039

ENSG00000116675	DNAJC6	0.321	8.780	2.132	0.036
ENSG00000116750	UCHL5	0.215	6.172	2.058	0.043
ENSG00000116815	CD58	-0.474	1.238	-2.642	0.010
ENSG00000117153	KLHL12	0.211	5.733	2.046	0.044
ENSG00000117308	GALE	0.298	1.987	2.217	0.030
ENSG00000117360	PRPF3	-0.173	5.827	-2.145	0.035
ENSG00000117758	STX12	0.192	6.887	2.145	0.035
ENSG00000117862	TXNDC12	0.300	2.105	2.460	0.016
ENSG00000117906	RCN2	0.225	6.155	2.413	0.018
ENSG00000118058	KMT2A	-0.116	8.271	-2.103	0.039
ENSG00000118276	B4GALT6	0.285	6.065	2.037	0.045
ENSG00000118292	C1orf54	-0.350	1.358	-2.027	0.046
ENSG00000118308	LRMP	0.422	4.342	2.009	0.048
ENSG00000118402	ELOVL4	0.262	5.225	2.033	0.046
ENSG00000118680	MYL12B	0.283	5.740	2.100	0.039
ENSG00000119231	SENP5	0.173	6.643	2.227	0.029
ENSG00000119630	PGF	0.634	-0.577	2.032	0.046
ENSG00000119681	LTBP2	-0.398	3.149	-2.355	0.021
ENSG00000119711	ALDH6A1	-0.246	7.564	-1.994	0.050
ENSG00000119760	SUPT7L	0.156	6.073	2.970	0.004
ENSG00000119801	YPEL5	0.292	6.851	2.508	0.014
ENSG00000119938	PPP1R3C	-0.333	6.079	-2.338	0.022
ENSG00000119965	C10orf88	0.270	3.613	2.510	0.014
ENSG00000120129	DUSP1	-0.604	4.316	-2.052	0.044
ENSG00000120265	PCMT1	0.321	6.610	2.067	0.042
ENSG00000120440	TTLL2	0.950	-1.377	3.201	0.002
ENSG00000120690	ELF1	-0.411	4.538	-2.453	0.016
ENSG00000120756	PLS1	0.349	3.081	2.008	0.048
ENSG00000121022	COPS5	0.200	4.829	2.497	0.015
ENSG00000121039	RDH10	-0.466	3.093	-2.314	0.023
ENSG00000121281	ADCY7	0.399	1.983	2.206	0.030
ENSG00000121764	HCRTR1	0.855	-1.679	2.339	0.022
ENSG00000122025	FLT3	0.764	1.983	3.175	0.002
ENSG00000122257	RBBP6	-0.177	6.885	-2.964	0.004
ENSG00000122420	PTGFR	0.589	1.456	2.168	0.033
ENSG00000122557	HERPUD2	-0.129	5.232	-2.425	0.018
ENSG00000122584	NXPH1	0.351	4.842	2.118	0.037
ENSG00000122786	CALD1	-0.394	6.315	-2.413	0.018
ENSG00000122873	CISD1	0.329	4.885	2.315	0.023
ENSG00000123080	CDKN2C	-0.373	1.583	-2.031	0.046
ENSG00000123416	TUBA1B	0.378	7.106	2.229	0.029
ENSG00000124659	TBCC	0.331	3.598	2.543	0.013
ENSG00000124733	MEA1	0.298	4.936	2.057	0.043
ENSG00000124802	EEF1E1	0.347	2.205	2.386	0.020
ENSG00000125148	MT2A	-0.540	4.532	-2.322	0.023
ENSG00000125246	CLYBL	-0.318	4.064	-2.393	0.019
ENSG00000125740	FOSB	-0.820	2.412	-2.330	0.022

ENSG00000125821	DTD1	0.219	3.322	2.323	0.023
ENSG00000125827	TMX4	0.305	7.752	2.479	0.015
ENSG00000126653	NSRP1	-0.285	5.378	-2.201	0.031
ENSG00000126785	RHOJ	-0.400	3.254	-3.262	0.002
ENSG00000127325	BEST3	-0.529	1.327	-2.581	0.012
ENSG00000127418	FGFRL1	-0.521	3.877	-2.320	0.023
ENSG00000127445	PIN1	0.338	5.599	2.108	0.038
ENSG00000127955	GNAI1	0.230	6.507	2.078	0.041
ENSG00000128607	KLHDC10	0.228	6.776	2.536	0.013
ENSG00000128654	MTX2	0.252	4.540	1.993	0.050
ENSG00000128739	SNRPN	0.268	6.187	2.132	0.036
ENSG00000128965	CHAC1	0.549	0.964	2.481	0.015
ENSG00000129084	PSMA1	0.304	3.595	2.862	0.005
ENSG00000129116	PALLD	-0.395	5.933	-2.412	0.018
ENSG00000129518	EAPP	0.239	4.758	2.332	0.022
ENSG00000130402	ACTN4	-0.243	6.028	-2.166	0.033
ENSG00000130520	LSM4	0.279	4.677	2.275	0.026
ENSG00000130699	TAF4	-0.191	3.845	-2.229	0.029
ENSG00000131023	LATS1	-0.139	6.376	-2.680	0.009
ENSG00000131196	NFATC1	-0.502	1.785	-2.077	0.041
ENSG00000131779	PEX11B	0.290	4.196	2.060	0.043
ENSG00000131871	VIMP	-0.231	3.710	-2.027	0.046
ENSG00000131981	LGALS3	-0.442	3.304	-2.722	0.008
ENSG00000132326	PER2	-0.258	5.065	-2.098	0.039
ENSG00000132424	PNISR	-0.117	8.401	-2.269	0.026
ENSG00000132622	HSPA12B	0.492	1.144	2.168	0.033
ENSG00000132669	RIN2	-0.277	5.899	-1.998	0.049
ENSG00000132671	SSTR4	0.719	-1.574	2.096	0.039
ENSG00000132819	RBM38	-0.385	1.869	-2.073	0.042
ENSG00000132846	ZBED3	-0.237	3.653	-2.278	0.026
ENSG00000132932	ATP8A2	0.306	7.507	2.122	0.037
ENSG00000133083	DCLK1	0.302	9.039	2.239	0.028
ENSG00000133392	MYH11	-1.002	3.544	-2.015	0.047
ENSG00000133703	KRAS	0.245	6.583	2.093	0.040
ENSG00000133731	IMPA1	0.294	5.349	2.345	0.022
ENSG00000133789	SWAP70	-0.235	5.681	-2.178	0.032
ENSG00000133962	CATSPERB	0.595	-0.701	2.441	0.017
ENSG00000133985	TTC9	0.304	5.117	2.200	0.031
ENSG00000134042	MRO	-0.391	6.602	-2.543	0.013
ENSG00000134070	IRAK2	0.414	2.553	2.493	0.015
ENSG00000134115	CNTN6	0.470	3.823	2.230	0.029
ENSG00000134375	TIMM17A	0.360	4.385	2.083	0.041
ENSG00000134443	GRP	1.051	-0.409	2.114	0.038
ENSG00000134684	YARS	0.272	6.174	2.344	0.022
ENSG00000134748	PRPF38A	-0.204	5.881	-2.357	0.021
ENSG00000135002	RFK	0.437	5.779	2.679	0.009
ENSG00000135318	NT5E	-0.269	4.860	-2.073	0.042

ENSG00000135625	EGR4	-0.659	0.495	-2.091	0.040
ENSG00000135775	COG2	-0.136	5.027	-2.035	0.045
ENSG00000135862	LAMC1	-0.321	5.509	-3.419	0.001
ENSG00000135953	MFSD9	0.225	2.419	2.226	0.029
ENSG00000135960	EDAR	-0.713	-1.180	-2.129	0.036
ENSG00000136040	PLXNC1	0.304	6.432	3.230	0.002
ENSG00000136159	NUDT15	0.223	3.060	1.995	0.050
ENSG00000136457	CHAD	-0.662	0.282	-3.230	0.002
ENSG00000136710	CCDC115	0.216	4.081	2.122	0.037
ENSG00000136718	IMP4	0.248	4.972	2.258	0.027
ENSG00000136826	KLF4	-0.837	1.911	-2.839	0.006
ENSG00000136938	ANP32B	-0.451	4.419	-2.034	0.045
ENSG00000136982	DSCC1	-0.355	1.292	-2.024	0.046
ENSG00000137076	TLN1	-0.317	6.531	-2.188	0.032
ENSG00000137267	TUBB2A	0.279	6.812	2.069	0.042
ENSG00000137331	IER3	-0.791	0.576	-3.047	0.003
ENSG00000137507	LRRC32	-0.706	1.348	-2.525	0.014
ENSG00000137693	YAP1	-0.438	5.471	-2.121	0.037
ENSG00000137700	SLC37A4	0.281	2.372	2.558	0.013
ENSG00000137842	TMEM62	0.184	4.104	2.579	0.012
ENSG00000137860	SLC28A2	-0.781	1.596	-2.234	0.028
ENSG00000138078	PREPL	0.269	9.057	2.042	0.045
ENSG00000138079	SLC3A1	0.399	1.029	2.074	0.041
ENSG00000138207	RBP4	0.348	3.367	2.195	0.031
ENSG00000138448	ITGAV	-0.244	7.765	-2.133	0.036
ENSG00000138663	COPS4	0.326	5.644	2.410	0.018
ENSG00000138669	PRKG2	0.329	4.044	2.093	0.040
ENSG00000138698	RAP1GDS1	0.260	6.442	2.041	0.045
ENSG00000138757	G3BP2	0.346	8.198	2.453	0.016
ENSG00000138769	CDKL2	0.282	4.932	2.669	0.009
ENSG00000139112	GABARAPL1	0.434	7.939	2.559	0.012
ENSG00000139163	ETNK1	0.218	7.129	2.072	0.042
ENSG00000139287	TPH2	0.661	0.577	2.938	0.004
ENSG00000139352	ASCL1	-0.381	3.258	-2.031	0.046
ENSG00000139620	KANSL2	0.175	3.806	2.253	0.027
ENSG00000139726	DENR	0.228	6.055	2.017	0.047
ENSG00000139800	ZIC5	-0.650	2.400	-2.642	0.010
ENSG00000139910	NOVA1	0.232	6.971	2.558	0.013
ENSG00000140015	KCNH5	0.569	5.496	2.023	0.047
ENSG00000140090	SLC24A4	-0.388	5.806	-2.995	0.004
ENSG00000140092	FBLN5	-0.397	3.095	-2.131	0.036
ENSG00000140280	LYSMD2	0.260	3.479	2.031	0.046
ENSG00000140287	HDC	1.756	-1.193	2.370	0.020
ENSG00000140299	BNIP2	-0.279	6.106	-2.034	0.045
ENSG00000140443	IGF1R	-0.142	6.886	-2.019	0.047
ENSG00000140497	SCAMP2	-0.308	3.475	-2.330	0.022
ENSG00000140577	CRTC3	-0.204	5.066	-2.238	0.028

ENSG00000140937	CDH11	-0.193	6.296	-2.138	0.036
ENSG00000140948	ZCCHC14	-0.168	6.034	-2.520	0.014
ENSG00000141232	TOB1	-0.380	5.131	-2.184	0.032
ENSG00000141433	ADCYAP1	1.369	3.772	3.401	0.001
ENSG00000141505	ASGR1	0.544	0.412	2.578	0.012
ENSG00000141905	NFIC	-0.303	5.662	-2.628	0.010
ENSG00000142168	SOD1	0.276	7.462	2.036	0.045
ENSG00000142178	SIK1	-1.111	0.255	-2.575	0.012
ENSG00000142252	GEMIN7	0.267	1.477	2.760	0.007
ENSG00000142530	FAM71E1	0.547	0.443	2.499	0.015
ENSG00000142611	PRDM16	-0.485	4.027	-2.477	0.015
ENSG00000142686	C1orf216	0.416	6.223	2.217	0.030
ENSG00000142867	BCL10	-0.155	3.529	-2.210	0.030
ENSG00000142871	CYR61	-0.635	2.869	-2.382	0.020
ENSG00000143127	ITGA10	-0.448	1.302	-2.039	0.045
ENSG00000143153	ATP1B1	0.314	9.024	2.192	0.031
ENSG00000143158	MPC2	0.309	5.472	2.128	0.037
ENSG00000143194	MAEL	0.574	1.377	2.147	0.035
ENSG00000143303	RRNAD1	-0.255	2.115	-2.179	0.032
ENSG00000143318	CASQ1	0.619	3.097	2.069	0.042
ENSG00000143367	TUFT1	0.377	3.147	2.856	0.006
ENSG00000143552	NUP210L	-0.604	1.686	-2.286	0.025
ENSG00000144043	TEX261	-0.247	4.307	-2.023	0.047
ENSG00000144182	LIPT1	-0.310	2.031	-2.660	0.010
ENSG00000144285	SCN1A	0.339	7.177	2.460	0.016
ENSG00000144407	PTH2R	0.493	1.448	2.032	0.046
ENSG00000144559	TAMM41	-0.213	4.299	-2.389	0.019
ENSG00000144560	VGLL4	-0.215	4.969	-2.880	0.005
ENSG00000144635	DYNC1LI1	0.225	5.854	2.222	0.029
ENSG00000144674	GOLGA4	-0.148	8.235	-2.047	0.044
ENSG00000144802	NFKBIZ	-0.420	3.144	-2.077	0.041
ENSG00000144834	TAGLN3	0.468	7.239	2.090	0.040
ENSG00000145012	LPP	-0.490	7.031	-2.807	0.006
ENSG00000145191	EIF2B5	0.147	4.945	2.064	0.042
ENSG00000145354	CISD2	0.212	4.456	2.057	0.043
ENSG00000145358	DDIT4L	-0.751	2.022	-3.452	0.001
ENSG00000145416	MARCHF1	0.211	5.887	2.237	0.028
ENSG00000145476	CYP4V2	-0.265	6.057	-2.031	0.046
ENSG00000145545	SRD5A1	0.367	5.436	2.856	0.006
ENSG00000145555	MYO10	-0.304	7.210	-2.783	0.007
ENSG00000145692	BHMT	0.464	0.682	2.167	0.033
ENSG00000145708	CRHBP	0.722	3.645	2.475	0.016
ENSG00000145725	PPIP5K2	0.151	6.760	2.009	0.048
ENSG00000145730	PAM	0.304	7.321	2.208	0.030
ENSG00000145779	TNFAIP8	-0.818	0.564	-2.248	0.027
ENSG00000145817	YIPF5	0.258	5.125	2.406	0.019
ENSG00000145888	GLRA1	1.102	-0.501	2.354	0.021

ENSG00000146112	PPP1R18	-0.303	3.613	-2.018	0.047
ENSG00000146151	HMGCLL1	0.301	5.486	2.525	0.014
ENSG00000146411	SLC2A12	-0.216	4.257	-2.158	0.034
ENSG00000146648	EGFR	-0.445	6.314	-2.649	0.010
ENSG00000146833	TRIM4	-0.216	4.562	-2.440	0.017
ENSG00000147416	ATP6V1B2	0.351	7.809	2.230	0.029
ENSG00000147642	SYBU	0.211	6.326	2.213	0.030
ENSG00000147647	DPYS	0.562	1.513	2.971	0.004
ENSG00000148604	RGR	-0.755	2.536	-2.023	0.047
ENSG00000148680	HTR7	0.531	2.027	2.622	0.011
ENSG00000148700	ADD3	-0.274	8.588	-2.132	0.036
ENSG00000148730	EIF4EBP2	-0.211	7.293	-2.631	0.010
ENSG00000148734	NPFFR1	-0.519	3.276	-2.286	0.025
ENSG00000148841	ITPRIP	-0.360	1.817	-2.023	0.047
ENSG00000149054	ZNF215	0.340	1.913	2.054	0.043
ENSG00000149179	C11orf49	0.140	4.688	2.295	0.024
ENSG00000149313	AASDHPPT	0.293	6.530	2.265	0.026
ENSG00000149656	LINC00266-1	-0.534	0.611	-2.186	0.032
ENSG00000150361	KLHL1	0.658	3.010	2.496	0.015
ENSG00000150401	DCUN1D2	0.168	4.267	2.092	0.040
ENSG00000150457	LATS2	-0.430	2.474	-2.080	0.041
ENSG00000150594	ADRA2A	0.602	2.184	2.336	0.022
ENSG00000150768	DLAT	0.236	5.724	2.364	0.021
ENSG00000150779	TIMM8B	0.323	2.880	2.311	0.024
ENSG00000150787	PTS	0.299	4.060	2.273	0.026
ENSG00000151136	BTBD11	0.453	4.154	2.682	0.009
ENSG00000151303	RP11-96C23.13	-0.645	-0.835	-2.393	0.019
ENSG00000151491	EPS8	-0.340	5.450	-2.151	0.035
ENSG00000152128	TMEM163	0.296	2.774	2.793	0.007
ENSG00000152253	SPC25	0.675	0.770	2.362	0.021
ENSG00000152332	UHMK1	0.312	8.385	2.548	0.013
ENSG00000152556	PFKM	0.175	6.898	2.004	0.049
ENSG00000152580	IGSF10	0.400	3.447	2.546	0.013
ENSG00000152932	RAB3C	0.458	8.740	2.218	0.030
ENSG00000153130	SCOC	0.344	6.886	2.682	0.009
ENSG00000153291	SLC25A27	0.222	6.234	2.067	0.042
ENSG00000153446	C16orf89	-0.404	2.779	-2.945	0.004
ENSG00000153558	FBXL2	0.197	5.516	2.152	0.035
ENSG00000153902	LGI4	-0.267	5.297	-2.224	0.029
ENSG00000153922	CHD1	-0.199	6.380	-2.696	0.009
ENSG00000153956	CACNA2D1	0.338	7.493	2.021	0.047
ENSG00000153982	GDPD1	0.227	4.262	2.490	0.015
ENSG00000154328	NEIL2	0.257	4.625	2.632	0.010
ENSG00000154473	BUB3	0.205	5.266	2.664	0.009
ENSG00000154518	ATP5G3	0.268	5.087	2.233	0.028
ENSG00000154582	TCEB1	0.252	4.618	2.072	0.042
ENSG00000154734	ADAMTS1	-0.783	4.467	-2.235	0.028

ENSG00000154898	CCDC144CP	-0.348	6.010	-2.354	0.021
ENSG00000154975	CA10	0.494	5.597	2.028	0.046
ENSG00000155099	TMEM55A	0.256	4.808	2.763	0.007
ENSG00000155158	TTC39B	0.322	5.957	2.850	0.006
ENSG00000155858	LSM11	0.385	5.000	2.137	0.036
ENSG00000155906	RMND1	0.216	4.223	2.476	0.016
ENSG00000155970	MICU3	0.296	6.350	2.224	0.029
ENSG00000156253	RWDD2B	0.319	3.774	2.096	0.039
ENSG00000156650	KAT6B	-0.138	7.216	-2.230	0.029
ENSG00000156689	GLYATL2	0.552	-0.275	2.138	0.036
ENSG00000156873	PHKG2	0.188	3.083	2.057	0.043
ENSG00000157778	PSMG3	0.362	2.877	2.125	0.037
ENSG00000157933	SKI	-0.270	6.233	-3.061	0.003
ENSG00000158156	XKR8	-0.330	1.113	-2.162	0.034
ENSG00000158467	AHCYL2	-0.188	6.637	-2.382	0.020
ENSG00000158636	EMSY	-0.125	5.712	-2.259	0.027
ENSG00000158711	ELK4	-0.212	6.279	-2.665	0.009
ENSG00000158793	NIT1	0.149	3.517	2.102	0.039
ENSG00000158985	CDC42SE2	0.285	7.259	1.993	0.050
ENSG00000159374	M1AP	0.534	0.537	2.622	0.011
ENSG00000159388	BTG2	-0.775	4.564	-2.680	0.009
ENSG00000159658	EFCAB14	-0.244	7.315	-2.101	0.039
ENSG00000160310	PRMT2	0.162	6.371	2.071	0.042
ENSG00000160606	TLCD1	-0.349	1.378	-2.008	0.048
ENSG00000160752	FDPS	0.240	4.064	2.018	0.047
ENSG00000160888	IER2	-0.310	2.163	-2.259	0.027
ENSG00000160917	CPSF4	0.162	3.594	2.184	0.032
ENSG00000161021	MAML1	-0.201	4.911	-3.189	0.002
ENSG00000161647	MPP3	0.390	2.386	3.318	0.001
ENSG00000161888	SPC24	0.642	-0.464	2.171	0.033
ENSG00000162139	NEU3	0.157	5.006	2.044	0.044
ENSG00000162194	LBHD1	0.331	0.280	2.127	0.037
ENSG00000162383	SLC1A7	1.268	-1.853	2.204	0.031
ENSG00000162430	SEPN1	-0.299	4.299	-2.512	0.014
ENSG00000162599	NFIA	-0.313	8.043	-2.304	0.024
ENSG00000162636	FAM102B	0.382	6.791	2.011	0.048
ENSG00000162642	C1orf52	0.201	4.788	2.096	0.039
ENSG00000162670	BRINP3	0.236	5.633	2.149	0.035
ENSG00000162757	C1orf74	0.268	1.857	2.217	0.030
ENSG00000162769	FLVCR1	0.204	4.400	2.034	0.045
ENSG00000162882	HAAO	-0.442	0.819	-2.193	0.031
ENSG00000162910	MRPL55	0.374	2.896	2.327	0.023
ENSG00000162929	KIAA1841	0.239	5.218	3.194	0.002
ENSG00000163016	ALMS1P1	-0.350	2.062	-2.217	0.030
ENSG00000163053	SLC16A14	0.256	5.085	2.225	0.029
ENSG00000163110	PDLIM5	-0.349	6.758	-2.262	0.027
ENSG00000163155	LYSMD1	0.211	3.239	2.131	0.036

ENSG00000163273	NPPC	0.814	-0.471	2.450	0.017
ENSG00000163444	TMEM183A	0.338	5.714	2.237	0.028
ENSG00000163449	TMEM169	0.381	3.495	2.369	0.020
ENSG00000163453	IGFBP7	-0.481	4.381	-2.343	0.022
ENSG00000163535	SGO2	-0.229	2.788	-2.288	0.025
ENSG00000163539	CLASP2	0.227	9.035	2.285	0.025
ENSG00000163541	SUCLG1	0.169	5.126	2.006	0.048
ENSG00000163577	EIF5A2	0.299	4.304	2.294	0.025
ENSG00000163626	COX18	0.195	3.997	2.323	0.023
ENSG00000163655	GMPS	0.178	6.894	2.104	0.039
ENSG00000163659	TIPARP	-0.395	5.193	-2.105	0.039
ENSG00000163666	HESX1	0.492	-0.685	2.258	0.027
ENSG00000163684	RPP14	0.221	4.792	2.631	0.010
ENSG00000163710	PCOLCE2	0.513	1.218	2.165	0.034
ENSG00000163875	MEAF6	0.338	6.170	2.804	0.006
ENSG00000163888	CAMK2N2	0.345	1.536	1.999	0.049
ENSG00000163898	LIPH	0.372	0.882	2.056	0.043
ENSG00000163935	SFMBT1	0.151	3.976	2.330	0.022
ENSG00000163950	SLBP	0.275	4.703	2.321	0.023
ENSG00000164032	H2AFZ	0.302	4.335	2.054	0.043
ENSG00000164038	SLC9B2	0.270	5.333	2.382	0.020
ENSG00000164056	SPRY1	-0.263	3.288	-2.161	0.034
ENSG00000164062	APEH	0.179	4.732	2.154	0.034
ENSG00000164070	HSPA4L	0.313	7.138	2.490	0.015
ENSG00000164172	MOCS2	0.276	6.096	2.309	0.024
ENSG00000164190	NIPBL	-0.189	7.483	-2.881	0.005
ENSG00000164284	GRPEL2	0.277	4.886	2.394	0.019
ENSG00000164326	CARTPT	0.910	2.003	2.115	0.038
ENSG00000164484	TMEM200A	0.569	2.527	2.267	0.026
ENSG00000164542	KIAA0895	0.196	4.893	2.558	0.013
ENSG00000164615	CAMLG	0.237	5.277	2.047	0.044
ENSG00000164692	COL1A2	-0.618	2.364	-2.019	0.047
ENSG00000164741	DLC1	-0.208	6.640	-2.494	0.015
ENSG00000164761	TNFRSF11B	-0.806	1.329	-2.036	0.045
ENSG00000164885	CDK5	0.364	3.797	2.103	0.039
ENSG00000165152	TMEM246	0.314	6.357	2.241	0.028
ENSG00000165264	NDUFB6	0.275	3.551	2.206	0.030
ENSG00000165474	GJB2	-0.648	0.277	-2.583	0.012
ENSG00000165494	PCF11	-0.161	6.849	-2.174	0.033
ENSG00000165526	RPUSD4	0.193	3.851	2.252	0.027
ENSG00000165572	KBTBD6	0.319	6.108	2.956	0.004
ENSG00000165731	RET	0.385	2.828	1.992	0.050
ENSG00000165832	TRUB1	0.326	5.684	2.159	0.034
ENSG00000165895	ARHGAP42	-0.344	4.575	-3.130	0.002
ENSG00000165943	MOAP1	0.337	7.604	2.193	0.031
ENSG00000166111	SVOP	0.337	6.524	2.176	0.033
ENSG00000166123	GPT2	-0.199	6.019	-2.056	0.043

ENSG00000166199	ALKBH3	0.212	3.336	2.216	0.030
ENSG00000166402	TUB	0.186	7.459	2.101	0.039
ENSG00000166407	LMO1	0.556	-0.864	2.030	0.046
ENSG00000166444	ST5	-0.149	4.855	-2.132	0.036
ENSG00000166603	MC4R	0.620	1.728	2.627	0.010
ENSG00000166716	ZNF592	-0.138	5.508	-2.041	0.045
ENSG00000166840	GLYATL1	0.834	-1.361	3.017	0.003
ENSG00000166848	TERF2IP	0.249	7.869	2.125	0.037
ENSG00000166896	ATP23	0.413	1.686	3.013	0.004
ENSG00000166946	CCNDBP1	0.214	5.092	2.039	0.045
ENSG00000167005	NUDT21	0.246	7.437	2.151	0.035
ENSG00000167103	PIP5KL1	0.767	-0.444	2.958	0.004
ENSG00000167107	ACSF2	-0.302	2.544	-2.024	0.046
ENSG00000167552	TUBA1A	0.281	8.431	2.280	0.025
ENSG00000167601	AXL	-0.295	5.000	-2.578	0.012
ENSG00000167604	NFKBID	-0.468	0.851	-2.559	0.012
ENSG00000167642	SPINT2	0.363	4.923	2.444	0.017
ENSG00000167699	GLOD4	0.233	5.614	2.722	0.008
ENSG00000168092	PAFAH1B2	0.267	7.428	2.612	0.011
ENSG00000168309	FAM107A	-0.475	9.326	-2.996	0.004
ENSG00000168310	IRF2	-0.288	4.565	-2.769	0.007
ENSG00000168411	RFWD3	0.247	3.359	2.511	0.014
ENSG00000168461	RAB31	-0.252	7.202	-2.059	0.043
ENSG00000168772	CXXC4	0.338	5.445	3.067	0.003
ENSG00000168824	NSG1	0.426	5.461	2.035	0.045
ENSG00000168843	FSTL5	0.334	5.847	2.494	0.015
ENSG00000168958	MFF	0.154	6.017	2.166	0.033
ENSG00000169139	UBE2V2	0.295	6.634	1.997	0.049
ENSG00000169255	B3GALNT1	0.297	5.943	2.438	0.017
ENSG00000169330	KIAA1024	0.378	4.151	2.091	0.040
ENSG00000169371	SNUPN	0.219	3.173	2.260	0.027
ENSG00000169607	CKAP2L	0.658	-0.859	2.206	0.030
ENSG00000169689	STRA13	0.318	1.566	1.994	0.050
ENSG00000169750	RAC3	0.500	2.480	2.431	0.017
ENSG00000170315	UBB	0.290	6.924	2.836	0.006
ENSG00000170370	EMX2	-0.421	4.308	-2.664	0.009
ENSG00000170417	TMEM182	0.415	1.554	2.588	0.012
ENSG00000170439	METTL7B	-0.568	0.436	-2.212	0.030
ENSG00000170627	GTSF1	-1.473	-2.551	-2.841	0.006
ENSG00000170653	ATF7	-0.287	4.159	-2.035	0.045
ENSG00000170745	KCNS3	0.452	2.411	2.536	0.013
ENSG00000170786	SDR16C5	0.528	1.511	2.010	0.048
ENSG00000170899	GSTA4	0.215	5.227	2.453	0.016
ENSG00000170927	PKHD1	0.842	-1.298	2.235	0.028
ENSG00000170962	PDGFD	0.428	1.929	2.306	0.024
ENSG00000171103	TRMT61B	0.206	3.291	2.826	0.006
ENSG00000171174	RBKS	-0.227	2.206	-1.998	0.049

ENSG00000171303	KCNK3	0.395	3.248	2.265	0.026
ENSG00000171314	PGAM1	0.248	4.842	2.059	0.043
ENSG00000171421	MRPL36	0.360	1.662	2.315	0.023
ENSG00000171425	ZNF581	-0.396	0.283	-2.085	0.040
ENSG00000171467	ZNF318	-0.108	6.455	-2.081	0.041
ENSG00000171517	LPAR3	-0.829	2.632	-2.763	0.007
ENSG00000171634	BPTF	-0.152	8.554	-2.085	0.040
ENSG00000171714	ANO5	0.250	6.151	2.223	0.029
ENSG00000171970	ZNF57	0.260	1.831	2.036	0.045
ENSG00000172020	GAP43	0.444	7.914	2.157	0.034
ENSG00000172270	BSG	-0.267	6.381	-2.186	0.032
ENSG00000172292	CERS6	0.303	6.237	2.005	0.049
ENSG00000172301	COPRS	0.274	5.142	2.146	0.035
ENSG00000172348	RCAN2	0.427	7.221	2.322	0.023
ENSG00000172421	EFCAB3	1.272	-2.534	2.856	0.006
ENSG00000172586	CHCHD1	0.243	2.575	2.107	0.038
ENSG00000172733	PURG	0.175	4.515	2.076	0.041
ENSG00000172738	TMEM217	0.450	-0.888	2.137	0.036
ENSG00000172795	DCP2	0.234	6.613	2.352	0.021
ENSG00000173210	ABLIM3	0.195	5.589	1.996	0.049
ENSG00000173402	DAG1	-0.209	5.755	-2.116	0.038
ENSG00000173404	INSM1	0.512	0.837	2.180	0.032
ENSG00000173706	HEG1	-0.292	5.677	-2.305	0.024
ENSG00000174099	MSRB3	-0.335	4.773	-2.141	0.036
ENSG00000174136	RGMB	0.250	4.004	2.024	0.046
ENSG00000174442	ZWILCH	0.289	2.977	2.021	0.047
ENSG00000174446	SNAPC5	0.286	4.092	2.378	0.020
ENSG00000174453	VWC2L	0.446	3.719	2.019	0.047
ENSG00000174456	C12orf76	0.269	5.637	2.736	0.008
ENSG00000174500	GCSAM	0.864	-0.897	3.306	0.001
ENSG00000174928	C3orf33	0.367	-0.568	2.042	0.045
ENSG00000175087	PDIK1L	0.232	4.765	2.258	0.027
ENSG00000175193	PARL	0.228	1.885	2.027	0.046
ENSG00000175215	CTDSP2	-0.265	6.024	-2.368	0.020
ENSG00000175352	NRIP3	0.398	5.839	2.257	0.027
ENSG00000175395	ZNF25	0.327	7.139	2.784	0.007
ENSG00000175426	PCSK1	0.801	5.838	2.901	0.005
ENSG00000175895	PLEKHF2	-0.266	3.676	-2.086	0.040
ENSG00000175928	LRRN1	-0.270	5.594	-2.140	0.036
ENSG00000176014	TUBB6	-0.462	2.364	-2.428	0.018
ENSG00000176087	SLC35A4	-0.237	3.440	-2.047	0.044
ENSG00000176401	EID2B	0.364	3.346	2.639	0.010
ENSG00000176410	DNAJC30	0.188	2.628	2.099	0.039
ENSG00000176783	RUFY1	-0.181	5.240	-2.557	0.013
ENSG00000176840	MIR7-3HG	0.445	2.615	2.030	0.046
ENSG00000177119	ANO6	-0.301	5.366	-2.044	0.044
ENSG00000177133	LINC00982	-0.336	3.757	-2.036	0.045

ENSG00000177337	DLGAP1-AS1	-0.368	3.392	-2.869	0.005
ENSG00000177432	NAP1L5	0.416	6.800	2.474	0.016
ENSG00000177556	ATOX1	0.170	3.221	2.296	0.024
ENSG00000177694	NAALADL2	-0.305	3.826	-2.249	0.027
ENSG00000177752	YIPF7	0.390	-0.142	2.228	0.029
ENSG00000177889	UBE2N	0.279	6.480	2.234	0.028
ENSG00000178031	ADAMTSL1	0.609	2.052	3.655	0.000
ENSG00000178078	STAP2	0.499	-0.244	2.382	0.020
ENSG00000178115	GOLGA8Q	-0.455	0.488	-2.183	0.032
ENSG00000178201	VN1R1	-0.256	3.137	-1.993	0.050
ENSG00000178338	ZNF354B	-0.153	4.475	-2.049	0.044
ENSG00000178397	FAM220A	0.419	4.196	2.859	0.005
ENSG00000178538	CA8	0.245	4.305	2.183	0.032
ENSG00000178567	EPM2AIP1	0.242	6.800	2.953	0.004
ENSG00000178662	CSRNP3	0.309	8.664	2.086	0.040
ENSG00000178761	FAM219B	0.147	4.586	2.234	0.028
ENSG00000178764	ZHX2	-0.364	4.765	-2.204	0.031
ENSG00000178951	ZBTB7A	-0.265	5.906	-2.111	0.038
ENSG00000179241	LDLRAD3	-0.281	3.716	-2.071	0.042
ENSG00000179277	MEIS3P1	-0.419	2.379	-2.102	0.039
ENSG00000179523	EIF3J-AS1	0.270	3.590	3.027	0.003
ENSG00000179598	PLD6	0.251	2.706	2.380	0.020
ENSG00000179833	SERTAD2	-0.220	4.174	-2.079	0.041
ENSG00000179922	ZNF784	-0.320	0.583	-2.135	0.036
ENSG00000179930	ZNF648	0.698	-0.596	2.919	0.005
ENSG00000179933	C14orf119	0.273	3.190	2.206	0.030
ENSG00000180263	FGD6	-0.207	6.420	-2.127	0.037
ENSG00000180347	CCDC129	0.465	0.984	2.002	0.049
ENSG00000180376	CCDC66	-0.157	6.067	-2.334	0.022
ENSG00000180447	GAS1	-0.475	1.986	-2.993	0.004
ENSG00000180543	TSPYL5	0.225	5.635	2.070	0.042
ENSG00000180758	GPR157	-0.627	-0.849	-2.440	0.017
ENSG00000180769	WDFY3-AS2	-0.326	4.449	-2.427	0.018
ENSG00000180773	SLC36A4	0.235	5.497	2.634	0.010
ENSG00000180953	ST20	0.319	0.801	2.214	0.030
ENSG00000180998	GPR137C	0.284	5.067	2.191	0.032
ENSG00000181097	RP11-429J17.2	-0.782	-1.455	-2.219	0.029
ENSG00000181458	TMEM45A	-0.620	0.793	-2.379	0.020
ENSG00000181852	RNF41	0.254	6.016	2.388	0.019
ENSG00000181873	IBA57	-0.207	3.681	-2.644	0.010
ENSG00000182013	PNMAL1	0.292	7.548	2.280	0.025
ENSG00000182158	CREB3L2	-0.373	4.382	-2.159	0.034
ENSG00000182180	MRPS16	0.184	4.946	2.071	0.042
ENSG00000182359	KBTBD3	0.202	4.385	2.114	0.038
ENSG00000182397	DNM1P46	-0.711	-0.371	-2.139	0.036
ENSG00000182631	RXFP3	1.008	-2.379	2.150	0.035
ENSG00000182636	NDN	0.329	5.220	2.023	0.047

ENSG00000182836	PLCXD3	0.485	5.873	2.717	0.008
ENSG00000183527	PSMG1	0.233	3.558	2.020	0.047
ENSG00000183579	ZNRF3	-0.309	5.203	-2.616	0.011
ENSG00000183580	FBXL7	-0.442	3.827	-2.887	0.005
ENSG00000183621	ZNF438	-0.211	4.117	-2.852	0.006
ENSG00000183654	MARCHF11	0.519	1.006	2.225	0.029
ENSG00000183696	UPP1	0.210	4.313	2.101	0.039
ENSG00000183762	KREMEN1	-0.319	4.156	-2.328	0.023
ENSG00000183779	ZNF703	-0.351	1.970	-2.231	0.029
ENSG00000183864	TOB2	-0.327	5.747	-2.200	0.031
ENSG00000184117	NIPSNAP1	0.254	5.164	2.329	0.023
ENSG00000184139	RPL7AP28	1.103	-2.081	2.076	0.041
ENSG00000184154	LRTOMT	-0.337	2.832	-2.133	0.036
ENSG00000184162	NR2C2AP	-0.307	0.887	-2.266	0.026
ENSG00000184232	OAF	-0.489	3.219	-2.462	0.016
ENSG00000184260	HIST2H2AC	-0.333	4.147	-2.148	0.035
ENSG00000184277	TM2D3	0.248	5.757	2.151	0.035
ENSG00000184635	ZNF93	0.255	2.265	2.030	0.046
ENSG00000184900	SUMO3	0.387	5.507	2.641	0.010
ENSG00000185115	NSMCE3	0.226	4.126	2.364	0.021
ENSG00000185149	NPY2R	0.731	1.868	2.747	0.008
ENSG00000185272	RBM11	0.327	2.899	2.104	0.039
ENSG00000185610	DBX2	-0.355	2.809	-2.177	0.033
ENSG00000185627	PSMD13	0.214	4.691	2.106	0.039
ENSG00000185650	ZFP36L1	-0.500	6.311	-2.043	0.044
ENSG00000185697	MYBL1	-0.296	3.135	-2.345	0.022
ENSG00000185933	CALHM1	0.627	0.624	2.046	0.044
ENSG00000186104	CYP2R1	0.225	3.509	2.252	0.027
ENSG00000186106	ANKRD46	0.287	5.908	2.314	0.023
ENSG00000186188	FFAR4	0.548	1.471	2.166	0.033
ENSG00000186352	ANKRD37	-0.654	1.643	-2.619	0.011
ENSG00000186469	GNG2	0.495	7.212	2.192	0.031
ENSG00000186523	FAM86B1	-0.477	2.865	-2.307	0.024
ENSG00000186577	C6orf1	0.371	3.326	2.548	0.013
ENSG00000186867	QRFPR	0.717	0.466	2.360	0.021
ENSG00000187068	C3orf70	-0.248	6.122	-2.187	0.032
ENSG00000187474	FPR3	-0.625	0.259	-2.091	0.040
ENSG00000187678	SPRY4	-0.300	4.563	-2.024	0.046
ENSG00000187772	LIN28B	0.395	1.026	1.999	0.049
ENSG00000187824	TMEM220	-0.309	3.245	-2.052	0.044
ENSG00000187837	HIST1H1C	-0.262	5.293	-2.260	0.027
ENSG00000187867	PALM3	0.478	-0.138	2.089	0.040
ENSG00000188042	ARL4C	0.400	5.825	2.297	0.024
ENSG00000188352	FOCAD	0.200	7.065	2.083	0.041
ENSG00000188647	PTAR1	-0.243	7.385	-2.085	0.040
ENSG00000188693	CYP51A1-AS1	0.365	0.009	2.110	0.038
ENSG00000188712	OR13D3P	0.421	0.607	2.126	0.037

ENSG00000188730	VWC2	0.374	4.797	2.020	0.047
ENSG00000188783	PRELP	-0.634	4.108	-3.273	0.002
ENSG00000188869	TMC3	0.690	-0.707	2.373	0.020
ENSG00000189058	APOD	-0.532	6.543	-2.457	0.016
ENSG00000189159	HN1	0.279	4.449	2.193	0.031
ENSG00000196150	ZNF250	0.172	4.304	2.346	0.022
ENSG00000196172	ZNF681	0.246	4.095	2.022	0.047
ENSG00000196230	TUBB	0.280	7.123	2.133	0.036
ENSG00000196277	GRM7	0.255	5.516	2.204	0.031
ENSG00000196350	ZNF729	1.032	-2.062	2.012	0.048
ENSG00000196367	TRRAP	-0.135	6.937	-2.185	0.032
ENSG00000196655	TRAPPC4	0.207	3.613	2.154	0.034
ENSG00000196890	HIST3H2BB	0.367	1.292	2.205	0.030
ENSG00000196975	ANXA4	-0.249	3.920	-2.231	0.029
ENSG00000197162	ZNF785	-0.166	3.438	-2.118	0.037
ENSG00000197322	C17orf102	0.458	1.518	2.260	0.027
ENSG00000197345	MRPL21	0.346	3.412	2.117	0.037
ENSG00000197496	SLC2A10	-0.410	2.460	-2.272	0.026
ENSG00000197705	KLHL14	0.639	1.031	2.490	0.015
ENSG00000197713	RPE	0.170	5.527	2.223	0.029
ENSG00000197808	ZNF461	0.179	4.113	2.057	0.043
ENSG00000197822	OCLN	-0.497	2.852	-2.169	0.033
ENSG00000197885	NKIRAS1	0.306	5.658	2.500	0.015
ENSG00000197927	C2orf27A	0.265	2.765	2.354	0.021
ENSG00000197959	DNM3	0.216	8.446	2.180	0.032
ENSG00000197977	ELOVL2	-0.321	5.314	-2.010	0.048
ENSG00000198246	SLC29A3	0.279	2.017	2.294	0.025
ENSG00000198300	PEG3	0.364	9.634	2.144	0.035
ENSG00000198380	GFPT1	0.209	6.834	2.145	0.035
ENSG00000198478	SH3BGRL2	0.291	6.769	2.315	0.023
ENSG00000198502	HLA-DRB5	-1.226	1.832	-2.663	0.009
ENSG00000198580	RP11-611L7.2	0.414	2.287	2.329	0.023
ENSG00000198612	COPS8	0.272	5.989	2.425	0.018
ENSG00000198720	ANKRD13B	-0.233	3.895	-2.138	0.036
ENSG00000198805	PNP	-0.470	2.537	-2.136	0.036
ENSG00000198825	INPP5F	0.461	7.837	2.807	0.006
ENSG00000198840	MT-ND3	-0.514	10.449	-2.317	0.023
ENSG00000198914	POU3F3	-0.244	6.021	-2.790	0.007
ENSG00000199377	RNU5F-1	0.898	-3.002	2.104	0.039
ENSG00000199575	SNORD114-1	0.785	0.176	3.164	0.002
ENSG00000199833	SNORD115-21	0.449	0.392	2.047	0.044
ENSG00000199960	SNORD115-14	0.525	-0.019	2.152	0.035
ENSG00000200156	RNU5B-1	1.249	0.887	2.688	0.009
ENSG00000200183	RNU6-238P	0.806	-2.008	2.065	0.042
ENSG00000200189	RNU6-832P	1.029	-3.706	2.499	0.015
ENSG00000200351	Y_RNA	-0.593	-1.505	-2.049	0.044
ENSG00000200354	SNORA71D	0.920	-0.932	2.100	0.039

ENSG00000200503	SNORD115-5	0.951	3.045	2.018	0.047
ENSG00000200593	SNORD115-33	0.857	-0.270	2.049	0.044
ENSG00000200680	SNORD115-4	1.043	-0.814	2.893	0.005
ENSG00000200719	RNA5SP260	-0.691	-1.954	-2.560	0.012
ENSG00000200753	SNORD56	-0.774	-1.579	-2.417	0.018
ENSG00000200795	RNU4-1	0.744	4.450	2.364	0.021
ENSG00000200903	RNU1-42P	1.022	-2.444	2.210	0.030
ENSG00000201021	RNU6-743P	0.954	-3.250	2.422	0.018
ENSG00000201240	SNORD114-9	0.852	-1.272	2.975	0.004
ENSG00000201247	SNORD114-13	0.528	-0.740	2.100	0.039
ENSG00000201291	RNU1-34P	1.135	-3.475	2.298	0.024
ENSG00000201331	SNORD115-23	0.792	1.592	2.254	0.027
ENSG00000201499	RNU6-312P	0.563	-1.640	2.023	0.047
ENSG00000201805	RNU6-1180P	1.601	-3.669	3.255	0.002
ENSG00000201839	SNORD114-3	0.564	0.871	2.630	0.010
ENSG00000202270	SNORD114-12	1.036	-1.833	2.585	0.012
ENSG00000202534	RNU6-1329P	1.272	-2.511	2.730	0.008
ENSG00000203307	RP11-375H19.2	-0.548	-0.668	-2.277	0.026
ENSG00000203326	ZNF525	0.240	4.107	2.394	0.019
ENSG00000203446	AC004988.1	-0.758	-0.905	-2.149	0.035
ENSG00000203644	RP11-332M2.1	-0.388	1.552	-2.845	0.006
ENSG00000203910	C1orf146	0.549	-1.338	2.057	0.043
ENSG00000203965	EFCAB7	0.172	4.918	2.435	0.017
ENSG00000204147	ASAH2B	0.312	3.580	2.318	0.023
ENSG00000204179	PTPN20	0.747	3.283	3.782	0.000
ENSG00000204338	CYP21A1P	-1.002	-1.407	-2.068	0.042
ENSG00000204396	VWA7	0.453	1.345	1.997	0.049
ENSG00000204789	ZNF204P	0.302	6.207	2.098	0.039
ENSG00000205060	SLC35B4	0.200	5.950	2.180	0.032
ENSG00000205089	CCNI2	-0.313	1.948	-2.427	0.018
ENSG00000205189	ZBTB10	-0.185	6.379	-2.122	0.037
ENSG00000205629	LCMT1	0.237	4.392	2.245	0.028
ENSG00000205669	ACOT6	-0.444	-0.019	-2.198	0.031
ENSG00000205696	ADARB2-AS1	-0.720	-0.110	-2.606	0.011
ENSG00000205981	DNAJC19	0.150	4.639	1.998	0.049
ENSG00000206145	P2RX6P	1.098	-1.414	2.682	0.009
ENSG00000206562	METTL6	0.146	4.710	2.423	0.018
ENSG00000206583	RNU6-1292P	0.876	-2.885	2.359	0.021
ENSG00000206678	RNU6-144P	1.074	-2.410	2.244	0.028
ENSG00000206702	RNU1-11P	0.969	0.246	2.847	0.006
ENSG00000206838	SNORA5A	0.654	0.074	2.323	0.023
ENSG00000206888	RNU6-48P	0.828	0.096	2.197	0.031
ENSG00000206899	RNU6-36P	0.955	2.863	2.284	0.025
ENSG00000206973	RNU6-1145P	0.857	-3.335	2.019	0.047
ENSG00000207001	SNORD116-2	0.543	-0.890	2.050	0.044
ENSG00000207264	RNU6-15P	0.824	0.000	2.352	0.021
ENSG00000207279	SNORD116-24	0.421	0.778	1.999	0.049

ENSG00000207340	RNVU1-1	0.865	1.188	2.349	0.021
ENSG00000207357	RNU6-2	0.952	2.908	2.669	0.009
ENSG00000207360	RNU6-14P	0.924	1.034	2.572	0.012
ENSG00000207507	RNU6-9	0.823	2.814	2.403	0.019
ENSG00000207523	SNORA66	0.725	1.790	2.095	0.039
ENSG00000208772	SNORD94	0.469	1.985	2.222	0.029
ENSG00000211450	C11orf31	0.230	2.833	2.284	0.025
ENSG00000211456	SACM1L	0.172	6.060	2.090	0.040
ENSG00000212461	SNORA17	0.621	-2.101	2.493	0.015
ENSG00000213619	NDUFS3	0.205	4.388	2.038	0.045
ENSG00000213977	TAX1BP3	-0.359	0.454	-2.134	0.036
ENSG00000214106	PAXIP1-AS2	-0.407	3.767	-2.663	0.009
ENSG00000214198	TTC41P	-0.439	1.545	-2.623	0.011
ENSG00000214338	SOGA3	0.592	-0.798	2.421	0.018
ENSG00000214366	EIF4EP5	0.617	-1.203	2.482	0.015
ENSG00000214376	VSTM5	0.557	2.148	3.175	0.002
ENSG00000214998	CCNB2P1	-0.724	-0.963	-2.614	0.011
ENSG00000215712	TMEM242	0.298	4.958	2.383	0.020
ENSG00000216809	RP11-57K17.1	0.523	1.138	2.624	0.010
ENSG00000216990	HSPD1P10	1.099	-2.176	2.655	0.010
ENSG00000217930	PAM16	-0.401	0.437	-2.620	0.011
ENSG00000218732	RP5-1046G13.2	0.991	-2.017	2.233	0.028
ENSG00000219430	MBL3P	0.968	-1.209	2.936	0.004
ENSG00000220305	HNRNPH1P1	-0.429	0.328	-2.221	0.029
ENSG00000220614	RP11-480N24.4	-0.452	-0.219	-2.308	0.024
ENSG00000221639	SNORA3	-0.601	-1.892	-2.038	0.045
ENSG00000221843	C2orf16	0.244	3.969	2.138	0.036
ENSG00000221926	TRIM16	0.254	2.844	2.795	0.007
ENSG00000222112	RN7SKP16	-0.568	-0.182	-2.281	0.025
ENSG00000222210	RN7SKP65	-1.107	-4.062	-2.108	0.038
ENSG00000222293	RNU2-36P	1.161	-3.391	2.639	0.010
ENSG00000223525	RABGAP1L-IT1	0.514	-0.826	2.109	0.038
ENSG00000223797	ENTPD3-AS1	-0.292	1.984	-2.366	0.021
ENSG00000223891	OSER1-AS1	0.193	3.365	2.106	0.038
ENSG00000223893	GNL2P1	0.809	-0.924	2.973	0.004
ENSG00000223942	RP11-252P19.2	-0.847	-2.993	-2.085	0.040
ENSG00000224195	RP11-574K11.5	0.881	-1.382	3.220	0.002
ENSG00000224354	MTND2P5	-0.602	-0.146	-2.209	0.030
ENSG00000224511	LINC00365	0.709	-1.647	2.070	0.042
ENSG00000224531	SMIM13	0.254	6.293	2.111	0.038
ENSG00000224593	RP11-30B1.1	-0.424	0.272	-2.048	0.044
ENSG00000224613	RP11-153F1.1	0.660	-0.758	2.089	0.040
ENSG00000224955	KCTD10P1	0.689	-0.562	2.284	0.025
ENSG00000225174	OSTM1-AS1	-0.667	0.231	-2.987	0.004
ENSG00000225205	AC093818.1	-0.527	0.465	-2.414	0.018
ENSG00000225213	RP11-197M22.2	0.480	-0.386	2.474	0.016
ENSG00000225356	RP11-433O3.1	0.882	-0.119	2.881	0.005

ENSG00000225439	BOLA3-AS1	0.259	3.041	2.269	0.026
ENSG00000225465	RFPL1S	0.442	6.074	2.255	0.027
ENSG00000225497	KCNMA1-AS2	-0.531	-0.459	-1.995	0.050
ENSG00000225554	RP11-527D7.1	0.628	0.179	2.890	0.005
ENSG00000225675	LINC01771	0.591	0.205	2.221	0.029
ENSG00000225726	AC007000.10	-0.433	0.395	-2.118	0.037
ENSG00000225746	SNHG23	0.324	6.122	2.533	0.013
ENSG00000225764	P3H2-AS1	0.593	-1.609	2.123	0.037
ENSG00000225808	DNAJC19P5	-0.551	-0.150	-2.324	0.023
ENSG00000226254	PTP4A1P3	0.661	-0.919	2.303	0.024
ENSG00000226377	AC084809.2	1.428	-2.419	3.283	0.002
ENSG00000226393	IFNA20P	-0.589	-0.869	-2.781	0.007
ENSG00000226403	RP11-31F19.1	-0.638	-1.208	-2.315	0.023
ENSG00000226471	CTA-292E10.6	-0.396	0.220	-2.251	0.027
ENSG00000226491	FTOP1	-0.343	0.520	-2.196	0.031
ENSG00000226636	RP5-1049N15.2	-0.752	-0.320	-2.228	0.029
ENSG00000226648	PLCG1-AS1	-0.293	0.520	-2.020	0.047
ENSG00000226695	ANKRD20A10P	0.809	-0.793	2.798	0.007
ENSG00000226717	PTPRD-AS2	0.827	-0.186	2.376	0.020
ENSG00000226762	RP11-298E9.6	0.884	0.146	2.033	0.046
ENSG00000226944	RP1-120G22.11	0.879	-2.222	2.358	0.021
ENSG00000227006	RP5-956O18.2	-0.374	0.218	-2.098	0.039
ENSG00000227053	RP11-395B7.4	0.822	-1.756	2.051	0.044
ENSG00000227071	FOCAD-AS1	0.591	-1.622	2.320	0.023
ENSG00000227170	AF178030.2	-0.539	0.714	-2.144	0.035
ENSG00000227218	RP11-203J24.8	-0.627	-0.227	-2.340	0.022
ENSG00000227359	AC017074.2	-0.683	-2.034	-2.126	0.037
ENSG00000227413	RP5-1042K10.12	-0.643	-0.563	-2.147	0.035
ENSG00000227470	AC073415.2	0.436	-0.459	2.340	0.022
ENSG00000227492	RP11-316M21.6	0.485	-0.752	2.302	0.024
ENSG00000227560	RP11-139K1.2	-0.686	-0.382	-2.637	0.010
ENSG00000227640	SOX21-AS1	-0.389	2.961	-2.573	0.012
ENSG00000227681	RP11-307P5.1	0.788	-0.558	2.777	0.007
ENSG00000227742	CALR4P	-1.230	-1.389	-2.832	0.006
ENSG00000228020	HNRNPA1P46	1.257	-1.861	2.090	0.040
ENSG00000228022	HCG20	-0.912	-1.877	-2.512	0.014
ENSG00000228079	AC012368.1	-1.073	-1.701	-3.625	0.001
ENSG00000228107	AP000692.9	-0.366	0.643	-2.166	0.033
ENSG00000228446	AC073052.1	-0.261	1.464	-2.071	0.042
ENSG00000228549	RP11-108M9.3	0.709	-0.754	2.157	0.034
ENSG00000228793	RP1-223B1.1	-1.422	-0.912	-2.424	0.018
ENSG00000228816	AK3P5	-0.483	-0.770	-2.030	0.046
ENSG00000228853	NEGR1-IT1	0.570	-0.316	2.445	0.017
ENSG00000228944	AC004485.3	-0.835	-2.203	-2.249	0.027
ENSG00000229330	AC006947.1	-0.608	-1.612	-2.250	0.027
ENSG00000229427	ANKRD26P4	-0.411	0.498	-2.274	0.026
ENSG00000229558	SACS-AS1	-0.429	0.553	-2.293	0.025

ENSG00000229598	PRDX3P1	-0.411	1.627	-2.144	0.035
ENSG00000229657	RP11-494K3.2	-0.658	1.718	-2.957	0.004
ENSG00000229839	AC018462.2	-0.367	0.807	-2.356	0.021
ENSG00000229847	EMX2OS	-0.450	6.513	-2.694	0.009
ENSG00000229938	MYO16-AS2	0.687	-0.948	2.243	0.028
ENSG00000229955	RP1-5O6.4	-0.766	-1.817	-2.482	0.015
ENSG00000230002	ALMS1-IT1	-0.480	1.565	-2.740	0.008
ENSG00000230071	RPL4P6	-0.394	0.751	-2.215	0.030
ENSG00000230074	RP11-195F19.9	0.464	-0.025	2.159	0.034
ENSG00000230319	AL022476.2	0.436	1.342	2.617	0.011
ENSG00000230735	RP11-413E1.4	-0.502	-0.258	-2.430	0.017
ENSG00000230989	HSBP1	0.230	6.354	2.165	0.034
ENSG00000231290	APCDD1L-AS1	0.542	-0.859	2.530	0.013
ENSG00000231607	DLEU2	-0.287	2.621	-2.270	0.026
ENSG00000231738	TSPAN19	0.493	0.323	2.388	0.019
ENSG00000231769	RP1-8B1.4	0.598	0.045	2.705	0.008
ENSG00000231784	DBIL5P	0.329	0.583	2.065	0.042
ENSG00000232040	ZBED9	0.309	3.610	2.760	0.007
ENSG00000232287	SLC6A1-AS1	-0.416	1.889	-2.394	0.019
ENSG00000232377	AC016910.1	0.614	-1.103	2.118	0.037
ENSG00000232407	RP11-641C17.2	-0.698	-1.762	-2.536	0.013
ENSG00000232480	TGFB2-AS1	-0.344	0.953	-2.137	0.036
ENSG00000232768	RP11-201O14.2	-0.675	-1.467	-2.333	0.022
ENSG00000232788	AC078883.3	-0.508	0.122	-2.241	0.028
ENSG00000232852	CICP4	-0.658	-0.718	-2.159	0.034
ENSG00000232903	LINC01166	0.822	0.252	3.462	0.001
ENSG00000233189	RPL12P29	-0.523	-0.544	-2.006	0.048
ENSG00000233208	LINC00642	1.193	-2.609	2.389	0.019
ENSG00000233325	MIPEPP3	0.329	0.432	2.162	0.034
ENSG00000233452	STXBP5-AS1	0.500	4.286	3.085	0.003
ENSG00000233642	GPR158-AS1	0.824	-2.511	2.107	0.038
ENSG00000233845	AC093732.1	-0.577	-0.845	-2.208	0.030
ENSG00000233892	PAIP1P1	-0.532	-0.171	-2.210	0.030
ENSG00000233906	RP11-315I14.2	0.944	-2.943	2.219	0.030
ENSG00000234056	LINC00463	-0.905	-1.293	-2.457	0.016
ENSG00000234177	LINC01114	-0.272	3.109	-2.081	0.041
ENSG00000234352	AC009264.1	0.410	1.456	2.093	0.040
ENSG00000234428	RP11-666F17.1	-0.383	1.133	-2.102	0.039
ENSG00000234586	RP11-382H24.2	0.989	-2.282	2.351	0.021
ENSG00000234650	PCCA-AS1	-0.352	0.168	-2.013	0.048
ENSG00000234675	RP11-242F11.2	0.717	-0.730	2.603	0.011
ENSG00000234751	AP002381.2	-0.468	-0.972	-2.077	0.041
ENSG00000234758	AC034228.4	-0.595	-0.839	-2.291	0.025
ENSG00000234801	MORF4	0.481	0.709	2.235	0.028
ENSG00000235111	RP1-29C18.8	-1.006	-1.661	-2.004	0.049
ENSG00000235279	RP11-539E19.2	-0.560	-0.168	-2.374	0.020
ENSG00000235347	RP1-37M3.8	-0.440	0.607	-2.202	0.031

ENSG00000235374	SSR4P1	0.373	-0.196	2.036	0.045
ENSG00000235711	ANKRD34C	0.693	2.879	2.787	0.007
ENSG00000235786	ZNRF3-IT1	-0.451	0.789	-2.373	0.020
ENSG00000236306	LINC01241	-0.718	-1.128	-2.338	0.022
ENSG00000236440	AC117947.1	0.526	0.856	2.858	0.005
ENSG00000236577	SNRPGP14	0.631	-1.611	2.344	0.022
ENSG00000236776	RPL21P23	-0.774	-1.444	-2.066	0.042
ENSG00000237133	AC020594.5	0.476	-1.120	2.017	0.047
ENSG00000237190	CDKN2AIPNL	0.350	4.094	2.235	0.028
ENSG00000237301	RP4-680D5.2	-0.515	-0.403	-2.377	0.020
ENSG00000237711	MTCO3P11	-0.631	-0.561	-2.582	0.012
ENSG00000237732	AC010980.2	0.363	1.899	2.105	0.039
ENSG00000237753	AC079922.3	0.403	1.367	2.490	0.015
ENSG00000237911	SRP68P3	-0.533	-0.275	-2.203	0.031
ENSG00000238018	AC093110.3	-0.304	2.746	-2.045	0.044
ENSG00000238058	RP11-432J22.2	0.274	0.506	2.034	0.045
ENSG00000238125	SLC9A3P2	1.682	-3.958	2.814	0.006
ENSG00000238173	RPL39P6	1.017	-2.613	2.118	0.037
ENSG00000238243	OR2W3	0.904	-0.662	2.580	0.012
ENSG00000238500	RNU7-87P	-0.492	-1.536	-2.068	0.042
ENSG00000238554	RNU1-88P	1.102	-3.043	2.155	0.034
ENSG00000238825	RNU1-13P	1.043	0.886	2.475	0.016
ENSG00000239151	RNU7-195P	-0.748	-1.675	-2.438	0.017
ENSG00000239224	RN7SL546P	1.041	-1.563	2.460	0.016
ENSG00000239398	RN7SL342P	1.518	-3.869	2.966	0.004
ENSG00000239437	RN7SL752P	0.580	6.885	2.793	0.007
ENSG00000239672	NME1	0.419	3.562	2.365	0.021
ENSG00000240253	FAR2P3	0.619	1.014	3.138	0.002
ENSG00000240328	RP11-841C19.1	-1.093	-1.815	-3.113	0.003
ENSG00000240531	RPL21P123	-0.477	-0.017	-2.806	0.006
ENSG00000240694	PNMA2	0.364	8.866	2.626	0.010
ENSG00000240808	CTD-3236F5.1	-0.728	0.187	-3.251	0.002
ENSG00000240888	RP13-635I23.3	-0.558	-1.276	-2.008	0.048
ENSG00000240891	PLCXD2	0.433	5.324	2.072	0.042
ENSG00000241112	RPL29P14	0.396	-0.474	2.040	0.045
ENSG00000241172	RN7SL70P	0.883	-3.625	2.225	0.029
ENSG00000241243	RN7SL629P	0.846	-0.187	2.677	0.009
ENSG00000241362	RPL36AP43	-0.654	-0.434	-2.769	0.007
ENSG00000241370	RPP21	-0.569	-0.448	-2.411	0.018
ENSG00000241461	RN7SL182P	0.752	-1.875	2.004	0.049
ENSG00000241529	RN7SL767P	0.533	5.177	2.953	0.004
ENSG00000241592	RP11-372E1.7	0.800	-0.387	3.605	0.001
ENSG00000241679	RP11-80H8.4	-0.647	-0.853	-2.474	0.016
ENSG00000241735	FABP5P3	0.558	-1.036	2.390	0.019
ENSG00000241983	RN7SL566P	0.543	-0.810	2.133	0.036
ENSG00000242121	RN7SL267P	0.819	-2.144	2.269	0.026
ENSG00000242173	ARHGDIG	0.522	4.063	2.078	0.041

ENSG00000242412	DBIL5P2	-0.563	-0.454	-2.331	0.022
ENSG00000242614	RN7SL164P	0.744	-0.720	2.494	0.015
ENSG00000242767	ZBTB20-AS4	-0.602	-0.660	-2.415	0.018
ENSG00000243155	RP11-46A10.5	-0.616	-1.049	-2.356	0.021
ENSG00000243260	RN7SL558P	0.550	-1.245	2.387	0.019
ENSG00000243364	EFNA4	-0.856	-1.728	-2.288	0.025
ENSG00000243384	RP11-475O23.2	-0.576	-0.215	-2.057	0.043
ENSG00000243424	RN7SL107P	0.397	2.012	2.295	0.025
ENSG00000243449	C4orf48	0.790	-0.202	3.170	0.002
ENSG00000243620	RP11-649A16.1	-1.292	0.070	-2.621	0.011
ENSG00000243872	RN7SL464P	0.712	-1.531	2.208	0.030
ENSG00000244018	RPL35P6	-0.472	0.277	-1.993	0.050
ENSG00000244060	RPS2P41	-0.540	-1.444	-2.001	0.049
ENSG00000244139	RN7SL397P	1.042	-3.383	2.112	0.038
ENSG00000244194	RN7SL218P	0.782	-0.068	2.089	0.040
ENSG00000244230	RN7SL151P	0.458	3.982	2.028	0.046
ENSG00000244289	RPS3AP35	0.608	-0.575	2.332	0.022
ENSG00000244632	RN7SL863P	1.120	-2.023	2.646	0.010
ENSG00000244642	RN7SL396P	0.540	8.675	2.917	0.005
ENSG00000246022	ALDH1L1-AS2	-0.448	0.844	-2.354	0.021
ENSG00000247595	SPTY2D1-AS1	-0.479	0.187	-2.231	0.029
ENSG00000248283	CCNL2P1	-0.363	2.507	-2.190	0.032
ENSG00000248285	RP11-158J3.2	1.050	-1.179	2.640	0.010
ENSG00000248319	LINC02275	0.708	-1.618	2.054	0.043
ENSG00000248559	RP11-215P8.3	0.353	0.536	2.309	0.024
ENSG00000248627	RP11-428L21.1	0.432	0.302	2.101	0.039
ENSG00000248629	RP11-154F14.2	-0.795	-1.638	-2.703	0.008
ENSG00000248725	RP11-218C23.1	-0.463	0.216	-2.326	0.023
ENSG00000248874	C5orf17	1.247	-1.956	2.186	0.032
ENSG00000248923	MTND5P11	-0.512	2.517	-1.995	0.050
ENSG00000248927	CTD-2334D19.1	0.498	0.897	2.520	0.014
ENSG00000249020	SNORA58	0.636	-0.844	2.257	0.027
ENSG00000249456	RP11-298J20.4	0.737	-2.354	2.539	0.013
ENSG00000249514	CTD-2266L18.1	-0.910	-1.733	-2.299	0.024
ENSG00000249550	LINC01234	0.798	2.229	2.884	0.005
ENSG00000249592	RP11-440L14.1	-0.355	2.205	-2.216	0.030
ENSG00000249678	RP11-619J20.1	0.586	-0.705	2.174	0.033
ENSG00000249780	RP11-352E6.2	1.142	-1.492	2.017	0.047
ENSG00000249916	CTD-2280E9.1	-0.981	-1.611	-3.270	0.002
ENSG00000250017	RP11-484L7.2	0.519	1.845	2.035	0.045
ENSG00000250033	SLC7A11-AS1	-0.521	2.827	-2.191	0.031
ENSG00000250041	CTD-2003C8.2	-0.594	-1.507	-2.363	0.021
ENSG00000250075	RP11-584P21.2	0.615	0.409	2.967	0.004
ENSG00000250238	Z95704.3	0.953	-1.655	2.993	0.004
ENSG00000250320	CTD-2269F5.1	0.404	0.995	2.526	0.014
ENSG00000250461	RP11-631M6.2	-0.482	3.875	-3.100	0.003
ENSG00000250508	RP11-757G1.6	-0.592	0.030	-2.796	0.007

ENSG00000250514	LINC02125	0.624	-0.651	2.254	0.027
ENSG00000250602	RP11-517I3.1	-0.577	1.432	-2.616	0.011
ENSG00000250749	RP11-451H23.1	-0.761	-1.962	-2.135	0.036
ENSG00000250910	AC097467.2	0.358	1.495	2.052	0.044
ENSG00000251076	RP11-526F3.1	-0.666	-1.472	-2.054	0.043
ENSG00000251128	WWC2-AS1	-0.706	-1.869	-2.011	0.048
ENSG00000251139	RP11-701P16.2	-0.896	-1.155	-2.846	0.006
ENSG00000251171	RP11-327O17.2	-0.371	0.003	-2.009	0.048
ENSG00000251209	LINC00923	0.480	1.763	2.276	0.026
ENSG00000251306	NDUFB4P2	0.500	0.146	2.250	0.027
ENSG00000251350	LINC02475	-0.636	1.186	-2.854	0.006
ENSG00000251562	MALAT1	-0.268	14.006	-2.564	0.012
ENSG00000251664	PCDHA12	-0.417	0.900	-2.599	0.011
ENSG00000251675	CTC-458I2.2	-1.173	-2.102	-3.071	0.003
ENSG00000251682	RP11-631M6.3	-0.374	2.543	-1.999	0.049
ENSG00000252643	RNU6-1136P	0.652	-1.531	2.083	0.041
ENSG00000252707	RNU11-2P	0.703	-0.358	3.044	0.003
ENSG00000252804	RNU6-958P	1.232	-2.943	2.372	0.020
ENSG00000253086	RNU6-537P	0.953	-2.818	2.009	0.048
ENSG00000253175	RP11-267M23.6	-0.744	-2.226	-2.269	0.026
ENSG00000253200	RP11-582J16.5	-0.314	1.097	-2.613	0.011
ENSG00000253300	RP11-108E14.1	0.744	-1.559	1.994	0.050
ENSG00000253305	PCDHGB6	-0.269	3.745	-2.087	0.040
ENSG00000253352	TUG1	-0.147	7.122	-2.088	0.040
ENSG00000253372	RP11-44N11.1	-0.454	1.383	-2.042	0.045
ENSG00000253456	CTD-2363C16.2	-1.510	-1.747	-3.830	0.000
ENSG00000253485	PCDHGA5	-0.247	3.026	-2.090	0.040
ENSG00000253537	PCDHGA7	-0.252	3.263	-2.279	0.025
ENSG00000253653	CTC-248O19.1	1.106	-2.223	2.965	0.004
ENSG00000253719	ATXN7L3B	0.262	7.467	2.489	0.015
ENSG00000253846	PCDHGA10	-0.280	4.217	-2.406	0.019
ENSG00000253910	PCDHGB2	-0.354	3.892	-2.824	0.006
ENSG00000254028	AC083843.2	-0.630	-1.392	-2.543	0.013
ENSG00000254060	RP11-172E10.1	-0.521	-0.586	-2.228	0.029
ENSG00000254221	PCDHGB1	-0.362	2.465	-3.234	0.002
ENSG00000254245	PCDHGA3	-0.303	3.234	-2.489	0.015
ENSG00000254260	RP11-369E15.1	1.218	-1.883	2.252	0.027
ENSG00000254358	CYCSP22	-0.638	-1.822	-2.222	0.029
ENSG00000254638	RP11-356J5.4	0.751	-1.887	2.293	0.025
ENSG00000254758	AP000889.1	0.518	0.059	2.100	0.039
ENSG00000254768	CTD-2140G10.2	0.865	-2.392	2.025	0.046
ENSG00000254901	BORCS8	0.254	2.837	2.081	0.041
ENSG00000254938	RP11-688I9.4	-0.340	2.074	-2.001	0.049
ENSG00000254968	RP11-65M17.3	0.760	-2.032	2.173	0.033
ENSG00000255076	CTD-2023J5.1	0.822	-2.020	2.456	0.016
ENSG00000255219	RP11-716H6.2	-0.701	-0.701	-2.084	0.041
ENSG00000255250	CTD-2005H7.2	-0.665	-1.370	-2.647	0.010

ENSG00000255581	RP11-69M1.4	0.540	0.451	2.409	0.018
ENSG00000255968	RP11-513G19.1	-0.494	0.694	-2.579	0.012
ENSG00000256001	RP11-575F12.2	0.639	-1.335	2.114	0.038
ENSG00000256159	RP11-820K3.4	-0.737	-1.396	-2.247	0.028
ENSG00000256235	SMIM3	-0.522	1.398	-2.622	0.011
ENSG00000256394	ASIC5	1.085	-3.651	2.137	0.036
ENSG00000256448	RP11-809N8.4	-0.998	-1.431	-2.890	0.005
ENSG00000256589	ENPP7P5	-0.360	1.083	-1.997	0.049
ENSG00000257126	FOXG1-AS1	-0.396	1.725	-3.047	0.003
ENSG00000257298	RP3-405J10.3	-0.355	1.152	-2.105	0.039
ENSG00000257308	RP11-471N19.1	1.211	-1.940	2.480	0.015
ENSG00000257943	RP11-167N24.3	-0.745	-2.583	-2.244	0.028
ENSG00000257951	RP11-554D14.4	0.793	-1.074	2.767	0.007
ENSG00000258072	RP11-554D14.2	0.611	0.423	2.781	0.007
ENSG00000258240	RP1-46F2.3	1.052	-2.129	2.683	0.009
ENSG00000258274	RP11-887P2.5	-0.390	1.789	-2.311	0.024
ENSG00000258384	AC068831.6	-0.908	-2.153	-2.452	0.017
ENSG00000258428	RP11-1085N6.2	-0.514	-1.324	-2.146	0.035
ENSG00000258667	HIF1A-AS2	-0.620	0.007	-2.168	0.033
ENSG00000258754	LINC01579	0.580	0.865	3.032	0.003
ENSG00000258940	RP11-407N17.5	0.699	-1.758	2.474	0.016
ENSG00000258983	RP11-507K2.2	-0.622	-0.308	-2.806	0.006
ENSG00000259055	RP11-1140I5.1	-0.438	1.785	-2.300	0.024
ENSG00000259356	RP11-90E5.1	-0.753	-1.031	-2.018	0.047
ENSG00000259415	RP11-7M10.2	-0.821	-1.415	-2.828	0.006
ENSG00000259425	RP11-566K19.5	0.363	0.361	2.012	0.048
ENSG00000259429	UBE2Q2P2	-0.382	2.623	-2.811	0.006
ENSG00000259446	RP11-489D6.2	-0.788	-1.647	-2.084	0.040
ENSG00000259543	CTD-2034I4.1	0.748	-1.707	2.022	0.047
ENSG00000259556	RP11-56B16.2	-0.451	0.168	-2.254	0.027
ENSG00000259782	CTD-2270L9.2	0.829	-1.786	2.664	0.009
ENSG00000259820	AC083843.1	-0.316	3.115	-2.074	0.041
ENSG00000259837	LINC01904	1.140	-2.576	2.017	0.047
ENSG00000260000	RP3-467N11.1	0.441	1.606	2.272	0.026
ENSG00000260025	RP11-490M8.1	0.399	1.402	2.266	0.026
ENSG00000260083	MIR762HG	0.671	-0.998	3.128	0.002
ENSG00000260166	RP11-863P13.6	1.072	-0.928	2.177	0.033
ENSG00000260213	RP11-303E16.3	-0.672	-1.405	-2.146	0.035
ENSG00000260274	RP11-817O13.8	0.721	-1.177	2.727	0.008
ENSG00000260277	RP11-101E7.2	-0.452	-0.029	-2.209	0.030
ENSG00000260317	RP11-48B3.4	-0.617	0.379	-2.268	0.026
ENSG00000260329	RP11-412D9.4	0.300	0.898	2.254	0.027
ENSG00000260360	RP11-533E19.5	-0.761	-1.585	-2.441	0.017
ENSG00000260369	CTD-2526A2.2	0.937	-2.627	2.105	0.039
ENSG00000260412	RP11-438B23.2	0.423	4.301	2.104	0.039
ENSG00000260455	NBAT1	-0.348	0.663	-2.031	0.046
ENSG00000260517	RP11-426C22.5	-0.526	-0.897	-2.421	0.018

ENSG00000260549	MT1L	-0.594	-0.295	-2.113	0.038
ENSG00000260690	CYCSP39	0.710	-2.063	2.454	0.016
ENSG00000260733	RP11-264L1.4	-0.445	0.346	-2.171	0.033
ENSG00000260838	RP11-531A24.3	0.530	1.531	2.133	0.036
ENSG00000260924	LINC01311	0.675	-1.397	2.242	0.028
ENSG00000260969	RP11-190D6.2	-0.677	-2.204	-2.029	0.046
ENSG00000261087	KB-1460A1.5	-0.240	5.082	-2.357	0.021
ENSG00000261103	RP11-298D21.3	0.820	-1.478	2.848	0.006
ENSG00000261210	CLEC19A	0.663	0.019	2.426	0.018
ENSG00000261216	RP11-166B2.5	-0.574	-1.055	-2.087	0.040
ENSG00000261242	CTD-2302E22.4	0.554	-0.939	2.208	0.030
ENSG00000261399	LA16c-329F2.1	-0.570	0.412	-2.949	0.004
ENSG00000261402	RP11-378I6.1	0.658	0.870	2.042	0.045
ENSG00000261542	RP11-16E18.3	0.398	1.049	2.510	0.014
ENSG00000261884	CTC-479C5.12	-0.318	1.326	-2.269	0.026
ENSG00000262038	RP11-177N22.3	-0.529	-1.593	-2.044	0.044
ENSG00000262663	RP11-497H17.1	-0.663	-0.958	-2.217	0.030
ENSG00000263142	LRRC37A17P	-0.273	3.974	-2.048	0.044
ENSG00000263426	RN7SL471P	0.781	4.212	2.499	0.015
ENSG00000263603	CTD-2349P21.5	-0.382	1.858	-2.080	0.041
ENSG00000263766	RP11-580I16.2	-0.448	-0.467	-2.225	0.029
ENSG00000263874	LINC00672	0.389	4.784	2.255	0.027
ENSG00000263968	RN7SL381P	0.474	2.437	2.187	0.032
ENSG00000263988	RN7SL147P	1.018	-0.787	3.047	0.003
ENSG00000264424	MYH4	0.877	-2.163	2.051	0.044
ENSG00000264434	CTD-2006O16.2	-0.864	-1.426	-2.590	0.011
ENSG00000264536	MIR5706	-0.719	-2.496	-2.112	0.038
ENSG00000265179	RP11-672L10.2	1.247	0.415	2.998	0.004
ENSG00000265917	MIR3685	0.765	-2.969	2.132	0.036
ENSG00000266127	ZNF415P1	-0.413	-0.082	-2.003	0.049
ENSG00000266405	CBX3P2	0.464	0.009	2.283	0.025
ENSG00000266477	RN7SL616P	1.058	-2.905	2.484	0.015
ENSG00000266875	RN7SL408P	0.975	-1.617	2.671	0.009
ENSG00000267028	TCF4-AS1	0.427	-0.153	2.362	0.021
ENSG00000267150	RP11-411B10.2	0.654	-1.111	2.497	0.015
ENSG00000267244	CTB-31O20.4	-0.466	-0.153	-2.354	0.021
ENSG00000267272	LINC01140	0.474	1.989	2.139	0.036
ENSG00000267321	LINC02001	0.354	1.351	2.621	0.011
ENSG00000267375	CTB-186G2.4	-0.782	-1.849	-2.751	0.007
ENSG00000267395	AC074212.6	-0.659	-1.345	-2.481	0.015
ENSG00000267475	CTD-2538C1.2	0.524	-0.323	2.318	0.023
ENSG00000267519	MIR24-2	-0.633	1.904	-2.557	0.013
ENSG00000267746	RP11-379L18.1	0.484	-0.785	2.086	0.040
ENSG00000268182	SMIM17	0.764	2.794	4.480	0.000
ENSG00000268879	IGFL1P1	0.740	-1.798	2.218	0.030
ENSG00000269399	CTD-3222D19.1[illegible]	0.358	0.525	2.348	0.022
ENSG00000269489	RP11-98D18.17	0.626	-2.249	2.020	0.047

ENSG00000269559	RP11-629B11.5	-0.565	-1.222	-2.036	0.045
ENSG00000269793	ZIM2-AS1	0.360	2.269	2.698	0.009
ENSG00000269873	CTD-2587H19.3	-0.620	-0.395	-2.715	0.008
ENSG00000269895	AP000654.4	-0.809	-1.447	-2.516	0.014
ENSG00000269921	RP11-646I6.5	0.375	-0.152	2.149	0.035
ENSG00000270059	RP11-88H12.2	0.568	-0.427	2.270	0.026
ENSG00000270077	KB-1958F4.1	-0.579	-0.802	-2.147	0.035
ENSG00000270108	RP11-73M18.6	-0.317	0.840	-2.114	0.038
ENSG00000270112	RP11-742D12.2	0.392	2.920	2.194	0.031
ENSG00000270170	NCBP2-AS2	0.344	2.629	2.364	0.021
ENSG00000270265	RP11-731D1.4	-0.882	-0.794	-2.548	0.013
ENSG00000270457	RP11-467C18.1	-0.796	-1.876	-2.214	0.030
ENSG00000270510	RP11-777F6.3	0.376	0.318	2.107	0.038
ENSG00000270562	RP11-154H23.3	-0.445	1.339	-2.370	0.020
ENSG00000270640	RP11-373D23.2	-1.215	-1.082	-2.909	0.005
ENSG00000270661	RP1-142L7.9	-0.896	-0.840	-2.943	0.004
ENSG00000270776	RP11-17G11.1	-0.670	-1.538	-2.238	0.028
ENSG00000270823	RP11-2E11.6	0.401	1.385	2.212	0.030
ENSG00000271040	RP5-933K21.3	0.686	-1.431	2.357	0.021
ENSG00000271424	RP11-647O20.1	0.597	-1.111	2.289	0.025
ENSG00000271499	CTC-559E9.9	0.620	-1.619	2.052	0.044
ENSG00000271601	LIX1L	-0.303	4.502	-2.752	0.007
ENSG00000271624	RP11-651K21.1	-0.387	0.967	-2.045	0.044
ENSG00000271918	CTD-2287O16.5	0.389	1.046	2.770	0.007
ENSG00000272020	RNU1-30P	0.702	-1.508	2.171	0.033
ENSG00000272137	RP11-177G23.2	-0.433	0.898	-2.112	0.038
ENSG00000272172	RP13-582O9.7	0.933	-2.176	3.053	0.003
ENSG00000272197	RN7SL803P	0.694	-1.916	2.062	0.043
ENSG00000272397	RP11-497D6.5	0.516	0.865	2.365	0.021
ENSG00000272460	SNORD115-40	0.700	-1.031	2.574	0.012
ENSG00000272483	RP11-169K17.3	-0.975	-1.369	-3.089	0.003
ENSG00000272551	RP11-324L17.1	1.262	-1.684	2.717	0.008
ENSG00000272562	RP11-396C23.4	0.858	-1.682	3.117	0.003
ENSG00000272604	RP11-251G23.5	0.365	1.468	2.285	0.025
ENSG00000272656	RP11-219D15.3	-0.814	-1.837	-2.580	0.012
ENSG00000272880	RP11-33M22.2	0.682	-1.185	2.233	0.028
ENSG00000272913	RP11-440D17.3	-1.180	-1.514	-3.334	0.001
ENSG00000273014	RP11-225B17.2	-0.284	0.617	-2.012	0.048
ENSG00000273026	RP11-422P24.10	0.767	-2.158	2.161	0.034
ENSG00000273483	RP4-671G15.2	-0.427	-0.539	-2.026	0.046
ENSG00000274001	RP11-5G9.5	0.613	-1.201	2.036	0.045
ENSG00000274346	RP11-40B20.1	0.703	-0.508	2.927	0.005
ENSG00000274512	TBC1D3L	-0.890	0.860	-2.279	0.025
ENSG00000274659	LINC02371	0.729	-1.606	2.324	0.023
ENSG00000274769	RP11-493E12.3	0.631	-1.602	2.266	0.026
ENSG00000275004	ZNF280B	0.258	4.472	2.299	0.024
ENSG00000275014	RN7SL166P	0.544	-0.532	2.637	0.010

ENSG00000275090	RN7SL296P	0.923	-1.871	2.127	0.037
ENSG00000275212	RP3-446N13.5	1.015	-1.697	2.587	0.012
ENSG00000275232	RP11-897M7.4	1.027	-3.128	1.994	0.050
ENSG00000276170	AC124789.1	-0.753	-1.173	-2.283	0.025
ENSG00000276174	RP11-687E1.2	-0.503	-0.990	-2.018	0.047
ENSG00000276317	RP11-93B14.10	-1.389	-2.486	-2.970	0.004
ENSG00000276547	PCDHGB5	-0.277	3.475	-2.034	0.045
ENSG00000276550	HERC2P2	-0.398	6.403	-2.029	0.046
ENSG00000276853	RP11-305O6.4	-0.409	-0.016	-2.088	0.040
ENSG00000277013	CTC-526N19.1	-0.796	-0.514	-2.021	0.047
ENSG00000277062	RN7SL88P	0.709	-0.128	2.026	0.046
ENSG00000277270	RP5-827E24.1	0.674	-0.070	3.276	0.002
ENSG00000277425	RP5-1116H23.3	-0.950	-1.158	-2.811	0.006
ENSG00000277452	RN7SL473P	0.808	-0.815	2.322	0.023
ENSG00000277496	RP11-93B14.9	-0.762	-0.507	-2.455	0.016
ENSG00000277531	LLNLF-187D8.1	0.362	5.839	3.368	0.001
ENSG00000277558	RP3-468O1.6	-0.892	-1.738	-2.687	0.009
ENSG00000277895	RP11-114F3.4	0.713	-1.561	2.503	0.014
ENSG00000277977	CTD-3018O17.5	-0.244	1.302	-2.062	0.043
ENSG00000278192	RP5-1056H1.2	-0.504	-0.080	-2.219	0.029
ENSG00000278434	RP11-709D24.8	-0.400	1.031	-2.400	0.019
ENSG00000278492	RP11-15A1.9	0.401	-0.229	2.025	0.046
ENSG00000278637	HIST1H4A	0.930	-1.652	2.321	0.023
ENSG00000278709	NKILA	0.525	-0.630	2.136	0.036
ENSG00000278768	BACE1-AS	-0.217	3.511	-2.442	0.017
ENSG00000278771	Metazoa_SRP	0.431	11.982	2.160	0.034
ENSG00000279026	RP3-414A15.12	-0.361	1.838	-2.164	0.034
ENSG00000279098	RP11-15B17.4	-0.739	-1.281	-2.554	0.013
ENSG00000279118	RP11-517I3.2	-0.433	4.524	-2.018	0.047
ENSG00000279191	RP11-803D5.1	-0.783	0.404	-2.438	0.017
ENSG00000279249	RP11-491F9.3	0.818	1.708	2.959	0.004
ENSG00000279259	RP11-334C17.3	0.561	-1.031	2.094	0.040
ENSG00000279349	RP11-147G7.3	-0.585	-0.655	-2.560	0.012
ENSG00000279565	RP4-570O12.3	0.490	-0.455	2.079	0.041
ENSG00000279568	RP11-20I23.5	-0.559	0.431	-2.786	0.007
ENSG00000279917	RP11-360N9.2	-0.460	0.611	-2.009	0.048
ENSG00000279980	GABARAPL3	0.467	0.319	2.733	0.008
ENSG00000280063	RP11-295D4.3	-0.468	1.520	-2.953	0.004
ENSG00000280268	RP11-147K16.3	-1.065	-3.173	-2.283	0.025
ENSG00000280281	RP11-340L3.1	0.882	-1.997	2.061	0.043
ENSG00000280284	RP11-154F14.3	1.053	-2.764	2.451	0.017
ENSG00000280350	LINC01726	-0.631	-0.668	-2.192	0.031
ENSG00000280399	RP11-80P20.3	0.293	2.176	2.010	0.048
ENSG00000280432	AP000962.1	-0.397	1.519	-2.118	0.037
ENSG00000280485	RP11-437J2.4	-0.357	0.692	-2.182	0.032
ENSG00000282390	RP11-29A19.2	-0.521	0.573	-2.519	0.014
ENSG00000282890	RP11-460M2.1	0.930	-1.889	2.191	0.032

ENSG00000283031	RP11-557N21.1	-0.449	0.641	-2.153	0.035
ENSG00000283057	RP11-417N10.5	-0.554	-0.814	-2.306	0.024
ENSG00000283058	RP11-358H12.1	0.432	1.618	2.018	0.047
ENSG00000283199	ABC13-4748860	-0.470	1.119	-2.266	0.026
ENSG00000283584	snoMe28S-Am2(	0.785	-1.917	2.196	0.031
ENSG00000283765	#N/A	-0.880	-2.072	-2.345	0.022
ENSG00000283828	#N/A	0.412	-0.360	2.199	0.031
ENSG00000284070	#N/A	-0.644	-0.961	-2.372	0.020
ENSG00000284523	#N/A	0.608	-1.352	2.078	0.041
ENSG00000284612	#N/A	0.924	-1.525	2.322	0.023
ENSG00000284808	#N/A	-1.160	-1.714	-3.013	0.004
ENSG00000285184	#N/A	-0.192	2.763	-2.084	0.040
ENSG00000285692	#N/A	-0.515	0.149	-2.102	0.039
ENSG00000285780	#N/A	0.732	-1.104	2.066	0.042
ENSG00000285980	#N/A	-0.343	2.008	-2.004	0.049

dlPFC

gene_id	gene_name	logFC	AveExpr	t	P.Value
ENSG00000000938	FGR	0.460	0.078	2.210	0.030
ENSG00000004776	HSPB6	-0.594	-0.761	-2.170	0.033
ENSG00000006788	MYH13	0.557	-2.035	2.079	0.041
ENSG00000008516	MMP25	0.738	-1.636	3.024	0.003
ENSG00000010319	SEMA3G	0.639	2.473	2.801	0.006
ENSG00000012779	ALOX5	0.469	1.286	2.098	0.039
ENSG00000019186	CYP24A1	1.046	-0.365	2.482	0.015
ENSG00000023330	ALAS1	0.206	4.913	2.131	0.036
ENSG00000027847	B4GALT7	0.175	2.427	2.112	0.038
ENSG00000033050	ABCF2	0.227	3.288	2.090	0.040
ENSG00000033867	SLC4A7	0.272	7.211	2.407	0.018
ENSG00000034510	TMSB10	0.264	8.701	2.037	0.045
ENSG00000035141	FAM136A	0.249	3.766	2.064	0.042
ENSG00000037280	FLT4	0.454	-0.264	2.255	0.027
ENSG00000038002	AGA	0.383	3.225	2.575	0.012
ENSG00000043462	LCP2	0.236	3.113	2.234	0.028
ENSG00000048162	NOP16	0.240	2.174	2.788	0.007
ENSG00000049541	RFC2	0.257	2.541	2.081	0.041
ENSG00000051523	CYBA	0.646	0.759	2.703	0.008
ENSG00000053501	USE1	0.303	2.714	2.449	0.016
ENSG00000057757	PITHD1	0.240	6.398	2.219	0.029
ENSG00000059378	PARP12	0.290	1.990	2.244	0.028
ENSG00000063180	CA11	0.250	6.660	2.156	0.034
ENSG00000064419	TNPO3	0.165	6.109	2.411	0.018
ENSG00000064652	SNX24	0.150	4.177	2.209	0.030
ENSG00000065526	SPEN	-0.134	7.797	-2.223	0.029
ENSG00000065970	FOXJ2	-0.128	4.236	-2.039	0.045
ENSG00000066735	KIF26A	0.691	0.149	3.740	0.000
ENSG00000069122	ADGRF5	0.260	5.402	2.256	0.027
ENSG00000071539	TRIP13	0.383	0.454	2.746	0.007
ENSG00000073282	TP63	0.842	-1.979	3.412	0.001
ENSG00000074964	ARHGEF10L	0.185	4.076	2.295	0.024
ENSG00000075673	ATP12A	0.804	-0.941	2.548	0.013
ENSG00000076944	STXBP2	0.470	-1.213	2.098	0.039
ENSG00000077312	SNRPA	0.192	2.702	2.197	0.031
ENSG00000077549	CAPZB	0.173	5.888	2.427	0.017
ENSG00000078304	PPP2R5C	0.171	7.424	2.090	0.040
ENSG00000081818	PCDHB4	-0.289	3.767	-2.619	0.011
ENSG00000084693	AGBL5	0.116	4.578	2.075	0.041
ENSG00000085491	SLC25A24	0.221	3.513	2.175	0.033
ENSG00000086730	LAT2	0.437	1.096	2.049	0.044
ENSG00000087995	METTL2A	0.127	4.001	2.066	0.042
ENSG00000089063	TMEM230	0.202	5.480	2.266	0.026
ENSG00000090487	SPG21	0.187	3.948	2.225	0.029
ENSG00000092203	TOX4	0.109	5.904	2.029	0.046

ENSG00000095485	CWF19L1	0.234	3.864	2.234	0.028
ENSG00000096433	ITPR3	0.408	2.086	2.728	0.008
ENSG00000100058	CRYBB2P1	-0.212	3.638	-2.040	0.045
ENSG00000100216	TOMM22	0.252	5.549	2.452	0.016
ENSG00000100249	C22orf31	-0.919	-2.370	-2.838	0.006
ENSG00000100297	MCM5	0.354	1.438	2.250	0.027
ENSG00000100461	RBM23	0.146	5.617	2.486	0.015
ENSG00000100473	COCH	0.263	1.678	2.236	0.028
ENSG00000100558	PLEK2	-0.593	-2.530	-2.050	0.044
ENSG00000100632	ERH	0.191	4.766	2.013	0.047
ENSG00000101246	ARFRP1	0.165	3.747	2.034	0.045
ENSG00000101773	RBBP8	0.183	4.717	2.055	0.043
ENSG00000102452	NALCN	0.164	7.455	2.062	0.042
ENSG00000102897	LYRM1	0.239	3.867	2.543	0.013
ENSG00000103313	MEFV	0.354	0.339	2.037	0.045
ENSG00000104231	ZFAND1	0.228	5.177	2.061	0.043
ENSG00000104863	LIN7B	0.353	2.263	2.161	0.034
ENSG00000104870	FCGRT	0.302	2.226	2.218	0.029
ENSG00000104903	LYL1	0.617	-1.106	2.016	0.047
ENSG00000104904	OAZ1	0.264	6.511	2.172	0.033
ENSG00000104972	LILRB1	0.644	-0.067	2.188	0.032
ENSG00000104973	MED25	-0.223	2.836	-2.105	0.038
ENSG00000105171	POP4	0.240	3.833	2.254	0.027
ENSG00000105928	DFNA5	-0.166	4.641	-2.030	0.046
ENSG00000106245	BUD31	0.234	3.946	2.121	0.037
ENSG00000106636	YKT6	0.195	5.091	3.077	0.003
ENSG00000106682	EIF4H	0.182	6.912	2.070	0.042
ENSG00000106991	ENG	0.337	1.790	2.099	0.039
ENSG00000107566	ERLIN1	0.113	3.913	2.026	0.046
ENSG00000107719	PALD1	0.314	3.258	2.484	0.015
ENSG00000107736	CDH23	0.317	2.043	2.213	0.030
ENSG00000108518	PFN1	0.286	3.927	2.607	0.011
ENSG00000108587	GOSR1	0.144	5.876	3.168	0.002
ENSG00000108771	DHX58	0.249	2.462	2.119	0.037
ENSG00000108830	RND2	0.224	4.462	2.466	0.016
ENSG00000109390	NDUFC1	0.237	3.767	2.347	0.021
ENSG00000109854	HTATIP2	0.212	3.244	2.040	0.045
ENSG00000110031	LPXN	0.169	3.568	2.053	0.043
ENSG00000110057	UNC93B1	0.279	0.861	2.028	0.046
ENSG00000110711	AIP	0.280	3.515	2.175	0.033
ENSG00000110719	TCIRG1	0.542	-0.218	2.424	0.018
ENSG00000111364	DDX55	0.144	4.281	2.037	0.045
ENSG00000111906	HDDC2	0.170	5.751	2.265	0.026
ENSG00000112096	SOD2	0.244	7.928	2.085	0.040
ENSG00000112651	MRPL2	0.166	3.083	2.032	0.045
ENSG00000112799	LY86	0.549	0.463	2.056	0.043
ENSG00000113272	THG1L	0.267	2.290	2.787	0.007

ENSG00000113552	GNPDA1	0.167	4.656	2.031	0.046
ENSG00000114125	RNF7	0.176	3.869	2.096	0.039
ENSG00000114383	TUSC2	0.183	4.345	2.067	0.042
ENSG00000115267	IFIH1	0.305	2.734	2.047	0.044
ENSG00000115325	DOK1	0.352	-0.194	2.086	0.040
ENSG00000115657	ABCB6	-0.287	1.568	-2.191	0.031
ENSG00000116883	RP11-268J15.5	-0.308	2.053	-1.995	0.049
ENSG00000116983	HPCAL4	0.195	8.470	2.305	0.024
ENSG00000117676	RPS6KA1	0.382	2.184	2.459	0.016
ENSG00000117862	TXNDC12	0.267	2.411	2.497	0.015
ENSG00000118520	ARG1	0.414	-0.216	2.341	0.022
ENSG00000118680	MYL12B	0.236	5.875	1.997	0.049
ENSG00000119508	NR4A3	-0.476	3.812	-2.253	0.027
ENSG00000119686	FLVCR2	0.537	-0.535	2.391	0.019
ENSG00000120129	DUSP1	-0.524	4.092	-2.698	0.008
ENSG00000120217	CD274	0.236	2.289	2.046	0.044
ENSG00000120306	CYSTM1	0.209	3.697	2.279	0.025
ENSG00000120738	EGR1	-0.492	4.530	-2.066	0.042
ENSG00000121005	CRISPLD1	0.321	3.130	2.093	0.039
ENSG00000121281	ADCY7	0.304	1.833	2.225	0.029
ENSG00000121377	TAS2R7	-0.548	-0.777	-2.048	0.044
ENSG00000122203	KIAA1191	0.163	6.400	2.278	0.025
ENSG00000122386	ZNF205	0.255	1.680	2.079	0.041
ENSG00000122952	ZWINT	0.334	1.075	2.011	0.048
ENSG00000123154	WDR83	0.298	1.226	2.076	0.041
ENSG00000123358	NR4A1	-0.644	2.929	-2.411	0.018
ENSG00000123416	TUBA1B	0.299	7.483	2.458	0.016
ENSG00000123552	USP45	0.167	6.007	2.284	0.025
ENSG00000124104	SNX21	0.137	3.225	2.048	0.044
ENSG00000124243	BCAS4	0.178	5.245	1.993	0.050
ENSG00000125319	C17orf53	-0.408	-1.174	-2.085	0.040
ENSG00000125384	PTGER2	-1.173	-2.293	-3.688	0.000
ENSG00000125449	ARMC7	0.247	1.359	2.628	0.010
ENSG00000125637	PSD4	0.233	2.547	2.068	0.042
ENSG00000125740	FOSB	-0.604	2.195	-2.552	0.013
ENSG00000125818	PSMF1	0.114	5.392	2.277	0.025
ENSG00000126231	PROZ	-0.950	-2.058	-2.885	0.005
ENSG00000126749	EMG1	0.134	3.501	2.100	0.039
ENSG00000127125	PPCS	0.238	4.015	2.215	0.030
ENSG00000127184	COX7C	0.325	6.028	2.105	0.038
ENSG00000128340	RAC2	0.587	-1.212	2.437	0.017
ENSG00000128581	IFT22	0.200	4.198	2.198	0.031
ENSG00000128604	IRF5	0.459	0.886	3.083	0.003
ENSG00000128917	DLL4	-0.421	0.897	-2.055	0.043
ENSG00000129103	SUMF2	0.184	4.244	2.215	0.030
ENSG00000129467	ADCY4	0.665	0.478	3.148	0.002
ENSG00000130307	USHBP1	0.643	-0.135	2.817	0.006

ENSG00000130312	MRPL34	0.227	3.000	2.208	0.030
ENSG00000130332	LSM7	0.256	1.243	2.143	0.035
ENSG00000130429	ARPC1B	0.311	-0.154	2.230	0.028
ENSG00000130520	LSM4	0.215	4.609	2.197	0.031
ENSG00000130717	UCK1	0.174	3.761	2.006	0.048
ENSG00000130770	ATPIF1	0.197	5.791	2.073	0.041
ENSG00000130813	C19orf66	0.165	3.200	2.298	0.024
ENSG00000130856	ZNF236	0.118	6.221	2.376	0.020
ENSG00000130956	HABP4	0.223	7.000	2.109	0.038
ENSG00000131408	NR1H2	0.162	4.200	1.999	0.049
ENSG00000131480	AOC2	-0.430	0.108	-2.259	0.027
ENSG00000131495	NDUFA2	0.292	2.556	2.173	0.033
ENSG00000131508	UBE2D2	0.152	5.740	2.009	0.048
ENSG00000131951	LRRC9	0.196	2.309	2.175	0.032
ENSG00000132622	HSPA12B	0.778	1.227	3.657	0.000
ENSG00000132749	TESMIN	0.421	-0.716	2.143	0.035
ENSG00000133055	MYBPH	-0.461	-0.099	-2.209	0.030
ENSG00000133193	FAM104A	0.136	3.503	2.034	0.045
ENSG00000133313	CNDP2	0.179	6.590	2.020	0.047
ENSG00000133398	MED10	0.239	3.491	2.590	0.011
ENSG00000133597	ADCK2	0.252	3.339	2.600	0.011
ENSG00000133665	DYDC2	0.521	0.096	2.226	0.029
ENSG00000133863	TEX15	-0.233	3.209	-2.069	0.042
ENSG00000134020	PEBP4	0.394	0.395	2.568	0.012
ENSG00000134326	CMPK2	0.233	2.831	2.088	0.040
ENSG00000134779	TPGS2	0.133	6.096	2.090	0.040
ENSG00000134940	ACRV1	0.484	-0.774	2.610	0.011
ENSG00000135124	P2RX4	0.235	2.778	2.356	0.021
ENSG00000135218	CD36	0.361	0.876	2.401	0.019
ENSG00000135363	LMO2	0.370	3.391	2.061	0.042
ENSG00000135960	EDAR	-0.646	-1.330	-2.921	0.005
ENSG00000136003	ISCU	0.219	6.796	2.074	0.041
ENSG00000136026	CKAP4	0.181	4.344	2.282	0.025
ENSG00000136169	SETDB2	0.123	5.140	1.992	0.050
ENSG00000136352	NKX2-1	-0.845	-2.470	-2.736	0.008
ENSG00000136425	CIB2	0.337	0.731	2.164	0.033
ENSG00000136718	IMP4	0.214	5.073	2.809	0.006
ENSG00000136891	TEX10	-0.103	5.321	-2.364	0.020
ENSG00000137038	TMEM261	0.186	4.524	2.004	0.048
ENSG00000137080	IFNA21	-0.587	-1.111	-2.175	0.033
ENSG00000137491	SLCO2B1	0.312	4.583	2.024	0.046
ENSG00000137747	TMPRSS13	1.105	-3.057	2.468	0.016
ENSG00000137817	PARP6	0.100	5.550	2.131	0.036
ENSG00000138375	SMARCAL1	0.147	3.414	2.850	0.006
ENSG00000138400	MDH1B	0.244	2.653	2.060	0.043
ENSG00000138821	SLC39A8	0.236	3.819	2.128	0.036
ENSG00000138829	FBN2	0.345	1.338	2.670	0.009

ENSG00000139209	SLC38A4	-0.529	-0.221	-2.963	0.004
ENSG00000139567	ACVRL1	0.549	1.853	2.459	0.016
ENSG00000139620	KANSL2	0.178	3.726	2.225	0.029
ENSG00000140105	WARS	0.307	6.175	1.996	0.049
ENSG00000140368	PSTPIP1	0.449	-0.435	2.542	0.013
ENSG00000140382	HMG20A	0.117	5.806	2.035	0.045
ENSG00000140941	MAP1LC3B	0.144	6.894	2.189	0.031
ENSG00000140987	ZSCAN32	0.326	-0.230	2.393	0.019
ENSG00000141480	ARRB2	0.165	4.214	2.439	0.017
ENSG00000141505	ASGR1	0.306	0.937	2.165	0.033
ENSG00000141968	VAV1	0.512	1.065	2.494	0.015
ENSG00000142676	RPL11	0.233	6.329	2.062	0.042
ENSG00000142871	CYR61	-0.613	2.157	-2.956	0.004
ENSG00000143222	UFC1	0.216	4.109	2.029	0.046
ENSG00000143318	CASQ1	0.359	3.811	2.084	0.040
ENSG00000143365	RORC	-0.728	-2.272	-2.128	0.036
ENSG00000143367	TUFT1	0.191	3.212	2.134	0.036
ENSG00000143520	FLG2	-0.584	-0.240	-2.457	0.016
ENSG00000143549	TPM3	0.253	7.576	2.274	0.026
ENSG00000143612	C1orf43	0.180	6.256	2.287	0.025
ENSG00000143727	ACP1	0.194	6.381	2.073	0.041
ENSG00000143970	ASXL2	-0.096	6.612	-2.165	0.033
ENSG00000144118	RALB	0.255	6.426	2.430	0.017
ENSG00000144659	SLC25A38	0.164	4.007	2.065	0.042
ENSG00000145283	SLC10A6	0.722	-1.681	2.302	0.024
ENSG00000145384	FABP2	-0.930	-2.259	-3.107	0.003
ENSG00000145494	NDUFS6	0.350	3.207	2.223	0.029
ENSG00000145545	SRD5A1	0.212	5.822	2.002	0.049
ENSG00000145723	GIN1	0.174	4.059	2.122	0.037
ENSG00000145868	FBXO38	0.130	6.045	2.281	0.025
ENSG00000145912	NHP2	0.390	2.856	2.535	0.013
ENSG00000145919	BOD1	0.138	4.530	2.194	0.031
ENSG00000147535	PLPP5	-0.134	3.639	-2.006	0.048
ENSG00000148335	NTMT1	0.217	1.781	2.065	0.042
ENSG00000148702	HABP2	-0.382	0.221	-2.239	0.028
ENSG00000148803	FUOM	0.919	-1.668	3.123	0.002
ENSG00000148926	ADM	-0.818	0.669	-2.354	0.021
ENSG00000149476	TKFC	0.192	2.869	2.069	0.042
ENSG00000149636	DSN1	0.210	2.889	2.052	0.043
ENSG00000149656	LINC00266-1	-0.539	0.525	-2.136	0.036
ENSG00000149761	NUDT22	0.276	1.215	2.645	0.010
ENSG00000149781	FERMT3	0.474	-0.145	2.108	0.038
ENSG00000150455	TIRAP	-0.240	1.650	-2.157	0.034
ENSG00000150681	RGS18	0.900	-0.530	2.178	0.032
ENSG00000151360	ALLC	-1.366	-2.497	-2.466	0.016
ENSG00000151365	THRSP	-0.628	-1.484	-2.309	0.023
ENSG00000151388	ADAMTS12	0.451	-0.912	2.026	0.046

ENSG00000151632	AKR1C2	0.310	2.181	2.174	0.033
ENSG00000151892	GFRA1	-0.267	5.340	-2.230	0.028
ENSG00000152315	KCNK13	0.402	-0.485	2.047	0.044
ENSG00000152380	FAM151B	0.138	2.631	2.034	0.045
ENSG00000152779	SLC16A12	0.718	-1.763	2.015	0.047
ENSG00000153214	TMEM87B	0.180	4.503	2.078	0.041
ENSG00000153234	NR4A2	-0.563	3.341	-2.480	0.015
ENSG00000153291	SLC25A27	0.170	6.228	2.115	0.037
ENSG00000154144	TBRG1	0.135	6.541	2.016	0.047
ENSG00000154330	PGM5	0.421	2.210	2.400	0.019
ENSG00000154734	ADAMTS1	-0.410	4.198	-2.098	0.039
ENSG00000156171	DRAM2	0.204	3.515	2.031	0.045
ENSG00000156384	SFR1	0.296	1.870	2.293	0.024
ENSG00000156427	FGF18	0.552	-1.419	2.280	0.025
ENSG00000156853	ZNF689	0.158	3.364	2.067	0.042
ENSG00000156928	MALSU1	0.186	3.201	2.206	0.030
ENSG00000156970	BUB1B	0.425	-0.280	2.388	0.019
ENSG00000157017	GHRL	-0.423	-0.110	-2.741	0.008
ENSG00000157510	AFAP1L1	0.270	1.769	2.052	0.043
ENSG00000157601	MX1	0.291	5.037	2.102	0.039
ENSG00000158457	TSPAN33	0.208	3.686	2.279	0.025
ENSG00000158636	EMSY	-0.118	5.644	-2.310	0.023
ENSG00000159111	MRPL10	0.161	3.508	2.153	0.034
ENSG00000159259	CHAF1B	0.233	2.755	2.690	0.009
ENSG00000159346	ADIPOR1	0.140	5.775	2.272	0.026
ENSG00000159388	BTG2	-0.551	4.043	-2.674	0.009
ENSG00000160075	SSU72	0.204	5.650	2.487	0.015
ENSG00000160124	CCDC58	0.206	2.632	2.229	0.029
ENSG00000160446	ZDHHC12	0.501	-0.607	2.150	0.035
ENSG00000160710	ADAR	0.103	8.391	2.075	0.041
ENSG00000160803	UBQLN4	0.174	5.256	2.781	0.007
ENSG00000160808	MYL3	0.752	-1.222	3.197	0.002
ENSG00000160870	CYP3A7	-0.479	0.569	-2.910	0.005
ENSG00000161647	MPP3	0.314	2.156	3.024	0.003
ENSG00000162755	KLHDC9	0.276	2.241	2.182	0.032
ENSG00000163053	SLC16A14	0.187	5.459	2.093	0.039
ENSG00000163138	PACRGL	0.133	4.919	2.333	0.022
ENSG00000163191	S100A11	0.948	-0.440	3.133	0.002
ENSG00000163378	EOGT	0.172	3.862	2.403	0.019
ENSG00000163479	SSR2	0.166	4.048	2.126	0.037
ENSG00000163608	NEPRO	0.133	5.663	2.207	0.030
ENSG00000163673	DCLK3	0.341	2.709	2.322	0.023
ENSG00000163923	RPL39L	0.394	0.496	2.568	0.012
ENSG00000164120	HPGD	0.595	-0.091	2.129	0.036
ENSG00000164123	C4orf45	0.343	-0.060	2.083	0.040
ENSG00000164142	FAM160A1	-0.267	1.800	-2.001	0.049
ENSG00000164167	LSM6	0.260	3.026	2.124	0.037

ENSG00000164867	NOS3	0.428	0.555	2.311	0.023
ENSG00000165185	KIAA1958	-0.175	5.191	-2.011	0.048
ENSG00000165457	FOLR2	0.725	-0.707	2.244	0.028
ENSG00000165879	FRAT1	0.288	0.979	2.063	0.042
ENSG00000166199	ALKBH3	0.247	3.349	2.481	0.015
ENSG00000166278	C2	0.381	-0.581	2.197	0.031
ENSG00000166426	CRABP1	0.866	-0.578	3.726	0.000
ENSG00000166582	CENPV	0.176	3.532	2.300	0.024
ENSG00000166825	ANPEP	0.549	-0.981	2.401	0.019
ENSG00000167604	NFKBID	-0.477	0.298	-2.536	0.013
ENSG00000167645	YIF1B	0.261	1.784	2.230	0.028
ENSG00000167658	EEF2	0.212	8.990	2.107	0.038
ENSG00000167754	KLK5	0.636	0.063	2.041	0.045
ENSG00000167778	SPRYD3	0.173	6.562	2.076	0.041
ENSG00000168079	SCARA5	0.516	0.398	2.248	0.027
ENSG00000168348	INSM2	0.334	1.193	2.342	0.022
ENSG00000168411	RFWD3	0.187	3.270	2.374	0.020
ENSG00000168418	KCNG4	-0.950	-1.772	-2.592	0.011
ENSG00000169220	RGS14	0.415	2.436	2.473	0.015
ENSG00000169429	CXCL8	1.017	-1.058	2.093	0.039
ENSG00000169738	DCXR	0.240	3.102	2.061	0.043
ENSG00000169902	TPST1	0.205	4.824	2.359	0.021
ENSG00000169964	TMEM42	0.184	2.618	2.581	0.012
ENSG00000170006	TMEM154	0.428	0.677	2.514	0.014
ENSG00000170231	FABP6	0.418	2.049	2.293	0.024
ENSG00000170345	FOS	-0.865	4.182	-2.563	0.012
ENSG00000170509	HSD17B13	0.385	-0.055	1.994	0.050
ENSG00000170540	ARL6IP1	0.192	8.630	1.995	0.049
ENSG00000170946	DNAJC24	0.155	4.586	2.093	0.039
ENSG00000170959	DCDC1	-0.223	2.471	-2.282	0.025
ENSG00000171135	JAGN1	0.204	2.409	2.010	0.048
ENSG00000171223	JUNB	-0.473	2.906	-2.052	0.043
ENSG00000171561	OR2AT4	-0.635	-0.043	-2.663	0.009
ENSG00000171722	C1orf111	-0.479	0.475	-2.508	0.014
ENSG00000171819	ANGPTL7	-0.372	0.365	-2.340	0.022
ENSG00000171864	PRND	1.553	-3.039	3.152	0.002
ENSG00000172331	BPGM	0.217	4.380	2.183	0.032
ENSG00000172432	GTPBP2	0.103	3.894	2.072	0.041
ENSG00000172572	PDE3A	-0.250	3.297	-2.023	0.046
ENSG00000172922	RNASEH2C	0.131	3.003	2.035	0.045
ENSG00000173404	INSM1	-0.394	0.391	-2.050	0.044
ENSG00000173442	EHBP1L1	0.233	3.002	2.518	0.014
ENSG00000173812	EIF1	0.151	6.838	2.076	0.041
ENSG00000174137	FAM53A	0.619	-1.431	2.101	0.039
ENSG00000174485	DENND4A	0.130	7.315	2.251	0.027
ENSG00000175544	CABP4	0.585	-1.255	2.227	0.029
ENSG00000175564	UCP3	-0.379	1.065	-2.407	0.018

ENSG00000175567	UCP2	0.608	0.136	2.974	0.004
ENSG00000175779	C15orf53	-0.558	-1.154	-2.464	0.016
ENSG00000177106	EPS8L2	0.362	1.211	2.192	0.031
ENSG00000177963	RIC8A	0.150	5.089	2.171	0.033
ENSG00000178175	ZNF366	0.471	1.052	2.399	0.019
ENSG00000178397	FAM220A	0.207	4.511	2.105	0.038
ENSG00000178878	APOLD1	-0.573	5.195	-2.037	0.045
ENSG00000179008	C14orf39	0.759	-2.177	2.800	0.006
ENSG00000179041	RRS1	0.226	2.314	2.461	0.016
ENSG00000179855	GIPC3	1.085	-2.075	3.285	0.002
ENSG00000179922	ZNF784	0.320	0.045	2.018	0.047
ENSG00000180479	ZNF571	-0.139	3.852	-2.475	0.015
ENSG00000180803	RP11-467D10.2	-0.617	-1.730	-2.416	0.018
ENSG00000182117	NOP10	0.265	3.049	2.247	0.027
ENSG00000182362	YBEY	0.269	1.935	2.215	0.030
ENSG00000182534	MXRA7	0.170	6.142	2.390	0.019
ENSG00000182853	VMO1	0.710	-2.675	2.048	0.044
ENSG00000182873	RP11-181G12.2	-0.496	-0.141	-2.643	0.010
ENSG00000183346	C10orf107	0.394	2.074	2.354	0.021
ENSG00000183833	MAATS1	-0.236	2.646	-2.392	0.019
ENSG00000183978	COA3	0.239	3.535	2.115	0.037
ENSG00000184601	C14orf180	-0.452	-0.821	-2.141	0.035
ENSG00000184708	EIF4ENIF1	0.094	5.214	2.062	0.042
ENSG00000184898	RBM43	0.211	2.592	2.048	0.044
ENSG00000184900	SUMO3	0.296	5.484	2.442	0.017
ENSG00000185149	NPY2R	0.386	2.373	2.020	0.047
ENSG00000185215	TNFAIP2	0.373	2.724	3.162	0.002
ENSG00000185880	TRIM69	0.176	6.175	2.154	0.034
ENSG00000186352	ANKRD37	-0.454	1.454	-2.266	0.026
ENSG00000187066	TMEM262	0.706	-2.035	2.204	0.030
ENSG00000187566	NHLRC1	0.248	1.328	2.027	0.046
ENSG00000187994	RINL	0.334	1.587	2.688	0.009
ENSG00000188305	C19orf35	0.639	-2.389	2.154	0.034
ENSG00000188315	C3orf62	-0.146	3.080	-2.043	0.044
ENSG00000188690	UROS	0.201	5.453	2.020	0.047
ENSG00000188785	ZNF548	-0.116	4.231	-2.239	0.028
ENSG00000189149	CRYM-AS1	0.548	-1.459	2.069	0.042
ENSG00000196411	EPHB4	0.337	0.550	2.066	0.042
ENSG00000196655	TRAPPC4	0.265	3.632	2.681	0.009
ENSG00000196844	PATE2	-0.268	0.845	-2.072	0.041
ENSG00000197585	AC107218.3	-0.425	-0.584	-2.471	0.016
ENSG00000197728	RPS26	0.739	2.122	2.365	0.020
ENSG00000197879	MYO1C	0.195	2.799	2.081	0.041
ENSG00000197956	S100A6	0.358	4.701	2.140	0.035
ENSG00000198001	IRAK4	0.221	2.688	2.153	0.034
ENSG00000198246	SLC29A3	0.254	1.581	2.355	0.021
ENSG00000198356	ASNA1	0.264	4.167	2.416	0.018

ENSG00000198711	SSBP3-AS1	-0.355	1.357	-2.215	0.030
ENSG00000198829	SUCNR1	1.081	-0.720	2.232	0.028
ENSG00000198844	ARHGEF15	0.548	0.114	2.770	0.007
ENSG00000198937	CCDC167	0.288	0.434	2.249	0.027
ENSG00000199266	SNORA60	0.574	-0.020	2.100	0.039
ENSG00000199585	RNA5SP119	-0.627	-1.384	-2.422	0.018
ENSG00000200502	Y_RNA	-0.500	-1.505	-2.267	0.026
ENSG00000200792	SNORA80A	0.853	-2.940	2.686	0.009
ENSG00000201326	SNORD115-22	-0.521	-0.061	-2.640	0.010
ENSG00000201643	SNORA14A	0.985	-2.397	2.888	0.005
ENSG00000201852	RNU6-702P	1.016	-3.928	2.228	0.029
ENSG00000202354	RNY3	-0.450	0.425	-2.081	0.041
ENSG00000202373	SNORD115-43	-0.549	-1.338	-2.632	0.010
ENSG00000203441	LINC00449	0.586	-2.446	2.057	0.043
ENSG00000204315	FKBPL	0.254	1.306	2.218	0.029
ENSG00000204338	CYP21A1P	-1.038	-2.537	-2.613	0.011
ENSG00000204392	LSM2	0.272	1.374	2.573	0.012
ENSG00000204498	NFKBIL1	0.192	1.934	2.186	0.032
ENSG00000204525	HLA-C	0.446	4.312	2.303	0.024
ENSG00000204556	CTD-2514C3.1	-0.597	-0.995	-2.104	0.038
ENSG00000204909	SPINK9	-0.429	0.255	-2.268	0.026
ENSG00000205309	NT5M	0.269	1.266	2.385	0.019
ENSG00000205659	LIN52	0.106	4.239	2.013	0.047
ENSG00000205744	DENND1C	0.403	-0.495	2.393	0.019
ENSG00000205758	CRYZL1	0.157	5.224	2.022	0.046
ENSG00000206145	P2RX6P	0.558	-0.641	2.229	0.029
ENSG00000206181	TCEB3B	-0.531	-0.575	-2.112	0.038
ENSG00000206562	METTL6	0.114	4.664	2.172	0.033
ENSG00000206786	RNU6-701P	-0.572	-1.823	-2.577	0.012
ENSG00000206812	RNU6-38P	-1.277	-4.571	-2.935	0.004
ENSG00000207110	RNU1-106P	0.954	-3.962	2.226	0.029
ENSG00000207425	Y_RNA	-0.669	-1.959	-2.371	0.020
ENSG00000207428	RNU6-95P	-0.641	-1.701	-2.055	0.043
ENSG00000207460	SNORD116-19	-0.412	0.306	-2.111	0.038
ENSG00000210176	MT-TH	1.015	-1.363	2.668	0.009
ENSG00000210195	MT-TT	0.741	-1.554	2.282	0.025
ENSG00000210196	MT-TP	0.341	1.588	2.014	0.047
ENSG00000212402	SNORA74B	-0.651	1.667	-2.686	0.009
ENSG00000212525	RNA5SP212	-0.696	-2.553	-2.099	0.039
ENSG00000212526	RNU6-466P	-0.685	-2.499	-1.995	0.049
ENSG00000213203	GIMAP1	0.601	0.212	2.320	0.023
ENSG00000213221	DNLZ	0.858	-2.394	2.672	0.009
ENSG00000213237	AC008155.1	-0.614	-1.468	-2.565	0.012
ENSG00000213484	EIF4A1P8	-0.538	-0.551	-2.614	0.011
ENSG00000213755	RP11-342F17.1	-0.492	-0.035	-2.452	0.016
ENSG00000214087	ARL16	0.180	3.692	2.100	0.039
ENSG00000214725	CDIPT-AS1	0.783	-1.926	2.790	0.007

ENSG00000215399	HMGB3P7	-0.500	0.607	-2.036	0.045
ENSG00000215421	ZNF407	0.110	6.159	2.070	0.042
ENSG00000217330	SSXP10	-0.454	-0.281	-2.170	0.033
ENSG00000217929	CICP18	-0.776	-0.909	-2.378	0.020
ENSG00000218631	RP3-395C13.1	0.555	-1.955	2.051	0.044
ENSG00000220804	LINC01881	0.261	2.552	2.357	0.021
ENSG00000221983	UBA52	0.167	6.212	2.163	0.033
ENSG00000222844	RNU6-321P	-0.628	-1.760	-2.212	0.030
ENSG00000222932	RNU6-172P	-0.590	-2.543	-2.117	0.037
ENSG00000223351	ZNF385D-AS2	-0.803	-1.491	-3.106	0.003
ENSG00000223583	RP3-476K8.4	0.582	-1.592	2.673	0.009
ENSG00000223797	ENTPD3-AS1	-0.263	1.601	-2.632	0.010
ENSG00000223799	IL10RB-AS1	-0.412	-0.180	-2.805	0.006
ENSG00000224032	EPB41L4A-AS1	0.231	4.134	2.351	0.021
ENSG00000224295	OLFM5P	-0.884	-2.159	-2.429	0.017
ENSG00000224414	AC010886.2	-0.426	0.489	-2.283	0.025
ENSG00000224613	RP11-153F1.1	0.412	-0.306	2.111	0.038
ENSG00000224616	RTCA-AS1	0.281	0.318	2.068	0.042
ENSG00000224731	AC016716.2	-0.431	1.702	-3.029	0.003
ENSG00000224973	LARGE-AS1	-0.375	2.052	-2.153	0.034
ENSG00000225032	RP11-228B15.4	-0.383	-0.535	-2.084	0.040
ENSG00000225365	AC078942.1	-0.644	-1.550	-2.310	0.023
ENSG00000225437	PACRG-AS2	-0.608	-1.763	-2.228	0.029
ENSG00000225492	GBP1P1	0.656	-0.035	2.830	0.006
ENSG00000225648	SBDSP1	0.159	4.255	2.517	0.014
ENSG00000225681	AC005730.2	-0.521	-1.392	-2.414	0.018
ENSG00000225726	AC007000.10	-0.559	-0.120	-2.434	0.017
ENSG00000225779	RP4-603I14.3	-0.776	-2.331	-2.869	0.005
ENSG00000226149	RP1-69D17.4	0.324	0.403	2.254	0.027
ENSG00000226276	AC093382.1	-0.997	-2.945	-2.592	0.011
ENSG00000226318	RP11-474D14.2	-0.321	2.255	-2.269	0.026
ENSG00000226323	AC006150.1	-0.489	-0.319	-2.092	0.040
ENSG00000226334	RP11-217B7.2	0.897	-3.120	2.301	0.024
ENSG00000226380	MIR29A	-0.238	4.594	-2.029	0.046
ENSG00000226393	IFNA20P	-0.401	-1.119	-2.027	0.046
ENSG00000226454	RP3-462C17.1	-0.516	0.169	-2.236	0.028
ENSG00000226580	RPL39P40	-0.836	-2.942	-2.088	0.040
ENSG00000226926	PDZPH1P	-0.581	-1.751	-2.171	0.033
ENSG00000227021	AC007557.3	-0.490	-0.904	-2.048	0.044
ENSG00000227073	SDHDP2	-0.569	-1.240	-2.437	0.017
ENSG00000227455	RP11-300M24.1	0.484	-0.498	2.879	0.005
ENSG00000227456	LINC00310	-0.432	0.237	-2.708	0.008
ENSG00000227646	STEAP2-AS1	0.576	-1.834	2.070	0.042
ENSG00000228022	HCG20	-0.586	-1.839	-2.115	0.037
ENSG00000228133	AC099684.1	-0.447	0.634	-2.446	0.017
ENSG00000228195	RPL5P27	-0.680	-0.424	-2.472	0.016
ENSG00000228352	RP11-537H15.3	0.571	-1.859	2.254	0.027

ENSG00000228391	AC011995.3	-0.810	-1.613	-2.789	0.007
ENSG00000228481	U51244.2	-0.439	0.221	-2.537	0.013
ENSG00000228680	AC004692.4	-0.436	-0.091	-2.170	0.033
ENSG00000228740	GABRG3-AS1	-0.543	0.087	-2.615	0.011
ENSG00000228793	RP1-223B1.1	-1.040	-1.243	-2.393	0.019
ENSG00000228839	PIK3IP1-AS1	-0.442	-1.389	-2.120	0.037
ENSG00000229042	RP1-232N11.2	-0.564	-0.579	-2.330	0.022
ENSG00000229117	RPL41	0.239	4.985	2.399	0.019
ENSG00000229267	AC072062.1	-0.522	-0.613	-2.117	0.037
ENSG00000229325	ACAP2-IT1	-0.247	2.437	-2.177	0.032
ENSG00000229536	LINC02572	0.891	-2.205	3.306	0.001
ENSG00000229673	LL22NC01-116C6.:	-1.100	-1.818	-3.655	0.000
ENSG00000229870	RP11-507K13.6	-0.615	-2.384	-2.227	0.029
ENSG00000229938	MYO16-AS2	-0.490	-1.509	-2.068	0.042
ENSG00000230054	RP11-402G3.5	-0.517	-0.878	-2.238	0.028
ENSG00000230155	RP3-477O4.14	-0.780	-2.312	-2.749	0.007
ENSG00000230189	GS1-124K5.2	-0.422	-1.229	-2.628	0.010
ENSG00000230212	AP000688.14	-0.552	-2.214	-2.255	0.027
ENSG00000230310	CTD-2192J16.11	-0.762	-2.100	-2.788	0.007
ENSG00000230470	GS1-115G20.1	-0.413	0.037	-2.211	0.030
ENSG00000230551	CTB-89H12.4	-0.247	4.784	-2.019	0.047
ENSG00000230593	AC090804.1	-0.477	-1.235	-2.196	0.031
ENSG00000230661	YY1P1	0.702	-2.024	2.301	0.024
ENSG00000230735	RP11-413E1.4	-0.367	-0.499	-2.282	0.025
ENSG00000230782	RP11-508N12.2	-0.365	0.181	-2.101	0.039
ENSG00000230826	RP11-417B4.2	-0.312	0.362	-2.262	0.026
ENSG00000231329	RP1-225E12.2	-0.244	0.973	-2.110	0.038
ENSG00000231336	AC017006.3	-0.661	-1.448	-3.229	0.002
ENSG00000231449	AC097359.2	-0.830	-1.820	-2.868	0.005
ENSG00000231744	RP11-164H5.1	-0.605	-0.582	-2.319	0.023
ENSG00000231758	AC092652.1	0.511	-1.498	2.341	0.022
ENSG00000231827	RP11-216N14.5	-0.719	-2.261	-3.043	0.003
ENSG00000232036	GS1-115G20.2	-0.467	-0.138	-2.409	0.018
ENSG00000232327	RP11-395C17.1	-0.728	-1.957	-1.997	0.049
ENSG00000232455	LARS2-AS1	-0.556	-2.004	-2.131	0.036
ENSG00000232466	RP11-337A23.5	-0.514	-1.091	-2.057	0.043
ENSG00000232762	RP4-784A16.4	-0.564	-1.654	-2.071	0.042
ENSG00000232859	LYRM9	0.220	3.399	2.238	0.028
ENSG00000232876	CTA-212D2.2	0.688	-1.535	2.254	0.027
ENSG00000232940	HCG25	-0.454	-1.225	-2.125	0.037
ENSG00000233329	AC068287.1	-0.698	-2.555	-2.030	0.046
ENSG00000233351	RP1-69D17.3	-0.613	-2.400	-2.022	0.046
ENSG00000233367	RP11-307L3.4	0.522	-1.567	2.493	0.015
ENSG00000233436	BTBD18	-0.289	1.757	-2.248	0.027
ENSG00000233519	RP11-177F11.1	-0.468	0.320	-2.252	0.027
ENSG00000233559	AC016831.7	-0.323	3.244	-1.991	0.050
ENSG00000233581	AC069155.1	-0.691	-1.747	-2.594	0.011

ENSG00000233623	PGAM1P11	-0.802	-2.578	-2.051	0.043
ENSG00000234336	JAZF1-AS1	-0.354	2.390	-2.119	0.037
ENSG00000234601	CHRM3-AS1	-0.449	-0.446	-2.604	0.011
ENSG00000234617	SNRK-AS1	-0.390	0.063	-2.293	0.024
ENSG00000234784	RP11-182I10.1	-0.789	-2.095	-2.550	0.013
ENSG00000235145	RPSAP16	-0.352	0.044	-2.070	0.042
ENSG00000235277	AF127577.8	-0.659	-1.861	-2.237	0.028
ENSG00000235279	RP11-539E19.2	-0.489	-1.017	-2.074	0.041
ENSG00000235284	SNORD62A	1.157	-3.762	3.148	0.002
ENSG00000235313	HM13-IT1	-0.657	-1.883	-2.195	0.031
ENSG00000235436	DPY19L2P4	-0.348	2.088	-2.383	0.020
ENSG00000235526	RP5-1028L10.2	-0.567	-0.285	-3.127	0.002
ENSG00000235579	AC007283.4	0.910	-2.730	2.933	0.004
ENSG00000235586	AC011247.3	-0.544	-1.921	-2.429	0.017
ENSG00000235621	LINC00494	0.854	-2.632	2.172	0.033
ENSG00000235628	TNR-IT1	-0.652	-0.986	-2.158	0.034
ENSG00000235785	AL109767.1	-0.674	-2.495	-2.150	0.035
ENSG00000235829	TBCAP2	-0.424	0.736	-2.114	0.038
ENSG00000235833	AC159540.14	-1.619	0.025	-2.380	0.020
ENSG00000235848	RMDN2-AS1	-0.463	-0.146	-2.543	0.013
ENSG00000235863	B3GALT4	0.226	1.163	2.113	0.038
ENSG00000235917	MTCO2P11	-0.786	-1.473	-2.606	0.011
ENSG00000236467	KCNMA1-AS1	-0.297	3.858	-2.048	0.044
ENSG00000236474	GCNT1P1	-0.355	0.010	-2.213	0.030
ENSG00000236532	LINC01695	0.495	-1.440	2.048	0.044
ENSG00000236592	S100A11P2	-0.662	-1.779	-1.990	0.050
ENSG00000236609	ZNF853	0.161	4.183	2.248	0.027
ENSG00000236758	MTUS2-AS2	-0.386	0.566	-2.150	0.035
ENSG00000236973	GAPDHP51	-0.808	-2.041	-2.929	0.004
ENSG00000237001	WASF3-AS1	-0.401	0.453	-2.157	0.034
ENSG00000237070	AC005550.3	-0.739	-1.937	-2.014	0.047
ENSG00000237088	RP11-73E6.2	-0.429	-0.740	-2.375	0.020
ENSG00000237329	RP11-201O14.1	-0.543	-1.774	-2.200	0.031
ENSG00000237402	CAMTA1-IT1	-0.697	-2.242	-2.319	0.023
ENSG00000237499	RP11-356I2.4	-0.610	-2.341	-2.053	0.043
ENSG00000237593	RP11-317B3.2	-0.485	-0.636	-2.423	0.018
ENSG00000237596	RP13-143G15.4	-0.459	1.919	-2.593	0.011
ENSG00000237646	HLCS-IT1	-0.405	-0.569	-2.156	0.034
ENSG00000237731	RNGTTP1	-0.501	-1.331	-2.245	0.027
ENSG00000237832	RP5-974N19.1	-0.616	-0.702	-2.820	0.006
ENSG00000237911	SRP68P3	-0.654	-0.639	-2.332	0.022
ENSG00000238078	LINC01352	0.688	-1.746	2.576	0.012
ENSG00000238117	AP004372.1	-0.717	-1.749	-2.427	0.017
ENSG00000238133	MLK7-AS1	-0.524	-1.556	-2.041	0.044
ENSG00000238363	SNORA13	-0.840	-3.441	-2.033	0.045
ENSG00000239212	RPL6P7	-0.608	0.094	-3.188	0.002
ENSG00000239465	RP11-330L19.2	0.542	-1.683	2.000	0.049

ENSG00000239570	SETP11	-0.375	1.078	-2.199	0.031
ENSG00000239648	MTND1P3	-0.556	-1.217	-2.263	0.026
ENSG00000239650	GUSBP4	-0.443	-0.699	-2.574	0.012
ENSG00000239713	APOBEC3G	0.472	0.071	2.392	0.019
ENSG00000240175	MAGI1-AS1	-0.400	-0.355	-2.012	0.048
ENSG00000240370	U47924.6	0.407	0.267	2.698	0.008
ENSG00000240622	RPL7P15	-0.477	0.111	-2.573	0.012
ENSG00000241156	RN7SL582P	-0.492	-0.776	-2.460	0.016
ENSG00000241217	RN7SL809P	-0.740	-2.090	-2.219	0.029
ENSG00000241229	RN7SL443P	-1.062	-2.149	-3.610	0.001
ENSG00000241462	RP11-579O24.1	-0.761	-0.841	-2.984	0.004
ENSG00000241739	CTD-2290C23.2	-0.548	-1.251	-2.234	0.028
ENSG00000241756	RN7SL83P	-0.594	-1.458	-2.321	0.023
ENSG00000241939	RN7SL517P	-0.530	-1.552	-2.774	0.007
ENSG00000242372	EIF6	0.216	2.731	2.034	0.045
ENSG00000242381	RN7SL741P	-0.568	-1.897	-2.252	0.027
ENSG00000242973	RP11-446F17.3	-0.534	-1.310	-2.223	0.029
ENSG00000243005	RN7SL16P	-0.695	-2.073	-2.361	0.021
ENSG00000243107	AC000120.7	-0.306	0.987	-2.087	0.040
ENSG00000243321	LINC01998	-0.601	-1.221	-2.182	0.032
ENSG00000243352	RN7SL8P	0.678	-3.184	2.010	0.048
ENSG00000243384	RP11-475O23.2	-0.471	-1.229	-2.049	0.044
ENSG00000243424	RN7SL107P	0.347	1.839	2.117	0.037
ENSG00000243516	RPL12P40	-0.725	-0.688	-3.666	0.000
ENSG00000243562	RN7SL838P	0.695	-1.717	2.537	0.013
ENSG00000243738	RN7SL181P	0.571	-1.557	2.134	0.036
ENSG00000243914	RPL5P14	-0.410	1.117	-2.001	0.049
ENSG00000244044	RN7SL735P	-0.433	-0.877	-2.246	0.027
ENSG00000244124	ATP1B3-AS1	-0.388	0.726	-2.087	0.040
ENSG00000244219	GS1-259H13.2	0.653	-2.236	2.457	0.016
ENSG00000244558	KCNK15-AS1	0.337	-0.090	2.063	0.042
ENSG00000244632	RN7SL863P	-0.538	-1.820	-2.005	0.048
ENSG00000245667	RP5-940J5.8	0.418	-1.804	2.038	0.045
ENSG00000245685	AF146191.4	-0.371	0.402	-2.190	0.031
ENSG00000247157	LINC01252	0.232	3.474	2.351	0.021
ENSG00000247271	ZBED5-AS1	-0.262	1.632	-2.270	0.026
ENSG00000247925	RP3-510L9.1	-0.447	-2.323	-2.030	0.046
ENSG00000248245	CTB-49A3.2	-0.432	-0.066	-2.046	0.044
ENSG00000248544	CTB-47B11.3	-0.402	0.772	-2.083	0.040
ENSG00000248624	RP11-5N11.2	-0.495	0.026	-2.182	0.032
ENSG00000248677	LINC02102	-0.529	-0.874	-2.494	0.015
ENSG00000248766	RP11-61K20.1	-0.943	-2.577	-2.278	0.025
ENSG00000248866	USP46-AS1	0.262	1.818	2.558	0.012
ENSG00000249028	RP5-951N9.1	-0.360	0.028	-2.098	0.039
ENSG00000249203	LINC02224	0.556	-1.321	2.105	0.038
ENSG00000249691	RP11-445J14.1	-0.444	-0.820	-2.046	0.044
ENSG00000249743	RP11-60A8.1	1.228	-2.615	2.743	0.007

ENSG00000249780	RP11-352E6.2	1.059	-1.590	2.583	0.012
ENSG00000249798	HSD3BP3	-0.500	-0.683	-2.067	0.042
ENSG00000249848	RP11-212F11.1	-0.497	0.091	-2.214	0.030
ENSG00000250120	PCDHA10	-0.228	3.881	-2.732	0.008
ENSG00000250260	RP11-325L7.2	-0.648	-1.542	-2.669	0.009
ENSG00000250410	RP11-714G18.1	-0.534	-0.269	-3.030	0.003
ENSG00000250479	CHCHD10	0.192	4.062	1.990	0.050
ENSG00000251023	RP11-549J18.1	-0.228	1.306	-2.009	0.048
ENSG00000251226	RP11-469N6.1	0.938	0.915	2.268	0.026
ENSG00000251370	CTD-2201E9.1	-0.386	-0.149	-2.016	0.047
ENSG00000251381	LINC00958	0.724	-0.570	3.110	0.003
ENSG00000251637	RP11-119D9.1	-0.529	-0.282	-2.283	0.025
ENSG00000251664	PCDHA12	-0.245	0.906	-2.229	0.029
ENSG00000251675	CTC-458I2.2	-0.665	-2.394	-2.640	0.010
ENSG00000251982	RN7SKP43	0.968	-2.960	2.588	0.011
ENSG00000252578	RNU6-135P	-0.776	-2.280	-2.859	0.005
ENSG00000252751	RNU6-143P	-0.559	-0.286	-2.517	0.014
ENSG00000253420	RP11-642D21.2	-0.411	0.398	-2.007	0.048
ENSG00000253547	RP11-959F10.5	-0.516	0.141	-1.997	0.049
ENSG00000253636	RP11-531A24.5	-0.365	2.373	-2.155	0.034
ENSG00000253642	RP11-317N12.1	-0.772	-1.771	-2.142	0.035
ENSG00000253773	C8orf37-AS1	0.578	-2.031	2.289	0.025
ENSG00000253805	RP11-1049H7.2	-0.520	-0.561	-2.599	0.011
ENSG00000254034	RP11-662B19.2	-0.387	0.380	-2.078	0.041
ENSG00000254126	CD8BP	0.579	-0.093	2.243	0.028
ENSG00000254317	RP11-473O4.5	-0.426	-0.455	-2.243	0.028
ENSG00000254362	RP11-14I17.3	-0.528	-1.562	-1.993	0.050
ENSG00000254365	CTB-180C19.1	-0.572	-1.435	-2.268	0.026
ENSG00000254427	RP11-430H10.1	0.579	-0.993	2.133	0.036
ENSG00000254484	CTA-797E19.1	-0.423	-1.016	-2.119	0.037
ENSG00000254843	RP11-648O15.1	-0.436	-0.342	-2.143	0.035
ENSG00000254896	OPCML-IT1	-0.347	1.831	-2.052	0.043
ENSG00000254972	RP11-167J8.3	-0.781	-2.159	-3.147	0.002
ENSG00000255167	RP11-670N15.1	-0.416	-0.503	-2.024	0.046
ENSG00000255185	PDXDC2P	-0.564	-0.792	-2.107	0.038
ENSG00000255434	CTD-2530H12.8	-0.360	-0.028	-2.019	0.047
ENSG00000255483	AP000889.2	-0.599	-1.412	-2.097	0.039
ENSG00000256025	CACNA1C-AS4	-0.351	0.976	-2.005	0.048
ENSG00000256137	RP11-174M13.2	-0.503	-0.686	-2.490	0.015
ENSG00000256159	RP11-820K3.4	-0.773	-1.270	-3.262	0.002
ENSG00000256325	RP11-611O2.1	-0.589	-1.493	-2.675	0.009
ENSG00000256377	RP11-1060J15.4	-0.677	-2.175	-2.100	0.039
ENSG00000256458	RP11-363J17.1	-0.462	-1.461	-2.108	0.038
ENSG00000256473	RP11-437F6.1	-0.529	-1.114	-2.171	0.033
ENSG00000256494	RP5-944M2.1	-0.643	-0.828	-2.180	0.032
ENSG00000256533	RP11-212D19.5	-0.355	0.070	-2.118	0.037
ENSG00000256540	RP11-598F7.6	-0.346	0.645	-2.041	0.044

ENSG00000256552	RP11-113C12.4	-0.389	-0.323	-2.099	0.039
ENSG00000256633	RP11-169D4.2	-0.627	-1.877	-2.209	0.030
ENSG00000256937	KRT17P8	0.497	-1.552	2.164	0.033
ENSG00000257185	LINC02293	-0.837	-2.687	-2.808	0.006
ENSG00000257198	FAM205BP	-0.708	-2.022	-1.993	0.050
ENSG00000257220	RP11-136F16.2	-0.518	-0.514	-2.571	0.012
ENSG00000257322	RP11-511B23.2	0.599	-1.276	2.306	0.024
ENSG00000257509	RP11-762I7.4	-0.519	-0.190	-2.758	0.007
ENSG00000257572	RP11-478B9.2	-0.602	0.217	-2.877	0.005
ENSG00000257718	RP11-396F22.1	0.561	-1.080	2.461	0.016
ENSG00000257792	OR5BJ1P	-0.612	-0.675	-2.546	0.013
ENSG00000257875	RP11-263K4.1	-0.377	0.071	-2.266	0.026
ENSG00000258068	RP11-328C8.5	-0.494	-0.948	-2.465	0.016
ENSG00000258336	DUX4L52	0.840	-2.219	2.186	0.032
ENSG00000258443	RP3-414A15.11	-0.358	-0.755	-2.060	0.043
ENSG00000258448	RP11-109N23.5	-0.487	-1.799	-2.017	0.047
ENSG00000258560	CTD-2376I20.1	-0.814	-2.416	-2.355	0.021
ENSG00000258631	RP11-739G5.1	-0.343	-1.142	-1.995	0.049
ENSG00000258766	DIO2-AS1	-0.655	-1.625	-2.520	0.014
ENSG00000258791	LINC00520	-0.571	-1.120	-2.027	0.046
ENSG00000258856	CTD-2325K12.1	-1.038	-1.846	-3.029	0.003
ENSG00000259013	RP11-1017G21.3	-0.653	-1.438	-2.847	0.006
ENSG00000259212	CTD-3065B20.2	-0.446	0.054	-2.714	0.008
ENSG00000259242	AC002306.1	0.538	-2.217	2.015	0.047
ENSG00000259258	RP11-325L12.3	-0.413	-0.009	-2.113	0.038
ENSG00000259356	RP11-90E5.1	-0.604	-1.583	-2.113	0.038
ENSG00000259359	RP11-327J17.2	-0.717	-2.506	-2.227	0.029
ENSG00000259417	LINC01314	-0.256	4.475	-2.119	0.037
ENSG00000259429	UBE2Q2P2	-0.286	2.256	-2.289	0.025
ENSG00000259478	RP11-753A21.1	-0.653	-2.490	-2.231	0.028
ENSG00000259528	RP11-272D12.2	-0.468	-0.610	-2.151	0.034
ENSG00000259591	RP11-554D20.1	-0.415	0.680	-2.089	0.040
ENSG00000259656	RP11-325E5.1	-0.786	-1.868	-2.807	0.006
ENSG00000259683	RP11-182J1.14	-0.679	1.033	-3.366	0.001
ENSG00000260000	RP3-467N11.1	0.291	1.336	2.222	0.029
ENSG00000260019	LINC01992	0.754	-1.204	2.061	0.043
ENSG00000260025	RP11-490M8.1	0.269	1.266	2.153	0.034
ENSG00000260115	CTC-420A11.2	-0.528	-1.537	-2.297	0.024
ENSG00000260185	RP11-432I5.6	-0.536	-2.754	-2.048	0.044
ENSG00000260255	RP11-185O21.1	-0.601	0.004	-2.808	0.006
ENSG00000260338	LINC01570	-0.638	-0.465	-2.360	0.021
ENSG00000260417	CTD-2542L18.1	-0.544	-0.746	-2.011	0.048
ENSG00000260528	FAM157C	-0.813	0.127	-2.118	0.037
ENSG00000260640	KB-1000E4.2	-0.735	-2.106	-2.337	0.022
ENSG00000260698	RP11-439E19.9	-0.589	-1.280	-2.671	0.009
ENSG00000260721	AF067845.1	-0.467	0.856	-2.223	0.029
ENSG00000260796	RP11-1348G14.5	-0.486	-0.695	-2.135	0.036

ENSG00000260806	RP11-872J21.3	0.616	-1.758	2.156	0.034
ENSG00000260927	RP11-459F6.3	-0.557	-1.447	-2.478	0.015
ENSG00000261095	RP11-899L11.1	-0.818	-2.634	-2.208	0.030
ENSG00000261131	RP11-93O14.2	0.680	-2.321	2.314	0.023
ENSG00000261426	AC144833.1	-0.390	2.601	-2.265	0.026
ENSG00000261778	RP5-1173P7.1	-0.581	-1.521	-2.283	0.025
ENSG00000261810	RP11-895K13.2	-0.474	0.218	-2.026	0.046
ENSG00000262075	DKFZP434A062	1.119	-2.147	3.713	0.000
ENSG00000262097	LINC02185	0.527	-1.318	2.016	0.047
ENSG00000262873	CTD-2561B21.11	-0.737	-1.894	-2.713	0.008
ENSG00000263063	RP11-388C12.1	-0.412	-1.001	-2.072	0.041
ENSG00000263300	RP5-1029F21.4	-0.656	-1.832	-2.233	0.028
ENSG00000263485	CTC-304I17.4	-0.440	0.471	-2.409	0.018
ENSG00000263528	IKBKE	0.585	-0.116	2.925	0.004
ENSG00000264529	RP11-556O9.3	-0.381	-0.038	-2.080	0.041
ENSG00000264569	RP13-650J16.1	-0.643	-2.078	-2.070	0.042
ENSG00000264659	CTD-2006K23.2	-0.728	-1.745	-2.315	0.023
ENSG00000264937	AC100830.5	-0.741	-1.560	-2.756	0.007
ENSG00000264954	PRR29-AS1	0.564	-1.035	2.342	0.022
ENSG00000265043	RP11-728E14.3	-0.480	-0.035	-2.116	0.037
ENSG00000265125	RP11-31I22.3	-0.436	1.200	-2.128	0.036
ENSG00000265222	RP11-466A19.8	-0.543	-1.632	-2.098	0.039
ENSG00000265494	RP11-131K5.2	-0.534	-1.333	-2.191	0.031
ENSG00000265689	RP11-118G23.1	-0.534	-0.623	-2.190	0.031
ENSG00000265787	CYP4F35P	-0.590	-1.288	-2.020	0.047
ENSG00000265801	RP11-720N19.2	-0.652	-1.547	-2.496	0.015
ENSG00000266371	RP11-142O6.1	-0.398	0.990	-2.351	0.021
ENSG00000266709	RP11-214O1.2	-0.585	-1.272	-2.283	0.025
ENSG00000267128	RP11-449J21.5	-0.291	1.378	-2.169	0.033
ENSG00000267278	MAP3K14-AS1	-0.343	0.234	-2.548	0.013
ENSG00000267321	LINC02001	0.216	1.089	2.165	0.033
ENSG00000267355	RPL9P29	-0.455	-1.472	-2.003	0.049
ENSG00000267390	RP11-635N19.1	0.513	-0.924	2.655	0.010
ENSG00000267783	CTB-55O6.10	-0.882	-2.982	-2.488	0.015
ENSG00000267787	RP11-35G9.5	-0.284	0.534	-2.081	0.041
ENSG00000267799	MAN1A2P1	-0.390	0.182	-2.263	0.026
ENSG00000267868	RP11-120K24.3	-0.468	-0.587	-2.188	0.032
ENSG00000268006	PTOV1-AS1	-0.306	1.176	-2.384	0.019
ENSG00000268058	BNIP3P40	-0.385	-0.002	-2.209	0.030
ENSG00000268093	AC022154.7	-0.522	-0.880	-2.269	0.026
ENSG00000268894	PLCE1-AS1	-0.436	-0.351	-2.329	0.022
ENSG00000269752	CTD-3149D2.3	-0.687	-2.305	-2.221	0.029
ENSG00000269843	CTC-490E21.10	-0.396	-0.173	-2.373	0.020
ENSG00000269891	ARHGAP19-SLIT1	-0.377	1.049	-2.162	0.034
ENSG00000269959	SPACA6P-AS	-0.487	0.290	-2.154	0.034
ENSG00000269967	RP11-84A19.4	-0.463	-0.540	-2.223	0.029
ENSG00000270001	RP11-218C14.8	-0.739	-2.240	-1.993	0.050

ENSG00000270020	RP11-463O9.9	0.646	-0.309	2.499	0.014
ENSG00000270077	KB-1958F4.1	-0.558	-1.084	-2.059	0.043
ENSG00000270083	RP1-257I20.14	-0.474	-0.883	-2.495	0.015
ENSG00000270130	RP11-214K3.23	-0.319	1.307	-2.071	0.041
ENSG00000270387	RP11-315D13.1	-0.558	-1.512	-2.014	0.047
ENSG00000270540	RP11-785G17.1	-0.680	-1.914	-2.357	0.021
ENSG00000270620	RP1-134O19.3	-0.617	-1.958	-2.335	0.022
ENSG00000270640	RP11-373D23.2	-0.970	-1.866	-2.844	0.006
ENSG00000270806	C17orf50	-0.744	-2.137	-2.302	0.024
ENSG00000270900	NUDT19P4	-0.583	-1.066	-2.596	0.011
ENSG00000271204	RP11-138A9.1	-0.328	3.888	-2.149	0.035
ENSG00000271789	RP5-1112D6.7	-0.308	1.319	-2.271	0.026
ENSG00000271986	RN7SL827P	0.499	-1.751	2.047	0.044
ENSG00000271993	RP11-285J16.1	-0.403	-0.077	-2.214	0.030
ENSG00000272084	RP5-1126H10.2	-0.483	-0.561	-2.647	0.010
ENSG00000272092	RP11-350N15.5	0.500	-1.156	2.059	0.043
ENSG00000272274	LINC00551	-0.894	-1.391	-2.376	0.020
ENSG00000272370	RP11-307L14.1	0.461	-1.298	2.244	0.028
ENSG00000272498	RP11-415F23.3	-0.389	0.371	-2.348	0.021
ENSG00000272529	RP11-415F23.4	-0.422	0.514	-2.391	0.019
ENSG00000272620	AFAP1-AS1	-0.345	1.774	-2.557	0.012
ENSG00000272702	RP11-44N22.3	-0.641	-1.337	-2.421	0.018
ENSG00000272721	RP11-778D9.12	-0.793	-2.562	-2.656	0.009
ENSG00000272744	RP11-367N14.3	-0.341	-0.172	-2.007	0.048
ENSG00000272769	RP11-725P16.2	-0.553	-0.866	-2.037	0.045
ENSG00000272779	LL22NC03-80A10.(	0.201	4.827	2.087	0.040
ENSG00000272844	RP11-484K9.4	-0.704	-1.859	-2.392	0.019
ENSG00000272892	RP11-57G10.8	0.718	-2.248	2.101	0.039
ENSG00000273026	RP11-422P24.10	0.671	-1.947	2.201	0.031
ENSG00000273148	RP5-1068E13.7	0.350	0.929	3.044	0.003
ENSG00000273372	SFTPD-AS1	-0.567	-0.075	-2.676	0.009
ENSG00000273391	RP11-634H22.1	-0.542	-1.655	-2.248	0.027
ENSG00000273474	RP11-295P9.12	-0.544	-1.287	-2.340	0.022
ENSG00000273611	ZNHIT3	0.239	4.257	2.171	0.033
ENSG00000273762	VN1R76P	-0.480	-0.438	-2.439	0.017
ENSG00000274002	RP11-66N24.6	0.428	-0.867	2.028	0.046
ENSG00000274038	RP11-66H6.4	-0.660	-2.291	-2.167	0.033
ENSG00000274210	RNVU1-27	0.548	-0.764	1.992	0.050
ENSG00000274425	AC114271.2	0.626	-2.063	2.266	0.026
ENSG00000274461	RP11-323P17.2	-0.339	0.998	-2.147	0.035
ENSG00000274963	RN7SL600P	-0.521	-1.844	-2.096	0.039
ENSG00000275064	CH17-262O2.1	-0.411	0.567	-1.996	0.049
ENSG00000275720	CTB-75G16.3	-0.739	-2.761	-2.155	0.034
ENSG00000275927	RP11-567P19.1	-0.429	-0.462	-2.095	0.039
ENSG00000276045	ORAI1	0.617	-1.198	2.785	0.007
ENSG00000276375	RP11-339B9.1	-0.929	-2.312	-2.141	0.035
ENSG00000276505	CMB9-94B1.2	-0.625	-2.596	-2.074	0.041

ENSG00000276527	RP11-442J21.2	-0.666	-2.096	-2.227	0.029
ENSG00000276545	PCDHGB9P	-0.312	1.262	-2.018	0.047
ENSG00000276573	RP11-128N14.5	-0.585	-1.093	-3.526	0.001
ENSG00000276980	CTD-3128G10.7	0.746	-0.968	2.608	0.011
ENSG00000277007	RP11-392O17.2	-0.415	1.610	-2.535	0.013
ENSG00000277224	HIST1H2BF	0.336	1.760	2.735	0.008
ENSG00000277795	AC005752.10	-0.425	0.518	-2.698	0.008
ENSG00000278934	CTD-2006M22.2	0.418	-0.824	2.109	0.038
ENSG00000278970	HEIH	0.184	2.306	2.111	0.038
ENSG00000279085	CTA-243E7.4	-0.531	-0.249	-2.137	0.036
ENSG00000279123	RP11-424M24.3	-0.651	-2.325	-2.066	0.042
ENSG00000279267	RP11-395L14.18	0.489	1.856	2.251	0.027
ENSG00000279283	RP11-417L19.5	-0.456	-0.024	-2.114	0.038
ENSG00000279314	RP1-257C22.2	-0.471	-0.313	-2.389	0.019
ENSG00000279317	AC006994.3	0.478	-0.874	2.072	0.041
ENSG00000279324	RP11-93G23.2	-0.484	0.988	-2.437	0.017
ENSG00000279373	RP11-650L12.4	-0.729	-1.979	-2.191	0.031
ENSG00000279539	AC006486.10	0.562	-1.009	2.231	0.028
ENSG00000279543	RP11-1147P1.1	-0.534	0.006	-2.180	0.032
ENSG00000279555	RP11-426D19.1	-0.458	1.614	-2.531	0.013
ENSG00000279584	AC005593.2	-0.937	-2.990	-2.251	0.027
ENSG00000279629	RP11-426J5.1	-0.628	-1.302	-2.543	0.013
ENSG00000279668	CTC-304I17.6	-0.386	3.749	-2.293	0.024
ENSG00000279670	RP11-70L8.5	-0.371	0.703	-2.208	0.030
ENSG00000279685	MAPT-IT1	-0.306	1.759	-2.057	0.043
ENSG00000279862	RP11-423E7.2	-0.549	-0.732	-2.475	0.015
ENSG00000279957	RP11-141B14.2	-0.564	-0.768	-2.187	0.032
ENSG00000280047	CTC-463A16.1	-0.276	2.393	-2.158	0.034
ENSG00000280080	U51561.1	-0.358	0.275	-2.172	0.033
ENSG00000280115	RP11-133M8.3	-0.438	-1.079	-2.014	0.047
ENSG00000280155	RP3-523C21.3	-0.527	-1.688	-2.039	0.045
ENSG00000280205	RP11-504I13.2	-0.588	-0.573	-2.354	0.021
ENSG00000280207	RP11-423H2.4	-0.800	-2.636	-2.384	0.019
ENSG00000280237	MIR4697HG	-0.227	4.925	-2.075	0.041
ENSG00000280321	RP11-1110F20.1	-0.528	-0.745	-2.265	0.026
ENSG00000280350	LINC01726	-0.661	-0.892	-2.275	0.026
ENSG00000280355	RP11-119F19.5	-0.310	2.181	-2.520	0.014
ENSG00000280422	RP11-155D18.13	-0.347	0.055	-2.139	0.035
ENSG00000280425	RP11-446E24.3	-0.736	-2.595	-2.467	0.016
ENSG00000280474	RP11-216B9.8	-0.708	-1.604	-2.510	0.014
ENSG00000280485	RP11-437J2.4	-0.460	0.204	-2.753	0.007
ENSG00000282390	RP11-29A19.2	-0.600	-0.232	-2.796	0.006
ENSG00000282989	KB-1396H2.2	-0.392	1.015	-2.091	0.040
ENSG00000283162	RP11-403F21.4	0.299	1.013	2.086	0.040
ENSG00000283402	RP11-108A14.2	-0.505	-0.842	-2.170	0.033
ENSG00000283405	RP5-872K7.8	-0.723	-2.522	-2.066	0.042
ENSG00000283417	RP11-118G23.3	-0.401	3.006	-2.054	0.043

ENSG00000283575	U6	1.042	-0.642	2.356	0.021
ENSG00000283597	FAM169B	0.789	-2.093	2.404	0.018
ENSG00000283973	#N/A	-0.579	-1.374	-2.217	0.029
ENSG00000284513	#N/A	-0.482	0.166	-2.343	0.022
ENSG00000284600	#N/A	-0.590	0.035	-2.957	0.004
ENSG00000284669	#N/A	-0.431	1.490	-2.246	0.027
ENSG00000284677	#N/A	-0.530	-0.691	-2.402	0.019
ENSG00000285080	#N/A	-0.601	-2.480	-2.419	0.018
ENSG00000285382	#N/A	0.495	-0.690	2.704	0.008
ENSG00000285555	#N/A	-0.375	1.635	-2.046	0.044
ENSG00000285600	#N/A	0.355	0.702	2.893	0.005
ENSG00000285603	#N/A	-0.548	-0.912	-2.386	0.019
ENSG00000285619	#N/A	-0.715	-2.211	-2.220	0.029
ENSG00000285621	#N/A	-0.449	0.114	-2.433	0.017
ENSG00000285781	#N/A	-0.363	0.805	-2.263	0.026
ENSG00000285870	#N/A	-0.683	-1.401	-3.097	0.003

Nac

gene_id	gene_name	logFC	AveExpr	t	P.Value
ENSG00000002016	RAD52	-0.282	4.055	-2.316	0.023
ENSG00000005238	FAM214B	0.234	3.617	2.332	0.023
ENSG00000005486	RHBDD2	0.530	5.103	3.002	0.004
ENSG00000006210	CX3CL1	0.396	5.100	2.234	0.029
ENSG00000007402	CACNA2D2	0.370	4.440	2.149	0.035
ENSG00000008283	CYB561	0.371	4.600	2.446	0.017
ENSG00000011478	QPCTL	0.292	1.407	2.141	0.036
ENSG00000012232	EXTL3	0.191	6.094	2.236	0.029
ENSG00000012822	CALCOCO1	0.208	7.024	2.206	0.031
ENSG00000014216	CAPN1	0.353	4.068	2.996	0.004
ENSG00000014257	ACPP	1.414	-0.519	2.638	0.010
ENSG00000019186	CYP24A1	1.025	0.410	2.067	0.042
ENSG00000027847	B4GALT7	0.279	2.164	2.202	0.031
ENSG00000028277	POU2F2	0.396	2.038	2.058	0.043
ENSG00000030582	GRN	0.331	2.650	2.294	0.025
ENSG00000031823	RANBP3	0.139	4.495	2.124	0.037
ENSG00000037897	METTL1	0.367	0.797	2.385	0.020
ENSG00000043143	JADE2	0.186	5.862	2.196	0.031
ENSG00000048028	USP28	-0.172	4.506	-2.371	0.020
ENSG00000054356	PTPRN	0.478	6.415	2.570	0.012
ENSG00000054392	HHAT	-0.281	2.816	-2.259	0.027
ENSG00000055118	KCNH2	0.359	3.298	2.099	0.039
ENSG00000059122	FLYWCH1	0.360	4.310	2.364	0.021
ENSG00000062485	CS	0.199	5.912	2.713	0.008
ENSG00000063761	ADCK1	0.253	2.572	2.080	0.041
ENSG00000064218	DMRT3	-0.982	-5.680	-2.324	0.023
ENSG00000065243	PKN2	-0.236	6.570	-2.943	0.004
ENSG00000065491	TBC1D22B	0.198	3.503	2.386	0.020
ENSG00000065802	ASB1	0.146	6.051	2.108	0.039
ENSG00000066735	KIF26A	0.629	-0.171	2.570	0.012
ENSG00000067221	STOML1	0.343	3.396	3.689	0.000
ENSG00000068885	IFT80	-0.321	5.110	-2.101	0.039
ENSG00000068971	PPP2R5B	0.336	5.134	3.188	0.002
ENSG00000069943	PIGB	0.170	3.473	2.004	0.049
ENSG00000070413	DGCR2	0.199	5.517	2.066	0.042
ENSG00000071205	ARHGAP10	-0.357	4.755	-2.024	0.047
ENSG00000072832	CRMP1	0.234	5.646	2.357	0.021
ENSG00000073756	PTGS2	-1.231	1.314	-3.589	0.001
ENSG00000074071	MRPS34	0.301	3.506	2.621	0.011
ENSG00000074527	NTN4	-0.284	4.064	-2.105	0.039
ENSG00000075340	ADD2	0.362	8.132	2.068	0.042
ENSG00000076944	STXBP2	0.618	-0.517	2.044	0.045
ENSG00000077312	SNRPA	0.242	2.988	2.154	0.035
ENSG00000077463	SIRT6	0.548	0.459	3.197	0.002
ENSG00000078725	BRINP1	0.289	6.333	2.361	0.021

ENSG00000082556	OPRK1	0.765	5.230	2.373	0.020
ENSG00000084070	SMAP2	0.255	6.371	2.379	0.020
ENSG00000084234	APLP2	0.188	8.859	2.010	0.048
ENSG00000085644	ZNF213	0.329	2.240	2.060	0.043
ENSG00000085840	ORC1	0.713	-1.613	2.369	0.021
ENSG00000087085	ACHE	0.622	3.718	2.556	0.013
ENSG00000088882	CPXM1	0.765	-2.222	2.135	0.036
ENSG00000089486	CDIP1	0.202	5.203	2.130	0.037
ENSG00000089597	GANAB	0.191	5.800	2.542	0.013
ENSG00000089693	MLF2	0.344	5.583	2.393	0.019
ENSG00000090316	MAEA	0.200	4.883	2.131	0.037
ENSG00000090432	MUL1	0.241	3.551	2.392	0.019
ENSG00000090447	TFAP4	0.226	2.327	2.078	0.041
ENSG00000090470	PDCD7	-0.179	4.397	-2.268	0.026
ENSG00000090924	PLEKHG2	0.533	0.470	2.059	0.043
ENSG00000091073	DTX2	0.492	0.903	2.303	0.024
ENSG00000091262	ABCC6	-0.434	-0.347	-2.019	0.047
ENSG00000091490	SEL1L3	-0.367	4.605	-2.632	0.010
ENSG00000091592	NLRP1	0.386	6.012	2.144	0.035
ENSG00000092096	SLC22A17	0.487	6.537	2.554	0.013
ENSG00000095739	BAMBI	-0.443	2.587	-2.206	0.031
ENSG00000097021	ACOT7	0.373	4.223	2.347	0.022
ENSG00000099284	H2AFY2	0.218	2.799	2.436	0.017
ENSG00000099341	PSMD8	0.268	4.868	2.293	0.025
ENSG00000099817	POLR2E	0.223	4.129	2.293	0.025
ENSG00000099994	SUSD2	1.019	-0.969	2.316	0.023
ENSG00000099998	GGT5	0.607	0.943	2.105	0.039
ENSG00000100056	DGCR14	0.215	2.870	2.173	0.033
ENSG00000100280	AP1B1	0.184	5.772	2.169	0.033
ENSG00000100429	HDAC10	0.442	-0.499	2.162	0.034
ENSG00000100867	DHRS2	0.949	-2.254	2.348	0.022
ENSG00000100884	CPNE6	0.265	4.499	2.019	0.047
ENSG00000100949	RABGGTA	0.404	2.392	3.649	0.000
ENSG00000100983	GSS	0.218	4.347	2.527	0.014
ENSG00000101152	DNAJC5	0.246	7.433	2.301	0.024
ENSG00000101180	HRH3	0.825	2.955	3.025	0.003
ENSG00000101188	NTSR1	1.051	-1.815	2.396	0.019
ENSG00000101210	EEF1A2	0.386	6.532	2.228	0.029
ENSG00000101298	SNPH	0.293	5.784	2.135	0.036
ENSG00000101337	TM9SF4	0.134	5.445	2.132	0.036
ENSG00000101363	MANBAL	0.269	3.156	2.799	0.007
ENSG00000101489	CELF4	0.469	6.301	2.572	0.012
ENSG00000103066	PLA2G15	0.240	1.691	2.182	0.032
ENSG00000103145	HCFC1R1	0.333	3.694	2.232	0.029
ENSG00000103152	MPG	0.393	0.871	2.110	0.038
ENSG00000103227	LMF1	0.361	2.388	2.186	0.032
ENSG00000103241	FOXF1	-0.840	0.173	-2.482	0.015

ENSG00000103319	EEF2K	0.265	4.827	2.610	0.011
ENSG00000103502	CDIPT	0.371	4.185	3.159	0.002
ENSG00000103522	IL21R	1.188	-0.942	2.872	0.005
ENSG00000103855	CD276	0.280	2.562	1.998	0.049
ENSG00000104228	TRIM35	0.179	4.601	2.030	0.046
ENSG00000104853	CLPTM1	0.272	5.923	2.240	0.028
ENSG00000104884	ERCC2	0.319	2.963	3.289	0.002
ENSG00000105197	TIMM50	0.181	4.433	2.228	0.029
ENSG00000105223	PLD3	0.422	6.145	2.622	0.011
ENSG00000105258	POLR2I	0.394	2.175	2.172	0.033
ENSG00000105298	CACTIN	0.249	2.694	2.400	0.019
ENSG00000105364	MRPL4	0.316	1.775	2.245	0.028
ENSG00000105520	PLPPR2	0.468	4.016	2.420	0.018
ENSG00000105568	PPP2R1A	0.246	6.428	2.272	0.026
ENSG00000105676	ARMC6	0.249	2.521	2.345	0.022
ENSG00000105723	GSK3A	0.319	3.427	2.067	0.042
ENSG00000105732	ZNF574	0.379	2.266	2.325	0.023
ENSG00000105880	DLX5	0.627	1.766	2.150	0.035
ENSG00000106330	MOSPD3	0.531	0.407	3.312	0.001
ENSG00000106348	IMPDH1	0.238	3.091	2.039	0.045
ENSG00000106351	AGFG2	0.279	4.593	2.110	0.038
ENSG00000106948	AKNA	0.300	4.292	2.598	0.011
ENSG00000107140	TESK1	0.243	3.332	2.225	0.029
ENSG00000107338	SHB	0.317	1.746	2.092	0.040
ENSG00000107742	SPOCK2	0.262	7.965	2.135	0.036
ENSG00000108828	VAT1	0.475	4.587	3.272	0.002
ENSG00000110002	VWA5A	0.419	5.345	2.811	0.006
ENSG00000110237	ARHGEF17	0.292	5.919	2.157	0.034
ENSG00000110693	SOX6	-0.262	6.229	-2.678	0.009
ENSG00000110721	CHKA	-0.191	5.448	-2.469	0.016
ENSG00000110841	PPFIBP1	-0.395	5.797	-2.078	0.041
ENSG00000110852	CLEC2B	-0.559	1.388	-2.150	0.035
ENSG00000110888	CAPRIN2	0.262	7.060	2.265	0.027
ENSG00000111012	CYP27B1	0.680	-1.174	2.022	0.047
ENSG00000111859	NEDD9	-0.422	4.352	-2.446	0.017
ENSG00000112273	HDGFL1	-1.089	-5.502	-2.005	0.049
ENSG00000112514	CUTA	0.327	4.496	2.205	0.031
ENSG00000112576	CCND3	0.225	3.780	2.267	0.026
ENSG00000112592	TBP	0.159	3.549	2.306	0.024
ENSG00000112640	PPP2R5D	0.275	5.195	2.177	0.033
ENSG00000112651	MRPL2	0.264	2.837	2.922	0.005
ENSG00000112715	VEGFA	-0.472	5.194	-2.218	0.030
ENSG00000112763	BTN2A1	0.195	4.702	2.108	0.039
ENSG00000113580	NR3C1	-0.200	7.101	-2.022	0.047
ENSG00000113739	STC2	-0.971	-1.286	-2.284	0.025
ENSG00000113758	DBN1	0.387	5.749	2.727	0.008
ENSG00000114383	TUSC2	0.307	3.859	2.536	0.013

ENSG00000115207	GTF3C2	0.123	4.082	2.003	0.049
ENSG00000115474	KCNJ13	-0.463	2.429	-2.992	0.004
ENSG00000115827	DCAF17	-0.184	5.401	-2.124	0.037
ENSG00000116044	NFE2L2	-0.266	5.795	-2.396	0.019
ENSG00000116514	RNF19B	0.183	4.408	2.120	0.037
ENSG00000116521	SCAMP3	0.216	3.455	2.013	0.048
ENSG00000116649	SRM	0.426	2.632	2.377	0.020
ENSG00000116663	FBXO6	0.369	1.139	2.564	0.012
ENSG00000116711	PLA2G4A	-0.637	1.926	-2.383	0.020
ENSG00000117472	TSPAN1	-0.590	-1.419	-2.108	0.039
ENSG00000117602	RCAN3	0.376	4.216	2.140	0.036
ENSG00000117682	DHDDS	0.147	4.816	2.139	0.036
ENSG00000118004	COLEC11	-1.007	-1.109	-2.257	0.027
ENSG00000118579	MED28	-0.097	6.232	-2.172	0.033
ENSG00000119042	SATB2	-0.517	5.154	-2.095	0.040
ENSG00000119333	WDR34	0.330	1.629	2.266	0.026
ENSG00000119431	HDHD3	0.295	2.285	2.190	0.032
ENSG00000119508	NR4A3	-0.837	4.032	-2.420	0.018
ENSG00000119574	ZBTB45	0.441	1.376	2.226	0.029
ENSG00000119777	TMEM214	0.285	3.152	2.107	0.039
ENSG00000119899	SLC17A5	0.238	4.421	2.523	0.014
ENSG00000120057	SFRP5	0.749	-0.469	2.350	0.022
ENSG00000120341	SEC16B	-1.063	-1.893	-2.548	0.013
ENSG00000121060	TRIM25	0.216	4.480	2.057	0.043
ENSG00000121749	TBC1D15	-0.168	5.883	-2.160	0.034
ENSG00000122008	POLK	-0.181	6.339	-2.082	0.041
ENSG00000122140	MRPS2	0.492	1.807	3.198	0.002
ENSG00000122386	ZNF205	0.369	1.531	2.266	0.027
ENSG00000122884	P4HA1	-0.324	5.554	-2.302	0.024
ENSG00000123989	CHPF	0.347	3.158	2.031	0.046
ENSG00000123999	INHA	0.613	0.021	2.294	0.025
ENSG00000125510	OPRL1	0.585	3.123	2.985	0.004
ENSG00000125651	GTF2F1	0.252	5.201	2.780	0.007
ENSG00000125652	ALKBH7	0.517	1.572	2.156	0.034
ENSG00000125841	NRSN2	0.529	4.693	2.926	0.005
ENSG00000125843	AP5S1	0.198	3.128	2.133	0.036
ENSG00000125871	MGME1	-0.303	2.553	-2.217	0.030
ENSG00000125895	TMEM74B	0.471	2.650	2.325	0.023
ENSG00000125898	FAM110A	0.839	-0.402	3.458	0.001
ENSG00000125912	NCLN	0.317	2.580	2.342	0.022
ENSG00000125966	MMP24	0.264	3.707	2.554	0.013
ENSG00000125971	DYNLRB1	0.322	6.237	2.288	0.025
ENSG00000126106	TMEM53	0.289	1.585	2.299	0.024
ENSG00000126247	CAPNS1	0.295	5.330	2.284	0.025
ENSG00000126602	TRAP1	0.216	4.670	2.314	0.024
ENSG00000127220	ABHD8	0.404	2.697	2.198	0.031
ENSG00000127483	HP1BP3	-0.104	7.801	-2.148	0.035

ENSG00000127561	SYNGR3	0.437	3.384	2.002	0.049
ENSG00000127837	AAMP	0.217	4.900	2.403	0.019
ENSG00000127838	PNKD	0.342	4.552	2.158	0.034
ENSG00000127952	STYXL1	-0.387	3.010	-2.828	0.006
ENSG00000128185	DGCR6L	0.290	2.419	2.093	0.040
ENSG00000128422	KRT17	-1.610	-4.230	-2.178	0.033
ENSG00000128482	RNF112	0.325	4.806	2.105	0.039
ENSG00000129038	LOXL1	0.460	0.375	2.374	0.020
ENSG00000129355	CDKN2D	0.288	4.061	2.038	0.045
ENSG00000129951	PLPPR3	0.568	2.844	2.046	0.044
ENSG00000129990	SYT5	0.535	5.156	2.235	0.029
ENSG00000130311	DDA1	0.240	4.136	2.250	0.028
ENSG00000130600	H19	0.811	-1.115	2.005	0.049
ENSG00000130640	TUBGCP2	0.189	5.156	2.435	0.017
ENSG00000130643	CALY	0.627	5.123	2.762	0.007
ENSG00000130813	C19orf66	0.296	2.820	2.885	0.005
ENSG00000131067	GGT7	0.289	3.681	2.054	0.044
ENSG00000131462	TUBG1	0.284	3.264	2.616	0.011
ENSG00000131652	THOC6	0.378	0.585	2.127	0.037
ENSG00000131773	KHDRBS3	-0.387	5.223	-2.125	0.037
ENSG00000132153	DHX30	0.151	5.833	2.045	0.045
ENSG00000132155	RAF1	-0.102	5.748	-2.055	0.044
ENSG00000132376	INPP5K	0.185	3.673	2.083	0.041
ENSG00000132471	WBP2	0.261	5.664	2.102	0.039
ENSG00000132507	EIF5A	0.254	4.149	2.147	0.035
ENSG00000132549	VPS13B	-0.093	7.560	-2.081	0.041
ENSG00000132589	FLOT2	0.263	5.109	2.555	0.013
ENSG00000132879	FBXO44	0.291	4.976	2.197	0.031
ENSG00000132970	WASF3	-0.156	6.911	-2.115	0.038
ENSG00000133027	PEMT	0.364	0.823	2.115	0.038
ENSG00000133597	ADCK2	0.246	2.923	2.257	0.027
ENSG00000133687	TMTC1	0.522	8.377	2.437	0.017
ENSG00000133710	SPINK5	-0.334	0.711	-2.267	0.026
ENSG00000134330	IAH1	0.163	4.375	2.452	0.017
ENSG00000134470	IL15RA	0.818	-0.773	2.493	0.015
ENSG00000134480	CCNH	-0.149	6.189	-2.197	0.031
ENSG00000134575	ACP2	0.216	3.322	2.102	0.039
ENSG00000134748	PRPF38A	-0.186	5.772	-2.079	0.041
ENSG00000135048	TMEM2	-0.415	4.967	-2.094	0.040
ENSG00000135503	ACVR1B	0.199	5.120	2.053	0.044
ENSG00000135723	FHOD1	0.310	2.540	2.152	0.035
ENSG00000135740	SLC9A5	0.435	2.881	2.204	0.031
ENSG00000135953	MFSD9	0.290	2.626	2.372	0.020
ENSG00000136158	SPRY2	-0.273	4.558	-2.270	0.026
ENSG00000136352	NKX2-1	0.886	0.967	2.016	0.048
ENSG00000136371	MTHFS	0.431	0.108	2.509	0.014
ENSG00000136527	TRA2B	-0.199	6.818	-2.016	0.048

ENSG00000136542	GALNT5	-1.363	-3.199	-2.035	0.046
ENSG00000137216	TMEM63B	0.212	4.711	2.040	0.045
ENSG00000137273	FOXF2	-0.553	0.128	-2.235	0.029
ENSG00000137309	HMGA1	0.422	3.177	2.578	0.012
ENSG00000137747	TMPRSS13	0.890	-2.397	2.068	0.042
ENSG00000137871	ZNF280D	-0.180	5.888	-2.040	0.045
ENSG00000138031	ADCY3	0.367	5.646	2.148	0.035
ENSG00000138286	FAM149B1	-0.120	5.319	-2.112	0.038
ENSG00000138376	BARD1	-0.383	4.201	-2.065	0.043
ENSG00000138468	SENP7	-0.204	6.209	-2.174	0.033
ENSG00000138771	SHROOM3	-0.398	3.578	-2.096	0.040
ENSG00000139178	C1RL	0.493	2.658	2.766	0.007
ENSG00000139410	SDSL	-0.683	-0.812	-2.091	0.040
ENSG00000140009	ESR2	0.303	1.456	2.383	0.020
ENSG00000140323	DISP2	0.429	4.914	2.286	0.025
ENSG00000140876	NUDT7	0.312	0.834	2.078	0.041
ENSG00000141026	MED9	0.184	4.355	2.007	0.049
ENSG00000141582	CBX4	0.217	4.042	2.293	0.025
ENSG00000141741	MIEN1	0.269	2.380	2.239	0.028
ENSG00000141968	VAV1	0.455	2.135	2.173	0.033
ENSG00000142046	TMEM91	0.350	2.794	2.110	0.038
ENSG00000142178	SIK1	-0.996	-0.385	-2.064	0.043
ENSG00000142453	CARM1	0.313	3.436	2.323	0.023
ENSG00000142657	PGD	0.239	4.961	2.075	0.042
ENSG00000142669	SH3BGRL3	0.354	2.718	2.285	0.025
ENSG00000142871	CYR61	-0.870	2.428	-2.587	0.012
ENSG00000143320	CRABP2	0.585	1.423	2.467	0.016
ENSG00000143552	NUP210L	-0.673	2.168	-2.717	0.008
ENSG00000143590	EFNA3	0.517	2.678	2.341	0.022
ENSG00000143627	PKLR	1.284	-3.026	2.585	0.012
ENSG00000143630	HCN3	0.509	1.475	2.562	0.013
ENSG00000143761	ARF1	0.219	6.873	2.385	0.020
ENSG00000143845	ETNK2	0.428	2.217	3.040	0.003
ENSG00000143921	ABCG8	0.459	0.710	2.053	0.044
ENSG00000143952	VPS54	-0.189	5.659	-2.280	0.026
ENSG00000144199	FAHD2B	0.216	3.440	2.059	0.043
ENSG00000144583	MARCHF4	0.582	3.392	2.126	0.037
ENSG00000144647	POMGNT2	0.296	4.143	2.653	0.010
ENSG00000144659	SLC25A38	0.215	4.098	2.245	0.028
ENSG00000144730	IL17RD	-0.263	5.057	-2.776	0.007
ENSG00000144824	PHLDB2	-0.479	3.329	-2.092	0.040
ENSG00000145555	MYO10	-0.248	7.164	-2.092	0.040
ENSG00000145675	PIK3R1	-0.218	8.355	-2.228	0.029
ENSG00000145715	RASA1	-0.136	6.542	-2.186	0.032
ENSG00000145934	TENM2	0.365	6.601	2.149	0.035
ENSG00000147526	TACC1	-0.166	8.397	-2.489	0.015
ENSG00000147813	NAPRT	0.639	-0.038	1.994	0.050

ENSG00000147862	NFIB	-0.273	7.367	-2.614	0.011
ENSG00000147955	SIGMAR1	0.257	3.502	2.658	0.010
ENSG00000148215	OR5C1	1.172	-2.272	2.769	0.007
ENSG00000148331	ASB6	0.209	3.745	2.092	0.040
ENSG00000149179	C11orf49	0.139	4.403	2.103	0.039
ENSG00000149541	B3GAT3	0.257	2.664	2.106	0.039
ENSG00000149582	TMEM25	0.298	5.010	2.285	0.025
ENSG00000149599	DUSP15	0.383	0.501	2.260	0.027
ENSG00000149658	YTHDF1	0.150	5.129	2.047	0.044
ENSG00000149735	GPHA2	1.040	-2.129	2.783	0.007
ENSG00000149781	FERMT3	0.513	0.587	2.105	0.039
ENSG00000149930	TAOK2	0.187	6.183	2.306	0.024
ENSG00000150961	SEC24D	-0.397	3.089	-2.018	0.047
ENSG00000151718	WWC2	-0.232	5.588	-2.516	0.014
ENSG00000151812	SLC35F4	0.476	1.752	2.488	0.015
ENSG00000152348	ATG10	-0.238	3.270	-2.030	0.046
ENSG00000152520	PAN3	-0.156	5.878	-2.367	0.021
ENSG00000153132	CLGN	0.476	3.373	2.033	0.046
ENSG00000153234	NR4A2	-1.726	1.359	-3.415	0.001
ENSG00000154096	THY1	0.498	7.395	2.224	0.029
ENSG00000154734	ADAMTS1	-0.672	4.318	-2.046	0.044
ENSG00000154898	CCDC144CP	-0.341	5.634	-2.089	0.040
ENSG00000155016	CYP2U1	-0.214	4.928	-2.296	0.025
ENSG00000155093	PTPRN2	0.290	7.060	2.090	0.040
ENSG00000155903	RASA2	-0.154	5.687	-2.098	0.039
ENSG00000156427	FGF18	-1.215	-3.786	-2.312	0.024
ENSG00000156471	PTDSS1	0.325	6.125	2.275	0.026
ENSG00000156587	UBE2L6	0.307	4.110	2.272	0.026
ENSG00000156689	GLYATL2	-0.725	-1.098	-2.009	0.048
ENSG00000156869	FRRS1	-0.549	-0.175	-2.114	0.038
ENSG00000156959	LHFPL4	0.344	4.065	2.524	0.014
ENSG00000157093	LYZL4	-1.251	-3.714	-2.372	0.020
ENSG00000157107	FCHO2	-0.189	5.553	-2.397	0.019
ENSG00000157557	ETS2	-0.355	5.497	-2.364	0.021
ENSG00000157895	C12orf43	0.212	3.957	2.504	0.015
ENSG00000157911	PEX10	0.204	2.557	2.117	0.038
ENSG00000158483	FAM86C1	0.314	1.248	2.487	0.015
ENSG00000158683	PKD1L1	0.331	2.892	2.121	0.037
ENSG00000158717	RNF166	0.351	2.231	2.846	0.006
ENSG00000158828	PINK1	0.233	5.376	2.125	0.037
ENSG00000158850	B4GALT3	0.295	3.633	3.241	0.002
ENSG00000159259	CHAF1B	0.301	2.578	2.408	0.019
ENSG00000159363	ATP13A2	0.318	4.750	2.006	0.049
ENSG00000159720	ATP6V0D1	0.258	5.016	2.221	0.030
ENSG00000159753	CARMIL2	0.396	2.598	2.091	0.040
ENSG00000159761	C16orf86	0.556	0.284	3.224	0.002
ENSG00000160113	NR2F6	0.392	1.889	2.525	0.014

ENSG00000160190	SLC37A1	0.241	3.304	2.462	0.016
ENSG00000160224	AIRE	0.736	-1.251	2.033	0.046
ENSG00000160446	ZDHHC12	0.543	-0.412	3.105	0.003
ENSG00000160447	PKN3	0.666	-0.925	2.291	0.025
ENSG00000160703	NLRX1	0.327	1.591	2.139	0.036
ENSG00000160710	ADAR	0.184	8.409	2.471	0.016
ENSG00000160716	CHRNB2	0.340	5.081	2.575	0.012
ENSG00000160741	CRTC2	0.324	2.645	2.181	0.032
ENSG00000160932	LY6E	0.358	3.823	2.039	0.045
ENSG00000160949	TONSL	0.425	0.212	2.326	0.023
ENSG00000160972	PPP1R16A	0.370	1.650	2.010	0.048
ENSG00000161048	NAPEPLD	-0.215	6.362	-2.207	0.031
ENSG00000161180	CCDC116	-0.856	-2.462	-2.128	0.037
ENSG00000161647	MPP3	0.322	2.187	2.249	0.028
ENSG00000161956	SENP3	0.321	2.173	2.438	0.017
ENSG00000161980	POLR3K	0.242	2.645	2.060	0.043
ENSG00000162066	AMDHD2	0.530	0.675	2.021	0.047
ENSG00000162069	BICDL2	1.386	-3.928	2.561	0.013
ENSG00000162139	NEU3	0.217	5.462	2.009	0.048
ENSG00000162426	SLC45A1	0.470	2.174	2.558	0.013
ENSG00000162526	TSSK3	0.791	-0.763	2.828	0.006
ENSG00000162878	PKDCC	-0.842	0.278	-2.661	0.010
ENSG00000163082	SGPP2	0.367	4.278	2.239	0.028
ENSG00000163171	CDC42EP3	0.365	4.128	1.995	0.050
ENSG00000163249	CCNYL1	-0.162	4.156	-2.019	0.047
ENSG00000163749	CCDC158	-0.528	1.771	-2.099	0.039
ENSG00000163795	ZNF513	0.313	1.930	2.451	0.017
ENSG00000163808	KIF15	-0.252	1.969	-2.273	0.026
ENSG00000163993	S100P	-0.639	-1.832	-2.077	0.041
ENSG00000164054	SHISA5	0.283	4.376	2.868	0.005
ENSG00000164620	RELL2	0.431	1.478	2.244	0.028
ENSG00000164885	CDK5	0.334	3.869	2.143	0.035
ENSG00000165006	UBAP1	0.159	5.289	2.161	0.034
ENSG00000165097	KDM1B	-0.174	5.264	-2.145	0.035
ENSG00000165548	TMEM63C	0.360	3.987	2.506	0.014
ENSG00000165644	COMTD1	0.549	-1.090	2.186	0.032
ENSG00000165879	FRAT1	0.448	1.035	2.862	0.006
ENSG00000165886	UBTD1	0.484	0.078	2.018	0.047
ENSG00000165899	OTOGL	-0.726	2.496	-3.096	0.003
ENSG00000166278	C2	0.573	-0.239	2.579	0.012
ENSG00000166323	C11orf65	-0.274	2.761	-2.190	0.032
ENSG00000166448	TMEM130	0.483	7.033	2.447	0.017
ENSG00000166484	MAPK7	0.350	1.575	2.294	0.025
ENSG00000166741	NNMT	-0.949	-2.186	-2.206	0.031
ENSG00000166845	C18orf54	-0.455	2.843	-2.329	0.023
ENSG00000167037	SGSM1	0.337	5.840	2.258	0.027
ENSG00000167100	SAMD14	0.382	3.019	2.088	0.040

ENSG00000167173	C15orf39	0.553	1.032	2.387	0.020
ENSG00000167555	ZNF528	-0.189	6.094	-2.390	0.020
ENSG00000167562	ZNF701	-0.204	3.801	-2.301	0.024
ENSG00000167770	OTUB1	0.313	4.306	2.439	0.017
ENSG00000167778	SPRYD3	0.315	6.460	2.175	0.033
ENSG00000168032	ENTPD3	0.501	5.237	2.016	0.048
ENSG00000168062	BATF2	0.913	-0.591	2.567	0.012
ENSG00000168124	OR1F1	0.751	-0.519	2.354	0.021
ENSG00000168158	OR2C1	0.651	-0.834	2.378	0.020
ENSG00000168298	HIST1H1E	0.338	5.263	2.490	0.015
ENSG00000168348	INSM2	0.650	0.540	2.739	0.008
ENSG00000168461	RAB31	-0.266	7.251	-2.373	0.020
ENSG00000168477	TNXB	0.404	3.068	1.998	0.050
ENSG00000168522	FNTA	-0.201	5.611	-2.342	0.022
ENSG00000168569	TMEM223	0.250	2.231	2.323	0.023
ENSG00000168824	NSG1	0.608	6.309	2.509	0.014
ENSG00000169006	NTSR2	0.360	4.814	2.131	0.037
ENSG00000169116	PARM1	0.407	7.169	2.116	0.038
ENSG00000169180	XPO6	0.115	6.248	2.126	0.037
ENSG00000169692	AGPAT2	0.480	0.300	2.317	0.023
ENSG00000170266	GLB1	0.172	4.040	2.259	0.027
ENSG00000170419	VSTM2A	0.556	6.787	2.116	0.038
ENSG00000170445	HARS	0.159	6.216	2.110	0.038
ENSG00000170448	NFXL1	-0.206	4.432	-3.109	0.003
ENSG00000170473	PYM1	0.227	2.926	2.236	0.029
ENSG00000171160	MORN4	0.266	4.472	2.602	0.011
ENSG00000171307	ZDHHC16	0.144	4.055	2.183	0.032
ENSG00000171448	ZBTB26	-0.178	3.865	-2.145	0.035
ENSG00000171462	DLK2	0.447	0.493	2.123	0.037
ENSG00000171552	BCL2L1	0.319	4.310	3.265	0.002
ENSG00000171703	TCEA2	0.263	3.912	2.634	0.010
ENSG00000171724	VAT1L	0.730	7.737	2.155	0.035
ENSG00000171766	GATM	-0.292	7.107	-2.071	0.042
ENSG00000171786	NHLH1	0.772	0.092	2.600	0.011
ENSG00000171944	OR52A5	-1.362	-5.406	-2.542	0.013
ENSG00000171951	SCG2	0.794	9.408	2.440	0.017
ENSG00000172247	C1QTNF4	0.903	2.097	2.086	0.041
ENSG00000172339	ALG14	0.196	4.300	2.099	0.039
ENSG00000172354	GNB2	0.359	4.152	2.217	0.030
ENSG00000172382	PRSS27	0.679	-0.440	3.278	0.002
ENSG00000172757	CFL1	0.177	6.711	2.057	0.043
ENSG00000172782	FADS6	0.444	0.897	2.473	0.016
ENSG00000172986	GXYLT2	-0.328	3.588	-2.636	0.010
ENSG00000172992	DCAKD	0.187	4.959	2.062	0.043
ENSG00000173456	RNF26	0.432	1.836	2.561	0.013
ENSG00000173465	SSSCA1	0.364	2.081	2.156	0.034
ENSG00000173482	PTPRM	0.274	7.530	2.121	0.037

ENSG00000173581	CCDC106	0.339	3.229	2.124	0.037
ENSG00000173825	TIGD3	0.511	-0.129	2.094	0.040
ENSG00000173826	KCNH6	0.743	-1.296	2.021	0.047
ENSG00000173950	XXYLT1	0.181	3.176	2.096	0.040
ENSG00000174236	REP15	-1.138	-2.922	-2.925	0.005
ENSG00000174332	GLIS1	0.641	0.577	2.270	0.026
ENSG00000174417	TRHR	0.972	-0.594	2.062	0.043
ENSG00000174516	PELI3	0.373	3.756	2.769	0.007
ENSG00000174527	MYO1H	0.542	-1.292	2.137	0.036
ENSG00000174871	CNIH2	0.397	2.647	2.060	0.043
ENSG00000174886	NDUFA11	0.493	0.989	2.995	0.004
ENSG00000174899	PQLC2L	0.728	0.247	2.347	0.022
ENSG00000174903	RAB1B	0.344	3.662	2.735	0.008
ENSG00000174912	METTL15P1	-0.453	1.249	-2.037	0.045
ENSG00000174938	SEZ6L2	0.432	5.057	2.196	0.031
ENSG00000174996	KLC2	0.296	4.348	2.285	0.025
ENSG00000175229	GAL3ST3	0.527	2.143	2.220	0.030
ENSG00000175283	DOLK	0.309	2.831	2.321	0.023
ENSG00000175416	CLTB	0.270	4.918	2.618	0.011
ENSG00000175520	UBQLN3	1.035	-2.565	2.031	0.046
ENSG00000175768	TOMM5	0.246	2.940	2.007	0.049
ENSG00000175806	MSRA	-0.203	4.559	-2.056	0.043
ENSG00000176095	IP6K1	0.204	5.738	2.067	0.042
ENSG00000176105	YES1	-0.295	5.853	-2.005	0.049
ENSG00000176124	DLEU1	-0.316	3.017	-2.134	0.036
ENSG00000176125	UFSP1	0.472	-1.202	2.053	0.044
ENSG00000176340	COX8A	0.337	1.934	2.333	0.022
ENSG00000176387	HSD11B2	0.927	-0.683	2.282	0.025
ENSG00000176723	ZNF843	0.273	1.508	2.135	0.036
ENSG00000176887	SOX11	0.401	4.975	2.232	0.029
ENSG00000176907	C8orf4	-0.792	1.589	-2.391	0.019
ENSG00000176946	THAP4	0.276	4.096	2.185	0.032
ENSG00000176978	DPP7	0.279	4.128	2.015	0.048
ENSG00000177042	TMEM80	0.324	2.299	2.413	0.018
ENSG00000177076	ACER2	-0.394	1.858	-2.051	0.044
ENSG00000177106	EPS8L2	0.483	1.630	2.078	0.041
ENSG00000177275	OR2AJ1	0.910	-1.638	2.208	0.030
ENSG00000177352	CCDC71	0.269	2.170	2.439	0.017
ENSG00000177427	MIEF2	0.194	2.663	2.001	0.049
ENSG00000177707	NECTIN3	-0.316	4.890	-2.415	0.018
ENSG00000178171	AMER3	0.379	4.776	2.283	0.025
ENSG00000178445	GLDC	-0.444	3.321	-3.194	0.002
ENSG00000178719	GRINA	0.354	4.972	2.250	0.027
ENSG00000179271	GADD45GIP1	0.395	2.999	3.374	0.001
ENSG00000179583	CIITA	0.372	3.232	2.171	0.033
ENSG00000179598	PLD6	0.307	2.916	2.027	0.046
ENSG00000179918	SEPHS2	0.265	3.618	2.181	0.033

ENSG00000180448	ARHGAP45	0.509	1.627	2.338	0.022
ENSG00000180626	ZNF594	-0.153	5.597	-2.335	0.022
ENSG00000181027	FKRP	0.206	3.189	2.054	0.044
ENSG00000181035	SLC25A42	0.285	3.373	2.178	0.033
ENSG00000181218	HIST3H2A	0.540	2.126	2.261	0.027
ENSG00000182000	RP11-651P23.2	-1.282	-3.067	-2.500	0.015
ENSG00000182400	TRAPPC6B	0.237	5.167	2.262	0.027
ENSG00000182809	CRIP2	0.502	3.425	2.461	0.016
ENSG00000182983	ZNF662	-0.246	3.778	-2.189	0.032
ENSG00000183020	AP2A2	0.193	5.812	2.078	0.041
ENSG00000183090	FREM3	-0.981	0.616	-2.042	0.045
ENSG00000183114	FAM43B	0.609	2.139	3.621	0.001
ENSG00000183317	EPHA10	0.431	3.723	2.515	0.014
ENSG00000183570	PCBP3	0.325	2.568	2.557	0.013
ENSG00000183691	NOG	0.472	2.064	2.000	0.049
ENSG00000183751	TBL3	0.242	2.455	2.164	0.034
ENSG00000183807	FAM162B	-0.506	-0.015	-1.997	0.050
ENSG00000183808	RBM12B	-0.181	5.845	-2.738	0.008
ENSG00000183873	SCN5A	0.666	0.933	2.149	0.035
ENSG00000183921	SDR42E2	-0.674	-1.697	-2.300	0.024
ENSG00000184226	PCDH9	-0.316	10.424	-2.387	0.020
ENSG00000184281	TSSC4	0.335	1.809	2.076	0.041
ENSG00000184436	THAP7	0.319	2.196	2.355	0.021
ENSG00000184489	PTP4A3	0.317	1.638	2.247	0.028
ENSG00000184497	TMEM255B	0.467	0.689	1.999	0.049
ENSG00000184544	DHRS7C	-0.953	-5.699	-2.583	0.012
ENSG00000184897	H1FX	0.555	2.077	2.301	0.024
ENSG00000184916	JAG2	0.307	2.621	2.148	0.035
ENSG00000185052	SLC24A3	0.400	4.788	2.239	0.028
ENSG00000185070	FLRT2	-0.248	7.224	-2.259	0.027
ENSG00000185090	MANEAL	0.578	2.024	3.619	0.001
ENSG00000185215	TNFAIP2	0.470	3.581	3.137	0.002
ENSG00000185436	IFNLR1	0.519	1.298	3.508	0.001
ENSG00000185483	ROR1	-0.422	1.908	-2.644	0.010
ENSG00000185499	MUC1	0.713	-0.649	2.154	0.035
ENSG00000185803	SLC52A2	0.401	0.502	2.202	0.031
ENSG00000186187	ZNRF1	0.281	3.459	2.363	0.021
ENSG00000186198	SLC51B	0.720	-0.211	2.122	0.037
ENSG00000186352	ANKRD37	-0.623	1.763	-2.460	0.016
ENSG00000186364	NUDT17	0.375	1.321	2.401	0.019
ENSG00000186777	ZNF732	0.550	2.734	2.099	0.039
ENSG00000186806	VSIG10L	0.572	0.400	2.919	0.005
ENSG00000186854	TRABD2A	-0.667	1.568	-2.738	0.008
ENSG00000187391	MAGI2	-0.126	7.928	-2.187	0.032
ENSG00000187486	KCNJ11	0.586	1.698	2.775	0.007
ENSG00000187504	RPL7P48	-0.780	-1.034	-2.016	0.048
ENSG00000187952	HS6ST1P1	1.116	-1.360	2.094	0.040

ENSG00000188011	RTP5	0.714	1.798	2.437	0.017
ENSG00000188603	CLN3	-0.370	0.977	-2.155	0.035
ENSG00000188647	PTAR1	-0.237	7.552	-2.146	0.035
ENSG00000188677	PARVB	0.274	4.741	2.645	0.010
ENSG00000188763	FZD9	0.679	-1.076	2.606	0.011
ENSG00000188991	SLC15A5	-0.595	-1.291	-2.019	0.047
ENSG00000189077	TMEM120A	0.284	2.365	2.275	0.026
ENSG00000189159	HN1	0.288	4.306	2.123	0.037
ENSG00000189190	ZNF600	-0.314	1.450	-2.036	0.045
ENSG00000189275	LINC01164	-1.675	-4.247	-2.316	0.023
ENSG00000189306	RRP7A	0.319	4.745	2.361	0.021
ENSG00000196159	FAT4	-0.446	5.349	-2.604	0.011
ENSG00000196236	XPNPEP3	-0.125	5.003	-2.072	0.042
ENSG00000196277	GRM7	0.271	5.397	2.070	0.042
ENSG00000196453	ZNF777	0.266	2.899	2.012	0.048
ENSG00000196465	MYL6B	0.245	4.542	2.118	0.038
ENSG00000196628	TCF4	-0.360	7.900	-2.680	0.009
ENSG00000196865	NHLRC2	-0.185	6.636	-2.563	0.012
ENSG00000196923	PDLIM7	0.458	2.045	2.135	0.036
ENSG00000197451	HNRNPAB	0.192	3.768	2.199	0.031
ENSG00000197584	KCNMB2	-0.507	1.509	-2.382	0.020
ENSG00000197586	ENTPD6	0.323	5.915	2.165	0.034
ENSG00000197608	ZNF841	-0.144	5.345	-2.138	0.036
ENSG00000197852	FAM212B	-0.411	-0.993	-2.028	0.046
ENSG00000197858	GPAA1	0.345	2.634	2.317	0.023
ENSG00000198113	TOR4A	0.559	-1.160	2.278	0.026
ENSG00000198218	QRICH1	0.161	5.599	2.329	0.023
ENSG00000198252	STYX	-0.192	4.974	-2.010	0.048
ENSG00000198315	ZKSCAN8	-0.133	5.953	-2.093	0.040
ENSG00000198561	CTNND1	-0.199	6.689	-2.124	0.037
ENSG00000198794	SCAMP5	0.301	6.380	2.254	0.027
ENSG00000198832	SELM	0.428	2.624	2.804	0.007
ENSG00000198925	ATG9A	0.262	4.125	2.924	0.005
ENSG00000199627	RNU6-1010P	-0.594	-1.140	-2.354	0.021
ENSG00000199753	SNORD104	0.697	-1.889	2.054	0.044
ENSG00000199880	RNU6-888P	0.673	-1.791	2.117	0.038
ENSG00000200215	SNORD113-6	0.658	-1.534	2.003	0.049
ENSG00000200243	RN7SKP79	0.613	1.752	2.471	0.016
ENSG00000200879	SNORD14E	-1.015	-1.205	-2.025	0.047
ENSG00000200913	SNORD46	1.162	-3.888	2.677	0.009
ENSG00000201025	SNORD74	-0.665	-1.734	-2.196	0.031
ENSG00000201820	Y_RNA	-0.736	-1.857	-2.726	0.008
ENSG00000202017	RNU6-806P	-1.455	-5.106	-2.760	0.007
ENSG00000202270	SNORD114-12	0.730	-2.367	2.064	0.043
ENSG00000203808	BVES-AS1	-0.445	-0.083	-2.291	0.025
ENSG00000204160	ZDHHC18	0.299	3.419	2.547	0.013
ENSG00000204175	GPRIN2	1.176	-1.290	2.498	0.015

ENSG00000204179	PTPN20	0.420	3.492	2.040	0.045
ENSG00000204310	AGPAT1	0.328	3.662	2.071	0.042
ENSG00000204531	POU5F1	0.623	-2.534	1.997	0.050
ENSG00000204619	PPP1R11	0.179	4.680	2.137	0.036
ENSG00000204909	SPINK9	-0.493	0.642	-2.326	0.023
ENSG00000205208	C4orf46	-0.306	2.601	-2.511	0.014
ENSG00000205268	PDE7A	-0.200	5.018	-2.262	0.027
ENSG00000205744	DENND1C	0.541	0.503	2.589	0.012
ENSG00000206605	RNU6-946P	-0.570	-2.051	-2.082	0.041
ENSG00000207001	SNORD116-2	0.918	-0.884	2.641	0.010
ENSG00000207178	RNU6-1122P	1.504	-4.597	2.957	0.004
ENSG00000207217	SNORA42	0.724	-2.397	2.131	0.037
ENSG00000207442	SNORD116-6	0.602	-0.979	2.011	0.048
ENSG00000207954	MIR138-1	0.996	0.618	2.729	0.008
ENSG00000210156	MT-TK	0.743	0.301	2.100	0.039
ENSG00000211445	GPX3	0.570	4.366	2.604	0.011
ENSG00000211454	AKR7L	0.354	0.625	2.022	0.047
ENSG00000212123	PRR22	0.729	-1.916	2.412	0.018
ENSG00000213023	SYT3	0.482	1.942	2.091	0.040
ENSG00000213221	DNLZ	0.755	-2.059	2.066	0.042
ENSG00000213465	ARL2	0.400	3.230	2.103	0.039
ENSG00000213672	NCKIPSD	0.371	4.058	2.488	0.015
ENSG00000213995	NAXD	0.145	4.971	2.299	0.024
ENSG00000214093	RP11-247I13.3	-0.737	-2.001	-2.257	0.027
ENSG00000214160	ALG3	0.287	1.898	2.065	0.043
ENSG00000214212	C19orf38	0.770	-1.507	2.037	0.045
ENSG00000214517	PPME1	0.219	5.834	2.274	0.026
ENSG00000214872	SMTNL1	0.723	-0.859	2.540	0.013
ENSG00000214922	HLA-F-AS1	0.383	1.772	2.390	0.019
ENSG00000214988	RPL7AP26	-1.087	-1.323	-3.198	0.002
ENSG00000215325	ASS1P10	0.928	-2.484	2.203	0.031
ENSG00000215386	MIR99AHG	-0.192	6.017	-2.022	0.047
ENSG00000215440	NPEPL1	0.393	1.376	2.097	0.040
ENSG00000216863	LY86-AS1	-0.625	2.588	-2.191	0.032
ENSG00000219445	RP11-3B12.3	0.503	-0.457	2.121	0.037
ENSG00000221643	SNORA77	-1.213	-3.562	-2.136	0.036
ENSG00000221983	UBA52	0.215	6.347	2.083	0.041
ENSG00000221988	PPT2	0.334	1.123	2.414	0.018
ENSG00000222365	SNORD12B	-0.573	-1.996	-2.041	0.045
ENSG00000222385	RN7SKP158	-0.491	-1.249	-2.037	0.045
ENSG00000223949	ROR1-AS1	-0.666	-0.808	-2.685	0.009
ENSG00000224733	RP1-86D1.5	-0.697	-0.258	-2.288	0.025
ENSG00000224790	AP000704.5	0.489	0.262	2.410	0.019
ENSG00000224802	TUBB4BP2	0.627	-1.971	2.030	0.046
ENSG00000225578	NCBP2-AS1	0.801	-2.025	2.309	0.024
ENSG00000225649	AC064875.2	-0.409	0.485	-2.016	0.048
ENSG00000225886	RP11-288L9.4	0.921	-2.060	2.253	0.027

ENSG00000226410	AC104058.1	-0.522	-0.803	-2.055	0.044
ENSG00000226416	MRPL23-AS1	0.613	-0.963	2.117	0.038
ENSG00000226650	KIF4B	0.418	1.234	2.857	0.006
ENSG00000226744	AC079781.5	0.710	-1.139	2.253	0.027
ENSG00000227045	RP11-98D18.1	0.688	-1.429	2.058	0.043
ENSG00000227053	RP11-395B7.4	-1.043	-3.835	-2.028	0.046
ENSG00000227230	RP11-261C10.5	-0.468	-0.310	-2.387	0.020
ENSG00000227256	MIS18A-AS1	0.737	-1.577	2.601	0.011
ENSG00000227294	AC016994.2	-0.956	-1.917	-3.039	0.003
ENSG00000227583	RP11-119F7.3	-0.514	-0.806	-2.046	0.044
ENSG00000227617	CERS6-AS1	1.076	-2.354	3.211	0.002
ENSG00000227666	CYCSP24	-0.343	0.247	-2.006	0.049
ENSG00000227671	RP11-488L18.4	-0.354	3.420	-3.145	0.002
ENSG00000227785	AC092013.1	-0.891	-0.557	-2.141	0.036
ENSG00000227945	RP11-103C16.2	-0.571	-0.171	-2.366	0.021
ENSG00000227958	RP11-399H11.3	-1.090	-5.752	-2.462	0.016
ENSG00000228022	HCG20	-0.647	-1.731	-2.179	0.033
ENSG00000228151	AC004941.3	0.903	-1.540	2.519	0.014
ENSG00000228209	AC073091.2	-1.444	-5.484	-2.520	0.014
ENSG00000228793	RP1-223B1.1	-1.160	-0.068	-2.582	0.012
ENSG00000228903	RASA4CP	0.454	0.762	2.210	0.030
ENSG00000228983	SLC47A1P1	0.615	-1.810	2.237	0.028
ENSG00000229690	MTCO2P1	-1.126	-2.474	-2.187	0.032
ENSG00000229797	AC140481.8	0.798	-2.216	2.134	0.036
ENSG00000229808	RP11-456P18.2	-0.386	0.264	-2.025	0.047
ENSG00000230022	FNTAP2	-0.544	0.323	-2.089	0.040
ENSG00000230224	PHBP9	0.289	0.649	2.004	0.049
ENSG00000230310	CTD-2192J16.11	0.981	-1.671	2.802	0.007
ENSG00000230370	RPL23AP52	0.473	-0.995	2.244	0.028
ENSG00000230734	RPL10P3	0.718	-2.237	2.252	0.027
ENSG00000231133	HAR1B	0.806	-1.102	2.449	0.017
ENSG00000231165	TRBV26OR9-2	-0.521	0.216	-2.855	0.006
ENSG00000231181	RP13-15M17.1	-0.821	-1.281	-2.574	0.012
ENSG00000231310	TBL1XR1-AS1	-0.454	0.309	-2.836	0.006
ENSG00000231449	AC097359.2	-0.733	-1.667	-2.342	0.022
ENSG00000231490	RPL7L1P2	-0.700	-0.257	-2.164	0.034
ENSG00000231625	CTD-2104P17.1	0.550	-1.040	2.600	0.011
ENSG00000231688	RPL21P43	0.691	-0.783	2.271	0.026
ENSG00000231742	LINC01273	0.780	-2.254	2.289	0.025
ENSG00000231890	DARS-AS1	0.517	-0.114	2.244	0.028
ENSG00000231920	NEBL-AS1	0.578	-1.162	2.016	0.048
ENSG00000232036	GS1-115G20.2	-0.671	-0.529	-2.461	0.016
ENSG00000232542	DPYD-IT1	-0.559	-1.292	-2.039	0.045
ENSG00000232756	RP5-1185I7.1	1.437	-2.416	3.170	0.002
ENSG00000232821	AC003986.6	-1.341	-4.966	-2.449	0.017
ENSG00000232825	RP5-896L10.1	0.672	0.437	2.197	0.031
ENSG00000232903	LINC01166	0.920	-1.029	2.147	0.035

ENSG00000232953	HSPA8P18	0.746	-0.626	2.013	0.048
ENSG00000233123	LINC01007	-1.374	-2.589	-2.129	0.037
ENSG00000233509	ZNF197-AS1	-0.514	-0.612	-2.675	0.009
ENSG00000233718	MYCNOS	0.681	-1.539	2.301	0.024
ENSG00000233757	AC092835.2	-0.141	3.822	-2.224	0.029
ENSG00000233840	PCDH9-AS4	-0.429	3.179	-2.468	0.016
ENSG00000233937	CTC-338M12.4	0.768	-1.830	2.017	0.047
ENSG00000234233	KCNH1-IT1	-0.514	0.433	-2.008	0.048
ENSG00000234358	AC003080.4	-0.330	1.334	-2.137	0.036
ENSG00000234492	RPL34-AS1	-0.412	0.506	-2.169	0.033
ENSG00000234604	RP1-206D15.5	-0.582	-1.029	-2.131	0.037
ENSG00000234724	HDAC1P1	-0.728	-1.947	-2.030	0.046
ENSG00000235271	LINC01422	0.719	-1.091	2.595	0.011
ENSG00000235277	AF127577.8	-0.863	-2.238	-2.454	0.017
ENSG00000235299	MRPL53P1	-0.450	-1.215	-2.242	0.028
ENSG00000235499	AC073046.25	-1.001	-2.633	-2.633	0.010
ENSG00000235527	HIPK1-AS1	-0.341	1.232	-2.648	0.010
ENSG00000235605	RP5-827C21.1	0.786	0.853	2.254	0.027
ENSG00000235674	LDHAP2	0.485	1.299	2.220	0.030
ENSG00000235772	AP001625.6	0.655	-0.226	2.577	0.012
ENSG00000235833	AC159540.14	-1.788	0.336	-2.283	0.025
ENSG00000236204	LINC01376	-0.337	0.928	-2.341	0.022
ENSG00000236699	ARHGEF38	0.424	-0.595	2.428	0.018
ENSG00000236779	RP11-430C7.2	0.533	-0.566	1.994	0.050
ENSG00000236797	SPA17P1	0.813	-0.379	2.475	0.016
ENSG00000236810	TCEB3-AS1	-0.374	0.274	-2.066	0.042
ENSG00000237194	SNAI1P1	1.064	-1.203	2.820	0.006
ENSG00000237212	RP11-569G13.2	0.657	-0.474	2.231	0.029
ENSG00000237250	RP11-193H5.1	-1.406	-5.144	-2.801	0.007
ENSG00000237512	UNC5B-AS1	-0.913	-2.751	-2.253	0.027
ENSG00000237638	LINC02245	0.812	-1.499	2.206	0.031
ENSG00000237766	GGTA2P	-0.381	0.336	-2.223	0.029
ENSG00000238243	OR2W3	0.984	-0.464	3.158	0.002
ENSG00000238386	RNU7-48P	-0.666	-2.212	-2.174	0.033
ENSG00000238390	SNORA81	-0.479	-0.483	-2.228	0.029
ENSG00000239419	RN7SL535P	-0.775	-2.064	-2.333	0.022
ENSG00000239445	ST3GAL6-AS1	0.955	-1.441	2.596	0.011
ENSG00000240449	RP4-584D14.5	0.496	-0.902	2.335	0.022
ENSG00000240898	RP11-48B14.1	-0.504	-1.184	-2.110	0.038
ENSG00000241058	NSUN6	-0.181	4.748	-2.057	0.043
ENSG00000241360	PDXP	1.397	-1.415	2.693	0.009
ENSG00000241487	RN7SL586P	-1.247	-3.317	-2.554	0.013
ENSG00000241684	ADAMTS9-AS2	-0.383	3.248	-2.042	0.045
ENSG00000241878	PISD	0.302	4.752	2.799	0.007
ENSG00000242101	RN7SL416P	-1.032	-3.702	-2.220	0.030
ENSG00000242125	SNHG3	0.249	2.821	2.203	0.031
ENSG00000242525	OR7E100P	0.698	-1.600	2.324	0.023

ENSG00000242612	DECR2	0.303	1.002	2.198	0.031
ENSG00000242889	RN7SL449P	-0.548	-0.256	-2.118	0.038
ENSG00000243321	LINC01998	-0.677	-0.638	-2.224	0.029
ENSG00000243477	NAT6	0.346	1.223	1.999	0.049
ENSG00000243508	RP11-775J23.2	-0.395	1.129	-2.096	0.040
ENSG00000243678	NME2	0.741	-0.245	2.794	0.007
ENSG00000243902	ELFN2	0.708	-0.740	2.398	0.019
ENSG00000244486	SCARF2	0.636	-1.655	2.064	0.043
ENSG00000245017	LINC02453	0.865	-2.084	2.333	0.022
ENSG00000245213	RP11-10K16.1	-0.318	1.802	-2.062	0.043
ENSG00000245688	CTB-26E19.1	-1.439	-3.798	-2.266	0.027
ENSG00000246640	RP11-1094H24.4	0.537	-1.038	2.331	0.023
ENSG00000247925	RP3-510L9.1	-0.627	-1.786	-2.331	0.023
ENSG00000247934	RP11-967K21.1	0.438	1.009	2.588	0.012
ENSG00000248125	CTB-73N10.1	-0.929	-2.219	-2.564	0.012
ENSG00000248150	LINC02150	0.609	-0.398	2.076	0.042
ENSG00000248249	RP11-598O12.1	-0.500	1.120	-2.174	0.033
ENSG00000248285	RP11-158J3.2	-1.058	-3.789	-2.079	0.041
ENSG00000248466	RP11-640B6.1	0.743	-2.092	2.290	0.025
ENSG00000248487	ABHD14A	0.408	1.901	2.305	0.024
ENSG00000249234	RP11-452J21.2	-0.589	-0.995	-2.714	0.008
ENSG00000249258	RP11-468H14.2	0.394	0.747	2.225	0.029
ENSG00000249363	CTB-78O21.1	-1.053	-1.521	-3.031	0.003
ENSG00000249547	RP11-453O5.1	-0.985	-5.358	-2.106	0.039
ENSG00000249562	RP11-116O11.2	-0.609	-1.310	-2.043	0.045
ENSG00000249673	NOP14-AS1	0.187	3.699	2.211	0.030
ENSG00000249678	RP11-619J20.1	-0.457	-0.935	-2.029	0.046
ENSG00000250064	RP11-123O22.1	-1.338	-3.873	-2.315	0.023
ENSG00000250378	RP11-119J18.1	0.899	-2.442	2.310	0.024
ENSG00000250643	RP11-93K22.6	-1.269	-3.627	-2.544	0.013
ENSG00000250706	RP11-18H21.2	-1.142	-5.393	-2.235	0.029
ENSG00000250934	RP11-71E19.1	0.536	0.217	2.378	0.020
ENSG00000250974	LINC02196	-0.743	-1.799	-2.182	0.032
ENSG00000251011	TMEM108-AS1	-0.782	1.171	-2.361	0.021
ENSG00000251136	RP11-37B2.1	-0.191	5.231	-2.020	0.047
ENSG00000251441	RTEL1P1	0.570	-0.089	2.087	0.040
ENSG00000251488	RP11-404I7.2	0.599	-1.271	2.445	0.017
ENSG00000251523	RP11-724M22.1	-0.647	-1.070	-2.335	0.022
ENSG00000251661	RP11-326C3.11	0.425	0.522	2.008	0.048
ENSG00000251682	RP11-631M6.3	-0.392	3.332	-2.273	0.026
ENSG00000252835	SCARNA21	0.427	3.966	2.209	0.030
ENSG00000253161	LINC01605	-0.991	-2.722	-2.277	0.026
ENSG00000253525	CTD-2114J12.1	-1.225	0.022	-2.459	0.016
ENSG00000253607	RP11-557C18.3	0.554	-1.010	2.273	0.026
ENSG00000253730	RP11-893F2.13	0.566	-1.643	2.144	0.035
ENSG00000253734	LINC01289	0.594	-0.176	2.274	0.026
ENSG00000253932	RP11-10C8.2	-0.462	1.355	-2.271	0.026

ENSG00000254163	CTC-340I23.2	-0.623	-1.742	-2.460	0.016
ENSG00000254260	RP11-369E15.1	-1.377	-5.187	-2.367	0.021
ENSG00000254587	RP11-360K13.1	-1.067	-3.548	-2.316	0.023
ENSG00000254781	GVINP2	0.512	0.217	2.322	0.023
ENSG00000254915	RP11-263C24.3	-0.711	-1.962	-2.032	0.046
ENSG00000254921	RP11-236J17.6	0.599	0.042	2.839	0.006
ENSG00000255000	AC139103.1	0.635	-1.498	2.073	0.042
ENSG00000255206	RP11-403D15.2	-0.629	0.196	-2.537	0.013
ENSG00000255219	RP11-716H6.2	-0.660	-0.007	-2.212	0.030
ENSG00000255384	RP11-770J1.4	0.302	0.297	2.134	0.036
ENSG00000255565	RP11-6B19.1	-0.486	-0.498	-2.042	0.045
ENSG00000256120	RP11-778H2.1	-0.735	-1.049	-2.735	0.008
ENSG00000256321	RP11-153K16.1	-1.242	-3.838	-2.205	0.031
ENSG00000256637	LINC01965	-0.839	-0.113	-2.610	0.011
ENSG00000256694	RP11-598F7.5	0.716	-1.869	2.121	0.037
ENSG00000256862	RP11-664D1.1	0.251	1.204	2.046	0.044
ENSG00000256922	RP11-749H20.1	-1.229	-5.065	-2.275	0.026
ENSG00000257045	RP11-486F17.1	-1.528	-3.794	-2.407	0.019
ENSG00000257557	RP11-84G21.1	-0.370	0.598	-2.197	0.031
ENSG00000257864	RP11-46I1.2	-0.681	-2.161	-2.160	0.034
ENSG00000258168	RP11-588H23.3	-0.324	2.846	-2.052	0.044
ENSG00000258199	RP11-977G19.5	0.416	0.901	2.712	0.008
ENSG00000258392	RPA2P1	0.921	-1.213	3.216	0.002
ENSG00000258455	RP11-665C16.5	-0.538	-0.948	-2.012	0.048
ENSG00000258537	FRMD6-AS2	-1.352	-4.108	-2.452	0.017
ENSG00000258640	RPL21P5	-0.464	-0.715	-2.176	0.033
ENSG00000258947	TUBB3	0.571	0.528	2.184	0.032
ENSG00000259091	LINC00517	-0.969	-2.037	-2.472	0.016
ENSG00000259362	RP11-307C19.1	0.651	-0.482	2.489	0.015
ENSG00000259548	RP11-38G5.2	0.507	-0.512	2.213	0.030
ENSG00000259642	ST20-AS1	0.302	1.055	2.264	0.027
ENSG00000260361	CTD-2313J17.5	0.746	-1.627	2.448	0.017
ENSG00000260398	RP11-594N15.3	0.412	3.381	2.241	0.028
ENSG00000260423	LINC02367	-0.777	-1.022	-2.554	0.013
ENSG00000260469	C15orf59-AS1	0.940	-2.234	2.310	0.024
ENSG00000260643	RP11-303E16.8	0.289	-0.044	2.154	0.035
ENSG00000260949	KB-1836B5.1	0.947	-2.079	2.363	0.021
ENSG00000260969	RP11-190D6.2	-0.719	-2.218	-2.084	0.041
ENSG00000260992	DOCK9-AS2	0.600	0.185	2.464	0.016
ENSG00000261136	RP11-37C7.3	0.482	0.735	2.271	0.026
ENSG00000261243	RP11-517C16.4	0.633	-2.125	2.193	0.032
ENSG00000261592	RP11-178L8.3	0.744	-1.623	2.363	0.021
ENSG00000261787	TCF24	-0.744	-2.012	-2.001	0.049
ENSG00000261842	RP11-143I21.1	0.957	-1.158	2.958	0.004
ENSG00000262681	RP11-311F12.1	1.055	-0.854	2.607	0.011
ENSG00000263050	RP11-667K14.3	0.282	1.144	2.024	0.047
ENSG00000263904	RP11-344B2.3	-0.849	-2.261	-2.082	0.041

ENSG00000264084	MIR5688	0.746	-2.231	2.389	0.020
ENSG00000264315	HNRNPA1P11	-1.590	-4.280	-2.113	0.038
ENSG00000264384	RN7SL431P	-0.423	0.569	-2.243	0.028
ENSG00000264754	CTD-2653B5.1	-0.515	-0.313	-2.033	0.046
ENSG00000264869	RP11-526H11.1	-1.307	-4.817	-2.684	0.009
ENSG00000264954	PRR29-AS1	0.588	0.008	2.170	0.033
ENSG00000264978	RN7SL630P	-0.710	-1.691	-2.391	0.019
ENSG00000265123	RN7SL200P	-0.554	-1.179	-2.064	0.043
ENSG00000265185	SNORD3B-1	0.892	3.533	2.112	0.038
ENSG00000266680	RP5-1148A21.3	0.637	-0.932	2.393	0.019
ENSG00000266994	RP11-127I20.7	-0.593	-2.052	-2.036	0.045
ENSG00000267039	RP11-58G13.1	-0.417	0.027	-2.166	0.034
ENSG00000267065	LINC02080	0.805	-1.169	2.070	0.042
ENSG00000267333	ASNSP6	0.775	-1.851	2.213	0.030
ENSG00000267402	TCF4-AS2	-0.479	-0.589	-2.193	0.032
ENSG00000267475	CTD-2538C1.2	0.526	-0.578	2.350	0.022
ENSG00000267673	FDX2	1.263	-2.672	3.717	0.000
ENSG00000267890	CTD-2126E3.4	1.135	-1.146	2.394	0.019
ENSG00000268403	CTD-2371O3.3	-0.454	0.803	-2.932	0.005
ENSG00000268743	CTD-2538G9.5	0.540	-0.979	2.101	0.039
ENSG00000268858	RP4-591C20.9	0.346	1.674	2.137	0.036
ENSG00000269867	CTD-2583A14.8	0.593	-1.344	2.073	0.042
ENSG00000270441	RP11-694I15.7	0.374	0.812	2.060	0.043
ENSG00000270640	RP11-373D23.2	-1.533	-1.471	-3.450	0.001
ENSG00000270876	ZNF30-AS1	-0.520	-1.007	-2.146	0.035
ENSG00000270878	CTD-2302E22.5	0.735	-1.035	2.714	0.008
ENSG00000270986	RP11-123C21.2	-0.451	-0.040	-2.476	0.016
ENSG00000271075	RP11-589M4.4	-0.604	-0.783	-2.326	0.023
ENSG00000271207	MTCO1P22	1.340	-0.956	2.322	0.023
ENSG00000271538	LINC02427	0.452	1.331	2.064	0.043
ENSG00000271858	CYB561D2	0.804	-2.332	2.474	0.016
ENSG00000272009	RP1-313I6.12	0.554	-1.270	2.299	0.024
ENSG00000272031	ANKRD34A	0.500	2.981	2.288	0.025
ENSG00000272040	CTC-366B18.4	-0.363	0.426	-2.097	0.040
ENSG00000272223	RP1-20C7.6	0.695	-2.355	2.444	0.017
ENSG00000272343	RP11-140I16.3	-0.511	-0.127	-2.195	0.031
ENSG00000272509	RP11-347C18.5	-0.444	-0.577	-2.429	0.018
ENSG00000272619	RP11-563K23.1	-0.332	0.292	-2.150	0.035
ENSG00000272721	RP11-778D9.12	-0.668	-2.487	-2.005	0.049
ENSG00000272734	ADIRF-AS1	0.463	-0.115	2.025	0.047
ENSG00000272758	RP11-299J3.8	-0.276	1.414	-2.355	0.021
ENSG00000272888	LINC01578	-0.196	6.328	-2.157	0.034
ENSG00000272913	RP11-440D17.3	0.517	-0.974	2.208	0.030
ENSG00000273062	RP11-428K3.1	0.780	-1.184	2.746	0.008
ENSG00000273084	RP11-1275H24.3	0.458	-0.162	2.399	0.019
ENSG00000273321	RP11-621L6.3	-0.725	-1.643	-2.247	0.028
ENSG00000273328	RP11-141M3.6	0.653	-1.491	2.138	0.036

ENSG00000273344	PAXIP1-AS1	0.319	2.719	2.281	0.026
ENSG00000273384	RP5-1098D14.1	0.793	-1.971	2.617	0.011
ENSG00000273893	RP1-101A2.1	0.274	0.891	2.106	0.039
ENSG00000274525	CTC-338M12.13	-0.546	-1.328	-2.080	0.041
ENSG00000274605	RP11-12G12.7	-0.383	-0.209	-2.112	0.038
ENSG00000275074	NUDT18	0.360	1.840	2.235	0.029
ENSG00000275197	RP11-295I5.4	-0.576	-1.776	-2.064	0.043
ENSG00000275232	RP11-897M7.4	-1.327	-5.616	-3.009	0.004
ENSG00000275468	LLNLR-304A6.2	0.842	-2.378	2.012	0.048
ENSG00000275560	RP11-180M15.7	-0.451	0.046	-2.148	0.035
ENSG00000276571	AC002550.6	-0.431	-0.049	-2.172	0.033
ENSG00000276653	RN7SL856P	-0.593	-1.216	-2.129	0.037
ENSG00000276696	Y_RNA	-0.458	-1.259	-2.051	0.044
ENSG00000277200	RP11-74E22.8	0.363	2.114	2.039	0.045
ENSG00000277692	RP11-358N2.2	0.529	-0.758	2.308	0.024
ENSG00000277708	PGBD4P2	0.951	-2.455	2.452	0.017
ENSG00000278058	RP11-70D24.2	-0.878	-1.809	-2.694	0.009
ENSG00000278535	DHRS11	0.323	2.771	2.900	0.005
ENSG00000278558	TMEM191B	1.186	-1.808	2.601	0.011
ENSG00000278627	RP11-506H21.5	0.451	0.383	2.931	0.005
ENSG00000278740	RP11-147L13.14	-0.380	0.657	-2.105	0.039
ENSG00000278925	AC005753.1	0.794	-0.601	2.263	0.027
ENSG00000279085	CTA-243E7.4	-0.621	-0.359	-2.019	0.047
ENSG00000279212	RP13-16H11.8	0.848	-2.967	2.045	0.045
ENSG00000279285	RP11-64C12.5	-1.266	-5.634	-2.740	0.008
ENSG00000279384	RP11-635L1.2	-0.433	0.313	-2.037	0.045
ENSG00000279727	LINC02033	0.593	0.327	1.997	0.050
ENSG00000279910	RP11-113E21.2	0.670	0.175	2.021	0.047
ENSG00000279995	RP11-1250I15.1	-0.636	-1.017	-2.457	0.016
ENSG00000280010	AP001350.4	-0.558	-0.115	-2.798	0.007
ENSG00000280035	RP11-10J21.2	-0.681	-0.588	-2.036	0.045
ENSG00000280088	RP11-135F9.3	0.367	2.661	2.027	0.046
ENSG00000280129	RP11-298I3.3	-0.470	-0.283	-2.110	0.038
ENSG00000280187	CTC-351M12.1	0.235	1.628	2.174	0.033
ENSG00000280243	MTCO1P1	-0.976	-1.403	-2.012	0.048
ENSG00000280281	RP11-340L3.1	0.735	-0.968	2.119	0.038
ENSG00000280310	RP11-326I19.2	0.631	-0.714	2.287	0.025
ENSG00000280432	AP000962.1	-0.446	1.276	-2.812	0.006
ENSG00000280739	EIF1B-AS1	-0.316	2.194	-2.543	0.013
ENSG00000281195	ZNF638-IT1	-0.207	4.446	-2.006	0.049
ENSG00000281248	LINC02536	-1.376	-5.105	-2.924	0.005
ENSG00000281344	HELLPAR	0.294	2.168	1.999	0.049
ENSG00000282022	RP11-259O18.5	0.598	-0.102	2.076	0.041
ENSG00000283653	RP11-373N22.4	-0.706	-1.092	-2.587	0.012
ENSG00000283891	MIR628	0.729	-0.754	2.036	0.045
ENSG00000284691	#N/A	0.299	1.867	2.120	0.037
ENSG00000285331	#N/A	0.226	2.401	2.469	0.016

ENSG00000285550	#N/A	-1.381	-5.688	-3.284	0.002
ENSG00000285586	#N/A	0.753	-0.904	2.528	0.014
ENSG00000285619	#N/A	0.770	-1.799	2.323	0.023
ENSG00000285633	#N/A	-1.262	-3.114	-2.078	0.041
ENSG00000285647	#N/A	1.171	-3.844	2.006	0.049
ENSG00000285697	#N/A	0.516	-0.888	2.408	0.019
ENSG00000285728	#N/A	0.361	-0.044	2.346	0.022
ENSG00000285825	#N/A	-0.507	-0.564	-2.307	0.024
ENSG00000285850	#N/A	-0.580	0.700	-2.181	0.033
ENSG00000285914	#N/A	-0.731	-5.699	-2.001	0.049

vSUB

gene_id	gene_name	logFC	AveExpr	t	P.Value
ENSG00000000938	FGR	0.654	0.317	2.046	0.045
ENSG00000002745	WNT16	0.779	0.879	2.044	0.046
ENSG00000003400	CASP10	0.390	2.674	2.226	0.030
ENSG00000005981	ASB4	0.603	1.671	2.308	0.025
ENSG00000006740	ARHGAP44	-0.321	5.849	-2.237	0.029
ENSG00000008311	AASS	0.252	5.586	2.018	0.048
ENSG00000008394	MGST1	0.691	4.284	3.308	0.002
ENSG00000010379	SLC6A13	-0.439	1.916	-2.089	0.041
ENSG00000011083	SLC6A7	-0.638	3.519	-2.294	0.025
ENSG00000011478	QPCTL	-0.329	1.333	-2.076	0.042
ENSG00000012779	ALOX5	0.523	2.136	2.067	0.043
ENSG00000013016	EHD3	-0.371	6.714	-2.662	0.010
ENSG00000018625	ATP1A2	0.303	9.549	2.440	0.018
ENSG00000021826	CPS1	0.471	3.132	2.435	0.018
ENSG00000023171	GRAMD1B	-0.278	7.559	-2.178	0.034
ENSG00000031081	ARHGAP31	0.280	5.573	2.145	0.036
ENSG00000033627	ATP6V0A1	-0.230	7.909	-2.011	0.049
ENSG00000034053	APBA2	-0.322	7.025	-2.419	0.019
ENSG00000042493	CAPG	0.623	2.124	2.705	0.009
ENSG00000046889	PREX2	0.296	8.239	2.345	0.022
ENSG00000050438	SLC4A8	-0.313	7.807	-2.155	0.035
ENSG00000054219	LY75	0.509	0.937	2.208	0.031
ENSG00000054356	PTPRN	-0.433	6.181	-2.157	0.035
ENSG00000055163	CYFIP2	-0.359	8.560	-2.202	0.032
ENSG00000055208	TAB2	0.220	6.746	2.487	0.016
ENSG00000058335	RASGRF1	-0.424	7.095	-2.079	0.042
ENSG00000058404	CAMK2B	-0.529	6.847	-2.642	0.011
ENSG00000060709	RIMBP2	-0.358	6.347	-2.035	0.046
ENSG00000064961	HMG20B	0.302	2.748	2.195	0.032
ENSG00000065665	SEC61A2	-0.260	5.232	-2.077	0.042
ENSG00000066027	PPP2R5A	0.208	5.490	2.127	0.038
ENSG00000066382	MPPED2	-0.312	4.721	-2.147	0.036
ENSG00000067182	TNFRSF1A	0.375	2.916	2.011	0.049
ENSG00000067191	CACNB1	-0.356	5.543	-2.217	0.031
ENSG00000067715	SYT1	-0.568	10.277	-2.296	0.025
ENSG00000068079	IFI35	0.441	1.111	2.360	0.022
ENSG00000069424	KCNAB2	-0.323	6.900	-2.266	0.027
ENSG00000069431	ABCC9	0.359	5.452	2.094	0.041
ENSG00000071242	RPS6KA2	0.329	6.394	2.517	0.015
ENSG00000072786	STK10	0.334	3.474	2.913	0.005
ENSG00000072952	MRVI1	0.431	6.687	2.432	0.018
ENSG00000072958	AP1M1	-0.348	6.037	-2.170	0.034
ENSG00000073670	ADAM11	-0.512	4.946	-2.825	0.006
ENSG00000074047	GLI2	0.496	0.990	2.107	0.040
ENSG00000074201	CLNS1A	0.222	6.512	2.022	0.048

ENSG00000075035	WSCD2	-0.518	5.454	-2.387	0.020
ENSG00000075043	KCNQ2	-0.383	7.253	-2.014	0.049
ENSG00000075340	ADD2	-0.444	8.284	-2.342	0.023
ENSG00000076248	UNG	0.205	3.780	2.106	0.040
ENSG00000076555	ACACB	0.324	5.427	2.270	0.027
ENSG00000077782	FGFR1	-0.356	5.734	-2.193	0.032
ENSG00000078053	AMPH	-0.486	6.340	-2.209	0.031
ENSG00000079101	CLUL1	-0.576	2.514	-2.088	0.041
ENSG00000079215	SLC1A3	0.409	9.510	2.508	0.015
ENSG00000079335	CDC14A	0.274	3.691	2.142	0.036
ENSG00000080189	SLC35C2	0.215	3.905	2.589	0.012
ENSG00000080493	SLC4A4	0.423	7.758	2.857	0.006
ENSG00000081818	PCDHB4	-0.281	3.742	-2.353	0.022
ENSG00000081853	PCDHGA2	0.224	2.918	2.006	0.050
ENSG00000083857	FAT1	0.237	8.113	2.186	0.033
ENSG00000085415	SEH1L	-0.279	5.681	-2.158	0.035
ENSG00000086730	LAT2	0.565	1.881	2.033	0.047
ENSG00000087074	PPP1R15A	-0.487	4.724	-2.229	0.030
ENSG00000089057	SLC23A2	-0.177	7.429	-2.442	0.018
ENSG00000089060	SLC8B1	0.252	2.977	2.341	0.023
ENSG00000089159	PXN	0.361	2.792	2.143	0.036
ENSG00000089177	KIF16B	0.205	5.687	2.102	0.040
ENSG00000089199	CHGB	-1.023	7.412	-3.343	0.001
ENSG00000093072	CECR1	0.292	3.529	2.061	0.044
ENSG00000096433	ITPR3	0.484	2.455	2.054	0.044
ENSG00000096654	ZNF184	-0.353	5.256	-2.016	0.049
ENSG00000099250	NRP1	-0.685	6.207	-2.649	0.010
ENSG00000099256	PRTFDC1	0.244	4.522	2.297	0.025
ENSG00000099284	H2AFY2	0.221	2.507	2.084	0.042
ENSG00000099937	SERPIND1	-0.371	1.815	-2.068	0.043
ENSG00000099957	P2RX6	-0.369	1.880	-2.138	0.037
ENSG00000100033	PRODH	0.491	4.859	2.686	0.009
ENSG00000100055	CYTH4	0.616	2.572	2.465	0.017
ENSG00000100060	MFNG	0.561	-0.572	2.032	0.047
ENSG00000100095	SEZ6L	-0.625	7.977	-2.350	0.022
ENSG00000100288	CHKB	-0.418	0.164	-2.028	0.047
ENSG00000100321	SYNGR1	-0.377	7.397	-2.503	0.015
ENSG00000100336	APOL4	2.154	-0.301	3.155	0.003
ENSG00000100427	MLC1	0.292	6.402	2.163	0.035
ENSG00000100504	PYGL	0.460	2.949	2.307	0.025
ENSG00000100767	PAPLN	0.403	3.887	2.345	0.022
ENSG00000100852	ARHGAP5	0.227	9.384	2.319	0.024
ENSG00000101198	NKAIN4	0.422	2.363	2.238	0.029
ENSG00000101210	EEF1A2	-0.385	7.231	-2.413	0.019
ENSG00000101342	TLDC2	0.633	-0.629	2.170	0.034
ENSG00000101367	MAPRE1	0.167	6.323	2.087	0.041
ENSG00000101542	CDH20	0.228	5.555	2.330	0.023

ENSG00000101751	POLI	0.181	5.996	2.341	0.023
ENSG00000102900	NUP93	-0.474	6.424	-2.787	0.007
ENSG00000103056	SMPD3	-0.646	4.796	-2.770	0.008
ENSG00000103653	CSK	0.263	3.056	2.091	0.041
ENSG00000103723	AP3B2	-0.311	6.114	-2.293	0.025
ENSG00000103740	ACSBG1	0.380	5.308	2.578	0.013
ENSG00000103942	HOMER2	-0.409	6.545	-2.560	0.013
ENSG00000104219	ZDHHC2	0.253	6.099	2.105	0.040
ENSG00000104760	FGL1	0.761	1.051	2.148	0.036
ENSG00000104870	FCGRT	0.371	2.705	2.082	0.042
ENSG00000104972	LILRB1	0.838	1.035	2.375	0.021
ENSG00000105193	RPS16	0.335	3.661	2.041	0.046
ENSG00000105287	PRKD2	0.473	1.981	2.903	0.005
ENSG00000105329	TGFB1	0.479	1.801	2.133	0.037
ENSG00000105699	LSR	0.345	1.688	2.126	0.038
ENSG00000105825	TFPI2	-0.793	-1.443	-2.217	0.031
ENSG00000105852	PON3	0.349	1.582	2.075	0.042
ENSG00000105854	PON2	0.379	6.527	2.982	0.004
ENSG00000105928	DFNA5	-0.310	4.531	-2.788	0.007
ENSG00000106069	CHN2	-0.479	2.173	-2.575	0.013
ENSG00000106299	WASL	-0.373	7.415	-2.185	0.033
ENSG00000106976	DNM1	-0.439	8.157	-2.439	0.018
ENSG00000107551	RASSF4	0.331	5.417	2.179	0.033
ENSG00000107736	CDH23	0.520	2.874	2.207	0.031
ENSG00000107738	C10orf54	0.614	3.315	3.132	0.003
ENSG00000107815	C10orf2	-0.219	2.287	-2.149	0.036
ENSG00000107938	EDRF1	-0.205	5.066	-2.273	0.027
ENSG00000108039	XPNPEP1	-0.165	5.095	-2.237	0.029
ENSG00000108242	CYP2C18	1.391	-2.187	2.856	0.006
ENSG00000108352	RAPGEFL1	-0.377	5.121	-2.351	0.022
ENSG00000108591	DRG2	-0.145	3.445	-2.145	0.036
ENSG00000108771	DHX58	0.373	2.602	2.783	0.007
ENSG00000108798	ABI3	0.635	1.131	2.350	0.022
ENSG00000109063	MYH3	0.439	2.485	2.264	0.027
ENSG00000109689	STIM2	-0.279	6.092	-2.510	0.015
ENSG00000110436	SLC1A2	0.446	12.531	2.205	0.031
ENSG00000110446	SLC15A3	0.485	0.980	2.021	0.048
ENSG00000110514	MADD	-0.336	7.184	-2.866	0.006
ENSG00000110675	ELMOD1	-0.512	6.539	-2.105	0.040
ENSG00000110848	CD69	-0.903	0.249	-2.356	0.022
ENSG00000111012	CYP27B1	-0.575	-1.761	-2.074	0.043
ENSG00000111058	ACSS3	0.294	5.014	2.137	0.037
ENSG00000111262	KCNA1	-0.761	6.390	-2.738	0.008
ENSG00000111275	ALDH2	0.257	6.114	2.686	0.009
ENSG00000111344	RASAL1	-0.357	4.494	-2.253	0.028
ENSG00000111783	RFX4	0.383	4.997	2.278	0.026
ENSG00000111961	SASH1	0.184	7.632	2.079	0.042

ENSG00000112186	CAP2	-0.531	7.261	-2.197	0.032
ENSG00000112290	WASF1	-0.500	6.778	-2.226	0.030
ENSG00000112303	VNN2	1.020	-0.464	2.728	0.008
ENSG00000112333	NR2E1	0.358	4.072	2.371	0.021
ENSG00000113161	HMGCR	-0.453	6.693	-2.213	0.031
ENSG00000113205	PCDHB3	-0.339	2.498	-2.017	0.048
ENSG00000113657	DPYSL3	0.393	7.310	3.516	0.001
ENSG00000113763	UNC5A	-0.554	4.665	-2.556	0.013
ENSG00000114248	LRRC31	0.787	-2.472	2.078	0.042
ENSG00000114554	PLXNA1	-0.327	6.056	-2.058	0.044
ENSG00000114738	MAPKAPK3	0.363	2.585	2.136	0.037
ENSG00000114739	ACVR2B	0.184	5.575	2.459	0.017
ENSG00000114790	ARHGEF26	0.226	5.378	2.152	0.036
ENSG00000115415	STAT1	0.375	6.155	2.575	0.013
ENSG00000115641	FHL2	-0.593	3.126	-2.864	0.006
ENSG00000115902	SLC1A4	0.346	6.346	3.045	0.004
ENSG00000115977	AAK1	-0.354	8.475	-3.216	0.002
ENSG00000116106	EPHA4	-0.623	7.748	-2.606	0.012
ENSG00000116117	PARD3B	0.362	4.899	2.290	0.026
ENSG00000116133	DHCR24	-0.380	6.684	-2.107	0.040
ENSG00000116641	DOCK7	0.209	7.137	2.410	0.019
ENSG00000116691	MIIP	0.439	1.362	2.916	0.005
ENSG00000117069	ST6GALNAC5	-0.675	5.823	-2.415	0.019
ENSG00000117154	IGSF21	-0.418	4.249	-2.161	0.035
ENSG00000117298	ECE1	0.297	4.628	2.093	0.041
ENSG00000117400	MPL	0.842	-2.048	2.320	0.024
ENSG00000117525	F3	0.464	6.103	2.478	0.016
ENSG00000118160	SLC8A2	-0.482	5.361	-2.189	0.033
ENSG00000118402	ELOVL4	-0.473	5.625	-2.415	0.019
ENSG00000118762	PKD2	0.201	5.906	2.445	0.018
ENSG00000119401	TRIM32	-0.232	4.644	-2.237	0.029
ENSG00000119408	NEK6	0.251	5.195	2.313	0.024
ENSG00000119471	HSDL2	0.277	6.382	2.444	0.018
ENSG00000119508	NR4A3	-0.706	4.737	-2.226	0.030
ENSG00000119535	CSF3R	0.692	2.843	2.058	0.044
ENSG00000119711	ALDH6A1	0.252	7.459	2.377	0.021
ENSG00000119917	IFIT3	0.310	4.968	2.125	0.038
ENSG00000119927	GPAM	0.374	6.160	2.398	0.020
ENSG00000119950	MXI1	0.229	6.623	2.157	0.035
ENSG00000120210	INSL6	0.435	0.274	2.198	0.032
ENSG00000120251	GRIA2	-0.434	9.926	-2.320	0.024
ENSG00000120318	ARAP3	0.482	1.694	2.524	0.014
ENSG00000120333	MRPS14	0.213	5.556	2.355	0.022
ENSG00000120334	CENPL	0.232	2.207	2.023	0.048
ENSG00000120669	SOHLH2	-0.826	-0.377	-2.079	0.042
ENSG00000120875	DUSP4	-1.035	4.405	-2.656	0.010
ENSG00000120896	SORBS3	0.315	3.157	2.018	0.048

ENSG00000121671	CRY2	-0.224	5.856	-2.108	0.039
ENSG00000121742	GJB6	0.544	5.508	2.374	0.021
ENSG00000121871	SLITRK3	-0.489	5.408	-2.776	0.007
ENSG00000122335	SERAC1	-0.295	4.954	-2.725	0.008
ENSG00000122877	EGR2	-1.109	0.540	-2.084	0.042
ENSG00000123104	ITPR2	0.314	7.831	2.847	0.006
ENSG00000123243	ITIH5	-0.399	4.476	-2.296	0.025
ENSG00000123358	NR4A1	-0.778	2.758	-2.267	0.027
ENSG00000123609	NMI	0.456	1.318	2.111	0.039
ENSG00000123636	BAZ2B	0.235	8.376	2.055	0.044
ENSG00000124104	SNX21	0.262	2.854	2.229	0.030
ENSG00000124140	SLC12A5	-0.379	7.094	-2.065	0.043
ENSG00000124171	PARD6B	-0.320	3.146	-2.161	0.035
ENSG00000124257	NEURL2	-0.537	-1.512	-2.480	0.016
ENSG00000124701	APOBEC2	0.677	-0.595	2.795	0.007
ENSG00000124782	RREB1	0.372	3.791	2.445	0.018
ENSG00000125285	SOX21	0.322	2.835	2.339	0.023
ENSG00000125378	BMP4	-0.786	-0.846	-2.129	0.038
ENSG00000125409	TEKT3	0.563	0.111	2.354	0.022
ENSG00000125730	C3	0.683	6.693	2.021	0.048
ENSG00000125740	FOSB	-1.183	1.521	-2.959	0.004
ENSG00000126785	RHOJ	0.449	2.980	2.439	0.018
ENSG00000127249	ATP13A4	0.297	6.262	2.383	0.021
ENSG00000127334	DYRK2	-0.372	5.657	-2.404	0.019
ENSG00000127831	VIL1	0.743	-1.630	2.288	0.026
ENSG00000128011	LRFN1	-0.414	1.624	-2.085	0.041
ENSG00000128284	APOL3	0.666	2.414	2.700	0.009
ENSG00000128394	APOBEC3F	0.564	-0.476	2.088	0.041
ENSG00000128422	KRT17	1.796	-0.962	2.059	0.044
ENSG00000128482	RNF112	-0.425	4.702	-2.771	0.008
ENSG00000128604	IRF5	0.628	1.237	2.321	0.024
ENSG00000128849	CGNL1	0.356	6.049	2.368	0.021
ENSG00000129667	RHBDF2	0.682	2.417	2.421	0.019
ENSG00000129990	SYT5	-0.532	5.121	-2.448	0.017
ENSG00000130176	CNN1	-0.519	0.622	-2.097	0.040
ENSG00000130202	NECTIN2	0.387	1.991	2.409	0.019
ENSG00000130226	DPP6	-0.430	7.408	-2.478	0.016
ENSG00000130844	ZNF331	-0.229	5.385	-2.071	0.043
ENSG00000130940	CASZ1	0.939	-0.886	3.290	0.002
ENSG00000131042	LILRB2	1.157	-1.388	2.546	0.014
ENSG00000131626	PPFIA1	0.167	6.790	2.052	0.045
ENSG00000131845	ZNF304	-0.255	4.038	-2.281	0.026
ENSG00000131941	RHPN2	0.447	2.793	2.250	0.028
ENSG00000131969	ABHD12B	0.595	1.722	2.086	0.041
ENSG00000131981	LGALS3	0.425	2.940	2.250	0.028
ENSG00000132026	RTBDN	-0.573	-0.191	-2.041	0.046
ENSG00000132109	TRIM21	0.410	1.855	2.276	0.027

ENSG00000132470	ITGB4	0.603	4.587	2.141	0.037
ENSG00000132622	HSPA12B	0.705	1.304	3.019	0.004
ENSG00000132639	SNAP25	-0.543	10.391	-2.327	0.023
ENSG00000132640	BTBD3	-0.422	8.141	-2.108	0.039
ENSG00000132793	LPIN3	0.507	0.914	2.550	0.013
ENSG00000133019	CHRM3	-0.516	5.787	-2.323	0.024
ENSG00000133026	MYH10	-0.268	8.114	-2.121	0.038
ENSG00000133216	EPHB2	-0.411	3.944	-3.322	0.002
ENSG00000133321	RARRES3	0.588	1.983	2.395	0.020
ENSG00000133816	MICAL2	-0.557	6.947	-2.550	0.013
ENSG00000134138	MEIS2	0.522	5.213	2.146	0.036
ENSG00000134243	SORT1	-0.328	9.159	-2.175	0.034
ENSG00000134318	ROCK2	-0.254	8.470	-2.016	0.048
ENSG00000134508	CABLES1	0.426	5.571	2.153	0.036
ENSG00000134569	LRP4	0.313	7.112	2.797	0.007
ENSG00000134698	AGO4	0.158	5.825	2.045	0.045
ENSG00000134716	CYP2J2	0.326	4.418	2.134	0.037
ENSG00000134769	DTNA	0.298	8.916	2.664	0.010
ENSG00000134824	FADS2	0.311	5.876	2.085	0.042
ENSG00000135069	PSAT1	0.260	6.017	2.317	0.024
ENSG00000135094	SDS	0.478	1.722	2.046	0.045
ENSG00000135248	FAM71F1	0.724	-0.094	2.837	0.006
ENSG00000135314	KHDC1	-0.327	1.938	-2.542	0.014
ENSG00000135333	EPHA7	-0.573	6.333	-2.382	0.021
ENSG00000135365	PHF21A	0.260	6.452	2.498	0.015
ENSG00000135454	B4GALNT1	-0.577	4.770	-3.151	0.003
ENSG00000135622	SEMA4F	-0.299	4.084	-2.133	0.037
ENSG00000135744	AGT	0.355	6.260	2.055	0.044
ENSG00000135821	GLUL	0.331	10.071	2.236	0.029
ENSG00000135838	NPL	0.487	4.231	2.832	0.006
ENSG00000135899	SP110	0.298	3.547	2.140	0.037
ENSG00000135953	MFSD9	-0.390	2.876	-2.852	0.006
ENSG00000135973	GPR45	-0.525	-0.504	-2.099	0.040
ENSG00000136044	APPL2	0.181	5.736	2.147	0.036
ENSG00000136114	THSD1	0.364	2.893	2.337	0.023
ENSG00000136156	ITM2B	-0.233	8.490	-2.111	0.039
ENSG00000136193	SCRN1	0.262	8.730	2.108	0.039
ENSG00000136531	SCN2A	-0.484	9.319	-2.071	0.043
ENSG00000136826	KLF4	-0.693	1.952	-2.307	0.025
ENSG00000136854	STXBP1	-0.367	7.982	-2.262	0.027
ENSG00000136869	TLR4	0.343	6.657	2.161	0.035
ENSG00000136870	ZNF189	-0.256	6.438	-2.075	0.042
ENSG00000137033	IL33	0.413	4.528	2.206	0.031
ENSG00000137216	TMEM63B	-0.250	4.991	-2.419	0.019
ENSG00000137478	FCHSD2	0.277	6.056	2.469	0.017
ENSG00000137491	SLCO2B1	0.447	5.303	2.494	0.016
ENSG00000137976	DNASE2B	1.111	-2.251	2.387	0.020

ENSG00000138028	CGREF1	-0.288	4.027	-2.028	0.047
ENSG00000138030	KHK	-0.338	1.739	-2.076	0.042
ENSG00000138166	DUSP5	-0.654	2.402	-2.267	0.027
ENSG00000138376	BARD1	0.377	4.374	2.279	0.026
ENSG00000138496	PARP9	0.412	4.213	2.535	0.014
ENSG00000138696	BMPR1B	0.432	5.871	3.172	0.002
ENSG00000138758	SEPTIN11	0.246	7.959	2.444	0.018
ENSG00000138834	MAPK8IP3	-0.199	7.367	-2.058	0.044
ENSG00000139155	SLCO1C1	0.398	5.296	2.155	0.035
ENSG00000139174	PRICKLE1	-0.387	4.440	-2.138	0.037
ENSG00000139219	COL2A1	-1.321	-1.998	-2.425	0.018
ENSG00000139323	POC1B	0.209	5.216	2.088	0.041
ENSG00000139567	ACVRL1	0.593	2.243	2.139	0.037
ENSG00000139835	GRTP1	0.532	0.423	2.510	0.015
ENSG00000139998	RAB15	-0.427	6.448	-2.532	0.014
ENSG00000140022	STON2	0.534	7.088	3.058	0.003
ENSG00000140464	PML	0.351	3.001	2.506	0.015
ENSG00000140548	ZNF710	0.353	4.206	3.131	0.003
ENSG00000140839	CLEC18B	-0.769	-0.888	-2.530	0.014
ENSG00000140853	NLRC5	0.621	1.984	2.730	0.008
ENSG00000141314	RHBDL3	-0.308	3.752	-2.213	0.031
ENSG00000141576	RNF157	-0.310	6.911	-2.901	0.005
ENSG00000141639	MAPK4	0.466	5.909	2.582	0.012
ENSG00000141736	ERBB2	0.354	2.957	2.186	0.033
ENSG00000141854	MIR1199	0.319	1.493	2.307	0.025
ENSG00000141968	VAV1	0.687	2.215	2.123	0.038
ENSG00000142149	HUNK	-0.473	4.018	-2.476	0.016
ENSG00000142347	MYO1F	0.484	2.565	2.473	0.016
ENSG00000142408	CACNG8	-0.832	7.206	-3.104	0.003
ENSG00000142765	SYTL1	0.826	-0.721	2.319	0.024
ENSG00000142798	HSPG2	0.427	1.896	2.130	0.037
ENSG00000143126	CELSR2	-0.324	7.596	-2.023	0.048
ENSG00000143153	ATP1B1	-0.456	9.725	-2.144	0.036
ENSG00000143401	ANP32E	0.192	6.509	2.059	0.044
ENSG00000143429	AC027612.6	-1.018	0.167	-2.392	0.020
ENSG00000143437	ARNT	0.174	5.298	2.313	0.024
ENSG00000143494	VASH2	-0.383	2.202	-2.572	0.013
ENSG00000143502	SUSD4	-0.697	5.830	-2.988	0.004
ENSG00000143514	TP53BP2	0.235	6.487	2.211	0.031
ENSG00000143603	KCNN3	0.492	7.191	3.020	0.004
ENSG00000143772	ITPKB	0.384	6.833	2.341	0.023
ENSG00000143793	C1orf35	0.286	2.370	2.397	0.020
ENSG00000144120	TMEM177	-0.271	2.429	-2.110	0.039
ENSG00000144218	AFF3	-0.440	6.766	-2.798	0.007
ENSG00000144290	SLC4A10	-0.433	8.037	-2.072	0.043
ENSG00000144468	RHBDD1	0.189	4.983	2.091	0.041
ENSG00000144668	ITGA9	-0.476	4.876	-2.566	0.013

ENSG00000144749	LRIG1	0.337	6.433	2.502	0.015
ENSG00000144908	ALDH1L1	0.657	5.071	3.447	0.001
ENSG00000144909	OSBPL11	0.329	5.617	2.826	0.006
ENSG00000145050	MANF	-0.311	3.279	-2.135	0.037
ENSG00000145087	STXBP5L	-0.483	8.090	-2.442	0.018
ENSG00000145555	MYO10	0.252	7.280	2.237	0.029
ENSG00000145920	CPLX2	-0.546	9.328	-2.440	0.018
ENSG00000146409	SLC18B1	0.256	4.762	2.225	0.030
ENSG00000146700	SSC4D	-0.728	-0.783	-3.147	0.003
ENSG00000147408	CSGALNACT1	0.277	5.810	2.117	0.039
ENSG00000147421	HMBOX1	0.198	6.355	2.477	0.016
ENSG00000147509	RGS20	0.465	3.711	2.078	0.042
ENSG00000147650	LRP12	-0.364	6.199	-2.021	0.048
ENSG00000148053	NTRK2	0.223	10.719	2.226	0.030
ENSG00000148175	STOM	0.361	6.574	2.301	0.025
ENSG00000148411	NACC2	0.282	6.832	2.447	0.017
ENSG00000148655	C10orf11	0.425	2.390	2.238	0.029
ENSG00000148672	GLUD1	0.341	8.085	2.208	0.031
ENSG00000148730	EIF4EBP2	0.201	7.143	2.045	0.045
ENSG00000149090	PAMR1	0.296	4.632	2.447	0.017
ENSG00000149781	FERMT3	0.539	0.347	2.374	0.021
ENSG00000150477	KIAA1328	0.190	4.927	2.480	0.016
ENSG00000151014	NOCT	-0.372	3.086	-2.441	0.018
ENSG00000151322	NPAS3	0.227	6.370	2.423	0.019
ENSG00000151623	NR3C2	-0.431	6.275	-2.793	0.007
ENSG00000151687	ANKAR	0.261	4.654	2.779	0.007
ENSG00000151838	CCDC175	0.487	1.483	2.106	0.040
ENSG00000152213	ARL11	1.010	-0.819	2.155	0.035
ENSG00000152315	KCNK13	0.869	-0.524	2.532	0.014
ENSG00000153048	CARHSP1	0.314	3.312	2.034	0.047
ENSG00000153208	MERTK	0.330	5.458	2.140	0.037
ENSG00000153822	KCNJ16	0.434	4.748	2.035	0.046
ENSG00000154040	CABYR	-0.202	3.620	-2.119	0.038
ENSG00000154065	ANKRD29	-0.333	4.196	-2.197	0.032
ENSG00000154096	THY1	-0.519	7.264	-2.243	0.029
ENSG00000154133	ROBO4	0.536	1.431	2.644	0.011
ENSG00000154217	PITPNC1	0.246	6.567	2.093	0.041
ENSG00000154310	TNIK	0.224	8.219	2.539	0.014
ENSG00000155363	MOV10	0.288	3.116	2.253	0.028
ENSG00000155366	RHOC	0.442	2.866	2.876	0.006
ENSG00000155465	SLC7A7	0.404	1.711	2.288	0.026
ENSG00000155511	GRIA1	-0.648	8.508	-2.397	0.020
ENSG00000156026	MCU	-0.269	4.438	-2.124	0.038
ENSG00000156097	GPR61	-0.319	3.993	-2.007	0.049
ENSG00000156265	MAP3K7CL	0.428	0.766	2.215	0.031
ENSG00000156466	GDF6	0.740	-0.786	2.020	0.048
ENSG00000156535	CD109	0.414	4.042	2.337	0.023

ENSG00000156587	UBE2L6	0.405	3.980	3.075	0.003
ENSG00000157350	ST3GAL2	-0.255	4.569	-2.100	0.040
ENSG00000157404	KIT	-0.701	6.299	-2.373	0.021
ENSG00000157542	KCNJ6	-0.962	7.603	-3.016	0.004
ENSG00000157916	RER1	-0.146	4.881	-2.348	0.022
ENSG00000158258	CLSTN2	-0.798	8.443	-2.787	0.007
ENSG00000158683	PKD1L1	0.416	1.851	2.241	0.029
ENSG00000158769	F11R	0.450	2.321	2.091	0.041
ENSG00000158955	WNT9B	-0.699	-1.300	-2.030	0.047
ENSG00000159082	SYNJ1	-0.379	7.817	-2.071	0.043
ENSG00000159403	C1R	0.664	2.192	2.275	0.027
ENSG00000159423	ALDH4A1	0.429	2.503	2.264	0.027
ENSG00000159658	EFCAB14	0.168	7.473	2.087	0.041
ENSG00000159685	CHCHD6	-0.362	3.934	-2.336	0.023
ENSG00000159720	ATP6V0D1	-0.315	5.391	-2.312	0.024
ENSG00000159753	CARMIL2	-0.418	3.289	-2.500	0.015
ENSG00000159904	ZNF890P	-0.489	1.418	-2.060	0.044
ENSG00000160050	CCDC28B	-0.266	1.461	-2.110	0.039
ENSG00000160183	TMPRSS3	1.069	-0.292	3.049	0.003
ENSG00000160233	LRRC3	0.600	-0.461	2.229	0.030
ENSG00000160606	TLCD1	0.400	1.060	2.230	0.030
ENSG00000160678	S100A1	0.477	2.741	2.903	0.005
ENSG00000160712	IL6R	0.434	3.587	2.562	0.013
ENSG00000160716	CHRNB2	-0.355	5.262	-2.222	0.030
ENSG00000160796	NBEAL2	0.547	2.415	2.587	0.012
ENSG00000161091	MFSD12	-0.299	3.930	-2.082	0.042
ENSG00000161791	FMNL3	0.283	5.564	2.047	0.045
ENSG00000162144	CYB561A3	0.217	2.342	2.008	0.049
ENSG00000162365	CYP4A22	-0.583	0.438	-2.486	0.016
ENSG00000162407	PLPP3	0.362	6.515	2.695	0.009
ENSG00000162747	FCGR3B	1.278	-0.446	2.254	0.028
ENSG00000162769	FLVCR1	-0.207	4.539	-2.200	0.032
ENSG00000162873	KLHDC8A	-0.608	1.799	-2.003	0.050
ENSG00000162989	KCNJ3	-0.550	6.325	-2.069	0.043
ENSG00000162998	FRZB	-0.542	2.226	-2.539	0.014
ENSG00000163083	INHBB	0.385	2.203	2.064	0.044
ENSG00000163093	BBS5	-0.195	2.566	-2.100	0.040
ENSG00000163239	TDRD10	0.491	-0.968	2.005	0.050
ENSG00000163249	CCNYL1	0.238	3.824	2.034	0.047
ENSG00000163285	GABRG1	0.345	7.141	2.383	0.020
ENSG00000163359	COL6A3	-1.000	2.243	-2.059	0.044
ENSG00000163406	SLC15A2	0.360	5.569	2.889	0.005
ENSG00000163449	TMEM169	-0.345	3.723	-2.264	0.027
ENSG00000163618	CADPS	-0.508	8.060	-2.718	0.009
ENSG00000163637	PRICKLE2	-0.323	6.877	-2.268	0.027
ENSG00000163686	ABHD6	-0.352	5.293	-2.311	0.024
ENSG00000163735	CXCL5	0.690	0.395	2.495	0.015

ENSG00000163817	SLC6A20	-0.878	1.088	-2.202	0.032
ENSG00000163840	DTX3L	0.446	3.959	2.329	0.023
ENSG00000163909	HEYL	0.657	1.314	2.520	0.015
ENSG00000163947	ARHGEF3	-0.431	6.032	-2.827	0.006
ENSG00000164089	ETNPPL	0.515	7.147	2.813	0.007
ENSG00000164099	PRSS12	-0.831	3.359	-3.371	0.001
ENSG00000164111	ANXA5	0.314	5.619	2.098	0.040
ENSG00000164122	ASB5	1.173	-0.540	3.176	0.002
ENSG00000164188	RANBP3L	0.390	6.292	2.248	0.028
ENSG00000164241	C5orf63	0.381	2.561	2.153	0.036
ENSG00000164292	RHOBTB3	0.298	7.909	2.276	0.027
ENSG00000164326	CARTPT	-1.295	2.505	-2.360	0.022
ENSG00000164414	SLC35A1	-0.254	3.271	-2.427	0.018
ENSG00000164976	KIAA1161	0.310	5.875	2.166	0.034
ENSG00000165152	TMEM246	-0.454	6.372	-2.514	0.015
ENSG00000165271	NOL6	-0.289	4.024	-2.717	0.009
ENSG00000165300	SLITRK5	-0.407	6.844	-2.326	0.024
ENSG00000165379	LRFN5	-0.458	5.660	-2.522	0.014
ENSG00000165434	PGM2L1	-0.527	8.507	-2.012	0.049
ENSG00000165458	INPPL1	0.265	4.518	2.063	0.044
ENSG00000165795	NDRG2	0.303	8.372	2.637	0.011
ENSG00000165806	CASP7	0.452	2.753	2.090	0.041
ENSG00000165816	VWA2	0.536	0.470	2.469	0.017
ENSG00000166206	GABRB3	-0.439	7.584	-2.011	0.049
ENSG00000166224	SGPL1	0.184	5.420	2.094	0.041
ENSG00000166257	SCN3B	-0.733	8.234	-2.935	0.005
ENSG00000166439	RNF169	0.148	5.915	2.098	0.040
ENSG00000166448	TMEM130	-0.379	7.044	-2.027	0.047
ENSG00000166471	TMEM41B	-0.209	5.773	-2.364	0.021
ENSG00000166548	TK2	0.140	5.238	2.319	0.024
ENSG00000166780	C16orf45	-0.296	6.396	-2.304	0.025
ENSG00000166908	PIP4K2C	-0.304	5.485	-2.007	0.049
ENSG00000167434	CA4	-0.742	1.196	-2.627	0.011
ENSG00000167601	AXL	0.271	4.702	2.350	0.022
ENSG00000167604	NFKBID	-0.653	0.309	-3.056	0.003
ENSG00000167654	ATCAY	-0.249	7.105	-2.046	0.045
ENSG00000167994	RAB3IL1	0.462	1.348	2.495	0.015
ENSG00000168077	SCARA3	0.383	5.687	2.366	0.021
ENSG00000168234	TTC39C	-0.408	4.917	-2.780	0.007
ENSG00000168546	GFRA2	-0.586	3.533	-2.267	0.027
ENSG00000168610	STAT3	0.253	6.019	2.081	0.042
ENSG00000168710	AHCYL1	0.379	8.320	2.783	0.007
ENSG00000168913	ENHO	0.462	1.742	2.319	0.024
ENSG00000169006	NTSR2	0.455	3.747	2.162	0.035
ENSG00000169047	IRS1	-0.307	3.942	-2.380	0.021
ENSG00000169118	CSNK1G1	-0.284	4.785	-2.139	0.037
ENSG00000169169	CPT1C	-0.291	4.578	-2.393	0.020

ENSG00000169418	NPR1	0.672	-0.529	2.361	0.022
ENSG00000169439	SDC2	0.313	5.683	2.083	0.042
ENSG00000169744	LDB2	-0.496	6.460	-2.480	0.016
ENSG00000169752	NRG4	0.649	2.567	2.182	0.033
ENSG00000169760	NLGN1	-0.254	6.949	-2.051	0.045
ENSG00000169951	ZNF764	-0.242	2.144	-2.187	0.033
ENSG00000169994	MYO7B	0.737	-0.427	2.193	0.032
ENSG00000170074	FAM153A	-0.613	3.925	-2.010	0.049
ENSG00000170165	CR848007.2	-0.908	2.282	-3.331	0.002
ENSG00000170175	CHRNB1	0.203	2.364	2.004	0.050
ENSG00000170374	SP7	0.723	-1.463	2.110	0.039
ENSG00000170396	ZNF804A	-0.590	4.157	-2.496	0.015
ENSG00000170412	GPRC5C	0.380	1.410	2.014	0.049
ENSG00000170439	METTL7B	-0.992	1.605	-2.003	0.050
ENSG00000170703	TTLL6	0.489	0.701	2.378	0.021
ENSG00000170915	PAQR8	0.277	7.332	2.280	0.026
ENSG00000170989	S1PR1	0.360	5.632	2.342	0.023
ENSG00000171132	PRKCE	-0.528	7.127	-3.007	0.004
ENSG00000171159	C9orf16	-0.456	2.845	-2.335	0.023
ENSG00000171243	SOSTDC1	-0.960	1.431	-2.547	0.014
ENSG00000171574	ZNF584	-0.200	3.140	-2.230	0.030
ENSG00000171603	CLSTN1	-0.259	8.562	-2.189	0.033
ENSG00000171617	ENC1	-0.648	9.007	-2.196	0.032
ENSG00000171681	ATF7IP	0.130	7.251	2.063	0.044
ENSG00000171700	RGS19	0.502	0.725	2.337	0.023
ENSG00000171786	NHLH1	-0.654	-0.494	-2.260	0.028
ENSG00000171791	BCL2	0.215	5.924	2.234	0.029
ENSG00000171804	WDR87	1.022	-1.687	2.536	0.014
ENSG00000171873	ADRA1D	-0.954	0.969	-2.202	0.032
ENSG00000171885	AQP4	0.431	10.148	2.049	0.045
ENSG00000171903	CYP4F11	0.406	2.677	2.309	0.025
ENSG00000171940	ZNF217	0.346	3.665	2.158	0.035
ENSG00000172201	ID4	0.543	6.035	2.797	0.007
ENSG00000172638	EFEMP2	0.298	2.803	2.582	0.012
ENSG00000172794	RAB37	-0.501	2.752	-2.618	0.011
ENSG00000173200	PARP15	0.788	-0.339	2.577	0.013
ENSG00000173262	SLC2A14	-0.783	0.782	-2.170	0.034
ENSG00000173442	EHBP1L1	0.432	3.557	2.902	0.005
ENSG00000173705	SUSD5	-0.297	4.042	-2.023	0.048
ENSG00000173862	RP11-89N17.1	0.799	-1.693	2.394	0.020
ENSG00000174469	CNTNAP2	-0.501	7.858	-2.308	0.025
ENSG00000174514	MFSD4A	-0.407	5.934	-2.089	0.041
ENSG00000174871	CNIH2	-0.623	3.425	-2.128	0.038
ENSG00000174990	CA5A	1.192	-2.566	2.745	0.008
ENSG00000175175	PPM1E	-0.775	6.980	-2.510	0.015
ENSG00000175182	FAM131A	-0.349	5.517	-2.161	0.035
ENSG00000175318	GRAMD2	0.654	2.125	2.524	0.014

ENSG00000175489	LRRC25	1.323	-1.925	2.435	0.018
ENSG00000175544	CABP4	0.792	-0.417	3.475	0.001
ENSG00000175772	LINC01106	-0.784	2.647	-2.090	0.041
ENSG00000175874	CREG2	-0.701	8.617	-2.515	0.015
ENSG00000176055	MBLAC2	-0.293	4.809	-2.327	0.023
ENSG00000176124	DLEU1	0.300	2.855	2.182	0.033
ENSG00000176244	ACBD7	0.292	5.533	2.079	0.042
ENSG00000176390	CRLF3	-0.216	5.348	-2.032	0.047
ENSG00000176422	SPRYD4	-0.216	3.431	-2.013	0.049
ENSG00000176697	BDNF	-0.878	3.971	-2.437	0.018
ENSG00000176749	CDK5R1	-0.314	6.782	-2.044	0.046
ENSG00000176834	VSIG10	0.228	3.659	2.201	0.032
ENSG00000176956	LY6H	-0.388	4.093	-2.027	0.047
ENSG00000177200	CHD9	0.167	8.659	2.415	0.019
ENSG00000177406	RP11-218M22.1	0.330	2.144	2.037	0.046
ENSG00000177409	SAMD9L	0.501	4.590	2.659	0.010
ENSG00000177707	NECTIN3	-0.320	5.518	-2.285	0.026
ENSG00000177791	MYOZ1	0.487	-0.894	2.187	0.033
ENSG00000177990	DPY19L2	-0.317	4.449	-2.058	0.044
ENSG00000178075	GRAMD1C	0.397	4.893	2.771	0.008
ENSG00000178093	TSSK6	0.608	-1.911	2.210	0.031
ENSG00000178358	OR2D3	-0.542	0.279	-2.036	0.046
ENSG00000178965	ERICH3	-0.486	6.644	-2.069	0.043
ENSG00000179058	C9orf50	1.130	-2.885	2.737	0.008
ENSG00000179066	CTD-2527I21.15	0.580	-1.185	2.119	0.038
ENSG00000179397	C1orf101	0.283	3.089	2.649	0.010
ENSG00000179399	GPC5	0.460	5.047	2.489	0.016
ENSG00000179583	CIITA	0.478	3.504	2.255	0.028
ENSG00000179855	GIPC3	1.368	-1.351	3.991	0.000
ENSG00000180155	LYNX1	-0.379	6.829	-2.448	0.017
ENSG00000180263	FGD6	0.288	6.132	2.322	0.024
ENSG00000180370	PAK2	0.172	6.390	2.006	0.050
ENSG00000180448	ARHGAP45	0.493	2.061	2.647	0.010
ENSG00000180628	PCGF5	0.205	7.421	2.326	0.024
ENSG00000180914	OXTR	0.842	3.007	2.571	0.013
ENSG00000180938	ZNF572	0.441	2.317	2.928	0.005
ENSG00000181192	DHTKD1	0.166	5.090	2.137	0.037
ENSG00000181350	LRRC75A	-0.294	3.166	-2.227	0.030
ENSG00000181381	DDX60L	0.399	3.990	2.291	0.026
ENSG00000181481	RNF135	0.342	1.838	2.236	0.029
ENSG00000182054	IDH2	0.308	5.773	3.032	0.004
ENSG00000182511	FES	0.388	0.996	2.189	0.033
ENSG00000182902	SLC25A18	0.337	5.927	2.647	0.010
ENSG00000183066	WBP2NL	0.385	2.457	2.554	0.013
ENSG00000183307	CECR6	-0.469	3.637	-2.275	0.027
ENSG00000183454	GRIN2A	-0.566	8.705	-2.295	0.025
ENSG00000183486	MX2	0.417	1.747	2.040	0.046

ENSG00000183496	MEX3B	-0.380	1.935	-2.116	0.039
ENSG00000183571	PGPEP1L	0.893	-1.567	2.407	0.019
ENSG00000183662	FAM19A1	-0.606	4.849	-2.094	0.041
ENSG00000183715	OPCML	-0.685	7.937	-3.180	0.002
ENSG00000183765	CHEK2	0.382	0.469	2.253	0.028
ENSG00000183889	AC138969.4	-1.164	1.520	-2.063	0.044
ENSG00000184154	LRTOMT	0.269	2.714	2.032	0.047
ENSG00000184584	TMEM173	0.543	0.783	2.342	0.023
ENSG00000184612	RPL7P26	-0.600	-0.150	-2.464	0.017
ENSG00000184613	NELL2	-0.617	7.959	-2.212	0.031
ENSG00000184828	ZBTB7C	0.629	1.660	2.292	0.026
ENSG00000184995	IFNE	1.046	-1.934	2.090	0.041
ENSG00000185052	SLC24A3	-0.656	5.785	-2.081	0.042
ENSG00000185090	MANEAL	-0.374	2.104	-2.374	0.021
ENSG00000185115	NSMCE3	-0.333	4.134	-2.170	0.034
ENSG00000185187	SIGIRR	0.442	0.232	2.025	0.048
ENSG00000185274	WBSCR17	-0.531	5.723	-2.275	0.027
ENSG00000185432	METTL7A	0.283	7.224	2.064	0.044
ENSG00000185442	FAM174B	-0.274	3.202	-2.406	0.019
ENSG00000185499	MUC1	0.789	-0.872	2.227	0.030
ENSG00000185742	C11orf87	-0.488	6.609	-2.078	0.042
ENSG00000185760	KCNQ5	-0.684	6.157	-2.103	0.040
ENSG00000185924	RTN4RL1	-0.598	3.797	-2.436	0.018
ENSG00000186063	AIDA	0.220	4.335	2.258	0.028
ENSG00000186094	AGBL4	-0.309	4.462	-2.089	0.041
ENSG00000186198	SLC51B	0.688	0.114	2.262	0.027
ENSG00000186472	PCLO	-0.382	9.662	-2.136	0.037
ENSG00000186594	MIR22HG	-0.293	3.022	-2.003	0.050
ENSG00000186868	MAPT	-0.211	8.053	-2.023	0.048
ENSG00000187091	PLCD1	0.291	3.261	2.117	0.039
ENSG00000187122	SLIT1	-0.438	6.643	-2.009	0.049
ENSG00000187323	DCC	-0.496	5.753	-2.523	0.014
ENSG00000188152	NUTM2G	-0.585	-0.381	-2.142	0.036
ENSG00000188211	NCR3LG1	-0.356	5.423	-2.399	0.020
ENSG00000188517	COL25A1	-1.008	4.532	-2.515	0.015
ENSG00000188559	RALGAPA2	0.222	5.767	2.292	0.026
ENSG00000188649	CC2D2B	0.306	3.520	2.401	0.020
ENSG00000188760	TMEM198	-0.417	2.038	-2.026	0.047
ENSG00000188763	FZD9	0.423	-0.478	2.049	0.045
ENSG00000188779	SKOR1	0.488	-0.713	2.435	0.018
ENSG00000188803	SHISA6	-0.797	5.435	-2.193	0.032
ENSG00000189171	S100A13	0.447	3.667	2.523	0.014
ENSG00000189280	GJB5	-0.973	-1.066	-2.399	0.020
ENSG00000196091	MYBPC1	0.473	4.699	2.156	0.035
ENSG00000196220	SRGAP3	-0.240	7.334	-2.110	0.039
ENSG00000196277	GRM7	-0.283	5.480	-2.171	0.034
ENSG00000196353	CPNE4	-0.729	4.757	-2.745	0.008

ENSG00000196366	C9orf163	-0.956	-2.617	-2.209	0.031
ENSG00000196581	AJAP1	-0.440	6.809	-2.414	0.019
ENSG00000196876	SCN8A	-0.524	8.548	-3.034	0.004
ENSG00000196923	PDLIM7	0.400	1.503	2.284	0.026
ENSG00000196935	SRGAP1	0.286	5.380	2.423	0.019
ENSG00000197249	SERPINA1	1.090	0.963	2.058	0.044
ENSG00000197471	SPN	0.643	1.066	2.072	0.043
ENSG00000197670	RP4-724E16.2	0.784	-0.747	2.960	0.004
ENSG00000197769	MAP1LC3C	-1.564	-3.148	-3.009	0.004
ENSG00000197934	CYYR1-AS1	0.576	2.170	2.156	0.035
ENSG00000198046	ZNF667	-0.327	5.806	-2.837	0.006
ENSG00000198075	SULT1C4	0.304	3.064	2.108	0.039
ENSG00000198113	TOR4A	1.039	-1.186	2.434	0.018
ENSG00000198185	ZNF334	0.421	4.695	3.330	0.002
ENSG00000198216	CACNA1E	-0.460	8.356	-2.067	0.043
ENSG00000198246	SLC29A3	0.472	1.455	2.501	0.015
ENSG00000198576	ARC	-0.772	2.161	-2.439	0.018
ENSG00000198626	RYR2	-0.370	9.037	-2.089	0.041
ENSG00000198794	SCAMP5	-0.297	6.843	-2.106	0.040
ENSG00000198909	MAP3K3	0.173	4.170	2.049	0.045
ENSG00000198929	NOS1AP	-0.605	5.827	-2.384	0.020
ENSG00000199453	SNORD115-12	-0.791	-1.859	-2.052	0.045
ENSG00000199489	SNORD115-25	-0.629	-1.116	-2.160	0.035
ENSG00000199627	RNU6-1010P	-0.667	-1.110	-2.301	0.025
ENSG00000200087	SNORA73B	0.627	3.972	2.064	0.044
ENSG00000200218	RNU6-697P	0.939	-1.822	2.460	0.017
ENSG00000200661	SNORD116-10	-0.741	-1.449	-2.291	0.026
ENSG00000200879	SNORD14E	-1.014	-1.265	-2.136	0.037
ENSG00000200882	RNU6-681P	-0.553	-1.100	-2.213	0.031
ENSG00000200987	SNORD115-30	-0.888	-3.212	-2.050	0.045
ENSG00000201121	RNY1P12	-0.648	-1.988	-2.200	0.032
ENSG00000201326	SNORD115-22	-1.000	-0.157	-3.852	0.000
ENSG00000201679	SNORD115-15	-0.980	-2.455	-2.322	0.024
ENSG00000201852	RNU6-702P	1.499	-4.062	2.270	0.027
ENSG00000202169	Y_RNA	0.761	-1.843	2.025	0.048
ENSG00000202380	RNU1-55P	1.330	-3.880	2.081	0.042
ENSG00000203435	E2F3P2	0.514	0.590	2.312	0.024
ENSG00000204397	CARD16	0.655	-0.320	2.215	0.031
ENSG00000204754	LINC01951	1.173	-3.094	2.208	0.031
ENSG00000205038	PKHD1L1	-1.282	-0.650	-2.260	0.028
ENSG00000205220	PSMB10	0.326	0.448	2.486	0.016
ENSG00000205643	CDPF1	0.371	0.765	2.536	0.014
ENSG00000205885	C1RL-AS1	0.496	1.127	2.277	0.027
ENSG00000205978	NYNRIN	0.333	2.740	2.242	0.029
ENSG00000206262	FOXL2NB	1.116	-1.551	2.986	0.004
ENSG00000206688	SNORD116-18	-0.592	0.220	-2.161	0.035
ENSG00000207001	SNORD116-2	-0.648	-1.026	-2.017	0.048

ENSG00000207954	MIR138-1	-0.666	0.518	-2.028	0.047
ENSG00000210195	MT-TT	0.887	-0.475	2.127	0.038
ENSG00000211445	GPX3	-0.643	3.171	-2.405	0.019
ENSG00000212719	C17orf51	-0.368	5.972	-2.647	0.010
ENSG00000213023	SYT3	-0.415	2.820	-2.313	0.024
ENSG00000213079	SCAF8	0.145	6.848	2.252	0.028
ENSG00000213277	MARCKSL1P1	0.829	-2.252	2.025	0.048
ENSG00000213445	SIPA1	0.534	2.243	3.251	0.002
ENSG00000213513	RP13-39P12.2	-0.662	-1.819	-2.118	0.038
ENSG00000214188	ST7-OT4	0.492	0.662	2.045	0.045
ENSG00000214353	VAC14-AS1	0.465	2.003	2.430	0.018
ENSG00000214517	PPME1	-0.305	5.876	-2.165	0.035
ENSG00000214575	CPEB1	-0.340	4.358	-3.085	0.003
ENSG00000215067	ALOX12-AS1	0.235	2.727	2.030	0.047
ENSG00000215184	RP11-8L18.2	0.655	-0.304	2.344	0.023
ENSG00000215218	UBE2QL1	-0.563	5.861	-2.670	0.010
ENSG00000215388	ACTG1P3	0.387	0.403	2.098	0.040
ENSG00000215788	TNFRSF25	-0.537	2.580	-2.389	0.020
ENSG00000215790	SLC35E2	-0.343	4.860	-2.140	0.037
ENSG00000215802	RFKP1	-0.785	-0.023	-2.642	0.011
ENSG00000216740	ANXA2P3	1.368	-1.298	3.514	0.001
ENSG00000218536	AP002530.2	-0.704	-0.909	-2.023	0.048
ENSG00000219941	KRT18P50	1.392	-1.696	3.806	0.000
ENSG00000221539	SNORD99	-0.606	-1.778	-2.320	0.024
ENSG00000221866	PLXNA4	-0.543	6.960	-2.544	0.014
ENSG00000221882	OR3A2	0.684	0.627	2.143	0.036
ENSG00000222344	RNU6-613P	-0.570	-1.833	-2.214	0.031
ENSG00000223436	AC011625.1	-0.773	-1.470	-2.372	0.021
ENSG00000223583	RP3-476K8.4	0.604	-1.023	2.528	0.014
ENSG00000223839	FAM95B1	-0.991	0.533	-2.082	0.042
ENSG00000223969	AC002456.2	0.668	-1.845	2.038	0.046
ENSG00000224014	RP11-488L18.6	-0.693	-1.520	-2.329	0.023
ENSG00000224081	LINC01057	0.448	2.142	2.148	0.036
ENSG00000224238	WARS2-IT1	0.480	-0.423	2.209	0.031
ENSG00000224536	RP11-134G8.7	0.478	0.215	2.193	0.032
ENSG00000224598	RPS5P2	-0.733	-1.885	-2.201	0.032
ENSG00000224616	RTCA-AS1	0.510	0.393	2.739	0.008
ENSG00000224883	CTA-250D10.19	-0.732	-1.199	-2.259	0.028
ENSG00000225194	LINC00092	0.388	1.280	2.118	0.038
ENSG00000225401	TGIF2P1	-0.894	-2.778	-2.170	0.034
ENSG00000225963	AC009950.2	0.334	0.781	2.257	0.028
ENSG00000226200	SGMS1-AS1	0.275	2.660	2.222	0.030
ENSG00000226323	AC006150.1	-0.797	-1.071	-2.038	0.046
ENSG00000226380	MIR29A	-0.402	4.723	-2.133	0.037
ENSG00000226887	ERVMER34-1	-0.567	-0.432	-2.112	0.039
ENSG00000226904	RP11-561O23.8	-0.623	1.665	-2.253	0.028
ENSG00000227045	RP11-98D18.1	-0.798	-1.758	-2.393	0.020

ENSG00000227742	CALR4P	-0.633	-0.891	-2.018	0.048
ENSG00000227950	RP11-312B8.2	-0.592	-0.887	-2.299	0.025
ENSG00000228127	LINC01649	-0.447	1.071	-2.163	0.035
ENSG00000228133	AC099684.1	-0.736	0.568	-2.230	0.030
ENSG00000228219	NPM1P30	0.420	0.498	2.188	0.033
ENSG00000228280	RP11-367B6.2	0.823	-1.098	2.084	0.042
ENSG00000228657	RPL23AP58	0.762	-1.836	2.308	0.025
ENSG00000228696	ARL17B	-0.745	1.948	-2.188	0.033
ENSG00000228861	RPL23AP12	-0.933	-2.282	-2.524	0.014
ENSG00000229146	SNX18P4	-0.789	0.109	-2.053	0.045
ENSG00000229154	KCNQ5-AS1	-0.924	-0.218	-2.089	0.041
ENSG00000229246	LINC00377	0.831	-1.477	2.057	0.044
ENSG00000229369	RP13-225O21.5	-0.499	-0.457	-2.061	0.044
ENSG00000229403	RP4-718N17.2	1.198	-2.588	2.249	0.028
ENSG00000229539	RP11-119B16.2	-0.425	-1.360	-2.045	0.045
ENSG00000229809	ZNF688	0.285	1.505	2.178	0.034
ENSG00000229852	RP11-398K22.12	-0.275	3.019	-2.530	0.014
ENSG00000229853	RP5-1049G16.4	0.590	-1.282	2.005	0.050
ENSG00000229980	TOB1-AS1	-0.446	0.347	-2.602	0.012
ENSG00000230026	RP11-382D8.5	-0.716	-1.050	-2.112	0.039
ENSG00000230184	SMYD3-IT1	-0.579	-1.171	-2.030	0.047
ENSG00000230303	RP11-213G2.2	-0.414	-0.059	-2.008	0.049
ENSG00000230435	AC004160.4	-0.925	-0.005	-2.004	0.050
ENSG00000230736	RP1-149A16.3	-0.701	-2.042	-2.033	0.047
ENSG00000230772	VN1R108P	-0.507	-1.150	-2.059	0.044
ENSG00000231437	LINC01750	-0.424	1.219	-2.044	0.045
ENSG00000231562	RP11-508N12.3	0.512	-0.394	2.375	0.021
ENSG00000231646	FSIP2-AS1	0.374	0.423	2.275	0.027
ENSG00000231654	RPS6KA2-AS1	1.201	-2.136	2.996	0.004
ENSG00000231793	DOC2GP	0.795	-2.060	2.402	0.020
ENSG00000231995	RP11-111F5.2	-1.203	-0.829	-2.471	0.016
ENSG00000232433	GXYLT1P3	-0.591	3.102	-2.377	0.021
ENSG00000232778	RPL23AP50	-0.536	-1.036	-2.022	0.048
ENSG00000232994	RPL7P14	0.564	-0.418	2.254	0.028
ENSG00000233966	UBE2SP1	-1.062	-0.634	-2.359	0.022
ENSG00000234084	RP3-388E23.2	-0.458	2.204	-2.342	0.023
ENSG00000234303	CLYBL-AS1	1.105	-2.506	2.260	0.028
ENSG00000234377	RNF219-AS1	0.329	4.598	2.342	0.023
ENSG00000235008	RP3-497J21.1	0.604	2.130	2.899	0.005
ENSG00000235363	SNRPGP10	-0.876	-0.055	-2.487	0.016
ENSG00000235833	AC159540.14	-2.089	-0.449	-2.477	0.016
ENSG00000235904	RBMS3-AS3	0.690	-0.257	2.753	0.008
ENSG00000236021	RP11-195C7.1	0.300	1.857	2.099	0.040
ENSG00000236136	ADORA2BP1	1.028	-2.388	2.297	0.025
ENSG00000236259	AC017083.2	-0.853	-2.190	-2.624	0.011
ENSG00000236279	CLEC2L	-0.573	2.790	-2.460	0.017
ENSG00000236397	DDX11L2	0.671	-1.002	2.391	0.020

ENSG00000236494	RP11-89N17.4	0.482	0.041	2.072	0.043
ENSG00000236519	LINC01424	0.597	-1.362	2.082	0.042
ENSG00000236740	RP11-411K7.1	1.265	-4.214	2.126	0.038
ENSG00000236816	ANKRD20A7P	-0.631	3.799	-2.682	0.010
ENSG00000236819	LINC01563	0.361	1.577	2.392	0.020
ENSG00000237065	NANOGP4	-0.556	0.511	-2.154	0.035
ENSG00000237076	RP5-836J3.1	-0.880	-1.895	-2.338	0.023
ENSG00000237090	RP11-342M1.6	-1.021	-2.899	-2.096	0.040
ENSG00000237575	PYY2	1.225	-3.870	2.221	0.030
ENSG00000237854	LINC00674	0.263	2.695	2.329	0.023
ENSG00000237938	RP11-288I21.1	-0.519	0.094	-2.075	0.043
ENSG00000238086	PPP1R26P1	1.034	-0.896	2.960	0.004
ENSG00000238164	RP3-395M20.8	0.696	-1.192	2.010	0.049
ENSG00000239382	ALKBH6	-0.347	1.985	-3.362	0.001
ENSG00000239389	PCDHA13	0.354	1.266	2.127	0.038
ENSG00000239726	RN7SL688P	0.842	-2.612	2.217	0.031
ENSG00000239883	PARGP1	0.210	5.279	2.037	0.046
ENSG00000240065	PSMB9	0.539	1.319	2.362	0.022
ENSG00000240522	RPL7AP10	-0.502	-0.782	-2.435	0.018
ENSG00000240567	LINC02067	1.028	-1.884	2.186	0.033
ENSG00000240764	PCDHGC5	-0.616	5.385	-2.165	0.035
ENSG00000240771	ARHGEF25	-0.484	4.782	-2.358	0.022
ENSG00000241316	SUCLG2-AS1	0.288	2.560	2.098	0.040
ENSG00000241553	ARPC4	-0.275	3.812	-2.131	0.037
ENSG00000241852	C8orf58	0.446	0.628	2.218	0.031
ENSG00000242282	AC108488.4	0.824	-1.632	3.053	0.003
ENSG00000242498	ARPIN	0.201	4.803	2.111	0.039
ENSG00000242551	POU5F1P6	1.105	-1.737	2.795	0.007
ENSG00000242759	LINC00882	-0.257	4.358	-2.076	0.042
ENSG00000243069	ARHGEF26-AS1	0.330	3.517	2.025	0.048
ENSG00000243256	RPL30P14	0.377	0.602	2.095	0.041
ENSG00000243516	RPL12P40	-0.773	-1.298	-2.413	0.019
ENSG00000243742	RPLP0P2	0.953	-0.569	2.177	0.034
ENSG00000244159	RP11-1070A24.1	1.015	-1.031	3.302	0.002
ENSG00000244482	LILRA6	0.838	-1.101	2.265	0.027
ENSG00000246375	RP11-10L7.1	1.146	0.095	2.218	0.031
ENSG00000246528	RP11-159H10.3	-0.697	-1.100	-2.563	0.013
ENSG00000246731	MGC16275	0.384	2.716	2.194	0.032
ENSG00000247131	RP11-588G21.2	-0.651	-1.781	-2.013	0.049
ENSG00000247213	LINC01498	-0.978	-2.760	-2.115	0.039
ENSG00000247287	RP11-902B17.1	0.638	0.930	2.249	0.028
ENSG00000248115	RP11-752D24.2	-0.385	0.798	-2.072	0.043
ENSG00000248210	LINC02355	-0.613	-0.018	-2.463	0.017
ENSG00000248249	RP11-598O12.1	0.594	0.965	2.552	0.013
ENSG00000248323	LUCAT1	0.341	2.294	2.044	0.046
ENSG00000248641	HMGA1P2	-0.830	-0.696	-2.275	0.027
ENSG00000248711	THUMPD3P1	-0.728	-1.289	-2.940	0.005

ENSG00000249049	RP11-763F8.1	-0.554	0.771	-2.318	0.024
ENSG00000249170	RP11-1J11.1	0.634	0.444	2.358	0.022
ENSG00000249242	TMEM150C	-0.263	5.303	-2.230	0.030
ENSG00000249352	LINC02198	0.823	-1.488	2.271	0.027
ENSG00000249588	CTC-537E7.1	1.128	-2.713	2.197	0.032
ENSG00000249631	RP11-281P23.2	1.355	-2.131	2.597	0.012
ENSG00000249695	RP11-598F7.4	-0.670	1.177	-2.410	0.019
ENSG00000249807	CTD-2199O4.1	-0.905	-1.562	-2.493	0.016
ENSG00000250106	ANKRD33B-AS1	-0.818	-1.631	-2.076	0.042
ENSG00000250131	RP11-130F10.1	0.522	0.350	2.165	0.035
ENSG00000250148	KRT8P31	0.733	0.091	2.640	0.011
ENSG00000250155	CTD-2353F22.1	0.519	2.339	2.654	0.010
ENSG00000250254	PTTG2	-0.620	-0.038	-2.192	0.032
ENSG00000250289	VWA8P1	1.007	-3.162	2.269	0.027
ENSG00000250303	RP11-356J5.12	0.296	2.521	2.598	0.012
ENSG00000250328	MGC32805	0.918	-0.956	2.556	0.013
ENSG00000250510	GPR162	-0.326	4.107	-2.105	0.040
ENSG00000250603	CTC-228N24.2	-0.731	-1.481	-2.252	0.028
ENSG00000250899	RP11-253E3.3	0.995	-2.474	2.084	0.042
ENSG00000250974	LINC02196	-1.310	-0.813	-2.149	0.036
ENSG00000251136	RP11-37B2.1	0.221	5.212	2.053	0.045
ENSG00000251381	LINC00958	1.116	-1.076	3.175	0.002
ENSG00000251488	RP11-404I7.2	0.666	-1.796	2.179	0.033
ENSG00000252087	RN7SKP248	-1.274	-2.934	-2.512	0.015
ENSG00000252396	RN7SKP195	-0.907	-0.168	-3.118	0.003
ENSG00000252494	RNU6-126P	-0.632	-2.182	-2.484	0.016
ENSG00000253230	LINC00599	-0.650	5.681	-3.170	0.002
ENSG00000253270	RP11-1105O14.1	0.531	0.012	2.009	0.049
ENSG00000253853	GS1-57L11.1	0.990	-0.760	2.032	0.047
ENSG00000253855	AC133633.1	1.161	-2.840	2.294	0.025
ENSG00000253878	RP11-347C18.3	0.779	-1.632	2.316	0.024
ENSG00000253943	KRT18P37	0.869	-1.714	2.421	0.019
ENSG00000253946	CTC-425K20.1	1.470	-2.435	2.127	0.038
ENSG00000253956	RP11-211F2.1	-0.850	-2.165	-2.022	0.048
ENSG00000254041	RP11-574O7.1	-1.298	-2.555	-2.894	0.005
ENSG00000254054	RP11-156K13.3	0.925	-1.435	2.169	0.034
ENSG00000254080	RP11-730G20.1	-0.655	0.280	-2.469	0.017
ENSG00000254093	PINX1	-0.251	2.107	-2.244	0.029
ENSG00000254126	CD8BP	0.590	0.496	2.122	0.038
ENSG00000254211	LINC01485	0.908	-2.015	2.563	0.013
ENSG00000254396	RP11-56F10.3	1.165	-1.735	2.926	0.005
ENSG00000254423	RP11-351I21.7	1.071	0.513	2.068	0.043
ENSG00000254471	RP11-885L14.1	-0.795	1.897	-2.073	0.043
ENSG00000254485	RP11-380O24.1	0.822	-0.685	2.886	0.005
ENSG00000254506	RP11-748H22.1	0.958	-0.594	2.893	0.005
ENSG00000254743	OR10V3P	1.318	-3.136	2.767	0.008
ENSG00000254758	AP000889.1	-0.621	-0.068	-2.180	0.033

ENSG00000254839	AF131215.6	-0.543	0.037	-2.018	0.048
ENSG00000255234	RP11-727A23.10	-0.571	-1.234	-2.161	0.035
ENSG00000255371	OPCML-IT2	-0.665	1.166	-2.332	0.023
ENSG00000255372	CTD-3064C13.1	-0.590	-0.349	-2.092	0.041
ENSG00000255413	RP11-436P7.2	1.305	-1.830	2.860	0.006
ENSG00000255483	AP000889.2	-0.566	-1.117	-2.048	0.045
ENSG00000255556	RP11-351I21.6	1.032	0.587	2.206	0.031
ENSG00000255660	RERG-AS1	0.791	-2.056	2.307	0.025
ENSG00000255823	MTRNR2L8	-1.119	0.049	-2.538	0.014
ENSG00000256234	RP11-283G6.4	0.470	-0.721	2.108	0.039
ENSG00000256315	AP000462.1	0.309	2.659	2.384	0.020
ENSG00000256596	RP11-522N14.2	-0.411	0.931	-2.024	0.048
ENSG00000256947	RP11-64D24.2	-0.698	-1.140	-2.067	0.043
ENSG00000256972	AP000462.3	0.396	1.204	2.063	0.044
ENSG00000257191	RP11-390N6.1	-0.451	1.239	-2.108	0.039
ENSG00000257497	RP11-585P4.5	0.524	-0.047	2.407	0.019
ENSG00000257647	RP11-701H24.3	-0.391	5.957	-2.062	0.044
ENSG00000257674	RP11-76E16.2	0.753	-1.013	2.539	0.014
ENSG00000257859	CASC18	1.118	-0.926	2.945	0.005
ENSG00000257923	CUX1	0.247	6.076	2.249	0.028
ENSG00000258279	LINC00592	1.679	-4.873	2.592	0.012
ENSG00000258311	RP11-644F5.10	0.557	-0.652	2.112	0.039
ENSG00000258352	RP11-23J18.1	1.140	-2.688	3.224	0.002
ENSG00000258454	RP11-361H10.3	-0.936	-2.264	-2.307	0.025
ENSG00000258679	RP11-74M13.4	-0.765	-0.827	-2.126	0.038
ENSG00000258743	LINC02301	1.032	-0.590	2.590	0.012
ENSG00000258909	RP11-164C12.2	0.729	-1.386	2.178	0.033
ENSG00000259013	RP11-1017G21.3	-0.504	-1.081	-2.250	0.028
ENSG00000259207	ITGB3	0.616	-0.448	2.064	0.044
ENSG00000259271	ANKRD62P1	-0.917	-0.665	-2.563	0.013
ENSG00000259281	LINGO1-AS2	-1.077	-2.450	-2.640	0.011
ENSG00000259291	RP11-617F23.1	0.294	4.556	2.206	0.031
ENSG00000259295	CSPG4P12	-0.819	1.445	-2.016	0.049
ENSG00000259318	RP11-454L9.2	0.396	0.892	2.057	0.044
ENSG00000259425	RP11-566K19.5	-0.442	0.114	-2.047	0.045
ENSG00000259463	RP11-540O11.7	-0.596	-1.212	-2.286	0.026
ENSG00000259592	PRELID1P4	0.530	-1.468	2.005	0.050
ENSG00000259617	RP11-540O11.6	-0.558	-1.213	-2.156	0.035
ENSG00000259751	RP11-83J16.3	0.678	-1.757	2.025	0.048
ENSG00000259804	CTD-2012K14.7	-0.581	-1.191	-2.029	0.047
ENSG00000259846	RP11-467L24.1	0.947	-0.643	2.066	0.043
ENSG00000259920	RP11-2E11.5	0.879	-0.809	2.886	0.005
ENSG00000259940	CTD-3203P2.1	-0.645	-1.949	-2.076	0.042
ENSG00000260144	RP11-361M10.3	0.605	-1.508	2.256	0.028
ENSG00000260281	ITFG1-AS1	-0.491	-1.137	-2.092	0.041
ENSG00000260369	CTD-2526A2.2	1.072	-3.387	2.412	0.019
ENSG00000260372	AQP4-AS1	0.472	3.391	2.785	0.007

ENSG00000260447	RP11-304L19.2	-0.876	-1.411	-2.618	0.011
ENSG00000260604	RP1-140K8.5	0.656	-0.416	2.029	0.047
ENSG00000260992	DOCK9-AS2	-0.659	-0.758	-2.170	0.034
ENSG00000261098	RP11-819C21.1	-0.252	3.861	-2.006	0.050
ENSG00000261115	TMEM178B	-0.381	7.210	-2.546	0.014
ENSG00000261572	RP11-384L8.1	0.776	-0.974	2.218	0.030
ENSG00000261600	RP11-575H3.1	-1.090	-1.506	-2.779	0.007
ENSG00000261633	RP11-405F3.5	0.567	-1.505	2.036	0.046
ENSG00000261684	RP11-265N6.1	-0.693	-1.482	-2.289	0.026
ENSG00000261786	RP4-555D20.2	-0.546	4.774	-2.288	0.026
ENSG00000261837	RP11-264L1.3	0.645	-0.157	2.463	0.017
ENSG00000262681	RP11-311F12.1	-0.935	-1.474	-2.840	0.006
ENSG00000262966	RP11-85B7.2	-0.593	-0.375	-2.160	0.035
ENSG00000263146	LINC01896	0.477	2.124	3.404	0.001
ENSG00000263327	TAPT1-AS1	0.275	3.327	2.218	0.031
ENSG00000263753	LINC00667	0.237	5.360	2.346	0.022
ENSG00000263990	CTC-542B22.2	-0.402	0.116	-2.283	0.026
ENSG00000264727	RP11-680C21.1	-0.745	0.901	-2.046	0.045
ENSG00000265043	RP11-728E14.3	-1.066	-0.626	-3.259	0.002
ENSG00000265265	RP11-822E23.7	-0.715	-1.012	-2.296	0.025
ENSG00000265452	MIR3682	0.425	0.224	2.380	0.021
ENSG00000265630	GLUD1P8	0.734	-1.531	2.022	0.048
ENSG00000265666	RARA-AS1	0.658	-1.210	2.312	0.024
ENSG00000265702	RP11-156L14.1	0.679	-1.118	2.269	0.027
ENSG00000266490	CTD-2349P21.9	-0.292	1.776	-2.080	0.042
ENSG00000266578	RP11-838N2.5	0.920	-1.615	2.655	0.010
ENSG00000266865	RP11-848P1.9	0.244	1.276	2.111	0.039
ENSG00000266994	RP11-127I20.7	-0.978	-2.571	-2.451	0.017
ENSG00000267091	CTBP2P7	-0.794	-1.913	-2.005	0.050
ENSG00000267213	AC007773.2	-0.551	-0.988	-2.293	0.026
ENSG00000267216	AC010642.1	-0.358	0.482	-2.189	0.033
ENSG00000267353	CTD-2162K18.3	-0.732	-1.606	-2.251	0.028
ENSG00000267592	CTC-507E2.2	0.845	-2.351	2.289	0.026
ENSG00000268070	AC006539.3	-0.660	-1.726	-2.513	0.015
ENSG00000268231	CTD-2560K21.6	-0.643	-1.385	-2.188	0.033
ENSG00000268455	RP11-359H18.2	-0.757	-0.779	-2.090	0.041
ENSG00000268659	RP11-310J24.3	0.729	-1.088	2.372	0.021
ENSG00000268945	CTD-2192J16.26	-1.076	-2.815	-2.041	0.046
ENSG00000269028	MTRNR2L12	-1.377	0.840	-2.139	0.037
ENSG00000269526	ERVV-1	-1.967	-2.469	-3.891	0.000
ENSG00000269925	RP3-467L1.6	-0.465	-0.089	-2.100	0.040
ENSG00000269945	RP6-91H8.5	-0.885	0.449	-2.192	0.032
ENSG00000270001	RP11-218C14.8	0.640	-1.640	2.103	0.040
ENSG00000270098	LDHAL6EP	0.787	-0.221	2.778	0.007
ENSG00000270112	RP11-742D12.2	-0.391	2.892	-2.629	0.011
ENSG00000270179	RP11-159N11.4	0.920	-1.126	3.226	0.002
ENSG00000270300	PHACTR2P1	0.369	0.903	2.032	0.047

ENSG00000270343	UNGP3	1.026	-1.102	3.262	0.002
ENSG00000270387	RP11-315D13.1	-0.880	-1.275	-2.125	0.038
ENSG00000270634	RP4-777G9.1	-0.796	-2.039	-2.081	0.042
ENSG00000270959	LPP-AS2	0.505	1.251	2.616	0.011
ENSG00000271447	MMP28	0.346	2.154	2.108	0.039
ENSG00000271797	CTC-428G20.6	0.701	-2.092	2.032	0.047
ENSG00000271947	RP11-439M11.1	1.067	-1.599	2.098	0.040
ENSG00000271978	RP11-428J1.4	0.310	0.787	2.027	0.047
ENSG00000272156	RP11-477N3.1	0.418	-0.715	2.167	0.034
ENSG00000272186	RP11-110I1.13	0.952	-2.576	2.024	0.048
ENSG00000272189	RP3-325F22.5	-0.840	1.035	-2.631	0.011
ENSG00000272269	RP11-500C11.3	-0.730	-2.486	-2.181	0.033
ENSG00000272279	RP11-157J24.2	1.114	-1.256	3.379	0.001
ENSG00000272375	RP11-51J9.6	-0.445	1.156	-2.010	0.049
ENSG00000272447	RP11-182L21.6	-0.427	0.633	-2.269	0.027
ENSG00000272518	RP11-26J3.3	0.304	1.740	2.291	0.026
ENSG00000272588	RP11-440L14.4	0.619	-0.542	2.046	0.045
ENSG00000272668	RP11-190A12.8	0.538	3.189	2.372	0.021
ENSG00000272689	RP4-539M6.21	0.662	-1.433	2.256	0.028
ENSG00000272734	ADIRF-AS1	0.594	0.075	2.337	0.023
ENSG00000272754	AL133245.2	0.699	-1.665	2.308	0.025
ENSG00000272844	RP11-484K9.4	-0.906	-2.129	-2.381	0.021
ENSG00000272853	RP11-398C13.6	-0.690	-1.178	-2.489	0.016
ENSG00000273079	GRIN2B	-0.554	9.819	-2.282	0.026
ENSG00000273271	AP000254.8	-0.373	0.499	-2.092	0.041
ENSG00000273368	RP11-376P6.3	0.742	-1.530	2.770	0.008
ENSG00000273771	RP11-236L14.2	0.711	-0.767	2.292	0.026
ENSG00000274020	LINC01138	0.311	2.820	2.141	0.036
ENSG00000274127	uc_338	-0.474	-0.348	-2.013	0.049
ENSG00000274441	CTD-2282P23.2	0.537	1.231	2.701	0.009
ENSG00000274677	RP11-505K9.5	-0.721	-1.077	-2.332	0.023
ENSG00000274718	RP11-346C4.3	-0.545	2.501	-2.228	0.030
ENSG00000274884	AL117190.1	-0.791	-2.488	-2.063	0.044
ENSG00000274893	RP11-15E1.5	-1.076	0.971	-2.774	0.007
ENSG00000275026	GXYLT1P4	-0.651	2.135	-2.195	0.032
ENSG00000275144	SNX18P5	-0.816	-0.250	-2.193	0.032
ENSG00000275160	RP11-187C18.3	-0.494	2.291	-2.891	0.005
ENSG00000275465	RP11-569G13.3	0.872	0.209	3.099	0.003
ENSG00000275620	FLJ16779	0.702	2.765	2.706	0.009
ENSG00000275763	C18orf65	0.823	-2.074	2.242	0.029
ENSG00000275839	CTC-421K24.1	-0.644	-0.669	-2.989	0.004
ENSG00000275880	RP11-90L1.8	-0.498	-1.328	-2.305	0.025
ENSG00000276128	RP11-459O16.8	-0.754	2.434	-3.151	0.003
ENSG00000276309	RN7SKP251	1.111	-1.674	2.311	0.024
ENSG00000276393	RP11-106D4.3	-0.931	-1.306	-2.769	0.008
ENSG00000276591	GXYLT1P5	-0.746	2.288	-3.074	0.003
ENSG00000276772	CTD-2515H24.2	0.944	-1.534	2.525	0.014

ENSG00000276952	RP5-965G21.6	-0.597	-0.522	-2.502	0.015
ENSG00000277233	Metazoa_SRP	0.653	-2.108	2.225	0.030
ENSG00000277692	RP11-358N2.2	-0.506	-0.568	-2.365	0.021
ENSG00000278156	TSC22D1-AS1	-0.388	2.431	-2.532	0.014
ENSG00000278175	GLIDR	-0.422	1.815	-2.169	0.034
ENSG00000278254	RP4-668E10.4	0.495	1.005	2.178	0.034
ENSG00000278396	RP11-120E11.2	-0.568	-1.314	-2.209	0.031
ENSG00000278464	RP11-96A15.1	0.663	-0.467	2.481	0.016
ENSG00000278891	RP11-246E12.2	0.786	-1.318	2.101	0.040
ENSG00000278898	CTD-2342N23.1	1.017	-2.274	2.021	0.048
ENSG00000279128	RP11-76C10.4	1.046	-2.783	2.127	0.038
ENSG00000279138	KB-1742H10.3	-0.655	2.947	-2.348	0.022
ENSG00000279189	RP11-861E23.2	-0.621	3.064	-2.297	0.025
ENSG00000279276	CTA-345G4.1	-0.999	-2.524	-2.062	0.044
ENSG00000279300	CTD-3149D2.2	-1.078	-1.085	-2.883	0.006
ENSG00000279304	RP11-573M3.6	0.919	-2.620	2.045	0.045
ENSG00000279332	RP11-799B12.1	-0.943	-0.741	-2.893	0.005
ENSG00000279354	RP11-776B3.1	-0.568	-1.157	-2.059	0.044
ENSG00000279372	RP11-1182P23.5	0.878	-2.130	2.523	0.014
ENSG00000279400	CTD-2353F22.2	0.457	4.464	2.387	0.020
ENSG00000279693	RP11-71L14.3	-0.513	0.412	-2.093	0.041
ENSG00000279708	CTD-2335A18.2	-1.064	-3.427	-2.073	0.043
ENSG00000279910	RP11-113E21.2	-0.685	-0.120	-2.175	0.034
ENSG00000280007	AC008079.10	-0.449	3.245	-2.225	0.030
ENSG00000280047	CTC-463A16.1	-0.369	2.343	-2.570	0.013
ENSG00000280053	RP11-61K12.2	-0.576	-0.395	-2.010	0.049
ENSG00000280087	CTB-129P6.7	0.815	-0.842	2.695	0.009
ENSG00000280088	RP11-135F9.3	-0.323	3.111	-2.067	0.043
ENSG00000280098	AC011155.1	-0.759	-1.119	-2.747	0.008
ENSG00000280099	RP11-33E12.2	-0.530	0.228	-2.061	0.044
ENSG00000280103	AC007792.1	-0.898	-2.253	-2.209	0.031
ENSG00000280157	RP11-520H14.7	-0.396	1.891	-2.302	0.025
ENSG00000280169	RP11-151A6.6	0.931	-2.135	3.037	0.004
ENSG00000280255	RP5-1007F24.1	-0.663	2.774	-2.199	0.032
ENSG00000280291	RP11-759A24.2	0.804	-0.634	2.027	0.047
ENSG00000280294	RP11-177H2.1	-0.325	2.688	-2.025	0.047
ENSG00000280304	CTD-2536I1.2	-0.561	1.253	-2.642	0.011
ENSG00000280415	RP4-800O15.2	1.337	-3.181	2.491	0.016
ENSG00000280422	RP11-155D18.13	-0.652	-0.005	-2.543	0.014
ENSG00000280435	RP4-555D20.1	-0.574	2.420	-2.585	0.012
ENSG00000281181	CH507-513H4.3	-1.750	2.805	-2.102	0.040
ENSG00000281183	NPTN-IT1	-0.330	3.935	-2.356	0.022
ENSG00000281383	CH507-513H4.5	-1.828	2.340	-2.219	0.030
ENSG00000281721	LINC01080	0.586	0.982	2.444	0.018
ENSG00000282997	RP11-95C14.1	0.524	0.460	2.368	0.021
ENSG00000283097	RP11-165N12.2	0.551	2.179	2.269	0.027
ENSG00000283341	RP11-472I20.4	-0.351	1.361	-2.113	0.039

ENSG00000283415	RP11-413N10.3	-0.545	0.988	-2.089	0.041
ENSG00000283646	LINC02009	1.392	-2.476	2.008	0.049
ENSG00000283757	#N/A	0.503	1.005	3.087	0.003
ENSG00000284677	#N/A	-0.646	-0.812	-2.032	0.047
ENSG00000285106	#N/A	-0.516	3.261	-2.164	0.035
ENSG00000285287	#N/A	-0.849	-1.044	-2.998	0.004
ENSG00000285554	#N/A	0.795	-1.844	2.246	0.029
ENSG00000285601	#N/A	-0.708	-1.162	-2.265	0.027
ENSG00000285619	#N/A	0.814	-1.859	2.295	0.025
ENSG00000285652	#N/A	-0.711	-0.436	-2.337	0.023
ENSG00000285663	#N/A	0.582	-0.806	2.002	0.050
ENSG00000285669	#N/A	-0.272	4.171	-2.002	0.050
ENSG00000285673	#N/A	0.611	-1.772	2.053	0.045
ENSG00000285728	#N/A	-0.368	-0.041	-2.228	0.030
ENSG00000285729	#N/A	0.562	-0.580	2.193	0.032
ENSG00000285851	#N/A	0.459	0.532	2.687	0.009
ENSG00000285867	#N/A	0.617	0.543	2.617	0.011
ENSG00000285909	#N/A	-0.695	-0.182	-3.119	0.003
ENSG00000285938	AC072022.2	0.511	1.215	2.305	0.025
ENSG00000285991	#N/A	-0.332	1.045	-2.148	0.036

Supplementary Table 6_list of DEGs in women

AntIns

gene_id	gene_name	logFC	AveExpr	t	P.Value
ENSG00000003436	TFPI	0.346	1.960	2.000	0.049
ENSG00000004779	NDUFAB1	0.236	5.230	1.998	0.049
ENSG00000005059	CCDC109B	0.398	2.355	2.210	0.030
ENSG00000005100	DHX33	-0.126	4.884	-2.254	0.027
ENSG00000005339	CREBBP	-0.215	6.869	-2.478	0.015
ENSG00000005469	CROT	0.230	4.114	2.133	0.036
ENSG00000005700	IBTK	0.202	6.732	2.082	0.040
ENSG00000006125	AP2B1	-0.156	8.560	-2.118	0.037
ENSG00000006128	TAC1	0.625	2.864	1.996	0.049
ENSG00000006432	MAP3K9	-0.216	6.760	-2.296	0.024
ENSG00000006576	PHTF2	0.201	5.565	2.238	0.028
ENSG00000006625	GGCT	0.475	4.104	2.680	0.009
ENSG00000007062	PROM1	0.494	2.847	2.598	0.011
ENSG00000008083	JARID2	-0.137	5.123	-2.324	0.023
ENSG00000008118	CAMK1G	-0.269	5.459	-2.198	0.031
ENSG00000009844	VTA1	0.338	6.294	2.236	0.028
ENSG00000011021	CLCN6	-0.215	6.039	-2.742	0.007
ENSG00000016391	CHDH	-0.264	3.979	-2.026	0.046
ENSG00000018510	AGPS	0.249	6.111	2.103	0.038
ENSG00000020129	NCDN	-0.354	7.893	-2.320	0.023
ENSG00000020426	MNAT1	0.196	5.545	2.057	0.043
ENSG00000021574	SPAST	0.275	6.128	2.617	0.011
ENSG00000023287	RB1CC1	0.241	8.708	1.996	0.049
ENSG00000023608	SNAPC1	0.267	2.639	2.035	0.045
ENSG00000028203	VEZT	0.209	7.575	2.276	0.025
ENSG00000029153	ARNTL2	0.282	5.749	2.372	0.020
ENSG00000029534	ANK1	-0.308	4.488	-2.068	0.042
ENSG00000029725	RABEP1	0.194	8.159	2.091	0.040
ENSG00000031691	CENPQ	0.342	2.137	2.022	0.046
ENSG00000032219	ARID4A	0.238	7.138	2.274	0.026
ENSG00000033178	UBA6	0.232	6.726	2.400	0.019
ENSG00000033867	SLC4A7	0.366	7.133	2.641	0.010
ENSG00000035141	FAM136A	0.284	3.780	2.095	0.039
ENSG00000035687	ADSS	0.291	5.789	2.298	0.024
ENSG00000036672	USP2	-0.288	4.078	-2.230	0.028
ENSG00000038002	AGA	0.321	3.203	2.075	0.041
ENSG00000038210	PI4K2B	0.324	1.788	2.537	0.013
ENSG00000038532	CLEC16A	-0.169	5.655	-2.089	0.040
ENSG00000039139	DNAH5	0.230	4.029	2.376	0.020
ENSG00000039319	ZFYVE16	0.237	7.365	2.096	0.039
ENSG00000041357	PSMA4	0.258	5.746	2.028	0.046
ENSG00000041515	MYO16	0.274	4.831	2.237	0.028
ENSG00000047410	TPR	0.188	8.065	2.197	0.031
ENSG00000047621	C12orf4	0.240	5.655	2.002	0.049

ENSG00000047936	ROS1	-0.697	3.053	-2.501	0.014
ENSG00000048140	TSPAN17	0.270	3.998	2.605	0.011
ENSG00000048544	MRPS10	0.267	5.095	2.002	0.049
ENSG00000049192	ADAMTS6	0.411	2.704	2.870	0.005
ENSG00000049246	PER3	-0.160	6.001	-2.011	0.048
ENSG00000049618	ARID1B	-0.143	7.421	-2.293	0.024
ENSG00000050165	DKK3	-0.234	8.747	-2.969	0.004
ENSG00000052802	MSMO1	0.308	5.898	2.219	0.029
ENSG00000053108	FSTL4	-0.419	4.794	-3.226	0.002
ENSG00000053438	NNAT	0.351	5.899	2.584	0.012
ENSG00000053524	MCF2L2	-0.150	6.886	-2.277	0.025
ENSG00000054282	SDCCAG8	0.246	6.896	2.344	0.021
ENSG00000054523	KIF1B	-0.120	10.055	-2.020	0.047
ENSG00000056050	HPF1	0.370	3.378	2.355	0.021
ENSG00000059769	DNAJC25	0.283	3.459	2.751	0.007
ENSG00000061794	MRPS35	0.296	5.661	2.178	0.032
ENSG00000062194	GPBP1	0.203	7.572	2.222	0.029
ENSG00000062485	CS	-0.144	6.124	-2.111	0.038
ENSG00000064309	CDON	-0.197	4.354	-1.991	0.050
ENSG00000064313	TAF2	0.173	5.524	1.991	0.050
ENSG00000064726	BTBD1	0.320	7.097	2.202	0.030
ENSG00000064886	CHI3L2	-1.349	-1.536	-2.344	0.022
ENSG00000064933	PMS1	0.234	5.180	2.260	0.026
ENSG00000064989	CALCRL	0.292	4.282	2.030	0.046
ENSG00000065060	UHRF1BP1	-0.122	5.211	-2.186	0.032
ENSG00000065548	ZC3H15	0.283	7.091	2.250	0.027
ENSG00000065911	MTHFD2	0.475	1.661	2.610	0.011
ENSG00000065978	YBX1	0.282	5.483	2.655	0.010
ENSG00000065989	PDE4A	-0.479	4.846	-4.019	0.000
ENSG00000066557	LRRC40	0.272	5.591	2.010	0.048
ENSG00000066735	KIF26A	0.633	0.114	2.991	0.004
ENSG00000067064	IDI1	0.371	6.543	2.528	0.013
ENSG00000067900	ROCK1	0.179	6.636	2.021	0.047
ENSG00000068885	IFT80	0.307	5.151	2.091	0.040
ENSG00000069869	NEDD4	0.267	3.501	2.864	0.005
ENSG00000070081	NUCB2	0.254	5.703	2.524	0.013
ENSG00000070501	POLB	0.262	3.863	2.368	0.020
ENSG00000071073	MGAT4A	0.194	6.827	2.090	0.040
ENSG00000071205	ARHGAP10	0.675	5.130	3.894	0.000
ENSG00000071994	PDCD2	0.212	4.968	2.604	0.011
ENSG00000072042	RDH11	0.292	6.379	2.913	0.005
ENSG00000072062	PRKACA	-0.268	5.455	-2.591	0.011
ENSG00000072182	ASIC4	0.369	1.437	2.229	0.028
ENSG00000072736	NFATC3	-0.167	4.925	-2.039	0.045
ENSG00000072756	TRNT1	0.219	4.753	2.297	0.024
ENSG00000073734	ABCB11	-0.525	-0.850	-2.044	0.044
ENSG00000074054	CLASP1	-0.098	7.856	-2.215	0.029

ENSG00000074211	PPP2R2C	-0.178	7.646	-2.340	0.022
ENSG00000074410	CA12	-0.749	1.590	-2.550	0.013
ENSG00000075073	TACR2	-0.302	1.632	-2.602	0.011
ENSG00000075223	SEMA3C	0.414	4.790	2.744	0.007
ENSG00000075239	ACAT1	0.220	5.371	2.051	0.043
ENSG00000075292	ZNF638	0.171	8.744	2.504	0.014
ENSG00000075413	MARK3	0.097	6.961	2.021	0.047
ENSG00000075785	RAB7A	-0.227	6.842	-3.286	0.001
ENSG00000076043	REXO2	0.201	3.991	2.526	0.013
ENSG00000076108	BAZ2A	-0.204	6.055	-2.163	0.033
ENSG00000076555	ACACB	-0.265	5.018	-2.034	0.045
ENSG00000077380	DYNC1I2	0.286	6.420	2.685	0.009
ENSG00000077935	SMC1B	0.520	-0.585	2.550	0.013
ENSG00000078674	PCM1	0.199	9.068	2.470	0.016
ENSG00000078699	CBFA2T2	-0.182	5.607	-2.703	0.008
ENSG00000079819	EPB41L2	0.127	8.297	2.114	0.038
ENSG00000080200	CRYBG3	0.250	6.051	2.218	0.029
ENSG00000080608	PUM3	0.201	5.521	2.209	0.030
ENSG00000081087	OSTM1	0.295	5.236	2.412	0.018
ENSG00000081248	CACNA1S	0.517	0.192	2.333	0.022
ENSG00000081818	PCDHB4	-0.369	3.749	-2.649	0.010
ENSG00000082898	XPO1	0.196	7.510	2.056	0.043
ENSG00000083290	ULK2	0.227	6.827	3.033	0.003
ENSG00000083312	TNPO1	0.182	7.370	2.081	0.041
ENSG00000083457	ITGAE	0.309	1.761	2.472	0.016
ENSG00000083535	PIBF1	0.240	5.279	2.112	0.038
ENSG00000083937	CHMP2B	0.286	5.544	2.085	0.040
ENSG00000084112	SSH1	-0.175	5.884	-2.279	0.025
ENSG00000084764	MAPRE3	-0.222	5.936	-2.299	0.024
ENSG00000086061	DNAJA1	0.245	7.954	2.175	0.032
ENSG00000086666	ZFAND6	0.267	5.133	2.335	0.022
ENSG00000086967	MYBPC2	0.868	-1.730	2.400	0.019
ENSG00000087191	PSMC5	-0.280	5.180	-3.176	0.002
ENSG00000087903	RFX2	-0.263	2.402	-2.015	0.047
ENSG00000088179	PTPN4	0.243	7.801	2.039	0.045
ENSG00000088205	DDX18	0.182	5.924	2.011	0.048
ENSG00000088367	EPB41L1	-0.255	8.219	-3.155	0.002
ENSG00000088387	DOCK9	0.169	8.184	2.863	0.005
ENSG00000088882	CPXM1	1.088	-2.170	2.640	0.010
ENSG00000089009	RPL6	0.330	6.238	2.031	0.046
ENSG00000089169	RPH3A	-0.289	7.452	-2.510	0.014
ENSG00000089335	ZNF302	0.207	5.856	2.016	0.047
ENSG00000089356	FXYD3	-0.743	-1.940	-2.487	0.015
ENSG00000090020	SLC9A1	-0.465	4.137	-3.442	0.001
ENSG00000090263	MRPS33	0.290	4.643	2.664	0.009
ENSG00000090266	NDUFB2	0.327	4.239	2.116	0.037
ENSG00000090520	DNAJB11	0.305	4.126	3.127	0.002

ENSG00000090621	PABPC4	0.147	5.197	2.294	0.024
ENSG00000090674	MCOLN1	-0.288	2.075	-2.045	0.044
ENSG00000090857	PDPR	-0.368	4.813	-2.392	0.019
ENSG00000090863	GLG1	-0.133	7.818	-2.150	0.034
ENSG00000090905	TNRC6A	-0.141	7.409	-2.198	0.031
ENSG00000091127	PUS7	0.193	4.457	2.177	0.032
ENSG00000091490	SEL1L3	0.194	5.215	2.109	0.038
ENSG00000092068	SLC7A8	-0.207	4.807	-2.464	0.016
ENSG00000092108	SCFD1	0.262	5.783	2.133	0.036
ENSG00000092140	G2E3	0.226	4.899	2.009	0.048
ENSG00000092208	GEMIN2	0.265	2.788	2.190	0.031
ENSG00000092847	AGO1	-0.133	7.203	-2.846	0.006
ENSG00000094804	CDC6	0.379	2.790	2.798	0.006
ENSG00000095209	TMEM38B	0.250	3.269	2.192	0.031
ENSG00000095564	BTAF1	0.180	6.893	2.235	0.028
ENSG00000095794	CREM	0.227	4.635	2.285	0.025
ENSG00000096070	BRPF3	-0.269	5.745	-2.712	0.008
ENSG00000096872	IFT74	0.377	4.196	2.748	0.007
ENSG00000097096	SYDE2	0.233	3.720	2.346	0.021
ENSG00000099260	PALMD	0.405	4.063	2.917	0.005
ENSG00000099365	STX1B	-0.310	6.999	-2.448	0.016
ENSG00000099377	HSD3B7	-0.501	-0.558	-2.038	0.045
ENSG00000099995	SF3A1	-0.209	5.693	-2.722	0.008
ENSG00000100302	RASD2	-0.472	3.697	-2.464	0.016
ENSG00000100350	FOXRED2	-0.279	4.796	-2.396	0.019
ENSG00000100354	TNRC6B	-0.112	8.324	-2.256	0.027
ENSG00000100364	KIAA0930	-0.217	6.698	-2.129	0.036
ENSG00000100442	FKBP3	0.264	5.538	2.056	0.043
ENSG00000100478	AP4S1	0.177	5.330	2.250	0.027
ENSG00000100567	PSMA3	0.205	5.251	2.328	0.022
ENSG00000100744	GSKIP	0.317	4.613	2.122	0.037
ENSG00000100883	SRP54	0.262	5.756	2.134	0.036
ENSG00000100982	PCIF1	-0.444	3.020	-3.108	0.003
ENSG00000101096	NFATC2	-0.326	2.642	-2.070	0.042
ENSG00000101126	ADNP	-0.107	7.207	-2.196	0.031
ENSG00000101132	PFDN4	0.352	4.712	2.005	0.048
ENSG00000101189	MRGBP	0.216	2.742	2.425	0.017
ENSG00000101236	RNF24	-0.179	5.525	-2.583	0.012
ENSG00000101251	SEL1L2	0.901	-2.019	2.471	0.016
ENSG00000101307	SIRPB1	-0.686	1.665	-2.338	0.022
ENSG00000101337	TM9SF4	-0.195	5.750	-2.539	0.013
ENSG00000101343	CRNKL1	0.175	6.117	1.996	0.049
ENSG00000101363	MANBAL	-0.236	3.376	-2.344	0.021
ENSG00000101596	SMCHD1	0.200	7.122	2.097	0.039
ENSG00000101624	CEP76	0.247	3.601	2.430	0.017
ENSG00000102595	UGGT2	0.257	6.568	2.461	0.016
ENSG00000102898	NUTF2	0.164	5.430	2.571	0.012

ENSG00000103018	CYB5B	0.216	6.352	2.214	0.030
ENSG00000103023	PRSS54	-0.680	-2.474	-2.232	0.028
ENSG00000103061	SLC7A6OS	0.221	2.351	2.485	0.015
ENSG00000103460	TOX3	0.297	3.244	2.159	0.034
ENSG00000103494	RPGRIP1L	0.239	5.780	2.482	0.015
ENSG00000103512	NOMO1	-0.255	5.963	-3.053	0.003
ENSG00000103522	IL21R	-0.986	-1.917	-2.755	0.007
ENSG00000103642	LACTB	0.230	3.771	2.191	0.031
ENSG00000103647	CORO2B	-0.198	6.559	-2.828	0.006
ENSG00000103832	GOLGA8UP	-0.667	-1.662	-2.419	0.018
ENSG00000104131	EIF3J	0.232	5.449	2.524	0.014
ENSG00000104164	BLOC1S6	0.279	6.992	2.179	0.032
ENSG00000104218	CSPP1	0.199	5.843	2.732	0.008
ENSG00000104320	NBN	0.225	6.480	2.116	0.037
ENSG00000104327	CALB1	0.493	5.853	2.348	0.021
ENSG00000104343	UBE2W	0.317	7.049	2.377	0.020
ENSG00000104408	EIF3E	0.324	6.040	2.285	0.025
ENSG00000104412	EMC2	0.225	5.717	2.011	0.048
ENSG00000104435	STMN2	0.309	8.998	2.055	0.043
ENSG00000104549	SQLE	0.277	5.618	2.357	0.021
ENSG00000104613	INTS10	0.128	5.940	2.013	0.047
ENSG00000104691	UBXN8	0.247	3.322	2.029	0.046
ENSG00000104765	BNIP3L	0.253	7.711	2.157	0.034
ENSG00000104853	CLPTM1	-0.270	6.120	-2.666	0.009
ENSG00000104888	SLC17A7	-0.322	7.455	-2.501	0.014
ENSG00000104967	NOVA2	-0.372	5.484	-2.508	0.014
ENSG00000105085	MED26	-0.320	1.596	-2.501	0.014
ENSG00000105270	CLIP3	-0.373	6.744	-2.432	0.017
ENSG00000105289	TJP3	0.650	-0.621	2.373	0.020
ENSG00000105419	MEIS3	0.299	5.100	2.079	0.041
ENSG00000105438	KDELR1	-0.222	4.178	-2.171	0.033
ENSG00000105576	TNPO2	-0.268	6.564	-2.240	0.028
ENSG00000105662	CRTC1	-0.350	5.110	-2.093	0.039
ENSG00000105699	LSR	0.423	1.522	2.785	0.007
ENSG00000105849	TWISTNB	0.243	4.820	2.140	0.035
ENSG00000105875	WDR91	-0.193	3.974	-1.999	0.049
ENSG00000105894	PTN	0.363	7.171	2.646	0.010
ENSG00000105971	CAV2	0.266	3.234	2.045	0.044
ENSG00000106078	COBL	-0.184	7.135	-2.560	0.012
ENSG00000106080	FKBP14	0.201	4.034	2.539	0.013
ENSG00000106113	CRHR2	-0.503	0.820	-2.446	0.017
ENSG00000106125	FAM188B	-0.413	0.854	-2.508	0.014
ENSG00000106245	BUD31	0.244	3.945	2.230	0.028
ENSG00000106355	LSM5	0.206	3.766	1.991	0.050
ENSG00000106443	PHF14	0.222	7.780	2.308	0.024
ENSG00000106554	CHCHD3	0.191	4.931	2.001	0.049
ENSG00000106571	GLI3	-0.422	4.036	-2.823	0.006

ENSG00000106591	MRPL32	0.236	4.669	2.453	0.016
ENSG00000106733	NMRK1	0.405	3.700	2.537	0.013
ENSG00000106993	CDC37L1	0.274	5.732	2.198	0.031
ENSG00000107249	GLIS3	-0.335	4.103	-2.509	0.014
ENSG00000107262	BAG1	0.221	4.647	2.038	0.045
ENSG00000107625	DDX50	0.238	5.211	2.384	0.019
ENSG00000107672	NSMCE4A	0.192	3.218	2.018	0.047
ENSG00000107742	SPOCK2	-0.224	7.978	-2.137	0.036
ENSG00000107949	BCCIP	0.208	4.996	2.852	0.005
ENSG00000107960	OBFC1	0.193	3.388	2.028	0.046
ENSG00000108055	SMC3	0.219	6.261	2.238	0.028
ENSG00000108306	FBXL20	-0.131	6.941	-2.539	0.013
ENSG00000108349	CASC3	-0.174	6.457	-2.306	0.024
ENSG00000108423	TUBD1	0.202	2.424	2.108	0.038
ENSG00000108561	C1QBP	0.318	5.545	2.698	0.008
ENSG00000108797	CNTNAP1	-0.284	6.514	-2.445	0.017
ENSG00000108960	MMD	0.565	6.800	3.651	0.000
ENSG00000109046	WSB1	0.265	7.506	2.373	0.020
ENSG00000109061	MYH1	-1.170	-1.839	-2.351	0.021
ENSG00000109111	SUPT6H	-0.153	6.901	-2.193	0.031
ENSG00000109118	PHF12	-0.181	4.595	-2.225	0.029
ENSG00000109255	NMU	0.866	-0.719	2.266	0.026
ENSG00000109475	RPL34	0.426	6.352	2.143	0.035
ENSG00000109576	AADAT	0.210	3.882	2.440	0.017
ENSG00000109689	STIM2	0.150	5.683	2.026	0.046
ENSG00000109758	HGFAC	0.992	-2.234	2.364	0.020
ENSG00000109814	UGDH	0.203	3.709	2.641	0.010
ENSG00000109881	CCDC34	0.265	3.618	2.257	0.027
ENSG00000109906	ZBTB16	-0.319	5.655	-2.115	0.037
ENSG00000109929	SC5D	0.256	6.552	2.059	0.043
ENSG00000110092	CCND1	0.250	4.332	2.051	0.043
ENSG00000110107	PRPF19	-0.254	5.977	-2.514	0.014
ENSG00000110237	ARHGEF17	-0.304	5.629	-2.208	0.030
ENSG00000110395	CBL	-0.121	6.355	-2.088	0.040
ENSG00000110723	EXPH5	0.305	4.622	2.233	0.028
ENSG00000110768	GTF2H1	0.229	5.857	2.082	0.040
ENSG00000111142	METAP2	0.171	6.135	2.048	0.044
ENSG00000111229	ARPC3	0.386	5.501	2.776	0.007
ENSG00000111231	GPN3	0.303	3.203	2.096	0.039
ENSG00000111254	AKAP3	0.405	1.221	2.106	0.038
ENSG00000111266	DUSP16	-0.181	5.551	-2.586	0.011
ENSG00000111540	RAB5B	-0.179	7.573	-2.884	0.005
ENSG00000111846	GCNT2	0.252	2.634	2.017	0.047
ENSG00000111850	SMIM8	0.190	4.909	2.015	0.047
ENSG00000111907	TPD52L1	-0.381	4.779	-2.203	0.030
ENSG00000111911	HINT3	0.242	6.339	2.118	0.037
ENSG00000112031	MTRF1L	0.264	3.534	2.287	0.025

ENSG00000112038	OPRM1	-0.408	3.774	-2.745	0.007
ENSG00000112182	BACH2	-0.198	3.841	-2.255	0.027
ENSG00000112210	RAB23	0.273	4.733	2.174	0.033
ENSG00000112280	COL9A1	0.592	2.164	3.910	0.000
ENSG00000112339	HBS1L	0.222	6.930	2.270	0.026
ENSG00000112414	ADGRG6	0.406	2.487	2.030	0.046
ENSG00000112419	PHACTR2	0.235	6.331	2.299	0.024
ENSG00000112559	MDFI	0.608	-0.965	2.607	0.011
ENSG00000112624	GLTSCR1L	-0.151	6.113	-2.488	0.015
ENSG00000112706	IMPG1	0.342	1.560	2.013	0.047
ENSG00000112739	PRPF4B	0.183	6.890	2.250	0.027
ENSG00000112972	HMGCS1	0.414	7.660	2.882	0.005
ENSG00000113013	HSPA9	0.212	8.040	2.008	0.048
ENSG00000113211	PCDHB6	-0.263	2.074	-2.098	0.039
ENSG00000113240	CLK4	0.197	6.156	2.110	0.038
ENSG00000113356	POLR3G	0.215	3.576	2.099	0.039
ENSG00000113387	SUB1	0.445	8.309	2.316	0.023
ENSG00000113389	NPR3	0.524	2.104	2.108	0.038
ENSG00000113391	FAM172A	0.213	6.150	2.156	0.034
ENSG00000113407	TARS	0.186	5.701	1.995	0.049
ENSG00000113525	IL5	-0.440	0.062	-2.538	0.013
ENSG00000113583	C5orf15	0.263	4.774	2.201	0.031
ENSG00000113643	RARS	0.210	5.908	2.008	0.048
ENSG00000113845	TIMMDC1	0.218	5.270	2.043	0.044
ENSG00000114107	CEP70	0.245	5.658	2.240	0.028
ENSG00000114125	RNF7	0.251	4.018	2.408	0.018
ENSG00000114126	TFDP2	0.150	6.025	2.324	0.023
ENSG00000114200	BCHE	0.504	3.022	3.291	0.001
ENSG00000114204	SERPINI2	0.379	1.347	2.053	0.043
ENSG00000114209	PDCD10	0.314	5.266	2.565	0.012
ENSG00000114316	USP4	-0.170	5.189	-2.399	0.019
ENSG00000114331	ACAP2	0.167	6.513	2.009	0.048
ENSG00000114446	IFT57	0.229	5.579	2.162	0.034
ENSG00000114450	GNB4	0.413	6.052	2.939	0.004
ENSG00000114480	GBE1	0.258	4.096	2.007	0.048
ENSG00000114520	SNX4	0.287	5.453	2.327	0.022
ENSG00000114942	EEF1B2	0.404	5.397	2.205	0.030
ENSG00000114999	TTL	0.187	7.024	2.196	0.031
ENSG00000115207	GTF3C2	-0.136	4.035	-2.154	0.034
ENSG00000115317	HTRA2	0.296	1.874	2.356	0.021
ENSG00000115353	TACR1	-0.432	2.105	-3.272	0.002
ENSG00000115380	EFEMP1	-0.561	5.732	-2.418	0.018
ENSG00000115507	OTX1	-0.499	0.334	-2.088	0.040
ENSG00000115524	SF3B1	0.199	8.461	2.292	0.024
ENSG00000115568	ZNF142	-0.270	4.695	-2.227	0.029
ENSG00000115705	TPO	-0.938	-1.280	-2.904	0.005
ENSG00000115816	CEBPZ	0.214	5.094	2.163	0.033

ENSG00000115875	SRSF7	0.219	6.292	2.321	0.023
ENSG00000116030	SUMO1	0.352	5.857	2.267	0.026
ENSG00000116128	BCL9	-0.241	4.665	-3.208	0.002
ENSG00000116266	STXBP3	0.269	6.107	2.294	0.024
ENSG00000116299	KIAA1324	-0.188	5.375	-2.177	0.032
ENSG00000116497	S100PBP	0.141	4.927	2.138	0.035
ENSG00000116604	MEF2D	-0.221	6.770	-2.108	0.038
ENSG00000116679	IVNS1ABP	0.215	6.510	2.127	0.036
ENSG00000116698	SMG7	-0.248	6.009	-2.915	0.005
ENSG00000116898	MRPS15	0.174	4.280	2.005	0.048
ENSG00000116906	GNPAT	0.235	4.976	2.150	0.034
ENSG00000117013	KCNQ4	-0.378	2.111	-2.200	0.031
ENSG00000117036	ETV3	-0.131	5.205	-2.024	0.046
ENSG00000117281	CD160	-0.421	1.078	-2.476	0.015
ENSG00000117360	PRPF3	0.129	5.605	2.087	0.040
ENSG00000117395	EBNA1BP2	0.220	5.424	2.056	0.043
ENSG00000117475	BLZF1	0.195	5.590	2.187	0.032
ENSG00000117595	IRF6	0.507	0.408	2.044	0.044
ENSG00000117616	RSRP1	0.211	6.142	2.235	0.028
ENSG00000117713	ARID1A	-0.276	6.566	-2.929	0.004
ENSG00000118058	KMT2A	-0.194	8.047	-3.141	0.002
ENSG00000118454	ANKRD13C	0.199	6.351	2.065	0.042
ENSG00000118680	MYL12B	0.272	6.012	1.993	0.050
ENSG00000118689	FOXO3	-0.139	6.689	-2.238	0.028
ENSG00000119013	NDUFB3	0.502	3.523	2.398	0.019
ENSG00000119314	PTBP3	0.166	6.169	2.209	0.030
ENSG00000119401	TRIM32	-0.136	4.912	-2.025	0.046
ENSG00000119636	BBOF1	0.220	3.392	2.342	0.022
ENSG00000119661	DNAL1	0.279	6.567	2.313	0.023
ENSG00000119698	PPP4R4	0.377	6.581	2.518	0.014
ENSG00000119705	SLIRP	0.339	4.501	2.459	0.016
ENSG00000119946	CNNM1	-0.196	5.631	-2.135	0.036
ENSG00000119986	AVPI1	-0.346	2.534	-2.442	0.017
ENSG00000120008	WDR11	0.140	6.245	2.147	0.035
ENSG00000120137	PANK3	0.213	7.988	2.069	0.042
ENSG00000120159	CAAP1	0.219	4.019	2.294	0.024
ENSG00000120251	GRIA2	0.207	10.224	2.062	0.042
ENSG00000120279	MYCT1	0.377	2.210	2.107	0.038
ENSG00000120437	ACAT2	0.367	4.066	2.020	0.047
ENSG00000120645	IQSEC3	-0.324	5.397	-2.279	0.025
ENSG00000120686	UFM1	0.305	6.698	2.298	0.024
ENSG00000120708	TGFBI	0.645	1.425	2.047	0.044
ENSG00000120756	PLS1	0.269	3.280	2.178	0.032
ENSG00000120832	MTERF2	0.193	4.503	2.868	0.005
ENSG00000120837	NFYB	0.295	5.573	2.301	0.024
ENSG00000120899	PTK2B	-0.288	7.302	-2.161	0.034
ENSG00000120992	LYPLA1	0.367	4.174	2.610	0.011

ENSG00000121022	COPS5	0.181	4.923	2.359	0.021
ENSG00000121289	CEP89	0.175	4.599	2.524	0.014
ENSG00000121390	PSPC1	0.131	6.324	2.097	0.039
ENSG00000121481	RNF2	0.251	5.284	2.015	0.047
ENSG00000121621	KIF18A	0.627	-0.968	2.326	0.022
ENSG00000121766	ZCCHC17	0.228	5.961	2.108	0.038
ENSG00000121769	FABP3	0.261	6.138	2.009	0.048
ENSG00000121797	CCRL2	-0.636	-1.241	-2.234	0.028
ENSG00000121851	POLR3GL	0.287	2.944	2.027	0.046
ENSG00000121864	ZNF639	0.240	5.248	2.110	0.038
ENSG00000122008	POLK	0.225	5.956	2.037	0.045
ENSG00000122034	GTF3A	0.212	5.209	2.027	0.046
ENSG00000122068	FYTTD1	0.218	6.260	2.027	0.046
ENSG00000122417	ODF2L	0.209	6.303	2.109	0.038
ENSG00000122420	PTGFR	0.731	1.096	2.758	0.007
ENSG00000122545	SEPTIN7	0.254	9.003	2.103	0.039
ENSG00000122574	WIPF3	-0.405	5.131	-3.445	0.001
ENSG00000122591	FAM126A	0.304	5.446	2.614	0.011
ENSG00000122779	TRIM24	0.136	5.409	2.057	0.043
ENSG00000122873	CISD1	0.362	5.132	2.135	0.036
ENSG00000122970	IFT81	0.282	5.022	3.030	0.003
ENSG00000123080	CDKN2C	-0.334	1.671	-2.120	0.037
ENSG00000123360	PDE1B	-0.255	5.134	-2.343	0.022
ENSG00000123384	LRP1	-0.300	8.695	-2.174	0.033
ENSG00000123600	METTL8	0.220	5.128	2.669	0.009
ENSG00000123643	SLC36A1	-0.245	5.484	-2.400	0.019
ENSG00000124134	KCNS1	-0.671	4.896	-3.371	0.001
ENSG00000124145	SDC4	-0.456	5.115	-2.165	0.033
ENSG00000124172	ATP5E	0.376	6.055	2.065	0.042
ENSG00000124191	TOX2	-0.358	2.460	-2.522	0.014
ENSG00000124222	STX16	-0.229	4.992	-2.526	0.013
ENSG00000124440	HIF3A	-0.487	3.461	-2.182	0.032
ENSG00000124535	WRNIP1	0.176	5.055	2.425	0.018
ENSG00000124766	SOX4	0.420	3.365	3.280	0.002
ENSG00000124786	SLC35B3	0.291	3.379	2.396	0.019
ENSG00000124827	GCM2	-0.444	0.659	-2.291	0.025
ENSG00000125378	BMP4	-0.767	-0.257	-2.672	0.009
ENSG00000125388	GRK4	0.199	4.656	2.413	0.018
ENSG00000125398	SOX9	-0.450	5.654	-2.528	0.013
ENSG00000125414	MYH2	-1.480	-2.746	-2.877	0.005
ENSG00000125631	HTR5BP	-0.465	-1.582	-2.135	0.036
ENSG00000125691	RPL23	0.347	6.034	2.544	0.013
ENSG00000125743	SNRPD2	0.249	4.557	2.091	0.040
ENSG00000125845	BMP2	0.291	1.891	2.380	0.020
ENSG00000125868	DSTN	0.309	8.138	2.014	0.047
ENSG00000125870	SNRPB2	0.300	4.630	2.046	0.044
ENSG00000125875	TBC1D20	-0.192	5.327	-3.160	0.002

ENSG00000125895	TMEM74B	0.262	2.172	2.000	0.049
ENSG00000125977	EIF2S2	0.347	5.044	2.587	0.011
ENSG00000126003	PLAGL2	-0.369	2.657	-2.684	0.009
ENSG00000126261	UBA2	0.253	6.390	2.107	0.038
ENSG00000126368	NR1D1	-0.401	3.901	-2.042	0.044
ENSG00000126524	SBDS	0.257	6.702	2.211	0.030
ENSG00000126777	KTN1	0.207	7.928	2.309	0.023
ENSG00000126804	ZBTB1	0.201	5.815	2.136	0.036
ENSG00000126883	NUP214	-0.222	5.368	-3.177	0.002
ENSG00000127084	FGD3	0.425	0.681	2.492	0.015
ENSG00000127125	PPCS	0.433	4.042	2.994	0.004
ENSG00000127252	HRASLS	0.369	2.769	3.636	0.000
ENSG00000127362	TAS2R3	-0.279	1.549	-2.105	0.038
ENSG00000127364	TAS2R4	-0.238	2.830	-2.112	0.038
ENSG00000127366	TAS2R5	-0.356	0.711	-2.000	0.049
ENSG00000127481	UBR4	-0.160	8.424	-2.465	0.016
ENSG00000127527	EPS15L1	-0.299	4.594	-2.559	0.012
ENSG00000127838	PNKD	-0.348	4.914	-2.527	0.013
ENSG00000127914	AKAP9	0.221	8.785	2.341	0.022
ENSG00000127955	GNAI1	0.291	6.714	2.211	0.030
ENSG00000127989	MTERF1	0.198	4.369	2.160	0.034
ENSG00000128272	ATF4	0.290	6.295	2.822	0.006
ENSG00000128510	CPA4	-0.744	-2.311	-2.036	0.045
ENSG00000128595	CALU	0.283	6.399	3.168	0.002
ENSG00000128609	NDUFA5	0.438	7.510	2.144	0.035
ENSG00000128708	HAT1	0.231	4.973	2.280	0.025
ENSG00000128805	ARHGAP22	0.271	3.507	2.096	0.039
ENSG00000128951	DUT	0.338	4.184	2.297	0.024
ENSG00000128989	ARPP19	0.356	9.762	2.459	0.016
ENSG00000129083	COPB1	0.230	5.718	2.299	0.024
ENSG00000129159	KCNC1	-0.239	5.891	-2.305	0.024
ENSG00000129244	ATP1B2	-0.477	7.105	-2.479	0.015
ENSG00000129292	PHF20L1	0.199	7.298	2.252	0.027
ENSG00000129351	ILF3	-0.177	6.608	-2.360	0.021
ENSG00000129451	KLK10	-0.936	-1.865	-2.599	0.011
ENSG00000129515	SNX6	0.278	5.344	2.032	0.045
ENSG00000129534	MIS18BP1	0.334	4.606	2.402	0.019
ENSG00000130244	FAM98C	0.207	3.204	2.295	0.024
ENSG00000130300	PLVAP	0.753	-0.112	2.575	0.012
ENSG00000130332	LSM7	0.357	1.317	2.605	0.011
ENSG00000130558	OLFM1	0.231	9.013	2.108	0.038
ENSG00000130635	COL5A1	0.481	1.729	2.463	0.016
ENSG00000130713	EXOSC2	0.194	4.276	2.117	0.037
ENSG00000130783	CCDC62	0.231	3.453	2.383	0.019
ENSG00000130997	POLN	0.200	3.994	2.157	0.034
ENSG00000131013	PPIL4	0.247	6.428	2.016	0.047
ENSG00000131051	RBM39	0.219	8.059	2.797	0.006

ENSG00000131127	ZNF141	0.257	5.058	2.768	0.007
ENSG00000131732	ZCCHC9	0.271	3.672	2.968	0.004
ENSG00000131771	PPP1R1B	-0.320	6.003	-2.383	0.019
ENSG00000131871	VIMP	0.248	3.551	2.453	0.016
ENSG00000131931	THAP1	0.265	3.424	2.187	0.032
ENSG00000131943	C19orf12	0.200	5.747	2.382	0.020
ENSG00000132383	RPA1	-0.256	4.692	-3.141	0.002
ENSG00000132394	EEFSEC	-0.366	1.395	-2.591	0.011
ENSG00000132432	SEC61G	0.522	3.037	2.992	0.004
ENSG00000132554	RGS22	0.299	3.551	2.039	0.045
ENSG00000132604	TERF2	-0.200	5.292	-2.881	0.005
ENSG00000132622	HSPA12B	0.540	1.098	2.026	0.046
ENSG00000132646	PCNA	0.262	3.597	2.126	0.037
ENSG00000132676	DAP3	0.163	5.243	2.164	0.033
ENSG00000132681	ATP1A4	-0.592	1.342	-2.366	0.020
ENSG00000132842	AP3B1	0.182	5.675	2.097	0.039
ENSG00000132854	KANK4	-0.497	0.797	-2.251	0.027
ENSG00000132950	ZMYM5	0.190	4.619	2.023	0.046
ENSG00000132964	CDK8	0.193	4.830	2.148	0.035
ENSG00000133121	STARD13	-0.212	5.886	-2.479	0.015
ENSG00000133401	PDZD2	-0.197	7.451	-2.010	0.048
ENSG00000133477	FAM83F	0.714	-0.644	2.357	0.021
ENSG00000133641	C12orf29	0.260	5.284	2.042	0.044
ENSG00000133661	SFTPD	-0.404	0.387	-2.427	0.017
ENSG00000133706	LARS	0.178	7.285	2.237	0.028
ENSG00000133800	LYVE1	0.516	2.921	1.996	0.049
ENSG00000134030	CTIF	-0.253	6.360	-2.234	0.028
ENSG00000134086	VHL	0.178	5.368	2.491	0.015
ENSG00000134243	SORT1	-0.183	8.613	-2.486	0.015
ENSG00000134255	CEPT1	0.181	5.111	2.253	0.027
ENSG00000134291	TMEM106C	0.148	4.205	1.994	0.049
ENSG00000134333	LDHA	0.275	7.927	2.244	0.027
ENSG00000134375	TIMM17A	0.478	4.695	2.345	0.021
ENSG00000134419	RPS15A	0.370	4.051	2.242	0.028
ENSG00000134508	CABLES1	-0.266	5.754	-2.143	0.035
ENSG00000134757	DSG3	1.296	-0.904	3.462	0.001
ENSG00000134853	PDGFRA	0.516	6.421	3.580	0.001
ENSG00000134874	DZIP1	0.139	6.928	2.085	0.040
ENSG00000134884	ARGLU1	0.155	7.756	2.287	0.025
ENSG00000134897	BIVM	0.203	4.529	2.142	0.035
ENSG00000135045	C9orf40	0.273	3.142	2.096	0.039
ENSG00000135127	BICDL1	0.227	4.880	2.287	0.025
ENSG00000135164	DMTF1	0.165	7.165	2.736	0.008
ENSG00000135218	CD36	0.425	1.415	2.034	0.045
ENSG00000135241	PNPLA8	0.253	6.812	2.006	0.048
ENSG00000135248	FAM71F1	0.494	-0.055	2.456	0.016
ENSG00000135269	TES	0.542	0.710	2.625	0.010

ENSG00000135315	CEP162	0.288	5.085	2.754	0.007
ENSG00000135404	CD63	0.265	5.240	2.019	0.047
ENSG00000135472	FAIM2	-0.326	8.305	-2.756	0.007
ENSG00000135486	HNRNPA1	0.238	6.700	2.419	0.018
ENSG00000135622	SEMA4F	-0.204	4.525	-2.044	0.044
ENSG00000135698	MPHOSPH6	0.233	4.791	2.306	0.024
ENSG00000135766	EGLN1	0.203	6.798	2.149	0.035
ENSG00000135913	USP37	0.196	5.177	2.685	0.009
ENSG00000135951	TSGA10	0.277	4.973	2.706	0.008
ENSG00000136051	KIAA1033	0.232	6.357	2.112	0.038
ENSG00000136159	NUDT15	0.260	3.267	2.209	0.030
ENSG00000136261	BZW2	0.201	4.199	2.021	0.047
ENSG00000136274	NACAD	-0.399	4.995	-2.258	0.027
ENSG00000136371	MTHFS	-0.337	-0.335	-2.034	0.045
ENSG00000136536	MARCHF7	0.185	6.423	2.012	0.047
ENSG00000136715	SAP130	-0.213	4.790	-2.962	0.004
ENSG00000136770	DNAJC1	0.228	4.235	2.417	0.018
ENSG00000136783	NIPSNAP3A	0.280	4.027	2.371	0.020
ENSG00000136810	TXN	0.433	4.360	2.221	0.029
ENSG00000136811	ODF2	0.145	4.659	2.021	0.047
ENSG00000136828	RALGPS1	-0.104	5.907	-2.125	0.037
ENSG00000136854	STXBP1	-0.390	8.441	-3.353	0.001
ENSG00000136999	NOV	0.342	4.370	2.088	0.040
ENSG00000137038	TMEM261	0.295	4.532	2.272	0.026
ENSG00000137166	FOXP4	-0.449	2.503	-2.152	0.034
ENSG00000137198	GMPR	-0.439	1.442	-2.021	0.047
ENSG00000137261	KIAA0319	-0.161	6.538	-2.358	0.021
ENSG00000137343	ATAT1	-0.214	3.791	-2.245	0.027
ENSG00000137500	CCDC90B	0.247	5.437	2.115	0.037
ENSG00000137574	TGS1	0.172	5.152	2.143	0.035
ENSG00000137692	DCUN1D5	0.318	4.525	2.611	0.011
ENSG00000137806	NDUFAF1	0.355	4.012	2.772	0.007
ENSG00000137821	LRRC49	0.193	5.735	2.061	0.042
ENSG00000137841	PLCB2	0.311	3.310	2.195	0.031
ENSG00000137955	RABGGTB	0.217	5.935	2.316	0.023
ENSG00000138035	PNPT1	0.251	4.809	2.168	0.033
ENSG00000138061	CYP1B1	0.480	3.690	2.552	0.013
ENSG00000138182	KIF20B	0.306	3.895	2.101	0.039
ENSG00000138190	EXOC6	0.301	6.002	2.160	0.034
ENSG00000138246	DNAJC13	0.178	6.897	2.261	0.026
ENSG00000138286	FAM149B1	0.119	5.174	2.189	0.031
ENSG00000138326	RPS24	0.277	7.254	2.101	0.039
ENSG00000138363	ATIC	-0.172	4.269	-2.258	0.027
ENSG00000138385	SSB	0.325	6.349	2.370	0.020
ENSG00000138413	IDH1	0.279	4.804	2.697	0.008
ENSG00000138495	COX17	0.367	3.475	2.246	0.027
ENSG00000138592	USP8	0.174	6.764	2.312	0.023

ENSG00000138594	TMOD3	0.151	5.556	2.040	0.045
ENSG00000138600	SPPL2A	0.231	5.213	2.112	0.038
ENSG00000138660	AP1AR	0.354	5.727	2.191	0.031
ENSG00000138669	PRKG2	0.534	4.247	3.527	0.001
ENSG00000138768	USO1	0.257	6.933	2.105	0.038
ENSG00000138795	LEF1	0.403	2.667	2.076	0.041
ENSG00000139146	FAM60A	0.563	0.528	2.778	0.007
ENSG00000139182	CLSTN3	-0.252	7.527	-1.996	0.049
ENSG00000139190	VAMP1	-0.327	4.913	-2.158	0.034
ENSG00000139200	PIANP	-0.450	3.745	-2.085	0.040
ENSG00000139219	COL2A1	-1.064	-2.323	-2.975	0.004
ENSG00000139220	PPFIA2	0.245	8.304	2.712	0.008
ENSG00000139266	MARCHF9	0.226	2.577	2.402	0.019
ENSG00000139343	SNRPF	0.281	3.073	2.179	0.032
ENSG00000139354	GAS2L3	0.427	2.218	2.161	0.034
ENSG00000139496	NUP58	0.212	6.612	2.094	0.039
ENSG00000139651	ZNF740	-0.130	4.579	-2.019	0.047
ENSG00000139679	LPAR6	0.390	4.174	2.370	0.020
ENSG00000139684	ESD	0.289	5.126	2.300	0.024
ENSG00000139687	RB1	0.237	5.902	2.326	0.022
ENSG00000139714	MORN3	0.251	3.020	2.255	0.027
ENSG00000139726	DENR	0.301	6.179	2.377	0.020
ENSG00000139767	SRRM4	-0.245	6.540	-2.335	0.022
ENSG00000139797	RNF113B	-0.861	-1.640	-2.266	0.026
ENSG00000139835	GRTP1	-0.600	-0.137	-2.953	0.004
ENSG00000139865	TTC6	0.394	3.138	2.252	0.027
ENSG00000140307	GTF2A2	0.332	3.283	2.540	0.013
ENSG00000140367	UBE2Q2	0.341	5.285	2.656	0.009
ENSG00000140374	ETFA	0.294	5.062	2.613	0.011
ENSG00000140403	DNAJA4	-0.310	6.594	-2.755	0.007
ENSG00000140443	IGF1R	-0.211	6.676	-2.818	0.006
ENSG00000140471	LINS1	0.242	4.619	2.036	0.045
ENSG00000140519	RHCG	-0.291	1.173	-2.214	0.030
ENSG00000141198	TOM1L1	0.189	4.807	2.159	0.034
ENSG00000141252	VPS53	-0.260	7.265	-3.519	0.001
ENSG00000141314	RHBDL3	-0.548	3.616	-3.010	0.003
ENSG00000141429	GALNT1	0.240	5.973	2.196	0.031
ENSG00000141434	MEP1B	-0.403	0.533	-2.432	0.017
ENSG00000141446	ESCO1	0.231	6.026	2.234	0.028
ENSG00000141542	RAB40B	0.283	6.294	3.068	0.003
ENSG00000141698	NT5C3B	0.291	4.989	2.529	0.013
ENSG00000141750	STAC2	-0.392	3.662	-2.285	0.025
ENSG00000141753	IGFBP4	0.388	2.319	1.997	0.049
ENSG00000141759	TXNL4A	0.199	5.370	2.307	0.024
ENSG00000141905	NFIC	-0.414	5.529	-2.920	0.005
ENSG00000142197	DOPEY2	-0.142	5.248	-2.114	0.038
ENSG00000142871	CYR61	-0.650	2.428	-2.575	0.012

ENSG00000143033	MTF2	0.166	5.531	2.013	0.047
ENSG00000143126	CELSR2	-0.339	7.559	-2.387	0.019
ENSG00000143156	NME7	0.281	4.685	3.342	0.001
ENSG00000143190	POU2F1	-0.187	6.270	-2.442	0.017
ENSG00000143195	ILDR2	0.165	7.094	2.048	0.044
ENSG00000143322	ABL2	-0.199	6.564	-3.657	0.000
ENSG00000143353	LYPLAL1	0.227	5.077	2.069	0.042
ENSG00000143442	POGZ	-0.165	6.714	-2.400	0.019
ENSG00000143452	HORMAD1	0.608	-0.199	2.832	0.006
ENSG00000143569	UBAP2L	-0.157	6.498	-2.518	0.014
ENSG00000143603	KCNN3	-0.295	6.732	-2.012	0.047
ENSG00000143614	GATAD2B	-0.230	6.682	-4.019	0.000
ENSG00000143621	ILF2	0.199	6.043	2.215	0.029
ENSG00000143633	C1orf131	0.193	3.058	2.178	0.032
ENSG00000143748	NVL	0.159	4.185	2.082	0.040
ENSG00000143772	ITPKB	-0.360	6.281	-2.114	0.038
ENSG00000143850	PLEKHA6	-0.200	6.707	-2.122	0.037
ENSG00000143858	SYT2	-0.655	3.535	-2.477	0.015
ENSG00000143870	PDIA6	0.358	5.205	3.212	0.002
ENSG00000143889	HNRNPLL	0.169	5.865	2.119	0.037
ENSG00000143933	CALM2	0.347	9.813	2.335	0.022
ENSG00000143977	SNRPG	0.549	2.195	3.285	0.001
ENSG00000143994	ABHD1	-0.299	0.939	-2.032	0.045
ENSG00000144026	ZNF514	0.210	5.042	2.217	0.029
ENSG00000144034	TPRKB	0.222	2.907	2.446	0.017
ENSG00000144161	ZC3H8	0.244	5.858	2.745	0.007
ENSG00000144331	ZNF385B	-0.200	4.668	-2.054	0.043
ENSG00000144407	PTH2R	-0.431	2.199	-2.300	0.024
ENSG00000144426	NBEAL1	0.226	6.108	2.675	0.009
ENSG00000144619	CNTN4	0.224	6.196	2.084	0.040
ENSG00000144677	CTDSPL	-0.179	4.923	-2.505	0.014
ENSG00000144711	IQSEC1	-0.175	7.588	-2.016	0.047
ENSG00000144747	TMF1	0.230	6.399	2.215	0.030
ENSG00000144847	IGSF11	-0.189	5.357	-2.007	0.048
ENSG00000144848	ATG3	0.287	5.078	2.300	0.024
ENSG00000144895	EIF2A	0.245	5.670	2.034	0.045
ENSG00000144935	TRPC1	0.238	6.094	2.188	0.031
ENSG00000145050	MANF	0.247	3.238	2.527	0.013
ENSG00000145293	ENOPH1	0.301	6.140	2.383	0.019
ENSG00000145335	SNCA	0.342	7.951	2.199	0.031
ENSG00000145425	RPS3A	0.545	5.198	2.250	0.027
ENSG00000145439	CBR4	0.203	5.661	2.083	0.040
ENSG00000145569	FAM105A	0.320	3.785	2.625	0.010
ENSG00000145734	BDP1	0.179	8.733	2.229	0.029
ENSG00000145782	ATG12	0.257	6.157	2.511	0.014
ENSG00000145819	ARHGAP26	-0.184	8.046	-2.642	0.010
ENSG00000145888	GLRA1	-0.637	-1.258	-2.460	0.016

ENSG00000145908	ZNF300	0.431	2.096	2.592	0.011
ENSG00000146005	PSD2	-0.268	6.317	-2.143	0.035
ENSG00000146007	ZMAT2	0.275	6.285	2.198	0.031
ENSG00000146281	PM20D2	0.275	4.554	2.905	0.005
ENSG00000146433	TMEM181	0.221	6.755	2.138	0.035
ENSG00000146469	VIP	0.542	3.908	2.188	0.031
ENSG00000146648	EGFR	-0.332	5.834	-2.325	0.023
ENSG00000146666	LINC00525	-1.023	-1.796	-3.025	0.003
ENSG00000146731	CCT6A	0.234	6.542	2.074	0.041
ENSG00000146757	ZNF92	0.327	4.263	2.270	0.026
ENSG00000146776	ATXN7L1	-0.154	4.608	-2.138	0.035
ENSG00000146834	MEPCE	-0.267	4.159	-2.533	0.013
ENSG00000146872	TLK2	0.217	5.229	2.024	0.046
ENSG00000147573	TRIM55	1.310	-3.424	2.539	0.013
ENSG00000147604	RPL7	0.650	5.367	2.460	0.016
ENSG00000147647	DPYS	0.359	1.846	2.826	0.006
ENSG00000147649	MTDH	0.182	7.220	2.101	0.039
ENSG00000147669	POLR2K	0.356	5.573	2.065	0.042
ENSG00000148123	PLPPR1	0.427	4.266	2.958	0.004
ENSG00000148219	ASTN2	-0.296	4.889	-2.978	0.004
ENSG00000148396	SEC16A	-0.245	5.927	-2.547	0.013
ENSG00000148429	USP6NL	0.167	4.676	2.138	0.035
ENSG00000148468	FAM171A1	-0.189	6.511	-2.321	0.023
ENSG00000148481	FAM188A	0.264	6.165	2.069	0.042
ENSG00000148660	CAMK2G	-0.207	7.339	-2.103	0.039
ENSG00000148680	HTR7	0.332	1.888	2.188	0.031
ENSG00000148690	FRA10AC1	0.274	5.778	2.413	0.018
ENSG00000148737	TCF7L2	-0.331	3.852	-2.669	0.009
ENSG00000148842	CNNM2	-0.141	5.840	-2.293	0.024
ENSG00000149311	ATM	0.225	7.643	2.431	0.017
ENSG00000149532	CPSF7	-0.148	5.590	-2.375	0.020
ENSG00000149577	SIDT2	-0.215	4.108	-2.062	0.042
ENSG00000149596	JPH2	0.917	-1.699	2.086	0.040
ENSG00000149972	CNTN5	0.334	5.272	2.174	0.033
ENSG00000150275	PCDH15	0.376	5.344	3.202	0.002
ENSG00000150361	KLHL1	0.368	2.593	2.000	0.049
ENSG00000150527	CTAGE5	0.234	5.013	2.252	0.027
ENSG00000150540	HNMT	0.258	4.557	1.993	0.050
ENSG00000150593	PDCD4	0.191	5.970	2.097	0.039
ENSG00000150672	DLG2	0.210	9.213	2.471	0.016
ENSG00000150787	PTS	0.370	4.110	2.191	0.031
ENSG00000150977	RILPL2	0.310	3.318	3.039	0.003
ENSG00000151005	TKTL2	0.929	-2.555	2.104	0.038
ENSG00000151135	TMEM263	0.250	5.680	2.371	0.020
ENSG00000151239	TWF1	0.268	5.230	2.015	0.047
ENSG00000151338	MIPOL1	0.210	5.194	2.155	0.034
ENSG00000151466	SCLT1	0.240	5.484	2.427	0.017

ENSG00000151552	QDPR	-0.348	5.886	-2.658	0.009
ENSG00000151692	RNF144A	-0.143	6.139	-2.341	0.022
ENSG00000151718	WWC2	0.178	5.613	2.995	0.004
ENSG00000152092	ASTN1	-0.149	7.987	-2.194	0.031
ENSG00000152104	PTPN14	-0.229	5.354	-2.599	0.011
ENSG00000152137	HSPB8	-0.320	5.199	-2.726	0.008
ENSG00000152208	GRID2	0.256	5.174	2.311	0.023
ENSG00000152291	TGOLN2	-0.140	7.898	-2.156	0.034
ENSG00000152380	FAM151B	0.225	2.807	2.399	0.019
ENSG00000152413	HOMER1	0.415	7.993	3.214	0.002
ENSG00000152463	OLAH	-0.517	-1.191	-2.024	0.046
ENSG00000152484	USP12	0.291	6.706	2.108	0.038
ENSG00000152492	CCDC50	0.191	6.404	2.043	0.044
ENSG00000152527	PLEKHH2	0.423	4.815	4.063	0.000
ENSG00000152578	GRIA4	0.243	7.916	2.219	0.029
ENSG00000152795	HNRNPDL	0.257	8.336	2.819	0.006
ENSG00000152942	RAD17	0.252	4.809	2.216	0.029
ENSG00000153037	SRP19	0.166	4.563	1.993	0.050
ENSG00000153130	SCOC	0.386	7.058	2.138	0.035
ENSG00000153140	CETN3	0.309	4.494	2.608	0.011
ENSG00000153237	CCDC148	0.259	4.152	2.143	0.035
ENSG00000153253	SCN3A	0.227	7.382	2.503	0.014
ENSG00000153339	TRAPPC8	0.238	6.514	2.237	0.028
ENSG00000153531	ADPRHL1	-0.417	2.471	-2.723	0.008
ENSG00000154122	ANKH	-0.119	7.130	-2.118	0.037
ENSG00000154240	CEP112	0.181	5.068	2.076	0.041
ENSG00000154370	TRIM11	0.291	3.443	2.577	0.012
ENSG00000154429	CCSAP	0.233	7.922	2.343	0.022
ENSG00000154478	GPR26	0.448	5.471	2.626	0.010
ENSG00000154485	MMP21	-0.571	-1.462	-2.290	0.025
ENSG00000154639	CXADR	0.422	5.691	3.028	0.003
ENSG00000154640	BTG3	0.330	2.313	2.484	0.015
ENSG00000154654	NCAM2	0.306	8.849	2.395	0.019
ENSG00000154928	EPHB1	-0.315	4.392	-3.110	0.003
ENSG00000155011	DKK2	0.485	2.242	1.990	0.050
ENSG00000155085	AK9	0.225	4.982	2.182	0.032
ENSG00000155096	AZIN1	0.311	8.145	2.642	0.010
ENSG00000155100	OTUD6B	0.206	5.083	2.135	0.036
ENSG00000155115	GTF3C6	0.368	3.811	2.607	0.011
ENSG00000155269	GPR78	0.701	-0.920	2.095	0.039
ENSG00000155304	HSPA13	0.307	7.013	2.129	0.036
ENSG00000155307	SAMSN1	0.646	1.808	2.272	0.026
ENSG00000155313	USP25	0.217	7.194	2.189	0.031
ENSG00000155330	C16orf87	0.253	5.291	2.194	0.031
ENSG00000155368	DBI	0.247	5.074	2.051	0.043
ENSG00000155393	HEATR3	0.212	4.271	2.188	0.032
ENSG00000155438	NIFK	0.354	3.929	2.544	0.013

ENSG00000155506	LARP1	-0.223	7.963	-2.945	0.004
ENSG00000155816	FMN2	-0.217	6.878	-3.128	0.002
ENSG00000155846	PPARGC1B	-0.240	5.089	-3.038	0.003
ENSG00000155876	RRAGA	-0.220	5.181	-2.143	0.035
ENSG00000155980	KIF5A	-0.280	10.332	-2.766	0.007
ENSG00000156136	DCK	0.306	5.528	2.055	0.043
ENSG00000156171	DRAM2	0.236	3.551	2.128	0.036
ENSG00000156261	CCT8	0.312	6.382	2.035	0.045
ENSG00000156304	SCAF4	-0.208	4.810	-2.645	0.010
ENSG00000156463	SH3RF2	-0.452	1.576	-2.557	0.012
ENSG00000156535	CD109	-0.308	3.454	-2.349	0.021
ENSG00000156711	MAPK13	-0.292	4.049	-2.379	0.020
ENSG00000156787	TBC1D31	0.262	5.132	2.884	0.005
ENSG00000156928	MALSU1	0.230	3.058	2.383	0.019
ENSG00000157045	NTAN1	0.284	2.950	2.462	0.016
ENSG00000157216	SSBP3	-0.209	5.330	-2.095	0.039
ENSG00000157617	C2CD2	-0.197	5.217	-2.367	0.020
ENSG00000157657	ZNF618	-0.270	3.021	-2.572	0.012
ENSG00000157693	C9orf91	-0.169	5.949	-2.219	0.029
ENSG00000157833	GAREM2	-0.368	4.157	-2.259	0.027
ENSG00000158109	TPRG1L	-0.256	6.318	-3.358	0.001
ENSG00000158158	CNNM4	-0.218	3.619	-2.360	0.021
ENSG00000158234	FAIM	0.214	3.655	2.173	0.033
ENSG00000158411	MITD1	0.234	3.996	2.184	0.032
ENSG00000158480	SPATA2	-0.220	4.600	-2.235	0.028
ENSG00000159167	STC1	0.680	-0.080	2.067	0.042
ENSG00000159256	MORC3	0.203	6.525	2.040	0.045
ENSG00000159479	MED8	0.161	4.441	2.099	0.039
ENSG00000159788	RGS12	0.242	5.690	2.359	0.021
ENSG00000159899	NPR2	-0.185	3.456	-2.072	0.041
ENSG00000159915	ZNF233	0.162	3.837	2.047	0.044
ENSG00000159961	OR3A3	-0.808	-1.508	-2.005	0.048
ENSG00000160117	ANKLE1	-0.442	-0.743	-2.317	0.023
ENSG00000160207	HSF2BP	0.263	0.960	2.036	0.045
ENSG00000160209	PDXK	-0.243	7.412	-2.383	0.019
ENSG00000160321	ZNF208	-0.625	2.919	-2.111	0.038
ENSG00000160584	SIK3	-0.191	7.271	-2.967	0.004
ENSG00000160695	VPS11	-0.150	5.543	-2.003	0.048
ENSG00000160710	ADAR	-0.123	8.414	-2.152	0.034
ENSG00000161405	IKZF3	-0.545	0.915	-2.074	0.041
ENSG00000161640	SIGLEC11	0.795	-1.612	2.153	0.034
ENSG00000161642	ZNF385A	-0.356	3.405	-2.010	0.048
ENSG00000161970	RPL26	0.437	5.971	2.643	0.010
ENSG00000161999	JMJD8	-0.322	2.015	-1.995	0.049
ENSG00000162104	ADCY9	-0.172	5.493	-2.025	0.046
ENSG00000162385	MAGOH	0.219	2.806	2.069	0.042
ENSG00000162390	ACOT11	-0.342	4.998	-2.934	0.004

ENSG00000162441	LZIC	0.212	5.037	2.389	0.019
ENSG00000162613	FUBP1	0.191	6.968	2.632	0.010
ENSG00000162621	LRRC53	0.426	2.610	2.079	0.041
ENSG00000162636	FAM102B	0.296	7.234	2.211	0.030
ENSG00000162642	C1orf52	0.270	5.042	2.480	0.015
ENSG00000162670	BRINP3	0.246	5.426	2.582	0.012
ENSG00000162687	KCNT2	0.303	5.944	2.866	0.005
ENSG00000162704	ARPC5	0.304	5.907	3.013	0.003
ENSG00000162722	TRIM58	0.533	1.953	2.411	0.018
ENSG00000162782	TDRD5	0.488	2.288	2.730	0.008
ENSG00000162813	BPNT1	0.246	3.734	2.241	0.028
ENSG00000162951	LRRTM1	-0.211	4.513	-2.201	0.030
ENSG00000163029	SMC6	0.229	6.043	2.333	0.022
ENSG00000163046	ANKRD30BL	0.408	3.726	2.300	0.024
ENSG00000163093	BBS5	0.176	2.492	2.327	0.022
ENSG00000163125	RPRD2	-0.189	6.492	-3.099	0.003
ENSG00000163156	SCNM1	0.207	3.228	2.086	0.040
ENSG00000163170	BOLA3	0.350	2.842	2.402	0.019
ENSG00000163531	NFASC	-0.268	7.918	-2.266	0.026
ENSG00000163539	CLASP2	0.245	9.271	2.162	0.034
ENSG00000163558	PRKCI	0.275	6.251	2.266	0.026
ENSG00000163605	PPP4R2	0.265	6.838	2.294	0.024
ENSG00000163636	PSMD6	0.185	6.241	2.137	0.036
ENSG00000163681	SLMAP	0.229	6.649	2.468	0.016
ENSG00000163683	SMIM14	0.279	6.787	2.058	0.043
ENSG00000163710	PCOLCE2	0.471	0.594	2.228	0.029
ENSG00000163714	U2SURP	0.176	7.391	2.055	0.043
ENSG00000163728	TTC14	0.232	6.640	2.310	0.023
ENSG00000163735	CXCL5	0.667	-0.460	2.231	0.028
ENSG00000163781	TOPBP1	0.213	6.088	2.203	0.030
ENSG00000163808	KIF15	0.352	1.796	2.805	0.006
ENSG00000163811	WDR43	0.183	5.588	2.058	0.043
ENSG00000163814	CDCP1	-0.551	-0.216	-2.753	0.007
ENSG00000163938	GNL3	0.281	5.628	2.173	0.033
ENSG00000163995	ABLIM2	-0.203	5.433	-2.081	0.041
ENSG00000164061	BSN	-0.350	8.555	-2.145	0.035
ENSG00000164120	HPGD	0.689	0.019	2.128	0.036
ENSG00000164163	ABCE1	0.359	5.839	2.552	0.013
ENSG00000164167	LSM6	0.271	3.092	2.148	0.035
ENSG00000164176	EDIL3	0.323	7.520	2.000	0.049
ENSG00000164211	STARD4	0.341	6.196	2.563	0.012
ENSG00000164221	CCDC112	0.337	4.762	2.678	0.009
ENSG00000164253	WDR41	0.185	6.217	2.090	0.040
ENSG00000164306	PRIMPOL	0.219	3.742	2.061	0.042
ENSG00000164434	FABP7	0.394	5.966	2.682	0.009
ENSG00000164483	SAMD3	0.406	1.250	2.099	0.039
ENSG00000164530	PI16	-0.527	0.444	-1.993	0.050

ENSG00000164616	FBXL21	0.508	0.665	2.046	0.044
ENSG00000164687	FABP5	0.378	2.076	3.000	0.004
ENSG00000164694	FNDC1	0.712	-1.630	2.098	0.039
ENSG00000164751	PEX2	0.227	4.586	2.304	0.024
ENSG00000164934	DCAF13	0.294	4.480	2.809	0.006
ENSG00000164985	PSIP1	0.219	7.745	2.088	0.040
ENSG00000165084	C8orf34	0.363	4.259	2.964	0.004
ENSG00000165115	KIF27	0.233	4.385	2.414	0.018
ENSG00000165125	TRPV6	-0.304	2.963	-2.401	0.019
ENSG00000165338	HECTD2	0.239	6.222	2.019	0.047
ENSG00000165478	HEPACAM	-0.290	5.740	-2.267	0.026
ENSG00000165487	MICU2	0.299	5.232	2.896	0.005
ENSG00000165525	NEMF	0.222	7.227	2.387	0.019
ENSG00000165548	TMEM63C	-0.344	4.385	-2.571	0.012
ENSG00000165606	DRGX	-0.725	0.278	-2.281	0.025
ENSG00000165685	TMEM52B	0.695	0.514	2.402	0.019
ENSG00000165689	SDCCAG3	0.249	3.178	3.259	0.002
ENSG00000165714	BORCS5	-0.170	5.085	-2.663	0.009
ENSG00000165724	ZMYND19	0.254	3.033	2.095	0.039
ENSG00000165732	DDX21	0.206	5.920	2.199	0.031
ENSG00000165810	BTNL9	0.406	3.040	2.286	0.025
ENSG00000165821	SALL2	-0.241	5.262	-2.230	0.028
ENSG00000165832	TRUB1	0.317	6.115	1.998	0.049
ENSG00000165929	TC2N	0.425	4.157	2.925	0.004
ENSG00000165934	CPSF2	0.153	6.197	2.011	0.048
ENSG00000165996	HACD1	0.343	2.086	2.706	0.008
ENSG00000166035	LIPC	0.425	0.199	2.308	0.023
ENSG00000166068	SPRED1	0.270	7.632	2.399	0.019
ENSG00000166130	IKBIP	0.462	2.021	3.047	0.003
ENSG00000166135	HIF1AN	-0.160	5.862	-2.344	0.022
ENSG00000166148	AVPR1A	0.552	0.871	2.459	0.016
ENSG00000166164	BRD7	0.203	5.432	2.305	0.024
ENSG00000166226	CCT2	0.308	6.269	2.308	0.023
ENSG00000166295	ANAPC16	0.200	4.679	2.016	0.047
ENSG00000166340	TPP1	-0.437	4.928	-3.015	0.003
ENSG00000166347	CYB5A	0.262	3.495	2.865	0.005
ENSG00000166436	TRIM66	-0.319	4.722	-3.731	0.000
ENSG00000166448	TMEM130	-0.314	7.596	-2.839	0.006
ENSG00000166503	RP11-382A20.3	0.261	7.973	2.333	0.022
ENSG00000166535	A2ML1	-0.381	2.790	-3.107	0.003
ENSG00000166562	SEC11C	0.276	5.578	2.121	0.037
ENSG00000166598	HSP90B1	0.273	8.432	2.723	0.008
ENSG00000166669	ATF7IP2	0.258	4.613	2.130	0.036
ENSG00000166716	ZNF592	-0.205	5.181	-2.340	0.022
ENSG00000166734	CASC4	0.226	7.624	1.993	0.050
ENSG00000166783	KIAA0430	-0.262	7.533	-3.681	0.000
ENSG00000166794	PPIB	0.297	4.448	3.481	0.001

ENSG00000166831	RBPMS2	-0.706	-1.379	-2.193	0.031
ENSG00000167004	PDIA3	0.295	5.514	2.161	0.034
ENSG00000167037	SGSM1	-0.231	5.908	-2.139	0.035
ENSG00000167182	SP2	-0.324	2.565	-2.476	0.015
ENSG00000167460	TPM4	0.214	5.967	2.215	0.030
ENSG00000167461	RAB8A	0.215	3.210	2.292	0.024
ENSG00000167552	TUBA1A	0.282	8.404	2.322	0.023
ENSG00000167637	ZNF283	0.175	4.548	2.371	0.020
ENSG00000167676	PLIN4	-0.617	1.766	-2.954	0.004
ENSG00000167711	SERPINF2	-0.754	-1.927	-1.991	0.050
ENSG00000167842	MIS12	0.224	3.685	2.075	0.041
ENSG00000167881	SRP68	-0.157	5.393	-2.337	0.022
ENSG00000167978	SRRM2	-0.266	8.670	-2.139	0.035
ENSG00000168014	C2CD3	-0.117	5.454	-2.003	0.048
ENSG00000168066	SF1	-0.179	6.250	-2.398	0.019
ENSG00000168090	COPS6	-0.211	4.795	-3.173	0.002
ENSG00000168275	COA6	0.357	3.243	2.236	0.028
ENSG00000168288	MMADHC	0.312	5.251	2.129	0.036
ENSG00000168300	PCMTD1	0.264	6.630	2.427	0.017
ENSG00000168438	CDC40	0.237	6.280	2.257	0.027
ENSG00000168481	LGI3	-0.395	5.353	-2.214	0.030
ENSG00000168556	ING2	0.244	3.781	2.555	0.012
ENSG00000168566	SNRNP48	0.199	4.526	2.273	0.026
ENSG00000168615	ADAM9	0.211	6.368	2.316	0.023
ENSG00000168661	ZNF30	0.185	2.978	2.193	0.031
ENSG00000168685	IL7R	0.679	0.609	2.220	0.029
ENSG00000168743	NPNT	0.443	3.576	2.775	0.007
ENSG00000168802	CHTF8	-0.156	3.215	-1.998	0.049
ENSG00000168827	GFM1	0.220	6.414	2.210	0.030
ENSG00000168830	HTR1E	-0.311	3.020	-2.634	0.010
ENSG00000168843	FSTL5	0.448	5.967	2.797	0.006
ENSG00000168876	ANKRD49	0.247	3.595	2.374	0.020
ENSG00000169019	COMMD8	0.416	3.600	2.563	0.012
ENSG00000169045	HNRNPH1	0.149	7.663	2.405	0.018
ENSG00000169126	ARMC4	0.383	0.864	2.594	0.011
ENSG00000169189	NSMCE1	0.248	3.558	2.299	0.024
ENSG00000169375	SIN3A	-0.111	5.934	-2.018	0.047
ENSG00000169564	PCBP1	-0.323	3.321	-2.009	0.048
ENSG00000169592	INO80E	0.223	2.964	2.031	0.046
ENSG00000169621	APLF	0.324	5.329	2.175	0.032
ENSG00000169826	CSGALNACT2	0.210	5.886	2.303	0.024
ENSG00000169855	ROBO1	0.176	7.138	2.478	0.015
ENSG00000169857	AVEN	0.229	2.302	2.548	0.013
ENSG00000169946	ZFPM2	-0.236	5.175	-3.123	0.002
ENSG00000169964	TMEM42	0.251	2.698	2.930	0.004
ENSG00000170017	ALCAM	0.366	7.715	3.004	0.004
ENSG00000170270	C14orf142	0.364	2.432	2.319	0.023

ENSG00000170542	SERPINB9	-0.220	4.210	-2.495	0.015
ENSG00000170653	ATF7	-0.259	3.768	-2.265	0.026
ENSG00000170791	CHCHD7	0.269	5.421	2.104	0.038
ENSG00000170860	LSM3	0.380	4.691	2.169	0.033
ENSG00000170946	DNAJC24	0.223	4.577	2.415	0.018
ENSG00000170961	HAS2	0.496	0.858	2.968	0.004
ENSG00000171055	FEZ2	0.229	6.791	2.369	0.020
ENSG00000171103	TRMT61B	0.205	3.248	2.269	0.026
ENSG00000171105	INSR	-0.138	6.457	-2.451	0.016
ENSG00000171208	NETO2	0.324	5.905	2.515	0.014
ENSG00000171217	CLDN20	-0.291	1.280	-2.088	0.040
ENSG00000171291	ZNF439	0.145	4.594	1.999	0.049
ENSG00000171435	KSR2	-0.277	7.087	-2.533	0.013
ENSG00000171451	DSEL	0.232	5.534	2.226	0.029
ENSG00000171456	ASXL1	-0.152	5.714	-2.438	0.017
ENSG00000171475	WIPF2	-0.181	6.604	-2.791	0.007
ENSG00000171497	PPID	0.270	5.347	2.105	0.038
ENSG00000171502	COL24A1	0.339	5.431	2.604	0.011
ENSG00000171530	TBCA	0.423	4.339	2.604	0.011
ENSG00000171603	CLSTN1	-0.202	9.183	-2.215	0.030
ENSG00000171791	BCL2	-0.225	5.739	-2.253	0.027
ENSG00000171819	ANGPTL7	-0.454	0.384	-2.257	0.027
ENSG00000171827	ZNF570	0.229	4.965	2.371	0.020
ENSG00000171865	RNASEH1	0.231	4.026	1.994	0.049
ENSG00000171903	CYP4F11	-0.559	2.451	-2.793	0.006
ENSG00000171951	SCG2	0.517	8.106	3.353	0.001
ENSG00000171984	C20orf196	-0.327	0.597	-2.172	0.033
ENSG00000171988	JMJD1C	0.172	8.025	2.295	0.024
ENSG00000172086	KRCC1	0.328	4.111	2.720	0.008
ENSG00000172115	CYCS	0.331	7.730	2.151	0.034
ENSG00000172172	MRPL13	0.295	3.385	2.071	0.042
ENSG00000172493	AFF1	-0.220	6.134	-2.712	0.008
ENSG00000172590	MRPL52	0.181	2.903	2.067	0.042
ENSG00000172716	SLFN11	0.497	3.000	2.927	0.004
ENSG00000172809	RPL38	0.481	6.549	2.797	0.006
ENSG00000172985	SH3RF3	0.187	4.289	2.150	0.035
ENSG00000172995	ARPP21	0.196	8.013	2.304	0.024
ENSG00000173011	TADA2B	-0.159	4.526	-2.108	0.038
ENSG00000173013	CCDC96	-0.442	0.010	-2.286	0.025
ENSG00000173065	FAM222B	-0.305	3.768	-2.700	0.008
ENSG00000173085	COQ2	0.325	2.031	2.360	0.021
ENSG00000173141	MRPL57	0.253	4.248	2.673	0.009
ENSG00000173145	NOC3L	0.249	4.553	2.081	0.041
ENSG00000173175	ADCY5	-0.283	5.890	-2.166	0.033
ENSG00000173402	DAG1	-0.269	5.558	-2.614	0.011
ENSG00000173451	THAP2	0.249	4.057	2.206	0.030
ENSG00000173452	TMEM196	0.381	4.683	2.736	0.008

ENSG00000173456	RNF26	-0.411	2.484	-2.471	0.016
ENSG00000173588	CEP83	0.305	5.033	2.770	0.007
ENSG00000173825	TIGD3	-0.424	-0.007	-2.022	0.046
ENSG00000173826	KCNH6	-0.893	-1.442	-2.500	0.014
ENSG00000173868	PHOSPHO1	-0.510	-0.107	-2.324	0.023
ENSG00000174038	C9orf131	-0.335	1.131	-1.999	0.049
ENSG00000174123	TLR10	0.629	1.179	1.999	0.049
ENSG00000174173	TRMT10C	0.318	4.625	2.104	0.038
ENSG00000174231	PRPF8	-0.288	8.119	-3.500	0.001
ENSG00000174306	ZHX3	-0.201	7.177	-2.678	0.009
ENSG00000174521	TTC9B	0.324	3.281	2.160	0.034
ENSG00000174574	AKIRIN1	0.189	5.647	2.149	0.035
ENSG00000174611	KY	-0.250	2.458	-2.146	0.035
ENSG00000174721	FGFBP3	0.363	1.299	2.484	0.015
ENSG00000174996	KLC2	-0.342	5.086	-2.126	0.037
ENSG00000175115	PACS1	-0.206	5.758	-2.429	0.017
ENSG00000175324	LSM1	0.330	2.930	2.505	0.014
ENSG00000175354	PTPN2	0.194	4.927	2.136	0.036
ENSG00000175455	CCDC14	0.219	6.423	2.153	0.034
ENSG00000175536	LIPT2	-0.409	0.096	-2.304	0.024
ENSG00000175938	ORAI3	-0.412	0.239	-2.469	0.016
ENSG00000175946	KLHL38	-0.965	-1.771	-2.308	0.024
ENSG00000175984	DENND2C	0.321	2.171	2.239	0.028
ENSG00000176049	JAKMIP2	0.161	6.900	2.062	0.042
ENSG00000176102	CSTF3	0.210	4.807	2.172	0.033
ENSG00000176153	GPX2	-0.434	0.188	-2.159	0.034
ENSG00000176182	MYPOP	-0.290	2.484	-2.073	0.041
ENSG00000176358	TAC4	-0.535	-0.782	-2.329	0.022
ENSG00000176435	CLEC14A	0.331	1.971	2.041	0.044
ENSG00000176584	DMBT1P1	0.756	-0.445	2.612	0.011
ENSG00000176641	RNF152	0.351	5.565	2.745	0.007
ENSG00000176681	LRRC37A	-0.662	3.404	-2.400	0.019
ENSG00000176853	FAM91A1	0.243	5.657	2.081	0.041
ENSG00000176857	GJA1P1	-0.440	2.400	-2.684	0.009
ENSG00000176887	SOX11	0.323	3.270	2.704	0.008
ENSG00000176974	SHMT1	-0.372	2.161	-2.541	0.013
ENSG00000176994	SMCR8	-0.155	5.572	-2.542	0.013
ENSG00000177225	PDDC1	0.193	4.930	2.098	0.039
ENSG00000177275	OR2AJ1	0.949	-1.751	2.575	0.012
ENSG00000177301	KCNA2	-0.235	8.631	-2.417	0.018
ENSG00000177459	ERICH5	0.446	-0.044	2.188	0.031
ENSG00000177535	OR2B11	0.776	-1.718	2.627	0.010
ENSG00000177675	CD163L1	0.418	2.636	2.674	0.009
ENSG00000177692	DNAJC28	0.297	3.459	2.085	0.040
ENSG00000177707	NECTIN3	0.390	6.125	2.683	0.009
ENSG00000177853	ZNF518A	0.247	6.569	2.483	0.015
ENSG00000177868	SVBP	0.252	2.862	2.281	0.025

ENSG00000177879	AP3S1	0.282	5.813	2.236	0.028
ENSG00000177932	ZNF354C	0.337	4.558	2.919	0.005
ENSG00000177993	ZNRF3-AS1	-0.433	0.521	-2.107	0.038
ENSG00000177994	C2orf73	0.273	0.568	2.024	0.046
ENSG00000178105	DDX10	0.199	5.688	2.200	0.031
ENSG00000178163	ZNF518B	0.227	6.409	2.747	0.007
ENSG00000178177	LCORL	0.289	5.473	2.819	0.006
ENSG00000178338	ZNF354B	0.191	4.300	2.223	0.029
ENSG00000178385	PLEKHM3	-0.161	7.068	-2.617	0.011
ENSG00000178719	GRINA	-0.399	5.384	-2.698	0.008
ENSG00000178734	LMO7DN	-0.683	-2.562	-2.057	0.043
ENSG00000178919	FOXE1	-0.997	-1.946	-2.574	0.012
ENSG00000179058	C9orf50	-0.847	-2.636	-2.197	0.031
ENSG00000179066	CTD-2527I21.15	-0.687	-2.128	-2.103	0.039
ENSG00000179134	SAMD4B	-0.212	5.469	-1.992	0.050
ENSG00000179270	C2orf71	-0.430	-0.195	-2.087	0.040
ENSG00000179314	WSCD1	0.251	4.902	2.228	0.029
ENSG00000179409	GEMIN4	-0.247	2.831	-2.219	0.029
ENSG00000179546	HTR1D	-0.426	0.435	-2.453	0.016
ENSG00000179841	AKAP5	0.324	7.188	2.038	0.045
ENSG00000179889	PDXDC1	0.185	6.334	2.539	0.013
ENSG00000179918	SEPHS2	-0.205	3.886	-2.035	0.045
ENSG00000179981	TSHZ1	-0.189	5.341	-2.433	0.017
ENSG00000180233	ZNRF2	0.260	4.536	2.141	0.035
ENSG00000180304	OAZ2	-0.131	5.452	-2.368	0.020
ENSG00000180329	CCDC43	0.251	4.751	2.040	0.045
ENSG00000180776	ZDHHC20	0.301	6.222	2.517	0.014
ENSG00000180817	PPA1	0.424	5.489	2.308	0.024
ENSG00000180828	BHLHE22	0.613	3.206	2.843	0.006
ENSG00000180878	C11orf42	-0.380	0.749	-2.499	0.014
ENSG00000181038	METTL23	0.242	2.169	2.149	0.035
ENSG00000181085	MAPK15	0.892	0.094	2.854	0.005
ENSG00000181222	POLR2A	-0.301	6.871	-3.068	0.003
ENSG00000181392	SYNE4	0.487	-0.581	2.077	0.041
ENSG00000181458	TMEM45A	0.427	0.266	2.320	0.023
ENSG00000181588	MEX3D	0.309	1.606	2.107	0.038
ENSG00000181610	MRPS23	0.226	6.083	2.021	0.047
ENSG00000181924	COA4	0.226	2.554	2.195	0.031
ENSG00000182004	SNRPE	0.369	3.232	2.278	0.025
ENSG00000182070	OR52A1	-1.072	-2.058	-2.299	0.024
ENSG00000182107	TMEM30B	0.650	-0.639	2.240	0.028
ENSG00000182257	PRR34	-0.407	0.312	-2.425	0.018
ENSG00000182324	KCNJ14	-0.378	0.604	-2.078	0.041
ENSG00000182347	PDSS1P1	-0.348	0.838	-2.001	0.049
ENSG00000182670	TTC3	0.286	9.438	2.998	0.004
ENSG00000182752	PAPPA	-0.351	2.423	-3.119	0.002
ENSG00000182771	GRID1	-0.156	4.930	-2.405	0.018

ENSG00000182934	SRPRA	-0.231	5.306	-2.791	0.007
ENSG00000183049	CAMK1D	-0.164	7.840	-2.123	0.037
ENSG00000183186	C2CD4C	0.450	3.136	2.653	0.010
ENSG00000183324	REC114	-0.536	-1.177	-2.317	0.023
ENSG00000183604	SMG1P5	-0.579	4.371	-2.366	0.020
ENSG00000183648	NDUFB1	0.336	4.576	2.213	0.030
ENSG00000183662	FAM19A1	0.314	5.705	2.151	0.034
ENSG00000183665	TRMT12	0.177	4.694	2.194	0.031
ENSG00000183765	CHEK2	0.490	0.371	2.968	0.004
ENSG00000183778	B3GALT5	-0.536	2.668	-3.997	0.000
ENSG00000183779	ZNF703	-0.342	1.760	-2.009	0.048
ENSG00000183856	IQGAP3	-0.529	0.603	-2.295	0.024
ENSG00000183955	KMT5A	0.152	4.196	2.081	0.040
ENSG00000184307	ZDHHC23	0.214	6.068	2.111	0.038
ENSG00000184500	PROS1	0.340	3.592	2.739	0.008
ENSG00000184544	DHRS7C	-0.927	-3.055	-2.169	0.033
ENSG00000184575	XPOT	0.252	6.901	2.215	0.029
ENSG00000184635	ZNF93	0.331	2.055	2.574	0.012
ENSG00000184669	OR7E14P	-0.664	-1.689	-2.186	0.032
ENSG00000184708	EIF4ENIF1	-0.138	5.191	-2.519	0.014
ENSG00000184984	CHRM5	-0.601	1.220	-2.467	0.016
ENSG00000185031	SLC2A3P2	-0.394	2.504	-2.566	0.012
ENSG00000185053	SGCZ	0.238	2.932	2.018	0.047
ENSG00000185088	RPS27L	0.228	4.699	2.035	0.045
ENSG00000185164	NOMO2	-0.464	5.652	-3.037	0.003
ENSG00000185236	RAB11B	-0.314	3.984	-2.435	0.017
ENSG00000185290	NUPR2	0.883	-1.113	2.562	0.012
ENSG00000185619	PCGF3	0.116	5.791	2.236	0.028
ENSG00000185666	SYN3	-0.359	4.841	-2.659	0.009
ENSG00000185697	MYBL1	0.223	3.118	2.069	0.042
ENSG00000185722	ANKFY1	-0.149	6.059	-2.644	0.010
ENSG00000185963	BICD2	0.126	5.982	2.075	0.041
ENSG00000186297	GABRA5	0.321	7.037	2.419	0.018
ENSG00000186369	LINC00643	0.520	5.464	2.522	0.014
ENSG00000186409	CCDC30	0.189	5.620	2.199	0.031
ENSG00000186470	BTN3A2	0.400	2.754	2.150	0.034
ENSG00000186638	KIF24	-0.240	2.447	-2.471	0.016
ENSG00000186765	FSCN2	-0.855	-1.800	-2.723	0.008
ENSG00000187079	TEAD1	-0.195	5.812	-2.021	0.047
ENSG00000187240	DYNC2H1	0.193	7.497	2.278	0.025
ENSG00000187258	NPSR1	-1.316	-1.369	-3.366	0.001
ENSG00000187323	DCC	-0.252	5.140	-2.319	0.023
ENSG00000187398	LUZP2	0.421	5.749	3.154	0.002
ENSG00000187416	LHFPL3	0.362	3.840	2.351	0.021
ENSG00000187474	FPR3	-0.573	-0.113	-1.996	0.049
ENSG00000187595	ZNF385C	-0.626	-0.355	-2.847	0.006
ENSG00000187605	TET3	-0.224	5.560	-2.928	0.004

ENSG00000187624	C17orf97	-0.327	1.914	-2.076	0.041
ENSG00000187672	ERC2	0.208	8.536	2.593	0.011
ENSG00000187783	TMEM72	-0.677	-2.298	-2.262	0.026
ENSG00000187987	ZSCAN23	0.225	3.267	2.059	0.043
ENSG00000187994	RINL	0.251	1.534	2.064	0.042
ENSG00000188070	C11orf95	0.235	4.892	2.714	0.008
ENSG00000188167	TMPPE	-0.228	3.493	-2.113	0.038
ENSG00000188171	ZNF626	0.296	5.045	2.822	0.006
ENSG00000188227	ZNF793	0.196	5.099	2.320	0.023
ENSG00000188243	COMMD6	0.300	3.561	2.087	0.040
ENSG00000188315	C3orf62	-0.195	3.169	-2.248	0.027
ENSG00000188342	GTF2F2	0.272	4.272	2.431	0.017
ENSG00000188493	C19orf54	-0.565	0.264	-2.780	0.007
ENSG00000188529	SRSF10	0.168	6.353	2.173	0.033
ENSG00000188612	SUMO2	0.333	5.318	2.133	0.036
ENSG00000188641	DPYD	0.238	5.695	2.615	0.011
ENSG00000188690	UROS	0.207	5.503	2.116	0.037
ENSG00000188779	SKOR1	-0.522	-0.577	-2.594	0.011
ENSG00000188786	MTF1	-0.320	4.241	-3.936	0.000
ENSG00000188859	FAM78B	-0.252	3.401	-2.134	0.036
ENSG00000188868	ZNF563	0.295	2.114	2.766	0.007
ENSG00000188895	MSL1	0.148	6.843	2.266	0.026
ENSG00000189114	BLOC1S3	-0.326	1.749	-2.681	0.009
ENSG00000189184	PCDH18	0.375	3.883	2.486	0.015
ENSG00000189238	LINC00943	0.615	1.602	2.766	0.007
ENSG00000189266	PNRC2	0.221	5.919	2.086	0.040
ENSG00000196072	BLOC1S2	0.284	5.436	2.024	0.046
ENSG00000196159	FAT4	0.304	5.522	3.001	0.004
ENSG00000196208	GREB1	-0.267	4.849	-3.124	0.002
ENSG00000196341	OR8D1	-0.684	-1.832	-2.115	0.037
ENSG00000196350	ZNF729	-0.933	-2.196	-2.070	0.042
ENSG00000196367	TRRAP	-0.171	6.589	-2.297	0.024
ENSG00000196531	NACA	0.309	7.108	2.482	0.015
ENSG00000196730	DAPK1	-0.209	6.555	-2.319	0.023
ENSG00000196968	FUT11	0.227	4.101	2.633	0.010
ENSG00000197006	METTL9	0.268	6.466	2.186	0.032
ENSG00000197077	KIAA1671	-0.254	6.647	-3.450	0.001
ENSG00000197081	IGF2R	-0.125	6.240	-2.050	0.044
ENSG00000197265	GTF2E2	0.256	3.718	2.679	0.009
ENSG00000197299	BLM	0.279	3.313	2.245	0.027
ENSG00000197322	C17orf102	-0.442	1.712	-2.412	0.018
ENSG00000197360	ZNF98	-0.452	1.824	-2.160	0.034
ENSG00000197372	ZNF675	0.272	5.111	2.513	0.014
ENSG00000197381	ADARB1	-0.260	6.069	-2.943	0.004
ENSG00000197479	PCDHB11	-0.339	3.434	-2.698	0.008
ENSG00000197584	KCNMB2	0.205	3.029	2.101	0.039
ENSG00000197696	NMB	0.626	-1.264	2.170	0.033

ENSG00000197705	KLHL14	0.512	1.790	2.342	0.022
ENSG00000197728	RPS26	0.558	2.026	2.262	0.026
ENSG00000197748	CFAP43	0.329	3.757	2.686	0.009
ENSG00000198053	SIRPA	-0.197	7.635	-2.476	0.015
ENSG00000198056	PRIM1	0.301	1.485	2.014	0.047
ENSG00000198093	ZNF649	0.191	4.034	2.202	0.030
ENSG00000198153	ZNF849P	0.815	-2.042	2.430	0.017
ENSG00000198155	ZNF876P	0.352	4.059	3.062	0.003
ENSG00000198298	ZNF485	0.310	2.281	2.702	0.008
ENSG00000198369	SPRED2	-0.189	5.595	-2.296	0.024
ENSG00000198453	ZNF568	0.145	4.853	2.072	0.041
ENSG00000198554	WDHD1	0.304	3.763	2.688	0.009
ENSG00000198612	COPS8	0.286	6.266	2.055	0.043
ENSG00000198668	CALM1	0.232	12.067	2.184	0.032
ENSG00000198673	FAM19A2	0.365	6.361	2.190	0.031
ENSG00000198677	TTC37	0.213	7.526	2.078	0.041
ENSG00000198707	CEP290	0.249	7.034	2.242	0.028
ENSG00000198723	C19orf45	-0.677	-1.981	-2.245	0.027
ENSG00000198794	SCAMP5	-0.234	7.288	-2.293	0.024
ENSG00000198829	SUCNR1	1.225	-0.636	2.594	0.011
ENSG00000198843	SELT	0.332	6.635	2.272	0.026
ENSG00000198846	TOX	-0.292	4.904	-2.802	0.006
ENSG00000198856	OSTC	0.407	3.253	2.649	0.010
ENSG00000198898	CAPZA2	0.398	7.066	2.241	0.028
ENSG00000198944	SOWAHA	0.417	5.783	2.986	0.004
ENSG00000199627	RNU6-1010P	-0.660	-1.148	-2.683	0.009
ENSG00000200296	RNU1-83P	0.961	-3.244	2.465	0.016
ENSG00000200638	SNORD115-37	0.627	-1.725	2.310	0.023
ENSG00000200661	SNORD116-10	-0.809	-1.699	-2.319	0.023
ENSG00000200783	RN7SKP180	0.740	1.138	2.405	0.018
ENSG00000201027	RN7SKP107	1.107	-3.280	2.472	0.016
ENSG00000201358	RN7SKP193	0.854	-2.420	1.992	0.050
ENSG00000201499	RNU6-312P	0.566	-1.735	2.094	0.039
ENSG00000203326	ZNF525	0.300	4.047	2.529	0.013
ENSG00000203643	AC012456.3	-0.692	-1.197	-2.075	0.041
ENSG00000203647	RP11-320P11.1	-0.555	-1.260	-2.053	0.043
ENSG00000203667	COX20	-0.314	2.072	-2.271	0.026
ENSG00000203799	CCDC162P	0.315	2.166	2.677	0.009
ENSG00000203867	RBM20	-0.462	2.071	-3.190	0.002
ENSG00000204569	PPP1R10	-0.160	5.432	-2.130	0.036
ENSG00000204581	AC096670.3	-0.568	-1.562	-2.346	0.021
ENSG00000204683	C10orf113	-0.482	0.757	-2.685	0.009
ENSG00000204764	RANBP17	0.277	4.064	2.121	0.037
ENSG00000204789	ZNF204P	0.395	6.356	2.856	0.005
ENSG00000204832	ST8SIA6-AS1	-0.627	-1.757	-2.128	0.036
ENSG00000204929	AC074391.1	0.291	1.285	2.081	0.041
ENSG00000204990	RP5-842K16.1	-0.422	1.221	-1.990	0.050

ENSG00000205038	PKHD1L1	-0.886	-1.414	-2.020	0.047
ENSG00000205108	FAM205A	-0.480	-0.596	-2.188	0.031
ENSG00000205189	ZBTB10	0.233	6.370	2.710	0.008
ENSG00000205302	SNX2	0.264	5.503	2.202	0.030
ENSG00000205571	SMN2	-0.838	2.852	-2.152	0.034
ENSG00000205581	HMGN1	0.226	5.248	2.633	0.010
ENSG00000205634	LINC00898	-0.790	-0.212	-2.730	0.008
ENSG00000205643	CDPF1	0.364	0.862	2.039	0.045
ENSG00000205758	CRYZL1	0.186	5.283	2.076	0.041
ENSG00000205940	HSP90AB2P	-0.524	3.363	-2.142	0.035
ENSG00000205981	DNAJC19	0.284	4.756	2.028	0.046
ENSG00000206014	OR7E161P	-0.771	-1.994	-2.487	0.015
ENSG00000206052	DOK6	0.326	7.924	2.287	0.025
ENSG00000206644	RNU6-1279P	-0.920	-2.559	-2.836	0.006
ENSG00000206739	Y_RNA	-0.709	-2.136	-2.784	0.007
ENSG00000206991	RNU6-610P	-0.592	-1.689	-2.533	0.013
ENSG00000207129	RNA5SP187	-0.634	-0.799	-3.168	0.002
ENSG00000207233	SNORA37	-0.861	-2.209	-2.560	0.012
ENSG00000207349	RNVU1-17	1.078	-3.471	2.117	0.037
ENSG00000207428	RNU6-95P	0.854	-1.670	2.279	0.025
ENSG00000207561	MIR635	-0.764	-2.236	-2.865	0.005
ENSG00000207750	MIR553	0.770	-2.115	2.170	0.033
ENSG00000211445	GPX3	-0.401	3.539	-2.331	0.022
ENSG00000212181	SNORA48	-0.689	-1.432	-2.975	0.004
ENSG00000212205	Y_RNA	-0.620	-2.323	-2.134	0.036
ENSG00000212864	RNF208	-0.488	1.421	-2.126	0.037
ENSG00000213058	RP4-765C7.2	-0.627	0.398	-2.611	0.011
ENSG00000213063	RPL29P7	-0.855	-2.339	-2.173	0.033
ENSG00000213088	ACKR1	-0.327	2.407	-2.122	0.037
ENSG00000213221	DNLZ	-0.734	-2.182	-2.052	0.043
ENSG00000213301	HMGB3P11	-0.624	-1.406	-2.384	0.019
ENSG00000213484	EIF4A1P8	-0.416	-0.588	-2.173	0.033
ENSG00000213513	RP13-39P12.2	-0.767	-1.529	-3.193	0.002
ENSG00000213519	AC132008.1	-0.759	-1.828	-2.617	0.011
ENSG00000213551	DNAJC9	0.248	4.337	2.707	0.008
ENSG00000213600	XXcos-LUCA16.1	0.886	-2.517	2.517	0.014
ENSG00000213672	NCKIPSD	-0.328	4.728	-2.204	0.030
ENSG00000213741	RPS29	0.219	4.867	2.621	0.010
ENSG00000213785	RP11-449L13.2	-0.461	-0.858	-2.100	0.039
ENSG00000213871	TAF9BP1	-0.291	1.530	-2.099	0.039
ENSG00000213889	PPM1N	0.571	-0.852	2.412	0.018
ENSG00000213891	RPL3P6	-0.383	0.794	-2.079	0.041
ENSG00000213949	ITGA1	0.364	4.365	2.080	0.041
ENSG00000213976	CTD-2561J22.2	0.386	1.533	2.329	0.022
ENSG00000214087	ARL16	0.207	3.888	2.192	0.031
ENSG00000214194	LINC00998	0.392	3.622	2.483	0.015
ENSG00000214211	CTAGE14P	-0.328	1.311	-2.469	0.016

ENSG00000214331	RP11-252A24.2	-0.310	3.323	-2.389	0.019
ENSG00000214357	NEURL1B	0.311	4.389	2.214	0.030
ENSG00000214381	LINC00488	0.650	0.917	2.127	0.036
ENSG00000214456	PLIN5	-0.750	1.061	-4.034	0.000
ENSG00000214549	SDHCP2	-0.574	-0.448	-2.700	0.008
ENSG00000214753	HNRNPUL2	-0.302	4.915	-3.255	0.002
ENSG00000214796	RP11-480I12.5	-0.308	0.649	-2.034	0.045
ENSG00000214842	RAD51AP2	0.369	1.953	2.097	0.039
ENSG00000215004	MESTP4	-0.530	-0.777	-2.136	0.036
ENSG00000215057	PKMP5	-0.720	0.821	-2.242	0.028
ENSG00000215187	FAM166B	-0.728	-1.757	-2.203	0.030
ENSG00000215190	LINC00680	0.255	3.320	2.359	0.021
ENSG00000215380	AC090625.1	-0.541	1.971	-2.058	0.043
ENSG00000215417	MIR17HG	0.595	0.113	2.405	0.018
ENSG00000215452	ZNF663P	-0.936	-2.306	-2.101	0.039
ENSG00000215458	AATBC	-0.470	0.655	-2.059	0.043
ENSG00000215493	XXbac-B562F10.1	-0.402	2.642	-2.144	0.035
ENSG00000216802	RP11-390P2.2	-0.639	1.486	-2.075	0.041
ENSG00000217227	RP1-319M7.2	-0.479	-0.572	-2.031	0.045
ENSG00000217801	RP11-465B22.3	0.381	1.125	2.486	0.015
ENSG00000218428	NIP7P3	-0.484	-0.985	-2.171	0.033
ENSG00000218857	RP1-209B5.2	-0.540	-0.314	-2.137	0.036
ENSG00000219445	RP11-3B12.3	-0.794	-1.437	-3.211	0.002
ENSG00000219941	KRT18P50	-0.725	-1.531	-2.251	0.027
ENSG00000219951	RP11-378G13.2	-0.538	-0.553	-2.440	0.017
ENSG00000220205	VAMP2	-0.286	7.257	-2.728	0.008
ENSG00000220884	MESTP1	-0.420	1.846	-2.015	0.047
ENSG00000221676	RNU6ATAC	-0.918	0.085	-2.640	0.010
ENSG00000221944	TIGD1	0.223	3.067	2.545	0.013
ENSG00000222069	RN7SKP285	-0.784	-2.478	-2.467	0.016
ENSG00000222404	RN7SKP281	1.272	-1.962	2.114	0.038
ENSG00000222974	RN7SKP228	0.670	-2.069	2.050	0.043
ENSG00000223341	RN7SKP3	0.883	-4.358	2.145	0.035
ENSG00000223427	AC016716.1	-0.432	2.203	-2.614	0.011
ENSG00000223459	TCAF1P1	-0.344	3.699	-2.002	0.049
ENSG00000223492	RP11-15M15.1	-0.648	-1.276	-2.346	0.021
ENSG00000223500	AC083862.6	-0.435	0.954	-2.028	0.046
ENSG00000223519	KIF28P	-0.384	0.669	-2.296	0.024
ENSG00000223528	RP11-223P11.3	-0.546	-0.554	-2.194	0.031
ENSG00000223536	AC008069.1	0.920	-1.594	2.227	0.029
ENSG00000223547	ZNF844	0.184	4.533	2.019	0.047
ENSG00000223548	AC034228.3	-0.434	0.061	-2.931	0.004
ENSG00000223583	RP3-476K8.4	0.606	-1.384	2.145	0.035
ENSG00000223656	HMGB3P10	0.832	-2.225	2.095	0.039
ENSG00000223756	TSSC2	-0.554	-0.727	-2.295	0.024
ENSG00000223899	SEC13P1	-0.509	-0.751	-2.379	0.020
ENSG00000223930	RP11-33A14.1	0.533	2.377	2.073	0.041

ENSG00000224414	AC010886.2	-0.494	0.606	-2.324	0.023
ENSG00000224448	GS1-259H13.7	-0.405	-0.597	-2.031	0.045
ENSG00000224479	AC136289.1	-0.273	2.145	-2.101	0.039
ENSG00000224563	LPP-AS1	-0.711	-2.132	-2.114	0.037
ENSG00000224609	HSD52	-0.384	0.822	-2.339	0.022
ENSG00000224635	RP4-564F22.5	0.792	-0.697	2.452	0.016
ENSG00000224699	LAMTOR5-AS1	-0.216	2.360	-2.042	0.044
ENSG00000224957	LINC01266	-0.489	1.006	-2.127	0.036
ENSG00000225017	AP004289.1	-0.430	-0.379	-2.239	0.028
ENSG00000225127	LINC00237	0.534	-0.044	2.584	0.012
ENSG00000225171	DUTP6	-0.625	-1.123	-2.574	0.012
ENSG00000225194	LINC00092	-0.440	1.333	-2.539	0.013
ENSG00000225217	HSPA7	-1.270	-1.406	-2.393	0.019
ENSG00000225292	RP11-57H14.3	-0.462	-0.907	-2.108	0.038
ENSG00000225335	XXbac-B476C20.9	0.497	-0.215	2.732	0.008
ENSG00000225349	RP11-820K3.2	-0.571	-1.062	-2.178	0.032
ENSG00000225439	BOLA3-AS1	-0.206	2.986	-2.116	0.037
ENSG00000225451	RP11-340I6.5	1.161	-2.114	2.010	0.048
ENSG00000225648	SBDSP1	0.179	4.297	2.102	0.039
ENSG00000225649	AC064875.2	0.364	1.615	2.027	0.046
ENSG00000225697	SLC26A6	0.317	1.440	2.207	0.030
ENSG00000225727	RP11-71C5.2	-0.548	0.118	-2.616	0.011
ENSG00000225921	NOL7	0.276	4.709	2.658	0.009
ENSG00000225963	AC009950.2	-0.377	0.230	-2.307	0.024
ENSG00000226237	GAS1RR	-0.418	0.587	-2.272	0.026
ENSG00000226239	RP1-310O13.7	0.509	-0.647	1.992	0.050
ENSG00000226272	ARHGAP26-AS1	-0.522	-0.775	-2.001	0.049
ENSG00000226288	OR52I2	-0.781	-1.358	-2.532	0.013
ENSG00000226519	LINC00390	0.550	0.607	2.203	0.030
ENSG00000226705	SDCBPP1	-0.830	-1.611	-2.409	0.018
ENSG00000226726	TMEM256P2	-0.956	-2.419	-2.571	0.012
ENSG00000226751	AF127936.5	0.843	0.569	2.587	0.011
ENSG00000226797	AC015923.1	-0.486	-0.001	-2.112	0.038
ENSG00000226833	AC097724.3	-0.761	-1.952	-2.481	0.015
ENSG00000226874	AC005154.8	-0.672	-0.585	-2.999	0.004
ENSG00000226939	AC062021.1	0.554	-1.156	2.121	0.037
ENSG00000227256	MIS18A-AS1	0.651	-1.838	2.293	0.024
ENSG00000227376	FTH1P16	-0.453	2.255	-2.056	0.043
ENSG00000227695	DNMBP-AS1	-0.833	-1.856	-2.517	0.014
ENSG00000227745	AC098826.5	0.643	2.935	3.436	0.001
ENSG00000227770	RPL7P12	0.835	-2.265	2.054	0.043
ENSG00000227782	CTC-529I10.2	-0.397	0.545	-2.131	0.036
ENSG00000227805	RP11-248J23.5	-1.193	-2.962	-2.830	0.006
ENSG00000227811	FAM212B-AS1	0.512	-0.251	2.451	0.016
ENSG00000227848	SUCLA2-AS1	0.540	-0.495	2.887	0.005
ENSG00000227914	RP11-130C19.3	-0.942	-1.635	-2.450	0.016
ENSG00000228024	CYP1D1P	-0.775	-0.686	-2.684	0.009

ENSG00000228061	Z83001.1	-0.352	1.700	-2.194	0.031
ENSG00000228223	HCG11	0.250	4.433	2.106	0.038
ENSG00000228351	AC018832.1	-0.420	-0.479	-2.230	0.028
ENSG00000228549	RP11-108M9.3	-0.599	-1.112	-2.093	0.039
ENSG00000228592	D21S2088E	0.924	-1.962	2.089	0.040
ENSG00000228625	RP11-1B20.1	-0.496	-0.095	-2.144	0.035
ENSG00000228718	LINC02521	-0.739	-2.261	-2.249	0.027
ENSG00000228729	RP11-211A18.2	-0.487	0.179	-2.466	0.016
ENSG00000228818	RP11-567G24.1	-0.455	3.635	-2.112	0.038
ENSG00000228824	MIR4500HG	0.369	3.927	2.283	0.025
ENSG00000229029	CDCA4P1	-0.406	0.025	-2.049	0.044
ENSG00000229105	ASTN2-AS1	-0.447	0.815	-2.794	0.006
ENSG00000229287	FABP5P4	-0.690	-1.771	-2.097	0.039
ENSG00000229327	RP11-135A24.2	0.554	-0.805	2.254	0.027
ENSG00000229393	RP3-395M20.3	0.750	-2.210	2.085	0.040
ENSG00000229502	RP11-52J3.2	-0.463	-0.090	-2.303	0.024
ENSG00000229585	RPL21P44	-0.381	-0.391	-2.116	0.037
ENSG00000229672	RP11-184A2.3	-0.445	-0.112	-2.137	0.036
ENSG00000229896	RP11-456H18.2	-0.826	-2.195	-2.060	0.043
ENSG00000230006	ANKRD36BP2	0.252	5.911	2.232	0.028
ENSG00000230223	ATXN8OS	0.616	-1.507	2.190	0.031
ENSG00000230237	AC010975.2	-0.474	-0.230	-2.339	0.022
ENSG00000230513	THAP7-AS1	-0.361	-0.067	-2.018	0.047
ENSG00000230552	AC092162.1	-0.471	0.324	-2.299	0.024
ENSG00000230561	CCDC192	-0.480	0.237	-2.572	0.012
ENSG00000230725	RP4-738P15.1	0.406	0.003	2.001	0.049
ENSG00000230849	GOT2P2	-0.548	0.606	-2.463	0.016
ENSG00000230894	RP11-67K19.3	-0.479	2.170	-3.284	0.002
ENSG00000230989	HSBP1	0.305	6.381	2.303	0.024
ENSG00000231006	RPL7P32	-0.491	0.927	-2.288	0.025
ENSG00000231007	CDC20P1	-0.697	-1.309	-2.515	0.014
ENSG00000231169	EEF1B2P1	-0.402	1.037	-2.069	0.042
ENSG00000231256	C17orf105	-0.640	-2.157	-2.163	0.033
ENSG00000231336	AC017006.3	-0.726	-1.751	-2.516	0.014
ENSG00000231378	NDUFAF4P4	-0.437	-0.029	-2.080	0.041
ENSG00000231424	RP1-45C12.1	0.857	-3.268	2.009	0.048
ENSG00000231500	RPS18	0.394	5.159	2.031	0.045
ENSG00000231561	CEACAMP5	-0.610	-0.852	-3.213	0.002
ENSG00000231606	RP11-344F13.1	-0.635	-0.454	-2.671	0.009
ENSG00000231663	RP5-827C21.4	-0.408	0.541	-2.233	0.028
ENSG00000231905	SETP10	-0.656	-1.266	-2.424	0.018
ENSG00000231942	HNRNPA1P36	-0.516	-1.393	-2.006	0.048
ENSG00000231965	AF131215.1	-0.615	0.434	-2.684	0.009
ENSG00000231995	RP11-111F5.2	-0.799	-0.415	-2.475	0.015
ENSG00000232006	AC005537.2	-0.466	0.054	-2.380	0.020
ENSG00000232027	RP11-275F13.3	-0.486	-0.148	-2.511	0.014
ENSG00000232043	RP4-530I15.9	-0.411	1.521	-2.040	0.044

ENSG00000232098	CTD-2619J13.14	-0.301	2.094	-2.059	0.043
ENSG00000232136	DUXAP7	-0.725	-1.797	-2.162	0.033
ENSG00000232142	RP11-367H5.8	-0.597	-1.087	-2.454	0.016
ENSG00000232196	MTRNR2L4	-0.568	-1.043	-2.076	0.041
ENSG00000232287	SLC6A1-AS1	-0.362	1.340	-2.208	0.030
ENSG00000232342	RP11-46O21.2	-0.606	-0.124	-2.668	0.009
ENSG00000232344	AC087163.2	-0.382	-0.262	-2.453	0.016
ENSG00000232439	RPL18AP7	-0.430	0.936	-2.491	0.015
ENSG00000232455	LARS2-AS1	-0.758	-1.997	-2.622	0.010
ENSG00000232479	AC010900.2	0.415	-0.549	2.207	0.030
ENSG00000232490	OSBPL10-AS1	-0.700	-0.203	-3.546	0.001
ENSG00000232528	RP4-673D20.3	-0.336	2.642	-2.207	0.030
ENSG00000232630	PRPS1P2	-0.270	1.683	-2.218	0.029
ENSG00000232751	RP11-296O14.1	-0.384	0.335	-2.033	0.045
ENSG00000232756	RP5-1185I7.1	-0.680	-2.324	-2.121	0.037
ENSG00000232759	AC002480.3	0.584	-1.672	1.992	0.050
ENSG00000232768	RP11-201O14.2	0.511	-1.639	2.039	0.045
ENSG00000232939	RP11-406O23.2	-0.719	-1.710	-2.009	0.048
ENSG00000232970	POLHP1	-0.539	-1.101	-2.001	0.049
ENSG00000232977	LINC00327	0.336	0.678	2.041	0.044
ENSG00000233016	SNHG7	0.383	2.204	2.284	0.025
ENSG00000233184	RP11-421L21.3	0.520	1.592	3.162	0.002
ENSG00000233332	RP4-799P18.2	-0.462	-0.613	-2.076	0.041
ENSG00000233436	BTBD18	-0.284	1.767	-2.033	0.045
ENSG00000233464	NANOGP11	-0.865	-1.084	-2.758	0.007
ENSG00000233487	RPSAP69	-0.290	1.515	-1.992	0.050
ENSG00000233503	HNRNPLP1	-0.477	1.641	-2.499	0.014
ENSG00000233524	RANP8	-0.576	-1.534	-2.109	0.038
ENSG00000233615	HNRNPA1P42	-0.451	1.223	-2.060	0.043
ENSG00000233622	CYP2T1P	-0.446	0.533	-2.039	0.045
ENSG00000233693	RP11-506O24.1	-0.389	1.222	-2.236	0.028
ENSG00000233892	PAIP1P1	-0.631	-0.775	-2.166	0.033
ENSG00000233994	GDI2P2	-0.317	3.772	-2.180	0.032
ENSG00000234084	RP3-388E23.2	-0.330	2.302	-2.058	0.043
ENSG00000234127	TRIM26	-0.286	4.321	-2.544	0.013
ENSG00000234492	RPL34-AS1	-0.313	0.723	-2.016	0.047
ENSG00000234509	AP000253.1	-0.655	-1.282	-2.527	0.013
ENSG00000234593	RP4-704D23.1	-0.479	1.709	-2.201	0.031
ENSG00000234608	MAPKAPK5-AS1	0.301	3.575	2.566	0.012
ENSG00000234685	NUS1P2	0.746	-2.270	2.247	0.027
ENSG00000234741	GAS5	0.314	4.302	2.607	0.011
ENSG00000235020	RP11-109I13.2	-0.345	1.609	-2.053	0.043
ENSG00000235092	ID2-AS1	-0.324	1.501	-2.407	0.018
ENSG00000235162	C12orf75	0.578	1.461	2.700	0.008
ENSG00000235204	RP11-121A14.2	-0.824	-1.916	-2.536	0.013
ENSG00000235272	FAM103A2P	-0.436	1.958	-2.361	0.021
ENSG00000235278	ZNF652P1	-0.668	-0.153	-3.147	0.002

ENSG00000235286	AP000925.2	-0.459	-0.319	-2.118	0.037
ENSG00000235347	RP1-37M3.8	-0.433	0.315	-2.026	0.046
ENSG00000235478	LINC01664	0.461	0.690	2.792	0.007
ENSG00000235482	RPL21P135	-0.640	-1.712	-2.344	0.021
ENSG00000235597	LINC01102	0.293	2.922	2.333	0.022
ENSG00000235631	RNF148	-0.459	0.851	-2.081	0.041
ENSG00000235740	PHACTR2-AS1	-0.468	-1.227	-2.029	0.046
ENSG00000235768	BRD7P5	-0.578	-0.205	-2.164	0.033
ENSG00000235816	PRELID1P3	-0.701	-1.335	-2.442	0.017
ENSG00000235940	MTND1P21	-0.972	-0.849	-4.132	0.000
ENSG00000235984	GPC5-AS1	-0.851	-1.171	-3.164	0.002
ENSG00000236008	LINC01814	-0.401	0.835	-2.068	0.042
ENSG00000236065	RP1-117O3.2	-0.367	-0.008	-2.008	0.048
ENSG00000236166	RP3-523C21.2	0.490	1.405	2.449	0.016
ENSG00000236200	KDM4A-AS1	-0.415	-0.217	-2.109	0.038
ENSG00000236212	AC009262.2	-0.934	-1.705	-3.408	0.001
ENSG00000236254	MTND4P14	-0.972	-1.333	-2.675	0.009
ENSG00000236259	AC017083.2	-0.624	-2.251	-2.188	0.031
ENSG00000236452	AC123023.1	0.895	-1.171	3.338	0.001
ENSG00000236554	ASNSP3	-0.733	-2.212	-2.084	0.040
ENSG00000236583	AP004290.1	-0.445	-0.728	-2.076	0.041
ENSG00000236668	RP11-85O21.2	-0.369	0.648	-2.248	0.027
ENSG00000236678	LINC00347	-0.961	-3.129	-2.080	0.041
ENSG00000236863	RPL23AP23	-0.624	-1.716	-2.133	0.036
ENSG00000236928	RP11-792A8.1	-0.742	-1.883	-2.117	0.037
ENSG00000236939	BAALC-AS2	-0.486	-0.885	-2.048	0.044
ENSG00000236963	LINC01141	0.383	1.378	2.224	0.029
ENSG00000236977	ANKRD44-IT1	-0.373	0.176	-1.992	0.050
ENSG00000237001	WASF3-AS1	-0.396	0.567	-2.623	0.010
ENSG00000237088	RP11-73E6.2	-0.596	-0.669	-2.495	0.015
ENSG00000237190	CDKN2AIPNL	0.270	4.511	2.171	0.033
ENSG00000237234	RP1-142L7.5	-0.434	-0.483	-2.006	0.048
ENSG00000237250	RP11-193H5.1	1.108	-0.443	2.896	0.005
ENSG00000237316	RP1-288L1.4	0.537	-0.228	2.180	0.032
ENSG00000237440	ZNF737	0.268	5.559	2.378	0.020
ENSG00000237575	PYY2	0.947	-3.043	2.590	0.011
ENSG00000237593	RP11-317B3.2	-0.567	-0.741	-2.002	0.049
ENSG00000237638	LINC02245	-0.923	-1.851	-2.500	0.014
ENSG00000237672	KRR1P1	-0.479	1.042	-2.735	0.008
ENSG00000237734	RP11-298E2.2	-0.727	-1.080	-2.631	0.010
ENSG00000237774	RP11-129B9.1	-0.766	-1.489	-2.503	0.014
ENSG00000237788	RP5-1041C10.3	-0.477	1.272	-2.297	0.024
ENSG00000237807	RP11-400K9.4	0.453	0.522	2.054	0.043
ENSG00000237879	LINC00398	-0.710	-1.704	-2.265	0.026
ENSG00000238065	AC010740.1	-0.520	-0.354	-2.334	0.022
ENSG00000238083	LRRC37A2	-0.496	4.591	-2.043	0.044
ENSG00000238090	AC006195.2	-0.942	-2.101	-2.521	0.014

ENSG00000238230	LINC00391	-0.912	-0.473	-3.035	0.003
ENSG00000238286	SLC35E1P1	-0.379	0.068	-2.017	0.047
ENSG00000238358	RP5-1121E10.2	-0.854	-1.980	-2.318	0.023
ENSG00000238741	SCARNA7	0.484	5.301	2.373	0.020
ENSG00000239332	LINC01119	-0.438	-0.530	-2.087	0.040
ENSG00000239351	NPM1P29	-0.504	0.428	-2.628	0.010
ENSG00000239377	RP4-584D14.6	0.774	-2.556	2.017	0.047
ENSG00000239468	RN7SL569P	1.331	-2.251	3.731	0.000
ENSG00000239494	RN7SL333P	-0.717	-2.200	-2.199	0.031
ENSG00000239508	PLCH1-AS1	-0.517	-1.040	-2.025	0.046
ENSG00000239748	RN7SL795P	-0.619	-1.712	-1.993	0.050
ENSG00000240057	RP11-572M11.4	-0.528	-1.023	-3.338	0.001
ENSG00000240086	RP11-657O9.1	-0.367	0.458	-2.652	0.010
ENSG00000240137	ERICH6-AS1	0.410	0.158	2.393	0.019
ENSG00000240305	RP11-1072C15.1	-0.771	-2.090	-2.657	0.009
ENSG00000240395	RPL5P23	-0.582	1.542	-2.308	0.024
ENSG00000240436	RP11-95I19.1	-0.735	-1.742	-2.182	0.032
ENSG00000240441	RP11-864J10.2	-0.509	-0.503	-2.488	0.015
ENSG00000240470	RN7SL346P	0.872	-3.937	2.291	0.024
ENSG00000240499	RP5-1101C3.1	-0.454	2.690	-2.146	0.035
ENSG00000240661	RP11-174O3.3	-1.223	-0.415	-2.326	0.022
ENSG00000240738	RP1-273G13.1	-1.138	-1.852	-3.479	0.001
ENSG00000240775	RP11-170N16.2	-1.096	-1.680	-3.535	0.001
ENSG00000241032	RN7SL709P	1.291	-3.151	2.652	0.010
ENSG00000241074	RN7SL813P	1.188	-3.308	2.804	0.006
ENSG00000241172	RN7SL70P	0.836	-3.920	2.346	0.021
ENSG00000241345	RP4-630C24.3	0.691	-0.630	2.102	0.039
ENSG00000241487	RN7SL586P	1.076	-3.823	2.775	0.007
ENSG00000241563	CORT	0.525	0.730	3.012	0.003
ENSG00000241756	RN7SL83P	-0.858	-1.423	-2.685	0.009
ENSG00000241772	AC092620.2	-0.542	-0.681	-2.168	0.033
ENSG00000241785	RN7SL390P	0.878	-3.049	2.261	0.026
ENSG00000241973	PI4KA	-0.237	8.526	-2.252	0.027
ENSG00000241978	AKAP2	-0.611	-1.528	-2.300	0.024
ENSG00000242247	ARFGAP3	0.387	4.941	3.314	0.001
ENSG00000242445	RPL7AP11	-0.679	0.019	-2.417	0.018
ENSG00000242525	OR7E100P	-1.173	-1.879	-4.264	0.000
ENSG00000242600	MBL1P	-0.407	-0.047	-2.049	0.044
ENSG00000242622	RP11-18H7.1	0.275	1.222	2.164	0.033
ENSG00000242798	RP11-506M12.1	0.820	-1.543	2.489	0.015
ENSG00000243137	PSG4	-0.613	-0.145	-2.160	0.034
ENSG00000243147	MRPL33	0.300	3.599	2.308	0.023
ENSG00000243370	RN7SL775P	0.637	-1.833	2.217	0.029
ENSG00000243445	RP11-715J22.1	-0.739	-1.443	-2.679	0.009
ENSG00000243446	RN7SL284P	0.865	-1.881	2.561	0.012
ENSG00000243704	RN7SL105P	1.052	0.120	2.313	0.023
ENSG00000244061	RP11-389C8.1	-0.920	-2.160	-2.542	0.013

ENSG00000244073	RPS4XP6	-0.468	2.021	-2.281	0.025
ENSG00000244139	RN7SL397P	0.858	-3.782	2.015	0.047
ENSG00000244165	P2RY11	-0.356	0.770	-2.044	0.044
ENSG00000244194	RN7SL218P	0.832	-0.155	2.021	0.047
ENSG00000244289	RPS3AP35	0.697	-1.363	2.066	0.042
ENSG00000244300	GATA2-AS1	0.711	-1.412	2.420	0.018
ENSG00000244327	RP11-383G6.3	-0.454	-0.564	-2.125	0.037
ENSG00000244568	RN7SL654P	1.011	-3.733	2.781	0.007
ENSG00000244731	C4A	-0.819	1.760	-2.174	0.033
ENSG00000244757	RP11-45A16.4	-0.541	-1.565	-2.081	0.041
ENSG00000244952	RP11-1000B6.5	-0.845	-1.459	-2.729	0.008
ENSG00000245080	MIR3150B	-0.766	-1.066	-2.053	0.043
ENSG00000245248	USP2-AS1	-0.458	0.444	-2.544	0.013
ENSG00000245526	LINC00461	0.180	6.195	2.055	0.043
ENSG00000245832	MIR4300HG	-0.575	-0.253	-2.200	0.031
ENSG00000245928	RP11-630D6.5	-0.592	-1.791	-2.152	0.034
ENSG00000245970	KB-1208A12.3	0.393	0.504	2.119	0.037
ENSG00000246090	RP11-696N14.1	0.460	0.247	2.312	0.023
ENSG00000246145	RRS1-AS1	-0.575	0.300	-2.450	0.016
ENSG00000246203	RP11-29H23.5	-0.441	-0.041	-2.053	0.043
ENSG00000246250	RP11-613D13.5	-0.321	1.176	-2.032	0.045
ENSG00000246982	RP1-179N16.6	0.333	1.327	2.050	0.043
ENSG00000247516	MIR4458HG	0.302	3.277	2.168	0.033
ENSG00000247708	STX18-AS1	-0.205	3.709	-2.350	0.021
ENSG00000247828	TMEM161B-AS1	0.227	5.726	2.465	0.016
ENSG00000248079	DPH6-AS1	0.380	0.917	2.230	0.028
ENSG00000248180	GAPDHP60	-0.614	2.041	-1.996	0.049
ENSG00000248216	KCTD9P5	-0.514	-0.886	-2.062	0.042
ENSG00000248249	RP11-598O12.1	-0.478	0.989	-2.114	0.038
ENSG00000248305	RP11-310A13.2	-0.697	-0.371	-2.472	0.015
ENSG00000248397	LINC00498	-0.710	-1.872	-2.108	0.038
ENSG00000248429	RP11-597D13.9	-0.532	2.237	-2.538	0.013
ENSG00000248559	RP11-215P8.3	0.358	0.854	2.049	0.044
ENSG00000248696	AC011406.2	-0.733	-2.022	-2.573	0.012
ENSG00000248738	RP11-87E22.2	0.479	2.455	2.212	0.030
ENSG00000248803	RP11-287J9.1	-0.633	-2.080	-2.107	0.038
ENSG00000248810	LINC02432	-0.894	-2.240	-2.264	0.026
ENSG00000248859	LINC01574	-0.843	-1.505	-2.558	0.012
ENSG00000248967	RP11-241J12.3	-0.532	-0.966	-2.592	0.011
ENSG00000249096	LINC02362	-0.486	-0.550	-2.516	0.014
ENSG00000249098	RP11-39E3.4	-0.595	-0.475	-2.358	0.021
ENSG00000249278	RP11-688H10.1	-1.180	-2.944	-2.757	0.007
ENSG00000249381	LINC00500	-1.042	-1.984	-2.601	0.011
ENSG00000249580	MTATP6P22	-0.805	-0.973	-2.471	0.016
ENSG00000249755	RP11-466G12.2	-0.564	-1.200	-2.110	0.038
ENSG00000249859	PVT1	-0.340	1.662	-2.339	0.022
ENSG00000249896	LINC02495	0.205	4.052	2.177	0.032

ENSG00000250056	LINC01018	-0.302	3.003	-2.502	0.014
ENSG00000250091	DNAH10OS	-0.259	3.399	-2.026	0.046
ENSG00000250441	RP11-47F1.1	-0.610	-1.734	-2.022	0.046
ENSG00000250500	RP11-119H12.3	-0.611	-0.538	-2.614	0.011
ENSG00000250514	LINC02125	-0.613	-0.947	-2.064	0.042
ENSG00000250860	CTD-2265D6.2	-0.593	-0.872	-2.082	0.040
ENSG00000251139	RP11-701P16.2	-0.821	-1.458	-2.724	0.008
ENSG00000251171	RP11-327O17.2	-0.472	-0.135	-2.369	0.020
ENSG00000251372	LINC00499	-0.503	1.924	-2.041	0.044
ENSG00000251552	COQ10BP2	-0.927	-1.753	-3.343	0.001
ENSG00000251596	HADHAP1	-0.389	3.546	-2.665	0.009
ENSG00000251687	RP11-181K12.2	-0.658	-1.674	-2.319	0.023
ENSG00000251805	SCARNA21	-0.783	-2.141	-2.861	0.005
ENSG00000251880	RNU7-75P	-0.656	-2.207	-2.102	0.039
ENSG00000252017	RNU6-1194P	-0.718	-1.642	-2.025	0.046
ENSG00000252034	RNY4P37	0.755	-1.615	2.133	0.036
ENSG00000252290	SNORA25	-0.996	-2.761	-2.474	0.015
ENSG00000252316	RNY4	-0.936	-2.656	-2.271	0.026
ENSG00000252643	RNU6-1136P	-0.666	-1.743	-2.077	0.041
ENSG00000252717	RNU6-352P	-0.776	-2.145	-2.766	0.007
ENSG00000252757	RN7SKP96	-0.462	-0.570	-1.997	0.049
ENSG00000253167	KIAA0196-AS1	-0.511	-0.630	-2.500	0.014
ENSG00000253259	RP11-737F9.2	-0.447	-1.062	-1.992	0.050
ENSG00000253276	CCDC71L	0.263	3.206	2.093	0.039
ENSG00000253330	RP11-697N18.3	-0.463	0.922	-2.166	0.033
ENSG00000253341	RP11-730G20.2	-0.470	3.895	-2.213	0.030
ENSG00000253384	CTD-2547L16.3	-0.547	-1.137	-1.989	0.050
ENSG00000253537	PCDHGA7	-0.254	2.924	-2.222	0.029
ENSG00000253764	RP11-439C15.4	-0.426	1.774	-2.643	0.010
ENSG00000253784	RP11-1145L24.1	-0.704	-1.834	-2.087	0.040
ENSG00000253849	RP11-759A9.1	-0.837	-1.340	-2.664	0.009
ENSG00000253873	PCDHGA11	-0.224	2.919	-2.090	0.040
ENSG00000253910	PCDHGB2	-0.276	3.345	-2.333	0.022
ENSG00000253915	MAPRE1P1	-0.319	2.435	-2.017	0.047
ENSG00000253955	CTB-33O18.3	-0.723	0.288	-3.539	0.001
ENSG00000254061	RP11-359B20.1	-0.594	-1.070	-2.418	0.018
ENSG00000254114	HMGN1P28	-0.835	-2.482	-2.300	0.024
ENSG00000254187	CTB-78F1.1	-0.262	2.404	-2.062	0.042
ENSG00000254302	RP11-431M3.1	-1.117	-2.091	-2.762	0.007
ENSG00000254398	RP11-578F21.2	-0.561	-0.342	-2.509	0.014
ENSG00000254415	SIGLEC14	1.192	-1.829	2.100	0.039
ENSG00000254454	RCC2P6	-0.661	0.625	-2.972	0.004
ENSG00000254561	RP11-196E1.3	-0.405	0.827	-2.243	0.028
ENSG00000254595	CTD-2010I16.1	-0.613	1.710	-3.869	0.000
ENSG00000254602	AP000662.4	0.299	0.459	2.156	0.034
ENSG00000254644	RP11-5A11.2	-0.583	-1.217	-2.040	0.045
ENSG00000254688	RP11-47J17.3	-0.596	-1.792	-2.146	0.035

ENSG00000254703	SENCR	0.828	-2.287	2.296	0.024
ENSG00000254973	RP11-429J17.7	0.606	-0.633	2.501	0.014
ENSG00000255004	RP1-68D18.3	-0.530	1.869	-2.270	0.026
ENSG00000255087	RP11-168K9.2	0.394	2.474	2.133	0.036
ENSG00000255121	RP11-110I1.12	-0.390	0.073	-2.320	0.023
ENSG00000255234	RP11-727A23.10	-0.588	-1.707	-2.351	0.021
ENSG00000255267	RP11-430H10.2	-0.501	-1.124	-2.175	0.032
ENSG00000255329	H2AFZP4	-0.861	-1.837	-2.990	0.004
ENSG00000255389	C6orf3	0.278	2.760	2.040	0.045
ENSG00000255455	RP11-890B15.3	-0.196	4.845	-2.575	0.012
ENSG00000255545	RP11-627G23.1	-0.619	2.393	-2.924	0.004
ENSG00000255571	MIR9-3HG	0.195	4.883	2.360	0.021
ENSG00000255622	PCDHB17P	-0.320	1.641	-2.371	0.020
ENSG00000255717	SNHG1	0.257	2.941	2.272	0.026
ENSG00000255968	RP11-513G19.1	-0.389	0.456	-2.092	0.039
ENSG00000256001	RP11-575F12.2	0.593	-1.360	2.338	0.022
ENSG00000256050	RP11-982M15.6	-0.426	-0.045	-2.027	0.046
ENSG00000256120	RP11-778H2.1	-0.420	-0.601	-2.030	0.046
ENSG00000256222	MTRNR2L3	-0.755	-1.825	-2.093	0.039
ENSG00000256525	POLG2	0.228	2.535	2.290	0.025
ENSG00000256537	SMIM10L1	0.260	5.396	2.049	0.044
ENSG00000256588	RP11-613F22.8	-0.588	-0.701	-2.117	0.037
ENSG00000256637	LINC01965	0.666	-0.160	2.938	0.004
ENSG00000256684	RP11-49K4.2	-1.061	-3.389	-2.393	0.019
ENSG00000256732	RP11-407A16.3	0.946	-2.102	2.422	0.018
ENSG00000257002	AP000438.2	0.656	-0.475	2.558	0.012
ENSG00000257004	RP11-180M15.3	-0.659	-2.521	-2.318	0.023
ENSG00000257052	RP11-881M11.2	-1.142	-2.391	-3.134	0.002
ENSG00000257060	RP11-266O8.1	-0.430	1.534	-3.069	0.003
ENSG00000257267	ZNF271P	0.221	5.517	2.211	0.030
ENSG00000257302	FAHD2P1	-0.715	-0.004	-3.326	0.001
ENSG00000257354	RP11-631N16.2	-0.309	2.715	-2.122	0.037
ENSG00000257472	RP11-159D23.2	-0.904	-1.793	-2.682	0.009
ENSG00000257545	RP11-144F15.1	-0.438	-0.968	-2.004	0.048
ENSG00000257591	ZNF625	0.403	-1.095	2.065	0.042
ENSG00000257605	RP11-680A11.5	0.690	-2.042	2.476	0.015
ENSG00000257735	RP11-370I10.6	-0.379	-1.069	-2.043	0.044
ENSG00000257748	LINC02281	0.907	-2.506	2.647	0.010
ENSG00000257943	RP11-167N24.3	-0.842	-2.369	-2.312	0.023
ENSG00000257964	RP11-133N21.10	0.419	-0.117	2.124	0.037
ENSG00000258012	RP11-603K19.1	-0.387	1.774	-2.157	0.034
ENSG00000258024	OR5BT1P	-0.602	-0.378	-2.797	0.006
ENSG00000258048	RP11-530C5.1	-1.067	-2.026	-3.384	0.001
ENSG00000258227	CLEC5A	-0.883	-0.345	-2.364	0.020
ENSG00000258279	LINC00592	0.822	-1.319	2.396	0.019
ENSG00000258289	CHURC1	0.325	6.182	2.417	0.018
ENSG00000258437	RP11-661G16.1	-0.529	-1.582	-2.073	0.041

ENSG00000258439	RP11-173A8.2	-0.643	-1.472	-2.170	0.033
ENSG00000258485	SRMP2	-0.395	0.314	-2.026	0.046
ENSG00000258548	LINC00645	0.809	-0.351	2.501	0.014
ENSG00000258619	RP11-134E15.1	-0.752	-2.182	-2.197	0.031
ENSG00000258682	CTD-2002H8.2	-0.366	0.764	-2.205	0.030
ENSG00000258741	RP11-386M24.4	-0.570	0.900	-2.550	0.013
ENSG00000258754	LINC01579	-0.735	0.435	-3.563	0.001
ENSG00000258824	CTD-2555O16.2	-0.293	0.356	-2.073	0.041
ENSG00000258909	RP11-164C12.2	-0.673	-1.373	-2.204	0.030
ENSG00000258930	RP5-1163L11.2	-0.449	0.670	-2.105	0.038
ENSG00000259056	DUXAP1	-0.719	-1.626	-2.436	0.017
ENSG00000259218	LINC00928	0.483	0.358	2.727	0.008
ENSG00000259241	RP11-313P18.2	-0.639	-0.221	-2.725	0.008
ENSG00000259345	RP11-624L4.1	-0.482	1.204	-2.479	0.015
ENSG00000259366	CTD-2647L4.4	-0.297	2.315	-2.389	0.019
ENSG00000259381	RP11-192M23.1	-0.414	0.926	-2.581	0.012
ENSG00000259395	RP11-475A13.2	1.133	-2.005	3.594	0.001
ENSG00000259423	RP11-265N7.2	-1.217	-2.857	-3.180	0.002
ENSG00000259481	RP11-39M21.1	-0.554	1.703	-3.064	0.003
ENSG00000259485	LINC02253	-0.886	-2.825	-2.123	0.037
ENSG00000259505	RP11-74D7.1	-0.872	-1.048	-2.527	0.013
ENSG00000259575	RP11-39M21.2	-0.487	0.343	-2.807	0.006
ENSG00000259581	TYRO3P	-0.613	2.372	-2.152	0.034
ENSG00000259591	RP11-554D20.1	-0.401	0.638	-2.229	0.029
ENSG00000259635	AC100830.3	-0.531	-2.159	-2.186	0.032
ENSG00000259686	HNRNPA1P71	-0.780	-1.918	-2.144	0.035
ENSG00000260077	RP11-254F7.2	-0.385	1.068	-2.263	0.026
ENSG00000260271	RP1-45N11.1	-0.906	-1.650	-3.171	0.002
ENSG00000260300	RP11-505K9.4	-0.483	-1.106	-2.157	0.034
ENSG00000260306	RP11-645C24.5	-0.517	-0.178	-2.141	0.035
ENSG00000260339	HEXA-AS1	0.425	-0.183	2.170	0.033
ENSG00000260422	RP1-155D22.2	-1.088	-2.403	-2.796	0.006
ENSG00000260461	RP11-541N10.3	-0.366	0.853	-2.253	0.027
ENSG00000260469	C15orf59-AS1	1.059	-2.593	2.594	0.011
ENSG00000260551	PWRN2	-0.962	0.635	-2.403	0.019
ENSG00000260583	AP000223.42	-0.269	1.146	-2.047	0.044
ENSG00000260588	RP11-930P14.2	-0.989	-2.520	-2.798	0.006
ENSG00000260599	CTC-457E21.1	-1.152	0.289	-3.464	0.001
ENSG00000260633	RP11-375I20.6	-1.266	-1.546	-3.836	0.000
ENSG00000260647	RP1-178F10.1	0.524	-1.422	2.160	0.034
ENSG00000260721	AF067845.1	-0.460	0.946	-2.040	0.045
ENSG00000260785	CASC17	-0.789	-2.050	-2.636	0.010
ENSG00000260855	RP11-439E19.10	-0.236	3.180	-2.106	0.038
ENSG00000260947	RP11-384P7.7	0.246	3.705	2.131	0.036
ENSG00000260949	KB-1836B5.1	0.528	-0.975	2.059	0.043
ENSG00000261003	RP1-140C12.2	-0.618	0.281	-2.319	0.023
ENSG00000261094	RP11-355O1.11	0.466	1.629	3.289	0.001

ENSG00000261096	RP11-690I21.2	-0.307	1.193	-2.104	0.038
ENSG00000261129	RP11-361D14.2	-0.909	-2.023	-2.248	0.027
ENSG00000261136	RP11-37C7.3	-0.492	-0.089	-2.503	0.014
ENSG00000261159	RP11-723O4.9	-0.815	-1.449	-2.707	0.008
ENSG00000261175	LINC02188	-0.599	-1.540	-2.298	0.024
ENSG00000261188	CTA-445C9.14	0.266	3.485	2.098	0.039
ENSG00000261202	RP3-496C20.1	0.484	-1.487	1.996	0.049
ENSG00000261336	EIF4BP5	-0.500	0.592	-2.507	0.014
ENSG00000261402	RP11-378I6.1	-0.524	1.431	-2.609	0.011
ENSG00000261404	AC009120.4	-0.381	0.927	-2.266	0.026
ENSG00000261432	LINC01613	-0.731	-2.108	-2.291	0.025
ENSG00000261527	RP11-343C2.10	0.576	-1.899	2.296	0.024
ENSG00000261617	LINC02177	-0.587	1.372	-2.663	0.009
ENSG00000261675	RP5-1119A7.17	-0.249	4.536	-2.247	0.027
ENSG00000261799	RP11-283I3.6	-0.250	3.885	-2.627	0.010
ENSG00000261801	LOXL1-AS1	-0.546	-0.649	-2.082	0.040
ENSG00000261824	LINC00662	0.158	4.674	2.376	0.020
ENSG00000262096	PCDHB19P	-0.291	2.107	-2.028	0.046
ENSG00000262267	U95743.1	0.579	0.036	2.548	0.013
ENSG00000262316	RP11-433P17.1	-0.611	-1.381	-2.291	0.025
ENSG00000263680	RP11-57A1.1	-0.690	-1.785	-2.473	0.015
ENSG00000263740	RN7SL4P	-0.596	12.080	-2.236	0.028
ENSG00000263797	RP11-146G7.2	-1.193	-2.971	-2.907	0.005
ENSG00000263878	DLGAP1-AS4	0.367	3.975	2.759	0.007
ENSG00000264016	CTC-297N7.9	-0.800	-1.994	-2.439	0.017
ENSG00000264275	RN7SL753P	0.865	-1.074	2.388	0.019
ENSG00000264520	RP4-777O23.2	-0.488	-0.642	-2.347	0.021
ENSG00000264895	RP11-421E14.2	0.346	2.371	2.065	0.042
ENSG00000264916	RN7SL230P	0.879	-1.348	2.062	0.042
ENSG00000264932	CTD-2104P17.2	0.516	-1.150	2.158	0.034
ENSG00000265279	CLUHP6	-0.841	-2.012	-3.010	0.003
ENSG00000265337	RP11-466A19.5	-0.588	-1.594	-2.166	0.033
ENSG00000265494	RP11-131K5.2	-0.599	-1.218	-2.058	0.043
ENSG00000265657	MIR3151	-1.074	-1.473	-3.423	0.001
ENSG00000265702	RP11-156L14.1	0.715	-1.284	2.420	0.018
ENSG00000265735	RN7SL5P	-0.537	11.124	-2.285	0.025
ENSG00000265802	RN7SL49P	0.507	0.068	2.327	0.022
ENSG00000265817	FSBP	0.461	1.602	2.710	0.008
ENSG00000265845	RP11-20B24.5	0.470	-0.916	2.253	0.027
ENSG00000265918	RN7SL543P	0.889	-2.388	2.156	0.034
ENSG00000266100	RP11-118E18.2	-0.656	-2.291	-2.261	0.026
ENSG00000266573	RP11-449D8.5	0.727	-1.151	2.524	0.014
ENSG00000266918	RP11-798G7.8	-0.682	0.739	-1.998	0.049
ENSG00000266921	RP11-15A1.7	0.376	0.212	2.296	0.024
ENSG00000267128	RP11-449J21.5	-0.465	1.419	-3.429	0.001
ENSG00000267131	RP11-332H18.5	-0.594	-1.948	-2.552	0.013
ENSG00000267150	RP11-411B10.2	-0.840	-1.261	-3.253	0.002

ENSG00000267193	RP11-116O18.3	-0.955	-0.884	-2.952	0.004
ENSG00000267207	RP11-264B14.1	-0.676	-2.405	-2.230	0.028
ENSG00000267262	CTC-232P5.3	-0.761	-2.229	-2.149	0.035
ENSG00000267361	LOC100421166	-0.384	1.200	-2.081	0.041
ENSG00000267508	ZNF285	0.244	3.829	2.721	0.008
ENSG00000267585	RP11-115P21.1	-0.853	-1.053	-3.357	0.001
ENSG00000267629	AC138430.4	-0.634	-0.672	-2.297	0.024
ENSG00000267724	RP11-49K24.8	-0.304	1.136	-2.097	0.039
ENSG00000267764	RP11-484L8.1	-0.652	-0.785	-2.631	0.010
ENSG00000267795	SMIM22	-0.718	-2.015	-2.202	0.030
ENSG00000267858	MZF1-AS1	0.366	1.448	2.459	0.016
ENSG00000268015	CTD-2525I3.3	-0.668	-1.379	-2.999	0.004
ENSG00000268038	LINC01785	-0.965	-1.220	-2.443	0.017
ENSG00000268055	AC067969.2	-0.848	-0.952	-2.497	0.015
ENSG00000268182	SMIM17	0.380	2.832	2.398	0.019
ENSG00000268201	CTD-3138B18.6	0.323	0.480	2.167	0.033
ENSG00000268230	CTD-2619J13.8	0.528	0.548	2.847	0.006
ENSG00000268355	AC006129.1	-1.041	-2.895	-2.392	0.019
ENSG00000268560	CTD-2332E11.2	-0.722	-2.036	-2.521	0.014
ENSG00000268658	LINC00664	-0.529	0.734	-2.050	0.044
ENSG00000268756	AC104534.2	-0.539	-2.391	-2.011	0.048
ENSG00000269001	CTD-2245F17.3	0.373	3.012	2.444	0.017
ENSG00000269138	ZNF209P	-0.474	1.207	-2.004	0.048
ENSG00000269189	RP11-573N10.1	-0.436	-0.460	-2.174	0.033
ENSG00000269373	CTD-2542C24.3	0.803	-2.662	2.416	0.018
ENSG00000269463	RP11-727F15.13	0.548	-1.828	2.097	0.039
ENSG00000269696	AC007228.11	0.306	1.826	2.712	0.008
ENSG00000269961	CTD-2033C11.1	-0.621	-1.894	-2.313	0.023
ENSG00000270317	AC004004.2	-1.212	-1.725	-3.286	0.001
ENSG00000270429	KNOP1P2	-0.748	-1.463	-2.461	0.016
ENSG00000270706	PRMT1P1	-0.472	0.511	-2.125	0.037
ENSG00000270708	RP11-568G11.5	-0.932	-2.434	-2.789	0.007
ENSG00000270722	RNVU1-31	0.792	-0.800	2.569	0.012
ENSG00000270765	GAS2L2	-0.640	-1.567	-2.219	0.029
ENSG00000270815	RP11-56O18.1	-0.885	-2.325	-2.547	0.013
ENSG00000270832	RP1-168P16.3	-0.490	-1.131	-2.001	0.049
ENSG00000271065	RP11-607P23.1	-0.952	-2.570	-2.385	0.019
ENSG00000271148	RP11-401N18.1	0.906	-2.478	2.076	0.041
ENSG00000271159	HINT1P2	-0.664	-1.003	-2.716	0.008
ENSG00000271264	MZT1P1	-0.505	-0.989	-2.439	0.017
ENSG00000271275	AC007326.9	-0.493	0.382	-1.998	0.049
ENSG00000271382	RP11-1060G2.2	-0.543	-0.894	-2.178	0.032
ENSG00000271817	U3	-0.845	-1.672	-2.990	0.004
ENSG00000271851	RP11-565F19.2	0.426	0.579	3.142	0.002
ENSG00000271871	AC005740.6	-0.630	-1.093	-2.286	0.025
ENSG00000271889	RP11-493E12.1	0.564	-0.115	2.513	0.014
ENSG00000271976	RP11-884K10.7	-0.236	2.755	-2.171	0.033

ENSG00000272031	ANKRD34A	-0.339	3.837	-2.682	0.009
ENSG00000272195	RP11-156E8.1	-0.713	-1.702	-2.427	0.017
ENSG00000272223	RP1-20C7.6	-1.085	-2.485	-3.827	0.000
ENSG00000272383	RP11-556O9.4	-0.571	-0.073	-2.349	0.021
ENSG00000272398	CD24	0.490	3.795	2.157	0.034
ENSG00000272419	RP11-403I13.8	-0.392	1.180	-2.149	0.035
ENSG00000272477	RP11-158G18.1	-0.423	0.454	-2.028	0.046
ENSG00000272529	RP11-415F23.4	-0.399	0.597	-2.014	0.047
ENSG00000272536	RN7SL840P	0.875	-3.143	2.259	0.027
ENSG00000272620	AFAP1-AS1	-0.311	1.847	-2.253	0.027
ENSG00000272977	CTA-390C10.10	-0.424	1.976	-2.709	0.008
ENSG00000273033	LINC02035	-0.191	4.039	-2.421	0.018
ENSG00000273216	AC002059.10	0.913	-2.115	2.735	0.008
ENSG00000273218	LLNLR-246C6.1	0.847	0.561	4.941	0.000
ENSG00000273238	RP11-1263C18.1	0.215	3.849	2.004	0.048
ENSG00000273245	RP11-434P11.2	-0.388	0.280	-1.999	0.049
ENSG00000273265	RP11-353K11.1	-0.487	-1.621	-2.031	0.045
ENSG00000273274	ZBTB8B	0.295	3.407	2.545	0.013
ENSG00000273580	RP11-609L3.2	0.596	-1.070	2.103	0.039
ENSG00000273759	RP4-563E14.1	-0.434	-0.458	-2.117	0.037
ENSG00000273771	RP11-236L14.2	0.617	-1.252	2.110	0.038
ENSG00000273783	CTD-2506P8.6	0.775	-1.615	2.886	0.005
ENSG00000274012	RN7SL2	-0.556	12.784	-2.537	0.013
ENSG00000274020	LINC01138	-0.254	2.931	-2.707	0.008
ENSG00000274105	RP11-278C7.3	-0.445	0.554	-2.222	0.029
ENSG00000274210	RNVU1-27	0.899	-0.924	2.209	0.030
ENSG00000274341	RP11-227G15.10	0.723	-1.768	2.845	0.006
ENSG00000274677	RP11-505K9.5	-0.603	-0.813	-2.045	0.044
ENSG00000274979	RP11-1143G9.5	-0.957	-2.259	-2.325	0.023
ENSG00000275066	SYNRG	-0.127	6.112	-2.163	0.033
ENSG00000275090	RN7SL296P	1.089	-1.541	2.076	0.041
ENSG00000275485	RP11-261P13.6	-0.706	-2.191	-2.120	0.037
ENSG00000275645	RP11-817O13.9	0.843	-2.388	2.567	0.012
ENSG00000275678	RP4-547N15.3	0.754	-1.574	2.180	0.032
ENSG00000275878	RN7SL43P	1.197	-3.807	2.358	0.021
ENSG00000275963	RP11-180M15.6	-0.325	1.283	-2.554	0.012
ENSG00000276047	RN7SL711P	-0.520	1.069	-2.190	0.031
ENSG00000276214	CTD-2146O16.1	-0.448	0.540	-2.049	0.044
ENSG00000276259	RP11-481J2.4	-0.349	0.585	-2.653	0.010
ENSG00000276291	FRG1HP	0.239	4.123	2.080	0.041
ENSG00000276337	RP11-106D4.2	-0.339	1.041	-2.022	0.046
ENSG00000276754	RP11-461A8.5	-0.869	-2.137	-2.850	0.006
ENSG00000276757	RN7SL192P	-0.559	-1.976	-2.066	0.042
ENSG00000277020	RP11-476H16.1	-0.647	-1.705	-2.003	0.048
ENSG00000277171	RP11-191H23.1	-0.471	0.222	-2.275	0.025
ENSG00000277425	RP5-1116H23.3	0.602	-1.573	2.186	0.032
ENSG00000277443	MARCKS	0.244	8.208	2.464	0.016

ENSG00000277863	RP11-282A11.4	-0.979	-2.166	-2.005	0.048
ENSG00000277959	RP11-45A17.2	0.473	-0.929	2.028	0.046
ENSG00000278383	RP4-694B14.8	-0.581	-0.436	-3.255	0.002
ENSG00000278472	RP11-74D7.3	-0.834	-1.771	-2.213	0.030
ENSG00000278532	RP11-17E3.1	0.923	-1.808	2.572	0.012
ENSG00000278576	RP11-507K2.6	-0.305	2.612	-2.283	0.025
ENSG00000278768	BACE1-AS	-0.247	3.344	-3.133	0.002
ENSG00000278891	RP11-246E12.2	-0.487	-0.903	-2.217	0.029
ENSG00000279012	OR51B2	-0.909	-2.461	-2.031	0.045
ENSG00000279090	RP11-684N3.1	-0.441	1.354	-2.434	0.017
ENSG00000279110	CTA-243E7.1	-0.328	2.573	-2.110	0.038
ENSG00000279117	CTD-2562J17.6	-0.173	5.777	-2.245	0.027
ENSG00000279118	RP11-517I3.2	-0.438	3.940	-2.393	0.019
ENSG00000279130	RP11-278J6.4	-1.226	-2.385	-3.413	0.001
ENSG00000279251	RP11-78O7.1	0.447	0.819	2.651	0.010
ENSG00000279310	RP11-123O10.1	-0.772	-1.004	-2.679	0.009
ENSG00000279433	RP11-4B16.1	-0.624	-1.180	-2.401	0.019
ENSG00000279498	RP11-72I2.2	-0.616	-1.770	-2.103	0.039
ENSG00000279679	RP11-278J6.5	-0.625	-1.400	-2.161	0.034
ENSG00000279853	RP5-844F9.1	-0.332	0.754	-2.044	0.044
ENSG00000279863	RP11-521C22.2	-0.389	1.285	-1.992	0.050
ENSG00000279887	RP11-264L1.1	-0.590	0.125	-2.964	0.004
ENSG00000279936	CTD-2086O20.1	-0.661	-1.012	-2.736	0.008
ENSG00000280098	AC011155.1	-0.969	-2.260	-2.776	0.007
ENSG00000280118	RP11-385H1.1	-0.360	1.292	-2.015	0.047
ENSG00000280149	RP4-751H13.5	0.436	0.021	2.448	0.016
ENSG00000280165	PCDH20	1.136	-1.744	2.907	0.005
ENSG00000280166	RP11-354E23.3	-0.717	-1.470	-2.162	0.033
ENSG00000280169	RP11-151A6.6	-0.759	-2.826	-2.048	0.044
ENSG00000280202	RP5-1180D12.1	-0.498	-1.124	-2.052	0.043
ENSG00000280205	RP11-504I13.2	-0.570	-0.522	-2.014	0.047
ENSG00000280248	CTD-2047H16.3	0.446	-0.077	2.110	0.038
ENSG00000280281	RP11-340L3.1	-0.811	-2.181	-2.105	0.038
ENSG00000280294	RP11-177H2.1	-0.312	2.871	-2.223	0.029
ENSG00000280485	RP11-437J2.4	-0.306	0.507	-2.022	0.046
ENSG00000280660	RP11-437J2.3	-0.289	4.795	-2.192	0.031
ENSG00000281248	LINC02536	0.972	-2.600	2.330	0.022
ENSG00000281406	BLACAT1	-0.602	-0.459	-2.694	0.009
ENSG00000281420	AP001052.1	0.588	-2.223	2.092	0.039
ENSG00000282057	RP4-621F18.2	-0.334	1.401	-2.202	0.030
ENSG00000282164	PEG13	-0.331	4.329	-2.749	0.007
ENSG00000282375	RP11-43A14.2	0.362	1.052	2.335	0.022
ENSG00000282834	RP11-712B9.6	-0.603	0.923	-2.667	0.009
ENSG00000282876	RP1-138B7.7	0.929	-2.327	2.429	0.017
ENSG00000282987	RP11-901H12.1	-1.024	-2.890	-2.508	0.014
ENSG00000283003	RP11-418I22.3	-0.710	-2.179	-2.036	0.045
ENSG00000283213	CTC-591M7.1	-0.441	0.263	-2.155	0.034

ENSG00000283312	RP11-562I5.1	0.550	0.394	2.291	0.025
ENSG00000283491	RP11-562I5.2	0.636	-0.252	2.471	0.016
ENSG00000283538	RP11-180P8.1	0.358	2.127	3.398	0.001
ENSG00000283913	#N/A	-0.416	0.600	-3.054	0.003
ENSG00000284024	#N/A	0.154	4.605	2.265	0.026
ENSG00000284052	#N/A	-0.313	2.965	-2.493	0.015
ENSG00000284633	#N/A	-0.450	-0.429	-2.368	0.020
ENSG00000284738	#N/A	0.385	-1.308	2.035	0.045
ENSG00000285215	#N/A	-0.400	0.563	-2.084	0.040
ENSG00000285230	RALY-AS1	-0.304	1.383	-2.094	0.039
ENSG00000285621	#N/A	-0.528	0.236	-3.242	0.002
ENSG00000285631	#N/A	-0.912	-1.871	-2.027	0.046
ENSG00000285691	#N/A	-0.933	-1.817	-2.461	0.016
ENSG00000285695	#N/A	-0.846	-2.658	-2.153	0.034
ENSG00000285735	#N/A	0.701	0.210	2.238	0.028
ENSG00000285774	#N/A	-0.557	0.078	-2.499	0.014
ENSG00000285791	#N/A	-0.292	1.044	-2.002	0.049
ENSG00000285813	#N/A	-0.384	2.585	-3.052	0.003
ENSG00000285906	#N/A	-0.505	-0.585	-2.235	0.028
ENSG00000285939	#N/A	0.389	1.080	2.017	0.047
ENSG00000285966	#N/A	0.366	0.868	2.031	0.045

OFC

gene_id	gene_name	logFC	AveExpr	t	P.Value
ENSG00000001617	SEMA3F	0.509	0.146	2.236	0.028
ENSG00000002330	BAD	0.291	2.193	2.093	0.039
ENSG00000002834	LASP1	0.172	6.100	2.417	0.018
ENSG00000003056	M6PR	0.155	5.385	1.991	0.050
ENSG00000004399	PLXND1	0.221	5.345	2.189	0.031
ENSG00000004660	CAMKK1	0.238	6.257	2.824	0.006
ENSG00000005379	TSPOAP1	0.280	7.461	2.722	0.008
ENSG00000005448	WDR54	0.211	2.952	2.087	0.040
ENSG00000006047	YBX2	0.450	0.097	2.052	0.043
ENSG00000006210	CX3CL1	0.283	5.564	2.499	0.014
ENSG00000006283	CACNA1G	0.378	4.837	2.888	0.005
ENSG00000006534	ALDH3B1	-0.423	0.601	-2.989	0.004
ENSG00000006740	ARHGAP44	0.141	5.898	2.009	0.048
ENSG00000007047	MARK4	0.214	4.693	2.148	0.035
ENSG00000007080	CCDC124	0.243	3.348	2.217	0.029
ENSG00000007171	NOS2	0.276	2.229	2.103	0.039
ENSG00000007202	KIAA0100	0.142	7.233	2.563	0.012
ENSG00000007264	MATK	0.321	4.085	2.825	0.006
ENSG00000008256	CYTH3	0.169	5.345	2.233	0.028
ENSG00000008324	SS18L2	0.249	2.296	2.192	0.031
ENSG00000008382	MPND	0.267	2.023	2.226	0.029
ENSG00000008853	RHOBTB2	0.227	5.967	2.129	0.036
ENSG00000010030	ETV7	-0.758	-1.523	-2.429	0.017
ENSG00000010244	ZNF207	-0.128	7.280	-2.004	0.048
ENSG00000011009	LYPLA2	0.259	2.080	2.302	0.024
ENSG00000011332	DPF1	0.247	4.039	2.593	0.011
ENSG00000011478	QPCTL	0.262	1.625	2.162	0.034
ENSG00000011485	PPP5C	0.130	5.451	2.037	0.045
ENSG00000015479	MATR3	-0.231	5.385	-2.162	0.034
ENSG00000015592	STMN4	0.262	6.437	3.199	0.002
ENSG00000016864	GLT8D1	0.149	4.573	2.012	0.048
ENSG00000019485	PRDM11	0.160	4.858	2.341	0.022
ENSG00000025770	NCAPH2	0.234	3.640	2.537	0.013
ENSG00000031698	SARS	0.187	7.292	2.062	0.042
ENSG00000032389	TSSC1	0.255	4.418	3.131	0.002
ENSG00000036530	CYP46A1	0.252	5.440	2.283	0.025
ENSG00000038002	AGA	0.328	3.219	2.262	0.026
ENSG00000038382	TRIO	0.173	8.111	2.840	0.006
ENSG00000039139	DNAH5	0.184	3.897	2.156	0.034
ENSG00000041515	MYO16	0.309	4.739	2.719	0.008
ENSG00000043514	TRIT1	0.135	4.790	2.017	0.047
ENSG00000044090	CUL7	0.187	4.266	2.961	0.004
ENSG00000048140	TSPAN17	0.406	3.915	4.156	0.000
ENSG00000048991	R3HDM1	0.173	8.734	2.192	0.031
ENSG00000050767	COL23A1	0.298	1.762	2.034	0.045

ENSG00000053372	MRTO4	0.235	4.267	2.425	0.018
ENSG00000055118	KCNH2	0.268	3.305	2.696	0.009
ENSG00000055813	CCDC85A	0.258	4.767	2.534	0.013
ENSG00000058404	CAMK2B	0.252	7.031	3.025	0.003
ENSG00000059573	ALDH18A1	0.147	4.427	2.080	0.041
ENSG00000059691	GATB	0.198	4.894	2.717	0.008
ENSG00000059915	PSD	0.227	6.584	2.016	0.047
ENSG00000060140	STYK1	0.369	3.740	2.359	0.021
ENSG00000060709	RIMBP2	0.217	6.703	2.431	0.017
ENSG00000062038	CDH3	0.258	1.945	2.097	0.039
ENSG00000062282	DGAT2	0.338	1.789	2.269	0.026
ENSG00000062524	LTK	0.380	0.950	2.163	0.033
ENSG00000063015	SEZ6	0.219	4.242	2.158	0.034
ENSG00000063244	U2AF2	0.115	5.490	2.083	0.040
ENSG00000063322	MED29	0.146	5.690	2.240	0.028
ENSG00000063438	AHRR	0.545	1.904	3.365	0.001
ENSG00000063854	HAGH	0.208	5.216	2.212	0.030
ENSG00000064545	TMEM161A	0.245	1.466	2.161	0.034
ENSG00000064601	CTSA	0.137	4.803	2.110	0.038
ENSG00000065320	NTN1	0.249	1.933	2.133	0.036
ENSG00000065518	NDUFB4	0.200	3.828	2.113	0.038
ENSG00000065618	COL17A1	-0.375	-0.036	-2.349	0.021
ENSG00000066248	NGEF	0.227	6.843	2.596	0.011
ENSG00000067221	STOML1	0.271	3.817	2.842	0.006
ENSG00000067369	TP53BP1	0.119	7.790	2.017	0.047
ENSG00000067601	PMS2P4	0.222	2.255	2.274	0.026
ENSG00000067606	PRKCZ	0.281	6.046	3.490	0.001
ENSG00000067836	ROGDI	0.205	4.550	2.225	0.029
ENSG00000068831	RASGRP2	0.245	2.930	2.345	0.021
ENSG00000068903	SIRT2	0.172	5.584	2.054	0.043
ENSG00000068971	PPP2R5B	0.167	5.366	2.240	0.028
ENSG00000069424	KCNAB2	0.158	7.209	2.025	0.046
ENSG00000070182	SPTB	0.281	6.017	3.020	0.003
ENSG00000070729	CNGB1	0.387	3.823	2.238	0.028
ENSG00000070808	CAMK2A	0.292	9.908	3.629	0.000
ENSG00000071205	ARHGAP10	0.476	5.235	2.663	0.009
ENSG00000071539	TRIP13	0.544	0.524	2.985	0.004
ENSG00000072110	ACTN1	0.170	6.680	2.277	0.025
ENSG00000072163	LIMS2	0.374	2.652	2.296	0.024
ENSG00000072310	SREBF1	-0.238	4.557	-2.018	0.047
ENSG00000072832	CRMP1	0.200	6.174	2.680	0.009
ENSG00000072954	TMEM38A	0.250	4.528	2.238	0.028
ENSG00000072958	AP1M1	0.251	5.973	2.728	0.008
ENSG00000073536	NLE1	0.188	3.135	2.133	0.036
ENSG00000073670	ADAM11	0.282	5.260	3.684	0.000
ENSG00000074416	MGLL	0.223	6.764	2.275	0.026
ENSG00000075035	WSCD2	0.170	5.202	2.079	0.041

ENSG00000075043	KCNQ2	0.293	7.321	2.731	0.008
ENSG00000075275	CELSR1	0.584	0.200	2.761	0.007
ENSG00000075426	FOSL2	0.250	5.966	2.065	0.042
ENSG00000076864	RAP1GAP	0.247	6.722	2.602	0.011
ENSG00000077235	GTF3C1	0.122	6.955	2.132	0.036
ENSG00000078304	PPP2R5C	0.164	7.403	2.318	0.023
ENSG00000079459	FDFT1	0.206	6.361	2.280	0.025
ENSG00000079689	SCGN	0.784	-1.178	2.369	0.020
ENSG00000079841	RIMS1	0.214	8.624	2.244	0.028
ENSG00000081248	CACNA1S	0.633	0.193	3.763	0.000
ENSG00000081760	AACS	0.217	5.950	2.760	0.007
ENSG00000081818	PCDHB4	-0.295	3.788	-2.362	0.021
ENSG00000082014	SMARCD3	0.175	5.220	2.176	0.032
ENSG00000083290	ULK2	0.166	6.741	2.387	0.019
ENSG00000083845	RPS5	0.260	5.311	2.266	0.026
ENSG00000085998	POMGNT1	0.166	4.856	2.247	0.027
ENSG00000086504	MRPL28	0.348	4.145	2.648	0.010
ENSG00000086827	ZW10	0.134	4.563	2.260	0.026
ENSG00000087095	NLK	0.186	8.035	2.028	0.046
ENSG00000087111	PIGS	0.151	4.211	2.455	0.016
ENSG00000087152	ATXN7L3	0.267	6.587	3.052	0.003
ENSG00000088035	ALG6	-0.206	3.346	-2.150	0.034
ENSG00000088325	TPX2	0.247	2.180	2.207	0.030
ENSG00000088356	PDRG1	0.270	2.027	2.652	0.010
ENSG00000088387	DOCK9	0.152	8.124	2.383	0.019
ENSG00000088538	DOCK3	0.124	8.224	2.016	0.047
ENSG00000089723	OTUB2	0.302	1.799	2.373	0.020
ENSG00000089847	ANKRD24	0.221	5.161	2.100	0.039
ENSG00000090104	RGS1	-1.142	2.820	-2.209	0.030
ENSG00000090266	NDUFB2	0.315	4.503	2.065	0.042
ENSG00000090372	STRN4	0.265	5.231	3.323	0.001
ENSG00000090565	RAB11FIP3	0.173	5.655	2.042	0.044
ENSG00000090615	GOLGA3	0.148	7.047	2.742	0.007
ENSG00000090971	NAT14	0.356	3.409	3.485	0.001
ENSG00000090975	PITPNM2	0.249	5.517	2.063	0.042
ENSG00000091622	PITPNM3	0.229	6.657	2.561	0.012
ENSG00000091656	ZFHX4	-0.311	6.014	-2.511	0.014
ENSG00000091732	ZC3HC1	0.147	2.645	2.000	0.049
ENSG00000092051	JPH4	0.181	7.321	2.060	0.043
ENSG00000092445	TYRO3	0.168	6.797	2.245	0.027
ENSG00000092841	MYL6	0.261	6.149	2.515	0.014
ENSG00000094804	CDC6	0.367	2.770	3.107	0.003
ENSG00000095203	EPB41L4B	0.183	6.646	2.291	0.025
ENSG00000095777	MYO3A	0.735	-0.288	3.192	0.002
ENSG00000095794	CREM	0.201	4.395	2.215	0.030
ENSG00000096080	MRPS18A	0.221	2.811	2.001	0.049
ENSG00000096384	HSP90AB1	0.214	9.665	2.305	0.024

ENSG00000096696	DSP	0.601	3.320	2.062	0.042
ENSG00000099260	PALMD	0.336	3.701	2.092	0.040
ENSG00000099341	PSMD8	0.257	5.236	2.411	0.018
ENSG00000099800	TIMM13	0.287	3.078	2.515	0.014
ENSG00000099804	CDC34	0.223	4.273	2.565	0.012
ENSG00000099817	POLR2E	0.242	4.634	2.639	0.010
ENSG00000099866	MADCAM1	0.449	0.040	2.513	0.014
ENSG00000099910	KLHL22	0.147	4.251	2.221	0.029
ENSG00000099953	MMP11	-0.785	-2.037	-2.229	0.029
ENSG00000099994	SUSD2	-0.655	-1.324	-2.255	0.027
ENSG00000099999	RNF215	0.157	3.313	2.060	0.043
ENSG00000100003	SEC14L2	0.226	4.446	2.091	0.040
ENSG00000100084	HIRA	0.156	2.905	2.109	0.038
ENSG00000100122	CRYBB1	0.460	-1.025	2.026	0.046
ENSG00000100218	RSPH14	0.340	0.958	2.824	0.006
ENSG00000100221	JOSD1	0.175	5.907	2.651	0.010
ENSG00000100228	RAB36	0.255	3.446	2.800	0.006
ENSG00000100239	PPP6R2	0.172	5.975	2.420	0.018
ENSG00000100263	RHBDD3	0.219	2.065	2.306	0.024
ENSG00000100276	RASL10A	0.361	2.477	2.319	0.023
ENSG00000100294	MCAT	0.263	2.589	2.866	0.005
ENSG00000100296	THOC5	0.117	5.434	2.455	0.016
ENSG00000100321	SYNGR1	0.192	7.823	2.258	0.027
ENSG00000100324	TAB1	0.163	3.865	2.003	0.049
ENSG00000100341	PNPLA5	0.301	0.361	2.203	0.030
ENSG00000100379	KCTD17	0.224	4.714	2.205	0.030
ENSG00000100418	DESI1	0.180	5.989	2.746	0.007
ENSG00000100478	AP4S1	0.174	5.323	2.692	0.009
ENSG00000100600	LGMN	0.247	4.720	3.422	0.001
ENSG00000100842	EFS	-0.357	2.418	-2.215	0.030
ENSG00000100865	CINP	0.195	4.495	2.724	0.008
ENSG00000100867	DHRS2	0.704	-1.789	2.293	0.024
ENSG00000100884	CPNE6	0.266	4.828	2.527	0.013
ENSG00000100897	DCAF11	0.270	6.145	3.393	0.001
ENSG00000100911	PSME2	0.209	3.186	2.112	0.038
ENSG00000101098	RIMS4	0.330	5.726	3.465	0.001
ENSG00000101134	DOK5	0.260	3.577	2.334	0.022
ENSG00000101198	NKAIN4	0.436	1.819	2.440	0.017
ENSG00000101210	EEF1A2	0.227	7.486	2.225	0.029
ENSG00000101298	SNPH	0.187	6.518	2.353	0.021
ENSG00000101350	KIF3B	0.204	7.436	2.577	0.012
ENSG00000101417	PXMP4	0.186	3.754	2.283	0.025
ENSG00000101445	PPP1R16B	0.212	7.815	2.987	0.004
ENSG00000101489	CELF4	0.208	6.901	2.356	0.021
ENSG00000101746	NOL4	0.192	6.210	2.184	0.032
ENSG00000102606	ARHGEF7	0.118	7.560	2.257	0.027
ENSG00000102898	NUTF2	0.177	5.350	2.501	0.014

ENSG00000102901	CENPT	0.202	4.193	2.267	0.026
ENSG00000102904	TSNAXIP1	0.246	2.339	2.146	0.035
ENSG00000102924	CBLN1	0.345	2.291	2.168	0.033
ENSG00000103034	NDRG4	0.167	9.145	2.062	0.042
ENSG00000103035	PSMD7	0.200	5.794	2.472	0.016
ENSG00000103066	PLA2G15	0.249	2.083	2.532	0.013
ENSG00000103145	HCFC1R1	0.218	3.752	2.265	0.026
ENSG00000103154	NECAB2	0.372	3.594	2.465	0.016
ENSG00000103264	FBXO31	0.132	5.304	2.123	0.037
ENSG00000103269	RHBDL1	0.222	1.963	2.188	0.032
ENSG00000103316	CRYM	0.347	5.903	2.142	0.035
ENSG00000103363	TCEB2	0.271	4.090	2.258	0.027
ENSG00000103423	DNAJA3	0.181	4.833	2.630	0.010
ENSG00000103460	TOX3	0.277	2.893	2.041	0.044
ENSG00000103489	XYLT1	0.222	6.290	2.653	0.010
ENSG00000103494	RPGRIP1L	0.152	5.734	2.124	0.037
ENSG00000103495	MAZ	0.293	3.429	2.735	0.008
ENSG00000103507	BCKDK	0.149	3.785	2.170	0.033
ENSG00000103510	KAT8	0.134	4.856	2.040	0.045
ENSG00000103528	SYT17	0.243	5.122	2.775	0.007
ENSG00000103832	GOLGA8UP	0.583	-1.370	2.009	0.048
ENSG00000103942	HOMER2	0.138	6.360	2.334	0.022
ENSG00000104059	FAM189A1	0.222	4.457	2.440	0.017
ENSG00000104154	SLC30A4	0.187	5.438	2.021	0.047
ENSG00000104327	CALB1	0.502	5.573	2.502	0.014
ENSG00000104549	SQLE	0.241	5.384	2.099	0.039
ENSG00000104679	R3HCC1	0.201	4.093	2.170	0.033
ENSG00000104783	KCNN4	-0.511	-1.225	-2.037	0.045
ENSG00000104863	LIN7B	0.257	2.366	2.141	0.035
ENSG00000104884	ERCC2	0.228	3.267	2.498	0.014
ENSG00000104886	PLEKHJ1	0.261	2.425	2.170	0.033
ENSG00000105058	FAM32A	0.244	5.318	2.319	0.023
ENSG00000105143	SLC1A6	0.291	2.452	2.310	0.023
ENSG00000105197	TIMM50	0.167	4.625	2.070	0.042
ENSG00000105245	NUMBL	0.230	4.708	2.050	0.044
ENSG00000105254	TBCB	0.231	6.196	2.282	0.025
ENSG00000105327	BBC3	0.355	0.808	2.539	0.013
ENSG00000105341	ATP5SL	0.260	3.585	3.209	0.002
ENSG00000105376	ICAM5	0.314	4.391	2.582	0.012
ENSG00000105419	MEIS3	0.490	5.067	3.610	0.001
ENSG00000105671	DDX49	0.254	3.350	2.206	0.030
ENSG00000105717	PBX4	0.318	0.339	2.150	0.035
ENSG00000106089	STX1A	0.334	6.099	3.053	0.003
ENSG00000106144	CASP2	-0.175	3.737	-2.143	0.035
ENSG00000106244	PDAP1	0.196	5.236	2.244	0.028
ENSG00000106348	IMPDH1	0.293	3.296	2.920	0.005
ENSG00000106462	EZH2	-0.216	1.976	-2.320	0.023

ENSG00000106554	CHCHD3	0.189	4.906	2.548	0.013
ENSG00000106571	GLI3	-0.414	4.049	-2.750	0.007
ENSG00000106608	URGCP	0.109	5.508	2.027	0.046
ENSG00000106636	YKT6	0.242	5.164	3.433	0.001
ENSG00000106682	EIF4H	0.198	6.866	2.187	0.032
ENSG00000106688	SLC1A1	0.235	5.730	2.181	0.032
ENSG00000106714	CNTNAP3	-0.210	4.136	-2.071	0.041
ENSG00000107130	NCS1	0.189	8.839	2.220	0.029
ENSG00000107223	EDF1	0.210	5.775	2.029	0.046
ENSG00000107242	PIP5K1B	0.271	4.840	2.513	0.014
ENSG00000107404	DVL1	0.263	4.854	2.149	0.035
ENSG00000107819	SFXN3	0.126	5.583	2.376	0.020
ENSG00000107874	CUEDC2	0.225	4.487	2.082	0.040
ENSG00000107959	PITRM1	0.118	6.150	2.257	0.027
ENSG00000108100	CCNY	0.211	6.740	2.407	0.018
ENSG00000108309	RUNDC3A	0.233	7.194	3.030	0.003
ENSG00000108352	RAPGEFL1	0.199	5.459	2.096	0.039
ENSG00000108590	MED31	0.280	2.318	2.093	0.039
ENSG00000108641	B9D1	0.166	2.919	2.057	0.043
ENSG00000108684	ASIC2	0.238	3.814	2.423	0.018
ENSG00000108771	DHX58	0.300	2.676	2.328	0.022
ENSG00000108819	PPP1R9B	0.216	6.808	2.126	0.037
ENSG00000108829	LRRC59	0.142	4.969	2.036	0.045
ENSG00000108840	HDAC5	0.168	5.981	2.149	0.035
ENSG00000108852	MPP2	0.163	4.978	2.450	0.016
ENSG00000108861	DUSP3	0.214	7.152	2.421	0.018
ENSG00000108960	MMD	0.393	6.327	2.469	0.016
ENSG00000109079	TNFAIP1	0.172	5.737	2.116	0.037
ENSG00000109103	UNC119	0.159	4.074	2.277	0.025
ENSG00000109390	NDUFC1	0.197	3.844	2.015	0.047
ENSG00000109685	WHSC1	0.151	7.355	3.087	0.003
ENSG00000109881	CCDC34	0.191	3.521	2.060	0.043
ENSG00000109943	CRTAM	0.564	-0.830	2.370	0.020
ENSG00000110013	SIAE	0.199	5.152	2.290	0.025
ENSG00000110076	NRXN2	0.228	6.549	2.245	0.027
ENSG00000110077	MS4A6A	-0.983	1.006	-2.545	0.013
ENSG00000110171	TRIM3	0.130	4.434	2.108	0.038
ENSG00000110328	GALNT18	0.229	4.786	2.867	0.005
ENSG00000110442	COMMD9	0.205	4.619	2.625	0.010
ENSG00000110497	AMBRA1	0.099	5.318	2.151	0.034
ENSG00000110619	CARS	0.215	4.993	3.087	0.003
ENSG00000110931	CAMKK2	0.203	7.412	2.322	0.023
ENSG00000110987	BCL7A	0.155	4.777	2.186	0.032
ENSG00000111087	GLI1	-0.552	-0.256	-2.493	0.015
ENSG00000111218	PRMT8	0.278	4.379	3.317	0.001
ENSG00000111229	ARPC3	0.308	5.425	2.203	0.030
ENSG00000111249	CUX2	0.378	5.555	2.844	0.006

ENSG00000111445	RFC5	0.159	3.797	2.350	0.021
ENSG00000111481	COPZ1	0.273	5.039	2.804	0.006
ENSG00000111640	GAPDH	0.232	8.538	2.312	0.023
ENSG00000111670	GNPTAB	0.144	7.496	2.160	0.034
ENSG00000111696	NT5DC3	0.169	6.897	2.004	0.048
ENSG00000111737	RAB35	0.201	4.307	2.302	0.024
ENSG00000112031	MTRF1L	0.182	3.659	2.088	0.040
ENSG00000112137	PHACTR1	0.171	7.969	2.138	0.035
ENSG00000112530	PACRG	0.239	2.637	2.430	0.017
ENSG00000112658	SRF	0.156	4.298	2.008	0.048
ENSG00000112715	VEGFA	0.502	5.194	2.679	0.009
ENSG00000112763	BTN2A1	0.154	4.726	2.067	0.042
ENSG00000112972	HMGCS1	0.272	7.437	2.054	0.043
ENSG00000113141	IK	0.270	5.752	2.120	0.037
ENSG00000113209	PCDHB5	-0.250	3.691	-2.665	0.009
ENSG00000113269	RNF130	0.111	6.992	1.992	0.050
ENSG00000113319	RASGRF2	0.213	8.225	2.121	0.037
ENSG00000113719	ERGIC1	0.134	5.859	2.245	0.027
ENSG00000113749	HRH2	0.243	4.708	2.358	0.021
ENSG00000113758	DBN1	0.158	5.643	2.138	0.036
ENSG00000113761	ZNF346	0.143	4.459	2.207	0.030
ENSG00000114423	CBLB	-0.124	4.524	-2.173	0.033
ENSG00000114544	SLC41A3	0.166	5.006	2.020	0.047
ENSG00000114767	RRP9	0.262	1.337	2.036	0.045
ENSG00000114812	VIPR1	0.195	4.312	2.167	0.033
ENSG00000114923	SLC4A3	0.252	5.298	2.582	0.012
ENSG00000114999	TTL	0.254	6.927	2.913	0.005
ENSG00000115041	KCNIP3	0.279	5.099	2.413	0.018
ENSG00000115155	OTOF	0.463	1.614	3.204	0.002
ENSG00000115226	FNDC4	0.314	4.580	2.845	0.006
ENSG00000115268	RPS15	0.258	4.798	2.246	0.027
ENSG00000115275	MOGS	0.213	3.122	2.917	0.005
ENSG00000115306	SPTBN1	0.165	11.298	2.085	0.040
ENSG00000115457	IGFBP2	0.404	2.219	2.169	0.033
ENSG00000115641	FHL2	0.164	3.726	2.012	0.048
ENSG00000115677	HDLBP	0.150	8.032	2.110	0.038
ENSG00000115685	PPP1R7	0.223	5.744	2.262	0.026
ENSG00000115694	STK25	0.191	5.818	2.814	0.006
ENSG00000116147	TNR	0.229	8.759	2.369	0.020
ENSG00000116194	ANGPTL1	-0.256	3.001	-2.078	0.041
ENSG00000116221	MRPL37	0.183	4.126	2.173	0.033
ENSG00000116254	CHD5	0.242	8.106	2.356	0.021
ENSG00000116337	AMPD2	0.193	5.495	2.707	0.008
ENSG00000116544	DLGAP3	0.311	5.470	2.168	0.033
ENSG00000116584	ARHGEF2	0.182	6.441	2.860	0.005
ENSG00000116586	LAMTOR2	0.219	2.793	2.201	0.031
ENSG00000116649	SRM	0.199	2.733	2.087	0.040

ENSG00000116857	TMEM9	0.235	5.058	2.705	0.008
ENSG00000116863	ADPRHL2	0.290	2.852	2.889	0.005
ENSG00000116991	SIPA1L2	0.165	7.100	2.300	0.024
ENSG00000117308	GALE	0.237	2.259	2.275	0.026
ENSG00000117395	EBNA1BP2	0.204	5.399	2.201	0.031
ENSG00000117410	ATP6V0B	0.341	3.994	2.617	0.011
ENSG00000117411	B4GALT2	0.464	4.428	3.397	0.001
ENSG00000117643	MAN1C1	0.189	5.129	2.467	0.016
ENSG00000117650	NEK2	0.782	0.470	2.747	0.007
ENSG00000117682	DHDDS	0.105	5.042	2.183	0.032
ENSG00000117691	NENF	0.206	4.004	2.022	0.046
ENSG00000118160	SLC8A2	0.258	5.950	2.126	0.037
ENSG00000118976	OTUD4P1	-0.377	2.631	-2.072	0.041
ENSG00000118985	ELL2	0.191	6.975	2.115	0.038
ENSG00000119013	NDUFB3	0.334	3.649	1.992	0.050
ENSG00000119383	PTPA	0.232	6.011	2.381	0.020
ENSG00000119421	NDUFA8	0.209	4.626	2.131	0.036
ENSG00000119487	MAPKAP1	0.162	6.181	2.314	0.023
ENSG00000119559	C19orf25	0.293	2.418	2.382	0.020
ENSG00000119599	DCAF4	0.209	3.613	2.607	0.011
ENSG00000119682	AREL1	0.136	5.939	2.083	0.040
ENSG00000119698	PPP4R4	0.298	6.207	2.235	0.028
ENSG00000119725	ZNF410	-0.270	2.231	-2.224	0.029
ENSG00000119782	FKBP1B	0.383	2.115	3.016	0.003
ENSG00000120337	TNFSF18	-0.517	0.148	-2.102	0.039
ENSG00000120458	MSANTD2	-0.141	3.898	-2.057	0.043
ENSG00000120616	EPC1	-0.126	5.733	-2.379	0.020
ENSG00000120658	ENOX1	0.169	4.813	2.172	0.033
ENSG00000120709	FAM53C	0.175	5.497	3.150	0.002
ENSG00000120725	SIL1	0.188	3.802	2.872	0.005
ENSG00000120756	PLS1	0.260	3.194	2.526	0.013
ENSG00000120820	GLT8D2	0.249	2.642	2.365	0.020
ENSG00000120903	CHRNA2	0.400	2.045	2.305	0.024
ENSG00000121297	TSHZ3	0.181	5.219	2.413	0.018
ENSG00000121413	ZSCAN18	0.169	5.205	2.478	0.015
ENSG00000121653	MAPK8IP1	0.300	6.115	2.944	0.004
ENSG00000121753	ADGRB2	0.227	6.507	2.042	0.044
ENSG00000121766	ZCCHC17	0.202	5.786	2.064	0.042
ENSG00000121769	FABP3	0.320	5.949	2.470	0.016
ENSG00000121988	ZRANB3	0.156	5.040	2.304	0.024
ENSG00000122034	GTF3A	0.239	5.246	2.207	0.030
ENSG00000122359	ANXA11	0.161	6.347	2.365	0.020
ENSG00000122432	SPATA1	-0.385	1.131	-2.568	0.012
ENSG00000122435	TRMT13	-0.163	4.122	-2.023	0.046
ENSG00000122674	CCZ1	-0.349	3.709	-2.509	0.014
ENSG00000122687	MRM2	0.191	3.805	2.003	0.049
ENSG00000122694	GLIPR2	0.407	1.538	2.040	0.045

ENSG00000122705	CLTA	0.198	5.989	1.995	0.049
ENSG00000122707	RECK	-0.127	4.054	-2.036	0.045
ENSG00000122952	ZWINT	0.343	1.316	2.306	0.024
ENSG00000123240	OPTN	0.149	7.941	2.094	0.039
ENSG00000123352	SPATS2	0.180	5.434	2.654	0.010
ENSG00000123360	PDE1B	0.165	5.155	2.157	0.034
ENSG00000123395	ATG101	0.287	2.892	2.543	0.013
ENSG00000123415	SMUG1	0.146	3.443	2.031	0.046
ENSG00000123685	BATF3	0.368	1.334	2.242	0.028
ENSG00000123992	DNPEP	0.213	2.917	2.118	0.037
ENSG00000124091	GCNT7	-0.398	0.617	-2.284	0.025
ENSG00000124212	PTGIS	0.478	1.881	2.755	0.007
ENSG00000124225	PMEPA1	0.168	4.814	2.010	0.048
ENSG00000124257	NEURL2	0.493	-1.354	2.202	0.031
ENSG00000124541	RRP36	0.186	4.229	2.078	0.041
ENSG00000124549	BTN2A3P	-0.531	-0.152	-2.137	0.036
ENSG00000124608	AARS2	0.195	3.921	2.523	0.014
ENSG00000124766	SOX4	0.295	3.296	2.259	0.027
ENSG00000125149	C16orf70	0.181	3.935	2.541	0.013
ENSG00000125650	PSPN	0.278	1.385	2.323	0.023
ENSG00000125651	GTF2F1	0.198	5.633	2.559	0.012
ENSG00000125656	CLPP	0.167	2.900	2.141	0.035
ENSG00000125744	RTN2	0.247	3.954	2.360	0.021
ENSG00000125835	SNRPB	0.287	2.165	2.511	0.014
ENSG00000125877	ITPA	0.242	2.905	2.428	0.017
ENSG00000125895	TMEM74B	0.212	2.424	2.055	0.043
ENSG00000125967	NECAB3	0.281	4.030	2.875	0.005
ENSG00000126070	AGO3	-0.113	6.861	-2.193	0.031
ENSG00000126107	HECTD3	0.149	4.345	2.025	0.046
ENSG00000126259	KIRREL2	0.391	-0.413	2.124	0.037
ENSG00000126267	COX6B1	0.267	5.003	2.267	0.026
ENSG00000126351	THRA	0.147	7.277	2.123	0.037
ENSG00000126457	PRMT1	0.249	4.446	2.052	0.043
ENSG00000126602	TRAP1	0.150	4.636	2.128	0.036
ENSG00000126934	MAP2K2	0.203	5.050	2.110	0.038
ENSG00000127220	ABHD8	0.281	3.624	2.115	0.037
ENSG00000127252	HRASLS	0.212	2.804	2.090	0.040
ENSG00000127362	TAS2R3	-0.252	1.784	-2.105	0.038
ENSG00000127364	TAS2R4	-0.248	2.966	-2.263	0.026
ENSG00000127366	TAS2R5	-0.480	0.882	-3.005	0.004
ENSG00000127445	PIN1	0.227	5.838	2.303	0.024
ENSG00000127530	OR7C1	-0.555	-0.175	-2.641	0.010
ENSG00000127561	SYNGR3	0.289	4.803	2.927	0.004
ENSG00000128203	ASPHD2	0.298	3.251	3.314	0.001
ENSG00000128266	GNAZ	0.195	6.384	2.283	0.025
ENSG00000128268	MGAT3	0.193	6.079	2.915	0.005
ENSG00000128272	ATF4	0.317	6.428	2.920	0.005

ENSG00000128285	MCHR1	0.384	3.241	2.036	0.045
ENSG00000128298	BAIAP2L2	0.322	1.287	2.716	0.008
ENSG00000128482	RNF112	0.194	4.908	2.472	0.016
ENSG00000128578	STRIP2	0.238	4.911	2.367	0.020
ENSG00000128581	IFT22	0.188	4.211	1.996	0.049
ENSG00000128694	OSGEPL1	-0.166	3.477	-2.235	0.028
ENSG00000129158	SERGEF	0.157	4.921	2.017	0.047
ENSG00000129355	CDKN2D	0.333	4.480	2.929	0.004
ENSG00000129654	FOXJ1	-0.680	-0.636	-2.337	0.022
ENSG00000129925	TMEM8A	0.271	2.665	2.288	0.025
ENSG00000129990	SYT5	0.278	5.562	2.145	0.035
ENSG00000130035	GALNT8	0.260	3.932	3.264	0.002
ENSG00000130226	DPP6	0.192	7.477	2.588	0.011
ENSG00000130227	XPO7	0.126	6.872	2.392	0.019
ENSG00000130244	FAM98C	0.230	3.391	2.315	0.023
ENSG00000130255	RPL36	0.341	4.993	2.041	0.045
ENSG00000130305	NSUN5	0.178	2.147	2.281	0.025
ENSG00000130477	UNC13A	0.263	8.739	2.751	0.007
ENSG00000130511	SSBP4	0.398	2.589	2.296	0.024
ENSG00000130558	OLFM1	0.255	9.005	2.407	0.018
ENSG00000130559	CAMSAP1	0.136	7.081	2.174	0.033
ENSG00000130584	ZBTB46	0.214	3.614	2.350	0.021
ENSG00000130635	COL5A1	0.393	2.312	2.061	0.042
ENSG00000130649	CYP2E1	0.242	3.687	2.062	0.042
ENSG00000130707	ASS1	0.329	3.787	2.288	0.025
ENSG00000130717	UCK1	0.188	3.717	2.308	0.024
ENSG00000130770	ATPIF1	0.183	5.767	2.286	0.025
ENSG00000130813	C19orf66	0.198	3.485	2.604	0.011
ENSG00000130997	POLN	0.223	4.089	2.274	0.026
ENSG00000131016	AKAP12	0.155	9.089	2.261	0.026
ENSG00000131023	LATS1	-0.101	6.188	-2.043	0.044
ENSG00000131143	COX4I1	0.210	7.019	2.059	0.043
ENSG00000131165	CHMP1A	0.259	4.137	2.689	0.009
ENSG00000131187	F12	0.377	0.052	2.550	0.013
ENSG00000131242	RAB11FIP4	0.221	7.962	2.488	0.015
ENSG00000131378	RFTN1	0.221	5.378	2.514	0.014
ENSG00000131469	RPL27	0.323	6.119	2.151	0.034
ENSG00000131473	ACLY	0.182	6.271	2.559	0.012
ENSG00000131503	ANKHD1	-0.227	2.791	-2.032	0.045
ENSG00000131885	KRT17P1	0.719	0.971	2.639	0.010
ENSG00000131914	LIN28A	-0.628	-1.709	-2.167	0.033
ENSG00000131969	ABHD12B	0.271	2.501	2.283	0.025
ENSG00000132026	RTBDN	0.362	0.364	2.277	0.025
ENSG00000132359	RAP1GAP2	0.204	6.961	2.554	0.013
ENSG00000132405	TBC1D14	0.144	5.679	2.193	0.031
ENSG00000132432	SEC61G	0.358	3.141	1.997	0.049
ENSG00000132522	GPS2	-0.284	0.569	-1.993	0.050

ENSG00000132535	DLG4	0.268	7.468	2.915	0.005
ENSG00000132563	REEP2	0.232	5.543	2.512	0.014
ENSG00000132589	FLOT2	0.157	5.840	2.184	0.032
ENSG00000132676	DAP3	0.145	5.258	2.131	0.036
ENSG00000132694	ARHGEF11	0.152	6.972	2.741	0.008
ENSG00000132855	ANGPTL3	-0.351	1.786	-2.340	0.022
ENSG00000132879	FBXO44	0.188	5.269	2.490	0.015
ENSG00000133026	MYH10	0.150	8.441	2.239	0.028
ENSG00000133107	TRPC4	0.260	3.265	2.390	0.019
ENSG00000133265	HSPBP1	0.278	3.651	2.416	0.018
ENSG00000133275	CSNK1G2	0.209	4.577	2.048	0.044
ENSG00000133424	LARGE1	0.196	6.520	2.212	0.030
ENSG00000133627	ACTR3B	0.191	5.108	2.279	0.025
ENSG00000133878	DUSP26	0.244	4.660	2.760	0.007
ENSG00000133962	CATSPERB	0.591	-0.430	3.104	0.003
ENSG00000134463	ECHDC3	0.551	-0.977	2.313	0.023
ENSG00000134779	TPGS2	0.195	6.078	2.771	0.007
ENSG00000134809	TIMM10	0.387	3.627	3.266	0.002
ENSG00000134853	PDGFRA	0.392	6.367	2.442	0.017
ENSG00000134917	ADAMTS8	0.394	3.723	3.354	0.001
ENSG00000135074	ADAM19	0.187	4.239	2.256	0.027
ENSG00000135094	SDS	-0.457	1.224	-2.010	0.048
ENSG00000135127	BICDL1	0.218	5.014	2.190	0.031
ENSG00000135245	HILPDA	0.907	2.179	2.669	0.009
ENSG00000135437	RDH5	-0.608	-1.236	-2.361	0.021
ENSG00000135447	PPP1R1A	0.268	3.717	2.298	0.024
ENSG00000135519	KCNH3	0.258	5.396	2.182	0.032
ENSG00000135540	NHSL1	-0.279	4.867	-2.264	0.026
ENSG00000135547	HEY2	-0.332	2.713	-2.334	0.022
ENSG00000135697	BCO1	0.365	1.761	2.102	0.039
ENSG00000135709	KIAA0513	0.158	8.503	2.094	0.039
ENSG00000135740	SLC9A5	0.234	2.138	2.108	0.038
ENSG00000135749	PCNX2	0.132	7.420	2.423	0.018
ENSG00000135778	NTPCR	0.222	6.036	2.263	0.026
ENSG00000135838	NPL	-0.325	3.754	-2.306	0.024
ENSG00000136026	CKAP4	0.218	4.311	2.577	0.012
ENSG00000136280	CCM2	0.188	4.643	1.992	0.050
ENSG00000136810	TXN	0.378	4.423	2.154	0.034
ENSG00000136842	TMOD1	0.207	4.663	2.024	0.046
ENSG00000136928	GABBR2	0.215	8.312	2.457	0.016
ENSG00000137074	APTX	0.163	4.188	2.329	0.022
ENSG00000137100	DCTN3	0.256	4.579	2.962	0.004
ENSG00000137101	CD72	-0.502	-0.970	-2.434	0.017
ENSG00000137198	GMPR	-0.535	1.414	-2.586	0.011
ENSG00000137216	TMEM63B	0.154	5.177	2.273	0.026
ENSG00000137273	FOXF2	0.408	0.896	2.512	0.014
ENSG00000137309	HMGA1	0.201	3.895	2.321	0.023

ENSG00000137312	FLOT1	0.221	6.240	2.985	0.004
ENSG00000137522	RNF121	0.162	3.935	2.364	0.020
ENSG00000137713	PPP2R1B	-0.213	4.553	-2.115	0.037
ENSG00000137825	ITPKA	0.447	4.092	3.436	0.001
ENSG00000137843	PAK6	0.250	3.491	2.359	0.021
ENSG00000137996	RTCA	0.168	5.403	2.020	0.047
ENSG00000138028	CGREF1	0.265	4.536	3.016	0.003
ENSG00000138073	PREB	0.153	4.061	2.071	0.042
ENSG00000138101	DTNB	0.130	5.300	2.288	0.025
ENSG00000138669	PRKG2	0.375	4.148	2.818	0.006
ENSG00000138764	CCNG2	0.171	6.225	2.236	0.028
ENSG00000138944	KIAA1644	0.255	7.022	2.529	0.013
ENSG00000139144	PIK3C2G	0.682	0.765	2.763	0.007
ENSG00000139193	CD27	-0.656	-1.968	-2.408	0.018
ENSG00000139220	PPFIA2	0.177	8.220	2.236	0.028
ENSG00000139351	SYCP3	-0.342	3.407	-2.503	0.014
ENSG00000139364	TMEM132B	0.151	8.551	2.038	0.045
ENSG00000139405	RITA1	0.223	3.069	2.107	0.038
ENSG00000139579	NABP2	0.188	4.058	2.106	0.038
ENSG00000139668	WDFY2	-0.162	5.017	-2.067	0.042
ENSG00000139714	MORN3	0.295	2.907	2.703	0.008
ENSG00000139874	SSTR1	0.436	3.697	3.702	0.000
ENSG00000139946	PELI2	-0.224	4.679	-2.191	0.031
ENSG00000139998	RAB15	0.229	6.651	2.265	0.026
ENSG00000140057	AK7	0.289	1.936	1.993	0.050
ENSG00000140104	C14orf79	0.316	2.676	3.284	0.002
ENSG00000140465	CYP1A1	0.734	-0.948	2.798	0.006
ENSG00000140632	GLYR1	0.176	6.529	2.890	0.005
ENSG00000140688	C16orf58	0.154	3.680	2.091	0.040
ENSG00000140691	ARMC5	0.216	2.458	2.129	0.036
ENSG00000140995	DEF8	0.244	5.112	2.698	0.008
ENSG00000141002	TCF25	0.155	6.155	2.559	0.012
ENSG00000141013	GAS8	0.228	4.574	2.111	0.038
ENSG00000141026	MED9	0.175	4.437	2.612	0.011
ENSG00000141098	GFOD2	0.195	5.066	2.393	0.019
ENSG00000141198	TOM1L1	0.195	4.672	2.395	0.019
ENSG00000141385	AFG3L2	0.180	5.909	2.136	0.036
ENSG00000141480	ARRB2	0.158	4.381	2.095	0.039
ENSG00000141504	SAT2	0.190	3.980	2.192	0.031
ENSG00000141542	RAB40B	0.253	6.173	2.932	0.004
ENSG00000141562	NARF	0.120	4.894	2.042	0.044
ENSG00000141622	RNF165	0.247	5.261	2.671	0.009
ENSG00000141698	NT5C3B	0.261	5.052	2.265	0.026
ENSG00000142039	CCDC97	-0.199	3.212	-2.131	0.036
ENSG00000142459	EVI5L	0.214	4.866	2.617	0.011
ENSG00000142657	PGD	0.168	5.028	2.133	0.036
ENSG00000142675	CNKSR1	-0.403	0.432	-2.326	0.022

ENSG00000142686	C1orf216	0.233	6.539	2.032	0.045
ENSG00000143179	UCK2	0.148	4.506	2.041	0.045
ENSG00000143190	POU2F1	-0.194	6.388	-2.641	0.010
ENSG00000143222	UFC1	0.274	4.234	2.461	0.016
ENSG00000143314	MRPL24	0.242	4.251	2.117	0.037
ENSG00000143340	FAM163A	0.612	1.765	3.159	0.002
ENSG00000143344	RGL1	0.178	7.118	2.262	0.026
ENSG00000143420	ENSA	0.201	7.791	2.268	0.026
ENSG00000143436	MRPL9	0.240	3.648	2.467	0.016
ENSG00000143590	EFNA3	0.284	2.741	2.262	0.026
ENSG00000143774	GUK1	0.326	5.979	2.148	0.035
ENSG00000143862	ARL8A	0.173	4.881	2.037	0.045
ENSG00000143870	PDIA6	0.243	5.159	2.288	0.025
ENSG00000144152	FBLN7	0.348	2.772	2.363	0.020
ENSG00000144161	ZC3H8	0.171	5.848	2.498	0.015
ENSG00000144230	GPR17	0.431	2.950	2.519	0.014
ENSG00000144550	CPNE9	0.304	3.235	2.334	0.022
ENSG00000144619	CNTN4	0.242	5.924	2.273	0.026
ENSG00000144668	ITGA9	0.152	4.937	2.165	0.033
ENSG00000144713	RPL32	0.383	6.252	2.261	0.026
ENSG00000145022	TCTA	0.169	4.200	2.467	0.016
ENSG00000145416	MARCHF1	0.206	5.848	2.088	0.040
ENSG00000145476	CYP4V2	-0.320	5.827	-2.505	0.014
ENSG00000145736	GTF2H2	-0.739	3.181	-3.384	0.001
ENSG00000145794	MEGF10	-0.300	6.168	-2.089	0.040
ENSG00000145832	SLC25A48	-0.308	2.797	-2.050	0.044
ENSG00000145901	TNIP1	0.170	4.790	2.099	0.039
ENSG00000145916	RMND5B	0.144	4.400	2.373	0.020
ENSG00000145919	BOD1	0.161	4.676	2.184	0.032
ENSG00000145934	TENM2	0.151	6.977	2.043	0.044
ENSG00000146007	ZMAT2	0.228	6.253	2.057	0.043
ENSG00000146232	NFKBIE	0.261	2.114	2.194	0.031
ENSG00000146477	SLC22A3	0.499	0.785	3.009	0.003
ENSG00000147457	CHMP7	0.153	5.042	2.127	0.036
ENSG00000147955	SIGMAR1	0.175	3.771	2.726	0.008
ENSG00000148082	SHC3	0.177	9.137	2.158	0.034
ENSG00000148123	PLPPR1	0.313	4.161	2.177	0.032
ENSG00000148290	SURF1	0.367	1.691	2.486	0.015
ENSG00000148300	REXO4	0.306	3.993	3.449	0.001
ENSG00000148331	ASB6	0.149	3.991	2.005	0.048
ENSG00000148334	PTGES2	0.209	4.719	2.053	0.043
ENSG00000148513	ANKRD30A	0.540	-0.811	2.167	0.033
ENSG00000148606	POLR3A	0.160	6.246	2.727	0.008
ENSG00000148655	C10orf11	-0.320	1.816	-2.160	0.034
ENSG00000148814	LRRC27	0.204	4.269	2.914	0.005
ENSG00000148843	PDCD11	0.126	5.119	2.252	0.027
ENSG00000148926	ADM	0.828	0.710	2.265	0.026

ENSG00000149091	DGKZ	0.360	6.307	2.334	0.022
ENSG00000149136	SSRP1	0.122	5.629	2.046	0.044
ENSG00000149428	HYOU1	0.142	6.982	2.029	0.046
ENSG00000149506	ZP1	0.637	-1.725	2.063	0.042
ENSG00000149527	PLCH2	0.455	4.851	3.047	0.003
ENSG00000149541	B3GAT3	0.226	2.799	2.482	0.015
ENSG00000149547	EI24	0.216	5.152	2.252	0.027
ENSG00000149571	KIRREL3	0.257	4.684	3.106	0.003
ENSG00000149609	C20orf144	0.422	0.039	2.120	0.037
ENSG00000149654	CDH22	0.296	4.130	2.272	0.026
ENSG00000149761	NUDT22	0.353	1.139	2.357	0.021
ENSG00000149792	MRPL49	0.252	4.153	2.739	0.008
ENSG00000149809	TM7SF2	0.246	3.420	2.682	0.009
ENSG00000149927	DOC2A	0.232	5.363	2.410	0.018
ENSG00000150275	PCDH15	0.252	5.352	2.119	0.037
ENSG00000150556	LYPD6B	0.336	1.195	2.441	0.017
ENSG00000150712	MTMR12	0.129	5.909	2.197	0.031
ENSG00000150977	RILPL2	0.311	3.245	3.161	0.002
ENSG00000151062	CACNA2D4	-0.547	-1.051	-2.421	0.018
ENSG00000151276	MAGI1	0.187	7.569	2.842	0.006
ENSG00000151640	DPYSL4	0.305	3.842	3.277	0.002
ENSG00000151952	TMEM132D	0.314	5.830	3.891	0.000
ENSG00000152128	TMEM163	0.206	2.747	2.121	0.037
ENSG00000152208	GRID2	0.215	4.909	2.132	0.036
ENSG00000152214	RIT2	0.341	3.795	2.447	0.017
ENSG00000152284	TCF7L1	-0.334	0.853	-2.194	0.031
ENSG00000152413	HOMER1	0.302	7.857	2.455	0.016
ENSG00000152463	OLAH	-0.620	-1.007	-2.458	0.016
ENSG00000152467	ZSCAN1	0.245	1.313	2.114	0.038
ENSG00000152936	LMNTD1	-0.599	-0.636	-2.410	0.018
ENSG00000153317	ASAP1	0.126	7.019	2.161	0.034
ENSG00000153406	NMRAL1	0.177	4.091	2.205	0.030
ENSG00000153487	ING1	0.162	3.999	2.421	0.018
ENSG00000153563	CD8A	0.390	1.090	3.495	0.001
ENSG00000153823	PID1	0.226	5.166	2.260	0.026
ENSG00000153832	FBXO36	0.172	3.157	2.025	0.046
ENSG00000154328	NEIL2	0.247	4.496	2.725	0.008
ENSG00000154358	OBSCN	0.247	4.434	2.077	0.041
ENSG00000154370	TRIM11	0.220	3.530	2.147	0.035
ENSG00000154478	GPR26	0.592	5.303	3.568	0.001
ENSG00000154898	CCDC144CP	-0.282	6.000	-2.428	0.017
ENSG00000154975	CA10	0.395	6.193	2.775	0.007
ENSG00000155265	GOLGA7B	0.197	4.624	2.780	0.007
ENSG00000155324	GRAMD3	-0.261	5.582	-2.077	0.041
ENSG00000156345	CDK20	0.248	4.178	3.185	0.002
ENSG00000156395	SORCS3	0.183	5.461	2.058	0.043
ENSG00000156535	CD109	-0.328	3.546	-2.172	0.033

ENSG00000156873	PHKG2	0.143	3.192	2.000	0.049
ENSG00000157020	SEC13	0.144	4.702	2.086	0.040
ENSG00000157045	NTAN1	0.294	3.106	2.348	0.021
ENSG00000157368	IL34	0.295	2.000	2.027	0.046
ENSG00000157613	CREB3L1	0.311	1.556	2.061	0.043
ENSG00000157741	UBN2	-0.110	7.167	-2.085	0.040
ENSG00000157782	CABP1	0.219	6.955	2.122	0.037
ENSG00000157851	DPYSL5	0.250	4.912	2.649	0.010
ENSG00000157881	PANK4	0.244	3.496	3.198	0.002
ENSG00000157895	C12orf43	0.196	4.680	2.860	0.005
ENSG00000158042	MRPL17	0.213	4.031	2.248	0.027
ENSG00000158062	UBXN11	0.203	3.144	2.074	0.041
ENSG00000158552	ZFAND2B	0.204	3.854	2.801	0.006
ENSG00000158773	USF1	0.194	4.596	2.095	0.039
ENSG00000158806	NPM2	0.231	4.554	2.667	0.009
ENSG00000158856	DMTN	0.196	6.700	2.101	0.039
ENSG00000158882	TOMM40L	0.291	4.055	2.921	0.005
ENSG00000159055	MIS18A	0.206	1.652	2.014	0.047
ENSG00000159128	IFNGR2	0.153	4.618	2.054	0.043
ENSG00000159199	ATP5G1	0.260	3.132	2.005	0.048
ENSG00000159352	PSMD4	0.273	5.986	2.277	0.025
ENSG00000159409	CELF3	0.271	5.546	2.223	0.029
ENSG00000159479	MED8	0.170	4.481	2.525	0.014
ENSG00000159625	DRC7	0.409	-0.591	2.124	0.037
ENSG00000159685	CHCHD6	0.272	3.929	2.673	0.009
ENSG00000159733	ZFYVE28	0.229	4.717	2.085	0.040
ENSG00000159753	CARMIL2	0.216	3.156	2.288	0.025
ENSG00000159784	FAM131B	0.170	6.831	2.080	0.041
ENSG00000159788	RGS12	0.198	5.496	2.050	0.044
ENSG00000159842	ABR	0.176	8.299	2.215	0.030
ENSG00000159884	CCDC107	0.931	-1.539	3.534	0.001
ENSG00000159917	ZNF235	-0.161	3.603	-2.137	0.036
ENSG00000160050	CCDC28B	0.230	1.770	2.173	0.033
ENSG00000160058	BSDC1	0.136	6.113	2.218	0.029
ENSG00000160097	FNDC5	0.250	4.880	2.525	0.013
ENSG00000160145	KALRN	0.186	9.190	3.003	0.004
ENSG00000160201	U2AF1	-0.778	2.005	-2.269	0.026
ENSG00000160321	ZNF208	-0.968	3.107	-3.796	0.000
ENSG00000160469	BRSK1	0.226	6.600	2.351	0.021
ENSG00000160688	FLAD1	0.174	3.022	2.111	0.038
ENSG00000160691	SHC1	0.270	3.372	2.400	0.019
ENSG00000160963	COL26A1	0.348	3.580	2.818	0.006
ENSG00000160993	ALKBH4	0.236	1.903	2.113	0.038
ENSG00000161013	MGAT4B	0.231	4.443	2.596	0.011
ENSG00000161016	RPL8	0.247	5.332	2.093	0.039
ENSG00000161082	CELF5	0.251	5.821	2.399	0.019
ENSG00000161179	YDJC	0.372	1.853	3.110	0.003

ENSG00000161328	LRRC56	0.440	0.573	2.365	0.020
ENSG00000161526	SAP30BP	0.207	5.169	3.009	0.003
ENSG00000161544	CYGB	0.267	2.253	2.273	0.026
ENSG00000161835	GRASP	0.273	3.554	2.303	0.024
ENSG00000161944	ASGR2	0.502	-0.976	2.085	0.040
ENSG00000161970	RPL26	0.367	5.923	2.614	0.011
ENSG00000161981	SNRNP25	0.352	3.748	2.629	0.010
ENSG00000162065	TBC1D24	0.167	5.778	2.375	0.020
ENSG00000162076	FLYWCH2	0.299	2.326	2.392	0.019
ENSG00000162222	TTC9C	0.150	3.731	2.100	0.039
ENSG00000162390	ACOT11	-0.289	5.322	-2.249	0.027
ENSG00000162461	SLC25A34	0.332	0.527	2.023	0.046
ENSG00000162545	CAMK2N1	0.287	8.663	3.106	0.003
ENSG00000162687	KCNT2	0.180	5.816	2.182	0.032
ENSG00000162699	DNAJA1P5	-0.511	1.500	-2.131	0.036
ENSG00000162706	CADM3	0.213	6.832	2.490	0.015
ENSG00000162722	TRIM58	0.558	1.707	2.300	0.024
ENSG00000162728	KCNJ9	0.315	6.713	3.377	0.001
ENSG00000162729	IGSF8	0.175	5.894	1.992	0.050
ENSG00000162734	PEA15	0.170	8.867	2.104	0.038
ENSG00000162738	VANGL2	-0.267	2.763	-2.622	0.010
ENSG00000162755	KLHDC9	0.252	2.431	2.310	0.023
ENSG00000162813	BPNT1	0.200	3.624	2.016	0.047
ENSG00000162814	SPATA17	0.356	1.054	2.072	0.041
ENSG00000162913	C1orf145	0.580	1.235	3.681	0.000
ENSG00000162931	TRIM17	0.246	3.310	2.242	0.028
ENSG00000162975	KCNF1	0.268	4.341	2.099	0.039
ENSG00000162999	DUSP19	0.218	4.715	2.172	0.033
ENSG00000163001	CFAP36	0.168	6.118	2.000	0.049
ENSG00000163331	DAPL1	-1.173	-2.962	-3.168	0.002
ENSG00000163362	C1orf106	0.499	-0.814	2.481	0.015
ENSG00000163382	NAXE	0.214	3.939	2.235	0.028
ENSG00000163517	HDAC11	0.232	4.721	2.348	0.021
ENSG00000163545	NUAK2	-0.544	-1.014	-2.300	0.024
ENSG00000163673	DCLK3	0.450	2.823	2.794	0.006
ENSG00000163703	CRELD1	0.193	5.047	3.185	0.002
ENSG00000163749	CCDC158	0.293	3.576	2.542	0.013
ENSG00000163872	YEATS2	0.098	6.731	2.279	0.025
ENSG00000163923	RPL39L	0.490	0.737	2.515	0.014
ENSG00000163930	BAP1	0.220	6.220	2.357	0.021
ENSG00000163956	LRPAP1	0.184	6.219	2.348	0.021
ENSG00000164062	APEH	0.189	4.821	2.748	0.007
ENSG00000164068	RNF123	0.181	4.267	2.089	0.040
ENSG00000164076	CAMKV	0.288	7.065	3.132	0.002
ENSG00000164087	POC1A	0.362	0.090	2.139	0.035
ENSG00000164211	STARD4	0.240	6.146	2.329	0.022
ENSG00000164326	CARTPT	0.688	3.671	2.545	0.013

ENSG00000164344	KLKB1	-0.460	2.019	-2.712	0.008
ENSG00000164483	SAMD3	0.356	0.088	2.058	0.043
ENSG00000164484	TMEM200A	0.411	2.667	2.157	0.034
ENSG00000164587	RPS14	0.317	5.228	2.494	0.015
ENSG00000164733	CTSB	0.155	7.718	2.113	0.038
ENSG00000164764	SBSPON	-0.386	1.644	-3.081	0.003
ENSG00000164849	GPR146	0.384	0.241	2.360	0.021
ENSG00000164879	CA3	0.316	0.543	2.170	0.033
ENSG00000164978	NUDT2	0.297	3.153	2.641	0.010
ENSG00000165478	HEPACAM	-0.260	5.831	-2.350	0.021
ENSG00000165688	PMPCA	0.169	4.269	2.330	0.022
ENSG00000165724	ZMYND19	0.200	3.040	2.016	0.047
ENSG00000165752	STK32C	0.341	3.763	2.963	0.004
ENSG00000165802	NSMF	0.248	6.909	2.393	0.019
ENSG00000165923	AGBL2	-0.548	-1.136	-2.044	0.044
ENSG00000166035	LIPC	0.410	0.205	2.126	0.037
ENSG00000166111	SVOP	0.275	6.768	2.774	0.007
ENSG00000166140	ZFYVE19	0.178	3.472	2.230	0.028
ENSG00000166148	AVPR1A	0.417	0.842	2.048	0.044
ENSG00000166153	DEPDC4	0.333	0.222	2.525	0.013
ENSG00000166313	APBB1	0.194	7.019	2.253	0.027
ENSG00000166323	C11orf65	-0.202	1.981	-2.051	0.044
ENSG00000166402	TUB	0.154	7.441	2.185	0.032
ENSG00000166441	RPL27A	0.223	5.470	2.077	0.041
ENSG00000166535	A2ML1	-0.298	2.944	-2.254	0.027
ENSG00000166682	TMPRSS5	-0.354	1.797	-2.261	0.026
ENSG00000166793	YPEL4	0.171	4.646	2.094	0.039
ENSG00000166819	PLIN1	-0.498	1.208	-3.001	0.004
ENSG00000166823	MESP1	0.405	-0.004	1.992	0.050
ENSG00000166847	DCTN5	0.152	7.753	2.081	0.041
ENSG00000166887	VPS39	0.104	6.396	2.076	0.041
ENSG00000166896	ATP23	0.365	1.822	3.558	0.001
ENSG00000166949	SMAD3	0.162	6.010	2.148	0.035
ENSG00000166959	MS4A8	0.623	-0.834	2.101	0.039
ENSG00000166960	CCDC178	0.532	0.714	3.025	0.003
ENSG00000166965	RCCD1	0.177	3.839	2.366	0.020
ENSG00000166986	MARS	0.124	6.004	2.262	0.026
ENSG00000167004	PDIA3	0.258	5.407	2.006	0.048
ENSG00000167074	TEF	0.182	6.430	2.306	0.024
ENSG00000167085	PHB	0.270	3.829	2.142	0.035
ENSG00000167094	TTC16	-0.416	-0.957	-2.005	0.048
ENSG00000167110	GOLGA2	0.177	5.858	2.847	0.006
ENSG00000167112	TRUB2	0.192	5.042	2.251	0.027
ENSG00000167118	URM1	0.209	3.166	2.047	0.044
ENSG00000167210	LOXHD1	0.388	1.391	2.114	0.038
ENSG00000167264	DUS2	0.245	2.291	2.240	0.028
ENSG00000167323	STIM1	0.149	5.882	2.185	0.032

ENSG00000167515	TRAPPC2L	0.263	4.020	2.599	0.011
ENSG00000167526	RPL13	0.282	6.012	2.300	0.024
ENSG00000167535	CACNB3	0.368	5.212	3.762	0.000
ENSG00000167552	TUBA1A	0.300	8.182	2.011	0.048
ENSG00000167588	GPD1	0.438	2.436	2.621	0.010
ENSG00000167654	ATCAY	0.230	7.658	2.930	0.004
ENSG00000167658	EEF2	0.191	8.974	2.068	0.042
ENSG00000167676	PLIN4	-0.472	1.890	-2.764	0.007
ENSG00000167680	SEMA6B	0.259	5.122	2.163	0.033
ENSG00000167711	SERPINF2	-0.644	-1.808	-1.997	0.049
ENSG00000167723	TRPV3	-0.278	3.435	-2.491	0.015
ENSG00000167733	HSD11B1L	0.232	3.498	2.169	0.033
ENSG00000167747	C19orf48	0.261	0.837	2.031	0.045
ENSG00000167771	RCOR2	0.309	2.534	2.582	0.012
ENSG00000167778	SPRYD3	0.144	6.530	2.113	0.038
ENSG00000167792	NDUFV1	0.157	6.448	2.093	0.039
ENSG00000167863	ATP5H	0.264	5.515	2.010	0.048
ENSG00000168060	NAALADL1	0.785	-2.027	2.226	0.029
ENSG00000168067	MAP4K2	0.166	4.771	2.414	0.018
ENSG00000168243	GNG4	0.341	4.843	3.636	0.000
ENSG00000168405	CMAHP	-0.332	3.664	-2.367	0.020
ENSG00000168490	PHYHIP	0.190	7.879	2.041	0.044
ENSG00000168495	POLR3D	0.181	5.018	2.761	0.007
ENSG00000168556	ING2	0.256	3.747	2.786	0.007
ENSG00000168569	TMEM223	0.226	2.494	2.066	0.042
ENSG00000168734	PKIG	0.278	5.073	2.916	0.005
ENSG00000168748	CA7	0.336	1.627	2.963	0.004
ENSG00000168802	CHTF8	0.165	3.367	2.060	0.043
ENSG00000168852	TPTE2P5	-0.242	1.981	-2.091	0.040
ENSG00000168924	LETM1	0.138	5.717	2.165	0.033
ENSG00000169087	HSPBAP1	-0.165	3.484	-2.085	0.040
ENSG00000169129	AFAP1L2	0.181	4.645	2.078	0.041
ENSG00000169181	GSG1L	0.379	3.349	3.221	0.002
ENSG00000169189	NSMCE1	0.282	3.553	2.623	0.010
ENSG00000169220	RGS14	0.302	2.549	2.161	0.034
ENSG00000169258	GPRIN1	0.244	4.888	2.527	0.013
ENSG00000169884	WNT10B	0.291	2.991	2.612	0.011
ENSG00000169926	KLF13	0.202	6.512	2.830	0.006
ENSG00000169976	SF3B5	0.237	2.778	2.170	0.033
ENSG00000170004	CHD3	0.214	7.910	2.552	0.013
ENSG00000170091	HMP19	0.327	8.202	2.299	0.024
ENSG00000170379	TCAF2	0.482	1.196	2.443	0.017
ENSG00000170439	METTL7B	-0.458	-0.054	-2.073	0.041
ENSG00000170515	PA2G4	0.201	4.994	2.431	0.017
ENSG00000170653	ATF7	-0.207	3.900	-2.105	0.038
ENSG00000170837	GPR27	0.207	3.762	2.501	0.014
ENSG00000170906	NDUFA3	0.215	3.248	2.200	0.031

ENSG00000170917	NUDT6	-0.203	3.048	-2.001	0.049
ENSG00000170961	HAS2	0.431	0.892	2.800	0.006
ENSG00000171130	ATP6V0E2	0.207	5.956	2.218	0.029
ENSG00000171160	MORN4	0.205	4.686	3.082	0.003
ENSG00000171217	CLDN20	-0.324	1.272	-2.456	0.016
ENSG00000171302	CANT1	0.145	3.769	2.199	0.031
ENSG00000171303	KCNK3	0.383	3.438	3.298	0.001
ENSG00000171462	DLK2	0.316	0.815	2.243	0.028
ENSG00000171631	P2RY6	0.661	-0.950	2.239	0.028
ENSG00000171720	HDAC3	0.149	4.147	2.340	0.022
ENSG00000171861	MRM3	0.236	2.352	2.321	0.023
ENSG00000171865	RNASEH1	0.216	3.935	2.306	0.024
ENSG00000171951	SCG2	0.379	7.812	2.085	0.040
ENSG00000172006	ZNF554	0.173	3.847	2.569	0.012
ENSG00000172053	QARS	0.183	4.291	2.332	0.022
ENSG00000172137	CALB2	0.555	3.813	3.058	0.003
ENSG00000172346	CSDC2	0.364	2.767	2.138	0.035
ENSG00000172375	C2CD2L	0.198	4.868	2.421	0.018
ENSG00000172404	DNAJB7	-0.285	0.371	-2.127	0.036
ENSG00000172426	RSPH9	0.408	0.595	3.359	0.001
ENSG00000172578	KLHL6	-0.688	0.835	-2.367	0.020
ENSG00000172602	RND1	0.297	4.496	2.605	0.011
ENSG00000172728	FUT10	-0.271	4.595	-2.066	0.042
ENSG00000172757	CFL1	0.200	7.220	2.285	0.025
ENSG00000172803	SNX32	0.305	3.490	2.204	0.030
ENSG00000172831	CES2	0.247	4.438	3.378	0.001
ENSG00000172932	ANKRD13D	0.192	4.404	2.635	0.010
ENSG00000173020	GRK2	0.199	6.027	2.150	0.034
ENSG00000173083	HPSE	-0.402	1.083	-2.018	0.047
ENSG00000173141	MRPL57	0.188	4.305	2.151	0.034
ENSG00000173200	PARP15	-0.642	-1.199	-2.409	0.018
ENSG00000173253	DMRT2	-0.669	-0.408	-2.245	0.027
ENSG00000173406	DAB1	0.226	4.660	2.248	0.027
ENSG00000173465	SSSCA1	0.228	2.659	2.468	0.016
ENSG00000173486	FKBP2	0.214	3.933	2.068	0.042
ENSG00000173581	CCDC106	0.234	3.055	2.044	0.044
ENSG00000173614	NMNAT1	0.207	3.350	2.538	0.013
ENSG00000173621	LRFN4	0.296	1.657	2.065	0.042
ENSG00000173889	PHC3	-0.104	7.396	-2.015	0.047
ENSG00000173898	SPTBN2	0.233	8.098	2.663	0.009
ENSG00000173991	TCAP	0.481	0.525	3.500	0.001
ENSG00000174151	CYB561D1	0.230	3.725	2.588	0.011
ENSG00000174233	ADCY6	0.207	4.705	2.120	0.037
ENSG00000174428	GTF2IRD2B	-0.314	2.526	-2.519	0.014
ENSG00000174444	RPL4	0.323	7.232	2.026	0.046
ENSG00000174473	GALNTL6	0.272	3.738	2.611	0.011
ENSG00000174482	LINGO2	0.260	5.127	2.490	0.015

ENSG00000174514	MFSD4A	0.259	6.534	2.372	0.020
ENSG00000174521	TTC9B	0.310	3.487	2.222	0.029
ENSG00000174718	KIAA1551	-0.099	6.390	-2.043	0.044
ENSG00000174775	HRAS	0.325	3.803	3.060	0.003
ENSG00000174871	CNIH2	0.328	2.820	2.406	0.018
ENSG00000174951	FUT1	0.304	2.468	2.632	0.010
ENSG00000174989	FBXW8	-0.152	3.852	-2.047	0.044
ENSG00000175048	ZDHHC14	0.252	5.016	3.332	0.001
ENSG00000175137	SH3BP5L	0.211	4.798	2.636	0.010
ENSG00000175182	FAM131A	0.287	5.805	3.517	0.001
ENSG00000175203	DCTN2	0.178	5.931	2.334	0.022
ENSG00000175274	TP53I11	0.498	4.267	2.835	0.006
ENSG00000175416	CLTB	0.201	5.506	2.038	0.045
ENSG00000175550	DRAP1	0.227	5.099	2.146	0.035
ENSG00000175664	TEX26	-0.410	0.550	-2.320	0.023
ENSG00000175787	ZNF169	0.146	3.428	2.085	0.040
ENSG00000175792	RUVBL1	0.203	4.000	2.425	0.018
ENSG00000175832	ETV4	0.402	0.062	2.124	0.037
ENSG00000175857	GAPT	-0.937	-1.815	-2.036	0.045
ENSG00000175866	BAIAP2	0.222	5.538	2.211	0.030
ENSG00000175946	KLHL38	-0.685	-1.573	-2.448	0.016
ENSG00000176022	B3GALT6	0.206	3.355	2.485	0.015
ENSG00000176171	BNIP3	0.250	6.590	2.686	0.009
ENSG00000176225	RTTN	-0.261	4.952	-2.535	0.013
ENSG00000176584	DMBT1P1	0.649	-0.468	2.036	0.045
ENSG00000176749	CDK5R1	0.186	7.217	1.994	0.049
ENSG00000176857	GJA1P1	-0.443	2.349	-3.245	0.002
ENSG00000176871	WSB2	0.313	7.481	3.106	0.003
ENSG00000176887	SOX11	0.323	3.247	3.191	0.002
ENSG00000176946	THAP4	0.148	4.241	2.048	0.044
ENSG00000176956	LY6H	0.371	4.364	3.248	0.002
ENSG00000177030	DEAF1	0.262	5.421	2.752	0.007
ENSG00000177042	TMEM80	0.216	2.506	2.166	0.033
ENSG00000177173	NAP1L4P1	-0.563	2.221	-2.057	0.043
ENSG00000177225	PDDC1	0.246	4.983	2.552	0.013
ENSG00000177370	TIMM22	0.171	3.933	2.834	0.006
ENSG00000177380	PPFIA3	0.217	5.749	2.450	0.016
ENSG00000177427	MIEF2	0.232	2.840	2.667	0.009
ENSG00000177519	RPRM	0.325	2.057	2.040	0.045
ENSG00000177614	PGBD5	0.180	7.037	2.348	0.021
ENSG00000177675	CD163L1	0.381	2.614	2.672	0.009
ENSG00000177706	FAM20C	0.247	3.533	2.034	0.045
ENSG00000177707	NECTIN3	0.219	5.980	2.067	0.042
ENSG00000177879	AP3S1	0.202	5.731	2.102	0.039
ENSG00000177963	RIC8A	0.210	5.090	2.876	0.005
ENSG00000177993	ZNRF3-AS1	-0.462	0.829	-2.414	0.018
ENSG00000178105	DDX10	0.175	5.688	2.537	0.013

ENSG00000178150	ZNF114	0.318	0.408	2.007	0.048
ENSG00000178217	SH2D4B	0.548	-0.787	3.096	0.003
ENSG00000178297	TMPRSS9	0.466	-0.166	2.300	0.024
ENSG00000178467	P4HTM	0.158	5.223	2.633	0.010
ENSG00000178723	GLULP4	-0.872	2.134	-2.300	0.024
ENSG00000178773	CPNE7	0.279	3.169	2.102	0.039
ENSG00000178796	RIIAD1	0.240	1.847	2.242	0.028
ENSG00000178952	TUFM	0.165	5.550	2.051	0.043
ENSG00000178982	EIF3K	0.269	5.076	3.368	0.001
ENSG00000178997	EXD1	0.876	-1.264	2.696	0.009
ENSG00000179038	RP11-157K17.5	-0.441	0.853	-2.434	0.017
ENSG00000179057	IGSF22	0.272	1.862	1.995	0.049
ENSG00000179218	CALR	0.200	7.007	2.300	0.024
ENSG00000179447	RP5-1027G4.3	-0.537	-0.912	-2.330	0.022
ENSG00000180189	HMGB1P14	-0.735	-0.024	-3.715	0.000
ENSG00000180304	OAZ2	0.111	5.486	2.097	0.039
ENSG00000180481	GLIPR1L2	-0.522	1.561	-2.326	0.023
ENSG00000180747	SMG1P3	-0.284	5.672	-2.006	0.048
ENSG00000180801	ARSJ	-0.275	3.281	-2.927	0.004
ENSG00000180875	GREM2	0.314	3.651	2.564	0.012
ENSG00000180901	KCTD2	0.147	6.018	2.128	0.036
ENSG00000181085	MAPK15	0.695	-0.591	2.505	0.014
ENSG00000181215	C4orf50	0.172	7.110	2.001	0.049
ENSG00000181234	TMEM132C	0.186	3.735	2.224	0.029
ENSG00000181418	DDN	0.402	7.161	3.231	0.002
ENSG00000181458	TMEM45A	0.393	0.332	2.170	0.033
ENSG00000181499	OR6T1	1.292	-2.676	3.345	0.001
ENSG00000181577	C6orf223	0.795	-0.897	3.274	0.002
ENSG00000181610	MRPS23	0.221	6.002	2.109	0.038
ENSG00000181652	ATG9B	0.359	0.863	2.407	0.018
ENSG00000181929	PRKAG1	0.203	5.043	2.513	0.014
ENSG00000181991	MRPS11	0.165	4.334	2.267	0.026
ENSG00000182158	CREB3L2	-0.219	3.932	-2.026	0.046
ENSG00000182199	SHMT2	0.244	3.002	2.719	0.008
ENSG00000182208	MOB2	0.204	3.289	2.217	0.029
ENSG00000182272	B4GALNT4	0.271	4.746	2.381	0.020
ENSG00000182347	PDSS1P1	-0.349	1.103	-3.243	0.002
ENSG00000182400	TRAPPC6B	0.188	5.126	2.070	0.042
ENSG00000182473	EXOC7	0.126	6.162	2.028	0.046
ENSG00000182631	RXFP3	1.098	-2.313	2.919	0.005
ENSG00000182670	TTC3	0.178	9.421	2.334	0.022
ENSG00000182732	RGS6	0.219	5.607	3.315	0.001
ENSG00000182774	RPS17	-0.277	0.952	-2.341	0.022
ENSG00000182870	GALNT9	0.238	4.608	2.112	0.038
ENSG00000182983	ZNF662	-0.179	4.001	-2.603	0.011
ENSG00000183010	PYCR1	0.297	0.778	2.020	0.047
ENSG00000183018	SPNS2	0.180	3.735	1.991	0.050

ENSG00000183036	PCP4	0.300	3.376	2.034	0.045
ENSG00000183186	C2CD4C	0.451	3.151	2.849	0.006
ENSG00000183207	RUVBL2	0.248	4.374	2.447	0.017
ENSG00000183317	EPHA10	0.253	3.607	2.415	0.018
ENSG00000183474	GTF2H2C	-0.600	3.089	-3.173	0.002
ENSG00000183562	CTC-343N3.1	-0.363	2.497	-2.663	0.009
ENSG00000183597	TANGO2	0.140	3.652	2.009	0.048
ENSG00000183604	SMG1P5	-0.736	4.534	-2.909	0.005
ENSG00000183729	NPBWR1	1.012	-0.941	3.084	0.003
ENSG00000183741	CBX6	0.261	7.717	2.992	0.004
ENSG00000183778	B3GALT5	-0.327	2.616	-2.986	0.004
ENSG00000183784	C9orf66	-0.967	-2.298	-2.400	0.019
ENSG00000183826	BTBD9	0.155	6.822	2.661	0.009
ENSG00000183856	IQGAP3	-0.554	0.455	-2.305	0.024
ENSG00000183873	SCN5A	0.422	1.524	2.284	0.025
ENSG00000183955	KMT5A	0.177	4.261	2.102	0.039
ENSG00000183978	COA3	0.283	3.599	2.161	0.034
ENSG00000184009	ACTG1	0.152	8.673	1.992	0.050
ENSG00000184185	KCNJ12	0.351	3.011	3.149	0.002
ENSG00000184207	PGP	0.208	3.732	2.527	0.013
ENSG00000184261	KCNK12	0.205	4.357	2.386	0.019
ENSG00000184608	FAM167A-AS1	-0.432	-0.487	-2.212	0.030
ENSG00000184887	BTBD6	0.238	4.762	2.822	0.006
ENSG00000185052	SLC24A3	0.254	3.826	2.114	0.038
ENSG00000185290	NUPR2	0.799	-0.974	3.058	0.003
ENSG00000185298	CCDC137	0.212	2.969	2.658	0.009
ENSG00000185306	C12orf56	-0.445	0.921	-2.617	0.011
ENSG00000185361	TNFAIP8L1	0.304	3.758	3.418	0.001
ENSG00000185386	MAPK11	0.270	3.773	2.254	0.027
ENSG00000185614	FAM212A	0.325	0.314	1.997	0.049
ENSG00000185627	PSMD13	0.201	4.730	2.346	0.021
ENSG00000185641	CTD-2287O16.1	-0.823	2.981	-2.089	0.040
ENSG00000185650	ZFP36L1	-0.340	5.466	-2.186	0.032
ENSG00000185710	SMG1P4	-0.651	4.190	-2.303	0.024
ENSG00000185829	ARL17A	-0.499	2.862	-2.224	0.029
ENSG00000185904	LINC00839	0.342	2.721	2.262	0.026
ENSG00000185909	KLHDC8B	0.350	2.851	3.283	0.002
ENSG00000185924	RTN4RL1	0.366	4.338	3.317	0.001
ENSG00000185963	BICD2	0.128	5.896	2.150	0.035
ENSG00000186105	LRRC70	-0.450	0.208	-2.057	0.043
ENSG00000186162	CIDECP	0.287	2.601	2.974	0.004
ENSG00000186184	POLR1D	0.153	4.601	2.228	0.029
ENSG00000186193	SAPCD2	0.346	1.518	2.072	0.041
ENSG00000186222	BLOC1S4	0.297	0.671	2.120	0.037
ENSG00000186329	TMEM212	-0.717	-1.818	-2.173	0.033
ENSG00000186364	NUDT17	0.352	0.877	2.776	0.007
ENSG00000186575	NF2	0.149	6.564	2.241	0.028

ENSG00000186577	C6orf1	0.215	3.365	2.243	0.028
ENSG00000186642	PDE2A	0.319	6.893	3.567	0.001
ENSG00000186665	C17orf58	0.258	2.837	3.021	0.003
ENSG00000186716	BCR	0.225	6.446	2.369	0.020
ENSG00000186732	MPPED1	0.211	6.200	2.327	0.022
ENSG00000186854	TRABD2A	0.249	3.713	2.001	0.049
ENSG00000187079	TEAD1	-0.211	5.872	-2.420	0.018
ENSG00000187122	SLIT1	0.227	6.422	2.113	0.038
ENSG00000187258	NPSR1	-0.929	-1.844	-2.657	0.009
ENSG00000187260	WDR86	0.414	1.194	2.158	0.034
ENSG00000187398	LUZP2	0.320	5.371	2.316	0.023
ENSG00000187479	C11orf96	0.695	-0.295	2.408	0.018
ENSG00000187566	NHLRC1	0.286	1.281	2.096	0.039
ENSG00000187672	ERC2	0.195	8.427	2.644	0.010
ENSG00000187699	C2orf88	-0.267	4.151	-2.123	0.037
ENSG00000187730	GABRD	0.224	4.445	2.001	0.049
ENSG00000187778	MCRS1	0.223	4.136	2.525	0.014
ENSG00000187848	P2RX2	1.510	-2.252	2.823	0.006
ENSG00000187954	CYHR1	0.172	4.188	2.223	0.029
ENSG00000188051	TMEM221	0.382	0.550	2.330	0.022
ENSG00000188070	C11orf95	0.246	4.899	2.771	0.007
ENSG00000188133	TMEM215	0.679	0.518	2.403	0.018
ENSG00000188186	LAMTOR4	0.301	3.687	2.258	0.027
ENSG00000188191	PRKAR1B	0.246	6.876	2.644	0.010
ENSG00000188290	HES4	0.554	0.696	2.494	0.015
ENSG00000188612	SUMO2	0.300	5.333	2.366	0.020
ENSG00000188626	GOLGA8M	-0.348	0.290	-2.118	0.037
ENSG00000188690	UROS	0.194	5.618	2.157	0.034
ENSG00000188895	MSL1	0.121	6.815	2.108	0.038
ENSG00000188976	NOC2L	0.215	4.467	2.531	0.013
ENSG00000189077	TMEM120A	0.337	2.132	2.391	0.019
ENSG00000189089	RIMKLBP1	-0.435	1.733	-2.084	0.040
ENSG00000189129	PLAC9	0.482	1.007	2.900	0.005
ENSG00000189159	HN1	0.261	4.399	2.513	0.014
ENSG00000189184	PCDH18	0.293	3.819	2.024	0.046
ENSG00000189238	LINC00943	0.506	1.507	2.153	0.034
ENSG00000189280	GJB5	0.398	-0.465	2.048	0.044
ENSG00000189306	RRP7A	0.255	4.404	2.405	0.018
ENSG00000196109	ZNF676	-0.525	1.349	-2.631	0.010
ENSG00000196123	KIAA0895L	0.162	4.957	2.173	0.033
ENSG00000196132	MYT1	0.248	3.485	2.151	0.034
ENSG00000196262	PPIA	0.222	6.781	2.043	0.044
ENSG00000196350	ZNF729	-0.780	-1.933	-2.214	0.030
ENSG00000196363	WDR5	0.163	4.043	2.607	0.011
ENSG00000196387	ZNF140	-0.123	3.813	-2.158	0.034
ENSG00000196544	BORCS6	0.307	0.726	2.037	0.045
ENSG00000196549	MME	0.277	1.498	2.143	0.035

ENSG00000196588	MKL1	0.187	4.798	2.144	0.035
ENSG00000196700	ZNF512B	0.180	4.571	2.033	0.045
ENSG00000196811	CHRNG	0.430	-0.472	2.048	0.044
ENSG00000196865	NHLRC2	-0.187	6.292	-2.803	0.006
ENSG00000196872	KIAA1211L	0.200	6.145	2.200	0.031
ENSG00000196935	SRGAP1	-0.195	4.786	-2.183	0.032
ENSG00000196968	FUT11	0.193	4.104	2.664	0.009
ENSG00000196975	ANXA4	-0.253	3.670	-2.342	0.022
ENSG00000197122	SRC	0.204	4.059	2.422	0.018
ENSG00000197134	ZNF257	-0.539	1.662	-2.826	0.006
ENSG00000197191	CYSRT1	0.674	-1.511	2.584	0.012
ENSG00000197261	C6orf141	0.971	0.060	3.311	0.001
ENSG00000197360	ZNF98	-0.367	2.234	-2.129	0.036
ENSG00000197467	COL13A1	0.471	-0.001	2.051	0.043
ENSG00000197562	RAB40C	0.302	4.479	2.870	0.005
ENSG00000197586	ENTPD6	0.178	5.552	2.137	0.036
ENSG00000197705	KLHL14	0.421	1.930	2.120	0.037
ENSG00000197728	RPS26	0.479	2.196	2.194	0.031
ENSG00000197744	PTMAP2	-0.620	3.387	-2.034	0.045
ENSG00000197785	ATAD3A	0.189	2.672	2.344	0.022
ENSG00000197822	OCLN	-0.392	2.384	-2.161	0.034
ENSG00000197859	ADAMTSL2	0.243	2.547	2.128	0.036
ENSG00000198133	TMEM229B	0.204	3.771	2.371	0.020
ENSG00000198171	DDRGK1	0.179	5.275	2.290	0.025
ENSG00000198208	RPS6KL1	0.165	4.919	2.207	0.030
ENSG00000198218	QRICH1	0.117	5.894	2.284	0.025
ENSG00000198258	UBL5	0.397	3.715	2.319	0.023
ENSG00000198315	ZKSCAN8	-0.151	5.948	-2.603	0.011
ENSG00000198417	MT1F	-0.362	2.323	-2.261	0.026
ENSG00000198520	C1orf228	0.345	1.547	2.452	0.016
ENSG00000198542	ITGBL1	0.639	-0.739	2.005	0.048
ENSG00000198546	ZNF511	0.229	1.410	2.302	0.024
ENSG00000198625	MDM4	-0.112	6.900	-2.132	0.036
ENSG00000198722	UNC13B	0.117	6.981	2.030	0.046
ENSG00000198799	LRIG2	-0.107	5.712	-2.589	0.011
ENSG00000198841	KTI12	0.256	0.710	2.157	0.034
ENSG00000198873	GRK5	0.219	3.689	2.227	0.029
ENSG00000198912	C1orf174	0.148	3.323	2.188	0.032
ENSG00000198915	RASGEF1A	0.253	5.228	3.123	0.002
ENSG00000198944	SOWAHA	0.380	5.614	2.778	0.007
ENSG00000199217	RNU6-1123P	-0.835	-2.051	-2.084	0.040
ENSG00000199466	Y_RNA	-0.764	-1.898	-2.638	0.010
ENSG00000199914	SNORD114-16	-0.613	-2.217	-2.136	0.036
ENSG00000200086	RNU6-433P	-0.500	-0.369	-2.793	0.006
ENSG00000201390	RNU6-1141P	-0.627	-2.393	-2.259	0.027
ENSG00000201533	RN7SKP237	0.663	-2.186	2.145	0.035
ENSG00000202172	RNU6-1327P	-1.031	-2.129	-3.040	0.003

ENSG00000202205	RNU6-1034P	-0.541	-2.324	-2.143	0.035
ENSG00000202261	SNORD115-44	-0.660	-1.153	-2.558	0.012
ENSG00000203499	FAM83H-AS1	0.564	-0.227	2.740	0.008
ENSG00000203685	STUM	0.390	6.804	2.509	0.014
ENSG00000203772	SPRN	0.282	3.862	2.022	0.046
ENSG00000203799	CCDC162P	0.336	2.223	2.964	0.004
ENSG00000203805	PLPP4	0.433	1.376	3.262	0.002
ENSG00000203814	HIST2H2BF	-0.290	2.991	-2.056	0.043
ENSG00000203865	ATP1A1-AS1	-0.284	1.851	-2.316	0.023
ENSG00000203867	RBM20	-0.292	2.087	-2.555	0.012
ENSG00000204099	NEU4	0.494	0.100	2.306	0.024
ENSG00000204175	GPRIN2	0.757	-1.569	2.250	0.027
ENSG00000204227	RING1	0.198	3.827	2.667	0.009
ENSG00000204366	ZBTB12	-0.349	0.417	-2.451	0.016
ENSG00000204389	HSPA1A	-0.605	5.422	-2.036	0.045
ENSG00000204568	MRPS18B	0.264	3.633	2.629	0.010
ENSG00000204623	ZNRD1ASP	-0.200	4.677	-2.546	0.013
ENSG00000204628	RACK1	0.205	6.758	2.520	0.014
ENSG00000204792	LINC01291	-2.067	-0.801	-2.059	0.043
ENSG00000204843	DCTN1	0.176	8.589	2.774	0.007
ENSG00000205106	DKFZp779M0652	0.745	-2.106	2.045	0.044
ENSG00000205363	C15orf59	0.229	5.568	2.279	0.025
ENSG00000205534	SMG1P2	-0.559	4.074	-2.212	0.030
ENSG00000205571	SMN2	-0.906	2.775	-2.492	0.015
ENSG00000205629	LCMT1	0.164	4.502	2.234	0.028
ENSG00000205790	DPP9-AS1	-0.860	-2.070	-2.942	0.004
ENSG00000206034	DEFB109P1B	-0.503	-0.235	-1.995	0.049
ENSG00000206052	DOK6	0.309	7.734	2.169	0.033
ENSG00000206149	HERC2P9	-0.291	5.682	-2.190	0.031
ENSG00000206344	HCG27	0.357	0.962	2.402	0.019
ENSG00000206585	RNVU1-7	0.785	8.445	2.138	0.036
ENSG00000206728	Y_RNA	-0.945	-1.561	-2.979	0.004
ENSG00000206847	Y_RNA	-0.506	-1.070	-2.216	0.029
ENSG00000206985	RNU6-198P	-0.632	-1.972	-2.140	0.035
ENSG00000207034	Y_RNA	-0.638	-1.699	-2.596	0.011
ENSG00000207428	RNU6-95P	0.683	-1.276	2.260	0.027
ENSG00000212124	TAS2R19	-0.259	1.585	-2.026	0.046
ENSG00000212125	TAS2R15P	-0.335	1.921	-2.698	0.008
ENSG00000212205	Y_RNA	-0.516	-2.155	-2.096	0.039
ENSG00000212766	EWSAT1	0.555	-0.318	2.880	0.005
ENSG00000212978	AC016747.3	0.284	2.457	2.647	0.010
ENSG00000213005	PTTG3P	-0.815	-0.939	-2.792	0.007
ENSG00000213046	EEF1A1P32	-0.481	-0.152	-2.124	0.037
ENSG00000213339	QTRT1	0.213	3.616	2.043	0.044
ENSG00000213376	GAPDHP71	-0.479	-0.087	-2.271	0.026
ENSG00000213484	EIF4A1P8	-0.521	-0.432	-2.274	0.026
ENSG00000213542	RP11-467H10.1	-0.379	-0.135	-2.025	0.046

ENSG00000213587	RP11-646D13.1	-0.798	-0.723	-3.777	0.000
ENSG00000213690	RP11-513D4.1	-0.680	-0.980	-2.364	0.020
ENSG00000213889	PPM1N	0.514	-0.794	2.273	0.026
ENSG00000213891	RPL3P6	-0.395	0.963	-2.808	0.006
ENSG00000213988	ZNF90	-0.340	0.077	-2.110	0.038
ENSG00000213995	NAXD	0.182	5.081	3.281	0.002
ENSG00000214029	ZNF891	-0.194	5.328	-2.549	0.013
ENSG00000214188	ST7-OT4	-0.429	0.151	-2.116	0.037
ENSG00000214211	CTAGE14P	-0.309	1.581	-2.449	0.016
ENSG00000214248	CTD-3193O13.12	0.924	-2.496	2.676	0.009
ENSG00000214357	NEURL1B	0.443	4.245	3.267	0.002
ENSG00000214456	PLIN5	-0.437	1.197	-2.391	0.019
ENSG00000214530	STARD10	0.258	2.676	2.550	0.013
ENSG00000214534	ZNF705E	-0.601	-1.501	-2.517	0.014
ENSG00000214549	SDHCP2	-0.417	-0.180	-2.216	0.029
ENSG00000214654	RP11-27I1.4	-0.262	1.400	-2.237	0.028
ENSG00000214671	RPL6P12	-1.202	-1.973	-3.485	0.001
ENSG00000214719	AC005562.1	0.342	-0.620	2.051	0.043
ENSG00000214976	VDAC2P1	-0.481	0.040	-2.506	0.014
ENSG00000215018	COL28A1	-0.273	2.948	-2.060	0.043
ENSG00000215021	PHB2	0.170	5.088	2.067	0.042
ENSG00000215158	RP11-1023L17.1	-0.286	3.915	-2.525	0.014
ENSG00000215184	RP11-8L18.2	-0.575	-1.434	-2.072	0.041
ENSG00000215190	LINC00680	0.209	3.376	2.143	0.035
ENSG00000215223	CYP51A1P3	-0.475	-0.052	-2.643	0.010
ENSG00000215277	RNF212B	-0.424	-0.495	-2.053	0.043
ENSG00000215302	CTD-3092A11.1	-0.431	2.281	-2.296	0.024
ENSG00000215305	VPS16	0.175	3.899	2.197	0.031
ENSG00000215380	AC090625.1	-0.639	2.328	-2.413	0.018
ENSG00000215712	TMEM242	0.222	5.117	2.159	0.034
ENSG00000216307	RP3-400B16.2	-0.567	-1.585	-2.402	0.019
ENSG00000216977	RPL21P65	-0.513	-1.169	-2.127	0.036
ENSG00000217165	ANKRD18EP	-0.258	2.427	-2.378	0.020
ENSG00000217414	RP11-528A10.2	-0.538	-0.075	-2.351	0.021
ENSG00000217801	RP11-465B22.3	0.294	0.922	2.217	0.029
ENSG00000218027	RP11-157J24.1	-0.509	0.063	-2.167	0.033
ENSG00000218186	KRT8P43	-0.536	-0.306	-2.106	0.038
ENSG00000218336	TENM3	0.236	7.201	2.127	0.036
ENSG00000218586	RP4-791C19.1	-0.768	1.716	-2.099	0.039
ENSG00000218857	RP1-209B5.2	-0.477	-0.187	-2.023	0.046
ENSG00000218996	RP1-99E18.2	-0.441	0.603	-2.364	0.020
ENSG00000219547	RPL17P25	-0.475	-0.636	-2.150	0.035
ENSG00000219941	KRT18P50	-0.603	-1.352	-1.999	0.049
ENSG00000220378	KRT8P42	-0.612	-0.558	-2.350	0.021
ENSG00000220506	RP3-415N12.1	-0.383	1.155	-2.439	0.017
ENSG00000220695	RP1-121G13.3	-0.720	1.559	-1.994	0.049
ENSG00000221817	PPP3CB-AS1	-0.328	2.473	-2.182	0.032

ENSG00000221890	NPTXR	0.347	7.591	2.704	0.008
ENSG00000221949	LINC01465	-0.413	-0.581	-2.096	0.039
ENSG00000221983	UBA52	0.174	6.256	2.117	0.037
ENSG00000222543	RN7SKP220	-0.679	-2.295	-2.009	0.048
ENSG00000222883	Y_RNA	-0.722	-1.654	-2.346	0.021
ENSG00000222969	RN7SKP8	0.580	-1.645	1.990	0.050
ENSG00000223152	RNU4-88P	-0.733	-2.504	-2.083	0.040
ENSG00000223324	RN7SKP273	-0.826	-2.303	-2.490	0.015
ENSG00000223374	AC005104.3	-0.562	-1.523	-2.111	0.038
ENSG00000223427	AC016716.1	-0.302	2.421	-2.308	0.024
ENSG00000223429	RP4-800M22.2	-0.422	0.277	-2.056	0.043
ENSG00000223430	AC011243.1	-0.963	-2.059	-2.542	0.013
ENSG00000223573	TINCR	0.400	2.034	2.348	0.021
ENSG00000223653	RP11-131L23.1	-0.408	2.046	-2.459	0.016
ENSG00000223768	LINC00205	0.390	5.566	2.720	0.008
ENSG00000223930	RP11-33A14.1	0.661	1.828	2.877	0.005
ENSG00000223949	ROR1-AS1	-0.514	-1.136	-2.119	0.037
ENSG00000223965	ZNF587P1	-0.737	-1.356	-2.062	0.042
ENSG00000223984	HNRNPRP1	-0.413	2.966	-2.051	0.043
ENSG00000224014	RP11-488L18.6	0.545	-1.113	2.331	0.022
ENSG00000224093	RP5-1033H22.2	-0.461	-0.028	-2.518	0.014
ENSG00000224138	AC000123.4	-0.327	0.628	-2.077	0.041
ENSG00000224237	MINOS1P3	-0.522	-0.334	-2.381	0.020
ENSG00000224238	WARS2-IT1	-0.497	-0.749	-2.085	0.040
ENSG00000224363	RP11-289H16.1	-0.565	0.002	-2.000	0.049
ENSG00000224404	LL22NC03-13G6.2	0.456	2.240	2.501	0.014
ENSG00000224412	RP11-351O1.3	-0.482	-0.404	-2.075	0.041
ENSG00000224442	AC017035.2	-0.471	-0.429	-2.267	0.026
ENSG00000224468	RP11-181K3.4	-0.606	-1.376	-1.990	0.050
ENSG00000224525	RP11-561I11.3	-0.799	-0.959	-2.943	0.004
ENSG00000224543	SNRPGP15	-0.401	0.107	-2.464	0.016
ENSG00000224700	RP11-697E14.2	-0.404	0.411	-2.341	0.022
ENSG00000224707	E2F3-IT1	-0.410	-0.353	-2.087	0.040
ENSG00000224731	AC016716.2	-0.313	2.080	-2.250	0.027
ENSG00000224733	RP1-86D1.5	-0.444	0.388	-2.035	0.045
ENSG00000224746	AC015987.1	-0.595	-1.821	-2.315	0.023
ENSG00000224892	RPS4XP16	-0.435	1.952	-2.127	0.036
ENSG00000224965	LINC02586	0.657	-2.395	2.045	0.044
ENSG00000225138	CTD-2228K2.7	0.389	2.349	2.092	0.039
ENSG00000225215	SMARCE1P1	-0.255	1.346	-2.264	0.026
ENSG00000225282	AP000350.6	-0.677	1.758	-2.322	0.023
ENSG00000225303	RP11-512N4.2	-0.537	-1.070	-2.178	0.032
ENSG00000225338	RPL23AP18	-0.449	-0.837	-2.025	0.046
ENSG00000225505	RP11-330C7.3	-0.365	0.348	-2.197	0.031
ENSG00000225539	LINC01821	0.398	1.487	2.159	0.034
ENSG00000225663	MCRIP1	0.272	3.649	2.410	0.018
ENSG00000225767	RP5-850O15.3	-0.519	0.781	-3.087	0.003

ENSG00000225826	LINC00626	-0.657	-1.571	-2.039	0.045
ENSG00000225900	HSPE1P13	-0.467	0.087	-2.060	0.043
ENSG00000225938	RP4-575N6.4	-0.620	-1.477	-2.534	0.013
ENSG00000226038	PPIAP21	0.461	-0.529	2.324	0.023
ENSG00000226193	RP3-398G3.5	-0.495	-1.863	-2.027	0.046
ENSG00000226259	GTF2H2B	-0.901	1.427	-2.483	0.015
ENSG00000226268	RP11-61N20.3	-0.679	1.424	-2.505	0.014
ENSG00000226330	RP11-739N20.2	-0.606	-0.981	-3.068	0.003
ENSG00000226438	RP11-420K8.1	-0.613	-0.656	-3.027	0.003
ENSG00000226440	RP11-506B6.6	-0.509	-0.732	-2.239	0.028
ENSG00000226519	LINC00390	0.509	0.294	2.273	0.026
ENSG00000226761	TAS2R46	-0.411	0.368	-2.334	0.022
ENSG00000226939	AC062021.1	0.527	-1.456	2.115	0.037
ENSG00000226994	AC012593.1	-0.636	0.709	-2.733	0.008
ENSG00000227018	IL6STP1	-0.914	1.720	-2.400	0.019
ENSG00000227034	RP11-234N17.1	-0.413	0.011	-2.223	0.029
ENSG00000227051	C14orf132	0.234	7.977	2.716	0.008
ENSG00000227057	WDR46	0.182	3.990	2.246	0.027
ENSG00000227107	AC096574.5	0.303	0.072	2.010	0.048
ENSG00000227170	AF178030.2	-0.488	0.410	-2.442	0.017
ENSG00000227176	AC092641.2	-0.352	0.202	-2.272	0.026
ENSG00000227215	RP11-445L13__B.3	-0.476	-1.270	-2.074	0.041
ENSG00000227230	RP11-261C10.5	-0.484	-0.898	-2.074	0.041
ENSG00000227288	RP5-837I24.1	-0.363	0.633	-2.208	0.030
ENSG00000227379	PPIAP2	-0.646	-1.167	-2.370	0.020
ENSG00000227523	RPS20P15	-0.415	-0.764	-1.999	0.049
ENSG00000227646	STEAP2-AS1	-0.576	-1.581	-2.595	0.011
ENSG00000227692	MED28P3	-0.599	-0.902	-2.462	0.016
ENSG00000227695	DNMBP-AS1	-0.642	-1.775	-2.047	0.044
ENSG00000227725	GCOM2	-0.572	1.616	-2.242	0.028
ENSG00000227745	AC098826.5	0.398	2.970	2.302	0.024
ENSG00000227911	LINC02344	-0.466	-1.321	-2.058	0.043
ENSG00000227929	SRGAP3-AS3	-0.356	0.839	-2.011	0.048
ENSG00000227948	AC003989.4	-0.495	-0.083	-2.484	0.015
ENSG00000228024	CYP1D1P	-0.547	-0.236	-2.110	0.038
ENSG00000228212	OFD1P17	-0.334	2.172	-2.025	0.046
ENSG00000228251	AC012442.6	0.567	-1.310	2.119	0.037
ENSG00000228351	AC018832.1	-0.408	-0.353	-2.048	0.044
ENSG00000228386	RP5-1125A11.4	-0.692	-1.448	-2.243	0.028
ENSG00000228510	RP11-339B21.8	-0.536	0.998	-2.599	0.011
ENSG00000228513	AC023271.1	-1.007	-1.849	-3.051	0.003
ENSG00000228599	RPL7P52	-0.402	0.459	-2.136	0.036
ENSG00000228604	RP5-872K7.7	-0.444	-0.086	-3.008	0.003
ENSG00000228634	RP4-534N18.2	-0.432	-0.778	-2.165	0.033
ENSG00000228696	ARL17B	-0.565	2.127	-2.295	0.024
ENSG00000228809	RP4-705D16.3	-0.934	-2.656	-2.607	0.011
ENSG00000228868	MYLKP1	-0.789	-1.602	-3.223	0.002

ENSG00000228914	OR1H1P	0.656	-0.875	2.443	0.017
ENSG00000228915	OR7E128P	-0.415	0.102	-2.071	0.041
ENSG00000228995	MTND1P9	-1.039	-1.492	-3.415	0.001
ENSG00000229287	FABP5P4	-0.859	-2.035	-2.845	0.006
ENSG00000229385	AC010975.1	-0.354	0.773	-2.162	0.034
ENSG00000229456	RLIMP1	-0.411	0.916	-2.232	0.028
ENSG00000229521	MYCBP2-AS2	-0.357	0.297	-2.151	0.034
ENSG00000229587	RP11-197P3.5	-0.431	-0.292	-2.287	0.025
ENSG00000229591	RP5-981O7.2	-0.344	0.038	-2.008	0.048
ENSG00000229598	PRDX3P1	-0.436	1.338	-2.286	0.025
ENSG00000229694	RP11-305L7.6	-0.649	-1.049	-2.820	0.006
ENSG00000229839	AC018462.2	-0.410	0.774	-2.104	0.038
ENSG00000229852	RP11-398K22.12	0.162	3.022	2.043	0.044
ENSG00000229867	STEAP3-AS1	0.517	-0.251	2.884	0.005
ENSG00000229981	LINC01435	0.461	-0.126	2.389	0.019
ENSG00000230022	FNTAP2	-0.333	0.371	-2.069	0.042
ENSG00000230051	CTA-929C8.8	0.591	0.111	2.038	0.045
ENSG00000230077	MTAPP2	-0.392	-0.772	-1.994	0.050
ENSG00000230237	AC010975.2	-0.469	-0.052	-2.229	0.029
ENSG00000230257	NFE4	-0.711	-1.777	-2.273	0.026
ENSG00000230280	HNRNPA1P59	-0.424	-0.081	-2.783	0.007
ENSG00000230287	RP11-305E17.4	-0.545	-1.103	-2.288	0.025
ENSG00000230300	STARD13-IT1	-0.327	0.911	-2.281	0.025
ENSG00000230303	RP11-213G2.2	-0.317	0.348	-2.122	0.037
ENSG00000230373	GOLGA6L5P	-0.466	1.048	-2.208	0.030
ENSG00000230417	LINC00856	-0.234	2.907	-2.053	0.043
ENSG00000230482	ATP5G2P3	-0.374	-0.478	-2.069	0.042
ENSG00000230551	CTB-89H12.4	-0.211	4.919	-2.288	0.025
ENSG00000230630	DNM3OS	-0.260	2.767	-2.229	0.029
ENSG00000230737	AC106870.1	-0.701	-1.660	-2.193	0.031
ENSG00000230806	SRGAP2-AS1	-0.486	0.364	-2.115	0.037
ENSG00000230820	AC000362.1	-0.776	-2.355	-2.061	0.042
ENSG00000230894	RP11-67K19.3	-0.338	2.467	-2.393	0.019
ENSG00000231063	AC006994.1	-0.564	-0.253	-2.330	0.022
ENSG00000231132	RP11-40C11.2	-0.524	0.153	-2.377	0.020
ENSG00000231181	RP13-15M17.1	-0.632	-1.298	-2.122	0.037
ENSG00000231195	IFNA11P	-1.068	-1.501	-3.246	0.002
ENSG00000231310	TBL1XR1-AS1	-0.514	-0.212	-2.876	0.005
ENSG00000231329	RP1-225E12.2	-0.395	1.112	-2.970	0.004
ENSG00000231378	NDUFAF4P4	-0.488	-0.104	-2.082	0.041
ENSG00000231395	AC124914.3	-0.501	-0.582	-2.666	0.009
ENSG00000231401	AC023481.1	-0.574	-0.054	-2.266	0.026
ENSG00000231409	RP11-83J16.1	-0.640	1.411	-2.355	0.021
ENSG00000231466	CTA-246H3.11	-0.314	1.026	-2.277	0.025
ENSG00000231527	RP11-374M1.2	0.489	-0.211	2.323	0.023
ENSG00000231564	EIF4A1P11	-0.496	-1.867	-1.999	0.049
ENSG00000231616	RP11-575L7.4	-0.362	0.654	-2.389	0.019

ENSG00000231721	LINC-PINT	-0.259	6.023	-2.373	0.020
ENSG00000231769	RP1-8B1.4	0.338	0.243	2.492	0.015
ENSG00000231799	RP13-93L13.2	-0.363	0.873	-2.164	0.033
ENSG00000231858	AC067945.4	-0.368	-0.081	-2.156	0.034
ENSG00000231940	RPS7P3	0.522	-1.116	2.261	0.026
ENSG00000232085	RP11-261C10.4	-0.457	-1.232	-2.091	0.040
ENSG00000232176	RP11-146N23.1	-0.380	2.351	-2.229	0.029
ENSG00000232395	RP3-399L15.1	-0.644	-2.164	-2.160	0.034
ENSG00000232451	AC016768.1	0.751	-2.739	2.022	0.046
ENSG00000232454	RP11-3J10.7	-0.473	1.592	-1.991	0.050
ENSG00000232474	NCKAP5-IT1	-0.805	-1.088	-2.590	0.011
ENSG00000232578	CTC-303L1.1	-0.277	1.687	-2.025	0.046
ENSG00000232757	ETF1P1	-0.402	-0.486	-2.231	0.028
ENSG00000232860	SMG7-AS1	0.465	-0.609	2.517	0.014
ENSG00000232912	RP5-1115A15.1	-0.340	0.403	-2.064	0.042
ENSG00000232995	RGS5	-0.368	1.274	-2.650	0.010
ENSG00000233008	LINC01725	-0.441	1.545	-2.318	0.023
ENSG00000233009	NALCN-AS1	-0.612	-0.040	-2.772	0.007
ENSG00000233016	SNHG7	0.398	2.518	2.840	0.006
ENSG00000233123	LINC01007	0.484	3.960	2.351	0.021
ENSG00000233337	UBE2FP3	-0.413	0.068	-2.215	0.030
ENSG00000233540	DNM3-IT1	-0.435	0.544	-2.517	0.014
ENSG00000233806	LINC01237	-0.420	-0.008	-2.316	0.023
ENSG00000233829	AC017078.1	-0.783	-1.641	-2.590	0.011
ENSG00000233894	RP4-650F12.2	0.412	-0.751	2.023	0.046
ENSG00000233998	SETP5	-0.786	-2.065	-2.369	0.020
ENSG00000234031	RPS3AP44	-0.396	0.772	-2.310	0.023
ENSG00000234040	RP11-254B13.4	-0.537	-1.407	-2.089	0.040
ENSG00000234144	COX6CP13	-0.590	-0.690	-2.850	0.006
ENSG00000234197	ETV5-AS1	-0.644	0.637	-2.739	0.008
ENSG00000234268	AP000936.1	-0.457	0.564	-2.845	0.006
ENSG00000234456	MAGI2-AS3	-0.227	6.706	-2.721	0.008
ENSG00000234562	TPMTP2	-0.646	-1.470	-2.276	0.025
ENSG00000234608	MAPKAPK5-AS1	0.262	3.572	2.394	0.019
ENSG00000234616	JRK	0.140	4.506	2.055	0.043
ENSG00000234817	RP3-400B16.1	-0.520	-0.867	-2.190	0.031
ENSG00000234841	RP11-119H12.4	-0.245	3.003	-2.274	0.026
ENSG00000234891	RP1-288L1.5	0.472	-1.089	2.089	0.040
ENSG00000234902	AC007879.2	0.869	-2.002	2.507	0.014
ENSG00000234937	RP11-101O6.2	-0.643	-1.074	-2.215	0.030
ENSG00000234943	AC092839.4	-0.508	0.530	-2.484	0.015
ENSG00000234953	RP5-837I24.2	-0.547	-0.781	-2.565	0.012
ENSG00000234982	RP11-280O24.3	-0.687	-1.294	-2.748	0.007
ENSG00000235026	DPP10-AS1	0.252	3.416	3.033	0.003
ENSG00000235092	ID2-AS1	-0.308	1.596	-2.240	0.028
ENSG00000235141	COX6CP17	-0.485	-1.326	-2.141	0.035
ENSG00000235159	RP6-109B7.4	-0.378	0.804	-2.390	0.019

ENSG00000235251	RP11-384B12.2	-0.651	1.011	-2.095	0.039
ENSG00000235597	LINC01102	0.269	3.122	2.414	0.018
ENSG00000235677	NPM1P26	-0.306	1.185	-2.484	0.015
ENSG00000235717	DPP10-AS2	-0.380	1.288	-2.054	0.043
ENSG00000235786	ZNRF3-IT1	-0.457	0.536	-2.089	0.040
ENSG00000236340	AC000099.1	-0.533	-0.539	-2.557	0.012
ENSG00000236349	SUCLG2P2	-0.655	0.917	-2.105	0.038
ENSG00000236397	DDX11L2	-0.819	-1.092	-2.653	0.010
ENSG00000236478	AC012513.4	-0.450	0.218	-2.144	0.035
ENSG00000236508	ATP13A5-AS1	0.575	-2.418	2.181	0.032
ENSG00000236581	STARD13-AS	-0.361	2.985	-2.761	0.007
ENSG00000236816	ANKRD20A7P	-0.586	3.041	-2.530	0.013
ENSG00000236818	ARL5AP2	-0.655	-0.997	-2.605	0.011
ENSG00000236948	RP11-154H17.1	0.923	-0.793	3.435	0.001
ENSG00000236973	GAPDHP51	-0.791	-1.817	-2.937	0.004
ENSG00000236991	EDRF1-AS1	-0.464	0.099	-2.537	0.013
ENSG00000237001	WASF3-AS1	-0.455	0.771	-2.548	0.013
ENSG00000237170	RPS7P15	-0.623	-0.862	-3.134	0.002
ENSG00000237190	CDKN2AIPNL	0.301	4.408	2.564	0.012
ENSG00000237234	RP1-142L7.5	-0.412	-0.502	-2.084	0.040
ENSG00000237250	RP11-193H5.1	0.850	-0.733	2.921	0.005
ENSG00000237263	MAPK6PS3	-0.432	1.523	-2.239	0.028
ENSG00000237296	SMG1P1	-0.549	3.862	-2.517	0.014
ENSG00000237317	RP4-809F4.1	-0.619	-1.713	-2.135	0.036
ENSG00000237321	RP11-374A22.1	-0.402	2.115	-2.057	0.043
ENSG00000237517	DGCR5	0.253	5.578	2.402	0.019
ENSG00000237522	NONOP2	-0.333	1.162	-2.626	0.010
ENSG00000237686	RP5-1120P11.1	0.413	0.517	2.182	0.032
ENSG00000237928	NFIA-AS2	-0.458	-0.673	-2.034	0.045
ENSG00000238227	C9orf69	0.203	3.805	2.229	0.029
ENSG00000238230	LINC00391	-0.431	-0.097	-2.001	0.049
ENSG00000238243	OR2W3	0.712	-0.519	2.232	0.028
ENSG00000238246	RP11-575A19.2	-0.520	-0.100	-2.421	0.018
ENSG00000238386	RNU7-48P	-0.475	-2.011	-2.002	0.049
ENSG00000239300	RP11-400L8.2	-0.447	-0.072	-2.236	0.028
ENSG00000239388	ASB14	-0.211	1.841	-2.053	0.043
ENSG00000239524	RPL32P34	0.983	-2.290	2.366	0.020
ENSG00000239602	RP11-449H3.1	-0.683	-2.014	-2.647	0.010
ENSG00000239627	RPL12P20	-0.444	1.076	-2.286	0.025
ENSG00000239671	CTD-3193K9.1	-0.596	-1.225	-2.389	0.019
ENSG00000239828	RP11-446H18.5	-0.331	2.680	-2.180	0.032
ENSG00000239835	RP11-767L7.1	-0.332	0.433	-2.156	0.034
ENSG00000239877	IGSF11-AS1	-0.403	1.395	-2.655	0.010
ENSG00000240048	DDX50P2	-0.509	1.676	-2.420	0.018
ENSG00000240057	RP11-572M11.4	-0.404	-0.850	-2.040	0.045
ENSG00000240074	RPL9P30	-0.764	-0.788	-2.972	0.004
ENSG00000240265	RP11-62G11.1	-0.778	-1.566	-2.245	0.027

ENSG00000240328	RP11-841C19.1	-0.734	-1.769	-2.210	0.030
ENSG00000240520	UOX	0.355	-0.370	2.081	0.041
ENSG00000240616	RPS6P25	-0.469	1.857	-2.550	0.013
ENSG00000240720	LRRD1	-0.473	-0.671	-2.410	0.018
ENSG00000240729	CTD-2301A4.3	-0.444	-0.349	-2.181	0.032
ENSG00000240869	RN7SL128P	0.614	6.389	2.401	0.019
ENSG00000240972	MIF	0.667	-1.946	2.115	0.037
ENSG00000241158	ADAMTS9-AS1	0.667	1.770	2.865	0.005
ENSG00000241217	RN7SL809P	-0.755	-1.743	-2.060	0.043
ENSG00000241416	KRT8P13	-0.693	-0.113	-2.227	0.029
ENSG00000241449	RP11-545G3.1	-0.495	-0.978	-2.161	0.034
ENSG00000241468	ATP5J2	0.274	4.016	2.414	0.018
ENSG00000241592	RP11-372E1.7	0.488	-0.271	2.572	0.012
ENSG00000241625	RN7SL18P	0.953	-3.322	2.463	0.016
ENSG00000241878	PISD	0.190	4.854	2.850	0.006
ENSG00000241990	PRR34-AS1	-0.260	1.428	-2.045	0.044
ENSG00000242121	RN7SL267P	-0.765	-2.322	-2.417	0.018
ENSG00000242163	RP11-299L17.1	-0.482	0.231	-2.258	0.027
ENSG00000242173	ARHGDIG	0.259	4.489	2.027	0.046
ENSG00000242175	RN7SL127P	-0.585	-1.304	-2.322	0.023
ENSG00000242290	RP11-197K3.1	-0.380	0.878	-2.312	0.023
ENSG00000242338	BMS1P4	-0.322	0.457	-2.349	0.021
ENSG00000242375	RP11-498P14.3	-0.363	0.513	-2.049	0.044
ENSG00000242612	DECR2	0.262	1.093	2.090	0.040
ENSG00000242816	RP11-768G7.3	-0.365	0.510	-2.048	0.044
ENSG00000243004	AC005062.2	0.386	0.312	2.056	0.043
ENSG00000243155	RP11-46A10.5	-0.529	-1.113	-2.507	0.014
ENSG00000243175	RPSAP36	-0.482	1.111	-2.606	0.011
ENSG00000243402	RP11-473O4.1	-0.476	0.487	-2.776	0.007
ENSG00000243663	RPS4XP14	-0.514	0.777	-2.523	0.014
ENSG00000243710	CFAP57	0.464	1.321	3.885	0.000
ENSG00000243849	CFAP44-AS1	-0.656	-1.833	-2.147	0.035
ENSG00000244038	DDOST	0.178	4.293	2.095	0.039
ENSG00000244187	TMEM141	0.353	1.618	2.893	0.005
ENSG00000244215	LINC02016	0.853	-1.904	2.876	0.005
ENSG00000244668	SNRPCP3	-1.134	-2.465	-3.257	0.002
ENSG00000244682	FCGR2C	-0.823	-0.014	-2.255	0.027
ENSG00000245105	A2M-AS1	-0.248	1.673	-2.206	0.030
ENSG00000245498	RP11-677M14.7	0.255	2.175	2.353	0.021
ENSG00000246250	RP11-613D13.5	-0.544	1.210	-3.313	0.001
ENSG00000246898	LINC00920	-0.735	-1.136	-2.146	0.035
ENSG00000247317	RP11-273G15.2	0.249	2.958	2.434	0.017
ENSG00000247595	SPTY2D1-AS1	-0.429	-0.166	-2.545	0.013
ENSG00000247596	TWF2	0.280	1.843	2.126	0.036
ENSG00000248008	NRAV	0.206	3.681	3.017	0.003
ENSG00000248200	RP11-115A14.1	-0.709	-1.650	-2.005	0.048
ENSG00000248216	KCTD9P5	-0.630	-0.822	-2.286	0.025

ENSG00000248397	LINC00498	-0.763	-1.756	-2.038	0.045
ENSG00000248429	RP11-597D13.9	-0.685	2.587	-3.946	0.000
ENSG00000248466	RP11-640B6.1	-0.971	-2.542	-2.271	0.026
ENSG00000248485	PCP4L1	0.345	4.267	2.343	0.022
ENSG00000248673	LINC01331	0.638	-0.038	2.852	0.005
ENSG00000248714	RP11-1079K10.3	-0.427	-0.879	-2.011	0.048
ENSG00000248725	RP11-218C23.1	-0.566	-0.143	-2.352	0.021
ENSG00000248738	RP11-87E22.2	0.490	2.337	2.680	0.009
ENSG00000248773	RP11-231L11.3	-0.460	-0.681	-2.100	0.039
ENSG00000248810	LINC02432	-1.004	-2.469	-2.164	0.033
ENSG00000248909	HMGB1P21	-0.773	-0.259	-3.531	0.001
ENSG00000248967	RP11-241J12.3	-0.680	-1.246	-3.289	0.001
ENSG00000249098	RP11-39E3.4	-0.457	-0.548	-2.191	0.031
ENSG00000249222	ATP5L2	-0.682	-0.457	-2.389	0.019
ENSG00000249278	RP11-688H10.1	-0.829	-2.716	-2.102	0.039
ENSG00000249304	RP11-26P13.2	-0.699	-1.234	-2.495	0.015
ENSG00000249381	LINC00500	-1.111	-2.029	-2.648	0.010
ENSG00000249436	CTD-2023N9.3	0.416	3.355	2.183	0.032
ENSG00000249471	ZNF324B	0.191	2.660	2.111	0.038
ENSG00000249485	RBBP4P1	-0.423	2.938	-2.286	0.025
ENSG00000249553	PPP2R2B-IT1	-0.339	1.144	-2.004	0.048
ENSG00000249577	CTD-2195M15.1	-0.635	0.184	-2.673	0.009
ENSG00000249684	RP11-423H2.3	0.627	0.231	2.921	0.005
ENSG00000249850	KRT18P31	-0.265	1.668	-2.052	0.043
ENSG00000249858	SNX5P1	-0.648	0.863	-2.059	0.043
ENSG00000249860	MTRNR2L5	-0.759	-2.419	-2.077	0.041
ENSG00000249896	LINC02495	0.199	3.919	2.063	0.042
ENSG00000249915	PDCD6	0.171	5.160	1.996	0.049
ENSG00000249916	CTD-2280E9.1	-1.011	-2.201	-2.996	0.004
ENSG00000249934	RP11-466G12.3	-0.762	-1.643	-2.501	0.014
ENSG00000250045	CBX3P3	-0.382	1.222	-2.593	0.011
ENSG00000250069	CTB-131B5.2	-0.775	-0.012	-3.056	0.003
ENSG00000250073	RP11-677M14.3	-0.323	-0.121	-2.149	0.035
ENSG00000250107	CACNA1G-AS1	0.585	-1.439	2.074	0.041
ENSG00000250189	RP11-362F19.2	-0.788	-2.439	-2.049	0.044
ENSG00000250329	KDELC1P1	-0.301	1.521	-2.226	0.029
ENSG00000250407	CTB-99A3.1	-0.494	-0.556	-2.184	0.032
ENSG00000250479	CHCHD10	0.213	4.223	2.020	0.047
ENSG00000250500	RP11-119H12.3	-0.681	-0.566	-2.626	0.010
ENSG00000250579	CTD-2297D10.2	1.075	-1.924	2.799	0.006
ENSG00000250584	LINC01511	0.504	0.725	2.504	0.014
ENSG00000250853	RNF138P1	-0.446	1.242	-3.399	0.001
ENSG00000250869	RP11-453N18.1	-0.388	-0.033	-2.088	0.040
ENSG00000250950	RP11-33B1.3	-0.488	0.076	-2.010	0.048
ENSG00000250980	RP11-529H22.1	-0.452	-0.747	-2.313	0.023
ENSG00000250986	LINC02600	0.447	1.495	3.492	0.001
ENSG00000251073	NUDT19P5	-0.789	-0.356	-3.586	0.001

ENSG00000251076	RP11-526F3.1	-0.750	-1.973	-2.089	0.040
ENSG00000251171	RP11-327O17.2	-0.509	-0.119	-2.907	0.005
ENSG00000251186	RP11-689P11.3	-0.571	-1.466	-2.234	0.028
ENSG00000251187	CTC-459M5.2	-0.677	-1.432	-2.947	0.004
ENSG00000251293	CTC-552D5.1	0.619	-0.404	3.068	0.003
ENSG00000251400	ALDH7A1P1	-0.466	3.447	-2.992	0.004
ENSG00000251579	RP11-39E3.5	-0.614	-1.263	-2.277	0.025
ENSG00000251596	HADHAP1	-0.314	3.465	-2.642	0.010
ENSG00000251609	SETP12	-0.994	-2.174	-3.311	0.001
ENSG00000251687	RP11-181K12.2	-0.563	-1.433	-2.234	0.028
ENSG00000251735	SNORA25	-0.582	-1.723	-2.311	0.023
ENSG00000252414	RNU6-100P	-0.519	-2.048	-2.022	0.046
ENSG00000252494	RNU6-126P	-0.674	-2.330	-2.143	0.035
ENSG00000252498	RNU6-1016P	-0.324	1.099	-2.158	0.034
ENSG00000252634	RN7SKP219	-0.820	-1.845	-2.516	0.014
ENSG00000252707	RNU11-2P	-0.372	0.062	-2.024	0.046
ENSG00000252772	RNU6-714P	-0.872	-2.639	-2.633	0.010
ENSG00000252782	RNU6-341P	-0.445	-1.670	-2.069	0.042
ENSG00000253104	RP11-855C21.1	-0.621	-1.878	-2.022	0.046
ENSG00000253138	LINC00967	-0.629	-1.887	-2.117	0.037
ENSG00000253197	CTD-3239E11.2	-0.381	-0.718	-2.139	0.035
ENSG00000253288	RP11-238K6.1	-0.560	0.929	-2.220	0.029
ENSG00000253406	AC012613.2	-0.393	1.445	-2.135	0.036
ENSG00000253456	CTD-2363C16.2	-0.804	-1.887	-2.308	0.023
ENSG00000253536	RP11-1174L13.2	-0.727	-1.528	-2.225	0.029
ENSG00000253558	CTD-2179L22.1	-0.412	-0.180	-2.022	0.046
ENSG00000253582	RP11-649G15.2	-0.449	1.057	-2.546	0.013
ENSG00000253602	RN7SL260P	0.972	-3.446	2.358	0.021
ENSG00000253633	KB-1980E6.3	0.551	0.059	2.615	0.011
ENSG00000253653	CTC-248O19.1	0.819	-2.597	2.434	0.017
ENSG00000253667	RP11-30L15.4	-0.394	1.571	-2.516	0.014
ENSG00000253671	RP11-806O11.1	-0.462	1.144	-2.037	0.045
ENSG00000253686	LINC01484	0.418	1.094	2.038	0.045
ENSG00000253762	RP11-579E24.2	0.553	0.029	3.044	0.003
ENSG00000253767	PCDHGA8	-0.254	2.589	-1.999	0.049
ENSG00000253824	KB-173C10.2	1.000	-2.292	2.552	0.013
ENSG00000253855	AC133633.1	-0.790	-2.237	-2.090	0.040
ENSG00000253932	RP11-10C8.2	-0.545	1.016	-2.545	0.013
ENSG00000254034	RP11-662B19.2	-0.434	0.556	-2.258	0.027
ENSG00000254050	RP11-577N1.1	-0.759	-1.073	-2.436	0.017
ENSG00000254051	RP11-403D15.1	-0.643	-1.299	-2.226	0.029
ENSG00000254163	CTC-340I23.2	-0.532	-1.387	-2.320	0.023
ENSG00000254266	PKIA-AS1	-0.391	2.157	-3.051	0.003
ENSG00000254330	RP11-1D12.1	-0.446	-0.223	-2.080	0.041
ENSG00000254396	RP11-56F10.3	0.759	-1.859	2.175	0.032
ENSG00000254397	CTD-2371O3.2	-0.454	-1.224	-2.093	0.039
ENSG00000254398	RP11-578F21.2	-0.538	-0.313	-2.390	0.019

ENSG00000254432	RP11-33I11.2	-0.859	-2.022	-3.262	0.002
ENSG00000254449	SF3A3P2	-0.586	2.153	-2.041	0.045
ENSG00000254590	RP11-334E6.2	-0.448	0.068	-2.502	0.014
ENSG00000254602	AP000662.4	0.287	0.526	2.198	0.031
ENSG00000254610	RP5-903G2.2	0.527	-0.882	2.489	0.015
ENSG00000254664	CTD-2560E9.3	0.637	-0.312	2.841	0.006
ENSG00000254708	RP1-145M24.1	-0.658	-0.026	-2.072	0.041
ENSG00000254726	MEX3A	0.371	0.534	2.103	0.039
ENSG00000254843	RP11-648O15.1	-0.496	-0.066	-2.659	0.009
ENSG00000254885	RP11-802F5.1	-0.888	-2.512	-2.312	0.023
ENSG00000254915	RP11-263C24.3	-0.672	-2.118	-2.247	0.027
ENSG00000255000	AC139103.1	-0.934	-1.794	-3.069	0.003
ENSG00000255087	RP11-168K9.2	0.459	2.174	2.467	0.016
ENSG00000255135	RP11-111M22.3	0.338	1.002	2.250	0.027
ENSG00000255182	CTD-2517M22.14	0.385	0.346	2.039	0.045
ENSG00000255197	RP11-750H9.5	-0.362	-0.139	-2.129	0.036
ENSG00000255220	DDX18P5	-0.368	0.624	-2.453	0.016
ENSG00000255234	RP11-727A23.10	-0.822	-1.640	-3.173	0.002
ENSG00000255296	RP11-757C15.4	-0.691	-1.688	-2.424	0.018
ENSG00000255334	RP11-708L7.6	-0.634	-1.695	-2.245	0.027
ENSG00000255423	EBLN2	-0.301	1.719	-2.094	0.039
ENSG00000255435	RP11-770J1.3	-0.421	-1.692	-2.087	0.040
ENSG00000255449	RP11-91P24.6	-0.613	-1.317	-2.202	0.030
ENSG00000255559	ZNF252P-AS1	-0.287	0.183	-2.074	0.041
ENSG00000255565	RP11-6B19.1	-0.728	-1.344	-2.714	0.008
ENSG00000255566	RP11-114F3.5	-0.757	-1.276	-2.264	0.026
ENSG00000255571	MIR9-3HG	0.151	5.097	2.030	0.046
ENSG00000255580	AP000462.2	-0.344	2.036	-2.161	0.034
ENSG00000255618	LINC02440	0.538	-1.120	2.292	0.024
ENSG00000255837	TAS2R20	-0.312	1.726	-2.138	0.036
ENSG00000256053	APOPT1	0.246	2.796	2.556	0.012
ENSG00000256128	LINC00944	0.646	-1.363	2.294	0.024
ENSG00000256139	RP11-968O1.5	-0.498	-0.402	-2.265	0.026
ENSG00000256181	AP000438.4	0.405	0.808	2.084	0.040
ENSG00000256193	LINC00507	0.530	4.988	2.327	0.022
ENSG00000256436	TAS2R31	-0.280	1.996	-2.206	0.030
ENSG00000256574	OR13A1	-1.104	-2.517	-2.928	0.004
ENSG00000256616	RP11-815J4.6	-0.541	2.436	-3.164	0.002
ENSG00000256637	LINC01965	0.566	0.190	2.434	0.017
ENSG00000256817	TPT1P12	-0.618	-1.895	-2.170	0.033
ENSG00000256847	CCND2P1	0.527	1.039	3.229	0.002
ENSG00000256863	RP11-959F10.6	0.449	0.970	2.051	0.044
ENSG00000256897	RP11-17G12.2	-0.683	-1.465	-2.241	0.028
ENSG00000257057	C11orf97	0.402	-0.116	2.265	0.026
ENSG00000257060	RP11-266O8.1	-0.333	1.701	-2.421	0.018
ENSG00000257150	PGAM1P5	-0.446	-0.561	-2.138	0.036
ENSG00000257354	RP11-631N16.2	-0.295	2.897	-2.117	0.037

ENSG00000257497	RP11-585P4.5	-0.572	0.071	-2.652	0.010
ENSG00000257621	PSMA3-AS1	-0.141	5.754	-2.089	0.040
ENSG00000257947	RP11-46I1.1	-0.514	-1.830	-2.003	0.048
ENSG00000257951	RP11-554D14.4	0.612	-1.318	2.119	0.037
ENSG00000257957	QRSL1P3	-0.393	1.149	-2.479	0.015
ENSG00000258033	RP11-350G24.2	-0.421	2.724	-3.157	0.002
ENSG00000258136	RP11-864J10.4	-0.409	0.116	-2.273	0.026
ENSG00000258204	RP11-90E9.1	-0.400	1.515	-2.107	0.038
ENSG00000258212	ZNF75BP	-0.407	-0.148	-2.275	0.026
ENSG00000258274	RP11-887P2.5	-0.322	1.562	-2.243	0.028
ENSG00000258279	LINC00592	1.286	-1.354	3.802	0.000
ENSG00000258376	RP4-647C14.2	-0.606	0.001	-2.763	0.007
ENSG00000258571	PTTG4P	-0.555	-0.937	-2.232	0.028
ENSG00000258640	RPL21P5	-0.634	-1.029	-2.511	0.014
ENSG00000258754	LINC01579	-0.468	0.652	-2.728	0.008
ENSG00000258891	RP5-1021I20.5	-0.396	0.712	-2.304	0.024
ENSG00000258900	HNRNPCP1	-0.287	1.076	-2.856	0.005
ENSG00000258972	NDUFB8P1	-0.700	-1.711	-2.350	0.021
ENSG00000258986	TMEM179	0.226	4.616	2.357	0.021
ENSG00000259073	FOXN3-AS2	-0.571	0.058	-3.207	0.002
ENSG00000259096	FAM35CP	-0.344	1.173	-2.048	0.044
ENSG00000259232	RP11-349G13.1	-0.614	-1.611	-2.474	0.015
ENSG00000259241	RP11-313P18.2	-0.638	-0.148	-2.852	0.005
ENSG00000259287	RP11-3D4.2	-0.394	-0.688	-2.048	0.044
ENSG00000259296	RP11-313P18.1	-0.466	0.257	-2.516	0.014
ENSG00000259378	DCAF13P3	-0.244	1.723	-2.277	0.025
ENSG00000259442	RP11-752G15.8	-0.418	-0.035	-2.562	0.012
ENSG00000259463	RP11-540O11.7	-0.477	-1.446	-2.095	0.039
ENSG00000259481	RP11-39M21.1	-0.374	2.195	-2.336	0.022
ENSG00000259505	RP11-74D7.1	-1.111	-0.535	-3.375	0.001
ENSG00000259528	RP11-272D12.2	-0.386	-0.341	-2.100	0.039
ENSG00000259562	RP11-762H8.2	-0.343	1.272	-2.103	0.039
ENSG00000259564	RP11-16B9.1	-0.470	-0.286	-2.322	0.023
ENSG00000259583	RP11-66B24.4	0.227	4.164	2.095	0.039
ENSG00000259591	RP11-554D20.1	-0.423	1.013	-2.256	0.027
ENSG00000259642	ST20-AS1	0.269	1.162	2.087	0.040
ENSG00000259644	RP11-680F8.4	-0.433	1.757	-2.599	0.011
ENSG00000259668	RP11-707P17.2	-0.352	0.798	-2.112	0.038
ENSG00000259688	RP11-139F4.2	0.853	-2.738	2.067	0.042
ENSG00000259740	RP11-142J21.2	-0.699	-2.308	-2.060	0.043
ENSG00000259823	LYPD8	0.340	2.773	2.789	0.007
ENSG00000259831	LINC00567	0.492	-0.889	2.305	0.024
ENSG00000260025	RP11-490M8.1	0.329	1.426	2.175	0.032
ENSG00000260060	RP11-388M20.1	-0.628	-0.958	-2.754	0.007
ENSG00000260166	RP11-863P13.6	0.437	-0.048	2.521	0.014
ENSG00000260194	RP11-357N13.2	-0.320	0.747	-2.283	0.025
ENSG00000260328	RP11-416I2.1	0.292	3.552	2.340	0.022

ENSG00000260455	NBAT1	0.372	0.238	2.276	0.025
ENSG00000260460	RP11-284F21.8	0.586	-0.188	2.557	0.012
ENSG00000260517	RP11-426C22.5	-0.662	-1.128	-2.730	0.008
ENSG00000260558	RP11-63M22.1	-0.412	0.032	-2.172	0.033
ENSG00000260576	EIF5A2P1	-0.459	-0.801	-2.218	0.029
ENSG00000260599	CTC-457E21.1	-0.742	1.101	-2.361	0.021
ENSG00000260628	RP11-1166P10.1	-0.442	0.430	-2.502	0.014
ENSG00000260733	RP11-264L1.4	-0.430	-0.092	-2.011	0.048
ENSG00000260744	RP11-329J18.3	-0.440	0.280	-2.305	0.024
ENSG00000260816	RP11-319G9.3	-0.379	2.119	-2.366	0.020
ENSG00000260891	RP11-96D1.5	-0.614	-0.677	-3.048	0.003
ENSG00000260896	PRCAT47	0.513	0.091	2.890	0.005
ENSG00000261037	CTC-471C19.1	0.337	3.293	2.248	0.027
ENSG00000261114	RP11-325K4.2	-0.604	-1.284	-2.083	0.040
ENSG00000261204	AC004449.6	1.090	-2.528	3.039	0.003
ENSG00000261236	BOP1	0.200	3.483	2.236	0.028
ENSG00000261360	CTD-2165H16.4	-0.439	0.824	-2.539	0.013
ENSG00000261468	RP11-1024P17.1	-0.910	-2.474	-2.256	0.027
ENSG00000261537	RP11-319G9.5	-0.426	-0.154	-2.123	0.037
ENSG00000261668	RP11-50D9.3	-0.473	0.838	-2.096	0.039
ENSG00000261997	RP11-212I21.4	-0.729	0.047	-2.159	0.034
ENSG00000262038	RP11-177N22.3	-0.614	-1.790	-2.207	0.030
ENSG00000262503	RP11-530N7.2	-0.399	0.880	-2.419	0.018
ENSG00000262580	RP11-334C17.5	-0.465	0.168	-2.331	0.022
ENSG00000262668	AJ003147.9	0.881	-2.666	2.429	0.017
ENSG00000262732	CTD-2135D7.2	-0.445	-0.083	-2.198	0.031
ENSG00000263177	MTND1P8	-0.586	0.052	-2.566	0.012
ENSG00000263400	TMEM220-AS1	-0.509	-0.599	-2.229	0.029
ENSG00000263477	RP11-338L22.2	-0.404	0.131	-2.390	0.019
ENSG00000263878	DLGAP1-AS4	0.247	4.161	2.207	0.030
ENSG00000263924	RP11-210K20.2	-0.705	-0.778	-3.094	0.003
ENSG00000264016	CTC-297N7.9	-0.723	-1.837	-2.662	0.009
ENSG00000264151	RP11-739N10.1	0.744	-1.801	2.382	0.020
ENSG00000264246	RP11-138C24.2	-0.640	-2.250	-2.066	0.042
ENSG00000264343	NOTCH2NL	-0.468	2.719	-2.607	0.011
ENSG00000264589	MAPT-AS1	0.274	2.553	2.346	0.021
ENSG00000264754	CTD-2653B5.1	-0.916	-0.893	-3.857	0.000
ENSG00000265194	RP11-70L8.4	-0.445	1.411	-3.258	0.002
ENSG00000265313	LINC01476	0.406	2.122	2.300	0.024
ENSG00000265366	GLUD1P7	0.461	-0.532	2.494	0.015
ENSG00000265394	RP11-1148O4.2	-0.775	-1.655	-2.972	0.004
ENSG00000265451	RP11-204L24.2	-0.564	-1.668	-2.165	0.033
ENSG00000265656	RP11-13N13.6	-0.827	-1.609	-2.377	0.020
ENSG00000265739	RP11-354P11.3	-0.410	-0.475	-2.247	0.027
ENSG00000265763	ZNF488	0.245	3.572	2.078	0.041
ENSG00000265778	RP11-17M16.2	-0.376	-0.069	-2.094	0.039
ENSG00000265908	RP11-20B24.6	-0.585	-1.285	-2.230	0.029

ENSG00000266805	RP11-61L19.1	-0.423	0.989	-2.302	0.024
ENSG00000267056	AC005336.4	-0.432	1.092	-2.255	0.027
ENSG00000267193	RP11-116O18.3	-0.694	-0.337	-2.373	0.020
ENSG00000267321	LINC02001	0.359	1.290	2.910	0.005
ENSG00000267353	CTD-2162K18.3	0.980	-2.392	2.857	0.005
ENSG00000267508	ZNF285	0.135	3.769	2.020	0.047
ENSG00000267524	RPSAP57	-0.509	-0.097	-2.156	0.034
ENSG00000267692	TAF9P3	-0.436	-0.486	-2.069	0.042
ENSG00000268225	CTD-2331H12.5	-0.274	1.138	-2.139	0.035
ENSG00000268659	RP11-310J24.3	-0.714	-2.087	-2.006	0.048
ENSG00000268705	BNIP3P26	-0.522	-0.622	-2.168	0.033
ENSG00000268717	BNIP3P33	-0.743	-0.733	-2.558	0.012
ENSG00000268830	BNIP3P32	-0.726	0.190	-2.528	0.013
ENSG00000269124	AC007193.10	-0.669	-1.272	-2.284	0.025
ENSG00000269235	ZNF350-AS1	0.420	0.106	2.460	0.016
ENSG00000269289	CTB-92J24.3	-0.698	-2.026	-2.195	0.031
ENSG00000269378	ITGB1P1	-0.664	0.896	-2.412	0.018
ENSG00000269793	ZIM2-AS1	0.230	2.330	2.035	0.045
ENSG00000269887	RP11-477H21.2	-0.353	1.454	-2.293	0.024
ENSG00000269907	RP11-330M2.4	-0.408	0.651	-2.401	0.019
ENSG00000269950	AP000962.2	-0.300	1.254	-2.144	0.035
ENSG00000269952	RP11-324I22.3	-0.555	-1.571	-2.048	0.044
ENSG00000270071	AP001172.2	-0.422	1.709	-3.069	0.003
ENSG00000270084	GAS5-AS1	-0.278	0.455	-2.171	0.033
ENSG00000270344	RP11-734K2.4	-0.271	4.121	-2.482	0.015
ENSG00000270462	RP11-342K6.3	-0.623	-0.316	-2.705	0.008
ENSG00000270540	RP11-785G17.1	-0.752	-1.744	-2.227	0.029
ENSG00000270585	RP11-568G11.4	-0.386	0.632	-2.294	0.024
ENSG00000270614	CTC-325H20.7	-0.512	0.389	-2.607	0.011
ENSG00000270661	RP1-142L7.9	-0.701	-1.547	-2.218	0.029
ENSG00000270733	RP11-231P20.5	-0.582	-2.100	-2.000	0.049
ENSG00000270776	RP11-17G11.1	-0.799	-1.645	-2.973	0.004
ENSG00000270842	RP11-57B24.2	-0.572	-0.109	-3.138	0.002
ENSG00000270885	RASL10B	0.297	4.490	2.462	0.016
ENSG00000270893	RP11-311B14.1	-0.547	-0.789	-2.175	0.033
ENSG00000270964	RP11-502I4.3	0.575	-0.968	3.016	0.003
ENSG00000271064	RP11-792A8.3	-0.376	0.093	-2.055	0.043
ENSG00000271159	HINT1P2	-0.489	-0.905	-2.541	0.013
ENSG00000271270	TMCC1-AS1	0.290	3.333	3.248	0.002
ENSG00000271327	RP11-1109F11.3	-0.395	1.014	-2.405	0.018
ENSG00000271428	RP1-224A6.8	-0.472	-0.047	-2.117	0.037
ENSG00000271500	RP11-288K12.1	-0.397	1.434	-1.991	0.050
ENSG00000271639	RP11-289A15.1	-0.407	0.323	-2.204	0.030
ENSG00000271710	NDUFA8P1	-0.723	-1.645	-2.595	0.011
ENSG00000271711	RP11-392O18.2	-0.575	0.529	-2.320	0.023
ENSG00000271771	RP11-1250I15.3	-0.520	-0.220	-2.633	0.010
ENSG00000271869	RP11-51J9.5	0.838	-2.249	2.441	0.017

ENSG00000272040	CTC-366B18.4	-0.295	0.150	-2.039	0.045
ENSG00000272072	CTA-363E19.2	-0.285	1.896	-2.527	0.013
ENSG00000272240	RP5-855D21.1	-0.350	0.498	-2.215	0.030
ENSG00000272247	RP11-379F4.9	-0.796	-2.145	-2.440	0.017
ENSG00000272542	RP11-255P5.2	-0.554	0.411	-1.999	0.049
ENSG00000272556	GTF2IP13	-0.366	0.749	-1.995	0.049
ENSG00000272564	RP11-548P2.2	-0.563	-1.084	-2.018	0.047
ENSG00000272599	RP11-152N13.16	-0.288	0.839	-2.198	0.031
ENSG00000272645	GTF2IP20	-0.280	2.044	-2.083	0.040
ENSG00000272732	RP5-1159O4.2	-0.364	1.670	-3.189	0.002
ENSG00000272750	RP11-378J18.8	-0.239	1.883	-2.110	0.038
ENSG00000272822	RP11-302B13.5	0.696	-2.013	2.935	0.004
ENSG00000272944	CTD-2308L22.1	0.480	0.204	2.908	0.005
ENSG00000273032	DGCR9	0.258	5.125	2.090	0.040
ENSG00000273035	RP11-449G16.1	-0.539	-0.254	-2.192	0.031
ENSG00000273238	RP11-1263C18.1	0.274	3.716	2.453	0.016
ENSG00000273247	RP11-83A24.2	-0.176	3.645	-2.068	0.042
ENSG00000273302	RP11-493E12.2	-0.415	1.302	-2.185	0.032
ENSG00000273419	RP4-751H13.7	-0.432	-0.422	-2.798	0.006
ENSG00000273604	C17orf96	0.392	3.179	2.681	0.009
ENSG00000273747	RP11-90B9.3	-0.697	-1.588	-2.509	0.014
ENSG00000273768	RNVU1-29	0.532	7.304	2.068	0.042
ENSG00000273891	RP11-500G22.5	-0.371	0.735	-2.274	0.026
ENSG00000273987	RP11-585P4.6	-0.361	0.055	-2.013	0.047
ENSG00000274038	RP11-66H6.4	-0.690	-2.320	-2.038	0.045
ENSG00000274105	RP11-278C7.3	-0.581	0.522	-3.021	0.003
ENSG00000274204	RP11-272L14.2	-0.542	-0.917	-2.508	0.014
ENSG00000274211	SOCS7	0.147	6.520	2.201	0.031
ENSG00000274253	RP11-566K19.6	0.280	3.692	2.753	0.007
ENSG00000274331	RP11-403A3.1	0.707	-1.471	2.060	0.043
ENSG00000274667	RP11-31H5.3	-0.751	-1.077	-3.288	0.001
ENSG00000274756	CTB-75G16.1	-0.424	-0.112	-2.191	0.031
ENSG00000275340	FGD5P1	0.377	0.850	2.445	0.017
ENSG00000275607	RP11-802O23.4	-0.666	-1.085	-2.759	0.007
ENSG00000275678	RP4-547N15.3	0.784	-1.730	2.519	0.014
ENSG00000275878	RN7SL43P	0.833	-3.867	2.138	0.035
ENSG00000276148	RP11-278C7.4	-0.439	-1.001	-2.203	0.030
ENSG00000276174	RP11-687E1.2	-0.448	-0.981	-2.037	0.045
ENSG00000276334	AL133243.1	-0.296	2.856	-2.419	0.018
ENSG00000276393	RP11-106D4.3	-0.700	-0.823	-2.784	0.007
ENSG00000277050	RP11-102G14.1	-0.549	-1.606	-2.063	0.042
ENSG00000277171	RP11-191H23.1	-0.557	0.409	-2.628	0.010
ENSG00000277511	CTD-2095E4.5	1.090	-2.648	2.590	0.011
ENSG00000277526	RP11-378A12.1	-0.603	-0.578	-2.124	0.037
ENSG00000277558	RP3-468O1.6	-0.611	-2.037	-2.329	0.022
ENSG00000277566	RP11-545P7.9	-0.543	-1.061	-2.186	0.032
ENSG00000277589	RP11-333J10.3	-0.802	-1.497	-3.030	0.003

ENSG00000277972	CISD3	0.303	2.217	2.509	0.014
ENSG00000278012	RP1-310O13.13	-0.634	-0.897	-2.693	0.009
ENSG00000278206	RP1-20N2.8	-0.380	-0.094	-2.296	0.024
ENSG00000278344	RP11-18C24.8	-0.906	-2.330	-2.724	0.008
ENSG00000278413	uc_338	-0.728	-1.536	-2.612	0.011
ENSG00000278472	RP11-74D7.3	-0.749	-1.627	-2.135	0.036
ENSG00000278576	RP11-507K2.6	-0.242	2.783	-2.188	0.032
ENSG00000278886	RP11-108A14.1	-0.668	-1.373	-2.460	0.016
ENSG00000278893	RP11-229O3.1	-0.528	-0.210	-2.801	0.006
ENSG00000279041	CTD-2373N4.3	-0.604	-0.566	-2.567	0.012
ENSG00000279118	RP11-517I3.2	-0.369	4.063	-2.179	0.032
ENSG00000279128	RP11-76C10.4	1.127	-0.967	2.645	0.010
ENSG00000279129	RP11-264L1.2	-0.406	1.617	-2.319	0.023
ENSG00000279133	RP11-342K2.1	-0.217	3.089	-2.185	0.032
ENSG00000279159	RP3-394A18.1	-0.272	5.179	-2.604	0.011
ENSG00000279281	RP11-226M10.3	-0.429	1.457	-2.572	0.012
ENSG00000279289	RP3-331H24.6	-0.375	2.556	-2.034	0.045
ENSG00000279304	RP11-573M3.6	-0.682	-2.218	-2.025	0.046
ENSG00000279314	RP1-257C22.2	-0.601	-0.086	-2.461	0.016
ENSG00000279377	AC003973.3	-0.948	1.706	-3.781	0.000
ENSG00000279530	AC092881.1	-0.348	2.917	-2.286	0.025
ENSG00000279537	RP11-133K1.8	0.489	-0.299	2.143	0.035
ENSG00000279565	RP4-570O12.3	0.568	-0.530	2.442	0.017
ENSG00000279570	CTD-2357A8.2	-0.377	-0.068	-1.990	0.050
ENSG00000279594	RP11-950C14.10	-0.542	-0.206	-2.542	0.013
ENSG00000279606	RP11-502F1.1	-0.642	-1.063	-2.086	0.040
ENSG00000279636	LINC00216	-0.296	2.280	-2.346	0.021
ENSG00000279673	RP11-185E8.2	0.414	0.101	2.318	0.023
ENSG00000279707	RP11-380M21.1	-0.675	-1.458	-2.256	0.027
ENSG00000279786	CTD-2014D20.1	-0.529	0.461	-2.438	0.017
ENSG00000279814	RP11-399K21.12	-0.587	-0.310	-2.268	0.026
ENSG00000279836	RP11-648O15.2	-0.447	-1.043	-2.035	0.045
ENSG00000279904	CTD-2015C24.1	0.266	1.421	2.247	0.027
ENSG00000279985	CTC-436P18.4	-0.428	0.398	-1.996	0.049
ENSG00000280079	CTC-260E6.4	-0.389	-0.323	-2.362	0.021
ENSG00000280115	RP11-133M8.3	-0.544	-0.912	-2.254	0.027
ENSG00000280129	RP11-298I3.3	-0.555	-0.958	-2.313	0.023
ENSG00000280182	RP11-626G3.1	0.456	-0.074	2.016	0.047
ENSG00000280190	RP11-319G9.1	-0.505	1.023	-2.790	0.007
ENSG00000280269	AP000577.2	-0.451	0.842	-2.875	0.005
ENSG00000280347	AC000123.2	-0.400	3.319	-3.428	0.001
ENSG00000280348	RP11-552M6.1	-0.472	0.933	-2.183	0.032
ENSG00000280376	RP11-93O14.3	-0.871	-2.047	-3.028	0.003
ENSG00000280392	RP11-357N13.6	-0.294	2.544	-2.038	0.045
ENSG00000280397	RP11-42F12.1	-0.831	-0.855	-2.839	0.006
ENSG00000280432	AP000962.1	-0.327	1.235	-2.372	0.020
ENSG00000280660	RP11-437J2.3	-0.347	4.864	-2.856	0.005

ENSG00000281021	RP1-12G14.9	-0.423	-0.211	-2.064	0.042
ENSG00000281026	N4BP2L2-IT2	-0.307	4.938	-2.419	0.018
ENSG00000281131	SCHLAP1	0.528	-0.343	2.331	0.022
ENSG00000281186	LINC00706	-1.080	-2.242	-2.232	0.028
ENSG00000281969	XXyac-YR38GF2.1	-0.504	2.257	-2.142	0.035
ENSG00000282100	HSP90AB4P	-0.270	1.813	-2.296	0.024
ENSG00000282863	RP11-90J7.3	-0.580	-1.379	-2.331	0.022
ENSG00000282864	RP11-487A2.1	-0.802	-0.545	-2.623	0.010
ENSG00000282865	RP11-306O1.2	-0.470	1.546	-2.030	0.046
ENSG00000282961	PRNCR1	-0.637	0.149	-2.661	0.009
ENSG00000283078	RP11-11M20.4	0.305	1.473	2.828	0.006
ENSG00000283125	RP11-299P2.2	-0.723	-1.088	-2.832	0.006
ENSG00000283445	RP11-63G10.4	0.507	0.494	2.503	0.014
ENSG00000283509	U6	-0.661	-1.312	-2.834	0.006
ENSG00000283646	LINC02009	0.650	0.632	3.119	0.003
ENSG00000284052	#N/A	-0.216	3.142	-2.026	0.046
ENSG00000284966	#N/A	-0.385	3.030	-3.242	0.002
ENSG00000285253	#N/A	-0.380	-0.908	-2.015	0.047
ENSG00000285287	#N/A	-0.541	-1.406	-2.163	0.033
ENSG00000285458	#N/A	-0.419	-1.037	-2.066	0.042
ENSG00000285577	#N/A	-0.997	-2.243	-2.957	0.004
ENSG00000285595	#N/A	-0.794	-1.584	-3.474	0.001
ENSG00000285608	#N/A	-0.693	-2.516	-2.003	0.049
ENSG00000285618	#N/A	-0.629	-0.551	-2.262	0.026
ENSG00000285621	#N/A	-0.342	0.529	-2.135	0.036
ENSG00000285622	#N/A	0.514	-0.819	2.976	0.004
ENSG00000285627	#N/A	-0.415	1.278	-2.225	0.029
ENSG00000285631	#N/A	-0.694	-1.359	-2.084	0.040
ENSG00000285634	#N/A	0.374	2.938	2.084	0.040
ENSG00000285691	#N/A	-1.085	-2.044	-2.714	0.008
ENSG00000285735	#N/A	0.719	0.493	2.634	0.010
ENSG00000285807	#N/A	-0.653	-1.233	-2.450	0.016
ENSG00000285813	#N/A	-0.205	2.839	-1.993	0.050
ENSG00000285830	#N/A	-0.357	-0.631	-2.000	0.049
ENSG00000285836	#N/A	-0.336	0.200	-1.995	0.049
ENSG00000285850	#N/A	-0.387	1.360	-1.996	0.049
ENSG00000285851	#N/A	-0.384	0.439	-2.245	0.027
ENSG00000285943	#N/A	-0.332	0.545	-2.400	0.019
ENSG00000285966	#N/A	0.465	0.656	2.111	0.038
ENSG00000285980	#N/A	-0.488	1.854	-3.381	0.001

vmPFC

gene_id	gene_name	logFC	AveExpr	t	P.Value
ENSG00000001617	SEMA3F	0.911	0.046	3.124	0.003
ENSG00000003989	SLC7A2	-0.497	5.021	-2.314	0.023
ENSG00000005339	CREBBP	-0.183	7.144	-2.162	0.034
ENSG00000007129	CEACAM21	0.994	-1.026	2.082	0.041
ENSG00000007372	PAX6	-0.372	4.892	-2.533	0.013
ENSG00000007908	SELE	-1.236	-0.173	-2.417	0.018
ENSG00000011105	TSPAN9	0.253	2.797	2.432	0.017
ENSG00000011376	LARS2	0.175	5.113	2.028	0.046
ENSG00000013503	POLR3B	0.173	4.607	2.164	0.034
ENSG00000016864	GLT8D1	0.145	4.551	2.176	0.033
ENSG00000019186	CYP24A1	1.127	-0.751	2.168	0.033
ENSG00000023041	ZDHHC6	0.154	4.386	2.106	0.038
ENSG00000023734	STRAP	0.251	5.984	2.223	0.029
ENSG00000037241	RPL26L1	0.476	2.140	2.716	0.008
ENSG00000038002	AGA	0.386	3.339	2.415	0.018
ENSG00000038945	MSR1	-0.731	2.267	-2.327	0.023
ENSG00000049246	PER3	-0.192	6.211	-2.024	0.046
ENSG00000049860	HEXB	0.185	4.629	2.284	0.025
ENSG00000053747	LAMA3	-0.309	3.901	-2.240	0.028
ENSG00000060718	COL11A1	0.241	5.598	2.386	0.020
ENSG00000062282	DGAT2	0.377	1.866	2.098	0.039
ENSG00000065183	WDR3	0.186	5.081	2.361	0.021
ENSG00000065882	TBC1D1	-0.220	5.837	-2.199	0.031
ENSG00000066405	CLDN18	-0.666	0.812	-2.283	0.025
ENSG00000068383	INPP5A	0.289	5.032	2.099	0.039
ENSG00000069702	TGFBR3	-0.362	4.643	-1.995	0.050
ENSG00000070501	POLB	0.186	3.866	2.092	0.040
ENSG00000072121	ZFYVE26	-0.131	5.632	-2.581	0.012
ENSG00000072163	LIMS2	0.389	3.462	2.160	0.034
ENSG00000072694	FCGR2B	-0.815	-0.933	-2.196	0.031
ENSG00000072954	TMEM38A	0.282	3.936	2.207	0.030
ENSG00000075089	ACTR6	0.237	4.799	2.641	0.010
ENSG00000075711	DLG1	0.218	7.859	2.106	0.039
ENSG00000075884	ARHGAP15	-0.545	1.799	-3.022	0.003
ENSG00000076043	REXO2	0.177	3.995	2.442	0.017
ENSG00000076108	BAZ2A	-0.187	6.373	-2.379	0.020
ENSG00000076321	KLHL20	0.169	4.689	1.999	0.049
ENSG00000077348	EXOSC5	0.342	1.232	2.134	0.036
ENSG00000080603	SRCAP	-0.306	2.307	-3.038	0.003
ENSG00000081087	OSTM1	0.204	4.973	2.456	0.016
ENSG00000081248	CACNA1S	0.824	0.255	2.975	0.004
ENSG00000082482	KCNK2	0.300	4.373	2.177	0.033
ENSG00000083845	RPS5	0.258	5.530	2.019	0.047
ENSG00000084112	SSH1	-0.222	6.033	-2.919	0.005
ENSG00000087510	TFAP2C	-1.120	-1.102	-2.677	0.009

ENSG00000088305	DNMT3B	-0.298	1.146	-2.155	0.034
ENSG00000090104	RGS1	-1.786	4.151	-3.123	0.003
ENSG00000090263	MRPS33	0.200	4.572	2.118	0.037
ENSG00000090339	ICAM1	-0.783	1.270	-2.017	0.047
ENSG00000090520	DNAJB11	0.257	4.142	3.101	0.003
ENSG00000090905	TNRC6A	-0.119	7.676	-2.068	0.042
ENSG00000090932	DLL3	0.465	1.241	2.467	0.016
ENSG00000092108	SCFD1	0.189	5.683	2.370	0.020
ENSG00000092758	COL9A3	0.543	2.585	2.153	0.034
ENSG00000092969	TGFB2	-0.357	5.521	-2.204	0.031
ENSG00000094880	CDC23	0.134	4.584	1.999	0.049
ENSG00000099800	TIMM13	0.391	3.054	2.574	0.012
ENSG00000099849	RASSF7	0.589	0.087	2.521	0.014
ENSG00000099992	TBC1D10A	0.255	1.982	2.174	0.033
ENSG00000100024	UPB1	0.833	-1.284	2.306	0.024
ENSG00000100196	KDELR3	-0.289	1.830	-2.065	0.042
ENSG00000100298	APOBEC3H	-0.897	-1.620	-2.547	0.013
ENSG00000100314	CABP7	0.404	1.911	1.992	0.050
ENSG00000100478	AP4S1	0.177	5.094	2.008	0.048
ENSG00000100483	VCPKMT	0.362	2.909	3.286	0.002
ENSG00000100883	SRP54	0.177	5.686	2.004	0.049
ENSG00000101019	UQCC1	0.153	5.521	2.359	0.021
ENSG00000101115	SALL4	-0.541	-0.879	-2.014	0.048
ENSG00000101194	SLC17A9	-0.467	-0.803	-1.996	0.050
ENSG00000101220	C20orf27	0.484	2.872	1.995	0.050
ENSG00000101444	AHCY	0.324	4.215	3.012	0.004
ENSG00000102780	DGKH	0.356	7.327	2.174	0.033
ENSG00000102908	NFAT5	-0.221	8.383	-2.802	0.006
ENSG00000102924	CBLN1	0.665	2.108	2.505	0.014
ENSG00000103024	NME3	0.388	1.575	2.192	0.031
ENSG00000103044	HAS3	-0.311	1.130	-2.016	0.047
ENSG00000103056	SMPD3	0.268	4.360	2.163	0.034
ENSG00000103415	HMOX2	0.254	5.482	2.118	0.037
ENSG00000103429	BFAR	0.151	4.435	2.029	0.046
ENSG00000104154	SLC30A4	0.247	5.134	2.502	0.014
ENSG00000104312	RIPK2	-0.302	3.346	-2.955	0.004
ENSG00000104327	CALB1	0.833	6.047	3.202	0.002
ENSG00000104408	EIF3E	0.224	6.152	2.049	0.044
ENSG00000104522	TSTA3	0.315	2.362	2.245	0.028
ENSG00000104691	UBXN8	0.248	3.253	2.222	0.029
ENSG00000104723	TUSC3	0.262	6.035	2.448	0.017
ENSG00000104774	MAN2B1	-0.307	2.675	-2.255	0.027
ENSG00000104888	SLC17A7	-1.201	6.784	-2.112	0.038
ENSG00000104946	TBC1D17	-0.160	4.010	-2.082	0.041
ENSG00000105173	CCNE1	0.362	1.385	2.708	0.008
ENSG00000105339	DENND3	-0.417	5.992	-2.413	0.018
ENSG00000105671	DDX49	0.296	3.321	2.082	0.041

ENSG00000105784	RUNDC3B	0.241	5.429	2.128	0.037
ENSG00000105851	PIK3CG	-0.473	2.261	-2.259	0.027
ENSG00000105894	PTN	0.200	7.422	2.069	0.042
ENSG00000105929	ATP6V0A4	0.559	-0.215	2.093	0.040
ENSG00000105983	LMBR1	0.192	6.639	2.223	0.029
ENSG00000106003	LFNG	0.530	2.581	2.435	0.017
ENSG00000106133	NSUN5P2	-0.811	0.229	-2.914	0.005
ENSG00000106400	ZNHIT1	0.261	4.088	2.542	0.013
ENSG00000106554	CHCHD3	0.172	4.878	2.179	0.032
ENSG00000106591	MRPL32	0.218	4.463	2.332	0.022
ENSG00000106633	GCK	0.523	0.613	2.028	0.046
ENSG00000106638	TBL2	0.167	3.897	2.263	0.027
ENSG00000107262	BAG1	0.241	4.746	2.408	0.018
ENSG00000107625	DDX50	0.177	5.177	2.169	0.033
ENSG00000107651	SEC23IP	0.127	5.494	2.577	0.012
ENSG00000107821	KAZALD1	0.743	-0.547	2.851	0.006
ENSG00000107937	GTPBP4	0.214	5.199	2.288	0.025
ENSG00000107949	BCCIP	0.150	5.036	2.211	0.030
ENSG00000108010	GLRX3	0.171	4.116	2.085	0.040
ENSG00000108018	SORCS1	0.280	5.701	2.015	0.047
ENSG00000108094	CUL2	0.203	5.774	2.096	0.039
ENSG00000108406	DHX40	0.172	5.229	2.057	0.043
ENSG00000108448	TRIM16L	0.267	2.620	2.394	0.019
ENSG00000108561	C1QBP	0.256	5.489	2.044	0.044
ENSG00000108588	CCDC47	0.153	6.805	2.046	0.044
ENSG00000109762	SNX25	0.193	5.443	2.322	0.023
ENSG00000109971	HSPA8	0.297	10.065	2.495	0.015
ENSG00000110077	MS4A6A	-1.142	1.890	-2.916	0.005
ENSG00000110079	MS4A4A	-0.936	0.425	-2.791	0.007
ENSG00000110324	IL10RA	-0.583	2.823	-2.969	0.004
ENSG00000110448	CD5	-0.511	0.452	-2.653	0.010
ENSG00000110619	CARS	0.185	4.874	2.144	0.035
ENSG00000110871	COQ5	0.210	3.504	2.273	0.026
ENSG00000111058	ACSS3	-0.351	4.992	-2.625	0.010
ENSG00000111252	SH2B3	-0.214	4.760	-2.694	0.009
ENSG00000111676	ATN1	-0.258	6.686	-2.347	0.022
ENSG00000112031	MTRF1L	0.257	3.614	2.955	0.004
ENSG00000112280	COL9A1	0.350	2.838	2.254	0.027
ENSG00000112297	AIM1	-0.269	3.128	-2.168	0.033
ENSG00000112624	GLTSCR1L	-0.142	6.140	-2.203	0.031
ENSG00000112742	TTK	0.736	-1.291	2.456	0.016
ENSG00000113209	PCDHB5	-0.225	3.715	-2.009	0.048
ENSG00000113407	TARS	0.185	5.590	2.084	0.041
ENSG00000113460	BRIX1	0.184	3.891	2.158	0.034
ENSG00000113494	PRLR	1.235	3.467	2.423	0.018
ENSG00000113621	TXNDC15	0.173	5.958	2.064	0.042
ENSG00000113966	ARL6	0.271	4.975	2.081	0.041

ENSG00000114251	WNT5A	-0.416	3.523	-2.888	0.005
ENSG00000114268	PFKFB4	0.259	2.336	2.096	0.039
ENSG00000114686	MRPL3	0.264	5.316	2.066	0.042
ENSG00000115138	POMC	1.463	-1.655	2.566	0.012
ENSG00000115233	PSMD14	0.195	5.393	2.065	0.042
ENSG00000115507	OTX1	-0.610	0.609	-2.654	0.010
ENSG00000115526	CHST10	0.184	4.601	2.153	0.034
ENSG00000115539	PDCL3	0.279	2.770	2.185	0.032
ENSG00000115718	PROC	0.480	-0.665	2.221	0.029
ENSG00000115827	DCAF17	0.189	5.132	2.850	0.006
ENSG00000116128	BCL9	-0.214	4.955	-2.697	0.009
ENSG00000116396	KCNC4	-0.314	5.182	-2.412	0.018
ENSG00000116459	ATP5F1	0.228	6.260	2.272	0.026
ENSG00000116586	LAMTOR2	0.332	2.918	2.600	0.011
ENSG00000116741	RGS2	0.435	4.494	2.440	0.017
ENSG00000116962	NID1	0.398	3.447	2.022	0.047
ENSG00000117543	DPH5	0.203	3.733	2.344	0.022
ENSG00000117594	HSD11B1	0.489	0.883	2.022	0.047
ENSG00000117643	MAN1C1	-0.291	4.873	-2.828	0.006
ENSG00000117713	ARID1A	-0.167	6.918	-2.294	0.025
ENSG00000117906	RCN2	0.224	6.155	2.415	0.018
ENSG00000118058	KMT2A	-0.154	8.271	-2.809	0.006
ENSG00000118402	ELOVL4	0.327	5.225	2.547	0.013
ENSG00000118976	OTUD4P1	-0.382	2.738	-2.019	0.047
ENSG00000119013	NDUFB3	0.417	3.478	2.439	0.017
ENSG00000119242	CCDC92	-0.253	6.378	-2.159	0.034
ENSG00000119888	EPCAM	1.231	1.424	3.297	0.001
ENSG00000119965	C10orf88	0.214	3.613	2.001	0.049
ENSG00000120210	INSL6	-0.333	0.101	-2.219	0.029
ENSG00000120440	TTLL2	1.011	-1.377	3.428	0.001
ENSG00000120697	ALG5	0.275	2.973	2.487	0.015
ENSG00000120899	PTK2B	-0.292	7.011	-2.039	0.045
ENSG00000121022	COPS5	0.174	4.829	2.186	0.032
ENSG00000121741	ZMYM2	0.121	7.987	2.202	0.031
ENSG00000122008	POLK	0.131	5.961	2.121	0.037
ENSG00000122012	SV2C	1.115	5.189	2.032	0.046
ENSG00000122643	NT5C3A	0.201	3.803	2.496	0.015
ENSG00000122707	RECK	-0.162	4.160	-2.217	0.030
ENSG00000123411	IKZF4	-0.190	3.691	-2.036	0.045
ENSG00000123610	TNFAIP6	0.642	0.394	2.113	0.038
ENSG00000124225	PMEPA1	0.345	5.033	2.048	0.044
ENSG00000124541	RRP36	0.263	4.274	2.795	0.007
ENSG00000124749	COL21A1	-0.327	4.351	-2.064	0.042
ENSG00000124802	EEF1E1	0.338	2.205	2.336	0.022
ENSG00000125037	EMC3	0.242	4.826	2.764	0.007
ENSG00000125245	GPR18	-0.307	2.493	-2.056	0.043
ENSG00000125304	TM9SF2	0.215	6.697	2.383	0.020

ENSG00000125347	IRF1	-0.428	2.100	-2.068	0.042
ENSG00000125652	ALKBH7	0.376	2.059	2.028	0.046
ENSG00000125743	SNRPD2	0.277	4.539	2.029	0.046
ENSG00000126460	PRRG2	0.621	-1.958	2.220	0.029
ENSG00000127124	HIVEP3	-0.370	5.711	-2.393	0.019
ENSG00000127125	PPCS	0.273	4.002	2.174	0.033
ENSG00000127527	EPS15L1	-0.178	4.998	-2.113	0.038
ENSG00000127922	SHFM1	0.175	4.816	2.081	0.041
ENSG00000128272	ATF4	0.309	6.261	2.179	0.032
ENSG00000128578	STRIP2	0.556	5.322	2.320	0.023
ENSG00000129103	SUMF2	0.204	4.142	2.390	0.019
ENSG00000129204	USP6	-0.431	2.782	-2.139	0.036
ENSG00000129480	DTD2	0.215	3.991	2.274	0.026
ENSG00000129636	ITFG1	0.268	6.620	1.995	0.050
ENSG00000129691	ASH2L	-0.169	4.817	-2.014	0.048
ENSG00000129993	CBFA2T3	-0.382	2.360	-2.047	0.044
ENSG00000130332	LSM7	0.571	1.468	3.440	0.001
ENSG00000130612	CYP2G1P	-0.444	0.224	-2.028	0.046
ENSG00000130643	CALY	0.419	5.256	2.285	0.025
ENSG00000130811	EIF3G	0.264	4.646	2.248	0.027
ENSG00000130921	C12orf65	0.141	3.866	2.230	0.029
ENSG00000131023	LATS1	-0.107	6.376	-2.064	0.042
ENSG00000131042	LILRB2	-0.915	-1.124	-2.402	0.019
ENSG00000131149	GSE1	-0.191	5.756	-1.996	0.049
ENSG00000131459	GFPT2	-0.265	4.092	-2.329	0.023
ENSG00000131966	ACTR10	0.257	5.731	2.368	0.020
ENSG00000132326	PER2	-0.254	5.065	-2.081	0.041
ENSG00000132388	UBE2G1	0.163	5.361	1.996	0.050
ENSG00000132432	SEC61G	0.457	3.189	3.269	0.002
ENSG00000132437	DDC	1.511	-1.306	2.468	0.016
ENSG00000132622	HSPA12B	0.482	1.144	2.125	0.037
ENSG00000132635	PCED1A	0.190	3.341	2.033	0.046
ENSG00000132749	TESMIN	0.946	-0.329	3.590	0.001
ENSG00000132958	TPTE2	0.502	-1.107	2.009	0.048
ENSG00000133105	RXFP2	1.033	-1.496	2.077	0.041
ENSG00000133116	KL	0.690	2.168	2.017	0.047
ENSG00000133961	NUMB	-0.135	5.970	-2.227	0.029
ENSG00000134057	CCNB1	0.232	3.002	2.005	0.049
ENSG00000134077	THUMPD3	0.136	5.007	2.064	0.042
ENSG00000134253	TRIM45	-0.203	2.975	-2.080	0.041
ENSG00000134452	FBXO18	0.132	5.047	2.352	0.021
ENSG00000134532	SOX5	-0.156	6.689	-2.039	0.045
ENSG00000135312	HTR1B	0.719	1.724	3.010	0.004
ENSG00000135336	ORC3	0.141	5.358	1.993	0.050
ENSG00000135549	PKIB	0.437	2.451	2.172	0.033
ENSG00000135587	SMPD2	-0.336	1.050	-2.158	0.034
ENSG00000135617	PRADC1	0.296	1.866	1.992	0.050

ENSG00000135925	WNT10A	1.446	-2.443	2.707	0.008
ENSG00000135972	MRPS9	0.305	4.299	2.980	0.004
ENSG00000136231	IGF2BP3	0.412	0.358	2.036	0.045
ENSG00000136247	ZDHHC4	0.180	3.506	2.159	0.034
ENSG00000136630	HLX	-0.544	-0.395	-2.043	0.045
ENSG00000136813	KIAA0368	0.166	8.083	2.097	0.039
ENSG00000137038	TMEM261	0.244	4.356	2.274	0.026
ENSG00000137080	IFNA21	0.649	-1.248	2.064	0.042
ENSG00000137142	IGFBPL1	0.727	0.181	3.224	0.002
ENSG00000137288	UQCC2	0.243	4.184	2.543	0.013
ENSG00000137331	IER3	-0.560	0.576	-2.168	0.033
ENSG00000137460	FHDC1	0.759	1.345	2.349	0.021
ENSG00000137462	TLR2	-0.752	2.684	-2.742	0.008
ENSG00000137821	LRRC49	0.260	5.729	3.353	0.001
ENSG00000137955	RABGGTB	0.159	5.973	2.056	0.043
ENSG00000138347	MYPN	-0.840	-1.656	-2.407	0.019
ENSG00000138395	CDK15	-0.860	-0.379	-2.813	0.006
ENSG00000138442	WDR12	0.267	5.456	2.418	0.018
ENSG00000138604	GLCE	0.353	6.078	2.690	0.009
ENSG00000138660	AP1AR	0.278	5.512	2.085	0.040
ENSG00000138668	HNRNPD	-0.158	6.836	-2.165	0.034
ENSG00000138670	RASGEF1B	0.360	4.588	2.250	0.027
ENSG00000138698	RAP1GDS1	0.258	6.442	2.030	0.046
ENSG00000138741	TRPC3	0.406	2.479	2.049	0.044
ENSG00000138798	EGF	-0.417	2.670	-2.250	0.027
ENSG00000138801	PAPSS1	0.206	6.270	2.213	0.030
ENSG00000138944	KIAA1644	-0.368	7.077	-2.062	0.043
ENSG00000138964	PARVG	-0.657	0.916	-2.845	0.006
ENSG00000139193	CD27	0.584	-1.767	2.008	0.048
ENSG00000139436	GIT2	-0.141	5.518	-2.180	0.032
ENSG00000139537	CCDC65	-0.358	4.068	-2.269	0.026
ENSG00000139547	RDH16	-0.394	0.273	-2.088	0.040
ENSG00000139718	SETD1B	-0.253	4.408	-2.523	0.014
ENSG00000139767	SRRM4	-0.430	6.536	-2.310	0.024
ENSG00000139800	ZIC5	-0.625	2.400	-2.541	0.013
ENSG00000139926	FRMD6	-0.283	4.477	-2.135	0.036
ENSG00000140030	GPR65	-0.846	0.799	-3.161	0.002
ENSG00000140307	GTF2A2	0.271	3.298	2.229	0.029
ENSG00000140948	ZCCHC14	-0.174	6.034	-2.630	0.010
ENSG00000141252	VPS53	-0.236	7.277	-2.754	0.007
ENSG00000141293	SKAP1	0.979	-1.341	2.882	0.005
ENSG00000141441	GAREM1	-0.207	5.320	-2.070	0.042
ENSG00000141905	NFIC	-0.295	5.662	-2.565	0.012
ENSG00000142046	TMEM91	0.475	1.951	2.440	0.017
ENSG00000142233	NTN5	-0.553	-0.996	-2.044	0.044
ENSG00000142530	FAM71E1	0.466	0.443	2.132	0.036
ENSG00000142657	PGD	0.240	5.119	2.098	0.039

ENSG00000143107	FNDC7	-0.659	0.344	-2.011	0.048
ENSG00000143226	FCGR2A	-0.685	2.525	-2.024	0.047
ENSG00000143442	POGZ	-0.131	6.933	-2.520	0.014
ENSG00000143575	HAX1	0.250	4.655	2.068	0.042
ENSG00000143621	ILF2	0.207	5.915	2.290	0.025
ENSG00000143643	TTC13	0.149	4.894	2.017	0.047
ENSG00000143653	SCCPDH	0.188	5.454	2.033	0.046
ENSG00000143839	REN	0.660	-2.044	2.083	0.041
ENSG00000143845	ETNK2	0.331	2.041	2.811	0.006
ENSG00000143858	SYT2	0.627	2.928	2.036	0.045
ENSG00000143867	OSR1	-0.751	0.086	-2.070	0.042
ENSG00000143870	PDIA6	0.249	5.152	2.981	0.004
ENSG00000144476	ACKR3	-0.423	2.661	-2.057	0.043
ENSG00000144792	ZNF660	-0.190	4.232	-2.325	0.023
ENSG00000144827	ABHD10	0.213	5.422	2.070	0.042
ENSG00000144847	IGSF11	-0.295	5.364	-3.092	0.003
ENSG00000145050	MANF	0.367	3.288	3.344	0.001
ENSG00000145244	CORIN	0.315	1.759	2.249	0.027
ENSG00000145337	PYURF	0.190	4.406	2.058	0.043
ENSG00000145358	DDIT4L	-0.440	2.022	-2.027	0.046
ENSG00000145730	PAM	0.363	7.321	2.651	0.010
ENSG00000145736	GTF2H2	-0.568	3.298	-2.369	0.020
ENSG00000145757	SPATA9	-0.483	-0.975	-2.189	0.032
ENSG00000145908	ZNF300	0.398	2.538	2.322	0.023
ENSG00000146001	PCDHB18P	-0.403	2.765	-3.166	0.002
ENSG00000146054	TRIM7	-0.347	0.930	-2.236	0.028
ENSG00000146350	TBC1D32	0.161	5.330	2.594	0.011
ENSG00000146409	SLC18B1	-0.297	4.783	-2.205	0.030
ENSG00000146433	TMEM181	0.225	6.458	2.448	0.017
ENSG00000146476	ARMT1	0.293	4.632	2.930	0.004
ENSG00000146477	SLC22A3	0.559	1.083	2.425	0.018
ENSG00000146731	CCT6A	0.232	6.308	2.184	0.032
ENSG00000147408	CSGALNACT1	-0.236	6.292	-2.108	0.038
ENSG00000147852	VLDLR	0.298	5.228	3.132	0.002
ENSG00000147862	NFIB	-0.148	7.934	-2.072	0.042
ENSG00000148090	AUH	0.204	4.431	2.036	0.045
ENSG00000148123	PLPPR1	0.449	4.646	2.445	0.017
ENSG00000148396	SEC16A	-0.128	6.078	-2.081	0.041
ENSG00000148604	RGR	-0.828	2.536	-2.215	0.030
ENSG00000148730	EIF4EBP2	-0.170	7.293	-2.136	0.036
ENSG00000149243	KLHL35	0.306	1.825	2.565	0.012
ENSG00000149532	CPSF7	-0.102	5.760	-2.015	0.047
ENSG00000149547	EI24	0.215	5.053	2.220	0.029
ENSG00000149548	CCDC15	-0.328	2.041	-2.012	0.048
ENSG00000149554	CHEK1	0.248	2.004	2.213	0.030
ENSG00000150361	KLHL1	0.615	3.010	2.338	0.022
ENSG00000150753	CCT5	0.284	5.951	2.467	0.016

ENSG00000150782	IL18	-0.576	1.889	-2.440	0.017
ENSG00000151388	ADAMTS12	0.785	0.035	2.219	0.029
ENSG00000151812	SLC35F4	0.627	0.769	2.131	0.036
ENSG00000152208	GRID2	0.325	5.622	2.509	0.014
ENSG00000152214	RIT2	0.478	3.288	2.284	0.025
ENSG00000152422	XRCC4	0.346	2.598	2.973	0.004
ENSG00000152475	ZNF837	0.481	-0.603	2.105	0.039
ENSG00000153037	SRP19	0.145	4.482	2.050	0.044
ENSG00000153094	BCL2L11	-0.276	2.837	-2.232	0.029
ENSG00000153253	SCN3A	0.257	7.418	2.389	0.019
ENSG00000153339	TRAPPC8	0.156	6.372	2.203	0.031
ENSG00000153902	LGI4	-0.303	5.297	-2.531	0.013
ENSG00000153904	DDAH1	-0.215	8.409	-2.133	0.036
ENSG00000153982	GDPD1	0.230	4.262	2.531	0.013
ENSG00000154174	TOMM70	0.311	7.146	2.270	0.026
ENSG00000154645	CHODL	1.183	0.373	2.192	0.031
ENSG00000154898	CCDC144CP	-0.330	6.010	-2.239	0.028
ENSG00000155304	HSPA13	0.317	6.694	2.509	0.014
ENSG00000155438	NIFK	0.227	3.838	2.380	0.020
ENSG00000155465	SLC7A7	-0.494	1.654	-2.650	0.010
ENSG00000155714	PDZD9	-0.293	0.040	-2.014	0.048
ENSG00000155906	RMND1	0.191	4.223	2.198	0.031
ENSG00000155926	SLA	-0.375	3.888	-2.168	0.033
ENSG00000156261	CCT8	0.256	6.164	2.171	0.033
ENSG00000156304	SCAF4	-0.131	5.115	-2.006	0.048
ENSG00000156574	NODAL	-1.056	-1.335	-3.231	0.002
ENSG00000156875	MFSD14A	0.330	1.678	2.221	0.029
ENSG00000156966	B3GNT7	0.379	2.537	2.327	0.023
ENSG00000157150	TIMP4	-0.291	2.561	-2.314	0.023
ENSG00000157349	DDX19B	-0.139	3.637	-2.203	0.031
ENSG00000157851	DPYSL5	0.347	5.426	2.308	0.024
ENSG00000157916	RER1	0.139	4.918	2.056	0.043
ENSG00000157933	SKI	-0.232	6.233	-2.642	0.010
ENSG00000157985	AGAP1	-0.233	8.064	-2.513	0.014
ENSG00000158716	DUSP23	0.356	1.651	2.261	0.027
ENSG00000159167	STC1	1.067	0.688	2.093	0.040
ENSG00000159239	C2orf81	0.283	3.083	2.003	0.049
ENSG00000159377	PSMB4	0.223	4.591	2.362	0.021
ENSG00000159763	PIP	0.760	-1.985	2.114	0.038
ENSG00000159884	CCDC107	0.710	-1.640	2.198	0.031
ENSG00000160588	MPZL3	0.665	0.532	2.775	0.007
ENSG00000161681	SHANK1	-0.392	5.990	-2.103	0.039
ENSG00000161800	RACGAP1	0.233	3.345	2.255	0.027
ENSG00000162139	NEU3	0.162	5.006	2.119	0.037
ENSG00000162396	PARS2	0.250	1.410	2.028	0.046
ENSG00000162433	AK4	-0.278	6.422	-2.531	0.013
ENSG00000162595	DIRAS3	1.208	3.582	2.287	0.025

ENSG00000162631	NTNG1	0.248	5.073	2.016	0.047
ENSG00000162639	HENMT1	0.346	3.577	2.410	0.018
ENSG00000162670	BRINP3	0.264	5.633	2.408	0.018
ENSG00000162722	TRIM58	0.855	1.823	2.987	0.004
ENSG00000162777	DENND2D	-0.737	0.358	-2.973	0.004
ENSG00000162782	TDRD5	0.811	2.171	3.115	0.003
ENSG00000162804	SNED1	-0.346	4.060	-2.482	0.015
ENSG00000162851	TFB2M	0.248	2.729	2.230	0.029
ENSG00000163125	RPRD2	-0.162	6.592	-2.432	0.017
ENSG00000163293	NIPAL1	0.818	0.568	3.741	0.000
ENSG00000163394	CCKAR	0.743	-0.899	2.009	0.048
ENSG00000163463	KRTCAP2	-0.319	0.668	-2.063	0.042
ENSG00000163468	CCT3	0.238	6.197	2.771	0.007
ENSG00000163517	HDAC11	0.234	4.736	2.044	0.044
ENSG00000163635	ATXN7	-0.249	2.401	-2.181	0.032
ENSG00000163814	CDCP1	-0.556	0.175	-2.091	0.040
ENSG00000164096	C4orf3	0.212	6.812	1.998	0.049
ENSG00000164125	FAM198B	-0.292	6.396	-2.087	0.040
ENSG00000164129	NPY5R	0.435	2.082	2.237	0.028
ENSG00000164142	FAM160A1	-0.307	1.809	-2.094	0.040
ENSG00000164163	ABCE1	0.212	5.570	2.209	0.030
ENSG00000164211	STARD4	0.261	5.659	2.097	0.039
ENSG00000164258	NDUFS4	0.309	4.901	2.110	0.038
ENSG00000164308	ERAP2	-0.534	3.106	-2.023	0.047
ENSG00000164326	CARTPT	1.184	2.003	2.758	0.007
ENSG00000164466	SFXN1	0.172	6.089	2.280	0.025
ENSG00000164659	KIAA1324L	0.254	6.201	2.141	0.036
ENSG00000164849	GPR146	0.346	0.465	2.031	0.046
ENSG00000164978	NUDT2	0.344	3.135	2.557	0.013
ENSG00000165119	HNRNPK	0.226	7.788	2.386	0.020
ENSG00000165572	KBTBD6	0.216	6.108	2.013	0.048
ENSG00000165730	STOX1	-0.237	5.916	-1.998	0.049
ENSG00000166226	CCT2	0.274	6.014	2.390	0.019
ENSG00000166337	TAF10	-0.229	2.828	-2.315	0.023
ENSG00000166340	TPP1	-0.347	5.229	-2.366	0.021
ENSG00000166407	LMO1	0.594	-0.864	2.180	0.032
ENSG00000166444	ST5	-0.140	4.855	-2.008	0.048
ENSG00000166535	A2ML1	-0.490	3.338	-2.981	0.004
ENSG00000166557	TMED3	0.227	3.820	2.291	0.025
ENSG00000166598	HSP90B1	0.234	8.374	2.550	0.013
ENSG00000166682	TMPRSS5	-0.476	2.152	-2.226	0.029
ENSG00000166783	KIAA0430	-0.257	7.472	-2.536	0.013
ENSG00000166794	PPIB	0.361	4.478	2.999	0.004
ENSG00000166828	SCNN1G	-1.264	-2.547	-2.614	0.011
ENSG00000166839	ANKDD1A	-0.164	4.396	-1.994	0.050
ENSG00000166922	SCG5	0.329	6.458	2.248	0.028
ENSG00000166927	MS4A7	-0.700	2.608	-2.244	0.028

ENSG00000167004	PDIA3	0.314	5.481	2.548	0.013
ENSG00000167085	PHB	0.273	3.797	2.210	0.030
ENSG00000167183	PRR15L	-0.711	-1.763	-2.296	0.024
ENSG00000167258	CDK12	-0.120	6.540	-2.166	0.033
ENSG00000167552	TUBA1A	0.289	8.431	2.354	0.021
ENSG00000167553	TUBA1C	-0.781	4.915	-2.205	0.030
ENSG00000167977	KCTD5	0.268	3.061	2.968	0.004
ENSG00000167978	SRRM2	-0.230	9.174	-2.007	0.048
ENSG00000168004	HRASLS5	-0.385	3.017	-3.007	0.004
ENSG00000168101	NUDT16L1	0.203	2.574	2.000	0.049
ENSG00000168137	SETD5	-0.141	7.474	-2.030	0.046
ENSG00000168374	ARF4	0.207	5.628	2.268	0.026
ENSG00000168405	CMAHP	-0.367	3.299	-2.058	0.043
ENSG00000168434	COG7	-0.171	3.644	-2.348	0.021
ENSG00000168556	ING2	0.193	3.832	2.097	0.039
ENSG00000168594	ADAM29	0.656	0.015	2.578	0.012
ENSG00000168772	CXXC4	0.294	5.445	2.676	0.009
ENSG00000168824	NSG1	0.551	5.461	2.650	0.010
ENSG00000168843	FSTL5	0.354	5.847	2.660	0.010
ENSG00000168961	LGALS9	-0.469	0.996	-2.084	0.041
ENSG00000169189	NSMCE1	0.253	3.697	2.005	0.049
ENSG00000169203	RP11-231C14.4	-0.508	3.049	-2.004	0.049
ENSG00000169288	MRPL1	0.267	3.798	2.554	0.013
ENSG00000169499	PLEKHA2	-0.270	5.877	-1.994	0.050
ENSG00000169679	BUB1	0.629	-0.694	2.326	0.023
ENSG00000169760	NLGN1	0.190	6.773	2.303	0.024
ENSG00000169976	SF3B5	0.258	2.880	2.033	0.046
ENSG00000170017	ALCAM	0.283	7.713	2.302	0.024
ENSG00000170091	HMP19	0.323	8.312	2.051	0.044
ENSG00000170340	B3GNT2	0.478	4.330	3.247	0.002
ENSG00000170370	EMX2	-0.435	4.308	-2.758	0.007
ENSG00000170417	TMEM182	0.420	1.554	2.626	0.010
ENSG00000170419	VSTM2A	0.305	6.834	2.156	0.034
ENSG00000170458	CD14	-0.759	1.616	-2.398	0.019
ENSG00000170542	SERPINB9	-0.228	4.154	-2.174	0.033
ENSG00000170909	OSCAR	-0.578	-0.372	-2.465	0.016
ENSG00000171302	CANT1	0.183	3.846	2.062	0.043
ENSG00000171451	DSEL	0.253	5.883	2.088	0.040
ENSG00000171566	PLRG1	0.180	6.081	2.029	0.046
ENSG00000171724	VAT1L	0.526	6.529	2.149	0.035
ENSG00000171903	CYP4F11	-0.405	2.848	-2.228	0.029
ENSG00000171951	SCG2	0.806	8.156	3.600	0.001
ENSG00000172172	MRPL13	0.260	3.413	2.472	0.016
ENSG00000172247	C1QTNF4	0.529	2.193	2.169	0.033
ENSG00000172346	CSDC2	0.437	3.305	2.007	0.048
ENSG00000172493	AFF1	-0.208	6.298	-2.068	0.042
ENSG00000172578	KLHL6	-0.630	1.601	-2.313	0.023

ENSG00000173065	FAM222B	-0.198	3.940	-2.064	0.042
ENSG00000173083	HPSE	-0.943	0.992	-3.284	0.002
ENSG00000173163	COMMD1	0.226	3.753	2.332	0.022
ENSG00000173369	C1QB	-0.760	3.055	-2.216	0.030
ENSG00000173404	INSM1	0.538	0.837	2.307	0.024
ENSG00000173809	TDRD12	0.527	0.479	2.186	0.032
ENSG00000174109	C16orf91	0.432	1.397	2.842	0.006
ENSG00000174292	TNK1	0.465	0.656	2.692	0.009
ENSG00000174600	CMKLR1	-0.508	2.199	-2.033	0.046
ENSG00000174899	PQLC2L	0.607	-0.585	2.071	0.042
ENSG00000175040	CHST2	0.315	4.323	2.129	0.037
ENSG00000175727	MLXIP	-0.251	5.111	-2.760	0.007
ENSG00000175857	GAPT	-1.611	-0.952	-3.081	0.003
ENSG00000175895	PLEKHF2	-0.291	3.676	-2.289	0.025
ENSG00000176142	TMEM39A	-0.238	3.779	-2.167	0.033
ENSG00000176396	EID2	0.315	3.373	2.272	0.026
ENSG00000176563	CNTD1	-0.297	0.858	-2.363	0.021
ENSG00000176641	RNF152	0.507	5.306	2.817	0.006
ENSG00000176681	LRRC37A	-0.693	3.821	-2.413	0.018
ENSG00000176857	GJA1P1	-0.432	2.786	-2.513	0.014
ENSG00000177283	FZD8	-0.395	2.279	-2.219	0.029
ENSG00000177688	SUMO4	-0.284	1.625	-2.041	0.045
ENSG00000178078	STAP2	0.491	-0.244	2.341	0.022
ENSG00000178104	PDE4DIP	-0.170	8.534	-2.606	0.011
ENSG00000178449	COX14	0.291	3.108	2.142	0.035
ENSG00000178723	GLULP4	-0.780	2.696	-2.166	0.033
ENSG00000178971	CTC1	-0.229	4.755	-3.249	0.002
ENSG00000179038	RP11-157K17.5	-0.369	1.087	-2.063	0.043
ENSG00000179218	CALR	0.240	7.205	2.391	0.019
ENSG00000179277	MEIS3P1	-0.454	2.379	-2.290	0.025
ENSG00000179314	WSCD1	0.238	5.272	2.134	0.036
ENSG00000179611	DGKZP1	-0.715	4.513	-2.052	0.044
ENSG00000179776	CDH5	0.464	2.795	2.006	0.048
ENSG00000179832	MROH1	-0.302	5.099	-2.225	0.029
ENSG00000179886	TIGD5	0.305	1.994	2.229	0.029
ENSG00000179899	PHC1P1	-0.412	3.253	-1.997	0.049
ENSG00000179933	C14orf119	0.323	3.190	2.620	0.011
ENSG00000180263	FGD6	-0.239	6.420	-2.461	0.016
ENSG00000180353	HCLS1	-0.491	3.394	-2.048	0.044
ENSG00000180425	C11orf71	0.244	3.699	2.348	0.021
ENSG00000180458	CTD-3064H18.4	0.477	0.427	2.231	0.029
ENSG00000180537	RNF182	-0.438	4.581	-2.768	0.007
ENSG00000180543	TSPYL5	0.222	5.635	2.053	0.044
ENSG00000180776	ZDHHC20	0.222	6.234	2.004	0.049
ENSG00000181195	PENK	1.692	2.885	2.050	0.044
ENSG00000181220	ZNF746	-0.163	4.468	-2.352	0.021
ENSG00000181626	ANKRD62	-0.761	1.762	-2.307	0.024

ENSG00000181827	RFX7	-0.161	5.968	-2.233	0.028
ENSG00000182257	PRR34	0.410	0.687	2.429	0.018
ENSG00000182405	PGBD4	0.237	3.912	2.003	0.049
ENSG00000182636	NDN	0.454	5.220	2.809	0.006
ENSG00000182774	RPS17	0.297	0.959	2.099	0.039
ENSG00000182973	CNOT10	0.153	4.310	2.562	0.012
ENSG00000183066	WBP2NL	-0.379	2.610	-2.269	0.026
ENSG00000183474	GTF2H2C	-0.429	3.149	-2.161	0.034
ENSG00000183495	EP400	-0.144	6.615	-2.067	0.042
ENSG00000183520	UTP11	0.221	4.479	2.567	0.012
ENSG00000183621	ZNF438	-0.159	4.117	-2.166	0.033
ENSG00000183655	KLHL25	0.292	3.258	2.405	0.019
ENSG00000183763	TRAIP	0.446	1.050	2.338	0.022
ENSG00000183888	C1orf64	0.681	-0.028	2.300	0.024
ENSG00000183889	AC138969.4	-0.737	2.150	-2.190	0.032
ENSG00000184164	CRELD2	0.237	3.495	2.358	0.021
ENSG00000184544	DHRS7C	-1.099	-3.683	-2.332	0.022
ENSG00000184564	SLITRK6	1.471	1.351	2.498	0.015
ENSG00000184635	ZNF93	0.264	2.265	2.109	0.038
ENSG00000184731	FAM110C	-0.470	3.368	-2.557	0.013
ENSG00000184840	TMED9	0.224	4.584	2.354	0.021
ENSG00000184857	TMEM186	0.236	1.401	2.025	0.046
ENSG00000185149	NPY2R	0.590	1.868	2.237	0.028
ENSG00000185480	PARPBP	0.421	0.908	2.429	0.018
ENSG00000185666	SYN3	-0.462	4.701	-2.829	0.006
ENSG00000185737	NRG3	-0.295	5.959	-2.369	0.020
ENSG00000186007	LEMD1	1.005	-2.085	2.543	0.013
ENSG00000186026	ZNF284	0.294	1.830	2.433	0.017
ENSG00000186399	GOLGA8R	-0.376	1.080	-2.050	0.044
ENSG00000186566	GPATCH8	-0.151	6.919	-2.627	0.010
ENSG00000187372	PCDHB13	-0.235	4.145	-2.072	0.042
ENSG00000187416	LHFPL3	0.329	4.275	2.167	0.033
ENSG00000187474	FPR3	-0.604	0.259	-2.035	0.045
ENSG00000187792	ZNF70	-0.210	4.152	-2.075	0.041
ENSG00000187837	HIST1H1C	-0.310	5.293	-2.690	0.009
ENSG00000188013	MEIS3P2	-0.677	1.990	-2.435	0.017
ENSG00000188234	AGAP4	-0.317	2.918	-2.244	0.028
ENSG00000188283	ZNF383	0.124	4.434	2.026	0.046
ENSG00000188290	HES4	0.698	0.831	2.945	0.004
ENSG00000188649	CC2D2B	-0.253	3.436	-2.236	0.028
ENSG00000188672	RHCE	0.410	0.064	2.144	0.035
ENSG00000188783	PRELP	-0.449	4.108	-2.330	0.022
ENSG00000188921	HACD4	-0.212	5.145	-2.080	0.041
ENSG00000188981	MSANTD1	0.377	1.975	2.654	0.010
ENSG00000189159	HN1	0.342	4.449	2.706	0.008
ENSG00000189280	GJB5	-0.700	-0.984	-2.685	0.009
ENSG00000189339	SLC35E2B	-0.321	5.314	-2.359	0.021

ENSG00000189377	CXCL17	-0.662	-0.483	-2.540	0.013
ENSG00000189423	USP32P3	-0.371	3.350	-2.484	0.015
ENSG00000196126	HLA-DRB1	-0.661	3.067	-2.060	0.043
ENSG00000196277	GRM7	0.246	5.516	2.137	0.036
ENSG00000196313	POM121	-0.212	5.279	-2.218	0.030
ENSG00000196428	TSC22D2	-0.196	6.530	-2.012	0.048
ENSG00000196510	ANAPC7	0.171	5.083	2.725	0.008
ENSG00000196655	TRAPPC4	0.252	3.613	2.636	0.010
ENSG00000196705	ZNF431	0.165	5.293	2.558	0.013
ENSG00000196735	HLA-DQA1	-1.085	1.345	-2.259	0.027
ENSG00000196968	FUT11	0.184	4.065	2.002	0.049
ENSG00000197043	ANXA6	0.323	6.566	2.301	0.024
ENSG00000197181	PIWIL2	0.791	1.285	3.093	0.003
ENSG00000197183	NOL4L	-0.201	5.624	-2.045	0.044
ENSG00000197223	C1D	0.280	3.333	2.535	0.013
ENSG00000197345	MRPL21	0.342	3.412	2.091	0.040
ENSG00000197406	DIO3	0.749	-1.076	2.222	0.029
ENSG00000197471	SPN	-0.628	0.765	-2.038	0.045
ENSG00000197479	PCDHB11	-0.452	3.498	-3.926	0.000
ENSG00000197728	RPS26	0.566	2.083	2.108	0.038
ENSG00000197837	HIST4H4	0.361	2.403	2.462	0.016
ENSG00000197903	HIST1H2BK	0.329	1.742	2.154	0.034
ENSG00000198300	PEG3	0.411	9.634	2.430	0.017
ENSG00000198301	SDAD1	0.194	5.839	2.000	0.049
ENSG00000198315	ZKSCAN8	-0.175	6.032	-2.500	0.015
ENSG00000198554	WDHD1	0.237	3.653	2.331	0.022
ENSG00000198837	DENND4B	-0.232	5.284	-2.474	0.016
ENSG00000198890	PRMT6	0.243	3.069	2.038	0.045
ENSG00000198898	CAPZA2	0.247	6.790	2.031	0.046
ENSG00000199691	RN7SKP173	0.445	0.905	2.640	0.010
ENSG00000199914	SNORD114-16	0.848	-2.474	2.072	0.042
ENSG00000200215	SNORD113-6	0.607	-0.836	2.053	0.044
ENSG00000200903	RNU1-42P	1.218	-2.444	2.562	0.012
ENSG00000201240	SNORD114-9	0.654	-1.272	2.283	0.025
ENSG00000201600	RN7SKP124	-0.431	-0.418	-2.024	0.046
ENSG00000201700	SNORD113-3	0.497	0.316	2.604	0.011
ENSG00000202048	SNORD114-20	0.804	-0.793	2.036	0.045
ENSG00000202079	Y_RNA	-0.677	-2.468	-2.420	0.018
ENSG00000202188	SNORD115-31	0.800	-2.141	2.493	0.015
ENSG00000203326	ZNF525	0.230	4.107	2.298	0.024
ENSG00000203327	AC012358.7	-0.556	0.129	-2.571	0.012
ENSG00000204186	ZDBF2	0.242	7.611	2.341	0.022
ENSG00000204220	PFDN6	0.331	3.952	2.225	0.029
ENSG00000204257	HLA-DMA	-0.779	2.084	-2.638	0.010
ENSG00000204287	HLA-DRA	-0.754	4.447	-2.152	0.035
ENSG00000204856	FAM216A	0.317	3.890	2.846	0.006
ENSG00000204990	RP5-842K16.1	-0.526	1.252	-2.308	0.024

ENSG00000205002	AARD	0.780	-0.351	2.681	0.009
ENSG00000205277	MUC12	-0.903	0.636	-2.622	0.011
ENSG00000205302	SNX2	0.172	5.388	2.195	0.031
ENSG00000205488	CALML3-AS1	0.868	-1.058	2.087	0.040
ENSG00000205571	SMN2	-0.795	2.792	-2.151	0.035
ENSG00000205696	ADARB2-AS1	-0.569	-0.110	-2.064	0.042
ENSG00000205746	RP11-1212A22.1	-0.574	1.917	-2.475	0.016
ENSG00000205808	PLPP6	0.174	3.266	2.077	0.041
ENSG00000205981	DNAJC19	0.181	4.639	2.414	0.018
ENSG00000206129	CTD-2008L17.2	-1.298	-1.731	-4.006	0.000
ENSG00000206562	METTL6	0.150	4.710	2.506	0.014
ENSG00000206702	RNU1-11P	1.089	0.246	3.150	0.002
ENSG00000206985	RNU6-198P	-0.758	-1.996	-2.284	0.025
ENSG00000206991	RNU6-610P	-0.695	-1.182	-2.785	0.007
ENSG00000207082	RNU6-171P	-0.454	-0.376	-2.087	0.040
ENSG00000207110	RNU1-106P	0.983	-2.711	2.134	0.036
ENSG00000207370	Y_RNA	-0.563	-1.692	-1.997	0.049
ENSG00000207609	MIR491	0.679	-0.153	2.366	0.021
ENSG00000208018	MIR645	-0.893	-2.373	-2.301	0.024
ENSG00000211450	C11orf31	0.213	2.833	2.127	0.037
ENSG00000211491	MIR320D1	0.383	-0.788	2.084	0.040
ENSG00000211644	IGLV1-51	-0.747	-2.036	-2.582	0.012
ENSG00000213073	RP11-288H12.3	-0.290	1.257	-2.045	0.044
ENSG00000213376	GAPDHP71	-0.575	0.494	-2.755	0.007
ENSG00000213512	GBP7	-0.646	-1.109	-2.404	0.019
ENSG00000213593	TMX2	0.168	4.958	2.036	0.045
ENSG00000213612	FAM220CP	-0.615	-0.031	-2.546	0.013
ENSG00000214106	PAXIP1-AS2	-0.315	3.767	-2.064	0.042
ENSG00000214331	RP11-252A24.2	-0.267	3.846	-2.049	0.044
ENSG00000214753	HNRNPUL2	-0.221	5.058	-2.364	0.021
ENSG00000214826	DDX12P	-0.703	-0.462	-2.275	0.026
ENSG00000214872	SMTNL1	-0.736	-0.418	-3.493	0.001
ENSG00000215018	COL28A1	-0.425	3.111	-2.998	0.004
ENSG00000215380	AC090625.1	-0.666	2.197	-2.552	0.013
ENSG00000215388	ACTG1P3	-0.359	0.824	-2.230	0.029
ENSG00000216192	MIR920	-0.584	-1.950	-2.097	0.039
ENSG00000217555	CKLF	-0.378	-0.017	-2.147	0.035
ENSG00000217929	CICP18	-0.674	-0.561	-2.410	0.018
ENSG00000218757	RP3-472M2.2	-0.373	0.840	-2.488	0.015
ENSG00000220008	LINGO3	0.758	0.961	2.356	0.021
ENSG00000220960	RP1-72A23.1	-0.857	-0.373	-2.589	0.012
ENSG00000221303	SNORA79	0.959	-2.023	2.169	0.033
ENSG00000221882	OR3A2	0.729	0.670	2.426	0.018
ENSG00000221888	OR1C1	1.387	-2.553	2.373	0.020
ENSG00000221926	TRIM16	0.199	2.844	2.188	0.032
ENSG00000221978	CCNL2	-0.153	6.104	-2.400	0.019
ENSG00000221986	MYBPHL	-0.729	-1.201	-2.624	0.010

ENSG00000222009	BTBD19	-0.420	1.593	-2.479	0.015
ENSG00000223152	RNU4-88P	0.779	-2.238	2.179	0.032
ENSG00000223351	ZNF385D-AS2	-0.608	-1.398	-2.047	0.044
ENSG00000223575	RBMX2P3	-0.632	-0.530	-3.083	0.003
ENSG00000223754	AC008073.9	-0.471	-1.044	-2.079	0.041
ENSG00000223756	TSSC2	-0.515	-0.277	-2.271	0.026
ENSG00000223959	AFG3L1P	-0.191	4.295	-2.317	0.023
ENSG00000224083	MTCO1P11	-0.464	0.246	-2.100	0.039
ENSG00000224232	AC110299.5	-0.611	-0.914	-2.200	0.031
ENSG00000224280	AC005014.5	-0.470	0.977	-2.014	0.048
ENSG00000224500	RP11-328K22.1	-0.662	-0.455	-2.441	0.017
ENSG00000224557	HLA-DPB2	1.044	0.621	2.340	0.022
ENSG00000224712	NPIPA3	-0.721	1.170	-2.319	0.023
ENSG00000225208	RP11-107I14.2	0.803	-1.093	2.207	0.030
ENSG00000225222	CHCHD4P5	-0.428	-0.018	-2.093	0.040
ENSG00000225224	RP4-814D15.2	-0.311	0.748	-2.322	0.023
ENSG00000225339	RP11-513I15.6	-0.434	-0.008	-2.450	0.017
ENSG00000225386	AC099754.1	-0.590	-1.695	-2.016	0.047
ENSG00000225648	SBDSP1	0.215	4.358	2.176	0.033
ENSG00000225774	SIRPAP1	-0.870	2.265	-2.719	0.008
ENSG00000225828	FAM229A	-0.489	-0.060	-2.739	0.008
ENSG00000225920	RIMKLBP2	-0.371	1.688	-2.427	0.018
ENSG00000226007	RP11-211N8.2	0.549	-0.735	2.107	0.038
ENSG00000226067	LINC00623	-0.334	0.605	-2.339	0.022
ENSG00000226288	OR52I2	-0.597	-0.927	-2.025	0.046
ENSG00000226375	RP3-395P12.2	-0.487	-0.352	-2.612	0.011
ENSG00000226403	RP11-31F19.1	-0.636	-1.208	-2.309	0.024
ENSG00000226478	UPF3AP1	-0.526	1.573	-2.347	0.022
ENSG00000226491	FTOP1	-0.352	0.520	-2.270	0.026
ENSG00000226564	FTH1P20	-0.575	3.777	-1.994	0.050
ENSG00000226648	PLCG1-AS1	-0.353	0.520	-2.439	0.017
ENSG00000227068	RP11-12A16.3	-0.658	-1.206	-2.288	0.025
ENSG00000227359	AC017074.2	-0.770	-2.034	-2.405	0.019
ENSG00000227374	RP11-109A6.3	0.561	-1.344	2.133	0.036
ENSG00000227878	AC114760.1	-1.162	-1.370	-2.880	0.005
ENSG00000228166	MTND1P11	-1.054	-1.619	-2.371	0.020
ENSG00000228288	PCAT6	-0.564	-1.179	-2.246	0.028
ENSG00000228549	RP11-108M9.3	0.712	-0.754	2.176	0.033
ENSG00000229122	AGBL5-IT1	0.331	0.647	2.099	0.039
ENSG00000229152	ANKRD10-IT1	-0.273	2.697	-2.070	0.042
ENSG00000229191	RP11-168O16.1	0.661	0.114	2.432	0.017
ENSG00000229214	LINC00242	0.383	-0.072	2.408	0.018
ENSG00000229622	MTND5P2	-0.923	-1.564	-2.400	0.019
ENSG00000229728	RP11-314N13.3	-0.472	0.457	-2.271	0.026
ENSG00000229991	AKR1B1P1	-0.556	-0.531	-2.022	0.047
ENSG00000230026	RP11-382D8.5	-0.525	-0.867	-1.996	0.049
ENSG00000230082	PRRT3-AS1	0.476	-0.630	2.170	0.033

ENSG00000230104	AC018712.2	0.937	-1.805	2.650	0.010
ENSG00000230223	ATXN8OS	0.704	-1.045	2.123	0.037
ENSG00000230303	RP11-213G2.2	-0.467	0.301	-2.641	0.010
ENSG00000230359	TPI1P2	-0.331	0.623	-2.045	0.044
ENSG00000230387	RP4-737E23.2	-1.109	-3.132	-2.089	0.040
ENSG00000230490	RP11-141M1.3	0.570	-0.721	2.162	0.034
ENSG00000230806	SRGAP2-AS1	-0.453	0.695	-2.041	0.045
ENSG00000230837	RPL31P2	-0.503	0.375	-2.009	0.048
ENSG00000231181	RP13-15M17.1	-0.770	-1.018	-2.960	0.004
ENSG00000231249	ITPR1-AS1	0.566	-0.491	2.640	0.010
ENSG00000231530	RP11-187A9.3	1.043	-1.388	3.189	0.002
ENSG00000231538	DPP10-AS3	-0.426	1.872	-2.077	0.041
ENSG00000231550	PTCHD3P2	-0.436	-0.456	-2.301	0.024
ENSG00000231784	DBIL5P	0.489	0.583	3.084	0.003
ENSG00000232136	DUXAP7	-0.467	-1.094	-2.020	0.047
ENSG00000232233	LINC02043	-0.755	-0.402	-2.367	0.020
ENSG00000232277	AC104651.3	-0.947	-1.484	-3.235	0.002
ENSG00000232407	RP11-641C17.2	-0.586	-1.762	-2.127	0.037
ENSG00000232448	RP11-416N4.1	0.727	-1.134	3.050	0.003
ENSG00000232466	RP11-337A23.5	-0.593	-0.854	-2.830	0.006
ENSG00000232499	RP11-512F24.1	-0.422	1.252	-2.399	0.019
ENSG00000232528	RP4-673D20.3	-0.446	2.815	-2.689	0.009
ENSG00000232531	AC027612.1	-0.576	0.715	-2.221	0.029
ENSG00000232913	PLCE1-AS2	-0.604	-0.345	-1.997	0.049
ENSG00000232970	POLHP1	-0.634	-0.812	-2.738	0.008
ENSG00000233016	SNHG7	0.344	2.438	2.048	0.044
ENSG00000233225	AC004987.9	-0.749	2.078	-2.651	0.010
ENSG00000233295	FAM90A20P	-0.738	0.200	-2.535	0.013
ENSG00000233416	AC012065.5	-0.388	0.922	-2.225	0.029
ENSG00000233654	AC093388.3	0.354	0.448	2.459	0.016
ENSG00000234056	LINC00463	-1.038	-1.293	-2.835	0.006
ENSG00000234160	RP11-613M10.6	0.440	-0.778	2.089	0.040
ENSG00000234255	AC012370.3	-0.641	-0.747	-2.715	0.008
ENSG00000234428	RP11-666F17.1	-0.588	1.133	-3.226	0.002
ENSG00000234465	PINLYP	0.286	1.527	2.033	0.046
ENSG00000234593	RP4-704D23.1	-0.504	1.763	-2.235	0.028
ENSG00000234685	NUS1P2	0.840	-2.803	2.407	0.018
ENSG00000234758	AC034228.4	-0.546	-0.839	-2.111	0.038
ENSG00000235035	AC007395.3	-0.734	-1.591	-2.191	0.032
ENSG00000235279	RP11-539E19.2	-0.691	-0.168	-2.928	0.004
ENSG00000235316	DUSP8P5	-0.337	1.726	-2.524	0.014
ENSG00000235347	RP1-37M3.8	-0.602	0.607	-3.024	0.003
ENSG00000235374	SSR4P1	0.395	-0.196	2.163	0.034
ENSG00000235532	LINC00402	-0.403	1.985	-2.126	0.037
ENSG00000235613	NSRP1P1	-0.308	3.018	-2.217	0.030
ENSG00000235665	LINC00298	-0.402	0.416	-2.291	0.025
ENSG00000235717	DPP10-AS2	-0.519	1.270	-2.117	0.038

ENSG00000236212	AC009262.2	-1.199	-2.823	-2.309	0.024
ENSG00000236306	LINC01241	-0.793	-1.128	-2.586	0.012
ENSG00000236567	TCF3P1	0.421	-0.389	2.198	0.031
ENSG00000237440	ZNF737	0.214	5.601	2.085	0.040
ENSG00000237481	RP4-803J11.2	-0.642	-1.391	-2.659	0.010
ENSG00000237522	NONOP2	-0.309	1.373	-2.325	0.023
ENSG00000237556	KCND3-AS1	-0.527	0.744	-2.314	0.023
ENSG00000237667	LINC01115	1.443	0.481	2.597	0.011
ENSG00000237887	RPL23AP32	-0.341	2.179	-2.194	0.031
ENSG00000237911	SRP68P3	-0.503	-0.275	-2.086	0.040
ENSG00000238082	AC009948.7	-0.484	2.194	-2.308	0.024
ENSG00000238083	LRRC37A2	-0.665	5.000	-2.615	0.011
ENSG00000238243	OR2W3	0.763	-0.662	2.172	0.033
ENSG00000238386	RNU7-48P	-0.631	-2.348	-2.231	0.029
ENSG00000238741	SCARNA7	0.371	5.216	2.800	0.006
ENSG00000239205	LINC02023	-0.850	-1.779	-2.158	0.034
ENSG00000239213	NCK1-AS1	-0.327	0.790	-2.181	0.032
ENSG00000239473	RPL7P38	0.437	-0.506	2.030	0.046
ENSG00000239602	RP11-449H3.1	-0.635	-1.954	-2.024	0.046
ENSG00000240024	LINC00888	0.291	3.698	2.023	0.047
ENSG00000240731	RP5-890O3.9	-0.323	0.581	-2.231	0.029
ENSG00000240823	RN7SL23P	0.904	-2.520	2.241	0.028
ENSG00000240859	AC093627.10	0.460	0.272	2.265	0.026
ENSG00000241157	RP11-3K24.1	-0.547	-1.273	-2.021	0.047
ENSG00000241399	CD302	-0.313	2.929	-2.108	0.038
ENSG00000241461	RN7SL182P	0.794	-1.875	2.096	0.039
ENSG00000241627	UBQLN4P1	-0.611	1.610	-2.332	0.022
ENSG00000241756	RN7SL83P	-0.810	-1.476	-2.167	0.033
ENSG00000241834	RN7SL149P	-0.983	-2.219	-2.386	0.020
ENSG00000241991	RP11-95I19.2	-0.512	-0.690	-2.104	0.039
ENSG00000242214	MTND3P6	-0.626	-1.241	-2.224	0.029
ENSG00000242330	RN7SL683P	-0.513	-0.769	-2.075	0.041
ENSG00000242689	CNTF	-0.502	0.116	-2.120	0.037
ENSG00000242748	RPL23AP81	-0.603	-1.646	-2.096	0.039
ENSG00000242818	RN7SL846P	0.574	-1.348	2.075	0.041
ENSG00000242970	AC068522.4	0.535	-1.158	2.073	0.042
ENSG00000242979	RPS15AP18	-0.681	-1.551	-2.051	0.044
ENSG00000242992	FTH1P4	-0.401	0.461	-2.177	0.033
ENSG00000243646	IL10RB	-0.383	0.120	-2.242	0.028
ENSG00000244038	DDOST	0.191	4.320	2.114	0.038
ENSG00000244044	RN7SL735P	-0.501	-0.179	-2.019	0.047
ENSG00000244159	RP11-1070A24.1	-0.838	-0.959	-2.276	0.026
ENSG00000244307	RN7SL395P	1.057	-3.416	2.200	0.031
ENSG00000244490	RWDD4P1	-0.393	0.561	-2.107	0.038
ENSG00000244509	APOBEC3C	-0.619	0.396	-2.409	0.018
ENSG00000244682	FCGR2C	-0.752	0.623	-2.181	0.032
ENSG00000246174	KCTD21-AS1	0.593	0.733	2.752	0.007

ENSG00000246225	RP11-17A1.3	0.596	-0.668	2.294	0.025
ENSG00000246528	RP11-159H10.3	0.471	-0.815	2.107	0.038
ENSG00000246596	RP11-1277A3.2	-0.221	3.904	-2.046	0.044
ENSG00000246662	LINC00535	0.284	1.682	2.426	0.018
ENSG00000246792	RP11-68L18.1	0.383	0.689	2.044	0.044
ENSG00000246851	RP11-544A12.8	-0.334	1.405	-2.018	0.047
ENSG00000247911	HMGN1P12	-0.927	-1.403	-3.629	0.001
ENSG00000248125	CTB-73N10.1	-0.639	-1.316	-2.052	0.044
ENSG00000248174	LINC02268	1.220	-1.340	2.129	0.036
ENSG00000248197	LINC00290	0.536	-1.138	2.067	0.042
ENSG00000248249	RP11-598O12.1	-0.589	1.292	-2.407	0.019
ENSG00000248305	RP11-310A13.2	-0.718	-0.466	-2.649	0.010
ENSG00000248394	FOSL1P1	-0.518	-0.677	-2.079	0.041
ENSG00000248429	RP11-597D13.9	-0.595	2.392	-2.849	0.006
ENSG00000248485	PCP4L1	0.664	4.385	2.239	0.028
ENSG00000248627	RP11-428L21.1	0.583	0.302	2.845	0.006
ENSG00000248863	RP11-83A24.1	-0.329	0.948	-2.200	0.031
ENSG00000248923	MTND5P11	-0.516	2.517	-2.020	0.047
ENSG00000249035	CTB-113P19.1	0.612	-1.084	2.023	0.047
ENSG00000249096	LINC02362	-0.620	-0.212	-2.835	0.006
ENSG00000249138	SLED1	-1.183	-1.388	-3.250	0.002
ENSG00000249216	RP11-227F19.5	-0.381	0.125	-2.397	0.019
ENSG00000249278	RP11-688H10.1	-1.271	-2.287	-3.253	0.002
ENSG00000249306	LINC01411	-0.990	2.439	-3.157	0.002
ENSG00000249514	CTD-2266L18.1	-1.223	-1.733	-3.070	0.003
ENSG00000249825	CTD-2201I18.1	-0.469	0.599	-2.839	0.006
ENSG00000249839	AC011330.5	-0.655	2.739	-2.139	0.036
ENSG00000249846	LINC02021	-0.435	0.754	-2.957	0.004
ENSG00000249853	HS3ST5	0.421	3.241	2.508	0.014
ENSG00000249859	PVT1	-0.445	1.717	-2.015	0.047
ENSG00000249917	LINC00536	-1.124	-3.044	-2.051	0.044
ENSG00000250033	SLC7A11-AS1	-0.517	2.827	-2.174	0.033
ENSG00000250254	PTTG2	-0.535	0.182	-2.617	0.011
ENSG00000250433	CLSTN2-AS1	-0.616	1.261	-2.160	0.034
ENSG00000250486	FAM218A	0.252	2.665	2.187	0.032
ENSG00000250541	RP11-302F12.10	-0.627	-1.394	-2.060	0.043
ENSG00000250761	RP11-711G10.1	-0.479	0.478	-2.310	0.024
ENSG00000251073	NUDT19P5	-0.794	-0.759	-2.100	0.039
ENSG00000251126	RP11-119H12.6	-0.638	-0.427	-2.719	0.008
ENSG00000251136	RP11-37B2.1	-0.171	5.379	-2.185	0.032
ENSG00000251165	F11-AS1	-0.538	0.054	-2.614	0.011
ENSG00000251186	RP11-689P11.3	-0.645	-1.360	-2.483	0.015
ENSG00000251354	RP11-697N18.1	-0.523	0.062	-2.310	0.024
ENSG00000251372	LINC00499	-1.267	1.533	-3.142	0.002
ENSG00000251408	RP11-586D19.2	0.446	1.077	2.518	0.014
ENSG00000251441	RTEL1P1	-0.513	0.039	-2.392	0.019
ENSG00000251460	RP11-1258F18.1	-0.910	-1.750	-2.583	0.012

ENSG00000251562	MALAT1	-0.218	14.006	-2.097	0.039
ENSG00000251580	LINC02482	0.375	0.346	2.212	0.030
ENSG00000252338	RNU6-503P	-0.824	-1.851	-2.119	0.037
ENSG00000252835	SCARNA21	0.284	3.944	2.032	0.046
ENSG00000253167	KIAA0196-AS1	-0.470	-0.275	-2.308	0.024
ENSG00000253175	RP11-267M23.6	-0.798	-2.226	-2.436	0.017
ENSG00000253519	AC106801.1	-0.773	-1.081	-2.615	0.011
ENSG00000253532	CTD-2340D6.1	-0.772	-1.533	-2.589	0.012
ENSG00000253663	NPM1P52	-1.019	-2.034	-2.301	0.024
ENSG00000253764	RP11-439C15.4	-0.342	2.287	-1.996	0.050
ENSG00000253807	LINC01170	0.638	-0.375	2.174	0.033
ENSG00000253875	RP11-16P20.3	-0.433	-0.530	-2.118	0.037
ENSG00000253908	RP11-252I14.2	-0.638	-0.069	-2.682	0.009
ENSG00000253910	PCDHGB2	-0.292	3.892	-2.333	0.022
ENSG00000253961	RP11-363L24.3	-0.744	-1.349	-2.605	0.011
ENSG00000254041	RP11-574O7.1	1.033	-3.219	2.153	0.035
ENSG00000254050	RP11-577N1.1	-0.781	-0.159	-2.542	0.013
ENSG00000254087	LYN	-0.419	3.452	-2.385	0.020
ENSG00000254165	RP11-503E24.2	-0.343	1.371	-2.015	0.047
ENSG00000254206	NPIPB11	-0.560	2.420	-2.248	0.028
ENSG00000254272	RP11-382J24.2	-0.376	0.606	-2.131	0.036
ENSG00000254297	CTC-286N12.1	-0.648	-1.304	-2.041	0.045
ENSG00000254302	RP11-431M3.1	-0.826	-1.426	-2.268	0.026
ENSG00000254484	CTA-797E19.1	-0.442	-0.423	-2.020	0.047
ENSG00000254528	RP11-728F11.4	-0.531	0.677	-2.022	0.047
ENSG00000254664	CTD-2560E9.3	0.778	-1.217	2.450	0.017
ENSG00000254681	PKD1P5	-0.764	2.309	-2.393	0.019
ENSG00000254827	SLC22A18AS	0.902	-3.430	2.008	0.048
ENSG00000254863	RP11-831A10.2	0.985	-1.575	2.160	0.034
ENSG00000254901	BORCS8	0.370	2.837	3.032	0.003
ENSG00000254986	DPP3	0.236	2.528	2.036	0.045
ENSG00000255040	RP11-677N16.1	-0.367	0.632	-2.051	0.044
ENSG00000255153	TOLLIP-AS1	0.622	-1.560	2.079	0.041
ENSG00000255321	RP11-91I20.4	-0.555	-0.800	-2.313	0.023
ENSG00000255545	RP11-627G23.1	-0.685	2.619	-3.530	0.001
ENSG00000255561	FDXACB1	0.326	0.921	2.096	0.039
ENSG00000256001	RP11-575F12.2	0.761	-1.335	2.532	0.013
ENSG00000256278	RP11-182J1.5	0.436	-0.733	2.083	0.041
ENSG00000256340	ABCC6P1	-0.726	-0.810	-2.676	0.009
ENSG00000256473	RP11-437F6.1	-0.616	-0.788	-2.559	0.012
ENSG00000256537	SMIM10L1	0.273	5.287	2.561	0.012
ENSG00000257835	RP1-97G4.1	-0.380	1.777	-2.150	0.035
ENSG00000257943	RP11-167N24.3	-1.403	-2.583	-4.205	0.000
ENSG00000258199	RP11-977G19.5	-0.282	0.998	-2.154	0.034
ENSG00000258216	RP11-654D12.2	0.839	-2.621	2.726	0.008
ENSG00000258227	CLEC5A	-1.270	0.232	-2.539	0.013
ENSG00000258274	RP11-887P2.5	-0.348	1.789	-2.068	0.042

ENSG00000258365	RP11-1105G2.3	-0.409	-0.199	-2.038	0.045
ENSG00000258399	MEG8	0.379	2.595	2.380	0.020
ENSG00000258733	LINC02328	-0.439	-0.095	-2.196	0.031
ENSG00000258891	RP5-1021I20.5	-0.350	0.855	-2.219	0.029
ENSG00000258912	LINC02316	-0.942	-2.122	-2.933	0.004
ENSG00000258930	RP5-1163L11.2	-0.353	0.979	-2.147	0.035
ENSG00000259045	MTCO1P2	0.708	0.270	2.133	0.036
ENSG00000259151	CAP2P1	-0.308	0.861	-2.102	0.039
ENSG00000259161	CTD-2017C7.3	-0.463	-0.259	-2.014	0.048
ENSG00000259216	RP11-227D13.2	-0.816	1.434	-2.215	0.030
ENSG00000259315	ACTG1P17	0.578	-1.575	2.030	0.046
ENSG00000259347	RP11-798K3.2	-0.537	-0.487	-2.228	0.029
ENSG00000259363	CTD-2054N24.2	0.622	-0.259	2.016	0.047
ENSG00000259380	RP11-346D14.1	-0.629	-0.118	-2.946	0.004
ENSG00000259425	RP11-566K19.5	0.408	0.361	2.270	0.026
ENSG00000259456	ADNP-AS1	0.316	0.545	2.238	0.028
ENSG00000259529	RP11-468E2.4	-0.598	-1.633	-2.320	0.023
ENSG00000259954	IL21R-AS1	1.040	-1.007	2.205	0.030
ENSG00000260271	RP1-45N11.1	-0.626	-1.584	-2.023	0.047
ENSG00000260288	RP11-24M17.4	0.756	-2.070	2.191	0.032
ENSG00000260305	NTRK3-AS1	0.569	-0.079	2.504	0.014
ENSG00000260433	LINC01917	0.807	-1.687	2.180	0.032
ENSG00000260455	NBAT1	0.453	0.663	2.654	0.010
ENSG00000260469	C15orf59-AS1	0.810	-1.620	2.061	0.043
ENSG00000260653	RP11-114G11.5	1.880	-2.198	2.496	0.015
ENSG00000260733	RP11-264L1.4	-0.547	0.346	-2.687	0.009
ENSG00000260792	LINC02280	0.700	-1.254	1.996	0.049
ENSG00000260793	RP5-882C2.2	-0.364	0.813	-2.140	0.036
ENSG00000260816	RP11-319G9.3	-0.495	2.378	-2.528	0.014
ENSG00000260830	RP11-524O1.4	-1.218	-2.662	-3.252	0.002
ENSG00000260896	PRCAT47	0.527	-0.032	2.897	0.005
ENSG00000261087	KB-1460A1.5	-0.249	5.082	-2.455	0.016
ENSG00000261220	RP11-629O1.2	-0.596	-0.364	-2.097	0.039
ENSG00000261390	MAFTRR	-0.621	-0.450	-2.079	0.041
ENSG00000261461	UBE2MP1	-0.610	1.485	-2.162	0.034
ENSG00000261634	RP11-352D13.6	1.219	-1.678	2.786	0.007
ENSG00000261654	RP11-96K19.4	-0.463	0.925	-2.325	0.023
ENSG00000262096	PCDHB19P	-0.322	2.179	-2.397	0.019
ENSG00000262668	AJ003147.9	1.105	-2.739	2.690	0.009
ENSG00000263400	TMEM220-AS1	-0.646	-0.048	-2.793	0.007
ENSG00000263426	RN7SL471P	0.738	4.212	2.365	0.021
ENSG00000263667	RP3-416J7.4	-1.306	-2.156	-2.011	0.048
ENSG00000263786	RP11-649A18.4	0.717	-1.422	2.690	0.009
ENSG00000264364	DYNLL2	0.236	7.640	2.108	0.038
ENSG00000264515	CTC-525D6.1	-0.451	1.960	-2.403	0.019
ENSG00000264617	AC144838.3	1.173	-2.667	2.165	0.034
ENSG00000264707	L3MBTL4-AS1	-0.372	1.360	-2.327	0.023

ENSG00000264869	RP11-526H11.1	-0.958	-2.050	-2.051	0.044
ENSG00000264937	AC100830.5	-0.717	-1.103	-2.879	0.005
ENSG00000265148	TSPOAP1-AS1	-0.425	0.131	-3.057	0.003
ENSG00000265203	RBP3	-0.670	-0.383	-2.688	0.009
ENSG00000265279	CLUHP6	-0.701	-1.707	-2.359	0.021
ENSG00000265337	RP11-466A19.5	-0.730	-1.369	-2.479	0.015
ENSG00000266397	RP11-737O24.1	-0.530	-0.787	-2.043	0.044
ENSG00000267107	PCAT19	0.625	0.011	2.315	0.023
ENSG00000267128	RP11-449J21.5	-0.288	1.707	-2.069	0.042
ENSG00000267179	CTD-2006C1.13	0.554	-1.782	2.101	0.039
ENSG00000267219	AC010504.2	-0.482	-0.362	-2.636	0.010
ENSG00000267508	ZNF285	0.177	3.900	2.447	0.017
ENSG00000267636	AC008992.1	-0.884	-2.294	-2.114	0.038
ENSG00000267733	RP11-64C12.3	-1.214	-1.152	-3.069	0.003
ENSG00000267766	RP11-299P2.1	-0.695	-0.894	-2.797	0.007
ENSG00000268055	AC067969.2	-0.947	-0.941	-2.445	0.017
ENSG00000268278	RP11-420K14.1	-0.499	-0.803	-2.006	0.048
ENSG00000268654	MIMT1	0.408	1.004	2.153	0.034
ENSG00000268751	SCGB1B2P	0.620	-1.228	2.045	0.044
ENSG00000268756	AC104534.2	-0.698	-1.949	-2.033	0.046
ENSG00000269473	CTD-2619J13.19	0.405	0.902	2.177	0.033
ENSG00000269570	CMB9-55A18.1	-0.412	0.573	-2.678	0.009
ENSG00000269707	RP11-13J10.1	0.394	0.209	2.096	0.039
ENSG00000269836	CTD-3032J10.4	-0.771	-1.672	-2.833	0.006
ENSG00000269845	RP11-420K14.6	-0.436	0.696	-2.484	0.015
ENSG00000269935	RP11-482M8.3	-0.597	0.827	-2.348	0.021
ENSG00000269976	RP11-130L8.2	-0.915	-2.438	-2.009	0.048
ENSG00000270139	AP001172.3	-0.494	0.681	-2.281	0.025
ENSG00000270324	RP11-180P8.4	0.708	-1.270	2.088	0.040
ENSG00000270578	RP11-712B9.5	-0.504	-0.521	-2.484	0.015
ENSG00000270728	RP4-657E11.10	-0.299	1.306	-2.233	0.028
ENSG00000270742	RP4-802A10.1	-0.510	0.783	-2.210	0.030
ENSG00000270776	RP11-17G11.1	-0.602	-1.538	-2.017	0.047
ENSG00000270948	RP11-460N20.7	-0.564	-0.344	-2.371	0.020
ENSG00000271075	RP11-589M4.4	-0.611	-0.437	-2.444	0.017
ENSG00000271167	LINC01109	0.560	-0.885	2.031	0.046
ENSG00000271392	RP1-161P9.5	0.653	-1.601	2.662	0.009
ENSG00000271538	LINC02427	0.431	1.404	2.335	0.022
ENSG00000271780	RP11-1017G21.5	0.330	1.640	2.550	0.013
ENSG00000271992	RP11-42O15.3	1.058	-2.813	2.462	0.016
ENSG00000272024	RP11-546K22.3	0.610	-1.232	2.212	0.030
ENSG00000272138	LINC01607	0.896	-1.524	2.701	0.009
ENSG00000272211	RP11-347P5.1	-0.569	0.021	-2.322	0.023
ENSG00000272391	POM121C	-0.195	4.982	-2.181	0.032
ENSG00000272460	SNORD115-40	0.555	-1.031	2.055	0.043
ENSG00000272483	RP11-169K17.3	-0.718	-1.369	-2.280	0.025
ENSG00000272660	RP11-145M9.6	-0.734	-1.676	-2.341	0.022

ENSG00000272667	RP11-395A13.2	-0.376	0.201	-2.009	0.048
ENSG00000272693	RP11-479O9.4	-0.424	-0.152	-2.561	0.012
ENSG00000272732	RP5-1159O4.2	-0.359	1.736	-2.223	0.029
ENSG00000272990	RP11-305K5.1	-0.381	0.941	-2.117	0.038
ENSG00000273008	RP11-351D16.3	0.629	-0.600	2.825	0.006
ENSG00000273018	CTD-2303H24.2	-0.782	2.739	-2.081	0.041
ENSG00000273061	CDC37L1-AS1	0.506	0.161	2.382	0.020
ENSG00000273139	XXbac-B444P24.1	0.681	-1.074	2.890	0.005
ENSG00000273198	GS1-304P7.3	0.837	-1.843	2.233	0.029
ENSG00000273293	RP11-445N20.3	0.546	-1.560	2.237	0.028
ENSG00000273855	RP11-133K1.12	0.750	-1.372	2.298	0.024
ENSG00000274114	CTC-281F24.3	0.643	1.039	2.171	0.033
ENSG00000274198	RP11-579A4.1	-0.712	-2.055	-2.201	0.031
ENSG00000274322	RP11-314N13.10	-0.697	-0.494	-2.356	0.021
ENSG00000274356	RP11-216L13.21	0.926	-2.841	2.043	0.045
ENSG00000274554	RP11-240G22.5	-0.319	1.204	-2.084	0.041
ENSG00000274659	LINC02371	0.769	-1.606	2.453	0.016
ENSG00000274767	AC131056.3	-1.449	-1.137	-2.973	0.004
ENSG00000274929	RP11-165D7.5	0.695	-1.018	2.730	0.008
ENSG00000275023	MLLT6	-0.224	6.827	-2.135	0.036
ENSG00000275131	CH17-472G23.4	-0.282	7.081	-2.406	0.019
ENSG00000275160	RP11-187C18.3	0.381	2.323	2.115	0.038
ENSG00000275846	RP11-457M11.7	-0.499	-0.245	-2.200	0.031
ENSG00000275963	RP11-180M15.6	-0.377	1.222	-2.676	0.009
ENSG00000276107	CTD-2033D15.2	-0.881	-0.996	-2.517	0.014
ENSG00000276269	RP11-403A3.2	-1.038	-2.240	-2.009	0.048
ENSG00000276591	GXYLT1P5	-0.627	1.988	-2.004	0.049
ENSG00000276704	RP11-128N14.4	-0.692	-1.268	-2.514	0.014
ENSG00000276710	CSPG4P10	-0.391	1.961	-2.286	0.025
ENSG00000276786	WI2-8325B5.1	0.732	-1.631	2.172	0.033
ENSG00000276853	RP11-305O6.4	-0.469	-0.016	-2.406	0.019
ENSG00000277170	RP11-295D4.4	-0.656	-1.210	-2.460	0.016
ENSG00000277795	AC005752.10	-0.436	0.301	-1.992	0.050
ENSG00000278600	RP11-81A1.6	0.192	2.926	2.215	0.030
ENSG00000278638	CTC-268N12.2	-0.576	-1.112	-2.011	0.048
ENSG00000278925	AC005753.1	-0.733	-0.212	-2.084	0.041
ENSG00000278943	RP11-142A22.3	-0.743	2.173	-2.065	0.042
ENSG00000279012	OR51B2	-1.378	-2.634	-3.005	0.004
ENSG00000279021	RP11-391L3.4	-0.347	1.402	-2.154	0.034
ENSG00000279075	RP11-480D4.7	1.172	-2.095	2.650	0.010
ENSG00000279085	CTA-243E7.4	-0.416	0.356	-2.014	0.048
ENSG00000279254	RP11-536C12.1	0.600	-0.917	2.010	0.048
ENSG00000279314	RP1-257C22.2	-0.661	-0.355	-2.161	0.034
ENSG00000279344	RP11-44F14.7	-0.405	0.672	-2.509	0.014
ENSG00000279415	RP11-1102P22.1	-0.664	-0.217	-2.993	0.004
ENSG00000279469	RP11-215P8.2	0.539	-0.931	2.205	0.030
ENSG00000279499	RP11-490N5.3	-0.686	-0.467	-2.606	0.011

ENSG00000279521	RP11-314P15.1	-1.338	-1.843	-2.560	0.012
ENSG00000279565	RP4-570O12.3	0.500	-0.455	2.127	0.037
ENSG00000279584	AC005593.2	0.968	-2.396	2.761	0.007
ENSG00000279785	GS1-124K5.13	0.904	-1.016	3.369	0.001
ENSG00000279875	RP11-474C8.7	-0.581	-0.809	-2.039	0.045
ENSG00000279943	FLJ38576	0.310	-0.046	1.999	0.049
ENSG00000280113	AL357140.1	-0.538	-1.608	-2.063	0.042
ENSG00000280190	RP11-319G9.1	-0.495	1.261	-1.992	0.050
ENSG00000280222	RP11-174G17.3	-0.546	2.563	-2.003	0.049
ENSG00000280239	CTB-50L17.8	-0.360	-0.029	-2.086	0.040
ENSG00000280350	LINC01726	-0.716	-0.668	-2.471	0.016
ENSG00000280353	CTC-453G23.7	0.810	-1.589	2.315	0.023
ENSG00000280423	RP11-299J5.1	-1.092	-1.654	-2.983	0.004
ENSG00000280432	AP000962.1	-0.530	1.519	-2.826	0.006
ENSG00000280485	RP11-437J2.4	-0.547	0.692	-3.347	0.001
ENSG00000280660	RP11-437J2.3	-0.318	5.016	-2.320	0.023
ENSG00000281021	RP1-12G14.9	-0.343	0.044	-2.077	0.041
ENSG00000281344	HELLPAR	-0.349	2.382	-2.208	0.030
ENSG00000281772	RP11-567F11.2	-0.588	-1.668	-2.223	0.029
ENSG00000282057	RP4-621F18.2	-0.544	1.283	-2.852	0.006
ENSG00000282556	RP11-304C12.5	0.571	-1.166	2.093	0.040
ENSG00000282608	ADORA3	-0.832	1.269	-2.420	0.018
ENSG00000282943	CTC-490G23.6	-0.534	0.230	-2.262	0.027
ENSG00000283052	LINC02456	-0.924	-1.753	-2.091	0.040
ENSG00000283162	RP11-403F21.4	0.458	1.190	2.454	0.016
ENSG00000283217	RP11-472I20.3	0.764	-1.570	2.075	0.041
ENSG00000283897	#N/A	0.695	-0.072	2.548	0.013
ENSG00000284770	TBCE	-0.179	3.337	-2.054	0.043
ENSG00000285041	#N/A	1.304	-3.161	2.103	0.039
ENSG00000285219	#N/A	0.251	4.305	2.202	0.031
ENSG00000285230	RALY-AS1	-0.372	1.387	-2.338	0.022
ENSG00000285556	#N/A	-0.607	-1.139	-2.139	0.036
ENSG00000285591	#N/A	-0.718	-1.132	-2.050	0.044
ENSG00000285618	#N/A	-0.665	-0.046	-2.564	0.012
ENSG00000285639	#N/A	-0.913	-1.241	-2.135	0.036
ENSG00000285684	#N/A	-0.453	1.239	-2.133	0.036
ENSG00000285813	#N/A	-0.316	2.609	-2.212	0.030
ENSG00000285850	#N/A	-0.419	1.740	-1.999	0.049

dlPFC

gene_id	gene_name	logFC	AveExpr	t	P.Value
ENSG00000000419	DPM1	0.365	4.141	3.136	0.002
ENSG00000001461	NIPAL3	0.263	7.114	3.203	0.002
ENSG00000001561	ENPP4	0.262	6.419	2.474	0.015
ENSG00000001617	SEMA3F	0.504	0.220	2.410	0.018
ENSG00000003056	M6PR	0.225	5.417	2.686	0.009
ENSG00000004142	POLDIP2	0.152	4.899	2.255	0.027
ENSG00000004700	RECQL	0.280	4.232	3.060	0.003
ENSG00000004766	VPS50	0.352	6.532	2.828	0.006
ENSG00000004776	HSPB6	-0.853	-0.761	-3.094	0.003
ENSG00000004779	NDUFAB1	0.283	5.253	2.244	0.028
ENSG00000004866	ST7	-0.113	4.757	-2.062	0.042
ENSG00000005020	SKAP2	0.231	5.635	2.277	0.025
ENSG00000005059	CCDC109B	0.635	1.943	3.735	0.000
ENSG00000005175	RPAP3	0.210	5.648	2.405	0.018
ENSG00000005194	CIAPIN1	0.275	4.276	2.236	0.028
ENSG00000005339	CREBBP	-0.240	6.846	-3.004	0.004
ENSG00000005381	MPO	-0.412	0.230	-2.047	0.044
ENSG00000005421	PON1	-0.460	0.262	-2.701	0.008
ENSG00000005448	WDR54	0.310	2.850	2.627	0.010
ENSG00000005700	IBTK	0.162	6.734	2.162	0.034
ENSG00000006007	GDE1	0.379	5.736	4.029	0.000
ENSG00000006071	ABCC8	-0.218	5.217	-2.181	0.032
ENSG00000006128	TAC1	0.875	2.812	3.418	0.001
ENSG00000006194	ZNF263	0.160	4.589	2.884	0.005
ENSG00000006459	KDM7A	0.152	5.997	2.193	0.031
ENSG00000006534	ALDH3B1	-0.335	0.442	-2.157	0.034
ENSG00000006625	GGCT	0.592	4.057	4.108	0.000
ENSG00000006712	PAF1	0.282	4.945	3.311	0.001
ENSG00000006715	VPS41	0.254	7.352	2.213	0.030
ENSG00000007062	PROM1	0.516	2.815	3.061	0.003
ENSG00000007080	CCDC124	0.260	3.387	2.177	0.032
ENSG00000007202	KIAA0100	0.156	7.241	2.612	0.011
ENSG00000007264	MATK	0.405	4.236	2.916	0.005
ENSG00000007372	PAX6	-0.313	4.315	-2.337	0.022
ENSG00000007516	BAIAP3	-0.358	5.316	-2.227	0.029
ENSG00000007908	SELE	-0.973	-0.672	-2.809	0.006
ENSG00000007923	DNAJC11	0.238	5.046	2.904	0.005
ENSG00000008018	PSMB1	0.249	5.531	2.481	0.015
ENSG00000008083	JARID2	-0.115	5.194	-2.319	0.023
ENSG00000008324	SS18L2	0.379	2.328	2.853	0.005
ENSG00000008441	NFIX	-0.251	6.815	-2.544	0.013
ENSG00000008869	HEATR5B	0.161	6.948	2.029	0.046
ENSG00000008952	SEC62	0.165	8.312	2.073	0.041
ENSG00000008988	RPS20	0.348	6.665	2.307	0.024
ENSG00000009307	CSDE1	0.238	9.070	2.530	0.013

ENSG00000009335	UBE3C	0.172	6.763	2.601	0.011
ENSG00000009413	REV3L	-0.125	7.395	-2.973	0.004
ENSG00000009724	MASP2	-0.336	2.644	-3.091	0.003
ENSG00000009830	POMT2	-0.178	3.691	-2.629	0.010
ENSG00000009844	VTA1	0.324	6.355	3.073	0.003
ENSG00000010219	DYRK4	0.159	3.399	2.421	0.018
ENSG00000010256	UQCRC1	0.324	5.541	2.240	0.028
ENSG00000010270	STARD3NL	0.330	5.225	3.108	0.003
ENSG00000010295	IFFO1	0.132	4.582	2.143	0.035
ENSG00000010379	SLC6A13	0.604	1.861	3.338	0.001
ENSG00000011009	LYPLA2	0.235	2.081	2.455	0.016
ENSG00000011243	AKAP8L	0.246	4.824	3.187	0.002
ENSG00000011260	UTP18	0.323	4.635	2.221	0.029
ENSG00000011332	DPF1	0.263	4.130	2.718	0.008
ENSG00000011376	LARS2	0.221	5.017	2.656	0.010
ENSG00000011465	DCN	0.423	4.478	3.032	0.003
ENSG00000011485	PPP5C	0.191	5.471	2.610	0.011
ENSG00000011638	TMEM159	0.293	1.542	2.211	0.030
ENSG00000012232	EXTL3	0.124	6.181	2.032	0.045
ENSG00000013275	PSMC4	0.291	5.392	2.640	0.010
ENSG00000013374	NUB1	0.201	5.632	2.439	0.017
ENSG00000013561	RNF14	0.317	6.509	3.200	0.002
ENSG00000014123	UFL1	0.172	5.896	2.082	0.040
ENSG00000014164	ZC3H3	-0.309	1.984	-2.193	0.031
ENSG00000014641	MDH1	0.308	7.915	2.204	0.030
ENSG00000014824	SLC30A9	0.268	7.618	2.379	0.020
ENSG00000015171	ZMYND11	0.143	8.019	2.298	0.024
ENSG00000015479	MATR3	-0.272	5.262	-2.260	0.026
ENSG00000015592	STMN4	0.288	6.407	2.880	0.005
ENSG00000016391	CHDH	-0.294	3.891	-2.172	0.033
ENSG00000016864	GLT8D1	0.169	4.450	2.712	0.008
ENSG00000017797	RALBP1	0.155	7.378	2.202	0.031
ENSG00000018189	RUFY3	0.299	8.982	2.472	0.016
ENSG00000018510	AGPS	0.261	6.163	2.785	0.007
ENSG00000019186	CYP24A1	1.000	-0.365	2.392	0.019
ENSG00000020129	NCDN	-0.214	7.852	-2.038	0.045
ENSG00000021461	CYP3A43	-0.583	-0.518	-2.788	0.007
ENSG00000021574	SPAST	0.256	6.101	3.108	0.003
ENSG00000022277	RTFDC1	0.358	6.006	3.070	0.003
ENSG00000023041	ZDHHC6	0.223	4.425	2.864	0.005
ENSG00000023287	RB1CC1	0.242	8.748	2.716	0.008
ENSG00000023572	GLRX2	0.244	5.521	2.518	0.014
ENSG00000023697	DERA	0.253	3.126	2.155	0.034
ENSG00000023734	STRAP	0.299	6.098	2.518	0.014
ENSG00000024526	DEPDC1	0.276	0.704	2.112	0.038
ENSG00000025156	HSF2	0.232	4.661	2.116	0.037
ENSG00000028310	BRD9	0.124	5.162	2.339	0.022

ENSG00000029153	ARNTL2	0.246	5.609	2.595	0.011
ENSG00000029363	BCLAF1	0.183	7.808	2.392	0.019
ENSG00000029364	SLC39A9	0.179	6.386	2.567	0.012
ENSG00000029725	RABEP1	0.224	8.198	2.704	0.008
ENSG00000031691	CENPQ	0.411	2.134	2.493	0.015
ENSG00000031698	SARS	0.300	7.418	2.848	0.006
ENSG00000032219	ARID4A	0.155	7.076	2.209	0.030
ENSG00000032389	TSSC1	0.210	4.316	2.129	0.036
ENSG00000033178	UBA6	0.181	6.711	2.545	0.013
ENSG00000033867	SLC4A7	0.283	7.211	2.515	0.014
ENSG00000034510	TMSB10	0.347	8.701	2.673	0.009
ENSG00000035141	FAM136A	0.340	3.766	2.831	0.006
ENSG00000035681	NSMAF	-0.163	5.208	-3.196	0.002
ENSG00000035687	ADSS	0.321	5.836	2.521	0.014
ENSG00000035928	RFC1	0.187	6.057	2.191	0.031
ENSG00000036054	TBC1D23	0.270	5.692	3.274	0.002
ENSG00000036549	ZZZ3	0.134	6.822	2.146	0.035
ENSG00000037241	RPL26L1	0.538	2.299	2.488	0.015
ENSG00000037280	FLT4	0.461	-0.264	2.277	0.025
ENSG00000037749	MFAP3	0.221	4.717	3.830	0.000
ENSG00000037897	METTL1	0.356	0.698	2.547	0.013
ENSG00000038002	AGA	0.419	3.225	2.825	0.006
ENSG00000038295	TLL1	0.320	3.501	2.879	0.005
ENSG00000039123	SKIV2L2	0.254	6.469	2.634	0.010
ENSG00000040633	PHF23	0.188	4.108	2.450	0.016
ENSG00000041357	PSMA4	0.398	5.753	3.134	0.002
ENSG00000041802	LSG1	0.173	5.235	2.025	0.046
ENSG00000042429	MED17	-0.308	3.079	-2.565	0.012
ENSG00000042753	AP2S1	0.375	3.816	2.520	0.014
ENSG00000042781	USH2A	-0.251	2.261	-2.836	0.006
ENSG00000043462	LCP2	0.218	3.113	2.066	0.042
ENSG00000044115	CTNNA1	0.272	6.715	2.694	0.009
ENSG00000044524	EPHA3	0.232	4.503	2.013	0.047
ENSG00000044574	HSPA5	0.225	6.881	2.129	0.036
ENSG00000047315	POLR2B	0.272	7.256	2.418	0.018
ENSG00000047410	TPR	0.154	8.033	2.152	0.034
ENSG00000047621	C12orf4	0.241	5.697	2.714	0.008
ENSG00000047936	ROS1	-0.585	3.975	-2.807	0.006
ENSG00000048140	TSPAN17	0.362	3.819	3.995	0.000
ENSG00000048162	NOP16	0.209	2.174	2.437	0.017
ENSG00000048342	CC2D2A	-0.171	5.231	-2.272	0.026
ENSG00000048392	RRM2B	0.293	6.172	2.693	0.009
ENSG00000048544	MRPS10	0.310	5.084	3.185	0.002
ENSG00000048545	GUCA1A	0.855	-0.813	3.069	0.003
ENSG00000049167	ERCC8	0.161	3.499	2.128	0.036
ENSG00000049239	H6PD	-0.258	4.081	-2.334	0.022
ENSG00000049246	PER3	-0.271	5.915	-2.946	0.004

ENSG00000049618	ARID1B	-0.230	7.400	-4.247	0.000
ENSG00000049656	CLPTM1L	0.425	4.705	3.668	0.000
ENSG00000049860	HEXB	0.235	4.810	2.558	0.012
ENSG00000050344	NFE2L3	-0.327	3.820	-2.367	0.020
ENSG00000050405	LIMA1	0.208	4.293	2.451	0.016
ENSG00000051341	POLQ	-0.349	3.043	-2.731	0.008
ENSG00000052723	SIKE1	0.141	5.822	2.024	0.046
ENSG00000052802	MSMO1	0.407	5.871	2.901	0.005
ENSG00000053108	FSTL4	-0.326	4.977	-2.937	0.004
ENSG00000053328	METTL24	-0.215	1.501	-2.065	0.042
ENSG00000053372	MRTO4	0.256	4.274	2.538	0.013
ENSG00000053501	USE1	0.299	2.714	2.412	0.018
ENSG00000053524	MCF2L2	-0.149	6.946	-2.064	0.042
ENSG00000053702	NRIP2	-0.220	3.012	-2.256	0.027
ENSG00000054179	ENTPD2	-0.420	0.028	-2.252	0.027
ENSG00000054282	SDCCAG8	0.241	6.989	2.591	0.011
ENSG00000054598	FOXC1	0.340	1.721	1.996	0.049
ENSG00000055147	FAM114A2	0.185	4.037	2.123	0.037
ENSG00000055609	KMT2C	-0.114	8.408	-2.529	0.013
ENSG00000055813	CCDC85A	0.248	4.771	2.548	0.013
ENSG00000056050	HPF1	0.418	3.540	3.079	0.003
ENSG00000056097	ZFR	0.189	7.715	2.499	0.014
ENSG00000056736	IL17RB	-0.209	4.019	-2.105	0.038
ENSG00000056972	TRAF3IP2	-0.291	3.663	-2.523	0.014
ENSG00000057608	GDI2	0.303	7.425	3.011	0.003
ENSG00000057757	PITHD1	0.233	6.398	2.159	0.034
ENSG00000058063	ATP11B	0.147	6.315	2.051	0.043
ENSG00000058091	CDK14	0.248	8.206	2.116	0.037
ENSG00000058404	CAMK2B	0.235	7.042	2.203	0.030
ENSG00000058799	YIPF1	0.301	3.717	3.534	0.001
ENSG00000058804	NDC1	0.275	4.764	3.145	0.002
ENSG00000059588	TARBP1	-0.274	6.875	-2.566	0.012
ENSG00000059758	CDK17	0.201	7.348	2.329	0.022
ENSG00000059769	DNAJC25	0.257	3.543	2.851	0.006
ENSG00000060140	STYK1	0.485	3.845	3.850	0.000
ENSG00000060237	WNK1	-0.152	8.830	-2.090	0.040
ENSG00000060558	GNA15	0.517	0.804	2.508	0.014
ENSG00000060718	COL11A1	0.253	5.345	2.258	0.027
ENSG00000060762	MPC1	0.231	5.096	2.809	0.006
ENSG00000061794	MRPS35	0.301	5.657	2.508	0.014
ENSG00000062038	CDH3	0.275	1.640	2.134	0.036
ENSG00000062282	DGAT2	0.310	1.922	2.110	0.038
ENSG00000062716	VMP1	0.264	6.359	3.125	0.002
ENSG00000062725	APPBP2	0.197	7.069	2.258	0.027
ENSG00000063169	GLTSCR1	-0.344	1.629	-2.071	0.042
ENSG00000063180	CA11	0.386	6.660	3.331	0.001
ENSG00000063854	HAGH	0.257	5.132	2.105	0.038

ENSG00000064102	ASUN	0.192	4.367	2.397	0.019
ENSG00000064205	WISP2	-0.468	0.078	-2.274	0.026
ENSG00000064309	CDON	-0.299	4.439	-3.363	0.001
ENSG00000064419	TNPO3	0.173	6.109	2.538	0.013
ENSG00000064651	SLC12A2	0.268	7.463	2.181	0.032
ENSG00000064652	SNX24	0.188	4.177	2.777	0.007
ENSG00000064655	EYA2	-0.407	2.299	-2.642	0.010
ENSG00000064726	BTBD1	0.361	7.123	3.617	0.001
ENSG00000064995	TAF11	0.160	3.986	3.061	0.003
ENSG00000065060	UHRF1BP1	-0.137	5.229	-3.168	0.002
ENSG00000065154	OAT	0.281	7.152	2.269	0.026
ENSG00000065427	KARS	0.260	5.488	2.631	0.010
ENSG00000065526	SPEN	-0.203	7.797	-3.358	0.001
ENSG00000065548	ZC3H15	0.333	7.083	3.106	0.003
ENSG00000065559	MAP2K4	0.259	7.825	2.151	0.034
ENSG00000065613	SLK	0.181	7.850	2.240	0.028
ENSG00000065675	PRKCQ	0.396	3.552	2.673	0.009
ENSG00000065883	CDK13	-0.073	7.286	-2.212	0.030
ENSG00000065911	MTHFD2	0.445	1.679	2.201	0.031
ENSG00000065970	FOXJ2	-0.143	4.236	-2.276	0.025
ENSG00000065978	YBX1	0.393	5.442	2.543	0.013
ENSG00000065989	PDE4A	-0.402	5.051	-3.332	0.001
ENSG00000066027	PPP2R5A	0.167	5.213	2.204	0.030
ENSG00000066032	CTNNA2	0.175	8.233	2.212	0.030
ENSG00000066044	ELAVL1	-0.144	5.307	-2.033	0.045
ENSG00000066382	MPPED2	-0.153	4.779	-2.199	0.031
ENSG00000066405	CLDN18	-0.488	0.010	-2.596	0.011
ENSG00000066422	ZBTB11	0.171	6.591	2.104	0.038
ENSG00000066455	GOLGA5	0.163	5.420	2.109	0.038
ENSG00000066557	LRRC40	0.294	5.612	2.945	0.004
ENSG00000066629	EML1	0.184	5.351	2.297	0.024
ENSG00000066827	ZFAT	-0.273	3.977	-3.244	0.002
ENSG00000066926	FECH	0.146	4.780	2.155	0.034
ENSG00000067064	IDI1	0.445	6.560	3.908	0.000
ENSG00000067066	SP100	0.280	3.286	2.224	0.029
ENSG00000067221	STOML1	0.218	3.818	2.250	0.027
ENSG00000067248	DHX29	0.139	5.825	2.062	0.042
ENSG00000067606	PRKCZ	0.209	5.983	2.338	0.022
ENSG00000067704	IARS2	0.265	6.212	2.795	0.006
ENSG00000068028	RASSF1	0.291	1.801	2.825	0.006
ENSG00000068831	RASGRP2	0.248	2.935	2.571	0.012
ENSG00000068912	ERLEC1	0.348	6.131	2.506	0.014
ENSG00000069020	MAST4	-0.117	6.983	-2.100	0.039
ENSG00000069248	NUP133	0.203	6.157	2.363	0.021
ENSG00000069275	NUCKS1	0.158	9.439	2.183	0.032
ENSG00000069329	VPS35	0.297	7.771	2.569	0.012
ENSG00000069345	DNAJA2	0.330	7.146	2.695	0.009

ENSG00000069493	CLEC2D	-0.247	2.247	-2.203	0.030
ENSG00000069849	ATP1B3	0.207	5.473	2.318	0.023
ENSG00000069943	PIGB	-0.173	2.984	-2.420	0.018
ENSG00000069956	MAPK6	0.256	7.004	2.511	0.014
ENSG00000069998	CECR5	0.261	2.696	2.695	0.009
ENSG00000070081	NUCB2	0.151	5.630	2.359	0.021
ENSG00000070367	EXOC5	0.196	7.156	2.589	0.011
ENSG00000070526	ST6GALNAC1	0.427	0.745	2.002	0.049
ENSG00000070540	WIPI1	0.158	2.943	2.302	0.024
ENSG00000070718	AP3M2	0.189	6.667	2.019	0.047
ENSG00000070778	PTPN21	-0.233	4.208	-3.522	0.001
ENSG00000070785	EIF2B3	0.255	4.257	2.653	0.010
ENSG00000070961	ATP2B1	0.234	10.032	2.020	0.047
ENSG00000071073	MGAT4A	0.284	6.822	3.692	0.000
ENSG00000071082	RPL31	0.565	6.973	2.085	0.040
ENSG00000071127	WDR1	0.155	5.372	2.047	0.044
ENSG00000071189	SNX13	0.163	7.253	2.422	0.018
ENSG00000071205	ARHGAP10	0.669	5.185	4.163	0.000
ENSG00000071462	WBSCR22	0.267	3.789	2.794	0.006
ENSG00000071537	SEL1L	0.167	7.546	2.205	0.030
ENSG00000071539	TRIP13	0.282	0.454	2.022	0.046
ENSG00000071967	CYBRD1	-0.427	5.485	-2.141	0.035
ENSG00000071994	PDCD2	0.132	4.864	2.158	0.034
ENSG00000072042	RDH11	0.346	6.401	3.777	0.000
ENSG00000072121	ZFYVE26	-0.166	5.354	-3.057	0.003
ENSG00000072163	LIMS2	0.393	2.431	2.266	0.026
ENSG00000072182	ASIC4	0.251	1.396	2.057	0.043
ENSG00000072274	TFRC	0.379	6.915	3.022	0.003
ENSG00000072401	UBE2D1	0.230	4.610	2.377	0.020
ENSG00000072422	RHOBTB1	0.151	4.632	2.352	0.021
ENSG00000072657	TRHDE	0.249	7.066	2.161	0.034
ENSG00000072736	NFATC3	-0.141	5.087	-2.451	0.016
ENSG00000072756	TRNT1	0.219	4.734	3.030	0.003
ENSG00000072786	STK10	0.213	2.922	2.162	0.034
ENSG00000072803	FBXW11	0.215	7.166	2.705	0.008
ENSG00000072858	SIDT1	-0.230	5.067	-2.903	0.005
ENSG00000073737	DHRS9	0.912	0.027	2.547	0.013
ENSG00000073792	IGF2BP2	0.883	-1.373	2.866	0.005
ENSG00000073849	ST6GAL1	0.227	5.550	2.530	0.013
ENSG00000073921	PICALM	0.209	7.819	2.421	0.018
ENSG00000074054	CLASP1	-0.110	7.881	-2.505	0.014
ENSG00000074071	MRPS34	0.289	3.646	2.243	0.028
ENSG00000074201	CLNS1A	0.215	6.264	2.135	0.036
ENSG00000074416	MGLL	0.222	6.699	2.603	0.011
ENSG00000074696	HACD3	0.199	7.687	2.465	0.016
ENSG00000074800	ENO1	0.274	8.134	2.107	0.038
ENSG00000074842	MYDGF	0.478	2.228	3.451	0.001

ENSG00000075089	ACTR6	0.386	5.159	2.941	0.004
ENSG00000075239	ACAT1	0.275	5.391	2.610	0.011
ENSG00000075336	TIMM21	0.183	4.642	2.512	0.014
ENSG00000075415	SLC25A3	0.203	7.897	2.473	0.015
ENSG00000075461	CACNG4	0.294	2.830	2.417	0.018
ENSG00000075568	TMEM131	-0.095	6.357	-2.282	0.025
ENSG00000075711	DLG1	0.205	8.042	2.381	0.020
ENSG00000075826	SEC31B	-0.293	3.634	-2.184	0.032
ENSG00000075945	KIFAP3	0.283	7.544	2.282	0.025
ENSG00000075975	MKRN2	0.133	5.269	2.230	0.028
ENSG00000076003	MCM6	0.263	3.874	2.348	0.021
ENSG00000076043	REXO2	0.246	3.982	3.687	0.000
ENSG00000076108	BAZ2A	-0.198	6.025	-2.695	0.009
ENSG00000076201	PTPN23	-0.240	4.432	-2.486	0.015
ENSG00000076555	ACACB	-0.319	4.934	-2.379	0.020
ENSG00000077147	TM9SF3	0.234	7.836	2.704	0.008
ENSG00000077152	UBE2T	0.497	2.556	3.078	0.003
ENSG00000077238	IL4R	0.472	1.705	2.065	0.042
ENSG00000077254	USP33	0.195	7.502	2.067	0.042
ENSG00000077327	SPAG6	0.372	1.412	2.128	0.036
ENSG00000077380	DYNC1I2	0.356	6.419	2.594	0.011
ENSG00000077616	NAALAD2	-0.250	5.241	-2.592	0.011
ENSG00000077935	SMC1B	-0.407	-0.861	-2.039	0.045
ENSG00000078098	FAP	-0.272	1.887	-2.171	0.033
ENSG00000078124	ACER3	0.266	5.877	3.091	0.003
ENSG00000078304	PPP2R5C	0.244	7.424	2.983	0.004
ENSG00000078403	MLLT10	-0.172	5.861	-3.511	0.001
ENSG00000078549	ADCYAP1R1	-0.178	6.978	-1.997	0.049
ENSG00000078668	VDAC3	0.262	6.261	2.134	0.036
ENSG00000078674	PCM1	0.128	9.029	2.099	0.039
ENSG00000078687	TNRC6C	-0.151	6.552	-2.670	0.009
ENSG00000078699	CBFA2T2	-0.170	5.571	-2.946	0.004
ENSG00000078747	ITCH	0.245	7.124	2.563	0.012
ENSG00000078795	PKD2L2	-0.323	0.939	-2.216	0.029
ENSG00000078808	SDF4	0.203	4.927	2.571	0.012
ENSG00000079332	SAR1A	0.282	7.075	3.047	0.003
ENSG00000079459	FDFT1	0.191	6.398	2.369	0.020
ENSG00000079462	PAFAH1B3	0.312	1.738	2.225	0.029
ENSG00000079616	KIF22	0.167	3.012	2.088	0.040
ENSG00000079785	DDX1	0.297	6.974	2.122	0.037
ENSG00000079819	EPB41L2	0.177	8.325	2.964	0.004
ENSG00000079841	RIMS1	0.265	8.610	2.737	0.008
ENSG00000079974	RABL2B	-0.280	3.461	-2.227	0.029
ENSG00000080200	CRYBG3	0.297	6.218	2.703	0.008
ENSG00000080603	SRCAP	-0.404	1.950	-2.648	0.010
ENSG00000080644	CHRNA3	0.663	-1.052	2.323	0.023
ENSG00000080845	DLGAP4	-0.319	5.550	-2.367	0.020

ENSG00000081087	OSTM1	0.309	5.251	3.326	0.001
ENSG00000081138	CDH7	0.233	4.838	2.127	0.036
ENSG00000081177	EXD2	0.120	5.557	2.787	0.007
ENSG00000081181	ARG2	0.221	2.646	2.216	0.029
ENSG00000081721	DUSP12	0.301	2.946	2.565	0.012
ENSG00000081760	AACS	0.134	5.893	2.021	0.047
ENSG00000081818	PCDHB4	-0.387	3.767	-3.516	0.001
ENSG00000082014	SMARCD3	0.191	5.003	2.512	0.014
ENSG00000082146	STRADB	0.158	3.663	2.084	0.040
ENSG00000082153	BZW1	0.306	6.112	3.012	0.003
ENSG00000082293	COL19A1	0.302	4.140	2.361	0.021
ENSG00000082482	KCNK2	0.244	4.052	2.345	0.021
ENSG00000082996	RNF13	0.291	5.989	2.369	0.020
ENSG00000083099	LYRM2	0.194	5.718	2.542	0.013
ENSG00000083290	ULK2	0.172	6.698	2.809	0.006
ENSG00000083457	ITGAE	0.291	1.756	2.148	0.035
ENSG00000083520	DIS3	0.161	6.144	2.093	0.039
ENSG00000083720	OXCT1	0.316	7.502	2.565	0.012
ENSG00000083844	ZNF264	-0.102	6.092	-2.308	0.024
ENSG00000083937	CHMP2B	0.262	5.516	2.681	0.009
ENSG00000084072	PPIE	0.183	4.165	2.092	0.040
ENSG00000084073	ZMPSTE24	0.195	5.926	2.152	0.034
ENSG00000084090	STARD7	0.177	7.282	2.265	0.026
ENSG00000084234	APLP2	0.181	9.126	2.552	0.013
ENSG00000084623	EIF3I	0.339	4.728	3.054	0.003
ENSG00000084652	TXLNA	0.135	5.476	2.570	0.012
ENSG00000084733	RAB10	0.204	7.058	3.245	0.002
ENSG00000085231	AK6	0.424	3.676	3.407	0.001
ENSG00000085276	MECOM	0.417	3.116	2.472	0.016
ENSG00000085365	SCAMP1	0.285	7.556	2.478	0.015
ENSG00000085449	WDFY1	0.248	6.045	3.330	0.001
ENSG00000085465	OVGP1	-0.407	2.207	-2.591	0.011
ENSG00000085721	RRN3	0.211	5.431	2.522	0.014
ENSG00000085760	MTIF2	0.309	5.490	2.703	0.008
ENSG00000085788	DDHD2	0.256	7.742	2.655	0.010
ENSG00000085832	EPS15	0.224	8.950	2.389	0.019
ENSG00000085872	CHERP	-0.293	2.930	-2.280	0.025
ENSG00000085978	ATG16L1	0.135	5.968	2.050	0.044
ENSG00000085998	POMGNT1	0.193	4.747	2.301	0.024
ENSG00000086061	DNAJA1	0.363	7.908	3.244	0.002
ENSG00000086065	CHMP5	0.535	5.794	2.939	0.004
ENSG00000086289	EPDR1	0.236	6.965	2.093	0.039
ENSG00000086300	SNX10	0.298	6.985	2.049	0.044
ENSG00000086475	SEPHS1	0.172	4.859	1.992	0.050
ENSG00000086504	MRPL28	0.365	4.137	2.893	0.005
ENSG00000086598	TMED2	0.192	6.559	2.492	0.015
ENSG00000086666	ZFAND6	0.274	5.053	3.178	0.002

ENSG00000086730	LAT2	0.427	1.096	1.992	0.050
ENSG00000086827	ZW10	0.113	4.559	2.095	0.039
ENSG00000086967	MYBPC2	1.050	-1.717	2.746	0.007
ENSG00000087087	SRRT	0.149	4.952	2.011	0.048
ENSG00000087095	NLK	0.241	8.178	2.988	0.004
ENSG00000087152	ATXN7L3	0.277	6.661	2.806	0.006
ENSG00000087245	MMP2	0.469	0.286	2.234	0.028
ENSG00000087258	GNAO1	-0.203	8.106	-2.263	0.026
ENSG00000087301	TXNDC16	0.189	5.870	2.587	0.011
ENSG00000087302	C14orf166	0.301	5.478	2.753	0.007
ENSG00000087502	ERGIC2	0.278	6.126	3.219	0.002
ENSG00000087589	CASS4	0.470	0.574	2.443	0.017
ENSG00000087903	RFX2	-0.344	2.237	-2.389	0.019
ENSG00000087995	METTL2A	0.124	4.001	2.033	0.045
ENSG00000088179	PTPN4	0.193	7.941	2.449	0.016
ENSG00000088205	DDX18	0.266	5.929	3.145	0.002
ENSG00000088305	DNMT3B	-0.354	0.799	-2.617	0.011
ENSG00000088320	REM1	-1.062	-2.629	-3.056	0.003
ENSG00000088356	PDRG1	0.387	2.107	3.972	0.000
ENSG00000088367	EPB41L1	-0.191	8.264	-2.402	0.019
ENSG00000088387	DOCK9	0.170	8.161	2.695	0.009
ENSG00000088543	C3orf18	0.212	3.946	2.656	0.010
ENSG00000088727	KIF9	0.180	2.549	2.075	0.041
ENSG00000088836	SLC4A11	-0.481	-0.069	-2.497	0.015
ENSG00000088876	ZNF343	-0.128	4.071	-2.132	0.036
ENSG00000088882	CPXM1	1.053	-2.367	3.060	0.003
ENSG00000088930	XRN2	0.205	5.531	2.324	0.023
ENSG00000088992	TESC	0.540	2.474	2.730	0.008
ENSG00000089009	RPL6	0.534	6.241	2.518	0.014
ENSG00000089050	RBBP9	0.191	3.821	2.230	0.029
ENSG00000089063	TMEM230	0.190	5.480	2.137	0.036
ENSG00000089123	TASP1	0.258	4.589	2.239	0.028
ENSG00000089154	GCN1	0.115	5.461	2.043	0.044
ENSG00000089157	RPLP0	0.567	4.643	2.341	0.022
ENSG00000089220	PEBP1	0.263	8.553	2.363	0.020
ENSG00000089234	BRAP	0.159	5.529	2.344	0.022
ENSG00000089248	ERP29	0.320	5.115	3.133	0.002
ENSG00000089280	FUS	-0.284	5.808	-3.143	0.002
ENSG00000089335	ZNF302	0.180	5.879	2.514	0.014
ENSG00000089639	GMIP	0.341	1.636	2.741	0.008
ENSG00000089723	OTUB2	0.322	1.713	2.287	0.025
ENSG00000089775	ZBTB25	-0.180	5.458	-2.864	0.005
ENSG00000090013	BLVRB	0.335	2.251	2.155	0.034
ENSG00000090020	SLC9A1	-0.292	4.231	-2.545	0.013
ENSG00000090054	SPTLC1	0.242	5.676	2.812	0.006
ENSG00000090263	MRPS33	0.288	4.529	3.504	0.001
ENSG00000090266	NDUFB2	0.528	4.315	3.097	0.003

ENSG00000090372	STRN4	0.223	5.181	2.531	0.013
ENSG00000090376	IRAK3	0.451	3.061	2.415	0.018
ENSG00000090432	MUL1	0.188	3.684	2.698	0.008
ENSG00000090520	DNAJB11	0.233	4.108	2.956	0.004
ENSG00000090530	P3H2	-0.340	2.151	-3.764	0.000
ENSG00000090615	GOLGA3	0.120	7.063	2.004	0.048
ENSG00000090621	PABPC4	0.157	5.167	2.427	0.017
ENSG00000090857	PDPR	-0.419	4.848	-3.049	0.003
ENSG00000090905	TNRC6A	-0.253	7.405	-3.958	0.000
ENSG00000090989	EXOC1	0.208	6.534	2.371	0.020
ENSG00000091127	PUS7	0.169	4.426	2.234	0.028
ENSG00000091137	SLC26A4	-0.218	3.678	-2.095	0.039
ENSG00000091140	DLD	0.260	6.883	2.445	0.017
ENSG00000091164	TXNL1	0.181	7.267	2.410	0.018
ENSG00000091428	RAPGEF4	0.291	9.067	2.364	0.020
ENSG00000091483	FH	0.246	5.586	2.321	0.023
ENSG00000091527	CDV3	0.212	6.878	2.888	0.005
ENSG00000091640	SPAG7	0.286	4.332	2.507	0.014
ENSG00000091656	ZFHX4	-0.340	5.872	-2.650	0.010
ENSG00000091831	ESR1	-0.462	2.940	-3.224	0.002
ENSG00000091844	RGS17	0.170	5.363	2.377	0.020
ENSG00000092108	SCFD1	0.303	5.719	2.340	0.022
ENSG00000092148	HECTD1	0.189	8.793	2.189	0.031
ENSG00000092199	HNRNPC	0.210	7.984	2.401	0.019
ENSG00000092208	GEMIN2	0.333	2.847	2.722	0.008
ENSG00000092758	COL9A3	0.336	2.507	2.010	0.048
ENSG00000092841	MYL6	0.361	6.076	3.709	0.000
ENSG00000093000	NUP50	0.167	6.429	2.564	0.012
ENSG00000093144	ECHDC1	0.235	5.161	3.060	0.003
ENSG00000093183	SEC22C	0.158	6.104	2.415	0.018
ENSG00000093217	XYLB	-0.220	3.208	-2.332	0.022
ENSG00000094804	CDC6	0.378	2.744	3.169	0.002
ENSG00000094880	CDC23	0.174	4.626	2.994	0.004
ENSG00000094914	AAAS	0.183	3.314	2.448	0.016
ENSG00000094916	CBX5	-0.139	8.738	-2.419	0.018
ENSG00000095380	NANS	0.217	3.841	2.792	0.007
ENSG00000095464	PDE6C	-0.356	0.969	-2.129	0.036
ENSG00000095539	SEMA4G	-0.265	2.046	-2.400	0.019
ENSG00000095564	BTAF1	0.110	6.862	2.004	0.048
ENSG00000095574	IKZF5	-0.169	4.997	-2.589	0.011
ENSG00000095587	TLL2	0.242	3.345	2.239	0.028
ENSG00000095596	CYP26A1	0.740	-1.352	2.338	0.022
ENSG00000095637	SORBS1	-0.157	8.661	-2.484	0.015
ENSG00000095794	CREM	0.311	4.379	3.358	0.001
ENSG00000095951	HIVEP1	-0.186	6.781	-2.764	0.007
ENSG00000096070	BRPF3	-0.220	5.669	-2.616	0.011
ENSG00000096092	TMEM14A	0.352	4.812	2.128	0.036

ENSG00000096093	EFHC1	-0.115	4.771	-2.078	0.041
ENSG00000096384	HSP90AB1	0.314	9.804	2.690	0.009
ENSG00000096401	CDC5L	0.170	6.548	2.033	0.045
ENSG00000096433	ITPR3	0.510	2.086	3.369	0.001
ENSG00000096696	DSP	0.696	2.898	3.582	0.001
ENSG00000096872	IFT74	0.239	4.064	1.996	0.049
ENSG00000099204	ABLIM1	-0.168	8.043	-2.577	0.012
ENSG00000099260	PALMD	0.325	3.374	2.500	0.014
ENSG00000099337	KCNK6	0.360	0.537	2.373	0.020
ENSG00000099341	PSMD8	0.276	5.171	2.518	0.014
ENSG00000099365	STX1B	-0.213	7.195	-2.015	0.047
ENSG00000099381	SETD1A	-0.259	3.601	-2.413	0.018
ENSG00000099795	NDUFB7	0.320	3.736	2.037	0.045
ENSG00000099800	TIMM13	0.510	2.971	3.788	0.000
ENSG00000099901	RANBP1	0.417	4.125	2.599	0.011
ENSG00000099917	MED15	-0.191	4.470	-2.139	0.035
ENSG00000100028	SNRPD3	0.220	5.093	2.890	0.005
ENSG00000100029	PES1	0.255	4.289	2.607	0.011
ENSG00000100055	CYTH4	0.582	1.863	3.098	0.003
ENSG00000100065	CARD10	0.619	-0.794	2.398	0.019
ENSG00000100124	ANKRD54	0.176	3.357	2.234	0.028
ENSG00000100142	POLR2F	0.246	4.385	2.756	0.007
ENSG00000100154	TTC28	-0.206	5.430	-3.724	0.000
ENSG00000100170	SLC5A1	0.882	-2.340	2.715	0.008
ENSG00000100196	KDELR3	-0.332	1.627	-2.969	0.004
ENSG00000100201	DDX17	-0.109	9.465	-2.088	0.040
ENSG00000100216	TOMM22	0.241	5.549	2.347	0.021
ENSG00000100219	XBP1	0.382	4.247	3.915	0.000
ENSG00000100220	RTCB	0.327	5.207	2.867	0.005
ENSG00000100239	PPP6R2	0.160	5.854	2.103	0.039
ENSG00000100276	RASL10A	0.392	2.070	3.108	0.003
ENSG00000100296	THOC5	0.159	5.457	2.683	0.009
ENSG00000100302	RASD2	-0.307	3.934	-2.113	0.038
ENSG00000100314	CABP7	0.361	1.083	2.056	0.043
ENSG00000100319	ZMAT5	0.274	1.125	2.005	0.048
ENSG00000100345	MYH9	0.210	6.974	2.230	0.028
ENSG00000100350	FOXRED2	-0.355	4.645	-3.179	0.002
ENSG00000100354	TNRC6B	-0.140	8.298	-3.246	0.002
ENSG00000100379	KCTD17	0.247	4.635	2.325	0.023
ENSG00000100387	RBX1	0.217	3.924	2.777	0.007
ENSG00000100393	EP300	-0.168	6.968	-3.198	0.002
ENSG00000100410	PHF5A	0.289	3.371	3.085	0.003
ENSG00000100417	PMM1	0.383	3.122	2.701	0.008
ENSG00000100422	CERK	0.202	6.111	2.198	0.031
ENSG00000100442	FKBP3	0.258	5.649	2.086	0.040
ENSG00000100478	AP4S1	0.189	5.360	2.617	0.011
ENSG00000100479	POLE2	-0.335	1.465	-2.130	0.036

ENSG00000100483	VCPKMT	0.367	3.272	2.991	0.004
ENSG00000100505	TRIM9	-0.136	7.768	-2.113	0.038
ENSG00000100519	PSMC6	0.482	5.740	2.524	0.014
ENSG00000100522	GNPNAT1	0.203	3.829	2.105	0.038
ENSG00000100528	CNIH1	0.162	5.693	2.281	0.025
ENSG00000100554	ATP6V1D	0.394	6.294	2.588	0.011
ENSG00000100564	PIGH	0.284	2.445	2.487	0.015
ENSG00000100567	PSMA3	0.184	5.192	2.046	0.044
ENSG00000100568	VTI1B	0.193	6.295	2.634	0.010
ENSG00000100575	TIMM9	0.186	2.854	2.136	0.036
ENSG00000100591	AHSA1	0.299	6.284	2.613	0.011
ENSG00000100600	LGMN	0.229	4.603	2.695	0.009
ENSG00000100603	SNW1	0.269	5.502	2.700	0.008
ENSG00000100612	DHRS7	0.251	4.927	2.432	0.017
ENSG00000100632	ERH	0.241	4.766	2.545	0.013
ENSG00000100744	GSKIP	0.346	4.556	2.655	0.010
ENSG00000100764	PSMC1	0.981	3.130	3.356	0.001
ENSG00000100767	PAPLN	-0.512	3.489	-2.705	0.008
ENSG00000100804	PSMB5	0.384	5.160	2.883	0.005
ENSG00000100823	APEX1	0.283	5.315	3.556	0.001
ENSG00000100883	SRP54	0.245	5.760	2.378	0.020
ENSG00000100897	DCAF11	0.213	6.065	2.434	0.017
ENSG00000100911	PSME2	0.300	2.911	2.701	0.008
ENSG00000100934	SEC23A	0.256	6.871	2.137	0.036
ENSG00000100938	GMPR2	0.224	5.468	2.607	0.011
ENSG00000100979	PLTP	-0.377	3.530	-2.315	0.023
ENSG00000100982	PCIF1	-0.243	2.983	-2.568	0.012
ENSG00000100983	GSS	0.277	4.286	3.489	0.001
ENSG00000100997	ABHD12	0.233	5.479	2.275	0.026
ENSG00000101000	PROCR	0.471	-0.474	2.014	0.047
ENSG00000101003	GINS1	0.191	2.295	2.062	0.042
ENSG00000101019	UQCC1	0.177	5.638	3.062	0.003
ENSG00000101079	NDRG3	0.232	7.532	2.200	0.031
ENSG00000101098	RIMS4	0.205	5.616	2.234	0.028
ENSG00000101126	ADNP	-0.092	7.131	-2.101	0.039
ENSG00000101132	PFDN4	0.392	4.755	2.611	0.011
ENSG00000101146	RAE1	0.188	3.817	2.463	0.016
ENSG00000101166	PRELID3B	0.244	4.601	2.395	0.019
ENSG00000101182	PSMA7	0.343	4.937	2.557	0.012
ENSG00000101189	MRGBP	0.192	2.766	2.138	0.036
ENSG00000101191	DIDO1	-0.082	6.112	-2.012	0.048
ENSG00000101194	SLC17A9	0.631	-1.268	2.913	0.005
ENSG00000101199	ARFGAP1	0.221	4.450	2.443	0.017
ENSG00000101230	ISM1	0.328	0.508	2.081	0.041
ENSG00000101246	ARFRP1	0.301	3.747	3.698	0.000
ENSG00000101247	NDUFAF5	0.225	5.262	2.042	0.044
ENSG00000101266	CSNK2A1	0.132	6.926	2.949	0.004

ENSG00000101306	MYLK2	0.694	-1.425	2.123	0.037
ENSG00000101310	SEC23B	0.241	5.301	2.535	0.013
ENSG00000101350	KIF3B	0.218	7.538	2.967	0.004
ENSG00000101365	IDH3B	0.290	5.195	2.683	0.009
ENSG00000101412	E2F1	0.321	0.997	2.301	0.024
ENSG00000101417	PXMP4	0.167	3.670	2.567	0.012
ENSG00000101444	AHCY	0.278	4.193	3.072	0.003
ENSG00000101445	PPP1R16B	0.184	7.870	2.448	0.016
ENSG00000101473	ACOT8	0.217	3.724	2.682	0.009
ENSG00000101489	CELF4	0.178	6.841	2.336	0.022
ENSG00000101493	ZNF516	-0.233	4.469	-2.073	0.041
ENSG00000101608	MYL12A	0.303	3.164	2.602	0.011
ENSG00000101752	MIB1	0.212	8.014	2.481	0.015
ENSG00000101782	RIOK3	0.187	5.399	2.314	0.023
ENSG00000102543	CDADC1	0.231	4.221	2.682	0.009
ENSG00000102572	STK24	0.189	5.437	2.279	0.025
ENSG00000102580	DNAJC3	0.214	5.954	2.615	0.011
ENSG00000102738	MRPS31	0.223	4.558	2.726	0.008
ENSG00000102753	KPNA3	0.198	6.369	2.486	0.015
ENSG00000102755	FLT1	0.430	6.318	2.558	0.012
ENSG00000102802	MEDAG	0.623	-0.703	2.677	0.009
ENSG00000102871	TRADD	0.366	0.233	2.179	0.032
ENSG00000102897	LYRM1	0.252	3.867	2.692	0.009
ENSG00000102898	NUTF2	0.216	5.347	3.304	0.001
ENSG00000102900	NUP93	0.107	6.402	2.055	0.043
ENSG00000102908	NFAT5	-0.229	8.347	-3.631	0.000
ENSG00000102910	LONP2	0.098	7.391	2.166	0.033
ENSG00000102924	CBLN1	0.287	1.945	2.129	0.036
ENSG00000102978	POLR2C	0.244	4.551	2.833	0.006
ENSG00000103018	CYB5B	0.257	6.322	3.514	0.001
ENSG00000103034	NDRG4	0.178	9.202	2.428	0.017
ENSG00000103035	PSMD7	0.335	5.876	3.408	0.001
ENSG00000103091	WDR59	-0.125	4.687	-2.084	0.040
ENSG00000103126	AXIN1	0.181	3.683	2.611	0.011
ENSG00000103148	NPRL3	-0.342	3.291	-2.838	0.006
ENSG00000103150	MLYCD	-0.239	4.387	-2.181	0.032
ENSG00000103174	NAGPA	0.202	3.989	2.368	0.020
ENSG00000103175	WFDC1	0.262	2.721	2.452	0.016
ENSG00000103274	NUBP1	0.248	1.695	2.381	0.020
ENSG00000103316	CRYM	0.465	5.846	2.961	0.004
ENSG00000103319	EEF2K	-0.252	4.814	-2.561	0.012
ENSG00000103356	EARS2	0.138	5.035	2.474	0.015
ENSG00000103363	TCEB2	0.296	3.940	2.256	0.027
ENSG00000103415	HMOX2	0.285	5.748	2.660	0.009
ENSG00000103423	DNAJA3	0.266	4.861	3.297	0.001
ENSG00000103429	BFAR	0.203	4.420	2.725	0.008
ENSG00000103490	PYCARD	1.257	-1.955	3.530	0.001

ENSG00000103494	RPGRIP1L	0.133	5.774	2.054	0.043
ENSG00000103496	STX4	0.297	3.486	3.175	0.002
ENSG00000103510	KAT8	0.136	4.687	2.418	0.018
ENSG00000103549	RNF40	0.150	5.367	2.358	0.021
ENSG00000103591	AAGAB	0.314	6.071	2.928	0.004
ENSG00000103642	LACTB	0.224	3.662	2.451	0.016
ENSG00000103742	IGDCC4	-0.452	2.600	-2.744	0.007
ENSG00000103769	RAB11A	0.278	7.700	2.748	0.007
ENSG00000103855	CD276	-0.213	2.930	-2.230	0.029
ENSG00000103888	CEMIP	0.195	4.493	2.208	0.030
ENSG00000103978	TMEM87A	0.290	4.676	2.271	0.026
ENSG00000104064	GABPB1	0.220	3.501	2.502	0.014
ENSG00000104093	DMXL2	0.178	8.968	2.002	0.049
ENSG00000104131	EIF3J	0.216	5.523	2.734	0.008
ENSG00000104140	RHOV	0.366	0.804	2.234	0.028
ENSG00000104154	SLC30A4	0.196	5.549	2.446	0.017
ENSG00000104164	BLOC1S6	0.264	6.923	2.688	0.009
ENSG00000104213	PDGFRL	-0.330	-0.362	-2.014	0.047
ENSG00000104221	BRF2	0.397	1.933	2.904	0.005
ENSG00000104299	INTS9	-0.125	2.636	-2.158	0.034
ENSG00000104313	EYA1	-0.456	3.341	-2.566	0.012
ENSG00000104320	NBN	0.205	6.473	2.737	0.008
ENSG00000104327	CALB1	0.492	5.547	2.733	0.008
ENSG00000104343	UBE2W	0.247	7.101	3.512	0.001
ENSG00000104368	PLAT	0.235	3.051	2.024	0.046
ENSG00000104381	GDAP1	0.192	7.753	2.036	0.045
ENSG00000104388	RAB2A	0.237	7.310	2.276	0.025
ENSG00000104408	EIF3E	0.345	6.001	2.353	0.021
ENSG00000104435	STMN2	0.339	9.294	2.337	0.022
ENSG00000104442	ARMC1	0.165	6.011	2.007	0.048
ENSG00000104447	TRPS1	-0.192	6.818	-2.005	0.048
ENSG00000104522	TSTA3	0.299	2.339	2.648	0.010
ENSG00000104549	SQLE	0.433	5.437	4.112	0.000
ENSG00000104643	MTMR9	0.187	6.880	2.206	0.030
ENSG00000104660	LEPROTL1	0.187	5.588	2.588	0.011
ENSG00000104671	DCTN6	0.202	5.070	2.067	0.042
ENSG00000104679	R3HCC1	0.331	4.045	3.253	0.002
ENSG00000104687	GSR	0.213	5.386	2.492	0.015
ENSG00000104691	UBXN8	0.339	3.252	2.990	0.004
ENSG00000104723	TUSC3	0.243	6.137	2.188	0.032
ENSG00000104765	BNIP3L	0.288	7.793	3.101	0.003
ENSG00000104872	PIH1D1	0.276	4.202	2.271	0.026
ENSG00000104886	PLEKHJ1	0.355	2.366	2.928	0.004
ENSG00000104904	OAZ1	0.254	6.511	2.094	0.039
ENSG00000104946	TBC1D17	-0.188	3.705	-2.130	0.036
ENSG00000104964	AES	0.213	6.727	2.266	0.026
ENSG00000104967	NOVA2	-0.342	5.503	-2.707	0.008

ENSG00000104979	C19orf53	0.240	3.756	2.005	0.048
ENSG00000104998	IL27RA	0.319	1.276	2.330	0.022
ENSG00000105048	TNNT1	0.471	1.736	2.631	0.010
ENSG00000105058	FAM32A	0.420	5.356	3.598	0.001
ENSG00000105171	POP4	0.438	3.833	4.140	0.000
ENSG00000105173	CCNE1	0.347	1.725	3.451	0.001
ENSG00000105176	URI1	0.171	6.460	2.263	0.026
ENSG00000105185	PDCD5	0.301	4.375	2.039	0.045
ENSG00000105186	ANKRD27	0.148	5.518	2.022	0.046
ENSG00000105193	RPS16	0.417	3.279	2.199	0.031
ENSG00000105197	TIMM50	0.244	4.591	3.062	0.003
ENSG00000105254	TBCB	0.318	6.137	3.043	0.003
ENSG00000105255	FSD1	0.301	3.782	2.988	0.004
ENSG00000105270	CLIP3	-0.439	6.652	-2.841	0.006
ENSG00000105327	BBC3	0.333	0.695	2.076	0.041
ENSG00000105341	ATP5SL	0.173	3.524	2.069	0.042
ENSG00000105404	RABAC1	0.289	2.063	2.382	0.020
ENSG00000105419	MEIS3	0.435	4.765	2.963	0.004
ENSG00000105518	TMEM205	0.192	2.964	2.521	0.014
ENSG00000105605	CACNG7	-0.271	4.419	-2.038	0.045
ENSG00000105609	LILRB5	1.537	-1.510	4.129	0.000
ENSG00000105618	PRPF31	0.198	3.940	2.087	0.040
ENSG00000105619	TFPT	0.282	3.085	2.320	0.023
ENSG00000105640	RPL18A	0.284	3.194	2.000	0.049
ENSG00000105662	CRTC1	-0.363	5.025	-2.498	0.014
ENSG00000105671	DDX49	0.374	3.317	3.068	0.003
ENSG00000105676	ARMC6	0.164	2.847	1.997	0.049
ENSG00000105732	ZNF574	-0.295	2.724	-2.940	0.004
ENSG00000105792	CFAP69	-0.281	4.242	-2.677	0.009
ENSG00000105829	BET1	0.210	3.660	2.374	0.020
ENSG00000105835	NAMPT	0.293	5.688	2.374	0.020
ENSG00000105849	TWISTNB	0.216	4.797	2.339	0.022
ENSG00000105851	PIK3CG	0.428	2.230	2.738	0.008
ENSG00000105852	PON3	-0.619	0.162	-2.439	0.017
ENSG00000105887	MTPN	0.254	8.048	2.750	0.007
ENSG00000105929	ATP6V0A4	0.544	-1.071	2.343	0.022
ENSG00000105983	LMBR1	0.225	6.998	2.884	0.005
ENSG00000105989	WNT2	0.647	1.162	3.543	0.001
ENSG00000105993	DNAJB6	0.295	5.594	2.139	0.035
ENSG00000106028	SSBP1	0.241	5.103	2.156	0.034
ENSG00000106034	CPED1	0.474	2.695	2.156	0.034
ENSG00000106052	TAX1BP1	0.262	7.820	2.506	0.014
ENSG00000106089	STX1A	0.315	5.955	2.762	0.007
ENSG00000106125	FAM188B	-0.450	0.823	-2.785	0.007
ENSG00000106128	GHRHR	-0.522	-1.659	-2.075	0.041
ENSG00000106153	CHCHD2	0.513	4.502	2.477	0.015
ENSG00000106244	PDAP1	0.280	5.245	2.395	0.019

ENSG00000106245	BUD31	0.398	3.946	3.614	0.001
ENSG00000106258	CYP3A5	-0.331	3.007	-2.519	0.014
ENSG00000106263	EIF3B	0.263	5.362	2.697	0.008
ENSG00000106346	USP42	-0.166	4.214	-3.149	0.002
ENSG00000106348	IMPDH1	0.287	3.246	2.537	0.013
ENSG00000106367	AP1S1	0.385	4.524	2.587	0.011
ENSG00000106399	RPA3	0.509	2.959	3.292	0.001
ENSG00000106400	ZNHIT1	0.222	3.873	2.637	0.010
ENSG00000106443	PHF14	0.172	7.760	2.435	0.017
ENSG00000106526	ACTR3C	-0.295	2.384	-2.365	0.020
ENSG00000106536	POU6F2	-0.217	4.339	-2.102	0.039
ENSG00000106554	CHCHD3	0.306	4.974	3.204	0.002
ENSG00000106560	GIMAP2	0.567	0.702	2.511	0.014
ENSG00000106571	GLI3	-0.598	3.796	-3.179	0.002
ENSG00000106588	PSMA2	0.244	2.878	2.935	0.004
ENSG00000106591	MRPL32	0.183	4.589	2.203	0.030
ENSG00000106603	COA1	-0.099	5.288	-2.219	0.029
ENSG00000106605	BLVRA	0.266	4.126	2.262	0.026
ENSG00000106615	RHEB	0.492	5.544	2.751	0.007
ENSG00000106636	YKT6	0.258	5.091	4.066	0.000
ENSG00000106638	TBL2	0.147	3.779	2.432	0.017
ENSG00000106648	GALNTL5	0.434	1.397	2.302	0.024
ENSG00000106682	EIF4H	0.239	6.912	2.731	0.008
ENSG00000106686	SPATA6L	-0.248	3.405	-2.864	0.005
ENSG00000106688	SLC1A1	0.236	5.838	2.675	0.009
ENSG00000106714	CNTNAP3	-0.249	4.002	-2.052	0.043
ENSG00000106803	SEC61B	0.236	2.553	2.088	0.040
ENSG00000106823	ECM2	-0.290	2.776	-2.384	0.019
ENSG00000106993	CDC37L1	0.283	5.794	2.498	0.014
ENSG00000107165	TYRP1	0.392	1.787	2.110	0.038
ENSG00000107175	CREB3	0.299	4.242	2.646	0.010
ENSG00000107223	EDF1	0.340	5.705	2.591	0.011
ENSG00000107249	GLIS3	-0.521	3.892	-3.259	0.002
ENSG00000107262	BAG1	0.349	4.687	3.276	0.002
ENSG00000107290	SETX	0.131	8.021	2.188	0.032
ENSG00000107362	ABHD17B	0.284	4.892	2.776	0.007
ENSG00000107438	PDLIM1	0.544	-0.413	2.115	0.037
ENSG00000107443	CCNJ	-0.196	3.088	-2.225	0.029
ENSG00000107554	DNMBP	-0.197	3.821	-3.159	0.002
ENSG00000107562	CXCL12	0.371	2.643	2.199	0.031
ENSG00000107581	EIF3A	0.148	7.816	2.192	0.031
ENSG00000107614	TRDMT1	-0.245	3.878	-3.618	0.001
ENSG00000107625	DDX50	0.267	5.209	2.210	0.030
ENSG00000107651	SEC23IP	0.112	5.402	2.415	0.018
ENSG00000107719	PALD1	0.306	3.258	2.413	0.018
ENSG00000107736	CDH23	-0.311	2.043	-2.161	0.034
ENSG00000107789	MINPP1	0.191	3.648	2.410	0.018

ENSG00000107815	C10orf2	0.210	2.388	2.281	0.025
ENSG00000107821	KAZALD1	0.769	-0.037	3.601	0.001
ENSG00000107833	NPM3	0.614	1.722	4.019	0.000
ENSG00000107854	TNKS2	0.167	6.703	2.087	0.040
ENSG00000107897	ACBD5	0.227	6.631	2.659	0.009
ENSG00000107937	GTPBP4	0.318	5.300	3.233	0.002
ENSG00000107949	BCCIP	0.194	4.863	2.544	0.013
ENSG00000107959	PITRM1	0.200	6.193	2.867	0.005
ENSG00000107960	OBFC1	0.325	3.460	3.267	0.002
ENSG00000108001	EBF3	-0.360	0.825	-2.014	0.047
ENSG00000108094	CUL2	0.262	6.028	2.641	0.010
ENSG00000108100	CCNY	0.255	6.735	2.922	0.004
ENSG00000108175	ZMIZ1	-0.233	5.963	-2.281	0.025
ENSG00000108176	DNAJC12	0.287	4.246	2.899	0.005
ENSG00000108239	TBC1D12	0.236	5.747	2.004	0.048
ENSG00000108255	CRYBA1	-0.490	-0.980	-2.018	0.047
ENSG00000108298	RPL19	0.442	6.516	2.273	0.026
ENSG00000108306	FBXL20	-0.128	6.934	-2.641	0.010
ENSG00000108349	CASC3	-0.109	6.496	-2.172	0.033
ENSG00000108384	RAD51C	0.233	4.736	2.159	0.034
ENSG00000108389	MTMR4	0.166	7.004	2.755	0.007
ENSG00000108405	P2RX1	-0.786	-2.321	-2.224	0.029
ENSG00000108406	DHX40	0.197	5.195	2.750	0.007
ENSG00000108424	KPNB1	0.176	7.594	2.469	0.016
ENSG00000108468	CBX1	0.231	5.888	2.532	0.013
ENSG00000108474	PIGL	-0.183	3.935	-2.699	0.008
ENSG00000108557	RAI1	-0.253	4.916	-2.015	0.047
ENSG00000108561	C1QBP	0.361	5.566	2.920	0.005
ENSG00000108588	CCDC47	0.199	6.905	2.568	0.012
ENSG00000108622	ICAM2	0.545	-0.126	2.262	0.026
ENSG00000108666	C17orf75	0.249	5.011	2.396	0.019
ENSG00000108684	ASIC2	0.219	3.858	2.536	0.013
ENSG00000108788	MLX	0.223	3.386	2.927	0.004
ENSG00000108826	MRPL27	0.260	3.695	2.552	0.013
ENSG00000108861	DUSP3	0.250	7.294	2.840	0.006
ENSG00000108932	SLC16A6	0.276	3.377	2.156	0.034
ENSG00000108946	PRKAR1A	0.287	9.323	2.608	0.011
ENSG00000108953	YWHAE	0.250	8.775	2.494	0.015
ENSG00000108960	MMD	0.549	6.378	3.774	0.000
ENSG00000108984	MAP2K6	0.215	6.052	2.819	0.006
ENSG00000109016	DHRS7B	0.190	3.741	2.324	0.023
ENSG00000109079	TNFAIP1	0.156	5.720	2.087	0.040
ENSG00000109118	PHF12	-0.179	4.572	-2.892	0.005
ENSG00000109180	OCIAD1	0.156	6.997	2.041	0.045
ENSG00000109255	NMU	1.121	-0.767	3.020	0.003
ENSG00000109270	LAMTOR3	0.291	6.214	2.708	0.008
ENSG00000109332	UBE2D3	0.169	7.785	2.515	0.014

ENSG00000109390	NDUFC1	0.219	3.767	2.176	0.032
ENSG00000109445	ZNF330	0.209	4.237	1.997	0.049
ENSG00000109466	KLHL2	0.323	6.887	2.938	0.004
ENSG00000109475	RPL34	0.612	6.427	2.265	0.026
ENSG00000109501	WFS1	-0.343	4.770	-3.126	0.002
ENSG00000109519	GRPEL1	0.286	4.837	3.439	0.001
ENSG00000109572	CLCN3	0.223	8.313	2.734	0.008
ENSG00000109606	DHX15	0.156	6.614	2.438	0.017
ENSG00000109610	SOD3	-0.534	-0.198	-2.008	0.048
ENSG00000109670	FBXW7	0.232	9.185	2.183	0.032
ENSG00000109738	GLRB	0.274	6.688	2.111	0.038
ENSG00000109762	SNX25	0.268	5.397	2.629	0.010
ENSG00000109771	LRP2BP	-0.360	4.607	-4.060	0.000
ENSG00000109790	KLHL5	0.185	6.208	2.361	0.021
ENSG00000109819	PPARGC1A	-0.150	6.540	-2.378	0.020
ENSG00000109854	HTATIP2	0.257	3.244	2.488	0.015
ENSG00000109861	CTSC	0.309	3.251	2.252	0.027
ENSG00000109881	CCDC34	0.398	3.641	3.056	0.003
ENSG00000109906	ZBTB16	-0.213	5.936	-2.076	0.041
ENSG00000109917	ZPR1	0.178	5.967	2.223	0.029
ENSG00000109919	MTCH2	0.233	5.834	2.835	0.006
ENSG00000109929	SC5D	0.269	6.619	3.175	0.002
ENSG00000109971	HSPA8	0.336	10.571	2.928	0.004
ENSG00000110013	SIAE	0.226	5.262	2.967	0.004
ENSG00000110046	ATG2A	-0.215	3.626	-2.000	0.049
ENSG00000110075	PPP6R3	0.141	6.879	2.329	0.022
ENSG00000110092	CCND1	0.309	4.245	2.564	0.012
ENSG00000110200	ANAPC15	0.295	2.062	2.531	0.013
ENSG00000110321	EIF4G2	0.195	9.057	2.611	0.011
ENSG00000110330	BIRC2	0.171	6.050	2.275	0.026
ENSG00000110435	PDHX	0.220	6.188	2.095	0.039
ENSG00000110442	COMMD9	0.163	4.562	2.577	0.012
ENSG00000110455	ACCS	-0.321	2.444	-2.394	0.019
ENSG00000110619	CARS	0.291	5.099	3.931	0.000
ENSG00000110700	RPS13	0.483	4.365	2.246	0.027
ENSG00000110711	AIP	0.354	3.515	2.756	0.007
ENSG00000110723	EXPH5	0.318	4.566	2.490	0.015
ENSG00000110768	GTF2H1	0.226	5.837	2.143	0.035
ENSG00000110871	COQ5	0.264	3.521	2.892	0.005
ENSG00000110887	DAO	-0.563	-0.123	-2.324	0.023
ENSG00000110917	MLEC	0.220	6.572	3.165	0.002
ENSG00000110934	BIN2	0.601	1.582	2.175	0.033
ENSG00000110955	ATP5B	0.333	8.014	3.190	0.002
ENSG00000111011	RSRC2	0.176	6.933	2.702	0.008
ENSG00000111087	GLI1	-0.576	-0.732	-2.162	0.034
ENSG00000111142	METAP2	0.196	6.223	2.543	0.013
ENSG00000111144	LTA4H	0.207	5.379	2.512	0.014

ENSG00000111181	SLC6A12	0.352	2.678	2.773	0.007
ENSG00000111206	FOXM1	-0.448	-0.512	-2.077	0.041
ENSG00000111229	ARPC3	0.558	5.354	3.057	0.003
ENSG00000111231	GPN3	0.355	3.217	3.317	0.001
ENSG00000111237	VPS29	0.293	5.270	2.847	0.006
ENSG00000111254	AKAP3	0.304	1.224	2.036	0.045
ENSG00000111266	DUSP16	-0.141	5.557	-2.113	0.038
ENSG00000111276	CDKN1B	0.151	6.040	2.065	0.042
ENSG00000111339	ART4	-0.624	-0.121	-2.751	0.007
ENSG00000111344	RASAL1	0.231	4.040	2.002	0.049
ENSG00000111348	ARHGDIB	0.531	3.271	2.601	0.011
ENSG00000111412	C12orf49	0.220	6.276	2.816	0.006
ENSG00000111452	ADGRD1	0.403	1.209	2.412	0.018
ENSG00000111615	KRR1	0.150	6.222	2.084	0.040
ENSG00000111639	MRPL51	0.307	5.018	3.194	0.002
ENSG00000111647	UHRF1BP1L	0.200	7.231	2.165	0.033
ENSG00000111652	COPS7A	0.182	5.799	2.046	0.044
ENSG00000111653	ING4	0.333	4.320	4.121	0.000
ENSG00000111669	TPI1	0.436	7.680	3.170	0.002
ENSG00000111670	GNPTAB	0.148	7.481	2.574	0.012
ENSG00000111676	ATN1	-0.391	6.197	-2.674	0.009
ENSG00000111678	C12orf57	0.341	2.320	2.648	0.010
ENSG00000111711	GOLT1B	0.226	5.370	2.393	0.019
ENSG00000111716	LDHB	0.382	2.942	3.401	0.001
ENSG00000111725	PRKAB1	-0.137	3.520	-2.462	0.016
ENSG00000111726	CMAS	0.290	6.304	2.039	0.045
ENSG00000111727	HCFC2	0.142	5.866	2.359	0.021
ENSG00000111737	RAB35	0.284	4.232	3.915	0.000
ENSG00000111752	PHC1	-0.310	3.811	-2.654	0.010
ENSG00000111775	COX6A1	0.336	4.722	2.596	0.011
ENSG00000111783	RFX4	-0.390	4.318	-2.039	0.045
ENSG00000111786	SRSF9	0.123	4.047	2.148	0.035
ENSG00000111799	COL12A1	-0.254	5.480	-2.416	0.018
ENSG00000111802	TDP2	0.188	5.382	2.133	0.036
ENSG00000111832	RWDD1	0.202	6.034	2.238	0.028
ENSG00000111837	MAK	-0.340	2.945	-2.176	0.032
ENSG00000111843	TMEM14C	0.213	4.394	2.366	0.020
ENSG00000111845	PAK1IP1	0.429	4.025	3.250	0.002
ENSG00000111850	SMIM8	0.146	4.900	2.002	0.049
ENSG00000111907	TPD52L1	-0.462	4.659	-2.695	0.009
ENSG00000111911	HINT3	0.239	6.305	3.093	0.003
ENSG00000111962	UST	0.203	4.631	2.013	0.047
ENSG00000112031	MTRF1L	0.285	3.669	3.194	0.002
ENSG00000112038	OPRM1	-0.355	3.726	-2.817	0.006
ENSG00000112078	KCTD20	0.154	6.279	2.221	0.029
ENSG00000112081	SRSF3	0.222	6.912	2.905	0.005
ENSG00000112096	SOD2	0.284	7.928	2.429	0.017

ENSG00000112110	MRPL18	0.289	4.451	2.414	0.018
ENSG00000112130	RNF8	0.186	5.124	2.100	0.039
ENSG00000112175	BMP5	1.440	-1.805	3.715	0.000
ENSG00000112182	BACH2	-0.233	3.797	-2.927	0.004
ENSG00000112210	RAB23	0.238	4.660	2.444	0.017
ENSG00000112280	COL9A1	0.475	2.028	2.855	0.005
ENSG00000112297	AIM1	-0.356	2.863	-3.279	0.002
ENSG00000112305	SMAP1	0.210	4.851	2.162	0.034
ENSG00000112306	RPS12	0.660	5.186	2.295	0.024
ENSG00000112335	SNX3	0.213	6.954	2.367	0.020
ENSG00000112339	HBS1L	0.314	7.030	3.371	0.001
ENSG00000112357	PEX7	0.306	2.146	2.564	0.012
ENSG00000112367	FIG4	0.269	5.744	2.776	0.007
ENSG00000112414	ADGRG6	0.363	2.059	2.009	0.048
ENSG00000112419	PHACTR2	0.187	6.269	2.687	0.009
ENSG00000112473	SLC39A7	0.274	4.470	3.605	0.001
ENSG00000112559	MDFI	0.890	-1.277	3.908	0.000
ENSG00000112624	GLTSCR1L	-0.190	6.184	-3.598	0.001
ENSG00000112679	DUSP22	0.149	5.050	2.293	0.024
ENSG00000112685	EXOC2	0.185	5.693	2.031	0.046
ENSG00000112695	COX7A2	0.578	5.112	3.170	0.002
ENSG00000112697	TMEM30A	0.182	8.128	1.992	0.050
ENSG00000112706	IMPG1	0.314	1.528	2.419	0.018
ENSG00000112739	PRPF4B	0.144	6.914	2.690	0.009
ENSG00000112761	WISP3	-0.672	-1.046	-2.822	0.006
ENSG00000112763	BTN2A1	0.172	4.742	2.657	0.009
ENSG00000112837	TBX18	0.406	1.455	2.274	0.026
ENSG00000112855	HARS2	0.197	4.424	2.820	0.006
ENSG00000112874	NUDT12	0.287	4.815	2.556	0.012
ENSG00000112877	CEP72	0.171	1.950	2.039	0.045
ENSG00000112902	SEMA5A	0.180	6.049	2.277	0.025
ENSG00000112972	HMGCS1	0.548	7.621	4.276	0.000
ENSG00000112992	NNT	0.137	7.440	2.046	0.044
ENSG00000113013	HSPA9	0.347	8.065	2.935	0.004
ENSG00000113048	MRPS27	0.182	5.457	2.282	0.025
ENSG00000113068	PFDN1	0.276	4.932	2.856	0.005
ENSG00000113073	SLC4A9	-0.372	-0.515	-2.145	0.035
ENSG00000113141	IK	0.393	5.762	2.229	0.029
ENSG00000113161	HMGCR	0.310	7.036	2.724	0.008
ENSG00000113209	PCDHB5	-0.220	3.560	-2.003	0.049
ENSG00000113248	PCDHB15	-0.178	3.350	-2.406	0.018
ENSG00000113282	CLINT1	0.214	6.314	3.070	0.003
ENSG00000113312	TTC1	0.320	6.014	2.578	0.012
ENSG00000113318	MSH3	0.150	5.627	2.071	0.042
ENSG00000113328	CCNG1	0.241	4.947	2.636	0.010
ENSG00000113384	GOLPH3	0.211	5.538	2.971	0.004
ENSG00000113387	SUB1	0.376	8.282	2.417	0.018

ENSG00000113391	FAM172A	0.116	6.024	2.061	0.043
ENSG00000113407	TARS	0.286	5.690	2.872	0.005
ENSG00000113456	RAD1	0.161	5.083	2.033	0.045
ENSG00000113460	BRIX1	0.296	4.047	3.155	0.002
ENSG00000113525	IL5	-0.522	-0.051	-2.485	0.015
ENSG00000113532	ST8SIA4	0.315	4.334	2.612	0.011
ENSG00000113552	GNPDA1	0.212	4.656	2.583	0.012
ENSG00000113558	SKP1	0.172	7.664	2.116	0.037
ENSG00000113575	PPP2CA	0.223	7.649	2.388	0.019
ENSG00000113583	C5orf15	0.254	4.749	2.854	0.005
ENSG00000113595	TRIM23	0.298	7.097	2.597	0.011
ENSG00000113597	TRAPPC13	0.242	5.716	2.784	0.007
ENSG00000113621	TXNDC15	0.183	6.097	2.167	0.033
ENSG00000113638	TTC33	0.228	5.784	2.145	0.035
ENSG00000113643	RARS	0.193	5.901	2.124	0.037
ENSG00000113712	CSNK1A1	0.281	6.505	2.595	0.011
ENSG00000113719	ERGIC1	0.129	5.766	2.492	0.015
ENSG00000113734	BNIP1	0.257	2.435	2.271	0.026
ENSG00000113811	SELK	0.272	4.313	2.223	0.029
ENSG00000113845	TIMMDC1	0.293	5.263	2.884	0.005
ENSG00000113851	CRBN	0.182	6.785	2.382	0.020
ENSG00000113966	ARL6	0.376	5.519	3.039	0.003
ENSG00000113971	NPHP3	-0.351	2.814	-2.420	0.018
ENSG00000114013	CD86	0.778	0.956	2.156	0.034
ENSG00000114023	FAM162A	0.197	5.689	2.040	0.045
ENSG00000114098	ARMC8	0.151	6.274	2.080	0.041
ENSG00000114125	RNF7	0.330	3.869	3.928	0.000
ENSG00000114126	TFDP2	0.137	6.018	2.144	0.035
ENSG00000114200	BCHE	0.445	2.884	2.918	0.005
ENSG00000114209	PDCD10	0.253	5.308	2.577	0.012
ENSG00000114316	USP4	-0.154	5.253	-2.087	0.040
ENSG00000114346	ECT2	0.407	4.235	3.676	0.000
ENSG00000114354	TFG	0.103	5.307	2.361	0.021
ENSG00000114383	TUSC2	0.260	4.345	2.943	0.004
ENSG00000114388	NPRL2	0.255	3.225	3.232	0.002
ENSG00000114391	RPL24	0.754	5.769	2.385	0.019
ENSG00000114405	C3orf14	0.281	5.352	2.764	0.007
ENSG00000114423	CBLB	-0.134	4.387	-2.247	0.027
ENSG00000114446	IFT57	0.289	5.643	2.627	0.010
ENSG00000114450	GNB4	0.307	5.539	3.200	0.002
ENSG00000114480	GBE1	0.378	4.019	3.213	0.002
ENSG00000114503	NCBP2	0.146	5.982	2.205	0.030
ENSG00000114520	SNX4	0.347	5.453	3.461	0.001
ENSG00000114686	MRPL3	0.345	5.563	2.660	0.009
ENSG00000114739	ACVR2B	-0.121	5.588	-2.437	0.017
ENSG00000114744	COMMD2	0.260	4.933	3.154	0.002
ENSG00000114854	TNNC1	1.315	-3.093	3.263	0.002

ENSG00000114902	SPCS1	0.148	5.602	2.291	0.025
ENSG00000114933	INO80D	-0.168	6.520	-3.457	0.001
ENSG00000114942	EEF1B2	0.667	5.381	2.930	0.004
ENSG00000114956	DGUOK	0.161	3.074	2.025	0.046
ENSG00000114999	TTL	0.323	7.002	4.524	0.000
ENSG00000115041	KCNIP3	0.232	4.946	2.056	0.043
ENSG00000115053	NCL	0.196	8.206	2.402	0.019
ENSG00000115091	ACTR3	0.272	6.961	3.147	0.002
ENSG00000115128	SF3B6	0.347	3.729	2.746	0.007
ENSG00000115145	STAM2	0.153	6.281	2.273	0.026
ENSG00000115226	FNDC4	0.297	4.564	3.107	0.003
ENSG00000115233	PSMD14	0.349	5.688	2.845	0.006
ENSG00000115234	SNX17	0.199	4.971	2.142	0.035
ENSG00000115239	ASB3	-0.253	2.600	-3.284	0.002
ENSG00000115241	PPM1G	0.340	5.397	3.434	0.001
ENSG00000115252	PDE1A	0.288	7.075	2.226	0.029
ENSG00000115271	GCA	0.338	5.020	2.771	0.007
ENSG00000115307	AUP1	0.143	4.342	2.085	0.040
ENSG00000115310	RTN4	0.251	10.331	2.261	0.026
ENSG00000115317	HTRA2	0.327	1.972	3.088	0.003
ENSG00000115353	TACR1	-0.336	2.260	-2.881	0.005
ENSG00000115380	EFEMP1	-0.630	5.556	-2.359	0.021
ENSG00000115446	UNC50	0.190	4.539	2.723	0.008
ENSG00000115457	IGFBP2	0.532	2.391	3.081	0.003
ENSG00000115474	KCNJ13	-0.337	1.895	-2.578	0.012
ENSG00000115484	CCT4	0.287	6.144	2.713	0.008
ENSG00000115514	TXNDC9	0.349	3.892	2.681	0.009
ENSG00000115520	COQ10B	0.268	5.376	2.600	0.011
ENSG00000115524	SF3B1	0.164	8.428	2.417	0.018
ENSG00000115539	PDCL3	0.429	2.963	2.999	0.004
ENSG00000115568	ZNF142	-0.207	4.682	-2.390	0.019
ENSG00000115616	SLC9A2	0.467	2.216	3.317	0.001
ENSG00000115661	STK16	0.292	2.713	3.294	0.001
ENSG00000115677	HDLBP	0.209	8.069	2.828	0.006
ENSG00000115685	PPP1R7	0.380	5.866	3.408	0.001
ENSG00000115705	TPO	-0.724	-0.709	-2.432	0.017
ENSG00000115758	ODC1	0.340	4.725	2.955	0.004
ENSG00000115762	PLEKHB2	0.211	7.538	2.209	0.030
ENSG00000115816	CEBPZ	0.209	5.123	2.420	0.018
ENSG00000115825	PRKD3	-0.171	5.529	-2.225	0.029
ENSG00000115828	QPCT	0.622	2.619	3.459	0.001
ENSG00000115839	RAB3GAP1	0.199	7.398	2.040	0.045
ENSG00000115844	DLX2	-0.369	0.254	-2.006	0.048
ENSG00000115875	SRSF7	0.224	6.250	2.734	0.008
ENSG00000115884	SDC1	0.382	0.076	2.291	0.025
ENSG00000115946	PNO1	0.284	3.204	2.743	0.007
ENSG00000115963	RND3	0.430	4.302	2.532	0.013

ENSG00000116030	SUMO1	0.453	5.865	2.994	0.004
ENSG00000116120	FARSB	0.259	5.777	2.417	0.018
ENSG00000116128	BCL9	-0.240	4.631	-3.089	0.003
ENSG00000116161	CACYBP	0.304	6.810	2.169	0.033
ENSG00000116194	ANGPTL1	-0.376	2.756	-3.071	0.003
ENSG00000116199	FAM20B	0.210	7.373	2.347	0.021
ENSG00000116205	TCEANC2	0.120	4.903	2.042	0.044
ENSG00000116209	TMEM59	0.213	7.130	2.429	0.017
ENSG00000116237	ICMT	0.196	5.586	2.468	0.016
ENSG00000116288	PARK7	0.389	6.464	2.340	0.022
ENSG00000116299	KIAA1324	-0.199	5.250	-2.525	0.014
ENSG00000116396	KCNC4	-0.312	4.829	-2.837	0.006
ENSG00000116406	EDEM3	0.224	6.814	2.400	0.019
ENSG00000116459	ATP5F1	0.371	6.524	3.273	0.002
ENSG00000116489	CAPZA1	0.296	6.271	3.098	0.003
ENSG00000116586	LAMTOR2	0.282	2.762	2.476	0.015
ENSG00000116604	MEF2D	-0.250	6.578	-2.430	0.017
ENSG00000116652	DLEU2L	-0.315	2.556	-2.330	0.022
ENSG00000116679	IVNS1ABP	0.309	6.520	3.153	0.002
ENSG00000116698	SMG7	-0.256	6.069	-3.314	0.001
ENSG00000116701	NCF2	0.665	0.906	2.817	0.006
ENSG00000116703	PDC	-0.514	1.592	-3.164	0.002
ENSG00000116711	PLA2G4A	0.428	2.869	2.954	0.004
ENSG00000116717	GADD45A	0.364	3.586	2.119	0.037
ENSG00000116731	PRDM2	-0.161	7.762	-2.031	0.045
ENSG00000116752	BCAS2	0.367	5.478	2.218	0.029
ENSG00000116774	OLFML3	0.735	1.989	2.453	0.016
ENSG00000116819	TFAP2E	-0.367	0.998	-2.371	0.020
ENSG00000116863	ADPRHL2	0.357	2.843	3.373	0.001
ENSG00000116885	OSCP1	0.196	3.688	2.140	0.035
ENSG00000116898	MRPS15	0.306	4.327	3.117	0.003
ENSG00000116906	GNPAT	0.283	5.005	3.110	0.003
ENSG00000116918	TSNAX	0.262	5.883	2.626	0.010
ENSG00000116922	C1orf109	0.405	3.630	3.917	0.000
ENSG00000116983	HPCAL4	0.174	8.470	2.057	0.043
ENSG00000116991	SIPA1L2	0.164	7.099	2.040	0.045
ENSG00000117009	KMO	-0.285	2.017	-2.453	0.016
ENSG00000117020	AKT3	0.181	9.547	2.089	0.040
ENSG00000117118	SDHB	0.216	5.104	2.211	0.030
ENSG00000117133	RPF1	0.258	4.018	2.428	0.017
ENSG00000117143	UAP1	0.269	5.136	2.540	0.013
ENSG00000117152	RGS4	0.366	9.314	2.521	0.014
ENSG00000117153	KLHL12	0.250	5.860	2.516	0.014
ENSG00000117281	CD160	-0.460	0.982	-2.882	0.005
ENSG00000117362	APH1A	0.167	3.573	2.019	0.047
ENSG00000117395	EBNA1BP2	0.336	5.384	2.799	0.006
ENSG00000117410	ATP6V0B	0.421	3.943	2.842	0.006

ENSG00000117411	B4GALT2	0.417	4.354	2.947	0.004
ENSG00000117448	AKR1A1	0.246	3.941	2.621	0.010
ENSG00000117475	BLZF1	0.186	5.606	2.686	0.009
ENSG00000117481	NSUN4	0.113	4.129	2.027	0.046
ENSG00000117505	DR1	0.183	6.325	2.783	0.007
ENSG00000117528	ABCD3	0.222	6.110	2.390	0.019
ENSG00000117543	DPH5	0.213	3.368	2.806	0.006
ENSG00000117586	TNFSF4	-0.318	2.147	-2.911	0.005
ENSG00000117595	IRF6	0.743	-0.041	3.064	0.003
ENSG00000117602	RCAN3	0.297	3.251	2.141	0.035
ENSG00000117614	SYF2	0.263	4.707	2.412	0.018
ENSG00000117625	RCOR3	-0.172	6.200	-2.723	0.008
ENSG00000117632	STMN1	0.246	9.001	2.319	0.023
ENSG00000117650	NEK2	0.662	0.609	2.660	0.009
ENSG00000117682	DHDDS	0.112	4.985	2.097	0.039
ENSG00000117691	NENF	0.233	3.823	2.279	0.025
ENSG00000117697	NSL1	0.166	4.714	2.616	0.011
ENSG00000117713	ARID1A	-0.273	6.424	-3.391	0.001
ENSG00000117748	RPA2	0.358	4.594	2.839	0.006
ENSG00000117758	STX12	0.253	6.929	2.745	0.007
ENSG00000117862	TXNDC12	0.316	2.411	2.962	0.004
ENSG00000117906	RCN2	0.186	6.184	2.226	0.029
ENSG00000118046	STK11	0.217	4.491	2.389	0.019
ENSG00000118058	KMT2A	-0.253	8.008	-4.288	0.000
ENSG00000118245	TNP1	-0.952	-2.119	-2.715	0.008
ENSG00000118307	CASC1	0.217	3.517	2.179	0.032
ENSG00000118363	SPCS2	0.242	4.269	2.751	0.007
ENSG00000118402	ELOVL4	0.316	5.895	2.792	0.007
ENSG00000118407	FILIP1	0.313	4.750	2.275	0.026
ENSG00000118454	ANKRD13C	0.201	6.389	2.606	0.011
ENSG00000118508	RAB32	0.511	0.017	2.518	0.014
ENSG00000118640	VAMP8	0.708	0.126	2.981	0.004
ENSG00000118680	MYL12B	0.404	5.875	3.428	0.001
ENSG00000118689	FOXO3	-0.147	6.811	-2.538	0.013
ENSG00000118804	STBD1	0.291	0.449	2.071	0.042
ENSG00000118849	RARRES1	-0.350	1.214	-2.707	0.008
ENSG00000118855	MFSD1	0.199	4.552	2.042	0.044
ENSG00000118873	RAB3GAP2	0.224	6.907	2.403	0.019
ENSG00000118971	CCND2	0.255	8.518	2.929	0.004
ENSG00000118985	ELL2	0.262	7.107	3.202	0.002
ENSG00000119013	NDUFB3	0.677	3.723	3.268	0.002
ENSG00000119138	KLF9	0.177	7.219	2.083	0.040
ENSG00000119185	ITGB1BP1	0.207	5.607	2.471	0.016
ENSG00000119203	CPSF3	0.279	4.442	2.530	0.013
ENSG00000119227	PIGZ	-0.276	2.638	-2.248	0.027
ENSG00000119231	SENP5	0.164	6.854	2.395	0.019
ENSG00000119242	CCDC92	-0.200	6.270	-2.456	0.016

ENSG00000119318	RAD23B	0.208	7.049	2.747	0.007
ENSG00000119328	FAM206A	-0.156	3.833	-2.602	0.011
ENSG00000119335	SET	0.346	7.588	3.077	0.003
ENSG00000119383	PTPA	0.309	6.002	2.912	0.005
ENSG00000119392	GLE1	0.143	4.377	2.481	0.015
ENSG00000119421	NDUFA8	0.346	4.778	2.844	0.006
ENSG00000119446	RBM18	0.260	5.625	2.729	0.008
ENSG00000119487	MAPKAP1	0.159	6.142	2.486	0.015
ENSG00000119541	VPS4B	0.189	5.695	2.193	0.031
ENSG00000119596	YLPM1	-0.109	7.786	-2.122	0.037
ENSG00000119616	FCF1	0.322	3.585	3.847	0.000
ENSG00000119636	BBOF1	0.172	3.419	2.168	0.033
ENSG00000119638	NEK9	-0.149	5.229	-2.159	0.034
ENSG00000119661	DNAL1	0.265	6.505	2.927	0.004
ENSG00000119682	AREL1	0.185	6.009	2.809	0.006
ENSG00000119698	PPP4R4	0.294	6.238	2.235	0.028
ENSG00000119705	SLIRP	0.451	4.461	2.934	0.004
ENSG00000119725	ZNF410	-0.384	2.018	-3.237	0.002
ENSG00000119737	GPR75	-0.389	4.918	-3.408	0.001
ENSG00000119772	DNMT3A	-0.122	5.618	-2.248	0.027
ENSG00000119777	TMEM214	-0.191	3.571	-2.057	0.043
ENSG00000119782	FKBP1B	0.252	2.133	2.008	0.048
ENSG00000119801	YPEL5	0.233	7.167	2.232	0.028
ENSG00000119820	YIPF4	0.117	6.915	2.060	0.043
ENSG00000119844	AFTPH	0.202	6.703	2.243	0.028
ENSG00000119862	LGALSL	0.273	4.655	2.833	0.006
ENSG00000119865	CNRIP1	0.152	5.848	2.254	0.027
ENSG00000119866	BCL11A	-0.214	6.277	-2.389	0.019
ENSG00000119888	EPCAM	1.134	0.884	3.067	0.003
ENSG00000119912	IDE	0.135	5.303	2.242	0.028
ENSG00000119965	C10orf88	0.298	3.869	3.105	0.003
ENSG00000119969	HELLS	-0.280	3.057	-2.740	0.008
ENSG00000120049	KCNIP2	-0.205	4.705	-2.136	0.036
ENSG00000120057	SFRP5	-0.514	-1.357	-1.998	0.049
ENSG00000120137	PANK3	0.233	7.987	3.681	0.000
ENSG00000120156	TEK	0.263	4.359	2.372	0.020
ENSG00000120159	CAAP1	0.231	4.056	3.064	0.003
ENSG00000120210	INSL6	-0.499	0.105	-2.404	0.019
ENSG00000120265	PCMT1	0.357	7.149	2.564	0.012
ENSG00000120279	MYCT1	0.377	2.089	2.229	0.029
ENSG00000120327	PCDHB14	-0.156	4.505	-2.331	0.022
ENSG00000120328	PCDHB12	-0.164	3.686	-2.072	0.041
ENSG00000120329	SLC25A2	-0.522	-1.441	-2.132	0.036
ENSG00000120437	ACAT2	0.715	4.139	3.612	0.001
ENSG00000120438	TCP1	0.426	6.750	2.542	0.013
ENSG00000120526	NUDCD1	0.288	4.889	2.740	0.008
ENSG00000120549	KIAA1217	-0.146	6.257	-2.004	0.048

ENSG00000120616	EPC1	-0.163	5.576	-3.101	0.003
ENSG00000120656	TAF12	0.338	3.222	2.900	0.005
ENSG00000120685	PROSER1	-0.126	4.472	-2.180	0.032
ENSG00000120686	UFM1	0.303	6.601	3.342	0.001
ENSG00000120688	WBP4	0.241	5.596	2.307	0.024
ENSG00000120693	SMAD9	-0.218	4.534	-2.157	0.034
ENSG00000120697	ALG5	0.280	2.888	2.292	0.025
ENSG00000120705	ETF1	0.248	6.052	2.770	0.007
ENSG00000120708	TGFBI	0.772	1.599	2.456	0.016
ENSG00000120709	FAM53C	0.164	5.427	3.015	0.003
ENSG00000120725	SIL1	0.170	3.768	2.439	0.017
ENSG00000120727	PAIP2	0.259	5.991	2.884	0.005
ENSG00000120729	MYOT	-0.263	2.624	-2.328	0.022
ENSG00000120756	PLS1	0.251	3.486	2.402	0.019
ENSG00000120798	NR2C1	-0.144	5.228	-2.657	0.009
ENSG00000120805	ARL1	0.252	6.926	2.653	0.010
ENSG00000120837	NFYB	0.289	5.614	2.324	0.023
ENSG00000120907	ADRA1A	-0.275	4.490	-3.050	0.003
ENSG00000120925	RNF170	0.224	5.588	2.915	0.005
ENSG00000120948	TARDBP	-0.163	5.356	-2.033	0.045
ENSG00000120963	ZNF706	-0.149	6.247	-2.248	0.027
ENSG00000120992	LYPLA1	0.332	4.179	3.470	0.001
ENSG00000121022	COPS5	0.271	4.992	3.345	0.001
ENSG00000121058	COIL	0.246	4.251	3.042	0.003
ENSG00000121067	SPOP	0.215	5.605	2.547	0.013
ENSG00000121073	SLC35B1	0.294	4.072	3.518	0.001
ENSG00000121207	LRAT	-0.557	1.347	-2.738	0.008
ENSG00000121211	MND1	0.472	-0.016	2.210	0.030
ENSG00000121281	ADCY7	0.446	1.833	3.253	0.002
ENSG00000121310	ECHDC2	-0.347	3.761	-2.713	0.008
ENSG00000121350	PYROXD1	0.291	3.759	2.363	0.021
ENSG00000121361	KCNJ8	0.277	2.109	2.235	0.028
ENSG00000121410	A1BG	-0.403	-0.406	-2.224	0.029
ENSG00000121446	RGSL1	-0.960	-2.015	-2.887	0.005
ENSG00000121454	LHX4	-0.374	2.271	-2.594	0.011
ENSG00000121577	POPDC2	-0.387	-0.817	-2.177	0.032
ENSG00000121579	NAA50	0.228	6.576	3.070	0.003
ENSG00000121653	MAPK8IP1	0.213	6.100	2.206	0.030
ENSG00000121766	ZCCHC17	0.349	5.780	3.307	0.001
ENSG00000121769	FABP3	0.399	6.072	3.113	0.003
ENSG00000121775	TMEM39B	-0.183	2.141	-2.132	0.036
ENSG00000121851	POLR3GL	0.416	2.875	3.765	0.000
ENSG00000121864	ZNF639	0.239	5.267	3.033	0.003
ENSG00000122008	POLK	0.122	5.951	2.122	0.037
ENSG00000122026	RPL21	0.598	4.135	2.450	0.016
ENSG00000122033	MTIF3	0.158	4.791	2.036	0.045
ENSG00000122034	GTF3A	0.331	5.207	2.804	0.006

ENSG00000122068	FYTTD1	0.251	6.249	3.186	0.002
ENSG00000122218	COPA	0.142	7.644	2.044	0.044
ENSG00000122257	RBBP6	-0.135	6.638	-2.757	0.007
ENSG00000122406	RPL5	0.549	6.579	2.401	0.019
ENSG00000122420	PTGFR	0.470	0.799	2.481	0.015
ENSG00000122490	PQLC1	0.200	4.562	2.052	0.043
ENSG00000122545	SEPTIN7	0.303	9.103	2.703	0.008
ENSG00000122550	KLHL7	0.270	5.640	2.936	0.004
ENSG00000122565	CBX3	0.311	5.571	2.180	0.032
ENSG00000122574	WIPF3	-0.377	5.350	-3.363	0.001
ENSG00000122591	FAM126A	0.302	5.320	2.860	0.005
ENSG00000122643	NT5C3A	0.304	3.831	2.834	0.006
ENSG00000122687	MRM2	0.235	3.856	2.616	0.011
ENSG00000122692	SMU1	0.301	6.362	2.547	0.013
ENSG00000122694	GLIPR2	0.381	1.588	2.046	0.044
ENSG00000122705	CLTA	0.282	5.965	2.692	0.009
ENSG00000122756	CNTFR	-0.287	4.049	-1.990	0.050
ENSG00000122786	CALD1	0.206	6.014	2.696	0.009
ENSG00000122861	PLAU	0.656	-1.477	2.623	0.010
ENSG00000122862	SRGN	0.617	3.364	2.509	0.014
ENSG00000122873	CISD1	0.374	5.275	2.567	0.012
ENSG00000122912	SLC25A16	0.195	5.833	2.708	0.008
ENSG00000122952	ZWINT	0.438	1.075	2.640	0.010
ENSG00000122970	IFT81	0.186	4.973	2.103	0.039
ENSG00000123064	DDX54	0.241	3.806	3.093	0.003
ENSG00000123066	MED13L	-0.097	7.742	-2.955	0.004
ENSG00000123091	RNF11	0.226	8.272	2.662	0.009
ENSG00000123106	CCDC91	0.240	6.148	2.130	0.036
ENSG00000123136	DDX39A	0.363	2.560	2.883	0.005
ENSG00000123144	C19orf43	0.180	4.538	2.083	0.040
ENSG00000123154	WDR83	0.627	1.226	4.356	0.000
ENSG00000123240	OPTN	0.194	8.010	2.154	0.034
ENSG00000123297	TSFM	0.164	4.188	2.005	0.048
ENSG00000123338	NCKAP1L	0.514	2.930	2.111	0.038
ENSG00000123349	PFDN5	0.321	6.020	2.339	0.022
ENSG00000123395	ATG101	0.490	2.915	3.990	0.000
ENSG00000123411	IKZF4	-0.301	3.272	-3.070	0.003
ENSG00000123415	SMUG1	0.173	3.408	2.736	0.008
ENSG00000123427	METTL21B	-0.424	0.427	-2.052	0.043
ENSG00000123444	KBTBD4	0.192	2.849	2.044	0.044
ENSG00000123505	AMD1	0.191	7.437	2.303	0.024
ENSG00000123545	NDUFAF4	0.328	5.893	2.122	0.037
ENSG00000123552	USP45	0.179	6.007	2.442	0.017
ENSG00000123600	METTL8	0.147	5.147	2.325	0.023
ENSG00000123684	LPGAT1	0.227	8.110	2.228	0.029
ENSG00000123737	EXOSC9	0.254	4.287	2.909	0.005
ENSG00000123975	CKS2	0.569	0.192	2.590	0.011

ENSG00000124003	MOGAT1	-0.430	-0.956	-2.084	0.040
ENSG00000124019	FAM124B	0.843	-0.295	3.088	0.003
ENSG00000124091	GCNT7	-0.360	0.378	-2.303	0.024
ENSG00000124134	KCNS1	-0.296	5.733	-1.995	0.049
ENSG00000124145	SDC4	-0.634	5.014	-2.433	0.017
ENSG00000124172	ATP5E	0.530	6.025	2.696	0.009
ENSG00000124205	EDN3	0.439	1.471	2.069	0.042
ENSG00000124207	CSE1L	0.275	6.490	2.616	0.011
ENSG00000124215	CDH26	-0.346	2.046	-2.424	0.018
ENSG00000124222	STX16	-0.306	5.077	-3.882	0.000
ENSG00000124224	PPP4R1L	-0.384	1.908	-3.236	0.002
ENSG00000124228	DDX27	0.199	4.238	2.239	0.028
ENSG00000124383	MPHOSPH10	0.253	5.313	2.660	0.009
ENSG00000124440	HIF3A	-0.524	3.368	-2.345	0.021
ENSG00000124444	ZNF576	0.180	3.063	2.259	0.027
ENSG00000124535	WRNIP1	0.163	4.965	2.528	0.013
ENSG00000124541	RRP36	0.239	4.269	2.796	0.006
ENSG00000124562	SNRPC	0.522	3.100	3.856	0.000
ENSG00000124596	OARD1	0.152	4.322	2.052	0.043
ENSG00000124608	AARS2	0.193	3.781	2.407	0.018
ENSG00000124614	RPS10	0.666	1.552	2.352	0.021
ENSG00000124635	HIST1H2BJ	0.403	0.779	2.370	0.020
ENSG00000124659	TBCC	0.268	3.839	2.547	0.013
ENSG00000124678	TCP11	-0.586	-1.837	-2.095	0.039
ENSG00000124702	KLHDC3	0.284	6.758	2.355	0.021
ENSG00000124733	MEA1	0.225	4.958	2.359	0.021
ENSG00000124766	SOX4	0.365	3.044	2.889	0.005
ENSG00000124767	GLO1	0.243	6.446	2.685	0.009
ENSG00000124784	RIOK1	0.297	3.963	2.700	0.008
ENSG00000124785	NRN1	0.293	7.374	2.349	0.021
ENSG00000124786	SLC35B3	0.265	3.408	2.751	0.007
ENSG00000124787	RPP40	0.353	1.643	2.176	0.032
ENSG00000124802	EEF1E1	0.475	2.516	3.330	0.001
ENSG00000124813	RUNX2	-0.238	2.807	-2.257	0.027
ENSG00000124827	GCM2	-0.409	0.624	-1.990	0.050
ENSG00000124835	AC105760.3	-0.662	-0.613	-2.741	0.008
ENSG00000124942	AHNAK	-0.436	8.870	-2.821	0.006
ENSG00000125037	EMC3	0.350	5.032	3.401	0.001
ENSG00000125245	GPR18	-0.430	2.244	-2.963	0.004
ENSG00000125246	CLYBL	-0.318	3.608	-2.463	0.016
ENSG00000125304	TM9SF2	0.263	6.811	2.977	0.004
ENSG00000125386	FAM193A	-0.128	5.596	-2.085	0.040
ENSG00000125388	GRK4	0.204	4.694	2.406	0.018
ENSG00000125398	SOX9	-0.562	5.484	-2.642	0.010
ENSG00000125445	MRPS7	0.259	4.506	2.725	0.008
ENSG00000125454	SLC25A19	0.245	1.016	2.197	0.031
ENSG00000125482	TTF1	0.189	4.474	2.145	0.035

ENSG00000125505	MBOAT7	-0.249	5.195	-2.058	0.043
ENSG00000125629	INSIG2	0.207	5.529	2.227	0.029
ENSG00000125631	HTR5BP	-0.462	-1.562	-2.507	0.014
ENSG00000125691	RPL23	0.476	6.025	2.994	0.004
ENSG00000125703	ATG4C	0.243	5.059	2.386	0.019
ENSG00000125743	SNRPD2	0.384	4.556	3.119	0.003
ENSG00000125744	RTN2	0.292	4.007	2.441	0.017
ENSG00000125746	EML2	0.197	4.533	2.465	0.016
ENSG00000125753	VASP	0.315	1.830	2.501	0.014
ENSG00000125779	PANK2	0.231	5.565	3.234	0.002
ENSG00000125810	CD93	0.650	1.623	2.077	0.041
ENSG00000125817	CENPB	0.209	4.925	2.130	0.036
ENSG00000125820	NKX2-2	-0.429	0.495	-2.124	0.037
ENSG00000125835	SNRPB	0.305	2.150	2.506	0.014
ENSG00000125845	BMP2	0.253	1.795	2.507	0.014
ENSG00000125870	SNRPB2	0.430	4.559	3.292	0.001
ENSG00000125877	ITPA	0.365	2.832	2.982	0.004
ENSG00000125901	MRPS26	0.235	3.171	2.093	0.039
ENSG00000125944	HNRNPR	0.150	7.221	2.207	0.030
ENSG00000125945	ZNF436	0.241	5.636	2.905	0.005
ENSG00000125977	EIF2S2	0.546	5.087	2.974	0.004
ENSG00000125991	ERGIC3	0.260	4.692	2.357	0.021
ENSG00000125995	ROMO1	0.768	1.208	2.895	0.005
ENSG00000126003	PLAGL2	-0.412	2.730	-3.438	0.001
ENSG00000126088	UROD	0.323	4.627	2.510	0.014
ENSG00000126091	ST3GAL3	-0.287	3.379	-2.206	0.030
ENSG00000126249	PDCD2L	0.268	1.434	2.161	0.034
ENSG00000126259	KIRREL2	0.762	-0.431	3.917	0.000
ENSG00000126261	UBA2	0.361	6.360	2.778	0.007
ENSG00000126267	COX6B1	0.311	4.901	2.252	0.027
ENSG00000126368	NR1D1	-0.504	3.988	-2.702	0.008
ENSG00000126432	PRDX5	0.308	6.040	2.466	0.016
ENSG00000126457	PRMT1	0.409	4.385	2.509	0.014
ENSG00000126464	PRR12	-0.378	4.357	-2.517	0.014
ENSG00000126524	SBDS	0.236	6.664	2.703	0.008
ENSG00000126581	BECN1	0.275	5.637	2.754	0.007
ENSG00000126698	DNAJC8	0.328	5.875	2.695	0.009
ENSG00000126705	AHDC1	-0.316	4.381	-2.199	0.031
ENSG00000126821	SGPP1	0.312	4.995	3.159	0.002
ENSG00000126838	PZP	-0.400	2.670	-2.772	0.007
ENSG00000126856	PRDM7	-0.729	-2.607	-2.174	0.033
ENSG00000126858	RHOT1	0.223	6.705	2.554	0.013
ENSG00000126883	NUP214	-0.223	5.329	-3.663	0.000
ENSG00000126934	MAP2K2	0.230	4.879	2.316	0.023
ENSG00000127022	CANX	0.243	8.972	2.707	0.008
ENSG00000127080	IPPK	0.286	2.622	2.620	0.010
ENSG00000127084	FGD3	0.489	0.442	2.466	0.016

ENSG00000127125	PPCS	0.369	4.015	3.443	0.001
ENSG00000127184	COX7C	0.386	6.028	2.510	0.014
ENSG00000127252	HRASLS	0.304	2.805	3.172	0.002
ENSG00000127337	YEATS4	0.431	3.164	2.880	0.005
ENSG00000127362	TAS2R3	-0.486	1.623	-3.864	0.000
ENSG00000127364	TAS2R4	-0.310	2.802	-2.768	0.007
ENSG00000127366	TAS2R5	-0.480	0.696	-2.782	0.007
ENSG00000127527	EPS15L1	-0.299	4.619	-3.129	0.002
ENSG00000127540	UQCR11	0.363	3.388	2.777	0.007
ENSG00000127561	SYNGR3	0.209	4.784	2.321	0.023
ENSG00000127588	GNG13	-0.610	-1.541	-2.090	0.040
ENSG00000127831	VIL1	-0.956	-1.786	-3.576	0.001
ENSG00000127837	AAMP	0.176	5.015	2.176	0.032
ENSG00000127870	RNF6	0.325	6.757	2.897	0.005
ENSG00000127884	ECHS1	0.345	4.745	2.712	0.008
ENSG00000127922	SHFM1	0.187	4.616	2.119	0.037
ENSG00000127947	PTPN12	-0.169	5.896	-2.739	0.008
ENSG00000127951	FGL2	0.401	3.506	2.270	0.026
ENSG00000127955	GNAI1	0.255	6.740	2.353	0.021
ENSG00000127995	CASD1	0.245	6.831	2.251	0.027
ENSG00000128266	GNAZ	0.165	6.416	2.147	0.035
ENSG00000128268	MGAT3	0.125	6.151	2.031	0.045
ENSG00000128272	ATF4	0.454	6.411	4.611	0.000
ENSG00000128285	MCHR1	0.379	3.498	2.420	0.018
ENSG00000128463	EMC4	0.235	5.368	2.389	0.019
ENSG00000128482	RNF112	0.243	4.808	2.996	0.004
ENSG00000128563	PRKRIP1	0.177	3.810	2.421	0.018
ENSG00000128590	DNAJB9	0.271	5.521	2.971	0.004
ENSG00000128595	CALU	0.283	6.273	3.771	0.000
ENSG00000128607	KLHDC10	0.137	6.714	2.311	0.023
ENSG00000128609	NDUFA5	0.396	7.665	2.510	0.014
ENSG00000128626	MRPS12	0.327	1.008	2.306	0.024
ENSG00000128641	MYO1B	0.284	5.860	2.371	0.020
ENSG00000128654	MTX2	0.367	4.938	2.896	0.005
ENSG00000128656	CHN1	0.263	10.085	2.107	0.038
ENSG00000128833	MYO5C	-0.221	4.111	-2.085	0.040
ENSG00000128951	DUT	0.375	4.272	2.280	0.025
ENSG00000128989	ARPP19	0.400	9.683	3.627	0.000
ENSG00000129048	ACKR4	-0.472	0.828	-2.873	0.005
ENSG00000129071	MBD4	0.151	4.942	2.137	0.036
ENSG00000129083	COPB1	0.275	5.659	2.947	0.004
ENSG00000129084	PSMA1	0.213	3.723	2.177	0.032
ENSG00000129103	SUMF2	0.267	4.244	3.227	0.002
ENSG00000129116	PALLD	-0.231	5.328	-2.067	0.042
ENSG00000129128	SPCS3	0.247	7.126	2.790	0.007
ENSG00000129159	KCNC1	-0.277	6.016	-2.663	0.009
ENSG00000129244	ATP1B2	-0.469	7.050	-2.230	0.028

ENSG00000129255	MPDU1	0.244	2.745	2.531	0.013
ENSG00000129315	CCNT1	0.161	6.125	2.756	0.007
ENSG00000129347	KRI1	0.421	3.194	3.592	0.001
ENSG00000129355	CDKN2D	0.361	4.492	3.587	0.001
ENSG00000129451	KLK10	-0.857	-2.161	-2.267	0.026
ENSG00000129460	NGDN	0.295	3.811	2.309	0.023
ENSG00000129473	BCL2L2	0.140	7.125	2.030	0.046
ENSG00000129515	SNX6	0.300	5.387	2.673	0.009
ENSG00000129518	EAPP	0.260	4.896	2.366	0.020
ENSG00000129534	MIS18BP1	0.233	4.684	2.459	0.016
ENSG00000129596	CDO1	0.254	4.733	2.438	0.017
ENSG00000129636	ITFG1	0.336	7.068	2.401	0.019
ENSG00000129654	FOXJ1	-0.991	-1.401	-2.662	0.009
ENSG00000129810	SGO1	-0.548	-1.028	-2.079	0.041
ENSG00000129925	TMEM8A	0.309	2.581	2.550	0.013
ENSG00000129933	MAU2	-0.164	4.500	-2.024	0.046
ENSG00000129993	CBFA2T3	-0.335	1.995	-2.083	0.040
ENSG00000130023	ERMARD	-0.167	4.169	-2.333	0.022
ENSG00000130035	GALNT8	0.188	3.940	2.288	0.025
ENSG00000130037	KCNA5	-0.281	1.962	-2.000	0.049
ENSG00000130176	CNN1	0.500	1.061	2.789	0.007
ENSG00000130204	TOMM40	0.330	3.549	2.990	0.004
ENSG00000130244	FAM98C	0.225	3.247	2.679	0.009
ENSG00000130255	RPL36	0.465	4.768	2.133	0.036
ENSG00000130300	PLVAP	0.880	-0.262	2.372	0.020
ENSG00000130309	COLGALT1	0.166	3.564	2.358	0.021
ENSG00000130332	LSM7	0.340	1.243	2.840	0.006
ENSG00000130349	C6orf203	0.208	3.677	2.212	0.030
ENSG00000130511	SSBP4	0.387	2.332	2.493	0.015
ENSG00000130520	LSM4	0.291	4.609	2.978	0.004
ENSG00000130528	HRC	0.551	0.039	2.710	0.008
ENSG00000130558	OLFM1	0.337	9.003	3.067	0.003
ENSG00000130560	UBAC1	0.255	4.293	2.214	0.030
ENSG00000130595	TNNT3	0.756	-1.744	2.483	0.015
ENSG00000130707	ASS1	0.532	3.714	2.765	0.007
ENSG00000130713	EXOSC2	0.256	4.241	3.009	0.003
ENSG00000130714	POMT1	0.162	4.136	2.304	0.024
ENSG00000130724	CHMP2A	0.281	4.952	2.854	0.005
ENSG00000130755	GMFG	0.462	0.752	2.089	0.040
ENSG00000130770	ATPIF1	0.228	5.791	2.402	0.019
ENSG00000130772	MED18	0.302	1.551	2.595	0.011
ENSG00000130811	EIF3G	0.343	4.554	2.792	0.007
ENSG00000130818	ZNF426	0.126	5.005	2.022	0.046
ENSG00000130935	NOL11	0.283	4.231	2.615	0.011
ENSG00000130956	HABP4	0.271	7.000	2.559	0.012
ENSG00000131016	AKAP12	0.192	9.278	2.889	0.005
ENSG00000131019	ULBP3	0.511	-0.731	2.774	0.007

ENSG00000131023	LATS1	-0.112	6.148	-2.907	0.005
ENSG00000131069	ACSS2	0.147	4.079	2.005	0.048
ENSG00000131095	GFAP	-0.607	9.891	-2.312	0.023
ENSG00000131100	ATP6V1E1	0.380	7.237	2.152	0.034
ENSG00000131127	ZNF141	0.139	5.031	2.425	0.018
ENSG00000131236	CAP1	0.382	5.927	3.048	0.003
ENSG00000131238	PPT1	0.198	6.330	2.348	0.021
ENSG00000131242	RAB11FIP4	0.158	7.941	2.236	0.028
ENSG00000131370	SH3BP5	0.172	3.726	2.061	0.042
ENSG00000131378	RFTN1	0.186	5.228	2.016	0.047
ENSG00000131408	NR1H2	0.220	4.200	2.716	0.008
ENSG00000131437	KIF3A	0.179	8.784	2.036	0.045
ENSG00000131469	RPL27	0.404	6.014	2.675	0.009
ENSG00000131473	ACLY	0.174	6.378	2.004	0.048
ENSG00000131475	VPS25	0.369	2.690	2.664	0.009
ENSG00000131495	NDUFA2	0.293	2.556	2.193	0.031
ENSG00000131503	ANKHD1	-0.420	2.597	-3.294	0.001
ENSG00000131508	UBE2D2	0.208	5.740	2.751	0.007
ENSG00000131558	EXOC4	0.161	6.568	2.348	0.021
ENSG00000131652	THOC6	0.334	0.961	2.331	0.022
ENSG00000131730	CKMT2	-0.353	2.630	-3.086	0.003
ENSG00000131747	TOP2A	1.094	-2.356	2.491	0.015
ENSG00000131771	PPP1R1B	-0.228	5.800	-2.104	0.038
ENSG00000131791	PRKAB2	0.233	5.452	2.549	0.013
ENSG00000131844	MCCC2	-0.171	5.028	-2.810	0.006
ENSG00000131871	VIMP	0.251	3.451	2.386	0.019
ENSG00000131943	C19orf12	0.187	5.533	2.565	0.012
ENSG00000131966	ACTR10	0.289	5.932	2.619	0.011
ENSG00000131969	ABHD12B	0.265	2.631	3.238	0.002
ENSG00000132170	PPARG	0.205	2.879	2.032	0.045
ENSG00000132204	LINC00470	-0.661	0.267	-3.011	0.003
ENSG00000132254	ARFIP2	0.193	4.732	2.524	0.014
ENSG00000132305	IMMT	0.191	6.301	2.611	0.011
ENSG00000132313	MRPL35	0.254	6.628	2.955	0.004
ENSG00000132321	IQCA1	-0.229	4.811	-2.280	0.025
ENSG00000132330	SCLY	-0.432	1.665	-2.898	0.005
ENSG00000132341	RAN	0.333	7.555	2.722	0.008
ENSG00000132357	CARD6	0.524	1.374	2.892	0.005
ENSG00000132405	TBC1D14	0.185	5.643	2.587	0.011
ENSG00000132423	COQ3	0.288	3.054	2.000	0.049
ENSG00000132432	SEC61G	0.674	3.062	3.057	0.003
ENSG00000132434	LANCL2	0.167	6.241	2.065	0.042
ENSG00000132467	UTP3	0.298	4.522	3.731	0.000
ENSG00000132470	ITGB4	-0.692	3.556	-2.445	0.017
ENSG00000132475	H3F3B	0.242	6.829	2.085	0.040
ENSG00000132478	UNK	-0.228	3.345	-3.423	0.001
ENSG00000132510	KDM6B	-0.322	2.889	-2.225	0.029

ENSG00000132581	SDF2	0.321	3.186	3.208	0.002
ENSG00000132603	NIP7	0.303	3.317	3.122	0.002
ENSG00000132622	HSPA12B	0.560	1.227	2.621	0.010
ENSG00000132646	PCNA	0.309	3.558	2.397	0.019
ENSG00000132661	NXT1	0.377	-0.361	2.142	0.035
ENSG00000132664	POLR3F	0.186	4.819	2.183	0.032
ENSG00000132676	DAP3	0.228	5.222	3.440	0.001
ENSG00000132716	DCAF8	-0.129	5.665	-2.465	0.016
ENSG00000132768	DPH2	0.208	2.696	2.999	0.004
ENSG00000132792	CTNNBL1	0.240	4.073	2.399	0.019
ENSG00000132824	SERINC3	0.316	8.362	3.391	0.001
ENSG00000132842	AP3B1	0.176	5.702	2.206	0.030
ENSG00000132849	PATJ	-0.153	5.205	-2.441	0.017
ENSG00000132855	ANGPTL3	-0.407	1.691	-2.683	0.009
ENSG00000132912	DCTN4	0.137	6.627	2.186	0.032
ENSG00000132938	MTUS2	-0.178	5.884	-2.082	0.040
ENSG00000132963	POMP	0.615	4.448	2.922	0.004
ENSG00000132964	CDK8	0.259	4.809	3.265	0.002
ENSG00000133107	TRPC4	0.260	3.221	2.468	0.016
ENSG00000133111	RFXAP	-0.236	3.239	-3.232	0.002
ENSG00000133112	TPT1	0.350	7.251	2.544	0.013
ENSG00000133116	KL	0.702	1.460	3.984	0.000
ENSG00000133119	RFC3	0.307	3.012	2.718	0.008
ENSG00000133226	SRRM1	-0.167	6.552	-3.068	0.003
ENSG00000133275	CSNK1G2	0.238	4.468	2.399	0.019
ENSG00000133313	CNDP2	0.208	6.590	2.348	0.021
ENSG00000133316	WDR74	0.256	2.820	2.759	0.007
ENSG00000133393	FOPNL	0.293	4.821	3.226	0.002
ENSG00000133398	MED10	0.232	3.491	2.518	0.014
ENSG00000133519	ZDHHC8P1	-0.374	2.098	-2.745	0.007
ENSG00000133574	GIMAP4	0.601	3.107	2.849	0.006
ENSG00000133606	MKRN1	0.202	6.182	2.315	0.023
ENSG00000133641	C12orf29	0.210	5.315	2.128	0.036
ENSG00000133657	ATP13A3	0.161	6.939	2.352	0.021
ENSG00000133665	DYDC2	0.657	0.096	2.826	0.006
ENSG00000133706	LARS	0.267	7.351	2.908	0.005
ENSG00000133742	CA1	-0.364	1.655	-2.296	0.024
ENSG00000133800	LYVE1	0.476	2.591	2.043	0.044
ENSG00000133835	HSD17B4	0.213	7.061	2.294	0.024
ENSG00000133872	SARAF	0.314	8.700	2.803	0.006
ENSG00000133935	C14orf1	0.186	3.449	2.041	0.044
ENSG00000133958	UNC79	-0.150	7.100	-2.497	0.015
ENSG00000133961	NUMB	-0.098	5.834	-2.091	0.040
ENSG00000133962	CATSPERB	0.532	-0.471	2.298	0.024
ENSG00000134001	EIF2S1	0.267	6.461	2.387	0.019
ENSG00000134007	ADAM20	-0.321	3.030	-2.546	0.013
ENSG00000134014	ELP3	0.240	6.052	2.779	0.007

ENSG00000134030	CTIF	-0.156	6.439	-2.069	0.042
ENSG00000134056	MRPS36	0.529	3.474	3.716	0.000
ENSG00000134057	CCNB1	0.357	3.288	3.399	0.001
ENSG00000134058	CDK7	0.426	3.273	2.992	0.004
ENSG00000134072	CAMK1	0.321	2.688	2.747	0.007
ENSG00000134077	THUMPD3	0.150	4.992	2.264	0.026
ENSG00000134086	VHL	0.163	5.218	2.659	0.009
ENSG00000134115	CNTN6	0.467	4.456	2.705	0.008
ENSG00000134153	EMC7	0.239	3.665	2.258	0.027
ENSG00000134245	WNT2B	-0.235	4.092	-2.374	0.020
ENSG00000134248	LAMTOR5	0.419	3.116	3.368	0.001
ENSG00000134253	TRIM45	-0.204	2.564	-2.244	0.028
ENSG00000134265	NAPG	0.259	7.681	2.268	0.026
ENSG00000134278	SPIRE1	0.134	7.320	2.074	0.041
ENSG00000134294	SLC38A2	0.287	7.297	2.412	0.018
ENSG00000134308	YWHAQ	0.194	7.141	1.993	0.050
ENSG00000134313	KIDINS220	0.172	9.181	2.295	0.024
ENSG00000134318	ROCK2	0.200	8.804	2.236	0.028
ENSG00000134333	LDHA	0.439	7.918	3.693	0.000
ENSG00000134343	ANO3	0.330	6.526	2.415	0.018
ENSG00000134375	TIMM17A	0.709	4.854	3.754	0.000
ENSG00000134419	RPS15A	0.533	4.129	2.416	0.018
ENSG00000134440	NARS	0.318	7.622	2.906	0.005
ENSG00000134443	GRP	0.756	-2.159	2.442	0.017
ENSG00000134452	FBXO18	0.176	4.982	2.579	0.012
ENSG00000134453	RBM17	0.151	6.036	2.178	0.032
ENSG00000134684	YARS	0.215	6.511	2.258	0.027
ENSG00000134690	CDCA8	-0.312	0.520	-2.022	0.046
ENSG00000134697	GNL2	0.329	4.595	3.131	0.002
ENSG00000134698	AGO4	-0.140	5.716	-2.914	0.005
ENSG00000134717	BTF3L4	0.213	5.868	2.529	0.013
ENSG00000134744	ZCCHC11	-0.106	6.689	-2.022	0.046
ENSG00000134757	DSG3	1.093	-0.034	2.832	0.006
ENSG00000134809	TIMM10	0.412	3.494	3.447	0.001
ENSG00000134852	CLOCK	0.138	7.416	2.444	0.017
ENSG00000134853	PDGFRA	0.505	6.229	4.041	0.000
ENSG00000134882	UBAC2	0.119	4.661	2.004	0.048
ENSG00000134900	TPP2	0.151	6.366	1.990	0.050
ENSG00000134910	STT3A	0.189	5.334	2.650	0.010
ENSG00000134954	ETS1	0.209	4.454	2.209	0.030
ENSG00000134970	TMED7	0.209	6.207	2.366	0.020
ENSG00000134982	APC	0.171	10.238	2.169	0.033
ENSG00000134987	WDR36	0.173	5.930	2.264	0.026
ENSG00000134996	OSTF1	0.479	4.932	3.310	0.001
ENSG00000135002	RFK	0.293	5.929	1.998	0.049
ENSG00000135045	C9orf40	0.218	3.157	2.087	0.040
ENSG00000135047	CTSL	0.367	5.048	3.479	0.001

ENSG00000135049	AGTPBP1	0.248	7.733	2.439	0.017
ENSG00000135070	ISCA1	0.200	6.001	2.004	0.048
ENSG00000135083	CCNJL	-0.301	0.769	-2.032	0.045
ENSG00000135116	HRK	0.361	4.559	3.138	0.002
ENSG00000135127	BICDL1	0.278	4.814	2.812	0.006
ENSG00000135148	TRAFD1	0.249	3.987	3.033	0.003
ENSG00000135211	TMEM60	0.392	2.145	3.042	0.003
ENSG00000135218	CD36	0.464	0.876	3.092	0.003
ENSG00000135241	PNPLA8	0.268	6.830	2.802	0.006
ENSG00000135269	TES	0.414	0.586	2.228	0.029
ENSG00000135317	SNX14	0.217	6.429	2.228	0.029
ENSG00000135363	LMO2	0.399	3.391	2.219	0.029
ENSG00000135378	PRRG4	0.648	-0.358	2.006	0.048
ENSG00000135387	CAPRIN1	0.152	7.773	2.154	0.034
ENSG00000135392	DNAJC14	0.253	3.530	3.320	0.001
ENSG00000135404	CD63	0.415	5.174	2.632	0.010
ENSG00000135472	FAIM2	-0.192	8.354	-2.107	0.038
ENSG00000135486	HNRNPA1	0.287	6.685	2.561	0.012
ENSG00000135506	OS9	0.203	6.355	2.555	0.012
ENSG00000135535	CD164	0.161	6.355	2.095	0.039
ENSG00000135540	NHSL1	-0.345	4.678	-2.534	0.013
ENSG00000135549	PKIB	0.371	1.957	2.021	0.047
ENSG00000135597	REPS1	-0.186	6.463	-2.921	0.005
ENSG00000135624	CCT7	0.298	6.212	2.626	0.010
ENSG00000135643	KCNMB4	0.185	6.512	2.277	0.025
ENSG00000135698	MPHOSPH6	0.197	4.751	2.384	0.019
ENSG00000135720	DYNC1LI2	0.198	8.271	2.214	0.030
ENSG00000135766	EGLN1	0.116	6.720	2.366	0.020
ENSG00000135829	DHX9	0.257	6.896	2.401	0.019
ENSG00000135838	NPL	-0.347	3.540	-2.312	0.023
ENSG00000135900	MRPL44	0.419	3.941	3.401	0.001
ENSG00000135912	TTLL4	-0.320	3.601	-3.072	0.003
ENSG00000135913	USP37	0.151	5.054	2.571	0.012
ENSG00000135914	HTR2B	-0.434	1.512	-3.238	0.002
ENSG00000135930	EIF4E2	0.271	5.721	3.237	0.002
ENSG00000135932	CAB39	0.244	7.833	2.403	0.019
ENSG00000135953	MFSD9	0.193	2.656	2.253	0.027
ENSG00000135972	MRPS9	0.298	4.492	2.924	0.004
ENSG00000135976	ANKRD36	-0.316	7.472	-2.710	0.008
ENSG00000136021	SCYL2	0.184	6.304	2.640	0.010
ENSG00000136026	CKAP4	0.267	4.344	3.384	0.001
ENSG00000136045	PWP1	0.272	5.735	2.571	0.012
ENSG00000136048	DRAM1	0.277	1.168	2.165	0.033
ENSG00000136051	KIAA1033	0.221	6.427	2.460	0.016
ENSG00000136100	VPS36	0.193	6.673	2.661	0.009
ENSG00000136143	SUCLA2	0.429	6.787	2.954	0.004
ENSG00000136155	SCEL	0.610	-1.544	2.006	0.048

ENSG00000136159	NUDT15	0.246	3.338	2.496	0.015
ENSG00000136167	LCP1	0.595	2.780	2.253	0.027
ENSG00000136238	RAC1	0.142	6.519	2.020	0.047
ENSG00000136240	KDELR2	0.179	4.835	3.126	0.002
ENSG00000136243	NUPL2	0.177	4.240	2.284	0.025
ENSG00000136247	ZDHHC4	0.296	3.595	2.816	0.006
ENSG00000136270	TBRG4	0.219	3.422	2.424	0.018
ENSG00000136274	NACAD	-0.327	4.991	-2.319	0.023
ENSG00000136279	DBNL	0.144	6.072	2.206	0.030
ENSG00000136280	CCM2	0.314	4.775	2.577	0.012
ENSG00000136371	MTHFS	-0.305	-0.365	-2.179	0.032
ENSG00000136404	TM6SF1	0.327	3.728	2.779	0.007
ENSG00000136450	SRSF1	0.185	4.793	2.403	0.019
ENSG00000136463	TACO1	0.234	3.253	2.403	0.019
ENSG00000136492	BRIP1	-0.310	1.411	-2.282	0.025
ENSG00000136521	NDUFB5	0.223	6.764	2.708	0.008
ENSG00000136522	MRPL47	0.428	4.053	3.272	0.002
ENSG00000136535	TBR1	-0.273	6.321	-2.479	0.015
ENSG00000136628	EPRS	0.234	7.319	2.596	0.011
ENSG00000136709	WDR33	-0.090	6.297	-2.085	0.040
ENSG00000136710	CCDC115	0.217	4.247	2.777	0.007
ENSG00000136715	SAP130	-0.231	4.744	-3.209	0.002
ENSG00000136718	IMP4	0.205	5.073	2.696	0.009
ENSG00000136731	UGGT1	0.105	6.810	2.031	0.046
ENSG00000136758	YME1L1	0.196	6.722	2.644	0.010
ENSG00000136770	DNAJC1	0.195	4.261	2.001	0.049
ENSG00000136783	NIPSNAP3A	0.349	3.877	3.083	0.003
ENSG00000136810	TXN	0.613	4.415	2.979	0.004
ENSG00000136811	ODF2	0.159	4.699	2.280	0.025
ENSG00000136813	KIAA0368	0.148	8.228	1.998	0.049
ENSG00000136819	C9orf78	0.226	5.481	2.169	0.033
ENSG00000136828	RALGPS1	-0.148	5.907	-2.618	0.011
ENSG00000136830	FAM129B	0.318	2.806	2.399	0.019
ENSG00000136854	STXBP1	-0.244	8.513	-2.353	0.021
ENSG00000136856	SLC2A8	0.206	1.822	2.043	0.044
ENSG00000136869	TLR4	-0.385	5.734	-1.999	0.049
ENSG00000136875	PRPF4	0.212	4.656	2.569	0.012
ENSG00000136888	ATP6V1G1	0.371	5.358	3.043	0.003
ENSG00000136930	PSMB7	0.349	5.507	2.978	0.004
ENSG00000136933	RABEPK	0.285	3.879	3.682	0.000
ENSG00000136940	PDCL	0.192	4.806	2.425	0.018
ENSG00000136942	RPL35	0.408	5.065	2.127	0.036
ENSG00000136950	ARPC5L	0.216	4.499	2.221	0.029
ENSG00000136999	NOV	0.371	4.187	3.292	0.001
ENSG00000137038	TMEM261	0.258	4.524	2.799	0.006
ENSG00000137040	RANBP6	0.267	6.861	2.149	0.035
ENSG00000137054	POLR1E	0.310	3.609	3.137	0.002

ENSG00000137055	PLAA	0.232	5.499	2.886	0.005
ENSG00000137074	APTX	0.238	4.188	2.745	0.007
ENSG00000137080	IFNA21	-0.626	-1.111	-2.332	0.022
ENSG00000137100	DCTN3	0.354	4.458	2.976	0.004
ENSG00000137106	GRHPR	0.198	4.648	2.162	0.034
ENSG00000137124	ALDH1B1	0.286	2.870	2.674	0.009
ENSG00000137142	IGFBPL1	0.559	-0.102	2.775	0.007
ENSG00000137166	FOXP4	-0.413	2.506	-2.502	0.014
ENSG00000137168	PPIL1	0.221	4.137	2.295	0.024
ENSG00000137210	TMEM14B	0.123	4.028	2.169	0.033
ENSG00000137288	UQCC2	0.207	4.023	2.220	0.029
ENSG00000137312	FLOT1	0.223	6.304	2.595	0.011
ENSG00000137337	MDC1	-0.169	4.699	-2.265	0.026
ENSG00000137393	RNF144B	0.310	3.749	2.564	0.012
ENSG00000137414	FAM8A1	0.156	6.379	2.006	0.048
ENSG00000137500	CCDC90B	0.228	5.305	2.265	0.026
ENSG00000137522	RNF121	0.195	3.954	2.760	0.007
ENSG00000137547	MRPL15	0.293	4.283	2.596	0.011
ENSG00000137561	TTPA	-0.397	2.034	-2.100	0.039
ENSG00000137563	GGH	0.386	3.722	2.681	0.009
ENSG00000137575	SDCBP	0.269	6.230	2.828	0.006
ENSG00000137692	DCUN1D5	0.301	4.563	2.748	0.007
ENSG00000137700	SLC37A4	0.181	2.324	2.250	0.027
ENSG00000137714	FDX1	0.253	4.198	2.409	0.018
ENSG00000137760	ALKBH8	0.248	4.337	2.515	0.014
ENSG00000137776	SLTM	0.164	7.002	2.088	0.040
ENSG00000137806	NDUFAF1	0.434	4.036	3.879	0.000
ENSG00000137807	KIF23	0.301	0.902	2.399	0.019
ENSG00000137821	LRRC49	0.197	5.845	2.632	0.010
ENSG00000137824	RMDN3	0.146	4.978	2.027	0.046
ENSG00000137825	ITPKA	0.379	4.211	2.959	0.004
ENSG00000137857	DUOX1	-0.274	2.371	-2.143	0.035
ENSG00000137872	SEMA6D	-0.141	6.571	-2.147	0.035
ENSG00000137876	RSL24D1	0.459	5.843	3.175	0.002
ENSG00000137947	GTF2B	0.270	4.588	2.686	0.009
ENSG00000137955	RABGGTB	0.193	5.952	2.624	0.010
ENSG00000137996	RTCA	0.257	5.519	2.590	0.011
ENSG00000138018	EPT1	0.242	7.430	2.485	0.015
ENSG00000138061	CYP1B1	0.514	3.289	3.231	0.002
ENSG00000138069	RAB1A	0.243	6.728	2.616	0.011
ENSG00000138073	PREB	0.141	4.002	2.135	0.036
ENSG00000138107	ACTR1A	0.209	6.915	2.804	0.006
ENSG00000138119	MYOF	0.350	3.144	2.213	0.030
ENSG00000138138	ATAD1	0.248	6.699	2.489	0.015
ENSG00000138175	ARL3	0.203	5.723	2.311	0.023
ENSG00000138190	EXOC6	0.242	5.821	2.364	0.020
ENSG00000138246	DNAJC13	0.174	6.879	2.631	0.010

ENSG00000138271	GPR87	-0.600	1.439	-3.462	0.001
ENSG00000138326	RPS24	0.333	7.194	2.377	0.020
ENSG00000138356	AOX1	0.340	0.806	2.022	0.046
ENSG00000138363	ATIC	-0.196	4.265	-2.550	0.013
ENSG00000138375	SMARCAL1	-0.127	3.414	-2.461	0.016
ENSG00000138381	ASNSD1	0.299	5.741	2.671	0.009
ENSG00000138382	METTL5	0.329	4.075	2.620	0.010
ENSG00000138385	SSB	0.357	6.339	2.462	0.016
ENSG00000138395	CDK15	-0.632	-1.268	-2.613	0.011
ENSG00000138398	PPIG	0.192	7.481	2.500	0.014
ENSG00000138399	FASTKD1	0.136	5.201	2.509	0.014
ENSG00000138413	IDH1	0.285	4.736	2.909	0.005
ENSG00000138430	OLA1	0.308	6.436	2.056	0.043
ENSG00000138433	CIR1	0.208	4.995	2.326	0.022
ENSG00000138442	WDR12	0.271	5.952	2.845	0.006
ENSG00000138459	SLC35A5	0.266	5.536	2.689	0.009
ENSG00000138495	COX17	0.497	3.534	2.697	0.009
ENSG00000138592	USP8	0.182	6.795	3.043	0.003
ENSG00000138593	SECISBP2L	0.153	8.746	2.080	0.041
ENSG00000138594	TMOD3	0.109	5.435	2.155	0.034
ENSG00000138629	UBL7	0.260	3.779	2.159	0.034
ENSG00000138653	NDST4	0.348	0.689	2.043	0.044
ENSG00000138660	AP1AR	0.368	5.781	2.946	0.004
ENSG00000138663	COPS4	0.330	5.792	2.458	0.016
ENSG00000138668	HNRNPD	-0.227	6.693	-3.018	0.003
ENSG00000138669	PRKG2	0.457	4.246	3.286	0.001
ENSG00000138670	RASGEF1B	0.298	4.537	2.700	0.008
ENSG00000138678	GPAT3	0.501	2.344	2.883	0.005
ENSG00000138686	BBS7	0.279	6.610	2.551	0.013
ENSG00000138698	RAP1GDS1	0.373	6.976	2.822	0.006
ENSG00000138709	LARP1B	0.135	6.298	2.007	0.048
ENSG00000138735	PDE5A	-0.228	5.058	-2.718	0.008
ENSG00000138757	G3BP2	0.254	8.626	2.115	0.037
ENSG00000138758	SEPTIN11	0.119	8.326	2.093	0.039
ENSG00000138768	USO1	0.274	6.998	2.759	0.007
ENSG00000138795	LEF1	0.545	2.662	3.569	0.001
ENSG00000138796	HADH	-0.255	4.426	-4.332	0.000
ENSG00000138801	PAPSS1	0.285	6.407	2.731	0.008
ENSG00000138821	SLC39A8	0.261	3.819	2.355	0.021
ENSG00000138867	GUCD1	0.170	4.052	2.156	0.034
ENSG00000139112	GABARAPL1	0.272	8.363	2.269	0.026
ENSG00000139146	FAM60A	0.521	0.466	2.630	0.010
ENSG00000139160	ETFBKMT	-0.139	3.609	-2.433	0.017
ENSG00000139168	ZCRB1	0.273	5.448	2.659	0.009
ENSG00000139197	PEX5	0.157	4.866	2.012	0.047
ENSG00000139200	PIANP	-0.585	3.631	-2.882	0.005
ENSG00000139266	MARCHF9	0.165	2.616	2.358	0.021

ENSG00000139287	TPH2	0.514	1.401	2.898	0.005
ENSG00000139291	TMEM19	0.220	4.885	2.212	0.030
ENSG00000139324	TMTC3	0.223	6.278	2.951	0.004
ENSG00000139343	SNRPF	0.405	3.079	2.768	0.007
ENSG00000139344	AMDHD1	0.582	-0.416	2.566	0.012
ENSG00000139351	SYCP3	-0.542	3.083	-3.285	0.002
ENSG00000139354	GAS2L3	0.338	1.707	2.091	0.040
ENSG00000139428	MMAB	0.212	3.651	2.241	0.028
ENSG00000139436	GIT2	-0.199	5.368	-2.911	0.005
ENSG00000139496	NUP58	0.181	6.622	2.415	0.018
ENSG00000139505	MTMR6	0.284	6.425	2.722	0.008
ENSG00000139624	CERS5	-0.174	4.566	-2.231	0.028
ENSG00000139668	WDFY2	-0.228	4.872	-2.403	0.019
ENSG00000139684	ESD	0.270	5.120	2.916	0.005
ENSG00000139687	RB1	0.269	5.939	3.964	0.000
ENSG00000139697	SBNO1	0.195	8.080	2.709	0.008
ENSG00000139714	MORN3	0.305	2.851	3.043	0.003
ENSG00000139718	SETD1B	-0.341	4.021	-2.745	0.007
ENSG00000139726	DENR	0.315	6.163	3.419	0.001
ENSG00000139767	SRRM4	-0.317	6.556	-3.475	0.001
ENSG00000139797	RNF113B	-0.824	-1.647	-3.224	0.002
ENSG00000139826	ABHD13	0.197	5.512	2.517	0.014
ENSG00000139835	GRTP1	-0.393	-0.114	-2.069	0.042
ENSG00000139842	CUL4A	0.144	5.801	2.244	0.028
ENSG00000139874	SSTR1	0.239	3.627	2.031	0.045
ENSG00000139908	TSSK4	-0.220	2.242	-2.177	0.032
ENSG00000139910	NOVA1	-0.140	7.228	-2.104	0.038
ENSG00000139921	TMX1	0.218	4.792	2.266	0.026
ENSG00000139985	ADAM21	-0.288	2.618	-2.056	0.043
ENSG00000139998	RAB15	0.276	6.751	2.813	0.006
ENSG00000140015	KCNH5	0.253	7.079	2.123	0.037
ENSG00000140043	PTGR2	-0.268	2.654	-2.755	0.007
ENSG00000140057	AK7	0.474	2.008	3.587	0.001
ENSG00000140090	SLC24A4	-0.366	5.354	-3.415	0.001
ENSG00000140105	WARS	0.383	6.175	2.488	0.015
ENSG00000140259	MFAP1	0.304	5.006	3.349	0.001
ENSG00000140264	SERF2	0.217	4.191	2.297	0.024
ENSG00000140280	LYSMD2	0.211	3.804	2.031	0.046
ENSG00000140307	GTF2A2	0.405	3.391	3.246	0.002
ENSG00000140350	ANP32A	0.207	6.165	3.133	0.002
ENSG00000140367	UBE2Q2	0.314	5.303	2.437	0.017
ENSG00000140374	ETFA	0.287	5.057	2.523	0.014
ENSG00000140391	TSPAN3	0.165	7.640	2.166	0.033
ENSG00000140403	DNAJA4	-0.311	6.476	-2.977	0.004
ENSG00000140416	TPM1	0.178	6.405	2.425	0.018
ENSG00000140443	IGF1R	-0.168	6.690	-2.303	0.024
ENSG00000140455	USP3	-0.168	3.646	-2.296	0.024

ENSG00000140463	BBS4	0.185	4.244	2.449	0.016
ENSG00000140471	LINS1	0.260	4.594	2.793	0.007
ENSG00000140474	ULK3	0.214	4.851	2.904	0.005
ENSG00000140527	WDR93	-0.462	0.163	-2.745	0.007
ENSG00000140534	TICRR	-0.390	0.351	-2.373	0.020
ENSG00000140543	DET1	-0.274	0.991	-2.016	0.047
ENSG00000140577	CRTC3	-0.179	4.732	-2.091	0.040
ENSG00000140598	EFL1	0.177	4.459	2.650	0.010
ENSG00000140740	UQCRC2	0.219	7.075	2.319	0.023
ENSG00000140743	CDR2	0.190	4.283	2.033	0.045
ENSG00000140798	ABCC12	0.426	2.715	2.679	0.009
ENSG00000140807	NKD1	0.201	4.300	2.043	0.044
ENSG00000140829	DHX38	0.177	5.101	2.271	0.026
ENSG00000140830	TXNL4B	0.185	3.322	2.101	0.039
ENSG00000140948	ZCCHC14	-0.171	5.729	-2.904	0.005
ENSG00000140988	RPS2	0.436	3.818	2.219	0.029
ENSG00000140995	DEF8	0.208	4.989	2.053	0.043
ENSG00000141002	TCF25	0.192	6.106	2.266	0.026
ENSG00000141030	COPS3	0.271	5.638	2.345	0.021
ENSG00000141034	GID4	0.142	4.150	2.829	0.006
ENSG00000141098	GFOD2	0.241	5.102	3.181	0.002
ENSG00000141127	PRPSAP2	0.239	4.289	2.313	0.023
ENSG00000141219	C17orf80	0.111	4.889	2.194	0.031
ENSG00000141232	TOB1	-0.230	4.605	-2.355	0.021
ENSG00000141252	VPS53	-0.215	7.261	-2.948	0.004
ENSG00000141314	RHBDL3	-0.302	4.280	-2.401	0.019
ENSG00000141367	CLTC	0.210	10.044	1.995	0.049
ENSG00000141378	PTRH2	0.147	3.702	2.243	0.028
ENSG00000141385	AFG3L2	0.336	5.945	3.096	0.003
ENSG00000141424	SLC39A6	0.217	6.168	2.799	0.006
ENSG00000141425	RPRD1A	0.227	7.849	2.583	0.012
ENSG00000141429	GALNT1	0.328	6.004	3.505	0.001
ENSG00000141434	MEP1B	-0.548	0.506	-3.365	0.001
ENSG00000141447	OSBPL1A	0.199	8.178	2.091	0.040
ENSG00000141469	SLC14A1	-0.899	4.530	-2.451	0.016
ENSG00000141480	ARRB2	0.149	4.214	2.201	0.031
ENSG00000141504	SAT2	0.241	3.770	2.397	0.019
ENSG00000141526	SLC16A3	0.314	0.924	2.067	0.042
ENSG00000141542	RAB40B	0.289	6.050	3.651	0.000
ENSG00000141543	EIF4A3	0.366	3.542	3.016	0.003
ENSG00000141562	NARF	0.148	4.807	2.923	0.004
ENSG00000141580	WDR45B	0.163	5.416	2.496	0.015
ENSG00000141698	NT5C3B	0.370	5.018	3.139	0.002
ENSG00000141699	FAM134C	0.190	5.372	3.327	0.001
ENSG00000141759	TXNL4A	0.206	5.292	2.450	0.016
ENSG00000141905	NFIC	-0.376	5.891	-2.888	0.005
ENSG00000141968	VAV1	0.515	1.065	2.516	0.014

ENSG00000142039	CCDC97	-0.260	3.153	-2.538	0.013
ENSG00000142166	IFNAR1	0.149	6.969	2.594	0.011
ENSG00000142168	SOD1	0.364	7.582	2.436	0.017
ENSG00000142173	COL6A2	0.485	1.073	2.851	0.006
ENSG00000142186	SCYL1	0.176	5.327	2.078	0.041
ENSG00000142188	TMEM50B	0.173	5.375	2.399	0.019
ENSG00000142230	SAE1	0.288	5.994	2.855	0.005
ENSG00000142396	ERVK3-1	-0.253	2.540	-2.302	0.024
ENSG00000142444	C19orf52	0.208	2.441	2.012	0.047
ENSG00000142507	PSMB6	0.306	4.130	2.068	0.042
ENSG00000142534	RPS11	0.383	6.416	2.215	0.030
ENSG00000142541	RPL13A	0.846	5.274	2.364	0.020
ENSG00000142599	RERE	-0.157	7.695	-2.551	0.013
ENSG00000142657	PGD	0.329	5.165	2.787	0.007
ENSG00000142676	RPL11	0.284	6.329	2.506	0.014
ENSG00000142864	SERBP1	0.263	6.641	2.843	0.006
ENSG00000142871	CYR61	-0.470	2.157	-2.264	0.026
ENSG00000142892	PIGK	0.236	5.805	2.226	0.029
ENSG00000142937	RPS8	0.463	6.006	2.206	0.030
ENSG00000143013	LMO4	0.237	8.310	2.090	0.040
ENSG00000143106	PSMA5	0.342	5.655	2.768	0.007
ENSG00000143126	CELSR2	-0.258	7.585	-2.577	0.012
ENSG00000143155	TIPRL	0.259	6.194	2.611	0.011
ENSG00000143156	NME7	0.160	4.626	2.496	0.015
ENSG00000143158	MPC2	0.402	5.918	3.365	0.001
ENSG00000143162	CREG1	0.160	4.894	2.198	0.031
ENSG00000143164	DCAF6	0.258	7.662	2.262	0.026
ENSG00000143179	UCK2	0.186	4.576	2.664	0.009
ENSG00000143183	TMCO1	0.228	6.174	2.555	0.012
ENSG00000143190	POU2F1	-0.319	6.208	-3.950	0.000
ENSG00000143198	MGST3	0.175	6.337	2.101	0.039
ENSG00000143222	UFC1	0.304	4.109	2.861	0.005
ENSG00000143252	SDHC	-0.144	7.016	-2.348	0.021
ENSG00000143256	PFDN2	0.280	4.381	2.640	0.010
ENSG00000143314	MRPL24	0.270	4.146	2.626	0.010
ENSG00000143321	HDGF	0.138	6.293	2.223	0.029
ENSG00000143322	ABL2	-0.164	6.600	-2.963	0.004
ENSG00000143341	HMCN1	0.560	1.751	2.793	0.007
ENSG00000143344	RGL1	0.182	7.179	2.591	0.011
ENSG00000143367	TUFT1	0.197	3.212	2.221	0.029
ENSG00000143369	ECM1	-0.298	2.529	-2.256	0.027
ENSG00000143420	ENSA	0.279	7.839	3.327	0.001
ENSG00000143436	MRPL9	0.275	3.669	2.468	0.016
ENSG00000143437	ARNT	-0.123	5.343	-2.652	0.010
ENSG00000143442	POGZ	-0.294	6.623	-4.293	0.000
ENSG00000143452	HORMAD1	0.572	-0.323	2.778	0.007
ENSG00000143499	SMYD2	0.383	5.775	2.910	0.005

ENSG00000143543	JTB	0.345	2.898	2.836	0.006
ENSG00000143549	TPM3	0.376	7.576	3.381	0.001
ENSG00000143575	HAX1	0.446	4.664	3.200	0.002
ENSG00000143603	KCNN3	-0.453	6.414	-2.853	0.005
ENSG00000143614	GATAD2B	-0.189	6.667	-3.083	0.003
ENSG00000143621	ILF2	0.296	5.959	3.224	0.002
ENSG00000143622	RIT1	0.154	4.519	2.076	0.041
ENSG00000143632	ACTA1	0.431	0.082	2.606	0.011
ENSG00000143633	C1orf131	0.159	2.977	2.247	0.027
ENSG00000143653	SCCPDH	0.264	5.477	2.351	0.021
ENSG00000143727	ACP1	0.261	6.381	2.787	0.007
ENSG00000143742	SRP9	0.207	6.640	2.489	0.015
ENSG00000143756	FBXO28	0.203	6.307	2.392	0.019
ENSG00000143774	GUK1	0.368	5.955	2.013	0.047
ENSG00000143786	CNIH3	0.204	4.347	2.476	0.015
ENSG00000143793	C1orf35	0.304	2.335	3.070	0.003
ENSG00000143799	PARP1	0.206	6.775	2.151	0.034
ENSG00000143801	PSEN2	0.159	3.990	2.071	0.042
ENSG00000143815	LBR	0.195	3.926	2.147	0.035
ENSG00000143819	EPHX1	-0.241	4.099	-2.031	0.046
ENSG00000143851	PTPN7	-0.467	-0.580	-2.098	0.039
ENSG00000143862	ARL8A	0.189	4.998	2.442	0.017
ENSG00000143870	PDIA6	0.462	5.235	3.447	0.001
ENSG00000143933	CALM2	0.433	9.880	2.989	0.004
ENSG00000143947	RPS27A	0.965	5.029	2.482	0.015
ENSG00000143951	WDPCP	-0.131	4.905	-2.025	0.046
ENSG00000143970	ASXL2	-0.117	6.612	-2.641	0.010
ENSG00000143977	SNRPG	0.480	2.111	2.527	0.013
ENSG00000144029	MRPS5	0.194	5.354	2.415	0.018
ENSG00000144031	ANKRD53	-0.384	-0.139	-2.057	0.043
ENSG00000144034	TPRKB	0.196	2.927	2.516	0.014
ENSG00000144118	RALB	0.319	6.426	3.043	0.003
ENSG00000144161	ZC3H8	0.199	5.845	3.058	0.003
ENSG00000144199	FAHD2B	-0.218	3.389	-2.697	0.008
ENSG00000144227	NXPH2	0.436	2.046	2.002	0.049
ENSG00000144231	POLR2D	0.182	5.013	2.911	0.005
ENSG00000144285	SCN1A	0.239	8.577	2.009	0.048
ENSG00000144320	KIAA1715	0.198	7.071	2.535	0.013
ENSG00000144357	UBR3	0.194	8.339	2.287	0.025
ENSG00000144401	METTL21A	0.165	4.077	2.389	0.019
ENSG00000144451	SPAG16	0.198	5.393	2.036	0.045
ENSG00000144550	CPNE9	0.322	3.144	2.037	0.045
ENSG00000144554	FANCD2	0.261	1.617	2.298	0.024
ENSG00000144559	TAMM41	-0.197	3.978	-2.109	0.038
ENSG00000144566	RAB5A	0.196	6.586	2.685	0.009
ENSG00000144567	FAM134A	0.171	6.684	2.000	0.049
ENSG00000144597	EAF1	0.172	5.368	2.216	0.029

ENSG00000144619	CNTN4	0.269	6.156	2.379	0.020
ENSG00000144635	DYNC1LI1	0.205	6.201	2.105	0.038
ENSG00000144644	GADL1	1.103	-3.056	2.369	0.020
ENSG00000144649	FAM198A	-0.507	2.202	-2.866	0.005
ENSG00000144659	SLC25A38	0.218	4.007	2.755	0.007
ENSG00000144677	CTDSPL	-0.152	4.854	-2.104	0.038
ENSG00000144713	RPL32	0.513	6.035	2.346	0.021
ENSG00000144744	UBA3	0.182	5.644	2.283	0.025
ENSG00000144746	ARL6IP5	0.290	6.776	2.605	0.011
ENSG00000144771	LRTM1	-0.539	1.804	-3.021	0.003
ENSG00000144792	ZNF660	-0.296	3.920	-3.430	0.001
ENSG00000144827	ABHD10	0.342	5.622	3.205	0.002
ENSG00000144847	IGSF11	-0.255	5.308	-3.056	0.003
ENSG00000144848	ATG3	0.365	5.122	3.044	0.003
ENSG00000144857	BOC	-0.283	2.918	-2.091	0.040
ENSG00000144867	SRPRB	0.314	4.589	3.577	0.001
ENSG00000144868	TMEM108	-0.244	4.420	-2.945	0.004
ENSG00000144895	EIF2A	0.274	5.697	2.742	0.007
ENSG00000144935	TRPC1	0.220	6.054	2.723	0.008
ENSG00000144959	NCEH1	0.207	7.223	2.350	0.021
ENSG00000145012	LPP	-0.231	6.546	-2.489	0.015
ENSG00000145050	MANF	0.401	3.130	4.054	0.000
ENSG00000145075	CCDC39	-0.380	0.988	-2.775	0.007
ENSG00000145191	EIF2B5	0.177	4.948	2.384	0.019
ENSG00000145216	FIP1L1	0.130	5.215	2.140	0.035
ENSG00000145220	LYAR	0.281	2.591	2.385	0.019
ENSG00000145241	CENPC	-0.131	6.315	-2.051	0.043
ENSG00000145242	EPHA5	0.317	6.691	2.436	0.017
ENSG00000145293	ENOPH1	0.352	6.098	3.325	0.001
ENSG00000145332	KLHL8	0.237	6.186	2.579	0.012
ENSG00000145335	SNCA	0.351	7.884	2.468	0.016
ENSG00000145337	PYURF	0.235	4.364	2.677	0.009
ENSG00000145354	CISD2	0.202	4.737	2.260	0.026
ENSG00000145391	SETD7	0.136	7.159	2.013	0.047
ENSG00000145425	RPS3A	0.780	5.225	2.599	0.011
ENSG00000145428	RNF175	0.261	4.406	2.203	0.030
ENSG00000145476	CYP4V2	-0.336	5.578	-2.590	0.011
ENSG00000145494	NDUFS6	0.583	3.207	3.711	0.000
ENSG00000145536	ADAMTS16	-0.288	2.259	-2.801	0.006
ENSG00000145545	SRD5A1	0.281	5.822	2.655	0.010
ENSG00000145569	FAM105A	0.409	3.616	3.303	0.001
ENSG00000145681	HAPLN1	0.350	2.975	2.183	0.032
ENSG00000145708	CRHBP	0.624	3.227	2.628	0.010
ENSG00000145721	LIX1	-0.380	4.783	-2.105	0.038
ENSG00000145723	GIN1	0.181	4.059	2.222	0.029
ENSG00000145736	GTF2H2	-0.780	3.212	-2.838	0.006
ENSG00000145740	SLC30A5	0.157	4.933	2.218	0.029

ENSG00000145741	BTF3	0.288	5.607	2.224	0.029
ENSG00000145779	TNFAIP8	0.404	0.326	2.334	0.022
ENSG00000145781	COMMD10	0.282	3.601	2.909	0.005
ENSG00000145782	ATG12	0.225	6.115	3.427	0.001
ENSG00000145817	YIPF5	0.263	5.485	2.608	0.011
ENSG00000145819	ARHGAP26	-0.122	8.272	-1.998	0.049
ENSG00000145850	TIMD4	1.154	-2.839	3.214	0.002
ENSG00000145860	RNF145	0.228	6.301	2.490	0.015
ENSG00000145868	FBXO38	0.115	6.045	2.024	0.046
ENSG00000145888	GLRA1	-0.577	-1.141	-2.801	0.006
ENSG00000145901	TNIP1	0.164	4.852	1.990	0.050
ENSG00000145912	NHP2	0.466	2.856	3.040	0.003
ENSG00000145975	FAM217A	-0.654	-1.263	-2.730	0.008
ENSG00000146001	PCDHB18P	-0.266	2.684	-2.184	0.032
ENSG00000146007	ZMAT2	0.429	6.307	3.610	0.001
ENSG00000146070	PLA2G7	0.404	2.767	2.766	0.007
ENSG00000146147	MLIP	-0.267	5.939	-2.883	0.005
ENSG00000146151	HMGCLL1	0.224	5.553	2.142	0.035
ENSG00000146223	RPL7L1	0.253	6.345	3.236	0.002
ENSG00000146232	NFKBIE	0.279	2.046	2.570	0.012
ENSG00000146386	ABRACL	0.346	3.104	2.386	0.019
ENSG00000146411	SLC2A12	-0.218	3.920	-2.088	0.040
ENSG00000146433	TMEM181	0.252	6.549	2.978	0.004
ENSG00000146476	ARMT1	0.329	5.143	2.993	0.004
ENSG00000146540	C7orf50	0.246	4.234	2.337	0.022
ENSG00000146648	EGFR	-0.432	5.955	-2.679	0.009
ENSG00000146666	LINC00525	-0.646	-1.632	-2.262	0.026
ENSG00000146700	SSC4D	0.554	-1.064	2.932	0.004
ENSG00000146731	CCT6A	0.341	6.557	2.753	0.007
ENSG00000146757	ZNF92	0.226	4.320	2.470	0.016
ENSG00000146776	ATXN7L1	-0.207	4.536	-2.803	0.006
ENSG00000146872	TLK2	0.258	5.319	2.065	0.042
ENSG00000146963	LUC7L2	-0.212	5.215	-2.360	0.021
ENSG00000147419	CCDC25	0.274	6.587	2.780	0.007
ENSG00000147439	BIN3	0.332	2.291	3.134	0.002
ENSG00000147471	PROSC	0.133	5.335	2.153	0.034
ENSG00000147533	GOLGA7	0.183	5.702	2.057	0.043
ENSG00000147535	PLPP5	-0.161	3.639	-2.405	0.018
ENSG00000147576	ADHFE1	-0.391	2.235	-2.779	0.007
ENSG00000147604	RPL7	0.956	5.371	2.528	0.013
ENSG00000147649	MTDH	0.180	7.221	2.715	0.008
ENSG00000147669	POLR2K	0.330	5.660	2.692	0.009
ENSG00000147676	MAL2	0.363	6.421	2.580	0.012
ENSG00000147677	EIF3H	0.214	6.040	2.657	0.009
ENSG00000147684	NDUFB9	0.303	5.247	2.350	0.021
ENSG00000147852	VLDLR	0.235	5.257	2.562	0.012
ENSG00000147996	CBWD5	-0.512	4.320	-2.746	0.007

ENSG00000148123	PLPPR1	0.496	4.064	3.445	0.001
ENSG00000148153	INIP	0.149	5.766	2.197	0.031
ENSG00000148154	UGCG	0.364	5.788	2.693	0.009
ENSG00000148200	NR6A1	-0.219	2.605	-2.304	0.024
ENSG00000148229	POLE3	0.330	4.955	3.269	0.002
ENSG00000148290	SURF1	0.293	1.610	2.290	0.025
ENSG00000148291	SURF2	0.419	2.797	3.240	0.002
ENSG00000148296	SURF6	0.190	4.890	2.347	0.021
ENSG00000148300	REXO4	0.315	3.892	3.496	0.001
ENSG00000148303	RPL7A	0.526	5.650	2.501	0.014
ENSG00000148331	ASB6	0.147	3.986	2.188	0.032
ENSG00000148334	PTGES2	0.271	4.725	2.010	0.048
ENSG00000148335	NTMT1	0.350	1.781	3.341	0.001
ENSG00000148396	SEC16A	-0.196	5.911	-2.804	0.006
ENSG00000148429	USP6NL	0.185	4.616	2.736	0.008
ENSG00000148444	COMMD3	0.265	2.341	2.485	0.015
ENSG00000148450	MSRB2	0.145	3.601	2.023	0.046
ENSG00000148481	FAM188A	0.346	6.322	3.073	0.003
ENSG00000148482	SLC39A12	-0.527	4.597	-2.026	0.046
ENSG00000148484	RSU1	0.178	5.012	2.668	0.009
ENSG00000148513	ANKRD30A	0.640	-0.106	2.232	0.028
ENSG00000148516	ZEB1	0.123	7.493	2.325	0.023
ENSG00000148572	NRBF2	0.258	4.154	2.583	0.012
ENSG00000148655	C10orf11	-0.405	1.491	-2.654	0.010
ENSG00000148690	FRA10AC1	0.214	5.876	2.057	0.043
ENSG00000148704	VAX1	-0.317	1.203	-2.381	0.020
ENSG00000148730	EIF4EBP2	-0.175	7.121	-2.236	0.028
ENSG00000148734	NPFFR1	-0.530	2.246	-2.234	0.028
ENSG00000148737	TCF7L2	-0.406	3.823	-3.696	0.000
ENSG00000148832	PAOX	0.349	0.607	2.019	0.047
ENSG00000148834	GSTO1	0.411	4.587	2.555	0.012
ENSG00000148835	TAF5	0.192	3.015	2.131	0.036
ENSG00000148843	PDCD11	0.197	5.089	3.137	0.002
ENSG00000148925	BTBD10	0.324	7.300	2.702	0.008
ENSG00000148943	LIN7C	0.177	7.135	2.378	0.020
ENSG00000148948	LRRC4C	0.154	6.887	2.026	0.046
ENSG00000149100	EIF3M	0.190	5.823	2.240	0.028
ENSG00000149182	ARFGAP2	0.243	5.669	2.572	0.012
ENSG00000149196	HIKESHI	0.224	4.128	2.141	0.035
ENSG00000149273	RPS3	0.288	5.639	2.135	0.036
ENSG00000149289	ZC3H12C	-0.188	5.436	-2.193	0.031
ENSG00000149313	AASDHPPT	0.233	7.010	2.033	0.045
ENSG00000149357	LAMTOR1	0.218	4.457	2.464	0.016
ENSG00000149474	KAT14	0.201	5.196	2.802	0.006
ENSG00000149480	MTA2	0.170	4.642	2.558	0.012
ENSG00000149532	CPSF7	-0.159	5.501	-3.218	0.002
ENSG00000149541	B3GAT3	0.240	2.712	2.756	0.007

ENSG00000149547	EI24	0.312	5.167	3.302	0.001
ENSG00000149557	FEZ1	0.245	7.430	2.678	0.009
ENSG00000149564	ESAM	0.334	1.758	2.795	0.006
ENSG00000149575	SCN2B	0.220	7.139	2.565	0.012
ENSG00000149639	SOGA1	-0.227	6.185	-2.708	0.008
ENSG00000149656	LINC00266-1	-0.547	0.525	-2.166	0.033
ENSG00000149761	NUDT22	0.243	1.215	2.327	0.022
ENSG00000149792	MRPL49	0.194	4.057	2.364	0.020
ENSG00000149806	FAU	0.617	4.093	2.458	0.016
ENSG00000149809	TM7SF2	0.362	3.453	3.620	0.001
ENSG00000149923	PPP4C	0.210	1.940	2.120	0.037
ENSG00000150051	MKX	0.376	4.509	2.915	0.005
ENSG00000150093	ITGB1	0.218	6.059	2.264	0.026
ENSG00000150275	PCDH15	0.249	5.079	2.111	0.038
ENSG00000150281	CTF1	-0.536	-0.315	-2.204	0.030
ENSG00000150316	CWC15	0.300	5.265	2.523	0.014
ENSG00000150361	KLHL1	0.379	1.787	2.559	0.012
ENSG00000150403	TMCO3	0.145	5.117	2.487	0.015
ENSG00000150459	SAP18	0.176	7.271	2.097	0.039
ENSG00000150477	KIAA1328	-0.111	4.831	-2.184	0.032
ENSG00000150593	PDCD4	0.209	5.885	2.718	0.008
ENSG00000150625	GPM6A	0.204	10.487	2.420	0.018
ENSG00000150672	DLG2	0.164	9.148	2.085	0.040
ENSG00000150681	RGS18	0.872	-0.530	2.127	0.036
ENSG00000150753	CCT5	0.439	6.036	3.080	0.003
ENSG00000150768	DLAT	0.174	5.952	2.086	0.040
ENSG00000150776	C11orf57	0.174	5.343	2.399	0.019
ENSG00000150779	TIMM8B	0.526	2.970	3.363	0.001
ENSG00000150787	PTS	0.448	4.080	3.030	0.003
ENSG00000150977	RILPL2	0.366	3.068	3.726	0.000
ENSG00000151067	CACNA1C	-0.185	6.521	-2.238	0.028
ENSG00000151135	TMEM263	0.288	5.458	3.612	0.001
ENSG00000151151	IPMK	0.168	4.448	2.414	0.018
ENSG00000151229	SLC2A13	0.245	8.180	2.134	0.036
ENSG00000151239	TWF1	0.277	5.225	3.112	0.003
ENSG00000151327	FAM177A1	0.165	5.436	2.244	0.028
ENSG00000151353	TMEM18	0.201	4.300	2.526	0.013
ENSG00000151366	NDUFC2	0.417	4.221	3.552	0.001
ENSG00000151465	CDC123	0.359	4.672	2.573	0.012
ENSG00000151500	THYN1	0.245	5.158	2.347	0.021
ENSG00000151553	FAM160B1	0.171	5.871	2.201	0.031
ENSG00000151575	TEX9	-0.210	2.882	-2.075	0.041
ENSG00000151611	MMAA	0.195	4.014	2.583	0.012
ENSG00000151612	ZNF827	-0.112	5.782	-2.074	0.041
ENSG00000151617	EDNRA	0.318	2.191	2.956	0.004
ENSG00000151632	AKR1C2	0.500	2.181	3.513	0.001
ENSG00000151687	ANKAR	-0.167	4.164	-2.694	0.009

ENSG00000151694	ADAM17	-0.123	5.766	-2.003	0.049
ENSG00000151704	KCNJ1	0.618	-0.964	2.226	0.029
ENSG00000151743	AMN1	0.239	4.820	2.454	0.016
ENSG00000151790	TDO2	0.811	1.514	3.564	0.001
ENSG00000151948	GLT1D1	0.274	5.137	2.467	0.016
ENSG00000152092	ASTN1	-0.138	7.832	-2.026	0.046
ENSG00000152102	FAM168B	0.145	7.901	2.275	0.026
ENSG00000152104	PTPN14	-0.234	5.589	-2.943	0.004
ENSG00000152133	GPATCH11	0.266	5.558	2.839	0.006
ENSG00000152137	HSPB8	-0.258	5.480	-2.431	0.017
ENSG00000152147	GEMIN6	0.304	3.740	3.131	0.002
ENSG00000152192	POU4F1	-0.865	-1.970	-2.170	0.033
ENSG00000152208	GRID2	0.215	4.946	2.285	0.025
ENSG00000152214	RIT2	0.526	3.961	3.670	0.000
ENSG00000152217	SETBP1	-0.150	6.605	-2.108	0.038
ENSG00000152219	ARL14EP	0.252	5.665	2.716	0.008
ENSG00000152223	EPG5	-0.101	6.460	-2.374	0.020
ENSG00000152234	ATP5A1	0.309	8.418	2.699	0.008
ENSG00000152270	PDE3B	-0.126	5.411	-2.122	0.037
ENSG00000152413	HOMER1	0.448	8.012	3.586	0.001
ENSG00000152422	XRCC4	0.436	2.516	3.924	0.000
ENSG00000152484	USP12	0.313	6.696	3.477	0.001
ENSG00000152492	CCDC50	0.141	6.390	2.253	0.027
ENSG00000152503	TRIM36	0.271	5.898	2.112	0.038
ENSG00000152520	PAN3	-0.097	5.485	-2.117	0.037
ENSG00000152527	PLEKHH2	0.258	4.684	2.252	0.027
ENSG00000152601	MBNL1	0.128	7.561	2.010	0.048
ENSG00000152661	GJA1	-0.513	8.087	-2.196	0.031
ENSG00000152683	SLC30A6	0.136	4.589	2.206	0.030
ENSG00000152684	PELO	0.223	4.327	2.810	0.006
ENSG00000152767	FARP1	-0.141	7.643	-2.111	0.038
ENSG00000152778	IFIT5	0.240	5.464	2.259	0.027
ENSG00000152795	HNRNPDL	0.198	8.336	2.299	0.024
ENSG00000152904	GGPS1	0.140	4.283	2.006	0.048
ENSG00000152939	MARVELD2	0.459	0.800	3.047	0.003
ENSG00000152942	RAD17	0.232	4.721	2.405	0.018
ENSG00000153015	CWC27	0.167	5.107	2.059	0.043
ENSG00000153037	SRP19	0.120	4.553	2.257	0.027
ENSG00000153086	ACMSD	-0.386	0.401	-2.325	0.023
ENSG00000153130	SCOC	0.293	7.059	2.210	0.030
ENSG00000153140	CETN3	0.229	4.466	2.380	0.020
ENSG00000153147	SMARCA5	0.183	7.952	1.994	0.050
ENSG00000153179	RASSF3	0.298	3.807	2.552	0.013
ENSG00000153214	TMEM87B	0.256	4.503	2.961	0.004
ENSG00000153310	FAM49B	0.247	7.107	2.387	0.019
ENSG00000153339	TRAPPC8	0.247	6.502	3.082	0.003
ENSG00000153347	FAM81B	-0.711	-0.300	-2.846	0.006

ENSG00000153391	INO80C	-0.227	1.772	-2.291	0.025
ENSG00000153406	NMRAL1	0.221	3.991	2.398	0.019
ENSG00000153561	RMND5A	0.241	7.466	2.912	0.005
ENSG00000153767	GTF2E1	0.267	3.919	3.144	0.002
ENSG00000153774	CFDP1	0.268	5.150	3.134	0.002
ENSG00000153815	CMIP	-0.230	6.149	-2.090	0.040
ENSG00000153879	CEBPG	0.222	5.855	2.490	0.015
ENSG00000153930	ANKFN1	-0.341	2.120	-2.678	0.009
ENSG00000153944	MSI2	-0.219	7.148	-2.192	0.031
ENSG00000153975	ZUFSP	0.200	3.935	2.511	0.014
ENSG00000153982	GDPD1	0.288	4.808	2.838	0.006
ENSG00000154144	TBRG1	0.166	6.541	2.487	0.015
ENSG00000154153	FAM134B	0.260	6.391	2.047	0.044
ENSG00000154174	TOMM70	0.297	7.370	2.711	0.008
ENSG00000154175	ABI3BP	-0.303	2.326	-2.212	0.030
ENSG00000154227	CERS3	0.826	-1.419	2.379	0.020
ENSG00000154328	NEIL2	0.259	4.452	3.024	0.003
ENSG00000154429	CCSAP	0.205	7.889	2.412	0.018
ENSG00000154478	GPR26	0.596	5.726	3.830	0.000
ENSG00000154479	CCDC173	0.444	3.258	2.434	0.017
ENSG00000154485	MMP21	-0.686	-1.649	-2.796	0.006
ENSG00000154518	ATP5G3	0.335	5.414	3.051	0.003
ENSG00000154553	PDLIM3	-0.313	3.183	-2.501	0.014
ENSG00000154582	TCEB1	0.325	4.869	2.859	0.005
ENSG00000154639	CXADR	0.329	5.475	3.092	0.003
ENSG00000154654	NCAM2	0.292	8.665	2.916	0.005
ENSG00000154655	L3MBTL4	0.188	4.623	2.221	0.029
ENSG00000154719	MRPL39	0.297	4.108	2.502	0.014
ENSG00000154723	ATP5J	0.546	4.218	3.022	0.003
ENSG00000154783	FGD5	0.348	2.675	3.623	0.001
ENSG00000154832	CXXC1	0.194	3.459	2.464	0.016
ENSG00000154874	CCDC144B	-0.527	6.378	-2.735	0.008
ENSG00000154898	CCDC144CP	-0.373	5.873	-2.639	0.010
ENSG00000154975	CA10	0.372	6.432	2.838	0.006
ENSG00000155011	DKK2	0.670	1.843	3.348	0.001
ENSG00000155085	AK9	0.165	5.050	2.042	0.044
ENSG00000155096	AZIN1	0.298	8.060	3.168	0.002
ENSG00000155100	OTUD6B	0.245	5.070	2.745	0.007
ENSG00000155115	GTF3C6	0.509	3.717	2.983	0.004
ENSG00000155189	AGPAT5	0.216	5.992	2.780	0.007
ENSG00000155287	SLC25A28	-0.158	3.644	-2.295	0.024
ENSG00000155304	HSPA13	0.367	6.988	3.302	0.001
ENSG00000155313	USP25	0.151	7.191	2.230	0.028
ENSG00000155324	GRAMD3	-0.327	5.388	-2.197	0.031
ENSG00000155329	ZCCHC10	0.193	3.694	2.016	0.047
ENSG00000155438	NIFK	0.262	3.841	2.581	0.012
ENSG00000155506	LARP1	-0.168	7.877	-2.479	0.015

ENSG00000155508	CNOT8	0.222	5.395	2.617	0.011
ENSG00000155530	LRGUK	0.313	2.241	2.445	0.017
ENSG00000155629	PIK3AP1	0.504	2.181	2.112	0.038
ENSG00000155657	TTN	-0.267	7.906	-3.249	0.002
ENSG00000155660	PDIA4	0.249	4.715	2.548	0.013
ENSG00000155760	FZD7	-0.482	1.081	-2.402	0.019
ENSG00000155816	FMN2	-0.203	6.993	-2.605	0.011
ENSG00000155827	RNF20	0.142	6.376	2.071	0.042
ENSG00000155846	PPARGC1B	-0.172	5.089	-2.338	0.022
ENSG00000155868	MED7	0.153	4.340	2.199	0.031
ENSG00000155926	SLA	0.424	2.645	2.214	0.030
ENSG00000155975	VPS37A	0.168	5.911	2.140	0.035
ENSG00000156030	ELMSAN1	-0.177	5.260	-2.076	0.041
ENSG00000156136	DCK	0.241	5.477	2.336	0.022
ENSG00000156162	DPY19L4	0.204	5.068	2.088	0.040
ENSG00000156170	NDUFAF6	0.201	4.009	2.290	0.025
ENSG00000156239	N6AMT1	0.217	4.597	2.563	0.012
ENSG00000156256	USP16	0.195	5.961	2.159	0.034
ENSG00000156261	CCT8	0.476	6.370	2.864	0.005
ENSG00000156273	BACH1	0.200	6.122	2.500	0.014
ENSG00000156304	SCAF4	-0.269	4.788	-3.467	0.001
ENSG00000156345	CDK20	0.162	4.059	2.077	0.041
ENSG00000156374	PCGF6	0.283	2.213	2.220	0.029
ENSG00000156411	C14orf2	0.434	5.833	2.861	0.005
ENSG00000156463	SH3RF2	-0.489	1.981	-3.367	0.001
ENSG00000156467	UQCRB	0.351	7.754	2.255	0.027
ENSG00000156471	PTDSS1	0.228	5.855	2.096	0.039
ENSG00000156508	EEF1A1	0.359	8.702	2.232	0.028
ENSG00000156535	CD109	-0.461	3.338	-2.863	0.005
ENSG00000156650	KAT6B	-0.135	6.882	-3.264	0.002
ENSG00000156671	SAMD8	0.157	7.768	2.927	0.004
ENSG00000156831	NSMCE2	0.203	3.620	2.065	0.042
ENSG00000156873	PHKG2	0.216	3.068	2.443	0.017
ENSG00000156875	MFSD14A	0.394	1.678	2.687	0.009
ENSG00000156928	MALSU1	0.181	3.201	2.164	0.033
ENSG00000156966	B3GNT7	0.321	1.871	2.047	0.044
ENSG00000156973	PDE6D	0.147	3.458	2.458	0.016
ENSG00000156976	EIF4A2	0.395	9.679	2.600	0.011
ENSG00000157036	EXOG	-0.140	4.190	-2.637	0.010
ENSG00000157045	NTAN1	0.402	2.974	2.949	0.004
ENSG00000157368	IL34	0.464	1.977	3.103	0.003
ENSG00000157388	CACNA1D	-0.170	6.272	-2.151	0.034
ENSG00000157429	ZNF19	-0.238	1.839	-2.390	0.019
ENSG00000157500	APPL1	0.204	7.600	2.723	0.008
ENSG00000157510	AFAP1L1	0.285	1.769	2.149	0.035
ENSG00000157554	ERG	0.342	2.420	2.145	0.035
ENSG00000157741	UBN2	-0.185	7.059	-3.175	0.002

ENSG00000157778	PSMG3	0.334	3.293	2.662	0.009
ENSG00000157782	CABP1	0.193	6.991	2.349	0.021
ENSG00000157800	SLC37A3	0.213	5.295	2.726	0.008
ENSG00000157833	GAREM2	-0.332	3.962	-2.049	0.044
ENSG00000157881	PANK4	0.321	3.462	3.068	0.003
ENSG00000157895	C12orf43	0.149	4.752	2.199	0.031
ENSG00000157933	SKI	-0.217	5.820	-2.043	0.044
ENSG00000157999	ANKRD61	-0.516	0.084	-2.634	0.010
ENSG00000158042	MRPL17	0.297	4.063	3.479	0.001
ENSG00000158077	NLRP14	-0.481	0.316	-2.576	0.012
ENSG00000158092	NCK1	0.188	4.208	2.301	0.024
ENSG00000158161	EYA3	-0.146	5.096	-3.306	0.001
ENSG00000158169	FANCC	-0.332	3.299	-2.807	0.006
ENSG00000158220	ESYT3	-0.271	4.333	-2.911	0.005
ENSG00000158296	SLC13A3	-0.257	3.578	-2.302	0.024
ENSG00000158321	AUTS2	-0.318	5.905	-3.989	0.000
ENSG00000158373	HIST1H2BD	0.286	3.797	2.664	0.009
ENSG00000158406	HIST1H4H	0.454	3.325	2.562	0.012
ENSG00000158636	EMSY	-0.179	5.644	-3.486	0.001
ENSG00000158669	GPAT4	0.207	4.764	2.797	0.006
ENSG00000158716	DUSP23	0.499	1.326	3.678	0.000
ENSG00000158747	NBL1	0.218	5.378	2.613	0.011
ENSG00000158773	USF1	0.280	4.597	2.747	0.007
ENSG00000158806	NPM2	0.280	4.480	2.925	0.004
ENSG00000158864	NDUFS2	0.255	6.236	2.279	0.025
ENSG00000158882	TOMM40L	0.430	4.125	3.786	0.000
ENSG00000158985	CDC42SE2	0.216	7.568	2.214	0.030
ENSG00000158987	RAPGEF6	-0.232	4.501	-2.550	0.013
ENSG00000159055	MIS18A	0.357	1.576	2.899	0.005
ENSG00000159110	IFNAR2	-0.289	0.251	-2.046	0.044
ENSG00000159128	IFNGR2	0.216	4.620	2.568	0.012
ENSG00000159131	GART	0.125	5.060	2.686	0.009
ENSG00000159140	SON	-0.111	8.304	-2.629	0.010
ENSG00000159199	ATP5G1	0.447	3.061	2.509	0.014
ENSG00000159200	RCAN1	0.195	5.624	2.405	0.018
ENSG00000159210	SNF8	0.152	4.461	2.708	0.008
ENSG00000159256	MORC3	0.184	6.547	2.351	0.021
ENSG00000159322	ADPGK	0.135	4.267	2.496	0.015
ENSG00000159346	ADIPOR1	0.126	5.775	2.044	0.044
ENSG00000159352	PSMD4	0.424	5.959	2.530	0.013
ENSG00000159377	PSMB4	0.349	4.685	2.950	0.004
ENSG00000159399	HK2	0.603	2.378	2.704	0.008
ENSG00000159423	ALDH4A1	-0.386	2.673	-2.016	0.047
ENSG00000159479	MED8	0.182	4.440	2.663	0.009
ENSG00000159592	GPBP1L1	0.150	6.330	2.717	0.008
ENSG00000159685	CHCHD6	0.306	3.840	2.528	0.013
ENSG00000159692	CTBP1	0.163	5.708	2.173	0.033

ENSG00000159723	AGRP	0.759	-2.110	2.656	0.009
ENSG00000159784	FAM131B	0.178	6.913	2.548	0.013
ENSG00000159873	CCDC117	0.173	4.629	2.193	0.031
ENSG00000159915	ZNF233	0.174	3.788	2.346	0.021
ENSG00000159958	TNFRSF13C	0.666	0.163	2.389	0.019
ENSG00000160049	DFFA	0.164	5.409	2.730	0.008
ENSG00000160058	BSDC1	0.192	6.088	2.762	0.007
ENSG00000160075	SSU72	0.166	5.650	2.022	0.046
ENSG00000160097	FNDC5	0.206	4.913	2.050	0.044
ENSG00000160183	TMPRSS3	-0.924	-0.962	-2.085	0.040
ENSG00000160201	U2AF1	-0.915	1.938	-2.244	0.028
ENSG00000160214	RRP1	0.282	3.815	2.534	0.013
ENSG00000160229	ZNF66	-0.261	2.899	-2.676	0.009
ENSG00000160256	FAM207A	0.572	0.768	2.833	0.006
ENSG00000160321	ZNF208	-0.824	3.313	-3.176	0.002
ENSG00000160323	ADAMTS13	0.242	1.791	2.037	0.045
ENSG00000160551	TAOK1	0.121	9.188	2.245	0.027
ENSG00000160563	MED27	0.303	2.339	2.727	0.008
ENSG00000160584	SIK3	-0.189	7.295	-3.046	0.003
ENSG00000160588	MPZL3	0.329	0.591	2.082	0.040
ENSG00000160691	SHC1	0.336	3.276	3.368	0.001
ENSG00000160714	UBE2Q1	0.165	5.767	2.259	0.027
ENSG00000160746	ANO10	0.151	4.643	2.015	0.047
ENSG00000160752	FDPS	0.510	4.114	4.533	0.000
ENSG00000160799	CCDC12	0.359	3.102	2.667	0.009
ENSG00000160838	LRRC71	-0.725	-1.481	-2.869	0.005
ENSG00000160870	CYP3A7	-0.386	0.569	-2.338	0.022
ENSG00000160957	RECQL4	0.291	1.200	2.102	0.039
ENSG00000161013	MGAT4B	0.196	4.219	2.506	0.014
ENSG00000161040	FBXL13	-0.164	2.907	-2.183	0.032
ENSG00000161057	PSMC2	0.367	6.110	2.977	0.004
ENSG00000161204	ABCF3	0.159	5.353	2.390	0.019
ENSG00000161249	DMKN	-0.332	1.359	-2.052	0.043
ENSG00000161267	BDH1	-0.143	4.989	-2.119	0.037
ENSG00000161526	SAP30BP	0.266	5.110	3.327	0.001
ENSG00000161640	SIGLEC11	0.809	-1.858	2.138	0.036
ENSG00000161642	ZNF385A	-0.313	3.559	-2.313	0.023
ENSG00000161643	SIGLEC16	-0.629	-1.420	-2.205	0.030
ENSG00000161800	RACGAP1	0.241	3.474	2.997	0.004
ENSG00000161835	GRASP	0.253	3.510	2.096	0.039
ENSG00000161920	MED11	0.286	1.401	2.769	0.007
ENSG00000161929	SCIMP	-0.337	0.743	-2.132	0.036
ENSG00000161944	ASGR2	0.490	-0.676	2.226	0.029
ENSG00000161955	TNFSF13	-0.480	0.684	-2.193	0.031
ENSG00000161970	RPL26	0.543	5.888	2.843	0.006
ENSG00000161980	POLR3K	0.284	2.743	2.846	0.006
ENSG00000161981	SNRNP25	0.542	3.797	3.548	0.001

ENSG00000162069	BICDL2	0.572	-1.430	2.293	0.024
ENSG00000162076	FLYWCH2	0.328	2.177	2.188	0.032
ENSG00000162191	UBXN1	0.252	4.555	2.497	0.015
ENSG00000162236	STX5	0.180	3.113	2.235	0.028
ENSG00000162244	RPL29	0.373	4.162	2.081	0.041
ENSG00000162367	TAL1	0.518	1.370	2.181	0.032
ENSG00000162368	CMPK1	0.294	6.709	2.863	0.005
ENSG00000162385	MAGOH	0.319	2.733	2.667	0.009
ENSG00000162390	ACOT11	-0.489	4.911	-3.653	0.000
ENSG00000162402	USP24	0.106	7.551	2.030	0.046
ENSG00000162434	JAK1	0.133	7.371	2.089	0.040
ENSG00000162441	LZIC	0.252	5.044	3.010	0.003
ENSG00000162551	ALPL	0.366	2.639	2.193	0.031
ENSG00000162598	C1orf87	-1.006	-2.888	-1.995	0.049
ENSG00000162599	NFIA	-0.261	7.453	-2.327	0.022
ENSG00000162616	DNAJB4	0.200	6.582	2.012	0.048
ENSG00000162621	LRRC53	0.324	2.279	2.293	0.024
ENSG00000162636	FAM102B	0.337	7.087	3.041	0.003
ENSG00000162639	HENMT1	0.371	3.751	2.786	0.007
ENSG00000162642	C1orf52	0.336	5.008	3.481	0.001
ENSG00000162670	BRINP3	0.255	5.247	3.112	0.003
ENSG00000162687	KCNT2	0.216	5.854	2.618	0.011
ENSG00000162694	EXTL2	0.252	5.806	2.201	0.031
ENSG00000162699	DNAJA1P5	-0.664	1.445	-3.262	0.002
ENSG00000162704	ARPC5	0.349	5.809	3.479	0.001
ENSG00000162714	ZNF496	-0.110	4.633	-2.024	0.046
ENSG00000162722	TRIM58	0.534	2.157	2.794	0.006
ENSG00000162728	KCNJ9	0.286	6.831	3.602	0.001
ENSG00000162757	C1orf74	0.228	1.894	2.285	0.025
ENSG00000162782	TDRD5	0.725	1.872	3.463	0.001
ENSG00000162813	BPNT1	0.288	3.601	2.651	0.010
ENSG00000162814	SPATA17	0.313	1.220	2.182	0.032
ENSG00000162817	C1orf115	0.239	7.485	2.236	0.028
ENSG00000162851	TFB2M	0.366	2.928	3.301	0.001
ENSG00000162852	CNST	0.253	6.733	2.364	0.020
ENSG00000162910	MRPL55	0.379	3.010	3.756	0.000
ENSG00000162927	PUS10	-0.185	3.571	-2.233	0.028
ENSG00000162951	LRRTM1	-0.241	4.389	-2.586	0.011
ENSG00000162959	MEMO1	-0.255	2.999	-2.983	0.004
ENSG00000162961	DPY30	0.385	3.999	2.911	0.005
ENSG00000162972	C2orf47	0.283	2.969	2.874	0.005
ENSG00000162980	ARL5A	0.156	5.668	2.103	0.039
ENSG00000162994	CLHC1	-0.184	4.001	-2.436	0.017
ENSG00000162997	PRORSD1P	-0.345	1.296	-2.406	0.018
ENSG00000162998	FRZB	0.366	2.310	2.431	0.017
ENSG00000162999	DUSP19	0.237	4.761	2.426	0.017
ENSG00000163026	WDCP	0.199	3.255	2.232	0.028

ENSG00000163125	RPRD2	-0.247	6.482	-3.942	0.000
ENSG00000163132	MSX1	-0.451	0.626	-2.247	0.027
ENSG00000163155	LYSMD1	0.190	3.299	2.322	0.023
ENSG00000163156	SCNM1	0.289	3.126	3.101	0.003
ENSG00000163159	VPS72	0.198	4.708	2.276	0.025
ENSG00000163170	BOLA3	0.480	2.979	3.563	0.001
ENSG00000163319	MRPS18C	0.288	3.001	2.229	0.029
ENSG00000163380	LMOD3	0.413	0.745	2.431	0.017
ENSG00000163382	NAXE	0.210	3.882	2.272	0.026
ENSG00000163444	TMEM183A	0.261	6.152	2.314	0.023
ENSG00000163449	TMEM169	0.258	4.072	2.679	0.009
ENSG00000163466	ARPC2	0.238	6.166	3.176	0.002
ENSG00000163468	CCT3	0.344	6.334	3.160	0.002
ENSG00000163479	SSR2	0.322	4.048	4.128	0.000
ENSG00000163481	RNF25	0.207	3.442	2.276	0.025
ENSG00000163492	CCDC141	-0.309	2.178	-2.026	0.046
ENSG00000163512	AZI2	0.249	4.841	2.541	0.013
ENSG00000163513	TGFBR2	0.411	3.679	2.339	0.022
ENSG00000163517	HDAC11	0.194	4.751	2.149	0.035
ENSG00000163520	FBLN2	0.673	1.686	3.772	0.000
ENSG00000163527	STT3B	0.261	6.699	2.964	0.004
ENSG00000163528	CHCHD4	0.236	3.425	2.140	0.035
ENSG00000163531	NFASC	-0.203	7.987	-2.509	0.014
ENSG00000163536	SERPINI1	0.350	8.189	2.447	0.017
ENSG00000163539	CLASP2	0.249	9.305	2.495	0.015
ENSG00000163541	SUCLG1	0.268	5.223	2.554	0.013
ENSG00000163558	PRKCI	0.252	6.202	2.514	0.014
ENSG00000163584	RPL22L1	0.434	1.405	2.939	0.004
ENSG00000163605	PPP4R2	0.288	6.829	2.968	0.004
ENSG00000163606	CD200R1	0.417	0.257	2.455	0.016
ENSG00000163617	CCDC191	-0.169	4.411	-2.311	0.023
ENSG00000163624	CDS1	0.173	5.872	2.009	0.048
ENSG00000163634	THOC7	0.294	5.345	2.291	0.025
ENSG00000163635	ATXN7	-0.454	2.166	-3.853	0.000
ENSG00000163636	PSMD6	0.165	6.223	2.205	0.030
ENSG00000163673	DCLK3	0.386	2.709	2.633	0.010
ENSG00000163681	SLMAP	0.189	6.665	2.616	0.011
ENSG00000163682	RPL9	0.620	5.053	2.392	0.019
ENSG00000163683	SMIM14	0.204	6.780	2.128	0.036
ENSG00000163684	RPP14	0.129	4.980	2.178	0.032
ENSG00000163686	ABHD6	0.225	5.210	2.543	0.013
ENSG00000163703	CRELD1	0.170	5.026	2.281	0.025
ENSG00000163755	HPS3	0.153	4.490	2.187	0.032
ENSG00000163781	TOPBP1	0.273	6.059	3.123	0.002
ENSG00000163793	DNAJC5G	-0.498	1.852	-2.468	0.016
ENSG00000163798	SLC4A1AP	0.261	6.110	2.292	0.024
ENSG00000163807	KIAA1143	0.165	5.670	2.068	0.042

ENSG00000163810	TGM4	-0.652	-2.025	-2.013	0.047
ENSG00000163811	WDR43	0.180	5.560	2.015	0.047
ENSG00000163812	ZDHHC3	0.143	5.307	2.133	0.036
ENSG00000163818	LZTFL1	0.197	5.998	2.063	0.042
ENSG00000163832	ELP6	0.235	3.299	2.655	0.010
ENSG00000163864	NMNAT3	-0.216	4.084	-2.015	0.047
ENSG00000163873	GRIK3	0.261	6.242	2.537	0.013
ENSG00000163882	POLR2H	0.175	3.339	2.405	0.018
ENSG00000163898	LIPH	0.412	0.747	2.421	0.018
ENSG00000163902	RPN1	0.301	6.060	2.980	0.004
ENSG00000163918	RFC4	0.210	3.030	2.730	0.008
ENSG00000163930	BAP1	0.250	6.233	2.384	0.019
ENSG00000163938	GNL3	0.342	5.646	2.917	0.005
ENSG00000163945	UVSSA	-0.432	4.324	-3.538	0.001
ENSG00000163950	SLBP	0.240	4.846	2.380	0.020
ENSG00000163964	PIGX	0.205	5.287	2.232	0.028
ENSG00000164002	EXO5	0.297	2.457	2.995	0.004
ENSG00000164022	AIMP1	0.218	5.009	2.560	0.012
ENSG00000164024	METAP1	0.193	5.090	2.031	0.045
ENSG00000164031	DNAJB14	0.261	7.805	2.395	0.019
ENSG00000164032	H2AFZ	0.477	4.553	2.568	0.012
ENSG00000164038	SLC9B2	0.171	5.685	2.038	0.045
ENSG00000164048	ZNF589	-0.143	4.598	-2.194	0.031
ENSG00000164049	FBXW12	-0.389	0.624	-2.491	0.015
ENSG00000164051	CCDC51	0.260	2.579	2.166	0.033
ENSG00000164056	SPRY1	-0.275	3.161	-2.614	0.011
ENSG00000164061	BSN	-0.299	8.428	-2.137	0.036
ENSG00000164062	APEH	0.227	4.826	2.937	0.004
ENSG00000164087	POC1A	0.332	0.152	2.209	0.030
ENSG00000164096	C4orf3	0.150	6.808	2.175	0.032
ENSG00000164109	MAD2L1	0.340	2.741	2.125	0.037
ENSG00000164111	ANXA5	0.257	5.067	2.073	0.041
ENSG00000164112	TMEM155	0.526	5.420	3.464	0.001
ENSG00000164120	HPGD	0.746	-0.091	2.688	0.009
ENSG00000164125	FAM198B	-0.240	6.287	-2.115	0.037
ENSG00000164134	NAA15	0.113	6.531	2.092	0.040
ENSG00000164163	ABCE1	0.339	5.814	2.779	0.007
ENSG00000164167	LSM6	0.283	3.026	2.306	0.024
ENSG00000164168	TMEM184C	0.156	5.755	2.468	0.016
ENSG00000164172	MOCS2	0.235	6.249	2.124	0.037
ENSG00000164182	NDUFAF2	0.525	3.109	2.810	0.006
ENSG00000164187	LMBRD2	0.227	7.815	2.268	0.026
ENSG00000164209	SLC25A46	0.257	7.473	2.450	0.016
ENSG00000164211	STARD4	0.499	6.289	4.613	0.000
ENSG00000164221	CCDC112	0.193	4.671	2.141	0.035
ENSG00000164252	AGGF1	0.145	5.034	2.016	0.047
ENSG00000164253	WDR41	0.201	6.241	2.897	0.005

ENSG00000164258	NDUFS4	0.418	5.117	2.588	0.011
ENSG00000164303	ENPP6	0.381	3.037	2.876	0.005
ENSG00000164309	CMYA5	-0.231	5.747	-2.649	0.010
ENSG00000164318	EGFLAM	0.758	-1.709	2.245	0.027
ENSG00000164323	CFAP97	0.161	6.014	2.185	0.032
ENSG00000164331	ANKRA2	0.228	3.743	2.080	0.041
ENSG00000164332	UBLCP1	0.217	5.708	2.279	0.025
ENSG00000164346	NSA2	0.354	4.988	2.047	0.044
ENSG00000164347	GFM2	0.236	5.607	2.180	0.032
ENSG00000164402	SEPTIN8	0.195	7.806	2.541	0.013
ENSG00000164405	UQCRQ	0.485	4.933	3.226	0.002
ENSG00000164466	SFXN1	0.192	6.280	2.938	0.004
ENSG00000164484	TMEM200A	0.461	3.095	2.496	0.015
ENSG00000164494	PDSS2	0.269	3.572	3.072	0.003
ENSG00000164574	GALNT10	0.238	4.055	2.479	0.015
ENSG00000164587	RPS14	0.381	5.233	2.275	0.026
ENSG00000164591	MYOZ3	0.264	1.403	2.058	0.043
ENSG00000164603	BMT2	0.198	4.827	2.113	0.038
ENSG00000164609	SLU7	0.221	7.061	2.328	0.022
ENSG00000164611	PTTG1	0.621	0.767	2.966	0.004
ENSG00000164631	ZNF12	0.171	5.389	2.581	0.012
ENSG00000164645	C7orf62	-0.404	0.713	-2.192	0.031
ENSG00000164649	CDCA7L	0.279	2.324	2.241	0.028
ENSG00000164663	USP49	-0.326	4.622	-3.564	0.001
ENSG00000164683	HEY1	0.264	4.518	2.778	0.007
ENSG00000164687	FABP5	0.303	1.997	2.000	0.049
ENSG00000164733	CTSB	0.211	7.764	2.549	0.013
ENSG00000164751	PEX2	0.236	4.558	2.836	0.006
ENSG00000164761	TNFRSF11B	-0.645	0.696	-2.506	0.014
ENSG00000164764	SBSPON	-0.317	1.468	-2.580	0.012
ENSG00000164776	PHKG1	-0.540	1.570	-3.180	0.002
ENSG00000164815	ORC5	0.307	4.556	2.559	0.012
ENSG00000164867	NOS3	0.550	0.555	2.919	0.005
ENSG00000164885	CDK5	0.381	4.137	2.552	0.013
ENSG00000164902	PHAX	0.192	6.291	2.259	0.027
ENSG00000164919	COX6C	0.361	6.710	2.460	0.016
ENSG00000164933	SLC25A32	-0.488	0.655	-2.838	0.006
ENSG00000164934	DCAF13	0.354	4.473	4.235	0.000
ENSG00000164935	DCSTAMP	-0.396	-0.013	-2.127	0.036
ENSG00000164978	NUDT2	0.384	3.000	3.183	0.002
ENSG00000164985	PSIP1	0.191	7.712	2.219	0.029
ENSG00000165023	DIRAS2	0.247	8.982	2.093	0.039
ENSG00000165072	MAMDC2	-0.248	1.966	-2.095	0.039
ENSG00000165084	C8orf34	0.300	4.055	2.660	0.009
ENSG00000165119	HNRNPK	0.325	7.841	2.420	0.018
ENSG00000165138	ANKS6	-0.200	4.143	-2.119	0.037
ENSG00000165185	KIAA1958	-0.189	5.191	-2.171	0.033

ENSG00000165204	OR1K1	-0.752	-2.142	-2.162	0.034
ENSG00000165238	WNK2	-0.251	6.111	-2.095	0.039
ENSG00000165264	NDUFB6	0.433	3.644	2.722	0.008
ENSG00000165280	VCP	0.243	7.210	2.820	0.006
ENSG00000165282	PIGO	-0.186	3.282	-2.396	0.019
ENSG00000165283	STOML2	0.328	3.949	2.978	0.004
ENSG00000165300	SLITRK5	-0.172	7.309	-2.164	0.033
ENSG00000165449	SLC16A9	-0.608	3.582	-2.618	0.011
ENSG00000165478	HEPACAM	-0.316	5.630	-2.421	0.018
ENSG00000165501	LRR1	-0.321	0.103	-2.151	0.034
ENSG00000165502	RPL36AL	0.235	5.782	2.619	0.011
ENSG00000165506	DNAAF2	0.205	3.224	2.444	0.017
ENSG00000165525	NEMF	0.227	7.286	2.346	0.021
ENSG00000165572	KBTBD6	0.197	6.036	2.155	0.034
ENSG00000165606	DRGX	-0.750	0.716	-2.943	0.004
ENSG00000165629	ATP5C1	0.377	5.974	2.650	0.010
ENSG00000165630	PRPF18	0.289	4.064	2.190	0.031
ENSG00000165633	VSTM4	-0.144	4.197	-2.139	0.035
ENSG00000165637	VDAC2	0.376	4.931	2.468	0.016
ENSG00000165660	FAM175B	0.266	5.039	3.576	0.001
ENSG00000165672	PRDX3	0.378	5.800	3.426	0.001
ENSG00000165678	GHITM	0.334	8.401	2.906	0.005
ENSG00000165685	TMEM52B	0.772	0.354	2.460	0.016
ENSG00000165688	PMPCA	0.224	4.269	2.070	0.042
ENSG00000165714	BORCS5	-0.265	5.130	-4.598	0.000
ENSG00000165724	ZMYND19	0.254	2.984	3.017	0.003
ENSG00000165732	DDX21	0.277	5.933	3.649	0.000
ENSG00000165752	STK32C	0.294	3.717	2.087	0.040
ENSG00000165810	BTNL9	0.426	3.260	2.226	0.029
ENSG00000165813	CCDC186	0.182	6.976	2.362	0.021
ENSG00000165816	VWA2	0.370	0.119	2.296	0.024
ENSG00000165821	SALL2	-0.252	5.089	-3.094	0.003
ENSG00000165832	TRUB1	0.278	6.117	2.280	0.025
ENSG00000165837	ERICH6B	-0.474	-1.360	-2.403	0.019
ENSG00000165898	ISCA2	0.193	3.726	2.772	0.007
ENSG00000165916	PSMC3	0.386	5.909	2.471	0.016
ENSG00000165929	TC2N	0.476	4.041	2.939	0.004
ENSG00000165934	CPSF2	0.148	6.202	2.669	0.009
ENSG00000165943	MOAP1	0.252	8.121	2.224	0.029
ENSG00000165948	IFI27L1	0.502	1.384	3.372	0.001
ENSG00000165949	IFI27	0.387	2.653	2.207	0.030
ENSG00000165973	NELL1	0.372	5.285	2.888	0.005
ENSG00000165996	HACD1	0.234	1.843	2.052	0.043
ENSG00000166068	SPRED1	0.222	7.519	2.709	0.008
ENSG00000166111	SVOP	0.319	6.872	3.197	0.002
ENSG00000166128	RAB8B	0.248	6.092	3.402	0.001
ENSG00000166130	IKBIP	0.379	1.806	2.890	0.005

ENSG00000166136	NDUFB8	0.287	4.012	2.555	0.012
ENSG00000166147	FBN1	-0.233	5.702	-2.911	0.005
ENSG00000166148	AVPR1A	0.592	0.597	3.062	0.003
ENSG00000166164	BRD7	0.249	5.347	2.103	0.039
ENSG00000166171	DPCD	0.448	3.468	3.343	0.001
ENSG00000166181	API5	0.270	6.404	2.707	0.008
ENSG00000166183	ASPG	0.973	-2.927	2.528	0.013
ENSG00000166197	NOLC1	0.154	6.923	2.079	0.041
ENSG00000166226	CCT2	0.411	6.279	3.096	0.003
ENSG00000166260	COX11	0.160	5.928	2.249	0.027
ENSG00000166262	FAM227B	-0.283	2.178	-2.681	0.009
ENSG00000166265	CYYR1	0.342	2.249	2.379	0.020
ENSG00000166266	CUL5	0.203	6.910	2.137	0.036
ENSG00000166275	BORCS7	0.242	3.882	2.003	0.049
ENSG00000166321	NUDT13	-0.348	2.308	-3.223	0.002
ENSG00000166333	ILK	-0.349	1.251	-2.028	0.046
ENSG00000166337	TAF10	-0.293	2.443	-3.212	0.002
ENSG00000166340	TPP1	-0.496	4.972	-3.164	0.002
ENSG00000166347	CYB5A	0.244	3.423	2.631	0.010
ENSG00000166352	C11orf74	0.267	3.721	2.164	0.033
ENSG00000166402	TUB	0.155	7.470	2.370	0.020
ENSG00000166411	IDH3A	0.247	5.633	2.175	0.033
ENSG00000166436	TRIM66	-0.274	4.749	-3.491	0.001
ENSG00000166441	RPL27A	0.324	5.387	2.452	0.016
ENSG00000166448	TMEM130	-0.236	7.453	-2.422	0.018
ENSG00000166451	CENPN	0.397	1.293	2.912	0.005
ENSG00000166452	AKIP1	0.233	2.892	2.491	0.015
ENSG00000166471	TMEM41B	0.179	5.774	2.289	0.025
ENSG00000166478	ZNF143	0.242	4.374	3.136	0.002
ENSG00000166479	TMX3	0.208	7.017	2.320	0.023
ENSG00000166501	PRKCB	0.254	9.910	2.325	0.023
ENSG00000166503	RP11-382A20.3	0.191	7.996	2.253	0.027
ENSG00000166510	CCDC68	0.270	3.705	2.052	0.043
ENSG00000166535	A2ML1	-0.507	2.553	-3.279	0.002
ENSG00000166557	TMED3	0.265	3.945	4.157	0.000
ENSG00000166562	SEC11C	0.240	5.543	2.193	0.031
ENSG00000166582	CENPV	0.182	3.532	2.372	0.020
ENSG00000166595	FAM96B	0.264	2.712	2.371	0.020
ENSG00000166598	HSP90B1	0.348	8.341	3.010	0.003
ENSG00000166689	PLEKHA7	-0.374	2.730	-3.122	0.002
ENSG00000166710	B2M	0.328	6.862	2.045	0.044
ENSG00000166716	ZNF592	-0.184	5.151	-2.620	0.010
ENSG00000166734	CASC4	0.206	7.547	2.388	0.019
ENSG00000166741	NNMT	0.550	0.399	2.965	0.004
ENSG00000166747	AP1G1	0.157	7.247	2.768	0.007
ENSG00000166762	CATSPER2	-0.440	4.580	-2.919	0.005
ENSG00000166783	KIAA0430	-0.226	7.745	-3.021	0.003

ENSG00000166794	PPIB	0.355	4.279	3.355	0.001
ENSG00000166797	FAM96A	0.292	3.215	3.143	0.002
ENSG00000166819	PLIN1	-0.592	0.930	-2.899	0.005
ENSG00000166833	NAV2	-0.228	6.404	-2.375	0.020
ENSG00000166847	DCTN5	0.162	7.786	2.559	0.012
ENSG00000166855	CLPX	0.140	6.139	2.102	0.039
ENSG00000166856	GPR182	-0.398	0.122	-2.229	0.029
ENSG00000166896	ATP23	0.340	1.844	3.186	0.002
ENSG00000166902	MRPL16	0.217	4.145	2.716	0.008
ENSG00000166913	YWHAB	0.232	9.042	2.067	0.042
ENSG00000166922	SCG5	0.354	6.903	2.749	0.007
ENSG00000166946	CCNDBP1	0.218	5.394	2.388	0.019
ENSG00000166960	CCDC178	0.433	0.565	2.492	0.015
ENSG00000166971	AKTIP	0.241	5.610	2.206	0.030
ENSG00000166986	MARS	0.135	5.935	2.179	0.032
ENSG00000167004	PDIA3	0.528	5.470	2.714	0.008
ENSG00000167005	NUDT21	0.239	7.671	2.199	0.031
ENSG00000167085	PHB	0.426	3.948	2.817	0.006
ENSG00000167088	SNRPD1	0.295	5.348	2.958	0.004
ENSG00000167106	FAM102A	-0.256	5.062	-2.719	0.008
ENSG00000167110	GOLGA2	0.224	5.879	2.804	0.006
ENSG00000167112	TRUB2	0.304	5.041	3.551	0.001
ENSG00000167118	URM1	0.216	3.163	2.295	0.024
ENSG00000167182	SP2	-0.466	2.487	-3.525	0.001
ENSG00000167186	COQ7	0.178	4.627	2.061	0.043
ENSG00000167193	CRK	0.196	6.036	2.609	0.011
ENSG00000167258	CDK12	-0.128	6.254	-2.815	0.006
ENSG00000167264	DUS2	0.250	2.169	2.116	0.037
ENSG00000167283	ATP5L	0.285	5.310	2.738	0.008
ENSG00000167325	RRM1	0.294	5.047	2.725	0.008
ENSG00000167363	FN3K	-0.231	3.640	-2.435	0.017
ENSG00000167460	TPM4	0.351	5.960	3.582	0.001
ENSG00000167461	RAB8A	0.377	3.251	3.332	0.001
ENSG00000167468	GPX4	0.415	5.367	2.182	0.032
ENSG00000167508	MVD	0.208	3.600	2.053	0.043
ENSG00000167515	TRAPPC2L	0.289	3.892	2.752	0.007
ENSG00000167524	SGK494	-0.322	3.644	-2.122	0.037
ENSG00000167526	RPL13	0.308	5.786	2.482	0.015
ENSG00000167550	RHEBL1	0.486	0.604	2.393	0.019
ENSG00000167552	TUBA1A	0.447	8.243	3.472	0.001
ENSG00000167588	GPD1	0.551	2.662	3.189	0.002
ENSG00000167600	CYP2S1	-0.628	-1.196	-3.086	0.003
ENSG00000167632	TRAPPC9	-0.143	5.160	-2.296	0.024
ENSG00000167642	SPINT2	0.363	5.117	2.884	0.005
ENSG00000167658	EEF2	0.210	8.990	2.088	0.040
ENSG00000167671	UBXN6	0.256	5.331	2.648	0.010
ENSG00000167676	PLIN4	-0.656	1.776	-3.166	0.002

ENSG00000167695	FAM57A	-0.235	2.275	-2.205	0.030
ENSG00000167699	GLOD4	0.171	5.465	2.183	0.032
ENSG00000167721	TSR1	0.243	5.133	2.883	0.005
ENSG00000167792	NDUFV1	0.197	6.492	2.243	0.028
ENSG00000167797	CDK2AP2	0.295	1.919	2.563	0.012
ENSG00000167842	MIS12	0.164	3.714	2.042	0.044
ENSG00000167862	MRPL58	0.328	2.582	2.346	0.021
ENSG00000167863	ATP5H	0.521	5.464	2.867	0.005
ENSG00000167904	TMEM68	0.201	4.214	2.386	0.019
ENSG00000167967	E4F1	0.221	3.211	2.458	0.016
ENSG00000167978	SRRM2	-0.333	8.726	-2.790	0.007
ENSG00000168000	BSCL2	0.282	2.667	2.503	0.014
ENSG00000168002	POLR2G	0.330	2.838	3.065	0.003
ENSG00000168014	C2CD3	-0.131	5.514	-2.632	0.010
ENSG00000168028	RPSA	0.757	5.452	2.381	0.020
ENSG00000168036	CTNNB1	0.236	7.977	2.632	0.010
ENSG00000168040	FADD	0.420	0.556	3.076	0.003
ENSG00000168056	LTBP3	-0.233	4.600	-2.113	0.038
ENSG00000168066	SF1	-0.189	6.258	-2.842	0.006
ENSG00000168067	MAP4K2	0.154	4.816	2.064	0.042
ENSG00000168092	PAFAH1B2	0.270	7.677	2.699	0.008
ENSG00000168101	NUDT16L1	0.283	2.282	2.838	0.006
ENSG00000168116	KIAA1586	0.202	5.401	2.057	0.043
ENSG00000168118	RAB4A	0.190	5.641	2.031	0.045
ENSG00000168216	LMBRD1	0.178	6.375	2.169	0.033
ENSG00000168246	UBTD2	0.220	4.231	2.726	0.008
ENSG00000168259	DNAJC7	0.237	6.477	2.629	0.010
ENSG00000168275	COA6	0.367	3.181	2.851	0.006
ENSG00000168286	THAP11	0.346	2.340	2.718	0.008
ENSG00000168288	MMADHC	0.363	5.259	3.016	0.003
ENSG00000168291	PDHB	0.385	5.615	3.489	0.001
ENSG00000168300	PCMTD1	0.159	6.689	2.423	0.018
ENSG00000168306	ACOX2	-0.394	1.203	-2.457	0.016
ENSG00000168374	ARF4	0.275	5.754	3.351	0.001
ENSG00000168397	ATG4B	0.189	4.601	2.276	0.025
ENSG00000168405	CMAHP	-0.472	3.467	-3.710	0.000
ENSG00000168496	FEN1	0.288	3.397	3.087	0.003
ENSG00000168594	ADAM29	-0.519	0.136	-3.131	0.002
ENSG00000168615	ADAM9	0.221	6.415	2.552	0.013
ENSG00000168653	NDUFS5	0.368	6.534	2.668	0.009
ENSG00000168658	VWA3B	-0.484	-0.470	-2.095	0.039
ENSG00000168675	LDLRAD4	0.164	7.208	2.247	0.027
ENSG00000168685	IL7R	0.825	-0.052	3.054	0.003
ENSG00000168701	TMEM208	0.393	3.015	3.751	0.000
ENSG00000168724	DNAJC21	0.168	6.545	2.150	0.035
ENSG00000168734	PKIG	0.253	4.854	2.763	0.007
ENSG00000168743	NPNT	0.268	2.980	2.192	0.031

ENSG00000168765	GSTM4	-0.232	3.704	-2.461	0.016
ENSG00000168772	CXXC4	0.208	5.418	2.412	0.018
ENSG00000168781	PPIP5K1	-0.255	5.239	-2.024	0.046
ENSG00000168813	ZNF507	-0.127	6.339	-2.726	0.008
ENSG00000168818	STX18	0.199	4.209	2.176	0.032
ENSG00000168827	GFM1	0.200	6.487	2.450	0.016
ENSG00000168830	HTR1E	-0.368	3.015	-3.422	0.001
ENSG00000168843	FSTL5	0.367	5.717	2.734	0.008
ENSG00000168876	ANKRD49	0.188	3.556	2.481	0.015
ENSG00000168883	USP39	0.152	4.038	2.240	0.028
ENSG00000168884	TNIP2	0.279	1.876	2.055	0.043
ENSG00000168894	RNF181	0.466	2.666	3.758	0.000
ENSG00000168924	LETM1	0.140	5.701	2.376	0.020
ENSG00000168958	MFF	0.154	6.115	2.193	0.031
ENSG00000169006	NTSR2	-0.499	3.061	-2.266	0.026
ENSG00000169019	COMMD8	0.489	3.566	3.427	0.001
ENSG00000169020	ATP5I	0.355	4.002	2.513	0.014
ENSG00000169021	UQCRFS1	0.212	5.203	2.062	0.042
ENSG00000169045	HNRNPH1	0.132	7.615	2.326	0.022
ENSG00000169075	RP3-509L4.3	-0.569	1.427	-3.210	0.002
ENSG00000169085	C8orf46	0.417	4.403	3.327	0.001
ENSG00000169087	HSPBAP1	-0.205	3.381	-2.709	0.008
ENSG00000169129	AFAP1L2	0.273	4.558	3.088	0.003
ENSG00000169139	UBE2V2	0.225	7.113	2.074	0.041
ENSG00000169189	NSMCE1	0.362	3.509	3.035	0.003
ENSG00000169193	CCDC126	0.231	4.234	2.597	0.011
ENSG00000169217	CD2BP2	0.270	5.289	3.678	0.000
ENSG00000169241	SLC50A1	0.347	1.373	3.154	0.002
ENSG00000169255	B3GALNT1	0.283	5.858	2.653	0.010
ENSG00000169288	MRPL1	0.287	4.127	2.192	0.031
ENSG00000169313	P2RY12	0.629	4.920	2.033	0.045
ENSG00000169371	SNUPN	0.260	3.197	2.825	0.006
ENSG00000169490	TM2D2	0.233	5.214	2.415	0.018
ENSG00000169507	SLC38A11	-0.338	3.488	-2.379	0.020
ENSG00000169567	HINT1	0.477	5.751	3.054	0.003
ENSG00000169604	ANTXR1	-0.272	5.359	-2.356	0.021
ENSG00000169612	FAM103A1	0.276	2.075	2.671	0.009
ENSG00000169621	APLF	0.322	5.390	2.734	0.008
ENSG00000169689	STRA13	0.495	1.633	3.475	0.001
ENSG00000169714	CNBP	0.161	7.484	2.036	0.045
ENSG00000169740	ZNF32	0.228	3.584	2.453	0.016
ENSG00000169764	UGP2	0.266	7.805	2.558	0.012
ENSG00000169813	HNRNPF	0.211	5.173	2.505	0.014
ENSG00000169814	BTD	-0.146	3.857	-2.531	0.013
ENSG00000169826	CSGALNACT2	0.159	5.994	2.294	0.024
ENSG00000169860	P2RY1	-0.494	4.483	-2.071	0.042
ENSG00000169884	WNT10B	0.207	2.855	2.060	0.043

ENSG00000169896	ITGAM	0.602	1.850	1.990	0.050
ENSG00000169902	TPST1	0.210	4.824	2.419	0.018
ENSG00000169908	TM4SF1	0.551	3.023	2.230	0.028
ENSG00000169946	ZFPM2	-0.245	5.380	-3.152	0.002
ENSG00000169976	SF3B5	0.318	2.728	2.720	0.008
ENSG00000170004	CHD3	0.191	7.713	2.381	0.020
ENSG00000170017	ALCAM	0.318	7.403	2.782	0.007
ENSG00000170075	GPR37L1	-0.358	5.844	-2.098	0.039
ENSG00000170113	NIPA1	0.210	7.190	2.369	0.020
ENSG00000170142	UBE2E1	0.193	5.515	2.462	0.016
ENSG00000170165	CR848007.2	-0.841	1.768	-2.570	0.012
ENSG00000170231	FABP6	0.430	2.049	2.369	0.020
ENSG00000170270	C14orf142	0.299	2.357	2.675	0.009
ENSG00000170290	SLN	0.798	0.470	2.390	0.019
ENSG00000170291	ELP5	0.289	3.690	3.104	0.003
ENSG00000170293	CMTM8	0.389	-0.413	2.279	0.025
ENSG00000170310	STX8	0.269	4.070	2.457	0.016
ENSG00000170315	UBB	0.178	6.908	2.102	0.039
ENSG00000170340	B3GNT2	0.313	4.494	2.762	0.007
ENSG00000170417	TMEM182	0.294	1.753	2.215	0.030
ENSG00000170445	HARS	0.162	6.335	2.020	0.047
ENSG00000170473	PYM1	0.233	3.165	2.461	0.016
ENSG00000170502	NUDT9	0.164	4.393	1.998	0.049
ENSG00000170515	PA2G4	0.161	5.068	2.072	0.041
ENSG00000170523	KRT83	0.938	-1.814	2.191	0.031
ENSG00000170606	HSPA4	0.276	7.823	2.563	0.012
ENSG00000170653	ATF7	-0.383	3.769	-3.396	0.001
ENSG00000170745	KCNS3	0.421	2.900	2.527	0.013
ENSG00000170786	SDR16C5	0.431	2.872	2.652	0.010
ENSG00000170791	CHCHD7	0.242	5.492	2.242	0.028
ENSG00000170832	USP32	0.188	7.380	2.091	0.040
ENSG00000170846	AC093323.3	0.253	4.285	2.971	0.004
ENSG00000170854	MINA	0.167	4.643	2.065	0.042
ENSG00000170855	TRIAP1	0.325	3.070	2.429	0.017
ENSG00000170860	LSM3	0.408	4.523	2.700	0.008
ENSG00000170876	TMEM43	0.132	4.755	2.471	0.016
ENSG00000170881	RNF139	0.190	4.787	2.297	0.024
ENSG00000170906	NDUFA3	0.289	3.096	2.103	0.039
ENSG00000170919	TPT1-AS1	-0.439	4.193	-2.927	0.004
ENSG00000170921	TANC2	-0.134	8.305	-2.031	0.046
ENSG00000170927	PKHD1	-0.506	-1.335	-2.241	0.028
ENSG00000170946	DNAJC24	0.189	4.586	2.557	0.012
ENSG00000170961	HAS2	0.353	0.517	2.302	0.024
ENSG00000170967	DDI1	-1.229	-2.677	-2.734	0.008
ENSG00000171044	XKR6	-0.366	3.802	-3.254	0.002
ENSG00000171055	FEZ2	0.215	6.753	2.844	0.006
ENSG00000171067	C11orf24	0.243	2.916	2.208	0.030

ENSG00000171115	GIMAP8	0.539	2.301	2.216	0.029
ENSG00000171121	KCNMB3	-0.356	1.681	-2.479	0.015
ENSG00000171130	ATP6V0E2	0.212	5.993	2.992	0.004
ENSG00000171135	JAGN1	0.206	2.409	2.037	0.045
ENSG00000171202	TMEM126A	0.499	3.057	3.197	0.002
ENSG00000171204	TMEM126B	0.254	4.817	2.374	0.020
ENSG00000171208	NETO2	0.316	5.940	2.744	0.007
ENSG00000171217	CLDN20	-0.424	1.098	-2.638	0.010
ENSG00000171224	C10orf35	0.406	2.583	3.107	0.003
ENSG00000171262	FAM98B	-0.136	5.571	-2.111	0.038
ENSG00000171295	ZNF440	0.159	4.212	3.144	0.002
ENSG00000171303	KCNK3	0.271	3.336	2.277	0.025
ENSG00000171421	MRPL36	0.352	1.667	2.711	0.008
ENSG00000171435	KSR2	-0.303	7.128	-2.918	0.005
ENSG00000171444	MCC	-0.264	5.556	-2.243	0.028
ENSG00000171451	DSEL	0.194	5.272	2.504	0.014
ENSG00000171456	ASXL1	-0.119	5.647	-2.550	0.013
ENSG00000171466	ZNF562	0.108	5.789	2.061	0.043
ENSG00000171467	ZNF318	-0.092	6.303	-2.002	0.049
ENSG00000171475	WIPF2	-0.146	6.677	-2.338	0.022
ENSG00000171490	RSL1D1	0.229	6.465	3.122	0.002
ENSG00000171492	LRRC8D	0.207	5.647	2.086	0.040
ENSG00000171497	PPID	0.332	5.374	2.799	0.006
ENSG00000171502	COL24A1	0.341	5.366	2.104	0.038
ENSG00000171530	TBCA	0.531	4.251	2.663	0.009
ENSG00000171566	PLRG1	0.168	6.076	2.060	0.043
ENSG00000171631	P2RY6	0.718	-1.165	2.493	0.015
ENSG00000171681	ATF7IP	-0.124	6.998	-2.289	0.025
ENSG00000171700	RGS19	0.338	0.606	2.536	0.013
ENSG00000171757	LRRC34	-0.252	2.943	-2.932	0.004
ENSG00000171791	BCL2	-0.394	5.488	-3.714	0.000
ENSG00000171819	ANGPTL7	-0.629	0.365	-3.933	0.000
ENSG00000171827	ZNF570	0.199	4.974	2.840	0.006
ENSG00000171858	RPS21	0.847	3.858	2.485	0.015
ENSG00000171861	MRM3	0.264	2.338	2.338	0.022
ENSG00000171862	PTEN	0.201	7.791	2.813	0.006
ENSG00000171863	RPS7	0.701	4.529	2.443	0.017
ENSG00000171865	RNASEH1	0.424	3.992	2.791	0.007
ENSG00000171903	CYP4F11	-0.670	2.326	-2.821	0.006
ENSG00000171928	TVP23B	0.197	4.066	2.083	0.040
ENSG00000171951	SCG2	0.416	7.836	2.723	0.008
ENSG00000171960	PPIH	0.379	2.121	2.889	0.005
ENSG00000172007	RAB33B	0.175	4.442	2.185	0.032
ENSG00000172020	GAP43	0.425	8.474	3.324	0.001
ENSG00000172053	QARS	0.271	4.221	2.718	0.008
ENSG00000172086	KRCC1	0.214	3.917	2.068	0.042
ENSG00000172115	CYCS	0.357	7.857	3.002	0.004

ENSG00000172159	FRMD3	0.197	4.233	2.137	0.036
ENSG00000172164	SNTB1	-0.287	3.166	-2.539	0.013
ENSG00000172167	MTBP	-0.308	2.859	-3.312	0.001
ENSG00000172172	MRPL13	0.306	3.407	2.617	0.011
ENSG00000172239	PAIP1	0.466	4.902	2.690	0.009
ENSG00000172264	MACROD2	0.158	5.052	2.190	0.031
ENSG00000172331	BPGM	0.234	4.380	2.360	0.021
ENSG00000172336	POP7	0.318	2.964	2.727	0.008
ENSG00000172404	DNAJB7	-0.667	0.036	-4.344	0.000
ENSG00000172428	COPS9	0.572	2.904	2.682	0.009
ENSG00000172469	MANEA	0.209	4.113	2.246	0.027
ENSG00000172493	AFF1	-0.229	6.099	-3.141	0.002
ENSG00000172500	FIBP	0.230	4.501	2.687	0.009
ENSG00000172531	PPP1CA	0.345	4.239	3.757	0.000
ENSG00000172575	RASGRP1	0.289	6.540	2.457	0.016
ENSG00000172586	CHCHD1	0.466	2.615	3.661	0.000
ENSG00000172590	MRPL52	0.206	2.933	2.099	0.039
ENSG00000172602	RND1	0.234	4.533	2.321	0.023
ENSG00000172661	FAM21C	-0.191	5.402	-2.099	0.039
ENSG00000172716	SLFN11	0.418	2.785	2.880	0.005
ENSG00000172728	FUT10	-0.421	4.326	-3.153	0.002
ENSG00000172732	MUS81	0.238	3.229	2.796	0.006
ENSG00000172757	CFL1	0.190	7.243	2.024	0.046
ENSG00000172775	FAM192A	0.261	6.854	2.617	0.011
ENSG00000172795	DCP2	0.270	6.846	2.847	0.006
ENSG00000172809	RPL38	0.603	6.492	2.635	0.010
ENSG00000172819	RARG	-0.235	1.753	-2.075	0.041
ENSG00000172830	SSH3	-0.209	2.280	-2.451	0.016
ENSG00000172878	METAP1D	-0.206	3.274	-2.500	0.014
ENSG00000173020	GRK2	0.231	5.989	2.038	0.045
ENSG00000173065	FAM222B	-0.321	3.823	-2.624	0.010
ENSG00000173110	HSPA6	-1.339	-1.474	-2.195	0.031
ENSG00000173113	TRMT112	0.354	4.322	2.364	0.020
ENSG00000173141	MRPL57	0.211	4.237	2.749	0.007
ENSG00000173153	ESRRA	0.227	2.835	2.433	0.017
ENSG00000173207	CKS1B	0.338	-0.169	2.061	0.042
ENSG00000173221	GLRX	0.267	4.520	2.353	0.021
ENSG00000173226	IQCB1	0.235	4.731	2.279	0.025
ENSG00000173261	PLAC8L1	-0.439	-0.868	-2.331	0.022
ENSG00000173267	SNCG	0.383	5.288	2.335	0.022
ENSG00000173402	DAG1	-0.246	5.462	-2.493	0.015
ENSG00000173418	NAA20	0.190	4.793	2.110	0.038
ENSG00000173436	MINOS1	0.193	4.413	2.022	0.046
ENSG00000173442	EHBP1L1	0.306	3.002	3.288	0.001
ENSG00000173452	TMEM196	0.284	4.395	2.462	0.016
ENSG00000173457	PPP1R14B	0.430	1.161	2.004	0.048
ENSG00000173486	FKBP2	0.404	4.035	2.886	0.005

ENSG00000173517	PEAK1	-0.149	7.405	-2.418	0.018
ENSG00000173540	GMPPB	0.229	2.502	2.470	0.016
ENSG00000173545	ZNF622	0.254	4.754	2.356	0.021
ENSG00000173614	NMNAT1	0.167	3.319	2.409	0.018
ENSG00000173627	APOBEC4	-0.310	2.233	-2.234	0.028
ENSG00000173660	UQCRH	0.318	6.266	2.127	0.036
ENSG00000173692	PSMD1	0.284	7.333	2.692	0.009
ENSG00000173821	RNF213	-0.180	7.051	-2.174	0.033
ENSG00000173848	NET1	0.347	5.626	2.287	0.025
ENSG00000173889	PHC3	-0.140	7.340	-2.989	0.004
ENSG00000173915	USMG5	0.426	5.345	2.651	0.010
ENSG00000173933	RBM4	-0.198	4.190	-2.110	0.038
ENSG00000173992	CCS	0.217	2.538	2.084	0.040
ENSG00000174007	CEP19	0.330	3.467	3.035	0.003
ENSG00000174013	FBXO45	0.192	6.168	2.097	0.039
ENSG00000174109	C16orf91	0.644	1.517	4.396	0.000
ENSG00000174123	TLR10	0.693	1.015	2.518	0.014
ENSG00000174130	TLR6	0.601	2.149	2.240	0.028
ENSG00000174132	FAM174A	0.320	3.733	2.710	0.008
ENSG00000174151	CYB561D1	0.277	3.832	3.804	0.000
ENSG00000174173	TRMT10C	0.323	4.652	2.709	0.008
ENSG00000174243	DDX23	0.287	5.810	3.148	0.002
ENSG00000174306	ZHX3	-0.180	7.088	-2.949	0.004
ENSG00000174332	GLIS1	-0.433	1.248	-2.613	0.011
ENSG00000174370	C11orf45	0.415	0.991	3.288	0.001
ENSG00000174405	LIG4	0.235	6.154	2.481	0.015
ENSG00000174442	ZWILCH	0.357	3.200	2.796	0.006
ENSG00000174444	RPL4	0.485	7.114	2.232	0.028
ENSG00000174446	SNAPC5	0.252	4.135	2.500	0.014
ENSG00000174456	C12orf76	0.224	5.684	2.843	0.006
ENSG00000174501	ANKRD36C	-0.158	7.318	-2.086	0.040
ENSG00000174514	MFSD4A	0.228	6.650	2.334	0.022
ENSG00000174521	TTC9B	0.325	3.105	2.408	0.018
ENSG00000174547	MRPL11	0.251	2.327	2.347	0.021
ENSG00000174574	AKIRIN1	0.251	5.641	3.087	0.003
ENSG00000174579	MSL2	-0.115	5.732	-2.277	0.025
ENSG00000174611	KY	-0.194	2.561	-2.267	0.026
ENSG00000174695	TMEM167A	0.274	7.214	3.340	0.001
ENSG00000174744	BRMS1	0.310	2.438	2.429	0.017
ENSG00000174775	HRAS	0.483	3.811	3.958	0.000
ENSG00000174827	PDZK1	-0.469	1.555	-2.294	0.024
ENSG00000174891	RSRC1	0.211	5.818	2.446	0.017
ENSG00000174943	KCTD13	-0.215	4.411	-2.718	0.008
ENSG00000174944	P2RY14	-0.373	3.938	-3.322	0.001
ENSG00000174946	GPR171	-0.518	2.716	-3.675	0.000
ENSG00000175003	SLC22A1	-0.509	-0.720	-2.572	0.012
ENSG00000175066	GK5	-0.155	5.514	-2.732	0.008

ENSG00000175084	DES	0.825	-1.201	2.588	0.011
ENSG00000175104	TRAF6	-0.144	4.920	-2.806	0.006
ENSG00000175115	PACS1	-0.193	5.662	-2.653	0.010
ENSG00000175137	SH3BP5L	0.240	4.788	2.843	0.006
ENSG00000175166	PSMD2	0.234	6.611	2.188	0.032
ENSG00000175182	FAM131A	0.199	5.741	2.698	0.008
ENSG00000175197	DDIT3	0.365	2.409	2.971	0.004
ENSG00000175267	VWA3A	-0.417	3.640	-2.636	0.010
ENSG00000175311	ANKS4B	-0.447	0.469	-2.020	0.047
ENSG00000175324	LSM1	0.464	2.892	3.067	0.003
ENSG00000175348	TMEM9B	0.199	5.024	2.444	0.017
ENSG00000175352	NRIP3	0.367	6.243	3.142	0.002
ENSG00000175376	EIF1AD	0.145	3.962	2.187	0.032
ENSG00000175416	CLTB	0.276	5.422	2.550	0.013
ENSG00000175550	DRAP1	0.275	4.956	2.481	0.015
ENSG00000175567	UCP2	0.444	0.136	2.150	0.034
ENSG00000175581	MRPL48	0.346	3.486	3.060	0.003
ENSG00000175582	RAB6A	0.252	8.391	2.405	0.018
ENSG00000175611	LINC00476	-0.246	3.098	-2.555	0.012
ENSG00000175701	LINC00116	0.418	1.215	2.465	0.016
ENSG00000175727	MLXIP	-0.219	4.621	-2.222	0.029
ENSG00000175746	C15orf54	-0.711	-0.768	-3.012	0.003
ENSG00000175756	AURKAIP1	0.222	4.301	2.329	0.022
ENSG00000175792	RUVBL1	0.321	3.952	3.061	0.003
ENSG00000175820	CCDC168	-0.486	3.043	-2.612	0.011
ENSG00000175906	ARL4D	0.394	2.188	2.524	0.014
ENSG00000175920	DOK7	-0.630	-0.362	-2.478	0.015
ENSG00000175984	DENND2C	0.320	1.819	2.583	0.012
ENSG00000175985	PLEKHD1	0.261	1.942	2.103	0.039
ENSG00000176014	TUBB6	0.320	1.940	2.271	0.026
ENSG00000176018	LYSMD3	0.133	4.809	2.106	0.038
ENSG00000176024	ZNF613	0.180	2.840	2.402	0.019
ENSG00000176029	C11orf16	-0.778	-1.832	-3.015	0.003
ENSG00000176101	SSNA1	0.248	2.299	2.430	0.017
ENSG00000176102	CSTF3	0.179	4.729	2.461	0.016
ENSG00000176108	CHMP6	0.332	1.378	2.387	0.019
ENSG00000176124	DLEU1	-0.285	2.483	-2.220	0.029
ENSG00000176142	TMEM39A	-0.222	3.393	-2.278	0.025
ENSG00000176171	BNIP3	0.374	6.663	3.005	0.004
ENSG00000176208	ATAD5	-0.189	3.602	-2.144	0.035
ENSG00000176209	SMIM19	0.203	3.918	2.045	0.044
ENSG00000176268	CYCSP34	-0.423	0.132	-2.672	0.009
ENSG00000176390	CRLF3	-0.160	5.072	-2.029	0.046
ENSG00000176396	EID2	0.266	3.691	2.516	0.014
ENSG00000176401	EID2B	0.270	3.205	2.317	0.023
ENSG00000176407	KCMF1	0.147	6.437	2.096	0.039
ENSG00000176422	SPRYD4	-0.255	3.319	-2.567	0.012

ENSG00000176435	CLEC14A	0.434	1.859	3.197	0.002
ENSG00000176510	OR10AC1	0.737	-1.915	2.224	0.029
ENSG00000176542	USF3	-0.122	6.581	-2.346	0.021
ENSG00000176597	B3GNT5	-0.294	3.820	-2.375	0.020
ENSG00000176624	MEX3C	0.133	5.181	2.025	0.046
ENSG00000176641	RNF152	0.478	5.289	4.374	0.000
ENSG00000176681	LRRC37A	-0.884	3.395	-2.477	0.015
ENSG00000176783	RUFY1	0.158	4.996	2.251	0.027
ENSG00000176853	FAM91A1	0.283	5.685	3.186	0.002
ENSG00000176857	GJA1P1	-0.516	2.135	-2.891	0.005
ENSG00000176871	WSB2	0.276	7.450	2.870	0.005
ENSG00000176887	SOX11	0.206	3.101	2.495	0.015
ENSG00000177030	DEAF1	0.204	5.291	2.012	0.048
ENSG00000177150	FAM210A	0.208	4.166	3.069	0.003
ENSG00000177156	TALDO1	0.311	4.746	2.847	0.006
ENSG00000177181	RIMKLA	0.304	6.467	3.022	0.003
ENSG00000177225	PDDC1	0.139	4.852	1.997	0.049
ENSG00000177275	OR2AJ1	0.632	-1.528	2.069	0.042
ENSG00000177350	RPL13AP3	-0.467	-1.486	-2.126	0.037
ENSG00000177383	MAGEF1	0.193	5.313	2.095	0.039
ENSG00000177398	UMODL1	-0.567	-1.431	-2.426	0.017
ENSG00000177463	NR2C2	-0.082	7.138	-2.046	0.044
ENSG00000177469	PTRF	0.332	3.773	2.225	0.029
ENSG00000177479	ARIH2	0.150	6.072	2.234	0.028
ENSG00000177548	RABEP2	0.261	1.749	2.276	0.025
ENSG00000177663	IL17RA	0.237	3.659	2.174	0.033
ENSG00000177666	PNPLA2	-0.310	1.864	-2.269	0.026
ENSG00000177675	CD163L1	0.627	2.883	3.747	0.000
ENSG00000177683	THAP5	0.159	5.871	2.257	0.027
ENSG00000177688	SUMO4	-0.293	1.136	-2.536	0.013
ENSG00000177692	DNAJC28	0.269	3.593	2.646	0.010
ENSG00000177707	NECTIN3	0.300	6.200	2.409	0.018
ENSG00000177728	TMEM94	-0.252	5.322	-2.423	0.018
ENSG00000177731	FLII	0.164	5.238	2.136	0.036
ENSG00000177830	CHID1	0.303	3.242	2.561	0.012
ENSG00000177868	SVBP	0.194	2.901	2.205	0.030
ENSG00000177879	AP3S1	0.328	5.721	3.068	0.003
ENSG00000177885	GRB2	0.158	6.382	2.030	0.046
ENSG00000177889	UBE2N	0.197	6.783	2.086	0.040
ENSG00000177932	ZNF354C	0.255	4.635	3.025	0.003
ENSG00000177954	RPS27	0.958	3.858	2.105	0.038
ENSG00000177963	RIC8A	0.190	5.089	2.740	0.008
ENSG00000177971	IMP3	0.496	2.736	3.087	0.003
ENSG00000177993	ZNRF3-AS1	-0.648	0.336	-2.799	0.006
ENSG00000178015	GPR150	-0.469	-0.189	-2.408	0.018
ENSG00000178021	TSPYL6	-0.552	1.528	-3.557	0.001
ENSG00000178035	IMPDH2	0.264	4.568	2.941	0.004

ENSG00000178053	MLF1	0.275	4.155	2.712	0.008
ENSG00000178074	C2orf69	0.202	5.726	2.174	0.033
ENSG00000178105	DDX10	0.205	5.633	2.466	0.016
ENSG00000178127	NDUFV2	-0.259	2.412	-2.110	0.038
ENSG00000178163	ZNF518B	0.160	6.298	2.371	0.020
ENSG00000178172	SPINK6	-0.612	-1.343	-2.244	0.028
ENSG00000178175	ZNF366	0.462	1.052	2.335	0.022
ENSG00000178234	GALNT11	0.204	6.574	2.331	0.022
ENSG00000178307	TMEM11	0.188	3.170	2.151	0.034
ENSG00000178358	OR2D3	-0.421	0.240	-2.039	0.045
ENSG00000178381	ZFAND2A	0.265	3.830	2.042	0.044
ENSG00000178397	FAM220A	0.215	4.511	2.198	0.031
ENSG00000178401	DNAJC22	0.505	1.073	2.420	0.018
ENSG00000178440	LINC00843	-0.436	0.529	-2.391	0.019
ENSG00000178449	COX14	0.342	3.125	2.834	0.006
ENSG00000178460	MCMDC2	-0.222	2.121	-2.071	0.042
ENSG00000178585	CTNNBIP1	0.260	4.450	3.648	0.000
ENSG00000178607	ERN1	-0.175	2.710	-2.458	0.016
ENSG00000178636	RP11-455G16.1	-0.810	-1.828	-2.841	0.006
ENSG00000178719	GRINA	-0.322	5.486	-3.083	0.003
ENSG00000178761	FAM219B	0.119	4.435	2.447	0.017
ENSG00000178796	RIIAD1	0.258	1.673	2.459	0.016
ENSG00000178802	MPI	0.148	4.710	2.057	0.043
ENSG00000178913	TAF7	0.166	6.579	1.993	0.050
ENSG00000178952	TUFM	0.240	5.629	2.651	0.010
ENSG00000178971	CTC1	-0.224	4.435	-3.144	0.002
ENSG00000178980	SEPW1	0.305	5.852	2.512	0.014
ENSG00000178982	EIF3K	0.342	5.084	3.090	0.003
ENSG00000178988	MRFAP1L1	0.216	6.880	2.335	0.022
ENSG00000178997	EXD1	0.859	-1.370	2.886	0.005
ENSG00000179010	MRFAP1	0.205	7.602	2.408	0.018
ENSG00000179038	RP11-157K17.5	-0.471	0.684	-2.539	0.013
ENSG00000179085	DPM3	0.320	0.962	2.164	0.033
ENSG00000179094	PER1	-0.314	4.342	-2.290	0.025
ENSG00000179134	SAMD4B	-0.249	5.486	-2.347	0.021
ENSG00000179141	MTUS2-AS1	-0.656	0.823	-3.440	0.001
ENSG00000179148	ALOXE3	-0.617	-0.577	-2.709	0.008
ENSG00000179218	CALR	0.282	6.968	3.208	0.002
ENSG00000179270	C2orf71	-0.437	-0.190	-2.366	0.020
ENSG00000179295	PTPN11	0.180	8.743	2.061	0.042
ENSG00000179299	NSUN7	-0.536	0.015	-3.469	0.001
ENSG00000179314	WSCD1	0.303	4.662	3.125	0.002
ENSG00000179331	RAB39A	0.436	2.924	2.713	0.008
ENSG00000179361	ARID3B	-0.310	1.444	-2.379	0.020
ENSG00000179387	ELMOD2	0.168	4.909	2.337	0.022
ENSG00000179406	LINC00174	-0.273	2.963	-2.221	0.029
ENSG00000179409	GEMIN4	-0.156	2.815	-2.032	0.045

ENSG00000179447	RP5-1027G4.3	-0.542	-1.370	-2.103	0.039
ENSG00000179454	KLHL28	0.158	6.068	2.120	0.037
ENSG00000179456	ZBTB18	0.217	8.017	2.469	0.016
ENSG00000179520	SLC17A8	0.509	1.673	2.271	0.026
ENSG00000179532	DNHD1	-0.322	4.709	-2.950	0.004
ENSG00000179562	GCC1	0.159	3.970	2.087	0.040
ENSG00000179818	PCBP1-AS1	-0.257	4.978	-2.766	0.007
ENSG00000179833	SERTAD2	-0.224	4.040	-2.736	0.008
ENSG00000179889	PDXDC1	0.148	6.312	2.153	0.034
ENSG00000179933	C14orf119	0.379	3.364	3.363	0.001
ENSG00000179935	LINC00652	0.403	1.271	2.864	0.005
ENSG00000179941	BBS10	0.209	5.161	2.645	0.010
ENSG00000179950	PUF60	0.200	5.095	2.222	0.029
ENSG00000179958	DCTPP1	0.316	1.872	2.377	0.020
ENSG00000180116	C12orf40	-0.382	1.220	-2.525	0.013
ENSG00000180139	ACTA2-AS1	-0.385	2.637	-3.846	0.000
ENSG00000180189	HMGB1P14	-0.600	-0.142	-2.646	0.010
ENSG00000180219	FAM71C	-0.399	0.696	-2.187	0.032
ENSG00000180245	RRH	-0.439	0.453	-2.686	0.009
ENSG00000180329	CCDC43	0.299	4.809	2.954	0.004
ENSG00000180385	EMC3-AS1	-0.261	1.747	-2.303	0.024
ENSG00000180423	HARBI1	0.226	1.668	2.059	0.043
ENSG00000180425	C11orf71	0.211	3.459	2.511	0.014
ENSG00000180458	CTD-3064H18.4	0.473	-0.167	2.510	0.014
ENSG00000180479	ZNF571	-0.140	3.852	-2.500	0.014
ENSG00000180525	PRR26	-0.442	3.848	-2.927	0.004
ENSG00000180537	RNF182	-0.321	4.557	-2.117	0.037
ENSG00000180596	HIST1H2BC	0.239	4.156	2.054	0.043
ENSG00000180616	SSTR2	0.260	5.804	2.035	0.045
ENSG00000180660	MAB21L1	-0.435	2.040	-2.816	0.006
ENSG00000180667	YOD1	0.173	5.413	2.101	0.039
ENSG00000180694	TMEM64	0.272	4.288	2.769	0.007
ENSG00000180776	ZDHHC20	0.288	6.362	2.606	0.011
ENSG00000180801	ARSJ	-0.298	3.010	-2.702	0.008
ENSG00000180803	RP11-467D10.2	-0.769	-1.730	-3.006	0.004
ENSG00000180817	PPA1	0.582	5.600	2.676	0.009
ENSG00000180822	PSMG4	-0.177	3.937	-2.458	0.016
ENSG00000180828	BHLHE22	0.365	2.791	2.497	0.015
ENSG00000180875	GREM2	0.306	3.692	2.716	0.008
ENSG00000180992	MRPL14	0.308	1.907	2.452	0.016
ENSG00000181004	BBS12	0.266	3.428	2.415	0.018
ENSG00000181026	AEN	0.265	2.282	2.519	0.014
ENSG00000181085	MAPK15	1.072	-0.993	3.638	0.000
ENSG00000181163	NPM1	0.383	6.544	2.150	0.035
ENSG00000181195	PENK	0.622	1.361	2.351	0.021
ENSG00000181211	HECW1-IT1	-0.592	2.556	-3.368	0.001
ENSG00000181274	FRAT2	0.473	2.519	3.847	0.000

ENSG00000181291	TMEM132E	-0.314	2.359	-2.180	0.032
ENSG00000181458	TMEM45A	0.446	0.147	2.202	0.030
ENSG00000181467	RAP2B	0.180	5.686	2.169	0.033
ENSG00000181523	SGSH	-0.332	1.914	-2.566	0.012
ENSG00000181610	MRPS23	0.316	6.090	3.081	0.003
ENSG00000181619	GPR135	-0.273	3.727	-2.793	0.006
ENSG00000181634	TNFSF15	0.595	-0.630	2.224	0.029
ENSG00000181751	C5orf30	0.206	5.330	2.648	0.010
ENSG00000181800	CELF2-AS1	-0.337	4.144	-2.019	0.047
ENSG00000181827	RFX7	-0.255	5.847	-3.804	0.000
ENSG00000181847	TIGIT	-0.759	-2.157	-2.297	0.024
ENSG00000181894	ZNF329	0.181	4.569	2.532	0.013
ENSG00000181896	ZNF101	0.203	3.331	3.177	0.002
ENSG00000181915	ADO	0.185	5.669	2.070	0.042
ENSG00000181924	COA4	0.204	2.575	2.249	0.027
ENSG00000181929	PRKAG1	0.187	4.879	2.951	0.004
ENSG00000181991	MRPS11	0.183	4.256	2.585	0.012
ENSG00000182004	SNRPE	0.482	3.268	2.844	0.006
ENSG00000182108	DEXI	0.384	1.797	3.260	0.002
ENSG00000182117	NOP10	0.348	3.049	2.963	0.004
ENSG00000182118	FAM89A	0.514	1.482	2.859	0.005
ENSG00000182132	KCNIP1	0.262	3.846	2.991	0.004
ENSG00000182180	MRPS16	0.160	5.088	2.379	0.020
ENSG00000182199	SHMT2	0.300	3.013	3.098	0.003
ENSG00000182208	MOB2	0.238	3.256	2.297	0.024
ENSG00000182230	FAM153B	-0.657	2.396	-2.527	0.013
ENSG00000182263	FIGN	-0.245	4.975	-3.315	0.001
ENSG00000182272	B4GALNT4	0.202	4.559	1.991	0.050
ENSG00000182307	C8orf33	0.256	4.319	3.578	0.001
ENSG00000182347	PDSS1P1	-0.585	0.796	-3.290	0.001
ENSG00000182400	TRAPPC6B	0.285	5.212	3.273	0.002
ENSG00000182481	KPNA2	0.345	4.915	3.520	0.001
ENSG00000182511	FES	0.301	1.099	2.189	0.031
ENSG00000182512	GLRX5	0.231	4.237	2.056	0.043
ENSG00000182566	CLEC4G	0.737	-0.479	2.738	0.008
ENSG00000182621	PLCB1	0.233	9.011	2.294	0.024
ENSG00000182628	SKA2	0.162	5.491	2.272	0.026
ENSG00000182670	TTC3	0.238	9.452	2.306	0.024
ENSG00000182718	ANXA2	0.479	1.853	2.093	0.039
ENSG00000182722	SEPHS1P1	-0.488	0.990	-2.941	0.004
ENSG00000182752	PAPPA	-0.254	2.602	-2.144	0.035
ENSG00000182827	ACBD3	0.220	5.808	3.129	0.002
ENSG00000182831	C16orf72	0.128	7.184	2.390	0.019
ENSG00000182858	ALG12	-0.195	2.518	-2.303	0.024
ENSG00000182899	RPL35A	0.353	5.706	2.454	0.016
ENSG00000182973	CNOT10	0.130	4.280	2.316	0.023
ENSG00000182983	ZNF662	-0.263	3.764	-3.369	0.001

ENSG00000183010	PYCR1	0.320	0.781	2.184	0.032
ENSG00000183011	NAA38	0.333	2.856	2.254	0.027
ENSG00000183036	PCP4	0.491	3.882	2.702	0.008
ENSG00000183066	WBP2NL	-0.569	2.225	-3.728	0.000
ENSG00000183077	AFMID	0.227	3.498	2.027	0.046
ENSG00000183090	FREM3	0.591	5.413	2.530	0.013
ENSG00000183117	CSMD1	-0.209	7.515	-2.622	0.010
ENSG00000183207	RUVBL2	0.335	4.427	2.371	0.020
ENSG00000183258	DDX41	0.191	5.178	2.122	0.037
ENSG00000183423	LRIT3	-0.537	0.570	-2.838	0.006
ENSG00000183431	SF3A3	0.340	5.477	2.725	0.008
ENSG00000183474	GTF2H2C	-0.575	3.003	-2.115	0.037
ENSG00000183475	ASB7	0.129	4.149	2.111	0.038
ENSG00000183495	EP400	-0.231	6.198	-2.912	0.005
ENSG00000183513	COA5	0.171	4.901	2.140	0.035
ENSG00000183520	UTP11	0.227	4.559	2.830	0.006
ENSG00000183562	CTC-343N3.1	-0.289	2.373	-2.047	0.044
ENSG00000183576	SETD3	0.200	6.923	2.487	0.015
ENSG00000183580	FBXL7	-0.305	3.294	-2.236	0.028
ENSG00000183597	TANGO2	0.269	3.712	2.762	0.007
ENSG00000183604	SMG1P5	-1.089	4.362	-3.100	0.003
ENSG00000183621	ZNF438	-0.162	3.675	-3.040	0.003
ENSG00000183647	ZNF530	-0.210	1.075	-2.059	0.043
ENSG00000183648	NDUFB1	0.534	4.635	3.128	0.002
ENSG00000183662	FAM19A1	0.333	5.545	2.489	0.015
ENSG00000183665	TRMT12	0.161	4.637	2.219	0.029
ENSG00000183684	ALYREF	0.288	3.371	2.484	0.015
ENSG00000183696	UPP1	-0.181	4.338	-2.050	0.044
ENSG00000183726	TMEM50A	0.264	5.528	3.264	0.002
ENSG00000183735	TBK1	0.251	5.874	2.370	0.020
ENSG00000183742	MACC1	-0.387	-0.448	-2.184	0.032
ENSG00000183778	B3GALT5	-0.369	2.560	-3.251	0.002
ENSG00000183828	NUDT14	0.320	2.457	2.023	0.046
ENSG00000183833	MAATS1	-0.435	2.646	-4.418	0.000
ENSG00000183856	IQGAP3	-0.748	0.884	-3.031	0.003
ENSG00000183955	KMT5A	0.239	4.117	3.366	0.001
ENSG00000183978	COA3	0.262	3.535	2.326	0.022
ENSG00000184005	ST6GALNAC3	0.311	4.143	2.737	0.008
ENSG00000184076	UQCR10	0.254	4.279	2.186	0.032
ENSG00000184117	NIPSNAP1	0.233	5.195	2.745	0.007
ENSG00000184160	ADRA2C	0.466	0.890	2.899	0.005
ENSG00000184164	CRELD2	0.283	3.140	3.000	0.004
ENSG00000184178	SCFD2	0.158	4.429	2.390	0.019
ENSG00000184182	UBE2F	0.215	3.956	2.390	0.019
ENSG00000184185	KCNJ12	0.291	3.053	2.584	0.012
ENSG00000184203	PPP1R2	0.287	6.025	2.264	0.026
ENSG00000184207	PGP	0.184	3.614	2.864	0.005

ENSG00000184209	SNRNP35	0.179	2.853	2.097	0.039
ENSG00000184220	CMSS1	0.221	4.846	2.385	0.019
ENSG00000184313	MROH7	-0.413	1.734	-2.119	0.037
ENSG00000184349	EFNA5	-0.164	5.510	-2.325	0.023
ENSG00000184384	MAML2	-0.202	4.527	-2.160	0.034
ENSG00000184432	COPB2	0.182	7.030	2.203	0.030
ENSG00000184465	WDR27	-0.210	4.820	-2.224	0.029
ENSG00000184500	PROS1	0.406	3.531	3.565	0.001
ENSG00000184517	ZFP1	0.218	5.420	2.580	0.012
ENSG00000184575	XPOT	0.330	6.883	3.192	0.002
ENSG00000184584	TMEM173	0.360	0.605	2.221	0.029
ENSG00000184588	PDE4B	0.177	6.952	2.518	0.014
ENSG00000184608	FAM167A-AS1	-0.652	-0.807	-3.084	0.003
ENSG00000184611	KCNH7	0.227	6.280	2.323	0.023
ENSG00000184612	RPL7P26	-0.599	-0.035	-2.862	0.005
ENSG00000184617	ZNF840P	-0.450	-0.641	-2.210	0.030
ENSG00000184752	NDUFA12	0.457	4.947	2.833	0.006
ENSG00000184787	UBE2G2	-0.070	5.876	-2.025	0.046
ENSG00000184840	TMED9	0.229	4.561	2.377	0.020
ENSG00000184857	TMEM186	0.276	1.300	2.243	0.028
ENSG00000184900	SUMO3	0.337	5.484	2.793	0.007
ENSG00000184933	OR6A2	-0.329	1.539	-2.062	0.042
ENSG00000184949	FAM227A	-0.345	3.334	-2.887	0.005
ENSG00000184983	NDUFA6	0.343	5.097	3.163	0.002
ENSG00000184984	CHRM5	-0.702	1.316	-2.737	0.008
ENSG00000185031	SLC2A3P2	-0.665	2.296	-3.229	0.002
ENSG00000185033	SEMA4B	-0.269	3.436	-2.076	0.041
ENSG00000185052	SLC24A3	0.208	3.726	2.001	0.049
ENSG00000185088	RPS27L	0.214	4.726	2.350	0.021
ENSG00000185127	C6orf120	0.266	4.891	2.763	0.007
ENSG00000185236	RAB11B	-0.269	4.053	-2.582	0.012
ENSG00000185245	GP1BA	-0.320	0.608	-2.084	0.040
ENSG00000185298	CCDC137	0.261	2.908	3.055	0.003
ENSG00000185305	ARL15	0.309	5.442	2.619	0.011
ENSG00000185306	C12orf56	-0.408	0.908	-2.283	0.025
ENSG00000185414	MRPL30	0.206	4.684	2.104	0.038
ENSG00000185475	TMEM179B	0.161	2.098	2.196	0.031
ENSG00000185495	RP11-504P24.3	-0.483	3.782	-3.684	0.000
ENSG00000185624	P4HB	0.204	5.342	2.363	0.020
ENSG00000185627	PSMD13	0.376	4.721	3.146	0.002
ENSG00000185666	SYN3	-0.458	5.065	-3.964	0.000
ENSG00000185674	LYG2	-0.506	0.398	-2.877	0.005
ENSG00000185721	DRG1	0.296	4.980	2.523	0.014
ENSG00000185722	ANKFY1	-0.152	6.054	-3.054	0.003
ENSG00000185787	MORF4L1	0.390	6.770	2.072	0.041
ENSG00000185823	NPAP1	-0.644	1.235	-2.908	0.005
ENSG00000185860	CCDC190	-0.780	0.455	-2.614	0.011

ENSG00000185909	KLHDC8B	0.309	2.673	2.634	0.010
ENSG00000185920	PTCH1	-0.428	4.848	-3.775	0.000
ENSG00000185933	CALHM1	0.355	1.522	2.576	0.012
ENSG00000185947	ZNF267	0.228	4.205	2.341	0.022
ENSG00000185950	IRS2	-0.161	6.358	-2.055	0.043
ENSG00000185958	FAM186A	-0.392	0.118	-2.038	0.045
ENSG00000185963	BICD2	0.157	5.935	2.953	0.004
ENSG00000186010	NDUFA13	0.384	2.400	2.567	0.012
ENSG00000186038	HTR3E	-0.902	-1.626	-2.948	0.004
ENSG00000186130	ZBTB6	0.188	4.534	2.248	0.027
ENSG00000186141	POLR3C	0.195	4.383	2.370	0.020
ENSG00000186280	KDM4D	-0.308	0.722	-2.227	0.029
ENSG00000186281	GPAT2	-0.297	0.895	-2.039	0.045
ENSG00000186297	GABRA5	0.327	6.338	2.718	0.008
ENSG00000186340	THBS2	0.397	4.549	2.234	0.028
ENSG00000186369	LINC00643	0.371	5.364	2.532	0.013
ENSG00000186432	KPNA4	0.180	6.867	2.321	0.023
ENSG00000186453	FAM228A	-0.267	0.685	-2.158	0.034
ENSG00000186566	GPATCH8	-0.218	6.715	-3.133	0.002
ENSG00000186567	CEACAM19	-0.350	0.896	-2.004	0.048
ENSG00000186594	MIR22HG	0.297	3.240	2.614	0.011
ENSG00000186638	KIF24	-0.213	2.290	-2.010	0.048
ENSG00000186665	C17orf58	0.293	2.730	3.142	0.002
ENSG00000186666	BCDIN3D	0.184	1.746	2.099	0.039
ENSG00000186676	EEF1GP1	-0.522	-0.421	-2.261	0.026
ENSG00000186687	LYRM7	0.164	6.537	2.022	0.046
ENSG00000186732	MPPED1	0.193	6.274	2.462	0.016
ENSG00000186792	HYAL3	0.321	1.190	2.021	0.047
ENSG00000186854	TRABD2A	0.336	3.695	2.609	0.011
ENSG00000186867	QRFPR	0.460	0.473	2.047	0.044
ENSG00000186889	TMEM17	0.246	2.009	2.085	0.040
ENSG00000186951	PPARA	-0.299	4.972	-2.254	0.027
ENSG00000187051	RPS19BP1	0.369	4.030	2.892	0.005
ENSG00000187079	TEAD1	-0.314	5.743	-3.096	0.003
ENSG00000187105	HEATR4	-0.188	2.592	-2.049	0.044
ENSG00000187134	AKR1C1	0.316	2.811	3.072	0.003
ENSG00000187229	RP11-161I2.1	-0.373	1.287	-2.110	0.038
ENSG00000187258	NPSR1	-0.915	-1.947	-2.854	0.005
ENSG00000187260	WDR86	0.452	0.904	2.188	0.031
ENSG00000187372	PCDHB13	-0.234	4.099	-2.512	0.014
ENSG00000187391	MAGI2	-0.120	8.078	-2.668	0.009
ENSG00000187398	LUZP2	0.374	5.425	3.612	0.001
ENSG00000187416	LHFPL3	0.396	3.708	3.401	0.001
ENSG00000187446	CHP1	0.251	6.610	3.138	0.002
ENSG00000187474	FPR3	-0.613	-0.328	-2.373	0.020
ENSG00000187514	PTMA	0.256	7.479	2.314	0.023
ENSG00000187522	HSPA14	0.194	3.694	2.223	0.029

ENSG00000187605	TET3	-0.248	5.660	-3.508	0.001
ENSG00000187672	ERC2	0.171	8.327	2.251	0.027
ENSG00000187699	C2orf88	-0.250	4.124	-2.093	0.039
ENSG00000187713	TMEM203	0.212	3.443	2.340	0.022
ENSG00000187815	ZFP69	0.218	2.659	2.393	0.019
ENSG00000187902	SHISA7	-0.379	4.610	-2.572	0.012
ENSG00000187994	RINL	0.251	1.587	2.025	0.046
ENSG00000188010	MORN2	0.405	3.036	2.775	0.007
ENSG00000188026	RILPL1	0.145	4.048	2.081	0.041
ENSG00000188039	NWD1	-0.443	4.173	-2.095	0.039
ENSG00000188070	C11orf95	0.235	4.771	2.664	0.009
ENSG00000188092	GPR89B	-0.483	3.421	-2.108	0.038
ENSG00000188133	TMEM215	0.810	0.397	2.837	0.006
ENSG00000188171	ZNF626	0.145	5.031	2.008	0.048
ENSG00000188186	LAMTOR4	0.491	3.608	2.909	0.005
ENSG00000188234	AGAP4	-0.469	2.477	-2.099	0.039
ENSG00000188243	COMMD6	0.372	3.472	2.635	0.010
ENSG00000188266	HYKK	-0.180	2.455	-2.220	0.029
ENSG00000188290	HES4	0.508	-0.043	2.348	0.021
ENSG00000188315	C3orf62	-0.236	3.080	-3.282	0.002
ENSG00000188316	ENO4	-0.300	1.477	-2.189	0.031
ENSG00000188342	GTF2F2	0.230	4.179	2.222	0.029
ENSG00000188379	IFNA2	-1.025	-2.523	-2.248	0.027
ENSG00000188517	COL25A1	0.282	3.796	2.023	0.046
ENSG00000188529	SRSF10	0.127	6.295	2.317	0.023
ENSG00000188554	NBR1	0.157	7.195	2.277	0.025
ENSG00000188580	NKAIN2	0.178	5.854	2.248	0.027
ENSG00000188596	CFAP54	-0.230	3.566	-2.248	0.027
ENSG00000188612	SUMO2	0.591	5.389	3.251	0.002
ENSG00000188613	NANOS1	-0.261	1.442	-2.178	0.032
ENSG00000188624	IGFL3	-0.660	-0.960	-2.454	0.016
ENSG00000188636	LDOC1L	0.159	6.211	2.375	0.020
ENSG00000188649	CC2D2B	-0.255	3.086	-2.085	0.040
ENSG00000188674	C2orf80	0.378	5.065	2.445	0.017
ENSG00000188687	SLC4A5	-0.445	-0.147	-2.216	0.029
ENSG00000188690	UROS	0.284	5.453	2.862	0.005
ENSG00000188693	CYP51A1-AS1	-0.325	-0.390	-2.032	0.045
ENSG00000188725	SMIM15	0.201	5.113	2.729	0.008
ENSG00000188729	OSTN	0.740	2.009	2.448	0.017
ENSG00000188761	BCL2L15	-0.423	1.024	-2.818	0.006
ENSG00000188771	PLET1	-0.550	-2.029	-2.001	0.049
ENSG00000188783	PRELP	-0.460	3.109	-2.822	0.006
ENSG00000188786	MTF1	-0.269	4.225	-3.731	0.000
ENSG00000188807	TMEM201	-0.226	2.560	-2.181	0.032
ENSG00000188846	RPL14	0.233	6.913	2.511	0.014
ENSG00000188859	FAM78B	-0.251	3.741	-2.520	0.014
ENSG00000188895	MSL1	0.142	6.773	2.608	0.011

ENSG00000188921	HACD4	-0.382	5.178	-3.263	0.002
ENSG00000188976	NOC2L	0.340	4.508	3.143	0.002
ENSG00000188991	SLC15A5	-0.968	-2.286	-2.483	0.015
ENSG00000189043	NDUFA4	0.471	6.105	2.664	0.009
ENSG00000189045	ANKDD1B	-0.442	0.781	-2.826	0.006
ENSG00000189099	PRSS48	-0.385	0.467	-2.247	0.027
ENSG00000189114	BLOC1S3	-0.237	1.755	-2.014	0.047
ENSG00000189129	PLAC9	0.375	0.900	2.299	0.024
ENSG00000189144	ZNF573	0.164	4.453	2.702	0.008
ENSG00000189159	HN1	0.353	4.449	3.360	0.001
ENSG00000189184	PCDH18	0.267	3.583	2.573	0.012
ENSG00000189195	BTBD8	0.241	8.959	2.090	0.040
ENSG00000189212	DPY19L2P1	-0.280	5.498	-2.234	0.028
ENSG00000189238	LINC00943	0.638	0.894	2.801	0.006
ENSG00000189266	PNRC2	0.213	5.793	2.959	0.004
ENSG00000189306	RRP7A	0.273	4.389	2.485	0.015
ENSG00000189367	KIAA0408	-0.287	1.789	-2.109	0.038
ENSG00000189403	HMGB1	0.219	7.034	2.769	0.007
ENSG00000189423	USP32P3	-0.352	3.163	-2.303	0.024
ENSG00000196072	BLOC1S2	0.360	5.463	3.109	0.003
ENSG00000196104	SPOCK3	0.366	7.079	2.250	0.027
ENSG00000196109	ZNF676	-0.444	1.515	-2.590	0.011
ENSG00000196167	COLCA1	-0.346	1.998	-2.684	0.009
ENSG00000196208	GREB1	-0.235	4.950	-3.622	0.001
ENSG00000196220	SRGAP3	-0.135	7.630	-2.126	0.037
ENSG00000196230	TUBB	0.209	7.036	2.196	0.031
ENSG00000196233	LCOR	-0.136	7.145	-2.751	0.007
ENSG00000196262	PPIA	0.313	6.782	2.738	0.008
ENSG00000196268	ZNF493	-0.184	6.227	-2.159	0.034
ENSG00000196295	AC005154.6	-0.307	6.344	-3.353	0.001
ENSG00000196305	IARS	0.308	7.120	2.787	0.007
ENSG00000196313	POM121	-0.302	4.882	-2.456	0.016
ENSG00000196350	ZNF729	-0.815	-1.812	-2.193	0.031
ENSG00000196365	LONP1	0.216	5.079	2.156	0.034
ENSG00000196367	TRRAP	-0.162	6.545	-2.765	0.007
ENSG00000196418	ZNF124	0.338	4.847	2.114	0.038
ENSG00000196419	XRCC6	0.366	6.932	2.676	0.009
ENSG00000196428	TSC22D2	-0.155	6.776	-2.023	0.046
ENSG00000196449	YRDC	0.172	3.101	2.384	0.019
ENSG00000196460	RFX8	-0.307	0.880	-2.335	0.022
ENSG00000196510	ANAPC7	0.150	5.223	2.163	0.033
ENSG00000196531	NACA	0.362	7.043	2.755	0.007
ENSG00000196553	LINC00238	-0.411	0.776	-2.629	0.010
ENSG00000196562	SULF2	0.199	5.235	2.318	0.023
ENSG00000196597	ZNF782	-0.171	5.087	-2.736	0.008
ENSG00000196600	SLC22A25	-0.427	2.374	-2.958	0.004
ENSG00000196636	SDHAF3	0.230	2.350	2.048	0.044

ENSG00000196655	TRAPPC4	0.226	3.632	2.297	0.024
ENSG00000196668	LINC00173	-0.440	0.430	-2.265	0.026
ENSG00000196683	TOMM7	0.365	5.066	2.454	0.016
ENSG00000196689	TRPV1	-0.220	2.216	-2.077	0.041
ENSG00000196696	PDXDC2P	-0.328	1.649	-2.200	0.031
ENSG00000196782	MAML3	-0.234	3.237	-2.607	0.011
ENSG00000196792	STRN3	0.135	6.469	2.125	0.037
ENSG00000196810	CTBP1-AS2	-0.200	3.532	-2.750	0.007
ENSG00000196844	PATE2	-0.312	0.845	-2.407	0.018
ENSG00000196865	NHLRC2	-0.179	6.296	-3.135	0.002
ENSG00000196935	SRGAP1	-0.283	4.580	-3.319	0.001
ENSG00000196943	NOP9	0.124	4.139	1.991	0.050
ENSG00000196950	SLC39A10	0.343	8.690	2.561	0.012
ENSG00000196951	SCOC-AS1	0.325	0.397	2.577	0.012
ENSG00000196968	FUT11	0.371	4.186	5.216	0.000
ENSG00000197006	METTL9	0.260	6.479	2.951	0.004
ENSG00000197013	ZNF429	0.153	4.574	2.151	0.034
ENSG00000197024	ZNF398	-0.156	5.369	-3.363	0.001
ENSG00000197037	ZSCAN25	-0.110	4.103	-2.265	0.026
ENSG00000197043	ANXA6	0.398	7.326	2.641	0.010
ENSG00000197045	GMFB	0.292	7.853	2.541	0.013
ENSG00000197057	DTHD1	-0.485	-1.006	-2.040	0.045
ENSG00000197077	KIAA1671	-0.203	6.597	-2.580	0.012
ENSG00000197085	NPSR1-AS1	-0.357	0.668	-2.416	0.018
ENSG00000197093	GAL3ST4	0.435	1.260	2.118	0.037
ENSG00000197111	PCBP2	-0.165	5.043	-2.483	0.015
ENSG00000197134	ZNF257	-0.490	1.614	-2.586	0.011
ENSG00000197168	NEK5	-0.296	2.693	-2.825	0.006
ENSG00000197208	SLC22A4	-0.405	1.897	-4.843	0.000
ENSG00000197217	ENTPD4	0.202	6.935	2.527	0.013
ENSG00000197223	C1D	0.385	3.533	2.833	0.006
ENSG00000197261	C6orf141	0.721	0.424	2.322	0.023
ENSG00000197265	GTF2E2	0.194	3.679	2.419	0.018
ENSG00000197301	RP11-366L20.2	-0.691	-1.870	-2.239	0.028
ENSG00000197322	C17orf102	-0.425	2.018	-2.342	0.022
ENSG00000197329	PELI1	0.214	5.058	2.493	0.015
ENSG00000197345	MRPL21	0.573	3.508	3.247	0.002
ENSG00000197360	ZNF98	-0.403	2.108	-2.266	0.026
ENSG00000197362	ZNF786	0.160	3.832	2.183	0.032
ENSG00000197372	ZNF675	0.147	5.031	2.008	0.048
ENSG00000197375	SLC22A5	-0.299	3.869	-3.167	0.002
ENSG00000197381	ADARB1	-0.196	6.095	-2.375	0.020
ENSG00000197451	HNRNPAB	0.181	3.994	2.341	0.022
ENSG00000197461	PDGFA	0.178	4.666	2.976	0.004
ENSG00000197479	PCDHB11	-0.334	3.397	-3.264	0.002
ENSG00000197498	RPF2	0.291	4.631	2.969	0.004
ENSG00000197536	C5orf56	-0.347	3.120	-2.714	0.008

ENSG00000197555	SIPA1L1	-0.150	7.616	-2.579	0.012
ENSG00000197584	KCNMB2	0.190	2.993	2.121	0.037
ENSG00000197585	AC107218.3	-0.590	-0.584	-3.410	0.001
ENSG00000197586	ENTPD6	0.225	5.562	2.145	0.035
ENSG00000197614	MFAP5	-0.708	-1.935	-2.352	0.021
ENSG00000197619	ZNF615	0.163	4.795	2.432	0.017
ENSG00000197632	SERPINB2	1.133	-2.729	2.773	0.007
ENSG00000197658	SLC22A24	-0.623	1.397	-3.334	0.001
ENSG00000197705	KLHL14	0.481	1.663	2.244	0.028
ENSG00000197728	RPS26	1.005	2.122	3.215	0.002
ENSG00000197756	RPL37A	0.403	6.709	2.289	0.025
ENSG00000197780	TAF13	0.437	5.417	3.385	0.001
ENSG00000197816	CCDC180	-0.394	2.731	-2.701	0.008
ENSG00000197826	C4orf22	0.306	0.822	2.151	0.034
ENSG00000197837	HIST4H4	0.247	2.385	2.382	0.020
ENSG00000197860	SGTB	0.295	7.993	2.467	0.016
ENSG00000197882	OR7E13P	-0.484	-0.428	-2.659	0.009
ENSG00000197885	NKIRAS1	0.218	5.915	2.099	0.039
ENSG00000197956	S100A6	0.333	4.701	1.993	0.050
ENSG00000197958	RPL12	0.657	4.693	2.288	0.025
ENSG00000197961	ZNF121	0.126	5.882	2.548	0.013
ENSG00000197965	MPZL1	0.198	5.092	3.077	0.003
ENSG00000197978	GOLGA6L9	-0.416	1.756	-2.119	0.037
ENSG00000198010	DLGAP2	-0.292	6.607	-3.201	0.002
ENSG00000198042	MAK16	0.335	4.548	3.073	0.003
ENSG00000198113	TOR4A	0.801	-1.611	2.574	0.012
ENSG00000198160	MIER1	0.114	6.327	2.002	0.049
ENSG00000198162	MAN1A2	0.275	8.995	2.628	0.010
ENSG00000198171	DDRGK1	0.198	5.106	2.973	0.004
ENSG00000198189	HSD17B11	0.301	4.057	2.564	0.012
ENSG00000198198	SZT2	-0.183	5.591	-2.185	0.032
ENSG00000198242	RPL23A	0.297	4.147	2.351	0.021
ENSG00000198258	UBL5	0.672	3.479	2.958	0.004
ENSG00000198301	SDAD1	0.294	6.108	2.542	0.013
ENSG00000198315	ZKSCAN8	-0.234	5.843	-3.729	0.000
ENSG00000198331	HYLS1	0.201	3.452	1.996	0.049
ENSG00000198355	PIM3	0.256	2.718	3.051	0.003
ENSG00000198356	ASNA1	0.222	4.167	2.042	0.044
ENSG00000198369	SPRED2	-0.199	5.637	-2.680	0.009
ENSG00000198399	ITSN2	0.127	7.198	2.098	0.039
ENSG00000198431	TXNRD1	0.307	6.241	2.828	0.006
ENSG00000198440	ZNF583	0.163	4.725	2.513	0.014
ENSG00000198453	ZNF568	0.179	4.807	3.099	0.003
ENSG00000198467	TPM2	0.424	2.892	2.327	0.022
ENSG00000198492	YTHDF2	0.162	5.280	2.013	0.047
ENSG00000198498	TMA16	0.261	3.173	2.633	0.010
ENSG00000198513	ATL1	0.302	6.740	2.272	0.026

ENSG00000198535	C2CD4A	0.708	-2.212	2.354	0.021
ENSG00000198547	C20orf203	-0.670	-0.978	-2.782	0.007
ENSG00000198554	WDHD1	0.240	3.736	2.384	0.019
ENSG00000198556	ZNF789	-0.198	3.273	-2.040	0.045
ENSG00000198590	C3orf35	-0.216	1.754	-2.115	0.037
ENSG00000198612	COPS8	0.313	6.273	2.778	0.007
ENSG00000198633	ZNF534	-0.344	2.273	-2.442	0.017
ENSG00000198646	NCOA6	-0.174	6.000	-2.054	0.043
ENSG00000198648	STK39	0.266	7.192	2.879	0.005
ENSG00000198668	CALM1	0.268	12.064	2.845	0.006
ENSG00000198673	FAM19A2	0.331	6.420	2.751	0.007
ENSG00000198677	TTC37	0.215	7.545	2.241	0.028
ENSG00000198690	FAN1	0.149	5.477	2.534	0.013
ENSG00000198711	SSBP3-AS1	-0.449	1.357	-2.778	0.007
ENSG00000198722	UNC13B	0.144	6.808	2.465	0.016
ENSG00000198729	PPP1R14C	0.467	4.284	3.422	0.001
ENSG00000198730	CTR9	0.166	5.673	2.310	0.023
ENSG00000198734	F5	-0.326	3.245	-2.317	0.023
ENSG00000198746	GPATCH3	0.217	2.891	2.196	0.031
ENSG00000198755	RPL10A	0.394	5.240	2.011	0.048
ENSG00000198783	ZNF830	0.243	4.110	2.847	0.006
ENSG00000198799	LRIG2	-0.122	5.699	-2.428	0.017
ENSG00000198822	GRM3	0.187	6.077	2.096	0.039
ENSG00000198824	CHAMP1	-0.200	4.150	-2.262	0.026
ENSG00000198829	SUCNR1	1.260	-0.720	2.613	0.011
ENSG00000198836	OPA1	0.256	8.267	2.471	0.016
ENSG00000198839	ZNF277	0.277	4.526	2.570	0.012
ENSG00000198843	SELT	0.361	6.618	3.245	0.002
ENSG00000198846	TOX	-0.232	5.014	-2.656	0.010
ENSG00000198856	OSTC	0.490	3.134	3.204	0.002
ENSG00000198862	LTN1	0.187	6.723	2.321	0.023
ENSG00000198898	CAPZA2	0.530	7.165	3.359	0.001
ENSG00000198937	CCDC167	0.309	0.434	2.413	0.018
ENSG00000198944	SOWAHA	0.315	5.339	2.405	0.018
ENSG00000198945	L3MBTL3	0.246	3.694	2.634	0.010
ENSG00000199218	RN7SKP184	0.950	-1.384	2.049	0.044
ENSG00000199311	SNORD115-34	-0.684	-2.076	-2.171	0.033
ENSG00000199420	RN7SKP170	1.794	-1.899	2.717	0.008
ENSG00000199459	Y_RNA	-0.776	-2.526	-2.180	0.032
ENSG00000199585	RNA5SP119	-0.544	-1.384	-2.109	0.038
ENSG00000199592	RNA5SP321	-0.383	-0.734	-2.007	0.048
ENSG00000199627	RNU6-1010P	-0.899	-1.327	-3.754	0.000
ENSG00000199683	RN7SKP185	0.463	-0.086	2.005	0.048
ENSG00000199691	RN7SKP173	0.644	0.985	2.240	0.028
ENSG00000199719	RN7SKP74	-0.560	-0.770	-2.760	0.007
ENSG00000199933	RNY1P16	-0.671	-1.239	-2.385	0.019
ENSG00000200003	RNU6-986P	-0.992	-1.300	-3.294	0.001

ENSG00000200075	SNORA25	-0.561	-1.458	-2.697	0.008
ENSG00000200091	RN7SKP163	-0.393	0.663	-2.795	0.006
ENSG00000200114	RNA5SP123	-0.683	-1.157	-3.532	0.001
ENSG00000200218	RNU6-697P	-0.984	-2.431	-2.258	0.027
ENSG00000200351	Y_RNA	-0.590	-1.791	-2.602	0.011
ENSG00000200361	Y_RNA	-0.301	-0.814	-2.000	0.049
ENSG00000200406	SNORD114-23	-0.524	-1.632	-2.373	0.020
ENSG00000200478	SNORD115-41	-0.612	-0.837	-2.425	0.018
ENSG00000200550	RNU6-137P	-0.658	-2.146	-2.207	0.030
ENSG00000200594	RNU6-824P	-0.711	-2.989	-2.164	0.033
ENSG00000200638	SNORD115-37	-0.582	-1.867	-2.268	0.026
ENSG00000200719	RNA5SP260	-0.930	-2.468	-3.074	0.003
ENSG00000200753	SNORD56	-0.718	-2.310	-2.308	0.024
ENSG00000200783	RN7SKP180	0.647	1.379	2.603	0.011
ENSG00000200885	RNU1-146P	-0.526	-0.962	-2.373	0.020
ENSG00000201025	SNORD74	-0.569	-2.100	-2.402	0.019
ENSG00000201027	RN7SKP107	1.719	-3.273	3.090	0.003
ENSG00000201161	RN7SKP10	1.008	-3.454	2.253	0.027
ENSG00000201289	RN7SKP76	-0.578	-2.180	-2.062	0.042
ENSG00000201348	RNU105B	-0.616	-0.818	-2.251	0.027
ENSG00000201358	RN7SKP193	1.156	-2.045	2.725	0.008
ENSG00000201364	RN7SKP37	0.795	-1.241	2.850	0.006
ENSG00000201367	RNU6-522P	-0.752	-2.134	-2.618	0.011
ENSG00000201390	RNU6-1141P	-0.734	-2.605	-2.453	0.016
ENSG00000201498	Y_RNA	-0.814	-3.205	-2.113	0.038
ENSG00000201516	SNORA51	-0.720	-1.447	-2.387	0.019
ENSG00000201544	SNORA16B	-0.888	-2.012	-3.355	0.001
ENSG00000201581	RN7SKP78	-0.761	-1.406	-2.763	0.007
ENSG00000201600	RN7SKP124	-0.416	-0.762	-2.115	0.037
ENSG00000201662	RNU6-60P	-0.562	-1.131	-2.387	0.019
ENSG00000201794	RN7SKP130	2.055	-2.501	2.967	0.004
ENSG00000201847	SNORD31	-0.689	-1.350	-2.747	0.007
ENSG00000202017	RNU6-806P	-0.753	-2.266	-2.819	0.006
ENSG00000202141	Y_RNA	-0.492	-1.026	-2.888	0.005
ENSG00000202240	RNU6-737P	-0.863	-4.132	-2.066	0.042
ENSG00000202347	RNU1-16P	-0.509	-0.271	-2.764	0.007
ENSG00000203326	ZNF525	0.278	4.020	3.240	0.002
ENSG00000203327	AC012358.7	-0.498	-0.346	-2.543	0.013
ENSG00000203435	E2F3P2	-0.375	0.614	-2.105	0.038
ENSG00000203446	AC004988.1	-0.677	-1.606	-2.743	0.007
ENSG00000203601	LINC00970	-0.656	-1.540	-2.537	0.013
ENSG00000203643	AC012456.3	-0.960	-1.597	-2.470	0.016
ENSG00000203644	RP11-332M2.1	-0.449	1.287	-3.551	0.001
ENSG00000203667	COX20	-0.321	2.078	-2.461	0.016
ENSG00000203685	STUM	0.317	6.642	2.227	0.029
ENSG00000203724	C1orf53	0.499	-1.429	2.060	0.043
ENSG00000203730	TEDDM1	-1.089	-2.179	-3.182	0.002

ENSG00000203734	ECT2L	-0.475	1.684	-3.583	0.001
ENSG00000203739	RP11-296O14.3	-0.349	1.820	-2.873	0.005
ENSG00000203799	CCDC162P	0.272	2.168	2.451	0.016
ENSG00000203808	BVES-AS1	-0.330	0.226	-2.210	0.030
ENSG00000203855	HSD3BP4	-0.401	1.681	-2.448	0.017
ENSG00000203865	ATP1A1-AS1	-0.428	1.675	-3.583	0.001
ENSG00000203876	ADD3-AS1	-0.466	2.019	-3.042	0.003
ENSG00000204010	IFIT1B	-0.474	-1.471	-1.997	0.049
ENSG00000204084	INPP5B	-0.152	4.683	-2.353	0.021
ENSG00000204149	AGAP6	-0.313	2.898	-2.228	0.029
ENSG00000204209	DAXX	0.149	3.926	2.243	0.028
ENSG00000204220	PFDN6	0.365	4.049	2.896	0.005
ENSG00000204228	HSD17B8	0.384	-0.655	2.395	0.019
ENSG00000204237	OXLD1	0.276	2.501	2.548	0.013
ENSG00000204264	PSMB8	0.444	1.287	2.039	0.045
ENSG00000204301	NOTCH4	0.225	2.490	2.278	0.025
ENSG00000204308	RNF5	0.277	3.876	3.103	0.003
ENSG00000204315	FKBPL	0.235	1.306	2.068	0.042
ENSG00000204356	NELFE	0.271	3.660	2.754	0.007
ENSG00000204370	SDHD	0.369	3.846	3.584	0.001
ENSG00000204406	MBD5	-0.165	6.869	-2.944	0.004
ENSG00000204444	APOM	-0.405	-0.323	-2.481	0.015
ENSG00000204469	PRRC2A	-0.235	6.900	-2.147	0.035
ENSG00000204472	AIF1	0.901	1.579	2.059	0.043
ENSG00000204560	DHX16	0.162	4.151	2.169	0.033
ENSG00000204568	MRPS18B	0.360	3.664	3.374	0.001
ENSG00000204569	PPP1R10	-0.168	5.337	-2.399	0.019
ENSG00000204574	ABCF1	0.277	5.429	3.029	0.003
ENSG00000204581	AC096670.3	-0.609	-1.829	-2.037	0.045
ENSG00000204611	ZNF616	0.170	3.650	2.718	0.008
ENSG00000204623	ZNRD1ASP	-0.302	4.535	-2.984	0.004
ENSG00000204628	RACK1	0.242	6.593	2.957	0.004
ENSG00000204650	LINC02210	-0.244	5.315	-2.815	0.006
ENSG00000204683	C10orf113	-0.744	0.642	-4.536	0.000
ENSG00000204706	MAMDC2-AS1	-0.403	2.086	-2.997	0.004
ENSG00000204764	RANBP17	0.248	4.119	2.227	0.029
ENSG00000204789	ZNF204P	0.251	6.373	2.557	0.012
ENSG00000204842	ATXN2	-0.115	6.808	-2.736	0.008
ENSG00000204852	TCTN1	-0.195	4.212	-2.933	0.004
ENSG00000204856	FAM216A	0.258	4.221	2.440	0.017
ENSG00000204866	IGFL2	-0.461	0.257	-2.423	0.018
ENSG00000204899	MZT1	0.306	5.365	2.718	0.008
ENSG00000204956	PCDHGA1	-0.334	1.856	-3.072	0.003
ENSG00000204962	PCDHA8	-0.474	1.057	-3.102	0.003
ENSG00000204969	PCDHA2	-0.285	0.819	-2.563	0.012
ENSG00000204990	RP5-842K16.1	-0.523	1.206	-2.887	0.005
ENSG00000205133	TRIQK	0.216	5.719	2.477	0.015

ENSG00000205138	SDHAF1	0.326	2.247	2.742	0.007
ENSG00000205212	CCDC144NL	-0.440	1.568	-2.108	0.038
ENSG00000205221	VIT	0.562	0.214	2.800	0.006
ENSG00000205302	SNX2	0.337	5.416	3.164	0.002
ENSG00000205323	SARNP	-0.404	0.875	-2.931	0.004
ENSG00000205339	IPO7	0.286	7.588	2.522	0.014
ENSG00000205363	C15orf59	0.197	5.436	2.233	0.028
ENSG00000205500	AC013472.3	-0.483	0.822	-2.994	0.004
ENSG00000205531	NAP1L4	0.239	6.368	2.192	0.031
ENSG00000205544	TMEM256	0.311	1.608	2.273	0.026
ENSG00000205571	SMN2	-1.055	2.694	-2.403	0.019
ENSG00000205629	LCMT1	0.224	4.417	2.259	0.027
ENSG00000205634	LINC00898	-0.647	1.226	-4.065	0.000
ENSG00000205682	RP11-798L4.1	-0.344	1.774	-2.370	0.020
ENSG00000205704	LINC00634	0.293	1.764	2.610	0.011
ENSG00000205707	LYRM5	0.159	4.418	2.142	0.035
ENSG00000205758	CRYZL1	0.158	5.224	2.037	0.045
ENSG00000205771	CATSPER2P1	-0.418	1.558	-2.379	0.020
ENSG00000205898	RP11-3B12.4	-0.654	-1.181	-2.928	0.004
ENSG00000205940	HSP90AB2P	-0.658	3.200	-2.003	0.048
ENSG00000205981	DNAJC19	0.256	4.781	3.206	0.002
ENSG00000206028	CTA-373H7.7	-0.548	-0.909	-2.188	0.032
ENSG00000206052	DOK6	0.268	7.751	2.103	0.039
ENSG00000206199	ANKUB1	-0.559	-0.493	-2.818	0.006
ENSG00000206384	COL6A6	-0.226	3.094	-2.098	0.039
ENSG00000206418	RAB12	0.216	5.908	2.428	0.017
ENSG00000206432	TMEM200C	0.189	4.848	2.286	0.025
ENSG00000206448	PPIAP30	-0.792	-1.037	-2.756	0.007
ENSG00000206527	HACD2	0.202	6.156	2.887	0.005
ENSG00000206530	CFAP44	-0.235	4.786	-3.074	0.003
ENSG00000206535	LNP1	0.300	4.036	2.523	0.014
ENSG00000206552	KRBOX1-AS1	-0.746	-2.187	-2.129	0.036
ENSG00000206561	COLQ	-0.325	2.143	-2.057	0.043
ENSG00000206562	METTL6	0.116	4.664	2.202	0.030
ENSG00000206605	RNU6-946P	-0.760	-2.547	-2.864	0.005
ENSG00000206621	SNORD116-14	-0.424	0.474	-2.605	0.011
ENSG00000206623	RNU6-979P	-0.576	-0.546	-2.408	0.018
ENSG00000206684	RNU6-157P	-0.654	-2.127	-2.620	0.010
ENSG00000206728	Y_RNA	-0.597	-1.638	-2.389	0.019
ENSG00000206746	RNU6-142P	-0.537	-1.608	-2.010	0.048
ENSG00000206847	Y_RNA	-0.750	-1.120	-2.771	0.007
ENSG00000206889	RNU6-1200P	-0.437	0.117	-2.217	0.029
ENSG00000206985	RNU6-198P	-0.611	-2.141	-2.170	0.033
ENSG00000206991	RNU6-610P	-1.063	-1.981	-3.927	0.000
ENSG00000207001	SNORD116-2	-0.483	-1.016	-2.070	0.042
ENSG00000207008	SNORA54	0.662	-0.206	2.837	0.006
ENSG00000207034	Y_RNA	-0.837	-1.868	-2.676	0.009

ENSG00000207072	RNU6-39P	0.822	1.033	2.607	0.011
ENSG00000207129	RNA5SP187	-0.684	-0.954	-3.284	0.002
ENSG00000207197	SNORD116-12	-0.475	-0.972	-2.011	0.048
ENSG00000207205	RNVU1-15	1.326	-1.454	3.379	0.001
ENSG00000207248	RNU6-1005P	-0.626	-2.046	-2.929	0.004
ENSG00000207279	SNORD116-24	-0.505	-0.088	-2.838	0.006
ENSG00000207286	Y_RNA	-0.859	-1.829	-3.967	0.000
ENSG00000207318	RNU6-1184P	-0.533	-1.428	-2.057	0.043
ENSG00000207322	RNU1-89P	0.781	-2.048	2.256	0.027
ENSG00000207325	RNY1P4	-0.412	-0.897	-2.106	0.038
ENSG00000207368	Y_RNA	-0.791	-2.354	-2.553	0.013
ENSG00000207375	SNORD116-23	-0.347	0.050	-2.242	0.028
ENSG00000207425	Y_RNA	-0.586	-1.959	-2.032	0.045
ENSG00000207452	RNU6-606P	-0.574	-0.700	-2.542	0.013
ENSG00000207460	SNORD116-19	-0.629	0.306	-3.239	0.002
ENSG00000207461	RNU6-253P	-0.695	-5.677	-2.187	0.032
ENSG00000207507	RNU6-9	-0.444	1.623	-2.054	0.043
ENSG00000211450	C11orf31	0.220	2.764	2.143	0.035
ENSG00000211454	AKR7L	-0.293	0.598	-2.140	0.035
ENSG00000211456	SACM1L	0.173	6.378	2.008	0.048
ENSG00000211460	TSN	0.192	6.183	2.651	0.010
ENSG00000211772	TRBC2	0.505	1.258	2.272	0.026
ENSG00000212125	TAS2R15P	-0.444	1.797	-3.529	0.001
ENSG00000212126	TAS2R50	-0.412	0.786	-2.219	0.029
ENSG00000212127	TAS2R14	-0.344	2.597	-2.693	0.009
ENSG00000212128	TAS2R13	-0.372	2.583	-2.708	0.008
ENSG00000212175	SNORA12	-0.668	-1.675	-2.779	0.007
ENSG00000212181	SNORA48	-0.633	-1.654	-2.754	0.007
ENSG00000212380	SNORD115-45	-0.412	1.877	-2.628	0.010
ENSG00000212402	SNORA74B	-0.667	1.667	-2.740	0.008
ENSG00000212428	SNORD115	-0.441	0.894	-2.190	0.031
ENSG00000212461	SNORA17	-0.527	-2.153	-2.025	0.046
ENSG00000212482	RNU6-530P	-0.563	0.048	-2.644	0.010
ENSG00000212607	SNORA3B	-0.710	-0.010	-2.244	0.028
ENSG00000212625	RNA5SP397	-0.740	-2.197	-2.844	0.006
ENSG00000212695	RP11-134K1.2	-0.614	-0.917	-2.481	0.015
ENSG00000212961	HNRNPA1P40	-0.577	-1.381	-2.079	0.041
ENSG00000212978	AC016747.3	0.298	2.419	2.951	0.004
ENSG00000213046	EEF1A1P32	-0.514	-0.324	-2.568	0.012
ENSG00000213050	TPM3P1	-0.833	-0.864	-2.809	0.006
ENSG00000213055	EEF1B2P7	-0.561	-0.017	-2.501	0.014
ENSG00000213057	C1orf220	-0.272	1.274	-2.157	0.034
ENSG00000213073	RP11-288H12.3	-0.577	0.833	-3.861	0.000
ENSG00000213090	AC007256.5	-0.489	3.151	-2.280	0.025
ENSG00000213115	AC104131.1	0.848	-2.471	2.037	0.045
ENSG00000213203	GIMAP1	0.515	0.212	1.990	0.050
ENSG00000213213	CCDC183	0.284	0.332	1.992	0.050

ENSG00000213281	NRAS	0.206	5.571	2.837	0.006
ENSG00000213363	RPS3P6	-0.431	1.331	-2.003	0.049
ENSG00000213366	GSTM2	-0.229	3.448	-2.051	0.043
ENSG00000213424	KRT222	0.309	4.328	2.416	0.018
ENSG00000213463	SYNJ2BP	0.118	6.412	2.490	0.015
ENSG00000213484	EIF4A1P8	-0.824	-0.551	-3.982	0.000
ENSG00000213512	GBP7	-0.608	-1.236	-2.421	0.018
ENSG00000213513	RP13-39P12.2	-0.975	-1.382	-3.893	0.000
ENSG00000213519	AC132008.1	-0.927	-1.833	-2.600	0.011
ENSG00000213523	SRA1	0.178	3.527	2.272	0.026
ENSG00000213540	RP11-486G15.1	-0.573	-0.491	-2.983	0.004
ENSG00000213585	VDAC1	0.348	7.371	2.656	0.010
ENSG00000213587	RP11-646D13.1	-0.530	-0.929	-2.009	0.048
ENSG00000213592	AP000662.9	-0.619	-1.285	-2.573	0.012
ENSG00000213593	TMX2	0.273	5.082	2.574	0.012
ENSG00000213608	SLC25A14P1	-0.743	0.147	-2.500	0.014
ENSG00000213619	NDUFS3	0.243	4.488	2.602	0.011
ENSG00000213639	PPP1CB	0.165	8.262	2.107	0.038
ENSG00000213659	RSU1P3	-0.575	0.438	-3.126	0.002
ENSG00000213672	NCKIPSD	-0.236	4.871	-2.199	0.031
ENSG00000213676	ATF6B	-0.226	4.025	-2.387	0.019
ENSG00000213693	SEC14L1P1	-0.464	2.492	-2.290	0.025
ENSG00000213711	PHBP7	-0.533	-0.208	-2.223	0.029
ENSG00000213719	CLIC1	0.613	1.711	2.962	0.004
ENSG00000213741	RPS29	0.162	4.909	1.999	0.049
ENSG00000213742	ZNF337-AS1	-0.295	1.441	-2.601	0.011
ENSG00000213799	ZNF845	0.259	3.240	2.455	0.016
ENSG00000213871	TAF9BP1	-0.548	1.545	-3.263	0.002
ENSG00000213877	CFL1P7	-0.600	-1.475	-2.098	0.039
ENSG00000213949	ITGA1	0.301	4.326	2.353	0.021
ENSG00000213950	RPS10P2	-0.437	-0.794	-2.026	0.046
ENSG00000213963	AC074286.1	-0.249	2.888	-2.237	0.028
ENSG00000213967	ZNF726	-0.287	1.798	-2.999	0.004
ENSG00000213970	RP11-264F23.1	-0.459	-0.940	-2.194	0.031
ENSG00000213973	ZNF99	-0.330	1.418	-1.998	0.049
ENSG00000213976	CTD-2561J22.2	0.278	1.439	2.094	0.039
ENSG00000213995	NAXD	0.167	5.010	3.059	0.003
ENSG00000214026	MRPL23	0.413	2.202	2.159	0.034
ENSG00000214029	ZNF891	-0.221	5.174	-2.376	0.020
ENSG00000214039	LINC02418	0.548	-0.351	2.706	0.008
ENSG00000214062	RPL7P17	-0.595	-1.030	-2.346	0.021
ENSG00000214067	RP11-549L6.2	-0.419	0.657	-2.538	0.013
ENSG00000214087	ARL16	0.262	3.692	3.064	0.003
ENSG00000214106	PAXIP1-AS2	-0.297	3.323	-2.150	0.035
ENSG00000214114	MYCBP	-0.334	3.492	-2.743	0.007
ENSG00000214128	TMEM213	-0.801	-1.616	-2.285	0.025
ENSG00000214142	RPL7P60	-1.045	-1.811	-2.990	0.004

ENSG00000214188	ST7-OT4	-0.632	-0.090	-3.323	0.001
ENSG00000214190	RNF152P1	-0.478	-1.252	-2.151	0.034
ENSG00000214194	LINC00998	0.335	3.390	3.421	0.001
ENSG00000214211	CTAGE14P	-0.467	1.328	-2.949	0.004
ENSG00000214216	IQCJ	-0.431	2.947	-2.745	0.007
ENSG00000214244	SETP21	-0.484	1.783	-2.797	0.006
ENSG00000214331	RP11-252A24.2	-0.553	3.289	-4.597	0.000
ENSG00000214353	VAC14-AS1	-0.509	1.583	-3.224	0.002
ENSG00000214357	NEURL1B	0.300	4.081	2.267	0.026
ENSG00000214381	LINC00488	0.930	0.028	2.806	0.006
ENSG00000214413	BBIP1	-0.143	4.981	-2.331	0.022
ENSG00000214456	PLIN5	-0.640	0.805	-3.245	0.002
ENSG00000214457	RPL7P29	-0.515	0.830	-3.471	0.001
ENSG00000214510	SPINK13	-0.523	-0.445	-2.148	0.035
ENSG00000214530	STARD10	0.281	2.562	2.498	0.014
ENSG00000214549	SDHCP2	-0.791	-0.490	-3.445	0.001
ENSG00000214671	RPL6P12	-1.060	-2.016	-3.655	0.000
ENSG00000214688	C10orf105	-1.150	-1.447	-2.719	0.008
ENSG00000214700	C12orf71	-0.853	-2.346	-2.243	0.028
ENSG00000214753	HNRNPUL2	-0.291	5.137	-3.685	0.000
ENSG00000214761	HNRNPA1P15	-0.814	-0.810	-2.918	0.005
ENSG00000214814	FER1L6	0.586	-0.067	2.408	0.018
ENSG00000214872	SMTNL1	-0.508	-0.830	-2.046	0.044
ENSG00000214903	RPS15AP3	-0.357	0.120	-2.196	0.031
ENSG00000214988	RPL7AP26	-0.546	-0.608	-2.243	0.028
ENSG00000215018	COL28A1	-0.377	2.711	-2.904	0.005
ENSG00000215022	RP1-257A7.4	-0.483	1.869	-3.099	0.003
ENSG00000215114	UBXN2B	0.144	6.189	2.205	0.030
ENSG00000215126	CBWD7	-0.491	4.136	-2.265	0.026
ENSG00000215158	RP11-1023L17.1	-0.329	3.824	-2.194	0.031
ENSG00000215190	LINC00680	0.221	3.335	2.093	0.039
ENSG00000215244	RP11-563J2.2	-0.477	1.452	-2.298	0.024
ENSG00000215252	GOLGA8B	-0.613	6.216	-2.070	0.042
ENSG00000215283	HMGB3P24	-0.541	-1.016	-2.256	0.027
ENSG00000215297	RP11-62E14.2	-0.783	-1.437	-3.390	0.001
ENSG00000215305	VPS16	0.247	3.836	2.479	0.015
ENSG00000215325	ASS1P10	-0.382	-0.488	-2.111	0.038
ENSG00000215347	SLC25A5P1	-0.523	-1.011	-2.344	0.021
ENSG00000215367	TMED11P	-0.550	-1.214	-2.130	0.036
ENSG00000215380	AC090625.1	-0.804	1.876	-2.680	0.009
ENSG00000215386	MIR99AHG	-0.162	5.820	-2.374	0.020
ENSG00000215388	ACTG1P3	-0.559	0.159	-2.411	0.018
ENSG00000215399	HMGB3P7	-0.497	0.607	-2.028	0.046
ENSG00000215424	MCM3AP-AS1	-0.297	2.977	-3.292	0.001
ENSG00000215452	ZNF663P	-0.745	-2.735	-2.132	0.036
ENSG00000215478	CES5AP1	-0.588	-2.005	-2.024	0.046
ENSG00000215483	LINC00598	-0.435	0.716	-2.163	0.033

ENSG00000215520	RPS4XP4	-1.423	-3.052	-3.960	0.000
ENSG00000215712	TMEM242	0.262	5.163	2.657	0.009
ENSG00000215717	TMEM167B	0.218	5.455	2.772	0.007
ENSG00000215796	RP11-551G24.2	-0.587	-0.676	-2.166	0.033
ENSG00000215802	RFKP1	-0.609	0.163	-2.709	0.008
ENSG00000215845	TSTD1	0.414	2.142	3.356	0.001
ENSG00000215859	RP6-74O6.2	-0.551	1.842	-3.029	0.003
ENSG00000215875	ST13P20	-0.444	0.750	-2.483	0.015
ENSG00000215887	ZNF859P	-0.699	0.386	-3.689	0.000
ENSG00000215893	RPL23AP17	-0.898	-2.537	-2.371	0.020
ENSG00000216192	MIR920	-0.642	-2.525	-2.101	0.039
ENSG00000216316	RP3-354N19.3	-0.763	-0.912	-2.618	0.011
ENSG00000216613	RP3-406A7.5	-0.388	0.480	-2.114	0.038
ENSG00000216636	RPL7P25	-0.693	-1.385	-2.878	0.005
ENSG00000216895	AC009403.2	-0.288	0.909	-2.112	0.038
ENSG00000216915	RP1-97D16.1	-0.552	0.405	-2.010	0.048
ENSG00000217334	RP11-33E24.3	-1.115	-3.095	-2.377	0.020
ENSG00000217488	RP11-474L11.5	-0.493	0.882	-2.840	0.006
ENSG00000217495	RP1-95L4.2	-0.771	-0.652	-4.292	0.000
ENSG00000217612	RP1-28C20.1	-0.560	0.056	-2.493	0.015
ENSG00000217648	RP1-95L4.4	-0.294	2.061	-2.216	0.029
ENSG00000217653	RP11-63K6.1	-0.724	-2.337	-2.145	0.035
ENSG00000217783	LDHAL6FP	-0.413	-0.540	-2.176	0.032
ENSG00000217801	RP11-465B22.3	0.283	0.579	2.054	0.043
ENSG00000217897	HSPE1P8	-0.684	-1.083	-2.278	0.025
ENSG00000218020	THAP12P5	-0.981	-2.476	-2.625	0.010
ENSG00000218186	KRT8P43	-0.618	-0.631	-2.449	0.016
ENSG00000218226	TATDN2P2	-0.353	1.571	-2.488	0.015
ENSG00000218313	RP11-393I2.2	-0.434	1.459	-2.533	0.013
ENSG00000218424	NDUFS5P1	-0.460	0.061	-2.308	0.024
ENSG00000218428	NIP7P3	-0.718	-0.820	-3.493	0.001
ENSG00000218475	ACTG1P18	-0.491	1.401	-3.230	0.002
ENSG00000218596	RP11-2J18.1	-0.473	0.590	-3.038	0.003
ENSG00000218676	BRD7P4	-0.590	1.767	-2.063	0.042
ENSG00000218757	RP3-472M2.2	-0.413	0.713	-2.865	0.005
ENSG00000218823	PAPOLB	-0.443	0.520	-2.013	0.047
ENSG00000218996	RP1-99E18.2	-0.448	0.573	-2.387	0.019
ENSG00000219085	NPM1P37	-0.241	1.283	-2.059	0.043
ENSG00000219222	RPL12P47	-0.876	-2.196	-2.975	0.004
ENSG00000219355	RPL31P52	-0.618	-1.637	-2.286	0.025
ENSG00000219384	RP11-491H9.3	-0.515	-0.868	-2.394	0.019
ENSG00000219433	BTBD10P2	-0.444	-0.130	-2.438	0.017
ENSG00000219445	RP11-3B12.3	-0.876	-1.601	-2.824	0.006
ENSG00000219547	RPL17P25	-0.817	-0.790	-3.319	0.001
ENSG00000219665	CTD-2006C1.2	-0.232	1.748	-2.070	0.042
ENSG00000219881	GAPDHP42	-0.439	0.559	-2.585	0.012
ENSG00000219928	RP11-40C6.2	-0.628	-1.157	-2.153	0.034

ENSG00000219951	RP11-378G13.2	-0.539	-0.701	-2.170	0.033
ENSG00000220323	HIST2H2BD	-0.385	1.270	-3.122	0.002
ENSG00000220392	FCF1P5	-0.400	-0.004	-2.024	0.046
ENSG00000220506	RP3-415N12.1	-0.536	0.812	-2.959	0.004
ENSG00000220614	RP11-480N24.4	-0.477	-0.731	-2.146	0.035
ENSG00000220744	RPL5P18	-0.349	0.989	-2.013	0.047
ENSG00000220848	RPS18P9	-0.238	1.679	-2.315	0.023
ENSG00000221063	MIR1296	-0.737	-1.441	-3.278	0.002
ENSG00000221265	MIR1255A	-0.794	-2.415	-2.310	0.023
ENSG00000221562	RNU6ATAC10P	-0.569	-0.800	-2.232	0.028
ENSG00000221630	MIR1179	-0.602	-1.868	-2.471	0.016
ENSG00000221650	MIR1267	-0.375	-0.595	-2.161	0.034
ENSG00000221838	AP4M1	0.369	1.622	2.945	0.004
ENSG00000221843	C2orf16	-0.262	4.114	-3.088	0.003
ENSG00000221909	FAM200A	0.239	3.440	2.532	0.013
ENSG00000221923	ZNF880	0.247	5.087	3.108	0.003
ENSG00000221983	UBA52	0.172	6.212	2.217	0.029
ENSG00000221990	EXOC3-AS1	0.362	0.933	2.899	0.005
ENSG00000222024	AC004945.1	-0.415	2.403	-3.177	0.002
ENSG00000222041	LINC00152	-0.359	2.460	-2.252	0.027
ENSG00000222051	RNU6-1165P	-0.656	-2.404	-2.374	0.020
ENSG00000222078	RN7SKP110	-0.809	-2.478	-2.471	0.016
ENSG00000222164	RN7SKP266	-0.515	-1.455	-2.082	0.040
ENSG00000222317	RNA5SP118	-0.733	-1.754	-2.984	0.004
ENSG00000222365	SNORD12B	-0.531	-2.300	-2.040	0.045
ENSG00000222404	RN7SKP281	2.418	-1.597	3.338	0.001
ENSG00000222413	RN7SKP125	-0.475	-1.417	-2.031	0.045
ENSG00000222445	RN7SKP56	-0.611	-1.351	-2.467	0.016
ENSG00000222594	RN7SKP235	-0.681	-1.616	-2.781	0.007
ENSG00000222649	Y_RNA	-0.656	-2.863	-2.080	0.041
ENSG00000222685	RN7SKP119	1.727	-2.101	3.490	0.001
ENSG00000222985	RNU2-14P	-0.963	-2.937	-2.259	0.027
ENSG00000223044	RNU6-130P	-0.445	-1.547	-2.236	0.028
ENSG00000223117	RN7SKP296	-0.928	-2.183	-3.249	0.002
ENSG00000223190	RN7SKP100	-0.775	-0.913	-3.484	0.001
ENSG00000223324	RN7SKP273	-0.688	-2.130	-2.079	0.041
ENSG00000223409	RPL17P17	-0.654	-0.787	-2.236	0.028
ENSG00000223427	AC016716.1	-0.537	2.137	-3.191	0.002
ENSG00000223442	TH2LCRR	-0.311	-0.089	-2.227	0.029
ENSG00000223450	RP11-52I18.1	-0.639	-1.799	-2.342	0.022
ENSG00000223460	GAPDHP69	-0.670	-0.929	-2.295	0.024
ENSG00000223519	KIF28P	-0.463	0.551	-2.658	0.009
ENSG00000223522	AC093690.1	-0.426	1.755	-2.792	0.007
ENSG00000223528	RP11-223P11.3	-0.401	-0.446	-2.083	0.040
ENSG00000223584	TVP23CP1	-0.537	-0.982	-2.046	0.044
ENSG00000223642	AC008277.1	-0.375	1.228	-2.393	0.019
ENSG00000223653	RP11-131L23.1	-0.625	1.758	-4.050	0.000

ENSG00000223694	ADH5P3	-0.424	1.550	-2.941	0.004
ENSG00000223724	RAD17P2	-0.657	0.150	-3.354	0.001
ENSG00000223754	AC008073.9	-1.153	-1.480	-4.958	0.000
ENSG00000223770	AC006145.4	-0.375	2.458	-2.265	0.026
ENSG00000223787	RP4-593M8.1	-0.618	-0.729	-2.697	0.008
ENSG00000223812	RP11-197K6.1	-0.375	3.058	-2.265	0.026
ENSG00000223842	RP11-135J2.3	-0.419	0.063	-2.119	0.037
ENSG00000223843	EFCAB6-AS1	-0.481	-0.539	-2.323	0.023
ENSG00000223864	NPM1P19	-0.584	-0.511	-2.624	0.010
ENSG00000223878	AC005517.3	-0.614	-0.275	-2.649	0.010
ENSG00000223884	AC019048.1	-0.570	0.867	-2.875	0.005
ENSG00000223896	CCNJP2	-0.534	-0.394	-2.947	0.004
ENSG00000223899	SEC13P1	-0.527	-0.852	-2.051	0.043
ENSG00000223922	ASS1P2	-0.598	-0.556	-2.877	0.005
ENSG00000223965	ZNF587P1	-0.624	-1.438	-2.389	0.019
ENSG00000224003	YES1P1	-0.438	2.968	-2.913	0.005
ENSG00000224007	AC019070.1	-0.539	0.379	-2.819	0.006
ENSG00000224032	EPB41L4A-AS1	0.299	4.134	3.046	0.003
ENSG00000224055	GAPDHP55	-0.412	0.713	-2.481	0.015
ENSG00000224057	EGFR-AS1	-0.557	-0.878	-2.338	0.022
ENSG00000224078	SNHG14	-0.202	10.038	-3.040	0.003
ENSG00000224080	UBE2FP1	-0.619	-0.788	-2.737	0.008
ENSG00000224083	MTCO1P11	-0.916	-0.175	-4.276	0.000
ENSG00000224093	RP5-1033H22.2	-0.540	-0.473	-2.950	0.004
ENSG00000224117	PTPN2P2	-0.662	1.087	-3.119	0.003
ENSG00000224121	ATG12P2	-0.597	-0.078	-2.956	0.004
ENSG00000224222	RP11-262I2.2	-0.466	-0.258	-2.058	0.043
ENSG00000224238	WARS2-IT1	-0.769	-1.067	-3.094	0.003
ENSG00000224255	PDCL3P6	-0.435	0.138	-2.033	0.045
ENSG00000224257	VWC2L-IT1	-0.826	-2.419	-2.979	0.004
ENSG00000224273	AC005077.7	-0.531	-0.665	-2.102	0.039
ENSG00000224310	LINC01567	-0.414	2.674	-2.460	0.016
ENSG00000224315	RPL7P7	-0.446	0.334	-2.415	0.018
ENSG00000224323	DPRXP1	-0.564	-0.992	-2.509	0.014
ENSG00000224356	RP11-151A6.4	-0.590	-1.289	-2.035	0.045
ENSG00000224367	OACYLP	0.565	-1.337	2.332	0.022
ENSG00000224414	AC010886.2	-0.539	0.489	-2.876	0.005
ENSG00000224442	AC017035.2	-0.566	-0.668	-2.983	0.004
ENSG00000224448	GS1-259H13.7	-0.525	-0.638	-2.082	0.041
ENSG00000224479	AC136289.1	-0.368	2.217	-2.825	0.006
ENSG00000224493	RP11-87O11.1	-0.562	-0.164	-2.820	0.006
ENSG00000224494	HNRNPA3P14	-0.547	-0.789	-2.282	0.025
ENSG00000224500	RP11-328K22.1	-0.505	-0.576	-2.100	0.039
ENSG00000224530	PPIAP10	-0.972	-3.104	-2.219	0.029
ENSG00000224531	SMIM13	0.274	6.834	2.877	0.005
ENSG00000224541	AP001439.2	-0.730	-0.501	-3.697	0.000
ENSG00000224543	SNRPGP15	-0.610	-0.213	-2.724	0.008

ENSG00000224660	SH3BP5-AS1	-0.402	3.119	-3.362	0.001
ENSG00000224668	IPO8P1	-0.485	3.002	-2.098	0.039
ENSG00000224700	RP11-697E14.2	-0.398	0.096	-2.332	0.022
ENSG00000224707	E2F3-IT1	-0.443	-0.607	-2.582	0.012
ENSG00000224727	FCF1P7	-0.525	0.499	-2.509	0.014
ENSG00000224731	AC016716.2	-0.524	1.702	-3.664	0.000
ENSG00000224751	SHMT1P1	-0.468	-0.830	-2.373	0.020
ENSG00000224752	VN1R21P	-0.963	-1.617	-3.898	0.000
ENSG00000224808	SRGAP3-AS1	-0.667	-1.415	-2.638	0.010
ENSG00000224812	TMEM72-AS1	-0.259	2.379	-2.255	0.027
ENSG00000224819	AC093843.1	-0.298	1.214	-2.715	0.008
ENSG00000224822	THRB-IT1	-0.412	2.826	-2.474	0.015
ENSG00000224870	RP4-758J18.2	0.184	3.094	2.165	0.033
ENSG00000224877	C17orf89	0.348	3.265	2.865	0.005
ENSG00000224891	AC007899.3	-0.786	-1.808	-3.038	0.003
ENSG00000224934	RP11-441O15.3	-0.391	3.182	-2.653	0.010
ENSG00000224945	RP11-82L18.2	-0.331	2.261	-2.675	0.009
ENSG00000224957	LINC01266	-0.478	1.129	-2.409	0.018
ENSG00000224973	LARGE-AS1	-0.494	2.052	-2.812	0.006
ENSG00000224986	PPP1R8P1	-0.431	1.239	-2.575	0.012
ENSG00000225014	KCTD9P1	-0.382	2.152	-2.344	0.022
ENSG00000225078	RP11-123N4.4	-0.664	-0.551	-2.949	0.004
ENSG00000225087	RP4-660H19.1	-0.592	-1.565	-2.000	0.049
ENSG00000225111	AC083900.1	-0.491	-0.482	-2.467	0.016
ENSG00000225118	RP11-477L16.2	-0.505	-1.623	-2.036	0.045
ENSG00000225181	RP11-388K2.1	-0.624	-1.998	-2.036	0.045
ENSG00000225194	LINC00092	-0.694	1.184	-4.039	0.000
ENSG00000225213	RP11-197M22.2	-0.456	-0.549	-2.565	0.012
ENSG00000225215	SMARCE1P1	-0.414	1.123	-2.407	0.018
ENSG00000225216	AC007362.3	-0.543	-1.393	-2.644	0.010
ENSG00000225263	PCDH9-AS3	-0.300	2.420	-2.246	0.027
ENSG00000225303	RP11-512N4.2	-0.545	-1.098	-2.401	0.019
ENSG00000225313	RP11-415J8.3	-0.303	1.798	-2.465	0.016
ENSG00000225349	RP11-820K3.2	-0.875	-1.119	-3.760	0.000
ENSG00000225423	TNPO1P1	-0.444	1.779	-1.996	0.049
ENSG00000225460	RP13-93L13.1	-0.840	-0.628	-2.392	0.019
ENSG00000225465	RFPL1S	0.260	6.686	2.038	0.045
ENSG00000225473	ATP13A4-AS1	-0.741	-0.807	-2.848	0.006
ENSG00000225497	KCNMA1-AS2	-0.673	-0.620	-2.734	0.008
ENSG00000225498	AC002064.5	-0.559	-1.057	-2.171	0.033
ENSG00000225505	RP11-330C7.3	-0.557	0.265	-3.183	0.002
ENSG00000225527	RP11-383B4.4	-0.485	0.732	-2.516	0.014
ENSG00000225542	ZNF385D-AS1	-0.508	-0.777	-2.222	0.029
ENSG00000225572	DOCK4-AS1	-0.378	3.359	-2.798	0.006
ENSG00000225648	SBDSP1	0.155	4.255	2.449	0.016
ENSG00000225650	EIF2S2P5	-0.524	-0.500	-2.586	0.011
ENSG00000225652	KCNMA1-AS3	-0.515	1.003	-2.905	0.005

ENSG00000225663	MCRIP1	0.261	3.551	2.692	0.009
ENSG00000225665	SAR1P3	-0.769	-0.780	-2.287	0.025
ENSG00000225670	CADM3-AS1	-0.487	2.068	-3.425	0.001
ENSG00000225675	LINC01771	-0.437	0.175	-2.852	0.005
ENSG00000225681	AC005730.2	-0.669	-1.392	-3.072	0.003
ENSG00000225711	RP11-345I18.4	-0.479	-0.340	-2.316	0.023
ENSG00000225738	NRG3-AS1	-0.588	1.628	-3.610	0.001
ENSG00000225767	RP5-850O15.3	-0.502	0.693	-3.350	0.001
ENSG00000225779	RP4-603I14.3	-0.859	-2.331	-3.147	0.002
ENSG00000225805	CTD-2313N18.7	-0.535	-2.081	-2.155	0.034
ENSG00000225811	RP11-547I7.1	-0.494	-0.495	-2.829	0.006
ENSG00000225826	LINC00626	-0.618	-1.336	-2.629	0.010
ENSG00000225855	RUSC1-AS1	-0.302	1.564	-2.382	0.020
ENSG00000225885	AC023590.1	-0.418	3.063	-2.867	0.005
ENSG00000225891	RP3-476K8.3	-0.375	-0.629	-2.131	0.036
ENSG00000225893	RP11-6J24.3	-0.917	-1.242	-3.596	0.001
ENSG00000225911	AC009310.1	-0.593	-0.391	-2.462	0.016
ENSG00000225920	RIMKLBP2	-0.419	1.437	-2.220	0.029
ENSG00000225921	NOL7	0.332	4.682	2.764	0.007
ENSG00000225928	CACYBPP1	-0.560	-0.257	-2.288	0.025
ENSG00000225938	RP4-575N6.4	-0.699	-1.617	-2.241	0.028
ENSG00000225948	RP11-554I8.1	-0.790	-2.537	-2.111	0.038
ENSG00000226026	RP11-57H12.3	-0.477	0.838	-2.874	0.005
ENSG00000226029	LINC01772	-0.393	1.928	-2.560	0.012
ENSG00000226045	AC009274.6	-0.676	-1.999	-2.455	0.016
ENSG00000226070	RP11-552E20.4	-0.423	0.125	-2.070	0.042
ENSG00000226083	SLC39A12-AS1	-1.102	-1.404	-2.746	0.007
ENSG00000226091	LINC00937	-0.422	1.718	-2.457	0.016
ENSG00000226101	LINC02097	-0.702	-0.717	-3.037	0.003
ENSG00000226128	RPL26P9	-0.613	-1.592	-2.266	0.026
ENSG00000226167	AP4B1-AS1	-0.442	0.277	-2.131	0.036
ENSG00000226200	SGMS1-AS1	-0.313	2.491	-3.065	0.003
ENSG00000226259	GTF2H2B	-0.878	1.537	-2.008	0.048
ENSG00000226266	AC009961.3	-0.279	1.361	-2.350	0.021
ENSG00000226272	ARHGAP26-AS1	-0.768	-0.700	-4.018	0.000
ENSG00000226285	AC091813.2	-0.974	-2.212	-2.825	0.006
ENSG00000226288	OR52I2	-0.944	-1.326	-2.986	0.004
ENSG00000226306	NPY6R	-0.455	2.393	-2.527	0.013
ENSG00000226314	ZNF192P1	-0.360	3.115	-3.039	0.003
ENSG00000226318	RP11-474D14.2	-0.284	2.255	-1.996	0.049
ENSG00000226328	NUP50-AS1	0.305	3.316	3.036	0.003
ENSG00000226352	RP11-523H24.3	-0.484	-0.131	-3.148	0.002
ENSG00000226367	ST7-AS2	-0.471	2.782	-3.270	0.002
ENSG00000226375	RP3-395P12.2	-0.710	-0.605	-3.543	0.001
ENSG00000226380	MIR29A	-0.307	4.594	-2.610	0.011
ENSG00000226383	LINC01876	-1.350	-2.791	-2.606	0.011
ENSG00000226387	SORCS3-AS1	-0.586	0.687	-2.663	0.009

ENSG00000226393	IFNA20P	-0.431	-1.119	-2.169	0.033
ENSG00000226438	RP11-420K8.1	-0.596	-1.031	-2.973	0.004
ENSG00000226469	ADAM1B	-0.317	0.785	-2.034	0.045
ENSG00000226476	LINC01748	-0.227	3.488	-2.221	0.029
ENSG00000226491	FTOP1	-0.500	0.399	-2.450	0.016
ENSG00000226493	RP11-524O24.2	-0.671	-0.119	-3.413	0.001
ENSG00000226522	AC004744.3	-0.641	-1.598	-2.429	0.017
ENSG00000226533	BTBD9-AS1	-0.393	0.198	-2.259	0.027
ENSG00000226562	CYP4F26P	-0.724	-0.334	-2.940	0.004
ENSG00000226604	PAPPA-AS2	-1.106	-2.282	-2.672	0.009
ENSG00000226626	NOP56P2	-0.795	0.006	-3.690	0.000
ENSG00000226632	UBE2V1P1	-0.378	1.338	-2.051	0.043
ENSG00000226642	ACTG1P12	-0.426	1.116	-2.161	0.034
ENSG00000226648	PLCG1-AS1	-0.360	0.216	-2.524	0.014
ENSG00000226686	LINC01535	-0.442	1.847	-2.096	0.039
ENSG00000226695	ANKRD20A10P	0.495	-0.249	2.088	0.040
ENSG00000226711	FAM66C	-0.353	4.369	-3.636	0.000
ENSG00000226744	AC079781.5	-0.600	-0.915	-2.996	0.004
ENSG00000226751	AF127936.5	0.899	0.490	2.745	0.007
ENSG00000226759	DAB1-AS1	-0.482	1.984	-2.990	0.004
ENSG00000226761	TAS2R46	-0.390	0.291	-2.241	0.028
ENSG00000226770	AC000124.1	-0.750	-2.075	-2.213	0.030
ENSG00000226797	AC015923.1	-0.620	-0.243	-2.811	0.006
ENSG00000226816	AC005082.12	0.683	-1.164	2.225	0.029
ENSG00000226849	RP4-635E18.7	-0.599	-1.573	-2.185	0.032
ENSG00000226853	AC010894.3	-0.699	-1.801	-2.176	0.032
ENSG00000226874	AC005154.8	-0.742	-0.684	-3.325	0.001
ENSG00000226876	RP11-36N20.1	-0.350	1.457	-2.040	0.045
ENSG00000226877	RP11-575L7.2	-0.524	-0.695	-2.511	0.014
ENSG00000226891	LINC01359	-0.490	3.182	-2.849	0.006
ENSG00000226899	RP11-298J20.3	-0.464	-0.491	-2.145	0.035
ENSG00000226946	AC062022.1	-0.665	-1.437	-2.348	0.021
ENSG00000226978	MAGI2-AS2	-0.748	-1.071	-3.397	0.001
ENSG00000226984	RP4-591B8.2	-0.570	-1.630	-2.245	0.027
ENSG00000227006	RP5-956O18.2	-0.468	-0.227	-2.498	0.015
ENSG00000227014	AC007285.6	-0.331	0.549	-2.282	0.025
ENSG00000227017	AC007036.6	-0.532	-0.474	-2.734	0.008
ENSG00000227028	SLC8A1-AS1	-0.490	3.496	-3.451	0.001
ENSG00000227068	RP11-12A16.3	-0.562	-1.594	-2.423	0.018
ENSG00000227072	RP3-466I7.1	-0.870	-0.983	-3.964	0.000
ENSG00000227073	SDHDP2	-0.775	-1.240	-3.294	0.001
ENSG00000227082	CH17-437K3.1	0.259	3.559	2.202	0.030
ENSG00000227110	LMCD1-AS1	-0.382	2.965	-3.014	0.003
ENSG00000227124	ZNF717	-0.117	4.933	-2.450	0.016
ENSG00000227165	WDR11-AS1	-0.345	2.279	-2.894	0.005
ENSG00000227170	AF178030.2	-0.599	0.276	-3.042	0.003
ENSG00000227192	RP1-45I4.3	-0.469	-0.882	-2.038	0.045

ENSG00000227197	AC009518.8	-0.423	0.120	-2.323	0.023
ENSG00000227215	RP11-445L13__B.3	-0.797	-1.405	-3.559	0.001
ENSG00000227227	AC017101.10	-0.381	-0.124	-2.259	0.027
ENSG00000227248	FAM155A-IT1	-0.444	3.041	-2.807	0.006
ENSG00000227249	AC000374.1	-0.491	-0.622	-2.235	0.028
ENSG00000227267	RP11-305O4.2	-0.555	-1.137	-2.050	0.044
ENSG00000227279	AC015933.2	-0.539	-1.061	-2.719	0.008
ENSG00000227294	AC016994.2	-0.692	-0.866	-3.254	0.002
ENSG00000227321	MTND4P15	-0.372	-0.563	-2.103	0.039
ENSG00000227331	AC005042.2	-0.418	0.423	-2.525	0.013
ENSG00000227352	ARHGEF7-AS1	-0.569	-1.446	-2.441	0.017
ENSG00000227374	RP11-109A6.3	-0.578	-1.910	-2.185	0.032
ENSG00000227383	RP11-571F15.2	-0.531	-0.781	-2.522	0.014
ENSG00000227388	RP11-112J3.16	-0.374	1.077	-2.696	0.009
ENSG00000227394	AC007386.3	-0.588	-0.080	-2.873	0.005
ENSG00000227395	EIF2AP4	-0.676	0.423	-3.290	0.001
ENSG00000227398	KIF9-AS1	-0.207	1.856	-2.364	0.020
ENSG00000227409	ZMYM4-AS1	-0.377	-1.092	-2.417	0.018
ENSG00000227417	RP11-397P13.6	-0.604	0.051	-2.981	0.004
ENSG00000227436	FCF1P1	-0.615	-0.925	-2.532	0.013
ENSG00000227487	NCAM1-AS1	-0.435	0.337	-2.856	0.005
ENSG00000227500	SCAMP4	-0.249	2.991	-2.193	0.031
ENSG00000227532	AC002542.2	-0.666	-1.666	-2.758	0.007
ENSG00000227554	RP11-444D13.1	-0.603	-0.518	-2.730	0.008
ENSG00000227573	RP5-1166A24.1	-0.849	-1.664	-2.540	0.013
ENSG00000227599	RP4-753D4.2	-0.487	0.361	-2.506	0.014
ENSG00000227638	HNRNPA1P14	-0.514	1.240	-2.381	0.020
ENSG00000227692	MED28P3	-0.799	-1.045	-3.385	0.001
ENSG00000227719	AC006042.6	-0.485	0.635	-2.564	0.012
ENSG00000227748	RP11-497D6.3	-0.826	-0.946	-3.808	0.000
ENSG00000227773	ASH1L-IT1	-0.500	-0.781	-2.086	0.040
ENSG00000227782	CTC-529I10.2	-0.412	0.464	-2.375	0.020
ENSG00000227815	RP11-195C7.3	-0.529	-1.452	-2.484	0.015
ENSG00000227848	SUCLA2-AS1	0.366	-0.436	2.098	0.039
ENSG00000227911	LINC02344	-0.906	-1.633	-3.678	0.000
ENSG00000227913	KRT8P44	-0.693	-1.724	-2.911	0.005
ENSG00000227914	RP11-130C19.3	-0.902	-1.753	-3.365	0.001
ENSG00000227927	MACROD2-IT1	-0.408	-0.374	-2.206	0.030
ENSG00000227929	SRGAP3-AS3	-0.450	0.475	-2.310	0.023
ENSG00000227948	AC003989.4	-0.454	-0.226	-2.719	0.008
ENSG00000227950	RP11-312B8.2	-0.730	-1.316	-3.447	0.001
ENSG00000227953	LINC01341	-0.424	1.284	-2.301	0.024
ENSG00000227963	RP5-1074L1.1	-0.639	-1.171	-2.954	0.004
ENSG00000228021	RP11-383C5.3	-0.482	-0.615	-2.260	0.027
ENSG00000228024	CYP1D1P	-0.868	-0.578	-2.713	0.008
ENSG00000228027	RP1-251M9.3	-0.431	0.959	-2.415	0.018
ENSG00000228036	HSPD1P9	-0.780	-3.115	-2.147	0.035

ENSG00000228061	Z83001.1	-0.356	1.660	-2.531	0.013
ENSG00000228097	MTATP6P11	-0.739	-1.739	-2.171	0.033
ENSG00000228108	AC092839.3	-0.469	-0.636	-2.554	0.013
ENSG00000228135	LINC01494	-0.474	-0.427	-2.671	0.009
ENSG00000228195	RPL5P27	-0.746	-0.424	-2.701	0.008
ENSG00000228204	RP4-724E13.2	-0.503	-0.147	-2.900	0.005
ENSG00000228212	OFD1P17	-0.512	2.006	-2.025	0.046
ENSG00000228219	NPM1P30	-0.434	0.008	-2.187	0.032
ENSG00000228222	AC074363.1	-0.291	0.736	-2.234	0.028
ENSG00000228242	AC093495.4	-0.419	0.780	-3.081	0.003
ENSG00000228274	RP3-508I15.9	-0.241	1.555	-2.013	0.047
ENSG00000228337	RP11-804H8.5	-0.824	-1.387	-2.888	0.005
ENSG00000228341	CTB-20D2.1	-0.568	-1.492	-2.254	0.027
ENSG00000228391	AC011995.3	-0.642	-1.613	-2.173	0.033
ENSG00000228393	LINC01004	-0.344	1.117	-2.523	0.014
ENSG00000228395	RP11-216B9.6	-0.250	0.388	-2.046	0.044
ENSG00000228409	CCT6P1	-0.348	2.654	-4.063	0.000
ENSG00000228474	OST4	0.268	2.367	2.080	0.041
ENSG00000228481	U51244.2	-0.468	0.221	-2.681	0.009
ENSG00000228484	RP11-106M7.1	-0.451	1.123	-2.657	0.009
ENSG00000228506	RP11-98I9.4	-0.247	1.144	-2.143	0.035
ENSG00000228509	AC006460.2	-0.485	-1.147	-2.119	0.037
ENSG00000228510	RP11-339B21.8	-0.515	1.104	-2.104	0.038
ENSG00000228536	RP11-392O17.1	-0.465	0.439	-2.017	0.047
ENSG00000228568	AC006461.2	-0.462	-1.461	-1.996	0.049
ENSG00000228577	AC010731.2	-0.524	0.488	-2.761	0.007
ENSG00000228592	D21S2088E	1.512	-2.044	3.597	0.001
ENSG00000228599	RPL7P52	-0.614	0.296	-3.068	0.003
ENSG00000228625	RP11-1B20.1	-0.535	-0.144	-2.839	0.006
ENSG00000228653	HNRNPCP7	-0.349	1.700	-2.456	0.016
ENSG00000228663	PSMD10P1	-0.539	1.498	-3.790	0.000
ENSG00000228667	RP11-129O7.2	-0.530	-1.159	-2.326	0.023
ENSG00000228672	PROB1	-0.343	0.195	-2.107	0.038
ENSG00000228677	TTC3-AS1	-0.487	0.423	-2.328	0.022
ENSG00000228680	AC004692.4	-0.621	-0.091	-3.050	0.003
ENSG00000228686	RP11-492I21.1	-0.537	-0.208	-2.647	0.010
ENSG00000228696	ARL17B	-0.661	2.067	-2.233	0.028
ENSG00000228729	RP11-211A18.2	-0.632	0.200	-3.323	0.001
ENSG00000228737	AC008781.7	-0.663	-2.010	-2.299	0.024
ENSG00000228740	GABRG3-AS1	-0.576	0.087	-2.751	0.007
ENSG00000228741	SPATA13	-1.028	-2.314	-2.899	0.005
ENSG00000228748	RP13-39P12.3	-0.604	-0.829	-2.834	0.006
ENSG00000228753	EIF4BP2	-0.566	1.021	-2.495	0.015
ENSG00000228810	PABPC1P11	-0.575	2.715	-3.088	0.003
ENSG00000228818	RP11-567G24.1	-0.661	3.493	-1.992	0.050
ENSG00000228824	MIR4500HG	0.321	3.607	2.580	0.012
ENSG00000228937	AC005539.2	-0.729	-0.652	-3.241	0.002

ENSG00000228939	AKT3-IT1	-0.399	1.016	-2.355	0.021
ENSG00000228976	SUMO2P8	-0.563	-0.716	-2.464	0.016
ENSG00000228995	MTND1P9	-0.549	-1.607	-2.337	0.022
ENSG00000228999	AC068490.1	0.699	2.332	2.720	0.008
ENSG00000229020	AKR7A2P1	-0.568	-0.500	-2.148	0.035
ENSG00000229029	CDCA4P1	-0.520	0.057	-3.201	0.002
ENSG00000229036	VDAC1P8	-0.405	2.876	-4.100	0.000
ENSG00000229048	DUTP1	-0.452	-0.752	-2.088	0.040
ENSG00000229052	RP11-386I23.1	-0.502	2.618	-2.958	0.004
ENSG00000229056	AC020571.3	-0.479	0.297	-2.510	0.014
ENSG00000229105	ASTN2-AS1	-0.420	0.891	-2.531	0.013
ENSG00000229110	AC006355.3	-0.685	0.766	-2.645	0.010
ENSG00000229117	RPL41	0.318	4.985	3.187	0.002
ENSG00000229127	AC007038.7	-0.353	3.069	-3.019	0.003
ENSG00000229152	ANKRD10-IT1	-0.337	2.468	-2.473	0.015
ENSG00000229154	KCNQ5-AS1	-0.713	1.038	-3.304	0.001
ENSG00000229190	RP11-390B4.3	-0.548	-1.194	-2.029	0.046
ENSG00000229206	RP11-397O4.1	-0.618	2.667	-3.111	0.003
ENSG00000229240	LINC00710	-0.404	3.707	-2.310	0.023
ENSG00000229287	FABP5P4	-1.055	-2.373	-2.502	0.014
ENSG00000229294	RP5-1044H5.1	-0.656	0.926	-3.454	0.001
ENSG00000229312	RP11-470P21.2	-0.584	-0.180	-3.218	0.002
ENSG00000229359	PIN1P1	-0.559	1.180	-2.245	0.027
ENSG00000229427	ANKRD26P4	-0.444	0.058	-2.322	0.023
ENSG00000229447	RP11-490K7.4	-0.546	-0.915	-2.819	0.006
ENSG00000229458	RP11-202D18.2	-0.363	2.058	-2.790	0.007
ENSG00000229502	RP11-52J3.2	-0.769	-0.028	-4.350	0.000
ENSG00000229589	ACVR2B-AS1	-0.347	0.681	-2.558	0.012
ENSG00000229591	RP5-981O7.2	-0.801	-0.097	-4.068	0.000
ENSG00000229622	MTND5P2	-0.683	-1.930	-2.654	0.010
ENSG00000229623	METTL21AP1	-0.478	-0.079	-2.388	0.019
ENSG00000229666	MAST4-AS1	-0.563	0.225	-3.308	0.001
ENSG00000229668	RP11-317F20.2	-0.543	-1.398	-2.225	0.029
ENSG00000229673	LL22NC01-116C6.1	-0.722	-1.818	-2.387	0.019
ENSG00000229688	ISPD-AS1	-0.579	0.713	-3.350	0.001
ENSG00000229694	RP11-305L7.6	-0.517	-1.325	-2.590	0.011
ENSG00000229727	AC013460.1	-0.545	0.923	-2.667	0.009
ENSG00000229739	RP11-295K2.3	-0.212	2.222	-2.202	0.030
ENSG00000229751	RP11-142M10.2	-0.358	0.372	-2.272	0.026
ENSG00000229756	RPL31P20	-0.676	-0.020	-2.824	0.006
ENSG00000229800	ATP8A2P2	-0.393	1.688	-2.681	0.009
ENSG00000229808	RP11-456P18.2	-0.587	0.227	-3.931	0.000
ENSG00000229816	DDX50P1	-0.310	2.368	-2.409	0.018
ENSG00000229865	RPL31P54	-1.063	-2.192	-2.620	0.010
ENSG00000229892	RP4-730D4.1	-0.582	-1.260	-2.222	0.029
ENSG00000229894	RP11-668G10.2	-0.549	1.010	-2.374	0.020
ENSG00000229956	ZRANB2-AS2	-0.228	3.216	-2.044	0.044

ENSG00000229975	LIPT1P1	-0.445	1.950	-2.472	0.016
ENSG00000230001	RP11-70J12.1	-0.855	-0.334	-3.325	0.001
ENSG00000230002	ALMS1-IT1	-0.289	1.350	-1.991	0.050
ENSG00000230042	AK3P3	-0.350	0.832	-2.341	0.022
ENSG00000230046	BIRC6-AS1	-0.506	-0.868	-2.219	0.029
ENSG00000230051	CTA-929C8.8	0.801	0.392	3.265	0.002
ENSG00000230102	LINC02028	-0.826	-0.738	-3.587	0.001
ENSG00000230126	FGF12-AS2	-0.422	0.770	-2.025	0.046
ENSG00000230154	AC018463.4	-0.525	0.416	-2.426	0.017
ENSG00000230169	RPL30P16	-0.666	-1.107	-2.544	0.013
ENSG00000230177	RP5-1112D6.4	-0.286	0.893	-2.087	0.040
ENSG00000230185	C9orf147	-0.510	-0.286	-2.740	0.008
ENSG00000230224	PHBP9	-0.438	0.409	-2.723	0.008
ENSG00000230225	MTND5P14	-0.598	-1.005	-2.291	0.025
ENSG00000230237	AC010975.2	-0.708	-0.264	-3.014	0.003
ENSG00000230257	NFE4	-0.790	-2.182	-2.732	0.008
ENSG00000230286	AC013472.4	-0.568	-2.214	-2.018	0.047
ENSG00000230287	RP11-305E17.4	-0.490	-1.307	-2.128	0.036
ENSG00000230300	STARD13-IT1	-0.593	0.853	-3.796	0.000
ENSG00000230303	RP11-213G2.2	-0.610	0.069	-3.458	0.001
ENSG00000230314	ELOVL2-AS1	0.467	-1.174	2.043	0.044
ENSG00000230322	RP3-323N1.2	-0.482	2.048	-3.082	0.003
ENSG00000230370	RPL23AP52	-0.640	-1.445	-2.791	0.007
ENSG00000230393	AC092667.2	0.485	-0.647	2.219	0.029
ENSG00000230417	LINC00856	-0.472	2.469	-3.779	0.000
ENSG00000230437	RP11-102J14.1	-0.679	-2.519	-2.043	0.044
ENSG00000230453	ANKRD18B	-0.332	4.069	-2.486	0.015
ENSG00000230470	GS1-115G20.1	-0.564	0.037	-3.007	0.004
ENSG00000230482	ATP5G2P3	-0.560	-0.708	-2.541	0.013
ENSG00000230510	PPP5D1	-0.202	1.697	-2.333	0.022
ENSG00000230530	LIMD1-AS1	-0.696	-2.294	-2.130	0.036
ENSG00000230537	RP11-305L7.1	-0.565	-2.263	-2.052	0.043
ENSG00000230551	CTB-89H12.4	-0.334	4.784	-2.720	0.008
ENSG00000230552	AC092162.1	-0.537	0.218	-3.332	0.001
ENSG00000230585	PHBP12	-0.371	-0.080	-2.397	0.019
ENSG00000230612	AC004237.1	-0.471	1.205	-2.434	0.017
ENSG00000230676	RP11-65J3.3	0.829	-2.365	2.668	0.009
ENSG00000230731	RP11-478K15.6	-0.644	-1.216	-2.248	0.027
ENSG00000230732	AC127904.2	-0.433	1.532	-2.473	0.015
ENSG00000230736	RP1-149A16.3	-0.866	-1.911	-3.361	0.001
ENSG00000230737	AC106870.1	-0.678	-2.148	-2.473	0.015
ENSG00000230777	RP11-318C24.1	-0.584	-0.951	-2.074	0.041
ENSG00000230782	RP11-508N12.2	-0.562	0.181	-3.215	0.002
ENSG00000230783	AC009961.2	-0.639	-1.035	-3.488	0.001
ENSG00000230789	ARHGAP26-IT1	-0.729	0.642	-3.888	0.000
ENSG00000230815	RP11-49O14.3	-0.582	-1.484	-2.331	0.022
ENSG00000230826	RP11-417B4.2	-0.577	0.362	-4.148	0.000

ENSG00000230828	RP11-492I2.1	-0.851	-0.440	-3.391	0.001
ENSG00000230849	GOT2P2	-0.631	0.724	-2.332	0.022
ENSG00000230894	RP11-67K19.3	-0.425	2.355	-3.408	0.001
ENSG00000230910	RP3-525N10.2	-0.411	5.445	-3.610	0.001
ENSG00000230911	PPIHP1	-0.404	0.348	-2.219	0.029
ENSG00000230921	HAO2-IT1	-0.721	-2.146	-2.213	0.030
ENSG00000230928	RP11-34A14.3	-0.511	-1.643	-2.263	0.026
ENSG00000230935	RPS3P1	-0.582	-1.982	-2.135	0.036
ENSG00000230946	HNRNPA1P68	-0.715	0.447	-3.001	0.004
ENSG00000230989	HSBP1	0.364	6.296	3.277	0.002
ENSG00000231006	RPL7P32	-0.589	0.952	-2.309	0.023
ENSG00000231010	RP6-109B7.2	-0.378	0.040	-2.089	0.040
ENSG00000231063	AC006994.1	-0.827	-0.476	-4.265	0.000
ENSG00000231079	AC105402.4	-0.459	2.577	-2.966	0.004
ENSG00000231084	CTA-253N17.1	-0.482	-1.553	-2.153	0.034
ENSG00000231104	RP11-354M20.3	-0.297	1.191	-2.119	0.037
ENSG00000231125	AF129075.5	-0.376	-0.119	-2.257	0.027
ENSG00000231132	RP11-40C11.2	-0.672	-0.243	-3.391	0.001
ENSG00000231181	RP13-15M17.1	-0.568	-1.445	-2.275	0.026
ENSG00000231222	ARF4P4	-0.756	-2.499	-2.286	0.025
ENSG00000231252	RP11-436K8.1	-0.489	1.922	-2.098	0.039
ENSG00000231322	RPL13AP17	-0.335	2.029	-2.167	0.033
ENSG00000231329	RP1-225E12.2	-0.245	0.973	-2.106	0.038
ENSG00000231333	RPL34P6	-0.502	-0.140	-2.015	0.047
ENSG00000231334	AC104781.1	-0.576	1.173	-2.849	0.006
ENSG00000231336	AC017006.3	-0.469	-1.448	-2.280	0.025
ENSG00000231345	BEND3P1	-0.910	-1.187	-2.386	0.019
ENSG00000231355	AP000302.58	-0.910	-2.031	-3.038	0.003
ENSG00000231365	RP11-418J17.1	-0.285	2.845	-2.291	0.025
ENSG00000231378	NDUFAF4P4	-0.639	-0.260	-2.666	0.009
ENSG00000231383	FGF12-AS1	-0.493	1.908	-2.919	0.005
ENSG00000231395	AC124914.3	-0.622	-0.825	-2.784	0.007
ENSG00000231401	AC023481.1	-0.630	-0.144	-3.154	0.002
ENSG00000231496	RP1-251M9.2	-0.490	0.580	-2.546	0.013
ENSG00000231500	RPS18	0.719	5.027	2.491	0.015
ENSG00000231519	AC007285.7	-0.635	-1.453	-2.361	0.021
ENSG00000231538	DPP10-AS3	-0.552	1.404	-3.095	0.003
ENSG00000231551	RP11-495P10.1	-0.343	3.236	-2.942	0.004
ENSG00000231564	EIF4A1P11	-0.625	-1.913	-2.400	0.019
ENSG00000231606	RP11-344F13.1	-0.588	-0.135	-2.542	0.013
ENSG00000231615	RP11-296O14.2	-0.415	2.649	-3.004	0.004
ENSG00000231616	RP11-575L7.4	-0.498	0.443	-2.956	0.004
ENSG00000231628	RP3-355L5.4	-0.355	0.226	-2.102	0.039
ENSG00000231638	AC011738.4	-0.530	2.102	-3.287	0.001
ENSG00000231672	DIRC3	-0.250	2.710	-2.362	0.021
ENSG00000231697	NANOGP5	-0.448	0.446	-2.175	0.033
ENSG00000231699	AC020550.7	-0.865	-2.179	-2.147	0.035

ENSG00000231721	LINC-PINT	-0.355	5.889	-3.707	0.000
ENSG00000231726	HMGN2P38	-0.632	-0.692	-2.756	0.007
ENSG00000231731	AC010976.2	-0.311	2.329	-2.235	0.028
ENSG00000231760	RP11-350J20.5	-0.439	-0.754	-2.302	0.024
ENSG00000231829	RP11-310E22.5	-0.478	-0.867	-2.047	0.044
ENSG00000231845	HMGB3P14	-0.636	-0.488	-2.969	0.004
ENSG00000231858	AC067945.4	-0.456	-0.125	-2.509	0.014
ENSG00000231887	PRH1	-0.206	2.912	-2.106	0.038
ENSG00000231889	TRAF3IP2-AS1	-0.247	3.664	-3.502	0.001
ENSG00000231905	SETP10	-0.571	-1.162	-2.444	0.017
ENSG00000231965	AF131215.1	-0.516	0.546	-2.870	0.005
ENSG00000231981	RPL7L1P12	-0.471	-0.774	-2.203	0.030
ENSG00000231985	RP11-82L20.1	-0.468	-0.102	-2.197	0.031
ENSG00000232006	AC005537.2	-0.281	0.461	-2.029	0.046
ENSG00000232027	RP11-275F13.3	-0.681	-0.166	-3.587	0.001
ENSG00000232036	GS1-115G20.2	-0.451	-0.138	-2.328	0.022
ENSG00000232043	RP4-530I15.9	-0.423	1.492	-2.767	0.007
ENSG00000232059	RP11-173A6.2	-0.607	-1.548	-2.224	0.029
ENSG00000232098	CTD-2619J13.14	-0.316	2.242	-2.268	0.026
ENSG00000232104	RFX3-AS1	-0.309	1.982	-2.049	0.044
ENSG00000232131	NCOA7-AS1	-0.437	-0.965	-2.140	0.035
ENSG00000232136	DUXAP7	-0.789	-1.623	-3.512	0.001
ENSG00000232184	RP11-370K11.1	-0.427	0.327	-2.334	0.022
ENSG00000232196	MTRNR2L4	-0.758	-1.140	-2.736	0.008
ENSG00000232228	RP11-77O7.1	-0.449	-1.131	-2.001	0.049
ENSG00000232277	AC104651.3	-0.712	-1.814	-2.464	0.016
ENSG00000232284	GNG12-AS1	-0.295	1.784	-2.282	0.025
ENSG00000232292	ALDH7A1P2	-0.763	-0.736	-3.475	0.001
ENSG00000232295	RP11-154D6.1	-0.481	0.997	-3.088	0.003
ENSG00000232303	DFFBP1	-0.511	-0.923	-2.557	0.012
ENSG00000232311	RP1-249I4.2	-0.420	-0.848	-2.326	0.022
ENSG00000232342	RP11-46O21.2	-0.474	-0.204	-2.797	0.006
ENSG00000232344	AC087163.2	-0.363	-0.345	-1.991	0.050
ENSG00000232388	LINC00493	0.224	2.216	2.062	0.042
ENSG00000232411	AC009495.3	-0.689	-2.055	-2.207	0.030
ENSG00000232422	KNOP1P4	-0.405	1.686	-2.015	0.047
ENSG00000232439	RPL18AP7	-0.506	0.939	-3.769	0.000
ENSG00000232450	RP4-730K3.3	-0.494	-0.551	-2.207	0.030
ENSG00000232455	LARS2-AS1	-0.685	-2.004	-2.613	0.011
ENSG00000232466	RP11-337A23.5	-0.517	-1.091	-2.065	0.042
ENSG00000232486	RP11-18B3.2	-0.551	0.714	-3.331	0.001
ENSG00000232490	OSBPL10-AS1	-0.471	-0.193	-2.346	0.021
ENSG00000232493	RPL12P11	-0.672	-1.553	-2.875	0.005
ENSG00000232514	RP11-329A14.2	-0.819	-1.642	-2.947	0.004
ENSG00000232528	RP4-673D20.3	-0.575	2.440	-3.330	0.001
ENSG00000232546	RP11-458F8.1	-0.494	-2.021	-2.100	0.039
ENSG00000232568	RPL23AP35	-0.747	-1.335	-3.283	0.002

ENSG00000232578	CTC-303L1.1	-0.622	1.465	-2.986	0.004
ENSG00000232587	EEF1A1P3	-0.461	1.209	-3.367	0.001
ENSG00000232591	RP5-1031D4.2	1.214	-3.050	2.238	0.028
ENSG00000232608	TIMM9P2	-0.402	0.146	-2.182	0.032
ENSG00000232682	RP11-388P9.2	-0.469	0.756	-2.399	0.019
ENSG00000232693	AC012370.2	-0.836	0.034	-3.972	0.000
ENSG00000232696	AC017006.2	-0.678	-0.914	-2.443	0.017
ENSG00000232712	KIZ-AS1	-0.366	2.234	-2.401	0.019
ENSG00000232729	AC083884.8	-0.445	2.595	-2.867	0.005
ENSG00000232739	RP11-25G10.2	-0.484	0.696	-2.751	0.007
ENSG00000232754	RP1-85F18.6	-0.532	-2.169	-2.049	0.044
ENSG00000232778	RPL23AP50	-0.740	-1.406	-3.076	0.003
ENSG00000232780	GAPDHP74	-0.580	0.111	-2.983	0.004
ENSG00000232821	AC003986.6	0.918	-2.412	2.195	0.031
ENSG00000232872	CTAGE3P	-0.312	1.409	-2.200	0.031
ENSG00000232875	HMGN2P35	-0.881	-1.531	-2.959	0.004
ENSG00000232876	CTA-212D2.2	0.972	-1.535	3.180	0.002
ENSG00000232885	GPC5-AS2	-0.747	-0.534	-2.583	0.012
ENSG00000232912	RP5-1115A15.1	-0.634	0.167	-3.403	0.001
ENSG00000232913	PLCE1-AS2	-0.439	-1.179	-2.017	0.047
ENSG00000232953	HSPA8P18	-0.710	-0.715	-2.086	0.040
ENSG00000232956	SNHG15	0.329	1.384	2.730	0.008
ENSG00000232978	RP11-146N23.4	-0.451	-1.153	-2.152	0.034
ENSG00000232995	RGS5	-0.383	1.140	-2.608	0.011
ENSG00000233006	AC034220.3	-0.254	2.886	-2.920	0.005
ENSG00000233008	LINC01725	-0.602	1.400	-3.477	0.001
ENSG00000233009	NALCN-AS1	-0.350	0.077	-2.068	0.042
ENSG00000233012	HDAC1P2	-0.364	2.496	-2.265	0.026
ENSG00000233016	SNHG7	0.562	2.118	3.690	0.000
ENSG00000233021	RP11-490E15.2	-0.533	-0.508	-2.425	0.018
ENSG00000233026	MTCO1P5	-0.655	-1.411	-2.594	0.011
ENSG00000233029	RP11-439A17.9	-0.692	-0.665	-2.238	0.028
ENSG00000233072	RPS15AP6	-0.452	-0.038	-2.104	0.038
ENSG00000233107	SUCLA2P2	-0.773	-1.080	-2.443	0.017
ENSG00000233110	RP11-301L8.2	-0.517	1.032	-2.748	0.007
ENSG00000233123	LINC01007	0.416	4.145	2.163	0.033
ENSG00000233143	DIRC3-AS1	-0.336	0.391	-2.188	0.032
ENSG00000233148	SYF2P2	-0.669	-1.779	-2.101	0.039
ENSG00000233167	EEF1A1P26	-0.524	0.134	-2.533	0.013
ENSG00000233216	RP11-414C16.1	-0.768	-2.902	-2.506	0.014
ENSG00000233237	LINC00472	-0.349	3.037	-2.244	0.028
ENSG00000233264	AC006042.8	-0.457	3.068	-3.120	0.003
ENSG00000233321	RP11-482E14.1	-0.584	0.900	-3.008	0.003
ENSG00000233340	RP11-25C19.3	-0.463	-0.154	-2.995	0.004
ENSG00000233355	CHRM3-AS2	-0.482	3.526	-3.039	0.003
ENSG00000233384	RP11-100E13.1	0.335	0.611	2.274	0.026
ENSG00000233416	AC012065.5	-0.428	0.735	-2.883	0.005

ENSG00000233436	BTBD18	-0.399	1.757	-3.078	0.003
ENSG00000233524	RANP8	-0.517	-1.420	-2.175	0.032
ENSG00000233532	LINC00460	0.546	0.245	2.483	0.015
ENSG00000233559	AC016831.7	-0.495	3.244	-3.028	0.003
ENSG00000233562	RP11-548N1.1	-1.045	-1.731	-3.350	0.001
ENSG00000233578	EIF4EP1	-0.522	-0.604	-2.218	0.029
ENSG00000233609	RP11-62H7.2	-0.574	-0.639	-2.833	0.006
ENSG00000233613	DCUN1D2-AS	-0.441	-0.128	-2.882	0.005
ENSG00000233615	HNRNPA1P42	-0.676	1.240	-3.011	0.003
ENSG00000233621	LINC01137	0.461	-0.939	2.131	0.036
ENSG00000233635	AC037445.1	-0.509	-0.386	-2.485	0.015
ENSG00000233647	NENFP1	-0.509	-1.606	-1.998	0.049
ENSG00000233690	EBAG9P1	-0.380	-0.173	-2.078	0.041
ENSG00000233693	RP11-506O24.1	-0.552	1.217	-3.220	0.002
ENSG00000233716	AC074367.1	-0.439	0.761	-2.174	0.033
ENSG00000233729	AC016909.1	-0.513	0.154	-2.053	0.043
ENSG00000233735	RP11-567G24.3	-0.618	0.133	-3.759	0.000
ENSG00000233756	DSCAM-IT1	-0.443	1.803	-2.269	0.026
ENSG00000233783	AP001442.2	-0.426	3.189	-2.718	0.008
ENSG00000233821	ENOX1-AS1	-0.369	2.520	-2.394	0.019
ENSG00000233828	LINC01949	-0.695	-2.272	-2.169	0.033
ENSG00000233877	RP11-182I10.2	-0.556	-0.988	-2.419	0.018
ENSG00000233892	PAIP1P1	-0.571	-0.746	-2.461	0.016
ENSG00000233900	RPL17P35	-0.752	-1.008	-3.050	0.003
ENSG00000233926	RP11-154D17.1	-0.977	-2.593	-3.013	0.003
ENSG00000233927	RPS28	0.794	2.459	2.456	0.016
ENSG00000233994	GDI2P2	-0.580	3.597	-2.364	0.020
ENSG00000233998	SETP5	-0.892	-2.377	-2.385	0.019
ENSG00000234040	RP11-254B13.4	-0.553	-1.503	-2.347	0.021
ENSG00000234065	MTND4P26	-0.434	-0.686	-2.111	0.038
ENSG00000234073	AC011816.1	-0.437	-0.358	-2.005	0.048
ENSG00000234084	RP3-388E23.2	-0.599	2.313	-3.714	0.000
ENSG00000234091	RP11-353M9.1	-0.406	-0.702	-2.024	0.046
ENSG00000234136	AC055764.1	-0.542	-1.096	-2.260	0.026
ENSG00000234144	COX6CP13	-0.826	-0.934	-3.683	0.000
ENSG00000234149	RP11-77G23.2	-0.665	-1.723	-2.599	0.011
ENSG00000234159	RBPMSLP	-0.702	-1.832	-2.592	0.011
ENSG00000234193	AC097461.4	-0.637	-0.838	-2.775	0.007
ENSG00000234197	ETV5-AS1	-0.540	0.424	-2.108	0.038
ENSG00000234263	RP3-325F22.3	-0.416	1.085	-2.431	0.017
ENSG00000234323	LINC01505	-0.392	0.288	-2.281	0.025
ENSG00000234324	RPL9P2	-0.985	-1.705	-3.105	0.003
ENSG00000234358	AC003080.4	-0.507	0.929	-3.591	0.001
ENSG00000234383	CTBP2P8	-0.412	1.186	-2.783	0.007
ENSG00000234423	LINC01250	-0.312	2.572	-2.676	0.009
ENSG00000234445	FGF14-AS1	-0.476	2.471	-3.398	0.001
ENSG00000234456	MAGI2-AS3	-0.350	6.657	-4.362	0.000

ENSG00000234459	AC002064.4	-0.421	0.442	-2.809	0.006
ENSG00000234473	ZNF101P2	-0.326	1.098	-2.036	0.045
ENSG00000234497	ERICH3-AS1	-0.404	0.621	-2.755	0.007
ENSG00000234509	AP000253.1	-0.454	-1.287	-2.172	0.033
ENSG00000234562	TPMTP2	-0.944	-1.910	-3.105	0.003
ENSG00000234584	AC019186.1	-0.436	-0.196	-3.085	0.003
ENSG00000234588	FABP5P14	-0.873	-1.908	-2.444	0.017
ENSG00000234593	RP4-704D23.1	-0.556	1.812	-2.907	0.005
ENSG00000234604	RP1-206D15.5	-0.631	-1.404	-2.528	0.013
ENSG00000234608	MAPKAPK5-AS1	0.346	3.477	3.425	0.001
ENSG00000234650	PCCA-AS1	-0.429	-0.120	-2.262	0.026
ENSG00000234661	CHL1-AS1	-0.282	1.495	-2.058	0.043
ENSG00000234665	RP11-262H14.3	-1.021	0.074	-2.271	0.026
ENSG00000234678	RP11-465N4.4	-0.424	0.581	-2.649	0.010
ENSG00000234683	RP5-837I24.5	-0.845	-1.461	-4.052	0.000
ENSG00000234684	SDCBP2-AS1	-0.399	3.001	-3.495	0.001
ENSG00000234685	NUS1P2	0.983	-2.307	3.306	0.001
ENSG00000234718	AC007161.5	-0.499	-0.145	-2.790	0.007
ENSG00000234722	LINC01287	-0.719	-1.214	-3.183	0.002
ENSG00000234740	RP11-272P10.2	-0.320	1.131	-2.815	0.006
ENSG00000234741	GAS5	0.336	4.255	2.371	0.020
ENSG00000234751	AP002381.2	-0.502	-1.552	-2.002	0.049
ENSG00000234758	AC034228.4	-0.473	-1.093	-2.052	0.043
ENSG00000234784	RP11-182I10.1	-0.715	-2.095	-2.278	0.025
ENSG00000234818	AC092687.5	-0.647	-1.914	-2.807	0.006
ENSG00000234840	LINC01239	-0.662	-1.571	-2.745	0.007
ENSG00000234841	RP11-119H12.4	-0.464	2.793	-3.353	0.001
ENSG00000234881	PIGFP2	-0.538	-0.259	-2.674	0.009
ENSG00000234883	MIR155HG	-0.523	1.124	-3.472	0.001
ENSG00000234899	SOX9-AS1	-0.254	2.181	-2.287	0.025
ENSG00000234911	TEX21P	-0.323	0.267	-2.007	0.048
ENSG00000234937	RP11-101O6.2	-0.710	-1.128	-2.339	0.022
ENSG00000234942	GRID1-AS1	-0.373	0.778	-2.238	0.028
ENSG00000234943	AC092839.4	-0.444	0.364	-2.698	0.008
ENSG00000234953	RP5-837I24.2	-0.740	-0.664	-3.738	0.000
ENSG00000234982	RP11-280O24.3	-0.709	-1.754	-2.708	0.008
ENSG00000234995	AC013429.5	-0.899	-1.977	-3.062	0.003
ENSG00000234999	SNRPCP19	-0.778	-2.580	-2.235	0.028
ENSG00000235008	RP3-497J21.1	-0.374	1.905	-2.819	0.006
ENSG00000235016	SEMA3F-AS1	-0.374	0.596	-2.690	0.009
ENSG00000235020	RP11-109I13.2	-0.480	1.435	-2.885	0.005
ENSG00000235026	DPP10-AS1	0.195	3.186	2.491	0.015
ENSG00000235045	RPL7P8	-0.861	-3.319	-2.119	0.037
ENSG00000235120	BSN-AS1	-0.922	-2.002	-2.786	0.007
ENSG00000235123	DSCAM-AS1	-0.491	0.232	-2.195	0.031
ENSG00000235129	FABP7P2	-0.673	-1.546	-2.170	0.033
ENSG00000235141	COX6CP17	-0.472	-1.593	-2.059	0.043

ENSG00000235149	RP11-45I20.1	-0.611	-1.360	-2.996	0.004
ENSG00000235183	RP11-613C6.4	-0.575	-0.177	-2.265	0.026
ENSG00000235194	PPP1R3E	-0.224	4.530	-2.532	0.013
ENSG00000235248	OR13C1P	-0.433	0.226	-2.094	0.039
ENSG00000235253	AC010240.2	-0.558	0.740	-3.094	0.003
ENSG00000235258	NDUFB4P6	-0.515	-0.833	-2.294	0.024
ENSG00000235272	FAM103A2P	-0.598	1.817	-2.278	0.025
ENSG00000235279	RP11-539E19.2	-0.706	-1.017	-2.970	0.004
ENSG00000235286	AP000925.2	-0.495	-0.309	-2.163	0.033
ENSG00000235308	RP11-82K18.2	-1.154	-2.521	-3.470	0.001
ENSG00000235310	GXYLT1P6	-0.438	2.420	-2.067	0.042
ENSG00000235335	AC016723.4	-0.682	-1.068	-2.505	0.014
ENSG00000235337	AC114765.2	-0.874	-0.298	-4.283	0.000
ENSG00000235347	RP1-37M3.8	-0.493	0.301	-2.553	0.013
ENSG00000235369	RPL36AP15	-0.409	-0.972	-2.078	0.041
ENSG00000235376	RPEL1	-0.515	1.125	-2.077	0.041
ENSG00000235410	RP11-397C18.2	-0.660	0.898	-2.795	0.006
ENSG00000235429	AC083875.2	-0.658	-2.308	-2.039	0.045
ENSG00000235436	DPY19L2P4	-0.305	2.088	-2.094	0.039
ENSG00000235470	NEURL1-AS1	-0.513	-1.410	-2.222	0.029
ENSG00000235481	UBE2R2-AS1	-0.436	-1.286	-1.994	0.049
ENSG00000235489	DBF4P1	-0.509	0.810	-2.220	0.029
ENSG00000235518	AC011196.3	-0.442	0.923	-2.742	0.007
ENSG00000235522	AC009505.2	-0.423	0.138	-2.488	0.015
ENSG00000235530	AC087294.2	-0.517	0.361	-3.287	0.001
ENSG00000235593	RBMS3-AS1	-0.744	-2.203	-2.538	0.013
ENSG00000235597	LINC01102	0.253	3.108	2.064	0.042
ENSG00000235609	AF127936.9	0.278	3.008	2.724	0.008
ENSG00000235613	NSRP1P1	-0.405	2.467	-2.076	0.041
ENSG00000235619	RPL36AP33	-0.508	-0.456	-2.313	0.023
ENSG00000235641	LINC00484	-0.416	-0.595	-2.169	0.033
ENSG00000235661	MIR670HG	-0.980	-2.621	-2.325	0.023
ENSG00000235674	LDHAP2	-0.550	1.222	-2.263	0.026
ENSG00000235677	NPM1P26	-0.390	0.942	-2.134	0.036
ENSG00000235701	PCBP2P1	-0.427	-0.432	-2.158	0.034
ENSG00000235716	KRT18P46	-0.560	-0.812	-2.510	0.014
ENSG00000235717	DPP10-AS2	-0.794	0.938	-3.366	0.001
ENSG00000235731	LINC02250	-0.539	0.621	-3.010	0.003
ENSG00000235772	AP001625.6	-0.596	-0.527	-2.607	0.011
ENSG00000235784	HNRNPA1P29	-0.629	-0.985	-2.239	0.028
ENSG00000235829	TBCAP2	-0.503	0.736	-2.495	0.015
ENSG00000235837	AC073333.8	-0.449	-0.959	-2.307	0.024
ENSG00000235863	B3GALT4	0.229	1.163	2.143	0.035
ENSG00000235902	RP11-626E13.1	-0.494	0.656	-2.625	0.010
ENSG00000235910	APOA1-AS	-0.434	0.335	-2.320	0.023
ENSG00000235914	MACROD2-AS1	-0.462	1.113	-2.511	0.014
ENSG00000235917	MTCO2P11	-0.733	-1.473	-2.394	0.019

ENSG00000235920	AC073109.2	-0.437	1.332	-2.445	0.017
ENSG00000235947	EGOT	-0.519	0.901	-2.622	0.010
ENSG00000235958	UBOX5-AS1	-0.408	-0.314	-2.301	0.024
ENSG00000235967	COX5BP3	-0.766	-1.602	-2.362	0.021
ENSG00000235978	AC018816.3	-0.477	-0.166	-2.431	0.017
ENSG00000235984	GPC5-AS1	-1.045	-1.438	-4.352	0.000
ENSG00000235993	AC007559.1	-0.610	-1.347	-2.773	0.007
ENSG00000236007	MTCO1P46	-0.430	-0.056	-2.009	0.048
ENSG00000236015	AC011290.5	-0.401	0.750	-2.118	0.037
ENSG00000236027	PATE3	-0.486	-0.305	-2.416	0.018
ENSG00000236031	RP11-269F20.1	-0.429	1.258	-3.051	0.003
ENSG00000236051	MYCBP2-AS1	-0.478	0.237	-3.129	0.002
ENSG00000236054	LL22NC03-104C7.1	-0.580	-0.640	-2.412	0.018
ENSG00000236073	RP11-487E1.2	-0.631	-1.708	-3.152	0.002
ENSG00000236114	RP11-517P14.7	-0.311	1.837	-2.536	0.013
ENSG00000236115	RP11-62C3.6	-0.535	-2.252	-2.276	0.025
ENSG00000236166	RP3-523C21.2	0.337	1.294	2.119	0.037
ENSG00000236184	TCEA1P4	-0.290	1.098	-2.362	0.021
ENSG00000236197	AC002429.5	-0.311	2.856	-2.280	0.025
ENSG00000236200	KDM4A-AS1	-0.492	-0.290	-2.458	0.016
ENSG00000236240	GPC5-IT1	-0.698	0.090	-2.293	0.024
ENSG00000236254	MTND4P14	-0.835	-1.141	-2.739	0.008
ENSG00000236295	AC007563.1	-0.541	1.096	-2.587	0.011
ENSG00000236297	RP11-175P19.2	-0.606	-1.702	-2.161	0.034
ENSG00000236306	LINC01241	-0.685	-1.888	-2.174	0.033
ENSG00000236340	AC000099.1	-0.669	-0.819	-2.348	0.021
ENSG00000236430	KRT8P29	-0.569	-1.419	-2.238	0.028
ENSG00000236434	RP11-296A18.6	-0.833	-1.741	-3.179	0.002
ENSG00000236440	AC117947.1	-0.381	0.605	-2.032	0.045
ENSG00000236451	AC067956.1	-0.242	3.241	-2.746	0.007
ENSG00000236467	KCNMA1-AS1	-0.537	3.858	-3.687	0.000
ENSG00000236474	GCNT1P1	-0.926	0.010	-5.749	0.000
ENSG00000236475	TRIM26BP	-0.550	-1.531	-2.606	0.011
ENSG00000236478	AC012513.4	-0.752	0.048	-3.166	0.002
ENSG00000236508	ATP13A5-AS1	0.566	-2.342	2.028	0.046
ENSG00000236519	LINC01424	-0.675	-1.411	-2.931	0.004
ENSG00000236520	GPC6-AS1	-0.495	-1.030	-2.090	0.040
ENSG00000236549	AC079807.3	-0.501	0.630	-2.692	0.009
ENSG00000236581	STARD13-AS	-0.446	2.823	-2.756	0.007
ENSG00000236583	AP004290.1	-0.573	-0.646	-2.289	0.025
ENSG00000236627	RP11-252P19.1	-0.705	0.166	-2.337	0.022
ENSG00000236676	RP5-837I24.4	-0.481	0.570	-2.554	0.013
ENSG00000236732	AC094019.4	-0.886	-0.949	-3.361	0.001
ENSG00000236758	MTUS2-AS2	-0.684	0.566	-3.796	0.000
ENSG00000236772	RP5-1184F4.5	-0.550	-0.265	-2.701	0.008
ENSG00000236778	INTS6-AS1	-0.271	1.957	-2.206	0.030
ENSG00000236782	RP11-96L14.7	-0.453	-1.389	-1.997	0.049

ENSG00000236795	AC006004.1	-0.525	-0.059	-2.423	0.018
ENSG00000236804	RPS3AP12	-0.735	-1.945	-2.803	0.006
ENSG00000236816	ANKRD20A7P	-0.715	2.997	-2.883	0.005
ENSG00000236830	CBR3-AS1	-0.257	1.593	-2.157	0.034
ENSG00000236841	AC007750.5	0.437	0.291	2.207	0.030
ENSG00000236860	RPL39P29	-0.380	2.021	-2.678	0.009
ENSG00000236886	AC007563.5	-0.469	-0.886	-2.071	0.042
ENSG00000236892	RP11-482E14.2	-0.633	0.016	-2.967	0.004
ENSG00000236928	RP11-792A8.1	-0.658	-1.853	-2.282	0.025
ENSG00000236939	BAALC-AS2	-0.485	-0.890	-2.372	0.020
ENSG00000236948	RP11-154H17.1	0.751	-1.216	2.477	0.015
ENSG00000236963	LINC01141	0.514	0.921	3.194	0.002
ENSG00000236973	GAPDHP51	-0.580	-2.041	-2.096	0.039
ENSG00000236977	ANKRD44-IT1	-0.404	0.085	-2.205	0.030
ENSG00000236986	RP11-544A12.4	-0.679	0.335	-3.876	0.000
ENSG00000236991	EDRF1-AS1	-0.532	-0.171	-2.767	0.007
ENSG00000236998	RP11-203L2.3	-0.422	-0.073	-2.118	0.037
ENSG00000237001	WASF3-AS1	-0.590	0.453	-3.162	0.002
ENSG00000237009	GLIS3-AS1	-1.026	-2.777	-2.874	0.005
ENSG00000237027	RP11-16C18.3	-0.730	-2.506	-2.228	0.029
ENSG00000237031	AC008067.2	-0.494	1.916	-2.894	0.005
ENSG00000237074	RP11-6J21.2	-0.851	-0.942	-3.511	0.001
ENSG00000237076	RP5-836J3.1	-0.746	-2.319	-2.310	0.023
ENSG00000237088	RP11-73E6.2	-0.555	-0.740	-3.068	0.003
ENSG00000237115	RP1-131F15.2	-0.528	-0.699	-2.011	0.048
ENSG00000237130	MRPS6P2	-0.769	-1.834	-2.847	0.006
ENSG00000237135	DDX10P1	-0.296	1.494	-2.085	0.040
ENSG00000237159	CNTFR-AS1	-0.474	-0.010	-2.086	0.040
ENSG00000237170	RPS7P15	-0.628	-1.115	-2.864	0.005
ENSG00000237172	B3GNT9	-0.307	1.494	-2.106	0.038
ENSG00000237190	CDKN2AIPNL	0.373	4.456	3.252	0.002
ENSG00000237194	SNAI1P1	-1.114	-1.657	-3.006	0.004
ENSG00000237200	ZBTB40-IT1	-0.549	-1.755	-2.324	0.023
ENSG00000237217	AC008074.5	-0.461	-1.005	-2.081	0.041
ENSG00000237249	RP1-102G20.2	-0.769	-0.469	-2.930	0.004
ENSG00000237250	RP11-193H5.1	0.869	-0.926	2.841	0.006
ENSG00000237263	MAPK6PS3	-0.529	1.395	-2.116	0.037
ENSG00000237303	HIGD1AP8	-0.609	-1.600	-2.440	0.017
ENSG00000237310	GS1-124K5.4	0.364	-0.481	2.086	0.040
ENSG00000237321	RP11-374A22.1	-0.595	2.018	-3.118	0.003
ENSG00000237327	UPP2-IT1	-0.400	-1.219	-2.104	0.038
ENSG00000237353	PATE4	-0.458	0.598	-2.475	0.015
ENSG00000237359	RP11-509J21.2	-0.408	0.018	-2.131	0.036
ENSG00000237373	BRWD1-IT1	-0.737	-1.316	-2.714	0.008
ENSG00000237399	PITRM1-AS1	-0.425	1.389	-2.632	0.010
ENSG00000237406	NDUFA9P1	-0.605	2.316	-2.507	0.014
ENSG00000237437	ASS1P12	-0.502	-0.425	-2.481	0.015

ENSG00000237440	ZNF737	0.187	5.567	2.326	0.023
ENSG00000237470	DCLRE1CP1	-0.557	-2.267	-2.084	0.040
ENSG00000237479	AC007557.2	-0.580	-0.536	-2.008	0.048
ENSG00000237491	RP11-206L10.9	-0.554	2.814	-3.179	0.002
ENSG00000237499	RP11-356I2.4	-0.843	-2.341	-2.838	0.006
ENSG00000237522	NONOP2	-0.317	1.031	-2.578	0.012
ENSG00000237593	RP11-317B3.2	-0.709	-0.636	-3.530	0.001
ENSG00000237596	RP13-143G15.4	-0.560	1.919	-3.143	0.002
ENSG00000237633	AC104623.2	-0.474	0.240	-3.066	0.003
ENSG00000237645	RP11-521A24.1	-0.663	-1.513	-2.617	0.011
ENSG00000237646	HLCS-IT1	-0.528	-0.569	-2.783	0.007
ENSG00000237651	C2orf74	0.298	0.577	2.136	0.036
ENSG00000237663	DNAJC19P7	-0.705	-2.505	-2.194	0.031
ENSG00000237667	LINC01115	1.433	0.141	2.179	0.032
ENSG00000237711	MTCO3P11	-0.549	-0.695	-2.316	0.023
ENSG00000237731	RNGTTP1	-0.683	-1.331	-3.055	0.003
ENSG00000237827	RP11-332O19.2	-0.617	0.144	-3.346	0.001
ENSG00000237851	RP1-67K17.4	-0.557	0.014	-2.418	0.018
ENSG00000237872	POU5F1P4	-0.758	-1.760	-2.646	0.010
ENSG00000237883	DGUOK-AS1	-0.365	-0.813	-2.108	0.038
ENSG00000237887	RPL23AP32	-0.347	1.667	-2.386	0.019
ENSG00000237928	NFIA-AS2	-0.711	-1.247	-3.495	0.001
ENSG00000237930	AC007563.4	-0.783	-1.254	-3.126	0.002
ENSG00000237964	AC068042.1	-0.531	-0.832	-2.246	0.027
ENSG00000237986	CELF2-AS2	-0.406	3.766	-2.498	0.014
ENSG00000237993	RP11-159M11.2	-0.550	-0.925	-2.372	0.020
ENSG00000238008	COX6CP10	-0.785	-1.428	-2.894	0.005
ENSG00000238018	AC093110.3	-0.606	2.270	-3.111	0.003
ENSG00000238083	LRRC37A2	-0.756	4.554	-2.853	0.005
ENSG00000238085	RP11-435F13.2	-0.325	-0.698	-2.068	0.042
ENSG00000238090	AC006195.2	-0.697	-2.137	-2.809	0.006
ENSG00000238113	LINC01410	-0.422	3.329	-2.386	0.019
ENSG00000238133	MLK7-AS1	-0.572	-1.556	-2.215	0.030
ENSG00000238171	AC068196.1	-0.865	-2.219	-3.199	0.002
ENSG00000238173	RPL39P6	-1.417	-2.761	-3.464	0.001
ENSG00000238198	RP11-31F15.2	-0.378	1.364	-2.208	0.030
ENSG00000238228	OR7E7P	-0.553	0.728	-2.914	0.005
ENSG00000238230	LINC00391	-0.623	-0.631	-2.035	0.045
ENSG00000238250	ST6GAL2-IT1	-0.612	-0.609	-2.758	0.007
ENSG00000238259	AC067940.1	-0.493	-0.920	-2.132	0.036
ENSG00000238273	AC012360.6	-0.417	0.763	-2.105	0.038
ENSG00000238280	RP11-436D10.3	-0.659	-1.758	-2.276	0.025
ENSG00000238311	snoU13	-0.684	-1.775	-2.433	0.017
ENSG00000238324	RN7SKP198	-0.685	-1.475	-2.238	0.028
ENSG00000238358	RP5-1121E10.2	-1.417	-2.352	-4.031	0.000
ENSG00000238386	RNU7-48P	-0.710	-2.154	-2.671	0.009
ENSG00000238713	Y_RNA	-0.472	-1.699	-2.164	0.033

ENSG00000238741	SCARNA7	0.526	5.380	2.945	0.004
ENSG00000238755	LINC02006	-0.596	-2.493	-2.097	0.039
ENSG00000238777	RNU7-141P	-0.599	-1.775	-2.054	0.043
ENSG00000239002	SCARNA10	0.595	-1.568	2.178	0.032
ENSG00000239151	RNU7-195P	-0.850	-1.973	-2.796	0.006
ENSG00000239211	RN7SL563P	1.252	-3.460	2.154	0.034
ENSG00000239213	NCK1-AS1	-0.387	0.338	-2.616	0.011
ENSG00000239224	RN7SL546P	0.684	-1.298	2.337	0.022
ENSG00000239291	RP5-867C24.1	-0.620	-0.357	-3.083	0.003
ENSG00000239300	RP11-400L8.2	-0.476	-0.217	-3.132	0.002
ENSG00000239344	RP11-771F20.1	-0.455	0.750	-2.396	0.019
ENSG00000239351	NPM1P29	-0.728	0.254	-3.676	0.000
ENSG00000239453	SIDT1-AS1	-0.539	0.457	-2.822	0.006
ENSG00000239466	RN7SL552P	-0.609	-1.585	-2.663	0.009
ENSG00000239498	AC114765.1	-0.295	1.599	-2.403	0.019
ENSG00000239503	MARK2P8	-0.395	-0.757	-2.137	0.036
ENSG00000239519	CADM2-AS1	-0.357	2.686	-2.086	0.040
ENSG00000239524	RPL32P34	0.930	-3.025	2.389	0.019
ENSG00000239570	SETP11	-0.529	1.078	-3.079	0.003
ENSG00000239586	MTND1P16	-0.642	0.263	-2.721	0.008
ENSG00000239595	RN7SL79P	2.098	-1.516	2.980	0.004
ENSG00000239605	C2orf61	-0.628	-2.118	-2.059	0.043
ENSG00000239614	HMGN1P7	-0.455	-1.788	-2.131	0.036
ENSG00000239627	RPL12P20	-0.512	0.906	-3.249	0.002
ENSG00000239636	RP4-728D4.2	-0.369	-0.493	-2.013	0.047
ENSG00000239648	MTND1P3	-0.511	-1.217	-2.071	0.041
ENSG00000239650	GUSBP4	-0.385	-0.699	-2.235	0.028
ENSG00000239653	PSMD6-AS2	-0.430	2.484	-3.229	0.002
ENSG00000239665	RP11-295P9.3	-0.119	5.427	-2.306	0.024
ENSG00000239672	NME1	0.343	3.894	2.950	0.004
ENSG00000239686	RP11-665C16.1	-0.475	-0.138	-2.025	0.046
ENSG00000239789	MRPS17	0.255	2.305	2.186	0.032
ENSG00000239804	RP11-379B18.1	-0.721	-1.738	-2.828	0.006
ENSG00000239821	RN7SL513P	1.331	-3.538	2.336	0.022
ENSG00000239822	RN7SL754P	-0.913	-1.962	-3.326	0.001
ENSG00000239835	RP11-767L7.1	-0.435	0.314	-2.478	0.015
ENSG00000239840	RPL23AP72	-0.668	-2.570	-2.209	0.030
ENSG00000239856	RN7SL225P	-0.610	-2.138	-2.029	0.046
ENSG00000239868	RN7SL34P	1.128	-1.904	2.406	0.018
ENSG00000239877	IGSF11-AS1	-0.533	1.277	-2.658	0.009
ENSG00000239883	PARGP1	-0.231	5.310	-2.804	0.006
ENSG00000239888	RN7SL792P	1.058	-2.387	2.353	0.021
ENSG00000239899	RN7SL674P	0.640	6.747	2.111	0.038
ENSG00000239926	PRDX3P4	-0.485	1.597	-3.042	0.003
ENSG00000239932	RN7SL606P	0.889	-3.136	2.193	0.031
ENSG00000239942	RN7SL394P	1.725	-0.659	2.398	0.019
ENSG00000240024	LINC00888	0.353	3.733	2.600	0.011

ENSG00000240053	LY6G5B	-0.363	0.892	-2.034	0.045
ENSG00000240083	RPS3AP22	-0.420	0.406	-2.314	0.023
ENSG00000240086	RP11-657O9.1	-0.396	0.423	-3.257	0.002
ENSG00000240167	RPS7P7	-0.526	-0.883	-2.195	0.031
ENSG00000240174	RP11-851M3.1	-0.497	0.360	-2.348	0.021
ENSG00000240175	MAGI1-AS1	-0.457	-0.355	-2.271	0.026
ENSG00000240183	RN7SL297P	0.873	-2.620	2.019	0.047
ENSG00000240204	SMKR1	0.364	1.782	2.554	0.013
ENSG00000240225	ZNF542P	0.183	3.872	2.291	0.025
ENSG00000240230	COX19	0.241	3.417	3.203	0.002
ENSG00000240291	RP11-499P20.2	-0.484	1.079	-3.267	0.002
ENSG00000240303	ACAD11	-0.453	-0.118	-2.336	0.022
ENSG00000240317	RN7SL764P	1.022	-3.403	2.373	0.020
ENSG00000240393	RP11-768G7.1	-0.292	3.289	-2.307	0.024
ENSG00000240401	AC012358.8	-0.389	1.990	-2.770	0.007
ENSG00000240418	CTD-2013N17.1	-0.673	-1.765	-2.586	0.011
ENSG00000240436	RP11-95I19.1	-0.867	-1.593	-2.788	0.007
ENSG00000240470	RN7SL346P	1.051	-4.110	2.329	0.022
ENSG00000240474	RN7SL116P	1.026	-0.187	2.464	0.016
ENSG00000240499	RP5-1101C3.1	-0.539	3.049	-2.832	0.006
ENSG00000240520	UOX	0.427	-0.565	2.150	0.035
ENSG00000240622	RPL7P15	-0.685	0.111	-3.687	0.000
ENSG00000240661	RP11-174O3.3	-0.952	-0.400	-2.007	0.048
ENSG00000240692	RN7SL538P	-0.464	-0.179	-2.042	0.044
ENSG00000240751	RP11-553D4.2	-0.526	0.019	-2.302	0.024
ENSG00000240777	RP11-58O15.1	-0.837	-1.413	-2.819	0.006
ENSG00000240793	UBA52P8	-0.472	-0.890	-2.015	0.047
ENSG00000240808	CTD-3236F5.1	-0.762	-0.299	-3.922	0.000
ENSG00000240849	TMEM189	0.189	4.307	2.613	0.011
ENSG00000240857	RDH14	0.356	3.842	3.047	0.003
ENSG00000240861	RP11-572F4.1	-0.818	-2.273	-2.847	0.006
ENSG00000240873	RPS29P22	-0.453	0.126	-2.561	0.012
ENSG00000240922	LSAMP-AS1	-0.376	1.554	-2.353	0.021
ENSG00000240936	RN7SL554P	1.533	-3.692	2.881	0.005
ENSG00000240959	ST13P14	-0.796	-1.102	-3.035	0.003
ENSG00000240997	RN7SL183P	1.458	-3.165	2.224	0.029
ENSG00000241002	RP11-119H12.1	-0.595	-0.972	-2.092	0.040
ENSG00000241074	RN7SL813P	1.607	-3.544	2.887	0.005
ENSG00000241104	CEACAMP10	-0.868	-1.934	-2.677	0.009
ENSG00000241111	PRICKLE2-AS1	-0.607	1.034	-3.551	0.001
ENSG00000241155	ARHGAP31-AS1	-0.476	-1.198	-2.261	0.026
ENSG00000241157	RP11-3K24.1	-0.618	-1.478	-2.345	0.021
ENSG00000241162	RN7SL617P	1.731	-1.340	2.579	0.012
ENSG00000241163	LINC00877	-0.437	0.180	-2.053	0.043
ENSG00000241172	RN7SL70P	1.260	-4.254	2.850	0.006
ENSG00000241211	IQCJ-SCHIP1-AS1	-0.412	0.725	-2.574	0.012
ENSG00000241213	LINC02024	-0.553	3.247	-3.521	0.001

ENSG00000241218	RP11-446H18.1	-0.354	0.809	-2.183	0.032
ENSG00000241258	CRCP	0.140	4.806	2.156	0.034
ENSG00000241288	LINC02614	-0.387	2.983	-2.364	0.020
ENSG00000241362	RPL36AP43	-0.478	-1.158	-2.185	0.032
ENSG00000241391	RN7SL234P	1.530	-2.903	2.767	0.007
ENSG00000241420	RN7SL505P	1.332	-1.165	2.569	0.012
ENSG00000241429	EEF1A1P25	-0.464	0.314	-2.232	0.028
ENSG00000241461	RN7SL182P	0.992	-1.114	2.047	0.044
ENSG00000241468	ATP5J2	0.389	3.850	2.962	0.004
ENSG00000241487	RN7SL586P	1.104	-4.015	2.348	0.021
ENSG00000241535	CBX5P1	-0.602	0.347	-3.032	0.003
ENSG00000241546	RP11-767L7.2	-0.478	-1.444	-2.208	0.030
ENSG00000241563	CORT	0.389	0.667	2.086	0.040
ENSG00000241568	RN7SL338P	-0.711	-0.422	-3.012	0.003
ENSG00000241597	CTD-2007H13.1	-0.656	-0.721	-3.332	0.001
ENSG00000241625	RN7SL18P	1.211	-2.636	2.952	0.004
ENSG00000241634	RP11-381E24.1	-0.640	-0.360	-2.901	0.005
ENSG00000241651	RP11-214O14.1	-0.646	-1.500	-2.221	0.029
ENSG00000241738	ZNF90P1	-0.720	0.355	-3.410	0.001
ENSG00000241739	CTD-2290C23.2	-0.812	-1.251	-3.270	0.002
ENSG00000241756	RN7SL83P	-0.633	-1.458	-2.464	0.016
ENSG00000241772	AC092620.2	-0.699	-0.633	-3.514	0.001
ENSG00000241782	RP11-91P24.1	-0.742	-2.123	-2.414	0.018
ENSG00000241787	MTND4P16	-0.804	0.804	-2.856	0.005
ENSG00000241818	RP11-1000B6.2	-0.487	-0.684	-2.799	0.006
ENSG00000241834	RN7SL149P	1.299	-2.197	2.573	0.012
ENSG00000241837	ATP5O	0.337	4.605	3.011	0.003
ENSG00000241907	RPS20P4	-0.500	-0.404	-2.268	0.026
ENSG00000241913	RP5-1073F15.1	-0.602	-2.202	-2.076	0.041
ENSG00000241935	HOGA1	-0.505	0.779	-2.710	0.008
ENSG00000241962	RP11-111H13.1	-0.344	0.283	-2.613	0.011
ENSG00000241990	PRR34-AS1	-0.333	1.224	-2.519	0.014
ENSG00000241991	RP11-95I19.2	-0.508	-0.784	-2.379	0.020
ENSG00000242028	HYPK	-0.353	1.443	-2.211	0.030
ENSG00000242083	RPL7AP31	-0.672	-0.894	-2.407	0.018
ENSG00000242094	FOXP1-IT1	-0.349	1.518	-2.691	0.009
ENSG00000242103	RP11-10G15.4	-0.471	1.057	-2.558	0.012
ENSG00000242110	AMACR	0.365	2.149	2.123	0.037
ENSG00000242154	RP4-778K6.3	-0.481	-0.181	-3.078	0.003
ENSG00000242170	RN7SL329P	1.495	1.149	2.381	0.020
ENSG00000242173	ARHGDIG	0.284	4.486	2.525	0.013
ENSG00000242175	RN7SL127P	-0.591	-1.760	-2.132	0.036
ENSG00000242214	MTND3P6	-0.887	-1.033	-2.931	0.004
ENSG00000242221	PSG2	-0.777	-1.869	-2.512	0.014
ENSG00000242247	ARFGAP3	0.312	4.793	3.053	0.003
ENSG00000242290	RP11-197K3.1	-0.713	0.667	-3.445	0.001
ENSG00000242318	RP11-810D13.1	-0.651	-0.503	-2.905	0.005

ENSG00000242330	RN7SL683P	-0.550	-1.041	-2.550	0.013
ENSG00000242337	TFP1	-0.351	1.185	-2.535	0.013
ENSG00000242338	BMS1P4	-0.397	0.263	-3.125	0.002
ENSG00000242341	RN7SL646P	0.999	-4.118	2.223	0.029
ENSG00000242353	RPL12P30	-0.570	-1.213	-2.319	0.023
ENSG00000242370	KCNAB1-AS1	-0.457	-0.423	-2.253	0.027
ENSG00000242372	EIF6	0.254	2.731	2.393	0.019
ENSG00000242385	LINC00901	-0.387	2.617	-2.013	0.047
ENSG00000242412	DBIL5P2	-0.552	-0.451	-2.353	0.021
ENSG00000242419	PCDHGC4	-0.189	3.881	-2.020	0.047
ENSG00000242428	C3orf67-AS1	0.413	0.542	2.272	0.026
ENSG00000242485	MRPL20	0.320	5.084	2.831	0.006
ENSG00000242488	RP11-270M14.1	-0.651	-1.137	-2.467	0.016
ENSG00000242525	OR7E100P	-0.831	-1.858	-2.704	0.008
ENSG00000242553	AP001432.14	-0.386	0.190	-2.490	0.015
ENSG00000242593	RP5-921G16.1	-0.400	1.453	-2.748	0.007
ENSG00000242600	MBL1P	-0.735	-0.235	-3.776	0.000
ENSG00000242616	GNG10	1.115	-2.644	3.893	0.000
ENSG00000242661	RPS3AP43	-0.595	-0.823	-2.588	0.011
ENSG00000242689	CNTF	-0.452	-0.048	-3.186	0.002
ENSG00000242759	LINC00882	-0.180	4.603	-2.734	0.008
ENSG00000242768	RP11-556G22.1	-0.473	3.453	-2.376	0.020
ENSG00000242791	RP11-651P23.5	-0.505	0.049	-3.143	0.002
ENSG00000242816	RP11-768G7.3	-0.655	0.219	-3.578	0.001
ENSG00000242836	MTCO3P35	-0.941	-0.185	-2.932	0.004
ENSG00000242880	RP11-190P13.2	-0.516	-0.761	-2.599	0.011
ENSG00000242888	RP11-436M15.1	-0.665	-0.450	-3.126	0.002
ENSG00000242894	RN7SL634P	1.687	-2.710	2.596	0.011
ENSG00000242950	ERVW-1	-0.281	3.188	-2.106	0.038
ENSG00000242958	RP11-662B19.1	-0.606	-1.652	-1.998	0.049
ENSG00000242973	RP11-446F17.3	-0.490	-1.310	-2.039	0.045
ENSG00000242979	RPS15AP18	-0.749	-2.193	-2.074	0.041
ENSG00000242986	RP11-1110J8.1	-0.530	-1.324	-2.214	0.030
ENSG00000242999	RN7SL239P	-0.583	-1.055	-2.926	0.004
ENSG00000243053	RPL31P58	-0.533	-1.105	-2.278	0.025
ENSG00000243069	ARHGEF26-AS1	-0.412	2.981	-2.704	0.008
ENSG00000243137	PSG4	-0.587	-0.189	-2.588	0.011
ENSG00000243147	MRPL33	0.400	3.678	2.885	0.005
ENSG00000243193	CTA-360L10.1	-0.510	1.393	-2.834	0.006
ENSG00000243256	RPL30P14	-0.532	0.005	-2.204	0.030
ENSG00000243319	FGF14-IT1	-0.399	4.666	-2.849	0.006
ENSG00000243368	MCCC1-AS1	-0.413	0.178	-2.190	0.031
ENSG00000243384	RP11-475O23.2	-0.538	-1.229	-2.292	0.024
ENSG00000243402	RP11-473O4.1	-0.414	0.371	-2.418	0.018
ENSG00000243415	RP11-274H2.2	-0.416	-0.420	-2.413	0.018
ENSG00000243562	RN7SL838P	0.834	-1.717	3.044	0.003
ENSG00000243574	RP11-264K23.1	-0.438	-0.726	-2.199	0.031

ENSG00000243609	RPS2P44	-0.542	-0.718	-2.694	0.009
ENSG00000243621	AC003989.3	-0.386	-1.032	-2.045	0.044
ENSG00000243649	CFB	-0.531	-0.327	-3.018	0.003
ENSG00000243658	MTND5P16	-0.584	1.536	-2.977	0.004
ENSG00000243701	DUBR	-0.189	4.247	-2.198	0.031
ENSG00000243704	RN7SL105P	1.524	0.194	2.763	0.007
ENSG00000243715	CACNA2D3-AS1	-0.680	2.298	-3.371	0.001
ENSG00000243753	HLA-L	-0.255	3.473	-2.162	0.034
ENSG00000243770	RN7SL65P	-0.722	-2.160	-2.459	0.016
ENSG00000243799	PEX5L-AS1	-0.383	1.933	-2.280	0.025
ENSG00000243813	RP11-678L1.1	-0.399	1.318	-2.081	0.041
ENSG00000243836	WDR86-AS1	1.026	-1.834	3.333	0.001
ENSG00000243854	RN7SL67P	-0.840	-0.430	-3.738	0.000
ENSG00000243870	RN7SL236P	0.998	-3.683	2.135	0.036
ENSG00000243915	THAP12P2	-0.304	3.032	-2.543	0.013
ENSG00000243960	RP11-552M11.4	-0.443	-0.314	-2.908	0.005
ENSG00000244038	DDOST	0.273	4.316	2.512	0.014
ENSG00000244044	RN7SL735P	-0.557	-0.877	-2.850	0.006
ENSG00000244060	RPS2P41	-0.571	-1.934	-2.035	0.045
ENSG00000244139	RN7SL397P	1.146	-4.057	2.227	0.029
ENSG00000244159	RP11-1070A24.1	-1.107	-1.852	-3.199	0.002
ENSG00000244187	TMEM141	0.279	1.623	2.778	0.007
ENSG00000244194	RN7SL218P	1.517	-0.417	3.022	0.003
ENSG00000244242	IFITM10	0.305	1.303	2.257	0.027
ENSG00000244249	RP11-441M10.1	-0.598	1.559	-3.088	0.003
ENSG00000244264	RN7SL597P	0.910	-2.424	2.240	0.028
ENSG00000244289	RPS3AP35	-0.661	-1.531	-2.215	0.030
ENSG00000244327	RP11-383G6.3	-0.563	-0.928	-2.121	0.037
ENSG00000244357	RN7SL145P	-0.604	-1.389	-2.246	0.027
ENSG00000244378	RPS2P45	-0.734	-0.972	-3.748	0.000
ENSG00000244389	RN7SL242P	1.853	-0.034	2.669	0.009
ENSG00000244451	RPL34P21	-0.677	-0.869	-2.633	0.010
ENSG00000244480	AC005154.7	-0.363	0.918	-2.230	0.028
ENSG00000244490	RWDD4P1	-0.478	0.289	-2.002	0.049
ENSG00000244513	CTD-2013N24.2	-0.292	1.317	-2.484	0.015
ENSG00000244556	ODCP	-0.470	0.710	-3.159	0.002
ENSG00000244568	RN7SL654P	1.244	-3.881	2.466	0.016
ENSG00000244632	RN7SL863P	-0.941	-1.820	-3.506	0.001
ENSG00000244687	UBE2V1	-0.381	3.081	-2.720	0.008
ENSG00000244730	RP13-1056D16.2	-0.483	-0.003	-2.641	0.010
ENSG00000244731	C4A	-0.752	1.294	-2.099	0.039
ENSG00000244733	RP11-506M13.3	-0.404	1.199	-2.181	0.032
ENSG00000244932	RP11-529F4.1	-0.434	0.433	-2.285	0.025
ENSG00000244968	LIFR-AS1	-0.173	2.840	-2.105	0.038
ENSG00000245322	RP11-15B17.1	-0.462	-1.072	-2.068	0.042
ENSG00000245384	AC004053.1	-0.372	1.288	-3.371	0.001
ENSG00000245534	RORA-AS1	-0.620	0.653	-3.634	0.000

ENSG00000245552	RP11-712B9.2	-0.389	1.841	-3.212	0.002
ENSG00000245556	SCAMP1-AS1	0.313	1.710	3.175	0.002
ENSG00000245614	DDX11-AS1	-0.473	-0.689	-1.999	0.049
ENSG00000245667	RP5-940J5.8	-0.665	-1.804	-3.222	0.002
ENSG00000245711	NADK2-AS1	-0.560	0.651	-3.075	0.003
ENSG00000245768	RP11-410D17.2	-0.387	0.043	-2.455	0.016
ENSG00000245832	MIR4300HG	-0.737	-0.313	-2.951	0.004
ENSG00000245910	SNHG6	0.395	3.854	2.356	0.021
ENSG00000245928	RP11-630D6.5	-0.570	-1.815	-2.330	0.022
ENSG00000245958	RP11-33B1.1	-0.360	5.115	-3.198	0.002
ENSG00000246100	LINC00900	-0.314	1.532	-2.820	0.006
ENSG00000246145	RRS1-AS1	-0.558	0.041	-2.203	0.030
ENSG00000246203	RP11-29H23.5	-0.524	-0.011	-2.109	0.038
ENSG00000246223	LINC01550	-0.484	0.465	-3.360	0.001
ENSG00000246250	RP11-613D13.5	-0.717	1.117	-3.757	0.000
ENSG00000246366	LACTB2-AS1	-0.417	-0.170	-2.152	0.034
ENSG00000246422	CTD-2024I7.13	-0.594	0.469	-3.034	0.003
ENSG00000246477	AF131216.6	-0.437	1.962	-3.194	0.002
ENSG00000246851	RP11-544A12.8	-0.673	1.061	-4.030	0.000
ENSG00000246863	RP11-325N19.3	0.750	-2.493	2.144	0.035
ENSG00000246922	UBAP1L	-0.294	4.811	-2.384	0.019
ENSG00000247077	PGAM5	0.179	4.508	2.135	0.036
ENSG00000247137	RP11-727A23.5	-0.284	2.918	-2.050	0.044
ENSG00000247317	RP11-273G15.2	0.228	2.882	2.242	0.028
ENSG00000247345	RP11-266N13.2	-0.810	-0.606	-4.081	0.000
ENSG00000247416	RP11-629G13.1	-0.441	-0.151	-2.425	0.018
ENSG00000247516	MIR4458HG	0.222	3.215	2.138	0.036
ENSG00000247572	CKMT2-AS1	-0.188	3.343	-2.277	0.025
ENSG00000247624	CPEB2-AS1	-0.367	0.906	-2.578	0.012
ENSG00000247679	RP11-1277A3.1	-0.285	1.379	-2.392	0.019
ENSG00000247708	STX18-AS1	-0.263	3.662	-3.998	0.000
ENSG00000247763	RP11-447H19.1	-0.416	0.100	-2.215	0.030
ENSG00000247911	HMGN1P12	-0.854	-1.706	-2.907	0.005
ENSG00000247925	RP3-510L9.1	-0.508	-2.323	-2.275	0.026
ENSG00000247934	RP11-967K21.1	-0.500	0.758	-3.198	0.002
ENSG00000248019	FAM13A-AS1	-0.336	3.142	-3.012	0.003
ENSG00000248050	RP11-422N16.3	-0.338	3.265	-2.946	0.004
ENSG00000248124	RRN3P1	-0.250	3.438	-2.239	0.028
ENSG00000248125	CTB-73N10.1	-0.576	-1.066	-2.289	0.025
ENSG00000248152	RP11-650J17.1	-0.445	1.911	-3.180	0.002
ENSG00000248200	RP11-115A14.1	-0.742	-1.603	-2.495	0.015
ENSG00000248206	RP11-739P1.2	-0.329	0.369	-2.171	0.033
ENSG00000248216	KCTD9P5	-0.641	-0.899	-2.068	0.042
ENSG00000248221	STX18-IT1	-0.555	-1.460	-2.506	0.014
ENSG00000248222	CTB-174D11.1	-0.540	0.884	-2.724	0.008
ENSG00000248245	CTB-49A3.2	-0.685	-0.066	-3.221	0.002
ENSG00000248249	RP11-598O12.1	-0.757	0.684	-2.952	0.004

ENSG00000248254	AC092597.3	-0.561	-1.498	-3.019	0.003
ENSG00000248283	CCNL2P1	-0.530	2.186	-2.970	0.004
ENSG00000248323	LUCAT1	-0.425	1.539	-2.367	0.020
ENSG00000248340	RP11-293A21.2	-1.264	-2.244	-4.043	0.000
ENSG00000248343	RP11-556G22.2	-0.641	2.822	-3.416	0.001
ENSG00000248360	LINC00504	-0.318	0.371	-2.028	0.046
ENSG00000248397	LINC00498	-1.792	-2.481	-4.604	0.000
ENSG00000248415	GAPDHP61	-0.639	1.083	-2.245	0.027
ENSG00000248429	RP11-597D13.9	-0.712	2.301	-4.212	0.000
ENSG00000248458	RP4-598P13.1	-0.494	0.676	-2.364	0.020
ENSG00000248466	RP11-640B6.1	-0.803	-2.391	-2.516	0.014
ENSG00000248483	POU5F2	-0.269	3.329	-2.019	0.047
ENSG00000248485	PCP4L1	0.398	4.303	2.682	0.009
ENSG00000248557	RP11-390G14.1	-1.062	-1.503	-4.292	0.000
ENSG00000248610	HSPA8P4	-0.283	2.392	-2.187	0.032
ENSG00000248637	RP11-485C11.1	-0.983	-1.711	-3.795	0.000
ENSG00000248673	LINC01331	0.482	-0.349	2.123	0.037
ENSG00000248677	LINC02102	-0.591	-0.874	-2.754	0.007
ENSG00000248708	LINC02144	0.671	-2.049	2.130	0.036
ENSG00000248725	RP11-218C23.1	-0.706	-0.547	-2.050	0.044
ENSG00000248734	CTD-2260A17.1	-0.451	-0.017	-2.705	0.008
ENSG00000248779	RP11-53O19.2	-0.351	1.106	-2.110	0.038
ENSG00000248803	RP11-287J9.1	-0.756	-1.824	-2.685	0.009
ENSG00000248810	LINC02432	-1.325	-2.968	-2.650	0.010
ENSG00000248813	RP11-5N11.4	-0.694	-1.855	-2.220	0.029
ENSG00000248816	RP11-18D7.3	-0.475	-0.830	-2.462	0.016
ENSG00000248863	RP11-83A24.1	-0.515	0.392	-2.823	0.006
ENSG00000248878	RP11-5N11.1	-0.591	-1.252	-2.094	0.039
ENSG00000248884	CTC-537E7.3	-0.990	-2.387	-3.014	0.003
ENSG00000248885	RP11-950K24.2	-0.644	0.502	-2.368	0.020
ENSG00000248935	CTD-2254N19.1	-0.650	-0.709	-2.529	0.013
ENSG00000248978	RP11-98H4.1	-0.548	-0.499	-2.360	0.021
ENSG00000248979	LAMTOR3P2	-0.656	-0.997	-2.742	0.007
ENSG00000249028	RP5-951N9.1	-0.657	0.028	-3.823	0.000
ENSG00000249047	COX6B1P5	-0.765	-1.998	-2.286	0.025
ENSG00000249057	MAST4-IT1	-0.488	-0.972	-2.008	0.048
ENSG00000249068	CTC-287O8.1	-0.458	-0.650	-2.019	0.047
ENSG00000249089	AIG1P1	-0.496	-0.914	-2.418	0.018
ENSG00000249098	RP11-39E3.4	-0.659	-0.641	-2.284	0.025
ENSG00000249106	RP11-806K15.1	-0.446	3.617	-3.584	0.001
ENSG00000249112	RP11-43D2.2	-0.397	-0.808	-2.095	0.039
ENSG00000249114	RP11-5N11.3	-0.664	-1.780	-2.173	0.033
ENSG00000249169	CTC-529L17.1	0.445	-1.136	2.152	0.034
ENSG00000249175	CTC-484P3.3	-0.568	0.620	-2.664	0.009
ENSG00000249206	GCNT1P2	-0.899	-1.183	-3.463	0.001
ENSG00000249274	PDLIM1P4	-0.404	-0.442	-2.350	0.021
ENSG00000249278	RP11-688H10.1	-1.086	-2.724	-3.416	0.001

ENSG00000249304	RP11-26P13.2	-0.821	-1.604	-2.759	0.007
ENSG00000249309	RP11-153M7.5	-0.503	-1.299	-2.309	0.023
ENSG00000249348	UGDH-AS1	-0.374	1.205	-3.008	0.003
ENSG00000249363	CTB-78O21.1	-0.572	-1.174	-2.331	0.022
ENSG00000249373	RP11-650J17.2	-0.376	2.140	-2.179	0.032
ENSG00000249379	RP1-27K12.4	-0.419	-1.218	-2.224	0.029
ENSG00000249381	LINC00500	-1.535	-2.535	-4.144	0.000
ENSG00000249436	CTD-2023N9.3	0.416	3.071	2.284	0.025
ENSG00000249506	ZEB2P1	-0.428	0.563	-2.078	0.041
ENSG00000249514	CTD-2266L18.1	-1.150	-2.309	-3.117	0.003
ENSG00000249540	RP11-789L4.1	-0.578	-1.401	-2.623	0.010
ENSG00000249545	CTC-558O19.1	-0.774	-1.858	-2.982	0.004
ENSG00000249558	RCC2P4	-0.735	-1.246	-2.761	0.007
ENSG00000249580	MTATP6P22	-0.993	-0.650	-3.411	0.001
ENSG00000249584	LINC02225	-0.739	-1.198	-2.429	0.017
ENSG00000249588	CTC-537E7.1	-1.231	-2.326	-3.234	0.002
ENSG00000249593	CTB-46B19.2	-0.439	0.924	-2.851	0.006
ENSG00000249635	RP11-710F7.3	-0.498	-1.441	-2.056	0.043
ENSG00000249678	RP11-619J20.1	-0.414	-0.559	-2.206	0.030
ENSG00000249691	RP11-445J14.1	-0.633	-0.820	-2.933	0.004
ENSG00000249717	RP11-44F21.3	-0.495	1.328	-2.410	0.018
ENSG00000249781	LINC02112	-0.740	-1.202	-2.609	0.011
ENSG00000249848	RP11-212F11.1	-0.522	0.091	-2.291	0.025
ENSG00000249859	PVT1	-0.470	1.721	-3.661	0.000
ENSG00000249863	RP11-177C12.1	-0.385	-0.311	-2.354	0.021
ENSG00000249896	LINC02495	0.210	3.952	2.132	0.036
ENSG00000249915	PDCD6	0.286	5.125	3.172	0.002
ENSG00000249934	RP11-466G12.3	-0.510	-1.492	-2.280	0.025
ENSG00000250017	RP11-484L7.2	-0.457	2.318	-2.538	0.013
ENSG00000250027	RP11-563E2.2	-0.339	1.185	-2.463	0.016
ENSG00000250033	SLC7A11-AS1	-0.764	1.816	-3.386	0.001
ENSG00000250045	CBX3P3	-0.635	0.995	-2.854	0.005
ENSG00000250062	RP11-778J15.1	-0.399	1.992	-2.423	0.018
ENSG00000250068	RP11-576C12.1	-0.422	-1.204	-2.378	0.020
ENSG00000250069	CTB-131B5.2	-0.760	-0.202	-3.455	0.001
ENSG00000250091	DNAH10OS	-0.275	3.337	-2.197	0.031
ENSG00000250092	RP11-556G22.3	-0.517	3.405	-3.186	0.002
ENSG00000250106	ANKRD33B-AS1	-1.009	-1.973	-3.322	0.001
ENSG00000250116	RP11-417F21.1	-0.418	0.834	-2.067	0.042
ENSG00000250120	PCDHA10	-0.189	3.881	-2.258	0.027
ENSG00000250130	RP11-616K22.1	-0.389	0.538	-2.016	0.047
ENSG00000250156	LINC02060	-0.453	1.107	-2.291	0.025
ENSG00000250170	RASA2-IT1	-0.869	-0.448	-3.994	0.000
ENSG00000250208	FZD10-AS1	0.599	-0.740	2.229	0.029
ENSG00000250220	RP11-692D12.1	-0.468	0.125	-2.923	0.004
ENSG00000250243	RP11-120A1.1	-0.507	2.357	-2.414	0.018
ENSG00000250244	CTB-49A3.4	-0.720	-1.560	-2.527	0.013

ENSG00000250254	PTTG2	-0.632	-0.195	-2.920	0.005
ENSG00000250280	RP11-305O6.3	-0.555	0.629	-3.000	0.004
ENSG00000250289	VWA8P1	-0.820	-2.098	-2.700	0.008
ENSG00000250306	CTC-498M16.3	-0.651	-1.545	-2.558	0.012
ENSG00000250317	SMIM20	0.258	3.268	2.357	0.021
ENSG00000250326	RP11-284M14.1	-0.382	0.134	-2.100	0.039
ENSG00000250328	MGC32805	-0.696	-1.714	-2.231	0.028
ENSG00000250354	RP11-76G10.1	-0.795	-0.521	-4.462	0.000
ENSG00000250361	GYPB	-0.447	1.228	-2.153	0.034
ENSG00000250371	RP11-22A3.2	-0.538	0.653	-2.779	0.007
ENSG00000250377	CTC-467M3.3	0.437	-1.577	2.109	0.038
ENSG00000250407	CTB-99A3.1	-0.444	-0.709	-2.321	0.023
ENSG00000250409	CTB-3M24.3	-0.877	-1.279	-3.153	0.002
ENSG00000250410	RP11-714G18.1	-0.512	-0.269	-2.882	0.005
ENSG00000250420	AACSP1	-0.513	-1.121	-2.451	0.016
ENSG00000250422	RP11-484L7.1	-0.541	2.463	-2.609	0.011
ENSG00000250433	CLSTN2-AS1	-0.627	1.137	-2.503	0.014
ENSG00000250490	LINC02145	-0.592	-0.365	-2.585	0.012
ENSG00000250497	AC007126.1	-0.862	-1.542	-2.819	0.006
ENSG00000250500	RP11-119H12.3	-0.901	-0.597	-3.525	0.001
ENSG00000250508	RP11-757G1.6	-0.513	-0.383	-2.600	0.011
ENSG00000250539	KRT8P33	-0.323	0.269	-2.002	0.049
ENSG00000250541	RP11-302F12.10	-0.614	-1.992	-2.042	0.044
ENSG00000250569	NTAN1P2	-0.435	0.869	-2.347	0.021
ENSG00000250582	SMAD1-AS2	-0.811	0.157	-3.757	0.000
ENSG00000250592	RP11-39E3.3	-0.634	-1.213	-2.638	0.010
ENSG00000250602	RP11-517I3.1	-0.489	0.678	-2.401	0.019
ENSG00000250608	RP11-933H2.4	-0.541	2.074	-3.296	0.001
ENSG00000250630	MTCO1P35	-0.769	-2.442	-2.383	0.019
ENSG00000250634	LINC01182	-0.997	-1.654	-3.522	0.001
ENSG00000250651	PABPC1P7	-0.660	-0.815	-2.668	0.009
ENSG00000250656	ST3GAL1P1	-0.663	-1.316	-2.826	0.006
ENSG00000250670	AC004063.1	-0.576	-2.146	-2.318	0.023
ENSG00000250726	RP11-616K6.1	-1.049	-2.246	-2.932	0.004
ENSG00000250761	RP11-711G10.1	-0.621	0.028	-3.732	0.000
ENSG00000250853	RNF138P1	-0.518	0.985	-2.491	0.015
ENSG00000250860	CTD-2265D6.2	-0.645	-0.993	-2.479	0.015
ENSG00000250869	RP11-453N18.1	-0.517	-0.179	-2.211	0.030
ENSG00000250878	METTL21EP	-0.400	1.755	-2.301	0.024
ENSG00000250892	RP11-1365D11.1	-0.731	-1.870	-2.222	0.029
ENSG00000250903	GMDS-AS1	-0.262	4.008	-3.243	0.002
ENSG00000250910	AC097467.2	-0.377	1.602	-2.622	0.010
ENSG00000250930	LNX1-AS1	-0.449	1.525	-2.745	0.007
ENSG00000250933	GAPDHP66	-0.394	0.448	-2.290	0.025
ENSG00000250938	RP11-679C8.2	-0.361	0.799	-2.225	0.029
ENSG00000250979	RP11-656G20.1	-0.387	1.215	-2.797	0.006
ENSG00000250986	LINC02600	0.478	1.382	3.864	0.000

ENSG00000251001	CTD-2193P3.1	-0.678	-1.372	-2.280	0.025
ENSG00000251008	ORAOV1P1	-0.874	-2.428	-2.349	0.021
ENSG00000251011	TMEM108-AS1	-0.500	3.017	-3.052	0.003
ENSG00000251023	RP11-549J18.1	-0.247	1.306	-2.169	0.033
ENSG00000251034	RP11-582J16.4	-0.318	2.907	-2.387	0.019
ENSG00000251073	NUDT19P5	-0.711	-1.046	-2.649	0.010
ENSG00000251076	RP11-526F3.1	-0.589	-1.938	-1.997	0.049
ENSG00000251095	RP11-115D19.1	-0.474	1.129	-2.710	0.008
ENSG00000251128	WWC2-AS1	-0.760	-2.262	-2.754	0.007
ENSG00000251136	RP11-37B2.1	-0.155	5.010	-2.149	0.035
ENSG00000251165	F11-AS1	-0.799	-0.285	-3.265	0.002
ENSG00000251171	RP11-327O17.2	-0.385	-0.203	-2.065	0.042
ENSG00000251209	LINC00923	-0.448	1.996	-2.816	0.006
ENSG00000251248	RP11-223C24.2	-0.758	-1.426	-2.601	0.011
ENSG00000251276	MAGI2-AS1	-0.588	0.179	-2.585	0.012
ENSG00000251287	ALG1L2	-0.767	-1.810	-3.091	0.003
ENSG00000251294	RP11-116O11.1	-0.333	2.691	-2.336	0.022
ENSG00000251299	SLC25A15P3	-0.490	-1.262	-2.033	0.045
ENSG00000251306	NDUFB4P2	-0.457	0.219	-2.348	0.021
ENSG00000251323	RP11-452H21.4	-0.482	-1.082	-2.060	0.043
ENSG00000251348	HSPD1P11	-0.495	1.351	-3.040	0.003
ENSG00000251354	RP11-697N18.1	-0.492	-0.272	-2.396	0.019
ENSG00000251364	CTD-2516F10.2	-0.273	2.567	-2.149	0.035
ENSG00000251372	LINC00499	-0.843	1.374	-2.922	0.004
ENSG00000251402	FAM90A25P	-0.535	0.221	-2.066	0.042
ENSG00000251405	CTB-109A12.1	-0.498	1.265	-3.273	0.002
ENSG00000251442	LINC01094	-0.435	2.269	-2.021	0.047
ENSG00000251455	RP11-164P12.3	-0.468	-0.742	-2.204	0.030
ENSG00000251456	RP11-436H11.5	-0.455	-1.257	-2.116	0.037
ENSG00000251463	FKBP4P1	-0.683	1.325	-2.140	0.035
ENSG00000251520	RP11-46A10.6	-0.425	-0.103	-2.530	0.013
ENSG00000251557	HNRNPKP3	-0.599	-1.201	-2.319	0.023
ENSG00000251562	MALAT1	-0.323	13.629	-2.779	0.007
ENSG00000251596	HADHAP1	-0.487	3.286	-2.564	0.012
ENSG00000251614	HSPA8P19	-0.524	-0.674	-2.183	0.032
ENSG00000251615	RP11-774O3.3	-0.258	2.974	-2.499	0.014
ENSG00000251623	RP11-826N14.4	-0.543	1.674	-3.369	0.001
ENSG00000251637	RP11-119D9.1	-0.643	-0.282	-2.759	0.007
ENSG00000251661	RP11-326C3.11	-0.550	-0.164	-2.343	0.022
ENSG00000251664	PCDHA12	-0.237	0.906	-2.158	0.034
ENSG00000251666	ZNF346-IT1	-0.252	1.182	-2.049	0.044
ENSG00000251739	RNU6-1053P	-0.719	-2.503	-2.203	0.030
ENSG00000251785	RNA5SP20	-0.517	-1.532	-2.406	0.018
ENSG00000251867	RP11-48B3.5	-0.341	1.417	-2.902	0.005
ENSG00000251880	RNU7-75P	-0.557	-2.109	-2.312	0.023
ENSG00000251892	RNU7-84P	-0.730	-1.058	-2.971	0.004
ENSG00000251972	RNU6-123P	-0.485	-1.289	-2.066	0.042

ENSG00000251982	RN7SKP43	-0.772	-2.960	-2.057	0.043
ENSG00000252017	RNU6-1194P	-0.707	-1.616	-2.488	0.015
ENSG00000252026	RNU6-1262P	-0.644	-2.203	-2.615	0.011
ENSG00000252080	RNU7-99P	-0.488	-2.250	-2.064	0.042
ENSG00000252130	RNU6-1045P	-0.645	-1.925	-2.244	0.028
ENSG00000252172	RNU6-720P	-0.619	-0.650	-2.799	0.006
ENSG00000252202	Y_RNA	-0.702	-2.166	-1.998	0.049
ENSG00000252326	SNORD116-25	-0.424	-0.529	-2.466	0.016
ENSG00000252355	RN7SKP287	-0.523	-1.454	-2.054	0.043
ENSG00000252396	RN7SKP195	-0.702	-0.199	-3.322	0.001
ENSG00000252414	RNU6-100P	-0.819	-2.164	-2.969	0.004
ENSG00000252452	RNU6-107P	-0.482	-1.183	-2.442	0.017
ENSG00000252498	RNU6-1016P	-0.296	0.893	-2.100	0.039
ENSG00000252549	RNU6-759P	-0.424	-0.351	-2.155	0.034
ENSG00000252578	RNU6-135P	-0.799	-2.280	-2.925	0.004
ENSG00000252598	RNA5SP460	-0.728	-2.190	-2.620	0.010
ENSG00000252643	RNU6-1136P	-0.619	-1.621	-2.334	0.022
ENSG00000252717	RNU6-352P	-0.779	-2.046	-2.536	0.013
ENSG00000252720	RNU6-1258P	-0.517	-0.413	-2.893	0.005
ENSG00000252751	RNU6-143P	-0.567	-0.286	-2.557	0.012
ENSG00000252757	RN7SKP96	-0.614	-0.548	-2.736	0.008
ENSG00000252874	Y_RNA	-0.818	-1.659	-3.083	0.003
ENSG00000252950	RNA5SP490	-0.546	-1.903	-2.144	0.035
ENSG00000252980	RNU6-367P	-0.771	-1.617	-2.615	0.011
ENSG00000253042	SNORA70	-0.417	-0.424	-2.292	0.024
ENSG00000253107	CTD-2544L4.1	-0.963	-1.019	-3.579	0.001
ENSG00000253121	RP11-659E9.4	-0.747	-2.658	-2.227	0.029
ENSG00000253137	RP11-509P12.1	-0.499	1.633	-2.934	0.004
ENSG00000253159	PCDHGA12	-0.338	3.257	-2.468	0.016
ENSG00000253162	RP11-279L11.1	-0.506	-1.121	-2.461	0.016
ENSG00000253167	KIAA0196-AS1	-0.401	-0.584	-2.022	0.046
ENSG00000253175	RP11-267M23.6	-0.654	-2.483	-2.078	0.041
ENSG00000253180	RP11-410L14.1	-0.562	0.369	-2.492	0.015
ENSG00000253194	RP11-351A11.1	-0.386	0.861	-2.165	0.033
ENSG00000253197	CTD-3239E11.2	-0.380	-0.644	-2.023	0.046
ENSG00000253199	LINC02153	-0.812	-0.456	-3.590	0.001
ENSG00000253226	HAUS1P3	-0.633	-0.188	-3.035	0.003
ENSG00000253259	RP11-737F9.2	-0.654	-1.069	-2.924	0.004
ENSG00000253267	DLGAP2-AS1	-0.602	-0.466	-2.385	0.019
ENSG00000253270	RP11-1105O14.1	-0.557	-0.297	-2.802	0.006
ENSG00000253273	RP11-99A14.1	-0.420	-0.135	-2.237	0.028
ENSG00000253284	RP11-282K24.3	-0.395	3.684	-2.675	0.009
ENSG00000253305	PCDHGB6	-0.268	3.079	-2.441	0.017
ENSG00000253320	AZIN1-AS1	-0.207	3.411	-2.622	0.010
ENSG00000253341	RP11-730G20.2	-0.641	3.924	-2.239	0.028
ENSG00000253357	CTB-78F1.2	-0.678	0.000	-3.894	0.000
ENSG00000253372	RP11-44N11.1	-0.516	0.533	-2.500	0.014

ENSG00000253385	KB-1254G8.1	-0.452	-0.341	-2.062	0.042
ENSG00000253420	RP11-642D21.2	-0.457	0.398	-2.231	0.028
ENSG00000253463	HMGB1P19	-0.338	-0.056	-2.219	0.029
ENSG00000253469	CTC-264O10.1	-0.825	-0.756	-3.768	0.000
ENSG00000253485	PCDHGA5	-0.363	2.444	-3.198	0.002
ENSG00000253516	HMGB1P41	-0.533	0.943	-3.827	0.000
ENSG00000253523	RP11-1112C15.2	-0.971	-1.446	-3.767	0.000
ENSG00000253527	CTB-105L4.2	-0.774	-1.796	-2.779	0.007
ENSG00000253537	PCDHGA7	-0.453	2.819	-4.734	0.000
ENSG00000253547	RP11-959F10.5	-0.560	0.141	-2.153	0.034
ENSG00000253548	PYDC2	-0.823	-2.570	-2.102	0.039
ENSG00000253567	RP11-181B11.2	-0.491	-1.209	-2.159	0.034
ENSG00000253576	GS1-5L10.1	-0.403	-0.909	-2.015	0.047
ENSG00000253582	RP11-649G15.2	-0.443	1.014	-2.550	0.013
ENSG00000253621	RPSAP74	-0.931	-2.629	-2.714	0.008
ENSG00000253636	RP11-531A24.5	-0.513	2.373	-3.009	0.003
ENSG00000253660	CTC-353G13.1	-0.555	-1.051	-2.373	0.020
ENSG00000253667	RP11-30L15.4	-0.579	1.429	-2.235	0.028
ENSG00000253671	RP11-806O11.1	-0.721	0.902	-2.517	0.014
ENSG00000253678	RP11-981G7.3	-0.452	2.705	-2.069	0.042
ENSG00000253704	RP11-267M23.4	-0.626	-0.393	-3.526	0.001
ENSG00000253711	RP11-787D18.1	-0.466	-0.193	-2.521	0.014
ENSG00000253713	CTC-264O10.2	-0.555	-0.683	-2.431	0.017
ENSG00000253717	KB-1299A7.2	-0.819	-1.779	-2.977	0.004
ENSG00000253726	RP11-313C15.1	-0.596	1.357	-3.273	0.002
ENSG00000253731	PCDHGA6	-0.301	3.105	-2.909	0.005
ENSG00000253734	LINC01289	-0.442	0.972	-2.442	0.017
ENSG00000253738	OTUD6B-AS1	0.270	6.439	2.471	0.016
ENSG00000253764	RP11-439C15.4	-0.489	1.700	-2.947	0.004
ENSG00000253766	RP11-804N13.1	-0.552	-1.183	-2.499	0.014
ENSG00000253767	PCDHGA8	-0.439	2.321	-3.150	0.002
ENSG00000253784	RP11-1145L24.1	-0.572	-1.621	-2.238	0.028
ENSG00000253805	RP11-1049H7.2	-0.454	-0.561	-2.261	0.026
ENSG00000253846	PCDHGA10	-0.310	3.714	-3.531	0.001
ENSG00000253857	RP11-386G21.2	-0.421	0.630	-2.082	0.040
ENSG00000253873	PCDHGA11	-0.278	2.889	-2.822	0.006
ENSG00000253875	RP11-16P20.3	-0.642	-0.893	-2.646	0.010
ENSG00000253908	RP11-252I14.2	-0.499	-0.305	-2.039	0.045
ENSG00000253910	PCDHGB2	-0.447	3.230	-4.507	0.000
ENSG00000253915	MAPRE1P1	-0.598	2.424	-2.959	0.004
ENSG00000253925	CTB-178M22.1	-0.510	-0.243	-2.120	0.037
ENSG00000253932	RP11-10C8.2	-0.426	0.722	-2.373	0.020
ENSG00000253947	CTB-77H17.1	-0.748	-1.505	-2.817	0.006
ENSG00000253953	PCDHGB4	-0.271	2.605	-2.488	0.015
ENSG00000253956	RP11-211F2.1	-0.592	-0.957	-2.597	0.011
ENSG00000253961	RP11-363L24.3	-1.118	-1.743	-4.561	0.000
ENSG00000253971	CDC42P3	-0.883	-1.042	-3.203	0.002

ENSG00000253975	RP11-577N1.2	-0.734	-0.795	-2.368	0.020
ENSG00000253978	CTB-178M22.2	-0.490	-0.591	-2.360	0.021
ENSG00000253992	RP11-737F9.1	-0.449	-1.730	-2.085	0.040
ENSG00000254040	RP11-421P23.2	-0.752	-0.766	-3.074	0.003
ENSG00000254048	RP11-328K2.1	-0.686	-0.293	-2.898	0.005
ENSG00000254049	NRG1-IT3	-0.698	-1.614	-2.784	0.007
ENSG00000254050	RP11-577N1.1	-0.804	-1.463	-2.281	0.025
ENSG00000254101	LINC02055	0.483	3.010	2.678	0.009
ENSG00000254141	RP11-642D21.1	-0.356	0.921	-2.034	0.045
ENSG00000254163	CTC-340I23.2	-0.523	-1.447	-2.377	0.020
ENSG00000254165	RP11-503E24.2	-0.468	1.138	-2.992	0.004
ENSG00000254166	CASC19	-0.525	-0.595	-2.850	0.006
ENSG00000254221	PCDHGB1	-0.463	2.059	-3.548	0.001
ENSG00000254244	PAICSP4	-0.454	3.456	-2.481	0.015
ENSG00000254251	RP11-662G23.1	-0.641	-0.670	-2.746	0.007
ENSG00000254260	RP11-369E15.1	-0.449	-0.154	-2.285	0.025
ENSG00000254266	PKIA-AS1	-0.365	2.067	-2.929	0.004
ENSG00000254272	RP11-382J24.2	-0.367	0.248	-2.297	0.024
ENSG00000254297	CTC-286N12.1	-0.691	-2.001	-2.375	0.020
ENSG00000254315	RP11-267M23.3	-0.757	-2.524	-2.442	0.017
ENSG00000254330	RP11-1D12.1	-0.528	-0.547	-2.381	0.020
ENSG00000254363	CTB-131B5.5	-0.657	0.604	-2.409	0.018
ENSG00000254365	CTB-180C19.1	-0.695	-1.435	-2.747	0.007
ENSG00000254440	PBOV1	-0.346	2.492	-2.150	0.034
ENSG00000254441	RP11-718B12.5	-0.745	-0.838	-3.058	0.003
ENSG00000254450	ALG9-IT1	-0.627	-1.315	-2.439	0.017
ENSG00000254477	AP000640.10	-0.357	0.224	-2.192	0.031
ENSG00000254484	CTA-797E19.1	-0.589	-1.016	-2.912	0.005
ENSG00000254506	RP11-748H22.1	0.480	-0.360	2.341	0.022
ENSG00000254522	RP11-113K21.1	-0.759	-2.334	-2.202	0.031
ENSG00000254538	RP11-463D19.1	-0.457	1.371	-2.936	0.004
ENSG00000254542	NAV2-AS3	-0.548	-1.063	-2.502	0.014
ENSG00000254556	AF131215.4	-0.496	1.945	-3.406	0.001
ENSG00000254560	BBOX1-AS1	-0.626	-1.730	-2.508	0.014
ENSG00000254577	RP11-484D2.4	-0.644	-0.993	-2.380	0.020
ENSG00000254587	RP11-360K13.1	-0.702	-1.203	-2.705	0.008
ENSG00000254595	CTD-2010I16.1	-0.564	1.794	-3.866	0.000
ENSG00000254604	AP000487.6	-0.692	-0.778	-3.225	0.002
ENSG00000254622	NAV2-AS4	-0.523	0.023	-2.805	0.006
ENSG00000254644	RP11-5A11.2	-0.735	-1.121	-3.097	0.003
ENSG00000254649	RP11-452H21.2	-0.844	-1.939	-3.341	0.001
ENSG00000254664	CTD-2560E9.3	0.859	-0.316	3.939	0.000
ENSG00000254705	RP11-675M1.2	-0.518	0.753	-3.218	0.002
ENSG00000254717	GLYATL1P2	-0.631	-0.896	-2.883	0.005
ENSG00000254731	CTD-2005H7.1	-0.758	-2.700	-2.268	0.026
ENSG00000254743	OR10V3P	-1.013	-3.118	-2.270	0.026
ENSG00000254787	CTD-2537O9.1	-0.316	0.826	-2.093	0.039

ENSG00000254788	CKLF-CMTM1	0.584	-2.291	2.412	0.018
ENSG00000254839	AF131215.6	-0.651	-0.021	-3.254	0.002
ENSG00000254843	RP11-648O15.1	-0.553	-0.342	-2.708	0.008
ENSG00000254890	RP11-10A7.1	-0.669	-0.584	-2.757	0.007
ENSG00000254895	MTCO3P15	-0.793	-2.139	-2.245	0.027
ENSG00000254896	OPCML-IT1	-0.417	1.831	-2.448	0.016
ENSG00000254897	RP11-482L11.1	-0.425	0.288	-2.597	0.011
ENSG00000254901	BORCS8	0.322	2.888	3.119	0.003
ENSG00000254936	AF131215.3	-0.412	2.674	-2.054	0.043
ENSG00000254938	RP11-688I9.4	-0.500	1.631	-3.074	0.003
ENSG00000254960	KIRREL3-AS2	-0.400	1.928	-3.071	0.003
ENSG00000254968	RP11-65M17.3	-0.850	-3.089	-2.210	0.030
ENSG00000254972	RP11-167J8.3	-0.741	-2.159	-2.943	0.004
ENSG00000254985	RSF1-IT2	-0.405	0.068	-2.178	0.032
ENSG00000254996	ANKHD1-EIF4EBP3	-0.220	2.318	-2.433	0.017
ENSG00000254999	BRK1	0.251	5.275	2.764	0.007
ENSG00000255004	RP1-68D18.3	-0.768	1.574	-2.287	0.025
ENSG00000255028	RP11-708B6.2	-0.598	-1.413	-2.209	0.030
ENSG00000255040	RP11-677N16.1	-0.544	0.905	-2.456	0.016
ENSG00000255082	GRM5-AS1	-0.298	1.698	-2.032	0.045
ENSG00000255087	RP11-168K9.2	0.365	1.766	2.286	0.025
ENSG00000255097	RP11-5L12.1	-0.402	-0.695	-2.129	0.036
ENSG00000255105	RP11-5A11.1	-0.627	-0.868	-2.174	0.033
ENSG00000255112	CHMP1B	0.162	5.516	1.995	0.049
ENSG00000255122	RP11-303G3.6	-0.598	-1.781	-2.039	0.045
ENSG00000255135	RP11-111M22.3	0.289	0.834	2.063	0.042
ENSG00000255146	RP11-659P15.1	0.807	-2.765	2.269	0.026
ENSG00000255158	RP11-754B17.1	-0.538	-0.577	-2.563	0.012
ENSG00000255167	RP11-670N15.1	-0.460	-0.503	-2.243	0.028
ENSG00000255185	PDXDC2P	-0.569	-0.792	-2.125	0.037
ENSG00000255207	RP1-305G21.1	-0.865	-2.251	-2.435	0.017
ENSG00000255234	RP11-727A23.10	-0.858	-1.694	-2.580	0.012
ENSG00000255268	RP11-707M1.9	-0.571	-1.110	-2.668	0.009
ENSG00000255280	CTD-2530H12.5	-0.793	-2.625	-2.595	0.011
ENSG00000255296	RP11-757C15.4	-0.743	-2.035	-2.529	0.013
ENSG00000255299	RP11-655C2.3	-0.912	-2.679	-2.644	0.010
ENSG00000255302	EID1	0.249	8.114	2.399	0.019
ENSG00000255311	RP11-160H12.2	-0.363	2.935	-2.220	0.029
ENSG00000255319	ENPP7P8	-0.519	0.260	-2.947	0.004
ENSG00000255329	H2AFZP4	-0.428	-1.621	-2.051	0.043
ENSG00000255334	RP11-708L7.6	-0.726	-2.073	-2.613	0.011
ENSG00000255371	OPCML-IT2	-0.511	1.296	-3.080	0.003
ENSG00000255396	AP000857.2	-0.305	0.674	-2.168	0.033
ENSG00000255403	AP000641.1	-0.299	2.050	-2.129	0.036
ENSG00000255409	RSF1-IT1	-0.754	-2.213	-2.231	0.028
ENSG00000255417	MTCO2P15	-0.540	-1.680	-2.009	0.048
ENSG00000255423	EBLN2	-0.293	1.548	-2.073	0.041

ENSG00000255434	CTD-2530H12.8	-0.420	-0.028	-2.342	0.022
ENSG00000255455	RP11-890B15.3	-0.182	4.823	-2.370	0.020
ENSG00000255483	AP000889.2	-0.936	-1.412	-3.285	0.002
ENSG00000255487	RP11-513D5.5	-0.559	-1.965	-2.114	0.038
ENSG00000255506	RP11-203F8.1	-1.169	-1.275	-3.966	0.000
ENSG00000255508	RP11-864I4.1	-0.541	-1.800	-2.101	0.039
ENSG00000255520	RP11-390K5.3	-0.813	-2.486	-2.639	0.010
ENSG00000255537	AP000708.1	0.305	0.555	2.167	0.033
ENSG00000255538	OR10V2P	-0.634	-1.612	-2.477	0.015
ENSG00000255545	RP11-627G23.1	-0.676	2.195	-3.221	0.002
ENSG00000255547	RPA2P3	-0.785	-0.058	-3.099	0.003
ENSG00000255555	AP000857.3	-0.353	-0.094	-2.335	0.022
ENSG00000255559	ZNF252P-AS1	-0.370	-0.005	-2.270	0.026
ENSG00000255580	AP000462.2	-0.416	1.795	-2.438	0.017
ENSG00000255622	PCDHB17P	-0.529	1.753	-4.353	0.000
ENSG00000255642	PABPC1P4	-0.400	3.064	-2.080	0.041
ENSG00000255670	RP11-253I19.3	-0.512	-0.289	-2.632	0.010
ENSG00000255690	TRIL	-0.467	5.119	-2.193	0.031
ENSG00000255786	RP11-809N8.6	-0.525	-1.837	-2.036	0.045
ENSG00000255794	RMST	-0.241	4.365	-2.362	0.021
ENSG00000255837	TAS2R20	-0.450	1.660	-2.942	0.004
ENSG00000255853	RP11-407A16.1	-0.605	-0.463	-2.237	0.028
ENSG00000255920	CCND2-AS2	-0.379	1.443	-2.490	0.015
ENSG00000255966	RP5-940J5.3	-0.846	-2.015	-3.102	0.003
ENSG00000255980	AP000439.1	0.970	-1.100	2.092	0.040
ENSG00000256001	RP11-575F12.2	0.432	-1.269	2.127	0.036
ENSG00000256019	TAS2R63P	-0.388	-0.291	-2.251	0.027
ENSG00000256025	CACNA1C-AS4	-0.517	0.976	-2.931	0.004
ENSG00000256053	APOPT1	0.209	2.727	2.688	0.009
ENSG00000256064	RP11-117L5.4	-0.504	-0.714	-2.241	0.028
ENSG00000256070	RP11-56H16.1	-0.728	-1.945	-2.478	0.015
ENSG00000256073	URB1-AS1	0.384	0.256	2.417	0.018
ENSG00000256085	RP11-337L12.1	-0.356	1.253	-2.038	0.045
ENSG00000256101	RP11-90D4.3	-0.678	0.222	-3.125	0.002
ENSG00000256120	RP11-778H2.1	-0.615	-0.799	-3.037	0.003
ENSG00000256139	RP11-968O1.5	-0.562	-0.683	-2.738	0.008
ENSG00000256162	SMLR1	-0.404	1.269	-2.624	0.010
ENSG00000256188	TAS2R30	-0.301	2.074	-2.005	0.048
ENSG00000256229	ZNF486	0.197	2.250	2.102	0.039
ENSG00000256234	RP11-283G6.4	-0.420	-1.257	-2.090	0.040
ENSG00000256250	RP11-989F5.1	-0.546	-1.387	-2.293	0.024
ENSG00000256271	CACNA1C-AS2	-0.539	-0.782	-2.721	0.008
ENSG00000256315	AP000462.1	-0.416	2.170	-2.765	0.007
ENSG00000256340	ABCC6P1	-0.640	-1.073	-2.663	0.009
ENSG00000256399	RP11-274J7.3	-0.675	-0.814	-3.172	0.002
ENSG00000256420	OSBPL9P4	-0.464	0.538	-2.447	0.017
ENSG00000256436	TAS2R31	-0.434	1.855	-2.984	0.004

ENSG00000256458	RP11-363J17.1	-0.676	-1.461	-3.076	0.003
ENSG00000256533	RP11-212D19.5	-0.488	0.070	-2.907	0.005
ENSG00000256537	SMIM10L1	0.265	5.332	2.892	0.005
ENSG00000256538	RP11-847H18.3	0.912	-0.499	2.950	0.004
ENSG00000256564	RP11-424M22.3	-0.479	2.035	-2.371	0.020
ENSG00000256571	RP11-274J7.2	-1.196	-1.830	-3.865	0.000
ENSG00000256581	NLRP9P1	-0.468	-0.712	-2.123	0.037
ENSG00000256609	RP11-64B16.4	-0.610	-0.937	-2.078	0.041
ENSG00000256637	LINC01965	0.502	-0.027	3.042	0.003
ENSG00000256654	RP11-429A20.4	-0.416	2.166	-2.145	0.035
ENSG00000256667	KLRA1P	-0.238	2.187	-2.198	0.031
ENSG00000256682	TAS2R12	-0.386	1.177	-2.334	0.022
ENSG00000256712	RP11-785H5.2	-0.403	0.572	-2.376	0.020
ENSG00000256721	CACNA1C-IT3	-0.632	0.045	-2.835	0.006
ENSG00000256769	CACNA1C-AS3	-0.672	-2.245	-2.407	0.018
ENSG00000256817	TPT1P12	-1.036	-2.324	-3.063	0.003
ENSG00000256894	RP11-283G6.3	-0.386	0.093	-2.456	0.016
ENSG00000256971	LINC00508	-0.549	-0.207	-2.016	0.047
ENSG00000256972	AP000462.3	-0.628	0.527	-3.100	0.003
ENSG00000256973	RP11-359J14.2	-0.392	2.401	-2.218	0.029
ENSG00000256981	TAS2R18	-0.399	1.783	-2.289	0.025
ENSG00000257004	RP11-180M15.3	-0.612	-2.586	-2.246	0.027
ENSG00000257022	RP11-729I10.2	-0.711	-2.210	-2.658	0.009
ENSG00000257045	RP11-486F17.1	-0.508	0.287	-2.635	0.010
ENSG00000257056	LINC02282	-0.362	2.076	-2.319	0.023
ENSG00000257057	C11orf97	0.548	-0.145	2.917	0.005
ENSG00000257060	RP11-266O8.1	-0.365	1.300	-2.641	0.010
ENSG00000257083	RP11-123O10.3	-0.708	-1.032	-2.916	0.005
ENSG00000257095	RP11-364C11.2	-0.571	0.155	-3.548	0.001
ENSG00000257169	RP11-117N2.2	-0.428	0.381	-2.413	0.018
ENSG00000257176	RP11-996F15.2	-0.291	1.992	-2.527	0.013
ENSG00000257180	RP11-509E10.1	-0.455	1.398	-2.764	0.007
ENSG00000257191	RP11-390N6.1	-0.393	1.758	-2.329	0.022
ENSG00000257220	RP11-136F16.2	-0.699	-0.514	-3.483	0.001
ENSG00000257246	PHBP19	-0.494	0.330	-2.829	0.006
ENSG00000257261	RP11-96H19.1	-0.308	0.457	-2.247	0.027
ENSG00000257267	ZNF271P	0.239	5.523	3.396	0.001
ENSG00000257279	RP11-231I16.1	-0.413	1.159	-2.495	0.015
ENSG00000257283	RP11-1060G2.1	-0.500	-1.485	-2.287	0.025
ENSG00000257302	FAHD2P1	-0.573	-0.322	-2.393	0.019
ENSG00000257331	RP11-478B9.3	-0.658	0.339	-2.468	0.016
ENSG00000257354	RP11-631N16.2	-0.296	2.734	-2.188	0.032
ENSG00000257438	RP11-114F10.3	-0.442	1.316	-2.828	0.006
ENSG00000257458	RP11-473C19.1	-0.573	1.937	-3.153	0.002
ENSG00000257472	RP11-159D23.2	-0.699	-1.850	-2.087	0.040
ENSG00000257489	RP11-175P13.3	-0.694	-1.949	-2.847	0.006
ENSG00000257545	RP11-144F15.1	-0.434	-1.090	-2.071	0.041

ENSG00000257572	RP11-478B9.2	-0.500	0.217	-2.387	0.019
ENSG00000257621	PSMA3-AS1	-0.162	5.532	-2.250	0.027
ENSG00000257658	RP3-521E19.3	-0.547	-1.145	-2.283	0.025
ENSG00000257674	RP11-76E16.2	-0.639	-1.422	-2.380	0.020
ENSG00000257680	RP11-367O10.1	-0.453	2.782	-3.449	0.001
ENSG00000257711	RP11-482D24.2	-0.633	-0.747	-2.902	0.005
ENSG00000257727	CNPY2	0.241	2.092	2.897	0.005
ENSG00000257752	RP11-474L23.3	-0.747	-0.763	-3.642	0.000
ENSG00000257763	OR5BK1P	-0.438	1.167	-2.417	0.018
ENSG00000257792	OR5BJ1P	-0.486	-0.675	-2.008	0.048
ENSG00000257818	C1GALT1P1	-0.454	1.231	-3.200	0.002
ENSG00000257835	RP1-97G4.1	-0.606	1.718	-3.813	0.000
ENSG00000257864	RP11-46I1.2	-0.924	-2.452	-2.945	0.004
ENSG00000257880	RP11-769N19.2	-0.592	1.114	-3.269	0.002
ENSG00000257894	RP1-78O14.1	-0.511	2.404	-2.829	0.006
ENSG00000257897	RP11-812D23.1	-0.407	-0.059	-2.114	0.038
ENSG00000257918	RP11-482D24.3	-0.690	-1.162	-2.687	0.009
ENSG00000257923	CUX1	-0.132	6.268	-2.057	0.043
ENSG00000257943	RP11-167N24.3	-0.685	-1.840	-2.465	0.016
ENSG00000257947	RP11-46I1.1	-0.777	-1.808	-2.748	0.007
ENSG00000257954	RP11-161H23.10	-0.488	2.662	-1.994	0.050
ENSG00000257957	QRSL1P3	-0.368	1.081	-2.053	0.043
ENSG00000258007	RP11-350G24.1	-0.557	-0.141	-2.547	0.013
ENSG00000258033	RP11-350G24.2	-0.424	2.622	-2.114	0.038
ENSG00000258039	RP11-406H4.1	-0.606	1.118	-2.861	0.005
ENSG00000258048	RP11-530C5.1	-0.581	-1.757	-2.317	0.023
ENSG00000258066	RP11-781A6.1	-0.641	0.389	-2.890	0.005
ENSG00000258068	RP11-328C8.5	-0.577	-0.948	-2.861	0.005
ENSG00000258172	RP11-1105G2.4	-0.791	-2.365	-2.902	0.005
ENSG00000258204	RP11-90E9.1	-0.669	1.438	-3.010	0.003
ENSG00000258212	ZNF75BP	-0.520	-0.117	-2.336	0.022
ENSG00000258225	RP11-136F16.1	-0.544	-0.978	-2.698	0.008
ENSG00000258274	RP11-887P2.5	-0.491	1.407	-3.363	0.001
ENSG00000258302	RP11-981P6.1	-0.430	3.335	-2.846	0.006
ENSG00000258337	RP11-115H15.2	-0.422	-0.326	-2.081	0.041
ENSG00000258352	RP11-23J18.1	-0.717	-2.565	-2.377	0.020
ENSG00000258365	RP11-1105G2.3	-0.468	-0.686	-2.469	0.016
ENSG00000258375	RP11-617J18.1	-0.363	1.080	-2.116	0.037
ENSG00000258392	RPA2P1	-0.701	-0.959	-2.943	0.004
ENSG00000258428	RP11-1085N6.2	-0.653	-1.741	-2.531	0.013
ENSG00000258437	RP11-661G16.1	-0.595	-1.684	-2.408	0.018
ENSG00000258445	RP11-307P22.1	-0.556	0.947	-2.923	0.004
ENSG00000258455	RP11-665C16.5	-0.811	-1.350	-2.712	0.008
ENSG00000258490	RP11-862P13.1	-0.397	0.322	-2.524	0.014
ENSG00000258510	RP11-493G17.4	-0.439	0.066	-2.113	0.038
ENSG00000258515	RP11-203M5.7	-0.424	0.560	-2.588	0.011
ENSG00000258526	RP11-111A21.1	-0.510	0.271	-3.613	0.001

ENSG00000258546	CENPUP2	-0.479	-0.878	-2.183	0.032
ENSG00000258553	RP11-16B13.1	-0.649	-1.965	-2.508	0.014
ENSG00000258571	PTTG4P	-0.760	-1.174	-3.024	0.003
ENSG00000258577	SNRPGP1	-0.497	-1.326	-2.094	0.039
ENSG00000258580	RP11-320M16.2	-0.524	0.389	-3.390	0.001
ENSG00000258619	RP11-134E15.1	-0.854	-2.438	-2.238	0.028
ENSG00000258624	SERPINE4P	-0.734	-1.558	-2.677	0.009
ENSG00000258631	RP11-739G5.1	-0.412	-1.142	-2.396	0.019
ENSG00000258634	RP4-773N10.4	-0.211	2.151	-2.026	0.046
ENSG00000258637	RP11-242P2.1	-0.440	1.551	-2.482	0.015
ENSG00000258646	RP11-950C14.3	-0.457	-1.140	-2.243	0.028
ENSG00000258662	RP11-588P7.2	-0.382	1.572	-2.194	0.031
ENSG00000258696	CTD-2058B24.3	0.597	-1.796	2.194	0.031
ENSG00000258719	RP11-232C2.3	-0.693	-0.221	-3.739	0.000
ENSG00000258730	ITPK1-AS1	-0.477	1.196	-2.187	0.032
ENSG00000258743	LINC02301	0.612	0.565	2.750	0.007
ENSG00000258754	LINC01579	-0.477	0.754	-2.955	0.004
ENSG00000258796	CTD-2560C21.1	-0.449	0.524	-2.775	0.007
ENSG00000258824	CTD-2555O16.2	-0.246	0.365	-2.001	0.049
ENSG00000258829	CTD-2243E23.1	-0.424	2.330	-2.706	0.008
ENSG00000258876	RP11-270M14.5	-0.623	-2.261	-2.477	0.015
ENSG00000258890	CEP95	-0.190	5.315	-2.218	0.029
ENSG00000258904	RP11-638I2.2	-0.640	-0.727	-3.203	0.002
ENSG00000258909	RP11-164C12.2	-1.000	-1.591	-3.499	0.001
ENSG00000258912	LINC02316	-0.578	-2.339	-1.996	0.049
ENSG00000258928	RP11-463J10.2	-0.464	-0.823	-2.245	0.027
ENSG00000258930	RP5-1163L11.2	-0.523	0.509	-2.139	0.035
ENSG00000258944	RP4-647C14.3	-0.785	-1.201	-2.398	0.019
ENSG00000258951	KRT18P7	-0.452	-0.245	-2.110	0.038
ENSG00000258957	RP11-363J20.1	-0.486	-0.453	-2.669	0.009
ENSG00000258966	GTF3AP2	-0.821	-2.539	-2.535	0.013
ENSG00000258972	NDUFB8P1	-0.618	-1.919	-2.229	0.029
ENSG00000258983	RP11-507K2.2	-0.495	-0.706	-2.330	0.022
ENSG00000258986	TMEM179	0.304	4.683	3.234	0.002
ENSG00000258993	RP11-368P15.2	-0.583	-1.646	-2.510	0.014
ENSG00000259008	RP11-932A10.1	-0.704	-1.035	-2.356	0.021
ENSG00000259030	FPGT-TNNI3K	-0.332	1.981	-2.473	0.015
ENSG00000259055	RP11-1140I5.1	-0.382	1.622	-2.545	0.013
ENSG00000259056	DUXAP1	-0.575	-1.811	-2.117	0.037
ENSG00000259079	RP1-261D10.1	-0.506	1.318	-2.250	0.027
ENSG00000259096	FAM35CP	-0.525	1.018	-2.100	0.039
ENSG00000259100	RP11-1074O12.1	-0.552	0.517	-2.401	0.019
ENSG00000259103	RP11-270M14.4	-0.392	0.588	-2.050	0.044
ENSG00000259116	RP11-973N13.4	-0.355	0.481	-2.084	0.040
ENSG00000259118	RP11-840I19.3	-0.363	0.103	-2.202	0.030
ENSG00000259153	RP6-65G23.3	0.286	1.906	2.272	0.026
ENSG00000259158	ADAM20P1	-0.321	3.426	-2.138	0.036

ENSG00000259163	RP11-1078H9.5	-0.488	0.392	-3.372	0.001
ENSG00000259165	DDX18P1	-0.514	1.385	-2.793	0.007
ENSG00000259175	RP11-379K22.2	-0.360	1.643	-2.230	0.028
ENSG00000259221	CTD-2050N2.1	-0.418	-0.521	-2.294	0.024
ENSG00000259228	HNRNPA1P62	-0.507	-0.075	-2.009	0.048
ENSG00000259241	RP11-313P18.2	-0.647	-0.289	-3.237	0.002
ENSG00000259257	RP11-616M17.1	-0.446	0.014	-2.249	0.027
ENSG00000259258	RP11-325L12.3	-0.658	-0.009	-3.343	0.001
ENSG00000259269	RP11-624L4.2	-0.485	-1.060	-2.131	0.036
ENSG00000259276	RP11-815J21.3	-0.610	-2.067	-2.214	0.030
ENSG00000259277	RP13-126C7.1	-0.430	-0.114	-2.232	0.028
ENSG00000259287	RP11-3D4.2	-0.649	-0.943	-3.496	0.001
ENSG00000259296	RP11-313P18.1	-0.518	-0.060	-2.664	0.009
ENSG00000259300	TUBBP8	-0.823	-2.106	-2.393	0.019
ENSG00000259314	CTD-3065B20.3	-0.513	-0.024	-2.661	0.009
ENSG00000259321	RP11-468E2.5	0.685	-2.042	2.367	0.020
ENSG00000259322	RP11-762H8.1	-0.506	-0.294	-3.011	0.003
ENSG00000259326	RP11-102L12.2	-0.426	2.652	-3.110	0.003
ENSG00000259339	TGIF1P1	-0.731	-1.366	-2.885	0.005
ENSG00000259345	RP11-624L4.1	-0.637	1.008	-3.614	0.001
ENSG00000259347	RP11-798K3.2	-0.485	-0.898	-2.534	0.013
ENSG00000259358	RP11-456J20.1	-0.348	3.182	-2.629	0.010
ENSG00000259359	RP11-327J17.2	-0.726	-2.506	-2.243	0.028
ENSG00000259363	CTD-2054N24.2	0.768	-1.457	2.116	0.037
ENSG00000259366	CTD-2647L4.4	-0.414	2.330	-3.637	0.000
ENSG00000259370	RP11-1069G10.1	-0.332	2.050	-2.699	0.008
ENSG00000259377	CTD-2308G16.1	-0.393	0.466	-3.130	0.002
ENSG00000259379	MTND5P32	-0.655	-1.273	-2.224	0.029
ENSG00000259381	RP11-192M23.1	-0.587	0.865	-3.061	0.003
ENSG00000259390	RP11-27M9.1	-0.486	-1.108	-2.364	0.020
ENSG00000259407	RP11-158M2.3	-0.463	-0.215	-2.258	0.027
ENSG00000259408	RP11-3D4.3	-0.685	-0.682	-3.544	0.001
ENSG00000259411	HNRNPA1P45	-0.777	-2.006	-2.095	0.039
ENSG00000259431	THTPA	0.249	1.415	2.195	0.031
ENSG00000259432	RP11-37C7.2	-0.628	-2.041	-2.201	0.031
ENSG00000259440	NPM1P43	-0.582	0.055	-2.359	0.021
ENSG00000259447	RP11-462P6.1	-0.593	-0.491	-3.239	0.002
ENSG00000259463	RP11-540O11.7	-0.523	-1.585	-2.351	0.021
ENSG00000259478	RP11-753A21.1	-0.850	-2.490	-2.882	0.005
ENSG00000259481	RP11-39M21.1	-0.773	1.677	-4.163	0.000
ENSG00000259482	RORA-AS2	-0.933	-2.034	-3.269	0.002
ENSG00000259485	LINC02253	-0.958	-2.514	-2.920	0.005
ENSG00000259488	RP11-154J22.1	-0.350	0.297	-2.343	0.022
ENSG00000259494	MRPL46	-0.187	2.456	-2.261	0.026
ENSG00000259505	RP11-74D7.1	-1.412	-0.739	-4.330	0.000
ENSG00000259512	HNRNPA1P5	-0.534	-0.346	-2.486	0.015
ENSG00000259544	RP11-158M2.2	-0.782	-2.241	-2.406	0.018

ENSG00000259554	RP11-408J6.1	-0.469	-0.821	-2.053	0.043
ENSG00000259562	RP11-762H8.2	-0.387	1.025	-2.336	0.022
ENSG00000259563	CTD-2329K10.1	-0.565	-1.527	-2.653	0.010
ENSG00000259564	RP11-16B9.1	-0.517	-0.229	-3.466	0.001
ENSG00000259575	RP11-39M21.2	-0.592	0.363	-2.793	0.006
ENSG00000259577	RP11-430B1.2	0.334	1.213	2.165	0.033
ENSG00000259587	RP11-463I20.2	-0.717	-2.219	-2.129	0.036
ENSG00000259589	RP11-317G6.1	-0.604	-1.123	-2.072	0.041
ENSG00000259591	RP11-554D20.1	-0.540	0.680	-2.701	0.008
ENSG00000259600	RP11-925D8.3	-0.974	-1.849	-3.321	0.001
ENSG00000259617	RP11-540O11.6	-0.648	-1.681	-2.232	0.028
ENSG00000259644	RP11-680F8.4	-0.404	1.510	-2.127	0.036
ENSG00000259668	RP11-707P17.2	-0.388	0.617	-2.246	0.027
ENSG00000259677	RP11-493E3.1	-0.795	-1.464	-2.770	0.007
ENSG00000259686	HNRNPA1P71	-1.239	-1.413	-4.369	0.000
ENSG00000259699	HMGB1P8	-0.494	0.068	-2.383	0.019
ENSG00000259707	RP11-752G15.4	-0.485	-0.692	-2.133	0.036
ENSG00000259728	LINC00933	-0.539	-1.583	-2.197	0.031
ENSG00000259756	RP11-625H11.2	-0.463	-1.291	-2.485	0.015
ENSG00000259782	CTD-2270L9.2	-0.801	-2.633	-2.183	0.032
ENSG00000259807	RP11-426C22.4	-0.464	-0.329	-2.071	0.042
ENSG00000259814	RP11-300A12.2	-0.545	-0.263	-2.812	0.006
ENSG00000259820	AC083843.1	-0.284	3.068	-2.081	0.041
ENSG00000259823	LYPD8	0.249	2.834	2.238	0.028
ENSG00000259831	LINC00567	0.626	-0.941	2.285	0.025
ENSG00000259846	RP11-467L24.1	0.819	-1.617	2.099	0.039
ENSG00000259866	RP11-177N22.2	-0.748	-1.269	-3.601	0.001
ENSG00000259905	PWRN1	-0.565	2.458	-3.252	0.002
ENSG00000259915	RP11-410E4.1	-0.483	0.263	-2.326	0.023
ENSG00000259920	RP11-2E11.5	-0.383	-0.505	-2.139	0.035
ENSG00000259946	RP11-490G2.2	-0.400	3.215	-2.835	0.006
ENSG00000259963	RP11-185J20.2	-0.757	-0.922	-2.020	0.047
ENSG00000259972	AC009120.6	-0.413	2.603	-3.323	0.001
ENSG00000259986	RP11-382A20.4	-0.412	0.292	-2.207	0.030
ENSG00000260007	RP11-315D16.2	-0.388	1.824	-2.645	0.010
ENSG00000260011	RP13-20L14.1	-0.595	-2.423	-1.997	0.049
ENSG00000260012	RP11-329J18.4	-0.562	-0.148	-2.333	0.022
ENSG00000260032	NORAD	0.163	10.363	2.084	0.040
ENSG00000260033	CTC-527H23.1	-0.500	-0.355	-2.678	0.009
ENSG00000260034	LCMT1-AS2	-0.433	3.199	-2.854	0.005
ENSG00000260084	RP11-615I2.1	-0.646	-1.969	-2.261	0.026
ENSG00000260115	CTC-420A11.2	-0.541	-1.537	-2.363	0.021
ENSG00000260137	RP11-258F22.2	-0.701	-2.007	-2.077	0.041
ENSG00000260171	RP11-95H11.1	-0.475	2.077	-3.311	0.001
ENSG00000260194	RP11-357N13.2	-0.438	0.621	-2.657	0.009
ENSG00000260209	RP11-680F20.10	-0.547	-0.365	-2.821	0.006
ENSG00000260231	JHDM1D-AS1	0.262	1.363	2.363	0.021

ENSG00000260255	RP11-185O21.1	-0.499	0.004	-2.332	0.022
ENSG00000260306	RP11-645C24.5	-0.967	-0.394	-3.930	0.000
ENSG00000260314	MRC1	0.703	2.855	2.019	0.047
ENSG00000260385	RP11-450H6.3	-0.661	-1.221	-2.897	0.005
ENSG00000260398	RP11-594N15.3	-0.435	2.535	-3.511	0.001
ENSG00000260418	RP3-406A7.7	-0.607	-0.130	-2.571	0.012
ENSG00000260422	RP1-155D22.2	-1.002	-2.692	-2.365	0.020
ENSG00000260451	GEMIN8P2	-1.266	-3.111	-3.064	0.003
ENSG00000260452	TPRKBP2	-0.630	-1.518	-2.274	0.026
ENSG00000260455	NBAT1	0.417	0.193	2.734	0.008
ENSG00000260461	RP11-541N10.3	-0.458	1.041	-3.275	0.002
ENSG00000260536	RP5-1085F17.4	-0.278	-0.051	-2.098	0.039
ENSG00000260551	PWRN2	-0.904	0.351	-2.670	0.009
ENSG00000260563	RP13-516M14.1	-0.460	1.647	-3.502	0.001
ENSG00000260565	CTD-2270P14.1	-0.291	5.558	-2.377	0.020
ENSG00000260576	EIF5A2P1	-0.557	-0.801	-2.853	0.005
ENSG00000260588	RP11-930P14.2	-0.621	-2.076	-2.036	0.045
ENSG00000260599	CTC-457E21.1	-0.945	0.831	-2.713	0.008
ENSG00000260600	RP11-109D24.1	-0.364	1.166	-2.635	0.010
ENSG00000260618	RP11-23N2.4	-0.660	0.406	-3.625	0.001
ENSG00000260621	RP11-413H22.2	-0.624	-2.420	-2.129	0.036
ENSG00000260635	RP11-21M24.3	-0.674	-2.203	-2.613	0.011
ENSG00000260650	RP11-403P17.2	-0.988	-2.433	-2.589	0.011
ENSG00000260721	AF067845.1	-0.496	0.856	-2.343	0.022
ENSG00000260733	RP11-264L1.4	-0.614	-0.311	-3.034	0.003
ENSG00000260744	RP11-329J18.3	-0.436	-0.035	-2.201	0.031
ENSG00000260751	CTD-2196E14.6	-0.472	-0.638	-2.369	0.020
ENSG00000260796	RP11-1348G14.5	-0.512	-0.695	-2.257	0.027
ENSG00000260816	RP11-319G9.3	-0.665	1.818	-3.829	0.000
ENSG00000260832	CTD-3253I12.1	-0.471	0.298	-2.218	0.029
ENSG00000260835	RP11-468I15.1	-0.666	0.479	-3.458	0.001
ENSG00000260855	RP11-439E19.10	-0.348	3.070	-2.975	0.004
ENSG00000260862	RP11-22H5.2	-0.534	0.601	-2.704	0.008
ENSG00000260876	LINC01229	-0.673	-2.760	-2.047	0.044
ENSG00000260918	RP11-731J8.2	-0.291	7.126	-2.846	0.006
ENSG00000260948	RP11-552M11.8	-0.413	2.754	-3.347	0.001
ENSG00000260969	RP11-190D6.2	-0.948	-2.918	-3.262	0.002
ENSG00000260990	RP3-518E13.2	-0.498	-1.053	-2.179	0.032
ENSG00000260995	RP11-165H23.1	-0.900	-1.739	-2.818	0.006
ENSG00000261003	RP1-140C12.2	-0.545	0.376	-2.315	0.023
ENSG00000261019	RP11-111K18.2	-0.431	0.715	-3.091	0.003
ENSG00000261026	CTD-3247F14.2	-0.445	0.333	-2.532	0.013
ENSG00000261040	WFDC21P	-0.615	-2.469	-2.061	0.043
ENSG00000261051	RP11-274H2.5	-0.545	0.327	-2.756	0.007
ENSG00000261054	RP11-6O2.4	-0.557	-1.361	-2.110	0.038
ENSG00000261056	RP11-454F8.2	-0.494	1.742	-3.445	0.001
ENSG00000261072	RP11-5N19.3	-0.497	0.716	-3.108	0.003

ENSG00000261087	KB-1460A1.5	-0.240	4.973	-2.304	0.024
ENSG00000261096	RP11-690I21.2	-0.324	1.312	-2.416	0.018
ENSG00000261098	RP11-819C21.1	-0.320	4.051	-3.145	0.002
ENSG00000261136	RP11-37C7.3	-0.539	-0.081	-2.690	0.009
ENSG00000261191	RP11-16L14.2	-1.049	-2.939	-3.014	0.003
ENSG00000261198	RP11-26O3.1	-0.628	-1.178	-2.378	0.020
ENSG00000261216	RP11-166B2.5	-0.683	-1.363	-2.456	0.016
ENSG00000261229	RP11-38G5.4	-0.657	-0.961	-3.445	0.001
ENSG00000261236	BOP1	0.198	3.392	2.292	0.024
ENSG00000261242	CTD-2302E22.4	0.592	-1.068	2.369	0.020
ENSG00000261267	RP11-44I10.3	-0.362	-0.224	-2.100	0.039
ENSG00000261292	RP11-389G6.3	0.367	4.688	2.758	0.007
ENSG00000261326	LINC01355	-0.251	3.698	-2.166	0.033
ENSG00000261336	EIF4BP5	-0.657	0.508	-2.538	0.013
ENSG00000261371	PECAM1	0.499	4.498	2.567	0.012
ENSG00000261379	RP11-395N3.1	-0.823	-1.643	-2.783	0.007
ENSG00000261390	MAFTRR	-0.641	-1.629	-2.391	0.019
ENSG00000261410	RP11-543N12.1	-0.554	-1.384	-2.145	0.035
ENSG00000261423	RP11-1007O24.3	-0.320	0.979	-2.242	0.028
ENSG00000261424	ATP5F1P7	-0.633	-0.487	-2.298	0.024
ENSG00000261426	AC144833.1	-0.500	2.601	-2.897	0.005
ENSG00000261438	RP11-399O19.9	-0.551	-0.880	-2.208	0.030
ENSG00000261439	DKFZP434H168	-0.394	1.694	-2.538	0.013
ENSG00000261448	CTD-2576D5.4	-0.752	-2.148	-2.754	0.007
ENSG00000261451	RP11-981G7.1	-0.470	5.023	-2.890	0.005
ENSG00000261520	DLGAP1-AS5	-0.344	4.097	-2.475	0.015
ENSG00000261528	AC002400.1	-0.485	1.196	-2.371	0.020
ENSG00000261529	RP13-487P22.1	-0.431	5.243	-2.715	0.008
ENSG00000261534	RP11-244O19.1	-0.240	3.894	-2.159	0.034
ENSG00000261544	RP11-16E23.3	-0.601	1.177	-2.759	0.007
ENSG00000261560	RP11-166B2.3	-0.439	0.076	-2.505	0.014
ENSG00000261572	RP11-384L8.1	0.592	-0.337	2.330	0.022
ENSG00000261610	AP000265.1	-0.758	-2.202	-2.116	0.037
ENSG00000261617	LINC02177	-0.466	1.569	-2.120	0.037
ENSG00000261629	RP5-842K16.2	-0.910	-1.093	-3.333	0.001
ENSG00000261647	IMPDH1P11	-0.439	1.581	-2.437	0.017
ENSG00000261652	C15orf65	0.281	0.861	2.440	0.017
ENSG00000261668	RP11-50D9.3	-0.575	0.709	-2.518	0.014
ENSG00000261716	RP11-196G18.22	-0.302	3.345	-2.769	0.007
ENSG00000261758	RP11-102M11.2	-0.816	-1.974	-2.876	0.005
ENSG00000261795	RP11-90P13.1	1.052	-1.972	2.205	0.030
ENSG00000261799	RP11-283I3.6	-0.321	3.871	-3.158	0.002
ENSG00000261823	RP11-48G14.1	-0.553	0.443	-3.143	0.002
ENSG00000261837	RP11-264L1.3	-0.541	-1.108	-2.522	0.014
ENSG00000261838	RP11-303E16.6	-0.547	-0.870	-2.322	0.023
ENSG00000261842	RP11-143I21.1	-0.648	-1.314	-2.192	0.031
ENSG00000261868	MFSD1P1	-0.444	0.692	-2.227	0.029

ENSG00000261970	RP11-342L8.2	-0.514	0.411	-2.675	0.009
ENSG00000262001	DLGAP1-AS2	-0.429	3.449	-2.702	0.008
ENSG00000262020	RP11-66H6.3	-0.670	-1.947	-2.262	0.026
ENSG00000262096	PCDHB19P	-0.334	2.094	-2.988	0.004
ENSG00000262118	MTCO1P28	-0.594	0.516	-2.813	0.006
ENSG00000262209	PCDHGB3	-0.418	2.143	-4.075	0.000
ENSG00000262228	RP11-676J12.4	-0.628	-1.212	-2.572	0.012
ENSG00000262265	RP5-867C24.4	-0.359	1.811	-2.180	0.032
ENSG00000262267	U95743.1	0.677	-0.667	2.491	0.015
ENSG00000262316	RP11-433P17.1	-0.857	-1.454	-2.946	0.004
ENSG00000262454	MIR193BHG	0.209	3.976	2.075	0.041
ENSG00000262482	LA16c-321D4.2	-0.575	-2.298	-2.184	0.032
ENSG00000262503	RP11-530N7.2	-0.412	0.718	-2.827	0.006
ENSG00000262516	RP11-95J11.1	-0.720	-1.417	-2.679	0.009
ENSG00000262529	CTA-276F8.2	-0.467	1.552	-2.522	0.014
ENSG00000262580	RP11-334C17.5	-0.522	-0.232	-2.381	0.020
ENSG00000262652	RP13-638C3.2	-0.650	-1.162	-2.005	0.048
ENSG00000262663	RP11-497H17.1	-0.579	-1.399	-2.238	0.028
ENSG00000262732	CTD-2135D7.2	-0.716	-0.338	-3.557	0.001
ENSG00000262905	RP5-1029F21.2	-0.646	-1.480	-2.158	0.034
ENSG00000262943	ALOX12P2	-0.292	3.341	-1.994	0.050
ENSG00000263006	ROCK1P1	-0.826	2.636	-3.756	0.000
ENSG00000263069	CTD-2047H16.4	-0.369	2.089	-2.807	0.006
ENSG00000263072	ZNF213-AS1	-0.234	3.158	-2.343	0.022
ENSG00000263089	RP11-166P13.4	-0.428	0.822	-2.187	0.032
ENSG00000263177	MTND1P8	-0.483	0.013	-2.133	0.036
ENSG00000263189	RP11-618P13.1	-0.538	-0.885	-2.082	0.040
ENSG00000263219	RYKP1	-0.467	1.812	-2.167	0.033
ENSG00000263220	RP11-420A6.2	-0.552	-1.346	-2.439	0.017
ENSG00000263276	RP11-96D1.10	-0.432	-0.580	-2.359	0.021
ENSG00000263316	RP11-530N7.3	-0.343	0.742	-2.076	0.041
ENSG00000263326	RP11-266L9.6	-0.492	0.444	-2.399	0.019
ENSG00000263382	RP11-552O4.1	-1.276	-2.220	-3.910	0.000
ENSG00000263393	RP11-53I6.4	-0.464	-0.396	-2.650	0.010
ENSG00000263400	TMEM220-AS1	-0.607	-1.018	-2.649	0.010
ENSG00000263426	RN7SL471P	0.660	4.036	2.356	0.021
ENSG00000263485	CTC-304I17.4	-0.484	0.471	-2.628	0.010
ENSG00000263494	AC004702.2	-0.623	-2.099	-2.470	0.016
ENSG00000263499	RP11-118E18.4	-0.445	-0.275	-2.429	0.017
ENSG00000263528	IKBKE	0.432	-0.116	2.135	0.036
ENSG00000263597	MIR3936	-0.475	-1.123	-2.504	0.014
ENSG00000263634	MIR3919	-0.934	-2.810	-3.306	0.001
ENSG00000263680	RP11-57A1.1	-0.471	-2.020	-1.996	0.049
ENSG00000263766	RP11-580I16.2	-0.441	-0.832	-2.404	0.018
ENSG00000263823	RP11-326K13.4	0.582	-1.899	1.998	0.049
ENSG00000263826	RP11-573D15.9	-0.230	1.714	-2.035	0.045
ENSG00000263847	RP11-143J12.3	-0.495	-0.621	-2.792	0.007

ENSG00000263874	LINC00672	0.280	5.131	2.555	0.012
ENSG00000263905	RN7SL555P	2.002	-2.280	2.972	0.004
ENSG00000263969	RN7SL678P	0.883	-3.169	2.082	0.040
ENSG00000263993	RN7SL786P	1.144	-3.801	2.270	0.026
ENSG00000264017	RN7SL336P	0.799	-0.687	2.812	0.006
ENSG00000264044	RP11-192H23.7	-0.441	-0.133	-2.307	0.024
ENSG00000264050	RP11-22N12.2	-0.834	-2.284	-2.781	0.007
ENSG00000264057	RP11-583F2.1	-0.656	0.440	-2.539	0.013
ENSG00000264112	RP11-159D12.2	-0.388	4.654	-2.838	0.006
ENSG00000264169	RN7SL665P	0.989	1.174	2.792	0.007
ENSG00000264176	MAGOH2P	-0.463	0.031	-2.065	0.042
ENSG00000264217	RPL35AP25	-0.512	-1.548	-2.035	0.045
ENSG00000264246	RP11-138C24.2	-0.684	-2.411	-2.180	0.032
ENSG00000264247	LINC00909	0.181	3.750	2.380	0.020
ENSG00000264315	HNRNPA1P11	-0.659	2.128	-2.624	0.010
ENSG00000264364	DYNLL2	0.177	7.794	2.216	0.030
ENSG00000264434	CTD-2006O16.2	-1.170	-2.424	-3.598	0.001
ENSG00000264513	RP11-556O9.2	-0.419	0.949	-2.960	0.004
ENSG00000264520	RP4-777O23.2	-0.521	-0.870	-2.623	0.010
ENSG00000264536	MIR5706	-0.727	-2.616	-2.351	0.021
ENSG00000264575	LINC00526	0.343	-0.064	2.226	0.029
ENSG00000264577	AC010761.8	0.354	-0.173	2.213	0.030
ENSG00000264596	RP11-678G15.1	-0.465	-0.408	-2.478	0.015
ENSG00000264630	PRKCA-AS1	-0.638	1.648	-4.117	0.000
ENSG00000264707	L3MBTL4-AS1	-0.349	0.732	-2.390	0.019
ENSG00000264720	MIR3117	-0.445	-1.731	-2.040	0.045
ENSG00000264754	CTD-2653B5.1	-0.599	-1.239	-2.045	0.044
ENSG00000264924	RP11-799B12.2	-0.507	0.343	-2.476	0.015
ENSG00000264954	PRR29-AS1	0.532	-1.035	2.210	0.030
ENSG00000265078	RN7SL664P	0.815	-1.350	2.138	0.036
ENSG00000265113	RP11-70O5.2	-0.569	-1.740	-2.045	0.044
ENSG00000265115	RP11-31I22.2	-0.568	0.066	-2.455	0.016
ENSG00000265125	RP11-31I22.3	-0.451	1.200	-2.175	0.033
ENSG00000265158	LRRC37A7P	-0.688	-2.278	-2.011	0.048
ENSG00000265164	MIR2681	-0.644	-0.886	-3.331	0.001
ENSG00000265194	RP11-70L8.4	-0.385	1.257	-2.520	0.014
ENSG00000265204	RP11-403A21.3	-0.652	-1.185	-2.828	0.006
ENSG00000265218	RP11-927P21.1	-0.540	1.533	-2.525	0.013
ENSG00000265241	RBM8A	0.269	5.761	3.277	0.002
ENSG00000265261	RP11-162A12.3	-0.474	-0.104	-2.477	0.015
ENSG00000265279	CLUHP6	-0.819	-2.107	-2.834	0.006
ENSG00000265313	LINC01476	0.328	2.128	2.001	0.049
ENSG00000265327	RN7SL411P	0.997	-0.485	2.521	0.014
ENSG00000265354	TIMM23	0.341	4.617	2.656	0.010
ENSG00000265386	RN7SL219P	1.253	-2.948	2.232	0.028
ENSG00000265451	RP11-204L24.2	-0.685	-1.824	-2.544	0.013
ENSG00000265656	RP11-13N13.6	-0.654	-1.649	-2.126	0.037

ENSG00000265657	MIR3151	-0.912	-1.319	-3.378	0.001
ENSG00000265666	RARA-AS1	0.504	-0.995	2.266	0.026
ENSG00000265689	RP11-118G23.1	-0.490	-0.623	-2.007	0.048
ENSG00000265775	CTC-304I17.3	-0.522	-0.565	-2.107	0.038
ENSG00000265918	RN7SL543P	1.298	-2.894	2.158	0.034
ENSG00000265992	ESRG	-0.769	-0.361	-3.308	0.001
ENSG00000266111	RP11-296K13.4	-0.349	-0.121	-1.995	0.049
ENSG00000266126	RP11-209D14.4	-0.441	-0.923	-2.079	0.041
ENSG00000266173	STRADA	-0.341	4.500	-3.209	0.002
ENSG00000266187	RN7SL480P	1.384	-3.605	2.877	0.005
ENSG00000266304	RP11-484N16.1	-0.789	-2.865	-2.411	0.018
ENSG00000266306	CTD-2008P7.6	-0.697	-1.522	-2.219	0.029
ENSG00000266369	RP11-344E13.4	-0.668	-0.015	-2.758	0.007
ENSG00000266371	RP11-142O6.1	-0.540	0.990	-3.174	0.002
ENSG00000266401	RP11-874J12.4	-0.466	2.796	-3.248	0.002
ENSG00000266412	NCOA4	0.214	6.925	2.673	0.009
ENSG00000266466	RP11-822E23.2	-0.504	-0.748	-2.512	0.014
ENSG00000266472	MRPS21	0.237	5.710	2.380	0.020
ENSG00000266513	RP11-874J12.3	-0.451	0.503	-2.428	0.017
ENSG00000266554	LINC01443	-0.773	-1.484	-2.872	0.005
ENSG00000266673	RP11-822E23.3	-0.954	-2.232	-2.134	0.036
ENSG00000266696	RP11-30L3.2	-0.444	-0.116	-2.939	0.004
ENSG00000266805	RP11-61L19.1	-0.532	0.732	-2.317	0.023
ENSG00000266846	RP11-793A3.2	-0.395	-1.094	-2.318	0.023
ENSG00000266896	RP1-266L20.9	-0.532	-0.919	-2.561	0.012
ENSG00000266910	CTC-448F2.6	-0.384	1.561	-2.823	0.006
ENSG00000266932	AC006116.13	-0.586	-0.283	-3.388	0.001
ENSG00000266977	CTC-459F4.5	-0.550	-1.710	-2.100	0.039
ENSG00000266988	RP11-749H17.1	-0.705	-1.995	-2.098	0.039
ENSG00000266993	RP4-657D16.3	-0.516	0.211	-2.762	0.007
ENSG00000267002	RP11-242D8.1	-0.275	4.451	-2.825	0.006
ENSG00000267034	RP11-384O8.1	0.242	2.514	2.322	0.023
ENSG00000267039	RP11-58G13.1	-0.622	-0.486	-3.079	0.003
ENSG00000267056	AC005336.4	-0.849	0.903	-3.268	0.002
ENSG00000267107	PCAT19	0.420	-0.135	2.014	0.047
ENSG00000267128	RP11-449J21.5	-0.488	1.378	-3.612	0.001
ENSG00000267131	RP11-332H18.5	-0.966	-2.182	-3.297	0.001
ENSG00000267136	RP11-53B2.3	-0.380	3.045	-2.628	0.010
ENSG00000267150	RP11-411B10.2	-0.521	-1.079	-2.101	0.039
ENSG00000267199	RP11-861E21.2	-0.526	1.325	-3.532	0.001
ENSG00000267203	SNRPGP4	-0.664	1.256	-3.285	0.002
ENSG00000267207	RP11-264B14.1	-1.030	-2.546	-3.784	0.000
ENSG00000267311	RP11-99A1.2	-0.695	-0.758	-2.810	0.006
ENSG00000267376	ATP5LP6	-0.738	-2.781	-2.170	0.033
ENSG00000267463	UBE2V2P2	-0.658	-1.504	-2.101	0.039
ENSG00000267503	RP11-53B2.4	-0.427	0.888	-2.731	0.008
ENSG00000267504	RP11-845C23.2	-0.736	-0.557	-3.115	0.003

ENSG00000267508	ZNF285	0.139	3.707	2.366	0.020
ENSG00000267515	RP11-861E21.3	-0.682	-1.245	-2.737	0.008
ENSG00000267524	RPSAP57	-0.679	-0.199	-3.395	0.001
ENSG00000267529	RP11-53B2.1	-0.354	1.354	-2.173	0.033
ENSG00000267560	RP11-173A16.1	-0.338	0.895	-2.055	0.043
ENSG00000267585	RP11-115P21.1	-0.651	-0.856	-2.819	0.006
ENSG00000267590	NDUFA3P1	-0.624	-1.757	-2.316	0.023
ENSG00000267606	AC006116.21	-0.659	-1.869	-2.878	0.005
ENSG00000267632	RP11-400F19.18	-0.313	2.603	-2.358	0.021
ENSG00000267636	AC008992.1	-0.838	-2.380	-2.509	0.014
ENSG00000267638	RP11-546M21.6	-0.742	-2.741	-2.105	0.038
ENSG00000267640	CTD-2554C21.2	0.327	2.881	3.061	0.003
ENSG00000267666	AC004156.3	-0.773	-1.632	-2.871	0.005
ENSG00000267690	LDLRAD4-AS1	-0.541	0.948	-3.000	0.004
ENSG00000267694	RP11-691H4.4	-0.415	0.076	-2.488	0.015
ENSG00000267702	RP11-53B2.2	-0.335	2.493	-2.256	0.027
ENSG00000267710	AC006116.20	-0.469	1.259	-3.220	0.002
ENSG00000267724	RP11-49K24.8	-0.293	1.180	-2.871	0.005
ENSG00000267733	RP11-64C12.3	-0.621	-0.219	-2.408	0.018
ENSG00000267734	RP4-604K5.3	-0.590	-0.068	-2.853	0.005
ENSG00000267766	RP11-299P2.1	-0.895	-1.703	-3.143	0.002
ENSG00000267783	CTB-55O6.10	0.990	-2.982	2.789	0.007
ENSG00000267787	RP11-35G9.5	-0.435	0.534	-3.172	0.002
ENSG00000267799	MAN1A2P1	0.372	0.182	2.157	0.034
ENSG00000267800	RP11-49K24.5	-0.629	-1.473	-2.757	0.007
ENSG00000267904	CTC-429P9.5	0.190	1.885	2.045	0.044
ENSG00000267934	CTB-176F20.3	-0.513	3.732	-3.063	0.003
ENSG00000268038	LINC01785	-0.955	-0.670	-2.800	0.006
ENSG00000268043	NBPF12	-0.354	4.574	-2.045	0.044
ENSG00000268055	AC067969.2	-0.948	-0.583	-2.645	0.010
ENSG00000268058	BNIP3P40	-0.545	-0.002	-3.117	0.003
ENSG00000268067	OR5AH1P	-0.550	-0.869	-2.250	0.027
ENSG00000268093	AC022154.7	-0.462	-0.880	-2.007	0.048
ENSG00000268119	CTD-2561J22.5	-0.437	2.896	-2.449	0.016
ENSG00000268182	SMIM17	0.330	2.836	2.458	0.016
ENSG00000268257	AIRN	-0.423	0.028	-2.261	0.026
ENSG00000268279	RP11-434D12.1	-0.697	-2.116	-2.773	0.007
ENSG00000268362	CTD-2017D11.1	-0.298	2.192	-2.169	0.033
ENSG00000268364	SMC5-AS1	-0.297	0.829	-2.365	0.020
ENSG00000268388	FENDRR	0.647	0.523	2.886	0.005
ENSG00000268442	CTD-2027I19.2	-0.362	0.917	-2.449	0.016
ENSG00000268471	MIR4453	-0.194	2.532	-2.378	0.020
ENSG00000268472	RP11-356J5.13	-0.706	-1.489	-2.824	0.006
ENSG00000268573	RP11-158H5.7	-0.296	1.547	-2.071	0.041
ENSG00000268584	RP11-464F9.20	0.401	-1.069	2.005	0.048
ENSG00000268658	LINC00664	-0.664	0.530	-2.290	0.025
ENSG00000268686	AC010524.2	0.700	-0.713	2.709	0.008

ENSG00000268717	BNIP3P33	-1.433	-1.189	-3.732	0.000
ENSG00000268830	BNIP3P32	-0.938	-0.295	-2.252	0.027
ENSG00000268873	RP11-138H11.1	-0.914	-0.470	-3.899	0.000
ENSG00000269138	ZNF209P	-0.490	1.356	-2.152	0.034
ENSG00000269176	RP11-727F15.12	0.412	-0.674	2.252	0.027
ENSG00000269246	CTC-246B18.10	0.699	-2.129	2.222	0.029
ENSG00000269343	ZNF587B	-0.240	3.494	-2.212	0.030
ENSG00000269514	RP11-370I10.12	-0.389	1.896	-2.444	0.017
ENSG00000269570	CMB9-55A18.1	-0.343	0.564	-2.333	0.022
ENSG00000269752	CTD-3149D2.3	-0.666	-2.305	-2.107	0.038
ENSG00000269845	RP11-420K14.6	-0.533	0.320	-2.366	0.020
ENSG00000269887	RP11-477H21.2	-0.460	1.187	-2.938	0.004
ENSG00000269893	SNHG8	0.431	2.268	2.737	0.008
ENSG00000269935	RP11-482M8.3	-0.370	0.184	-2.056	0.043
ENSG00000269938	RP11-214K3.20	-0.496	2.378	-3.441	0.001
ENSG00000269944	RP11-770J1.7	-0.521	-0.201	-2.550	0.013
ENSG00000269945	RP6-91H8.5	-0.306	2.136	-2.131	0.036
ENSG00000269950	AP000962.2	-0.653	1.012	-3.596	0.001
ENSG00000269951	RP11-797A18.6	-0.437	1.910	-3.137	0.002
ENSG00000269978	RP11-338N10.3	-0.403	-1.612	-2.011	0.048
ENSG00000269985	RP1-232P20.1	-0.536	-0.041	-2.881	0.005
ENSG00000269997	RP11-214K3.21	-0.534	1.920	-3.638	0.000
ENSG00000270000	RP3-449M8.9	-0.836	-2.516	-2.517	0.014
ENSG00000270011	ZNF559-ZNF177	-0.378	-0.086	-2.466	0.016
ENSG00000270048	RP11-214K3.22	-0.547	0.520	-2.591	0.011
ENSG00000270061	RP11-214K3.19	-0.389	1.159	-2.429	0.017
ENSG00000270071	AP001172.2	-0.449	1.510	-3.021	0.003
ENSG00000270095	RP11-214K3.18	-0.625	0.723	-3.893	0.000
ENSG00000270098	LDHAL6EP	-0.812	-0.870	-2.968	0.004
ENSG00000270111	RP11-271F18.4	0.522	1.687	3.266	0.002
ENSG00000270116	AP001429.1	-0.450	0.746	-2.712	0.008
ENSG00000270127	RP11-526I2.5	0.477	0.451	2.315	0.023
ENSG00000270130	RP11-214K3.23	-0.549	1.307	-3.553	0.001
ENSG00000270133	CTC-303L1.2	-0.376	-0.277	-2.177	0.032
ENSG00000270139	AP001172.3	-0.388	0.249	-2.019	0.047
ENSG00000270174	RP1-256G22.2	-0.320	0.369	-2.026	0.046
ENSG00000270207	RP11-157E16.1	-0.439	-0.281	-2.182	0.032
ENSG00000270218	RP11-325L12.5	-0.527	-0.312	-2.469	0.016
ENSG00000270257	RP11-17P16.2	-0.712	-2.396	-2.139	0.035
ENSG00000270269	IMMP1LP1	-0.783	-1.947	-2.610	0.011
ENSG00000270285	RP11-1029M24.4	-0.730	-2.290	-2.541	0.013
ENSG00000270300	PHACTR2P1	-0.540	0.366	-3.501	0.001
ENSG00000270317	AC004004.2	-0.859	-1.223	-2.071	0.042
ENSG00000270344	RP11-734K2.4	-0.378	3.861	-3.339	0.001
ENSG00000270402	CTC-412M14.5	-0.349	0.279	-2.150	0.034
ENSG00000270426	RP11-326I11.5	-0.338	0.465	-2.125	0.037
ENSG00000270427	NRBF2P5	-0.629	-0.999	-2.933	0.004

ENSG00000270462	RP11-342K6.3	-0.522	-0.368	-2.941	0.004
ENSG00000270480	RP11-57B24.1	-0.309	0.655	-2.014	0.047
ENSG00000270510	RP11-777F6.3	-0.270	0.539	-2.087	0.040
ENSG00000270528	RP11-674I16.2	-0.913	-1.223	-4.201	0.000
ENSG00000270540	RP11-785G17.1	-0.913	-1.914	-3.145	0.002
ENSG00000270562	RP11-154H23.3	-0.391	1.153	-2.495	0.015
ENSG00000270571	RP11-355F16.1	-0.494	-0.335	-2.482	0.015
ENSG00000270574	RP11-171I2.2	-0.560	-1.382	-2.784	0.007
ENSG00000270575	RP11-21M7.2	-0.471	-0.385	-2.385	0.019
ENSG00000270585	RP11-568G11.4	-0.671	0.362	-3.505	0.001
ENSG00000270589	RP11-348N5.7	-0.463	1.846	-3.433	0.001
ENSG00000270604	HCG17	-0.269	0.904	-2.260	0.027
ENSG00000270614	CTC-325H20.7	-0.578	0.326	-2.471	0.016
ENSG00000270620	RP1-134O19.3	-0.536	-1.958	-2.025	0.046
ENSG00000270634	RP4-777G9.1	-0.694	-1.782	-2.587	0.011
ENSG00000270681	RP11-372K14.2	-0.272	1.274	-2.173	0.033
ENSG00000270696	RP11-342K6.1	-0.265	4.101	-2.832	0.006
ENSG00000270728	RP4-657E11.10	-0.432	0.901	-3.163	0.002
ENSG00000270742	RP4-802A10.1	-0.723	0.062	-2.852	0.006
ENSG00000270764	CTB-152G17.5	-1.098	-2.244	-3.288	0.001
ENSG00000270772	PDHA1P1	-0.606	-1.560	-2.496	0.015
ENSG00000270808	RP11-574K11.27	-0.765	-2.424	-2.666	0.009
ENSG00000270814	TMEM261P1	-0.518	2.763	-3.655	0.000
ENSG00000270863	DDX55P1	-0.492	1.990	-3.147	0.002
ENSG00000270874	RP11-420K10.1	-0.728	-0.015	-3.544	0.001
ENSG00000270885	RASL10B	0.290	4.575	2.050	0.044
ENSG00000270893	RP11-311B14.1	-0.460	-0.792	-2.212	0.030
ENSG00000270900	NUDT19P4	-0.474	-1.066	-2.100	0.039
ENSG00000270903	HNRNPA3P9	-0.564	0.267	-2.183	0.032
ENSG00000270915	RP11-27M5.1	-0.752	-1.889	-2.773	0.007
ENSG00000270923	TAS2R6P	-0.652	0.082	-3.099	0.003
ENSG00000270941	RP5-966M1.5	-0.690	-1.722	-2.790	0.007
ENSG00000270947	AC025811.3	-0.779	0.351	-2.770	0.007
ENSG00000270959	LPP-AS2	-0.376	0.970	-2.451	0.016
ENSG00000270965	RP11-169K19.1	-0.607	-0.421	-2.241	0.028
ENSG00000270986	RP11-123C21.2	-0.409	-0.220	-2.208	0.030
ENSG00000270988	RP11-439C15.5	-0.636	-1.140	-2.461	0.016
ENSG00000270994	RP11-293B7.1	-0.767	-1.338	-3.182	0.002
ENSG00000271070	GMCL1P2	-0.557	-1.783	-2.209	0.030
ENSG00000271096	SUMO2P14	-0.511	-1.669	-2.014	0.047
ENSG00000271100	RP11-697H9.5	-0.630	-0.516	-2.243	0.028
ENSG00000271146	RP11-479F13.1	-0.613	-1.151	-2.177	0.032
ENSG00000271159	HINT1P2	-0.686	-1.091	-3.042	0.003
ENSG00000271204	RP11-138A9.1	-0.638	3.888	-4.167	0.000
ENSG00000271259	RP11-1109F11.5	-0.512	-0.843	-2.428	0.017
ENSG00000271264	MZT1P1	-0.577	-0.982	-2.207	0.030
ENSG00000271277	RP11-82O2.2	-0.474	-0.592	-2.038	0.045

ENSG00000271278	TCEB1P33	-0.767	-1.796	-3.122	0.002
ENSG00000271314	RP11-435O5.6	-0.674	-1.227	-3.183	0.002
ENSG00000271327	RP11-1109F11.3	-0.325	0.887	-2.295	0.024
ENSG00000271356	RP11-335E8.1	-0.571	-0.086	-2.629	0.010
ENSG00000271386	SRGNP1	-1.191	-2.400	-2.937	0.004
ENSG00000271401	RP11-171I2.3	-0.403	0.815	-2.788	0.007
ENSG00000271428	RP1-224A6.8	-0.647	-0.325	-3.037	0.003
ENSG00000271461	RP4-673D20.6	-0.416	3.030	-2.111	0.038
ENSG00000271553	RP11-274B21.10	-0.554	1.018	-3.288	0.001
ENSG00000271554	RP4-665N4.8	-0.523	0.169	-2.966	0.004
ENSG00000271590	RP11-181E10.3	-0.428	-0.093	-2.038	0.045
ENSG00000271639	RP11-289A15.1	-0.418	0.288	-2.071	0.042
ENSG00000271659	RP11-435O5.4	-0.397	-0.744	-2.243	0.028
ENSG00000271662	RP11-141C7.3	-0.666	-0.745	-2.271	0.026
ENSG00000271664	RP4-800G7.3	-0.390	0.124	-2.328	0.022
ENSG00000271710	NDUFA8P1	-0.621	-1.668	-2.358	0.021
ENSG00000271730	RP11-787I22.3	-0.586	-2.070	-2.067	0.042
ENSG00000271732	RP5-1182A14.5	-0.962	-2.751	-2.695	0.009
ENSG00000271761	XXbac-BPGBPG55C	-0.543	-0.624	-2.014	0.047
ENSG00000271778	RP11-379F4.8	-0.526	0.705	-2.986	0.004
ENSG00000271780	RP11-1017G21.5	0.297	1.423	2.390	0.019
ENSG00000271789	RP5-1112D6.7	-0.397	1.319	-2.931	0.004
ENSG00000271795	CTC-251D13.1	-0.342	0.776	-2.070	0.042
ENSG00000271811	RP1-79C4.4	-0.430	0.307	-2.297	0.024
ENSG00000271817	U3	-0.991	-1.846	-3.788	0.000
ENSG00000271828	CTD-2310F14.1	-0.388	0.334	-2.284	0.025
ENSG00000271833	RP11-356B19.11	-0.524	0.924	-2.926	0.004
ENSG00000271843	RP11-245J9.5	-0.386	-0.188	-2.275	0.026
ENSG00000271904	CTC-498M16.4	-0.568	-0.602	-2.404	0.018
ENSG00000271916	RP11-884K10.6	-0.615	-0.911	-2.399	0.019
ENSG00000271917	RP11-506O24.2	-0.537	0.354	-3.304	0.001
ENSG00000271919	RP11-269G24.8	-0.682	-0.873	-2.868	0.005
ENSG00000271933	RP11-338I21.1	-0.292	0.506	-2.074	0.041
ENSG00000271966	RP11-7F18.2	-0.599	-1.628	-2.207	0.030
ENSG00000272009	RP1-313I6.12	-0.536	-1.474	-2.543	0.013
ENSG00000272031	ANKRD34A	-0.256	3.869	-1.995	0.049
ENSG00000272037	KB-431C1.5	-0.566	0.901	-3.134	0.002
ENSG00000272047	GTF2H5	0.186	5.659	2.107	0.038
ENSG00000272054	RP11-423P10.2	-0.362	2.219	-3.020	0.003
ENSG00000272070	AC005618.6	-0.229	1.821	-2.126	0.037
ENSG00000272072	CTA-363E19.2	-0.360	1.800	-3.062	0.003
ENSG00000272077	RP11-348P10.2	-0.277	1.873	-2.882	0.005
ENSG00000272103	CTD-2653M23.3	-0.445	0.657	-2.196	0.031
ENSG00000272143	FGF14-AS2	-0.421	2.735	-3.010	0.003
ENSG00000272167	PROX1-AS1	-0.539	-1.016	-2.321	0.023
ENSG00000272211	RP11-347P5.1	-0.682	-0.634	-2.028	0.046
ENSG00000272215	U7	-0.910	-1.934	-3.066	0.003

ENSG00000272249	KB-1958F4.2	-0.683	-1.281	-2.551	0.013
ENSG00000272254	RP11-531A24.7	-0.600	-1.482	-2.330	0.022
ENSG00000272316	XXbac-BPGBPG55C	-0.399	4.145	-2.829	0.006
ENSG00000272320	RP11-506K6.4	-0.298	2.293	-2.124	0.037
ENSG00000272341	RP1-151F17.2	-0.348	2.743	-2.851	0.006
ENSG00000272343	RP11-140I16.3	0.365	0.467	2.511	0.014
ENSG00000272356	RP5-1112D6.8	-0.323	2.160	-2.700	0.008
ENSG00000272371	RP11-25O10.2	-0.457	-2.199	-2.000	0.049
ENSG00000272374	RP3-329A5.8	-0.428	1.683	-2.612	0.011
ENSG00000272381	RP11-192P3.4	-0.499	-0.709	-2.390	0.019
ENSG00000272383	RP11-556O9.4	-0.503	-0.124	-2.490	0.015
ENSG00000272391	POM121C	-0.346	4.603	-2.547	0.013
ENSG00000272397	RP11-497D6.5	-0.597	1.573	-2.884	0.005
ENSG00000272425	RP11-363E6.4	-0.378	0.935	-3.131	0.002
ENSG00000272444	RP11-1017G21.6	0.353	0.368	2.581	0.012
ENSG00000272465	RP1-136B1.1	-0.528	-1.040	-2.157	0.034
ENSG00000272469	RN7SL760P	-0.568	-1.611	-2.319	0.023
ENSG00000272483	RP11-169K17.3	-0.781	-2.016	-3.076	0.003
ENSG00000272485	RP11-284J1.1	-0.494	-0.112	-2.671	0.009
ENSG00000272505	RP11-981G7.6	-0.344	4.856	-2.693	0.009
ENSG00000272514	CFAP206	-0.433	-0.695	-2.191	0.031
ENSG00000272529	RP11-415F23.4	-0.608	0.514	-3.412	0.001
ENSG00000272542	RP11-255P5.2	-0.538	0.440	-2.447	0.017
ENSG00000272604	RP11-251G23.5	-0.267	1.431	-2.032	0.045
ENSG00000272610	MAGI1-IT1	-0.628	1.034	-3.317	0.001
ENSG00000272627	RP11-354E23.5	-0.589	-1.266	-2.049	0.044
ENSG00000272630	RP11-344N10.5	-0.342	1.043	-2.422	0.018
ENSG00000272631	RP11-359E3.4	-0.356	1.643	-2.904	0.005
ENSG00000272650	RP11-571I18.4	-0.455	0.069	-3.141	0.002
ENSG00000272654	RP11-422P24.11	0.344	-0.413	2.280	0.025
ENSG00000272663	RP11-191L17.1	0.475	-0.842	2.468	0.016
ENSG00000272711	RP11-259N19.1	1.026	-2.500	2.528	0.013
ENSG00000272732	RP5-1159O4.2	-0.542	1.447	-3.888	0.000
ENSG00000272746	RP11-53B2.6	-0.413	0.329	-2.417	0.018
ENSG00000272750	RP11-378J18.8	-0.289	1.773	-2.651	0.010
ENSG00000272758	RP11-299J3.8	-0.329	0.976	-2.529	0.013
ENSG00000272779	LL22NC03-80A10.6	0.292	4.827	3.038	0.003
ENSG00000272841	RP3-428L16.2	0.399	1.603	3.313	0.001
ENSG00000272856	RP11-710E1.2	-0.753	-2.610	-2.320	0.023
ENSG00000272871	RP11-408A13.4	-0.351	1.512	-2.863	0.005
ENSG00000272886	DCP1A	-0.093	5.018	-2.127	0.036
ENSG00000272894	RP5-1159O4.1	-0.340	2.448	-2.337	0.022
ENSG00000272909	CTD-2555O16.4	-0.300	1.522	-2.843	0.006
ENSG00000272912	RP11-724N1.1	-0.876	-1.908	-3.079	0.003
ENSG00000272914	RP11-330O11.3	-0.501	-0.418	-2.888	0.005
ENSG00000272979	RP11-647K16.1	-0.487	-1.327	-2.265	0.026
ENSG00000272983	RP11-508N22.12	-0.353	3.845	-2.920	0.005

ENSG00000273007	RP11-170N16.3	-0.376	0.619	-2.720	0.008
ENSG00000273018	CTD-2303H24.2	-0.821	2.768	-2.380	0.020
ENSG00000273055	CTB-13F3.1	-0.464	-0.764	-2.237	0.028
ENSG00000273091	AP000255.6	-0.595	-0.493	-2.712	0.008
ENSG00000273139	XXbac-B444P24.14	0.627	-1.336	2.982	0.004
ENSG00000273173	SNURF	-0.404	2.699	-2.968	0.004
ENSG00000273183	RP11-5C23.2	-0.349	-0.410	-2.010	0.048
ENSG00000273198	GS1-304P7.3	-0.567	-1.405	-2.450	0.016
ENSG00000273238	RP11-1263C18.1	0.318	3.671	2.926	0.004
ENSG00000273245	RP11-434P11.2	-0.478	0.314	-2.898	0.005
ENSG00000273247	RP11-83A24.2	-0.284	3.419	-2.780	0.007
ENSG00000273264	RP11-131L23.2	-0.517	-1.803	-2.108	0.038
ENSG00000273265	RP11-353K11.1	-0.834	-1.667	-3.121	0.002
ENSG00000273291	RP11-136C24.3	-0.728	-1.440	-2.790	0.007
ENSG00000273294	C1QTNF3-AMACR	-0.916	-1.996	-2.680	0.009
ENSG00000273306	RP11-527J8.1	-0.316	0.410	-2.367	0.020
ENSG00000273312	RP11-425A6.5	0.327	0.562	2.161	0.034
ENSG00000273319	RP11-138A9.2	-0.422	4.427	-2.663	0.009
ENSG00000273328	RP11-141M3.6	-0.774	-2.140	-2.066	0.042
ENSG00000273372	SFTPD-AS1	-0.762	-0.075	-3.573	0.001
ENSG00000273373	RP5-1074L1.4	-0.344	2.949	-2.866	0.005
ENSG00000273381	RP11-305L7.7	-0.404	0.956	-2.810	0.006
ENSG00000273384	RP5-1098D14.1	-0.728	-1.996	-2.288	0.025
ENSG00000273442	AC006946.17	-0.628	-0.613	-2.969	0.004
ENSG00000273444	RP5-1147A1.2	-1.351	-3.416	-3.126	0.002
ENSG00000273447	AC004067.5	0.531	-1.157	2.532	0.013
ENSG00000273448	RP11-166O4.6	-0.444	1.549	-2.914	0.005
ENSG00000273451	RP4-569M23.4	-0.486	0.659	-2.979	0.004
ENSG00000273476	RP11-108L7.14	-0.902	-2.274	-2.792	0.007
ENSG00000273486	RP11-731C17.2	-0.468	1.036	-3.814	0.000
ENSG00000273507	RP11-50D16.4	-0.562	0.002	-2.145	0.035
ENSG00000273559	CWC25	0.162	4.061	2.345	0.021
ENSG00000273597	RP11-392A14.9	-0.848	-1.816	-2.918	0.005
ENSG00000273604	C17orf96	0.393	3.050	2.563	0.012
ENSG00000273611	ZNHIT3	0.364	4.257	3.317	0.001
ENSG00000273659	RP11-58C22.2	-0.698	-2.149	-2.457	0.016
ENSG00000273749	CYFIP1	0.243	5.812	2.477	0.015
ENSG00000273762	VN1R76P	-0.704	-0.438	-3.563	0.001
ENSG00000273765	RP11-370I10.11	-0.510	-0.779	-2.122	0.037
ENSG00000273792	RP11-554D20.2	-0.492	0.839	-2.472	0.016
ENSG00000273982	RP11-332H18.7	-1.032	-2.935	-2.692	0.009
ENSG00000273989	RP11-425D17.2	-0.784	-1.073	-3.149	0.002
ENSG00000274020	LINC01138	-0.349	2.823	-3.013	0.003
ENSG00000274031	RP11-413H22.3	-0.545	-0.055	-3.534	0.001
ENSG00000274038	RP11-66H6.4	-0.658	-2.291	-2.140	0.035
ENSG00000274064	RP11-570L14.1	-0.970	-2.753	-2.285	0.025
ENSG00000274093	RP11-140H17.2	-0.387	0.375	-2.231	0.028

ENSG00000274105	RP11-278C7.3	-0.385	0.510	-2.346	0.021
ENSG00000274118	Y_RNA	-0.610	0.057	-2.303	0.024
ENSG00000274124	RP11-474C8.8	-1.132	-2.845	-3.222	0.002
ENSG00000274210	RNVU1-27	0.778	-0.764	2.829	0.006
ENSG00000274238	RP11-115D19.3	-0.613	-0.883	-2.882	0.005
ENSG00000274253	RP11-566K19.6	0.262	3.587	2.467	0.016
ENSG00000274265	CH17-189H20.1	-0.328	1.457	-2.028	0.046
ENSG00000274308	RP11-19G24.1	-0.527	-0.783	-2.059	0.043
ENSG00000274317	LINC02334	-0.447	-0.478	-2.160	0.034
ENSG00000274331	RP11-403A3.1	0.636	-1.109	2.118	0.037
ENSG00000274346	RP11-40B20.1	-0.766	-1.086	-3.376	0.001
ENSG00000274357	Metazoa_SRP	1.613	-2.903	2.464	0.016
ENSG00000274363	RP11-104O19.4	-0.567	-1.593	-2.390	0.019
ENSG00000274397	CTD-2540L5.9	-0.400	1.860	-2.756	0.007
ENSG00000274421	RP11-386J22.3	-0.504	0.486	-2.994	0.004
ENSG00000274461	RP11-323P17.2	-0.346	0.998	-2.176	0.032
ENSG00000274551	RP11-434C1.6	-0.639	-0.017	-2.713	0.008
ENSG00000274554	RP11-240G22.5	-0.436	0.782	-3.084	0.003
ENSG00000274578	RP11-430E17.1	-0.408	1.649	-3.060	0.003
ENSG00000274614	RP11-17E4.1	-0.585	0.170	-3.157	0.002
ENSG00000274654	CTD-3247H4.2	-0.738	-0.571	-3.614	0.001
ENSG00000274677	RP11-505K9.5	-0.818	-0.822	-3.216	0.002
ENSG00000274712	RP11-147L13.15	-0.273	2.928	-3.037	0.003
ENSG00000274756	CTB-75G16.1	-0.552	-0.363	-2.564	0.012
ENSG00000274759	RNA5SP527	-0.879	-0.797	-3.434	0.001
ENSG00000274767	AC131056.3	-1.003	-1.353	-2.480	0.015
ENSG00000274769	RP11-493E12.3	-0.516	-1.607	-2.371	0.020
ENSG00000274776	RP11-687F6.5	-0.335	2.299	-3.004	0.004
ENSG00000274949	RP5-1009E24.9	0.867	-3.129	2.469	0.016
ENSG00000274964	RP11-817I4.1	-0.607	2.066	-4.098	0.000
ENSG00000274987	RP11-295I5.3	-0.312	0.553	-2.182	0.032
ENSG00000275005	RP11-234G16.5	-0.661	-1.516	-2.304	0.024
ENSG00000275017	RP11-317B17.4	-0.306	0.937	-2.121	0.037
ENSG00000275029	HMGB1P24	-0.314	0.883	-2.209	0.030
ENSG00000275052	PPP4R3B	0.137	6.906	2.401	0.019
ENSG00000275056	RP11-127I20.8	-0.392	0.141	-2.204	0.030
ENSG00000275131	CH17-472G23.4	-0.408	6.621	-2.385	0.019
ENSG00000275191	RP11-36I17.2	-0.481	-1.223	-2.206	0.030
ENSG00000275227	uc_338	-0.373	-0.609	-2.151	0.034
ENSG00000275294	LINC02340	-0.414	-1.037	-2.218	0.029
ENSG00000275332	CTD-2017F17.1	-0.847	-2.813	-2.244	0.028
ENSG00000275367	RP11-266K4.14	-0.317	3.859	-2.308	0.024
ENSG00000275377	MIR1299	-0.520	-1.699	-2.249	0.027
ENSG00000275381	RP11-401N16.2	-0.679	-1.421	-2.379	0.020
ENSG00000275383	RP11-615I2.6	-0.518	1.727	-2.802	0.006
ENSG00000275413	CTC-529I10.1	-0.631	0.452	-3.688	0.000
ENSG00000275427	CTD-2281E23.1	-0.430	2.186	-2.047	0.044

ENSG00000275465	RP11-569G13.3	-0.578	-0.599	-2.609	0.011
ENSG00000275481	RP11-474P2.6	-0.446	1.215	-2.669	0.009
ENSG00000275540	CTC-559E9.12	0.531	-1.746	2.249	0.027
ENSG00000275576	RP5-836N17.4	-0.589	-1.918	-2.085	0.040
ENSG00000275700	AATF	0.204	4.744	2.504	0.014
ENSG00000275720	CTB-75G16.3	-0.745	-2.761	-2.173	0.033
ENSG00000275734	RP11-368I7.6	-0.443	0.716	-2.353	0.021
ENSG00000275759	RP11-131L12.3	-0.459	0.597	-2.387	0.019
ENSG00000275769	RP11-771K4.3	-0.485	-0.686	-2.353	0.021
ENSG00000275803	RN7SL736P	0.920	-0.961	2.194	0.031
ENSG00000275805	RP11-349H17.2	-0.421	0.328	-2.412	0.018
ENSG00000275839	CTC-421K24.1	-0.606	-0.766	-2.737	0.008
ENSG00000275953	RP11-753G20.2	-0.923	-2.945	-2.313	0.023
ENSG00000275981	AC018816.1	-0.639	-1.861	-2.448	0.017
ENSG00000276023	DUSP14	0.201	3.440	2.196	0.031
ENSG00000276030	CTD-2027I19.3	-0.366	-0.802	-2.040	0.045
ENSG00000276107	CTD-2033D15.2	-0.710	-1.561	-2.866	0.005
ENSG00000276115	RP11-817I4.2	-0.437	1.753	-3.301	0.001
ENSG00000276148	RP11-278C7.4	-0.614	-1.175	-3.443	0.001
ENSG00000276179	DLG2-AS1_2	-0.561	-0.124	-2.937	0.004
ENSG00000276180	HIST1H4I	0.571	0.769	3.587	0.001
ENSG00000276214	CTD-2146O16.1	-0.488	0.555	-2.919	0.005
ENSG00000276281	RN7SL126P	0.945	-3.370	2.249	0.027
ENSG00000276334	AL133243.1	-0.381	2.759	-2.538	0.013
ENSG00000276337	RP11-106D4.2	-0.508	0.903	-3.227	0.002
ENSG00000276390	RP1-197B17.5	-0.649	-1.663	-2.739	0.008
ENSG00000276393	RP11-106D4.3	-0.564	-1.036	-2.152	0.034
ENSG00000276417	RP11-266K4.13	-0.592	2.313	-3.678	0.000
ENSG00000276449	AC004076.5	-0.345	2.611	-2.930	0.004
ENSG00000276517	AL133243.2	-0.347	3.290	-2.717	0.008
ENSG00000276542	RP11-115D19.2	-0.697	0.444	-2.981	0.004
ENSG00000276545	PCDHGB9P	-0.478	1.262	-3.072	0.003
ENSG00000276547	PCDHGB5	-0.189	2.836	-2.073	0.041
ENSG00000276578	LLNLR-285B5.1	-0.507	-0.525	-2.771	0.007
ENSG00000276588	RN7SL322P	1.039	-3.301	2.083	0.040
ENSG00000276591	GXYLT1P5	-0.660	2.122	-2.294	0.024
ENSG00000276649	RP5-858L17.1	-0.321	2.693	-2.125	0.037
ENSG00000276656	MIR6083	-0.705	-2.548	-2.323	0.023
ENSG00000276662	RP11-631M21.7	-0.441	-0.323	-2.136	0.036
ENSG00000276702	RP11-3D4.4	-0.420	-1.400	-2.007	0.048
ENSG00000276704	RP11-128N14.4	-0.789	-1.169	-3.303	0.001
ENSG00000276740	RP11-381O6.1	-0.398	1.970	-2.107	0.038
ENSG00000276759	RP11-80I3.1	-1.358	-2.167	-3.981	0.000
ENSG00000276805	RP11-291L22.9	-0.371	1.302	-2.123	0.037
ENSG00000276809	RP11-10E18.7	0.314	0.821	2.204	0.030
ENSG00000276868	RP11-318K12.3	-0.546	-1.635	-2.183	0.032
ENSG00000276916	RP11-230F18.6	-0.593	-0.495	-2.926	0.004

ENSG00000276925	RP11-708J19.3	-0.453	-1.347	-2.052	0.043
ENSG00000276931	RP11-161M6.6	-0.441	-0.839	-2.468	0.016
ENSG00000276966	HIST1H4E	0.360	5.461	2.053	0.043
ENSG00000276975	HYDIN2	-0.478	5.428	-2.609	0.011
ENSG00000277062	RN7SL88P	0.689	-0.765	2.159	0.034
ENSG00000277136	Metazoa_SRP	1.411	-3.733	2.648	0.010
ENSG00000277151	RP11-380B4.3	-0.349	2.546	-2.884	0.005
ENSG00000277171	RP11-191H23.1	-0.657	0.235	-3.147	0.002
ENSG00000277173	CTD-2024F21.1	-0.805	-2.749	-2.089	0.040
ENSG00000277224	HIST1H2BF	0.246	1.760	2.020	0.047
ENSG00000277258	PCGF2	-0.326	1.734	-2.656	0.009
ENSG00000277290	RP11-326C3.16	-0.464	-0.373	-2.197	0.031
ENSG00000277301	RP5-1184F4.7	-0.572	-1.189	-2.316	0.023
ENSG00000277342	RP11-843B15.4	-0.530	0.357	-3.461	0.001
ENSG00000277481	PKD1L3	-0.389	-0.149	-2.019	0.047
ENSG00000277500	RP11-456J20.2	-0.728	-0.842	-2.995	0.004
ENSG00000277534	RP11-9E17.1	-0.344	3.696	-3.299	0.001
ENSG00000277548	RP11-139H15.6	-0.576	-0.030	-3.208	0.002
ENSG00000277566	RP11-545P7.9	-0.596	-1.476	-2.174	0.033
ENSG00000277589	RP11-333J10.3	-1.216	-1.652	-4.567	0.000
ENSG00000277595	RP11-946P6.6	-0.452	-0.388	-2.590	0.011
ENSG00000277695	RP11-115D19.4	-0.622	-1.268	-2.384	0.019
ENSG00000277705	RP11-434C1.5	-0.907	-1.409	-3.478	0.001
ENSG00000277728	RP11-143K11.7	-0.485	-1.215	-2.089	0.040
ENSG00000277769	RBM22P4	-0.774	-0.154	-4.106	0.000
ENSG00000277778	PGM5P2	-0.281	2.497	-2.009	0.048
ENSG00000277791	PSMB3	0.339	3.664	2.475	0.015
ENSG00000277795	AC005752.10	-0.437	0.518	-2.778	0.007
ENSG00000277829	RP5-1054A22.4	-0.455	-0.040	-1.997	0.049
ENSG00000277840	RP11-103B5.4	-0.389	0.609	-2.040	0.045
ENSG00000277972	CISD3	0.360	2.191	2.888	0.005
ENSG00000277977	CTD-3018O17.5	0.223	1.492	2.217	0.029
ENSG00000278053	DDX52	0.147	5.078	2.263	0.026
ENSG00000278090	RP11-6O2.2	-0.705	-0.822	-2.592	0.011
ENSG00000278156	TSC22D1-AS1	-0.237	2.305	-2.144	0.035
ENSG00000278193	RP11-313C15.2	-0.805	-1.021	-2.535	0.013
ENSG00000278206	RP1-20N2.8	-0.422	-0.319	-2.490	0.015
ENSG00000278255	RP1-53O8.2	-0.497	0.624	-2.523	0.014
ENSG00000278259	MYO19	-0.182	3.567	-2.170	0.033
ENSG00000278266	RP11-575F12.3	0.387	1.486	2.814	0.006
ENSG00000278318	ZNF229	0.302	3.485	2.580	0.012
ENSG00000278330	RP11-4B16.4	-0.649	-0.648	-2.597	0.011
ENSG00000278396	RP11-120E11.2	-0.735	-1.499	-2.770	0.007
ENSG00000278413	uc_338	-0.523	-1.474	-2.201	0.031
ENSG00000278462	RP11-165D6.1	-0.746	-0.215	-3.337	0.001
ENSG00000278464	RP11-96A15.1	-0.482	-0.718	-2.127	0.036
ENSG00000278472	RP11-74D7.3	-1.165	-1.763	-2.988	0.004

ENSG00000278576	RP11-507K2.6	-0.390	2.714	-3.512	0.001
ENSG00000278588	HIST1H2BI	0.829	-2.183	2.157	0.034
ENSG00000278601	RP11-348N5.9	-0.406	-1.090	-2.197	0.031
ENSG00000278638	CTC-268N12.2	-0.655	-1.631	-2.801	0.006
ENSG00000278668	RP11-227G15.11	-0.429	0.127	-2.146	0.035
ENSG00000278768	BACE1-AS	-0.229	3.393	-2.918	0.005
ENSG00000278811	LINC00624	-0.379	1.410	-2.025	0.046
ENSG00000278829	RP11-358B23.7	-0.330	-0.220	-2.107	0.038
ENSG00000278831	RP11-12J10.4	-0.390	-0.796	-2.099	0.039
ENSG00000278861	RP11-338K17.6	-0.500	-0.507	-2.858	0.005
ENSG00000278873	PRO1804	-0.304	2.641	-2.134	0.036
ENSG00000278875	CTC-439O9.1	-0.541	1.006	-2.939	0.004
ENSG00000278879	AP000560.3	-0.374	2.158	-2.650	0.010
ENSG00000278891	RP11-246E12.2	-0.641	-1.104	-2.805	0.006
ENSG00000278893	RP11-229O3.1	-0.659	-0.342	-3.501	0.001
ENSG00000278896	RP11-474P2.7	-0.566	0.663	-3.125	0.002
ENSG00000278917	RP11-15A1.4	-0.294	1.472	-2.496	0.015
ENSG00000278975	RP11-185J20.1	-0.539	3.134	-2.976	0.004
ENSG00000278983	RP11-426J5.3	-0.351	1.157	-1.990	0.050
ENSG00000278985	RP11-303E16.9	-0.544	1.196	-3.291	0.001
ENSG00000278991	RP11-797A18.5	-0.431	2.561	-3.099	0.003
ENSG00000279006	CTA-243E7.2	-0.598	0.081	-3.296	0.001
ENSG00000279021	RP11-391L3.4	-0.632	0.683	-3.083	0.003
ENSG00000279022	RP11-250B2.4	-0.710	-1.131	-2.742	0.007
ENSG00000279029	RP11-144I2.1	-0.502	1.106	-3.016	0.003
ENSG00000279050	PWAR1	-0.469	5.372	-3.323	0.001
ENSG00000279067	GS1-25M2.1	-0.766	-1.357	-2.674	0.009
ENSG00000279068	CH17-140K24.5	-0.474	-1.750	-1.991	0.050
ENSG00000279078	SND1-IT1	-0.357	2.150	-2.663	0.009
ENSG00000279089	RP11-481C4.2	-0.217	2.598	-2.126	0.037
ENSG00000279091	RP11-461F11.2	-0.769	-0.730	-2.946	0.004
ENSG00000279098	RP11-15B17.4	-0.516	-1.542	-2.084	0.040
ENSG00000279099	RP11-595O22.1	-0.715	2.782	-4.098	0.000
ENSG00000279103	CTC-204F22.1	-0.353	1.332	-2.490	0.015
ENSG00000279104	RP11-138B9.1	-0.611	-1.576	-2.243	0.028
ENSG00000279108	CTC-490E21.11	-0.612	0.767	-2.832	0.006
ENSG00000279110	CTA-243E7.1	-0.414	2.483	-2.549	0.013
ENSG00000279114	RP3-425C14.5	-0.663	-0.758	-2.641	0.010
ENSG00000279118	RP11-517I3.2	-0.497	3.741	-2.329	0.022
ENSG00000279123	RP11-424M24.3	-1.079	-2.325	-3.389	0.001
ENSG00000279129	RP11-264L1.2	-0.458	1.270	-2.349	0.021
ENSG00000279130	RP11-278J6.4	-0.846	-1.937	-3.048	0.003
ENSG00000279133	RP11-342K2.1	-0.320	2.978	-2.785	0.007
ENSG00000279159	RP3-394A18.1	-0.423	4.898	-3.731	0.000
ENSG00000279175	CTA-414D7.1	-0.499	0.881	-2.248	0.027
ENSG00000279189	RP11-861E23.2	-0.451	2.906	-3.131	0.002
ENSG00000279190	RP11-534P6.1	-1.096	-2.398	-2.511	0.014

ENSG00000279197	CTB-3M24.1	-0.849	-1.535	-2.567	0.012
ENSG00000279235	RP11-16K12.2	-0.552	-0.196	-3.445	0.001
ENSG00000279265	AC000123.3	-0.509	-0.494	-2.611	0.011
ENSG00000279275	RP11-756J15.2	-0.332	0.267	-2.032	0.045
ENSG00000279277	RP11-861A13.3	-0.279	1.723	-2.240	0.028
ENSG00000279278	CH17-264L24.1	-0.310	3.012	-2.070	0.042
ENSG00000279285	RP11-64C12.5	-0.980	-1.721	-2.417	0.018
ENSG00000279289	RP3-331H24.6	-0.430	2.409	-2.298	0.024
ENSG00000279300	CTD-3149D2.2	-0.713	-0.832	-2.976	0.004
ENSG00000279302	MIR3622A	-0.570	1.154	-2.641	0.010
ENSG00000279304	RP11-573M3.6	-0.685	-2.177	-2.693	0.009
ENSG00000279310	RP11-123O10.1	-0.752	-0.973	-2.860	0.005
ENSG00000279314	RP1-257C22.2	-0.548	-0.313	-2.761	0.007
ENSG00000279315	RP11-57H14.5	-0.395	0.200	-1.994	0.049
ENSG00000279319	RP11-693M3.1	-0.459	2.148	-3.103	0.003
ENSG00000279320	RP11-329N15.3	-0.725	-1.290	-2.640	0.010
ENSG00000279324	RP11-93G23.2	-0.602	0.988	-3.021	0.003
ENSG00000279344	RP11-44F14.7	-0.602	0.374	-3.642	0.000
ENSG00000279347	RP11-85I17.2	-0.554	-1.008	-2.051	0.044
ENSG00000279358	RP11-688I9.5	-0.533	2.585	-2.908	0.005
ENSG00000279373	RP11-650L12.4	-0.840	-1.979	-2.522	0.014
ENSG00000279377	AC003973.3	-0.895	1.892	-3.312	0.001
ENSG00000279384	RP11-635L1.2	-0.723	-0.293	-3.274	0.002
ENSG00000279388	RP11-897M7.2	-1.164	-3.281	-2.602	0.011
ENSG00000279406	RP11-327E2.5	0.406	-1.242	2.063	0.042
ENSG00000279416	RP11-849I19.2	-0.478	-0.634	-2.300	0.024
ENSG00000279425	CTD-2017D11.2	-0.428	2.524	-3.423	0.001
ENSG00000279432	RP11-135N5.3	-0.475	2.134	-2.930	0.004
ENSG00000279439	CTD-2576N18.1	-0.777	-1.954	-2.655	0.010
ENSG00000279463	RP11-157H4.1	-0.502	-0.870	-2.747	0.007
ENSG00000279465	RP11-44D5.2	-0.658	-2.244	-2.231	0.028
ENSG00000279474	CTD-2286N8.1	-0.601	-0.803	-2.546	0.013
ENSG00000279481	RP11-181K12.1	-0.674	0.024	-3.365	0.001
ENSG00000279486	OR2AG1	-0.407	1.788	-2.043	0.044
ENSG00000279497	RP11-654C20.1	-0.482	1.694	-2.731	0.008
ENSG00000279498	RP11-72I2.2	-0.605	-1.784	-2.312	0.023
ENSG00000279513	RP4-675C20.4	-0.381	1.262	-2.443	0.017
ENSG00000279523	RP11-461O7.2	-0.754	-2.065	-2.314	0.023
ENSG00000279544	AL133243.4	-0.351	2.404	-2.189	0.031
ENSG00000279555	RP11-426D19.1	-0.418	1.614	-2.297	0.024
ENSG00000279594	RP11-950C14.10	-0.329	-0.313	-2.274	0.026
ENSG00000279598	RP11-65L3.3	-0.393	1.258	-2.396	0.019
ENSG00000279600	RP11-637C24.5	-0.573	-0.170	-2.129	0.036
ENSG00000279606	RP11-502F1.1	-0.604	-1.505	-2.085	0.040
ENSG00000279608	RP11-182N22.9	-0.325	2.455	-2.430	0.017
ENSG00000279623	RP11-399C16.3	-0.530	-1.471	-2.206	0.030
ENSG00000279645	RP11-759A24.1	-0.646	-1.513	-2.566	0.012

ENSG00000279649	RP11-96D1.8	-0.356	0.973	-2.022	0.046
ENSG00000279652	RP1-78B3.1	-0.467	2.613	-2.987	0.004
ENSG00000279659	RP11-177G23.1	-0.465	2.540	-2.924	0.004
ENSG00000279662	RP11-609N14.4	-0.376	1.991	-2.153	0.034
ENSG00000279668	CTC-304I17.6	-0.471	3.749	-2.782	0.007
ENSG00000279670	RP11-70L8.5	-0.630	0.703	-3.716	0.000
ENSG00000279674	RP11-1086I4.2	-0.397	4.462	-2.211	0.030
ENSG00000279702	RP11-347N5.3	-0.469	1.945	-2.431	0.017
ENSG00000279711	AP004782.1	-0.465	3.109	-2.397	0.019
ENSG00000279712	CTA-280A3.2	-0.541	2.034	-2.212	0.030
ENSG00000279727	LINC02033	-0.542	-0.497	-2.978	0.004
ENSG00000279735	AC124312.1	-0.585	1.956	-3.223	0.002
ENSG00000279739	CTB-105N12.2	-0.531	0.492	-2.705	0.008
ENSG00000279742	RP11-700A24.1	-0.372	0.037	-2.075	0.041
ENSG00000279766	RP11-642A1.2	-0.540	-0.952	-2.330	0.022
ENSG00000279786	CTD-2014D20.1	-0.691	0.165	-2.674	0.009
ENSG00000279829	RP11-63I8.1	-0.741	-0.987	-2.223	0.029
ENSG00000279834	RP11-775C24.4	-0.361	0.977	-2.085	0.040
ENSG00000279837	RP11-504G3.2	-0.576	-1.497	-2.009	0.048
ENSG00000279853	RP5-844F9.1	-0.374	0.829	-2.664	0.009
ENSG00000279858	RP11-618M23.5	-0.512	-1.267	-2.325	0.023
ENSG00000279863	RP11-521C22.2	-0.399	1.355	-2.595	0.011
ENSG00000279880	RP11-855A2.2	-0.454	0.595	-2.505	0.014
ENSG00000279883	CTD-2315M5.2	-0.592	0.266	-2.950	0.004
ENSG00000279885	RP11-637N19.1	-0.609	1.185	-3.849	0.000
ENSG00000279891	FLJ42393	-0.483	-0.113	-2.445	0.017
ENSG00000279897	BIRC6-AS2	-0.499	0.796	-3.278	0.002
ENSG00000279948	CTD-2233K9.1	-0.302	2.060	-2.254	0.027
ENSG00000279954	WI2-85898F10.2	-0.702	-2.213	-2.482	0.015
ENSG00000279957	RP11-141B14.2	-0.542	-0.768	-2.092	0.040
ENSG00000279958	RP11-12O16.1	-0.629	-0.087	-2.820	0.006
ENSG00000279985	CTC-436P18.4	-0.405	0.270	-2.419	0.018
ENSG00000279991	RP11-190D6.1	-0.553	-0.678	-2.424	0.018
ENSG00000280000	RP11-509E10.3	-0.428	1.328	-2.248	0.027
ENSG00000280005	RP11-9N12.3	-0.699	-2.517	-2.575	0.012
ENSG00000280037	RP11-434P11.1	-0.574	0.168	-3.820	0.000
ENSG00000280053	RP11-61K12.2	-0.519	-0.366	-2.289	0.025
ENSG00000280055	TMEM75	-0.566	-0.290	-2.737	0.008
ENSG00000280057	RP1-168L15.6	-0.347	0.456	-2.419	0.018
ENSG00000280067	CTD-2600H12.2	-0.518	1.833	-3.322	0.001
ENSG00000280069	CTD-2349P21.3	-0.464	2.738	-3.086	0.003
ENSG00000280078	RP11-361H10.5	-0.603	-1.142	-2.714	0.008
ENSG00000280098	AC011155.1	-0.909	-1.932	-3.780	0.000
ENSG00000280104	CTB-3M24.2	-0.619	0.102	-2.740	0.008
ENSG00000280105	RP11-334E15.1	-0.296	2.434	-2.064	0.042
ENSG00000280107	AL022393.9	-0.546	0.537	-2.557	0.012
ENSG00000280114	RP5-875O13.7	-0.571	-0.895	-2.203	0.030

ENSG00000280115	RP11-133M8.3	-0.722	-1.079	-3.331	0.001
ENSG00000280138	RP11-463O12.5	-0.415	4.168	-2.679	0.009
ENSG00000280157	RP11-520H14.7	-0.402	2.088	-2.680	0.009
ENSG00000280165	PCDH20	0.566	-1.899	2.086	0.040
ENSG00000280168	RP11-1090M7.3	-0.506	-0.899	-2.305	0.024
ENSG00000280190	RP11-319G9.1	-0.600	0.788	-2.993	0.004
ENSG00000280202	RP5-1180D12.1	-0.567	-1.074	-2.499	0.014
ENSG00000280205	RP11-504I13.2	-0.573	-0.573	-2.279	0.025
ENSG00000280227	RP11-454F8.3	-0.477	-1.462	-2.291	0.025
ENSG00000280257	RP11-340F16.2	-0.585	0.494	-2.578	0.012
ENSG00000280269	AP000577.2	-0.476	0.641	-3.086	0.003
ENSG00000280274	RP11-556H2.1	-0.649	-1.642	-2.158	0.034
ENSG00000280286	FRG1KP	-0.600	1.406	-2.179	0.032
ENSG00000280294	RP11-177H2.1	-0.362	2.950	-3.373	0.001
ENSG00000280295	RP11-358B23.6	-0.507	-0.592	-2.568	0.012
ENSG00000280321	RP11-1110F20.1	-0.510	-0.745	-2.174	0.033
ENSG00000280327	LA16c-313F4.1	-0.695	-2.136	-2.465	0.016
ENSG00000280336	CH17-140K24.3	-0.397	-0.211	-1.991	0.050
ENSG00000280347	AC000123.2	-0.515	3.303	-3.564	0.001
ENSG00000280348	RP11-552M6.1	-0.804	0.864	-4.710	0.000
ENSG00000280350	LINC01726	-0.775	-0.892	-2.652	0.010
ENSG00000280355	RP11-119F19.5	-0.286	2.181	-2.325	0.023
ENSG00000280365	RP11-9H20.2	-0.530	1.115	-2.481	0.015
ENSG00000280374	RP11-337N6.3	-0.442	0.018	-2.769	0.007
ENSG00000280378	RP4-758J24.6	-0.653	-0.051	-2.945	0.004
ENSG00000280383	CTA-941F9.10	-0.415	3.517	-3.516	0.001
ENSG00000280392	RP11-357N13.6	-0.527	2.294	-3.421	0.001
ENSG00000280397	RP11-42F12.1	-0.522	-0.917	-2.046	0.044
ENSG00000280399	RP11-80P20.3	-0.398	1.952	-2.876	0.005
ENSG00000280425	RP11-446E24.3	-0.713	-2.595	-2.396	0.019
ENSG00000280432	AP000962.1	-0.669	1.187	-4.519	0.000
ENSG00000280485	RP11-437J2.4	-0.792	0.204	-4.709	0.000
ENSG00000280620	SCAANT1	-0.757	-1.911	-2.493	0.015
ENSG00000280650	KCNIP4-IT1	-0.387	7.399	-2.652	0.010
ENSG00000280660	RP11-437J2.3	-0.590	4.551	-3.743	0.000
ENSG00000280776	LINC01202	0.406	1.724	2.196	0.031
ENSG00000280780	JAKMIP2-AS1	-0.352	0.667	-2.224	0.029
ENSG00000280828	RP11-274B21.1	-0.269	1.222	-2.246	0.027
ENSG00000280913	CTSLP2	-0.406	-1.094	-1.999	0.049
ENSG00000280927	CTBP1-AS	-0.386	0.053	-1.991	0.050
ENSG00000280958	RP4-587D13.2	-0.539	0.635	-3.070	0.003
ENSG00000281008	RP4-587D13.1	-0.425	-0.136	-2.370	0.020
ENSG00000281026	N4BP2L2-IT2	-0.369	4.819	-2.686	0.009
ENSG00000281100	RP11-640L9.2	-0.336	1.009	-2.228	0.029
ENSG00000281120	RP5-1093G23.1	-0.624	0.051	-3.127	0.002
ENSG00000281195	ZNF638-IT1	-0.381	3.874	-2.869	0.005
ENSG00000281207	SLFNL1-AS1	-0.442	0.154	-2.391	0.019

ENSG00000281344	HELLPAR	-0.361	2.028	-2.094	0.039
ENSG00000281392	LINC00506	-0.342	1.607	-2.683	0.009
ENSG00000281649	EBLN3	0.153	6.270	2.106	0.038
ENSG00000281691	RBM5-AS1	-0.478	-0.189	-2.275	0.026
ENSG00000281880	PAUPAR	-0.816	-1.040	-3.104	0.003
ENSG00000282021	CTD-2336O2.3	-0.614	-1.517	-2.155	0.034
ENSG00000282022	RP11-259O18.5	-0.457	-0.052	-2.322	0.023
ENSG00000282057	RP4-621F18.2	-0.336	1.604	-2.681	0.009
ENSG00000282121	RP11-388P9.3	-0.622	-2.158	-2.354	0.021
ENSG00000282164	PEG13	-0.205	4.594	-2.217	0.029
ENSG00000282390	RP11-29A19.2	-0.445	-0.232	-2.061	0.042
ENSG00000282834	RP11-712B9.6	-0.656	0.826	-3.241	0.002
ENSG00000282855	RP11-50D9.4	-0.536	1.762	-2.685	0.009
ENSG00000282864	RP11-487A2.1	-1.073	-0.906	-3.495	0.001
ENSG00000282865	RP11-306O1.2	-0.595	1.253	-2.237	0.028
ENSG00000282943	CTC-490G23.6	-0.437	0.087	-2.509	0.014
ENSG00000282977	PCBP2-OT1	-0.556	-1.460	-2.082	0.040
ENSG00000282997	RP11-95C14.1	-1.065	-0.734	-3.312	0.001
ENSG00000283058	RP11-358H12.1	-0.355	1.572	-2.139	0.035
ENSG00000283097	RP11-165N12.2	-0.729	1.128	-2.570	0.012
ENSG00000283122	HYMAI	-0.569	0.587	-3.646	0.000
ENSG00000283131	RP11-35D5.4	-0.639	-1.185	-2.160	0.034
ENSG00000283156	RP11-646I6.3	-0.435	2.686	-3.725	0.000
ENSG00000283208	RP11-226E21.4	-0.435	2.923	-3.085	0.003
ENSG00000283213	CTC-591M7.1	-0.422	0.458	-2.163	0.033
ENSG00000283221	RP11-297M9.2	-0.612	-0.960	-3.224	0.002
ENSG00000283235	RP11-844P9.6	-0.624	-2.286	-2.084	0.040
ENSG00000283236	AC074141.3	-0.270	2.851	-2.452	0.016
ENSG00000283267	CTB-16J4.1	-0.561	-0.092	-2.217	0.029
ENSG00000283355	RP11-739P1.3	-0.364	1.017	-2.207	0.030
ENSG00000283415	RP11-413N10.3	-0.453	1.046	-2.284	0.025
ENSG00000283417	RP11-118G23.3	-0.474	3.006	-2.423	0.018
ENSG00000283458	RP11-1I2.1	1.244	-2.233	2.702	0.008
ENSG00000283489	U6	-0.554	-1.614	-2.173	0.033
ENSG00000283503	LINC01902	-0.368	3.266	-2.299	0.024
ENSG00000283509	U6	-0.500	-1.475	-2.456	0.016
ENSG00000283529	RP4-791K14.2	-0.389	1.259	-2.445	0.017
ENSG00000283584	snoMe28S-Am263	-0.719	-2.158	-2.769	0.007
ENSG00000283913	#N/A	-0.439	0.568	-3.418	0.001
ENSG00000284052	#N/A	-0.480	3.002	-4.078	0.000
ENSG00000284196	#N/A	-0.500	1.341	-2.768	0.007
ENSG00000284376	#N/A	-0.917	-2.169	-2.603	0.011
ENSG00000284428	#N/A	-0.377	3.496	-3.049	0.003
ENSG00000284606	#N/A	-0.634	2.951	-2.104	0.038
ENSG00000284612	#N/A	0.806	-2.616	2.069	0.042
ENSG00000284657	#N/A	-0.498	-1.432	-2.010	0.048
ENSG00000284770	TBCE	-0.209	3.129	-2.415	0.018

ENSG00000284773	#N/A	-0.554	-0.475	-2.333	0.022
ENSG00000284808	#N/A	-0.864	-2.749	-2.318	0.023
ENSG00000284848	LINC02359	-0.628	0.921	-3.091	0.003
ENSG00000284862	#N/A	-0.416	0.943	-3.150	0.002
ENSG00000284902	#N/A	-0.436	-0.458	-2.217	0.029
ENSG00000284946	#N/A	-0.380	2.451	-2.830	0.006
ENSG00000284966	#N/A	-0.413	2.773	-3.334	0.001
ENSG00000284977	#N/A	-0.589	3.392	-2.400	0.019
ENSG00000285103	#N/A	-0.435	3.781	-3.144	0.002
ENSG00000285106	#N/A	-0.508	3.137	-3.301	0.001
ENSG00000285215	#N/A	-0.556	0.515	-2.348	0.021
ENSG00000285230	RALY-AS1	-0.496	1.632	-4.030	0.000
ENSG00000285366	#N/A	-0.542	0.353	-2.738	0.008
ENSG00000285382	#N/A	-0.430	-0.690	-2.344	0.022
ENSG00000285388	#N/A	-0.706	-2.325	-2.101	0.039
ENSG00000285399	#N/A	-0.384	2.275	-2.717	0.008
ENSG00000285424	#N/A	-0.622	-1.421	-2.214	0.030
ENSG00000285476	#N/A	-0.485	2.743	-2.394	0.019
ENSG00000285509	#N/A	-0.379	-0.339	-2.102	0.039
ENSG00000285518	#N/A	-0.707	-0.981	-4.014	0.000
ENSG00000285520	#N/A	-0.704	0.579	-3.632	0.000
ENSG00000285532	#N/A	-0.474	-0.700	-2.403	0.019
ENSG00000285548	#N/A	-0.393	1.860	-2.347	0.021
ENSG00000285555	#N/A	-0.555	1.635	-3.009	0.003
ENSG00000285579	#N/A	-0.772	-0.845	-3.411	0.001
ENSG00000285582	#N/A	-0.344	4.802	-2.176	0.032
ENSG00000285603	#N/A	-0.657	-0.912	-2.846	0.006
ENSG00000285605	#N/A	-0.569	0.383	-2.940	0.004
ENSG00000285606	#N/A	-0.464	0.812	-2.399	0.019
ENSG00000285608	#N/A	-0.985	-2.949	-2.331	0.022
ENSG00000285612	#N/A	-0.728	-1.480	-3.524	0.001
ENSG00000285618	#N/A	-0.888	-0.960	-2.654	0.010
ENSG00000285621	#N/A	-0.723	0.114	-3.896	0.000
ENSG00000285634	#N/A	0.414	2.865	2.347	0.021
ENSG00000285658	#N/A	-0.381	0.267	-2.141	0.035
ENSG00000285664	#N/A	-0.468	0.514	-2.900	0.005
ENSG00000285691	#N/A	-1.134	-2.499	-2.479	0.015
ENSG00000285704	#N/A	-0.415	2.044	-2.689	0.009
ENSG00000285717	#N/A	-0.494	1.169	-2.261	0.026
ENSG00000285725	#N/A	-0.366	1.233	-2.352	0.021
ENSG00000285731	#N/A	-0.472	-0.131	-2.464	0.016
ENSG00000285734	#N/A	-0.433	0.959	-2.067	0.042
ENSG00000285735	#N/A	0.606	0.281	2.063	0.042
ENSG00000285739	#N/A	-0.311	3.750	-2.388	0.019
ENSG00000285740	#N/A	-0.709	-2.067	-2.224	0.029
ENSG00000285750	#N/A	-0.476	0.446	-2.664	0.009
ENSG00000285774	#N/A	-0.586	-0.298	-2.711	0.008

ENSG00000285781	#N/A	-0.323	0.805	-2.000	0.049
ENSG00000285782	#N/A	-0.699	-2.129	-2.283	0.025
ENSG00000285791	#N/A	-0.270	0.970	-2.054	0.043
ENSG00000285803	#N/A	-0.680	0.575	-2.932	0.004
ENSG00000285804	#N/A	-0.530	0.524	-2.851	0.006
ENSG00000285813	#N/A	-0.269	2.739	-2.171	0.033
ENSG00000285820	#N/A	-0.459	-0.159	-2.299	0.024
ENSG00000285830	#N/A	-0.409	-0.873	-2.290	0.025
ENSG00000285836	#N/A	-0.493	0.057	-3.312	0.001
ENSG00000285841	#N/A	-0.483	2.625	-3.057	0.003
ENSG00000285849	#N/A	-0.495	0.219	-2.790	0.007
ENSG00000285867	#N/A	-0.443	-0.164	-2.271	0.026
ENSG00000285868	#N/A	-0.506	1.001	-3.282	0.002
ENSG00000285871	#N/A	-0.563	-0.867	-2.325	0.023
ENSG00000285879	#N/A	-0.497	0.415	-2.737	0.008
ENSG00000285882	#N/A	-0.494	-0.432	-2.218	0.029
ENSG00000285904	#N/A	-0.743	-0.636	-2.690	0.009
ENSG00000285906	#N/A	-0.698	-0.948	-2.618	0.011
ENSG00000285908	#N/A	-0.592	-0.116	-2.069	0.042
ENSG00000285909	#N/A	-0.416	-0.169	-2.428	0.017
ENSG00000285931	#N/A	-0.471	-0.374	-2.522	0.014
ENSG00000285933	#N/A	-0.444	-0.511	-2.099	0.039
ENSG00000285943	#N/A	-0.291	0.430	-2.028	0.046
ENSG00000285948	#N/A	-0.511	-1.149	-2.615	0.011
ENSG00000285977	#N/A	-0.480	1.889	-3.309	0.001
ENSG00000285979	#N/A	-0.257	2.373	-2.158	0.034
ENSG00000285980	#N/A	-0.434	1.496	-2.492	0.015
ENSG00000285994	#N/A	-0.464	2.111	-2.867	0.005

Nac

gene_id	gene_name	logFC	AveExpr	t	P.Value
ENSG00000003147	ICA1	-0.408	5.871	-2.825	0.006
ENSG00000004948	CALCR	2.497	-0.095	2.200	0.031
ENSG00000007129	CEACAM21	1.120	-1.271	2.153	0.035
ENSG00000043039	BARX2	-2.186	-3.129	-2.658	0.010
ENSG00000049130	KITLG	-0.734	5.342	-2.775	0.007
ENSG00000052126	PLEKHA5	-0.339	7.539	-2.503	0.015
ENSG00000055813	CCDC85A	-0.567	2.940	-2.186	0.032
ENSG00000064300	NGFR	1.978	-0.383	2.303	0.024
ENSG00000065665	SEC61A2	0.248	5.013	2.060	0.043
ENSG00000070159	PTPN3	-1.517	1.889	-2.520	0.014
ENSG00000070961	ATP2B1	-0.771	10.259	-2.031	0.046
ENSG00000072818	ACAP1	0.362	2.691	2.203	0.031
ENSG00000073756	PTGS2	-1.043	1.314	-2.321	0.023
ENSG00000073803	MAP3K13	-0.222	6.136	-2.021	0.047
ENSG00000074706	IPCEF1	-0.567	6.622	-2.032	0.046
ENSG00000078967	UBE2D4	-0.174	4.390	-2.013	0.048
ENSG00000079841	RIMS1	-0.423	8.567	-2.396	0.019
ENSG00000083290	ULK2	-0.213	6.701	-2.080	0.041
ENSG00000088179	PTPN4	-0.361	7.337	-2.815	0.006
ENSG00000094796	KRT31	-2.246	-5.159	-2.564	0.012
ENSG00000100626	GALNT16	0.506	4.765	2.517	0.014
ENSG00000100987	VSX1	0.621	0.843	2.526	0.014
ENSG00000102524	TNFSF13B	0.420	1.350	2.044	0.045
ENSG00000102967	DHODH	0.287	3.499	2.194	0.032
ENSG00000103187	COTL1	0.500	4.352	2.265	0.027
ENSG00000105011	ASF1B	1.170	-2.480	2.237	0.028
ENSG00000105137	SYDE1	0.538	0.449	2.125	0.037
ENSG00000105607	GCDH	-0.278	3.424	-2.048	0.044
ENSG00000105697	HAMP	1.459	-0.695	2.123	0.037
ENSG00000106278	PTPRZ1	0.272	9.398	2.085	0.041
ENSG00000106330	MOSPD3	0.425	0.407	2.022	0.047
ENSG00000106511	MEOX2	0.956	-0.308	2.341	0.022
ENSG00000106648	GALNTL5	-0.976	-0.985	-2.080	0.041
ENSG00000107518	ATRNL1	-0.611	8.566	-2.849	0.006
ENSG00000108924	HLF	-0.442	7.225	-2.141	0.036
ENSG00000110318	CEP126	-0.466	6.787	-2.238	0.028
ENSG00000111341	MGP	-0.672	1.638	-2.101	0.039
ENSG00000111801	BTN3A3	0.410	3.528	2.122	0.037
ENSG00000112715	VEGFA	0.758	5.194	2.709	0.008
ENSG00000113361	CDH6	-0.715	5.810	-2.265	0.027
ENSG00000113615	SEC24A	0.229	5.075	2.232	0.029
ENSG00000114268	PFKFB4	0.491	1.824	2.088	0.040
ENSG00000115423	DNAH6	-0.755	6.749	-2.676	0.009
ENSG00000115641	FHL2	-0.480	2.155	-2.170	0.033
ENSG00000115718	PROC	0.964	-0.762	3.049	0.003

ENSG00000115844	DLX2	1.175	-1.124	2.226	0.029
ENSG00000116106	EPHA4	-0.577	6.926	-2.562	0.013
ENSG00000117009	KMO	-0.617	0.650	-1.996	0.050
ENSG00000117020	AKT3	-0.301	9.045	-2.087	0.040
ENSG00000117650	NEK2	-1.298	-1.780	-2.158	0.034
ENSG00000118420	UBE3D	-0.260	4.480	-2.000	0.049
ENSG00000119508	NR4A3	-0.940	4.032	-2.065	0.043
ENSG00000120057	SFRP5	0.843	-0.469	2.015	0.048
ENSG00000120903	CHRNA2	-1.558	-0.169	-2.474	0.016
ENSG00000122591	FAM126A	0.393	5.926	2.104	0.039
ENSG00000123219	CENPK	-0.635	1.123	-2.248	0.028
ENSG00000123473	STIL	-0.273	2.986	-2.278	0.026
ENSG00000123610	TNFAIP6	0.840	0.572	2.065	0.043
ENSG00000123965	PMS2P5	0.862	1.732	2.794	0.007
ENSG00000124302	CHST8	1.698	-0.901	2.502	0.015
ENSG00000124788	ATXN1	-0.334	8.111	-2.565	0.012
ENSG00000125850	OVOL2	-1.732	-4.260	-2.069	0.042
ENSG00000127528	KLF2	-0.817	0.423	-2.203	0.031
ENSG00000128656	CHN1	-0.616	9.015	-2.208	0.030
ENSG00000128833	MYO5C	-0.597	4.676	-2.440	0.017
ENSG00000129244	ATP1B2	0.656	6.962	2.165	0.034
ENSG00000129450	SIGLEC9	1.154	-0.794	2.183	0.032
ENSG00000130363	RSPH3	-0.386	5.566	-2.740	0.008
ENSG00000130762	ARHGEF16	1.177	-1.863	2.168	0.033
ENSG00000130921	C12orf65	0.192	3.608	2.039	0.045
ENSG00000131351	HAUS8	0.500	0.529	2.620	0.011
ENSG00000131849	ZNF132	-0.184	3.783	-2.125	0.037
ENSG00000132164	SLC6A11	0.831	4.997	2.066	0.042
ENSG00000133026	MYH10	-0.314	7.904	-2.260	0.027
ENSG00000133027	PEMT	0.580	0.823	2.562	0.013
ENSG00000133121	STARD13	-0.288	5.605	-2.011	0.048
ENSG00000134259	NGF	-1.262	-2.831	-2.077	0.041
ENSG00000136240	KDELR2	0.224	4.963	1.994	0.050
ENSG00000137135	ARHGEF39	0.677	-0.690	2.456	0.016
ENSG00000137364	TPMT	-0.291	4.475	-2.065	0.043
ENSG00000138311	ZNF365	-0.397	6.125	-2.063	0.043
ENSG00000138653	NDST4	1.303	0.834	2.158	0.034
ENSG00000138758	SEPTIN11	-0.247	8.308	-2.003	0.049
ENSG00000139174	PRICKLE1	-0.476	3.262	-2.428	0.018
ENSG00000139364	TMEM132B	-0.397	8.208	-2.064	0.043
ENSG00000140009	ESR2	0.334	1.456	1.995	0.050
ENSG00000140488	CELF6	1.131	-2.086	2.680	0.009
ENSG00000141194	OR4D1	1.204	-3.409	1.995	0.050
ENSG00000143627	PKLR	1.471	-3.026	2.263	0.027
ENSG00000143630	HCN3	0.525	1.475	2.014	0.048
ENSG00000143942	CHAC2	0.382	1.790	2.133	0.036
ENSG00000144045	DQX1	1.267	-2.720	2.038	0.045

ENSG00000144119	C1QL2	0.750	0.179	2.373	0.020
ENSG00000144455	SUMF1	-0.244	4.608	-2.185	0.032
ENSG00000144644	GADL1	-1.460	-3.791	-2.184	0.032
ENSG00000145087	STXBP5L	-0.503	7.676	-2.046	0.044
ENSG00000145824	CXCL14	0.752	4.385	2.700	0.009
ENSG00000145850	TIMD4	1.213	-2.634	2.074	0.042
ENSG00000145982	FARS2	-0.275	4.276	-2.022	0.047
ENSG00000148704	VAX1	0.731	1.869	2.218	0.030
ENSG00000149599	DUSP15	0.522	0.501	2.344	0.022
ENSG00000152465	NMT2	-0.236	5.291	-2.277	0.026
ENSG00000153395	LPCAT1	0.228	4.695	2.223	0.029
ENSG00000154240	CEP112	-0.486	5.239	-2.472	0.016
ENSG00000155530	LRGUK	-0.398	3.120	-2.013	0.048
ENSG00000156966	B3GNT7	0.474	2.075	2.194	0.031
ENSG00000157315	TMED6	-0.636	-0.720	-2.297	0.025
ENSG00000157927	RADIL	0.429	0.988	2.138	0.036
ENSG00000158077	NLRP14	-0.547	1.051	-2.021	0.047
ENSG00000159399	HK2	0.658	3.122	2.038	0.045
ENSG00000159618	ADGRG5	1.383	-2.465	2.138	0.036
ENSG00000160087	UBE2J2	0.290	2.922	2.312	0.024
ENSG00000160188	RSPH1	-0.652	4.216	-2.258	0.027
ENSG00000160691	SHC1	0.546	3.051	2.481	0.015
ENSG00000161405	IKZF3	-1.017	-0.686	-2.444	0.017
ENSG00000161904	LEMD2	0.216	4.188	2.457	0.016
ENSG00000162383	SLC1A7	1.709	-2.733	2.493	0.015
ENSG00000162836	ACP6	0.416	4.292	2.395	0.019
ENSG00000163132	MSX1	-0.982	-0.354	-2.666	0.009
ENSG00000163634	THOC7	-0.343	5.398	-2.143	0.035
ENSG00000163817	SLC6A20	-0.807	-0.002	-2.480	0.015
ENSG00000164307	ERAP1	0.277	5.342	2.272	0.026
ENSG00000164951	PDP1	-0.792	8.424	-2.245	0.028
ENSG00000165084	C8orf34	-0.561	4.589	-2.161	0.034
ENSG00000165202	OR1Q1	1.208	-2.381	2.423	0.018
ENSG00000165209	STRBP	-0.366	6.671	-2.785	0.007
ENSG00000165879	FRAT1	0.411	1.035	1.997	0.050
ENSG00000166407	LMO1	1.015	-1.924	2.127	0.037
ENSG00000166573	GALR1	0.707	2.373	2.222	0.029
ENSG00000166579	NDEL1	-0.291	6.172	-2.003	0.049
ENSG00000167173	C15orf39	0.877	1.032	2.891	0.005
ENSG00000167394	ZNF668	0.394	0.903	2.248	0.028
ENSG00000167536	DHRS13	0.710	-0.549	2.048	0.044
ENSG00000168079	SCARA5	-1.522	-2.151	-2.314	0.024
ENSG00000168418	KCNG4	0.861	-1.106	2.291	0.025
ENSG00000168454	TXNDC2	1.500	-3.199	2.239	0.028
ENSG00000169064	ZBBX	-0.720	4.522	-2.036	0.045
ENSG00000169442	CD52	-0.869	-1.460	-2.164	0.034
ENSG00000169764	UGP2	-0.312	7.476	-2.555	0.013

ENSG00000170289	CNGB3	-0.557	0.636	-2.189	0.032
ENSG00000171864	PRND	-2.521	-3.523	-3.318	0.001
ENSG00000171944	OR52A5	-1.599	-5.406	-2.277	0.026
ENSG00000172059	KLF11	0.488	1.948	2.062	0.043
ENSG00000172403	SYNPO2	-0.443	6.031	-2.380	0.020
ENSG00000173212	MAB21L3	1.039	-0.798	2.701	0.009
ENSG00000173585	CCR9	1.073	-2.415	2.136	0.036
ENSG00000173805	HAP1	0.766	3.374	2.280	0.026
ENSG00000173838	MARCHF10	-0.728	0.708	-2.032	0.046
ENSG00000174417	TRHR	1.928	-0.594	3.142	0.002
ENSG00000175449	RFESD	-0.497	1.259	-2.323	0.023
ENSG00000175544	CABP4	1.277	-1.308	3.115	0.003
ENSG00000176014	TUBB6	0.467	2.396	2.325	0.023
ENSG00000176399	DMRTA1	-0.598	1.437	-2.896	0.005
ENSG00000176406	RIMS2	-0.312	8.705	-2.002	0.049
ENSG00000176410	DNAJC30	-0.287	2.292	-2.088	0.040
ENSG00000177201	OR2T12	1.143	-1.709	2.058	0.043
ENSG00000177519	RPRM	1.535	-0.143	2.524	0.014
ENSG00000179038	RP11-157K17.5	0.612	1.229	2.397	0.019
ENSG00000179133	C10orf67	-0.549	1.542	-2.024	0.047
ENSG00000179218	CALR	0.284	7.225	2.071	0.042
ENSG00000179270	C2orf71	-0.696	-0.294	-2.490	0.015
ENSG00000179846	NKPD1	0.807	-0.500	2.185	0.032
ENSG00000180509	KCNE1	-0.954	-0.254	-2.650	0.010
ENSG00000181026	AEN	0.519	2.073	2.087	0.040
ENSG00000181541	MAB21L2	0.636	0.310	2.158	0.034
ENSG00000182108	DEXI	0.452	2.164	2.640	0.010
ENSG00000183049	CAMK1D	-0.537	7.309	-2.040	0.045
ENSG00000183386	FHL3	0.799	-0.696	2.077	0.041
ENSG00000183775	KCTD16	-0.545	7.128	-2.030	0.046
ENSG00000183784	C9orf66	1.208	-1.953	2.063	0.043
ENSG00000184385	UMODL1-AS1	-1.316	-1.905	-2.179	0.033
ENSG00000184530	C6orf58	-0.841	-2.114	-2.027	0.046
ENSG00000184544	DHRS7C	-1.379	-5.699	-2.845	0.006
ENSG00000184613	NELL2	-0.604	6.818	-2.124	0.037
ENSG00000185046	ANKS1B	-0.327	8.336	-2.244	0.028
ENSG00000185112	FAM43A	0.521	0.924	2.135	0.036
ENSG00000185436	IFNLR1	0.472	1.298	2.424	0.018
ENSG00000185739	SRL	-0.678	-0.744	-2.107	0.039
ENSG00000186654	PRR5	0.490	0.869	2.014	0.048
ENSG00000186854	TRABD2A	-0.923	1.568	-2.879	0.005
ENSG00000187676	B3GLCT	-0.662	6.616	-2.197	0.031
ENSG00000187840	EIF4EBP1	0.902	-0.460	2.667	0.009
ENSG00000187848	P2RX2	2.187	-4.950	3.191	0.002
ENSG00000187912	CLEC17A	1.217	-1.377	2.023	0.047
ENSG00000189195	BTBD8	-0.382	8.288	-2.014	0.048
ENSG00000189325	C6orf222	1.627	-4.709	2.145	0.035

ENSG00000196378	ZNF34	0.252	3.163	2.316	0.023
ENSG00000196968	FUT11	0.307	4.011	2.213	0.030
ENSG00000198586	TLK1	-0.253	7.370	-2.538	0.013
ENSG00000198691	ABCA4	-0.961	-0.858	-2.176	0.033
ENSG00000198796	ALPK2	-2.092	-3.754	-2.759	0.007
ENSG00000198821	CD247	-0.683	0.749	-2.587	0.012
ENSG00000198855	FICD	0.308	3.123	2.243	0.028
ENSG00000199395	RNA5SP93	-1.157	-2.282	-2.388	0.020
ENSG00000199629	RNU1-14P	1.292	-5.026	2.101	0.039
ENSG00000199855	RNU6-490P	0.727	-1.130	2.244	0.028
ENSG00000200189	RNU6-832P	-1.346	-5.393	-2.359	0.021
ENSG00000203499	FAM83H-AS1	-0.982	-0.128	-2.166	0.034
ENSG00000203711	C6orf99	-0.832	-0.440	-2.159	0.034
ENSG00000203805	PLPP4	0.671	1.688	2.166	0.034
ENSG00000204175	GPRIN2	1.370	-1.290	2.225	0.029
ENSG00000204531	POU5F1	1.111	-2.534	2.710	0.008
ENSG00000204767	FAM196B	0.593	2.494	1.994	0.050
ENSG00000205220	PSMB10	0.460	0.451	2.062	0.043
ENSG00000205336	ADGRG1	0.569	5.330	2.363	0.021
ENSG00000205959	RP11-689P11.2	-0.581	1.126	-2.015	0.048
ENSG00000206609	SNORD116-11	-0.799	-1.153	-2.995	0.004
ENSG00000207116	RNU6-31P	-1.122	-0.819	-2.003	0.049
ENSG00000211772	TRBC2	-1.672	-1.371	-3.077	0.003
ENSG00000213046	EEF1A1P32	-0.680	1.082	-2.416	0.018
ENSG00000213126	AC092642.1	-1.222	-2.473	-2.094	0.040
ENSG00000213316	LTC4S	0.823	-1.845	2.276	0.026
ENSG00000213654	GPSM3	0.660	0.195	2.269	0.026
ENSG00000213741	RPS29	0.281	4.976	2.360	0.021
ENSG00000213889	PPM1N	0.693	-0.497	2.046	0.044
ENSG00000214331	RP11-252A24.2	-0.441	3.849	-2.057	0.043
ENSG00000214872	SMTNL1	0.871	-0.859	2.328	0.023
ENSG00000215417	MIR17HG	0.709	1.085	2.467	0.016
ENSG00000216863	LY86-AS1	-0.834	2.588	-2.217	0.030
ENSG00000218577	RP3-416J7.1	-2.205	-1.947	-2.699	0.009
ENSG00000218631	RP3-395C13.1	-0.768	-1.472	-2.048	0.044
ENSG00000218996	RP1-99E18.2	-0.657	0.497	-2.772	0.007
ENSG00000222112	RN7SKP16	0.602	0.242	2.248	0.028
ENSG00000222317	RNA5SP118	-0.714	-0.978	-2.076	0.042
ENSG00000222990	RNU4-22P	-0.748	-1.821	-2.197	0.031
ENSG00000223812	RP11-197K6.1	-0.954	1.670	-2.080	0.041
ENSG00000224429	LINC00539	0.390	0.135	2.019	0.047
ENSG00000224530	PPIAP10	1.269	-2.942	2.044	0.045
ENSG00000224613	RP11-153F1.1	-0.979	-1.588	-2.480	0.016
ENSG00000224822	THRB-IT1	-0.638	1.861	-2.277	0.026
ENSG00000225234	TRAPPC12-AS1	0.571	0.882	2.207	0.031
ENSG00000225675	LINC01771	0.953	-1.326	2.023	0.047
ENSG00000225938	RP4-575N6.4	0.701	-0.952	2.105	0.039

ENSG00000226421	SLC25A5P5	-1.072	-0.893	-3.035	0.003
ENSG00000226476	LINC01748	-0.460	4.871	-2.187	0.032
ENSG00000226620	LINC00343	-1.991	-4.222	-2.270	0.026
ENSG00000227165	WDR11-AS1	0.338	2.537	2.213	0.030
ENSG00000227245	RP11-136C24.2	0.807	-1.300	2.125	0.037
ENSG00000227495	AC109333.10	0.977	-2.152	2.044	0.045
ENSG00000228020	HNRNPA1P46	-1.778	-4.339	-2.329	0.023
ENSG00000228038	VN1R51P	-0.794	-0.014	-2.045	0.045
ENSG00000228106	RP11-452F19.3	0.475	0.717	1.997	0.050
ENSG00000228166	MTND1P11	1.569	-3.000	2.066	0.042
ENSG00000228288	PCAT6	1.058	-2.479	2.009	0.048
ENSG00000228672	PROB1	-0.638	0.172	-2.112	0.038
ENSG00000229255	RP11-407H12.8	-2.681	-4.737	-3.841	0.000
ENSG00000229543	RP11-90J7.2	1.034	-2.351	2.306	0.024
ENSG00000229618	AC011288.2	-1.029	0.462	-2.018	0.047
ENSG00000229619	MBNL1-AS1	-0.364	2.925	-2.019	0.047
ENSG00000230124	ACBD6	-0.189	4.720	-2.060	0.043
ENSG00000230777	RP11-318C24.1	-0.832	-0.631	-2.241	0.028
ENSG00000230833	RPEP3	1.361	-1.994	2.891	0.005
ENSG00000231028	LINC00271	0.392	1.875	2.011	0.048
ENSG00000231312	AC007246.3	-0.323	4.300	-2.399	0.019
ENSG00000231345	BEND3P1	-1.466	-0.855	-2.295	0.025
ENSG00000231612	RP11-522M21.3	-0.934	-2.174	-2.131	0.037
ENSG00000231689	LINC01090	-0.723	0.053	-2.243	0.028
ENSG00000233123	LINC01007	-1.982	-2.589	-2.353	0.021
ENSG00000233230	AC079807.2	0.476	-0.533	2.083	0.041
ENSG00000233329	AC068287.1	1.203	-2.960	2.173	0.033
ENSG00000233469	ST6GALNAC4P1	1.072	-2.034	2.272	0.026
ENSG00000233621	LINC01137	1.007	-1.454	2.027	0.046
ENSG00000233797	UFL1-AS1	-0.665	-1.028	-2.018	0.047
ENSG00000233799	AC139887.4	1.272	-1.809	2.919	0.005
ENSG00000234031	RPS3AP44	-0.943	0.832	-2.214	0.030
ENSG00000234527	PCDH9-AS1	0.921	-1.432	2.032	0.046
ENSG00000234586	RP11-382H24.2	1.447	-3.963	2.192	0.032
ENSG00000234661	CHL1-AS1	0.581	1.631	2.561	0.013
ENSG00000234772	LINC00412	-0.860	-1.520	-1.996	0.050
ENSG00000234817	RP3-400B16.1	0.533	-0.228	2.483	0.015
ENSG00000234998	RP11-439A17.10	1.048	-1.673	3.477	0.001
ENSG00000235140	RP11-135D11.2	1.000	-2.554	2.141	0.036
ENSG00000235280	MCF2L-AS1	1.049	-1.287	2.574	0.012
ENSG00000235298	RP11-575L7.8	0.512	-0.831	2.094	0.040
ENSG00000236107	AC010127.3	-0.371	3.122	-2.032	0.046
ENSG00000236408	RP11-476H24.1	-0.680	0.332	-2.124	0.037
ENSG00000236451	AC067956.1	-0.742	1.245	-2.047	0.044
ENSG00000236452	AC123023.1	-1.500	-3.024	-2.182	0.032
ENSG00000236782	RP11-96L14.7	1.035	-1.508	2.189	0.032
ENSG00000236861	AC006378.2	0.765	-0.755	3.462	0.001

ENSG00000237317	RP4-809F4.1	-0.863	-1.399	-2.179	0.033
ENSG00000237387	CTA-407F11.7	0.769	-0.778	2.120	0.038
ENSG00000237586	RP11-383J24.2	1.308	-2.386	3.099	0.003
ENSG00000237684	RPSAP35	-2.247	-5.005	-2.719	0.008
ENSG00000237709	EEF1A1P28	-1.148	-1.970	-2.562	0.013
ENSG00000237713	AC006000.5	-1.599	-2.443	-2.275	0.026
ENSG00000238004	AC009227.3	1.092	-2.540	2.066	0.042
ENSG00000238045	AC009133.12	0.500	0.440	2.666	0.009
ENSG00000238086	PPP1R26P1	0.773	-0.970	2.019	0.047
ENSG00000238279	BX470102.3	1.220	-2.312	2.261	0.027
ENSG00000238288	MTND3P23	0.785	-1.414	2.121	0.037
ENSG00000238372	snoU13	-1.521	-4.658	-2.135	0.036
ENSG00000238713	Y_RNA	-0.777	-1.191	-2.260	0.027
ENSG00000239122	RNU2-11P	0.902	-2.377	2.084	0.041
ENSG00000239486	RP11-163E9.1	1.030	-1.117	2.484	0.015
ENSG00000240591	RP11-247I13.11	0.910	-1.910	2.479	0.016
ENSG00000242103	RP11-10G15.4	-0.830	0.576	-2.443	0.017
ENSG00000242125	SNHG3	0.326	2.821	2.198	0.031
ENSG00000242525	OR7E100P	1.156	-1.600	2.938	0.004
ENSG00000242818	RN7SL846P	0.897	-1.425	2.367	0.021
ENSG00000243094	RP11-1084A12.1	0.758	-1.220	2.252	0.027
ENSG00000243574	RP11-264K23.1	0.572	-0.232	2.274	0.026
ENSG00000243678	NME2	0.868	-0.245	2.496	0.015
ENSG00000244588	RAD21L1	1.637	-1.585	2.467	0.016
ENSG00000246250	RP11-613D13.5	0.458	1.434	2.308	0.024
ENSG00000246863	RP11-325N19.3	1.170	-1.936	2.522	0.014
ENSG00000247556	OIP5-AS1	-0.417	9.103	-2.552	0.013
ENSG00000248161	RP11-499E18.1	-0.662	-0.256	-2.475	0.016
ENSG00000248174	LINC02268	1.944	-1.634	2.479	0.016
ENSG00000248294	CTD-2023N9.2	-1.508	-3.526	-2.226	0.029
ENSG00000248371	LINC02056	-0.668	-0.960	-2.055	0.044
ENSG00000248599	FLJ42969	1.073	-2.176	2.084	0.041
ENSG00000248746	ACTN3	-1.027	-1.965	-2.377	0.020
ENSG00000248837	RP11-412P11.1	-1.452	-5.017	-2.011	0.048
ENSG00000249605	CTC-218H9.1	-0.668	0.087	-2.537	0.013
ENSG00000251008	ORAOV1P1	0.793	-1.216	2.181	0.032
ENSG00000251056	ANKRD20A17P	0.983	0.970	2.067	0.042
ENSG00000251220	RFPL4AP3	-1.541	-5.213	-2.189	0.032
ENSG00000251423	CTB-51A17.1	-1.508	-2.705	-2.014	0.048
ENSG00000252438	SNORD45	-0.982	-1.556	-2.155	0.034
ENSG00000253107	CTD-2544L4.1	-0.707	-0.618	-2.330	0.023
ENSG00000253121	RP11-659E9.4	-1.498	-2.383	-3.038	0.003
ENSG00000253175	RP11-267M23.6	1.009	-2.153	2.088	0.040
ENSG00000253218	KLF3P1	0.573	-0.145	2.065	0.043
ENSG00000253300	RP11-108E14.1	1.219	-2.384	2.080	0.041
ENSG00000253311	LINC01847	-0.735	-1.104	-2.092	0.040
ENSG00000253317	RP11-142A23.1	-1.556	-4.687	-2.007	0.049

ENSG00000253503	RP11-653B10.1	-1.566	-3.944	-2.139	0.036
ENSG00000253965	CTC-329D1.3	0.822	-1.662	2.279	0.026
ENSG00000254459	RP11-91P24.7	1.189	-1.560	2.588	0.012
ENSG00000254461	RP11-755F10.3	0.728	0.814	2.605	0.011
ENSG00000254577	RP11-484D2.4	-0.660	-0.617	-2.185	0.032
ENSG00000254582	PSMA2P1	0.684	-0.915	2.245	0.028
ENSG00000254689	LINC02235	0.918	-1.747	2.417	0.018
ENSG00000255384	RP11-770J1.4	0.418	0.297	2.246	0.028
ENSG00000255471	RP11-736K20.5	-0.435	2.291	-2.702	0.009
ENSG00000255489	CTC-830E23.1	0.954	-1.292	2.103	0.039
ENSG00000256234	RP11-283G6.4	0.573	-0.580	2.135	0.036
ENSG00000257433	RP1-197B17.3	0.633	0.098	2.413	0.018
ENSG00000257842	LINC02588	0.880	-0.419	3.004	0.004
ENSG00000258204	RP11-90E9.1	-0.587	1.999	-2.068	0.042
ENSG00000258422	RP11-486O13.2	-0.881	-1.547	-2.169	0.033
ENSG00000258520	RP11-363J20.2	0.728	-1.033	2.293	0.025
ENSG00000258548	LINC00645	1.159	2.748	2.112	0.038
ENSG00000258719	RP11-232C2.3	-0.610	-0.538	-2.120	0.037
ENSG00000259056	DUXAP1	-0.790	-0.306	-2.029	0.046
ENSG00000259363	CTD-2054N24.2	1.504	-0.499	4.120	0.000
ENSG00000259678	RP11-707P17.1	-0.531	-0.543	-2.092	0.040
ENSG00000259846	RP11-467L24.1	-0.836	-1.711	-2.001	0.049
ENSG00000259867	RP11-525K10.3	-0.771	-1.547	-2.211	0.030
ENSG00000259895	RP11-715J22.2	1.149	-1.567	2.359	0.021
ENSG00000260103	RP11-10O17.1	0.772	-1.135	2.048	0.044
ENSG00000260317	RP11-48B3.4	-0.454	0.631	-2.018	0.047
ENSG00000260588	RP11-930P14.2	1.393	-2.685	2.518	0.014
ENSG00000260971	RP11-504A18.1	0.967	-0.574	2.326	0.023
ENSG00000261402	RP11-378I6.1	-1.120	1.777	-2.039	0.045
ENSG00000261757	AC005592.3	1.029	-1.452	2.038	0.045
ENSG00000261838	RP11-303E16.6	0.843	-0.697	2.521	0.014
ENSG00000261842	RP11-143I21.1	0.896	-1.158	2.106	0.039
ENSG00000262248	RP11-235E17.5	0.716	-1.089	2.159	0.034
ENSG00000262296	RP11-55L4.1	-1.495	-4.733	-2.127	0.037
ENSG00000263155	MYZAP	1.600	-2.870	2.523	0.014
ENSG00000263667	RP3-416J7.4	-1.817	-2.277	-2.370	0.020
ENSG00000264472	RP11-861L17.3	-0.686	-0.376	-2.519	0.014
ENSG00000264577	AC010761.8	0.675	0.045	2.745	0.008
ENSG00000265798	RP11-271K11.5	0.551	0.608	2.821	0.006
ENSG00000266489	RP11-178F10.3	-1.952	-4.411	-2.253	0.027
ENSG00000267083	KRT18P61	-0.610	-0.583	-2.172	0.033
ENSG00000268093	AC022154.7	0.854	-0.703	2.537	0.013
ENSG00000268455	RP11-359H18.2	1.550	-2.038	2.606	0.011
ENSG00000268945	CTD-2192J16.26	1.520	-2.603	2.684	0.009
ENSG00000269296	AC005614.3	-1.239	-2.425	-2.170	0.033
ENSG00000269559	RP11-629B11.5	0.759	-1.138	2.233	0.029
ENSG00000269873	CTD-2587H19.3	0.827	-0.634	1.999	0.049

ENSG00000270265	RP11-731D1.4	-1.478	-2.651	-2.191	0.032
ENSG00000270620	RP1-134O19.3	-0.681	-1.198	-2.415	0.018
ENSG00000270948	RP11-460N20.7	0.815	-0.982	2.576	0.012
ENSG00000271275	AC007326.9	-0.874	-0.120	-2.488	0.015
ENSG00000271811	RP1-79C4.4	0.780	0.535	2.149	0.035
ENSG00000271976	RP11-884K10.7	0.293	2.876	2.268	0.026
ENSG00000272103	CTD-2653M23.3	0.509	0.824	2.241	0.028
ENSG00000272361	GS1-166A23.2	-0.676	0.050	-2.198	0.031
ENSG00000272540	XXbac-BPG252P9.9	0.918	-2.000	2.114	0.038
ENSG00000272734	ADIRF-AS1	0.867	-0.115	2.895	0.005
ENSG00000272769	RP11-725P16.2	1.174	-1.186	2.840	0.006
ENSG00000272914	RP11-330O11.3	0.748	-0.588	2.589	0.012
ENSG00000273091	AP000255.6	-0.516	0.171	-2.070	0.042
ENSG00000273293	RP11-445N20.3	-0.712	-1.504	-2.201	0.031
ENSG00000273329	RP11-448A19.1	0.377	3.810	2.232	0.029
ENSG00000273492	AP000230.1	0.602	2.022	2.066	0.042
ENSG00000273712	RP5-874C20.7	-0.770	-1.941	-2.297	0.025
ENSG00000273890	RP11-603J24.21	1.063	-1.737	2.619	0.011
ENSG00000274238	RP11-115D19.3	-0.889	-1.224	-2.168	0.034
ENSG00000274363	RP11-104O19.4	0.689	-0.993	2.328	0.023
ENSG00000274507	RP5-965G21.5	0.946	-1.515	2.281	0.026
ENSG00000275450	PTGER4P2-CDK2AP2	1.064	-2.028	2.397	0.019
ENSG00000276107	CTD-2033D15.2	-1.003	-0.410	-2.473	0.016
ENSG00000276663	RP11-407G23.7	0.471	0.610	2.097	0.040
ENSG00000276730	Hammerhead_HH9	0.645	-1.260	2.055	0.044
ENSG00000277112	RP11-755J8.1	0.822	0.714	2.358	0.021
ENSG00000277954	RP11-679B19.1	-0.462	2.639	-2.131	0.037
ENSG00000278231	RP5-906C1.1	1.328	-1.074	2.905	0.005
ENSG00000278932	bP-21264C1.2	0.778	1.925	2.293	0.025
ENSG00000279130	RP11-278J6.4	-1.511	-3.016	-2.770	0.007
ENSG00000279180	RP11-417O18.1	1.275	-2.090	2.430	0.018
ENSG00000279208	bP-21264C1.1	1.242	1.407	2.584	0.012
ENSG00000279266	RP11-371I20.2	0.956	-1.544	2.066	0.042
ENSG00000279570	CTD-2357A8.2	0.641	0.291	2.018	0.047
ENSG00000279641	CTD-2545G14.6	0.854	-1.405	2.446	0.017
ENSG00000280010	AP001350.4	-0.549	-0.115	-2.095	0.040
ENSG00000280384	RP4-695O20.1	1.093	-1.326	2.633	0.010
ENSG00000280406	RP11-379L18.2	0.577	-0.450	2.042	0.045
ENSG00000283025	RP11-477N12.5	-1.753	-5.083	-2.251	0.027
ENSG00000283103	LLNLR-245B6.1	0.373	0.755	2.055	0.044
ENSG00000285509	#N/A	0.794	-0.360	2.633	0.010
ENSG00000285578	#N/A	-2.630	0.498	-2.795	0.007
ENSG00000285727	#N/A	-1.170	-2.080	-2.200	0.031
ENSG00000285754	#N/A	-1.425	-5.254	-2.336	0.022
ENSG00000285991	#N/A	-0.367	0.882	-2.067	0.042

vSUB

gene_id	gene_name	logFC	AveExpr	t	P.Value
ENSG00000000419	DPM1	0.364	3.891	2.033	0.047
ENSG00000000460	C1orf112	0.301	2.561	2.332	0.023
ENSG00000001561	ENPP4	0.332	6.473	2.313	0.024
ENSG00000003393	ALS2	0.200	6.580	2.476	0.016
ENSG00000003509	NDUFAF7	0.208	4.734	2.016	0.049
ENSG00000004897	CDC27	0.211	6.950	2.045	0.045
ENSG00000004948	CALCR	1.148	-0.690	2.346	0.022
ENSG00000005700	IBTK	0.203	6.803	2.058	0.044
ENSG00000006576	PHTF2	0.267	5.430	3.386	0.001
ENSG00000006652	IFRD1	0.241	5.694	2.287	0.026
ENSG00000007372	PAX6	-0.408	4.616	-2.227	0.030
ENSG00000010219	DYRK4	0.190	3.377	2.189	0.033
ENSG00000011451	WIZ	-0.382	4.051	-2.344	0.023
ENSG00000016391	CHDH	-0.277	4.299	-2.146	0.036
ENSG00000016864	GLT8D1	0.225	4.381	2.047	0.045
ENSG00000018189	RUFY3	0.359	8.768	2.337	0.023
ENSG00000018510	AGPS	0.249	6.186	2.111	0.039
ENSG00000021574	SPAST	0.186	6.478	2.083	0.042
ENSG00000023041	ZDHHC6	0.220	4.494	2.336	0.023
ENSG00000025156	HSF2	0.316	4.512	2.401	0.020
ENSG00000026652	AGPAT4	0.234	6.602	2.040	0.046
ENSG00000027847	B4GALT7	-0.191	2.301	-2.032	0.047
ENSG00000031081	ARHGAP31	-0.271	5.573	-2.077	0.042
ENSG00000031691	CENPQ	0.365	2.589	2.207	0.031
ENSG00000033178	UBA6	0.208	6.837	2.331	0.023
ENSG00000035687	ADSS	0.398	5.880	2.625	0.011
ENSG00000039523	FAM65A	-0.394	4.507	-2.954	0.005
ENSG00000041988	THAP3	0.320	2.365	2.925	0.005
ENSG00000047188	YTHDC2	0.211	6.451	2.279	0.026
ENSG00000047932	GOPC	0.215	6.000	2.027	0.047
ENSG00000048162	NOP16	0.227	2.052	2.170	0.034
ENSG00000048392	RRM2B	0.286	6.180	2.045	0.045
ENSG00000048544	MRPS10	0.288	4.940	2.250	0.028
ENSG00000049239	H6PD	-0.281	4.536	-2.561	0.013
ENSG00000050555	LAMC3	-0.436	1.141	-2.396	0.020
ENSG00000053900	ANAPC4	0.270	5.251	2.570	0.013
ENSG00000054116	TRAPPC3	0.293	4.463	2.469	0.017
ENSG00000055118	KCNH2	-0.389	3.045	-2.391	0.020
ENSG00000055208	TAB2	-0.178	6.746	-2.017	0.048
ENSG00000056050	HPF1	0.348	3.609	2.296	0.025
ENSG00000056487	PHF21B	-0.304	2.599	-2.038	0.046
ENSG00000057757	PITHD1	0.356	5.980	2.110	0.039
ENSG00000058063	ATP11B	0.229	6.257	2.966	0.004
ENSG00000059915	PSD	-0.498	6.482	-2.334	0.023
ENSG00000061337	LZTS1	-0.520	4.769	-2.053	0.045

ENSG00000063169	GLTSCR1	-0.479	1.722	-2.251	0.028
ENSG00000063245	EPN1	-0.254	4.808	-2.142	0.036
ENSG00000063660	GPC1	-0.331	4.939	-2.544	0.014
ENSG00000064995	TAF11	0.189	3.910	2.466	0.017
ENSG00000065150	IPO5	0.241	7.511	2.093	0.041
ENSG00000065328	MCM10	0.858	-2.360	2.080	0.042
ENSG00000065548	ZC3H15	0.299	7.034	2.079	0.042
ENSG00000065665	SEC61A2	0.308	5.232	2.453	0.017
ENSG00000065675	PRKCQ	0.375	3.604	2.019	0.048
ENSG00000066379	ZNRD1	0.387	1.022	2.625	0.011
ENSG00000066651	TRMT11	0.244	5.547	2.626	0.011
ENSG00000066827	ZFAT	-0.224	3.804	-2.747	0.008
ENSG00000068489	PRR11	-0.281	1.352	-2.181	0.033
ENSG00000070367	EXOC5	0.207	7.187	2.008	0.049
ENSG00000070444	MNT	-0.326	4.029	-2.031	0.047
ENSG00000070495	JMJD6	0.222	4.548	2.120	0.038
ENSG00000070785	EIF2B3	0.264	4.121	2.228	0.030
ENSG00000070831	CDC42	0.491	4.650	2.281	0.026
ENSG00000071073	MGAT4A	0.307	6.593	2.384	0.020
ENSG00000072041	SLC6A15	0.476	6.375	2.318	0.024
ENSG00000072042	RDH11	0.236	6.218	2.049	0.045
ENSG00000072071	ADGRL1	-0.301	6.555	-2.061	0.044
ENSG00000072135	PTPN18	-0.255	4.267	-2.114	0.039
ENSG00000072849	DERL2	0.170	4.232	2.023	0.048
ENSG00000072952	MRVI1	-0.379	6.687	-2.144	0.036
ENSG00000073150	PANX2	-0.451	3.259	-2.102	0.040
ENSG00000073921	PICALM	0.210	8.061	2.030	0.047
ENSG00000075043	KCNQ2	-0.437	7.253	-2.304	0.025
ENSG00000075413	MARK3	0.107	7.000	2.216	0.031
ENSG00000075461	CACNG4	-0.446	3.428	-2.236	0.029
ENSG00000076003	MCM6	0.286	3.838	2.237	0.029
ENSG00000076043	REXO2	0.290	3.984	3.175	0.002
ENSG00000077152	UBE2T	0.757	1.959	2.591	0.012
ENSG00000077254	USP33	0.261	7.346	2.224	0.030
ENSG00000077380	DYNC1I2	0.314	6.665	2.194	0.032
ENSG00000077585	GPR137B	-0.299	5.024	-3.145	0.003
ENSG00000078081	LAMP3	-0.902	-1.638	-2.307	0.025
ENSG00000078098	FAP	-0.612	1.019	-2.163	0.035
ENSG00000078124	ACER3	0.247	6.214	2.464	0.017
ENSG00000078246	TULP3	-0.318	3.922	-2.690	0.009
ENSG00000078668	VDAC3	0.383	5.891	2.192	0.032
ENSG00000078967	UBE2D4	-0.155	4.503	-2.263	0.027
ENSG00000079313	REXO1	-0.466	3.894	-2.571	0.013
ENSG00000079335	CDC14A	-0.433	3.691	-3.366	0.001
ENSG00000079432	CIC	-0.368	5.417	-2.425	0.018
ENSG00000080819	CPOX	0.367	4.620	2.568	0.013
ENSG00000080845	DLGAP4	-0.688	5.668	-3.211	0.002

ENSG00000081307	UBA5	0.243	5.839	2.387	0.020
ENSG00000081853	PCDHGA2	-0.279	2.918	-2.555	0.013
ENSG00000081870	HSPB11	0.232	3.612	2.404	0.019
ENSG00000082515	MRPL22	0.220	3.309	2.033	0.047
ENSG00000082805	ERC1	-0.200	8.121	-2.261	0.028
ENSG00000082898	XPO1	0.184	7.555	2.095	0.041
ENSG00000083520	DIS3	0.194	6.118	2.040	0.046
ENSG00000084112	SSH1	-0.197	5.732	-2.297	0.025
ENSG00000085832	EPS15	0.237	8.635	2.100	0.040
ENSG00000085872	CHERP	-0.384	2.883	-2.165	0.035
ENSG00000087053	MTMR2	0.246	5.823	2.359	0.022
ENSG00000087263	OGFOD1	0.148	5.164	2.109	0.039
ENSG00000087903	RFX2	-0.423	2.615	-2.592	0.012
ENSG00000088836	SLC4A11	-0.688	0.988	-3.046	0.003
ENSG00000090520	DNAJB11	0.268	4.285	2.388	0.020
ENSG00000090857	PDPR	-0.374	5.077	-2.093	0.041
ENSG00000091127	PUS7	0.219	4.336	2.753	0.008
ENSG00000091844	RGS17	0.279	5.269	2.172	0.034
ENSG00000092199	HNRNPC	0.212	8.131	2.242	0.029
ENSG00000092208	GEMIN2	0.303	2.834	2.256	0.028
ENSG00000092820	EZR	-0.289	6.859	-2.171	0.034
ENSG00000094880	CDC23	0.267	4.463	2.331	0.023
ENSG00000095015	MAP3K1	-0.214	5.133	-2.071	0.043
ENSG00000095380	NANS	0.296	3.592	2.362	0.022
ENSG00000095485	CWF19L1	0.260	3.737	2.082	0.042
ENSG00000095587	TLL2	0.403	2.858	2.113	0.039
ENSG00000096092	TMEM14A	0.506	4.447	2.185	0.033
ENSG00000097046	CDC7	0.335	3.870	2.177	0.034
ENSG00000099204	ABLIM1	-0.251	8.083	-2.950	0.005
ENSG00000099308	MAST3	-0.363	6.593	-2.136	0.037
ENSG00000099625	CBARP	-0.587	2.341	-2.806	0.007
ENSG00000099814	CEP170B	-0.444	5.931	-2.756	0.008
ENSG00000099822	HCN2	-0.537	4.810	-3.590	0.001
ENSG00000099864	PALM	-0.411	5.860	-2.516	0.015
ENSG00000099904	ZDHHC8	-0.311	2.975	-2.064	0.044
ENSG00000099953	MMP11	-0.803	-2.583	-2.110	0.039
ENSG00000100241	SBF1	-0.314	6.531	-2.332	0.023
ENSG00000100304	TTLL12	-0.272	3.717	-2.518	0.015
ENSG00000100350	FOXRED2	-0.371	4.312	-2.425	0.018
ENSG00000100387	RBX1	0.278	3.873	3.040	0.004
ENSG00000100433	KCNK10	0.334	4.167	2.264	0.027
ENSG00000100483	VCPKMT	0.515	3.304	2.360	0.022
ENSG00000100519	PSMC6	0.482	5.681	2.228	0.030
ENSG00000100554	ATP6V1D	0.404	6.013	2.139	0.037
ENSG00000100564	PIGH	0.352	2.426	2.721	0.009
ENSG00000100567	PSMA3	0.254	5.418	2.453	0.017
ENSG00000100575	TIMM9	0.251	2.828	2.158	0.035

ENSG00000100767	PAPLN	-0.388	3.887	-2.280	0.026
ENSG00000100902	PSMA6	0.256	3.053	2.566	0.013
ENSG00000100916	BRMS1L	0.291	4.663	2.084	0.042
ENSG00000101104	PABPC1L	-0.336	2.700	-2.042	0.046
ENSG00000101236	RNF24	-0.221	5.457	-2.013	0.049
ENSG00000101266	CSNK2A1	0.182	6.891	2.723	0.009
ENSG00000101298	SNPH	-0.352	6.403	-2.490	0.016
ENSG00000101363	MANBAL	-0.291	3.359	-2.900	0.005
ENSG00000101400	SNTA1	-0.302	5.011	-2.248	0.028
ENSG00000101782	RIOK3	0.233	5.314	2.351	0.022
ENSG00000102805	CLN5	0.220	4.625	2.231	0.030
ENSG00000102897	LYRM1	0.272	3.836	2.153	0.036
ENSG00000102981	PARD6A	-0.353	1.923	-2.201	0.032
ENSG00000102996	MMP15	-0.379	1.902	-2.239	0.029
ENSG00000103150	MLYCD	-0.389	4.733	-2.964	0.004
ENSG00000103248	MTHFSD	-0.189	2.767	-2.390	0.020
ENSG00000103540	CCP110	0.274	7.510	2.243	0.029
ENSG00000103657	HERC1	0.233	9.121	2.094	0.041
ENSG00000103707	MTFMT	0.291	3.151	2.451	0.017
ENSG00000104381	GDAP1	0.286	7.439	2.004	0.050
ENSG00000104643	MTMR9	0.274	6.513	2.012	0.049
ENSG00000104804	TULP2	-0.766	-1.991	-2.190	0.033
ENSG00000104853	CLPTM1	-0.206	5.928	-2.039	0.046
ENSG00000105063	PPP6R1	-0.344	5.462	-2.384	0.020
ENSG00000105085	MED26	-0.339	1.458	-2.494	0.016
ENSG00000105088	OLFM2	-0.411	4.840	-2.936	0.005
ENSG00000105171	POP4	0.329	3.734	2.161	0.035
ENSG00000105204	DYRK1B	-0.353	2.989	-2.749	0.008
ENSG00000105576	TNPO2	-0.247	6.295	-2.318	0.024
ENSG00000105618	PRPF31	0.190	3.865	2.106	0.040
ENSG00000105642	KCNN1	-0.520	4.389	-2.429	0.018
ENSG00000105662	CRTC1	-0.540	4.579	-2.620	0.011
ENSG00000105737	GRIK5	-0.387	4.640	-2.112	0.039
ENSG00000105738	SIPA1L3	-0.555	5.351	-2.383	0.020
ENSG00000105819	PMPCB	0.269	5.538	2.171	0.034
ENSG00000105829	BET1	0.326	3.744	2.758	0.008
ENSG00000105849	TWISTNB	0.293	4.653	2.233	0.029
ENSG00000105889	STEAP1B	0.445	1.474	2.034	0.047
ENSG00000105989	WNT2	1.171	-0.856	2.626	0.011
ENSG00000105993	DNAJB6	0.258	5.671	2.138	0.037
ENSG00000106086	PLEKHA8	0.178	4.782	2.298	0.025
ENSG00000106330	MOSPD3	-0.476	0.501	-2.590	0.012
ENSG00000106571	GLI3	-0.584	4.483	-3.637	0.001
ENSG00000106588	PSMA2	0.360	2.893	3.328	0.002
ENSG00000106733	NMRK1	0.528	3.339	2.539	0.014
ENSG00000107263	RAPGEF1	-0.341	6.231	-3.453	0.001
ENSG00000107362	ABHD17B	0.276	5.327	2.275	0.027

ENSG00000107521	HPS1	-0.185	3.864	-2.259	0.028
ENSG00000107554	DNMBP	-0.203	4.009	-2.083	0.042
ENSG00000107679	PLEKHA1	0.364	6.911	2.773	0.007
ENSG00000108010	GLRX3	0.320	4.108	2.670	0.010
ENSG00000108176	DNAJC12	0.399	4.223	2.555	0.013
ENSG00000108262	GIT1	-0.522	6.483	-3.079	0.003
ENSG00000108509	CAMTA2	-0.408	5.170	-2.477	0.016
ENSG00000108515	ENO3	0.533	-0.251	2.515	0.015
ENSG00000108666	C17orf75	0.363	4.635	2.317	0.024
ENSG00000109046	WSB1	0.207	7.972	2.017	0.048
ENSG00000109062	SLC9A3R1	-0.349	3.962	-2.383	0.021
ENSG00000109066	TMEM104	-0.350	2.834	-3.030	0.004
ENSG00000109184	DCUN1D4	0.206	7.094	2.035	0.046
ENSG00000109255	NMU	1.431	-2.684	2.132	0.037
ENSG00000109501	WFS1	-0.314	5.181	-2.194	0.032
ENSG00000109805	NCAPG	0.549	-0.268	2.368	0.021
ENSG00000109854	HTATIP2	0.402	3.277	2.385	0.020
ENSG00000109956	B3GAT1	-0.407	7.434	-2.280	0.026
ENSG00000110075	PPP6R3	0.141	6.935	2.073	0.043
ENSG00000110092	CCND1	-0.276	5.000	-2.021	0.048
ENSG00000110619	CARS	0.297	4.921	3.205	0.002
ENSG00000110696	C11orf58	0.215	7.145	2.051	0.045
ENSG00000110811	P3H3	-0.442	2.418	-2.111	0.039
ENSG00000110871	COQ5	0.301	3.598	2.147	0.036
ENSG00000110911	SLC11A2	0.197	6.200	2.133	0.037
ENSG00000111052	LIN7A	0.428	5.993	2.018	0.048
ENSG00000111110	PPM1H	0.284	6.616	2.146	0.036
ENSG00000111237	VPS29	0.388	5.016	2.395	0.020
ENSG00000111300	NAA25	0.264	6.434	3.218	0.002
ENSG00000111615	KRR1	0.219	6.102	2.002	0.050
ENSG00000111711	GOLT1B	0.322	5.211	2.522	0.014
ENSG00000111716	LDHB	0.476	2.733	2.791	0.007
ENSG00000111726	CMAS	0.426	6.087	2.101	0.040
ENSG00000112031	MTRF1L	0.295	3.600	3.093	0.003
ENSG00000112033	PPARD	-0.299	4.166	-2.414	0.019
ENSG00000112200	ZNF451	0.142	7.418	2.138	0.037
ENSG00000112282	MED23	0.173	5.812	2.216	0.031
ENSG00000112339	HBS1L	0.368	7.081	3.192	0.002
ENSG00000112685	EXOC2	0.207	5.647	2.181	0.033
ENSG00000112787	FBRSL1	-0.384	3.487	-2.119	0.038
ENSG00000112855	HARS2	0.203	4.570	2.445	0.018
ENSG00000112972	HMGCS1	0.441	7.241	2.013	0.049
ENSG00000112977	DAP	-0.319	3.692	-2.666	0.010
ENSG00000113083	LOX	0.379	2.412	2.240	0.029
ENSG00000113211	PCDHB6	-0.446	2.220	-3.478	0.001
ENSG00000113384	GOLPH3	0.178	5.451	2.235	0.029
ENSG00000113460	BRIX1	0.326	3.905	2.615	0.011

ENSG00000113558	SKP1	0.210	7.562	2.118	0.038
ENSG00000113595	TRIM23	0.358	6.692	2.100	0.040
ENSG00000113597	TRAPPC13	0.246	5.554	2.008	0.049
ENSG00000113638	TTC33	0.328	5.501	2.261	0.028
ENSG00000113851	CRBN	0.221	6.704	2.484	0.016
ENSG00000114054	PCCB	0.250	4.524	2.222	0.030
ENSG00000114098	ARMC8	0.189	6.230	2.215	0.031
ENSG00000114405	C3orf14	0.393	4.938	2.121	0.038
ENSG00000114416	FXR1	0.205	7.569	2.814	0.007
ENSG00000114626	ABTB1	-0.444	3.196	-2.672	0.010
ENSG00000114790	ARHGEF26	-0.251	5.378	-2.390	0.020
ENSG00000115241	PPM1G	0.234	5.199	2.036	0.046
ENSG00000115252	PDE1A	0.690	7.019	2.233	0.029
ENSG00000115255	REEP6	-0.398	1.421	-2.157	0.035
ENSG00000115257	PCSK4	-0.488	0.165	-2.357	0.022
ENSG00000115271	GCA	0.405	4.823	2.219	0.030
ENSG00000115392	FANCL	0.365	4.699	2.055	0.044
ENSG00000115421	PAPOLG	0.242	5.428	2.561	0.013
ENSG00000115540	MOB4	0.323	5.421	2.057	0.044
ENSG00000115568	ZNF142	-0.248	4.461	-2.286	0.026
ENSG00000115828	QPCT	0.564	2.472	2.093	0.041
ENSG00000115839	RAB3GAP1	0.274	7.148	2.271	0.027
ENSG00000115902	SLC1A4	-0.256	6.346	-2.256	0.028
ENSG00000116001	TIA1	0.190	6.360	2.226	0.030
ENSG00000116062	MSH6	0.182	5.945	2.406	0.019
ENSG00000116406	EDEM3	0.268	6.698	2.249	0.028
ENSG00000116544	DLGAP3	-0.769	5.182	-3.058	0.003
ENSG00000116675	DNAJC6	0.315	8.922	2.031	0.047
ENSG00000116906	GNPAT	0.241	5.013	2.245	0.029
ENSG00000117410	ATP6V0B	0.465	3.701	2.835	0.006
ENSG00000117569	PTBP2	0.270	6.941	2.326	0.024
ENSG00000117616	RSRP1	0.190	6.164	2.276	0.027
ENSG00000117632	STMN1	0.543	8.014	2.621	0.011
ENSG00000117713	ARID1A	-0.216	6.652	-2.226	0.030
ENSG00000117862	TXNDC12	0.388	2.070	2.641	0.011
ENSG00000118246	FASTKD2	0.237	5.782	2.168	0.034
ENSG00000118655	DCLRE1B	-0.248	2.639	-2.545	0.014
ENSG00000118804	STBD1	0.559	-0.659	2.123	0.038
ENSG00000118985	ELL2	0.374	6.726	2.719	0.009
ENSG00000119041	GTF3C3	0.251	5.173	2.417	0.019
ENSG00000119227	PIGZ	-0.327	2.356	-2.071	0.043
ENSG00000119242	CCDC92	-0.313	6.261	-2.152	0.036
ENSG00000119640	ACYP1	0.375	3.508	2.871	0.006
ENSG00000119688	ABCD4	0.238	4.357	2.065	0.043
ENSG00000119715	ESRRB	-0.557	-0.017	-2.688	0.009
ENSG00000119737	GPR75	-0.274	5.277	-2.066	0.043
ENSG00000119820	YIPF4	0.189	7.046	2.008	0.049

ENSG00000119862	LGALSL	0.316	4.316	2.040	0.046
ENSG00000119888	EPCAM	0.785	2.804	2.271	0.027
ENSG00000119938	PPP1R3C	-0.373	6.122	-2.554	0.013
ENSG00000119965	C10orf88	0.358	3.758	2.634	0.011
ENSG00000119969	HELLS	0.323	3.204	2.263	0.027
ENSG00000120129	DUSP1	-0.609	4.457	-2.416	0.019
ENSG00000120438	TCP1	0.384	6.639	2.111	0.039
ENSG00000120645	IQSEC3	-0.528	5.347	-2.763	0.008
ENSG00000120656	TAF12	0.289	3.235	2.186	0.033
ENSG00000120699	EXOSC8	0.242	3.306	2.073	0.043
ENSG00000120733	KDM3B	-0.121	6.932	-2.302	0.025
ENSG00000120802	TMPO	0.247	5.358	2.230	0.030
ENSG00000120896	SORBS3	-0.393	3.157	-2.539	0.014
ENSG00000120942	UBIAD1	-0.197	3.146	-2.398	0.020
ENSG00000121022	COPS5	0.184	4.885	2.050	0.045
ENSG00000121486	TRMT1L	0.212	5.098	2.154	0.035
ENSG00000121753	ADGRB2	-0.360	6.346	-2.032	0.047
ENSG00000121964	GTDC1	0.284	6.888	2.298	0.025
ENSG00000122068	FYTTD1	0.198	6.242	2.019	0.048
ENSG00000122432	SPATA1	-0.340	1.273	-2.062	0.044
ENSG00000122515	ZMIZ2	-0.458	4.948	-2.756	0.008
ENSG00000122643	NT5C3A	0.260	3.949	2.044	0.046
ENSG00000122873	CISD1	0.554	4.885	2.359	0.022
ENSG00000123119	NECAB1	0.494	7.971	2.356	0.022
ENSG00000123213	NLN	0.193	5.814	2.111	0.039
ENSG00000123297	TSFM	0.304	3.907	2.362	0.022
ENSG00000123358	NR4A1	-1.016	2.758	-2.920	0.005
ENSG00000123384	LRP1	-0.221	8.653	-2.151	0.036
ENSG00000123416	TUBA1B	0.362	6.683	2.004	0.050
ENSG00000123473	STIL	-0.193	2.969	-2.507	0.015
ENSG00000123607	TTC21B	0.215	6.186	2.271	0.027
ENSG00000123737	EXOSC9	0.250	4.135	2.122	0.038
ENSG00000123975	CKS2	0.844	0.025	2.087	0.041
ENSG00000123989	CHPF	-0.336	3.667	-2.011	0.049
ENSG00000124207	CSE1L	0.282	6.508	2.019	0.048
ENSG00000124253	PCK1	-1.695	-0.869	-2.826	0.006
ENSG00000124370	MCEE	0.286	2.322	2.197	0.032
ENSG00000124406	ATP8A1	0.405	8.960	2.046	0.045
ENSG00000124562	SNRPC	0.419	2.911	2.219	0.030
ENSG00000124602	UNC5CL	0.540	-0.376	2.309	0.025
ENSG00000124802	EEF1E1	0.542	2.379	2.469	0.017
ENSG00000125144	MT1G	-0.531	1.143	-2.328	0.023
ENSG00000125246	CLYBL	-0.376	4.014	-2.386	0.020
ENSG00000125386	FAM193A	-0.194	5.773	-2.243	0.029
ENSG00000125398	SOX9	-0.457	6.186	-2.553	0.013
ENSG00000125503	PPP1R12C	-0.247	4.826	-2.115	0.039
ENSG00000125629	INSIG2	0.242	5.360	2.240	0.029

ENSG00000125637	PSD4	-0.352	3.007	-2.319	0.024
ENSG00000125648	SLC25A23	-0.501	6.735	-2.568	0.013
ENSG00000125703	ATG4C	0.322	5.489	2.210	0.031
ENSG00000125843	AP5S1	-0.217	3.065	-2.505	0.015
ENSG00000126005	MMP24-AS1	-0.256	3.064	-2.319	0.024
ENSG00000126461	SCAF1	-0.383	4.651	-2.228	0.030
ENSG00000126705	AHDC1	-0.400	4.123	-2.343	0.023
ENSG00000126821	SGPP1	0.291	4.986	2.146	0.036
ENSG00000127337	YEATS4	0.493	2.974	2.251	0.028
ENSG00000127527	EPS15L1	-0.322	4.825	-2.081	0.042
ENSG00000127528	KLF2	-0.672	0.779	-2.352	0.022
ENSG00000127585	FBXL16	-0.624	6.480	-2.413	0.019
ENSG00000127616	SMARCA4	-0.177	6.856	-2.011	0.049
ENSG00000127666	TICAM1	-0.480	1.043	-2.631	0.011
ENSG00000127863	TNFRSF19	-0.361	2.333	-2.060	0.044
ENSG00000127955	GNAI1	0.369	6.880	2.805	0.007
ENSG00000127980	PEX1	0.173	6.146	2.042	0.046
ENSG00000127989	MTERF1	0.192	4.381	2.165	0.035
ENSG00000127993	RBM48	0.270	4.966	3.103	0.003
ENSG00000128059	PPAT	0.295	4.340	2.534	0.014
ENSG00000128228	SDF2L1	0.563	0.049	2.425	0.018
ENSG00000128272	ATF4	0.302	6.218	2.575	0.013
ENSG00000128534	LSM8	0.196	4.353	2.038	0.046
ENSG00000128951	DUT	0.392	4.433	2.289	0.026
ENSG00000129084	PSMA1	0.331	3.484	2.097	0.040
ENSG00000129214	SHBG	-0.649	-0.958	-2.451	0.017
ENSG00000129244	ATP1B2	-0.490	7.379	-2.127	0.038
ENSG00000129911	KLF16	-0.482	2.121	-2.019	0.048
ENSG00000129951	PLPPR3	-0.386	3.120	-2.037	0.046
ENSG00000130287	NCAN	-0.308	7.250	-2.349	0.022
ENSG00000130382	MLLT1	-0.468	4.903	-3.042	0.004
ENSG00000130653	PNPLA7	-0.493	3.318	-2.654	0.010
ENSG00000130669	PAK4	-0.315	1.502	-2.132	0.037
ENSG00000130695	CEP85	0.298	4.635	2.937	0.005
ENSG00000130720	FIBCD1	-1.558	0.989	-2.574	0.013
ENSG00000130758	MAP3K10	-0.388	4.958	-2.314	0.024
ENSG00000130881	LRP3	-0.445	4.360	-2.360	0.022
ENSG00000131069	ACSS2	0.201	4.148	2.097	0.040
ENSG00000131188	PRR7	-0.505	1.066	-2.094	0.041
ENSG00000131196	NFATC1	-0.452	1.687	-2.668	0.010
ENSG00000131375	CAPN7	0.166	5.771	2.169	0.034
ENSG00000131398	KCNC3	-0.541	3.979	-2.527	0.014
ENSG00000131477	RAMP2	-0.555	-0.054	-2.275	0.027
ENSG00000131591	C1orf159	-0.323	1.653	-2.168	0.034
ENSG00000131653	TRAF7	-0.262	2.897	-2.189	0.033
ENSG00000131759	RARA	-0.397	1.983	-2.025	0.048
ENSG00000131791	PRKAB2	0.228	5.246	2.478	0.016

ENSG00000131876	SNRPA1	0.299	3.269	2.110	0.039
ENSG00000132005	RFX1	-0.426	1.164	-2.546	0.014
ENSG00000132321	IQCA1	-0.294	5.384	-2.232	0.030
ENSG00000132326	PER2	-0.354	4.756	-2.543	0.014
ENSG00000132361	CLUH	-0.202	4.616	-2.077	0.042
ENSG00000132406	TMEM128	0.355	2.907	2.817	0.007
ENSG00000132581	SDF2	0.250	3.060	2.038	0.046
ENSG00000132669	RIN2	-0.194	6.233	-2.027	0.047
ENSG00000132793	LPIN3	-0.507	0.914	-2.686	0.009
ENSG00000133119	RFC3	0.270	3.019	2.071	0.043
ENSG00000133243	BTBD2	-0.434	4.735	-3.173	0.002
ENSG00000133519	ZDHHC8P1	-0.474	2.048	-2.735	0.008
ENSG00000133641	C12orf29	0.284	4.993	2.169	0.034
ENSG00000133731	IMPA1	0.292	5.512	2.196	0.032
ENSG00000133863	TEX15	-0.349	3.945	-2.105	0.040
ENSG00000134056	MRPS36	0.402	3.265	2.003	0.050
ENSG00000134058	CDK7	0.390	3.200	2.313	0.024
ENSG00000134152	KATNBL1	0.185	5.152	2.060	0.044
ENSG00000134198	TSPAN2	0.574	2.215	2.276	0.027
ENSG00000134294	SLC38A2	0.361	7.188	2.666	0.010
ENSG00000134375	TIMM17A	0.542	4.556	2.280	0.026
ENSG00000134759	ELP2	0.165	6.675	2.217	0.031
ENSG00000134852	CLOCK	0.205	7.274	2.495	0.015
ENSG00000134897	BIVM	0.183	4.372	2.182	0.033
ENSG00000134900	TPP2	0.214	6.286	2.385	0.020
ENSG00000135045	C9orf40	0.362	2.604	2.420	0.019
ENSG00000135049	AGTPBP1	0.325	7.911	2.221	0.030
ENSG00000135094	SDS	-0.695	1.722	-3.125	0.003
ENSG00000135249	RINT1	0.196	4.169	2.027	0.047
ENSG00000135250	SRPK2	0.250	8.033	2.185	0.033
ENSG00000135439	AGAP2	-0.603	7.178	-2.656	0.010
ENSG00000135540	NHSL1	-0.415	5.528	-2.947	0.005
ENSG00000135547	HEY2	-0.494	3.594	-3.056	0.003
ENSG00000135577	NMBR	1.168	-1.384	2.932	0.005
ENSG00000135625	EGR4	-0.986	-0.090	-2.332	0.023
ENSG00000135655	USP15	0.246	6.596	2.475	0.016
ENSG00000135679	MDM2	0.144	6.701	2.053	0.045
ENSG00000135900	MRPL44	0.341	3.616	2.014	0.049
ENSG00000135932	CAB39	0.253	7.523	2.035	0.046
ENSG00000135972	MRPS9	0.269	4.507	2.145	0.036
ENSG00000136002	ARHGEF4	-0.333	6.799	-3.440	0.001
ENSG00000136158	SPRY2	-0.183	4.918	-2.028	0.047
ENSG00000136205	TNS3	-0.285	6.575	-2.055	0.044
ENSG00000136261	BZW2	0.372	3.566	2.164	0.035
ENSG00000136297	MMD2	-0.500	4.017	-2.995	0.004
ENSG00000136504	KAT7	0.154	6.066	2.030	0.047
ENSG00000136699	SMPD4	-0.210	3.561	-2.021	0.048

ENSG00000136738	STAM	0.245	5.473	2.180	0.033
ENSG00000136826	KLF4	-0.822	1.952	-2.671	0.010
ENSG00000136840	ST6GALNAC4	-0.292	1.853	-2.002	0.050
ENSG00000136848	DAB2IP	-0.354	5.833	-2.474	0.016
ENSG00000136875	PRPF4	0.256	4.374	2.154	0.035
ENSG00000136943	CTSV	0.845	0.086	2.696	0.009
ENSG00000136986	DERL1	0.208	4.739	2.207	0.031
ENSG00000137074	APTX	0.175	4.240	2.005	0.050
ENSG00000137218	FRS3	-0.378	2.410	-2.686	0.009
ENSG00000137486	ARRB1	-0.275	6.051	-2.757	0.008
ENSG00000137692	DCUN1D5	0.314	4.640	2.163	0.035
ENSG00000137747	TMPRSS13	-1.272	-0.753	-2.035	0.047
ENSG00000137876	RSL24D1	0.420	5.644	2.308	0.025
ENSG00000137941	TTLL7	0.278	8.812	2.335	0.023
ENSG00000137955	RABGGTB	0.230	6.026	2.212	0.031
ENSG00000137996	RTCA	0.293	5.335	2.296	0.025
ENSG00000138018	EPT1	0.317	7.171	2.037	0.046
ENSG00000138032	PPM1B	0.253	5.780	2.733	0.008
ENSG00000138346	DNA2	0.323	3.441	2.803	0.007
ENSG00000138381	ASNSD1	0.313	5.459	2.069	0.043
ENSG00000138399	FASTKD1	0.229	5.131	2.414	0.019
ENSG00000138430	OLA1	0.447	6.166	2.319	0.024
ENSG00000138449	SLC40A1	-0.319	4.094	-2.126	0.038
ENSG00000138698	RAP1GDS1	0.415	6.945	2.256	0.028
ENSG00000139163	ETNK1	0.297	7.121	2.255	0.028
ENSG00000139428	MMAB	0.202	3.680	2.081	0.042
ENSG00000139496	NUP58	0.229	6.589	2.165	0.035
ENSG00000139620	KANSL2	0.285	3.883	2.742	0.008
ENSG00000139697	SBNO1	0.181	7.840	2.044	0.046
ENSG00000139718	SETD1B	-0.387	3.882	-2.400	0.020
ENSG00000139726	DENR	0.284	5.898	2.333	0.023
ENSG00000140471	LINS1	0.314	4.700	2.076	0.042
ENSG00000140522	RLBP1	-0.668	1.252	-2.707	0.009
ENSG00000140836	ZFHX3	-0.350	5.527	-2.486	0.016
ENSG00000140948	ZCCHC14	-0.196	5.797	-2.127	0.038
ENSG00000141198	TOM1L1	0.284	4.028	2.006	0.050
ENSG00000141252	VPS53	-0.283	7.206	-2.355	0.022
ENSG00000141447	OSBPL1A	0.304	7.661	2.200	0.032
ENSG00000141452	C18orf8	0.233	4.141	2.519	0.015
ENSG00000141503	MINK1	-0.251	6.829	-2.081	0.042
ENSG00000141668	CBLN2	0.808	3.415	2.197	0.032
ENSG00000141905	NFIC	-0.380	5.458	-2.067	0.043
ENSG00000142039	CCDC97	-0.317	3.152	-2.476	0.016
ENSG00000142235	LMTK3	-0.424	4.686	-2.248	0.028
ENSG00000142453	CARM1	-0.199	3.558	-2.009	0.049
ENSG00000142459	EVI5L	-0.212	4.831	-2.060	0.044
ENSG00000142611	PRDM16	-0.380	3.695	-2.450	0.017

ENSG00000142751	GPN2	0.148	4.165	2.210	0.031
ENSG00000142784	WDTC1	-0.192	5.267	-2.053	0.045
ENSG00000142875	PRKACB	0.366	9.132	2.065	0.043
ENSG00000142959	BEST4	-0.832	-1.607	-2.063	0.044
ENSG00000143155	TIPRL	0.343	5.877	2.046	0.045
ENSG00000143436	MRPL9	0.356	3.428	2.323	0.024
ENSG00000143819	EPHX1	-0.346	4.689	-2.409	0.019
ENSG00000143970	ASXL2	-0.131	6.862	-2.140	0.037
ENSG00000144029	MRPS5	0.252	5.399	2.923	0.005
ENSG00000144034	TPRKB	0.272	3.044	2.079	0.042
ENSG00000144048	DUSP11	0.180	4.330	2.118	0.039
ENSG00000144161	ZC3H8	0.216	5.332	2.006	0.050
ENSG00000144339	TMEFF2	0.491	5.505	2.775	0.007
ENSG00000144476	ACKR3	-0.386	2.117	-2.486	0.016
ENSG00000144677	CTDSPL	-0.237	4.695	-2.333	0.023
ENSG00000144744	UBA3	0.230	5.576	2.133	0.037
ENSG00000145012	LPP	-0.250	6.855	-2.121	0.038
ENSG00000145113	MUC4	0.614	0.060	2.169	0.034
ENSG00000145191	EIF2B5	0.174	4.783	2.086	0.041
ENSG00000145217	SLC26A1	-0.443	0.932	-2.668	0.010
ENSG00000145293	ENOPH1	0.311	6.108	2.336	0.023
ENSG00000145335	SNCA	0.570	7.019	2.578	0.013
ENSG00000145349	CAMK2D	0.415	7.088	2.122	0.038
ENSG00000145354	CISD2	0.331	4.452	2.435	0.018
ENSG00000145451	GLRA3	0.716	4.559	2.132	0.037
ENSG00000145715	RASA1	0.179	6.668	2.121	0.038
ENSG00000145782	ATG12	0.227	6.168	2.589	0.012
ENSG00000145860	RNF145	0.307	6.200	2.131	0.037
ENSG00000146083	RNF44	-0.381	3.953	-2.608	0.012
ENSG00000146282	RARS2	0.219	4.542	2.252	0.028
ENSG00000146576	C7orf26	-0.197	2.609	-2.074	0.043
ENSG00000146731	CCT6A	0.324	6.372	2.076	0.042
ENSG00000147548	WHSC1L1	0.123	7.914	2.177	0.034
ENSG00000147649	MTDH	0.198	7.207	2.331	0.023
ENSG00000147799	ARHGAP39	-0.409	3.634	-2.725	0.009
ENSG00000147854	UHRF2	0.243	5.633	2.753	0.008
ENSG00000148090	AUH	0.346	4.505	2.111	0.039
ENSG00000148143	ZNF462	-0.249	7.011	-2.267	0.027
ENSG00000148153	INIP	0.205	5.592	2.439	0.018
ENSG00000148154	UGCG	0.447	5.470	2.382	0.021
ENSG00000148158	SNX30	0.209	6.867	2.014	0.049
ENSG00000148400	NOTCH1	-0.290	4.892	-2.267	0.027
ENSG00000148572	NRBF2	0.288	3.839	2.434	0.018
ENSG00000148634	HERC4	0.210	7.098	2.508	0.015
ENSG00000148688	RPP30	0.314	4.592	2.502	0.015
ENSG00000148730	EIF4EBP2	-0.217	7.143	-2.214	0.031
ENSG00000148834	GSTO1	0.405	4.286	2.221	0.030

ENSG00000149090	PAMR1	-0.342	4.632	-2.826	0.006
ENSG00000149328	GLB1L2	-0.399	3.904	-2.100	0.040
ENSG00000149926	FAM57B	-0.666	1.606	-3.033	0.004
ENSG00000150753	CCT5	0.402	6.007	2.005	0.050
ENSG00000150764	DIXDC1	0.277	6.807	2.104	0.040
ENSG00000150787	PTS	0.437	4.083	2.491	0.016
ENSG00000151092	NGLY1	0.261	5.015	2.557	0.013
ENSG00000151413	NUBPL	0.173	3.825	2.143	0.036
ENSG00000151631	AKR1C6P	0.639	-1.885	2.422	0.019
ENSG00000151743	AMN1	0.322	4.553	2.246	0.029
ENSG00000151929	BAG3	-0.573	4.581	-2.282	0.026
ENSG00000152034	MCHR2	1.561	-0.222	2.064	0.044
ENSG00000152076	CCDC74B	-0.481	0.616	-2.042	0.046
ENSG00000152795	HNRNPDL	0.254	8.381	2.531	0.014
ENSG00000152894	PTPRK	0.376	6.744	2.152	0.036
ENSG00000153046	CDYL	-0.236	6.453	-2.288	0.026
ENSG00000153130	SCOC	0.399	7.025	2.181	0.033
ENSG00000153214	TMEM87B	0.231	4.528	2.040	0.046
ENSG00000153443	UBALD1	-0.398	1.525	-2.059	0.044
ENSG00000153558	FBXL2	0.247	5.190	2.024	0.048
ENSG00000153561	RMND5A	0.290	7.175	2.113	0.039
ENSG00000153714	LURAP1L	0.507	1.488	2.574	0.013
ENSG00000153944	MSI2	-0.198	7.619	-2.040	0.046
ENSG00000153975	ZUFSP	0.219	3.958	2.223	0.030
ENSG00000153976	HS3ST3A1	1.184	-2.685	2.341	0.023
ENSG00000153982	GDPD1	0.365	4.639	2.362	0.022
ENSG00000154079	SDHAF4	0.356	3.027	2.060	0.044
ENSG00000154118	JPH3	-0.404	6.516	-2.270	0.027
ENSG00000154144	TBRG1	0.278	5.861	2.041	0.046
ENSG00000154153	FAM134B	0.414	5.970	2.349	0.022
ENSG00000154473	BUB3	0.230	5.320	2.421	0.019
ENSG00000154639	CXADR	0.329	5.640	2.089	0.041
ENSG00000154814	OXNAD1	0.140	4.953	2.274	0.027
ENSG00000154856	APCDD1	-0.356	5.253	-2.774	0.007
ENSG00000154889	MPPE1	0.269	3.383	2.224	0.030
ENSG00000155034	FBXL18	-0.311	2.998	-2.112	0.039
ENSG00000155100	OTUD6B	0.189	5.295	2.113	0.039
ENSG00000155254	MARVELD1	-0.331	1.527	-2.459	0.017
ENSG00000155545	MIER3	0.176	5.176	2.151	0.036
ENSG00000155858	LSM11	0.546	4.803	2.485	0.016
ENSG00000155906	RMND1	0.243	4.205	2.090	0.041
ENSG00000156049	GNA14	-0.397	3.121	-2.012	0.049
ENSG00000156374	PCGF6	0.306	2.347	2.022	0.048
ENSG00000156502	SUPV3L1	0.206	4.818	2.451	0.017
ENSG00000156795	WDYHV1	0.327	3.107	2.505	0.015
ENSG00000157150	TIMP4	-0.429	2.220	-2.408	0.019
ENSG00000157350	ST3GAL2	-0.294	4.569	-2.428	0.018

ENSG00000157653	C9orf43	-0.347	0.701	-2.035	0.046
ENSG00000157657	ZNF618	-0.251	3.204	-2.043	0.046
ENSG00000157933	SKI	-0.422	6.051	-2.821	0.007
ENSG00000158109	TPRG1L	-0.286	6.039	-2.216	0.031
ENSG00000158321	AUTS2	-0.244	6.287	-2.182	0.033
ENSG00000158411	MITD1	0.206	4.274	2.246	0.029
ENSG00000158683	PKD1L1	0.411	1.851	2.203	0.032
ENSG00000158773	USF1	0.292	4.322	2.149	0.036
ENSG00000158864	NDUFS2	0.311	6.048	2.021	0.048
ENSG00000158985	CDC42SE2	0.253	7.181	2.108	0.039
ENSG00000159210	SNF8	0.169	4.425	2.395	0.020
ENSG00000159231	CBR3	-0.459	-0.097	-2.402	0.020
ENSG00000159409	CELF3	-0.460	5.172	-2.113	0.039
ENSG00000159423	ALDH4A1	-0.401	2.503	-2.172	0.034
ENSG00000159459	UBR1	0.228	6.887	2.184	0.033
ENSG00000159593	NAE1	0.234	5.506	2.035	0.046
ENSG00000159753	CARMIL2	-0.505	3.289	-3.060	0.003
ENSG00000160321	ZNF208	-0.735	3.723	-2.590	0.012
ENSG00000160563	MED27	0.317	2.217	2.478	0.016
ENSG00000160703	NLRX1	-0.234	1.468	-2.162	0.035
ENSG00000160801	PTH1R	-0.372	2.049	-2.164	0.035
ENSG00000160877	NACC1	-0.277	4.704	-2.138	0.037
ENSG00000160972	PPP1R16A	-0.364	2.018	-2.686	0.009
ENSG00000161509	GRIN2C	-0.428	4.057	-2.664	0.010
ENSG00000161526	SAP30BP	0.192	5.113	2.328	0.023
ENSG00000161542	PRPSAP1	0.213	4.614	2.340	0.023
ENSG00000161681	SHANK1	-0.722	5.506	-2.065	0.043
ENSG00000161905	ALOX15	-0.750	-1.097	-2.131	0.037
ENSG00000161912	ADCY10P1	0.302	3.767	2.075	0.042
ENSG00000161929	SCIMP	-0.477	1.157	-2.563	0.013
ENSG00000161980	POLR3K	0.260	2.439	2.113	0.039
ENSG00000161999	JMJD8	-0.272	1.770	-2.053	0.045
ENSG00000162222	TTC9C	0.273	3.301	2.337	0.023
ENSG00000162384	C1orf123	0.191	4.236	2.073	0.043
ENSG00000162430	SEPN1	-0.411	4.064	-2.391	0.020
ENSG00000162512	SDC3	-0.289	6.947	-2.287	0.026
ENSG00000162521	RBBP4	0.174	6.703	2.249	0.028
ENSG00000162576	MXRA8	-0.574	2.904	-3.483	0.001
ENSG00000162599	NFIA	-0.258	7.880	-2.140	0.037
ENSG00000162639	HENMT1	0.505	3.378	2.302	0.025
ENSG00000162961	DPY30	0.348	3.921	2.303	0.025
ENSG00000162972	C2orf47	0.287	2.838	2.478	0.016
ENSG00000163032	VSNL1	0.643	9.159	2.009	0.049
ENSG00000163171	CDC42EP3	0.318	3.921	2.003	0.050
ENSG00000163319	MRPS18C	0.390	3.007	2.995	0.004
ENSG00000163347	CLDN1	-0.426	1.836	-2.097	0.040
ENSG00000163393	SLC22A15	0.258	5.683	2.207	0.031

ENSG00000163395	IGFN1	-0.739	1.576	-2.233	0.029
ENSG00000163468	CCT3	0.258	6.236	2.004	0.050
ENSG00000163539	CLASP2	0.266	9.221	2.243	0.029
ENSG00000163605	PPP4R2	0.254	6.518	2.249	0.028
ENSG00000163607	GTPBP8	0.261	3.337	2.260	0.028
ENSG00000163682	RPL9	0.580	5.208	2.167	0.034
ENSG00000163683	SMIM14	0.302	6.856	2.550	0.013
ENSG00000163808	KIF15	0.252	1.936	2.046	0.045
ENSG00000163882	POLR2H	0.220	3.279	2.570	0.013
ENSG00000163938	GNL3	0.364	5.303	2.367	0.021
ENSG00000163964	PIGX	0.301	5.144	2.229	0.030
ENSG00000164031	DNAJB14	0.315	7.526	2.079	0.042
ENSG00000164061	BSN	-0.444	7.841	-2.057	0.044
ENSG00000164109	MAD2L1	0.631	2.380	2.197	0.032
ENSG00000164176	EDIL3	0.403	8.143	2.137	0.037
ENSG00000164180	TMEM161B	0.205	5.269	2.312	0.024
ENSG00000164209	SLC25A46	0.339	7.151	2.092	0.041
ENSG00000164252	AGGF1	0.248	4.801	2.343	0.023
ENSG00000164284	GRPEL2	0.343	4.820	2.429	0.018
ENSG00000164347	GFM2	0.330	5.395	2.251	0.028
ENSG00000164379	FOXQ1	-0.594	0.345	-2.328	0.023
ENSG00000164385	LINC01600	-0.489	0.178	-2.031	0.047
ENSG00000164465	DCBLD1	0.484	3.612	2.233	0.029
ENSG00000164494	PDSS2	0.229	3.672	2.106	0.040
ENSG00000164850	GPER1	-0.395	1.950	-2.147	0.036
ENSG00000164941	INTS8	0.198	5.455	2.146	0.036
ENSG00000164976	KIAA1161	-0.300	5.875	-2.097	0.040
ENSG00000165238	WNK2	-0.426	6.046	-2.350	0.022
ENSG00000165506	DNAAF2	0.259	3.077	2.051	0.045
ENSG00000165553	NGB	1.076	-1.643	2.059	0.044
ENSG00000165630	PRPF18	0.294	4.150	2.251	0.028
ENSG00000165637	VDAC2	0.362	4.726	2.139	0.037
ENSG00000165802	NSMF	-0.548	7.147	-2.453	0.017
ENSG00000165804	ZNF219	-0.404	2.728	-2.568	0.013
ENSG00000165821	SALL2	-0.309	5.410	-2.483	0.016
ENSG00000165915	SLC39A13	-0.257	3.105	-2.620	0.011
ENSG00000165973	NELL1	0.512	4.970	2.220	0.030
ENSG00000166037	CEP57	0.155	5.829	2.014	0.049
ENSG00000166123	GPT2	-0.291	5.941	-2.440	0.018
ENSG00000166181	API5	0.290	6.177	2.044	0.046
ENSG00000166188	ZNF319	-0.360	2.087	-2.906	0.005
ENSG00000166226	CCT2	0.386	6.024	2.238	0.029
ENSG00000166341	DCHS1	-0.321	4.444	-2.184	0.033
ENSG00000166352	C11orf74	0.322	3.471	2.062	0.044
ENSG00000166359	WDR88	-0.420	0.603	-2.745	0.008
ENSG00000166716	ZNF592	-0.314	5.407	-2.831	0.006
ENSG00000166897	ELFN2	-0.385	5.323	-2.028	0.047

ENSG00000167100	SAMD14	-0.505	3.083	-2.633	0.011
ENSG00000167182	SP2	-0.386	2.537	-2.645	0.011
ENSG00000167261	DPEP2	-0.607	-0.872	-2.135	0.037
ENSG00000167325	RRM1	0.303	4.899	2.466	0.017
ENSG00000167447	SMG8	0.290	3.675	2.149	0.036
ENSG00000167491	GATAD2A	-0.320	4.961	-2.241	0.029
ENSG00000167595	PROSER3	-0.450	1.949	-2.396	0.020
ENSG00000167632	TRAPPC9	-0.144	5.166	-2.014	0.049
ENSG00000167676	PLIN4	-0.416	2.217	-2.014	0.049
ENSG00000167680	SEMA6B	-0.459	4.902	-2.518	0.015
ENSG00000167701	GPT	-0.433	0.474	-2.052	0.045
ENSG00000167779	IGFBP6	0.426	1.663	2.075	0.043
ENSG00000167904	TMEM68	0.241	4.219	2.217	0.031
ENSG00000167962	ZNF598	-0.272	2.308	-2.007	0.049
ENSG00000167971	CASKIN1	-0.457	5.043	-2.863	0.006
ENSG00000168056	LTBP3	-0.267	4.906	-2.159	0.035
ENSG00000168066	SF1	-0.174	6.379	-2.273	0.027
ENSG00000168077	SCARA3	-0.355	5.687	-2.205	0.031
ENSG00000168298	HIST1H1E	-0.248	5.446	-2.181	0.033
ENSG00000168306	ACOX2	-0.360	1.767	-2.505	0.015
ENSG00000168615	ADAM9	0.268	6.372	2.553	0.013
ENSG00000169018	FEM1B	0.309	7.795	2.363	0.022
ENSG00000169217	CD2BP2	0.186	5.115	2.328	0.023
ENSG00000169564	PCBP1	-0.257	3.104	-2.056	0.044
ENSG00000169836	TACR3	0.861	1.090	2.322	0.024
ENSG00000169955	ZNF747	-0.199	2.959	-2.071	0.043
ENSG00000170075	GPR37L1	-0.296	5.977	-2.240	0.029
ENSG00000170165	CR848007.2	-0.665	2.282	-2.399	0.020
ENSG00000170185	USP38	0.172	5.007	2.157	0.035
ENSG00000170345	FOS	-0.804	5.002	-2.008	0.049
ENSG00000170370	EMX2	-0.370	4.218	-2.239	0.029
ENSG00000170442	KRT86	-0.476	-0.136	-2.112	0.039
ENSG00000170632	ARMC10	0.252	4.436	2.899	0.005
ENSG00000170791	CHCHD7	0.250	5.304	2.122	0.038
ENSG00000171109	MFN1	0.220	6.003	2.233	0.029
ENSG00000171202	TMEM126A	0.347	2.895	2.100	0.040
ENSG00000171223	JUNB	-0.649	3.188	-2.462	0.017
ENSG00000171497	PPID	0.352	5.097	2.476	0.016
ENSG00000171791	BCL2	-0.238	5.924	-2.470	0.017
ENSG00000171819	ANGPTL7	-0.384	0.654	-2.036	0.046
ENSG00000171903	CYP4F11	-0.435	2.677	-2.535	0.014
ENSG00000171992	SYNPO	-0.530	7.159	-2.158	0.035
ENSG00000172244	C5orf34	0.305	2.490	2.077	0.042
ENSG00000172331	BPGM	0.277	4.349	2.011	0.049
ENSG00000172346	CSDC2	-0.397	3.115	-2.040	0.046
ENSG00000172354	GNB2	-0.259	3.967	-2.321	0.024
ENSG00000172403	SYNPO2	-0.366	6.524	-2.440	0.018

ENSG00000172458	IL17D	-0.244	4.611	-2.116	0.039
ENSG00000172548	NIPAL4	0.503	1.856	2.063	0.044
ENSG00000172590	MRPL52	0.215	3.038	2.122	0.038
ENSG00000172640	OR10AD1	-0.453	0.641	-2.196	0.032
ENSG00000172687	ZNF738	0.491	3.275	3.472	0.001
ENSG00000172731	LRRC20	-0.275	3.751	-2.273	0.027
ENSG00000172780	RAB43	-0.543	-1.180	-2.063	0.044
ENSG00000172992	DCAKD	-0.248	4.894	-2.597	0.012
ENSG00000173040	EVC2	-0.507	1.211	-2.542	0.014
ENSG00000173110	HSPA6	-1.510	-0.795	-2.426	0.018
ENSG00000173207	CKS1B	0.575	0.118	2.456	0.017
ENSG00000173264	GPR137	-0.326	3.031	-2.270	0.027
ENSG00000173295	FAM86B3P	-0.447	0.748	-2.051	0.045
ENSG00000173320	STOX2	0.221	7.491	2.132	0.037
ENSG00000173334	TRIB1	-0.419	4.220	-2.687	0.009
ENSG00000173402	DAG1	-0.294	5.595	-2.757	0.008
ENSG00000173451	THAP2	0.214	4.057	2.207	0.031
ENSG00000173456	RNF26	-0.315	2.257	-2.053	0.045
ENSG00000173517	PEAK1	-0.183	7.574	-2.094	0.041
ENSG00000173545	ZNF622	0.260	4.591	2.041	0.046
ENSG00000173599	PC	-0.249	4.741	-2.034	0.047
ENSG00000173641	HSPB7	-0.934	-1.333	-2.299	0.025
ENSG00000173875	ZNF791	0.152	5.540	2.215	0.031
ENSG00000174004	NRROS	-0.653	-0.551	-2.706	0.009
ENSG00000174099	MSRB3	-0.263	4.635	-2.083	0.042
ENSG00000174306	ZHX3	-0.253	7.300	-3.357	0.001
ENSG00000174442	ZWILCH	0.434	2.978	2.972	0.004
ENSG00000174446	SNAPC5	0.345	3.782	2.313	0.024
ENSG00000174456	C12orf76	0.279	5.574	2.308	0.025
ENSG00000174871	CNIH2	-0.725	3.425	-2.514	0.015
ENSG00000174990	CA5A	0.866	-2.566	2.032	0.047
ENSG00000175115	PACS1	-0.227	5.605	-2.227	0.030
ENSG00000175155	YPEL2	0.286	5.895	2.034	0.047
ENSG00000175221	MED16	-0.307	3.172	-2.788	0.007
ENSG00000175520	UBQLN3	-1.233	-2.315	-2.928	0.005
ENSG00000175604	RP11-276H1.3	0.483	-0.464	2.249	0.028
ENSG00000176087	SLC35A4	-0.217	3.305	-2.013	0.049
ENSG00000176102	CSTF3	0.229	4.761	2.427	0.018
ENSG00000176182	MYPOP	-0.343	2.412	-2.206	0.031
ENSG00000176410	DNAJC30	-0.253	2.351	-2.462	0.017
ENSG00000176532	PRR15	0.815	-1.783	2.005	0.050
ENSG00000176533	GNG7	-0.463	5.171	-2.197	0.032
ENSG00000176731	C8orf59	0.250	2.683	2.040	0.046
ENSG00000176857	GJA1P1	-0.438	2.348	-2.217	0.031
ENSG00000177000	MTHFR	-0.195	4.451	-2.193	0.032
ENSG00000177169	ULK1	-0.647	4.155	-3.472	0.001
ENSG00000177201	OR2T12	1.136	-0.704	2.409	0.019

ENSG00000177212	OR2T33	0.710	-0.544	2.032	0.047
ENSG00000177283	FZD8	-0.510	1.940	-2.432	0.018
ENSG00000177453	NIM1K	0.261	4.036	2.191	0.033
ENSG00000177465	ACOT4	-0.321	1.588	-2.144	0.036
ENSG00000177728	TMEM94	-0.308	5.338	-2.694	0.009
ENSG00000177736	FXNP2	-0.563	-0.035	-2.172	0.034
ENSG00000177807	KCNJ10	-0.292	7.273	-2.480	0.016
ENSG00000177993	ZNRF3-AS1	-0.487	0.887	-2.062	0.044
ENSG00000178074	C2orf69	0.293	5.558	2.097	0.040
ENSG00000178093	TSSK6	-0.640	-1.911	-2.381	0.021
ENSG00000178209	PLEC	-0.304	7.688	-2.332	0.023
ENSG00000178358	OR2D3	-0.730	0.279	-2.762	0.008
ENSG00000178764	ZHX2	-0.284	4.782	-2.207	0.031
ENSG00000178951	ZBTB7A	-0.400	5.841	-2.737	0.008
ENSG00000179104	TMTC2	0.340	6.156	2.036	0.046
ENSG00000179523	EIF3J-AS1	0.246	3.378	2.566	0.013
ENSG00000179772	FOXS1	-0.974	-2.409	-2.174	0.034
ENSG00000179902	C1orf194	-0.624	0.207	-2.347	0.022
ENSG00000180185	FAHD1	0.350	3.763	2.177	0.034
ENSG00000180233	ZNRF2	0.256	4.487	2.515	0.015
ENSG00000180329	CCDC43	0.309	4.732	2.511	0.015
ENSG00000180488	FAM73A	0.292	7.126	2.247	0.029
ENSG00000180667	YOD1	0.210	5.030	2.026	0.047
ENSG00000180776	ZDHHC20	0.365	6.820	2.645	0.011
ENSG00000180881	CAPS2	0.247	5.276	2.305	0.025
ENSG00000180921	FAM83H	-0.493	1.744	-2.309	0.025
ENSG00000180953	ST20	0.367	0.655	2.215	0.031
ENSG00000181163	NPM1	0.353	6.944	2.158	0.035
ENSG00000181751	C5orf30	0.306	4.850	2.941	0.005
ENSG00000182093	WRB	0.278	4.538	2.189	0.033
ENSG00000182584	ACTL10	-0.464	-0.541	-2.189	0.033
ENSG00000182612	TSPAN10	-0.863	-1.493	-2.119	0.038
ENSG00000183248	PRR36	-0.667	2.088	-2.099	0.040
ENSG00000183729	NPBWR1	1.759	-3.808	2.289	0.026
ENSG00000183762	KREMEN1	-0.447	4.515	-3.200	0.002
ENSG00000183765	CHEK2	0.377	0.469	2.204	0.032
ENSG00000183779	ZNF703	-0.521	1.892	-2.525	0.014
ENSG00000183891	TTC32	0.328	2.572	2.266	0.027
ENSG00000184139	RPL7AP28	1.386	-2.319	2.537	0.014
ENSG00000184232	OAF	-0.527	3.424	-2.178	0.034
ENSG00000184271	POU6F1	-0.285	5.074	-2.436	0.018
ENSG00000184277	TM2D3	0.411	5.636	2.961	0.004
ENSG00000184374	COLEC10	0.591	-0.997	2.022	0.048
ENSG00000184384	MAML2	-0.251	5.297	-2.055	0.044
ENSG00000184545	DUSP8	-0.621	4.467	-3.019	0.004
ENSG00000184731	FAM110C	0.453	2.674	2.182	0.033
ENSG00000184922	FMNL1	-0.537	3.879	-3.096	0.003

ENSG00000185133	INPP5J	-0.427	2.471	-2.106	0.040
ENSG00000185236	RAB11B	-0.258	4.136	-2.212	0.031
ENSG00000185272	RBM11	0.477	2.900	2.715	0.009
ENSG00000185627	PSMD13	0.276	4.725	2.211	0.031
ENSG00000185650	ZFP36L1	-0.466	6.052	-2.515	0.015
ENSG00000185668	POU3F1	-0.859	1.415	-2.076	0.042
ENSG00000185721	DRG1	0.333	4.645	2.163	0.035
ENSG00000185800	DMWD	-0.281	4.829	-2.091	0.041
ENSG00000185818	NAT8L	-0.283	6.252	-2.611	0.012
ENSG00000185950	IRS2	-0.279	6.346	-2.115	0.039
ENSG00000186104	CYP2R1	0.214	3.315	2.049	0.045
ENSG00000186111	PIP5K1C	-0.513	5.464	-2.439	0.018
ENSG00000186231	KLHL32	0.320	5.435	2.355	0.022
ENSG00000186666	BCDIN3D	0.277	1.629	2.167	0.034
ENSG00000187068	C3orf70	-0.298	6.306	-2.350	0.022
ENSG00000187522	HSPA14	0.366	3.654	2.770	0.008
ENSG00000187536	TPM3P7	-0.438	1.160	-2.151	0.036
ENSG00000187837	HIST1H1C	-0.405	4.890	-2.653	0.010
ENSG00000187902	SHISA7	-0.529	4.699	-2.347	0.022
ENSG00000188001	TPRG1	-0.382	0.705	-2.261	0.028
ENSG00000188162	OTOG	-0.973	-2.047	-2.428	0.018
ENSG00000188215	DCUN1D3	0.186	4.271	2.020	0.048
ENSG00000188283	ZNF383	0.167	4.564	2.690	0.009
ENSG00000188295	ZNF669	0.326	2.492	2.227	0.030
ENSG00000188305	C19orf35	-1.115	-2.181	-3.170	0.002
ENSG00000188322	SBK1	-0.442	3.656	-2.109	0.039
ENSG00000188529	SRSF10	0.184	6.411	2.749	0.008
ENSG00000188735	TMEM120B	-0.378	3.547	-2.858	0.006
ENSG00000188766	SPRED3	-0.627	3.537	-2.589	0.012
ENSG00000188783	PRELP	-0.574	3.629	-2.699	0.009
ENSG00000188992	LIPI	0.661	-0.688	2.112	0.039
ENSG00000189046	ALKBH2	0.322	2.128	2.088	0.041
ENSG00000189376	C8orf76	0.327	1.326	2.808	0.007
ENSG00000189403	HMGB1	0.190	7.183	2.079	0.042
ENSG00000196072	BLOC1S2	0.352	5.359	2.546	0.014
ENSG00000196208	GREB1	-0.228	5.112	-2.159	0.035
ENSG00000196497	IPO4	-0.515	-1.430	-2.020	0.048
ENSG00000196557	CACNA1H	-0.706	4.424	-2.482	0.016
ENSG00000196576	PLXNB2	-0.279	4.691	-2.059	0.044
ENSG00000196591	HDAC2	0.223	5.745	2.047	0.045
ENSG00000196781	TLE1	-0.306	4.904	-2.435	0.018
ENSG00000196810	CTBP1-AS2	-0.229	3.701	-2.194	0.032
ENSG00000196911	KPNA5	0.198	6.465	2.037	0.046
ENSG00000196917	HCAR1	-0.715	-1.763	-2.108	0.039
ENSG00000196951	SCOC-AS1	0.363	0.535	2.043	0.046
ENSG00000196968	FUT11	0.225	4.196	2.051	0.045
ENSG00000197077	KIAA1671	-0.338	6.957	-2.151	0.036

ENSG00000197136	PCNX3	-0.276	4.257	-2.201	0.032
ENSG00000197181	PIWIL2	0.841	1.372	2.626	0.011
ENSG00000197223	C1D	0.404	3.243	2.407	0.019
ENSG00000197261	C6orf141	1.790	-2.105	2.217	0.031
ENSG00000197265	GTF2E2	0.216	3.717	2.176	0.034
ENSG00000197363	ZNF517	-0.276	2.772	-2.542	0.014
ENSG00000197461	PDGFA	0.207	4.728	2.241	0.029
ENSG00000197483	ZNF628	-0.550	-0.234	-2.214	0.031
ENSG00000197632	SERPINB2	1.168	-5.327	2.202	0.032
ENSG00000197978	GOLGA6L9	-0.443	1.854	-2.137	0.037
ENSG00000198053	SIRPA	-0.249	7.177	-2.299	0.025
ENSG00000198417	MT1F	-0.453	2.155	-2.285	0.026
ENSG00000198598	MMP17	-0.448	3.324	-2.081	0.042
ENSG00000198673	FAM19A2	0.759	5.111	2.483	0.016
ENSG00000198677	TTC37	0.245	7.354	2.069	0.043
ENSG00000198712	MT-CO2	-0.747	11.501	-2.483	0.016
ENSG00000198727	MT-CYB	-0.691	10.736	-2.257	0.028
ENSG00000198763	MT-ND2	-0.727	10.414	-2.166	0.034
ENSG00000198783	ZNF830	0.268	3.866	2.266	0.027
ENSG00000198786	MT-ND5	-0.607	10.836	-2.180	0.033
ENSG00000198804	MT-CO1	-0.717	14.378	-2.474	0.016
ENSG00000198838	RYR3	-0.361	8.164	-2.613	0.011
ENSG00000198840	MT-ND3	-0.582	9.981	-2.144	0.036
ENSG00000198886	MT-ND4	-0.687	11.557	-2.121	0.038
ENSG00000198888	MT-ND1	-0.779	10.520	-2.275	0.027
ENSG00000198899	MT-ATP6	-0.895	10.684	-2.254	0.028
ENSG00000198938	MT-CO3	-0.802	12.628	-2.414	0.019
ENSG00000199332	Y_RNA	-0.689	-1.759	-2.010	0.049
ENSG00000199390	SNORD114-7	0.836	-0.528	2.427	0.018
ENSG00000199812	RNU6-428P	-1.223	-4.763	-2.032	0.047
ENSG00000199880	RNU6-888P	-0.676	-2.306	-2.018	0.048
ENSG00000200480	SNORD114-28	1.202	-1.706	3.513	0.001
ENSG00000200610	Y_RNA	0.957	-3.064	2.155	0.035
ENSG00000200823	SNORD114-2	0.748	0.028	2.375	0.021
ENSG00000201358	RN7SKP193	1.273	-1.834	2.549	0.013
ENSG00000201499	RNU6-312P	0.701	-1.709	2.346	0.022
ENSG00000201566	Y_RNA	0.694	-0.756	2.013	0.049
ENSG00000201600	RN7SKP124	-0.486	-0.467	-2.115	0.039
ENSG00000201607	RNU4-16P	-0.692	-2.224	-2.171	0.034
ENSG00000201950	SNORD113-9	0.914	-1.528	2.405	0.019
ENSG00000201992	SNORD115-35	0.610	-0.239	2.014	0.049
ENSG00000202314	SNORD6	0.436	-0.995	2.167	0.034
ENSG00000203280	CTA-221G9.12	-0.508	-0.102	-2.131	0.037
ENSG00000203435	E2F3P2	-0.446	0.590	-2.030	0.047
ENSG00000203643	AC012456.3	-0.605	-0.415	-2.080	0.042
ENSG00000203722	RAET1G	-0.749	-1.069	-2.487	0.016
ENSG00000203761	MSTO2P	-0.370	1.822	-2.017	0.048

ENSG00000204314	PRRT1	-0.558	3.174	-2.258	0.028
ENSG00000204392	LSM2	0.317	1.365	2.282	0.026
ENSG00000204469	PRRC2A	-0.333	6.726	-2.335	0.023
ENSG00000204713	TRIM27	0.166	4.933	2.450	0.017
ENSG00000204856	FAM216A	0.299	4.063	2.177	0.034
ENSG00000204965	PCDHA5	-0.273	2.066	-2.054	0.045
ENSG00000204977	TRIM13	0.171	5.612	2.203	0.032
ENSG00000205085	FAM71F2	-0.401	3.078	-2.072	0.043
ENSG00000205181	LINC00654	-0.297	3.581	-2.035	0.046
ENSG00000205336	ADGRG1	-0.365	5.731	-2.132	0.037
ENSG00000205339	IPO7	0.276	7.575	2.186	0.033
ENSG00000205707	LYRM5	0.215	4.726	2.358	0.022
ENSG00000205758	CRYZL1	0.210	5.355	2.389	0.020
ENSG00000205771	CATSPER2P1	-0.385	1.693	-2.021	0.048
ENSG00000205809	KLRC2	-0.981	-0.333	-2.431	0.018
ENSG00000205981	DNAJC19	0.286	4.676	2.361	0.022
ENSG00000206034	DEFB109P1B	-0.593	-0.193	-2.153	0.036
ENSG00000206848	RNU6-890P	0.752	-1.866	2.562	0.013
ENSG00000207312	RNU6-429P	0.761	-2.291	2.011	0.049
ENSG00000207816	MIR124-2	0.908	-2.494	2.243	0.029
ENSG00000207954	MIR138-1	-0.684	0.518	-2.150	0.036
ENSG00000210082	MT-RNR2	-1.369	9.193	-3.861	0.000
ENSG00000210127	MT-TA	-1.071	0.788	-2.391	0.020
ENSG00000210196	MT-TP	-0.658	2.383	-2.011	0.049
ENSG00000211456	SACM1L	0.257	6.100	2.161	0.035
ENSG00000211459	MT-RNR1	-1.487	6.373	-3.421	0.001
ENSG00000211644	IGLV1-51	0.716	-2.224	2.149	0.036
ENSG00000212297	RNU6-821P	0.960	-2.959	2.123	0.038
ENSG00000212447	SNORD90	1.372	-4.190	2.785	0.007
ENSG00000213005	PTTG3P	-0.992	-0.677	-2.429	0.018
ENSG00000213109	RP11-55K22.2	-0.874	-1.985	-2.465	0.017
ENSG00000213185	FAM24B	0.382	0.151	2.408	0.019
ENSG00000213424	KRT222	0.566	3.911	2.672	0.010
ENSG00000213471	TTLL13P	-0.716	-1.725	-2.396	0.020
ENSG00000213513	RP13-39P12.2	-0.741	-1.819	-2.387	0.020
ENSG00000213551	DNAJC9	0.208	4.206	2.048	0.045
ENSG00000213741	RPS29	0.279	4.854	3.203	0.002
ENSG00000213976	CTD-2561J22.2	0.378	1.688	2.339	0.023
ENSG00000214078	CPNE1	0.225	3.743	2.717	0.009
ENSG00000214106	PAXIP1-AS2	-0.282	3.897	-2.418	0.019
ENSG00000214140	PRCD	0.603	0.984	2.961	0.004
ENSG00000214211	CTAGE14P	-0.365	1.549	-2.034	0.047
ENSG00000214290	COLCA2	-0.485	0.849	-2.330	0.023
ENSG00000214331	RP11-252A24.	-0.368	3.564	-2.648	0.010
ENSG00000214413	BBIP1	0.166	5.138	2.187	0.033
ENSG00000214688	C10orf105	-1.027	0.550	-2.209	0.031
ENSG00000214842	RAD51AP2	0.883	0.891	2.566	0.013

ENSG00000215380	AC090625.1	-0.654	2.035	-2.635	0.011
ENSG00000215790	SLC35E2	-0.339	4.860	-2.121	0.038
ENSG00000215912	TTC34	-0.479	0.638	-2.223	0.030
ENSG00000216316	RP3-354N19.3	-0.700	-0.301	-2.036	0.046
ENSG00000216687	RP11-278J20.2	-0.567	-0.667	-2.030	0.047
ENSG00000217128	FNIP1	0.191	6.923	2.367	0.021
ENSG00000218186	KRT8P43	-0.779	-0.606	-2.319	0.024
ENSG00000218416	PP14571	-0.483	1.305	-2.242	0.029
ENSG00000220008	LINGO3	-0.639	1.058	-2.496	0.015
ENSG00000221491	SNORA2C	0.929	-2.917	2.592	0.012
ENSG00000221818	EBF2	1.609	-1.870	2.631	0.011
ENSG00000221819	GAS8-AS1	-0.593	-0.453	-2.190	0.033
ENSG00000222255	RNU6-101P	0.788	-2.313	2.417	0.019
ENSG00000222317	RNA5SP118	-0.771	-1.408	-2.430	0.018
ENSG00000223427	AC016716.1	-0.332	2.059	-2.154	0.035
ENSG00000223503	RP11-29H23.6	-0.791	-1.995	-2.201	0.032
ENSG00000223558	TRIM60P17	0.674	-0.999	2.005	0.050
ENSG00000223658	C1GALT1C1L	-0.775	-0.833	-2.625	0.011
ENSG00000224023	FLJ37035	0.235	1.799	2.155	0.035
ENSG00000224083	MTCO1P11	-0.771	-0.076	-3.032	0.004
ENSG00000224247	AJ009632.3	1.560	-4.175	2.060	0.044
ENSG00000224348	RP11-306K13.	-0.632	-0.735	-2.178	0.034
ENSG00000224468	RP11-181K3.4	-0.605	-1.272	-2.194	0.032
ENSG00000224613	RP11-153F1.1	0.825	-1.240	2.640	0.011
ENSG00000224635	RP4-564F22.5	0.648	-0.555	2.032	0.047
ENSG00000224697	NEFMP1	-0.755	-0.990	-2.538	0.014
ENSG00000224707	E2F3-IT1	-0.393	-0.431	-2.077	0.042
ENSG00000224746	AC015987.1	0.773	-1.422	2.576	0.013
ENSG00000224764	RP11-54O15.3	0.709	-1.631	2.369	0.021
ENSG00000224786	CETN4P	0.448	1.342	2.118	0.038
ENSG00000224805	LINC00853	0.975	-2.968	2.082	0.042
ENSG00000225194	LINC00092	-0.510	1.280	-2.795	0.007
ENSG00000225386	AC099754.1	-0.786	-1.938	-2.154	0.035
ENSG00000225630	MTND2P28	-1.235	6.820	-2.025	0.048
ENSG00000225706	PTPRD-AS1	0.341	2.783	2.158	0.035
ENSG00000225731	AP001627.1	-0.576	0.198	-3.124	0.003
ENSG00000225793	RP1-234P15.4	0.397	0.428	2.043	0.046
ENSG00000225806	RP1-309F20.3	0.661	-1.356	2.045	0.045
ENSG00000225938	RP4-575N6.4	-0.607	-1.136	-2.180	0.033
ENSG00000225975	LINC01534	0.278	2.498	2.080	0.042
ENSG00000226083	SLC39A12-AS1	-0.675	-0.388	-2.232	0.030
ENSG00000226259	GTF2H2B	-0.943	1.604	-2.074	0.043
ENSG00000226288	OR52I2	-0.672	-1.148	-2.286	0.026
ENSG00000226624	CTA-351J1.1	-0.609	-2.008	-2.268	0.027
ENSG00000226746	SMCR5	-0.500	0.658	-2.189	0.033
ENSG00000226761	TAS2R46	0.453	0.426	2.140	0.037
ENSG00000227034	RP11-234N17.	-0.754	0.105	-2.467	0.017

ENSG00000227107	AC096574.5	-0.570	-0.685	-2.144	0.036
ENSG00000227195	MIR663AHG	-0.823	-1.278	-2.502	0.015
ENSG00000227288	RP5-837I24.1	-0.624	-0.066	-2.105	0.040
ENSG00000227470	AC073415.2	0.611	-0.568	2.356	0.022
ENSG00000227544	AC018647.3	0.564	3.568	2.135	0.037
ENSG00000227627	RP1-101K10.6	0.456	-0.789	2.022	0.048
ENSG00000228013	RP11-350G8.5	-0.730	-1.434	-2.610	0.012
ENSG00000228126	FALEC	-0.606	-0.712	-2.308	0.025
ENSG00000228252	COL6A4P2	-0.552	0.593	-2.319	0.024
ENSG00000228358	LINC02263	1.853	-2.955	2.505	0.015
ENSG00000228434	AC004951.6	-0.562	0.279	-2.274	0.027
ENSG00000228594	FNDC10	-0.540	1.204	-2.901	0.005
ENSG00000228606	RP11-574F21.[illegible]	0.376	0.036	2.210	0.031
ENSG00000229348	HYI-AS1	-0.773	-1.855	-2.685	0.009
ENSG00000229520	LINC00404	-0.568	-1.123	-2.131	0.037
ENSG00000229690	MTCO2P1	-0.960	-2.690	-2.165	0.035
ENSG00000229739	RP11-295K2.3	0.304	2.130	2.546	0.014
ENSG00000229847	EMX2OS	-0.382	6.452	-2.179	0.033
ENSG00000230042	AK3P3	-0.330	1.053	-2.385	0.020
ENSG00000230133	LINC01721	1.337	-1.178	2.484	0.016
ENSG00000230237	AC010975.2	-0.495	-0.491	-2.028	0.047
ENSG00000230470	GS1-115G20.1	-0.531	0.147	-2.785	0.007
ENSG00000230487	PSMG3-AS1	-0.373	3.069	-2.921	0.005
ENSG00000230555	RP11-517P14.[illegible]	-0.555	-0.634	-2.296	0.025
ENSG00000230731	RP11-478K15.[illegible]	-1.126	-1.784	-2.665	0.010
ENSG00000230733	AC092171.4	-0.425	3.741	-2.408	0.019
ENSG00000230741	SC22CB-1D7.1	-0.753	-1.324	-2.070	0.043
ENSG00000230805	AL132709.1	-1.021	-2.349	-2.083	0.042
ENSG00000230839	RP5-968J1.1	-0.450	1.147	-2.656	0.010
ENSG00000231064	MIR92B	-0.459	1.550	-2.654	0.010
ENSG00000231113	RP3-475N16.1	-0.325	1.688	-2.018	0.048
ENSG00000231419	LINC00689	-0.939	-0.426	-2.790	0.007
ENSG00000231546	RPL3P5	-0.665	-0.120	-2.096	0.040
ENSG00000231615	RP11-296O14.	-0.318	2.868	-2.160	0.035
ENSG00000231713	AF064860.7	-0.825	-1.192	-2.243	0.029
ENSG00000231795	ITCH-IT1	0.598	-1.216	2.041	0.046
ENSG00000231804	RP11-317B17.	0.935	-2.197	2.320	0.024
ENSG00000231806	PCAT7	-1.828	-1.820	-2.739	0.008
ENSG00000231940	RPS7P3	-0.595	-1.593	-2.005	0.050
ENSG00000232085	RP11-261C10.[illegible]	0.454	-0.602	2.019	0.048
ENSG00000232117	LINC00384	-0.595	-0.597	-2.071	0.043
ENSG00000232200	RP4-564O4.1	1.026	-5.443	2.006	0.050
ENSG00000232273	FTH1P1	-0.647	-1.735	-2.049	0.045
ENSG00000232434	C9orf172	-0.713	2.831	-2.945	0.005
ENSG00000232528	RP4-673D20.3	-0.592	2.585	-2.885	0.005
ENSG00000232568	RPL23AP35	-0.598	-0.511	-2.730	0.008
ENSG00000232913	PLCE1-AS2	-0.701	-0.277	-2.680	0.010

ENSG00000233038	AC011899.9	-0.809	0.268	-2.577	0.013
ENSG00000233157	RP3-388M5.8	-0.814	-0.578	-2.025	0.048
ENSG00000233175	CTD-2020K17.	-0.586	0.262	-2.212	0.031
ENSG00000233216	RP11-414C16.	-0.711	-2.102	-2.210	0.031
ENSG00000233220	LINC00167	-0.758	-1.704	-2.329	0.023
ENSG00000233293	RP11-410N8.3	-0.757	-1.660	-2.158	0.035
ENSG00000233517	AC005162.5	-0.625	0.382	-2.163	0.035
ENSG00000233560	KRT8P39	-0.414	0.358	-2.092	0.041
ENSG00000233725	LINC00284	0.631	-0.048	2.495	0.015
ENSG00000233828	LINC01949	0.605	-1.509	2.057	0.044
ENSG00000234377	RNF219-AS1	-0.316	4.598	-2.250	0.028
ENSG00000234409	CCDC188	-0.582	-1.172	-2.073	0.043
ENSG00000234478	RP11-275I14.4	0.612	-1.110	2.575	0.013
ENSG00000234534	CSNK1G2P1	-0.640	-0.737	-2.623	0.011
ENSG00000234758	AC034228.4	-0.533	-1.023	-2.324	0.024
ENSG00000234860	RP11-547C13.	-0.545	-1.622	-2.014	0.049
ENSG00000234917	RP5-994D16.3	0.673	-0.726	3.631	0.001
ENSG00000235079	ZRANB2-AS1	0.556	-1.158	2.281	0.026
ENSG00000235290	HLA-W	-0.542	-1.501	-2.010	0.049
ENSG00000235389	RP11-134K1.3	0.538	-0.025	2.276	0.027
ENSG00000235413	KRT18P63	0.568	-1.905	2.066	0.043
ENSG00000235547	NDUFB11P1	-0.608	-1.227	-2.189	0.033
ENSG00000235605	RP5-827C21.1	-0.665	0.325	-2.133	0.037
ENSG00000235641	LINC00484	0.618	-0.331	2.207	0.031
ENSG00000236254	MTND4P14	-0.698	-1.007	-2.173	0.034
ENSG00000236274	RP4-728D4.3	-0.438	-0.111	-2.143	0.036
ENSG00000236383	LINC00854	-0.500	0.510	-2.031	0.047
ENSG00000236501	AC079305.11	0.486	-0.900	2.108	0.039
ENSG00000236700	LINC01010	0.858	0.010	3.018	0.004
ENSG00000236740	RP11-411K7.1	1.656	-4.214	2.699	0.009
ENSG00000236796	RP11-462B18.	0.631	-1.212	2.221	0.030
ENSG00000236797	SPA17P1	-1.038	-1.120	-2.490	0.016
ENSG00000236816	ANKRD20A7P	-0.639	3.799	-2.692	0.009
ENSG00000236830	CBR3-AS1	-0.281	1.604	-2.059	0.044
ENSG00000237194	SNAI1P1	-0.858	-1.748	-2.383	0.020
ENSG00000237418	AC069257.6	0.556	-1.120	2.018	0.048
ENSG00000237667	LINC01115	2.005	-0.077	2.700	0.009
ENSG00000237705	AC008937.2	-0.486	-0.226	-2.399	0.020
ENSG00000237773	AC003075.4	0.522	0.246	2.461	0.017
ENSG00000238221	RP11-69L16.4	0.903	-2.544	2.740	0.008
ENSG00000238243	OR2W3	1.026	-0.672	2.680	0.010
ENSG00000238276	RP11-245J24.1	-0.571	0.657	-2.316	0.024
ENSG00000239672	NME1	0.334	3.710	2.014	0.049
ENSG00000239857	GET4	-0.418	-1.215	-2.179	0.033
ENSG00000239932	RN7SL606P	1.350	-3.072	2.643	0.011
ENSG00000240097	RP11-641C17.	0.564	-1.385	2.251	0.028
ENSG00000240184	PCDHGC3	-0.326	6.443	-2.963	0.004

ENSG00000240344	PPIL3	0.293	3.710	2.466	0.017
ENSG00000240393	RP11-768G7.1	-0.368	3.571	-2.152	0.036
ENSG00000240710	RP11-430C7.4	-0.558	1.070	-2.175	0.034
ENSG00000240775	RP11-170N16.	-0.704	-1.018	-2.658	0.010
ENSG00000240857	RDH14	0.409	3.700	2.540	0.014
ENSG00000241002	RP11-119H12.	-0.602	-0.525	-2.019	0.048
ENSG00000241163	LINC00877	0.522	1.442	2.124	0.038
ENSG00000241345	RP4-630C24.3	1.063	0.884	2.591	0.012
ENSG00000242121	RN7SL267P	0.821	-1.692	2.191	0.033
ENSG00000242715	CCDC169	0.602	2.154	2.108	0.039
ENSG00000242779	ZNF702P	0.751	2.135	2.602	0.012
ENSG00000242889	RN7SL449P	-0.477	-0.257	-2.145	0.036
ENSG00000242931	RPL7P49	-0.524	0.497	-2.036	0.046
ENSG00000243280	RP11-477G18.	0.651	-1.171	2.131	0.037
ENSG00000243323	PTPRVP	-0.582	-0.600	-2.075	0.042
ENSG00000243398	RN7SL141P	-0.866	-2.311	-2.233	0.029
ENSG00000243406	MRPS31P5	0.428	1.619	2.446	0.018
ENSG00000243444	PALM2	0.244	4.850	2.127	0.038
ENSG00000244061	RP11-389C8.1	1.097	-2.319	2.535	0.014
ENSG00000244383	FAM3D-AS1	-0.754	-1.176	-2.812	0.007
ENSG00000244480	AC005154.7	-0.358	1.141	-2.072	0.043
ENSG00000245667	RP5-940J5.8	-0.740	-1.613	-2.617	0.011
ENSG00000245688	CTB-26E19.1	-1.126	-3.261	-2.037	0.046
ENSG00000245750	DRAIC	-0.283	2.427	-2.129	0.038
ENSG00000245970	KB-1208A12.3	0.467	0.615	2.362	0.022
ENSG00000246100	LINC00900	-0.404	1.789	-3.130	0.003
ENSG00000246203	RP11-29H23.5	-0.527	0.119	-2.199	0.032
ENSG00000247345	RP11-266N13.	-0.563	-0.719	-2.147	0.036
ENSG00000247903	RP11-421F16.3	-0.198	3.645	-2.135	0.037
ENSG00000247934	RP11-967K21.	-0.444	0.948	-2.600	0.012
ENSG00000248049	UBA6-AS1	-0.254	4.449	-2.214	0.031
ENSG00000248249	RP11-598O12.	-0.490	0.965	-2.121	0.038
ENSG00000248429	RP11-597D13.	-0.568	2.653	-2.539	0.014
ENSG00000248449	PCDHGB8P	-0.408	1.943	-2.586	0.012
ENSG00000248527	MTATP6P1	-0.898	8.141	-2.168	0.034
ENSG00000248874	C5orf17	-1.551	-1.956	-2.319	0.024
ENSG00000248896	CTD-2135J3.3	-0.894	-1.666	-2.691	0.009
ENSG00000249114	RP11-5N11.3	-0.715	-2.209	-2.032	0.047
ENSG00000249352	LINC02198	-0.836	-1.488	-2.456	0.017
ENSG00000249381	LINC00500	-1.135	-1.735	-2.209	0.031
ENSG00000249476	CTD-2587M2.1	0.566	0.476	2.057	0.044
ENSG00000249502	AC006160.5	0.486	-0.260	2.205	0.031
ENSG00000249513	RP11-404I7.1	0.691	-1.361	2.870	0.006
ENSG00000249514	CTD-2266L18.1	-0.859	-1.405	-2.250	0.028
ENSG00000249661	TNRC18P1	-0.438	1.156	-2.311	0.024
ENSG00000249673	NOP14-AS1	-0.208	3.665	-2.209	0.031
ENSG00000249846	LINC02021	-0.547	0.451	-2.766	0.008

ENSG00000250039	RP11-17E2.2	-0.886	-1.817	-2.155	0.035
ENSG00000250091	DNAH10OS	-0.444	3.616	-2.698	0.009
ENSG00000250155	CTD-2353F22.	-0.441	2.339	-2.274	0.027
ENSG00000250254	PTTG2	-0.591	-0.038	-2.119	0.038
ENSG00000250267	RP11-122A21.	0.536	-0.636	2.676	0.010
ENSG00000250303	RP11-356J5.12	0.283	2.521	2.455	0.017
ENSG00000250397	RP11-1391J7.1	-0.665	-0.616	-2.082	0.042
ENSG00000250461	RP11-631M6.2	-0.423	3.980	-2.382	0.021
ENSG00000250514	LINC02125	0.756	-0.760	2.447	0.017
ENSG00000250602	RP11-517I3.1	-0.442	1.551	-2.207	0.031
ENSG00000250761	RP11-711G10.	-0.460	0.279	-2.074	0.043
ENSG00000250988	SNHG21	0.541	-0.633	2.186	0.033
ENSG00000251034	RP11-582J16.4	-0.388	3.294	-2.252	0.028
ENSG00000251322	SHANK3	-0.642	5.945	-2.868	0.006
ENSG00000251372	LINC00499	-1.041	1.631	-2.760	0.008
ENSG00000251408	RP11-586D19.	-0.519	0.583	-2.158	0.035
ENSG00000251421	CTC-369A16.2	0.840	-0.846	2.025	0.048
ENSG00000251441	RTEL1P1	-0.605	-0.229	-2.048	0.045
ENSG00000251484	RP5-1065J22.4	-0.539	-1.255	-2.090	0.041
ENSG00000251596	HADHAP1	-0.506	3.601	-3.034	0.004
ENSG00000251609	SETP12	-0.890	-1.908	-2.508	0.015
ENSG00000251675	CTC-458I2.2	0.631	-1.849	2.015	0.049
ENSG00000252892	RNU6-548P	-0.482	-1.075	-2.099	0.040
ENSG00000252985	SNORD116	0.628	-0.799	2.128	0.038
ENSG00000253086	RNU6-537P	-1.153	-4.892	-2.405	0.019
ENSG00000253121	RP11-659E9.4	0.993	-2.405	2.230	0.030
ENSG00000253159	PCDHGA12	-0.320	3.750	-2.195	0.032
ENSG00000253194	RP11-351A11.	-0.543	1.669	-2.141	0.036
ENSG00000253485	PCDHGA5	-0.262	2.760	-2.147	0.036
ENSG00000253523	RP11-1112C15	-0.622	-1.027	-2.086	0.041
ENSG00000253764	RP11-439C15.4	-0.540	1.873	-2.265	0.027
ENSG00000253767	PCDHGA8	-0.314	2.862	-2.032	0.047
ENSG00000253846	PCDHGA10	-0.275	4.131	-2.897	0.005
ENSG00000253873	PCDHGA11	-0.311	3.410	-2.507	0.015
ENSG00000253910	PCDHGB2	-0.354	3.537	-2.942	0.005
ENSG00000254027	RP11-363E6.3	-0.636	-1.340	-2.011	0.049
ENSG00000254122	PCDHGB7	-0.269	4.087	-2.229	0.030
ENSG00000254221	PCDHGB1	-0.338	2.532	-2.193	0.032
ENSG00000254343	RP11-760H22.	0.386	1.792	2.234	0.029
ENSG00000254865	RP11-748C4.1	-0.408	0.719	-2.342	0.023
ENSG00000254921	RP11-236J17.6	-0.460	-0.438	-2.018	0.048
ENSG00000255158	RP11-754B17.	-0.693	-0.183	-2.356	0.022
ENSG00000255267	RP11-430H10.	-0.632	-1.706	-2.108	0.039
ENSG00000255330	RP3-403A15.5	0.393	2.017	2.407	0.019
ENSG00000255346	NOX5	-0.697	0.643	-2.086	0.041
ENSG00000255445	RP11-573M3.3	0.488	-0.133	2.373	0.021
ENSG00000255520	RP11-390K5.3	-1.036	-2.114	-2.655	0.010

ENSG00000255545	RP11-627G23.	-0.512	2.201	-2.349	0.022
ENSG00000255622	PCDHB17P	-0.314	1.994	-2.085	0.041
ENSG00000255717	SNHG1	0.251	3.183	2.056	0.044
ENSG00000255823	MTRNR2L8	-1.629	0.049	-3.884	0.000
ENSG00000256070	RP11-56H16.1	1.586	-2.664	2.910	0.005
ENSG00000256282	RP11-504G3.4	-0.558	-0.175	-2.315	0.024
ENSG00000256473	RP11-437F6.1	-0.940	-0.977	-2.180	0.033
ENSG00000256616	RP11-815J4.6	-0.624	2.532	-2.103	0.040
ENSG00000256618	MTRNR2L1	-1.340	-2.801	-2.320	0.024
ENSG00000256694	RP11-598F7.5	-0.596	-1.089	-2.208	0.031
ENSG00000256802	RP11-680F8.1	-0.560	0.208	-2.034	0.047
ENSG00000256817	TPT1P12	0.665	-1.884	2.478	0.016
ENSG00000257097	CLIP1-AS1	-0.433	1.012	-2.521	0.014
ENSG00000257139	RP11-320P7.2	0.698	-0.569	2.089	0.041
ENSG00000257303	RP11-977G19.	-0.520	-0.894	-2.068	0.043
ENSG00000258135	LINC02396	-0.705	-1.750	-2.133	0.037
ENSG00000258220	LINC02424	1.710	-3.910	2.720	0.009
ENSG00000258289	CHURC1	0.239	6.344	2.065	0.043
ENSG00000258344	RP11-968A15.	-0.853	-1.323	-2.970	0.004
ENSG00000258909	RP11-164C12.	-0.654	-1.386	-2.045	0.045
ENSG00000259228	HNRNPA1P62	0.597	-0.406	2.062	0.044
ENSG00000259411	HNRNPA1P45	-0.924	-1.668	-2.635	0.011
ENSG00000259429	UBE2Q2P2	-0.351	2.463	-2.003	0.050
ENSG00000259575	RP11-39M21.2	-0.438	0.670	-2.150	0.036
ENSG00000259628	RP11-467H10.	-0.747	-1.232	-2.613	0.011
ENSG00000259805	RP11-382A20.	-0.902	-1.441	-2.328	0.023
ENSG00000259926	RP11-44F14.5	0.874	-1.382	2.446	0.018
ENSG00000260086	RP11-42I10.1	-0.652	-1.679	-2.122	0.038
ENSG00000260095	RP11-715J22.3	-0.719	-2.008	-2.112	0.039
ENSG00000260271	RP1-45N11.1	-0.671	-1.859	-2.164	0.035
ENSG00000260293	RP11-715J22.6	-0.453	2.155	-2.635	0.011
ENSG00000260300	RP11-505K9.4	-0.653	-0.876	-2.049	0.045
ENSG00000260317	RP11-48B3.4	0.638	0.401	3.450	0.001
ENSG00000260385	RP11-450H6.3	-0.624	-0.727	-2.126	0.038
ENSG00000260409	RP11-403B2.7	0.878	0.349	2.388	0.020
ENSG00000260492	CISTR	-0.487	-1.012	-2.024	0.048
ENSG00000260526	RP11-73K9.2	0.403	0.891	2.297	0.025
ENSG00000260541	LA16c-429E7.1	-0.814	-2.136	-2.660	0.010
ENSG00000260616	RP11-327F22.4	-0.496	-1.319	-2.210	0.031
ENSG00000260743	RP11-255C15.	0.597	0.012	2.466	0.017
ENSG00000260891	RP11-96D1.5	0.559	-0.458	2.759	0.008
ENSG00000260971	RP11-504A18.	0.663	-0.169	2.341	0.023
ENSG00000261007	RP11-382N13.	-0.702	1.035	-2.008	0.049
ENSG00000261221	ZNF865	-0.434	1.785	-2.911	0.005
ENSG00000261324	RP11-174G6.5	0.233	2.548	2.199	0.032
ENSG00000261366	MANEA-AS1	0.360	1.205	2.700	0.009
ENSG00000261542	RP11-16E18.3	0.458	1.286	2.085	0.042

ENSG00000261678	SCRT1	-0.465	3.101	-2.100	0.040
ENSG00000261824	LINC00662	0.165	4.574	2.267	0.027
ENSG00000262209	PCDHGB3	-0.266	2.598	-2.047	0.045
ENSG00000262445	CTD-2545H1.2	-0.920	-1.070	-2.533	0.014
ENSG00000263006	ROCK1P1	-0.546	2.473	-2.310	0.025
ENSG00000263264	CTB-133G6.1	-0.586	-1.503	-2.045	0.045
ENSG00000263382	RP11-552O4.1	-0.881	-2.435	-2.382	0.021
ENSG00000263843	RP11-649A18.	-0.512	-0.949	-2.360	0.022
ENSG00000263874	LINC00672	0.436	4.388	2.668	0.010
ENSG00000264116	RP11-321M21	0.989	-2.057	2.136	0.037
ENSG00000265100	RP11-147L13.2	-0.425	0.861	-2.052	0.045
ENSG00000265123	RN7SL200P	0.766	-1.366	2.852	0.006
ENSG00000265279	CLUHP6	-0.879	-1.850	-2.687	0.009
ENSG00000265643	RP11-465I4.2	-0.643	0.015	-2.092	0.041
ENSG00000265681	RPL17	0.312	1.174	2.710	0.009
ENSG00000266100	RP11-118E18.1	-0.689	-1.586	-2.264	0.027
ENSG00000266111	RP11-296K13.4	-0.370	0.306	-2.097	0.040
ENSG00000266306	CTD-2008P7.6	-0.819	-1.829	-2.488	0.016
ENSG00000266456	RP11-806L2.2	0.603	1.795	2.380	0.021
ENSG00000266598	RP11-583F2.5	-0.586	0.120	-2.343	0.023
ENSG00000267056	AC005336.4	-0.518	1.273	-2.577	0.013
ENSG00000267058	RP11-15A1.3	0.295	0.933	2.105	0.040
ENSG00000267091	CTBP2P7	-1.060	-1.913	-2.623	0.011
ENSG00000267136	RP11-53B2.3	-0.329	3.280	-2.267	0.027
ENSG00000267191	RP11-15A1.2	0.377	-0.129	2.056	0.044
ENSG00000267202	RP11-797E24.1	-1.094	-2.280	-2.519	0.015
ENSG00000267265	CTC-550B14.7	-0.523	-0.882	-2.098	0.040
ENSG00000267321	LINC02001	0.307	1.130	2.417	0.019
ENSG00000267364	RP11-47L3.1	1.612	-4.035	2.455	0.017
ENSG00000267372	AC005330.2	-0.581	1.002	-2.310	0.024
ENSG00000267457	RP5-837J1.4	0.593	0.284	2.030	0.047
ENSG00000267543	RP11-666A8.7	0.661	-1.588	2.237	0.029
ENSG00000267666	AC004156.3	-0.925	-1.906	-2.507	0.015
ENSG00000267680	ZNF224	0.221	3.794	2.304	0.025
ENSG00000267766	RP11-299P2.1	-0.941	-0.877	-3.585	0.001
ENSG00000267778	AC004221.2	-0.526	-1.024	-2.211	0.031
ENSG00000267799	MAN1A2P1	0.833	-0.536	2.528	0.014
ENSG00000267939	CTD-2325M2.1	-0.492	-1.456	-2.184	0.033
ENSG00000268027	AC006129.2	0.882	-1.195	2.172	0.034
ENSG00000268170	RP11-556I13.2	0.682	0.138	2.053	0.045
ENSG00000268182	SMIM17	0.595	2.824	2.487	0.016
ENSG00000268659	RP11-310J24.3	-0.642	-1.088	-2.201	0.032
ENSG00000268858	RP4-591C20.9	-0.293	1.350	-2.091	0.041
ENSG00000268873	RP11-138H11.	-0.527	-0.017	-2.679	0.010
ENSG00000268996	MAN1B1-AS1	-0.389	0.261	-2.310	0.025
ENSG00000269028	MTRNR2L12	-2.259	0.840	-3.722	0.000
ENSG00000269069	CTC-471F3.5	-0.288	3.698	-2.022	0.048

ENSG00000269189	RP11-573N10.	-0.698	-0.167	-2.636	0.011
ENSG00000269356	RP11-120K24.	-0.507	-0.198	-2.378	0.021
ENSG00000269514	RP11-370I10.1	-0.475	2.309	-2.573	0.013
ENSG00000269570	CMB9-55A18.	0.454	0.814	2.697	0.009
ENSG00000269964	MEI4	-0.527	0.821	-2.174	0.034
ENSG00000270059	RP11-88H12.2	0.943	-0.898	2.522	0.014
ENSG00000270071	AP001172.2	-0.388	2.007	-2.190	0.033
ENSG00000270133	CTC-303L1.2	0.634	-0.722	2.230	0.030
ENSG00000270528	RP11-674I16.2	-0.615	-1.277	-2.340	0.023
ENSG00000270681	RP11-372K14.	0.367	1.492	2.091	0.041
ENSG00000270739	RP11-608O8.2	0.485	0.089	2.092	0.041
ENSG00000270959	LPP-AS2	-0.517	1.251	-2.723	0.009
ENSG00000271133	CTA-293F17.1	0.970	-1.903	2.299	0.025
ENSG00000271259	RP11-1109F11	-0.451	-0.675	-2.040	0.046
ENSG00000271386	SRGNP1	-0.702	-1.356	-2.061	0.044
ENSG00000271387	RP11-382D12.	-0.886	-2.279	-2.078	0.042
ENSG00000271427	RP11-188D8.1	-0.597	-1.592	-2.064	0.044
ENSG00000271849	CTC-332L22.1	0.775	-1.637	2.379	0.021
ENSG00000271870	RP11-97C16.1	0.397	0.926	2.075	0.043
ENSG00000272109	CTD-2260A17.	0.696	-1.633	2.037	0.046
ENSG00000272156	RP11-477N3.1	0.447	-0.715	2.332	0.023
ENSG00000272182	RP11-802O23.	0.989	-0.968	2.567	0.013
ENSG00000272254	RP11-531A24.	0.734	-1.251	2.577	0.013
ENSG00000272301	RP11-111M22	0.448	0.869	2.084	0.042
ENSG00000272343	RP11-140I16.3	0.447	-0.003	2.239	0.029
ENSG00000272425	RP11-363E6.4	-0.393	1.067	-2.495	0.015
ENSG00000272432	RP3-465N24.6	1.384	-2.587	3.306	0.002
ENSG00000272515	RP11-29P20.1	0.636	0.512	2.053	0.045
ENSG00000272562	RP11-396C23.	0.772	-1.260	2.761	0.008
ENSG00000272597	RP11-446H18.	0.567	-0.007	2.623	0.011
ENSG00000272636	DOC2B	-0.508	5.818	-2.248	0.028
ENSG00000272848	RP1-137D17.2	-0.822	0.200	-2.777	0.007
ENSG00000272862	RP11-814H16.	1.218	-2.381	2.712	0.009
ENSG00000272910	RP11-15L13.4	0.363	-0.083	2.163	0.035
ENSG00000272918	CTB-152G17.6	0.211	4.036	2.130	0.037
ENSG00000273058	RP11-385F5.5	-0.523	-1.301	-2.151	0.036
ENSG00000273419	RP4-751H13.7	-0.522	-0.612	-2.423	0.019
ENSG00000273473	LL09NC01-139	-0.992	-2.143	-2.832	0.006
ENSG00000273557	CTD-2024I7.1	-0.525	-0.488	-2.312	0.024
ENSG00000273855	RP11-133K1.1	-0.616	-1.666	-2.093	0.041
ENSG00000273899	NOL12	-0.308	1.179	-2.022	0.048
ENSG00000274001	RP11-5G9.5	0.604	-0.620	2.176	0.034
ENSG00000274093	RP11-140H17.	0.370	0.589	2.291	0.026
ENSG00000274159	AL133243.3	0.853	-2.495	2.400	0.020
ENSG00000274253	RP11-566K19.	0.399	3.258	2.521	0.014
ENSG00000274400	RP11-294N21.	0.655	-0.915	2.609	0.012
ENSG00000274554	RP11-240G22.	-0.389	1.191	-2.228	0.030

ENSG00000274712	RP11-147L13.1	-0.268	3.327	-2.352	0.022
ENSG00000274810	NPHP3-ACAD1	0.586	-0.984	2.481	0.016
ENSG00000274943	RP11-351C21.	0.812	-1.273	2.935	0.005
ENSG00000274997	HIST1H2AH	1.132	-2.221	2.086	0.041
ENSG00000275144	SNX18P5	-0.797	-0.250	-2.135	0.037
ENSG00000275457	RP1-198K11.5	0.795	-2.046	2.023	0.048
ENSG00000275678	RP4-547N15.3	-0.837	-0.973	-2.059	0.044
ENSG00000275833	RP11-479O9.3	-0.538	-1.746	-2.084	0.042
ENSG00000275835	TUBGCP5	0.243	5.322	2.202	0.032
ENSG00000275963	RP11-180M15	-0.377	1.057	-2.295	0.025
ENSG00000276282	RP11-210K20.	0.531	-0.081	2.550	0.013
ENSG00000276488	CTD-2537I9.1	-0.752	-1.092	-2.076	0.042
ENSG00000276649	RP5-858L17.1	-0.262	3.056	-2.059	0.044
ENSG00000276653	RN7SL856P	-0.713	-1.133	-2.623	0.011
ENSG00000276754	RP11-461A8.5	-0.708	-1.996	-2.064	0.044
ENSG00000277200	RP11-74E22.8	-0.411	2.392	-2.394	0.020
ENSG00000278053	DDX52	0.180	5.007	2.149	0.036
ENSG00000278514	AC068831.16	1.325	-3.343	2.526	0.014
ENSG00000278588	HIST1H2BI	1.171	-2.872	2.122	0.038
ENSG00000278709	NKILA	0.735	-1.157	2.092	0.041
ENSG00000278922	AC002310.14	-0.583	-1.089	-2.321	0.024
ENSG00000279006	CTA-243E7.2	-0.744	0.737	-2.783	0.007
ENSG00000279080	CTA-228A9.4	-0.394	0.813	-2.040	0.046
ENSG00000279092	RP11-1H8.5	-0.641	0.810	-2.080	0.042
ENSG00000279110	CTA-243E7.1	-0.601	3.248	-2.455	0.017
ENSG00000279117	CTD-2562J17.	-0.316	5.538	-2.421	0.019
ENSG00000279144	RP11-894J14.2	-0.993	-2.535	-2.343	0.023
ENSG00000279197	CTB-3M24.1	-1.319	-3.869	-2.127	0.038
ENSG00000279275	RP11-756J15.2	-0.416	0.370	-2.296	0.025
ENSG00000279462	RP11-604N13.	-0.757	-0.151	-2.665	0.010
ENSG00000279469	RP11-215P8.2	0.759	-1.083	2.594	0.012
ENSG00000279575	RP11-271F18.3	1.241	-2.106	2.660	0.010
ENSG00000279678	RP11-797E24.1	-0.730	-1.702	-2.244	0.029
ENSG00000279696	RP11-178H8.7	0.376	2.040	2.635	0.011
ENSG00000279748	CTD-3222D19.	-0.643	-1.247	-2.525	0.014
ENSG00000279770	LINC00552	-1.005	-0.364	-2.512	0.015
ENSG00000279794	CTD-2621I17.3	-0.552	-0.816	-2.331	0.023
ENSG00000279819	RP11-142L16.2	0.588	2.178	2.115	0.039
ENSG00000279829	RP11-63I8.1	-0.653	-0.602	-2.683	0.010
ENSG00000279834	RP11-775C24.	0.557	0.543	2.418	0.019
ENSG00000279856	CTD-2300H10.	1.621	-4.404	2.697	0.009
ENSG00000279879	RP11-1072C15	-0.445	0.234	-2.290	0.026
ENSG00000279977	CTD-3222D19.	-0.502	0.115	-2.233	0.029
ENSG00000279993	RP11-76C10.3	1.146	-2.311	2.050	0.045
ENSG00000280076	RP11-326A13.	-0.873	-1.412	-2.073	0.043
ENSG00000280120	RP11-546D6.3	0.335	1.520	2.326	0.024
ENSG00000280197	C18orf12	-1.144	-3.047	-2.425	0.018

ENSG00000280206	CTB-193M12.5	0.653	0.600	2.397	0.020
ENSG00000280222	RP11-174G17.	-0.657	2.204	-2.492	0.016
ENSG00000280284	RP11-154F14.3	1.260	-2.728	2.353	0.022
ENSG00000280285	RP11-630A13.	-0.615	1.224	-2.017	0.048
ENSG00000280303	ERICD	-0.542	0.045	-2.858	0.006
ENSG00000280385	AP000648.5	0.286	5.248	2.353	0.022
ENSG00000280485	RP11-437J2.4	-0.425	0.550	-2.212	0.031
ENSG00000280660	RP11-437J2.3	-0.371	5.023	-2.346	0.022
ENSG00000280850	RP4-655C5.9	-0.659	-1.022	-2.367	0.021
ENSG00000281181	CH507-513H4.	-2.647	2.805	-3.137	0.003
ENSG00000281383	CH507-513H4.	-2.665	2.340	-3.166	0.002
ENSG00000281912	LINC01144	-0.632	-1.873	-2.019	0.048
ENSG00000282961	PRNCR1	-0.572	0.565	-2.018	0.048
ENSG00000283529	RP4-791K14.2	-0.500	0.976	-2.076	0.042
ENSG00000283891	MIR628	0.840	-1.698	2.106	0.040
ENSG00000284968	#N/A	-0.270	1.767	-2.046	0.045
ENSG00000285269	#N/A	0.650	-0.286	2.145	0.036
ENSG00000285287	#N/A	-0.689	-1.044	-2.415	0.019
ENSG00000285596	#N/A	-0.560	0.724	-2.332	0.023
ENSG00000285618	#N/A	-0.706	0.005	-2.390	0.020
ENSG00000285653	#N/A	-0.572	-1.062	-2.166	0.034
ENSG00000285691	#N/A	-0.999	-1.510	-2.530	0.014
ENSG00000285695	#N/A	1.032	-1.733	2.448	0.017
ENSG00000285821	#N/A	-0.932	-0.547	-2.274	0.027
ENSG00000285847	#N/A	-0.507	0.888	-2.473	0.016

Supplementary Table 7_Gene Ontology Results of DEGs across brain regions in men

AntIns

GO.ID	Term	FET	BH	Category
GO:0007275	multicellular organism development	9.6E-10	1.50307E-05	BP
GO:0048856	anatomical structure development	4.9E-09	3.83597E-05	BP
GO:0032502	developmental process	0.000000012	5.32338E-05	BP
GO:0072359	circulatory system development	0.000000014	5.32338E-05	BP
GO:0032501	multicellular organismal process	0.000000017	5.32338E-05	BP
GO:0001944	vasculature development	0.000000024	6.08883E-05	BP
GO:0007155	cell adhesion	0.00000003	6.08883E-05	BP
GO:0022610	biological adhesion	0.000000035	6.08883E-05	BP
GO:0001568	blood vessel development	0.000000035	6.08883E-05	BP
GO:0072358	cardiovascular system development	0.000000039	6.10623E-05	BP
GO:0048731	system development	0.000000059	8.39785E-05	BP
GO:0035295	tube development	0.000000083	0.000108294	BP
GO:0030198	extracellular matrix organization	0.0000003	0.000361315	BP
GO:0030154	cell differentiation	0.00000052	0.00057409	BP
GO:0043062	extracellular structure organization	0.00000055	0.00057409	BP
GO:0048513	animal organ development	0.00000074	0.000724136	BP
GO:0048514	blood vessel morphogenesis	0.000001	0.000921	BP
GO:0048869	cellular developmental process	0.0000017	0.001478717	BP
GO:0035239	tube morphogenesis	0.000002	0.001648105	BP
GO:0045595	regulation of cell differentiation	0.0000036	0.00281826	BP
GO:0009653	anatomical structure morphogenesis	0.0000057	0.004249757	BP
GO:0045995	regulation of embryonic development	0.0000071	0.004901322	BP
GO:0009611	response to wounding	0.0000072	0.004901322	BP
GO:0048646	anatomical structure formation involved ...	0.000011	0.007176125	BP
GO:0045944	positive regulation of transcription by ...	0.000013	0.008118444	BP
GO:0009792	embryo development ending in birth or eg...	0.000014	0.008118444	BP
GO:0010470	regulation of gastrulation	0.000014	0.008118444	BP
GO:0002062	chondrocyte differentiation	0.000015	0.008387679	BP
GO:0043009	chordate embryonic development	0.000017	0.009178241	BP
GO:0009888	tissue development	0.000018	0.0093942	BP
GO:0045597	positive regulation of cell differentiat...	0.000022	0.010438	BP
GO:0030155	regulation of cell adhesion	0.000022	0.010438	BP
GO:0009887	animal organ morphogenesis	0.000022	0.010438	BP
GO:0050793	regulation of developmental process	0.00003	0.013815	BP
GO:0022008	neurogenesis	0.00004	0.017893714	BP
GO:0030182	neuron differentiation	0.000042	0.0182665	BP
GO:0044706	multi-multicellular organism process	0.000044	0.018619135	BP
GO:0098609	cell-cell adhesion	0.00005	0.020601316	BP
GO:0007399	nervous system development	0.000053	0.021277462	BP
GO:0009790	embryo development	0.000059	0.022530805	BP
GO:0009605	response to external stimulus	0.000059	0.022530805	BP
GO:0007166	cell surface receptor signaling pathway	0.000071	0.025620545	BP
GO:1904705	regulation of vascular smooth muscle cel...	0.000072	0.025620545	BP
GO:1990874	vascular smooth muscle cell proliferatio...	0.000072	0.025620545	BP
GO:0007565	female pregnancy	0.000081	0.027649596	BP
GO:0048660	regulation of smooth muscle cell prolife...	0.000082	0.027649596	BP
GO:0048568	embryonic organ development	0.000083	0.027649596	BP
GO:0048659	smooth muscle cell proliferation	0.000098	0.031314	BP
GO:0008219	cell death	0.0001	0.031314	BP

GO:0051216	cartilage development	0.0001	0.031314	BP
GO:0032967	positive regulation of collagen biosynth...	0.00011	0.03377	BP
GO:0042060	wound healing	0.00013	0.037692778	BP
GO:0033002	muscle cell proliferation	0.00013	0.037692778	BP
GO:0007507	heart development	0.00013	0.037692778	BP
GO:2000026	regulation of multicellular organismal d...	0.00014	0.039854182	BP
GO:0001525	angiogenesis	0.00015	0.041202632	BP
GO:0010714	positive regulation of collagen metaboli...	0.00015	0.041202632	BP
GO:0012501	programmed cell death	0.00019	0.051290172	BP
GO:0042127	regulation of cell proliferation	0.0002	0.053074576	BP
GO:0006915	apoptotic process	0.00021	0.0547995	BP
GO:1901652	response to peptide	0.00022	0.056467869	BP
GO:0038189	neuropilin signaling pathway	0.00023	0.057160476	BP
GO:0051094	positive regulation of developmental pro...	0.00023	0.057160476	BP
GO:0009791	post-embryonic development	0.00024	0.05871375	BP
GO:0045992	negative regulation of embryonic develop...	0.00026	0.060758507	BP
GO:0048699	generation of neurons	0.00026	0.060758507	BP
GO:0048663	neuron fate commitment	0.00026	0.060758507	BP
GO:0030206	chondroitin sulfate biosynthetic process	0.00033	0.074881304	BP
GO:0048706	embryonic skeletal system development	0.00033	0.074881304	BP
GO:0002521	leukocyte differentiation	0.00034	0.076048286	BP
GO:0010712	regulation of collagen metabolic process	0.00035	0.077182394	BP
GO:1901342	regulation of vasculature development	0.00044	0.093095676	BP
GO:1904035	regulation of epithelial cell apoptotic ...	0.00044	0.093095676	BP
GO:0090596	sensory organ morphogenesis	0.00044	0.093095676	BP
GO:0061448	connective tissue development	0.00046	0.0960296	BP
GO:1902532	negative regulation of intracellular sig...	0.00049	0.100946447	BP
GO:0030204	chondroitin sulfate metabolic process	0.0005	0.101668831	BP
GO:0001999	renal response to blood flow involved in...	0.00056	0.106532165	BP
GO:0002001	renin secretion into blood stream	0.00056	0.106532165	BP
GO:0043435	response to corticotropin-releasing horm...	0.00056	0.106532165	BP
GO:0071376	cellular response to corticotropin-relea...	0.00056	0.106532165	BP
GO:0010463	mesenchymal cell proliferation	0.00059	0.106532165	BP
GO:0009952	anterior/posterior pattern specification	0.00059	0.106532165	BP
GO:0002237	response to molecule of bacterial origin	0.0006	0.106532165	BP
GO:0006935	chemotaxis	0.00061	0.106532165	BP
GO:0048598	embryonic morphogenesis	0.00061	0.106532165	BP
GO:0032488	Cdc42 protein signal transduction	0.00061	0.106532165	BP
GO:0030326	embryonic limb morphogenesis	0.00062	0.106532165	BP
GO:0035113	embryonic appendage morphogenesis	0.00062	0.106532165	BP
GO:0040011	locomotion	0.00063	0.106532165	BP
GO:0038034	signal transduction in absence of ligand	0.00064	0.106532165	BP
GO:0097192	extrinsic apoptotic signaling pathway in...	0.00064	0.106532165	BP
GO:0071310	cellular response to organic substance	0.00065	0.106532165	BP
GO:0042330	taxis	0.00065	0.106532165	BP
GO:0021511	spinal cord patterning	0.00066	0.106532165	BP
GO:0032495	response to muramyl dipeptide	0.00066	0.106532165	BP
GO:0060713	labyrinthine layer morphogenesis	0.00066	0.106532165	BP
GO:0048589	developmental growth	0.00067	0.107042755	BP
GO:1901700	response to oxygen-containing compound	0.00068	0.10754303	BP
GO:0032964	collagen biosynthetic process	0.00069	0.1080333	BP
GO:0045785	positive regulation of cell adhesion	0.00071	0.110064059	BP

GO:0009966	regulation of signal transduction	0.00073	0.112055	BP
GO:0022407	regulation of cell-cell adhesion	0.00077	0.117047476	BP
GO:0007162	negative regulation of cell adhesion	0.0008	0.120438462	BP
GO:2001239	regulation of extrinsic apoptotic signal...	0.00081	0.120782571	BP
GO:0009968	negative regulation of signal transducti...	0.00083	0.120985909	BP
GO:0023051	regulation of signaling	0.00085	0.120985909	BP
GO:0048583	regulation of response to stimulus	0.00085	0.120985909	BP
GO:0003071	renal system process involved in regulat...	0.00085	0.120985909	BP
GO:2000352	negative regulation of endothelial cell ...	0.00085	0.120985909	BP
GO:0010646	regulation of cell communication	0.00086	0.121306486	BP
GO:0030208	dermatan sulfate biosynthetic process	0.00089	0.124176207	BP
GO:0007417	central nervous system development	0.00091	0.124176207	BP
GO:0001501	skeletal system development	0.00091	0.124176207	BP
GO:0032965	regulation of collagen biosynthetic proc...	0.00092	0.124176207	BP
GO:0050650	chondroitin sulfate proteoglycan biosynt...	0.00092	0.124176207	BP
GO:0007154	cell communication	0.00093	0.124453077	BP
GO:0060711	labyrinthine layer development	0.00095	0.126052119	BP
GO:0006979	response to oxidative stress	0.00102	0.131277923	BP
GO:0023052	signaling	0.00103	0.131277923	BP
GO:0001890	placenta development	0.00104	0.131277923	BP
GO:0035107	appendage morphogenesis	0.00104	0.131277923	BP
GO:0035108	limb morphogenesis	0.00104	0.131277923	BP
GO:0008284	positive regulation of cell proliferatio...	0.00105	0.131277923	BP
GO:0006928	movement of cell or subcellular componen...	0.00108	0.131277923	BP
GO:0048661	positive regulation of smooth muscle cel...	0.00108	0.131277923	BP
GO:1904738	vascular associated smooth muscle cell m...	0.00108	0.131277923	BP
GO:1904752	regulation of vascular associated smooth...	0.00108	0.131277923	BP
GO:0070432	regulation of nucleotide-binding oligome...	0.00109	0.131277923	BP
GO:0048585	negative regulation of response to stimu...	0.00109	0.131277923	BP
GO:0050654	chondroitin sulfate proteoglycan metabol...	0.0011	0.131470992	BP
GO:1904707	positive regulation of vascular smooth m...	0.00111	0.131661136	BP
GO:0045165	cell fate commitment	0.00114	0.134202857	BP
GO:0010033	response to organic substance	0.00116	0.135538209	BP
GO:0042221	response to chemical	0.00119	0.138013556	BP
GO:0002576	platelet degranulation	0.00121	0.13930125	BP
GO:0030205	dermatan sulfate metabolic process	0.00125	0.141820652	BP
GO:0030207	chondroitin sulfate catabolic process	0.00125	0.141820652	BP
GO:0002063	chondrocyte development	0.00127	0.143053165	BP
GO:0002053	positive regulation of mesenchymal cell ...	0.00136	0.152096571	BP
GO:0007498	mesoderm development	0.0014	0.155459574	BP
GO:0006027	glycosaminoglycan catabolic process	0.00141	0.155467394	BP
GO:0045893	positive regulation of transcription, DN...	0.00142	0.155475105	BP
GO:0030316	osteoclast differentiation	0.00144	0.155490207	BP
GO:0033993	response to lipid	0.00144	0.155490207	BP
GO:0050885	neuromuscular process controlling balanc...	0.00146	0.15657	BP
GO:0045596	negative regulation of cell differentiat...	0.00151	0.160830408	BP
GO:0010648	negative regulation of cell communicatio...	0.00162	0.171380676	BP
GO:0008283	cell proliferation	0.00166	0.173742194	BP
GO:0060669	embryonic placenta morphogenesis	0.00168	0.173742194	BP
GO:0023057	negative regulation of signaling	0.00168	0.173742194	BP
GO:0034616	response to laminar fluid shear stress	0.00169	0.173742194	BP
GO:0051254	positive regulation of RNA metabolic pro...	0.00171	0.173742194	BP

GO:0043067	regulation of programmed cell death	0.00171	0.173742194	BP
GO:0040007	growth	0.00172	0.173742194	BP
GO:0048705	skeletal system morphogenesis	0.00177	0.175433855	BP
GO:0030334	regulation of cell migration	0.00181	0.175433855	BP
GO:0007409	axonogenesis	0.00183	0.175433855	BP
GO:0009617	response to bacterium	0.00183	0.175433855	BP
GO:0021521	ventral spinal cord interneuron specific...	0.00185	0.175433855	BP
GO:0034115	negative regulation of heterotypic cell-...	0.00185	0.175433855	BP
GO:0060573	cell fate specification involved in patt...	0.00185	0.175433855	BP
GO:0060710	chorio-allantoic fusion	0.00185	0.175433855	BP
GO:0070587	regulation of cell-cell adhesion involve...	0.00185	0.175433855	BP
GO:0071499	cellular response to laminar fluid shear...	0.00185	0.175433855	BP
GO:0003205	cardiac chamber development	0.00186	0.175433855	BP
GO:0032496	response to lipopolysaccharide	0.00188	0.17614125	BP
GO:0042981	regulation of apoptotic process	0.00189	0.17614125	BP
GO:0007411	axon guidance	0.00197	0.181291579	BP
GO:0006026	aminoglycan catabolic process	0.00198	0.181291579	BP
GO:0007596	blood coagulation	0.00198	0.181291579	BP
GO:1903508	positive regulation of nucleic acid-temp...	0.002	0.18205814	BP
GO:1902680	positive regulation of RNA biosynthetic ...	0.00204	0.18414767	BP
GO:0097485	neuron projection guidance	0.00207	0.18414767	BP
GO:1903706	regulation of hemopoiesis	0.00207	0.18414767	BP
GO:0006950	response to stress	0.00207	0.18414767	BP
GO:0003073	regulation of systemic arterial blood pr...	0.00209	0.184876441	BP
GO:2000351	regulation of endothelial cell apoptotic...	0.00214	0.187184246	BP
GO:0050878	regulation of body fluid levels	0.00214	0.187184246	BP
GO:0050817	coagulation	0.00217	0.188753833	BP
GO:0050651	dermatan sulfate proteoglycan biosynthet...	0.00223	0.192308804	BP
GO:0032231	regulation of actin filament bundle asse...	0.00224	0.192308804	BP
GO:0007165	signal transduction	0.00226	0.192308804	BP
GO:0050896	response to stimulus	0.00226	0.192308804	BP
GO:0043583	ear development	0.00235	0.198886216	BP
GO:0001775	cell activation	0.00237	0.199500484	BP
GO:0007599	hemostasis	0.00248	0.207371968	BP
GO:0035886	vascular smooth muscle cell differentiat...	0.00249	0.207371968	BP
GO:0002520	immune system development	0.0026	0.215387302	BP
GO:0006029	proteoglycan metabolic process	0.00265	0.218050366	BP
GO:0001701	in utero embryonic development	0.00266	0.218050366	BP
GO:0010941	regulation of cell death	0.00273	0.220725512	BP
GO:1902105	regulation of leukocyte differentiation	0.0028	0.220725512	BP
GO:0048468	cell development	0.00284	0.220725512	BP
GO:0022612	gland morphogenesis	0.00285	0.220725512	BP
GO:0021859	pyramidal neuron differentiation	0.00287	0.220725512	BP
GO:0021910	smoothened signaling pathway involved in...	0.00287	0.220725512	BP
GO:0036166	phenotypic switching	0.00287	0.220725512	BP
GO:0070586	cell-cell adhesion involved in gastrulat...	0.00287	0.220725512	BP
GO:0071225	cellular response to muramyl dipeptide	0.00287	0.220725512	BP
GO:0097084	vascular smooth muscle cell development	0.00287	0.220725512	BP
GO:1900239	regulation of phenotypic switching	0.00287	0.220725512	BP
GO:0034114	regulation of heterotypic cell-cell adhe...	0.00289	0.220725512	BP
GO:0050655	dermatan sulfate proteoglycan metabolic ...	0.00289	0.220725512	BP
GO:1904754	positive regulation of vascular associat...	0.00289	0.220725512	BP

GO:0070887	cellular response to chemical stimulus	0.00292	0.221934175	BP
GO:0045664	regulation of neuron differentiation	0.00295	0.223131159	BP
GO:0048566	embryonic digestive tract development	0.00298	0.224316635	BP
GO:0060840	artery development	0.00302	0.225162571	BP
GO:1904019	epithelial cell apoptotic process	0.00302	0.225162571	BP
GO:0006024	glycosaminoglycan biosynthetic process	0.00307	0.22780564	BP
GO:0000904	cell morphogenesis involved in different...	0.00319	0.234487465	BP
GO:0051270	regulation of cellular component movemen...	0.00319	0.234487465	BP
GO:0061061	muscle structure development	0.00329	0.240708084	BP
GO:0034614	cellular response to reactive oxygen spe...	0.00333	0.242501442	BP
GO:0045765	regulation of angiogenesis	0.00341	0.247177639	BP
GO:0061564	axon development	0.00349	0.251810737	BP
GO:0006023	aminoglycan biosynthetic process	0.00355	0.253800685	BP
GO:0010811	positive regulation of cell-substrate ad...	0.00355	0.253800685	BP
GO:0021513	spinal cord dorsal/ventral patterning	0.00366	0.259296923	BP
GO:0045109	intermediate filament organization	0.00366	0.259296923	BP
GO:0034599	cellular response to oxidative stress	0.00369	0.26024473	BP
GO:0016477	cell migration	0.00371	0.260254133	BP
GO:2000145	regulation of cell motility	0.00373	0.260254133	BP
GO:0043066	negative regulation of apoptotic process	0.00374	0.260254133	BP
GO:0048662	negative regulation of smooth muscle cel...	0.00377	0.261180929	BP
GO:0051093	negative regulation of developmental pro...	0.00382	0.263479031	BP
GO:0048666	neuron development	0.0039	0.265412342	BP
GO:0048534	hematopoietic or lymphoid organ developm...	0.00393	0.265412342	BP
GO:0008630	intrinsic apoptotic signaling pathway in...	0.00394	0.265412342	BP
GO:0001570	vasculogenesis	0.00405	0.265412342	BP
GO:2000147	positive regulation of cell motility	0.00406	0.265412342	BP
GO:0051385	response to mineralocorticoid	0.00417	0.265412342	BP
GO:0002327	immature B cell differentiation	0.00418	0.265412342	BP
GO:0048251	elastic fiber assembly	0.00418	0.265412342	BP
GO:0060712	spongiotrophoblast layer development	0.00418	0.265412342	BP
GO:0070424	regulation of nucleotide-binding oligome...	0.00418	0.265412342	BP
GO:0072498	embryonic skeletal joint development	0.00418	0.265412342	BP
GO:2000121	regulation of removal of superoxide radi...	0.00418	0.265412342	BP
GO:0072577	endothelial cell apoptotic process	0.0043	0.265412342	BP
GO:0031589	cell-substrate adhesion	0.00434	0.265412342	BP
GO:0032677	regulation of interleukin-8 production	0.00435	0.265412342	BP
GO:0030166	proteoglycan biosynthetic process	0.00444	0.265412342	BP
GO:0021538	epithalamus development	0.00446	0.265412342	BP
GO:0021775	smoothened signaling pathway involved in...	0.00446	0.265412342	BP
GO:0021776	smoothened signaling pathway involved in...	0.00446	0.265412342	BP
GO:0021986	habenula development	0.00446	0.265412342	BP
GO:0048369	lateral mesoderm morphogenesis	0.00446	0.265412342	BP
GO:0048370	lateral mesoderm formation	0.00446	0.265412342	BP
GO:0048371	lateral mesodermal cell differentiation	0.00446	0.265412342	BP
GO:0051176	positive regulation of sulfur metabolic ...	0.00446	0.265412342	BP
GO:0060948	cardiac vascular smooth muscle cell deve...	0.00446	0.265412342	BP
GO:0070426	positive regulation of nucleotide-bindin...	0.00446	0.265412342	BP
GO:0070434	positive regulation of nucleotide-bindin...	0.00446	0.265412342	BP
GO:0090679	cell differentiation involved in phenoty...	0.00446	0.265412342	BP
GO:0097070	ductus arteriosus closure	0.00446	0.265412342	BP
GO:1904580	regulation of intracellular mRNA localiz...	0.00446	0.265412342	BP

GO:1904582	positive regulation of intracellular mRN...	0.00446	0.265412342	BP
GO:1905420	vascular smooth muscle cell differentiat...	0.00446	0.265412342	BP
GO:1905915	regulation of cell differentiation invol...	0.00446	0.265412342	BP
GO:1905930	regulation of vascular smooth muscle cel...	0.00446	0.265412342	BP
GO:0007369	gastrulation	0.0045	0.265412342	BP
GO:0002115	store-operated calcium entry	0.00456	0.265412342	BP
GO:0003081	regulation of systemic arterial blood pr...	0.00456	0.265412342	BP
GO:0003094	glomerular filtration	0.00456	0.265412342	BP
GO:0045624	positive regulation of T-helper cell dif...	0.00456	0.265412342	BP
GO:0048665	neuron fate specification	0.00456	0.265412342	BP
GO:0051412	response to corticosterone	0.00456	0.265412342	BP
GO:0071498	cellular response to fluid shear stress	0.00456	0.265412342	BP
GO:0030335	positive regulation of cell migration	0.00458	0.265589111	BP
GO:0070301	cellular response to hydrogen peroxide	0.00463	0.267497823	BP
GO:0007389	pattern specification process	0.00468	0.268405714	BP
GO:0007568	aging	0.00474	0.268405714	BP
GO:0048839	inner ear development	0.00479	0.268405714	BP
GO:0042733	embryonic digit morphogenesis	0.00483	0.268405714	BP
GO:0048844	artery morphogenesis	0.00483	0.268405714	BP
GO:0003002	regionalization	0.00483	0.268405714	BP
GO:0048812	neuron projection morphogenesis	0.00487	0.268405714	BP
GO:0003012	muscle system process	0.00488	0.268405714	BP
GO:0030097	hemopoiesis	0.00488	0.268405714	BP
GO:1901099	negative regulation of signal transducti...	0.00489	0.268405714	BP
GO:2000516	positive regulation of CD4-positive, alp...	0.00489	0.268405714	BP
GO:2001240	negative regulation of extrinsic apoptot...	0.00489	0.268405714	BP
GO:0048870	cell motility	0.00491	0.268405714	BP
GO:0051674	localization of cell	0.00491	0.268405714	BP
GO:0003014	renal system process	0.00492	0.268405714	BP
GO:0003279	cardiac septum development	0.00492	0.268405714	BP
GO:0040017	positive regulation of locomotion	0.00503	0.271710825	BP
GO:0008544	epidermis development	0.00504	0.271710825	BP
GO:0048736	appendage development	0.00505	0.271710825	BP
GO:0060173	limb development	0.00505	0.271710825	BP
GO:0043434	response to peptide hormone	0.00512	0.274533699	BP
GO:0048667	cell morphogenesis involved in neuron di...	0.00521	0.278406041	BP
GO:0051960	regulation of nervous system development	0.00534	0.282989696	BP
GO:0043069	negative regulation of programmed cell d...	0.00535	0.282989696	BP
GO:0045600	positive regulation of fat cell differen...	0.00535	0.282989696	BP
GO:0035272	exocrine system development	0.0055	0.288005017	BP
GO:0038066	p38MAPK cascade	0.0055	0.288005017	BP
GO:0060976	coronary vasculature development	0.0055	0.288005017	BP
GO:0097205	renal filtration	0.0056	0.292264	BP
GO:0051928	positive regulation of calcium ion trans...	0.00567	0.292937419	BP
GO:0010464	regulation of mesenchymal cell prolifera...	0.00568	0.292937419	BP
GO:1904036	negative regulation of epithelial cell a...	0.00568	0.292937419	BP
GO:0048608	reproductive structure development	0.00575	0.292937419	BP
GO:0031175	neuron projection development	0.00576	0.292937419	BP
GO:0040012	regulation of locomotion	0.00577	0.292937419	BP
GO:0007016	cytoskeletal anchoring at plasma membran...	0.0058	0.292937419	BP
GO:0007379	segment specification	0.0058	0.292937419	BP
GO:0060579	ventral spinal cord interneuron fate com...	0.0058	0.292937419	BP

GO:0060581	cell fate commitment involved in pattern...	0.0058	0.292937419	BP
GO:0032963	collagen metabolic process	0.00587	0.295519582	BP
GO:0035148	tube formation	0.00591	0.296579712	BP
GO:1902531	regulation of intracellular signal trans...	0.00619	0.309638435	BP
GO:0031960	response to corticosteroid	0.00627	0.311648857	BP
GO:0045619	regulation of lymphocyte differentiation	0.00627	0.311648857	BP
GO:0061458	reproductive system development	0.00638	0.315115647	BP
GO:0051272	positive regulation of cellular componen...	0.00638	0.315115647	BP
GO:0048704	embryonic skeletal system morphogenesis	0.00652	0.321017736	BP
GO:0097193	intrinsic apoptotic signaling pathway	0.00672	0.329827712	BP
GO:0060055	angiogenesis involved in wound healing	0.00679	0.330158478	BP
GO:0000122	negative regulation of transcription by ...	0.00679	0.330158478	BP
GO:0007423	sensory organ development	0.00679	0.330158478	BP
GO:0010243	response to organonitrogen compound	0.00684	0.33156	BP
GO:0001892	embryonic placenta development	0.00688	0.33246963	BP
GO:0050767	regulation of neurogenesis	0.00692	0.332831319	BP
GO:0072132	mesenchyme morphogenesis	0.00693	0.332831319	BP
GO:0120039	plasma membrane bounded cell projection ...	0.00697	0.333728716	BP
GO:0021536	diencephalon development	0.00718	0.341693799	BP
GO:0032233	positive regulation of actin filament bu...	0.00718	0.341693799	BP
GO:0051384	response to glucocorticoid	0.00732	0.346623343	BP
GO:0032990	cell part morphogenesis	0.00734	0.346623343	BP
GO:0050905	neuromuscular process	0.00735	0.346623343	BP
GO:0001893	maternal placenta development	0.00753	0.352985659	BP
GO:0007435	salivary gland morphogenesis	0.00753	0.352985659	BP
GO:0051716	cellular response to stimulus	0.0076	0.355203582	BP
GO:0051241	negative regulation of multicellular org...	0.00763	0.355544375	BP
GO:0045666	positive regulation of neuron differenti...	0.00767	0.356347745	BP
GO:0048858	cell projection morphogenesis	0.0077	0.356581387	BP
GO:0042093	T-helper cell differentiation	0.00774	0.356581387	BP
GO:0032060	bleb assembly	0.00775	0.356581387	BP
GO:0021953	central nervous system neuron differenti...	0.00781	0.356581387	BP
GO:0045935	positive regulation of nucleobase-contai...	0.00784	0.356581387	BP
GO:0060429	epithelium development	0.00787	0.356581387	BP
GO:0001708	cell fate specification	0.00788	0.356581387	BP
GO:0032637	interleukin-8 production	0.00788	0.356581387	BP
GO:1904427	positive regulation of calcium ion trans...	0.00788	0.356581387	BP
GO:0010810	regulation of cell-substrate adhesion	0.00809	0.363689743	BP
GO:0019430	removal of superoxide radicals	0.00813	0.363689743	BP
GO:0033081	regulation of T cell differentiation in ...	0.00813	0.363689743	BP
GO:0046697	decidualization	0.00813	0.363689743	BP
GO:0048562	embryonic organ morphogenesis	0.00842	0.369300978	BP
GO:0030217	T cell differentiation	0.00847	0.369300978	BP
GO:0007159	leukocyte cell-cell adhesion	0.00849	0.369300978	BP
GO:0051239	regulation of multicellular organismal p...	0.00856	0.369300978	BP
GO:0034405	response to fluid shear stress	0.00859	0.369300978	BP
GO:0022414	reproductive process	0.0086	0.369300978	BP
GO:0002287	alpha-beta T cell activation involved in...	0.00861	0.369300978	BP
GO:0002293	alpha-beta T cell differentiation involv...	0.00861	0.369300978	BP
GO:0002294	CD4-positive, alpha-beta T cell differen...	0.00861	0.369300978	BP
GO:0044273	sulfur compound catabolic process	0.00861	0.369300978	BP
GO:0006801	superoxide metabolic process	0.00862	0.369300978	BP

GO:0001922	B-1 B cell homeostasis	0.00868	0.369300978	BP
GO:0045415	negative regulation of interleukin-8 bio...	0.00868	0.369300978	BP
GO:0046959	habituation	0.00868	0.369300978	BP
GO:0048664	neuron fate determination	0.00868	0.369300978	BP
GO:0072131	kidney mesenchyme morphogenesis	0.00868	0.369300978	BP
GO:0072133	metanephric mesenchyme morphogenesis	0.00868	0.369300978	BP
GO:0090063	positive regulation of microtubule nucle...	0.00868	0.369300978	BP
GO:0000003	reproduction	0.00875	0.369387668	BP
GO:0052547	regulation of peptidase activity	0.00877	0.369387668	BP
GO:0022408	negative regulation of cell-cell adhesio...	0.0088	0.369387668	BP
GO:0045766	positive regulation of angiogenesis	0.0088	0.369387668	BP
GO:0051017	actin filament bundle assembly	0.0088	0.369387668	BP
GO:0050920	regulation of chemotaxis	0.00883	0.369655909	BP
GO:0003206	cardiac chamber morphogenesis	0.00905	0.3778556	BP
GO:0042471	ear morphogenesis	0.0091	0.378932713	BP
GO:1901701	cellular response to oxygen-containing c...	0.0092	0.381069841	BP
GO:0010035	response to inorganic substance	0.0092	0.381069841	BP
GO:1905114	cell surface receptor signaling pathway ...	0.0093	0.384195515	BP
GO:0042472	inner ear morphogenesis	0.00948	0.390600947	BP
GO:0060284	regulation of cell development	0.00957	0.393055938	BP
GO:1904018	positive regulation of vasculature devel...	0.00959	0.393055938	BP
GO:0010664	negative regulation of striated muscle c...	0.00964	0.393055938	BP
GO:0060045	positive regulation of cardiac muscle ce...	0.00964	0.393055938	BP
GO:0014020	primary neural tube formation	0.00975	0.395481218	BP
GO:0060079	excitatory postsynaptic potential	0.00975	0.395481218	BP
GO:1903707	negative regulation of hemopoiesis	0.00984	0.398100465	BP
GO:0001710	mesodermal cell fate commitment	0.01003	0.401636087	BP
GO:0003214	cardiac left ventricle morphogenesis	0.01003	0.401636087	BP
GO:0021514	ventral spinal cord interneuron differen...	0.01003	0.401636087	BP
GO:0070431	nucleotide-binding oligomerization domai...	0.01003	0.401636087	BP
GO:0046632	alpha-beta T cell differentiation	0.01022	0.407320431	BP
GO:0050921	positive regulation of chemotaxis	0.01025	0.407320431	BP
GO:0010720	positive regulation of cell development	0.01025	0.407320431	BP
GO:0061572	actin filament bundle organization	0.01033	0.409460278	BP
GO:0042542	response to hydrogen peroxide	0.01041	0.411341335	BP
GO:0001704	formation of primary germ layer	0.01043	0.411341335	BP
GO:0006954	inflammatory response	0.0105	0.413061558	BP
GO:0030323	respiratory tube development	0.01058	0.413485362	BP
GO:0050679	positive regulation of epithelial cell p...	0.01058	0.413485362	BP
GO:0000302	response to reactive oxygen species	0.01059	0.413485362	BP
GO:0051962	positive regulation of nervous system de...	0.01081	0.421025299	BP
GO:0032989	cellular component morphogenesis	0.01084	0.421146104	BP
GO:0008217	regulation of blood pressure	0.01088	0.421653861	BP
GO:0043502	regulation of muscle adaptation	0.01102	0.423931548	BP
GO:0030098	lymphocyte differentiation	0.01102	0.423931548	BP
GO:0007431	salivary gland development	0.01102	0.423931548	BP
GO:0034446	substrate adhesion-dependent cell spread...	0.01115	0.42788125	BP
GO:0050769	positive regulation of neurogenesis	0.01123	0.429807451	BP
GO:0009719	response to endogenous stimulus	0.01131	0.429807451	BP
GO:0071450	cellular response to oxygen radical	0.01131	0.429807451	BP
GO:0071451	cellular response to superoxide	0.01131	0.429807451	BP
GO:1903037	regulation of leukocyte cell-cell adhesi...	0.01144	0.433695109	BP

GO:0042692	muscle cell differentiation	0.01151	0.435294855	BP
GO:0045580	regulation of T cell differentiation	0.01157	0.436509614	BP
GO:0043523	regulation of neuron apoptotic process	0.01167	0.439224014	BP
GO:0048638	regulation of developmental growth	0.0117	0.439297122	BP
GO:1901698	response to nitrogen compound	0.01177	0.440868158	BP
GO:0010942	positive regulation of cell death	0.01184	0.442431695	BP
GO:0060548	negative regulation of cell death	0.01187	0.442496643	BP
GO:0048593	camera-type eye morphogenesis	0.0119	0.442561283	BP
GO:0021915	neural tube development	0.01205	0.446020922	BP
GO:0060401	cytosolic calcium ion transport	0.01205	0.446020922	BP
GO:0001838	embryonic epithelial tube formation	0.01227	0.451700775	BP
GO:0030856	regulation of epithelial cell differenti...	0.01227	0.451700775	BP
GO:0060078	regulation of postsynaptic membrane pote...	0.01229	0.451700775	BP
GO:0060537	muscle tissue development	0.01241	0.455043021	BP
GO:0001991	regulation of systemic arterial blood pr...	0.01267	0.459924375	BP
GO:0040037	negative regulation of fibroblast growth...	0.01267	0.459924375	BP
GO:0072189	ureter development	0.01267	0.459924375	BP
GO:1905048	regulation of metallopeptidase activity	0.01267	0.459924375	BP
GO:1903038	negative regulation of leukocyte cell-ce...	0.01269	0.459924375	BP
GO:0002292	T cell differentiation involved in immun...	0.01282	0.460752906	BP
GO:1990868	response to chemokine	0.01282	0.460752906	BP
GO:1990869	cellular response to chemokine	0.01282	0.460752906	BP
GO:0006816	calcium ion transport	0.01285	0.460752906	BP
GO:0070482	response to oxygen levels	0.01286	0.460752906	BP
GO:0007205	protein kinase C-activating G protein-co...	0.01317	0.467579796	BP
GO:0035767	endothelial cell chemotaxis	0.01317	0.467579796	BP
GO:0043372	positive regulation of CD4-positive, alp...	0.01317	0.467579796	BP
GO:0048333	mesodermal cell differentiation	0.01317	0.467579796	BP
GO:0010628	positive regulation of gene expression	0.01328	0.470418462	BP
GO:0043500	muscle adaptation	0.01352	0.476811447	BP
GO:0060021	roof of mouth development	0.01366	0.476811447	BP
GO:0003044	regulation of systemic arterial blood pr...	0.01389	0.476811447	BP
GO:0071675	regulation of mononuclear cell migration	0.01389	0.476811447	BP
GO:0071674	mononuclear cell migration	0.01406	0.476811447	BP
GO:0048729	tissue morphogenesis	0.01407	0.476811447	BP
GO:0003150	muscular septum morphogenesis	0.0141	0.476811447	BP
GO:0010286	heat acclimation	0.0141	0.476811447	BP
GO:0016479	negative regulation of transcription by ...	0.0141	0.476811447	BP
GO:0021520	spinal cord motor neuron cell fate speci...	0.0141	0.476811447	BP
GO:0033504	floor plate development	0.0141	0.476811447	BP
GO:0035860	glial cell-derived neurotrophic factor r...	0.0141	0.476811447	BP
GO:0048702	embryonic neurocranium morphogenesis	0.0141	0.476811447	BP
GO:0061043	regulation of vascular wound healing	0.0141	0.476811447	BP
GO:0070245	positive regulation of thymocyte apoptot...	0.0141	0.476811447	BP
GO:0070370	cellular heat acclimation	0.0141	0.476811447	BP
GO:0070487	monocyte aggregation	0.0141	0.476811447	BP
GO:0070640	vitamin D3 metabolic process	0.0141	0.476811447	BP
GO:0097283	keratinocyte apoptotic process	0.0141	0.476811447	BP
GO:1902172	regulation of keratinocyte apoptotic pro...	0.0141	0.476811447	BP
GO:1990049	retrograde neuronal dense core vesicle t...	0.0141	0.476811447	BP
GO:0007167	enzyme linked receptor protein signaling...	0.0142	0.478464452	BP
GO:0009967	positive regulation of signal transducti...	0.01421	0.478464452	BP

GO:0071495	cellular response to endogenous stimulus	0.01444	0.484461777	BP
GO:0055074	calcium ion homeostasis	0.01445	0.484461777	BP
GO:0001938	positive regulation of endothelial cell ...	0.01464	0.488738763	BP
GO:1902106	negative regulation of leukocyte differe...	0.01464	0.488738763	BP
GO:0002573	myeloid leukocyte differentiation	0.01472	0.490363915	BP
GO:0048584	positive regulation of response to stimu...	0.01489	0.494973949	BP
GO:0000303	response to superoxide	0.0152	0.501024	BP
GO:0048536	spleen development	0.0152	0.501024	BP
GO:0085029	extracellular matrix assembly	0.0152	0.501024	BP
GO:1902175	regulation of oxidative stress-induced i...	0.0152	0.501024	BP
GO:0099565	chemical synaptic transmission, postsyna...	0.0153	0.502435697	BP
GO:0031102	neuron projection regeneration	0.01539	0.502435697	BP
GO:0045670	regulation of osteoclast differentiation	0.01539	0.502435697	BP
GO:0034764	positive regulation of transmembrane tra...	0.0154	0.502435697	BP
GO:0030203	glycosaminoglycan metabolic process	0.01542	0.502435697	BP
GO:0097237	cellular response to toxic substance	0.01544	0.502435697	BP
GO:0001502	cartilage condensation	0.01566	0.502435697	BP
GO:0042474	middle ear morphogenesis	0.01566	0.502435697	BP
GO:0043217	myelin maintenance	0.01566	0.502435697	BP
GO:0048569	post-embryonic animal organ development	0.01566	0.502435697	BP
GO:0060831	smoothened signaling pathway involved in...	0.01566	0.502435697	BP
GO:1905564	positive regulation of vascular endothel...	0.01566	0.502435697	BP
GO:2001028	positive regulation of endothelial cell ...	0.01566	0.502435697	BP
GO:0000902	cell morphogenesis	0.01576	0.504610061	BP
GO:0034620	cellular response to unfolded protein	0.01597	0.510290388	BP
GO:0001841	neural tube formation	0.01626	0.518498615	BP
GO:0035710	CD4-positive, alpha-beta T cell activati...	0.0166	0.527193103	BP
GO:1900407	regulation of cellular response to oxida...	0.0166	0.527193103	BP
GO:0098742	cell-cell adhesion via plasma-membrane a...	0.01666	0.527792419	BP
GO:0051480	regulation of cytosolic calcium ion conc...	0.01671	0.527792419	BP
GO:0051402	neuron apoptotic process	0.01672	0.527792419	BP
GO:0016331	morphogenesis of embryonic epithelium	0.0168	0.529131145	BP
GO:0002699	positive regulation of immune effector p...	0.01683	0.529131145	BP
GO:1901653	cellular response to peptide	0.01692	0.530894669	BP
GO:0071396	cellular response to lipid	0.01708	0.53484312	BP
GO:0061041	regulation of wound healing	0.01721	0.537838263	BP
GO:0000305	response to oxygen radical	0.01742	0.539640474	BP
GO:0001569	branching involved in blood vessel morph...	0.01742	0.539640474	BP
GO:0060074	synapse maturation	0.01742	0.539640474	BP
GO:0042493	response to drug	0.01743	0.539640474	BP
GO:0003008	system process	0.01744	0.539640474	BP
GO:0043068	positive regulation of programmed cell d...	0.01774	0.547840592	BP
GO:0001843	neural tube closure	0.01787	0.549686817	BP
GO:2001243	negative regulation of intrinsic apoptot...	0.01787	0.549686817	BP
GO:0070997	neuron death	0.01797	0.550736498	BP
GO:0051707	response to other organism	0.01803	0.550736498	BP
GO:0072593	reactive oxygen species metabolic proces...	0.01805	0.550736498	BP
GO:0045637	regulation of myeloid cell differentiati...	0.01808	0.550736498	BP
GO:0060326	cell chemotaxis	0.01808	0.550736498	BP
GO:0072175	epithelial tube formation	0.01815	0.551795243	BP
GO:0043367	CD4-positive, alpha-beta T cell differen...	0.0183	0.554203288	BP
GO:0097120	receptor localization to synapse	0.0183	0.554203288	BP

GO:0002682	regulation of immune system process	0.01839	0.555853726	BP
GO:0051235	maintenance of location	0.01844	0.556291098	BP
GO:0072001	renal system development	0.01853	0.557931173	BP
GO:0043207	response to external biotic stimulus	0.01879	0.564673762	BP
GO:0030949	positive regulation of vascular endothel...	0.01902	0.567045342	BP
GO:0032717	negative regulation of interleukin-8 pro...	0.01902	0.567045342	BP
GO:0098743	cell aggregation	0.01902	0.567045342	BP
GO:0002688	regulation of leukocyte chemotaxis	0.01905	0.567045342	BP
GO:0060606	tube closure	0.01905	0.567045342	BP
GO:0050673	epithelial cell proliferation	0.01912	0.568049032	BP
GO:0010952	positive regulation of peptidase activit...	0.01916	0.568159318	BP
GO:0007610	behavior	0.01926	0.570045028	BP
GO:2001257	regulation of cation channel activity	0.01945	0.574482406	BP
GO:0009725	response to hormone	0.01951	0.574482406	BP
GO:0031325	positive regulation of cellular metaboli...	0.01952	0.574482406	BP
GO:0045622	regulation of T-helper cell differentiat...	0.01984	0.57801828	BP
GO:0001707	mesoderm formation	0.01988	0.57801828	BP
GO:0006874	cellular calcium ion homeostasis	0.01997	0.57801828	BP
GO:0003006	developmental process involved in reprod...	0.02011	0.57801828	BP
GO:0019221	cytokine-mediated signaling pathway	0.02011	0.57801828	BP
GO:0010975	regulation of neuron projection developm...	0.02024	0.57801828	BP
GO:0006022	aminoglycan metabolic process	0.02034	0.57801828	BP
GO:1903708	positive regulation of hemopoiesis	0.02034	0.57801828	BP
GO:0046631	alpha-beta T cell activation	0.0205	0.57801828	BP
GO:0010968	regulation of microtubule nucleation	0.0206	0.57801828	BP
GO:0019800	peptide cross-linking via chondroitin 4-...	0.0206	0.57801828	BP
GO:0035726	common myeloid progenitor cell prolifera...	0.0206	0.57801828	BP
GO:0042661	regulation of mesodermal cell fate speci...	0.0206	0.57801828	BP
GO:0043569	negative regulation of insulin-like grow...	0.0206	0.57801828	BP
GO:0045110	intermediate filament bundle assembly	0.0206	0.57801828	BP
GO:0046958	nonassociative learning	0.0206	0.57801828	BP
GO:0060022	hard palate development	0.0206	0.57801828	BP
GO:0060136	embryonic process involved in female pre...	0.0206	0.57801828	BP
GO:0060754	positive regulation of mast cell chemota...	0.0206	0.57801828	BP
GO:0061419	positive regulation of transcription fro...	0.0206	0.57801828	BP
GO:0090677	reversible differentiation	0.0206	0.57801828	BP
GO:1901341	positive regulation of store-operated ca...	0.0206	0.57801828	BP
GO:1901621	negative regulation of smoothened signal...	0.0206	0.57801828	BP
GO:1905770	regulation of mesodermal cell differenti...	0.0206	0.57801828	BP
GO:1905902	regulation of mesoderm formation	0.0206	0.57801828	BP
GO:2000321	positive regulation of T-helper 17 cell ...	0.0206	0.57801828	BP
GO:0006367	transcription initiation from RNA polyme...	0.02084	0.583706404	BP
GO:0071363	cellular response to growth factor stimu...	0.021	0.5871375	BP
GO:0045620	negative regulation of lymphocyte differ...	0.02104	0.587207273	BP
GO:0030879	mammary gland development	0.02123	0.590405169	BP
GO:1904064	positive regulation of cation transmembr...	0.02123	0.590405169	BP
GO:0045321	leukocyte activation	0.02137	0.59324484	BP
GO:0060135	maternal process involved in female preg...	0.02156	0.597060951	BP
GO:0071345	cellular response to cytokine stimulus	0.02162	0.597060951	BP
GO:0002376	immune system process	0.02164	0.597060951	BP
GO:0030162	regulation of proteolysis	0.02166	0.597060951	BP
GO:0065008	regulation of biological quality	0.02179	0.599588805	BP

GO:0008589	regulation of smoothened signaling pathw...	0.0221	0.606845612	BP
GO:0022603	regulation of anatomical structure morph...	0.02216	0.606845612	BP
GO:0036293	response to decreased oxygen levels	0.02217	0.606845612	BP
GO:1901214	regulation of neuron death	0.02224	0.607699267	BP
GO:0045601	regulation of endothelial cell different...	0.02245	0.611303739	BP
GO:0045880	positive regulation of smoothened signal...	0.02245	0.611303739	BP
GO:0070848	response to growth factor	0.02259	0.613075301	BP
GO:0007567	parturition	0.02275	0.613075301	BP
GO:0070242	thymocyte apoptotic process	0.02275	0.613075301	BP
GO:1902259	regulation of delayed rectifier potassiu...	0.02275	0.613075301	BP
GO:1904706	negative regulation of vascular smooth m...	0.02275	0.613075301	BP
GO:1905063	regulation of vascular smooth muscle cel...	0.02275	0.613075301	BP
GO:0050865	regulation of cell activation	0.02282	0.613905052	BP
GO:0010524	positive regulation of calcium ion trans...	0.02314	0.618264471	BP
GO:0042149	cellular response to glucose starvation	0.02314	0.618264471	BP
GO:0045773	positive regulation of axon extension	0.02314	0.618264471	BP
GO:0070098	chemokine-mediated signaling pathway	0.02314	0.618264471	BP
GO:0048332	mesoderm morphogenesis	0.02332	0.62095449	BP
GO:0051145	smooth muscle cell differentiation	0.02332	0.62095449	BP
GO:0030534	adult behavior	0.02347	0.622830153	BP
GO:0048592	eye morphogenesis	0.02347	0.622830153	BP
GO:2001236	regulation of extrinsic apoptotic signal...	0.02352	0.623100914	BP
GO:0022409	positive regulation of cell-cell adhesio...	0.02365	0.625486571	BP
GO:0090287	regulation of cellular response to growt...	0.02402	0.634147839	BP
GO:0040008	regulation of growth	0.02414	0.634147839	BP
GO:0048545	response to steroid hormone	0.02417	0.634147839	BP
GO:0050728	negative regulation of inflammatory resp...	0.02418	0.634147839	BP
GO:1903510	mucopolysaccharide metabolic process	0.02418	0.634147839	BP
GO:0001709	cell fate determination	0.02526	0.657229884	BP
GO:0001990	regulation of systemic arterial blood pr...	0.02526	0.657229884	BP
GO:0036003	positive regulation of transcription fro...	0.02526	0.657229884	BP
GO:0060674	placenta blood vessel development	0.02526	0.657229884	BP
GO:0050821	protein stabilization	0.02527	0.657229884	BP
GO:0045646	regulation of erythrocyte differentiatio...	0.02537	0.658736468	BP
GO:0032101	regulation of response to external stimu...	0.02568	0.665681722	BP
GO:1903034	regulation of response to wounding	0.02575	0.666392975	BP
GO:0060322	head development	0.02584	0.666710082	BP
GO:0007224	smoothened signaling pathway	0.02589	0.666710082	BP
GO:0042552	myelination	0.02589	0.666710082	BP
GO:0043065	positive regulation of apoptotic process	0.02602	0.66837423	BP
GO:0010557	positive regulation of macromolecule bio...	0.02604	0.66837423	BP
GO:0051090	regulation of DNA-binding transcription ...	0.0262	0.67138036	BP
GO:0007221	positive regulation of transcription of ...	0.02684	0.677461498	BP
GO:0030903	notochord development	0.02684	0.677461498	BP
GO:0042359	vitamin D metabolic process	0.02684	0.677461498	BP
GO:0071636	positive regulation of transforming grow...	0.02684	0.677461498	BP
GO:0072574	hepatocyte proliferation	0.02684	0.677461498	BP
GO:0072575	epithelial cell proliferation involved i...	0.02684	0.677461498	BP
GO:1900153	positive regulation of nuclear-transcrib...	0.02684	0.677461498	BP
GO:1902176	negative regulation of oxidative stress-...	0.02684	0.677461498	BP
GO:2001026	regulation of endothelial cell chemotaxi...	0.02684	0.677461498	BP
GO:0048565	digestive tract development	0.02687	0.677461498	BP

GO:0030901	midbrain development	0.02698	0.679141254	BP
GO:0060395	SMAD protein signal transduction	0.02715	0.680891616	BP
GO:0072376	protein activation cascade	0.02715	0.680891616	BP
GO:0051253	negative regulation of RNA metabolic pro...	0.02718	0.680891616	BP
GO:0050678	regulation of epithelial cell proliferat...	0.02772	0.681440815	BP
GO:0009636	response to toxic substance	0.02777	0.681440815	BP
GO:0015886	heme transport	0.0281	0.681440815	BP
GO:0021860	pyramidal neuron development	0.0281	0.681440815	BP
GO:0031077	post-embryonic camera-type eye developme...	0.0281	0.681440815	BP
GO:0031086	nuclear-transcribed mRNA catabolic proce...	0.0281	0.681440815	BP
GO:0033085	negative regulation of T cell differenti...	0.0281	0.681440815	BP
GO:0048842	positive regulation of axon extension in...	0.0281	0.681440815	BP
GO:0060012	synaptic transmission, glycinergic	0.0281	0.681440815	BP
GO:0060013	righting reflex	0.0281	0.681440815	BP
GO:0060020	Bergmann glial cell differentiation	0.0281	0.681440815	BP
GO:0060272	embryonic skeletal joint morphogenesis	0.0281	0.681440815	BP
GO:0060484	lung-associated mesenchyme development	0.0281	0.681440815	BP
GO:0060753	regulation of mast cell chemotaxis	0.0281	0.681440815	BP
GO:0060947	cardiac vascular smooth muscle cell diff...	0.0281	0.681440815	BP
GO:0071493	cellular response to UV-B	0.0281	0.681440815	BP
GO:0071676	negative regulation of mononuclear cell ...	0.0281	0.681440815	BP
GO:1905050	positive regulation of metallopeptidase ...	0.0281	0.681440815	BP
GO:2000318	positive regulation of T-helper 17 type ...	0.0281	0.681440815	BP
GO:2000542	negative regulation of gastrulation	0.0281	0.681440815	BP
GO:0048246	macrophage chemotaxis	0.02827	0.681440815	BP
GO:0051281	positive regulation of release of seques...	0.02827	0.681440815	BP
GO:0055023	positive regulation of cardiac muscle ti...	0.02827	0.681440815	BP
GO:0071634	regulation of transforming growth factor...	0.02827	0.681440815	BP
GO:0003231	cardiac ventricle development	0.02829	0.681440815	BP
GO:0032103	positive regulation of response to exter...	0.02845	0.681582248	BP
GO:0002694	regulation of leukocyte activation	0.02845	0.681582248	BP
GO:0007272	ensheathment of neurons	0.02847	0.681582248	BP
GO:0008366	axon ensheathment	0.02847	0.681582248	BP
GO:0035282	segmentation	0.02876	0.685959148	BP
GO:0051492	regulation of stress fiber assembly	0.02876	0.685959148	BP
GO:0007420	brain development	0.0289	0.685959148	BP
GO:0001666	response to hypoxia	0.02897	0.685959148	BP
GO:0008015	blood circulation	0.02913	0.685959148	BP
GO:0033077	T cell differentiation in thymus	0.0292	0.685959148	BP
GO:2001242	regulation of intrinsic apoptotic signal...	0.02939	0.685959148	BP
GO:0006959	humoral immune response	0.02984	0.685959148	BP
GO:0001961	positive regulation of cytokine-mediated...	0.03023	0.685959148	BP
GO:0006956	complement activation	0.03023	0.685959148	BP
GO:0090303	positive regulation of wound healing	0.03023	0.685959148	BP
GO:2000179	positive regulation of neural precursor ...	0.03023	0.685959148	BP
GO:2000514	regulation of CD4-positive, alpha-beta T...	0.03023	0.685959148	BP
GO:0055065	metal ion homeostasis	0.03026	0.685959148	BP
GO:0002702	positive regulation of production of mol...	0.03061	0.685959148	BP
GO:1902882	regulation of response to oxidative stre...	0.03061	0.685959148	BP
GO:0035556	intracellular signal transduction	0.03063	0.685959148	BP
GO:0090257	regulation of muscle system process	0.03063	0.685959148	BP
GO:0030595	leukocyte chemotaxis	0.03068	0.685959148	BP

GO:0023014	signal transduction by protein phosphory...	0.03082	0.685959148	BP
GO:0006986	response to unfolded protein	0.03101	0.685959148	BP
GO:0031099	regeneration	0.03101	0.685959148	BP
GO:0019725	cellular homeostasis	0.0312	0.685959148	BP
GO:0043524	negative regulation of neuron apoptotic ...	0.03124	0.685959148	BP
GO:0002043	blood vessel endothelial cell proliferat...	0.03129	0.685959148	BP
GO:0008090	retrograde axonal transport	0.03129	0.685959148	BP
GO:0060004	reflex	0.03129	0.685959148	BP
GO:0072576	liver morphogenesis	0.03129	0.685959148	BP
GO:0101023	vascular endothelial cell proliferation	0.03129	0.685959148	BP
GO:1905562	regulation of vascular endothelial cell ...	0.03129	0.685959148	BP
GO:0010508	positive regulation of autophagy	0.0313	0.685959148	BP
GO:0035418	protein localization to synapse	0.03136	0.685959148	BP
GO:0070373	negative regulation of ERK1 and ERK2 cas...	0.03136	0.685959148	BP
GO:1901031	regulation of response to reactive oxyge...	0.03149	0.685959148	BP
GO:0009891	positive regulation of biosynthetic proc...	0.03198	0.685959148	BP
GO:0051403	stress-activated MAPK cascade	0.0324	0.685959148	BP
GO:0090288	negative regulation of cellular response...	0.03269	0.685959148	BP
GO:0002720	positive regulation of cytokine producti...	0.03287	0.685959148	BP
GO:0097190	apoptotic signaling pathway	0.03294	0.685959148	BP
GO:0018209	peptidyl-serine modification	0.03319	0.685959148	BP
GO:0043281	regulation of cysteine-type endopeptidas...	0.03335	0.685959148	BP
GO:0010976	positive regulation of neuron projection...	0.0334	0.685959148	BP
GO:1900449	regulation of glutamate receptor signali...	0.03362	0.685959148	BP
GO:0051704	multi-organism process	0.03369	0.685959148	BP
GO:0050808	synapse organization	0.03383	0.685959148	BP
GO:0006875	cellular metal ion homeostasis	0.03402	0.685959148	BP
GO:0030168	platelet activation	0.03419	0.685959148	BP
GO:0055001	muscle cell development	0.03479	0.685959148	BP
GO:0009607	response to biotic stimulus	0.03481	0.685959148	BP
GO:0006968	cellular defense response	0.03491	0.685959148	BP
GO:0010662	regulation of striated muscle cell apopt...	0.03491	0.685959148	BP
GO:0021517	ventral spinal cord development	0.03491	0.685959148	BP
GO:0046627	negative regulation of insulin receptor ...	0.03491	0.685959148	BP
GO:0046685	response to arsenic-containing substance	0.03491	0.685959148	BP
GO:0071604	transforming growth factor beta producti...	0.03491	0.685959148	BP
GO:0051173	positive regulation of nitrogen compound...	0.03532	0.685959148	BP
GO:0007204	positive regulation of cytosolic calcium...	0.03562	0.685959148	BP
GO:0034767	positive regulation of ion transmembrane...	0.03574	0.685959148	BP
GO:0051147	regulation of muscle cell differentiatio...	0.03574	0.685959148	BP
GO:0060402	calcium ion transport into cytosol	0.03574	0.685959148	BP
GO:0003013	circulatory system process	0.03587	0.685959148	BP
GO:0010667	negative regulation of cardiac muscle ce...	0.03611	0.685959148	BP
GO:0030728	ovulation	0.03611	0.685959148	BP
GO:0044342	type B pancreatic cell proliferation	0.03611	0.685959148	BP
GO:0060561	apoptotic process involved in morphogene...	0.03611	0.685959148	BP
GO:0060716	labyrinthine layer blood vessel developm...	0.03611	0.685959148	BP
GO:1900151	regulation of nuclear-transcribed mRNA c...	0.03611	0.685959148	BP
GO:1901673	regulation of mitotic spindle assembly	0.03611	0.685959148	BP
GO:2000482	regulation of interleukin-8 secretion	0.03611	0.685959148	BP
GO:2000679	positive regulation of transcription reg...	0.03611	0.685959148	BP
GO:1901136	carbohydrate derivative catabolic proces...	0.03624	0.685959148	BP

GO:0043409	negative regulation of MAPK cascade	0.03625	0.685959148	BP
GO:0048639	positive regulation of developmental gro...	0.03625	0.685959148	BP
GO:0061138	morphogenesis of a branching epithelium	0.03625	0.685959148	BP
GO:0010647	positive regulation of cell communicatio...	0.03641	0.685959148	BP
GO:0002683	negative regulation of immune system pro...	0.03642	0.685959148	BP
GO:0001867	complement activation, lectin pathway	0.03651	0.685959148	BP
GO:0001976	neurological system process involved in ...	0.03651	0.685959148	BP
GO:0002741	positive regulation of cytokine secretio...	0.03651	0.685959148	BP
GO:0019227	neuronal action potential propagation	0.03651	0.685959148	BP
GO:0032253	dense core granule localization	0.03651	0.685959148	BP
GO:0045602	negative regulation of endothelial cell ...	0.03651	0.685959148	BP
GO:0045647	negative regulation of erythrocyte diffe...	0.03651	0.685959148	BP
GO:0090185	negative regulation of kidney developmen...	0.03651	0.685959148	BP
GO:0090400	stress-induced premature senescence	0.03651	0.685959148	BP
GO:0098870	action potential propagation	0.03651	0.685959148	BP
GO:0099519	dense core granule cytoskeletal transpor...	0.03651	0.685959148	BP
GO:0099541	trans-synaptic signaling by lipid	0.03651	0.685959148	BP
GO:0099542	trans-synaptic signaling by endocannabin...	0.03651	0.685959148	BP
GO:1901029	negative regulation of mitochondrial out...	0.03651	0.685959148	BP
GO:1901339	regulation of store-operated calcium cha...	0.03651	0.685959148	BP
GO:1901620	regulation of smoothened signaling pathw...	0.03651	0.685959148	BP
GO:1901950	dense core granule transport	0.03651	0.685959148	BP
GO:1902260	negative regulation of delayed rectifier...	0.03651	0.685959148	BP
GO:1902669	positive regulation of axon guidance	0.03651	0.685959148	BP
GO:0072507	divalent inorganic cation homeostasis	0.03718	0.685959148	BP
GO:0008285	negative regulation of cell proliferatio...	0.03762	0.685959148	BP
GO:0034097	response to cytokine	0.03778	0.685959148	BP
GO:0055123	digestive system development	0.03796	0.685959148	BP
GO:0051249	regulation of lymphocyte activation	0.03806	0.685959148	BP
GO:0000165	MAPK cascade	0.03809	0.685959148	BP
GO:0045786	negative regulation of cell cycle	0.03825	0.685959148	BP
GO:0002455	humoral immune response mediated by circ...	0.03854	0.685959148	BP
GO:0038083	peptidyl-tyrosine autophosphorylation	0.03854	0.685959148	BP
GO:0060421	positive regulation of heart growth	0.03854	0.685959148	BP
GO:1903539	protein localization to postsynaptic mem...	0.03854	0.685959148	BP
GO:0030199	collagen fibril organization	0.03856	0.685959148	BP
GO:0034113	heterotypic cell-cell adhesion	0.03856	0.685959148	BP
GO:0034762	regulation of transmembrane transport	0.03881	0.685959148	BP
GO:0001315	age-dependent response to reactive oxyge...	0.03908	0.685959148	BP
GO:0001979	regulation of systemic arterial blood pr...	0.03908	0.685959148	BP
GO:0002007	detection of hypoxic conditions in blood...	0.03908	0.685959148	BP
GO:0002041	intussusceptive angiogenesis	0.03908	0.685959148	BP
GO:0002332	transitional stage B cell differentiatio...	0.03908	0.685959148	BP
GO:0002333	transitional one stage B cell differenti...	0.03908	0.685959148	BP
GO:0003027	regulation of systemic arterial blood pr...	0.03908	0.685959148	BP
GO:0003029	detection of hypoxic conditions in blood...	0.03908	0.685959148	BP
GO:0003068	regulation of systemic arterial blood pr...	0.03908	0.685959148	BP
GO:0003069	acetylcholine-mediated vasodilation invo...	0.03908	0.685959148	BP
GO:0003070	regulation of systemic arterial blood pr...	0.03908	0.685959148	BP
GO:0003292	cardiac septum cell differentiation	0.03908	0.685959148	BP
GO:0003342	proepicardium development	0.03908	0.685959148	BP
GO:0003343	septum transversum development	0.03908	0.685959148	BP

GO:0006218	uridine catabolic process	0.03908	0.685959148	BP
GO:0009444	pyruvate oxidation	0.03908	0.685959148	BP
GO:0014822	detection of wounding	0.03908	0.685959148	BP
GO:0015730	propanoate transport	0.03908	0.685959148	BP
GO:0016131	brassinosteroid metabolic process	0.03908	0.685959148	BP
GO:0016132	brassinosteroid biosynthetic process	0.03908	0.685959148	BP
GO:0018400	peptidyl-proline hydroxylation to 3-hydr...	0.03908	0.685959148	BP
GO:0018969	thiocyanate metabolic process	0.03908	0.685959148	BP
GO:0021508	floor plate formation	0.03908	0.685959148	BP
GO:0021512	spinal cord anterior/posterior patternin...	0.03908	0.685959148	BP
GO:0021539	subthalamus development	0.03908	0.685959148	BP
GO:0021682	nerve maturation	0.03908	0.685959148	BP
GO:0030961	peptidyl-arginine hydroxylation	0.03908	0.685959148	BP
GO:0032690	negative regulation of interleukin-1 alp...	0.03908	0.685959148	BP
GO:0033514	L-lysine catabolic process to acetyl-CoA...	0.03908	0.685959148	BP
GO:0034147	regulation of toll-like receptor 5 signa...	0.03908	0.685959148	BP
GO:0034148	negative regulation of toll-like recepto...	0.03908	0.685959148	BP
GO:0034263	positive regulation of autophagy in resp...	0.03908	0.685959148	BP
GO:0035708	interleukin-4-dependent isotype switchin...	0.03908	0.685959148	BP
GO:0035713	response to nitrogen dioxide	0.03908	0.685959148	BP
GO:0035724	CD24 biosynthetic process	0.03908	0.685959148	BP
GO:0038097	positive regulation of mast cell activat...	0.03908	0.685959148	BP
GO:0038159	C-X-C chemokine receptor CXCR4 signaling...	0.03908	0.685959148	BP
GO:0038160	CXCL12-activated CXCR4 signaling pathway	0.03908	0.685959148	BP
GO:0038167	epidermal growth factor receptor signali...	0.03908	0.685959148	BP
GO:0038168	epidermal growth factor receptor signali...	0.03908	0.685959148	BP
GO:0038195	urokinase plasminogen activator signalin...	0.03908	0.685959148	BP
GO:0043063	intercellular bridge organization	0.03908	0.685959148	BP
GO:0046086	adenosine biosynthetic process	0.03908	0.685959148	BP
GO:0048567	ectodermal digestive tract morphogenesis	0.03908	0.685959148	BP
GO:0048613	embryonic ectodermal digestive tract mor...	0.03908	0.685959148	BP
GO:0050894	determination of affect	0.03908	0.685959148	BP
GO:0051041	positive regulation of calcium-independe...	0.03908	0.685959148	BP
GO:0051355	proprioception involved in equilibriocep...	0.03908	0.685959148	BP
GO:0060092	regulation of synaptic transmission, gly...	0.03908	0.685959148	BP
GO:0060461	right lung morphogenesis	0.03908	0.685959148	BP
GO:0060466	activation of meiosis involved in egg ac...	0.03908	0.685959148	BP
GO:0060922	atrioventricular node cell differentiati...	0.03908	0.685959148	BP
GO:0060928	atrioventricular node cell development	0.03908	0.685959148	BP
GO:0061185	negative regulation of dermatome develop...	0.03908	0.685959148	BP
GO:0061536	glycine secretion	0.03908	0.685959148	BP
GO:0061537	glycine secretion, neurotransmission	0.03908	0.685959148	BP
GO:0061757	leukocyte adhesion to arterial endotheli...	0.03908	0.685959148	BP
GO:0070429	negative regulation of nucleotide-bindin...	0.03908	0.685959148	BP
GO:0070483	detection of hypoxia	0.03908	0.685959148	BP
GO:0071275	cellular response to aluminum ion	0.03908	0.685959148	BP
GO:0071707	immunoglobulin heavy chain V-D-J recombi...	0.03908	0.685959148	BP
GO:0071954	chemokine (C-C motif) ligand 11 producti...	0.03908	0.685959148	BP
GO:0072027	connecting tubule development	0.03908	0.685959148	BP
GO:0072165	anterior mesonephric tubule development	0.03908	0.685959148	BP
GO:0072166	posterior mesonephric tubule development	0.03908	0.685959148	BP
GO:0072167	specification of mesonephric tubule iden...	0.03908	0.685959148	BP

GO:0072168	specification of anterior mesonephric tu...	0.03908	0.685959148	BP
GO:0072169	specification of posterior mesonephric t...	0.03908	0.685959148	BP
GO:0072184	renal vesicle progenitor cell differenti...	0.03908	0.685959148	BP
GO:0072258	metanephric interstitial fibroblast diff...	0.03908	0.685959148	BP
GO:0072259	metanephric interstitial fibroblast deve...	0.03908	0.685959148	BP
GO:0072268	pattern specification involved in metane...	0.03908	0.685959148	BP
GO:0072286	metanephric connecting tubule developmen...	0.03908	0.685959148	BP
GO:0072366	regulation of cellular ketone metabolic ...	0.03908	0.685959148	BP
GO:0072573	tolerance induction to lipopolysaccharid...	0.03908	0.685959148	BP
GO:0097022	lymphocyte migration into lymph node	0.03908	0.685959148	BP
GO:0097403	cellular response to raffinose	0.03908	0.685959148	BP
GO:0099627	neurotransmitter receptor cycle	0.03908	0.685959148	BP
GO:0099630	postsynaptic neurotransmitter receptor c...	0.03908	0.685959148	BP
GO:0100057	regulation of phenotypic switching by tr...	0.03908	0.685959148	BP
GO:0110057	regulation of blood vessel endothelial c...	0.03908	0.685959148	BP
GO:0110059	negative regulation of blood vessel endo...	0.03908	0.685959148	BP
GO:1900134	negative regulation of renin secretion i...	0.03908	0.685959148	BP
GO:1900281	positive regulation of CD4-positive, alp...	0.03908	0.685959148	BP
GO:1901202	negative regulation of extracellular mat...	0.03908	0.685959148	BP
GO:1901545	response to raffinose	0.03908	0.685959148	BP
GO:1901834	regulation of deadenylation-independent ...	0.03908	0.685959148	BP
GO:1901835	positive regulation of deadenylation-ind...	0.03908	0.685959148	BP
GO:1902380	positive regulation of endoribonuclease ...	0.03908	0.685959148	BP
GO:1903465	positive regulation of mitotic cell cycl...	0.03908	0.685959148	BP
GO:1903547	regulation of growth hormone activity	0.03908	0.685959148	BP
GO:1903570	regulation of protein kinase D signaling	0.03908	0.685959148	BP
GO:1903572	positive regulation of protein kinase D ...	0.03908	0.685959148	BP
GO:1903788	positive regulation of glutathione biosy...	0.03908	0.685959148	BP
GO:1904178	negative regulation of adipose tissue de...	0.03908	0.685959148	BP
GO:1904205	negative regulation of skeletal muscle h...	0.03908	0.685959148	BP
GO:1904246	negative regulation of polynucleotide ad...	0.03908	0.685959148	BP
GO:1904271	L-proline import across plasma membrane	0.03908	0.685959148	BP
GO:1904531	positive regulation of actin filament bi...	0.03908	0.685959148	BP
GO:1904555	L-proline transmembrane transport	0.03908	0.685959148	BP
GO:1904618	positive regulation of actin binding	0.03908	0.685959148	BP
GO:1904826	regulation of hydrogen sulfide biosynthe...	0.03908	0.685959148	BP
GO:1904828	positive regulation of hydrogen sulfide ...	0.03908	0.685959148	BP
GO:1904897	regulation of hepatic stellate cell prol...	0.03908	0.685959148	BP
GO:1904899	positive regulation of hepatic stellate ...	0.03908	0.685959148	BP
GO:1904997	regulation of leukocyte adhesion to arte...	0.03908	0.685959148	BP
GO:1904998	negative regulation of leukocyte adhesio...	0.03908	0.685959148	BP
GO:1905000	regulation of membrane repolarization du...	0.03908	0.685959148	BP
GO:1905647	proline import across plasma membrane	0.03908	0.685959148	BP
GO:1905672	negative regulation of lysosome organiza...	0.03908	0.685959148	BP
GO:1905917	positive regulation of cell differentiat...	0.03908	0.685959148	BP
GO:1905932	positive regulation of vascular smooth m...	0.03908	0.685959148	BP
GO:1990051	activation of protein kinase C activity	0.03908	0.685959148	BP
GO:1990256	signal clustering	0.03908	0.685959148	BP
GO:1990349	gap junction-mediated intercellular tran...	0.03908	0.685959148	BP
GO:1990771	clathrin-dependent extracellular exosome...	0.03908	0.685959148	BP
GO:1990922	hepatic stellate cell proliferation	0.03908	0.685959148	BP
GO:2000349	negative regulation of CD40 signaling pa...	0.03908	0.685959148	BP

GO:2000438	negative regulation of monocyte extravas...	0.03908	0.685959148	BP
GO:2000525	positive regulation of T cell costimulat...	0.03908	0.685959148	BP
GO:2000559	regulation of CD24 biosynthetic process	0.03908	0.685959148	BP
GO:2000560	positive regulation of CD24 biosynthetic...	0.03908	0.685959148	BP
GO:2000571	regulation of interleukin-4-dependent is...	0.03908	0.685959148	BP
GO:2000572	positive regulation of interleukin-4-dep...	0.03908	0.685959148	BP
GO:2000723	negative regulation of cardiac vascular ...	0.03908	0.685959148	BP
GO:2000830	positive regulation of parathyroid hormo...	0.03908	0.685959148	BP
GO:2001173	regulation of histone H2B conserved C-te...	0.03908	0.685959148	BP
GO:2001207	regulation of transcription elongation f...	0.03908	0.685959148	BP
GO:2001208	negative regulation of transcription elo...	0.03908	0.685959148	BP
GO:0023056	positive regulation of signaling	0.03916	0.686593639	BP
GO:0035967	cellular response to topologically incor...	0.03928	0.687927248	BP
GO:0007178	transmembrane receptor protein serine/th...	0.03941	0.689432816	BP
GO:0045185	maintenance of protein location	0.03985	0.695575753	BP
GO:1901343	negative regulation of vasculature devel...	0.03985	0.695575753	BP
GO:0060562	epithelial tube morphogenesis	0.03991	0.695847294	BP
GO:0046649	lymphocyte activation	0.04027	0.700737733	BP
GO:0010604	positive regulation of macromolecule met...	0.04028	0.700737733	BP
GO:0044272	sulfur compound biosynthetic process	0.04056	0.70482566	BP
GO:0070838	divalent metal ion transport	0.0408	0.7082102	BP
GO:0002009	morphogenesis of an epithelium	0.04098	0.708358546	BP
GO:0002690	positive regulation of leukocyte chemota...	0.04102	0.708358546	BP
GO:0003151	outflow tract morphogenesis	0.04102	0.708358546	BP
GO:0060411	cardiac septum morphogenesis	0.04102	0.708358546	BP
GO:0021510	spinal cord development	0.04108	0.708358546	BP
GO:0072384	organelle transport along microtubule	0.04108	0.708358546	BP
GO:0031281	positive regulation of cyclase activity	0.04128	0.710242813	BP
GO:0036499	PERK-mediated unfolded protein response	0.04128	0.710242813	BP
GO:0051496	positive regulation of stress fiber asse...	0.04162	0.71530663	BP
GO:0051924	regulation of calcium ion transport	0.04167	0.715380691	BP
GO:0072503	cellular divalent inorganic cation homeo...	0.04202	0.720599277	BP
GO:0010656	negative regulation of muscle cell apopt...	0.04238	0.722814444	BP
GO:0010658	striated muscle cell apoptotic process	0.04238	0.722814444	BP
GO:0045581	negative regulation of T cell differenti...	0.04238	0.722814444	BP
GO:1900077	negative regulation of cellular response...	0.04238	0.722814444	BP
GO:2000310	regulation of NMDA receptor activity	0.04238	0.722814444	BP
GO:0060759	regulation of response to cytokine stimu...	0.04248	0.723731621	BP
GO:0001822	kidney development	0.04302	0.73151	BP
GO:0007169	transmembrane receptor protein tyrosine ...	0.04303	0.73151	BP
GO:0002367	cytokine production involved in immune r...	0.04342	0.736251786	BP
GO:0098869	cellular oxidant detoxification	0.04342	0.736251786	BP
GO:0031328	positive regulation of cellular biosynth...	0.04345	0.736251786	BP
GO:0044344	cellular response to fibroblast growth f...	0.04355	0.73635243	BP
GO:0097553	calcium ion transmembrane import into cy...	0.04355	0.73635243	BP
GO:0051781	positive regulation of cell division	0.0437	0.738091586	BP
GO:0045930	negative regulation of mitotic cell cycl...	0.044	0.742064273	BP
GO:0042476	odontogenesis	0.04403	0.742064273	BP
GO:0030324	lung development	0.04415	0.743286613	BP
GO:0045604	regulation of epidermal cell differentia...	0.04482	0.751634575	BP
GO:0046635	positive regulation of alpha-beta T cell...	0.04482	0.751634575	BP
GO:1902369	negative regulation of RNA catabolic pro...	0.04482	0.751634575	BP

GO:0009893	positive regulation of metabolic process	0.04487	0.751634575	BP
GO:0042594	response to starvation	0.04522	0.751634575	BP
GO:0001503	ossification	0.04556	0.751634575	BP
GO:0007501	mesodermal cell fate specification	0.04575	0.751634575	BP
GO:0014877	response to muscle inactivity involved i...	0.04575	0.751634575	BP
GO:0014894	response to denervation involved in regu...	0.04575	0.751634575	BP
GO:0032490	detection of molecule of bacterial origi...	0.04575	0.751634575	BP
GO:0033690	positive regulation of osteoblast prolif...	0.04575	0.751634575	BP
GO:0045762	positive regulation of adenylate cyclase...	0.04575	0.751634575	BP
GO:0046602	regulation of mitotic centrosome separat...	0.04575	0.751634575	BP
GO:0060837	blood vessel endothelial cell differenti...	0.04575	0.751634575	BP
GO:0070234	positive regulation of T cell apoptotic ...	0.04575	0.751634575	BP
GO:0072203	cell proliferation involved in metanephr...	0.04575	0.751634575	BP
GO:0090336	positive regulation of brown fat cell di...	0.04575	0.751634575	BP
GO:0097709	connective tissue replacement	0.04575	0.751634575	BP
GO:1901678	iron coordination entity transport	0.04575	0.751634575	BP
GO:1902237	positive regulation of endoplasmic retic...	0.04575	0.751634575	BP
GO:1903817	negative regulation of voltage-gated pot...	0.04575	0.751634575	BP
GO:1903867	extraembryonic membrane development	0.04575	0.751634575	BP
GO:1905064	negative regulation of vascular smooth m...	0.04575	0.751634575	BP
GO:0110020	regulation of actomyosin structure organ...	0.04584	0.752323774	BP
GO:0003007	heart morphogenesis	0.04591	0.752683634	BP
GO:0002695	negative regulation of leukocyte activat...	0.04604	0.753237492	BP
GO:0097529	myeloid leukocyte migration	0.04604	0.753237492	BP
GO:0021515	cell differentiation in spinal cord	0.04642	0.753241696	BP
GO:0043370	regulation of CD4-positive, alpha-beta T...	0.04642	0.753241696	BP
GO:0048701	embryonic cranial skeleton morphogenesis	0.04642	0.753241696	BP
GO:0070423	nucleotide-binding oligomerization domai...	0.04642	0.753241696	BP
GO:0071622	regulation of granulocyte chemotaxis	0.04642	0.753241696	BP
GO:1904062	regulation of cation transmembrane trans...	0.04644	0.753241696	BP
GO:0003281	ventricular septum development	0.04648	0.753241696	BP
GO:0008344	adult locomotory behavior	0.04648	0.753241696	BP
GO:0052548	regulation of endopeptidase activity	0.04666	0.753241696	BP
GO:0072511	divalent inorganic cation transport	0.04672	0.753241696	BP
GO:0001919	regulation of receptor recycling	0.04681	0.753241696	BP
GO:0003272	endocardial cushion formation	0.04681	0.753241696	BP
GO:0010831	positive regulation of myotube different...	0.04681	0.753241696	BP
GO:0021904	dorsal/ventral neural tube patterning	0.04681	0.753241696	BP
GO:0060445	branching involved in salivary gland mor...	0.04681	0.753241696	BP
GO:2000108	positive regulation of leukocyte apoptot...	0.04681	0.753241696	BP
GO:0001655	urogenital system development	0.0473	0.760345072	BP
GO:1902107	positive regulation of leukocyte differe...	0.04757	0.763901015	BP
GO:0007156	homophilic cell adhesion via plasma memb...	0.04762	0.76392043	BP
GO:0014706	striated muscle tissue development	0.04788	0.767305179	BP
GO:0002011	morphogenesis of an epithelial sheet	0.04816	0.770215649	BP
GO:0045576	mast cell activation	0.04816	0.770215649	BP
GO:0046620	regulation of organ growth	0.04835	0.772465255	BP
GO:0048519	negative regulation of biological proces...	0.04845	0.772964725	BP
GO:0051209	release of sequestered calcium ion into ...	0.04848	0.772964725	BP
GO:0043408	regulation of MAPK cascade	0.04892	0.779186612	BP
GO:0080134	regulation of response to stress	0.04931	0.784600274	BP
GO:0002718	regulation of cytokine production involv...	0.04937	0.784755517	BP

GO:0042063	gliogenesis	0.04942	0.784755517	BP
GO:0051302	regulation of cell division	0.04988	0.791257508	BP
GO:0001968	fibronectin binding	0.00000012	0.00053988	MF
GO:0005201	extracellular matrix structural constitu...	0.00000046	0.00103477	MF
GO:0001228	DNA-binding transcription activator acti...	0.0000036	0.0053988	MF
GO:0005509	calcium ion binding	0.000011	0.01237225	MF
GO:0005539	glycosaminoglycan binding	0.000048	0.0431904	MF
GO:0030021	extracellular matrix structural constitu...	0.00017	0.127471667	MF
GO:0035259	glucocorticoid receptor binding	0.00024	0.154251429	MF
GO:0019956	chemokine binding	0.00095	0.53425625	MF
GO:0008022	protein C-terminus binding	0.0014	0.66258	MF
GO:0099106	ion channel regulator activity	0.00155	0.66258	MF
GO:0015459	potassium channel regulator activity	0.00162	0.66258	MF
GO:0000987	proximal promoter sequence-specific DNA ...	0.00268	0.9470395	MF
GO:0031681	G-protein beta-subunit binding	0.0033	0.9470395	MF
GO:0000977	RNA polymerase II regulatory region sequ...	0.0033	0.9470395	MF
GO:0000978	RNA polymerase II proximal promoter sequ...	0.00371	0.9470395	MF
GO:0001221	transcription cofactor binding	0.00373	0.9470395	MF
GO:0001012	RNA polymerase II regulatory region DNA ...	0.00378	0.9470395	MF
GO:0008134	transcription factor binding	0.00388	0.9470395	MF
GO:1990837	sequence-specific double-stranded DNA bi...	0.00409	0.9470395	MF
GO:0043183	vascular endothelial growth factor recep...	0.00421	0.9470395	MF
GO:0008201	heparin binding	0.00445	0.953359524	MF
GO:1990939	ATP-dependent microtubule motor activity	0.00479	0.979555	MF
GO:0000976	transcription regulatory region sequence...	0.00594	1	MF
GO:0005102	signaling receptor binding	0.00609	1	MF
GO:0071837	HMG box domain binding	0.00715	1	MF
GO:0030545	receptor regulator activity	0.00792	1	MF
GO:0016247	channel regulator activity	0.00802	1	MF
GO:0005198	structural molecule activity	0.00809	1	MF
GO:0004971	AMPA glutamate receptor activity	0.00821	1	MF
GO:0051525	NFAT protein binding	0.00821	1	MF
GO:0046982	protein heterodimerization activity	0.00848	1	MF
GO:0048018	receptor ligand activity	0.01067	1	MF
GO:0001618	virus receptor activity	0.01138	1	MF
GO:0104005	hijacked molecular function	0.01138	1	MF
GO:0019957	C-C chemokine binding	0.01172	1	MF
GO:0001664	G protein-coupled receptor binding	0.01208	1	MF
GO:0042803	protein homodimerization activity	0.01312	1	MF
GO:0004563	beta-N-acetylhexosaminidase activity	0.01334	1	MF
GO:0044212	transcription regulatory region DNA bind...	0.01339	1	MF
GO:0050840	extracellular matrix binding	0.01352	1	MF
GO:0001067	regulatory region nucleic acid binding	0.01366	1	MF
GO:1901681	sulfur compound binding	0.01374	1	MF
GO:0003690	double-stranded DNA binding	0.015	1	MF
GO:0051019	mitogen-activated protein kinase binding	0.01583	1	MF
GO:0046983	protein dimerization activity	0.01759	1	MF
GO:0001637	G protein-coupled chemoattractant recept...	0.01763	1	MF
GO:0004950	chemokine receptor activity	0.01763	1	MF
GO:0005246	calcium channel regulator activity	0.01882	1	MF
GO:0004931	extracellularly ATP-gated cation channel...	0.01951	1	MF
GO:0008172	S-methyltransferase activity	0.01951	1	MF

GO:0008510	sodium:bicarbonate symporter activity	0.01951	1	MF
GO:0055131	C3HC4-type RING finger domain binding	0.01951	1	MF
GO:0043565	sequence-specific DNA binding	0.01998	1	MF
GO:0003774	motor activity	0.02634	1	MF
GO:0015232	heme transporter activity	0.02663	1	MF
GO:0032051	clathrin light chain binding	0.02663	1	MF
GO:0035381	ATP-gated ion channel activity	0.02663	1	MF
GO:0008569	ATP-dependent microtubule motor activity...	0.02906	1	MF
GO:0001085	RNA polymerase II transcription factor b...	0.03022	1	MF
GO:0042169	SH2 domain binding	0.03188	1	MF
GO:0030674	protein binding, bridging	0.03294	1	MF
GO:0005540	hyaluronic acid binding	0.03356	1	MF
GO:0005230	extracellular ligand-gated ion channel a...	0.03414	1	MF
GO:0031628	opioid receptor binding	0.03463	1	MF
GO:0001010	RNA polymerase II sequence-specific DNA-...	0.03798	1	MF
GO:0004044	amidophosphoribosyltransferase activity	0.03798	1	MF
GO:0004377	GDP-Man:Man3GlcNAc2-PP-Dol alpha-1,2-man	0.03798	1	MF
GO:0004790	thioether S-methyltransferase activity	0.03798	1	MF
GO:0004900	erythropoietin receptor activity	0.03798	1	MF
GO:0008115	sarcosine oxidase activity	0.03798	1	MF
GO:0008119	thiopurine S-methyltransferase activity	0.03798	1	MF
GO:0008403	25-hydroxycholecalciferol-24-hydroxylase...	0.03798	1	MF
GO:0009918	sterol delta7 reductase activity	0.03798	1	MF
GO:0015143	urate transmembrane transporter activity	0.03798	1	MF
GO:0015552	propionate transmembrane transporter act...	0.03798	1	MF
GO:0016231	beta-N-acetylglucosaminidase activity	0.03798	1	MF
GO:0019912	cyclin-dependent protein kinase activati...	0.03798	1	MF
GO:0030290	sphingolipid activator protein activity	0.03798	1	MF
GO:0030342	1-alpha,25-dihydroxyvitamin D3 24-hydrox...	0.03798	1	MF
GO:0030377	urokinase plasminogen activator receptor...	0.03798	1	MF
GO:0030748	amine N-methyltransferase activity	0.03798	1	MF
GO:0031073	cholesterol 26-hydroxylase activity	0.03798	1	MF
GO:0031249	denatured protein binding	0.03798	1	MF
GO:0031700	adrenomedullin receptor binding	0.03798	1	MF
GO:0031737	CX3C chemokine receptor binding	0.03798	1	MF
GO:0031877	somatostatin receptor binding	0.03798	1	MF
GO:0032428	beta-N-acetylgalactosaminidase activity	0.03798	1	MF
GO:0032896	palmitoyl-CoA 9-desaturase activity	0.03798	1	MF
GO:0033871	[heparan sulfate]-glucosamine 3-sulfotra...	0.03798	1	MF
GO:0036393	thiocyanate peroxidase activity	0.03798	1	MF
GO:0038147	C-X-C motif chemokine 12 receptor activi...	0.03798	1	MF
GO:0045237	CXCR1 chemokine receptor binding	0.03798	1	MF
GO:0047323	[3-methyl-2-oxobutanoate dehydrogenase (...	0.03798	1	MF
GO:0047598	7-dehydrocholesterol reductase activity	0.03798	1	MF
GO:0047749	cholestanetriol 26-monooxygenase activit...	0.03798	1	MF
GO:0050031	L-pipecolate oxidase activity	0.03798	1	MF
GO:0050683	AF-1 domain binding	0.03798	1	MF
GO:0051786	all-trans-retinol 13,14-reductase activi...	0.03798	1	MF
GO:0061632	RNA lariat debranching enzyme activator ...	0.03798	1	MF
GO:0071566	UFM1 activating enzyme activity	0.03798	1	MF
GO:0098695	inositol 1,4,5-trisphosphate receptor ac...	0.03798	1	MF
GO:0099567	calcium ion binding involved in regulati...	0.03798	1	MF

GO:0102166	[protein]-3-O-(N-acetyl-D-glucosaminyl)-...	0.03798	1	MF
GO:0102167	[protein]-3-O-(N-acetyl-D-glucosaminyl)-...	0.03798	1	MF
GO:0102571	[protein]-3-O-(N-acetyl-D-glucosaminyl)-...	0.03798	1	MF
GO:0102707	S-adenosyl-L-methionine:beta-alanine N-m...	0.03798	1	MF
GO:0120153	calcium-dependent carbohydrate binding	0.03798	1	MF
GO:1901702	salt transmembrane transporter activity	0.03798	1	MF
GO:1990147	talin binding	0.03798	1	MF
GO:1990930	RNA N1-methyladenosine dioxygenase activ...	0.03798	1	MF
GO:1902936	phosphatidylinositol bisphosphate bindin...	0.03822	1	MF
GO:0140110	transcription regulator activity	0.04084	1	MF
GO:0015276	ligand-gated ion channel activity	0.0409	1	MF
GO:0022834	ligand-gated channel activity	0.0409	1	MF
GO:0046872	metal ion binding	0.0425	1	MF
GO:0051400	BH domain binding	0.04342	1	MF
GO:0061665	SUMO ligase activity	0.04342	1	MF
GO:0001223	transcription coactivator binding	0.04358	1	MF
GO:0051184	cofactor transmembrane transporter activ...	0.04358	1	MF
GO:0000981	DNA-binding transcription factor activit...	0.04599	1	MF
GO:0017147	Wnt-protein binding	0.04644	1	MF
GO:0042379	chemokine receptor binding	0.04908	1	MF
GO:0070530	K63-linked polyubiquitin modification-de...	0.04908	1	MF
GO:0062023	collagen-containing extracellular matrix	2.8E-11	5.558E-08	CC
GO:0031012	extracellular matrix	1.3E-10	1.29025E-07	CC
GO:0005604	basement membrane	0.000002	0.001323333	CC
GO:0071953	elastic fiber	0.00023	0.1141375	CC
GO:0005796	Golgi lumen	0.00039	0.132333333	CC
GO:0005576	extracellular region	0.0004	0.132333333	CC
GO:0044421	extracellular region part	0.00049	0.136744444	CC
GO:0043202	lysosomal lumen	0.00058	0.136744444	CC
GO:0044420	extracellular matrix component	0.00062	0.136744444	CC
GO:0009986	cell surface	0.00097	0.192545	CC
GO:0072562	blood microparticle	0.00239	0.431286364	CC
GO:0042827	platelet dense granule	0.00356	0.588883333	CC
GO:0031680	G-protein beta/gamma-subunit complex	0.00409	0.622439286	CC
GO:0005833	hemoglobin complex	0.00439	0.622439286	CC
GO:0005775	vacuolar lumen	0.00531	0.70269	CC
GO:0031089	platelet dense granule lumen	0.00568	0.704675	CC
GO:0005615	extracellular space	0.00652	0.761305882	CC
GO:0035976	transcription factor AP-1 complex	0.00855	0.92822381	CC
GO:0005640	nuclear outer membrane	0.00938	0.92822381	CC
GO:0031091	platelet alpha granule	0.00979	0.92822381	CC
GO:0005614	interstitial matrix	0.00982	0.92822381	CC
GO:0071014	post-mRNA release spliceosomal complex	0.0124	0.968543103	CC
GO:0005834	heterotrimeric G-protein complex	0.01282	0.968543103	CC
GO:1905360	GTPase complex	0.01282	0.968543103	CC
GO:0042383	sarcolemma	0.01297	0.968543103	CC
GO:0005577	fibrinogen complex	0.01388	0.968543103	CC
GO:0005955	calcineurin complex	0.01388	0.968543103	CC
GO:0030896	checkpoint clamp complex	0.01388	0.968543103	CC
GO:0005730	nucleolus	0.01415	0.968543103	CC
GO:0060205	cytoplasmic vesicle lumen	0.01504	0.990639063	CC
GO:0031983	vesicle lumen	0.01555	0.990639063	CC

GO:0098982	GABA-ergic synapse	0.01597	0.990639063	CC
GO:0005788	endoplasmic reticulum lumen	0.01946	1	CC
GO:0097136	Bcl-2 family protein complex	0.0203	1	CC
GO:0098888	extrinsic component of presynaptic membr...	0.0203	1	CC
GO:0031093	platelet alpha granule lumen	0.02083	1	CC
GO:0060077	inhibitory synapse	0.02228	1	CC
GO:0098794	postsynapse	0.02442	1	CC
GO:0019898	extrinsic component of membrane	0.026	1	CC
GO:0097440	apical dendrite	0.0263	1	CC
GO:0016011	dystroglycan complex	0.02769	1	CC
GO:0098642	network-forming collagen trimer	0.02769	1	CC
GO:0098645	collagen network	0.02769	1	CC
GO:0098690	glycinergic synapse	0.02769	1	CC
GO:0098644	complex of collagen trimers	0.0354	1	CC
GO:0098651	basement membrane collagen trimer	0.03599	1	CC
GO:0034774	secretory granule lumen	0.03648	1	CC
GO:0005886	plasma membrane	0.03661	1	CC
GO:0000274	mitochondrial proton-transporting ATP sy...	0.03878	1	CC
GO:0005591	collagen type VIII trimer	0.03878	1	CC
GO:0005598	short-chain collagen trimer	0.03878	1	CC
GO:0005889	potassium:proton exchanging ATPase compl...	0.03878	1	CC
GO:0014804	terminal cisterna lumen	0.03878	1	CC
GO:0030935	sheet-forming collagen trimer	0.03878	1	CC
GO:0032996	Bcl3-Bcl10 complex	0.03878	1	CC
GO:0045265	proton-transporting ATP synthase, stator...	0.03878	1	CC
GO:0055120	striated muscle dense body	0.03878	1	CC
GO:0061474	phagolysosome membrane	0.03878	1	CC
GO:0097728	9+0 motile cilium	0.03878	1	CC
GO:0098646	collagen sheet	0.03878	1	CC
GO:0098745	Dcp1-Dcp2 complex	0.03878	1	CC
GO:0098891	extrinsic component of presynaptic activ...	0.03878	1	CC
GO:0099182	presynaptic intermediate filament cytosk...	0.03878	1	CC
GO:0120134	proximal portion of axoneme	0.03878	1	CC
GO:0060076	excitatory synapse	0.04358	1	CC
GO:0099081	supramolecular polymer	0.04439	1	CC
GO:0001527	microfibril	0.0451	1	CC
GO:0098890	extrinsic component of postsynaptic memb...	0.0451	1	CC
GO:0099080	supramolecular complex	0.04513	1	CC
GO:0031225	anchored component of membrane	0.0477	1	CC

OFC

GO.ID	Term	FET	BH	Category
GO:0006413	translational initiation	0.000	1	BP
GO:0000184	nuclear-transcribed mRNA catabolic proce...	0.001	1	BP
GO:0006614	SRP-dependent cotranslational protein ta...	0.001	1	BP
GO:0014015	positive regulation of gliogenesis	0.001	1	BP
GO:0006613	cotranslational protein targeting to mem...	0.001	1	BP
GO:0000353	formation of quadruple SL/U4/U5/U6 snRNP	0.002	1	BP
GO:0000365	mRNA trans splicing, via spliceosome	0.002	1	BP
GO:0045291	mRNA trans splicing, SL addition	0.002	1	BP
GO:0060343	trabecula formation	0.002	1	BP
GO:0060347	heart trabecula formation	0.002	1	BP
GO:0045047	protein targeting to ER	0.002	1	BP
GO:0019935	cyclic-nucleotide-mediated signaling	0.002	1	BP
GO:0007189	adenylate cyclase-activating G protein-c...	0.003	1	BP
GO:0072599	establishment of protein localization to...	0.003	1	BP
GO:0032305	positive regulation of icosanoid secreti...	0.004	1	BP
GO:0072189	ureter development	0.004	1	BP
GO:0061383	trabecula morphogenesis	0.004	1	BP
GO:0006896	Golgi to vacuole transport	0.004	1	BP
GO:0015732	prostaglandin transport	0.004	1	BP
GO:0014013	regulation of gliogenesis	0.004	1	BP
GO:0003283	atrial septum development	0.005	1	BP
GO:0032303	regulation of icosanoid secretion	0.005	1	BP
GO:2000193	positive regulation of fatty acid transp...	0.005	1	BP
GO:0042446	hormone biosynthetic process	0.006	1	BP
GO:0072197	ureter morphogenesis	0.006	1	BP
GO:0019080	viral gene expression	0.006	1	BP
GO:0071715	icosanoid transport	0.008	1	BP
GO:1901571	fatty acid derivative transport	0.008	1	BP
GO:0042359	vitamin D metabolic process	0.008	1	BP
GO:0019933	cAMP-mediated signaling	0.008	1	BP
GO:0032020	ISG15-protein conjugation	0.009	1	BP
GO:0090238	positive regulation of arachidonic acid ...	0.009	1	BP
GO:1902412	regulation of mitotic cytokinesis	0.009	1	BP
GO:1904415	regulation of xenophagy	0.009	1	BP
GO:1904417	positive regulation of xenophagy	0.009	1	BP
GO:0048709	oligodendrocyte differentiation	0.009	1	BP
GO:0070972	protein localization to endoplasmic reti...	0.009	1	BP
GO:1905523	positive regulation of macrophage migrat...	0.009	1	BP
GO:0019083	viral transcription	0.010	1	BP
GO:0002181	cytoplasmic translation	0.011	1	BP
GO:0010753	positive regulation of cGMP-mediated sig...	0.012	1	BP
GO:0048280	vesicle fusion with Golgi apparatus	0.012	1	BP
GO:1905050	positive regulation of metallopeptidase ...	0.012	1	BP
GO:1905461	positive regulation of vascular associat...	0.012	1	BP
GO:0015701	bicarbonate transport	0.013	1	BP
GO:0032371	regulation of sterol transport	0.013	1	BP
GO:0032374	regulation of cholesterol transport	0.013	1	BP
GO:0002741	positive regulation of cytokine secretio...	0.015	1	BP
GO:0032308	positive regulation of prostaglandin sec...	0.015	1	BP
GO:0042368	vitamin D biosynthetic process	0.015	1	BP

GO:0044387	negative regulation of protein kinase ac...	0.015	1	BP
GO:0090237	regulation of arachidonic acid secretion	0.015	1	BP
GO:1902389	ceramide 1-phosphate transport	0.015	1	BP
GO:0006706	steroid catabolic process	0.016	1	BP
GO:0033688	regulation of osteoblast proliferation	0.016	1	BP
GO:0045687	positive regulation of glial cell differ...	0.018	1	BP
GO:0010657	muscle cell apoptotic process	0.018	1	BP
GO:2000191	regulation of fatty acid transport	0.018	1	BP
GO:0032306	regulation of prostaglandin secretion	0.020	1	BP
GO:0033690	positive regulation of osteoblast prolif...	0.020	1	BP
GO:0051255	spindle midzone assembly	0.020	1	BP
GO:0060033	anatomical structure regression	0.020	1	BP
GO:0070561	vitamin D receptor signaling pathway	0.020	1	BP
GO:0098792	xenophagy	0.020	1	BP
GO:0045214	sarcomere organization	0.020	1	BP
GO:1905517	macrophage migration	0.020	1	BP
GO:0000244	spliceosomal tri-snRNP complex assembly	0.021	1	BP
GO:0010661	positive regulation of muscle cell apopt...	0.021	1	BP
GO:0033687	osteoblast proliferation	0.021	1	BP
GO:0048714	positive regulation of oligodendrocyte d...	0.021	1	BP
GO:0045685	regulation of glial cell differentiation	0.022	1	BP
GO:0007188	adenylate cyclase-modulating G protein-c...	0.024	1	BP
GO:0032368	regulation of lipid transport	0.024	1	BP
GO:0002674	negative regulation of acute inflammator...	0.024	1	BP
GO:0048484	enteric nervous system development	0.024	1	BP
GO:0048672	positive regulation of collateral sprout...	0.024	1	BP
GO:0060579	ventral spinal cord interneuron fate com...	0.024	1	BP
GO:0060581	cell fate commitment involved in pattern...	0.024	1	BP
GO:1905288	vascular associated smooth muscle cell a...	0.024	1	BP
GO:1905459	regulation of vascular associated smooth...	0.024	1	BP
GO:0001315	age-dependent response to reactive oxyge...	0.025	1	BP
GO:0001797	negative regulation of type IIa hypersen...	0.025	1	BP
GO:0001811	negative regulation of type I hypersensi...	0.025	1	BP
GO:0001814	negative regulation of antibody-dependen...	0.025	1	BP
GO:0002041	intussusceptive angiogenesis	0.025	1	BP
GO:0002436	immune complex clearance by monocytes an...	0.025	1	BP
GO:0002450	B cell antigen processing and presentati...	0.025	1	BP
GO:0002622	regulation of B cell antigen processing ...	0.025	1	BP
GO:0002893	negative regulation of type II hypersens...	0.025	1	BP
GO:0003068	regulation of systemic arterial blood pr...	0.025	1	BP
GO:0003069	acetylcholine-mediated vasodilation invo...	0.025	1	BP
GO:0003070	regulation of systemic arterial blood pr...	0.025	1	BP
GO:0003285	septum secundum development	0.025	1	BP
GO:0003290	atrial septum secundum morphogenesis	0.025	1	BP
GO:0003327	type B pancreatic cell fate commitment	0.025	1	BP
GO:0006430	lysyl-tRNA aminoacylation	0.025	1	BP
GO:0016104	triterpenoid biosynthetic process	0.025	1	BP
GO:0019988	charged-tRNA amino acid modification	0.025	1	BP
GO:0021560	abducens nerve development	0.025	1	BP
GO:0021572	rhombomere 6 development	0.025	1	BP
GO:0021598	abducens nerve morphogenesis	0.025	1	BP
GO:0021599	abducens nerve formation	0.025	1	BP

GO:0035499	carnosine biosynthetic process	0.025	1	BP
GO:0036046	protein demalonylation	0.025	1	BP
GO:0036047	peptidyl-lysine demalonylation	0.025	1	BP
GO:0036048	protein desuccinylation	0.025	1	BP
GO:0036049	peptidyl-lysine desuccinylation	0.025	1	BP
GO:0038183	bile acid signaling pathway	0.025	1	BP
GO:0045234	protein palmitoleylation	0.025	1	BP
GO:0060580	ventral spinal cord interneuron fate det...	0.025	1	BP
GO:0060582	cell fate determination involved in patt...	0.025	1	BP
GO:0060682	primary ureteric bud growth	0.025	1	BP
GO:0060694	regulation of cholesterol transporter ac...	0.025	1	BP
GO:0060989	lipid tube assembly involved in organell...	0.025	1	BP
GO:0061698	protein deglutarylation	0.025	1	BP
GO:0061699	peptidyl-lysine deglutarylation	0.025	1	BP
GO:0071206	establishment of protein localization to...	0.025	1	BP
GO:0071908	determination of intestine left/right as...	0.025	1	BP
GO:0071909	determination of stomach left/right asym...	0.025	1	BP
GO:0071951	conversion of methionyl-tRNA to N-formyl...	0.025	1	BP
GO:0072343	pancreatic stellate cell proliferation	0.025	1	BP
GO:0090264	regulation of immune complex clearance b...	0.025	1	BP
GO:0099148	regulation of synaptic vesicle docking	0.025	1	BP
GO:1900369	negative regulation of RNA interference	0.025	1	BP
GO:1902167	positive regulation of intrinsic apoptot...	0.025	1	BP
GO:1903814	regulation of collecting lymphatic vesse...	0.025	1	BP
GO:1903815	negative regulation of collecting lympha...	0.025	1	BP
GO:1904803	regulation of translation involved in ce...	0.025	1	BP
GO:1904823	purine nucleobase transmembrane transpor...	0.025	1	BP
GO:1905917	positive regulation of cell differentiat...	0.025	1	BP
GO:1905932	positive regulation of vascular smooth m...	0.025	1	BP
GO:1990092	calcium-dependent self proteolysis	0.025	1	BP
GO:1990183	lymphatic vascular process in circulator...	0.025	1	BP
GO:1990186	regulation of lymphatic vessel size	0.025	1	BP
GO:1990192	collecting lymphatic vessel constriction	0.025	1	BP
GO:1990227	paranodal junction maintenance	0.025	1	BP
GO:1990261	pre-mRNA catabolic process	0.025	1	BP
GO:2000167	regulation of planar cell polarity pathw...	0.025	1	BP
GO:2000229	regulation of pancreatic stellate cell p...	0.025	1	BP
GO:2000230	negative regulation of pancreatic stella...	0.025	1	BP
GO:2000422	regulation of eosinophil chemotaxis	0.025	1	BP
GO:2000424	positive regulation of eosinophil chemot...	0.025	1	BP
GO:2000656	regulation of apolipoprotein binding	0.025	1	BP
GO:2000657	negative regulation of apolipoprotein bi...	0.025	1	BP
GO:2001136	negative regulation of endocytic recycli...	0.025	1	BP
GO:0003230	cardiac atrium development	0.025	1	BP
GO:0033555	multicellular organismal response to str...	0.026	1	BP
GO:0003006	developmental process involved in reprod...	0.026	1	BP
GO:0008217	regulation of blood pressure	0.027	1	BP
GO:0035270	endocrine system development	0.028	1	BP
GO:0000281	mitotic cytokinesis	0.028	1	BP
GO:0006953	acute-phase response	0.028	1	BP
GO:0071295	cellular response to vitamin	0.028	1	BP
GO:0042063	gliogenesis	0.028	1	BP

GO:0043392	negative regulation of DNA binding	0.028	1	BP
GO:0032310	prostaglandin secretion	0.029	1	BP
GO:0042362	fat-soluble vitamin biosynthetic process	0.029	1	BP
GO:0060413	atrial septum morphogenesis	0.029	1	BP
GO:0071621	granulocyte chemotaxis	0.029	1	BP
GO:0000956	nuclear-transcribed mRNA catabolic proce...	0.029	1	BP
GO:0061458	reproductive system development	0.030	1	BP
GO:0030199	collagen fibril organization	0.030	1	BP
GO:0032309	icosanoid secretion	0.031	1	BP
GO:0032892	positive regulation of organic acid tran...	0.031	1	BP
GO:1900181	negative regulation of protein localizat...	0.031	1	BP
GO:0042594	response to starvation	0.032	1	BP
GO:0001890	placenta development	0.033	1	BP
GO:0021696	cerebellar cortex morphogenesis	0.034	1	BP
GO:0061384	heart trabecula morphogenesis	0.034	1	BP
GO:1902742	apoptotic process involved in developmen...	0.034	1	BP
GO:0002739	regulation of cytokine secretion involve...	0.034	1	BP
GO:0010752	regulation of cGMP-mediated signaling	0.034	1	BP
GO:0021514	ventral spinal cord interneuron differen...	0.034	1	BP
GO:0048266	behavioral response to pain	0.034	1	BP
GO:0060009	Sertoli cell development	0.034	1	BP
GO:0090161	Golgi ribbon formation	0.034	1	BP
GO:0003073	regulation of systemic arterial blood pr...	0.034	1	BP
GO:0050911	detection of chemical stimulus involved ...	0.036	1	BP
GO:0014065	phosphatidylinositol 3-kinase signaling	0.037	1	BP
GO:0048246	macrophage chemotaxis	0.037	1	BP
GO:0006892	post-Golgi vesicle-mediated transport	0.037	1	BP
GO:0016553	base conversion or substitution editing	0.040	1	BP
GO:0021781	glial cell fate commitment	0.040	1	BP
GO:0034393	positive regulation of smooth muscle cel...	0.040	1	BP
GO:0035627	ceramide transport	0.040	1	BP
GO:0042448	progesterone metabolic process	0.040	1	BP
GO:0060850	regulation of transcription involved in ...	0.040	1	BP
GO:0061430	bone trabecula morphogenesis	0.040	1	BP
GO:1905048	regulation of metallopeptidase activity	0.040	1	BP
GO:2001212	regulation of vasculogenesis	0.040	1	BP
GO:0042116	macrophage activation	0.040	1	BP
GO:0007507	heart development	0.040	1	BP
GO:0030049	muscle filament sliding	0.040	1	BP
GO:0033275	actin-myosin filament sliding	0.040	1	BP
GO:1905521	regulation of macrophage migration	0.040	1	BP
GO:0002532	production of molecular mediator involve...	0.041	1	BP
GO:0071385	cellular response to glucocorticoid stim...	0.041	1	BP
GO:0015908	fatty acid transport	0.042	1	BP
GO:0090559	regulation of membrane permeability	0.042	1	BP
GO:0031668	cellular response to extracellular stimu...	0.042	1	BP
GO:0007030	Golgi organization	0.042	1	BP
GO:0043524	negative regulation of neuron apoptotic ...	0.042	1	BP
GO:0048015	phosphatidylinositol-mediated signaling	0.042	1	BP
GO:0007187	G protein-coupled receptor signaling pat...	0.043	1	BP
GO:0006891	intra-Golgi vesicle-mediated transport	0.044	1	BP
GO:0010659	cardiac muscle cell apoptotic process	0.044	1	BP

GO:0071868	cellular response to monoamine stimulus	0.044	1	BP
GO:0071870	cellular response to catecholamine stimu...	0.044	1	BP
GO:0014066	regulation of phosphatidylinositol 3-kin...	0.044	1	BP
GO:0002031	G protein-coupled receptor internalizati...	0.045	1	BP
GO:0010759	positive regulation of macrophage chemot...	0.045	1	BP
GO:0036158	outer dynein arm assembly	0.045	1	BP
GO:0071496	cellular response to external stimulus	0.045	1	BP
GO:0007411	axon guidance	0.046	1	BP
GO:0048017	inositol lipid-mediated signaling	0.046	1	BP
GO:0001836	release of cytochrome c from mitochondri...	0.047	1	BP
GO:0008207	C21-steroid hormone metabolic process	0.047	1	BP
GO:0021545	cranial nerve development	0.047	1	BP
GO:1901030	positive regulation of mitochondrial out...	0.047	1	BP
GO:0097485	neuron projection guidance	0.048	1	BP
GO:0033273	response to vitamin	0.048	1	BP
GO:0001306	age-dependent response to oxidative stre...	0.049	1	BP
GO:0001788	antibody-dependent cellular cytotoxicity	0.049	1	BP
GO:0001813	regulation of antibody-dependent cellula...	0.049	1	BP
GO:0001834	trophectodermal cell proliferation	0.049	1	BP
GO:0001920	negative regulation of receptor recyclin...	0.049	1	BP
GO:0002249	lymphocyte anergy	0.049	1	BP
GO:0002276	basophil activation involved in immune r...	0.049	1	BP
GO:0002316	follicular B cell differentiation	0.049	1	BP
GO:0002434	immune complex clearance	0.049	1	BP
GO:0002667	regulation of T cell anergy	0.049	1	BP
GO:0002728	negative regulation of natural killer ce...	0.049	1	BP
GO:0002870	T cell anergy	0.049	1	BP
GO:0002884	negative regulation of hypersensitivity	0.049	1	BP
GO:0002911	regulation of lymphocyte anergy	0.049	1	BP
GO:0002946	tRNA C5-cytosine methylation	0.049	1	BP
GO:0003312	pancreatic PP cell differentiation	0.049	1	BP
GO:0003326	pancreatic A cell fate commitment	0.049	1	BP
GO:0003329	pancreatic PP cell fate commitment	0.049	1	BP
GO:0006436	tryptophanyl-tRNA aminoacylation	0.049	1	BP
GO:0006481	C-terminal protein methylation	0.049	1	BP
GO:0006597	spermine biosynthetic process	0.049	1	BP
GO:0006722	triterpenoid metabolic process	0.049	1	BP
GO:0006780	uroporphyrinogen III biosynthetic proces...	0.049	1	BP
GO:0007354	zygotic determination of anterior/poster...	0.049	1	BP
GO:0007506	gonadal mesoderm development	0.049	1	BP
GO:0007571	age-dependent general metabolic decline	0.049	1	BP
GO:0009159	deoxyribonucleoside monophosphate catabo...	0.049	1	BP
GO:0009386	translational attenuation	0.049	1	BP
GO:0010593	negative regulation of lamellipodium ass...	0.049	1	BP
GO:0010693	negative regulation of alkaline phosphat...	0.049	1	BP
GO:0014717	regulation of satellite cell activation ...	0.049	1	BP
GO:0014718	positive regulation of satellite cell ac...	0.049	1	BP
GO:0015855	pyrimidine nucleobase transport	0.049	1	BP
GO:0019075	virus maturation	0.049	1	BP
GO:0021546	rhombomere development	0.049	1	BP
GO:0021571	rhombomere 5 development	0.049	1	BP
GO:0021603	cranial nerve formation	0.049	1	BP

GO:0021912	regulation of transcription from RNA pol...	0.049	1	BP
GO:0021913	regulation of transcription from RNA pol...	0.049	1	BP
GO:0032902	nerve growth factor production	0.049	1	BP
GO:0032971	regulation of muscle filament sliding	0.049	1	BP
GO:0033566	gamma-tubulin complex localization	0.049	1	BP
GO:0034196	acylglycerol transport	0.049	1	BP
GO:0034197	triglyceride transport	0.049	1	BP
GO:0035283	central nervous system segmentation	0.049	1	BP
GO:0035284	brain segmentation	0.049	1	BP
GO:0035498	carnosine metabolic process	0.049	1	BP
GO:0036304	umbilical cord morphogenesis	0.049	1	BP
GO:0043317	regulation of cytotoxic T cell degranula...	0.049	1	BP
GO:0043318	negative regulation of cytotoxic T cell ...	0.049	1	BP
GO:0045575	basophil activation	0.049	1	BP
GO:0046502	uroporphyrinogen III metabolic process	0.049	1	BP
GO:0051977	lysophospholipid transport	0.049	1	BP
GO:0060032	notochord regression	0.049	1	BP
GO:0060057	apoptotic process involved in mammary gl...	0.049	1	BP
GO:0060058	positive regulation of apoptotic process...	0.049	1	BP
GO:0060177	regulation of angiotensin metabolic proc...	0.049	1	BP
GO:0061027	umbilical cord development	0.049	1	BP
GO:0070315	G1 to G0 transition involved in cell dif...	0.049	1	BP
GO:0070541	response to platinum ion	0.049	1	BP
GO:0071306	cellular response to vitamin E	0.049	1	BP
GO:0071418	cellular response to amine stimulus	0.049	1	BP
GO:0090289	regulation of osteoclast proliferation	0.049	1	BP
GO:0090291	negative regulation of osteoclast prolif...	0.049	1	BP
GO:0110026	regulation of DNA strand resection invol...	0.049	1	BP
GO:0110112	regulation of lipid transporter activity	0.049	1	BP
GO:1900154	regulation of bone trabecula formation	0.049	1	BP
GO:1900155	negative regulation of bone trabecula fo...	0.049	1	BP
GO:1900169	regulation of glucocorticoid mediated si...	0.049	1	BP
GO:1900220	semaphorin-plexin signaling pathway invo...	0.049	1	BP
GO:1901558	response to metformin	0.049	1	BP
GO:1902162	regulation of DNA damage response, signa...	0.049	1	BP
GO:1902164	positive regulation of DNA damage respon...	0.049	1	BP
GO:1902283	negative regulation of primary amine oxi...	0.049	1	BP
GO:1902528	regulation of protein linear polyubiquit...	0.049	1	BP
GO:1902530	positive regulation of protein linear po...	0.049	1	BP
GO:1903519	regulation of mammary gland involution	0.049	1	BP
GO:1903521	positive regulation of mammary gland inv...	0.049	1	BP
GO:1903939	regulation of TORC2 signaling	0.049	1	BP
GO:1904027	negative regulation of collagen fibril o...	0.049	1	BP
GO:1904515	positive regulation of TORC2 signaling	0.049	1	BP
GO:1904700	granulosa cell apoptotic process	0.049	1	BP
GO:1904708	regulation of granulosa cell apoptotic p...	0.049	1	BP
GO:1905704	positive regulation of inhibitory synaps...	0.049	1	BP
GO:1990443	peptidyl-threonine autophosphorylation	0.049	1	BP
GO:2000184	positive regulation of progesterone bios...	0.049	1	BP
GO:2000355	negative regulation of ovarian follicle ...	0.049	1	BP
GO:2000418	positive regulation of eosinophil migrat...	0.049	1	BP
GO:0071384	cellular response to corticosteroid stim...	0.049	1	BP

GO:2001244	positive regulation of intrinsic apoptot...	0.049	1	BP
GO:0050907	detection of chemical stimulus involved ...	0.049	1	BP
GO:0031386	protein tag	0.002	1	MF
GO:0042296	ISG15 transferase activity	0.003	1	MF
GO:0005484	SNAP receptor activity	0.005	1	MF
GO:0016881	acid-amino acid ligase activity	0.005	1	MF
GO:0046965	retinoid X receptor binding	0.005	1	MF
GO:0051393	alpha-actinin binding	0.006	1	MF
GO:0016799	hydrolase activity, hydrolyzing N-glycos...	0.012	1	MF
GO:0003735	structural constituent of ribosome	0.014	1	MF
GO:0042805	actinin binding	0.014	1	MF
GO:1902388	ceramide 1-phosphate transporter activit...	0.015	1	MF
GO:0042974	retinoic acid receptor binding	0.017	1	MF
GO:0019864	IgG binding	0.019	1	MF
GO:0016879	ligase activity, forming carbon-nitrogen...	0.020	1	MF
GO:0030621	U4 snRNA binding	0.023	1	MF
GO:0035374	chondroitin sulfate binding	0.023	1	MF
GO:0035620	ceramide transporter activity	0.023	1	MF
GO:0000250	lanosterol synthase activity	0.024	1	MF
GO:0002953	5'-deoxynucleotidase activity	0.024	1	MF
GO:0003877	ATP adenylyltransferase activity	0.024	1	MF
GO:0003952	NAD+ synthase (glutamine-hydrolyzing) ac...	0.024	1	MF
GO:0004152	dihydroorotate dehydrogenase activity	0.024	1	MF
GO:0004441	inositol-1,4-bisphosphate 1-phosphatase ...	0.024	1	MF
GO:0004479	methionyl-tRNA formyltransferase activit...	0.024	1	MF
GO:0004632	phosphopantothenate--cysteine ligase act...	0.024	1	MF
GO:0004824	lysine-tRNA ligase activity	0.024	1	MF
GO:0004852	uroporphyrinogen-III synthase activity	0.024	1	MF
GO:0005275	amine transmembrane transporter activity	0.024	1	MF
GO:0005350	pyrimidine nucleobase transmembrane tran...	0.024	1	MF
GO:0005519	cytoskeletal regulatory protein binding	0.024	1	MF
GO:0008403	25-hydroxycholecalciferol-24-hydroxylase...	0.024	1	MF
GO:0016768	spermine synthase activity	0.024	1	MF
GO:0018423	protein C-terminal leucine carboxyl O-me...	0.024	1	MF
GO:0019770	IgG receptor activity	0.024	1	MF
GO:0019772	low-affinity IgG receptor activity	0.024	1	MF
GO:0030342	1-alpha,25-dihydroxyvitamin D3 24-hydrox...	0.024	1	MF
GO:0031073	cholesterol 26-hydroxylase activity	0.024	1	MF
GO:0031370	eukaryotic initiation factor 4G binding	0.024	1	MF
GO:0031559	oxidosqualene cyclase activity	0.024	1	MF
GO:0031700	adrenomedullin receptor binding	0.024	1	MF
GO:0036054	protein-malonyllysine demalonylase activ...	0.024	1	MF
GO:0036055	protein-succinyllysine desuccinylase act...	0.024	1	MF
GO:0038181	bile acid receptor activity	0.024	1	MF
GO:0038186	lithocholic acid receptor activity	0.024	1	MF
GO:0042300	beta-amyrin synthase activity	0.024	1	MF
GO:0047730	carnosine synthase activity	0.024	1	MF
GO:0047749	cholestanetriol 26-monooxygenase activit...	0.024	1	MF
GO:0047992	hydroxylysine kinase activity	0.024	1	MF
GO:0050254	rhodopsin kinase activity	0.024	1	MF
GO:0055103	ligase regulator activity	0.024	1	MF
GO:0061654	NEDD8 conjugating enzyme activity	0.024	1	MF

GO:0061697	protein-glutaryllysine deglutarylase act...	0.024	1	MF
GO:0070694	deoxyribonucleoside 5'-monophosphate N-g...	0.024	1	MF
GO:0102102	homocarnosine synthase activity	0.024	1	MF
GO:0120014	intermembrane phospholipid transfer acti...	0.024	1	MF
GO:1902098	calcitriol binding	0.024	1	MF
GO:1902121	lithocholic acid binding	0.024	1	MF
GO:1902271	D3 vitamins binding	0.024	1	MF
GO:0098632	cell-cell adhesion mediator activity	0.026	1	MF
GO:0000340	RNA 7-methylguanosine cap binding	0.033	1	MF
GO:0031432	titin binding	0.033	1	MF
GO:0046624	sphingolipid transporter activity	0.033	1	MF
GO:0004984	olfactory receptor activity	0.033	1	MF
GO:0004175	endopeptidase activity	0.039	1	MF
GO:0038024	cargo receptor activity	0.040	1	MF
GO:0010181	FMN binding	0.044	1	MF
GO:0019865	immunoglobulin binding	0.044	1	MF
GO:0070403	NAD+ binding	0.044	1	MF
GO:0045296	cadherin binding	0.044	1	MF
GO:0098631	cell adhesion mediator activity	0.046	1	MF
GO:0003865	3-oxo-5-alpha-steroid 4-dehydrogenase ac...	0.048	1	MF
GO:0003880	protein C-terminal carboxyl O-methyltran...	0.048	1	MF
GO:0004360	glutamine-fructose-6-phosphate transamin...	0.048	1	MF
GO:0004572	mannosyl-oligosaccharide 1,3-1,6-alpha-m...	0.048	1	MF
GO:0004830	tryptophan-tRNA ligase activity	0.048	1	MF
GO:0004873	asialoglycoprotein receptor activity	0.048	1	MF
GO:0004980	melanocyte-stimulating hormone receptor ...	0.048	1	MF
GO:0004998	transferrin receptor activity	0.048	1	MF
GO:0008294	calcium- and calmodulin-responsive adeny...	0.048	1	MF
GO:0016262	protein N-acetylglucosaminyltransferase ...	0.048	1	MF
GO:0016312	inositol bisphosphate phosphatase activi...	0.048	1	MF
GO:0017089	glycolipid transporter activity	0.048	1	MF
GO:0031720	haptoglobin binding	0.048	1	MF
GO:0033765	steroid dehydrogenase activity, acting o...	0.048	1	MF
GO:0038131	neuregulin receptor activity	0.048	1	MF
GO:0047017	prostaglandin-F synthase activity	0.048	1	MF
GO:0047751	cholestenone 5-alpha-reductase activity	0.048	1	MF
GO:0052593	tryptamine:oxygen oxidoreductase (deamin...	0.048	1	MF
GO:0052594	aminoacetone:oxygen oxidoreductase(deami...	0.048	1	MF
GO:0052595	aliphatic-amine oxidase activity	0.048	1	MF
GO:0052596	phenethylamine:oxygen oxidoreductase (de...	0.048	1	MF
GO:0098633	collagen fibril binding	0.048	1	MF
GO:1990698	palmitoleoyltransferase activity	0.048	1	MF
GO:0022626	cytosolic ribosome	0.000	0.5161	CC
GO:0022625	cytosolic large ribosomal subunit	0.001	0.585575	CC
GO:0042788	polysomal ribosome	0.001	0.681517	CC
GO:0044445	cytosolic part	0.001	0.699713	CC
GO:0044391	ribosomal subunit	0.006	1	CC
GO:0036157	outer dynein arm	0.006	1	CC
GO:0005840	ribosome	0.010	1	CC
GO:0000137	Golgi cis cisterna	0.011	1	CC
GO:0005801	cis-Golgi network	0.012	1	CC
GO:0005753	mitochondrial proton-transporting ATP sy...	0.014	1	CC

GO:0045259	proton-transporting ATP synthase complex	0.016	1 CC
GO:0001917	photoreceptor inner segment	0.016	1 CC
GO:0022627	cytosolic small ribosomal subunit	0.018	1 CC
GO:0016323	basolateral plasma membrane	0.021	1 CC
GO:0031201	SNARE complex	0.021	1 CC
GO:0005776	autophagosome	0.023	1 CC
GO:0005751	mitochondrial respiratory chain complex ...	0.024	1 CC
GO:0032311	angiogenin-PRI complex	0.025	1 CC
GO:0034423	autophagosome lumen	0.025	1 CC
GO:0097733	photoreceptor cell cilium	0.025	1 CC
GO:0015934	large ribosomal subunit	0.026	1 CC
GO:0097731	9+0 non-motile cilium	0.027	1 CC
GO:0015935	small ribosomal subunit	0.027	1 CC
GO:0005844	polysome	0.031	1 CC
GO:0005688	U6 snRNP	0.034	1 CC
GO:0005845	mRNA cap binding complex	0.034	1 CC
GO:0016281	eukaryotic translation initiation factor...	0.034	1 CC
GO:0097504	Gemini of coiled bodies	0.034	1 CC
GO:0099513	polymeric cytoskeletal fiber	0.036	1 CC
GO:0010369	chromocenter	0.039	1 CC
GO:0042622	photoreceptor outer segment membrane	0.039	1 CC
GO:0097381	photoreceptor disc membrane	0.039	1 CC
GO:0009925	basal plasma membrane	0.043	1 CC
GO:0005884	actin filament	0.044	1 CC
GO:0005858	axonemal dynein complex	0.045	1 CC
GO:0034518	RNA cap binding complex	0.045	1 CC
GO:0000308	cytoplasmic cyclin-dependent protein kin...	0.049	1 CC
GO:0005954	calcium- and calmodulin-dependent protei...	0.049	1 CC
GO:0033106	cis-Golgi network membrane	0.049	1 CC
GO:0097232	lamellar body membrane	0.049	1 CC
GO:0097233	alveolar lamellar body membrane	0.049	1 CC

vmPFC

GO.ID	Term	FET	BH	Category
GO:0051384	response to glucocorticoid	0.000	0.41752	BP
GO:0000353	formation of quadruple SL/U4/U5/U6 snRNP	0.000	0.41752	BP
GO:0000365	mRNA trans splicing, via spliceosome	0.000	0.41752	BP
GO:0045291	mRNA trans splicing, SL addition	0.000	0.41752	BP
GO:0071385	cellular response to glucocorticoid stim...	0.000	0.41752	BP
GO:0031960	response to corticosteroid	0.000	0.41752	BP
GO:0033619	membrane protein proteolysis	0.000	0.424976	BP
GO:0071384	cellular response to corticosteroid stim...	0.000	0.450139	BP
GO:0000387	spliceosomal snRNP assembly	0.000	0.661073	BP
GO:0007218	neuropeptide signaling pathway	0.001	1	BP
GO:0032088	negative regulation of NF-kappaB transcr...	0.001	1	BP
GO:0009719	response to endogenous stimulus	0.001	1	BP
GO:0007156	homophilic cell adhesion via plasma memb...	0.001	1	BP
GO:0021778	oligodendrocyte cell fate specification	0.002	1	BP
GO:0021779	oligodendrocyte cell fate commitment	0.002	1	BP
GO:0021780	glial cell fate specification	0.002	1	BP
GO:0000244	spliceosomal tri-snRNP complex assembly	0.002	1	BP
GO:1990778	protein localization to cell periphery	0.002	1	BP
GO:0031102	neuron projection regeneration	0.002	1	BP
GO:0071294	cellular response to zinc ion	0.003	1	BP
GO:0060441	epithelial tube branching involved in lu...	0.003	1	BP
GO:0031103	axon regeneration	0.003	1	BP
GO:0043967	histone H4 acetylation	0.003	1	BP
GO:0098742	cell-cell adhesion via plasma-membrane a...	0.003	1	BP
GO:0009725	response to hormone	0.003	1	BP
GO:1901388	regulation of transforming growth factor...	0.003	1	BP
GO:0003358	noradrenergic neuron development	0.003	1	BP
GO:0061104	adrenal chromaffin cell differentiation	0.003	1	BP
GO:0070253	somatostatin secretion	0.003	1	BP
GO:1902396	protein localization to bicellular tight...	0.003	1	BP
GO:0003012	muscle system process	0.003	1	BP
GO:0051969	regulation of transmission of nerve impu...	0.003	1	BP
GO:0008344	adult locomotory behavior	0.004	1	BP
GO:0045833	negative regulation of lipid metabolic p...	0.004	1	BP
GO:0014044	Schwann cell development	0.004	1	BP
GO:0043984	histone H4-K16 acetylation	0.004	1	BP
GO:0098609	cell-cell adhesion	0.004	1	BP
GO:0043584	nose development	0.005	1	BP
GO:0071396	cellular response to lipid	0.005	1	BP
GO:0042981	regulation of apoptotic process	0.005	1	BP
GO:0060449	bud elongation involved in lung branchin...	0.005	1	BP
GO:0060710	chorio-allantoic fusion	0.005	1	BP
GO:0006576	cellular biogenic amine metabolic proces...	0.005	1	BP
GO:0006006	glucose metabolic process	0.006	1	BP
GO:1901889	negative regulation of cell junction ass...	0.006	1	BP
GO:0043067	regulation of programmed cell death	0.007	1	BP
GO:0032526	response to retinoic acid	0.007	1	BP
GO:0021954	central nervous system neuron developmen...	0.007	1	BP
GO:1901652	response to peptide	0.007	1	BP
GO:0034329	cell junction assembly	0.007	1	BP

GO:0033993	response to lipid	0.007	1 BP
GO:0006626	protein targeting to mitochondrion	0.008	1 BP
GO:0007435	salivary gland morphogenesis	0.008	1 BP
GO:0000715	nucleotide-excision repair, DNA damage r...	0.008	1 BP
GO:0006094	gluconeogenesis	0.008	1 BP
GO:0009158	ribonucleoside monophosphate catabolic p...	0.008	1 BP
GO:0009169	purine ribonucleoside monophosphate cata...	0.008	1 BP
GO:0036363	transforming growth factor beta activati...	0.008	1 BP
GO:2000252	negative regulation of feeding behavior	0.008	1 BP
GO:0016051	carbohydrate biosynthetic process	0.009	1 BP
GO:0044706	multi-multicellular organism process	0.009	1 BP
GO:0007423	sensory organ development	0.009	1 BP
GO:0032870	cellular response to hormone stimulus	0.009	1 BP
GO:0002188	translation reinitiation	0.009	1 BP
GO:0006208	pyrimidine nucleobase catabolic process	0.009	1 BP
GO:0006210	thymine catabolic process	0.009	1 BP
GO:0019859	thymine metabolic process	0.009	1 BP
GO:0060164	regulation of timing of neuron different...	0.009	1 BP
GO:0061091	regulation of phospholipid translocation	0.009	1 BP
GO:0061092	positive regulation of phospholipid tran...	0.009	1 BP
GO:1902722	positive regulation of prolactin secreti...	0.009	1 BP
GO:2000619	negative regulation of histone H4-K16 ac...	0.009	1 BP
GO:0005996	monosaccharide metabolic process	0.010	1 BP
GO:0019319	hexose biosynthetic process	0.010	1 BP
GO:0061001	regulation of dendritic spine morphogene...	0.010	1 BP
GO:0010907	positive regulation of glucose metabolic...	0.010	1 BP
GO:0019318	hexose metabolic process	0.011	1 BP
GO:0048678	response to axon injury	0.011	1 BP
GO:0006839	mitochondrial transport	0.011	1 BP
GO:0007565	female pregnancy	0.011	1 BP
GO:0022011	myelination in peripheral nervous system	0.011	1 BP
GO:0032292	peripheral nervous system axon ensheathm...	0.011	1 BP
GO:0046856	phosphatidylinositol dephosphorylation	0.011	1 BP
GO:0000492	box C/D snoRNP assembly	0.011	1 BP
GO:0002862	negative regulation of inflammatory resp...	0.011	1 BP
GO:0009128	purine nucleoside monophosphate cataboli...	0.011	1 BP
GO:0014877	response to muscle inactivity involved i...	0.011	1 BP
GO:0014894	response to denervation involved in regu...	0.011	1 BP
GO:0030656	regulation of vitamin metabolic process	0.011	1 BP
GO:0051764	actin crosslink formation	0.011	1 BP
GO:0090192	regulation of glomerulus development	0.011	1 BP
GO:2000121	regulation of removal of superoxide radi...	0.011	1 BP
GO:0051017	actin filament bundle assembly	0.012	1 BP
GO:0006369	termination of RNA polymerase II transcr...	0.012	1 BP
GO:0007431	salivary gland development	0.012	1 BP
GO:0031099	regeneration	0.012	1 BP
GO:0050793	regulation of developmental process	0.012	1 BP
GO:0006939	smooth muscle contraction	0.012	1 BP
GO:0043065	positive regulation of apoptotic process	0.012	1 BP
GO:0006936	muscle contraction	0.013	1 BP
GO:0021513	spinal cord dorsal/ventral patterning	0.013	1 BP
GO:1901654	response to ketone	0.013	1 BP

GO:0021782	glial cell development	0.013	1 BP
GO:0071495	cellular response to endogenous stimulus	0.014	1 BP
GO:0014037	Schwann cell differentiation	0.014	1 BP
GO:0043433	negative regulation of DNA-binding trans...	0.014	1 BP
GO:0061572	actin filament bundle organization	0.014	1 BP
GO:0043068	positive regulation of programmed cell d...	0.014	1 BP
GO:0048545	response to steroid hormone	0.014	1 BP
GO:0043434	response to peptide hormone	0.014	1 BP
GO:0021885	forebrain cell migration	0.015	1 BP
GO:1904377	positive regulation of protein localizat...	0.015	1 BP
GO:0090101	negative regulation of transmembrane rec...	0.015	1 BP
GO:0097305	response to alcohol	0.015	1 BP
GO:0000338	protein deneddylation	0.016	1 BP
GO:0007016	cytoskeletal anchoring at plasma membran...	0.016	1 BP
GO:0014870	response to muscle inactivity	0.016	1 BP
GO:0060579	ventral spinal cord interneuron fate com...	0.016	1 BP
GO:0060581	cell fate commitment involved in pattern...	0.016	1 BP
GO:0045930	negative regulation of mitotic cell cycl...	0.016	1 BP
GO:0048665	neuron fate specification	0.016	1 BP
GO:0051895	negative regulation of focal adhesion as...	0.016	1 BP
GO:0050728	negative regulation of inflammatory resp...	0.016	1 BP
GO:0060997	dendritic spine morphogenesis	0.016	1 BP
GO:0007155	cell adhesion	0.016	1 BP
GO:0046364	monosaccharide biosynthetic process	0.017	1 BP
GO:0003357	noradrenergic neuron differentiation	0.017	1 BP
GO:0006710	androgen catabolic process	0.017	1 BP
GO:0015811	L-cystine transport	0.017	1 BP
GO:0015961	diadenosine polyphosphate catabolic proc...	0.017	1 BP
GO:0050748	negative regulation of lipoprotein metab...	0.017	1 BP
GO:0051971	positive regulation of transmission of n...	0.017	1 BP
GO:0060431	primary lung bud formation	0.017	1 BP
GO:0060455	negative regulation of gastric acid secr...	0.017	1 BP
GO:0071543	diphosphoinositol polyphosphate metaboli...	0.017	1 BP
GO:1901906	diadenosine pentaphosphate metabolic pro...	0.017	1 BP
GO:1901907	diadenosine pentaphosphate catabolic pro...	0.017	1 BP
GO:1901908	diadenosine hexaphosphate metabolic proc...	0.017	1 BP
GO:1901909	diadenosine hexaphosphate catabolic proc...	0.017	1 BP
GO:1901910	adenosine 5'-(hexahydrogen pentaphosphat...	0.017	1 BP
GO:1901911	adenosine 5'-(hexahydrogen pentaphosphat...	0.017	1 BP
GO:0022610	biological adhesion	0.018	1 BP
GO:0035051	cardiocyte differentiation	0.018	1 BP
GO:0072659	protein localization to plasma membrane	0.018	1 BP
GO:0072655	establishment of protein localization to...	0.018	1 BP
GO:0006509	membrane protein ectodomain proteolysis	0.018	1 BP
GO:0035019	somatic stem cell population maintenance	0.018	1 BP
GO:0051443	positive regulation of ubiquitin-protein...	0.018	1 BP
GO:0006915	apoptotic process	0.019	1 BP
GO:0021511	spinal cord patterning	0.019	1 BP
GO:0030150	protein import into mitochondrial matrix	0.019	1 BP
GO:0042402	cellular biogenic amine catabolic proces...	0.019	1 BP
GO:0060713	labyrinthine layer morphogenesis	0.019	1 BP
GO:0034637	cellular carbohydrate biosynthetic proce...	0.020	1 BP

GO:0000491	small nucleolar ribonucleoprotein comple...	0.020	1	BP
GO:0009125	nucleoside monophosphate catabolic proce...	0.020	1	BP
GO:0032060	bleb assembly	0.020	1	BP
GO:0046660	female sex differentiation	0.021	1	BP
GO:0070570	regulation of neuron projection regenera...	0.021	1	BP
GO:0070585	protein localization to mitochondrion	0.021	1	BP
GO:0030534	adult behavior	0.021	1	BP
GO:0007167	enzyme linked receptor protein signaling...	0.021	1	BP
GO:0015807	L-amino acid transport	0.021	1	BP
GO:0006112	energy reserve metabolic process	0.022	1	BP
GO:0010906	regulation of glucose metabolic process	0.022	1	BP
GO:0045185	maintenance of protein location	0.022	1	BP
GO:0044262	cellular carbohydrate metabolic process	0.023	1	BP
GO:0022414	reproductive process	0.023	1	BP
GO:0009310	amine catabolic process	0.023	1	BP
GO:0010226	response to lithium ion	0.023	1	BP
GO:0051043	regulation of membrane protein ectodomai...	0.023	1	BP
GO:0072012	glomerulus vasculature development	0.023	1	BP
GO:0000003	reproduction	0.023	1	BP
GO:0061512	protein localization to cilium	0.025	1	BP
GO:0005978	glycogen biosynthetic process	0.025	1	BP
GO:0009250	glucan biosynthetic process	0.025	1	BP
GO:0010043	response to zinc ion	0.025	1	BP
GO:0035296	regulation of tube diameter	0.025	1	BP
GO:0050880	regulation of blood vessel size	0.025	1	BP
GO:0097746	regulation of blood vessel diameter	0.025	1	BP
GO:0070848	response to growth factor	0.026	1	BP
GO:1901653	cellular response to peptide	0.026	1	BP
GO:0014854	response to inactivity	0.026	1	BP
GO:0014874	response to stimulus involved in regulat...	0.026	1	BP
GO:0021514	ventral spinal cord interneuron differen...	0.026	1	BP
GO:0021819	layer formation in cerebral cortex	0.026	1	BP
GO:0043970	histone H3-K9 acetylation	0.026	1	BP
GO:0048505	regulation of timing of cell differentia...	0.026	1	BP
GO:0048681	negative regulation of axon regeneration	0.026	1	BP
GO:0060253	negative regulation of glial cell prolif...	0.026	1	BP
GO:0071361	cellular response to ethanol	0.026	1	BP
GO:1902414	protein localization to cell junction	0.026	1	BP
GO:1902931	negative regulation of alcohol biosynthe...	0.026	1	BP
GO:0021953	central nervous system neuron differenti...	0.027	1	BP
GO:0043066	negative regulation of apoptotic process	0.027	1	BP
GO:2000736	regulation of stem cell differentiation	0.027	1	BP
GO:0035150	regulation of tube size	0.027	1	BP
GO:0006907	pinocytosis	0.027	1	BP
GO:0019430	removal of superoxide radicals	0.027	1	BP
GO:0032402	melanosome transport	0.027	1	BP
GO:0061003	positive regulation of dendritic spine m...	0.027	1	BP
GO:1903392	negative regulation of adherens junction...	0.027	1	BP
GO:0061951	establishment of protein localization to...	0.027	1	BP
GO:0003382	epithelial cell morphogenesis	0.027	1	BP
GO:0060428	lung epithelium development	0.027	1	BP
GO:0071392	cellular response to estradiol stimulus	0.027	1	BP

GO:0090322	regulation of superoxide metabolic proce...	0.027	1	BP
GO:0002865	negative regulation of acute inflammator...	0.028	1	BP
GO:0003310	pancreatic A cell differentiation	0.028	1	BP
GO:0006398	mRNA 3'-end processing by stem-loop bind...	0.028	1	BP
GO:0021520	spinal cord motor neuron cell fate speci...	0.028	1	BP
GO:0021796	cerebral cortex regionalization	0.028	1	BP
GO:0033152	immunoglobulin V(D)J recombination	0.028	1	BP
GO:0046113	nucleobase catabolic process	0.028	1	BP
GO:0090241	negative regulation of histone H4 acetyl...	0.028	1	BP
GO:0110011	regulation of basement membrane organiza...	0.028	1	BP
GO:1901642	nucleoside transmembrane transport	0.028	1	BP
GO:1901843	positive regulation of high voltage-gate...	0.028	1	BP
GO:1904259	regulation of basement membrane assembly...	0.028	1	BP
GO:1904261	positive regulation of basement membrane...	0.028	1	BP
GO:1904798	positive regulation of core promoter bin...	0.028	1	BP
GO:2000821	regulation of grooming behavior	0.028	1	BP
GO:2001197	basement membrane assembly involved in e...	0.028	1	BP
GO:0035272	exocrine system development	0.028	1	BP
GO:0043583	ear development	0.029	1	BP
GO:0010941	regulation of cell death	0.029	1	BP
GO:0097306	cellular response to alcohol	0.029	1	BP
GO:0051960	regulation of nervous system development	0.030	1	BP
GO:0010001	glial cell differentiation	0.030	1	BP
GO:1901264	carbohydrate derivative transport	0.030	1	BP
GO:0008285	negative regulation of cell proliferatio...	0.030	1	BP
GO:1904375	regulation of protein localization to ce...	0.031	1	BP
GO:0090288	negative regulation of cellular response...	0.032	1	BP
GO:0003209	cardiac atrium morphogenesis	0.032	1	BP
GO:0032401	establishment of melanosome localization	0.032	1	BP
GO:0051904	pigment granule transport	0.032	1	BP
GO:0061437	renal system vasculature development	0.032	1	BP
GO:0061440	kidney vasculature development	0.032	1	BP
GO:0097061	dendritic spine organization	0.032	1	BP
GO:0007417	central nervous system development	0.032	1	BP
GO:0045595	regulation of cell differentiation	0.032	1	BP
GO:0021781	glial cell fate commitment	0.033	1	BP
GO:0040034	regulation of development, heterochronic	0.033	1	BP
GO:0051044	positive regulation of membrane protein ...	0.033	1	BP
GO:0072189	ureter development	0.033	1	BP
GO:0090231	regulation of spindle checkpoint	0.033	1	BP
GO:0090266	regulation of mitotic cell cycle spindle...	0.033	1	BP
GO:1903504	regulation of mitotic spindle checkpoint	0.033	1	BP
GO:0016573	histone acetylation	0.034	1	BP
GO:0048663	neuron fate commitment	0.034	1	BP
GO:0060425	lung morphogenesis	0.034	1	BP
GO:0007044	cell-substrate junction assembly	0.035	1	BP
GO:0042063	gliogenesis	0.035	1	BP
GO:0072529	pyrimidine-containing compound catabolic...	0.035	1	BP
GO:0051321	meiotic cell cycle	0.035	1	BP
GO:0000278	mitotic cell cycle	0.035	1	BP
GO:0043966	histone H3 acetylation	0.036	1	BP
GO:0060135	maternal process involved in female preg...	0.036	1	BP

GO:0031398	positive regulation of protein ubiquitin...	0.037	1	BP
GO:0045471	response to ethanol	0.037	1	BP
GO:0051090	regulation of DNA-binding transcription ...	0.037	1	BP
GO:0015813	L-glutamate transmembrane transport	0.037	1	BP
GO:0021895	cerebral cortex neuron differentiation	0.037	1	BP
GO:0030431	sleep	0.037	1	BP
GO:0051220	cytoplasmic sequestering of protein	0.037	1	BP
GO:0051905	establishment of pigment granule localiz...	0.037	1	BP
GO:0060669	embryonic placenta morphogenesis	0.037	1	BP
GO:0071450	cellular response to oxygen radical	0.037	1	BP
GO:0071451	cellular response to superoxide	0.037	1	BP
GO:2000178	negative regulation of neural precursor ...	0.037	1	BP
GO:0043069	negative regulation of programmed cell d...	0.037	1	BP
GO:0048513	animal organ development	0.037	1	BP
GO:0006399	tRNA metabolic process	0.037	1	BP
GO:0000271	polysaccharide biosynthetic process	0.038	1	BP
GO:0030512	negative regulation of transforming grow...	0.038	1	BP
GO:1902808	positive regulation of cell cycle G1/S p...	0.038	1	BP
GO:0061351	neural precursor cell proliferation	0.039	1	BP
GO:0021955	central nervous system neuron axonogenes...	0.039	1	BP
GO:0031396	regulation of protein ubiquitination	0.039	1	BP
GO:0048839	inner ear development	0.039	1	BP
GO:0007548	sex differentiation	0.040	1	BP
GO:0006396	RNA processing	0.040	1	BP
GO:0001921	positive regulation of receptor recyclin...	0.040	1	BP
GO:0018410	C-terminal protein amino acid modificati...	0.040	1	BP
GO:0030540	female genitalia development	0.040	1	BP
GO:0042762	regulation of sulfur metabolic process	0.040	1	BP
GO:0048521	negative regulation of behavior	0.040	1	BP
GO:0061548	ganglion development	0.040	1	BP
GO:0070525	tRNA threonylcarbamoyladenosine metaboli...	0.040	1	BP
GO:0070571	negative regulation of neuron projection...	0.040	1	BP
GO:2000402	negative regulation of lymphocyte migrat...	0.040	1	BP
GO:0010675	regulation of cellular carbohydrate meta...	0.040	1	BP
GO:0002118	aggressive behavior	0.040	1	BP
GO:0006501	C-terminal protein lipidation	0.040	1	BP
GO:0010032	meiotic chromosome condensation	0.040	1	BP
GO:0010265	SCF complex assembly	0.040	1	BP
GO:0015858	nucleoside transport	0.040	1	BP
GO:0019249	lactate biosynthetic process	0.040	1	BP
GO:0019323	pentose catabolic process	0.040	1	BP
GO:0030091	protein repair	0.040	1	BP
GO:0042426	choline catabolic process	0.040	1	BP
GO:0046469	platelet activating factor metabolic pro...	0.040	1	BP
GO:0048549	positive regulation of pinocytosis	0.040	1	BP
GO:0048703	embryonic viscerocranium morphogenesis	0.040	1	BP
GO:0060754	positive regulation of mast cell chemota...	0.040	1	BP
GO:0070141	response to UV-A	0.040	1	BP
GO:1901621	negative regulation of smoothened signal...	0.040	1	BP
GO:1904796	regulation of core promoter binding	0.040	1	BP
GO:2000321	positive regulation of T-helper 17 cell ...	0.040	1	BP
GO:2000405	negative regulation of T cell migration	0.040	1	BP

GO:2000618	regulation of histone H4-K16 acetylation	0.040	1	BP
GO:2000739	regulation of mesenchymal stem cell diff...	0.040	1	BP
GO:0031589	cell-substrate adhesion	0.041	1	BP
GO:0006911	phagocytosis, engulfment	0.042	1	BP
GO:0010171	body morphogenesis	0.042	1	BP
GO:0030865	cortical cytoskeleton organization	0.042	1	BP
GO:0010812	negative regulation of cell-substrate ad...	0.042	1	BP
GO:0032400	melanosome localization	0.042	1	BP
GO:0060571	morphogenesis of an epithelial fold	0.042	1	BP
GO:1903421	regulation of synaptic vesicle recycling	0.042	1	BP
GO:0071363	cellular response to growth factor stimu...	0.043	1	BP
GO:0018393	internal peptidyl-lysine acetylation	0.043	1	BP
GO:0001953	negative regulation of cell-matrix adhes...	0.044	1	BP
GO:0045124	regulation of bone resorption	0.044	1	BP
GO:0046189	phenol-containing compound biosynthetic ...	0.044	1	BP
GO:0040008	regulation of growth	0.044	1	BP
GO:1903845	negative regulation of cellular response...	0.044	1	BP
GO:0010942	positive regulation of cell death	0.044	1	BP
GO:0042493	response to drug	0.045	1	BP
GO:0060541	respiratory system development	0.045	1	BP
GO:0060563	neuroepithelial cell differentiation	0.046	1	BP
GO:0034330	cell junction organization	0.046	1	BP
GO:0007043	cell-cell junction assembly	0.046	1	BP
GO:0071559	response to transforming growth factor b...	0.046	1	BP
GO:1901990	regulation of mitotic cell cycle phase t...	0.046	1	BP
GO:0051094	positive regulation of developmental pro...	0.046	1	BP
GO:1902600	proton transmembrane transport	0.047	1	BP
GO:0046620	regulation of organ growth	0.047	1	BP
GO:0051961	negative regulation of nervous system de...	0.048	1	BP
GO:0001696	gastric acid secretion	0.048	1	BP
GO:0006086	acetyl-CoA biosynthetic process from pyr...	0.048	1	BP
GO:0006098	pentose-phosphate shunt	0.048	1	BP
GO:0021542	dentate gyrus development	0.048	1	BP
GO:0032780	negative regulation of ATPase activity	0.048	1	BP
GO:0032988	ribonucleoprotein complex disassembly	0.048	1	BP
GO:0035067	negative regulation of histone acetylati...	0.048	1	BP
GO:0045116	protein neddylation	0.048	1	BP
GO:0050802	circadian sleep/wake cycle, sleep	0.048	1	BP
GO:0050884	neuromuscular process controlling postur...	0.048	1	BP
GO:0070593	dendrite self-avoidance	0.048	1	BP
GO:0070875	positive regulation of glycogen metaboli...	0.048	1	BP
GO:2000738	positive regulation of stem cell differe...	0.048	1	BP
GO:0012501	programmed cell death	0.048	1	BP
GO:0000303	response to superoxide	0.048	1	BP
GO:0008334	histone mRNA metabolic process	0.048	1	BP
GO:0021952	central nervous system projection neuron...	0.048	1	BP
GO:0048679	regulation of axon regeneration	0.048	1	BP
GO:0051875	pigment granule localization	0.048	1	BP
GO:0085029	extracellular matrix assembly	0.048	1	BP
GO:0048806	genitalia development	0.048	1	BP
GO:1900087	positive regulation of G1/S transition o...	0.048	1	BP
GO:0002064	epithelial cell development	0.049	1	BP

GO:1901988	negative regulation of cell cycle phase ...	0.050	1	BP
GO:0034754	cellular hormone metabolic process	0.050	1	BP
GO:0002162	dystroglycan binding	0.001	1	MF
GO:0047961	glycine N-acyltransferase activity	0.003	1	MF
GO:0050072	m7G(5')pppN diphosphatase activity	0.005	1	MF
GO:0019838	growth factor binding	0.007	1	MF
GO:0005520	insulin-like growth factor binding	0.008	1	MF
GO:0034596	phosphatidylinositol phosphate 4-phospha...	0.008	1	MF
GO:0031489	myosin V binding	0.008	1	MF
GO:0000293	ferric-chelate reductase activity	0.009	1	MF
GO:0050436	microfibril binding	0.009	1	MF
GO:0071209	U7 snRNA binding	0.009	1	MF
GO:0120160	intraciliary transport particle A bindin...	0.009	1	MF
GO:0017046	peptide hormone binding	0.009	1	MF
GO:0017069	snRNA binding	0.013	1	MF
GO:0005200	structural constituent of cytoskeleton	0.013	1	MF
GO:0030621	U4 snRNA binding	0.016	1	MF
GO:0000298	endopolyphosphatase activity	0.017	1	MF
GO:0004145	diamine N-acetyltransferase activity	0.017	1	MF
GO:0008486	diphosphoinositol-polyphosphate diphosph...	0.017	1	MF
GO:0008934	inositol monophosphate 1-phosphatase act...	0.017	1	MF
GO:0015184	L-cystine transmembrane transporter acti...	0.017	1	MF
GO:0034431	bis(5'-adenosyl)-hexaphosphatase activit...	0.017	1	MF
GO:0034432	bis(5'-adenosyl)-pentaphosphatase activi...	0.017	1	MF
GO:0043812	phosphatidylinositol-4-phosphate phospha...	0.017	1	MF
GO:0052832	inositol monophosphate 3-phosphatase act...	0.017	1	MF
GO:0052833	inositol monophosphate 4-phosphatase act...	0.017	1	MF
GO:0052834	inositol monophosphate phosphatase activ...	0.017	1	MF
GO:0052840	inositol diphosphate tetrakisphosphate d...	0.017	1	MF
GO:0052842	inositol diphosphate pentakisphosphate d...	0.017	1	MF
GO:0071208	histone pre-mRNA DCP binding	0.017	1	MF
GO:0097016	L27 domain binding	0.017	1	MF
GO:0003924	GTPase activity	0.018	1	MF
GO:0015179	L-amino acid transmembrane transporter a...	0.018	1	MF
GO:0005509	calcium ion binding	0.019	1	MF
GO:0016857	racemase and epimerase activity, acting ...	0.020	1	MF
GO:0071837	HMG box domain binding	0.020	1	MF
GO:0046332	SMAD binding	0.022	1	MF
GO:0003779	actin binding	0.022	1	MF
GO:0001221	transcription cofactor binding	0.023	1	MF
GO:0051015	actin filament binding	0.025	1	MF
GO:0019199	transmembrane receptor protein kinase ac...	0.025	1	MF
GO:0016410	N-acyltransferase activity	0.027	1	MF
GO:0050431	transforming growth factor beta binding	0.027	1	MF
GO:0002054	nucleobase binding	0.028	1	MF
GO:0004689	phosphorylase kinase activity	0.028	1	MF
GO:0005337	nucleoside transmembrane transporter act...	0.028	1	MF
GO:0016933	extracellularly glycine-gated ion channe...	0.028	1	MF
GO:0016934	extracellularly glycine-gated chloride c...	0.028	1	MF
GO:0042562	hormone binding	0.028	1	MF
GO:0015171	amino acid transmembrane transporter act...	0.030	1	MF
GO:0008565	protein transporter activity	0.032	1	MF

GO:0008134	transcription factor binding	0.034	1	MF
GO:0016746	transferase activity, transferring acyl ...	0.035	1	MF
GO:0098631	cell adhesion mediator activity	0.036	1	MF
GO:0005313	L-glutamate transmembrane transporter ac...	0.040	1	MF
GO:0015172	acidic amino acid transmembrane transpor...	0.040	1	MF
GO:0052745	inositol phosphate phosphatase activity	0.040	1	MF
GO:0010340	carboxyl-O-methyltransferase activity	0.040	1	MF
GO:0051998	protein carboxyl O-methyltransferase act...	0.040	1	MF
GO:0008509	anion transmembrane transporter activity	0.042	1	MF
GO:0098632	cell-cell adhesion mediator activity	0.046	1	MF
GO:0008656	cysteine-type endopeptidase activator ac...	0.048	1	MF
GO:0030021	extracellular matrix structural constitu...	0.048	1	MF
GO:0008092	cytoskeletal protein binding	0.049	1	MF
GO:0004402	histone acetyltransferase activity	0.049	1	MF
GO:0004714	transmembrane receptor protein tyrosine ...	0.049	1	MF
GO:0046540	U4/U6 x U5 tri-snRNP complex	0.000	0.054588	CC
GO:0097526	spliceosomal tri-snRNP complex	0.000	0.054588	CC
GO:0001405	PAM complex, Tim23 associated import mot...	0.002	1	CC
GO:0031372	UBC13-MMS2 complex	0.003	1	CC
GO:0120114	Sm-like protein family complex	0.004	1	CC
GO:0030532	small nuclear ribonucleoprotein complex	0.004	1	CC
GO:0005744	TIM23 mitochondrial import inner membran...	0.005	1	CC
GO:0032432	actin filament bundle	0.006	1	CC
GO:0097525	spliceosomal snRNP complex	0.007	1	CC
GO:0031371	ubiquitin conjugating enzyme complex	0.008	1	CC
GO:0001725	stress fiber	0.008	1	CC
GO:0097517	contractile actin filament bundle	0.008	1	CC
GO:0042641	actomyosin	0.009	1	CC
GO:0031262	Ndc80 complex	0.009	1	CC
GO:0031901	early endosome membrane	0.010	1	CC
GO:0098862	cluster of actin-based cell projections	0.011	1	CC
GO:0031312	extrinsic component of organelle membran...	0.012	1	CC
GO:0017053	transcriptional repressor complex	0.013	1	CC
GO:0000776	kinetochore	0.013	1	CC
GO:0070461	SAGA-type complex	0.014	1	CC
GO:0031966	mitochondrial membrane	0.014	1	CC
GO:0001726	ruffle	0.015	1	CC
GO:1902911	protein kinase complex	0.015	1	CC
GO:0031313	extrinsic component of endosome membrane	0.016	1	CC
GO:0005903	brush border	0.017	1	CC
GO:0005740	mitochondrial envelope	0.018	1	CC
GO:0005899	insulin receptor complex	0.018	1	CC
GO:0035867	alphav-beta3 integrin-IGF-1-IGF1R comple...	0.018	1	CC
GO:0000777	condensed chromosome kinetochore	0.023	1	CC
GO:0005682	U5 snRNP	0.024	1	CC
GO:0005881	cytoplasmic microtubule	0.025	1	CC
GO:0005687	U4 snRNP	0.027	1	CC
GO:0005688	U6 snRNP	0.027	1	CC
GO:0000125	PCAF complex	0.028	1	CC
GO:0005964	phosphorylase kinase complex	0.028	1	CC
GO:0097025	MPP7-DLG1-LIN7 complex	0.028	1	CC
GO:0097255	R2TP complex	0.028	1	CC

GO:1902562	H4 histone acetyltransferase complex	0.029	1	CC
GO:0000775	chromosome, centromeric region	0.031	1	CC
GO:0033176	proton-transporting V-type ATPase comple...	0.033	1	CC
GO:0031314	extrinsic component of mitochondrial inn...	0.033	1	CC
GO:0098845	postsynaptic endosome	0.033	1	CC
GO:0005741	mitochondrial outer membrane	0.034	1	CC
GO:0044429	mitochondrial part	0.034	1	CC
GO:0044428	nuclear part	0.039	1	CC
GO:0016459	myosin complex	0.040	1	CC
GO:0000123	histone acetyltransferase complex	0.041	1	CC
GO:0071204	histone pre-mRNA 3'end processing comple...	0.041	1	CC
GO:0098837	postsynaptic recycling endosome	0.041	1	CC
GO:0120111	neuron projection cytoplasm	0.046	1	CC
GO:0000779	condensed chromosome, centromeric region	0.048	1	CC
GO:0032426	stereocilium tip	0.049	1	CC
GO:0044815	DNA packaging complex	0.049	1	CC

dlPFC

GO.ID	Term	FET	BH	Category
GO:0051707	response to other organism	0.000	0.120037	BP
GO:0043207	response to external biotic stimulus	0.000	0.120037	BP
GO:0009607	response to biotic stimulus	0.000	0.120037	BP
GO:0006955	immune response	0.000	0.131966	BP
GO:0006952	defense response	0.000	0.131966	BP
GO:0002833	positive regulation of response to bioti...	0.000	0.131966	BP
GO:0098542	defense response to other organism	0.000	0.131966	BP
GO:0045087	innate immune response	0.000	0.133085	BP
GO:0002252	immune effector process	0.000	0.139173	BP
GO:0002831	regulation of response to biotic stimulu...	0.000	0.140913	BP
GO:0050776	regulation of immune response	0.000	0.169618	BP
GO:0009605	response to external stimulus	0.000	0.169618	BP
GO:0043435	response to corticotropin-releasing horm...	0.000	0.177446	BP
GO:0071376	cellular response to corticotropin-relea...	0.000	0.177446	BP
GO:0009617	response to bacterium	0.000	0.177446	BP
GO:0042448	progesterone metabolic process	0.000	0.23946	BP
GO:0042753	positive regulation of circadian rhythm	0.000	0.23946	BP
GO:0050778	positive regulation of immune response	0.000	0.247216	BP
GO:0002764	immune response-regulating signaling pat...	0.000	0.247216	BP
GO:0006779	porphyrin-containing compound biosynthet...	0.000	0.250512	BP
GO:0045088	regulation of innate immune response	0.000	0.26095	BP
GO:0002237	response to molecule of bacterial origin	0.000	0.317745	BP
GO:0002699	positive regulation of immune effector p...	0.001	0.317745	BP
GO:0033014	tetrapyrrole biosynthetic process	0.001	0.317745	BP
GO:0002221	pattern recognition receptor signaling p...	0.001	0.317745	BP
GO:0072358	cardiovascular system development	0.001	0.317745	BP
GO:0032655	regulation of interleukin-12 production	0.001	0.317745	BP
GO:0002376	immune system process	0.001	0.317745	BP
GO:0032618	interleukin-15 production	0.001	0.317745	BP
GO:1904344	regulation of gastric mucosal blood circ...	0.001	0.317745	BP
GO:1904346	positive regulation of gastric mucosal b...	0.001	0.317745	BP
GO:1990768	gastric mucosal blood circulation	0.001	0.317745	BP
GO:0002757	immune response-activating signal transd...	0.001	0.317745	BP
GO:0032615	interleukin-12 production	0.001	0.317745	BP
GO:0002253	activation of immune response	0.001	0.342761	BP
GO:0032496	response to lipopolysaccharide	0.001	0.342761	BP
GO:0033993	response to lipid	0.001	0.342761	BP
GO:0035912	dorsal aorta morphogenesis	0.001	0.362583	BP
GO:0002684	positive regulation of immune system pro...	0.001	0.36533	BP
GO:0034340	response to type I interferon	0.001	0.376514	BP
GO:0001944	vasculature development	0.001	0.376514	BP
GO:0045824	negative regulation of innate immune res...	0.001	0.376514	BP
GO:1990266	neutrophil migration	0.001	0.389604	BP
GO:0009725	response to hormone	0.001	0.398542	BP
GO:0006778	porphyrin-containing compound metabolic ...	0.001	0.433066	BP
GO:0035907	dorsal aorta development	0.001	0.433066	BP
GO:0048545	response to steroid hormone	0.001	0.433066	BP
GO:0031347	regulation of defense response	0.001	0.440353	BP
GO:0098586	cellular response to virus	0.001	0.456929	BP
GO:0014070	response to organic cyclic compound	0.002	0.482236	BP

GO:0039530	MDA-5 signaling pathway	0.002	0.543875	BP
GO:0042273	ribosomal large subunit biogenesis	0.002	0.543875	BP
GO:0006783	heme biosynthetic process	0.002	0.543875	BP
GO:0045576	mast cell activation	0.002	0.543875	BP
GO:0031120	snRNA pseudouridine synthesis	0.002	0.543875	BP
GO:0045938	positive regulation of circadian sleep/w...	0.002	0.543875	BP
GO:0046010	positive regulation of circadian sleep/w...	0.002	0.543875	BP
GO:0002224	toll-like receptor signaling pathway	0.002	0.553394	BP
GO:0071219	cellular response to molecule of bacteri...	0.002	0.599743	BP
GO:0001909	leukocyte mediated cytotoxicity	0.003	0.671736	BP
GO:0030431	sleep	0.003	0.671736	BP
GO:0090022	regulation of neutrophil chemotaxis	0.003	0.671736	BP
GO:0048514	blood vessel morphogenesis	0.003	0.708293	BP
GO:0030522	intracellular receptor signaling pathway	0.003	0.752918	BP
GO:0035239	tube morphogenesis	0.003	0.752918	BP
GO:0060337	type I interferon signaling pathway	0.003	0.752918	BP
GO:0071357	cellular response to type I interferon	0.003	0.752918	BP
GO:0002697	regulation of immune effector process	0.003	0.752918	BP
GO:0000303	response to superoxide	0.004	0.820427	BP
GO:0001525	angiogenesis	0.004	0.820427	BP
GO:0014805	smooth muscle adaptation	0.004	0.820427	BP
GO:0039534	negative regulation of MDA-5 signaling p...	0.004	0.820427	BP
GO:0070943	neutrophil mediated killing of symbiont ...	0.004	0.820427	BP
GO:2000182	regulation of progesterone biosynthetic ...	0.004	0.820427	BP
GO:0009615	response to virus	0.004	0.820427	BP
GO:0032695	negative regulation of interleukin-12 pr...	0.004	0.838343	BP
GO:0001568	blood vessel development	0.004	0.838343	BP
GO:0000305	response to oxygen radical	0.004	0.838343	BP
GO:1902622	regulation of neutrophil migration	0.004	0.838343	BP
GO:0043303	mast cell degranulation	0.004	0.863092	BP
GO:0045089	positive regulation of innate immune res...	0.004	0.8679	BP
GO:0006950	response to stress	0.005	0.869833	BP
GO:1903555	regulation of tumor necrosis factor supe...	0.005	0.869833	BP
GO:0002443	leukocyte mediated immunity	0.005	0.869833	BP
GO:0042168	heme metabolic process	0.005	0.869833	BP
GO:0002279	mast cell activation involved in immune ...	0.005	0.869833	BP
GO:0051607	defense response to virus	0.005	0.869833	BP
GO:0071222	cellular response to lipopolysaccharide	0.005	0.869833	BP
GO:1901342	regulation of vasculature development	0.005	0.869833	BP
GO:0006839	mitochondrial transport	0.005	0.869833	BP
GO:0002682	regulation of immune system process	0.005	0.907433	BP
GO:0097530	granulocyte migration	0.005	0.907433	BP
GO:0002448	mast cell mediated immunity	0.005	0.907433	BP
GO:0002218	activation of innate immune response	0.006	0.917767	BP
GO:0071706	tumor necrosis factor superfamily cytoki...	0.006	0.93064	BP
GO:0031349	positive regulation of defense response	0.006	0.93064	BP
GO:0050802	circadian sleep/wake cycle, sleep	0.006	0.93064	BP
GO:0035546	interferon-beta secretion	0.006	0.93064	BP
GO:0035547	regulation of interferon-beta secretion	0.006	0.93064	BP
GO:0040031	snRNA modification	0.006	0.93064	BP
GO:0042536	negative regulation of tumor necrosis fa...	0.006	0.93064	BP
GO:0042747	circadian sleep/wake cycle, REM sleep	0.006	0.93064	BP

GO:0051852	disruption by host of symbiont cells	0.006	0.93064	BP
GO:0051873	killing by host of symbiont cells	0.006	0.93064	BP
GO:0061484	hematopoietic stem cell homeostasis	0.006	0.93064	BP
GO:0070942	neutrophil mediated cytotoxicity	0.006	0.93064	BP
GO:2001186	negative regulation of CD8-positive, alp...	0.006	0.93064	BP
GO:0009409	response to cold	0.007	0.946669	BP
GO:0071216	cellular response to biotic stimulus	0.007	0.961989	BP
GO:0002444	myeloid leukocyte mediated immunity	0.007	0.961989	BP
GO:0002758	innate immune response-activating signal...	0.007	0.961989	BP
GO:0034097	response to cytokine	0.007	0.973457	BP
GO:0045747	positive regulation of Notch signaling p...	0.007	0.973457	BP
GO:1904705	regulation of vascular smooth muscle cel...	0.007	0.973457	BP
GO:1990874	vascular smooth muscle cell proliferatio...	0.007	0.973457	BP
GO:0001936	regulation of endothelial cell prolifera...	0.007	1	BP
GO:0042180	cellular ketone metabolic process	0.008	1	BP
GO:0019221	cytokine-mediated signaling pathway	0.008	1	BP
GO:0071495	cellular response to endogenous stimulus	0.008	1	BP
GO:1904707	positive regulation of vascular smooth m...	0.008	1	BP
GO:0031960	response to corticosteroid	0.008	1	BP
GO:0032870	cellular response to hormone stimulus	0.008	1	BP
GO:0033013	tetrapyrrole metabolic process	0.009	1	BP
GO:1904018	positive regulation of vasculature devel...	0.009	1	BP
GO:0042254	ribosome biogenesis	0.009	1	BP
GO:0071310	cellular response to organic substance	0.009	1	BP
GO:0003159	morphogenesis of an endothelium	0.009	1	BP
GO:0022410	circadian sleep/wake cycle process	0.009	1	BP
GO:0061154	endothelial tube morphogenesis	0.009	1	BP
GO:0001937	negative regulation of endothelial cell ...	0.009	1	BP
GO:0046890	regulation of lipid biosynthetic process	0.009	1	BP
GO:0006701	progesterone biosynthetic process	0.009	1	BP
GO:0031118	rRNA pseudouridine synthesis	0.009	1	BP
GO:0034137	positive regulation of toll-like recepto...	0.009	1	BP
GO:0051601	exocyst localization	0.009	1	BP
GO:0060331	negative regulation of response to inter...	0.009	1	BP
GO:0060336	negative regulation of interferon-gamma-...	0.009	1	BP
GO:0070339	response to bacterial lipopeptide	0.009	1	BP
GO:0071220	cellular response to bacterial lipoprote...	0.009	1	BP
GO:0071221	cellular response to bacterial lipopepti...	0.009	1	BP
GO:2001179	regulation of interleukin-10 secretion	0.009	1	BP
GO:2001300	lipoxin metabolic process	0.009	1	BP
GO:2001301	lipoxin biosynthetic process	0.009	1	BP
GO:0009719	response to endogenous stimulus	0.009	1	BP
GO:0048871	multicellular organismal homeostasis	0.010	1	BP
GO:0001974	blood vessel remodeling	0.010	1	BP
GO:0008207	C21-steroid hormone metabolic process	0.010	1	BP
GO:0043928	exonucleolytic nuclear-transcribed mRNA ...	0.010	1	BP
GO:1901030	positive regulation of mitochondrial out...	0.010	1	BP
GO:0097529	myeloid leukocyte migration	0.010	1	BP
GO:0061081	positive regulation of myeloid leukocyte...	0.011	1	BP
GO:0090023	positive regulation of neutrophil chemot...	0.011	1	BP
GO:1903556	negative regulation of tumor necrosis fa...	0.011	1	BP
GO:0045765	regulation of angiogenesis	0.011	1	BP

GO:0001906	cell killing	0.012	1	BP
GO:0046683	response to organophosphorus	0.012	1	BP
GO:0006120	mitochondrial electron transport, NADH t...	0.012	1	BP
GO:0030593	neutrophil chemotaxis	0.012	1	BP
GO:0035924	cellular response to vascular endothelia...	0.012	1	BP
GO:0031668	cellular response to extracellular stimu...	0.012	1	BP
GO:0031348	negative regulation of defense response	0.012	1	BP
GO:0032611	interleukin-1 beta production	0.012	1	BP
GO:0030728	ovulation	0.012	1	BP
GO:0042745	circadian sleep/wake cycle	0.012	1	BP
GO:0071624	positive regulation of granulocyte chemo...	0.012	1	BP
GO:0000291	nuclear-transcribed mRNA catabolic proce...	0.012	1	BP
GO:0002755	MyD88-dependent toll-like receptor signa...	0.012	1	BP
GO:0071622	regulation of granulocyte chemotaxis	0.012	1	BP
GO:0001910	regulation of leukocyte mediated cytotox...	0.013	1	BP
GO:0048844	artery morphogenesis	0.013	1	BP
GO:0032680	regulation of tumor necrosis factor prod...	0.013	1	BP
GO:0001542	ovulation from ovarian follicle	0.013	1	BP
GO:0015886	heme transport	0.013	1	BP
GO:0032493	response to bacterial lipoprotein	0.013	1	BP
GO:0039533	regulation of MDA-5 signaling pathway	0.013	1	BP
GO:0045188	regulation of circadian sleep/wake cycle...	0.013	1	BP
GO:0051818	disruption of cells of other organism in...	0.013	1	BP
GO:0051883	killing of cells in other organism invol...	0.013	1	BP
GO:1905065	positive regulation of vascular smooth m...	0.013	1	BP
GO:2000253	positive regulation of feeding behavior	0.013	1	BP
GO:0002250	adaptive immune response	0.014	1	BP
GO:0048512	circadian behavior	0.014	1	BP
GO:0035914	skeletal muscle cell differentiation	0.014	1	BP
GO:0071396	cellular response to lipid	0.014	1	BP
GO:0048520	positive regulation of behavior	0.014	1	BP
GO:0051349	positive regulation of lyase activity	0.014	1	BP
GO:0001935	endothelial cell proliferation	0.015	1	BP
GO:0032640	tumor necrosis factor production	0.015	1	BP
GO:0003044	regulation of systemic arterial blood pr...	0.015	1	BP
GO:0007622	rhythmic behavior	0.015	1	BP
GO:0033003	regulation of mast cell activation	0.015	1	BP
GO:0042304	regulation of fatty acid biosynthetic pr...	0.015	1	BP
GO:1901701	cellular response to oxygen-containing c...	0.015	1	BP
GO:0001818	negative regulation of cytokine producti...	0.015	1	BP
GO:0071345	cellular response to cytokine stimulus	0.015	1	BP
GO:0043434	response to peptide hormone	0.015	1	BP
GO:0071496	cellular response to external stimulus	0.016	1	BP
GO:0072359	circulatory system development	0.016	1	BP
GO:0019430	removal of superoxide radicals	0.016	1	BP
GO:0032691	negative regulation of interleukin-1 bet...	0.016	1	BP
GO:0060259	regulation of feeding behavior	0.016	1	BP
GO:1902624	positive regulation of neutrophil migrat...	0.016	1	BP
GO:2000108	positive regulation of leukocyte apoptot...	0.016	1	BP
GO:0072593	reactive oxygen species metabolic proces...	0.016	1	BP
GO:0001775	cell activation	0.017	1	BP
GO:0001955	blood vessel maturation	0.017	1	BP

GO:0006782	protoporphyrinogen IX biosynthetic proce...	0.017	1	BP
GO:0042118	endothelial cell activation	0.017	1	BP
GO:0042748	circadian sleep/wake cycle, non-REM slee...	0.017	1	BP
GO:0045602	negative regulation of endothelial cell ...	0.017	1	BP
GO:0060907	positive regulation of macrophage cytoki...	0.017	1	BP
GO:0072608	interleukin-10 secretion	0.017	1	BP
GO:0097084	vascular smooth muscle cell development	0.017	1	BP
GO:1900426	positive regulation of defense response ...	0.017	1	BP
GO:0034121	regulation of toll-like receptor signali...	0.017	1	BP
GO:0009755	hormone-mediated signaling pathway	0.018	1	BP
GO:0048661	positive regulation of smooth muscle cel...	0.018	1	BP
GO:0010565	regulation of cellular ketone metabolic ...	0.018	1	BP
GO:0002446	neutrophil mediated immunity	0.018	1	BP
GO:0009451	RNA modification	0.018	1	BP
GO:0002026	regulation of the force of heart contrac...	0.018	1	BP
GO:0003209	cardiac atrium morphogenesis	0.018	1	BP
GO:0031664	regulation of lipopolysaccharide-mediate...	0.018	1	BP
GO:0071377	cellular response to glucagon stimulus	0.018	1	BP
GO:2001025	positive regulation of response to drug	0.018	1	BP
GO:0002274	myeloid leukocyte activation	0.019	1	BP
GO:1901652	response to peptide	0.019	1	BP
GO:1901700	response to oxygen-containing compound	0.019	1	BP
GO:0043534	blood vessel endothelial cell migration	0.019	1	BP
GO:0043627	response to estrogen	0.019	1	BP
GO:0048646	anatomical structure formation involved ...	0.019	1	BP
GO:0001938	positive regulation of endothelial cell ...	0.020	1	BP
GO:0032612	interleukin-1 production	0.020	1	BP
GO:0002366	leukocyte activation involved in immune ...	0.020	1	BP
GO:0032692	negative regulation of interleukin-1 pro...	0.021	1	BP
GO:0071450	cellular response to oxygen radical	0.021	1	BP
GO:0071451	cellular response to superoxide	0.021	1	BP
GO:0001816	cytokine production	0.021	1	BP
GO:0003206	cardiac chamber morphogenesis	0.021	1	BP
GO:0002836	positive regulation of response to tumor...	0.021	1	BP
GO:0002839	positive regulation of immune response t...	0.021	1	BP
GO:0007342	fusion of sperm to egg plasma membrane i...	0.021	1	BP
GO:0032933	SREBP signaling pathway	0.021	1	BP
GO:0034135	regulation of toll-like receptor 2 signa...	0.021	1	BP
GO:0042363	fat-soluble vitamin catabolic process	0.021	1	BP
GO:0046886	positive regulation of hormone biosynthe...	0.021	1	BP
GO:0051593	response to folic acid	0.021	1	BP
GO:0070234	positive regulation of T cell apoptotic ...	0.021	1	BP
GO:0070391	response to lipoteichoic acid	0.021	1	BP
GO:0071223	cellular response to lipoteichoic acid	0.021	1	BP
GO:0071501	cellular response to sterol depletion	0.021	1	BP
GO:0072641	type I interferon secretion	0.021	1	BP
GO:0098856	intestinal lipid absorption	0.021	1	BP
GO:1901678	iron coordination entity transport	0.021	1	BP
GO:0014074	response to purine-containing compound	0.022	1	BP
GO:0002263	cell activation involved in immune respo...	0.022	1	BP
GO:0002275	myeloid cell activation involved in immu...	0.022	1	BP
GO:0006364	rRNA processing	0.023	1	BP

GO:1901028	regulation of mitochondrial outer membra...	0.023	1	BP
GO:0007263	nitric oxide mediated signal transductio...	0.023	1	BP
GO:0035767	endothelial cell chemotaxis	0.023	1	BP
GO:0006875	cellular metal ion homeostasis	0.024	1	BP
GO:0032103	positive regulation of response to exter...	0.024	1	BP
GO:0002703	regulation of leukocyte mediated immunit...	0.025	1	BP
GO:0032480	negative regulation of type I interferon...	0.025	1	BP
GO:1903428	positive regulation of reactive oxygen s...	0.025	1	BP
GO:0010257	NADH dehydrogenase complex assembly	0.025	1	BP
GO:0031341	regulation of cell killing	0.025	1	BP
GO:0032981	mitochondrial respiratory chain complex ...	0.025	1	BP
GO:0007005	mitochondrion organization	0.025	1	BP
GO:0042742	defense response to bacterium	0.025	1	BP
GO:0000255	allantoin metabolic process	0.026	1	BP
GO:0000256	allantoin catabolic process	0.026	1	BP
GO:0001315	age-dependent response to reactive oxyge...	0.026	1	BP
GO:0001927	exocyst assembly	0.026	1	BP
GO:0001928	regulation of exocyst assembly	0.026	1	BP
GO:0002041	intussusceptive angiogenesis	0.026	1	BP
GO:0002290	gamma-delta T cell activation involved i...	0.026	1	BP
GO:0002413	tolerance induction to tumor cell	0.026	1	BP
GO:0002416	IgG immunoglobulin transcytosis in epith...	0.026	1	BP
GO:0002843	regulation of tolerance induction to tum...	0.026	1	BP
GO:0002845	positive regulation of tolerance inducti...	0.026	1	BP
GO:0003068	regulation of systemic arterial blood pr...	0.026	1	BP
GO:0003069	acetylcholine-mediated vasodilation invo...	0.026	1	BP
GO:0003070	regulation of systemic arterial blood pr...	0.026	1	BP
GO:0003083	negative regulation of renal output by a...	0.026	1	BP
GO:0003106	negative regulation of glomerular filtra...	0.026	1	BP
GO:0006480	N-terminal protein amino acid methylatio...	0.026	1	BP
GO:0007499	ectoderm and mesoderm interaction	0.026	1	BP
GO:0009131	pyrimidine nucleoside monophosphate cata...	0.026	1	BP
GO:0009178	pyrimidine deoxyribonucleoside monophosp...	0.026	1	BP
GO:0009444	pyruvate oxidation	0.026	1	BP
GO:0010070	zygote asymmetric cell division	0.026	1	BP
GO:0010272	response to silver ion	0.026	1	BP
GO:0014895	smooth muscle hypertrophy	0.026	1	BP
GO:0015700	arsenite transport	0.026	1	BP
GO:0018011	N-terminal peptidyl-alanine methylation	0.026	1	BP
GO:0018012	N-terminal peptidyl-alanine trimethylati...	0.026	1	BP
GO:0018013	N-terminal peptidyl-glycine methylation	0.026	1	BP
GO:0018016	N-terminal peptidyl-proline dimethylatio...	0.026	1	BP
GO:0018194	peptidyl-alanine modification	0.026	1	BP
GO:0031049	programmed DNA elimination	0.026	1	BP
GO:0031052	chromosome breakage	0.026	1	BP
GO:0032658	regulation of interleukin-15 production	0.026	1	BP
GO:0032738	positive regulation of interleukin-15 pr...	0.026	1	BP
GO:0035351	heme transmembrane transport	0.026	1	BP
GO:0035548	negative regulation of interferon-beta s...	0.026	1	BP
GO:0035568	N-terminal peptidyl-proline methylation	0.026	1	BP
GO:0035570	N-terminal peptidyl-serine methylation	0.026	1	BP
GO:0035572	N-terminal peptidyl-serine dimethylation	0.026	1	BP

GO:0035573	N-terminal peptidyl-serine trimethylatio...	0.026	1	BP
GO:0035611	protein branching point deglutamylation	0.026	1	BP
GO:0035664	TIRAP-dependent toll-like receptor signa...	0.026	1	BP
GO:0035665	TIRAP-dependent toll-like receptor 4 sig...	0.026	1	BP
GO:0038097	positive regulation of mast cell activat...	0.026	1	BP
GO:0043251	sodium-dependent organic anion transport	0.026	1	BP
GO:0046079	dUMP catabolic process	0.026	1	BP
GO:0052550	response to defense-related reactive oxy...	0.026	1	BP
GO:0052567	response to defense-related host reactiv...	0.026	1	BP
GO:0060178	regulation of exocyst localization	0.026	1	BP
GO:0070130	negative regulation of mitochondrial tra...	0.026	1	BP
GO:0070543	response to linoleic acid	0.026	1	BP
GO:0070627	ferrous iron import	0.026	1	BP
GO:0070947	neutrophil mediated killing of fungus	0.026	1	BP
GO:0070953	regulation of neutrophil mediated killin...	0.026	1	BP
GO:0070965	positive regulation of neutrophil mediat...	0.026	1	BP
GO:0071231	cellular response to folic acid	0.026	1	BP
GO:0072301	regulation of metanephric glomerular mes...	0.026	1	BP
GO:0072303	positive regulation of glomerular metane...	0.026	1	BP
GO:0072564	blood microparticle formation	0.026	1	BP
GO:0072566	chemokine (C-X-C motif) ligand 1 product...	0.026	1	BP
GO:0090143	nucleoid organization	0.026	1	BP
GO:0090144	mitochondrial nucleoid organization	0.026	1	BP
GO:0097188	dentin mineralization	0.026	1	BP
GO:0098707	ferrous iron import across plasma membra...	0.026	1	BP
GO:0098758	response to interleukin-8	0.026	1	BP
GO:0098759	cellular response to interleukin-8	0.026	1	BP
GO:0110057	regulation of blood vessel endothelial c...	0.026	1	BP
GO:0110059	negative regulation of blood vessel endo...	0.026	1	BP
GO:0120054	intestinal motility	0.026	1	BP
GO:0120055	small intestinal transit	0.026	1	BP
GO:0120057	regulation of small intestinal transit	0.026	1	BP
GO:0120058	positive regulation of small intestinal ...	0.026	1	BP
GO:0150024	oxidised low-density lipoprotein particl...	0.026	1	BP
GO:1900369	negative regulation of RNA interference	0.026	1	BP
GO:1900390	regulation of iron ion import	0.026	1	BP
GO:1902203	negative regulation of hepatocyte growth...	0.026	1	BP
GO:1904438	regulation of ferrous iron import across...	0.026	1	BP
GO:1904439	negative regulation of ferrous iron impo...	0.026	1	BP
GO:1904983	glycine import into mitochondrion	0.026	1	BP
GO:1905222	atrioventricular canal morphogenesis	0.026	1	BP
GO:1905404	positive regulation of activated CD8-pos...	0.026	1	BP
GO:1905917	positive regulation of cell differentiat...	0.026	1	BP
GO:1905932	positive regulation of vascular smooth m...	0.026	1	BP
GO:1990758	mitotic sister chromatid biorientation	0.026	1	BP
GO:2000332	regulation of blood microparticle format...	0.026	1	BP
GO:2000334	positive regulation of blood micropartic...	0.026	1	BP
GO:2000338	regulation of chemokine (C-X-C motif) li...	0.026	1	BP
GO:2000340	positive regulation of chemokine (C-X-C ...	0.026	1	BP
GO:2001112	regulation of cellular response to hepat...	0.026	1	BP
GO:2001113	negative regulation of cellular response...	0.026	1	BP
GO:2001191	regulation of gamma-delta T cell activat...	0.026	1	BP

GO:2001193	positive regulation of gamma-delta T cel...	0.026	1	BP
GO:0032735	positive regulation of interleukin-12 pr...	0.026	1	BP
GO:0035886	vascular smooth muscle cell differentiat...	0.026	1	BP
GO:0006171	cAMP biosynthetic process	0.026	1	BP
GO:0045953	negative regulation of natural killer ce...	0.026	1	BP
GO:0046501	protoporphyrinogen IX metabolic process	0.026	1	BP
GO:0031670	cellular response to nutrient	0.026	1	BP
GO:0006213	pyrimidine nucleoside metabolic process	0.027	1	BP
GO:0045744	negative regulation of G protein-coupled...	0.027	1	BP
GO:0061383	trabecula morphogenesis	0.027	1	BP
GO:0048584	positive regulation of response to stimu...	0.027	1	BP
GO:0010035	response to inorganic substance	0.029	1	BP
GO:0001569	branching involved in blood vessel morph...	0.029	1	BP
GO:0003230	cardiac atrium development	0.029	1	BP
GO:0035909	aorta morphogenesis	0.029	1	BP
GO:0039528	cytoplasmic pattern recognition receptor...	0.029	1	BP
GO:0061844	antimicrobial humoral immune response me...	0.029	1	BP
GO:0009991	response to extracellular stimulus	0.029	1	BP
GO:0045766	positive regulation of angiogenesis	0.029	1	BP
GO:0046889	positive regulation of lipid biosyntheti...	0.030	1	BP
GO:0045834	positive regulation of lipid metabolic p...	0.030	1	BP
GO:0035295	tube development	0.031	1	BP
GO:0006954	inflammatory response	0.031	1	BP
GO:0043401	steroid hormone mediated signaling pathw...	0.031	1	BP
GO:0071407	cellular response to organic cyclic comp...	0.031	1	BP
GO:0002716	negative regulation of natural killer ce...	0.032	1	BP
GO:0006527	arginine catabolic process	0.032	1	BP
GO:0006991	response to sterol depletion	0.032	1	BP
GO:0030322	stabilization of membrane potential	0.032	1	BP
GO:0033147	negative regulation of intracellular est...	0.032	1	BP
GO:0039532	negative regulation of viral-induced cyt...	0.032	1	BP
GO:0043116	negative regulation of vascular permeabi...	0.032	1	BP
GO:0045026	plasma membrane fusion	0.032	1	BP
GO:0046007	negative regulation of activated T cell ...	0.032	1	BP
GO:0051447	negative regulation of meiotic cell cycl...	0.032	1	BP
GO:0061469	regulation of type B pancreatic cell pro...	0.032	1	BP
GO:0070213	protein auto-ADP-ribosylation	0.032	1	BP
GO:0070601	centromeric sister chromatid cohesion	0.032	1	BP
GO:0050878	regulation of body fluid levels	0.032	1	BP
GO:0038093	Fc receptor signaling pathway	0.032	1	BP
GO:0042775	mitochondrial ATP synthesis coupled elec...	0.032	1	BP
GO:0032648	regulation of interferon-beta production	0.033	1	BP
GO:0032418	lysosome localization	0.033	1	BP
GO:0042475	odontogenesis of dentin-containing tooth	0.033	1	BP
GO:1905954	positive regulation of lipid localizatio...	0.033	1	BP
GO:0022900	electron transport chain	0.033	1	BP
GO:0010033	response to organic substance	0.033	1	BP
GO:0008285	negative regulation of cell proliferatio...	0.033	1	BP
GO:0019216	regulation of lipid metabolic process	0.033	1	BP
GO:0042773	ATP synthesis coupled electron transport	0.034	1	BP
GO:0043900	regulation of multi-organism process	0.034	1	BP
GO:0043299	leukocyte degranulation	0.034	1	BP

GO:0071621	granulocyte chemotaxis	0.035	1	BP
GO:0030513	positive regulation of BMP signaling pat...	0.035	1	BP
GO:0035666	TRIF-dependent toll-like receptor signal...	0.035	1	BP
GO:0045601	regulation of endothelial cell different...	0.035	1	BP
GO:0051385	response to mineralocorticoid	0.035	1	BP
GO:0061036	positive regulation of cartilage develop...	0.035	1	BP
GO:0070887	cellular response to chemical stimulus	0.036	1	BP
GO:0032101	regulation of response to external stimu...	0.036	1	BP
GO:0051384	response to glucocorticoid	0.037	1	BP
GO:0071383	cellular response to steroid hormone sti...	0.037	1	BP
GO:0033108	mitochondrial respiratory chain complex ...	0.037	1	BP
GO:0000054	ribosomal subunit export from nucleus	0.037	1	BP
GO:0002834	regulation of response to tumor cell	0.037	1	BP
GO:0002837	regulation of immune response to tumor c...	0.037	1	BP
GO:0009111	vitamin catabolic process	0.037	1	BP
GO:0032352	positive regulation of hormone metabolic...	0.037	1	BP
GO:0033750	ribosome localization	0.037	1	BP
GO:0045187	regulation of circadian sleep/wake cycle...	0.037	1	BP
GO:0050665	hydrogen peroxide biosynthetic process	0.037	1	BP
GO:0070230	positive regulation of lymphocyte apopto...	0.037	1	BP
GO:0070486	leukocyte aggregation	0.037	1	BP
GO:2001185	regulation of CD8-positive, alpha-beta T...	0.037	1	BP
GO:0032608	interferon-beta production	0.038	1	BP
GO:0001990	regulation of systemic arterial blood pr...	0.038	1	BP
GO:0043304	regulation of mast cell degranulation	0.038	1	BP
GO:0061082	myeloid leukocyte cytokine production	0.038	1	BP
GO:0061384	heart trabecula morphogenesis	0.038	1	BP
GO:0050920	regulation of chemotaxis	0.039	1	BP
GO:0048660	regulation of smooth muscle cell prolife...	0.039	1	BP
GO:0030335	positive regulation of cell migration	0.040	1	BP
GO:0002886	regulation of myeloid leukocyte mediated...	0.040	1	BP
GO:0032720	negative regulation of tumor necrosis fa...	0.040	1	BP
GO:0006626	protein targeting to mitochondrion	0.040	1	BP
GO:0000288	nuclear-transcribed mRNA catabolic proce...	0.041	1	BP
GO:0003073	regulation of systemic arterial blood pr...	0.041	1	BP
GO:0019217	regulation of fatty acid metabolic proce...	0.041	1	BP
GO:0003203	endocardial cushion morphogenesis	0.042	1	BP
GO:0006471	protein ADP-ribosylation	0.042	1	BP
GO:0030857	negative regulation of epithelial cell d...	0.042	1	BP
GO:0033006	regulation of mast cell activation invol...	0.042	1	BP
GO:0042269	regulation of natural killer cell mediat...	0.042	1	BP
GO:0002181	cytoplasmic translation	0.042	1	BP
GO:0003014	renal system process	0.042	1	BP
GO:0048659	smooth muscle cell proliferation	0.043	1	BP
GO:0031663	lipopolysaccharide-mediated signaling pa...	0.043	1	BP
GO:0010935	regulation of macrophage cytokine produc...	0.043	1	BP
GO:0016553	base conversion or substitution editing	0.043	1	BP
GO:0051152	positive regulation of smooth muscle cel...	0.043	1	BP
GO:0060841	venous blood vessel development	0.043	1	BP
GO:0071428	rRNA-containing ribonucleoprotein comple...	0.043	1	BP
GO:1900029	positive regulation of ruffle assembly	0.043	1	BP
GO:1900225	regulation of NLRP3 inflammasome complex...	0.043	1	BP

GO:0034754	cellular hormone metabolic process	0.044	1	BP
GO:1990542	mitochondrial transmembrane transport	0.044	1	BP
GO:0030003	cellular cation homeostasis	0.044	1	BP
GO:0046148	pigment biosynthetic process	0.045	1	BP
GO:0050891	multicellular organismal water homeostas...	0.045	1	BP
GO:0097345	mitochondrial outer membrane permeabiliz...	0.045	1	BP
GO:0034641	cellular nitrogen compound metabolic pro...	0.045	1	BP
GO:0002707	negative regulation of lymphocyte mediat...	0.045	1	BP
GO:0048483	autonomic nervous system development	0.045	1	BP
GO:0060326	cell chemotaxis	0.047	1	BP
GO:0042476	odontogenesis	0.047	1	BP
GO:0002532	production of molecular mediator involve...	0.048	1	BP
GO:0050830	defense response to Gram-positive bacter...	0.048	1	BP
GO:0040017	positive regulation of locomotion	0.049	1	BP
GO:0002715	regulation of natural killer cell mediat...	0.049	1	BP
GO:0002756	MyD88-independent toll-like receptor sig...	0.049	1	BP
GO:0007094	mitotic spindle assembly checkpoint	0.049	1	BP
GO:0031577	spindle checkpoint	0.049	1	BP
GO:0033762	response to glucagon	0.049	1	BP
GO:0071173	spindle assembly checkpoint	0.049	1	BP
GO:0071174	mitotic spindle checkpoint	0.049	1	BP
GO:0060840	artery development	0.049	1	BP
GO:1901698	response to nitrogen compound	0.050	1	BP
GO:0001911	negative regulation of leukocyte mediate...	0.050	1	BP
GO:0001946	lymphangiogenesis	0.050	1	BP
GO:0002031	G protein-coupled receptor internalizati...	0.050	1	BP
GO:0002418	immune response to tumor cell	0.050	1	BP
GO:0003222	ventricular trabecula myocardium morphog...	0.050	1	BP
GO:0030540	female genitalia development	0.050	1	BP
GO:0032693	negative regulation of interleukin-10 pr...	0.050	1	BP
GO:0034134	toll-like receptor 2 signaling pathway	0.050	1	BP
GO:0044546	NLRP3 inflammasome complex assembly	0.050	1	BP
GO:0045091	regulation of single stranded viral RNA ...	0.050	1	BP
GO:0048485	sympathetic nervous system development	0.050	1	BP
GO:0048521	negative regulation of behavior	0.050	1	BP
GO:0072643	interferon-gamma secretion	0.050	1	BP
GO:2001028	positive regulation of endothelial cell ...	0.050	1	BP
GO:0017077	oxidative phosphorylation uncoupler acti...	0.001	1	MF
GO:0008097	5S rRNA binding	0.002	1	MF
GO:0003785	actin monomer binding	0.003	1	MF
GO:0035325	Toll-like receptor binding	0.003	1	MF
GO:0016655	oxidoreductase activity, acting on NAD(P...	0.004	1	MF
GO:0004957	prostaglandin E receptor activity	0.004	1	MF
GO:0016651	oxidoreductase activity, acting on NAD(P...	0.004	1	MF
GO:0017070	U6 snRNA binding	0.004	1	MF
GO:0004879	nuclear receptor activity	0.005	1	MF
GO:0098531	transcription factor activity, direct li...	0.005	1	MF
GO:0046914	transition metal ion binding	0.006	1	MF
GO:0034513	box H/ACA snoRNA binding	0.006	1	MF
GO:0035259	glucocorticoid receptor binding	0.007	1	MF
GO:1990446	U1 snRNP binding	0.009	1	MF
GO:0003707	steroid hormone receptor activity	0.010	1	MF

GO:0015232	heme transporter activity	0.013	1	MF
GO:0008198	ferrous iron binding	0.014	1	MF
GO:0009975	cyclase activity	0.014	1	MF
GO:0016849	phosphorus-oxygen lyase activity	0.014	1	MF
GO:0019239	deaminase activity	0.016	1	MF
GO:0050431	transforming growth factor beta binding	0.016	1	MF
GO:0051184	cofactor transmembrane transporter activ...	0.016	1	MF
GO:0004954	prostanoid receptor activity	0.017	1	MF
GO:0004955	prostaglandin receptor activity	0.017	1	MF
GO:0016813	hydrolase activity, acting on carbon-nit...	0.017	1	MF
GO:0070990	snRNP binding	0.017	1	MF
GO:0097371	MDM2/MDM4 family protein binding	0.017	1	MF
GO:0043021	ribonucleoprotein complex binding	0.018	1	MF
GO:0016427	tRNA (cytosine) methyltransferase activi...	0.021	1	MF
GO:0002953	5'-deoxynucleotidase activity	0.026	1	MF
GO:0003870	5-aminolevulinate synthase activity	0.026	1	MF
GO:0003948	N4-(beta-N-acetylglucosaminyl)-L-asparag...	0.026	1	MF
GO:0004037	allantoicase activity	0.026	1	MF
GO:0004051	arachidonate 5-lipoxygenase activity	0.026	1	MF
GO:0004371	glycerone kinase activity	0.026	1	MF
GO:0004632	phosphopantothenate--cysteine ligase act...	0.026	1	MF
GO:0004852	uroporphyrinogen-III synthase activity	0.026	1	MF
GO:0008403	25-hydroxycholecalciferol-24-hydroxylase...	0.026	1	MF
GO:0015105	arsenite transmembrane transporter activ...	0.026	1	MF
GO:0015439	heme-transporting ATPase activity	0.026	1	MF
GO:0016608	growth hormone-releasing hormone activit...	0.026	1	MF
GO:0016749	N-succinyltransferase activity	0.026	1	MF
GO:0019153	protein-disulfide reductase (glutathione...	0.026	1	MF
GO:0030107	HLA-A specific inhibitory MHC class I re...	0.026	1	MF
GO:0030109	HLA-B specific inhibitory MHC class I re...	0.026	1	MF
GO:0030342	1-alpha,25-dihydroxyvitamin D3 24-hydrox...	0.026	1	MF
GO:0030345	structural constituent of tooth enamel	0.026	1	MF
GO:0031700	adrenomedullin receptor binding	0.026	1	MF
GO:0031768	ghrelin receptor binding	0.026	1	MF
GO:0031859	platelet activating factor receptor bind...	0.026	1	MF
GO:0033862	UMP kinase activity	0.026	1	MF
GO:0034012	FAD-AMP lyase (cyclizing) activity	0.026	1	MF
GO:0036004	GAF domain binding	0.026	1	MF
GO:0036328	VEGF-C-activated receptor activity	0.026	1	MF
GO:0036373	L-fucose mutarotase activity	0.026	1	MF
GO:0043250	sodium-dependent organic anion transmemb...	0.026	1	MF
GO:0044105	L-xylulose reductase (NAD+) activity	0.026	1	MF
GO:0046525	xylosylprotein 4-beta-galactosyltransfer...	0.026	1	MF
GO:0047844	deoxycytidine deaminase activity	0.026	1	MF
GO:0047915	ganglioside galactosyltransferase activi...	0.026	1	MF
GO:0050038	L-xylulose reductase (NADP+) activity	0.026	1	MF
GO:0050354	triokinase activity	0.026	1	MF
GO:0052751	GDP-mannose hydrolase activity	0.026	1	MF
GO:0061632	RNA lariat debranching enzyme activator ...	0.026	1	MF
GO:0061714	folic acid receptor activity	0.026	1	MF
GO:0070037	rRNA (pseudouridine) methyltransferase a...	0.026	1	MF
GO:0070287	ferritin receptor activity	0.026	1	MF

GO:0070892	lipoteichoic acid receptor activity	0.026	1	MF
GO:0071885	N-terminal protein N-methyltransferase a...	0.026	1	MF
GO:0102008	cytosolic dipeptidase activity	0.026	1	MF
GO:0103046	alanylglutamate dipeptidase activity	0.026	1	MF
GO:0140260	mitochondrial proton-transporting ATP sy...	0.026	1	MF
GO:0150025	oxidised low-density lipoprotein particl...	0.026	1	MF
GO:1990930	RNA N1-methyladenosine dioxygenase activ...	0.026	1	MF
GO:0004016	adenylate cyclase activity	0.026	1	MF
GO:0016868	intramolecular transferase activity, pho...	0.026	1	MF
GO:0008028	monocarboxylic acid transmembrane transp...	0.026	1	MF
GO:0016229	steroid dehydrogenase activity	0.028	1	MF
GO:0030515	snoRNA binding	0.028	1	MF
GO:0017069	snRNA binding	0.030	1	MF
GO:0031386	protein tag	0.031	1	MF
GO:0005507	copper ion binding	0.036	1	MF
GO:0004953	icosanoid receptor activity	0.036	1	MF
GO:0015125	bile acid transmembrane transporter acti...	0.036	1	MF
GO:0022840	leak channel activity	0.036	1	MF
GO:0022842	narrow pore channel activity	0.036	1	MF
GO:0043023	ribosomal large subunit binding	0.036	1	MF
GO:0005085	guanyl-nucleotide exchange factor activi...	0.039	1	MF
GO:0003725	double-stranded RNA binding	0.039	1	MF
GO:0097617	annealing activity	0.042	1	MF
GO:0003735	structural constituent of ribosome	0.048	1	MF
GO:0044548	S100 protein binding	0.049	1	MF
GO:0048185	activin binding	0.049	1	MF
GO:0038024	cargo receptor activity	0.050	1	MF
GO:0120115	Lsm2-8 complex	0.000	0.02779	CC
GO:0005688	U6 snRNP	0.000	0.17865	CC
GO:0032420	stereocilium	0.001	0.628583	CC
GO:0032421	stereocilium bundle	0.002	0.744375	CC
GO:0005732	small nucleolar ribonucleoprotein comple...	0.003	0.940394	CC
GO:0035976	transcription factor AP-1 complex	0.004	0.940394	CC
GO:0072589	box H/ACA scaRNP complex	0.004	0.940394	CC
GO:0090661	box H/ACA telomerase RNP complex	0.004	0.940394	CC
GO:0098862	cluster of actin-based cell projections	0.005	1	CC
GO:1990904	ribonucleoprotein complex	0.005	1	CC
GO:0031429	box H/ACA snoRNP complex	0.006	1	CC
GO:1990726	Lsm1-7-Pat1 complex	0.006	1	CC
GO:0005747	mitochondrial respiratory chain complex ...	0.008	1	CC
GO:0030964	NADH dehydrogenase complex	0.008	1	CC
GO:0045271	respiratory chain complex I	0.008	1	CC
GO:0042788	polysomal ribosome	0.009	1	CC
GO:0072588	box H/ACA RNP complex	0.009	1	CC
GO:0015934	large ribosomal subunit	0.010	1	CC
GO:0045335	phagocytic vesicle	0.014	1	CC
GO:0098798	mitochondrial protein complex	0.015	1	CC
GO:0098803	respiratory chain complex	0.015	1	CC
GO:0016459	myosin complex	0.015	1	CC
GO:0098800	inner mitochondrial membrane protein com...	0.018	1	CC
GO:0005746	mitochondrial respiratory chain	0.019	1	CC
GO:0046540	U4/U6 x U5 tri-snRNP complex	0.019	1	CC

GO:0097526	spliceosomal tri-snRNP complex	0.019	1	CC
GO:0005889	potassium:proton exchanging ATPase compl...	0.026	1	CC
GO:0005914	spot adherens junction	0.026	1	CC
GO:0014804	terminal cisterna lumen	0.026	1	CC
GO:0036398	TCR signalosome	0.026	1	CC
GO:0043529	GET complex	0.026	1	CC
GO:0070176	DRM complex	0.026	1	CC
GO:0005576	extracellular region	0.029	1	CC
GO:0001726	ruffle	0.030	1	CC
GO:0070469	respiratory chain	0.031	1	CC
GO:0015629	actin cytoskeleton	0.033	1	CC
GO:0031966	mitochondrial membrane	0.034	1	CC
GO:0005844	polysome	0.035	1	CC
GO:0000940	condensed chromosome outer kinetochore	0.036	1	CC
GO:0071005	U2-type precatalytic spliceosome	0.039	1	CC
GO:0005743	mitochondrial inner membrane	0.040	1	CC
GO:0002102	podosome	0.041	1	CC
GO:0071011	precatalytic spliceosome	0.041	1	CC
GO:0000778	condensed nuclear chromosome kinetochore	0.042	1	CC
GO:0005681	spliceosomal complex	0.042	1	CC
GO:0044391	ribosomal subunit	0.046	1	CC
GO:1990204	oxidoreductase complex	0.047	1	CC
GO:0005859	muscle myosin complex	0.049	1	CC
GO:0042599	lamellar body	0.049	1	CC

Nac

GO.ID	Term	FET	BH	Category
GO:0009109	coenzyme catabolic process	0.000	1	BP
GO:0036474	cell death in response to hydrogen perox...	0.000	1	BP
GO:0051187	cofactor catabolic process	0.000	1	BP
GO:0006488	dolichol-linked oligosaccharide biosynth...	0.001	1	BP
GO:0045017	glycerolipid biosynthetic process	0.001	1	BP
GO:0006490	oligosaccharide-lipid intermediate biosy...	0.001	1	BP
GO:0009111	vitamin catabolic process	0.001	1	BP
GO:0043666	regulation of phosphoprotein phosphatase...	0.001	1	BP
GO:0046474	glycerophospholipid biosynthetic process	0.001	1	BP
GO:0033603	positive regulation of dopamine secretio...	0.001	1	BP
GO:0008654	phospholipid biosynthetic process	0.001	1	BP
GO:0006644	phospholipid metabolic process	0.001	1	BP
GO:0033280	response to vitamin D	0.001	1	BP
GO:0046337	phosphatidylethanolamine metabolic proce...	0.001	1	BP
GO:0034342	response to type III interferon	0.002	1	BP
GO:0042365	water-soluble vitamin catabolic process	0.002	1	BP
GO:1990708	conditioned place preference	0.002	1	BP
GO:0070085	glycosylation	0.002	1	BP
GO:0035304	regulation of protein dephosphorylation	0.002	1	BP
GO:0032225	regulation of synaptic transmission, dop...	0.002	1	BP
GO:0034356	NAD biosynthesis via nicotinamide ribosi...	0.002	1	BP
GO:0001963	synaptic transmission, dopaminergic	0.002	1	BP
GO:0006470	protein dephosphorylation	0.002	1	BP
GO:0009100	glycoprotein metabolic process	0.002	1	BP
GO:0042398	cellular modified amino acid biosyntheti...	0.002	1	BP
GO:0006750	glutathione biosynthetic process	0.002	1	BP
GO:1901032	negative regulation of response to react...	0.002	1	BP
GO:1903206	negative regulation of hydrogen peroxide...	0.002	1	BP
GO:2001039	negative regulation of cellular response...	0.002	1	BP
GO:0006486	protein glycosylation	0.002	1	BP
GO:0043413	macromolecule glycosylation	0.002	1	BP
GO:0090045	positive regulation of deacetylase activ...	0.003	1	BP
GO:0016311	dephosphorylation	0.003	1	BP
GO:0022603	regulation of anatomical structure morph...	0.003	1	BP
GO:0006629	lipid metabolic process	0.004	1	BP
GO:0019184	nonribosomal peptide biosynthetic proces...	0.004	1	BP
GO:1902176	negative regulation of oxidative stress-...	0.004	1	BP
GO:0044255	cellular lipid metabolic process	0.004	1	BP
GO:0010635	regulation of mitochondrial fusion	0.004	1	BP
GO:0018342	protein prenylation	0.004	1	BP
GO:0090336	positive regulation of brown fat cell di...	0.004	1	BP
GO:0097354	prenylation	0.004	1	BP
GO:0042369	vitamin D catabolic process	0.005	1	BP
GO:0045213	neurotransmitter receptor metabolic proc...	0.005	1	BP
GO:0060486	Clara cell differentiation	0.005	1	BP
GO:1903750	regulation of intrinsic apoptotic signal...	0.005	1	BP
GO:1903751	negative regulation of intrinsic apoptot...	0.005	1	BP
GO:2000466	negative regulation of glycogen (starch)...	0.005	1	BP
GO:0048268	clathrin coat assembly	0.005	1	BP
GO:0007411	axon guidance	0.005	1	BP

GO:0060969	negative regulation of gene silencing	0.005	1	BP
GO:0097485	neuron projection guidance	0.005	1	BP
GO:0050951	sensory perception of temperature stimul...	0.006	1	BP
GO:0019233	sensory perception of pain	0.006	1	BP
GO:2001243	negative regulation of intrinsic apoptot...	0.006	1	BP
GO:0006650	glycerophospholipid metabolic process	0.006	1	BP
GO:1902883	negative regulation of response to oxida...	0.007	1	BP
GO:2001234	negative regulation of apoptotic signali...	0.007	1	BP
GO:1901031	regulation of response to reactive oxyge...	0.007	1	BP
GO:0031952	regulation of protein autophosphorylatio...	0.007	1	BP
GO:0060425	lung morphogenesis	0.007	1	BP
GO:0043066	negative regulation of apoptotic process	0.008	1	BP
GO:0010827	regulation of glucose transmembrane tran...	0.008	1	BP
GO:0038003	opioid receptor signaling pathway	0.008	1	BP
GO:0060339	negative regulation of type I interferon...	0.008	1	BP
GO:0061469	regulation of type B pancreatic cell pro...	0.008	1	BP
GO:0150065	regulation of deacetylase activity	0.008	1	BP
GO:1903205	regulation of hydrogen peroxide-induced ...	0.008	1	BP
GO:0008610	lipid biosynthetic process	0.009	1	BP
GO:0018197	peptidyl-aspartic acid modification	0.009	1	BP
GO:0036476	neuron death in response to hydrogen per...	0.009	1	BP
GO:0051344	negative regulation of cyclic-nucleotide...	0.009	1	BP
GO:0060510	type II pneumocyte differentiation	0.009	1	BP
GO:1901748	leukotriene D4 metabolic process	0.009	1	BP
GO:1901750	leukotriene D4 biosynthetic process	0.009	1	BP
GO:1903207	regulation of hydrogen peroxide-induced ...	0.009	1	BP
GO:1903208	negative regulation of hydrogen peroxide...	0.009	1	BP
GO:0006370	7-methylguanosine mRNA capping	0.009	1	BP
GO:0046486	glycerolipid metabolic process	0.009	1	BP
GO:0012501	programmed cell death	0.010	1	BP
GO:0034219	carbohydrate transmembrane transport	0.010	1	BP
GO:0009452	7-methylguanosine RNA capping	0.010	1	BP
GO:0036260	RNA capping	0.010	1	BP
GO:0033605	positive regulation of catecholamine sec...	0.010	1	BP
GO:0043069	negative regulation of programmed cell d...	0.011	1	BP
GO:0010677	negative regulation of cellular carbohyd...	0.012	1	BP
GO:0014046	dopamine secretion	0.012	1	BP
GO:0014059	regulation of dopamine secretion	0.012	1	BP
GO:2001038	regulation of cellular response to drug	0.012	1	BP
GO:0110053	regulation of actin filament organizatio...	0.012	1	BP
GO:0097502	mannosylation	0.013	1	BP
GO:0070863	positive regulation of protein exit from...	0.013	1	BP
GO:0090335	regulation of brown fat cell differentia...	0.013	1	BP
GO:0051952	regulation of amine transport	0.014	1	BP
GO:2001023	regulation of response to drug	0.014	1	BP
GO:0009101	glycoprotein biosynthetic process	0.014	1	BP
GO:0006751	glutathione catabolic process	0.014	1	BP
GO:0014050	negative regulation of glutamate secreti...	0.014	1	BP
GO:0036481	intrinsic apoptotic signaling pathway in...	0.014	1	BP
GO:0043435	response to corticotropin-releasing horm...	0.014	1	BP
GO:0071376	cellular response to corticotropin-relea...	0.014	1	BP
GO:1901299	negative regulation of hydrogen peroxide...	0.014	1	BP

GO:0060548	negative regulation of cell death	0.015	1	BP
GO:0070268	cornification	0.015	1	BP
GO:0031346	positive regulation of cell projection o...	0.015	1	BP
GO:0008219	cell death	0.015	1	BP
GO:0050433	regulation of catecholamine secretion	0.015	1	BP
GO:1902175	regulation of oxidative stress-induced i...	0.016	1	BP
GO:2001024	negative regulation of response to drug	0.016	1	BP
GO:0010829	negative regulation of glucose transmemb...	0.016	1	BP
GO:0014048	regulation of glutamate secretion	0.016	1	BP
GO:0030836	positive regulation of actin filament de...	0.016	1	BP
GO:0031936	negative regulation of chromatin silenci...	0.016	1	BP
GO:0046341	CDP-diacylglycerol metabolic process	0.016	1	BP
GO:0050961	detection of temperature stimulus involv...	0.016	1	BP
GO:0050965	detection of temperature stimulus involv...	0.016	1	BP
GO:0090190	positive regulation of branching involve...	0.016	1	BP
GO:0072595	maintenance of protein localization in o...	0.016	1	BP
GO:0045666	positive regulation of neuron differenti...	0.016	1	BP
GO:0050432	catecholamine secretion	0.018	1	BP
GO:1904659	glucose transmembrane transport	0.018	1	BP
GO:0048566	embryonic digestive tract development	0.018	1	BP
GO:0006497	protein lipidation	0.019	1	BP
GO:0015837	amine transport	0.019	1	BP
GO:2001233	regulation of apoptotic signaling pathwa...	0.019	1	BP
GO:0043113	receptor clustering	0.019	1	BP
GO:0006646	phosphatidylethanolamine biosynthetic pr...	0.020	1	BP
GO:0015936	coenzyme A metabolic process	0.020	1	BP
GO:0045618	positive regulation of keratinocyte diff...	0.020	1	BP
GO:0061213	positive regulation of mesonephros devel...	0.020	1	BP
GO:0090141	positive regulation of mitochondrial fis...	0.020	1	BP
GO:0090189	regulation of branching involved in uret...	0.020	1	BP
GO:0010976	positive regulation of neuron projection...	0.020	1	BP
GO:0060761	negative regulation of response to cytok...	0.021	1	BP
GO:0010637	negative regulation of mitochondrial fus...	0.021	1	BP
GO:0032226	positive regulation of synaptic transmis...	0.021	1	BP
GO:0032277	negative regulation of gonadotropin secr...	0.021	1	BP
GO:0033578	protein glycosylation in Golgi	0.021	1	BP
GO:0033869	nucleoside bisphosphate catabolic proces...	0.021	1	BP
GO:0034031	ribonucleoside bisphosphate catabolic pr...	0.021	1	BP
GO:0034034	purine nucleoside bisphosphate catabolic...	0.021	1	BP
GO:0045719	negative regulation of glycogen biosynth...	0.021	1	BP
GO:0046826	negative regulation of protein export fr...	0.021	1	BP
GO:0061419	positive regulation of transcription fro...	0.021	1	BP
GO:0070874	negative regulation of glycogen metaboli...	0.021	1	BP
GO:0072526	pyridine-containing compound catabolic p...	0.021	1	BP
GO:1901727	positive regulation of histone deacetyla...	0.021	1	BP
GO:1903979	negative regulation of microglial cell a...	0.021	1	BP
GO:1903998	regulation of eating behavior	0.021	1	BP
GO:2000465	regulation of glycogen (starch) synthase...	0.021	1	BP
GO:0008645	hexose transmembrane transport	0.022	1	BP
GO:0008643	carbohydrate transport	0.022	1	BP
GO:0097164	ammonium ion metabolic process	0.022	1	BP
GO:0002832	negative regulation of response to bioti...	0.022	1	BP

GO:0045912	negative regulation of carbohydrate meta...	0.022	1	BP
GO:1900408	negative regulation of cellular response...	0.022	1	BP
GO:1903202	negative regulation of oxidative stress-...	0.022	1	BP
GO:0035303	regulation of dephosphorylation	0.022	1	BP
GO:0019886	antigen processing and presentation of e...	0.023	1	BP
GO:0042158	lipoprotein biosynthetic process	0.023	1	BP
GO:0033198	response to ATP	0.023	1	BP
GO:0061036	positive regulation of cartilage develop...	0.023	1	BP
GO:1905476	negative regulation of protein localizat...	0.023	1	BP
GO:0061217	regulation of mesonephros development	0.023	1	BP
GO:0071545	inositol phosphate catabolic process	0.023	1	BP
GO:0015749	monosaccharide transmembrane transport	0.024	1	BP
GO:0042982	amyloid precursor protein metabolic proc...	0.025	1	BP
GO:0045815	positive regulation of gene expression, ...	0.025	1	BP
GO:0036003	positive regulation of transcription fro...	0.026	1	BP
GO:1905332	positive regulation of morphogenesis of ...	0.026	1	BP
GO:0010921	regulation of phosphatase activity	0.026	1	BP
GO:0051898	negative regulation of protein kinase B ...	0.027	1	BP
GO:0051668	localization within membrane	0.027	1	BP
GO:0006915	apoptotic process	0.027	1	BP
GO:0002495	antigen processing and presentation of p...	0.028	1	BP
GO:0036473	cell death in response to oxidative stre...	0.029	1	BP
GO:0007600	sensory perception	0.029	1	BP
GO:0006489	dolichyl diphosphate biosynthetic proces...	0.029	1	BP
GO:0009134	nucleoside diphosphate catabolic process	0.029	1	BP
GO:0016926	protein desumoylation	0.029	1	BP
GO:0018344	protein geranylgeranylation	0.029	1	BP
GO:0032276	regulation of gonadotropin secretion	0.029	1	BP
GO:0042439	ethanolamine-containing compound metabol...	0.029	1	BP
GO:0046465	dolichyl diphosphate metabolic process	0.029	1	BP
GO:0048245	eosinophil chemotaxis	0.029	1	BP
GO:0061140	lung secretory cell differentiation	0.029	1	BP
GO:0061668	mitochondrial ribosome assembly	0.029	1	BP
GO:0071499	cellular response to laminar fluid shear...	0.029	1	BP
GO:1900045	negative regulation of protein K63-linke...	0.029	1	BP
GO:2000253	positive regulation of feeding behavior	0.029	1	BP
GO:0010811	positive regulation of cell-substrate ad...	0.029	1	BP
GO:0015872	dopamine transport	0.029	1	BP
GO:0061001	regulation of dendritic spine morphogene...	0.029	1	BP
GO:0002504	antigen processing and presentation of p...	0.029	1	BP
GO:0007015	actin filament organization	0.029	1	BP
GO:0006362	transcription elongation from RNA polyme...	0.029	1	BP
GO:0051954	positive regulation of amine transport	0.029	1	BP
GO:0046777	protein autophosphorylation	0.030	1	BP
GO:0006796	phosphate-containing compound metabolic ...	0.031	1	BP
GO:0061035	regulation of cartilage development	0.031	1	BP
GO:1901616	organic hydroxy compound catabolic proce...	0.031	1	BP
GO:0006793	phosphorus metabolic process	0.031	1	BP
GO:0045824	negative regulation of innate immune res...	0.032	1	BP
GO:0043900	regulation of multi-organism process	0.032	1	BP
GO:0010801	negative regulation of peptidyl-threonin...	0.032	1	BP
GO:0016048	detection of temperature stimulus	0.032	1	BP

GO:0032332	positive regulation of chondrocyte diffe...	0.032	1	BP
GO:0035269	protein O-linked mannosylation	0.032	1	BP
GO:0042053	regulation of dopamine metabolic process	0.032	1	BP
GO:0042069	regulation of catecholamine metabolic pr...	0.032	1	BP
GO:0070828	heterochromatin organization	0.032	1	BP
GO:0071498	cellular response to fluid shear stress	0.032	1	BP
GO:1902882	regulation of response to oxidative stre...	0.032	1	BP
GO:0006363	termination of RNA polymerase I transcri...	0.033	1	BP
GO:0009154	purine ribonucleotide catabolic process	0.033	1	BP
GO:0031935	regulation of chromatin silencing	0.033	1	BP
GO:0051930	regulation of sensory perception of pain	0.033	1	BP
GO:0043524	negative regulation of neuron apoptotic ...	0.034	1	BP
GO:0008543	fibroblast growth factor receptor signal...	0.034	1	BP
GO:0031424	keratinization	0.034	1	BP
GO:0044273	sulfur compound catabolic process	0.034	1	BP
GO:0060688	regulation of morphogenesis of a branchi...	0.034	1	BP
GO:0042221	response to chemical	0.035	1	BP
GO:0090407	organophosphate biosynthetic process	0.035	1	BP
GO:0032956	regulation of actin cytoskeleton organiz...	0.035	1	BP
GO:0009261	ribonucleotide catabolic process	0.036	1	BP
GO:0045684	positive regulation of epidermis develop...	0.036	1	BP
GO:0050873	brown fat cell differentiation	0.036	1	BP
GO:0051931	regulation of sensory perception	0.036	1	BP
GO:0034764	positive regulation of transmembrane tra...	0.036	1	BP
GO:0044342	type B pancreatic cell proliferation	0.037	1	BP
GO:0051497	negative regulation of stress fiber asse...	0.037	1	BP
GO:1901881	positive regulation of protein depolymer...	0.037	1	BP
GO:0007042	lysosomal lumen acidification	0.037	1	BP
GO:0043922	negative regulation by host of viral tra...	0.037	1	BP
GO:0051342	regulation of cyclic-nucleotide phosphod...	0.037	1	BP
GO:0071609	chemokine (C-C motif) ligand 5 productio...	0.037	1	BP
GO:1904714	regulation of chaperone-mediated autopha...	0.037	1	BP
GO:1905247	positive regulation of aspartic-type pep...	0.037	1	BP
GO:0046434	organophosphate catabolic process	0.038	1	BP
GO:0051937	catecholamine transport	0.038	1	BP
GO:0030218	erythrocyte differentiation	0.038	1	BP
GO:0030968	endoplasmic reticulum unfolded protein r...	0.039	1	BP
GO:0042157	lipoprotein metabolic process	0.039	1	BP
GO:0033273	response to vitamin	0.039	1	BP
GO:0006643	membrane lipid metabolic process	0.039	1	BP
GO:0007409	axonogenesis	0.039	1	BP
GO:0043112	receptor metabolic process	0.039	1	BP
GO:0001192	maintenance of transcriptional fidelity ...	0.040	1	BP
GO:0001193	maintenance of transcriptional fidelity ...	0.040	1	BP
GO:0002017	regulation of blood volume by renal aldo...	0.040	1	BP
GO:0002041	intussusceptive angiogenesis	0.040	1	BP
GO:0002264	endothelial cell activation involved in ...	0.040	1	BP
GO:0002380	immunoglobulin secretion involved in imm...	0.040	1	BP
GO:0002509	central tolerance induction to self anti...	0.040	1	BP
GO:0002785	negative regulation of antimicrobial pep...	0.040	1	BP
GO:0002787	negative regulation of antibacterial pep...	0.040	1	BP
GO:0003247	post-embryonic cardiac muscle cell growt...	0.040	1	BP

GO:0006386	termination of RNA polymerase III transc...	0.040	1	BP
GO:0006580	ethanolamine metabolic process	0.040	1	BP
GO:0008348	negative regulation of antimicrobial hum...	0.040	1	BP
GO:0009184	purine deoxyribonucleoside diphosphate c...	0.040	1	BP
GO:0009192	deoxyribonucleoside diphosphate cataboli...	0.040	1	BP
GO:0009444	pyruvate oxidation	0.040	1	BP
GO:0010335	response to non-ionic osmotic stress	0.040	1	BP
GO:0010904	regulation of UDP-glucose catabolic proc...	0.040	1	BP
GO:0010905	negative regulation of UDP-glucose catab...	0.040	1	BP
GO:0014822	detection of wounding	0.040	1	BP
GO:0015938	coenzyme A catabolic process	0.040	1	BP
GO:0016243	regulation of autophagosome size	0.040	1	BP
GO:0017190	N-terminal peptidyl-aspartic acid acetyl...	0.040	1	BP
GO:0018106	peptidyl-histidine phosphorylation	0.040	1	BP
GO:0018217	peptidyl-aspartic acid phosphorylation	0.040	1	BP
GO:0018277	protein deamination	0.040	1	BP
GO:0019357	nicotinate nucleotide biosynthetic proce...	0.040	1	BP
GO:0019358	nicotinate nucleotide salvage	0.040	1	BP
GO:0019365	pyridine nucleotide salvage	0.040	1	BP
GO:0021771	lateral geniculate nucleus development	0.040	1	BP
GO:0031940	positive regulation of chromatin silenci...	0.040	1	BP
GO:0032223	negative regulation of synaptic transmis...	0.040	1	BP
GO:0032361	pyridoxal phosphate catabolic process	0.040	1	BP
GO:0032690	negative regulation of interleukin-1 alp...	0.040	1	BP
GO:0034355	NAD salvage	0.040	1	BP
GO:0034971	histone H3-R17 methylation	0.040	1	BP
GO:0035752	lysosomal lumen pH elevation	0.040	1	BP
GO:0036114	medium-chain fatty-acyl-CoA catabolic pr...	0.040	1	BP
GO:0036116	long-chain fatty-acyl-CoA catabolic proc...	0.040	1	BP
GO:0036255	response to methylamine	0.040	1	BP
GO:0038097	positive regulation of mast cell activat...	0.040	1	BP
GO:0042820	vitamin B6 catabolic process	0.040	1	BP
GO:0043048	dolichyl monophosphate biosynthetic proc...	0.040	1	BP
GO:0044333	Wnt signaling pathway involved in digest...	0.040	1	BP
GO:0045212	neurotransmitter receptor biosynthetic p...	0.040	1	BP
GO:0045234	protein palmitoleylation	0.040	1	BP
GO:0045951	positive regulation of mitotic recombina...	0.040	1	BP
GO:0046057	dADP catabolic process	0.040	1	BP
GO:0046067	dGDP catabolic process	0.040	1	BP
GO:0046497	nicotinate nucleotide metabolic process	0.040	1	BP
GO:0046657	folic acid catabolic process	0.040	1	BP
GO:0046712	GDP catabolic process	0.040	1	BP
GO:0048567	ectodermal digestive tract morphogenesis	0.040	1	BP
GO:0048613	embryonic ectodermal digestive tract mor...	0.040	1	BP
GO:0048874	host-mediated regulation of intestinal m...	0.040	1	BP
GO:0051041	positive regulation of calcium-independe...	0.040	1	BP
GO:0051232	meiotic spindle elongation	0.040	1	BP
GO:0051413	response to cortisone	0.040	1	BP
GO:0052550	response to defense-related reactive oxy...	0.040	1	BP
GO:0052567	response to defense-related host reactiv...	0.040	1	BP
GO:0055108	Golgi to transport vesicle transport	0.040	1	BP
GO:0060234	neuroblast delamination	0.040	1	BP

GO:0060302	negative regulation of cytokine activity	0.040	1	BP
GO:0060461	right lung morphogenesis	0.040	1	BP
GO:0060668	regulation of branching involved in sali...	0.040	1	BP
GO:0062025	regulation of SCF-dependent proteasomal ...	0.040	1	BP
GO:0062026	negative regulation of SCF-dependent pro...	0.040	1	BP
GO:0062030	negative regulation of stress granule as...	0.040	1	BP
GO:0070105	positive regulation of interleukin-6-med...	0.040	1	BP
GO:0071454	cellular response to anoxia	0.040	1	BP
GO:0071471	cellular response to non-ionic osmotic s...	0.040	1	BP
GO:0090044	positive regulation of tubulin deacetyla...	0.040	1	BP
GO:0090362	positive regulation of platelet-derived ...	0.040	1	BP
GO:0090402	oncogene-induced cell senescence	0.040	1	BP
GO:0097166	lens epithelial cell proliferation	0.040	1	BP
GO:0097298	regulation of nucleus size	0.040	1	BP
GO:0097376	interneuron axon guidance	0.040	1	BP
GO:0097536	thymus epithelium morphogenesis	0.040	1	BP
GO:0099627	neurotransmitter receptor cycle	0.040	1	BP
GO:0099630	postsynaptic neurotransmitter receptor c...	0.040	1	BP
GO:0106047	polyamine deacetylation	0.040	1	BP
GO:0106048	spermidine deacetylation	0.040	1	BP
GO:1900369	negative regulation of RNA interference	0.040	1	BP
GO:1901609	negative regulation of vesicle transport...	0.040	1	BP
GO:1901627	negative regulation of postsynaptic memb...	0.040	1	BP
GO:1902684	negative regulation of receptor localiza...	0.040	1	BP
GO:1903165	response to polycyclic arene	0.040	1	BP
GO:1903259	exon-exon junction complex disassembly	0.040	1	BP
GO:1903442	response to lipoic acid	0.040	1	BP
GO:1903570	regulation of protein kinase D signaling	0.040	1	BP
GO:1903572	positive regulation of protein kinase D ...	0.040	1	BP
GO:1903610	regulation of calcium-dependent ATPase a...	0.040	1	BP
GO:1903611	negative regulation of calcium-dependent...	0.040	1	BP
GO:1903627	regulation of dUTP diphosphatase activit...	0.040	1	BP
GO:1903629	positive regulation of dUTP diphosphatas...	0.040	1	BP
GO:1903631	negative regulation of aminoacyl-tRNA li...	0.040	1	BP
GO:1903633	regulation of leucine-tRNA ligase activi...	0.040	1	BP
GO:1903634	negative regulation of leucine-tRNA liga...	0.040	1	BP
GO:1903743	negative regulation of anterograde synap...	0.040	1	BP
GO:1903788	positive regulation of glutathione biosy...	0.040	1	BP
GO:1903910	negative regulation of receptor clusteri...	0.040	1	BP
GO:1903965	monounsaturated fatty acid catabolic pro...	0.040	1	BP
GO:1904226	regulation of glycogen synthase activity...	0.040	1	BP
GO:1904227	negative regulation of glycogen synthase...	0.040	1	BP
GO:1904394	negative regulation of skeletal muscle a...	0.040	1	BP
GO:1904530	negative regulation of actin filament bi...	0.040	1	BP
GO:1904542	regulation of free ubiquitin chain polym...	0.040	1	BP
GO:1904544	positive regulation of free ubiquitin ch...	0.040	1	BP
GO:1904617	negative regulation of actin binding	0.040	1	BP
GO:1904681	response to 3-methylcholanthrene	0.040	1	BP
GO:1904692	positive regulation of type B pancreatic...	0.040	1	BP
GO:1904784	NLRP1 inflammasome complex assembly	0.040	1	BP
GO:1904806	regulation of protein oxidation	0.040	1	BP
GO:1904808	positive regulation of protein oxidation	0.040	1	BP

GO:1904983	glycine import into mitochondrion	0.040	1	BP
GO:1905547	regulation of telomeric heterochromatin ...	0.040	1	BP
GO:1905549	positive regulation of telomeric heteroc...	0.040	1	BP
GO:1905673	positive regulation of lysosome organiza...	0.040	1	BP
GO:1990134	epithelial cell apoptotic process involv...	0.040	1	BP
GO:1990386	mitotic cleavage furrow ingression	0.040	1	BP
GO:1990938	peptidyl-aspartic acid autophosphorylati...	0.040	1	BP
GO:2000056	regulation of Wnt signaling pathway invo...	0.040	1	BP
GO:2000057	negative regulation of Wnt signaling pat...	0.040	1	BP
GO:2000795	negative regulation of epithelial cell p...	0.040	1	BP
GO:2001109	regulation of lens epithelial cell proli...	0.040	1	BP
GO:2001111	positive regulation of lens epithelial c...	0.040	1	BP
GO:2001151	regulation of renal water transport	0.040	1	BP
GO:2001153	positive regulation of renal water trans...	0.040	1	BP
GO:0018345	protein palmitoylation	0.040	1	BP
GO:2000648	positive regulation of stem cell prolife...	0.040	1	BP
GO:0000731	DNA synthesis involved in DNA repair	0.040	1	BP
GO:0010810	regulation of cell-substrate adhesion	0.040	1	BP
GO:0050877	nervous system process	0.041	1	BP
GO:0043901	negative regulation of multi-organism pr...	0.042	1	BP
GO:0007035	vacuolar acidification	0.043	1	BP
GO:2000352	negative regulation of endothelial cell ...	0.043	1	BP
GO:0070301	cellular response to hydrogen peroxide	0.043	1	BP
GO:0050906	detection of stimulus involved in sensor...	0.044	1	BP
GO:0006654	phosphatidic acid biosynthetic process	0.044	1	BP
GO:0008216	spermidine metabolic process	0.047	1	BP
GO:0010269	response to selenium ion	0.047	1	BP
GO:0042363	fat-soluble vitamin catabolic process	0.047	1	BP
GO:0043117	positive regulation of vascular permeabi...	0.047	1	BP
GO:0046185	aldehyde catabolic process	0.047	1	BP
GO:0048570	notochord morphogenesis	0.047	1	BP
GO:0051231	spindle elongation	0.047	1	BP
GO:0070561	vitamin D receptor signaling pathway	0.047	1	BP
GO:1901298	regulation of hydrogen peroxide-mediated...	0.047	1	BP
GO:1901725	regulation of histone deacetylase activi...	0.047	1	BP
GO:1902915	negative regulation of protein polyubiqu...	0.047	1	BP
GO:2000121	regulation of removal of superoxide radi...	0.047	1	BP
GO:0006367	transcription initiation from RNA polyme...	0.047	1	BP
GO:0050896	response to stimulus	0.047	1	BP
GO:0001953	negative regulation of cell-matrix adhes...	0.048	1	BP
GO:0006361	transcription initiation from RNA polyme...	0.048	1	BP
GO:0043243	positive regulation of protein complex d...	0.048	1	BP
GO:0046473	phosphatidic acid metabolic process	0.048	1	BP
GO:0090313	regulation of protein targeting to membr...	0.048	1	BP
GO:0045606	positive regulation of epidermal cell di...	0.048	1	BP
GO:0046697	decidualization	0.048	1	BP
GO:0051457	maintenance of protein location in nucle...	0.048	1	BP
GO:0060259	regulation of feeding behavior	0.048	1	BP
GO:0061003	positive regulation of dendritic spine m...	0.048	1	BP
GO:0090312	positive regulation of protein deacetyla...	0.048	1	BP
GO:1903861	positive regulation of dendrite extensio...	0.048	1	BP
GO:1904738	vascular associated smooth muscle cell m...	0.048	1	BP

GO:1904752	regulation of vascular associated smooth...	0.048	1	BP
GO:0044282	small molecule catabolic process	0.049	1	BP
GO:0048469	cell maturation	0.049	1	BP
GO:0019208	phosphatase regulator activity	0.000	0.98978	MF
GO:0017110	nucleoside-diphosphatase activity	0.001	1	MF
GO:0019888	protein phosphatase regulator activity	0.001	1	MF
GO:0003824	catalytic activity	0.001	1	MF
GO:0001691	pseudophosphatase activity	0.002	1	MF
GO:0004577	N-acetylglucosaminyldiphosphodolichol N-...	0.002	1	MF
GO:0016492	G protein-coupled neurotensin receptor a...	0.002	1	MF
GO:0008318	protein prenyltransferase activity	0.002	1	MF
GO:0004659	prenyltransferase activity	0.002	1	MF
GO:0030145	manganese ion binding	0.003	1	MF
GO:0004985	opioid receptor activity	0.003	1	MF
GO:0019211	phosphatase activator activity	0.003	1	MF
GO:0035259	glucocorticoid receptor binding	0.003	1	MF
GO:0000048	peptidyltransferase activity	0.005	1	MF
GO:0000293	ferric-chelate reductase activity	0.005	1	MF
GO:0001093	TFIIB-class transcription factor binding	0.005	1	MF
GO:0004104	cholinesterase activity	0.005	1	MF
GO:0036374	glutathione hydrolase activity	0.005	1	MF
GO:0102953	hypoglycin A gamma-glutamyl transpeptida...	0.005	1	MF
GO:0103068	leukotriene C4 gamma-glutamyl transferas...	0.005	1	MF
GO:0016791	phosphatase activity	0.006	1	MF
GO:0030548	acetylcholine receptor regulator activit...	0.006	1	MF
GO:0099602	neurotransmitter receptor regulator acti...	0.006	1	MF
GO:0030276	clathrin binding	0.007	1	MF
GO:0008194	UDP-glycosyltransferase activity	0.007	1	MF
GO:0016740	transferase activity	0.008	1	MF
GO:0016755	transferase activity, transferring amino...	0.008	1	MF
GO:0042577	lipid phosphatase activity	0.008	1	MF
GO:0004305	ethanolamine kinase activity	0.009	1	MF
GO:0004663	Rab geranylgeranyltransferase activity	0.009	1	MF
GO:0043125	ErbB-3 class receptor binding	0.009	1	MF
GO:0042578	phosphoric ester hydrolase activity	0.010	1	MF
GO:0004553	hydrolase activity, hydrolyzing O-glycos...	0.013	1	MF
GO:0042165	neurotransmitter binding	0.013	1	MF
GO:0016594	glycine binding	0.014	1	MF
GO:0072542	protein phosphatase activator activity	0.014	1	MF
GO:0001784	phosphotyrosine residue binding	0.014	1	MF
GO:0017147	Wnt-protein binding	0.014	1	MF
GO:0000995	RNA polymerase III general transcription...	0.015	1	MF
GO:0003831	beta-N-acetylglucosaminylglycopeptide be...	0.015	1	MF
GO:0004661	protein geranylgeranyltransferase activi...	0.015	1	MF
GO:0034235	GPI anchor binding	0.015	1	MF
GO:0034711	inhibin binding	0.015	1	MF
GO:0070290	N-acylphosphatidylethanolamine-specific ...	0.015	1	MF
GO:0046982	protein heterodimerization activity	0.016	1	MF
GO:0016787	hydrolase activity	0.017	1	MF
GO:0016798	hydrolase activity, acting on glycosyl b...	0.019	1	MF
GO:0046965	retinoid X receptor binding	0.020	1	MF
GO:0140096	catalytic activity, acting on a protein	0.020	1	MF

GO:0055131	C3HC4-type RING finger domain binding	0.022	1	MF
GO:0016788	hydrolase activity, acting on ester bond...	0.025	1	MF
GO:0019212	phosphatase inhibitor activity	0.025	1	MF
GO:0016722	oxidoreductase activity, oxidizing metal...	0.029	1	MF
GO:0005549	odorant binding	0.029	1	MF
GO:0032051	clathrin light chain binding	0.029	1	MF
GO:0016757	transferase activity, transferring glyco...	0.030	1	MF
GO:0004879	nuclear receptor activity	0.030	1	MF
GO:0098531	transcription factor activity, direct li...	0.030	1	MF
GO:0046914	transition metal ion binding	0.032	1	MF
GO:0005242	inward rectifier potassium channel activ...	0.033	1	MF
GO:0017049	GTP-Rho binding	0.033	1	MF
GO:0140223	general transcription initiation factor ...	0.033	1	MF
GO:0008140	cAMP response element binding protein bi...	0.038	1	MF
GO:0016723	oxidoreductase activity, oxidizing metal...	0.038	1	MF
GO:0045309	protein phosphorylated amino acid bindin...	0.039	1	MF
GO:0000773	phosphatidyl-N-methylethanolamine N-meth...	0.040	1	MF
GO:0001532	interleukin-21 receptor activity	0.040	1	MF
GO:0001626	nociceptin receptor activity	0.040	1	MF
GO:0001642	group III metabotropic glutamate recepto...	0.040	1	MF
GO:0003881	CDP-diacylglycerol-inositol 3-phosphatid...	0.040	1	MF
GO:0003905	alkylbase DNA N-glycosylase activity	0.040	1	MF
GO:0004108	citrate (Si)-synthase activity	0.040	1	MF
GO:0004168	dolichol kinase activity	0.040	1	MF
GO:0004341	gluconolactonase activity	0.040	1	MF
GO:0004363	glutathione synthase activity	0.040	1	MF
GO:0004375	glycine dehydrogenase (decarboxylating) ...	0.040	1	MF
GO:0004498	calcidiol 1-monooxygenase activity	0.040	1	MF
GO:0004516	nicotinate phosphoribosyltransferase act...	0.040	1	MF
GO:0004608	phosphatidylethanolamine N-methyltransfe...	0.040	1	MF
GO:0004616	phosphogluconate dehydrogenase (decarbox...	0.040	1	MF
GO:0004673	protein histidine kinase activity	0.040	1	MF
GO:0004686	elongation factor-2 kinase activity	0.040	1	MF
GO:0004766	spermidine synthase activity	0.040	1	MF
GO:0004883	glucocorticoid receptor activity	0.040	1	MF
GO:0004997	thyrotropin-releasing hormone receptor a...	0.040	1	MF
GO:0005519	cytoskeletal regulatory protein binding	0.040	1	MF
GO:0008112	nicotinamide N-methyltransferase activit...	0.040	1	MF
GO:0008403	25-hydroxycholecalciferol-24-hydroxylase...	0.040	1	MF
GO:0008448	N-acetylglucosamine-6-phosphate deacetyl...	0.040	1	MF
GO:0008725	DNA-3-methyladenine glycosylase activity	0.040	1	MF
GO:0008894	guanosine-5'-triphosphate,3'-diphosphate...	0.040	1	MF
GO:0015067	amidinotransferase activity	0.040	1	MF
GO:0015068	glycine amidinotransferase activity	0.040	1	MF
GO:0015538	sialic acid:proton symporter activity	0.040	1	MF
GO:0016642	oxidoreductase activity, acting on the C...	0.040	1	MF
GO:0030272	5-formyltetrahydrofolate cyclo-ligase ac...	0.040	1	MF
GO:0030342	1-alpha,25-dihydroxyvitamin D3 24-hydrox...	0.040	1	MF
GO:0030527	structural constituent of chromatin	0.040	1	MF
GO:0030549	acetylcholine receptor activator activit...	0.040	1	MF
GO:0030760	pyridine N-methyltransferase activity	0.040	1	MF
GO:0031737	CX3C chemokine receptor binding	0.040	1	MF

GO:0033744	L-methionine:thioredoxin-disulfide S-oxi...	0.040	1	MF
GO:0033919	glucan 1,3-alpha-glucosidase activity	0.040	1	MF
GO:0034649	histone demethylase activity (H3-monomet...	0.040	1	MF
GO:0035642	histone methyltransferase activity (H3-R...	0.040	1	MF
GO:0035755	cardiolipin hydrolase activity	0.040	1	MF
GO:0036440	citrate synthase activity	0.040	1	MF
GO:0036456	L-methionine-(S)-S-oxide reductase activ...	0.040	1	MF
GO:0038048	dynorphin receptor activity	0.040	1	MF
GO:0042010	interleukin-15 receptor activity	0.040	1	MF
GO:0042131	thiamine phosphate phosphatase activity	0.040	1	MF
GO:0043733	DNA-3-methylbase glycosylase activity	0.040	1	MF
GO:0043916	DNA-7-methylguanine glycosylase activity	0.040	1	MF
GO:0044716	8-oxo-GDP phosphatase activity	0.040	1	MF
GO:0044717	8-hydroxy-dADP phosphatase activity	0.040	1	MF
GO:0045237	CXCR1 chemokine receptor binding	0.040	1	MF
GO:0046525	xylosylprotein 4-beta-galactosyltransfer...	0.040	1	MF
GO:0047419	N-acetylgalactosamine-6-phosphate deacet...	0.040	1	MF
GO:0047453	ATP-dependent NAD(P)H-hydrate dehydratas...	0.040	1	MF
GO:0047609	acetylputrescine deacetylase activity	0.040	1	MF
GO:0047611	acetylspermidine deacetylase activity	0.040	1	MF
GO:0051722	protein C-terminal methylesterase activi...	0.040	1	MF
GO:0051723	protein methylesterase activity	0.040	1	MF
GO:0052821	DNA-7-methyladenine glycosylase activity	0.040	1	MF
GO:0052822	DNA-3-methylguanine glycosylase activity	0.040	1	MF
GO:0052855	ADP-dependent NAD(P)H-hydrate dehydratas...	0.040	1	MF
GO:0052925	dol-P-Man:Man(5)GlcNAc(2)-PP-Dol alpha-1...	0.040	1	MF
GO:0070280	pyridoxal binding	0.040	1	MF
GO:0072555	17-beta-ketosteroid reductase activity	0.040	1	MF
GO:0072582	17-beta-hydroxysteroid dehydrogenase (NA...	0.040	1	MF
GO:0080101	phosphatidyl-N-dimethylethanolamine N-me...	0.040	1	MF
GO:0086060	voltage-gated sodium channel activity in...	0.040	1	MF
GO:0086061	voltage-gated sodium channel activity in...	0.040	1	MF
GO:0086063	voltage-gated sodium channel activity in...	0.040	1	MF
GO:0090600	alpha-1,3-glucosidase activity	0.040	1	MF
GO:0102200	N-acetylphosphatidylethanolamine-hydroly...	0.040	1	MF
GO:0120153	calcium-dependent carbohydrate binding	0.040	1	MF
GO:1990583	phospholipase D activator activity	0.040	1	MF
GO:0004721	phosphoprotein phosphatase activity	0.041	1	MF
GO:0016765	transferase activity, transferring alkyl...	0.042	1	MF
GO:0016746	transferase activity, transferring acyl ...	0.045	1	MF
GO:0003993	acid phosphatase activity	0.048	1	MF
GO:0004439	phosphatidylinositol-4,5-bisphosphate 5-...	0.048	1	MF
GO:0008242	omega peptidase activity	0.048	1	MF
GO:0015295	solute:proton symporter activity	0.050	1	MF
GO:0031224	intrinsic component of membrane	0.001	0.621967	CC
GO:0044425	membrane part	0.001	0.621967	CC
GO:0016021	integral component of membrane	0.001	0.621967	CC
GO:0031984	organelle subcompartment	0.004	1	CC
GO:0030672	synaptic vesicle membrane	0.006	1	CC
GO:0099501	exocytic vesicle membrane	0.006	1	CC
GO:0031252	cell leading edge	0.006	1	CC
GO:0030027	lamellipodium	0.008	1	CC

GO:0030658	transport vesicle membrane	0.008	1	CC
GO:0044194	cytolytic granule	0.009	1	CC
GO:0005802	trans-Golgi network	0.009	1	CC
GO:0070382	exocytic vesicle	0.012	1	CC
GO:0008021	synaptic vesicle	0.013	1	CC
GO:0098574	cytoplasmic side of lysosomal membrane	0.014	1	CC
GO:0042175	nuclear outer membrane-endoplasmic retic...	0.015	1	CC
GO:0005789	endoplasmic reticulum membrane	0.015	1	CC
GO:0098827	endoplasmic reticulum subcompartment	0.017	1	CC
GO:0150034	distal axon	0.019	1	CC
GO:0016020	membrane	0.019	1	CC
GO:0098992	neuronal dense core vesicle	0.021	1	CC
GO:0044432	endoplasmic reticulum part	0.024	1	CC
GO:0035578	azurophil granule lumen	0.028	1	CC
GO:0030133	transport vesicle	0.028	1	CC
GO:0098835	presynaptic endocytic zone membrane	0.029	1	CC
GO:0005794	Golgi apparatus	0.030	1	CC
GO:0005766	primary lysosome	0.031	1	CC
GO:0042582	azurophil granule	0.031	1	CC
GO:0000159	protein phosphatase type 2A complex	0.032	1	CC
GO:0005775	vacuolar lumen	0.034	1	CC
GO:0030132	clathrin coat of coated pit	0.037	1	CC
GO:0098843	postsynaptic endocytic zone	0.037	1	CC
GO:0014801	longitudinal sarcoplasmic reticulum	0.039	1	CC
GO:0016938	kinesin I complex	0.039	1	CC
GO:0043513	inhibin B complex	0.039	1	CC
GO:0070516	CAK-ERCC2 complex	0.039	1	CC
GO:0031968	organelle outer membrane	0.040	1	CC
GO:0005741	mitochondrial outer membrane	0.040	1	CC
GO:0000786	nucleosome	0.040	1	CC
GO:0019867	outer membrane	0.042	1	CC
GO:0098793	presynapse	0.043	1	CC
GO:0099503	secretory vesicle	0.044	1	CC
GO:0098833	presynaptic endocytic zone	0.046	1	CC

vSUB

GO.ID	Term	FET	BH	Category
GO:0023052	signaling	2.1E-17	2.74E-13	BP
GO:0007154	cell communication	3.5E-17	2.74E-13	BP
GO:0007268	chemical synaptic transmission	5.4E-15	2.11E-11	BP
GO:0098916	anterograde trans-synaptic signaling	5.4E-15	2.11E-11	BP
GO:0099537	trans-synaptic signaling	1E-14	3.13E-11	BP
GO:0099536	synaptic signaling	1.7E-14	4.44E-11	BP
GO:0007267	cell-cell signaling	3.6E-14	8.05E-11	BP
GO:0032879	regulation of localization	2.4E-12	4.7E-09	BP
GO:0023051	regulation of signaling	7.3E-12	1.27E-08	BP
GO:0007165	signal transduction	1E-11	1.44E-08	BP
GO:0050808	synapse organization	1.1E-11	1.44E-08	BP
GO:0051179	localization	1.1E-11	1.44E-08	BP
GO:0000902	cell morphogenesis	1.4E-11	1.69E-08	BP
GO:0032989	cellular component morphogenesis	2.4E-11	2.68E-08	BP
GO:0050896	response to stimulus	7.2E-11	7.52E-08	BP
GO:0010646	regulation of cell communication	1.2E-10	1.17E-07	BP
GO:0009653	anatomical structure morphogenesis	1.3E-10	1.2E-07	BP
GO:0048731	system development	1.8E-10	1.57E-07	BP
GO:0007166	cell surface receptor signaling pathway	2.5E-10	2.06E-07	BP
GO:0006811	ion transport	6.3E-10	4.93E-07	BP
GO:0007399	nervous system development	1.3E-09	9.69E-07	BP
GO:0006812	cation transport	1.6E-09	1.09E-06	BP
GO:0050804	modulation of chemical synaptic transmis...	1.6E-09	1.09E-06	BP
GO:0099177	regulation of trans-synaptic signaling	1.7E-09	1.11E-06	BP
GO:0040011	locomotion	2.1E-09	1.28E-06	BP
GO:0043269	regulation of ion transport	2.2E-09	1.28E-06	BP
GO:0051239	regulation of multicellular organismal p...	2.2E-09	1.28E-06	BP
GO:0000904	cell morphogenesis involved in different...	2.6E-09	1.45E-06	BP
GO:0032501	multicellular organismal process	4.8E-09	2.59E-06	BP
GO:0032990	cell part morphogenesis	5E-09	2.61E-06	BP
GO:0065008	regulation of biological quality	5.4E-09	2.73E-06	BP
GO:0048869	cellular developmental process	5.7E-09	2.79E-06	BP
GO:0007275	multicellular organism development	5.9E-09	2.8E-06	BP
GO:0051716	cellular response to stimulus	6.3E-09	2.9E-06	BP
GO:0120039	plasma membrane bounded cell projection ...	7.9E-09	3.53E-06	BP
GO:0048858	cell projection morphogenesis	1E-08	4.35E-06	BP
GO:0006810	transport	1.1E-08	4.65E-06	BP
GO:0006928	movement of cell or subcellular componen...	1.2E-08	4.94E-06	BP
GO:0051234	establishment of localization	1.3E-08	5.22E-06	BP
GO:0022603	regulation of anatomical structure morph...	1.6E-08	6.26E-06	BP
GO:0048856	anatomical structure development	1.7E-08	6.49E-06	BP
GO:0048699	generation of neurons	1.9E-08	7.08E-06	BP
GO:0016477	cell migration	2.1E-08	7.65E-06	BP
GO:2000026	regulation of multicellular organismal d...	2.7E-08	9.39E-06	BP
GO:0048167	regulation of synaptic plasticity	2.7E-08	9.39E-06	BP
GO:0048666	neuron development	3.4E-08	1.13E-05	BP
GO:0048468	cell development	3.4E-08	1.13E-05	BP
GO:0055085	transmembrane transport	3.5E-08	1.14E-05	BP
GO:0051049	regulation of transport	4.3E-08	1.35E-05	BP
GO:0022008	neurogenesis	4.3E-08	1.35E-05	BP

GO:0022610	biological adhesion	4.4E-08	1.35E-05	BP
GO:0030182	neuron differentiation	4.5E-08	1.35E-05	BP
GO:0048870	cell motility	4.8E-08	1.39E-05	BP
GO:0051674	localization of cell	4.8E-08	1.39E-05	BP
GO:0048812	neuron projection morphogenesis	5.2E-08	1.48E-05	BP
GO:0007155	cell adhesion	7.4E-08	2.07E-05	BP
GO:0030154	cell differentiation	8.4E-08	2.31E-05	BP
GO:0099173	postsynapse organization	1.3E-07	3.39E-05	BP
GO:0051270	regulation of cellular component movemen...	1.3E-07	3.39E-05	BP
GO:0032502	developmental process	1.3E-07	3.39E-05	BP
GO:0048667	cell morphogenesis involved in neuron di...	1.5E-07	3.85E-05	BP
GO:2000145	regulation of cell motility	1.6E-07	3.98E-05	BP
GO:0023056	positive regulation of signaling	1.6E-07	3.98E-05	BP
GO:0031175	neuron projection development	2E-07	4.89E-05	BP
GO:0009966	regulation of signal transduction	2.1E-07	5.06E-05	BP
GO:0050803	regulation of synapse structure or activ...	2.3E-07	5.46E-05	BP
GO:0010647	positive regulation of cell communicatio...	2.4E-07	5.61E-05	BP
GO:0050793	regulation of developmental process	2.8E-07	6.45E-05	BP
GO:0034762	regulation of transmembrane transport	2.9E-07	6.49E-05	BP
GO:0048583	regulation of response to stimulus	2.9E-07	6.49E-05	BP
GO:0050807	regulation of synapse organization	3.3E-07	7.28E-05	BP
GO:0030334	regulation of cell migration	3.4E-07	7.39E-05	BP
GO:0022604	regulation of cell morphogenesis	4.2E-07	9.01E-05	BP
GO:0030001	metal ion transport	4.5E-07	9.52E-05	BP
GO:0034220	ion transmembrane transport	4.9E-07	0.000102	BP
GO:0040012	regulation of locomotion	5.8E-07	0.000119	BP
GO:0098655	cation transmembrane transport	9E-07	0.000183	BP
GO:0065009	regulation of molecular function	1.2E-06	0.000238	BP
GO:0050806	positive regulation of synaptic transmis...	1.2E-06	0.000238	BP
GO:2000310	regulation of NMDA receptor activity	1.3E-06	0.000254	BP
GO:0034765	regulation of ion transmembrane transpor...	1.4E-06	0.000271	BP
GO:0099601	regulation of neurotransmitter receptor ...	1.8E-06	0.000344	BP
GO:0048168	regulation of neuronal synaptic plastici...	0.000002	0.000377	BP
GO:0022898	regulation of transmembrane transporter ...	2.2E-06	0.00041	BP
GO:1900449	regulation of glutamate receptor signali...	2.4E-06	0.000442	BP
GO:0120036	plasma membrane bounded cell projection ...	3.1E-06	0.000564	BP
GO:0032412	regulation of ion transmembrane transpor...	3.8E-06	0.000684	BP
GO:0007215	glutamate receptor signaling pathway	4.3E-06	0.000756	BP
GO:0065007	biological regulation	4.3E-06	0.000756	BP
GO:0006816	calcium ion transport	4.4E-06	0.000765	BP
GO:0007611	learning or memory	4.5E-06	0.000774	BP
GO:0030030	cell projection organization	0.000005	0.000849	BP
GO:0006935	chemotaxis	5.1E-06	0.000849	BP
GO:2001257	regulation of cation channel activity	5.1E-06	0.000849	BP
GO:0032409	regulation of transporter activity	5.4E-06	0.000881	BP
GO:0035637	multicellular organismal signaling	5.4E-06	0.000881	BP
GO:0042330	taxis	5.6E-06	0.000904	BP
GO:0061564	axon development	5.8E-06	0.000927	BP
GO:0098657	import into cell	0.000007	0.001107	BP
GO:0009611	response to wounding	7.1E-06	0.001112	BP
GO:0032940	secretion by cell	8.3E-06	0.001287	BP
GO:0035556	intracellular signal transduction	8.4E-06	0.001289	BP

GO:1904062	regulation of cation transmembrane trans...	9.2E-06	0.001398	BP
GO:0007610	behavior	9.7E-06	0.00146	BP
GO:0009605	response to external stimulus	0.000012	0.001789	BP
GO:0051960	regulation of nervous system development	0.000013	0.00192	BP
GO:0048813	dendrite morphogenesis	0.000015	0.002195	BP
GO:0007416	synapse assembly	0.000016	0.00232	BP
GO:0050890	cognition	0.000019	0.002704	BP
GO:0042221	response to chemical	0.000019	0.002704	BP
GO:0099003	vesicle-mediated transport in synapse	0.00002	0.002796	BP
GO:0007409	axonogenesis	0.00002	0.002796	BP
GO:0098609	cell-cell adhesion	0.000023	0.003104	BP
GO:0003008	system process	0.000023	0.003104	BP
GO:0010469	regulation of signaling receptor activit...	0.000023	0.003104	BP
GO:1903779	regulation of cardiac conduction	0.000023	0.003104	BP
GO:0048513	animal organ development	0.000026	0.003479	BP
GO:0050790	regulation of catalytic activity	0.000027	0.003583	BP
GO:0051963	regulation of synapse assembly	0.000028	0.003623	BP
GO:0050789	regulation of biological process	0.000028	0.003623	BP
GO:1901342	regulation of vasculature development	0.000028	0.003623	BP
GO:0070887	cellular response to chemical stimulus	0.000033	0.004201	BP
GO:0046903	secretion	0.000033	0.004201	BP
GO:0043085	positive regulation of catalytic activit...	0.000035	0.004419	BP
GO:1902531	regulation of intracellular signal trans...	0.00004	0.00501	BP
GO:0044093	positive regulation of molecular functio...	0.000045	0.005592	BP
GO:0051336	regulation of hydrolase activity	0.00005	0.006164	BP
GO:0070838	divalent metal ion transport	0.000051	0.00619	BP
GO:0007169	transmembrane receptor protein tyrosine ...	0.000051	0.00619	BP
GO:0050877	nervous system process	0.000057	0.006865	BP
GO:0051668	localization within membrane	0.000059	0.006998	BP
GO:0031290	retinal ganglion cell axon guidance	0.000059	0.006998	BP
GO:0099175	regulation of postsynapse organization	0.000062	0.007299	BP
GO:0001505	regulation of neurotransmitter levels	0.000064	0.007478	BP
GO:0051241	negative regulation of multicellular org...	0.000067	0.007771	BP
GO:0072511	divalent inorganic cation transport	0.000068	0.007829	BP
GO:0051240	positive regulation of multicellular org...	0.00007	0.008	BP
GO:0007167	enzyme linked receptor protein signaling...	0.000071	0.008055	BP
GO:0006836	neurotransmitter transport	0.000076	0.008439	BP
GO:0021952	central nervous system projection neuron...	0.000076	0.008439	BP
GO:0009967	positive regulation of signal transducti...	0.000076	0.008439	BP
GO:1905475	regulation of protein localization to me...	0.000086	0.009482	BP
GO:0099072	regulation of postsynaptic membrane neur...	0.000087	0.009526	BP
GO:0048729	tissue morphogenesis	0.000093	0.010112	BP
GO:0030036	actin cytoskeleton organization	0.000094	0.01015	BP
GO:0071310	cellular response to organic substance	0.000098	0.010438	BP
GO:0051094	positive regulation of developmental pro...	0.000098	0.010438	BP
GO:0072176	nephric duct development	0.0001	0.010579	BP
GO:0046777	protein autophosphorylation	0.00012	0.012526	BP
GO:0007423	sensory organ development	0.00012	0.012526	BP
GO:0023061	signal release	0.00013	0.013391	BP
GO:0006897	endocytosis	0.00013	0.013391	BP
GO:0051056	regulation of small GTPase mediated sign...	0.00018	0.018066	BP
GO:0030029	actin filament-based process	0.00018	0.018066	BP

GO:0015672	monovalent inorganic cation transport	0.00018	0.018066	BP
GO:0048522	positive regulation of cellular process	0.00018	0.018066	BP
GO:0060284	regulation of cell development	0.00019	0.018828	BP
GO:0045595	regulation of cell differentiation	0.00019	0.018828	BP
GO:0051924	regulation of calcium ion transport	0.0002	0.019694	BP
GO:0045765	regulation of angiogenesis	0.00021	0.02055	BP
GO:1903530	regulation of secretion by cell	0.00022	0.021395	BP
GO:0048489	synaptic vesicle transport	0.00023	0.021435	BP
GO:0097480	establishment of synaptic vesicle locali...	0.00023	0.021435	BP
GO:0072178	nephric duct morphogenesis	0.00023	0.021435	BP
GO:0050767	regulation of neurogenesis	0.00023	0.021435	BP
GO:0006865	amino acid transport	0.00023	0.021435	BP
GO:0061337	cardiac conduction	0.00023	0.021435	BP
GO:0021675	nerve development	0.00023	0.021435	BP
GO:0060291	long-term synaptic potentiation	0.00024	0.022235	BP
GO:0045664	regulation of neuron differentiation	0.00026	0.023668	BP
GO:0006887	exocytosis	0.00026	0.023668	BP
GO:0001568	blood vessel development	0.00026	0.023668	BP
GO:0050679	positive regulation of epithelial cell p...	0.00027	0.024295	BP
GO:0072358	cardiovascular system development	0.00027	0.024295	BP
GO:0016192	vesicle-mediated transport	0.00028	0.025051	BP
GO:0099590	neurotransmitter receptor internalizatio...	0.00029	0.025798	BP
GO:0007411	axon guidance	0.0003	0.026537	BP
GO:0097485	neuron projection guidance	0.00032	0.028147	BP
GO:0045055	regulated exocytosis	0.00033	0.028865	BP
GO:0021954	central nervous system neuron developmen...	0.00034	0.029411	BP
GO:0048172	regulation of short-term neuronal synapt...	0.00034	0.029411	BP
GO:0045576	mast cell activation	0.00035	0.03011	BP
GO:0009887	animal organ morphogenesis	0.00036	0.030633	BP
GO:0002252	immune effector process	0.00036	0.030633	BP
GO:0007605	sensory perception of sound	0.00037	0.031146	BP
GO:0001944	vasculature development	0.00037	0.031146	BP
GO:0010033	response to organic substance	0.00038	0.03148	BP
GO:0002230	positive regulation of defense response ...	0.00038	0.03148	BP
GO:0035235	ionotropic glutamate receptor signaling ...	0.00038	0.03148	BP
GO:0010959	regulation of metal ion transport	0.00039	0.032138	BP
GO:0015800	acidic amino acid transport	0.0004	0.032619	BP
GO:0051965	positive regulation of synapse assembly	0.0004	0.032619	BP
GO:0016079	synaptic vesicle exocytosis	0.00041	0.032752	BP
GO:0050794	regulation of cellular process	0.00041	0.032752	BP
GO:0048514	blood vessel morphogenesis	0.00041	0.032752	BP
GO:0035239	tube morphogenesis	0.00041	0.032752	BP
GO:0051345	positive regulation of hydrolase activit...	0.00042	0.033212	BP
GO:0035249	synaptic transmission, glutamatergic	0.00042	0.033212	BP
GO:0018108	peptidyl-tyrosine phosphorylation	0.00043	0.033495	BP
GO:0014047	glutamate secretion	0.00043	0.033495	BP
GO:0043303	mast cell degranulation	0.00043	0.033495	BP
GO:0001818	negative regulation of cytokine producti...	0.00044	0.034104	BP
GO:0010769	regulation of cell morphogenesis involve...	0.00045	0.034538	BP
GO:0052548	regulation of endopeptidase activity	0.00045	0.034538	BP
GO:1901652	response to peptide	0.00046	0.035133	BP
GO:0048518	positive regulation of biological proces...	0.00047	0.035722	BP

GO:0071804	cellular potassium ion transport	0.00048	0.035787	BP
GO:0071805	potassium ion transmembrane transport	0.00048	0.035787	BP
GO:0051093	negative regulation of developmental pro...	0.00048	0.035787	BP
GO:0021602	cranial nerve morphogenesis	0.00048	0.035787	BP
GO:0007274	neuromuscular synaptic transmission	0.00049	0.03585	BP
GO:0008045	motor neuron axon guidance	0.00049	0.03585	BP
GO:1905809	negative regulation of synapse organizat...	0.00049	0.03585	BP
GO:0016358	dendrite development	0.00049	0.03585	BP
GO:0018212	peptidyl-tyrosine modification	0.0005	0.036412	BP
GO:0002279	mast cell activation involved in immune ...	0.00051	0.036798	BP
GO:0099504	synaptic vesicle cycle	0.00051	0.036798	BP
GO:0050954	sensory perception of mechanical stimulu...	0.00053	0.037891	BP
GO:0097479	synaptic vesicle localization	0.00053	0.037891	BP
GO:0002376	immune system process	0.00054	0.038085	BP
GO:0048488	synaptic vesicle endocytosis	0.00054	0.038085	BP
GO:0140238	presynaptic endocytosis	0.00054	0.038085	BP
GO:0051272	positive regulation of cellular componen...	0.00055	0.038103	BP
GO:0043542	endothelial cell migration	0.00055	0.038103	BP
GO:0060322	head development	0.00055	0.038103	BP
GO:0048584	positive regulation of response to stimu...	0.00055	0.038103	BP
GO:0098662	inorganic cation transmembrane transport	0.00058	0.040005	BP
GO:0010975	regulation of neuron projection developm...	0.00059	0.040339	BP
GO:0048589	developmental growth	0.00059	0.040339	BP
GO:0002448	mast cell mediated immunity	0.0006	0.040492	BP
GO:0021955	central nervous system neuron axonogenes...	0.0006	0.040492	BP
GO:0051046	regulation of secretion	0.0006	0.040492	BP
GO:0002696	positive regulation of leukocyte activat...	0.00061	0.040642	BP
GO:0030335	positive regulation of cell migration	0.00061	0.040642	BP
GO:0042060	wound healing	0.00061	0.040642	BP
GO:0010594	regulation of endothelial cell migration	0.00062	0.041133	BP
GO:0001525	angiogenesis	0.00064	0.042281	BP
GO:0010634	positive regulation of epithelial cell m...	0.00067	0.043528	BP
GO:0007156	homophilic cell adhesion via plasma memb...	0.00067	0.043528	BP
GO:1904018	positive regulation of vasculature devel...	0.00067	0.043528	BP
GO:0017156	calcium ion regulated exocytosis	0.00067	0.043528	BP
GO:0019221	cytokine-mediated signaling pathway	0.00072	0.046391	BP
GO:0002009	morphogenesis of an epithelium	0.00072	0.046391	BP
GO:0032970	regulation of actin filament-based proce...	0.00073	0.046843	BP
GO:0035295	tube development	0.00074	0.047098	BP
GO:0030073	insulin secretion	0.00074	0.047098	BP
GO:0051128	regulation of cellular component organiz...	0.00076	0.047788	BP
GO:0030072	peptide hormone secretion	0.00076	0.047788	BP
GO:0001667	ameboidal-type cell migration	0.00076	0.047788	BP
GO:0048169	regulation of long-term neuronal synapti...	0.00078	0.048655	BP
GO:0006955	immune response	0.00078	0.048655	BP
GO:0035524	proline transmembrane transport	0.0008	0.049705	BP
GO:1902533	positive regulation of intracellular sig...	0.00081	0.050127	BP
GO:0007264	small GTPase mediated signal transductio...	0.00083	0.051163	BP
GO:0010631	epithelial cell migration	0.00085	0.05219	BP
GO:0010595	positive regulation of endothelial cell ...	0.00086	0.052393	BP
GO:0006820	anion transport	0.00086	0.052393	BP
GO:0032880	regulation of protein localization	0.00087	0.052797	BP

GO:0098660	inorganic ion transmembrane transport	0.00088	0.053198	BP
GO:0090132	epithelium migration	0.0009	0.054197	BP
GO:0001938	positive regulation of endothelial cell ...	0.00092	0.055189	BP
GO:0120035	regulation of plasma membrane bounded ce...	0.00097	0.057967	BP
GO:0048013	ephrin receptor signaling pathway	0.00102	0.060723	BP
GO:0048646	anatomical structure formation involved ...	0.00107	0.063219	BP
GO:0045321	leukocyte activation	0.00107	0.063219	BP
GO:0051656	establishment of organelle localization	0.0011	0.064504	BP
GO:0032956	regulation of actin cytoskeleton organiz...	0.0011	0.064504	BP
GO:0050867	positive regulation of cell activation	0.00111	0.064607	BP
GO:0090130	tissue migration	0.00111	0.064607	BP
GO:0007612	learning	0.00114	0.066107	BP
GO:2000147	positive regulation of cell motility	0.00115	0.066441	BP
GO:0006952	defense response	0.00116	0.066528	BP
GO:0044089	positive regulation of cellular componen...	0.00116	0.066528	BP
GO:0048523	negative regulation of cellular process	0.00118	0.067428	BP
GO:1990573	potassium ion import across plasma membr...	0.00121	0.068891	BP
GO:0040007	growth	0.00122	0.069208	BP
GO:0010632	regulation of epithelial cell migration	0.00124	0.070089	BP
GO:1901653	cellular response to peptide	0.00125	0.070148	BP
GO:0031344	regulation of cell projection organizati...	0.00125	0.070148	BP
GO:0009914	hormone transport	0.00129	0.072134	BP
GO:0043583	ear development	0.00133	0.074106	BP
GO:0007015	actin filament organization	0.00135	0.074689	BP
GO:0098742	cell-cell adhesion via plasma-membrane a...	0.00135	0.074689	BP
GO:0043087	regulation of GTPase activity	0.00136	0.074977	BP
GO:0052547	regulation of peptidase activity	0.00139	0.076362	BP
GO:0001780	neutrophil homeostasis	0.0014	0.076376	BP
GO:1903909	regulation of receptor clustering	0.0014	0.076376	BP
GO:1902905	positive regulation of supramolecular fi...	0.00141	0.076402	BP
GO:0001941	postsynaptic membrane organization	0.00142	0.076402	BP
GO:0015711	organic anion transport	0.00142	0.076402	BP
GO:1903522	regulation of blood circulation	0.00142	0.076402	BP
GO:0070588	calcium ion transmembrane transport	0.00143	0.076676	BP
GO:0006814	sodium ion transport	0.0015	0.080155	BP
GO:0060627	regulation of vesicle-mediated transport	0.00152	0.080948	BP
GO:0002792	negative regulation of peptide secretion	0.00154	0.081437	BP
GO:0007182	common-partner SMAD protein phosphorylat...	0.00155	0.081437	BP
GO:0061438	renal system vasculature morphogenesis	0.00155	0.081437	BP
GO:0061439	kidney vasculature morphogenesis	0.00155	0.081437	BP
GO:0090066	regulation of anatomical structure size	0.00157	0.082212	BP
GO:0090278	negative regulation of peptide hormone s...	0.00166	0.086062	BP
GO:0048010	vascular endothelial growth factor recep...	0.00166	0.086062	BP
GO:0007159	leukocyte cell-cell adhesion	0.00166	0.086062	BP
GO:1905477	positive regulation of protein localizat...	0.00167	0.086294	BP
GO:0019226	transmission of nerve impulse	0.00169	0.086755	BP
GO:0060350	endochondral bone morphogenesis	0.00169	0.086755	BP
GO:0006536	glutamate metabolic process	0.00173	0.08823	BP
GO:0048011	neurotrophin TRK receptor signaling path...	0.00173	0.08823	BP
GO:0072359	circulatory system development	0.00179	0.090699	BP
GO:0060341	regulation of cellular localization	0.00179	0.090699	BP
GO:0060560	developmental growth involved in morphog...	0.00181	0.091417	BP

GO:0048814	regulation of dendrite morphogenesis	0.00182	0.091626	BP
GO:0046879	hormone secretion	0.00184	0.092336	BP
GO:0006813	potassium ion transport	0.00186	0.093042	BP
GO:0051966	regulation of synaptic transmission, glu...	0.00189	0.094241	BP
GO:0003127	detection of nodal flow	0.00195	0.095113	BP
GO:0006982	response to lipid hydroperoxide	0.00195	0.095113	BP
GO:0007500	mesodermal cell fate determination	0.00195	0.095113	BP
GO:0010133	proline catabolic process to glutamate	0.00195	0.095113	BP
GO:0033563	dorsal/ventral axon guidance	0.00195	0.095113	BP
GO:0061441	renal artery morphogenesis	0.00195	0.095113	BP
GO:0072179	nephric duct formation	0.00195	0.095113	BP
GO:0060292	long-term synaptic depression	0.00207	0.100652	BP
GO:0051962	positive regulation of nervous system de...	0.00209	0.100997	BP
GO:0007259	JAK-STAT cascade	0.00209	0.100997	BP
GO:0002694	regulation of leukocyte activation	0.0021	0.101168	BP
GO:0030155	regulation of cell adhesion	0.00214	0.10263	BP
GO:0001775	cell activation	0.00215	0.10263	BP
GO:0050731	positive regulation of peptidyl-tyrosine...	0.00215	0.10263	BP
GO:0001817	regulation of cytokine production	0.00228	0.108176	BP
GO:0040013	negative regulation of locomotion	0.00228	0.108176	BP
GO:0048839	inner ear development	0.00232	0.109741	BP
GO:0036465	synaptic vesicle recycling	0.00235	0.110825	BP
GO:0045666	positive regulation of neuron differenti...	0.00238	0.11137	BP
GO:0043113	receptor clustering	0.00239	0.11137	BP
GO:0043550	regulation of lipid kinase activity	0.00239	0.11137	BP
GO:0097120	receptor localization to synapse	0.00239	0.11137	BP
GO:0097061	dendritic spine organization	0.00242	0.112433	BP
GO:0002062	chondrocyte differentiation	0.00246	0.113953	BP
GO:0045596	negative regulation of cell differentiat...	0.00249	0.115003	BP
GO:1990778	protein localization to cell periphery	0.0025	0.115125	BP
GO:0010107	potassium ion import	0.00255	0.117083	BP
GO:0072659	protein localization to plasma membrane	0.00261	0.118317	BP
GO:0015824	proline transport	0.00262	0.118317	BP
GO:0021612	facial nerve structural organization	0.00262	0.118317	BP
GO:0033085	negative regulation of T cell differenti...	0.00262	0.118317	BP
GO:0001816	cytokine production	0.00263	0.118317	BP
GO:0008643	carbohydrate transport	0.00264	0.118317	BP
GO:0070885	negative regulation of calcineurin-NFAT ...	0.00264	0.118317	BP
GO:0106057	negative regulation of calcineurin-media...	0.00264	0.118317	BP
GO:0017158	regulation of calcium ion-dependent exoc...	0.00266	0.118317	BP
GO:0051952	regulation of amine transport	0.00266	0.118317	BP
GO:1902803	regulation of synaptic vesicle transport	0.00266	0.118317	BP
GO:0030900	forebrain development	0.00269	0.119313	BP
GO:0044057	regulation of system process	0.00273	0.120405	BP
GO:0007420	brain development	0.00273	0.120405	BP
GO:0044092	negative regulation of molecular functio...	0.00277	0.121826	BP
GO:0002366	leukocyte activation involved in immune ...	0.00282	0.123677	BP
GO:0032689	negative regulation of interferon-gamma ...	0.00287	0.124652	BP
GO:0050709	negative regulation of protein secretion	0.00287	0.124652	BP
GO:0008016	regulation of heart contraction	0.00288	0.124652	BP
GO:0021545	cranial nerve development	0.00289	0.124652	BP
GO:0050691	regulation of defense response to virus ...	0.00289	0.124652	BP

GO:0097484	dendrite extension	0.00289	0.124652	BP
GO:0050771	negative regulation of axonogenesis	0.0029	0.12474	BP
GO:0050796	regulation of insulin secretion	0.00298	0.12748	BP
GO:1903305	regulation of regulated secretory pathwa...	0.00298	0.12748	BP
GO:0040017	positive regulation of locomotion	0.003	0.127986	BP
GO:0001936	regulation of endothelial cell prolifera...	0.0031	0.13118	BP
GO:0007613	memory	0.0031	0.13118	BP
GO:0097696	STAT cascade	0.0031	0.13118	BP
GO:0007417	central nervous system development	0.00314	0.132515	BP
GO:0002263	cell activation involved in immune respo...	0.00318	0.133842	BP
GO:0002431	Fc receptor mediated stimulatory signali...	0.0032	0.134323	BP
GO:1903038	negative regulation of leukocyte cell-ce...	0.00333	0.139081	BP
GO:0030308	negative regulation of cell growth	0.00334	0.139081	BP
GO:0034764	positive regulation of transmembrane tra...	0.00334	0.139081	BP
GO:0006883	cellular sodium ion homeostasis	0.00348	0.143763	BP
GO:0098884	postsynaptic neurotransmitter receptor i...	0.00348	0.143763	BP
GO:0140239	postsynaptic endocytosis	0.00348	0.143763	BP
GO:0048333	mesodermal cell differentiation	0.00349	0.143797	BP
GO:0043434	response to peptide hormone	0.00353	0.145064	BP
GO:0008360	regulation of cell shape	0.00359	0.146759	BP
GO:0045766	positive regulation of angiogenesis	0.00359	0.146759	BP
GO:0015849	organic acid transport	0.00365	0.148436	BP
GO:0046942	carboxylic acid transport	0.00365	0.148436	BP
GO:0043086	negative regulation of catalytic activit...	0.00366	0.148458	BP
GO:0042327	positive regulation of phosphorylation	0.00371	0.150097	BP
GO:0051051	negative regulation of transport	0.00377	0.152131	BP
GO:0042130	negative regulation of T cell proliferat...	0.00378	0.152143	BP
GO:0007269	neurotransmitter secretion	0.00382	0.153358	BP
GO:1901700	response to oxygen-containing compound	0.00385	0.154167	BP
GO:0030856	regulation of epithelial cell differenti...	0.00388	0.154972	BP
GO:0050730	regulation of peptidyl-tyrosine phosphor...	0.0039	0.155375	BP
GO:0022407	regulation of cell-cell adhesion	0.00403	0.158124	BP
GO:0042391	regulation of membrane potential	0.00405	0.158124	BP
GO:0099643	signal release from synapse	0.00405	0.158124	BP
GO:0003337	mesenchymal to epithelial transition inv...	0.00406	0.158124	BP
GO:0008354	germ cell migration	0.00406	0.158124	BP
GO:0021604	cranial nerve structural organization	0.00406	0.158124	BP
GO:0031630	regulation of synaptic vesicle fusion to...	0.00406	0.158124	BP
GO:0098976	excitatory chemical synaptic transmissio...	0.00406	0.158124	BP
GO:2000601	positive regulation of Arp2/3 complex-me...	0.00406	0.158124	BP
GO:1903037	regulation of leukocyte cell-cell adhesi...	0.00407	0.158124	BP
GO:1901699	cellular response to nitrogen compound	0.00409	0.158508	BP
GO:0017157	regulation of exocytosis	0.00411	0.158834	BP
GO:0010562	positive regulation of phosphorus metabo...	0.00413	0.158834	BP
GO:0045937	positive regulation of phosphate metabol...	0.00413	0.158834	BP
GO:0015837	amine transport	0.00416	0.158834	BP
GO:0032092	positive regulation of protein binding	0.00416	0.158834	BP
GO:0045597	positive regulation of cell differentiat...	0.00419	0.158834	BP
GO:0060997	dendritic spine morphogenesis	0.0042	0.158834	BP
GO:0031345	negative regulation of cell projection o...	0.0042	0.158834	BP
GO:0090276	regulation of peptide hormone secretion	0.0042	0.158834	BP
GO:0042036	negative regulation of cytokine biosynth...	0.00421	0.158834	BP

GO:2000311	regulation of AMPA receptor activity	0.00421	0.158834	BP
GO:0008038	neuron recognition	0.00428	0.161086	BP
GO:0051495	positive regulation of cytoskeleton orga...	0.00432	0.161814	BP
GO:0090596	sensory organ morphogenesis	0.00432	0.161814	BP
GO:0033005	positive regulation of mast cell activat...	0.00448	0.167407	BP
GO:0097435	supramolecular fiber organization	0.0045	0.167701	BP
GO:0050868	negative regulation of T cell activation	0.00452	0.167701	BP
GO:1903076	regulation of protein localization to pl...	0.00452	0.167701	BP
GO:0038179	neurotrophin signaling pathway	0.00455	0.168387	BP
GO:0008285	negative regulation of cell proliferatio...	0.00456	0.168387	BP
GO:1903034	regulation of response to wounding	0.00462	0.170201	BP
GO:0043547	positive regulation of GTPase activity	0.00464	0.170471	BP
GO:0061035	regulation of cartilage development	0.00466	0.170471	BP
GO:0071375	cellular response to peptide hormone sti...	0.00466	0.170471	BP
GO:0072657	protein localization to membrane	0.00469	0.171169	BP
GO:0051050	positive regulation of transport	0.00473	0.172227	BP
GO:0051250	negative regulation of lymphocyte activa...	0.00481	0.174692	BP
GO:0007632	visual behavior	0.00482	0.174692	BP
GO:0051099	positive regulation of binding	0.00484	0.175011	BP
GO:0106027	neuron projection organization	0.00491	0.177133	BP
GO:0051125	regulation of actin nucleation	0.00502	0.180685	BP
GO:1904950	negative regulation of establishment of ...	0.00512	0.183441	BP
GO:0150063	visual system development	0.00512	0.183441	BP
GO:2000300	regulation of synaptic vesicle exocytosi...	0.00514	0.183737	BP
GO:0035418	protein localization to synapse	0.00516	0.184032	BP
GO:0051129	negative regulation of cellular componen...	0.00521	0.185328	BP
GO:0060401	cytosolic calcium ion transport	0.00522	0.185328	BP
GO:0006835	dicarboxylic acid transport	0.00533	0.188379	BP
GO:0032231	regulation of actin filament bundle asse...	0.00533	0.188379	BP
GO:0032479	regulation of type I interferon producti...	0.00552	0.190694	BP
GO:0009084	glutamine family amino acid biosynthetic...	0.00566	0.190694	BP
GO:0061298	retina vasculature development in camera...	0.00566	0.190694	BP
GO:0072673	lamellipodium morphogenesis	0.00566	0.190694	BP
GO:0002767	immune response-inhibiting cell surface ...	0.00567	0.190694	BP
GO:0002774	Fc receptor mediated inhibitory signalin...	0.00567	0.190694	BP
GO:0006562	proline catabolic process	0.00567	0.190694	BP
GO:0009726	detection of endogenous stimulus	0.00567	0.190694	BP
GO:0021965	spinal cord ventral commissure morphogen...	0.00567	0.190694	BP
GO:0033058	directional locomotion	0.00567	0.190694	BP
GO:0033088	negative regulation of immature T cell p...	0.00567	0.190694	BP
GO:0045341	MHC class I biosynthetic process	0.00567	0.190694	BP
GO:0045343	regulation of MHC class I biosynthetic p...	0.00567	0.190694	BP
GO:0045345	positive regulation of MHC class I biosy...	0.00567	0.190694	BP
GO:0071462	cellular response to water stimulus	0.00567	0.190694	BP
GO:1901664	regulation of NAD+ ADP-ribosyltransferas...	0.00567	0.190694	BP
GO:1901666	positive regulation of NAD+ ADP-ribosylt...	0.00567	0.190694	BP
GO:1903279	regulation of calcium:sodium antiporter ...	0.00567	0.190694	BP
GO:1903976	negative regulation of glial cell migrat...	0.00567	0.190694	BP
GO:2001189	negative regulation of T cell activation...	0.00567	0.190694	BP
GO:0055074	calcium ion homeostasis	0.00568	0.190694	BP
GO:0009306	protein secretion	0.00568	0.190694	BP
GO:0015807	L-amino acid transport	0.0057	0.190694	BP

GO:1903670	regulation of sprouting angiogenesis	0.0057	0.190694	BP
GO:2001259	positive regulation of cation channel ac...	0.0057	0.190694	BP
GO:0002768	immune response-regulating cell surface ...	0.00573	0.191289	BP
GO:0048880	sensory system development	0.00577	0.192139	BP
GO:0048638	regulation of developmental growth	0.00578	0.192139	BP
GO:0048588	developmental cell growth	0.00583	0.192876	BP
GO:0048705	skeletal system morphogenesis	0.00584	0.192876	BP
GO:0051607	defense response to virus	0.00586	0.192876	BP
GO:0021561	facial nerve development	0.0059	0.192876	BP
GO:0021610	facial nerve morphogenesis	0.0059	0.192876	BP
GO:0099149	regulation of postsynaptic neurotransmit...	0.0059	0.192876	BP
GO:0001958	endochondral ossification	0.00595	0.192876	BP
GO:0003176	aortic valve development	0.00595	0.192876	BP
GO:0036075	replacement ossification	0.00595	0.192876	BP
GO:1905144	response to acetylcholine	0.00595	0.192876	BP
GO:1905145	cellular response to acetylcholine	0.00595	0.192876	BP
GO:0060402	calcium ion transport into cytosol	0.00595	0.192876	BP
GO:0010761	fibroblast migration	0.00598	0.193049	BP
GO:1901021	positive regulation of calcium ion trans...	0.00598	0.193049	BP
GO:0051248	negative regulation of protein metabolic...	0.00602	0.193941	BP
GO:0015844	monoamine transport	0.00613	0.196675	BP
GO:0051492	regulation of stress fiber assembly	0.00613	0.196675	BP
GO:0032606	type I interferon production	0.00632	0.202356	BP
GO:0071702	organic substance transport	0.00639	0.20418	BP
GO:0016049	cell growth	0.00644	0.205359	BP
GO:0051271	negative regulation of cellular componen...	0.00653	0.207805	BP
GO:0070661	leukocyte proliferation	0.00659	0.209183	BP
GO:0043534	blood vessel endothelial cell migration	0.0066	0.209183	BP
GO:0002682	regulation of immune system process	0.00665	0.209917	BP
GO:0050865	regulation of cell activation	0.00665	0.209917	BP
GO:0003333	amino acid transmembrane transport	0.00668	0.21044	BP
GO:0051251	positive regulation of lymphocyte activa...	0.00672	0.211275	BP
GO:0051592	response to calcium ion	0.00674	0.211479	BP
GO:0050433	regulation of catecholamine secretion	0.00677	0.211996	BP
GO:0051968	positive regulation of synaptic transmis...	0.00698	0.217701	BP
GO:1905476	negative regulation of protein localizat...	0.00698	0.217701	BP
GO:0002115	store-operated calcium entry	0.00703	0.218824	BP
GO:0022612	gland morphogenesis	0.00708	0.219818	BP
GO:0032870	cellular response to hormone stimulus	0.00709	0.219818	BP
GO:0045087	innate immune response	0.00712	0.220312	BP
GO:0032273	positive regulation of protein polymeriz...	0.0072	0.222218	BP
GO:0021537	telencephalon development	0.00721	0.222218	BP
GO:0060333	interferon-gamma-mediated signaling path...	0.00727	0.223189	BP
GO:0071277	cellular response to calcium ion	0.00727	0.223189	BP
GO:0050708	regulation of protein secretion	0.0074	0.226735	BP
GO:0002790	peptide secretion	0.00744	0.227516	BP
GO:1903531	negative regulation of secretion by cell	0.00754	0.229677	BP
GO:1902903	regulation of supramolecular fiber organ...	0.00754	0.229677	BP
GO:2000106	regulation of leukocyte apoptotic proces...	0.00759	0.230056	BP
GO:0002040	sprouting angiogenesis	0.00759	0.230056	BP
GO:0040008	regulation of growth	0.00761	0.230056	BP
GO:0044087	regulation of cellular component biogene...	0.00762	0.230056	BP

GO:0006874	cellular calcium ion homeostasis	0.00766	0.230056	BP
GO:0051961	negative regulation of nervous system de...	0.00767	0.230056	BP
GO:0048660	regulation of smooth muscle cell prolife...	0.00767	0.230056	BP
GO:1904064	positive regulation of cation transmembr...	0.00767	0.230056	BP
GO:0090183	regulation of kidney development	0.00772	0.230971	BP
GO:0046883	regulation of hormone secretion	0.00773	0.230971	BP
GO:0110053	regulation of actin filament organizatio...	0.00788	0.235004	BP
GO:0030278	regulation of ossification	0.00794	0.235895	BP
GO:0038093	Fc receptor signaling pathway	0.00794	0.235895	BP
GO:0023014	signal transduction by protein phosphory...	0.00797	0.236338	BP
GO:0007596	blood coagulation	0.00799	0.236483	BP
GO:1901701	cellular response to oxygen-containing c...	0.00801	0.236627	BP
GO:0098693	regulation of synaptic vesicle cycle	0.00812	0.236743	BP
GO:0050849	negative regulation of calcium-mediated ...	0.00813	0.236743	BP
GO:0061082	myeloid leukocyte cytokine production	0.00813	0.236743	BP
GO:0090218	positive regulation of lipid kinase acti...	0.00813	0.236743	BP
GO:0006930	substrate-dependent cell migration, cell...	0.00815	0.236743	BP
GO:0032230	positive regulation of synaptic transmis...	0.00815	0.236743	BP
GO:0045591	positive regulation of regulatory T cell...	0.00815	0.236743	BP
GO:0072283	metanephric renal vesicle morphogenesis	0.00815	0.236743	BP
GO:1990416	cellular response to brain-derived neuro...	0.00815	0.236743	BP
GO:0001935	endothelial cell proliferation	0.00817	0.236885	BP
GO:0030336	negative regulation of cell migration	0.00823	0.238183	BP
GO:0050432	catecholamine secretion	0.00836	0.241499	BP
GO:0060349	bone morphogenesis	0.00845	0.243649	BP
GO:0002274	myeloid leukocyte activation	0.00852	0.24473	BP
GO:0001654	eye development	0.00854	0.24473	BP
GO:0010721	negative regulation of cell development	0.00854	0.24473	BP
GO:0002695	negative regulation of leukocyte activat...	0.00855	0.24473	BP
GO:0051016	barbed-end actin filament capping	0.00859	0.244943	BP
GO:0072273	metanephric nephron morphogenesis	0.00859	0.244943	BP
GO:0051259	protein complex oligomerization	0.00861	0.244943	BP
GO:0031401	positive regulation of protein modificat...	0.00862	0.244943	BP
GO:0097191	extrinsic apoptotic signaling pathway	0.00865	0.245189	BP
GO:0050817	coagulation	0.00866	0.245189	BP
GO:0030534	adult behavior	0.0087	0.245434	BP
GO:0048659	smooth muscle cell proliferation	0.0087	0.245434	BP
GO:0051489	regulation of filopodium assembly	0.00872	0.245556	BP
GO:0021953	central nervous system neuron differenti...	0.00875	0.245638	BP
GO:0009612	response to mechanical stimulus	0.00877	0.245638	BP
GO:0051048	negative regulation of secretion	0.00877	0.245638	BP
GO:0002699	positive regulation of immune effector p...	0.00881	0.246318	BP
GO:0042127	regulation of cell proliferation	0.00905	0.252067	BP
GO:0071346	cellular response to interferon-gamma	0.00906	0.252067	BP
GO:0010771	negative regulation of cell morphogenesi...	0.00908	0.252067	BP
GO:0032414	positive regulation of ion transmembrane...	0.00908	0.252067	BP
GO:0048519	negative regulation of biological proces...	0.00913	0.253006	BP
GO:0034341	response to interferon-gamma	0.00924	0.254978	BP
GO:0042490	mechanoreceptor differentiation	0.00925	0.254978	BP
GO:0046427	positive regulation of JAK-STAT cascade	0.00925	0.254978	BP
GO:0042737	drug catabolic process	0.00928	0.255355	BP
GO:0001558	regulation of cell growth	0.00938	0.257654	BP

GO:0051954	positive regulation of amine transport	0.00941	0.258025	BP
GO:0045926	negative regulation of growth	0.00967	0.264447	BP
GO:0050769	positive regulation of neurogenesis	0.00969	0.264447	BP
GO:0030097	hemopoiesis	0.00973	0.264447	BP
GO:0051224	negative regulation of protein transport	0.00974	0.264447	BP
GO:0007599	hemostasis	0.00976	0.264447	BP
GO:0098656	anion transmembrane transport	0.00979	0.264447	BP
GO:0001504	neurotransmitter uptake	0.0098	0.264447	BP
GO:0031638	zymogen activation	0.0098	0.264447	BP
GO:0000165	MAPK cascade	0.00982	0.264447	BP
GO:0060041	retina development in camera-type eye	0.00983	0.264447	BP
GO:0051347	positive regulation of transferase activ...	0.00983	0.264447	BP
GO:0009064	glutamine family amino acid metabolic pr...	0.00991	0.266142	BP
GO:0071495	cellular response to endogenous stimulus	0.00993	0.266223	BP
GO:0048568	embryonic organ development	0.01008	0.269322	BP
GO:0050900	leukocyte migration	0.01008	0.269322	BP
GO:0007405	neuroblast proliferation	0.0102	0.272064	BP
GO:0009617	response to bacterium	0.0103	0.273339	BP
GO:0072507	divalent inorganic cation homeostasis	0.01034	0.273339	BP
GO:0031629	synaptic vesicle fusion to presynaptic a...	0.01037	0.273339	BP
GO:0032928	regulation of superoxide anion generatio...	0.01037	0.273339	BP
GO:0045662	negative regulation of myoblast differen...	0.01037	0.273339	BP
GO:0099500	vesicle fusion to plasma membrane	0.01037	0.273339	BP
GO:1903077	negative regulation of protein localizat...	0.01037	0.273339	BP
GO:0030004	cellular monovalent inorganic cation hom...	0.01045	0.27406	BP
GO:0060996	dendritic spine development	0.01045	0.27406	BP
GO:0072073	kidney epithelium development	0.01056	0.27406	BP
GO:0060047	heart contraction	0.01062	0.27406	BP
GO:0002275	myeloid cell activation involved in immu...	0.01075	0.27406	BP
GO:0032418	lysosome localization	0.01079	0.27406	BP
GO:0042475	odontogenesis of dentin-containing tooth	0.01079	0.27406	BP
GO:0043535	regulation of blood vessel endothelial c...	0.01079	0.27406	BP
GO:1901019	regulation of calcium ion transmembrane ...	0.01079	0.27406	BP
GO:0097106	postsynaptic density organization	0.01083	0.27406	BP
GO:1905314	semi-lunar valve development	0.01083	0.27406	BP
GO:0002363	alpha-beta T cell lineage commitment	0.01084	0.27406	BP
GO:0021783	preganglionic parasympathetic fiber deve...	0.01084	0.27406	BP
GO:0032700	negative regulation of interleukin-17 pr...	0.01084	0.27406	BP
GO:0061469	regulation of type B pancreatic cell pro...	0.01084	0.27406	BP
GO:1902946	protein localization to early endosome	0.01084	0.27406	BP
GO:2000392	regulation of lamellipodium morphogenesi...	0.01084	0.27406	BP
GO:0034097	response to cytokine	0.01087	0.27406	BP
GO:0032480	negative regulation of type I interferon...	0.01097	0.27406	BP
GO:0010536	positive regulation of activation of Jan...	0.01101	0.27406	BP
GO:0010815	bradykinin catabolic process	0.01101	0.27406	BP
GO:0019470	4-hydroxyproline catabolic process	0.01101	0.27406	BP
GO:0019740	nitrogen utilization	0.01101	0.27406	BP
GO:0032474	otolith morphogenesis	0.01101	0.27406	BP
GO:0033087	negative regulation of immature T cell p...	0.01101	0.27406	BP
GO:0048499	synaptic vesicle membrane organization	0.01101	0.27406	BP
GO:0070777	D-aspartate transport	0.01101	0.27406	BP
GO:0070779	D-aspartate import across plasma membran...	0.01101	0.27406	BP

GO:0072217	negative regulation of metanephros devel...	0.01101	0.27406	BP
GO:0072284	metanephric S-shaped body morphogenesis	0.01101	0.27406	BP
GO:0097350	neutrophil clearance	0.01101	0.27406	BP
GO:1900126	negative regulation of hyaluronan biosyn...	0.01101	0.27406	BP
GO:1903416	response to glycoside	0.01101	0.27406	BP
GO:1903463	regulation of mitotic cell cycle DNA rep...	0.01101	0.27406	BP
GO:2000669	negative regulation of dendritic cell ap...	0.01101	0.27406	BP
GO:0051588	regulation of neurotransmitter transport	0.01107	0.275116	BP
GO:0007229	integrin-mediated signaling pathway	0.0112	0.276895	BP
GO:0046717	acid secretion	0.0112	0.276895	BP
GO:0046888	negative regulation of hormone secretion	0.01123	0.276895	BP
GO:0072088	nephron epithelium morphogenesis	0.01123	0.276895	BP
GO:1904894	positive regulation of STAT cascade	0.01123	0.276895	BP
GO:0008283	cell proliferation	0.01127	0.277008	BP
GO:0001934	positive regulation of protein phosphory...	0.01127	0.277008	BP
GO:0010817	regulation of hormone levels	0.01134	0.278292	BP
GO:0051650	establishment of vesicle localization	0.01138	0.278455	BP
GO:1990138	neuron projection extension	0.0114	0.278455	BP
GO:0033674	positive regulation of kinase activity	0.0114	0.278455	BP
GO:0002697	regulation of immune effector process	0.01144	0.278997	BP
GO:0110020	regulation of actomyosin structure organ...	0.01163	0.28319	BP
GO:0055080	cation homeostasis	0.01182	0.287369	BP
GO:0046651	lymphocyte proliferation	0.01188	0.287731	BP
GO:0048562	embryonic organ morphogenesis	0.01188	0.287731	BP
GO:0031032	actomyosin structure organization	0.01189	0.287731	BP
GO:0098739	import across plasma membrane	0.01198	0.289015	BP
GO:1901343	negative regulation of vasculature devel...	0.01198	0.289015	BP
GO:0051707	response to other organism	0.0121	0.291461	BP
GO:0003012	muscle system process	0.01214	0.291975	BP
GO:0001658	branching involved in ureteric bud morph...	0.01225	0.2928	BP
GO:0008542	visual learning	0.01225	0.2928	BP
GO:0061001	regulation of dendritic spine morphogene...	0.01225	0.2928	BP
GO:0001655	urogenital system development	0.01227	0.2928	BP
GO:0032945	negative regulation of mononuclear cell ...	0.01233	0.2928	BP
GO:0046847	filopodium assembly	0.01233	0.2928	BP
GO:0050672	negative regulation of lymphocyte prolif...	0.01233	0.2928	BP
GO:0033081	regulation of T cell differentiation in ...	0.01238	0.2928	BP
GO:0045606	positive regulation of epidermal cell di...	0.01238	0.2928	BP
GO:1903861	positive regulation of dendrite extensio...	0.01238	0.2928	BP
GO:0045684	positive regulation of epidermis develop...	0.01238	0.2928	BP
GO:0055067	monovalent inorganic cation homeostasis	0.01242	0.292861	BP
GO:1903169	regulation of calcium ion transmembrane ...	0.01242	0.292861	BP
GO:0050770	regulation of axonogenesis	0.01247	0.293598	BP
GO:0046425	regulation of JAK-STAT cascade	0.01252	0.294333	BP
GO:0006873	cellular ion homeostasis	0.01258	0.2953	BP
GO:0043207	response to external biotic stimulus	0.01268	0.29704	BP
GO:0072080	nephron tubule development	0.01272	0.29704	BP
GO:0051260	protein homooligomerization	0.01272	0.29704	BP
GO:0045834	positive regulation of lipid metabolic p...	0.01273	0.29704	BP
GO:0051249	regulation of lymphocyte activation	0.01284	0.298716	BP
GO:0098542	defense response to other organism	0.01284	0.298716	BP
GO:0002684	positive regulation of immune system pro...	0.01293	0.300364	BP

GO:0032943	mononuclear cell proliferation	0.01296	0.300614	BP
GO:0055065	metal ion homeostasis	0.01316	0.304802	BP
GO:0050670	regulation of lymphocyte proliferation	0.01326	0.306664	BP
GO:0048598	embryonic morphogenesis	0.01328	0.306674	BP
GO:0043010	camera-type eye development	0.0134	0.308535	BP
GO:0050768	negative regulation of neurogenesis	0.0134	0.308535	BP
GO:0032233	positive regulation of actin filament bu...	0.0135	0.309926	BP
GO:0072028	nephron morphogenesis	0.0135	0.309926	BP
GO:2000146	negative regulation of cell motility	0.01353	0.31016	BP
GO:0045010	actin nucleation	0.01362	0.311767	BP
GO:0097194	execution phase of apoptosis	0.01377	0.314715	BP
GO:0032944	regulation of mononuclear cell prolifera...	0.01393	0.314715	BP
GO:0001710	mesodermal cell fate commitment	0.01399	0.314715	BP
GO:0021819	layer formation in cerebral cortex	0.01399	0.314715	BP
GO:0033605	positive regulation of catecholamine sec...	0.01399	0.314715	BP
GO:0043369	CD4-positive or CD8-positive, alpha-beta...	0.01399	0.314715	BP
GO:0048486	parasympathetic nervous system developme...	0.01399	0.314715	BP
GO:0051386	regulation of neurotrophin TRK receptor ...	0.01399	0.314715	BP
GO:0060391	positive regulation of SMAD protein sign...	0.01399	0.314715	BP
GO:0071340	skeletal muscle acetylcholine-gated chan...	0.01399	0.314715	BP
GO:0097104	postsynaptic membrane assembly	0.01399	0.314715	BP
GO:1902287	semaphorin-plexin signaling pathway invo...	0.01399	0.314715	BP
GO:0001974	blood vessel remodeling	0.01407	0.315156	BP
GO:0045616	regulation of keratinocyte differentiati...	0.01407	0.315156	BP
GO:0099084	postsynaptic specialization organization	0.01407	0.315156	BP
GO:0051346	negative regulation of hydrolase activit...	0.01424	0.318508	BP
GO:0001508	action potential	0.01435	0.320511	BP
GO:0002429	immune response-activating cell surface ...	0.01444	0.322061	BP
GO:0003433	chondrocyte development involved in endo...	0.01461	0.322169	BP
GO:0043368	positive T cell selection	0.01461	0.322169	BP
GO:0046337	phosphatidylethanolamine metabolic proce...	0.01461	0.322169	BP
GO:0061437	renal system vasculature development	0.01461	0.322169	BP
GO:0061440	kidney vasculature development	0.01461	0.322169	BP
GO:1904376	negative regulation of protein localizat...	0.01461	0.322169	BP
GO:2000209	regulation of anoikis	0.01461	0.322169	BP
GO:0022409	positive regulation of cell-cell adhesio...	0.01461	0.322169	BP
GO:0072001	renal system development	0.01463	0.322169	BP
GO:0034767	positive regulation of ion transmembrane...	0.01469	0.322311	BP
GO:1901698	response to nitrogen compound	0.01469	0.322311	BP
GO:0007162	negative regulation of cell adhesion	0.01472	0.322311	BP
GO:0098771	inorganic ion homeostasis	0.01473	0.322311	BP
GO:1904427	positive regulation of calcium ion trans...	0.01475	0.322311	BP
GO:0010977	negative regulation of neuron projection...	0.01476	0.322311	BP
GO:0061326	renal tubule development	0.01489	0.324697	BP
GO:0050801	ion homeostasis	0.01495	0.325552	BP
GO:0060429	epithelium development	0.01498	0.325753	BP
GO:0002791	regulation of peptide secretion	0.01507	0.327236	BP
GO:0043551	regulation of phosphatidylinositol 3-kin...	0.01509	0.327236	BP
GO:0003015	heart process	0.0152	0.329143	BP
GO:0061041	regulation of wound healing	0.01522	0.329143	BP
GO:0010951	negative regulation of endopeptidase act...	0.01536	0.331712	BP
GO:0016525	negative regulation of angiogenesis	0.01547	0.3336	BP

GO:0008015	blood circulation	0.01549	0.3336	BP
GO:0032411	positive regulation of transporter activ...	0.01552	0.333787	BP
GO:0042325	regulation of phosphorylation	0.01569	0.33698	BP
GO:0030003	cellular cation homeostasis	0.01576	0.33802	BP
GO:0032594	protein transport within lipid bilayer	0.01591	0.33888	BP
GO:0045429	positive regulation of nitric oxide bios...	0.01591	0.33888	BP
GO:0046676	negative regulation of insulin secretion	0.01591	0.33888	BP
GO:0071709	membrane assembly	0.01591	0.33888	BP
GO:1903672	positive regulation of sprouting angioge...	0.01591	0.33888	BP
GO:0071345	cellular response to cytokine stimulus	0.01593	0.33888	BP
GO:0055082	cellular chemical homeostasis	0.01601	0.34012	BP
GO:0008306	associative learning	0.01607	0.340222	BP
GO:0071158	positive regulation of cell cycle arrest	0.01607	0.340222	BP
GO:0032101	regulation of response to external stimu...	0.01608	0.340222	BP
GO:0035725	sodium ion transmembrane transport	0.01613	0.34036	BP
GO:0097553	calcium ion transmembrane import into cy...	0.01613	0.34036	BP
GO:0001503	ossification	0.01618	0.340956	BP
GO:0009719	response to endogenous stimulus	0.01627	0.342392	BP
GO:1904375	regulation of protein localization to ce...	0.01652	0.346586	BP
GO:0015749	monosaccharide transmembrane transport	0.01657	0.346586	BP
GO:1904892	regulation of STAT cascade	0.01657	0.346586	BP
GO:2000181	negative regulation of blood vessel morp...	0.01657	0.346586	BP
GO:0002250	adaptive immune response	0.01658	0.346586	BP
GO:0045747	positive regulation of Notch signaling p...	0.01668	0.347262	BP
GO:0009607	response to biotic stimulus	0.01688	0.347262	BP
GO:0051216	cartilage development	0.01689	0.347262	BP
GO:0060359	response to ammonium ion	0.01707	0.347262	BP
GO:0003180	aortic valve morphogenesis	0.01708	0.347262	BP
GO:0007413	axonal fasciculation	0.01708	0.347262	BP
GO:0015813	L-glutamate transmembrane transport	0.01708	0.347262	BP
GO:0035561	regulation of chromatin binding	0.01708	0.347262	BP
GO:0089718	amino acid import across plasma membrane	0.01708	0.347262	BP
GO:0106030	neuron projection fasciculation	0.01708	0.347262	BP
GO:1903859	regulation of dendrite extension	0.01708	0.347262	BP
GO:0006936	muscle contraction	0.01715	0.347262	BP
GO:0019216	regulation of lipid metabolic process	0.01725	0.347262	BP
GO:0046649	lymphocyte activation	0.01737	0.347262	BP
GO:0048878	chemical homeostasis	0.01748	0.347262	BP
GO:0070664	negative regulation of leukocyte prolife...	0.0175	0.347262	BP
GO:0051283	negative regulation of sequestering of c...	0.01757	0.347262	BP
GO:0010935	regulation of macrophage cytokine produc...	0.01759	0.347262	BP
GO:0035563	positive regulation of chromatin binding	0.01759	0.347262	BP
GO:0048532	anatomical structure arrangement	0.01759	0.347262	BP
GO:0060231	mesenchymal to epithelial transition	0.01759	0.347262	BP
GO:0072077	renal vesicle morphogenesis	0.01759	0.347262	BP
GO:0098712	L-glutamate import across plasma membran...	0.01759	0.347262	BP
GO:1901626	regulation of postsynaptic membrane orga...	0.01759	0.347262	BP
GO:1902285	semaphorin-plexin signaling pathway invo...	0.01759	0.347262	BP
GO:2001256	regulation of store-operated calcium ent...	0.01759	0.347262	BP
GO:0010720	positive regulation of cell development	0.01764	0.347262	BP
GO:0030038	contractile actin filament bundle assemb...	0.01771	0.347262	BP
GO:0043149	stress fiber assembly	0.01771	0.347262	BP

GO:0000820	regulation of glutamine family amino aci...	0.01781	0.347262	BP
GO:0002765	immune response-inhibiting signal transd...	0.01781	0.347262	BP
GO:0003339	regulation of mesenchymal to epithelial ...	0.01781	0.347262	BP
GO:0010533	regulation of activation of Janus kinase...	0.01781	0.347262	BP
GO:0021562	vestibulocochlear nerve development	0.01781	0.347262	BP
GO:0021631	optic nerve morphogenesis	0.01781	0.347262	BP
GO:0021636	trigeminal nerve morphogenesis	0.01781	0.347262	BP
GO:0021637	trigeminal nerve structural organization	0.01781	0.347262	BP
GO:0021960	anterior commissure morphogenesis	0.01781	0.347262	BP
GO:0032627	interleukin-23 production	0.01781	0.347262	BP
GO:0032667	regulation of interleukin-23 production	0.01781	0.347262	BP
GO:0033084	regulation of immature T cell proliferat...	0.01781	0.347262	BP
GO:0033564	anterior/posterior axon guidance	0.01781	0.347262	BP
GO:0043435	response to corticotropin-releasing horm...	0.01781	0.347262	BP
GO:0048170	positive regulation of long-term neurona...	0.01781	0.347262	BP
GO:0048840	otolith development	0.01781	0.347262	BP
GO:0060385	axonogenesis involved in innervation	0.01781	0.347262	BP
GO:0060666	dichotomous subdivision of terminal unit...	0.01781	0.347262	BP
GO:0070640	vitamin D3 metabolic process	0.01781	0.347262	BP
GO:0071286	cellular response to magnesium ion	0.01781	0.347262	BP
GO:0071376	cellular response to corticotropin-relea...	0.01781	0.347262	BP
GO:0072161	mesenchymal cell differentiation involve...	0.01781	0.347262	BP
GO:1904393	regulation of skeletal muscle acetylchol...	0.01781	0.347262	BP
GO:2001012	mesenchymal cell differentiation involve...	0.01781	0.347262	BP
GO:2001186	negative regulation of CD8-positive, alp...	0.01781	0.347262	BP
GO:0055078	sodium ion homeostasis	0.01791	0.348344	BP
GO:1904407	positive regulation of nitric oxide meta...	0.01791	0.348344	BP
GO:0051130	positive regulation of cellular componen...	0.01812	0.351991	BP
GO:0032535	regulation of cellular component size	0.01837	0.356405	BP
GO:0002433	immune response-regulating cell surface ...	0.01863	0.359224	BP
GO:0022404	molting cycle process	0.01863	0.359224	BP
GO:0022405	hair cycle process	0.01863	0.359224	BP
GO:0038096	Fc-gamma receptor signaling pathway invo...	0.01863	0.359224	BP
GO:0042472	inner ear morphogenesis	0.01863	0.359224	BP
GO:0071417	cellular response to organonitrogen comp...	0.01867	0.359553	BP
GO:0007266	Rho protein signal transduction	0.0188	0.361611	BP
GO:0034219	carbohydrate transmembrane transport	0.01891	0.362836	BP
GO:0042471	ear morphogenesis	0.01891	0.362836	BP
GO:0034103	regulation of tissue remodeling	0.019	0.363671	BP
GO:0071242	cellular response to ammonium ion	0.019	0.363671	BP
GO:0072503	cellular divalent inorganic cation homeo...	0.01908	0.364756	BP
GO:0033002	muscle cell proliferation	0.0194	0.369971	BP
GO:1903039	positive regulation of leukocyte cell-ce...	0.0194	0.369971	BP
GO:0045444	fat cell differentiation	0.01963	0.373432	BP
GO:0090257	regulation of muscle system process	0.01967	0.373432	BP
GO:0007271	synaptic transmission, cholinergic	0.0198	0.373432	BP
GO:0035767	endothelial cell chemotaxis	0.0198	0.373432	BP
GO:0048730	epidermis morphogenesis	0.0198	0.373432	BP
GO:0060384	innervation	0.0198	0.373432	BP
GO:0070723	response to cholesterol	0.0198	0.373432	BP
GO:0099563	modification of synaptic structure	0.0198	0.373432	BP
GO:0051282	regulation of sequestering of calcium io...	0.0198	0.373432	BP

GO:0003013	circulatory system process	0.01982	0.373432	BP
GO:0007010	cytoskeleton organization	0.01985	0.373473	BP
GO:0006875	cellular metal ion homeostasis	0.01996	0.373473	BP
GO:0048534	hematopoietic or lymphoid organ developm...	0.02	0.373473	BP
GO:0002028	regulation of sodium ion transport	0.02002	0.373473	BP
GO:0038094	Fc-gamma receptor signaling pathway	0.02002	0.373473	BP
GO:0070167	regulation of biomineral tissue developm...	0.02002	0.373473	BP
GO:0030501	positive regulation of bone mineralizati...	0.02007	0.373473	BP
GO:0048771	tissue remodeling	0.02015	0.373473	BP
GO:1903825	organic acid transmembrane transport	0.02016	0.373473	BP
GO:1905039	carboxylic acid transmembrane transport	0.02016	0.373473	BP
GO:0051648	vesicle localization	0.02018	0.373473	BP
GO:0032608	interferon-beta production	0.02018	0.373473	BP
GO:0051496	positive regulation of stress fiber asse...	0.02018	0.373473	BP
GO:0060675	ureteric bud morphogenesis	0.02018	0.373473	BP
GO:0061098	positive regulation of protein tyrosine ...	0.02018	0.373473	BP
GO:0050673	epithelial cell proliferation	0.02024	0.374141	BP
GO:0070663	regulation of leukocyte proliferation	0.02029	0.374623	BP
GO:0051937	catecholamine transport	0.02059	0.379715	BP
GO:0050776	regulation of immune response	0.02073	0.381847	BP
GO:0021700	developmental maturation	0.02085	0.383606	BP
GO:0051640	organelle localization	0.02101	0.386096	BP
GO:0051017	actin filament bundle assembly	0.02119	0.388947	BP
GO:0010466	negative regulation of peptidase activit...	0.02122	0.389042	BP
GO:0002831	regulation of response to biotic stimulu...	0.02128	0.389685	BP
GO:0015850	organic hydroxy compound transport	0.02134	0.389872	BP
GO:0043491	protein kinase B signaling	0.02134	0.389872	BP
GO:0051938	L-glutamate import	0.02167	0.392694	BP
GO:0070571	negative regulation of neuron projection...	0.02167	0.392694	BP
GO:0072074	kidney mesenchyme development	0.02167	0.392694	BP
GO:0072087	renal vesicle development	0.02167	0.392694	BP
GO:0090481	pyrimidine nucleotide-sugar transmembran...	0.02167	0.392694	BP
GO:1900452	regulation of long-term synaptic depress...	0.02167	0.392694	BP
GO:1902187	negative regulation of viral release fro...	0.02167	0.392694	BP
GO:0032270	positive regulation of cellular protein ...	0.02188	0.396041	BP
GO:0009888	tissue development	0.02201	0.397267	BP
GO:0045428	regulation of nitric oxide biosynthetic ...	0.0221	0.397267	BP
GO:0045604	regulation of epidermal cell differentia...	0.0221	0.397267	BP
GO:0048016	inositol phosphate-mediated signaling	0.0221	0.397267	BP
GO:0072171	mesonephric tubule morphogenesis	0.0221	0.397267	BP
GO:1902475	L-alpha-amino acid transmembrane transpo...	0.0221	0.397267	BP
GO:0010863	positive regulation of phospholipase C a...	0.02238	0.399093	BP
GO:0014911	positive regulation of smooth muscle cel...	0.02238	0.399093	BP
GO:0015701	bicarbonate transport	0.02238	0.399093	BP
GO:0033003	regulation of mast cell activation	0.02238	0.399093	BP
GO:0060351	cartilage development involved in endoch...	0.02238	0.399093	BP
GO:0090049	regulation of cell migration involved in...	0.02238	0.399093	BP
GO:2000107	negative regulation of leukocyte apoptot...	0.02238	0.399093	BP
GO:0003338	metanephros morphogenesis	0.02277	0.403292	BP
GO:0032647	regulation of interferon-alpha productio...	0.02277	0.403292	BP
GO:0045956	positive regulation of calcium ion-depen...	0.02277	0.403292	BP
GO:0085029	extracellular matrix assembly	0.02277	0.403292	BP

GO:0095500	acetylcholine receptor signaling pathway	0.02277	0.403292	BP
GO:1903831	signal transduction involved in cellular...	0.02277	0.403292	BP
GO:0050866	negative regulation of cell activation	0.02287	0.404605	BP
GO:0097190	apoptotic signaling pathway	0.02333	0.412277	BP
GO:0006468	protein phosphorylation	0.02349	0.414637	BP
GO:0051208	sequestering of calcium ion	0.02353	0.414875	BP
GO:0014065	phosphatidylinositol 3-kinase signaling	0.02365	0.416522	BP
GO:0031399	regulation of protein modification proce...	0.02372	0.417285	BP
GO:0006950	response to stress	0.02375	0.417344	BP
GO:0050870	positive regulation of T cell activation	0.02395	0.420387	BP
GO:0045637	regulation of myeloid cell differentiati...	0.02403	0.420423	BP
GO:0030500	regulation of bone mineralization	0.02405	0.420423	BP
GO:0046209	nitric oxide metabolic process	0.02405	0.420423	BP
GO:0008284	positive regulation of cell proliferatio...	0.0241	0.420423	BP
GO:0031663	lipopolysaccharide-mediated signaling pa...	0.02414	0.420423	BP
GO:0060113	inner ear receptor cell differentiation	0.02414	0.420423	BP
GO:1903307	positive regulation of regulated secreto...	0.02414	0.420423	BP
GO:0045446	endothelial cell differentiation	0.02432	0.423087	BP
GO:0045861	negative regulation of proteolysis	0.02441	0.423885	BP
GO:0043299	leukocyte degranulation	0.02442	0.423885	BP
GO:0050678	regulation of epithelial cell proliferat...	0.02455	0.424828	BP
GO:0061572	actin filament bundle organization	0.02455	0.424828	BP
GO:0042035	regulation of cytokine biosynthetic proc...	0.02461	0.424828	BP
GO:0048661	positive regulation of smooth muscle cel...	0.02461	0.424828	BP
GO:0051899	membrane depolarization	0.02461	0.424828	BP
GO:0032881	regulation of polysaccharide metabolic p...	0.02487	0.425416	BP
GO:0003158	endothelium development	0.02488	0.425416	BP
GO:0042133	neurotransmitter metabolic process	0.0249	0.425416	BP
GO:0030100	regulation of endocytosis	0.02502	0.425416	BP
GO:0050863	regulation of T cell activation	0.02502	0.425416	BP
GO:0002443	leukocyte mediated immunity	0.0254	0.425416	BP
GO:0014909	smooth muscle cell migration	0.02591	0.425416	BP
GO:0006216	cytidine catabolic process	0.02594	0.425416	BP
GO:0006537	glutamate biosynthetic process	0.02594	0.425416	BP
GO:0009972	cytidine deamination	0.02594	0.425416	BP
GO:0021785	branchiomotor neuron axon guidance	0.02594	0.425416	BP
GO:0031547	brain-derived neurotrophic factor recept...	0.02594	0.425416	BP
GO:0033080	immature T cell proliferation in thymus	0.02594	0.425416	BP
GO:0035095	behavioral response to nicotine	0.02594	0.425416	BP
GO:0035726	common myeloid progenitor cell prolifera...	0.02594	0.425416	BP
GO:0038113	interleukin-9-mediated signaling pathway	0.02594	0.425416	BP
GO:0038114	interleukin-21-mediated signaling pathwa...	0.02594	0.425416	BP
GO:0042268	regulation of cytolysis	0.02594	0.425416	BP
GO:0046087	cytidine metabolic process	0.02594	0.425416	BP
GO:0046487	glyoxylate metabolic process	0.02594	0.425416	BP
GO:0048549	positive regulation of pinocytosis	0.02594	0.425416	BP
GO:0051902	negative regulation of mitochondrial dep...	0.02594	0.425416	BP
GO:0060332	positive regulation of response to inter...	0.02594	0.425416	BP
GO:0060335	positive regulation of interferon-gamma-...	0.02594	0.425416	BP
GO:0071104	response to interleukin-9	0.02594	0.425416	BP
GO:0071355	cellular response to interleukin-9	0.02594	0.425416	BP
GO:0072050	S-shaped body morphogenesis	0.02594	0.425416	BP

GO:0090219	negative regulation of lipid kinase acti...	0.02594	0.425416	BP
GO:0098756	response to interleukin-21	0.02594	0.425416	BP
GO:0098757	cellular response to interleukin-21	0.02594	0.425416	BP
GO:0098880	maintenance of postsynaptic specializati...	0.02594	0.425416	BP
GO:1903911	positive regulation of receptor clusteri...	0.02594	0.425416	BP
GO:1903997	positive regulation of non-membrane span...	0.02594	0.425416	BP
GO:2001188	regulation of T cell activation via T ce...	0.02594	0.425416	BP
GO:0050778	positive regulation of immune response	0.02595	0.425416	BP
GO:0009065	glutamine family amino acid catabolic pr...	0.02599	0.425416	BP
GO:0032607	interferon-alpha production	0.02599	0.425416	BP
GO:0043090	amino acid import	0.02599	0.425416	BP
GO:0043552	positive regulation of phosphatidylinosi...	0.02599	0.425416	BP
GO:0060074	synapse maturation	0.02599	0.425416	BP
GO:0072210	metanephric nephron development	0.02599	0.425416	BP
GO:0099632	protein transport within plasma membrane	0.02599	0.425416	BP
GO:0099637	neurotransmitter receptor transport	0.02599	0.425416	BP
GO:0009615	response to virus	0.02614	0.425416	BP
GO:0010942	positive regulation of cell death	0.02618	0.425416	BP
GO:0006646	phosphatidylethanolamine biosynthetic pr...	0.02622	0.425416	BP
GO:0010934	macrophage cytokine production	0.02622	0.425416	BP
GO:0015780	nucleotide-sugar transmembrane transport	0.02622	0.425416	BP
GO:0019372	lipoxygenase pathway	0.02622	0.425416	BP
GO:0030949	positive regulation of vascular endothel...	0.02622	0.425416	BP
GO:0035729	cellular response to hepatocyte growth f...	0.02622	0.425416	BP
GO:0045618	positive regulation of keratinocyte diff...	0.02622	0.425416	BP
GO:0051127	positive regulation of actin nucleation	0.02622	0.425416	BP
GO:0051284	positive regulation of sequestering of c...	0.02622	0.425416	BP
GO:0090189	regulation of branching involved in uret...	0.02622	0.425416	BP
GO:0099558	maintenance of synapse structure	0.02622	0.425416	BP
GO:1900118	negative regulation of execution phase o...	0.02622	0.425416	BP
GO:2000811	negative regulation of anoikis	0.02622	0.425416	BP
GO:0031102	neuron projection regeneration	0.02631	0.426434	BP
GO:0031346	positive regulation of cell projection o...	0.02638	0.427127	BP
GO:0043270	positive regulation of ion transport	0.0265	0.428627	BP
GO:1903532	positive regulation of secretion by cell	0.02689	0.434199	BP
GO:0019220	regulation of phosphate metabolic proces...	0.0269	0.434199	BP
GO:0046928	regulation of neurotransmitter secretion	0.0274	0.441814	BP
GO:0044091	membrane biogenesis	0.02753	0.442865	BP
GO:1900274	regulation of phospholipase C activity	0.02753	0.442865	BP
GO:0060078	regulation of postsynaptic membrane pote...	0.02755	0.442865	BP
GO:0051174	regulation of phosphorus metabolic proce...	0.02765	0.444016	BP
GO:0009725	response to hormone	0.02774	0.445005	BP
GO:0050688	regulation of defense response to virus	0.02788	0.446793	BP
GO:0010976	positive regulation of neuron projection...	0.02796	0.447617	BP
GO:0051480	regulation of cytosolic calcium ion conc...	0.02799	0.44764	BP
GO:0045921	positive regulation of exocytosis	0.02805	0.447685	BP
GO:0071887	leukocyte apoptotic process	0.02805	0.447685	BP
GO:0042098	T cell proliferation	0.02828	0.450437	BP
GO:0050773	regulation of dendrite development	0.02828	0.450437	BP
GO:0032868	response to insulin	0.02849	0.453321	BP
GO:0002042	cell migration involved in sprouting ang...	0.0286	0.453387	BP
GO:0043536	positive regulation of blood vessel endo...	0.0286	0.453387	BP

GO:0051926	negative regulation of calcium ion trans...	0.0286	0.453387	BP
GO:1905330	regulation of morphogenesis of an epithe...	0.02861	0.453387	BP
GO:0071156	regulation of cell cycle arrest	0.02904	0.459736	BP
GO:0071407	cellular response to organic cyclic comp...	0.02907	0.459746	BP
GO:0003413	chondrocyte differentiation involved in ...	0.02948	0.463422	BP
GO:0006953	acute-phase response	0.02948	0.463422	BP
GO:0010742	macrophage derived foam cell differentia...	0.02948	0.463422	BP
GO:0036314	response to sterol	0.02948	0.463422	BP
GO:0051491	positive regulation of filopodium assemb...	0.02948	0.463422	BP
GO:0090077	foam cell differentiation	0.02948	0.463422	BP
GO:0043405	regulation of MAP kinase activity	0.02968	0.466098	BP
GO:0030162	regulation of proteolysis	0.02977	0.467043	BP
GO:1905114	cell surface receptor signaling pathway ...	0.0299	0.468302	BP
GO:0009953	dorsal/ventral pattern formation	0.02994	0.468302	BP
GO:2001057	reactive nitrogen species metabolic proc...	0.02994	0.468302	BP
GO:0010862	positive regulation of pathway-restricte...	0.03035	0.473137	BP
GO:0070169	positive regulation of biomineral tissue...	0.03035	0.473137	BP
GO:2000272	negative regulation of signaling recepto...	0.03035	0.473137	BP
GO:0007265	Ras protein signal transduction	0.03037	0.473137	BP
GO:0045860	positive regulation of protein kinase ac...	0.03054	0.475313	BP
GO:0051246	regulation of protein metabolic process	0.03078	0.477195	BP
GO:0035150	regulation of tube size	0.03083	0.477195	BP
GO:0032515	negative regulation of phosphoprotein ph...	0.03102	0.477195	BP
GO:0003414	chondrocyte morphogenesis involved in en...	0.03123	0.477195	BP
GO:0003429	growth plate cartilage chondrocyte morph...	0.03123	0.477195	BP
GO:0010523	negative regulation of calcium ion trans...	0.03123	0.477195	BP
GO:0034315	regulation of Arp2/3 complex-mediated ac...	0.03123	0.477195	BP
GO:0035970	peptidyl-threonine dephosphorylation	0.03123	0.477195	BP
GO:0036037	CD8-positive, alpha-beta T cell activati...	0.03123	0.477195	BP
GO:0044320	cellular response to leptin stimulus	0.03123	0.477195	BP
GO:0048311	mitochondrion distribution	0.03123	0.477195	BP
GO:0060572	morphogenesis of an epithelial bud	0.03123	0.477195	BP
GO:0061217	regulation of mesonephros development	0.03123	0.477195	BP
GO:0071397	cellular response to cholesterol	0.03123	0.477195	BP
GO:0072215	regulation of metanephros development	0.03123	0.477195	BP
GO:0090171	chondrocyte morphogenesis	0.03123	0.477195	BP
GO:0097091	synaptic vesicle clustering	0.03123	0.477195	BP
GO:1904754	positive regulation of vascular associat...	0.03123	0.477195	BP
GO:0043065	positive regulation of apoptotic process	0.03124	0.477195	BP
GO:0001822	kidney development	0.03168	0.482442	BP
GO:0043406	positive regulation of MAP kinase activi...	0.03168	0.482442	BP
GO:0001932	regulation of protein phosphorylation	0.03174	0.482442	BP
GO:0051047	positive regulation of secretion	0.03176	0.482442	BP
GO:0010522	regulation of calcium ion transport into...	0.03181	0.482442	BP
GO:0072009	nephron epithelium development	0.03181	0.482442	BP
GO:1904659	glucose transmembrane transport	0.03181	0.482442	BP
GO:0050878	regulation of body fluid levels	0.03183	0.482442	BP
GO:0030168	platelet activation	0.0319	0.483035	BP
GO:0045682	regulation of epidermis development	0.0321	0.485594	BP
GO:0007389	pattern specification process	0.03217	0.486183	BP
GO:0051896	regulation of protein kinase B signaling	0.03233	0.488112	BP
GO:0070201	regulation of establishment of protein l...	0.03236	0.488112	BP

GO:0030282	bone mineralization	0.03252	0.490054	BP
GO:0002764	immune response-regulating signaling pat...	0.03283	0.494249	BP
GO:0042110	T cell activation	0.03305	0.496927	BP
GO:0033036	macromolecule localization	0.03314	0.496927	BP
GO:0002520	immune system development	0.0332	0.496927	BP
GO:0010574	regulation of vascular endothelial growt...	0.03323	0.496927	BP
GO:0010644	cell communication by electrical couplin...	0.03323	0.496927	BP
GO:0045601	regulation of endothelial cell different...	0.03323	0.496927	BP
GO:0061036	positive regulation of cartilage develop...	0.03323	0.496927	BP
GO:0051247	positive regulation of protein metabolic...	0.0333	0.497445	BP
GO:0042531	positive regulation of tyrosine phosphor...	0.03336	0.497445	BP
GO:0045661	regulation of myoblast differentiation	0.03336	0.497445	BP
GO:0001707	mesoderm formation	0.03357	0.499625	BP
GO:0072078	nephron tubule morphogenesis	0.03357	0.499625	BP
GO:0002444	myeloid leukocyte mediated immunity	0.03362	0.499894	BP
GO:0010648	negative regulation of cell communicatio...	0.03393	0.502116	BP
GO:0043068	positive regulation of programmed cell d...	0.03408	0.502116	BP
GO:0007631	feeding behavior	0.03437	0.502116	BP
GO:0042476	odontogenesis	0.03437	0.502116	BP
GO:0023057	negative regulation of signaling	0.03495	0.502116	BP
GO:0051897	positive regulation of protein kinase B ...	0.03505	0.502116	BP
GO:0071772	response to BMP	0.03505	0.502116	BP
GO:0071773	cellular response to BMP stimulus	0.03505	0.502116	BP
GO:0001553	luteinization	0.03527	0.502116	BP
GO:0003330	regulation of extracellular matrix const...	0.03527	0.502116	BP
GO:0006068	ethanol catabolic process	0.03527	0.502116	BP
GO:0010966	regulation of phosphate transport	0.03527	0.502116	BP
GO:0016554	cytidine to uridine editing	0.03527	0.502116	BP
GO:0021559	trigeminal nerve development	0.03527	0.502116	BP
GO:0023041	neuronal signal transduction	0.03527	0.502116	BP
GO:0032762	mast cell cytokine production	0.03527	0.502116	BP
GO:0033083	regulation of immature T cell proliferat...	0.03527	0.502116	BP
GO:0042940	D-amino acid transport	0.03527	0.502116	BP
GO:0045348	positive regulation of MHC class II bios...	0.03527	0.502116	BP
GO:0045350	interferon-beta biosynthetic process	0.03527	0.502116	BP
GO:0045357	regulation of interferon-beta biosynthet...	0.03527	0.502116	BP
GO:0051388	positive regulation of neurotrophin TRK ...	0.03527	0.502116	BP
GO:0051964	negative regulation of synapse assembly	0.03527	0.502116	BP
GO:0060272	embryonic skeletal joint morphogenesis	0.03527	0.502116	BP
GO:0060836	lymphatic endothelial cell differentiati...	0.03527	0.502116	BP
GO:0072124	regulation of glomerular mesangial cell ...	0.03527	0.502116	BP
GO:0072177	mesonephric duct development	0.03527	0.502116	BP
GO:0086036	regulation of cardiac muscle cell membra...	0.03527	0.502116	BP
GO:0097048	dendritic cell apoptotic process	0.03527	0.502116	BP
GO:1900122	positive regulation of receptor binding	0.03527	0.502116	BP
GO:1900244	positive regulation of synaptic vesicle ...	0.03527	0.502116	BP
GO:1902474	positive regulation of protein localizat...	0.03527	0.502116	BP
GO:1902961	positive regulation of aspartic-type end...	0.03527	0.502116	BP
GO:1903995	regulation of non-membrane spanning prot...	0.03527	0.502116	BP
GO:1904180	negative regulation of membrane depolari...	0.03527	0.502116	BP
GO:1904749	regulation of protein localization to nu...	0.03527	0.502116	BP
GO:2000394	positive regulation of lamellipodium mor...	0.03527	0.502116	BP

GO:2000668	regulation of dendritic cell apoptotic p...	0.03527	0.502116	BP
GO:0002244	hematopoietic progenitor cell differenti...	0.0354	0.502116	BP
GO:0010243	response to organonitrogen compound	0.03563	0.502116	BP
GO:0030101	natural killer cell activation	0.03625	0.502116	BP
GO:0048857	neural nucleus development	0.03625	0.502116	BP
GO:0051932	synaptic transmission, GABAergic	0.03654	0.502116	BP
GO:0060443	mammary gland morphogenesis	0.03654	0.502116	BP
GO:0002320	lymphoid progenitor cell differentiation	0.03671	0.502116	BP
GO:0002360	T cell lineage commitment	0.03671	0.502116	BP
GO:0003159	morphogenesis of an endothelium	0.03671	0.502116	BP
GO:0003422	growth plate cartilage morphogenesis	0.03671	0.502116	BP
GO:0032660	regulation of interleukin-17 production	0.03671	0.502116	BP
GO:0032930	positive regulation of superoxide anion ...	0.03671	0.502116	BP
GO:0034104	negative regulation of tissue remodeling	0.03671	0.502116	BP
GO:0035728	response to hepatocyte growth factor	0.03671	0.502116	BP
GO:0042454	ribonucleoside catabolic process	0.03671	0.502116	BP
GO:0043649	dicarboxylic acid catabolic process	0.03671	0.502116	BP
GO:0061154	endothelial tube morphogenesis	0.03671	0.502116	BP
GO:0072111	cell proliferation involved in kidney de...	0.03671	0.502116	BP
GO:0099010	modification of postsynaptic structure	0.03671	0.502116	BP
GO:0048639	positive regulation of developmental gro...	0.03696	0.502116	BP
GO:0031116	positive regulation of microtubule polym...	0.03725	0.502116	BP
GO:0032228	regulation of synaptic transmission, GAB...	0.03725	0.502116	BP
GO:0045777	positive regulation of blood pressure	0.03725	0.502116	BP
GO:0046320	regulation of fatty acid oxidation	0.03725	0.502116	BP
GO:0051955	regulation of amino acid transport	0.03725	0.502116	BP
GO:0070570	regulation of neuron projection regenera...	0.03725	0.502116	BP
GO:1905332	positive regulation of morphogenesis of ...	0.03725	0.502116	BP
GO:0061448	connective tissue development	0.03743	0.502116	BP
GO:0008645	hexose transmembrane transport	0.03808	0.502116	BP
GO:0030838	positive regulation of actin filament po...	0.03808	0.502116	BP
GO:0042089	cytokine biosynthetic process	0.03808	0.502116	BP
GO:0051098	regulation of binding	0.0382	0.502116	BP
GO:0051209	release of sequestered calcium ion into ...	0.03827	0.502116	BP
GO:0048332	mesoderm morphogenesis	0.03907	0.502116	BP
GO:0045165	cell fate commitment	0.03925	0.502116	BP
GO:0042129	regulation of T cell proliferation	0.0396	0.502116	BP
GO:0043112	receptor metabolic process	0.03988	0.502116	BP
GO:0002063	chondrocyte development	0.03991	0.502116	BP
GO:0042771	intrinsic apoptotic signaling pathway in...	0.03991	0.502116	BP
GO:1903428	positive regulation of reactive oxygen s...	0.03991	0.502116	BP
GO:0032269	negative regulation of cellular protein ...	0.04003	0.502116	BP
GO:0046631	alpha-beta T cell activation	0.04033	0.502116	BP
GO:0042107	cytokine metabolic process	0.04034	0.502116	BP
GO:0042303	molting cycle	0.04034	0.502116	BP
GO:0042633	hair cycle	0.04034	0.502116	BP
GO:0050905	neuromuscular process	0.04034	0.502116	BP
GO:0071705	nitrogen compound transport	0.04058	0.502116	BP
GO:0043549	regulation of kinase activity	0.04113	0.502116	BP
GO:0002237	response to molecule of bacterial origin	0.04129	0.502116	BP
GO:0008361	regulation of cell size	0.04148	0.502116	BP
GO:0010573	vascular endothelial growth factor produ...	0.04153	0.502116	BP

GO:0010762	regulation of fibroblast migration	0.04153	0.502116	BP
GO:0042269	regulation of natural killer cell mediat...	0.04153	0.502116	BP
GO:0042554	superoxide anion generation	0.04153	0.502116	BP
GO:0044319	wound healing, spreading of cells	0.04153	0.502116	BP
GO:0090184	positive regulation of kidney developmen...	0.04153	0.502116	BP
GO:0090314	positive regulation of protein targeting...	0.04153	0.502116	BP
GO:0090505	epiboly involved in wound healing	0.04153	0.502116	BP
GO:0099068	postsynapse assembly	0.04153	0.502116	BP
GO:1900117	regulation of execution phase of apoptos...	0.04153	0.502116	BP
GO:0060993	kidney morphogenesis	0.04179	0.502116	BP
GO:0098659	inorganic cation import across plasma me...	0.04179	0.502116	BP
GO:0099587	inorganic ion import across plasma membr...	0.04179	0.502116	BP
GO:0010950	positive regulation of endopeptidase act...	0.04194	0.502116	BP
GO:0002683	negative regulation of immune system pro...	0.042	0.502116	BP
GO:0032332	positive regulation of chondrocyte diffe...	0.04265	0.502116	BP
GO:0035278	miRNA mediated inhibition of translation	0.04265	0.502116	BP
GO:0040033	negative regulation of translation, ncRN...	0.04265	0.502116	BP
GO:0045974	regulation of translation, ncRNA-mediate...	0.04265	0.502116	BP
GO:0048643	positive regulation of skeletal muscle t...	0.04265	0.502116	BP
GO:0061081	positive regulation of myeloid leukocyte...	0.04265	0.502116	BP
GO:0070102	interleukin-6-mediated signaling pathway	0.04265	0.502116	BP
GO:0071498	cellular response to fluid shear stress	0.04265	0.502116	BP
GO:0072202	cell differentiation involved in metanep...	0.04265	0.502116	BP
GO:0086014	atrial cardiac muscle cell action potent...	0.04265	0.502116	BP
GO:0086026	atrial cardiac muscle cell to AV node ce...	0.04265	0.502116	BP
GO:0086066	atrial cardiac muscle cell to AV node ce...	0.04265	0.502116	BP
GO:0098877	neurotransmitter receptor transport to p...	0.04265	0.502116	BP
GO:0098969	neurotransmitter receptor transport to p...	0.04265	0.502116	BP
GO:1903540	establishment of protein localization to...	0.04265	0.502116	BP
GO:0051223	regulation of protein transport	0.0429	0.502116	BP
GO:0002253	activation of immune response	0.04294	0.502116	BP
GO:0031103	axon regeneration	0.04345	0.502116	BP
GO:0032330	regulation of chondrocyte differentiatio...	0.04345	0.502116	BP
GO:0035176	social behavior	0.04345	0.502116	BP
GO:0051703	intraspecies interaction between organis...	0.04345	0.502116	BP
GO:0009416	response to light stimulus	0.04348	0.502116	BP
GO:0032102	negative regulation of response to exter...	0.04348	0.502116	BP
GO:0008104	protein localization	0.04364	0.502116	BP
GO:0060759	regulation of response to cytokine stimu...	0.0437	0.502116	BP
GO:0001970	positive regulation of activation of mem...	0.04416	0.502116	BP
GO:0002035	brain renin-angiotensin system	0.04416	0.502116	BP
GO:0002290	gamma-delta T cell activation involved i...	0.04416	0.502116	BP
GO:0002384	hepatic immune response	0.04416	0.502116	BP
GO:0002416	IgG immunoglobulin transcytosis in epith...	0.04416	0.502116	BP
GO:0003051	angiotensin-mediated drinking behavior	0.04416	0.502116	BP
GO:0003353	positive regulation of cilium movement	0.04416	0.502116	BP
GO:0006542	glutamine biosynthetic process	0.04416	0.502116	BP
GO:0007014	actin ubiquitination	0.04416	0.502116	BP
GO:0007199	G protein-coupled receptor signaling pat...	0.04416	0.502116	BP
GO:0009444	pyruvate oxidation	0.04416	0.502116	BP
GO:0009814	defense response, incompatible interacti...	0.04416	0.502116	BP
GO:0009817	defense response to fungus, incompatible...	0.04416	0.502116	BP

GO:0010814	substance P catabolic process	0.04416	0.502116	BP
GO:0010816	calcitonin catabolic process	0.04416	0.502116	BP
GO:0014006	regulation of microglia differentiation	0.04416	0.502116	BP
GO:0014008	positive regulation of microglia differe...	0.04416	0.502116	BP
GO:0014828	distal stomach smooth muscle contraction	0.04416	0.502116	BP
GO:0014873	response to muscle activity involved in ...	0.04416	0.502116	BP
GO:0015774	polysaccharide transport	0.04416	0.502116	BP
GO:0015782	CMP-N-acetylneuraminate transmembrane tr...	0.04416	0.502116	BP
GO:0015826	threonine transport	0.04416	0.502116	BP
GO:0019676	ammonia assimilation cycle	0.04416	0.502116	BP
GO:0021508	floor plate formation	0.04416	0.502116	BP
GO:0021569	rhombomere 3 development	0.04416	0.502116	BP
GO:0021593	rhombomere morphogenesis	0.04416	0.502116	BP
GO:0021594	rhombomere formation	0.04416	0.502116	BP
GO:0021615	glossopharyngeal nerve morphogenesis	0.04416	0.502116	BP
GO:0021644	vagus nerve morphogenesis	0.04416	0.502116	BP
GO:0021658	rhombomere 3 morphogenesis	0.04416	0.502116	BP
GO:0021660	rhombomere 3 formation	0.04416	0.502116	BP
GO:0021664	rhombomere 5 morphogenesis	0.04416	0.502116	BP
GO:0021666	rhombomere 5 formation	0.04416	0.502116	BP
GO:0021771	lateral geniculate nucleus development	0.04416	0.502116	BP
GO:0021793	chemorepulsion of branchiomotor axon	0.04416	0.502116	BP
GO:0021822	negative regulation of cell motility inv...	0.04416	0.502116	BP
GO:0031550	positive regulation of brain-derived neu...	0.04416	0.502116	BP
GO:0032601	connective tissue growth factor producti...	0.04416	0.502116	BP
GO:0032605	hepatocyte growth factor production	0.04416	0.502116	BP
GO:0032643	regulation of connective tissue growth f...	0.04416	0.502116	BP
GO:0032646	regulation of hepatocyte growth factor p...	0.04416	0.502116	BP
GO:0032686	negative regulation of hepatocyte growth...	0.04416	0.502116	BP
GO:0032723	positive regulation of connective tissue...	0.04416	0.502116	BP
GO:0032848	negative regulation of cellular pH reduc...	0.04416	0.502116	BP
GO:0033030	negative regulation of neutrophil apopto...	0.04416	0.502116	BP
GO:0034589	hydroxyproline transport	0.04416	0.502116	BP
GO:0034959	endothelin maturation	0.04416	0.502116	BP
GO:0035548	negative regulation of interferon-beta s...	0.04416	0.502116	BP
GO:0036392	chemokine (C-C motif) ligand 20 producti...	0.04416	0.502116	BP
GO:0036497	eIF2alpha dephosphorylation in response ...	0.04416	0.502116	BP
GO:0038097	positive regulation of mast cell activat...	0.04416	0.502116	BP
GO:0040040	thermosensory behavior	0.04416	0.502116	BP
GO:0043375	CD8-positive, alpha-beta T cell lineage ...	0.04416	0.502116	BP
GO:0043417	negative regulation of skeletal muscle t...	0.04416	0.502116	BP
GO:0044328	canonical Wnt signaling pathway involved...	0.04416	0.502116	BP
GO:0044329	canonical Wnt signaling pathway involved...	0.04416	0.502116	BP
GO:0044330	canonical Wnt signaling pathway involved...	0.04416	0.502116	BP
GO:0045079	negative regulation of chemokine biosynt...	0.04416	0.502116	BP
GO:0045355	negative regulation of interferon-alpha ...	0.04416	0.502116	BP
GO:0045358	negative regulation of interferon-beta b...	0.04416	0.502116	BP
GO:0045918	negative regulation of cytolysis	0.04416	0.502116	BP
GO:0046092	deoxycytidine metabolic process	0.04416	0.502116	BP
GO:0046671	negative regulation of retinal cell prog...	0.04416	0.502116	BP
GO:0046725	negative regulation by virus of viral pr...	0.04416	0.502116	BP
GO:0046814	coreceptor-mediated virion attachment to...	0.04416	0.502116	BP

GO:0048175	hepatocyte growth factor biosynthetic pr...	0.04416	0.502116	BP
GO:0048176	regulation of hepatocyte growth factor b...	0.04416	0.502116	BP
GO:0048178	negative regulation of hepatocyte growth...	0.04416	0.502116	BP
GO:0048264	determination of ventral identity	0.04416	0.502116	BP
GO:0048390	intermediate mesoderm morphogenesis	0.04416	0.502116	BP
GO:0048391	intermediate mesoderm formation	0.04416	0.502116	BP
GO:0048392	intermediate mesodermal cell differentia...	0.04416	0.502116	BP
GO:0048789	cytoskeletal matrix organization at acti...	0.04416	0.502116	BP
GO:0051892	negative regulation of cardioblast diffe...	0.04416	0.502116	BP
GO:0055018	regulation of cardiac muscle fiber devel...	0.04416	0.502116	BP
GO:0055020	positive regulation of cardiac muscle fi...	0.04416	0.502116	BP
GO:0060301	positive regulation of cytokine activity	0.04416	0.502116	BP
GO:0060503	bud dilation involved in lung branching	0.04416	0.502116	BP
GO:0060741	prostate gland stromal morphogenesis	0.04416	0.502116	BP
GO:0061149	BMP signaling pathway involved in ureter...	0.04416	0.502116	BP
GO:0061150	renal system segmentation	0.04416	0.502116	BP
GO:0061151	BMP signaling pathway involved in renal ...	0.04416	0.502116	BP
GO:0061155	pulmonary artery endothelial tube morpho...	0.04416	0.502116	BP
GO:0061216	regulation of transcription from RNA pol...	0.04416	0.502116	BP
GO:0061218	negative regulation of mesonephros devel...	0.04416	0.502116	BP
GO:0061682	seminal vesicle morphogenesis	0.04416	0.502116	BP
GO:0061757	leukocyte adhesion to arterial endotheli...	0.04416	0.502116	BP
GO:0070367	negative regulation of hepatocyte differ...	0.04416	0.502116	BP
GO:0070408	carbamoyl phosphate metabolic process	0.04416	0.502116	BP
GO:0070409	carbamoyl phosphate biosynthetic process	0.04416	0.502116	BP
GO:0070430	positive regulation of nucleotide-bindin...	0.04416	0.502116	BP
GO:0071449	cellular response to lipid hydroperoxide	0.04416	0.502116	BP
GO:0071464	cellular response to hydrostatic pressur...	0.04416	0.502116	BP
GO:0071893	BMP signaling pathway involved in nephri...	0.04416	0.502116	BP
GO:0071911	synchronous neurotransmitter secretion	0.04416	0.502116	BP
GO:0072046	establishment of planar polarity involve...	0.04416	0.502116	BP
GO:0072055	renal cortex development	0.04416	0.502116	BP
GO:0072059	cortical collecting duct development	0.04416	0.502116	BP
GO:0072096	negative regulation of branch elongation...	0.04416	0.502116	BP
GO:0072097	negative regulation of branch elongation...	0.04416	0.502116	BP
GO:0072099	anterior/posterior pattern specification...	0.04416	0.502116	BP
GO:0072100	specification of ureteric bud anterior/p...	0.04416	0.502116	BP
GO:0072101	specification of ureteric bud anterior/p...	0.04416	0.502116	BP
GO:0072181	mesonephric duct formation	0.04416	0.502116	BP
GO:0072192	ureter epithelial cell differentiation	0.04416	0.502116	BP
GO:0072200	negative regulation of mesenchymal cell ...	0.04416	0.502116	BP
GO:0072214	metanephric cortex development	0.04416	0.502116	BP
GO:0072219	metanephric cortical collecting duct dev...	0.04416	0.502116	BP
GO:0072308	negative regulation of metanephric nephr...	0.04416	0.502116	BP
GO:0072428	signal transduction involved in intra-S ...	0.04416	0.502116	BP
GO:0090191	negative regulation of branching involve...	0.04416	0.502116	BP
GO:0097115	neurexin clustering involved in presynap...	0.04416	0.502116	BP
GO:0097326	melanocyte adhesion	0.04416	0.502116	BP
GO:0097510	base-excision repair, AP site formation ...	0.04416	0.502116	BP
GO:0097639	L-lysine import across plasma membrane	0.04416	0.502116	BP
GO:0098746	fast, calcium ion-dependent exocytosis o...	0.04416	0.502116	BP
GO:0099551	trans-synaptic signaling by neuropeptide...	0.04416	0.502116	BP

GO:0106022	positive regulation of vesicle docking	0.04416	0.502116	BP
GO:0106028	neuron projection retraction	0.04416	0.502116	BP
GO:0110077	vesicle-mediated intercellular transport	0.04416	0.502116	BP
GO:0120064	stomach pylorus smooth muscle contractio...	0.04416	0.502116	BP
GO:0120065	pyloric antrum smooth muscle contraction	0.04416	0.502116	BP
GO:0120071	regulation of pyloric antrum smooth musc...	0.04416	0.502116	BP
GO:0120072	positive regulation of pyloric antrum sm...	0.04416	0.502116	BP
GO:0120158	positive regulation of collagen cataboli...	0.04416	0.502116	BP
GO:0150033	negative regulation of protein localizat...	0.04416	0.502116	BP
GO:1900477	negative regulation of G1/S transition o...	0.04416	0.502116	BP
GO:1901585	regulation of acid-sensing ion channel a...	0.04416	0.502116	BP
GO:1901609	negative regulation of vesicle transport...	0.04416	0.502116	BP
GO:1901627	negative regulation of postsynaptic memb...	0.04416	0.502116	BP
GO:1901631	positive regulation of presynaptic membr...	0.04416	0.502116	BP
GO:1901706	mesenchymal cell differentiation involve...	0.04416	0.502116	BP
GO:1901964	positive regulation of cell proliferatio...	0.04416	0.502116	BP
GO:1902378	VEGF-activated neuropilin signaling path...	0.04416	0.502116	BP
GO:1902464	regulation of histone H3-K27 trimethylat...	0.04416	0.502116	BP
GO:1902466	positive regulation of histone H3-K27 tr...	0.04416	0.502116	BP
GO:1902684	negative regulation of receptor localiza...	0.04416	0.502116	BP
GO:1902721	negative regulation of prolactin secreti...	0.04416	0.502116	BP
GO:1903235	positive regulation of calcium ion-depen...	0.04416	0.502116	BP
GO:1903280	negative regulation of calcium:sodium an...	0.04416	0.502116	BP
GO:1903465	positive regulation of mitotic cell cycl...	0.04416	0.502116	BP
GO:1903717	response to ammonia	0.04416	0.502116	BP
GO:1903718	cellular response to ammonia	0.04416	0.502116	BP
GO:1903743	negative regulation of anterograde synap...	0.04416	0.502116	BP
GO:1903881	regulation of interleukin-17-mediated si...	0.04416	0.502116	BP
GO:1903883	positive regulation of interleukin-17-me...	0.04416	0.502116	BP
GO:1903884	regulation of chemokine (C-C motif) liga...	0.04416	0.502116	BP
GO:1903886	positive regulation of chemokine (C-C mo...	0.04416	0.502116	BP
GO:1903910	negative regulation of receptor clusteri...	0.04416	0.502116	BP
GO:1903916	regulation of endoplasmic reticulum stre...	0.04416	0.502116	BP
GO:1903917	positive regulation of endoplasmic retic...	0.04416	0.502116	BP
GO:1903925	response to bisphenol A	0.04416	0.502116	BP
GO:1903926	cellular response to bisphenol A	0.04416	0.502116	BP
GO:1904274	tricellular tight junction assembly	0.04416	0.502116	BP
GO:1904341	regulation of colon smooth muscle contra...	0.04416	0.502116	BP
GO:1904343	positive regulation of colon smooth musc...	0.04416	0.502116	BP
GO:1904394	negative regulation of skeletal muscle a...	0.04416	0.502116	BP
GO:1904477	positive regulation of Ras GTPase bindin...	0.04416	0.502116	BP
GO:1904692	positive regulation of type B pancreatic...	0.04416	0.502116	BP
GO:1904782	negative regulation of NMDA glutamate re...	0.04416	0.502116	BP
GO:1904980	positive regulation of endosome organiza...	0.04416	0.502116	BP
GO:1904997	regulation of leukocyte adhesion to arte...	0.04416	0.502116	BP
GO:1904998	negative regulation of leukocyte adhesio...	0.04416	0.502116	BP
GO:1905008	regulation of L-lysine import across pla...	0.04416	0.502116	BP
GO:1905010	positive regulation of L-lysine import a...	0.04416	0.502116	BP
GO:1905040	otic placode development	0.04416	0.502116	BP
GO:1905099	positive regulation of guanyl-nucleotide...	0.04416	0.502116	BP
GO:1905219	regulation of platelet formation	0.04416	0.502116	BP
GO:1905221	positive regulation of platelet formatio...	0.04416	0.502116	BP

GO:1905313	transforming growth factor beta receptor...	0.04416	0.502116	BP
GO:1905518	regulation of presynaptic active zone as...	0.04416	0.502116	BP
GO:1905520	positive regulation of presynaptic activ...	0.04416	0.502116	BP
GO:1905541	regulation of L-arginine import across p...	0.04416	0.502116	BP
GO:1905589	positive regulation of L-arginine import...	0.04416	0.502116	BP
GO:1905608	positive regulation of presynapse assemb...	0.04416	0.502116	BP
GO:1905687	regulation of diacylglycerol kinase acti...	0.04416	0.502116	BP
GO:1905689	positive regulation of diacylglycerol ki...	0.04416	0.502116	BP
GO:1905875	negative regulation of postsynaptic dens...	0.04416	0.502116	BP
GO:1990402	embryonic liver development	0.04416	0.502116	BP
GO:1990765	colon smooth muscle contraction	0.04416	0.502116	BP
GO:1990959	eosinophil homeostasis	0.04416	0.502116	BP
GO:1990960	basophil homeostasis	0.04416	0.502116	BP
GO:2000004	regulation of metanephric S-shaped body ...	0.04416	0.502116	BP
GO:2000005	negative regulation of metanephric S-sha...	0.04416	0.502116	BP
GO:2000006	regulation of metanephric comma-shaped b...	0.04416	0.502116	BP
GO:2000007	negative regulation of metanephric comma...	0.04416	0.502116	BP
GO:2000212	negative regulation of glutamate metabol...	0.04416	0.502116	BP
GO:2000565	negative regulation of CD8-positive, alp...	0.04416	0.502116	BP
GO:2000690	regulation of cardiac muscle cell myobla...	0.04416	0.502116	BP
GO:2000691	negative regulation of cardiac muscle ce...	0.04416	0.502116	BP
GO:2000830	positive regulation of parathyroid hormo...	0.04416	0.502116	BP
GO:2001029	regulation of cellular glucuronidation	0.04416	0.502116	BP
GO:2001031	positive regulation of cellular glucuron...	0.04416	0.502116	BP
GO:2001108	positive regulation of Rho guanyl-nucleo...	0.04416	0.502116	BP
GO:2001191	regulation of gamma-delta T cell activat...	0.04416	0.502116	BP
GO:2001193	positive regulation of gamma-delta T cel...	0.04416	0.502116	BP
GO:0045665	negative regulation of neuron differenti...	0.04426	0.502272	BP
GO:2000116	regulation of cysteine-type endopeptidas...	0.04426	0.502272	BP
GO:1903827	regulation of cellular protein localizat...	0.04427	0.502272	BP
GO:0001942	hair follicle development	0.04448	0.50356	BP
GO:0051291	protein heterooligomerization	0.04468	0.50356	BP
GO:0061387	regulation of extent of cell growth	0.04468	0.50356	BP
GO:0071396	cellular response to lipid	0.04485	0.50356	BP
GO:0014910	regulation of smooth muscle cell migrati...	0.0451	0.50356	BP
GO:0035308	negative regulation of protein dephospho...	0.0451	0.50356	BP
GO:0045844	positive regulation of striated muscle t...	0.0451	0.50356	BP
GO:0048636	positive regulation of muscle organ deve...	0.0451	0.50356	BP
GO:0060193	positive regulation of lipase activity	0.0451	0.50356	BP
GO:0061333	renal tubule morphogenesis	0.0451	0.50356	BP
GO:0043648	dicarboxylic acid metabolic process	0.04513	0.50356	BP
GO:1903708	positive regulation of hemopoiesis	0.04551	0.50356	BP
GO:0035107	appendage morphogenesis	0.04562	0.50356	BP
GO:0035108	limb morphogenesis	0.04562	0.50356	BP
GO:0001955	blood vessel maturation	0.04567	0.50356	BP
GO:0002295	T-helper cell lineage commitment	0.04567	0.50356	BP
GO:0006538	glutamate catabolic process	0.04567	0.50356	BP
GO:0010958	regulation of amino acid import across p...	0.04567	0.50356	BP
GO:0021548	pons development	0.04567	0.50356	BP
GO:0021910	smoothened signaling pathway involved in...	0.04567	0.50356	BP
GO:0033079	immature T cell proliferation	0.04567	0.50356	BP
GO:0034310	primary alcohol catabolic process	0.04567	0.50356	BP

GO:0036066	protein O-linked fucosylation	0.04567	0.50356	BP
GO:0046133	pyrimidine ribonucleoside catabolic proc...	0.04567	0.50356	BP
GO:0060363	cranial suture morphogenesis	0.04567	0.50356	BP
GO:0060600	dichotomous subdivision of an epithelial...	0.04567	0.50356	BP
GO:0060907	positive regulation of macrophage cytoki...	0.04567	0.50356	BP
GO:0070778	L-aspartate transmembrane transport	0.04567	0.50356	BP
GO:0072110	glomerular mesangial cell proliferation	0.04567	0.50356	BP
GO:0090185	negative regulation of kidney developmen...	0.04567	0.50356	BP
GO:0090269	fibroblast growth factor production	0.04567	0.50356	BP
GO:0090270	regulation of fibroblast growth factor p...	0.04567	0.50356	BP
GO:0097113	AMPA glutamate receptor clustering	0.04567	0.50356	BP
GO:0097688	glutamate receptor clustering	0.04567	0.50356	BP
GO:0099545	trans-synaptic signaling by trans-synapt...	0.04567	0.50356	BP
GO:1900125	regulation of hyaluronan biosynthetic pr...	0.04567	0.50356	BP
GO:1902965	regulation of protein localization to ea...	0.04567	0.50356	BP
GO:1902966	positive regulation of protein localizat...	0.04567	0.50356	BP
GO:1905247	positive regulation of aspartic-type pep...	0.04567	0.50356	BP
GO:1990709	presynaptic active zone organization	0.04567	0.50356	BP
GO:0022408	negative regulation of cell-cell adhesio...	0.04582	0.504858	BP
GO:0060603	mammary gland duct morphogenesis	0.04608	0.505344	BP
GO:0070884	regulation of calcineurin-NFAT signaling...	0.04608	0.505344	BP
GO:0090322	regulation of superoxide metabolic proce...	0.04608	0.505344	BP
GO:0090504	epiboly	0.04608	0.505344	BP
GO:0097028	dendritic cell differentiation	0.04608	0.505344	BP
GO:0106056	regulation of calcineurin-mediated signa...	0.04608	0.505344	BP
GO:0070848	response to growth factor	0.04609	0.505344	BP
GO:0061024	membrane organization	0.04655	0.51003	BP
GO:0008347	glial cell migration	0.04718	0.514881	BP
GO:0019229	regulation of vasoconstriction	0.04718	0.514881	BP
GO:0050919	negative chemotaxis	0.04718	0.514881	BP
GO:0051646	mitochondrion localization	0.04718	0.514881	BP
GO:0090303	positive regulation of wound healing	0.04718	0.514881	BP
GO:0010038	response to metal ion	0.04719	0.514881	BP
GO:1903426	regulation of reactive oxygen species bi...	0.04727	0.515394	BP
GO:0006939	smooth muscle contraction	0.04765	0.519176	BP
GO:0048592	eye morphogenesis	0.04775	0.519904	BP
GO:0015698	inorganic anion transport	0.04781	0.520195	BP
GO:0071902	positive regulation of protein serine/th...	0.04807	0.522661	BP
GO:0006809	nitric oxide biosynthetic process	0.04833	0.524032	BP
GO:0033077	T cell differentiation in thymus	0.04833	0.524032	BP
GO:0035019	somatic stem cell population maintenance	0.04833	0.524032	BP
GO:1901863	positive regulation of muscle tissue dev...	0.04833	0.524032	BP
GO:0003177	pulmonary valve development	0.04904	0.527711	BP
GO:0007549	dosage compensation	0.04904	0.527711	BP
GO:0010884	positive regulation of lipid storage	0.04904	0.527711	BP
GO:0016082	synaptic vesicle priming	0.04904	0.527711	BP
GO:0033194	response to hydroperoxide	0.04904	0.527711	BP
GO:0035455	response to interferon-alpha	0.04904	0.527711	BP
GO:0036315	cellular response to sterol	0.04904	0.527711	BP
GO:0044342	type B pancreatic cell proliferation	0.04904	0.527711	BP
GO:0048670	regulation of collateral sprouting	0.04904	0.527711	BP
GO:0051497	negative regulation of stress fiber asse...	0.04904	0.527711	BP

GO:0072170	metanephric tubule development	0.04904	0.527711	BP
GO:2001236	regulation of extrinsic apoptotic signal...	0.04987	0.536274	BP
GO:0002262	myeloid cell homeostasis	0.04995	0.536765	BP
GO:1903706	regulation of hemopoiesis	0.04999	0.536827	BP
GO:0022890	inorganic cation transmembrane transport...	1.4E-09	6.3E-06	MF
GO:0005215	transporter activity	6.8E-09	1.05E-05	MF
GO:0008324	cation transmembrane transporter activit...	8.8E-09	1.05E-05	MF
GO:0046873	metal ion transmembrane transporter acti...	9.9E-09	1.05E-05	MF
GO:0015318	inorganic molecular entity transmembrane...	1.3E-08	1.05E-05	MF
GO:0038023	signaling receptor activity	1.4E-08	1.05E-05	MF
GO:0022857	transmembrane transporter activity	1.7E-08	1.09E-05	MF
GO:0005261	cation channel activity	4.9E-08	2.75E-05	MF
GO:0015075	ion transmembrane transporter activity	5.5E-08	2.75E-05	MF
GO:0060089	molecular transducer activity	1.4E-07	6.3E-05	MF
GO:0015267	channel activity	2.7E-07	0.000109	MF
GO:0022803	passive transmembrane transporter activi...	2.9E-07	0.000109	MF
GO:0001540	amyloid-beta binding	5.1E-07	0.000176	MF
GO:0022838	substrate-specific channel activity	5.5E-07	0.000177	MF
GO:0005216	ion channel activity	9.3E-07	0.000279	MF
GO:0015077	monovalent inorganic cation transmembran...	1.3E-06	0.000366	MF
GO:0004888	transmembrane signaling receptor activit...	1.4E-06	0.000371	MF
GO:0022836	gated channel activity	1.8E-06	0.00045	MF
GO:0022839	ion gated channel activity	2.7E-06	0.000639	MF
GO:0098772	molecular function regulator	2.9E-06	0.000652	MF
GO:0019199	transmembrane receptor protein kinase ac...	4.1E-06	0.000878	MF
GO:0015081	sodium ion transmembrane transporter act...	5.6E-06	0.001145	MF
GO:0015291	secondary active transmembrane transport...	8.5E-06	0.001663	MF
GO:0099094	ligand-gated cation channel activity	0.00001	0.001875	MF
GO:0008289	lipid binding	0.000012	0.00216	MF
GO:0015276	ligand-gated ion channel activity	0.000018	0.002999	MF
GO:0022834	ligand-gated channel activity	0.000018	0.002999	MF
GO:0005543	phospholipid binding	0.000021	0.003374	MF
GO:0043168	anion binding	0.000027	0.004049	MF
GO:0022804	active transmembrane transporter activit...	0.000027	0.004049	MF
GO:0042802	identical protein binding	0.000041	0.00595	MF
GO:0015085	calcium ion transmembrane transporter ac...	0.000046	0.006467	MF
GO:0042169	SH2 domain binding	0.00005	0.006817	MF
GO:0019838	growth factor binding	0.000054	0.007145	MF
GO:0015293	symporter activity	0.000066	0.008484	MF
GO:0030545	receptor regulator activity	0.000088	0.010998	MF
GO:0005509	calcium ion binding	0.00013	0.015807	MF
GO:0015294	solute:cation symporter activity	0.00016	0.018943	MF
GO:0005102	signaling receptor binding	0.00022	0.025379	MF
GO:0004714	transmembrane receptor protein tyrosine ...	0.00023	0.025869	MF
GO:0015079	potassium ion transmembrane transporter ...	0.00031	0.033207	MF
GO:0004971	AMPA glutamate receptor activity	0.00031	0.033207	MF
GO:0005244	voltage-gated ion channel activity	0.00036	0.03681	MF
GO:0022832	voltage-gated channel activity	0.00036	0.03681	MF
GO:0005230	extracellular ligand-gated ion channel a...	0.0004	0.037645	MF
GO:0035091	phosphatidylinositol binding	0.0004	0.037645	MF
GO:0015370	solute:sodium symporter activity	0.00041	0.037645	MF
GO:0022824	transmitter-gated ion channel activity	0.00041	0.037645	MF

GO:0022835	transmitter-gated channel activity	0.00041	0.037645	MF
GO:0016595	glutamate binding	0.00059	0.053088	MF
GO:0008066	glutamate receptor activity	0.00071	0.062633	MF
GO:0015193	L-proline transmembrane transporter acti...	0.00076	0.065755	MF
GO:0004970	ionotropic glutamate receptor activity	0.0008	0.067909	MF
GO:0099604	ligand-gated calcium channel activity	0.00088	0.073317	MF
GO:0022843	voltage-gated cation channel activity	0.00111	0.090798	MF
GO:0017048	Rho GTPase binding	0.00114	0.091531	MF
GO:0048018	receptor ligand activity	0.00116	0.091531	MF
GO:0042277	peptide binding	0.00118	0.091531	MF
GO:0008514	organic anion transmembrane transporter ...	0.00121	0.092268	MF
GO:0004713	protein tyrosine kinase activity	0.0013	0.09588	MF
GO:0005262	calcium channel activity	0.0013	0.09588	MF
GO:0005328	neurotransmitter:sodium symporter activi...	0.00135	0.097962	MF
GO:0008510	sodium:bicarbonate symporter activity	0.00147	0.104977	MF
GO:0019901	protein kinase binding	0.00154	0.107976	MF
GO:0019900	kinase binding	0.00156	0.107976	MF
GO:0019903	protein phosphatase binding	0.00162	0.11043	MF
GO:0033218	amide binding	0.00165	0.110796	MF
GO:0042803	protein homodimerization activity	0.00176	0.116445	MF
GO:0032396	inhibitory MHC class I receptor activity	0.00187	0.121929	MF
GO:0005267	potassium channel activity	0.00193	0.124044	MF
GO:0048365	Rac GTPase binding	0.0023	0.145742	MF
GO:0046983	protein dimerization activity	0.00243	0.151841	MF
GO:0005283	amino acid:sodium symporter activity	0.00247	0.152226	MF
GO:0019899	enzyme binding	0.00269	0.163545	MF
GO:0004867	serine-type endopeptidase inhibitor acti...	0.00336	0.201555	MF
GO:0005096	GTPase activator activity	0.00369	0.218438	MF
GO:0005343	organic acid:sodium symporter activity	0.00388	0.225527	MF
GO:0019902	phosphatase binding	0.00391	0.225527	MF
GO:0004672	protein kinase activity	0.00416	0.232726	MF
GO:0004675	transmembrane receptor protein serine/th...	0.00419	0.232726	MF
GO:0005416	amino acid:cation symporter activity	0.00419	0.232726	MF
GO:1902936	phosphatidylinositol bisphosphate bindin...	0.00428	0.234826	MF
GO:0017075	syntaxin-1 binding	0.00463	0.250968	MF
GO:0061135	endopeptidase regulator activity	0.00476	0.254943	MF
GO:0015179	L-amino acid transmembrane transporter a...	0.00489	0.258825	MF
GO:0016773	phosphotransferase activity, alcohol gro...	0.00495	0.258954	MF
GO:0005342	organic acid transmembrane transporter a...	0.00509	0.260226	MF
GO:0046943	carboxylic acid transmembrane transporte...	0.00509	0.260226	MF
GO:0005249	voltage-gated potassium channel activity	0.00543	0.261325	MF
GO:0005030	neurotrophin receptor activity	0.00546	0.261325	MF
GO:0005220	inositol 1,4,5-trisphosphate-sensitive c...	0.00546	0.261325	MF
GO:0034987	immunoglobulin receptor binding	0.00546	0.261325	MF
GO:0046573	lactonohydrolase activity	0.00546	0.261325	MF
GO:0102007	acyl-L-homoserine-lactone lactonohydrola...	0.00546	0.261325	MF
GO:0030234	enzyme regulator activity	0.00639	0.301016	MF
GO:0030695	GTPase regulator activity	0.00643	0.301016	MF
GO:0008047	enzyme activator activity	0.00649	0.301016	MF
GO:0005248	voltage-gated sodium channel activity	0.00657	0.301617	MF
GO:0030594	neurotransmitter receptor activity	0.0069	0.313567	MF
GO:0003779	actin binding	0.00712	0.320329	MF

GO:0015171	amino acid transmembrane transporter act...	0.00744	0.331411	MF
GO:0033130	acetylcholine receptor binding	0.00773	0.340954	MF
GO:0005326	neurotransmitter transporter activity	0.00797	0.348127	MF
GO:0004866	endopeptidase inhibitor activity	0.0081	0.350403	MF
GO:0005516	calmodulin binding	0.00854	0.362836	MF
GO:0019955	cytokine binding	0.00859	0.362836	MF
GO:0015662	ATPase activity, coupled to transmembran...	0.00871	0.362836	MF
GO:0051721	protein phosphatase 2A binding	0.00871	0.362836	MF
GO:0016301	kinase activity	0.00892	0.366464	MF
GO:1904315	transmitter-gated ion channel activity i...	0.00896	0.366464	MF
GO:1901681	sulfur compound binding	0.00953	0.386265	MF
GO:0008144	drug binding	0.0101	0.391265	MF
GO:0097367	carbohydrate derivative binding	0.01019	0.391265	MF
GO:0005452	inorganic anion exchanger activity	0.01029	0.391265	MF
GO:0015368	calcium:cation antiporter activity	0.01029	0.391265	MF
GO:0008509	anion transmembrane transporter activity	0.01044	0.391265	MF
GO:0005314	high-affinity glutamate transmembrane tr...	0.01061	0.391265	MF
GO:0015333	peptide:proton symporter activity	0.01061	0.391265	MF
GO:0015467	G-protein activated inward rectifier pot...	0.01061	0.391265	MF
GO:0015501	glutamate:sodium symporter activity	0.01061	0.391265	MF
GO:0023025	MHC class Ib protein complex binding	0.01061	0.391265	MF
GO:0048763	calcium-induced calcium release activity	0.01061	0.391265	MF
GO:0030414	peptidase inhibitor activity	0.01116	0.407087	MF
GO:0005544	calcium-dependent phospholipid binding	0.01122	0.407087	MF
GO:0019905	syntaxin binding	0.01143	0.411389	MF
GO:0015278	calcium-release channel activity	0.0116	0.414194	MF
GO:0043167	ion binding	0.01221	0.432542	MF
GO:0035254	glutamate receptor binding	0.01248	0.435252	MF
GO:0099529	neurotransmitter receptor activity invol...	0.01248	0.435252	MF
GO:0015125	bile acid transmembrane transporter acti...	0.01328	0.454636	MF
GO:0017154	semaphorin receptor activity	0.01328	0.454636	MF
GO:0005179	hormone activity	0.0134	0.454636	MF
GO:1901981	phosphatidylinositol phosphate binding	0.01344	0.454636	MF
GO:0060589	nucleoside-triphosphatase regulator acti...	0.01359	0.456279	MF
GO:0098960	postsynaptic neurotransmitter receptor a...	0.01531	0.51022	MF
GO:0004029	aldehyde dehydrogenase (NAD) activity	0.01672	0.540195	MF
GO:0005432	calcium:sodium antiporter activity	0.01717	0.540195	MF
GO:0022849	glutamate-gated calcium ion channel acti...	0.01717	0.540195	MF
GO:0030550	acetylcholine receptor inhibitor activit...	0.01717	0.540195	MF
GO:0031730	CCR5 chemokine receptor binding	0.01717	0.540195	MF
GO:0032393	MHC class I receptor activity	0.01717	0.540195	MF
GO:0038132	neuregulin binding	0.01717	0.540195	MF
GO:0042289	MHC class II protein binding	0.01717	0.540195	MF
GO:0032559	adenyl ribonucleotide binding	0.01791	0.559563	MF
GO:0030547	receptor inhibitor activity	0.01859	0.574085	MF
GO:0016903	oxidoreductase activity, acting on the a...	0.01863	0.574085	MF
GO:0072341	modified amino acid binding	0.01939	0.59344	MF
GO:0016247	channel regulator activity	0.01981	0.602197	MF
GO:0036094	small molecule binding	0.02016	0.606042	MF
GO:0005024	transforming growth factor beta-activate...	0.02061	0.606042	MF
GO:0005313	L-glutamate transmembrane transporter ac...	0.02061	0.606042	MF
GO:0015172	acidic amino acid transmembrane transpor...	0.02061	0.606042	MF

GO:0019865	immunoglobulin binding	0.02061	0.606042	MF
GO:0030554	adenyl nucleotide binding	0.02079	0.607365	MF
GO:0015297	antiporter activity	0.02226	0.646114	MF
GO:0001786	phosphatidylserine binding	0.02423	0.678373	MF
GO:0016597	amino acid binding	0.02423	0.678373	MF
GO:0016645	oxidoreductase activity, acting on the C...	0.02444	0.678373	MF
GO:0046875	ephrin receptor binding	0.02444	0.678373	MF
GO:0042166	acetylcholine binding	0.02495	0.678373	MF
GO:0004064	arylesterase activity	0.02503	0.678373	MF
GO:0004126	cytidine deaminase activity	0.02503	0.678373	MF
GO:0005004	GPI-linked ephrin receptor activity	0.02503	0.678373	MF
GO:0005042	netrin receptor activity	0.02503	0.678373	MF
GO:0023029	MHC class Ib protein binding	0.02503	0.678373	MF
GO:0035673	oligopeptide transmembrane transporter a...	0.02503	0.678373	MF
GO:0019904	protein domain specific binding	0.02521	0.67916	MF
GO:0030165	PDZ domain binding	0.02541	0.680474	MF
GO:0005524	ATP binding	0.02767	0.732141	MF
GO:0042287	MHC protein binding	0.02773	0.732141	MF
GO:0008083	growth factor activity	0.02901	0.732141	MF
GO:0005546	phosphatidylinositol-4,5-bisphosphate bi...	0.02936	0.732141	MF
GO:0035259	glucocorticoid receptor binding	0.02974	0.732141	MF
GO:0099106	ion channel regulator activity	0.03096	0.732141	MF
GO:0061134	peptidase regulator activity	0.03124	0.732141	MF
GO:0004972	NMDA glutamate receptor activity	0.03404	0.732141	MF
GO:0005025	transforming growth factor beta receptor...	0.03404	0.732141	MF
GO:0005031	tumor necrosis factor-activated receptor...	0.03404	0.732141	MF
GO:0017002	activin-activated receptor activity	0.03404	0.732141	MF
GO:0017153	sodium:dicarboxylate symporter activity	0.03404	0.732141	MF
GO:0031402	sodium ion binding	0.03404	0.732141	MF
GO:0098821	BMP receptor activity	0.03404	0.732141	MF
GO:0005160	transforming growth factor beta receptor...	0.03407	0.732141	MF
GO:0032555	purine ribonucleotide binding	0.03471	0.732141	MF
GO:0005005	transmembrane-ephrin receptor activity	0.03498	0.732141	MF
GO:0016620	oxidoreductase activity, acting on the a...	0.03508	0.732141	MF
GO:0008092	cytoskeletal protein binding	0.0378	0.732141	MF
GO:0017124	SH3 domain binding	0.03908	0.732141	MF
GO:0071949	FAD binding	0.03914	0.732141	MF
GO:0017076	purine nucleotide binding	0.04014	0.732141	MF
GO:0005003	ephrin receptor activity	0.04066	0.732141	MF
GO:0005242	inward rectifier potassium channel activ...	0.04066	0.732141	MF
GO:0015106	bicarbonate transmembrane transporter ac...	0.04066	0.732141	MF
GO:0016646	oxidoreductase activity, acting on the C...	0.04066	0.732141	MF
GO:0042562	hormone binding	0.0411	0.732141	MF
GO:0032553	ribonucleotide binding	0.0411	0.732141	MF
GO:0000246	delta24(24-1) sterol reductase activity	0.04332	0.732141	MF
GO:0001010	RNA polymerase II sequence-specific DNA-...	0.04332	0.732141	MF
GO:0001642	group III metabotropic glutamate recepto...	0.04332	0.732141	MF
GO:0003842	1-pyrroline-5-carboxylate dehydrogenase ...	0.04332	0.732141	MF
GO:0003947	(N-acetylneuraminyl)-galactosylglucosylc...	0.04332	0.732141	MF
GO:0004051	arachidonate 5-lipoxygenase activity	0.04332	0.732141	MF
GO:0004087	carbamoyl-phosphate synthase (ammonia) a...	0.04332	0.732141	MF
GO:0004356	glutamate-ammonia ligase activity	0.04332	0.732141	MF

GO:0004420	hydroxymethylglutaryl-CoA reductase (NAD...	0.04332	0.732141	MF
GO:0004454	ketohexokinase activity	0.04332	0.732141	MF
GO:0004491	methylmalonate-semialdehyde dehydrogenas...	0.04332	0.732141	MF
GO:0004498	calcidiol 1-monooxygenase activity	0.04332	0.732141	MF
GO:0004648	O-phospho-L-serine:2-oxoglutarate aminot...	0.04332	0.732141	MF
GO:0004657	proline dehydrogenase activity	0.04332	0.732141	MF
GO:0004753	saccharopine dehydrogenase activity	0.04332	0.732141	MF
GO:0004990	oxytocin receptor activity	0.04332	0.732141	MF
GO:0005020	stem cell factor receptor activity	0.04332	0.732141	MF
GO:0005456	CMP-N-acetylneuraminate transmembrane tr...	0.04332	0.732141	MF
GO:0008404	arachidonic acid 14,15-epoxygenase activ...	0.04332	0.732141	MF
GO:0008405	arachidonic acid 11,12-epoxygenase activ...	0.04332	0.732141	MF
GO:0008747	N-acetylneuraminate lyase activity	0.04332	0.732141	MF
GO:0008955	peptidoglycan glycosyltransferase activi...	0.04332	0.732141	MF
GO:0010465	nerve growth factor receptor activity	0.04332	0.732141	MF
GO:0015195	L-threonine transmembrane transporter ac...	0.04332	0.732141	MF
GO:0015292	uniporter activity	0.04332	0.732141	MF
GO:0016211	ammonia ligase activity	0.04332	0.732141	MF
GO:0016213	linoleoyl-CoA desaturase activity	0.04332	0.732141	MF
GO:0017159	pantetheine hydrolase activity	0.04332	0.732141	MF
GO:0018478	malonate-semialdehyde dehydrogenase (ace...	0.04332	0.732141	MF
GO:0018733	3,4-dihydrocoumarin hydrolase activity	0.04332	0.732141	MF
GO:0019978	interleukin-3 binding	0.04332	0.732141	MF
GO:0030107	HLA-A specific inhibitory MHC class I re...	0.04332	0.732141	MF
GO:0030109	HLA-B specific inhibitory MHC class I re...	0.04332	0.732141	MF
GO:0030379	neurotensin receptor activity, non-G pro...	0.04332	0.732141	MF
GO:0031703	type 2 angiotensin receptor binding	0.04332	0.732141	MF
GO:0031714	C5a anaphylatoxin chemotactic receptor b...	0.04332	0.732141	MF
GO:0031715	C5L2 anaphylatoxin chemotactic receptor ...	0.04332	0.732141	MF
GO:0034590	L-hydroxyproline transmembrane transport...	0.04332	0.732141	MF
GO:0035438	cyclic-di-GMP binding	0.04332	0.732141	MF
GO:0038164	thrombopoietin receptor activity	0.04332	0.732141	MF
GO:0042164	interleukin-12 alpha subunit binding	0.04332	0.732141	MF
GO:0042282	hydroxymethylglutaryl-CoA reductase acti...	0.04332	0.732141	MF
GO:0042988	X11-like protein binding	0.04332	0.732141	MF
GO:0043914	NADPH:sulfur oxidoreductase activity	0.04332	0.732141	MF
GO:0043924	suramin binding	0.04332	0.732141	MF
GO:0046789	host cell surface receptor binding	0.04332	0.732141	MF
GO:0046812	host cell surface binding	0.04332	0.732141	MF
GO:0047130	saccharopine dehydrogenase (NADP+, L-lys...	0.04332	0.732141	MF
GO:0047131	saccharopine dehydrogenase (NAD+, L-glut...	0.04332	0.732141	MF
GO:0047933	glucose-1,6-bisphosphate synthase activi...	0.04332	0.732141	MF
GO:0050459	ethanolamine-phosphate phospho-lyase act...	0.04332	0.732141	MF
GO:0050614	delta24-sterol reductase activity	0.04332	0.732141	MF
GO:0050683	AF-1 domain binding	0.04332	0.732141	MF
GO:0051722	protein C-terminal methylesterase activi...	0.04332	0.732141	MF
GO:0051723	protein methylesterase activity	0.04332	0.732141	MF
GO:0051916	granulocyte colony-stimulating factor bi...	0.04332	0.732141	MF
GO:0060175	brain-derived neurotrophic factor-activa...	0.04332	0.732141	MF
GO:0061507	cyclic-GMP-AMP binding	0.04332	0.732141	MF
GO:0061751	neutral sphingomyelin phosphodiesterase ...	0.04332	0.732141	MF
GO:0070119	ciliary neurotrophic factor binding	0.04332	0.732141	MF

GO:0071614	linoleic acid epoxygenase activity	0.04332	0.732141	MF
GO:0071916	dipeptide transmembrane transporter acti...	0.04332	0.732141	MF
GO:0086038	calcium:sodium antiporter activity invol...	0.04332	0.732141	MF
GO:0097161	DH domain binding	0.04332	0.732141	MF
GO:0099077	histone-dependent DNA binding	0.04332	0.732141	MF
GO:0102116	laurate hydroxylase activity	0.04332	0.732141	MF
GO:0103002	16-hydroxypalmitate dehydrogenase activi...	0.04332	0.732141	MF
GO:0120146	sulfatide binding	0.04332	0.732141	MF
GO:1902444	riboflavin binding	0.04332	0.732141	MF
GO:0005217	intracellular ligand-gated ion channel a...	0.04345	0.732141	MF
GO:0035255	ionotropic glutamate receptor binding	0.04345	0.732141	MF
GO:0008046	axon guidance receptor activity	0.04411	0.735003	MF
GO:0043121	neurotrophin binding	0.04411	0.735003	MF
GO:0097677	STAT family protein binding	0.04411	0.735003	MF
GO:0005089	Rho guanyl-nucleotide exchange factor ac...	0.04611	0.765494	MF
GO:0008157	protein phosphatase 1 binding	0.04677	0.772576	MF
GO:0004857	enzyme inhibitor activity	0.04688	0.772576	MF
GO:0035639	purine ribonucleoside triphosphate bindi...	0.04778	0.783644	MF
GO:0019829	cation-transporting ATPase activity	0.0479	0.783644	MF
GO:0005496	steroid binding	0.04891	0.797268	MF
GO:0017016	Ras GTPase binding	0.04919	0.798938	MF
GO:0005886	plasma membrane	1E-25	1.99E-22	CC
GO:0071944	cell periphery	3E-25	2.98E-22	CC
GO:0044459	plasma membrane part	2.6E-17	1.72E-14	CC
GO:0031226	intrinsic component of plasma membrane	9.2E-17	4.57E-14	CC
GO:0005887	integral component of plasma membrane	8.5E-15	3.37E-12	CC
GO:0031224	intrinsic component of membrane	6.6E-14	2.18E-11	CC
GO:0044425	membrane part	2.7E-13	6.7E-11	CC
GO:0044456	synapse part	2.7E-13	6.7E-11	CC
GO:0016020	membrane	5.4E-13	1.19E-10	CC
GO:0043235	receptor complex	7.5E-13	1.49E-10	CC
GO:0045202	synapse	9.2E-13	1.66E-10	CC
GO:0016021	integral component of membrane	4.7E-12	7.63E-10	CC
GO:0098794	postsynapse	5E-12	7.63E-10	CC
GO:0042995	cell projection	6.9E-12	9.78E-10	CC
GO:0097458	neuron part	7.8E-12	1.03E-09	CC
GO:0034703	cation channel complex	9.2E-12	1.11E-09	CC
GO:0098590	plasma membrane region	9.5E-12	1.11E-09	CC
GO:0120025	plasma membrane bounded cell projection	1.6E-11	1.76E-09	CC
GO:1902495	transmembrane transporter complex	6.8E-11	7.1E-09	CC
GO:0034702	ion channel complex	1.1E-10	1.09E-08	CC
GO:0099634	postsynaptic specialization membrane	1.2E-10	1.13E-08	CC
GO:1990351	transporter complex	1.6E-10	1.44E-08	CC
GO:0043005	neuron projection	2.2E-10	1.9E-08	CC
GO:0099572	postsynaptic specialization	1.2E-09	9.93E-08	CC
GO:0097060	synaptic membrane	1.3E-09	1.03E-07	CC
GO:0098839	postsynaptic density membrane	1.4E-09	1.07E-07	CC
GO:0030054	cell junction	1.5E-09	1.1E-07	CC
GO:0098984	neuron to neuron synapse	1.6E-09	1.13E-07	CC
GO:0099055	integral component of postsynaptic membr...	2.3E-09	1.57E-07	CC
GO:0099699	integral component of synaptic membrane	2.4E-09	1.59E-07	CC
GO:0032279	asymmetric synapse	2.9E-09	1.86E-07	CC

GO:0098978	glutamatergic synapse	3.1E-09	1.92E-07	CC
GO:0098936	intrinsic component of postsynaptic memb...	5.3E-09	3.19E-07	CC
GO:0099060	integral component of postsynaptic speci...	6.5E-09	3.74E-07	CC
GO:0014069	postsynaptic density	6.6E-09	3.74E-07	CC
GO:0045211	postsynaptic membrane	8.1E-09	4.47E-07	CC
GO:0036477	somatodendritic compartment	8.8E-09	4.72E-07	CC
GO:0099240	intrinsic component of synaptic membrane	1.1E-08	5.75E-07	CC
GO:0098948	intrinsic component of postsynaptic spec...	1.3E-08	6.62E-07	CC
GO:0009986	cell surface	1.4E-08	6.95E-07	CC
GO:0098793	presynapse	1.7E-08	8.23E-07	CC
GO:0030424	axon	5.6E-08	2.65E-06	CC
GO:0098797	plasma membrane protein complex	5.8E-08	2.68E-06	CC
GO:0099061	integral component of postsynaptic densi...	1.1E-07	4.96E-06	CC
GO:0099146	intrinsic component of postsynaptic dens...	2.3E-07	1.01E-05	CC
GO:0044463	cell projection part	3.8E-07	1.6E-05	CC
GO:0120038	plasma membrane bounded cell projection ...	3.8E-07	1.6E-05	CC
GO:0033267	axon part	4.5E-07	1.86E-05	CC
GO:0030425	dendrite	9.4E-07	3.81E-05	CC
GO:0097447	dendritic tree	0.000001	3.97E-05	CC
GO:0008328	ionotropic glutamate receptor complex	1.1E-06	4.2E-05	CC
GO:0031252	cell leading edge	1.1E-06	4.2E-05	CC
GO:0032281	AMPA glutamate receptor complex	1.2E-06	4.49E-05	CC
GO:0098878	neurotransmitter receptor complex	1.7E-06	6.25E-05	CC
GO:0150034	distal axon	1.8E-06	6.5E-05	CC
GO:0098802	plasma membrane receptor complex	2.7E-06	9.57E-05	CC
GO:0043197	dendritic spine	0.000003	0.000104	CC
GO:0044309	neuron spine	3.7E-06	0.000127	CC
GO:0044297	cell body	4.8E-06	0.000161	CC
GO:0043025	neuronal cell body	0.000005	0.000165	CC
GO:0044306	neuron projection terminus	8.3E-06	0.00027	CC
GO:0043679	axon terminus	0.000015	0.00048	CC
GO:0005576	extracellular region	0.000021	0.000662	CC
GO:0009897	external side of plasma membrane	0.000023	0.000713	CC
GO:0031982	vesicle	0.00007	0.002138	CC
GO:0031410	cytoplasmic vesicle	0.000072	0.002165	CC
GO:0097708	intracellular vesicle	0.000076	0.002252	CC
GO:0098552	side of membrane	0.000082	0.002394	CC
GO:0031594	neuromuscular junction	0.0002	0.005671	CC
GO:0030027	lamellipodium	0.0002	0.005671	CC
GO:0099503	secretory vesicle	0.00026	0.007269	CC
GO:0012505	endomembrane system	0.00034	0.009374	CC
GO:0070382	exocytic vesicle	0.0004	0.010877	CC
GO:0034705	potassium channel complex	0.00045	0.012071	CC
GO:0044304	main axon	0.00046	0.012175	CC
GO:0098858	actin-based cell projection	0.00048	0.012537	CC
GO:0043195	terminal bouton	0.00054	0.013921	CC
GO:0098805	whole membrane	0.00063	0.016033	CC
GO:0008076	voltage-gated potassium channel complex	0.00073	0.018342	CC
GO:0042629	mast cell granule	0.00081	0.020098	CC
GO:0031225	anchored component of membrane	0.00083	0.02034	CC
GO:0042734	presynaptic membrane	0.00094	0.022755	CC
GO:0031233	intrinsic component of external side of ...	0.00106	0.025351	CC

GO:0060076	excitatory synapse	0.00129	0.030484	CC
GO:0005615	extracellular space	0.00139	0.032461	CC
GO:0044421	extracellular region part	0.00143	0.033006	CC
GO:0016323	basolateral plasma membrane	0.00179	0.040841	CC
GO:0030673	axolemma	0.00184	0.041505	CC
GO:0030667	secretory granule membrane	0.00202	0.044773	CC
GO:0098796	membrane protein complex	0.00203	0.044773	CC
GO:0016529	sarcoplasmic reticulum	0.00234	0.051043	CC
GO:0032589	neuron projection membrane	0.00243	0.05243	CC
GO:0008021	synaptic vesicle	0.00246	0.052506	CC
GO:0016459	myosin complex	0.00305	0.064407	CC
GO:0045177	apical part of cell	0.00415	0.085477	CC
GO:0043198	dendritic shaft	0.00419	0.085477	CC
GO:0034704	calcium channel complex	0.00422	0.085477	CC
GO:0031362	anchored component of external side of p...	0.00422	0.085477	CC
GO:0042383	sarcolemma	0.00458	0.091509	CC
GO:0031300	intrinsic component of organelle membran...	0.00461	0.091509	CC
GO:0016528	sarcoplasm	0.00552	0.107618	CC
GO:0030426	growth cone	0.00553	0.107618	CC
GO:0031095	platelet dense tubular network membrane	0.00563	0.107648	CC
GO:0030659	cytoplasmic vesicle membrane	0.00564	0.107648	CC
GO:0012506	vesicle membrane	0.00591	0.111727	CC
GO:0016324	apical plasma membrane	0.00642	0.120224	CC
GO:0044433	cytoplasmic vesicle part	0.00712	0.132086	CC
GO:0030427	site of polarized growth	0.00725	0.133252	CC
GO:0031301	integral component of organelle membrane	0.00776	0.137887	CC
GO:0017146	NMDA selective glutamate receptor comple...	0.00778	0.137887	CC
GO:0031094	platelet dense tubular network	0.00778	0.137887	CC
GO:0044224	juxtaparanode region of axon	0.00778	0.137887	CC
GO:0030133	transport vesicle	0.00789	0.138599	CC
GO:0032982	myosin filament	0.00811	0.141214	CC
GO:0099056	integral component of presynaptic membra...	0.00902	0.155693	CC
GO:0098588	bounding membrane of organelle	0.00923	0.157944	CC
GO:0002116	semaphorin receptor complex	0.01036	0.175766	CC
GO:1990111	spermatoproteasome complex	0.01066	0.179323	CC
GO:0030017	sarcomere	0.01139	0.189993	CC
GO:0031256	leading edge membrane	0.01283	0.21223	CC
GO:0001518	voltage-gated sodium channel complex	0.01337	0.219334	CC
GO:0046658	anchored component of plasma membrane	0.01548	0.251111	CC
GO:0001726	ruffle	0.01556	0.251111	CC
GO:0030141	secretory granule	0.01633	0.260908	CC
GO:0005911	cell-cell junction	0.01643	0.260908	CC
GO:0098889	intrinsic component of presynaptic membr...	0.01704	0.267741	CC
GO:0043292	contractile fiber	0.01713	0.267741	CC
GO:0033268	node of Ranvier	0.02074	0.321632	CC
GO:0005891	voltage-gated calcium channel complex	0.021	0.32314	CC
GO:0098862	cluster of actin-based cell projections	0.02124	0.324318	CC
GO:0044449	contractile fiber part	0.02216	0.335783	CC
GO:0098589	membrane region	0.02239	0.336698	CC
GO:0031674	I band	0.02356	0.351629	CC
GO:0000932	P-body	0.02411	0.357152	CC
GO:0030016	myofibril	0.02502	0.364255	CC

GO:0098985	asymmetric, glutamatergic, excitatory sy...	0.02514	0.364255	CC
GO:0098992	neuronal dense core vesicle	0.02514	0.364255	CC
GO:0048786	presynaptic active zone	0.02577	0.370677	CC
GO:0098982	GABA-ergic synapse	0.02769	0.394888	CC
GO:0031253	cell projection membrane	0.02791	0.394888	CC
GO:0030175	filopodium	0.02805	0.394888	CC
GO:0045121	membrane raft	0.02916	0.407624	CC
GO:0005902	microvillus	0.02971	0.411785	CC
GO:0098688	parallel fiber to Purkinje cell synapse	0.02992	0.411785	CC
GO:0098857	membrane microdomain	0.03008	0.411785	CC
GO:0030018	Z disc	0.03262	0.443498	CC
GO:0010008	endosome membrane	0.03349	0.452229	CC
GO:0008290	F-actin capping protein complex	0.0342	0.455617	CC
GO:0048179	activin receptor complex	0.0342	0.455617	CC
GO:0048471	perinuclear region of cytoplasm	0.03484	0.458127	CC
GO:0015629	actin cytoskeleton	0.03485	0.458127	CC
GO:0031527	filopodium membrane	0.03519	0.459554	CC
GO:0016460	myosin II complex	0.0409	0.510134	CC
GO:0031045	dense core granule	0.0409	0.510134	CC
GO:0032433	filopodium tip	0.0409	0.510134	CC
GO:0005585	collagen type II trimer	0.04343	0.510134	CC
GO:0016028	rhabdomere	0.04343	0.510134	CC
GO:0034679	integrin alpha9-beta1 complex	0.04343	0.510134	CC
GO:0044228	host cell surface	0.04343	0.510134	CC
GO:0098897	spine apparatus membrane	0.04343	0.510134	CC
GO:0098952	intrinsic component of spine apparatus m...	0.04343	0.510134	CC
GO:0099065	integral component of spine apparatus me...	0.04343	0.510134	CC
GO:0120043	stereocilium shaft	0.04343	0.510134	CC
GO:1990031	pinceau fiber	0.04343	0.510134	CC
GO:1990673	intrinsic component of endoplasmic retic...	0.04343	0.510134	CC
GO:1990701	integral component of endoplasmic reticu...	0.04343	0.510134	CC
GO:0005794	Golgi apparatus	0.04357	0.510134	CC
GO:0033179	proton-transporting V-type ATPase, V0 do...	0.0443	0.510134	CC
GO:0044352	pinosome	0.0443	0.510134	CC
GO:0044354	macropinosome	0.0443	0.510134	CC
GO:0098843	postsynaptic endocytic zone	0.0443	0.510134	CC
GO:0032420	stereocilium	0.04446	0.510134	CC
GO:0045178	basal part of cell	0.04446	0.510134	CC
GO:0034706	sodium channel complex	0.04705	0.536749	CC
GO:0043296	apical junction complex	0.04765	0.540487	CC
GO:0009925	basal plasma membrane	0.04838	0.545649	CC
GO:0031091	platelet alpha granule	0.04945	0.554566	CC

Supplementary Table 8_Gene Ontology Results of DEGs across brain regions in women

AntIns

GO.ID	Term	FET	BH	Category
GO:0016071	mRNA metabolic process	0.000	0.002	BP
GO:0051641	cellular localization	0.000	0.003	BP
GO:0008380	RNA splicing	0.000	0.031	BP
GO:0000375	RNA splicing, via transesterification re...	0.000	0.069	BP
GO:0051649	establishment of localization in cell	0.000	0.069	BP
GO:0000377	RNA splicing, via transesterification re...	0.000	0.069	BP
GO:0000398	mRNA splicing, via spliceosome	0.000	0.069	BP
GO:0000387	spliceosomal snRNP assembly	0.000	0.072	BP
GO:0022406	membrane docking	0.000	0.092	BP
GO:0070727	cellular macromolecule localization	0.000	0.099	BP
GO:0140056	organelle localization by membrane tethe...	0.000	0.169	BP
GO:0046907	intracellular transport	0.000	0.169	BP
GO:0000956	nuclear-transcribed mRNA catabolic proce...	0.000	0.169	BP
GO:0006397	mRNA processing	0.000	**0.177**	BP
GO:0034613	cellular protein localization	0.000	0.177	BP
GO:0006412	translation	0.000	0.235	BP
GO:0043043	peptide biosynthetic process	0.000	0.235	BP
GO:0051169	nuclear transport	0.000	0.235	BP
GO:0000245	spliceosomal complex assembly	0.000	0.255	BP
GO:0022618	ribonucleoprotein complex assembly	0.000	0.313	BP
GO:0006913	nucleocytoplasmic transport	0.000	0.313	BP
GO:0006402	mRNA catabolic process	0.001	0.377	BP
GO:0071826	ribonucleoprotein complex subunit organi...	0.001	0.378	BP
GO:0006401	RNA catabolic process	0.001	0.378	BP
GO:0097065	anterior head development	0.001	0.388	BP
GO:0043604	amide biosynthetic process	0.001	0.418	BP
GO:0097711	ciliary basal body-plasma membrane docki...	0.001	0.418	BP
GO:0010605	negative regulation of macromolecule met...	0.001	0.418	BP
GO:0019076	viral release from host cell	0.001	0.418	BP
GO:0035890	exit from host	0.001	0.418	BP
GO:0035891	exit from host cell	0.001	0.418	BP
GO:0052126	movement in host environment	0.001	0.418	BP
GO:0052192	movement in environment of other organis...	0.001	0.418	BP
GO:0060271	cilium assembly	0.001	0.461	BP
GO:0008104	protein localization	0.001	0.461	BP
GO:0006353	DNA-templated transcription, termination	0.001	0.461	BP
GO:0043928	exonucleolytic nuclear-transcribed mRNA ...	0.001	0.461	BP
GO:0006886	intracellular protein transport	0.001	0.461	BP
GO:0060174	limb bud formation	0.001	0.474	BP
GO:0010629	negative regulation of gene expression	0.001	0.552	BP
GO:0008089	anterograde axonal transport	0.002	0.603	BP
GO:0010467	gene expression	0.002	0.641	BP
GO:0000291	nuclear-transcribed mRNA catabolic proce...	0.002	0.641	BP
GO:0060023	soft palate development	0.002	0.761	BP
GO:1990089	response to nerve growth factor	0.002	0.779	BP
GO:0022613	ribonucleoprotein complex biogenesis	0.002	0.811	BP
GO:0007217	tachykinin receptor signaling pathway	0.003	0.811	BP
GO:0070459	prolactin secretion	0.003	0.811	BP
GO:0044782	cilium organization	0.003	0.811	BP

GO:0009892	negative regulation of metabolic process	0.003	0.811	BP
GO:0033365	protein localization to organelle	0.003	0.823	BP
GO:0051640	organelle localization	0.003	0.877	BP
GO:0051170	import into nucleus	0.003	0.877	BP
GO:0045184	establishment of protein localization	0.003	0.877	BP
GO:0008334	histone mRNA metabolic process	0.003	0.877	BP
GO:0048524	positive regulation of viral process	0.003	0.877	BP
GO:2000134	negative regulation of G1/S transition o...	0.003	0.877	BP
GO:0048490	anterograde synaptic vesicle transport	0.003	0.877	BP
GO:0099514	synaptic vesicle cytoskeletal transport	0.003	0.877	BP
GO:0099517	synaptic vesicle transport along microtu...	0.003	0.877	BP
GO:0051168	nuclear export	0.004	0.877	BP
GO:0033036	macromolecule localization	0.004	0.877	BP
GO:0070925	organelle assembly	0.004	0.877	BP
GO:0060341	regulation of cellular localization	0.004	0.908	BP
GO:0015031	protein transport	0.004	0.908	BP
GO:0043517	positive regulation of DNA damage respon...	0.004	0.945	BP
GO:0006614	SRP-dependent cotranslational protein ta...	0.004	0.945	BP
GO:1902807	negative regulation of cell cycle G1/S p...	0.004	0.945	BP
GO:0006383	transcription by RNA polymerase III	0.004	0.945	BP
GO:0090304	nucleic acid metabolic process	0.004	0.945	BP
GO:0006457	protein folding	0.004	0.945	BP
GO:0044271	cellular nitrogen compound biosynthetic ...	0.004	0.945	BP
GO:0044085	cellular component biogenesis	0.005	0.945	BP
GO:1905515	non-motile cilium assembly	0.005	0.945	BP
GO:0007204	positive regulation of cytosolic calcium...	0.005	0.945	BP
GO:0000965	mitochondrial RNA 3'-end processing	0.005	0.945	BP
GO:0021631	optic nerve morphogenesis	0.005	0.945	BP
GO:0035790	platelet-derived growth factor receptor-...	0.005	0.945	BP
GO:0061484	hematopoietic stem cell homeostasis	0.005	0.945	BP
GO:0110011	regulation of basement membrane organiza...	0.005	0.945	BP
GO:1904259	regulation of basement membrane assembly...	0.005	0.945	BP
GO:1904261	positive regulation of basement membrane...	0.005	0.945	BP
GO:2001197	basement membrane assembly involved in e...	0.005	0.945	BP
GO:0048278	vesicle docking	0.005	0.949	BP
GO:0007018	microtubule-based movement	0.005	0.952	BP
GO:0022607	cellular component assembly	0.005	0.962	BP
GO:0006403	RNA localization	0.005	0.962	BP
GO:0019080	viral gene expression	0.006	0.962	BP
GO:0015833	peptide transport	0.006	0.962	BP
GO:0044794	positive regulation by host of viral pro...	0.006	0.962	BP
GO:0044849	estrous cycle	0.006	0.962	BP
GO:0050884	neuromuscular process controlling postur...	0.006	0.962	BP
GO:1901798	positive regulation of signal transducti...	0.006	0.962	BP
GO:0006613	cotranslational protein targeting to mem...	0.006	0.962	BP
GO:1903829	positive regulation of cellular protein ...	0.006	0.962	BP
GO:0045047	protein targeting to ER	0.006	0.962	BP
GO:0006725	cellular aromatic compound metabolic pro...	0.006	0.962	BP
GO:0048592	eye morphogenesis	0.007	0.962	BP
GO:0033327	Leydig cell differentiation	0.007	0.962	BP
GO:0034622	cellular protein-containing complex asse...	0.007	0.962	BP
GO:0016070	RNA metabolic process	0.007	0.962	BP

GO:1902186	regulation of viral release from host ce...	0.007	0.962	BP
GO:0003130	BMP signaling pathway involved in heart ...	0.007	0.962	BP
GO:0003133	endodermal-mesodermal cell signaling	0.007	0.962	BP
GO:0003134	endodermal-mesodermal cell signaling inv...	0.007	0.962	BP
GO:0006212	uracil catabolic process	0.007	0.962	BP
GO:0006393	termination of mitochondrial transcripti...	0.007	0.962	BP
GO:0009405	pathogenesis	0.007	0.962	BP
GO:0140042	lipid droplet formation	0.007	0.962	BP
GO:1902162	regulation of DNA damage response, signa...	0.007	0.962	BP
GO:1902164	positive regulation of DNA damage respon...	0.007	0.962	BP
GO:2000768	positive regulation of nephron tubule ep...	0.007	0.962	BP
GO:0006518	peptide metabolic process	0.007	0.991	BP
GO:0070972	protein localization to endoplasmic reti...	0.007	0.994	BP
GO:0098930	axonal transport	0.007	1.000	BP
GO:0140053	mitochondrial gene expression	0.008	1.000	BP
GO:0000959	mitochondrial RNA metabolic process	0.008	1.000	BP
GO:0006978	DNA damage response, signal transduction...	0.008	1.000	BP
GO:1902165	regulation of intrinsic apoptotic signal...	0.008	1.000	BP
GO:0006611	protein export from nucleus	0.009	1.000	BP
GO:0072599	establishment of protein localization to...	0.009	1.000	BP
GO:0003129	heart induction	0.009	1.000	BP
GO:0009249	protein lipoylation	0.009	1.000	BP
GO:0070141	response to UV-A	0.009	1.000	BP
GO:1902093	positive regulation of flagellated sperm...	0.009	1.000	BP
GO:0055074	calcium ion homeostasis	0.010	1.000	BP
GO:0008156	negative regulation of DNA replication	0.010	1.000	BP
GO:1901998	toxin transport	0.010	1.000	BP
GO:0010172	embryonic body morphogenesis	0.010	1.000	BP
GO:0010642	negative regulation of platelet-derived ...	0.010	1.000	BP
GO:0033033	negative regulation of myeloid cell apop...	0.010	1.000	BP
GO:0042886	amide transport	0.010	1.000	BP
GO:0019222	regulation of metabolic process	0.010	1.000	BP
GO:0000184	nuclear-transcribed mRNA catabolic proce...	0.010	1.000	BP
GO:0006139	nucleobase-containing compound metabolic...	0.010	1.000	BP
GO:0006874	cellular calcium ion homeostasis	0.010	1.000	BP
GO:0042772	DNA damage response, signal transduction...	0.010	1.000	BP
GO:0051151	negative regulation of smooth muscle cel...	0.010	1.000	BP
GO:0003179	heart valve morphogenesis	0.010	1.000	BP
GO:0046483	heterocycle metabolic process	0.011	1.000	BP
GO:0006413	translational initiation	0.011	1.000	BP
GO:0006275	regulation of DNA replication	0.012	1.000	BP
GO:0000244	spliceosomal tri-snRNP complex assembly	0.012	1.000	BP
GO:0010882	regulation of cardiac muscle contraction...	0.012	1.000	BP
GO:0033119	negative regulation of RNA splicing	0.012	1.000	BP
GO:0016050	vesicle organization	0.012	1.000	BP
GO:0047496	vesicle transport along microtubule	0.012	1.000	BP
GO:0072507	divalent inorganic cation homeostasis	0.012	1.000	BP
GO:2000113	negative regulation of cellular macromol...	0.012	1.000	BP
GO:0051480	regulation of cytosolic calcium ion conc...	0.012	1.000	BP
GO:0034641	cellular nitrogen compound metabolic pro...	0.013	1.000	BP
GO:0099537	trans-synaptic signaling	0.013	1.000	BP
GO:1903827	regulation of cellular protein localizat...	0.013	1.000	BP

GO:0003215	cardiac right ventricle morphogenesis	0.014	1.000	BP
GO:1904816	positive regulation of protein localizat...	0.014	1.000	BP
GO:0010468	regulation of gene expression	0.014	1.000	BP
GO:0010921	regulation of phosphatase activity	0.014	1.000	BP
GO:0007281	germ cell development	0.014	1.000	BP
GO:1990090	cellular response to nerve growth factor...	0.014	1.000	BP
GO:1903902	positive regulation of viral life cycle	0.014	1.000	BP
GO:1901360	organic cyclic compound metabolic proces...	0.014	1.000	BP
GO:0043516	regulation of DNA damage response, signa...	0.014	1.000	BP
GO:0019083	viral transcription	0.014	1.000	BP
GO:0001835	blastocyst hatching	0.014	1.000	BP
GO:0034453	microtubule anchoring	0.014	1.000	BP
GO:0035188	hatching	0.014	1.000	BP
GO:0071684	organism emergence from protective struc...	0.014	1.000	BP
GO:2000104	negative regulation of DNA-dependent DNA...	0.014	1.000	BP
GO:0046822	regulation of nucleocytoplasmic transpor...	0.015	1.000	BP
GO:0032984	protein-containing complex disassembly	0.015	1.000	BP
GO:0010948	negative regulation of cell cycle proces...	0.015	1.000	BP
GO:0099536	synaptic signaling	0.015	1.000	BP
GO:0030913	paranodal junction assembly	0.015	1.000	BP
GO:0033085	negative regulation of T cell differenti...	0.015	1.000	BP
GO:0035376	sterol import	0.015	1.000	BP
GO:0070508	cholesterol import	0.015	1.000	BP
GO:1900227	positive regulation of NLRP3 inflammasom...	0.015	1.000	BP
GO:0051656	establishment of organelle localization	0.016	1.000	BP
GO:0032206	positive regulation of telomere maintena...	0.016	1.000	BP
GO:0044419	interspecies interaction between organis...	0.016	1.000	BP
GO:0000288	nuclear-transcribed mRNA catabolic proce...	0.016	1.000	BP
GO:0006283	transcription-coupled nucleotide-excisio...	0.016	1.000	BP
GO:0060255	regulation of macromolecule metabolic pr...	0.016	1.000	BP
GO:0007507	heart development	0.017	1.000	BP
GO:0016082	synaptic vesicle priming	0.017	1.000	BP
GO:0034389	lipid droplet organization	0.017	1.000	BP
GO:1901673	regulation of mitotic spindle assembly	0.017	1.000	BP
GO:0090169	regulation of spindle assembly	0.018	1.000	BP
GO:0033157	regulation of intracellular protein tran...	0.018	1.000	BP
GO:1901991	negative regulation of mitotic cell cycl...	0.018	1.000	BP
GO:2000045	regulation of G1/S transition of mitotic...	0.018	1.000	BP
GO:0008298	intracellular mRNA localization	0.018	1.000	BP
GO:0048557	embryonic digestive tract morphogenesis	0.018	1.000	BP
GO:0044403	symbiont process	0.019	1.000	BP
GO:0010389	regulation of G2/M transition of mitotic...	0.019	1.000	BP
GO:0051817	modification of morphology or physiology...	0.019	1.000	BP
GO:0030330	DNA damage response, signal transduction...	0.019	1.000	BP
GO:0044774	mitotic DNA integrity checkpoint	0.019	1.000	BP
GO:0034645	cellular macromolecule biosynthetic proc...	0.019	1.000	BP
GO:0007268	chemical synaptic transmission	0.019	1.000	BP
GO:0098916	anterograde trans-synaptic signaling	0.019	1.000	BP
GO:0002188	translation reinitiation	0.020	1.000	BP
GO:0006208	pyrimidine nucleobase catabolic process	0.020	1.000	BP
GO:0006210	thymine catabolic process	0.020	1.000	BP
GO:0007621	negative regulation of female receptivit...	0.020	1.000	BP

GO:0010609	mRNA localization resulting in posttrans...	0.020	1.000	BP
GO:0014016	neuroblast differentiation	0.020	1.000	BP
GO:0014859	negative regulation of skeletal muscle c...	0.020	1.000	BP
GO:0016476	regulation of embryonic cell shape	0.020	1.000	BP
GO:0019859	thymine metabolic process	0.020	1.000	BP
GO:0019860	uracil metabolic process	0.020	1.000	BP
GO:0021965	spinal cord ventral commissure morphogen...	0.020	1.000	BP
GO:0031117	positive regulation of microtubule depol...	0.020	1.000	BP
GO:0032224	positive regulation of synaptic transmis...	0.020	1.000	BP
GO:0035359	negative regulation of peroxisome prolif...	0.020	1.000	BP
GO:0042078	germ-line stem cell division	0.020	1.000	BP
GO:0044502	positive regulation of signal transducti...	0.020	1.000	BP
GO:0048133	male germ-line stem cell asymmetric divi...	0.020	1.000	BP
GO:0051970	negative regulation of transmission of n...	0.020	1.000	BP
GO:0052470	modulation by host of symbiont signal tr...	0.020	1.000	BP
GO:0052525	positive regulation by host of symbiont ...	0.020	1.000	BP
GO:0052526	positive regulation by organism of signa...	0.020	1.000	BP
GO:0060221	retinal rod cell differentiation	0.020	1.000	BP
GO:0061209	cell proliferation involved in mesonephr...	0.020	1.000	BP
GO:0070859	positive regulation of bile acid biosynt...	0.020	1.000	BP
GO:0072138	mesenchymal cell proliferation involved ...	0.020	1.000	BP
GO:0072139	glomerular parietal epithelial cell diff...	0.020	1.000	BP
GO:0072190	ureter urothelium development	0.020	1.000	BP
GO:0075205	modulation by host of symbiont cAMP-medi...	0.020	1.000	BP
GO:0075206	positive regulation by host of symbiont ...	0.020	1.000	BP
GO:0090204	protein localization to nuclear pore	0.020	1.000	BP
GO:0098728	germline stem cell asymmetric division	0.020	1.000	BP
GO:1902722	positive regulation of prolactin secreti...	0.020	1.000	BP
GO:1902723	negative regulation of skeletal muscle s...	0.020	1.000	BP
GO:1904253	positive regulation of bile acid metabol...	0.020	1.000	BP
GO:1904761	negative regulation of myofibroblast dif...	0.020	1.000	BP
GO:1905292	regulation of neural crest cell differen...	0.020	1.000	BP
GO:1905294	positive regulation of neural crest cell...	0.020	1.000	BP
GO:1905310	regulation of cardiac neural crest cell ...	0.020	1.000	BP
GO:1905312	positive regulation of cardiac neural cr...	0.020	1.000	BP
GO:1990180	mitochondrial tRNA 3'-end processing	0.020	1.000	BP
GO:0051050	positive regulation of transport	0.020	1.000	BP
GO:0010970	transport along microtubule	0.020	1.000	BP
GO:0099111	microtubule-based transport	0.020	1.000	BP
GO:0000086	G2/M transition of mitotic cell cycle	0.020	1.000	BP
GO:0007019	microtubule depolymerization	0.020	1.000	BP
GO:0072594	establishment of protein localization to...	0.021	1.000	BP
GO:0006622	protein targeting to lysosome	0.021	1.000	BP
GO:0010881	regulation of cardiac muscle contraction...	0.021	1.000	BP
GO:1903055	positive regulation of extracellular mat...	0.021	1.000	BP
GO:0051279	regulation of release of sequestered cal...	0.021	1.000	BP
GO:0032386	regulation of intracellular transport	0.021	1.000	BP
GO:0006414	translational elongation	0.022	1.000	BP
GO:1901990	regulation of mitotic cell cycle phase t...	0.022	1.000	BP
GO:0140029	exocytic process	0.022	1.000	BP
GO:1904427	positive regulation of calcium ion trans...	0.022	1.000	BP
GO:0051236	establishment of RNA localization	0.023	1.000	BP

GO:0006369	termination of RNA polymerase II transcr...	0.023	1.000	BP
GO:0043243	positive regulation of protein complex d...	0.023	1.000	BP
GO:0099518	vesicle cytoskeletal trafficking	0.023	1.000	BP
GO:0043933	protein-containing complex subunit organ...	0.023	1.000	BP
GO:0010835	regulation of protein ADP-ribosylation	0.023	1.000	BP
GO:0031392	regulation of prostaglandin biosynthetic...	0.023	1.000	BP
GO:0033601	positive regulation of mammary gland epi...	0.023	1.000	BP
GO:0036289	peptidyl-serine autophosphorylation	0.023	1.000	BP
GO:0042780	tRNA 3'-end processing	0.023	1.000	BP
GO:0043497	regulation of protein heterodimerization...	0.023	1.000	BP
GO:0048227	plasma membrane to endosome transport	0.023	1.000	BP
GO:0051342	regulation of cyclic-nucleotide phosphod...	0.023	1.000	BP
GO:0051639	actin filament network formation	0.023	1.000	BP
GO:0060316	positive regulation of ryanodine-sensiti...	0.023	1.000	BP
GO:0060896	neural plate pattern specification	0.023	1.000	BP
GO:0072201	negative regulation of mesenchymal cell ...	0.023	1.000	BP
GO:0097011	cellular response to granulocyte macroph...	0.023	1.000	BP
GO:0097012	response to granulocyte macrophage colon...	0.023	1.000	BP
GO:1904354	negative regulation of telomere capping	0.023	1.000	BP
GO:0006904	vesicle docking involved in exocytosis	0.023	1.000	BP
GO:0038202	TORC1 signaling	0.023	1.000	BP
GO:0008088	axo-dendritic transport	0.023	1.000	BP
GO:0036158	outer dynein arm assembly	0.024	1.000	BP
GO:0042559	pteridine-containing compound biosynthet...	0.024	1.000	BP
GO:1902166	negative regulation of intrinsic apoptot...	0.024	1.000	BP
GO:1902187	negative regulation of viral release fro...	0.024	1.000	BP
GO:1904263	positive regulation of TORC1 signaling	0.024	1.000	BP
GO:1904814	regulation of protein localization to ch...	0.024	1.000	BP
GO:0032204	regulation of telomere maintenance	0.024	1.000	BP
GO:0051851	modification by host of symbiont morphol...	0.025	1.000	BP
GO:0010880	regulation of release of sequestered cal...	0.025	1.000	BP
GO:0033363	secretory granule organization	0.025	1.000	BP
GO:0044818	mitotic G2/M transition checkpoint	0.025	1.000	BP
GO:0061036	positive regulation of cartilage develop...	0.025	1.000	BP
GO:0010640	regulation of platelet-derived growth fa...	0.026	1.000	BP
GO:0016556	mRNA modification	0.026	1.000	BP
GO:0033081	regulation of T cell differentiation in ...	0.026	1.000	BP
GO:0032210	regulation of telomere maintenance via t...	0.026	1.000	BP
GO:0008584	male gonad development	0.026	1.000	BP
GO:0007223	Wnt signaling pathway, calcium modulatin...	0.026	1.000	BP
GO:0046825	regulation of protein export from nucleu...	0.026	1.000	BP
GO:0006366	transcription by RNA polymerase II	0.026	1.000	BP
GO:0031503	protein-containing complex localization	0.027	1.000	BP
GO:0043624	cellular protein complex disassembly	0.027	1.000	BP
GO:0030317	flagellated sperm motility	0.028	1.000	BP
GO:0035418	protein localization to synapse	0.028	1.000	BP
GO:0097722	sperm motility	0.028	1.000	BP
GO:0046546	development of primary male sexual chara...	0.028	1.000	BP
GO:0090307	mitotic spindle assembly	0.029	1.000	BP
GO:1903053	regulation of extracellular matrix organ...	0.030	1.000	BP
GO:1903432	regulation of TORC1 signaling	0.030	1.000	BP
GO:0043603	cellular amide metabolic process	0.030	1.000	BP

GO:0043244	regulation of protein complex disassembl...	0.031	1.000	BP
GO:0006390	mitochondrial transcription	0.031	1.000	BP
GO:0015936	coenzyme A metabolic process	0.031	1.000	BP
GO:0033599	regulation of mammary gland epithelial c...	0.031	1.000	BP
GO:0042749	regulation of circadian sleep/wake cycle	0.031	1.000	BP
GO:2000353	positive regulation of endothelial cell ...	0.031	1.000	BP
GO:2000738	positive regulation of stem cell differe...	0.031	1.000	BP
GO:0010558	negative regulation of macromolecule bio...	0.031	1.000	BP
GO:0070507	regulation of microtubule cytoskeleton o...	0.031	1.000	BP
GO:0002181	cytoplasmic translation	0.031	1.000	BP
GO:0072080	nephron tubule development	0.031	1.000	BP
GO:0035303	regulation of dephosphorylation	0.031	1.000	BP
GO:0090150	establishment of protein localization to...	0.032	1.000	BP
GO:0003170	heart valve development	0.032	1.000	BP
GO:0090329	regulation of DNA-dependent DNA replicat...	0.032	1.000	BP
GO:0031647	regulation of protein stability	0.032	1.000	BP
GO:0006617	SRP-dependent cotranslational protein ta...	0.033	1.000	BP
GO:0014816	skeletal muscle satellite cell different...	0.033	1.000	BP
GO:0048671	negative regulation of collateral sprout...	0.033	1.000	BP
GO:0072182	regulation of nephron tubule epithelial ...	0.033	1.000	BP
GO:0099641	anterograde axonal protein transport	0.033	1.000	BP
GO:1901317	regulation of flagellated sperm motility	0.033	1.000	BP
GO:1904869	regulation of protein localization to Ca...	0.033	1.000	BP
GO:1904871	positive regulation of protein localizat...	0.033	1.000	BP
GO:1905064	negative regulation of vascular smooth m...	0.033	1.000	BP
GO:0072503	cellular divalent inorganic cation homeo...	0.033	1.000	BP
GO:0050657	nucleic acid transport	0.033	1.000	BP
GO:0050658	RNA transport	0.033	1.000	BP
GO:0051172	negative regulation of nitrogen compound...	0.033	1.000	BP
GO:0032886	regulation of microtubule-based process	0.033	1.000	BP
GO:0017148	negative regulation of translation	0.033	1.000	BP
GO:0034605	cellular response to heat	0.033	1.000	BP
GO:2001257	regulation of cation channel activity	0.034	1.000	BP
GO:0045843	negative regulation of striated muscle t...	0.034	1.000	BP
GO:0010922	positive regulation of phosphatase activ...	0.034	1.000	BP
GO:0051281	positive regulation of release of seques...	0.034	1.000	BP
GO:0045930	negative regulation of mitotic cell cycl...	0.034	1.000	BP
GO:0030705	cytoskeleton-dependent intracellular tra...	0.035	1.000	BP
GO:1901021	positive regulation of calcium ion trans...	0.035	1.000	BP
GO:1901988	negative regulation of cell cycle phase ...	0.035	1.000	BP
GO:0051650	establishment of vesicle localization	0.035	1.000	BP
GO:0051235	maintenance of location	0.036	1.000	BP
GO:0016032	viral process	0.037	1.000	BP
GO:0000960	regulation of mitochondrial RNA cataboli...	0.037	1.000	BP
GO:0000964	mitochondrial RNA 5'-end processing	0.037	1.000	BP
GO:0003253	cardiac neural crest cell migration invo...	0.037	1.000	BP
GO:0003357	noradrenergic neuron differentiation	0.037	1.000	BP
GO:0018076	N-terminal peptidyl-lysine acetylation	0.037	1.000	BP
GO:0019482	beta-alanine metabolic process	0.037	1.000	BP
GO:0022615	protein to membrane docking	0.037	1.000	BP
GO:0032345	negative regulation of aldosterone metab...	0.037	1.000	BP
GO:0032348	negative regulation of aldosterone biosy...	0.037	1.000	BP

GO:0032847	regulation of cellular pH reduction	0.037	1.000	BP
GO:0036446	myofibroblast differentiation	0.037	1.000	BP
GO:0051138	positive regulation of NK T cell differe...	0.037	1.000	BP
GO:0051343	positive regulation of cyclic-nucleotide...	0.037	1.000	BP
GO:0060267	positive regulation of respiratory burst	0.037	1.000	BP
GO:0061428	negative regulation of transcription fro...	0.037	1.000	BP
GO:0071603	endothelial cell-cell adhesion	0.037	1.000	BP
GO:0072103	glomerulus vasculature morphogenesis	0.037	1.000	BP
GO:0072104	glomerular capillary formation	0.037	1.000	BP
GO:0072191	ureter smooth muscle development	0.037	1.000	BP
GO:0072193	ureter smooth muscle cell differentiatio...	0.037	1.000	BP
GO:0099041	vesicle tethering to Golgi	0.037	1.000	BP
GO:1902576	negative regulation of nuclear cell cycl...	0.037	1.000	BP
GO:1902725	negative regulation of satellite cell di...	0.037	1.000	BP
GO:1903690	negative regulation of wound healing, sp...	0.037	1.000	BP
GO:1904760	regulation of myofibroblast differentiat...	0.037	1.000	BP
GO:2000020	positive regulation of male gonad develo...	0.037	1.000	BP
GO:2000065	negative regulation of cortisol biosynth...	0.037	1.000	BP
GO:2000583	regulation of platelet-derived growth fa...	0.037	1.000	BP
GO:2000587	negative regulation of platelet-derived ...	0.037	1.000	BP
GO:2000698	positive regulation of epithelial cell d...	0.037	1.000	BP
GO:0003180	aortic valve morphogenesis	0.037	1.000	BP
GO:0035235	ionotropic glutamate receptor signaling ...	0.037	1.000	BP
GO:0009059	macromolecule biosynthetic process	0.037	1.000	BP
GO:0072384	organelle transport along microtubule	0.037	1.000	BP
GO:0061326	renal tubule development	0.037	1.000	BP
GO:0031324	negative regulation of cellular metaboli...	0.038	1.000	BP
GO:0048635	negative regulation of muscle organ deve...	0.038	1.000	BP
GO:0022898	regulation of transmembrane transporter ...	0.038	1.000	BP
GO:0051208	sequestering of calcium ion	0.038	1.000	BP
GO:0007158	neuron cell-cell adhesion	0.038	1.000	BP
GO:0021602	cranial nerve morphogenesis	0.038	1.000	BP
GO:0050792	regulation of viral process	0.038	1.000	BP
GO:0007346	regulation of mitotic cell cycle	0.039	1.000	BP
GO:0003008	system process	0.039	1.000	BP
GO:0046887	positive regulation of hormone secretion	0.039	1.000	BP
GO:0060249	anatomical structure homeostasis	0.039	1.000	BP
GO:0014808	release of sequestered calcium ion into ...	0.039	1.000	BP
GO:0051930	regulation of sensory perception of pain	0.039	1.000	BP
GO:0097242	amyloid-beta clearance	0.039	1.000	BP
GO:1903514	release of sequestered calcium ion into ...	0.039	1.000	BP
GO:0032409	regulation of transporter activity	0.039	1.000	BP
GO:0071705	nitrogen compound transport	0.039	1.000	BP
GO:0007156	homophilic cell adhesion via plasma memb...	0.040	1.000	BP
GO:0018394	peptidyl-lysine acetylation	0.040	1.000	BP
GO:1902806	regulation of cell cycle G1/S phase tran...	0.040	1.000	BP
GO:0007017	microtubule-based process	0.040	1.000	BP
GO:0034764	positive regulation of transmembrane tra...	0.040	1.000	BP
GO:0043903	regulation of symbiosis, encompassing mu...	0.041	1.000	BP
GO:0051702	interaction with symbiont	0.041	1.000	BP
GO:1903779	regulation of cardiac conduction	0.041	1.000	BP
GO:0050877	nervous system process	0.041	1.000	BP

GO:1990778	protein localization to cell periphery	0.041	1.000	BP
GO:0019439	aromatic compound catabolic process	0.041	1.000	BP
GO:1901987	regulation of cell cycle phase transitio...	0.041	1.000	BP
GO:0019438	aromatic compound biosynthetic process	0.041	1.000	BP
GO:0051049	regulation of transport	0.042	1.000	BP
GO:0045934	negative regulation of nucleobase-contai...	0.043	1.000	BP
GO:0010522	regulation of calcium ion transport into...	0.044	1.000	BP
GO:0000338	protein deneddylation	0.044	1.000	BP
GO:0009157	deoxyribonucleoside monophosphate biosyn...	0.044	1.000	BP
GO:0010960	magnesium ion homeostasis	0.044	1.000	BP
GO:0015937	coenzyme A biosynthetic process	0.044	1.000	BP
GO:0018065	protein-cofactor linkage	0.044	1.000	BP
GO:0030238	male sex determination	0.044	1.000	BP
GO:0033314	mitotic DNA replication checkpoint	0.044	1.000	BP
GO:0035646	endosome to melanosome transport	0.044	1.000	BP
GO:0035999	tetrahydrofolate interconversion	0.044	1.000	BP
GO:0043485	endosome to pigment granule transport	0.044	1.000	BP
GO:0048757	pigment granule maturation	0.044	1.000	BP
GO:0075522	IRES-dependent viral translational initi...	0.044	1.000	BP
GO:1903405	protein localization to nuclear body	0.044	1.000	BP
GO:1904851	positive regulation of establishment of ...	0.044	1.000	BP
GO:1904867	protein localization to Cajal body	0.044	1.000	BP
GO:2001279	regulation of unsaturated fatty acid bio...	0.044	1.000	BP
GO:0019433	triglyceride catabolic process	0.044	1.000	BP
GO:0035735	intraciliary transport involved in ciliu...	0.044	1.000	BP
GO:0048546	digestive tract morphogenesis	0.044	1.000	BP
GO:0090224	regulation of spindle organization	0.044	1.000	BP
GO:0002244	hematopoietic progenitor cell differenti...	0.044	1.000	BP
GO:0051648	vesicle localization	0.044	1.000	BP
GO:0050810	regulation of steroid biosynthetic proce...	0.044	1.000	BP
GO:0007004	telomere maintenance via telomerase	0.045	1.000	BP
GO:0033866	nucleoside bisphosphate biosynthetic pro...	0.045	1.000	BP
GO:0034030	ribonucleoside bisphosphate biosynthetic...	0.045	1.000	BP
GO:0034033	purine nucleoside bisphosphate biosynthe...	0.045	1.000	BP
GO:0018130	heterocycle biosynthetic process	0.045	1.000	BP
GO:1902749	regulation of cell cycle G2/M phase tran...	0.045	1.000	BP
GO:0007052	mitotic spindle organization	0.045	1.000	BP
GO:0051209	release of sequestered calcium ion into ...	0.045	1.000	BP
GO:0042558	pteridine-containing compound metabolic ...	0.045	1.000	BP
GO:0045070	positive regulation of viral genome repl...	0.045	1.000	BP
GO:0051931	regulation of sensory perception	0.045	1.000	BP
GO:0034654	nucleobase-containing compound biosynthe...	0.045	1.000	BP
GO:0009987	cellular process	0.046	1.000	BP
GO:0000963	mitochondrial RNA processing	0.047	1.000	BP
GO:0002320	lymphoid progenitor cell differentiation	0.047	1.000	BP
GO:0072234	metanephric nephron tubule development	0.047	1.000	BP
GO:0044839	cell cycle G2/M phase transition	0.047	1.000	BP
GO:0033059	cellular pigmentation	0.047	1.000	BP
GO:1901016	regulation of potassium ion transmembran...	0.047	1.000	BP
GO:1901862	negative regulation of muscle tissue dev...	0.047	1.000	BP
GO:2000112	regulation of cellular macromolecule bio...	0.047	1.000	BP
GO:0050821	protein stabilization	0.047	1.000	BP

GO:0035637	multicellular organismal signaling	0.047	1.000	BP
GO:1901362	organic cyclic compound biosynthetic pro...	0.047	1.000	BP
GO:0001889	liver development	0.048	1.000	BP
GO:0098781	ncRNA transcription	0.048	1.000	BP
GO:0048477	oogenesis	0.048	1.000	BP
GO:1904356	regulation of telomere maintenance via t...	0.048	1.000	BP
GO:1904377	positive regulation of protein localizat...	0.048	1.000	BP
GO:0006261	DNA-dependent DNA replication	0.050	1.000	BP
GO:0010923	negative regulation of phosphatase activ...	0.050	1.000	BP
GO:0048593	camera-type eye morphogenesis	0.050	1.000	BP
GO:2001022	positive regulation of response to DNA d...	0.050	1.000	BP
GO:0003723	RNA binding	0.000	0.001	MF
GO:0003676	nucleic acid binding	0.000	0.001	MF
GO:1901363	heterocyclic compound binding	0.000	0.052	MF
GO:0097159	organic cyclic compound binding	0.000	0.073	MF
GO:0005488	binding	0.001	0.555	MF
GO:0051082	unfolded protein binding	0.001	0.555	MF
GO:0003729	mRNA binding	0.001	0.758	MF
GO:0008312	7S RNA binding	0.001	0.787	MF
GO:0008494	translation activator activity	0.003	1.000	MF
GO:0005515	protein binding	0.004	1.000	MF
GO:0001222	transcription corepressor binding	0.004	1.000	MF
GO:0098847	sequence-specific single stranded DNA bi...	0.004	1.000	MF
GO:0002162	dystroglycan binding	0.004	1.000	MF
GO:0019894	kinesin binding	0.005	1.000	MF
GO:0003677	DNA binding	0.005	1.000	MF
GO:0099181	structural constituent of presynapse	0.005	1.000	MF
GO:0003735	structural constituent of ribosome	0.005	1.000	MF
GO:0039706	co-receptor binding	0.007	1.000	MF
GO:0002058	uracil binding	0.007	1.000	MF
GO:0002061	pyrimidine nucleobase binding	0.007	1.000	MF
GO:0005006	epidermal growth factor-activated recept...	0.007	1.000	MF
GO:0005009	insulin-activated receptor activity	0.007	1.000	MF
GO:0016415	octanoyltransferase activity	0.007	1.000	MF
GO:0031834	neurokinin receptor binding	0.007	1.000	MF
GO:0031835	substance P receptor binding	0.007	1.000	MF
GO:0140104	molecular carrier activity	0.010	1.000	MF
GO:0005520	insulin-like growth factor binding	0.010	1.000	MF
GO:0005049	nuclear export signal receptor activity	0.010	1.000	MF
GO:0140142	nucleocytoplasmic carrier activity	0.010	1.000	MF
GO:0004970	ionotropic glutamate receptor activity	0.014	1.000	MF
GO:0043047	single-stranded telomeric DNA binding	0.014	1.000	MF
GO:0003777	microtubule motor activity	0.015	1.000	MF
GO:0031995	insulin-like growth factor II binding	0.016	1.000	MF
GO:0001221	transcription cofactor binding	0.016	1.000	MF
GO:0008179	adenylate cyclase binding	0.019	1.000	MF
GO:0003726	double-stranded RNA adenosine deaminase ...	0.020	1.000	MF
GO:0003896	DNA primase activity	0.020	1.000	MF
GO:0004995	tachykinin receptor activity	0.020	1.000	MF
GO:0005010	insulin-like growth factor-activated rec...	0.020	1.000	MF
GO:0016941	natriuretic peptide receptor activity	0.020	1.000	MF
GO:0019776	Atg8 ligase activity	0.020	1.000	MF

GO:0030942	endoplasmic reticulum signal peptide bin...	0.020	1.000	MF
GO:0031997	N-terminal myristoylation domain binding	0.020	1.000	MF
GO:0061953	mRNA (adenine-N1-)-methyltransferase act...	0.020	1.000	MF
GO:0070053	thrombospondin receptor activity	0.020	1.000	MF
GO:1990460	leptin receptor binding	0.020	1.000	MF
GO:0019838	growth factor binding	0.021	1.000	MF
GO:0008092	cytoskeletal protein binding	0.021	1.000	MF
GO:0034237	protein kinase A regulatory subunit bind...	0.022	1.000	MF
GO:0000049	tRNA binding	0.024	1.000	MF
GO:0008046	axon guidance receptor activity	0.024	1.000	MF
GO:0008430	selenium binding	0.024	1.000	MF
GO:0005048	signal sequence binding	0.025	1.000	MF
GO:0017046	peptide hormone binding	0.025	1.000	MF
GO:0051219	phosphoprotein binding	0.026	1.000	MF
GO:0016790	thiolester hydrolase activity	0.028	1.000	MF
GO:0034062	5'-3' RNA polymerase activity	0.028	1.000	MF
GO:0097747	RNA polymerase activity	0.028	1.000	MF
GO:0008134	transcription factor binding	0.030	1.000	MF
GO:0090079	translation regulator activity, nucleic ...	0.032	1.000	MF
GO:0004690	cyclic nucleotide-dependent protein kina...	0.034	1.000	MF
GO:0004691	cAMP-dependent protein kinase activity	0.034	1.000	MF
GO:0019855	calcium channel inhibitor activity	0.034	1.000	MF
GO:0042301	phosphate ion binding	0.034	1.000	MF
GO:0003985	acetyl-CoA C-acetyltransferase activity	0.038	1.000	MF
GO:0004663	Rab geranylgeranyltransferase activity	0.038	1.000	MF
GO:0004839	ubiquitin activating enzyme activity	0.038	1.000	MF
GO:0004971	AMPA glutamate receptor activity	0.038	1.000	MF
GO:0005047	signal recognition particle binding	0.038	1.000	MF
GO:0005173	stem cell factor receptor binding	0.038	1.000	MF
GO:0016429	tRNA (adenine-N1-)-methyltransferase act...	0.038	1.000	MF
GO:0016807	cysteine-type carboxypeptidase activity	0.038	1.000	MF
GO:0034056	estrogen response element binding	0.038	1.000	MF
GO:0046912	transferase activity, transferring acyl ...	0.038	1.000	MF
GO:0070004	cysteine-type exopeptidase activity	0.038	1.000	MF
GO:0098882	structural constituent of presynaptic ac...	0.038	1.000	MF
GO:0001671	ATPase activator activity	0.039	1.000	MF
GO:0098918	structural constituent of synapse	0.040	1.000	MF
GO:0051018	protein kinase A binding	0.040	1.000	MF
GO:0034061	DNA polymerase activity	0.041	1.000	MF
GO:0008135	translation factor activity, RNA binding	0.043	1.000	MF
GO:0003774	motor activity	0.044	1.000	MF
GO:0008641	ubiquitin-like modifier activating enzym...	0.045	1.000	MF
GO:0015279	store-operated calcium channel activity	0.045	1.000	MF
GO:0098505	G-rich strand telomeric DNA binding	0.045	1.000	MF
GO:0008574	ATP-dependent microtubule motor activity...	0.046	1.000	MF
GO:0048027	mRNA 5'-UTR binding	0.046	1.000	MF
GO:0030594	neurotransmitter receptor activity	0.046	1.000	MF
GO:0019888	protein phosphatase regulator activity	0.047	1.000	MF
GO:0051010	microtubule plus-end binding	0.048	1.000	MF
GO:0015631	tubulin binding	0.049	1.000	MF
GO:0071005	U2-type precatalytic spliceosome	0.000	0.002	CC
GO:0071011	precatalytic spliceosome	0.000	0.002	CC

GO:0005684	U2-type spliceosomal complex	0.000	0.002	CC
GO:1990904	ribonucleoprotein complex	0.000	0.005	CC
GO:0005681	spliceosomal complex	0.000	0.005	CC
GO:0071007	U2-type catalytic step 2 spliceosome	0.000	0.005	CC
GO:0120114	Sm-like protein family complex	0.000	0.005	CC
GO:0071013	catalytic step 2 spliceosome	0.000	0.005	CC
GO:0005634	nucleus	0.000	0.008	CC
GO:0046540	U4/U6 x U5 tri-snRNP complex	0.000	0.012	CC
GO:0097526	spliceosomal tri-snRNP complex	0.000	0.012	CC
GO:0005654	nucleoplasm	0.000	0.014	CC
GO:0030532	small nuclear ribonucleoprotein complex	0.000	0.017	CC
GO:1990726	Lsm1-7-Pat1 complex	0.000	0.031	CC
GO:0034719	SMN-Sm protein complex	0.000	0.033	CC
GO:0097539	ciliary transition fiber	0.000	0.043	CC
GO:0044798	nuclear transcription factor complex	0.000	0.043	CC
GO:0097525	spliceosomal snRNP complex	0.000	0.044	CC
GO:0005840	ribosome	0.000	0.049	CC
GO:0043226	organelle	0.001	0.059	CC
GO:0043229	intracellular organelle	0.001	0.060	CC
GO:0097458	neuron part	0.001	0.065	CC
GO:0043005	neuron projection	0.001	0.072	CC
GO:0005689	U12-type spliceosomal complex	0.001	0.072	CC
GO:0005814	centriole	0.001	0.072	CC
GO:0044450	microtubule organizing center part	0.001	0.073	CC
GO:0032991	protein-containing complex	0.001	0.074	CC
GO:0044424	intracellular part	0.001	0.074	CC
GO:0031981	nuclear lumen	0.001	0.074	CC
GO:0043232	intracellular non-membrane-bounded organ...	0.001	0.079	CC
GO:0005622	intracellular	0.001	0.082	CC
GO:0120115	Lsm2-8 complex	0.001	0.082	CC
GO:0015935	small ribosomal subunit	0.001	0.082	CC
GO:0043228	non-membrane-bounded organelle	0.001	0.082	CC
GO:0034451	centriolar satellite	0.002	0.082	CC
GO:0031974	membrane-enclosed lumen	0.002	0.082	CC
GO:0043233	organelle lumen	0.002	0.082	CC
GO:0070013	intracellular organelle lumen	0.002	0.082	CC
GO:0044428	nuclear part	0.002	0.083	CC
GO:0005829	cytosol	0.002	0.098	CC
GO:0043231	intracellular membrane-bounded organelle	0.002	0.111	CC
GO:0044391	ribosomal subunit	0.002	0.115	CC
GO:0005686	U2 snRNP	0.003	0.157	CC
GO:0098982	GABA-ergic synapse	0.004	0.157	CC
GO:0030424	axon	0.004	0.157	CC
GO:0034663	endoplasmic reticulum chaperone complex	0.004	0.183	CC
GO:0022627	cytosolic small ribosomal subunit	0.006	0.237	CC
GO:0044451	nucleoplasm part	0.006	0.242	CC
GO:0044463	cell projection part	0.006	0.255	CC
GO:0120038	plasma membrane bounded cell projection ...	0.006	0.255	CC
GO:0043227	membrane-bounded organelle	0.007	0.287	CC
GO:0070822	Sin3-type complex	0.008	0.301	CC
GO:0034715	pICln-Sm protein complex	0.009	0.344	CC
GO:0061673	mitotic spindle astral microtubule	0.009	0.344	CC

GO:0005871	kinesin complex	0.010	0.344	CC
GO:0005952	cAMP-dependent protein kinase complex	0.010	0.345	CC
GO:0031970	organelle envelope lumen	0.010	0.346	CC
GO:0016605	PML body	0.010	0.355	CC
GO:0005623	cell	0.011	0.374	CC
GO:1902494	catalytic complex	0.011	0.374	CC
GO:0032838	plasma membrane bounded cell projection ...	0.012	0.403	CC
GO:0005687	U4 snRNP	0.014	0.423	CC
GO:0005688	U6 snRNP	0.014	0.423	CC
GO:0034709	methylosome	0.014	0.423	CC
GO:0005683	U7 snRNP	0.016	0.442	CC
GO:0005828	kinetochore microtubule	0.016	0.442	CC
GO:0030915	Smc5-Smc6 complex	0.016	0.442	CC
GO:0106068	SUMO ligase complex	0.016	0.442	CC
GO:0090575	RNA polymerase II transcription factor c...	0.016	0.442	CC
GO:0120025	plasma membrane bounded cell projection	0.016	0.442	CC
GO:0030425	dendrite	0.016	0.442	CC
GO:0030672	synaptic vesicle membrane	0.016	0.442	CC
GO:0099501	exocytic vesicle membrane	0.016	0.442	CC
GO:0044464	cell part	0.016	0.442	CC
GO:0097447	dendritic tree	0.017	0.447	CC
GO:0030055	cell-substrate junction	0.018	0.447	CC
GO:1904115	axon cytoplasm	0.018	0.447	CC
GO:0005694	chromosome	0.018	0.447	CC
GO:0005786	signal recognition particle, endoplasmic...	0.018	0.447	CC
GO:0016580	Sin3 complex	0.018	0.447	CC
GO:0044441	ciliary part	0.019	0.447	CC
GO:0005667	transcription factor complex	0.019	0.447	CC
GO:0005873	plus-end kinesin complex	0.020	0.447	CC
GO:0031510	SUMO activating enzyme complex	0.020	0.447	CC
GO:0035060	brahma complex	0.020	0.447	CC
GO:0044530	supraspliceosomal complex	0.020	0.447	CC
GO:0005925	focal adhesion	0.020	0.447	CC
GO:0005815	microtubule organizing center	0.020	0.447	CC
GO:0036064	ciliary basal body	0.020	0.447	CC
GO:0044445	cytosolic part	0.020	0.447	CC
GO:0005682	U5 snRNP	0.021	0.457	CC
GO:0030904	retromer complex	0.021	0.457	CC
GO:0005924	cell-substrate adherens junction	0.023	0.483	CC
GO:0033010	paranodal junction	0.023	0.487	CC
GO:1990316	Atg1/ULK1 kinase complex	0.023	0.487	CC
GO:0005813	centrosome	0.025	0.504	CC
GO:0055029	nuclear DNA-directed RNA polymerase comp...	0.025	0.504	CC
GO:0044427	chromosomal part	0.025	0.511	CC
GO:0000428	DNA-directed RNA polymerase complex	0.027	0.528	CC
GO:0022626	cytosolic ribosome	0.027	0.528	CC
GO:0036464	cytoplasmic ribonucleoprotein granule	0.029	0.565	CC
GO:0035770	ribonucleoprotein granule	0.030	0.568	CC
GO:0031083	BLOC-1 complex	0.031	0.568	CC
GO:0048500	signal recognition particle	0.031	0.568	CC
GO:0071004	U2-type prespliceosome	0.031	0.568	CC
GO:0071010	prespliceosome	0.031	0.568	CC

GO:0098554	cytoplasmic side of endoplasmic reticulu...	0.031	0.568	CC
GO:0030880	RNA polymerase complex	0.031	0.568	CC
GO:0043025	neuronal cell body	0.032	0.569	CC
GO:0000235	astral microtubule	0.033	0.569	CC
GO:0005641	nuclear envelope lumen	0.033	0.569	CC
GO:0005818	aster	0.033	0.569	CC
GO:0005832	chaperonin-containing T-complex	0.033	0.569	CC
GO:0033018	sarcoplasmic reticulum lumen	0.033	0.569	CC
GO:0090734	site of DNA damage	0.035	0.569	CC
GO:0042995	cell projection	0.037	0.569	CC
GO:0000798	nuclear cohesin complex	0.037	0.569	CC
GO:0005854	nascent polypeptide-associated complex	0.037	0.569	CC
GO:0005899	insulin receptor complex	0.037	0.569	CC
GO:0005968	Rab-protein geranylgeranyltransferase co...	0.037	0.569	CC
GO:0016602	CCAAT-binding factor complex	0.037	0.569	CC
GO:0030905	retromer, tubulation complex	0.037	0.569	CC
GO:0034991	nuclear meiotic cohesin complex	0.037	0.569	CC
GO:0060199	clathrin-sculpted glutamate transport ve...	0.037	0.569	CC
GO:0060203	clathrin-sculpted glutamate transport ve...	0.037	0.569	CC
GO:0072487	MSL complex	0.037	0.569	CC
GO:0097444	spine apparatus	0.037	0.569	CC
GO:0097487	multivesicular body, internal vesicle	0.037	0.569	CC
GO:0015630	microtubule cytoskeleton	0.037	0.569	CC
GO:0097729	9+2 motile cilium	0.038	0.569	CC
GO:0031092	platelet alpha granule membrane	0.038	0.569	CC
GO:0034045	phagophore assembly site membrane	0.038	0.569	CC
GO:0060077	inhibitory synapse	0.038	0.569	CC
GO:0097225	sperm midpiece	0.038	0.569	CC
GO:0016604	nuclear body	0.043	0.635	CC
GO:0005930	axoneme	0.044	0.635	CC
GO:0044305	calyx of Held	0.044	0.635	CC
GO:0099738	cell cortex region	0.045	0.635	CC
GO:0000795	synaptonemal complex	0.045	0.635	CC
GO:0005685	U1 snRNP	0.045	0.635	CC
GO:0042788	polysomal ribosome	0.045	0.635	CC
GO:0099086	synaptonemal structure	0.045	0.635	CC
GO:0036477	somatodendritic compartment	0.047	0.640	CC
GO:0099568	cytoplasmic region	0.047	0.640	CC
GO:0097014	ciliary plasm	0.047	0.640	CC
GO:0043034	costamere	0.047	0.640	CC
GO:0005758	mitochondrial intermembrane space	0.049	0.656	CC

OFC

GO.ID	Term	FET	BH	Category
GO:0007268	chemical synaptic transmission	0.000	0.000	BP
GO:0098916	anterograde trans-synaptic signaling	0.000	0.000	BP
GO:0099537	trans-synaptic signaling	0.000	0.000	BP
GO:0099536	synaptic signaling	0.000	0.000	BP
GO:0048167	regulation of synaptic plasticity	0.000	0.001	BP
GO:0050804	modulation of chemical synaptic transmis...	0.000	0.017	BP
GO:0099177	regulation of trans-synaptic signaling	0.000	0.017	BP
GO:1902683	regulation of receptor localization to s...	0.000	0.017	BP
GO:0031175	neuron projection development	0.000	0.035	BP
GO:0050808	synapse organization	0.000	0.053	BP
GO:0099173	postsynapse organization	0.000	0.104	BP
GO:0016081	synaptic vesicle docking	0.000	0.157	BP
GO:0007267	cell-cell signaling	0.000	0.179	BP
GO:0099565	chemical synaptic transmission, postsyna...	0.000	0.179	BP
GO:0007399	nervous system development	0.000	0.188	BP
GO:0023052	signaling	0.000	0.196	BP
GO:0051641	cellular localization	0.000	0.213	BP
GO:0050806	positive regulation of synaptic transmis...	0.000	0.213	BP
GO:0007154	cell communication	0.000	0.213	BP
GO:0048812	neuron projection morphogenesis	0.000	0.213	BP
GO:0010720	positive regulation of cell development	0.000	0.213	BP
GO:0007007	inner mitochondrial membrane organizatio...	0.000	0.213	BP
GO:0120035	regulation of plasma membrane bounded ce...	0.000	0.213	BP
GO:0120039	plasma membrane bounded cell projection ...	0.000	0.213	BP
GO:0030182	neuron differentiation	0.000	0.213	BP
GO:0050775	positive regulation of dendrite morphoge...	0.000	0.213	BP
GO:0099152	regulation of neurotransmitter receptor ...	0.000	0.213	BP
GO:1905749	regulation of endosome to plasma membran...	0.000	0.213	BP
GO:0050877	nervous system process	0.000	0.213	BP
GO:0003211	cardiac ventricle formation	0.000	0.213	BP
GO:0034201	response to oleic acid	0.000	0.213	BP
GO:0050909	sensory perception of taste	0.000	0.213	BP
GO:0048666	neuron development	0.000	0.213	BP
GO:0010975	regulation of neuron projection developm...	0.000	0.213	BP
GO:1902473	regulation of protein localization to sy...	0.000	0.213	BP
GO:0048858	cell projection morphogenesis	0.000	0.213	BP
GO:0031344	regulation of cell projection organizati...	0.001	0.229	BP
GO:0060079	excitatory postsynaptic potential	0.001	0.231	BP
GO:0051649	establishment of localization in cell	0.001	0.265	BP
GO:0098815	modulation of excitatory postsynaptic po...	0.001	0.270	BP
GO:0032990	cell part morphogenesis	0.001	0.271	BP
GO:0022008	neurogenesis	0.001	0.302	BP
GO:0097485	neuron projection guidance	0.001	0.302	BP
GO:2000463	positive regulation of excitatory postsy...	0.001	0.324	BP
GO:0031644	regulation of neurological system proces...	0.001	0.333	BP
GO:0003207	cardiac chamber formation	0.001	0.333	BP
GO:0060947	cardiac vascular smooth muscle cell diff...	0.001	0.333	BP
GO:0045664	regulation of neuron differentiation	0.001	0.333	BP
GO:0120036	plasma membrane bounded cell projection ...	0.001	0.348	BP
GO:0048699	generation of neurons	0.001	0.354	BP

GO:0050913	sensory perception of bitter taste	0.001	0.354	BP
GO:0090150	establishment of protein localization to...	0.001	0.354	BP
GO:0048667	cell morphogenesis involved in neuron di...	0.001	0.354	BP
GO:0046907	intracellular transport	0.001	0.354	BP
GO:0010646	regulation of cell communication	0.001	0.355	BP
GO:0010976	positive regulation of neuron projection...	0.001	0.355	BP
GO:0045666	positive regulation of neuron differenti...	0.001	0.386	BP
GO:0030030	cell projection organization	0.001	0.386	BP
GO:0031646	positive regulation of neurological syst...	0.002	0.386	BP
GO:0060997	dendritic spine morphogenesis	0.002	0.386	BP
GO:0061001	regulation of dendritic spine morphogene...	0.002	0.386	BP
GO:0007411	axon guidance	0.002	0.386	BP
GO:0010770	positive regulation of cell morphogenesi...	0.002	0.388	BP
GO:0051960	regulation of nervous system development	0.002	0.394	BP
GO:0048168	regulation of neuronal synaptic plastici...	0.002	0.402	BP
GO:0051962	positive regulation of nervous system de...	0.002	0.429	BP
GO:0070050	neuron cellular homeostasis	0.002	0.429	BP
GO:1903044	protein localization to membrane raft	0.002	0.429	BP
GO:0023051	regulation of signaling	0.002	0.429	BP
GO:0060078	regulation of postsynaptic membrane pote...	0.002	0.429	BP
GO:0050767	regulation of neurogenesis	0.002	0.433	BP
GO:0031346	positive regulation of cell projection o...	0.002	0.433	BP
GO:0001580	detection of chemical stimulus involved ...	0.002	0.457	BP
GO:0036474	cell death in response to hydrogen perox...	0.002	0.457	BP
GO:0000904	cell morphogenesis involved in different...	0.002	0.459	BP
GO:0050769	positive regulation of neurogenesis	0.002	0.464	BP
GO:0008277	regulation of G protein-coupled receptor...	0.002	0.472	BP
GO:0051650	establishment of vesicle localization	0.002	0.474	BP
GO:0106027	neuron projection organization	0.002	0.474	BP
GO:0097061	dendritic spine organization	0.003	0.495	BP
GO:0042407	cristae formation	0.003	0.556	BP
GO:0060284	regulation of cell development	0.003	0.556	BP
GO:0048814	regulation of dendrite morphogenesis	0.003	0.566	BP
GO:0051648	vesicle localization	0.003	0.624	BP
GO:0007006	mitochondrial membrane organization	0.004	0.683	BP
GO:0014047	glutamate secretion	0.004	0.712	BP
GO:0007269	neurotransmitter secretion	0.004	0.712	BP
GO:0061003	positive regulation of dendritic spine m...	0.004	0.712	BP
GO:0035304	regulation of protein dephosphorylation	0.004	0.712	BP
GO:0048172	regulation of short-term neuronal synapt...	0.004	0.718	BP
GO:0099643	signal release from synapse	0.004	0.736	BP
GO:0010769	regulation of cell morphogenesis involve...	0.005	0.761	BP
GO:0010948	negative regulation of cell cycle proces...	0.005	0.761	BP
GO:0045786	negative regulation of cell cycle	0.005	0.761	BP
GO:0006614	SRP-dependent cotranslational protein ta...	0.005	0.765	BP
GO:0016079	synaptic vesicle exocytosis	0.005	0.824	BP
GO:0099175	regulation of postsynapse organization	0.005	0.825	BP
GO:0051656	establishment of organelle localization	0.006	0.837	BP
GO:0007096	regulation of exit from mitosis	0.006	0.837	BP
GO:0007158	neuron cell-cell adhesion	0.006	0.837	BP
GO:0099509	regulation of presynaptic cytosolic calc...	0.006	0.837	BP
GO:0051301	cell division	0.006	0.837	BP

GO:0002542	Factor XII activation	0.006	0.837	BP
GO:0009386	translational attenuation	0.006	0.837	BP
GO:0043314	negative regulation of neutrophil degran...	0.006	0.837	BP
GO:0060488	orthogonal dichotomous subdivision of te...	0.006	0.837	BP
GO:0060489	planar dichotomous subdivision of termin...	0.006	0.837	BP
GO:0060490	lateral sprouting involved in lung morph...	0.006	0.837	BP
GO:0071418	cellular response to amine stimulus	0.006	0.837	BP
GO:1904046	negative regulation of vascular endothel...	0.006	0.837	BP
GO:0035418	protein localization to synapse	0.006	0.869	BP
GO:0050912	detection of chemical stimulus involved ...	0.007	0.891	BP
GO:0045047	protein targeting to ER	0.007	0.891	BP
GO:0006796	phosphate-containing compound metabolic ...	0.007	0.891	BP
GO:0006613	cotranslational protein targeting to mem...	0.007	0.891	BP
GO:0072657	protein localization to membrane	0.007	0.891	BP
GO:0050789	regulation of biological process	0.007	0.891	BP
GO:0022604	regulation of cell morphogenesis	0.007	0.891	BP
GO:0070727	cellular macromolecule localization	0.007	0.891	BP
GO:0032543	mitochondrial translation	0.007	0.891	BP
GO:0016310	phosphorylation	0.007	0.891	BP
GO:0097120	receptor localization to synapse	0.007	0.891	BP
GO:0099504	synaptic vesicle cycle	0.007	0.891	BP
GO:0021670	lateral ventricle development	0.007	0.891	BP
GO:0030322	stabilization of membrane potential	0.007	0.891	BP
GO:0051917	regulation of fibrinolysis	0.007	0.891	BP
GO:0098974	postsynaptic actin cytoskeleton organiza...	0.007	0.891	BP
GO:0034613	cellular protein localization	0.007	0.891	BP
GO:0043666	regulation of phosphoprotein phosphatase...	0.007	0.891	BP
GO:0048489	synaptic vesicle transport	0.007	0.891	BP
GO:0097480	establishment of synaptic vesicle locali...	0.007	0.891	BP
GO:0060029	convergent extension involved in organog...	0.008	0.891	BP
GO:0006468	protein phosphorylation	0.008	0.891	BP
GO:0140014	mitotic nuclear division	0.008	0.907	BP
GO:0099072	regulation of postsynaptic membrane neur...	0.008	0.908	BP
GO:0043114	regulation of vascular permeability	0.008	0.908	BP
GO:0010661	positive regulation of muscle cell apopt...	0.008	0.921	BP
GO:0099563	modification of synaptic structure	0.008	0.921	BP
GO:0097479	synaptic vesicle localization	0.008	0.921	BP
GO:0006793	phosphorus metabolic process	0.008	0.943	BP
GO:0048468	cell development	0.009	0.963	BP
GO:0007612	learning	0.009	1.000	BP
GO:0072599	establishment of protein localization to...	0.009	1.000	BP
GO:0044267	cellular protein metabolic process	0.009	1.000	BP
GO:0007165	signal transduction	0.010	1.000	BP
GO:0000028	ribosomal small subunit assembly	0.010	1.000	BP
GO:0002029	desensitization of G protein-coupled rec...	0.010	1.000	BP
GO:0022401	negative adaptation of signaling pathway	0.010	1.000	BP
GO:0007528	neuromuscular junction development	0.010	1.000	BP
GO:0033048	negative regulation of mitotic sister ch...	0.010	1.000	BP
GO:2001222	regulation of neuron migration	0.010	1.000	BP
GO:0035886	vascular smooth muscle cell differentiat...	0.010	1.000	BP
GO:0007409	axonogenesis	0.010	1.000	BP
GO:0002091	negative regulation of receptor internal...	0.010	1.000	BP

GO:0098962	regulation of postsynaptic neurotransmit...	0.010	1.000	BP
GO:0001505	regulation of neurotransmitter levels	0.010	1.000	BP
GO:1990138	neuron projection extension	0.011	1.000	BP
GO:0060341	regulation of cellular localization	0.011	1.000	BP
GO:0033046	negative regulation of sister chromatid ...	0.011	1.000	BP
GO:0051985	negative regulation of chromosome segreg...	0.011	1.000	BP
GO:0007611	learning or memory	0.012	1.000	BP
GO:0021549	cerebellum development	0.012	1.000	BP
GO:0048813	dendrite morphogenesis	0.012	1.000	BP
GO:0000910	cytokinesis	0.012	1.000	BP
GO:0140053	mitochondrial gene expression	0.012	1.000	BP
GO:0023058	adaptation of signaling pathway	0.012	1.000	BP
GO:2000049	positive regulation of cell-cell adhesio...	0.012	1.000	BP
GO:0061564	axon development	0.013	1.000	BP
GO:0045839	negative regulation of mitotic nuclear d...	0.013	1.000	BP
GO:0007186	G protein-coupled receptor signaling pat...	0.013	1.000	BP
GO:0051668	localization within membrane	0.013	1.000	BP
GO:0009966	regulation of signal transduction	0.013	1.000	BP
GO:0051693	actin filament capping	0.013	1.000	BP
GO:0060976	coronary vasculature development	0.013	1.000	BP
GO:0050807	regulation of synapse organization	0.013	1.000	BP
GO:0006612	protein targeting to membrane	0.014	1.000	BP
GO:0035303	regulation of dephosphorylation	0.014	1.000	BP
GO:0019538	protein metabolic process	0.014	1.000	BP
GO:0035358	regulation of peroxisome proliferator ac...	0.014	1.000	BP
GO:0099188	postsynaptic cytoskeleton organization	0.014	1.000	BP
GO:2001256	regulation of store-operated calcium ent...	0.014	1.000	BP
GO:0007005	mitochondrion organization	0.014	1.000	BP
GO:0042391	regulation of membrane potential	0.015	1.000	BP
GO:0051234	establishment of localization	0.015	1.000	BP
GO:0006904	vesicle docking involved in exocytosis	0.015	1.000	BP
GO:0070542	response to fatty acid	0.015	1.000	BP
GO:0043984	histone H4-K16 acetylation	0.015	1.000	BP
GO:0060996	dendritic spine development	0.016	1.000	BP
GO:0099003	vesicle-mediated transport in synapse	0.016	1.000	BP
GO:0007264	small GTPase mediated signal transductio...	0.016	1.000	BP
GO:0070125	mitochondrial translational elongation	0.016	1.000	BP
GO:0045597	positive regulation of cell differentiat...	0.017	1.000	BP
GO:0001907	killing by symbiont of host cells	0.017	1.000	BP
GO:0002254	kinin cascade	0.017	1.000	BP
GO:0002353	plasma kallikrein-kinin cascade	0.017	1.000	BP
GO:0002541	activation of plasma proteins involved i...	0.017	1.000	BP
GO:0010735	positive regulation of transcription via...	0.017	1.000	BP
GO:0014022	neural plate elongation	0.017	1.000	BP
GO:0022007	convergent extension involved in neural ...	0.017	1.000	BP
GO:0032849	positive regulation of cellular pH reduc...	0.017	1.000	BP
GO:0038086	VEGF-activated platelet-derived growth f...	0.017	1.000	BP
GO:0038091	positive regulation of cell proliferatio...	0.017	1.000	BP
GO:0044004	disruption by symbiont of host cell	0.017	1.000	BP
GO:0044211	CTP salvage	0.017	1.000	BP
GO:0044533	positive regulation of apoptotic process...	0.017	1.000	BP
GO:0046016	positive regulation of transcription by ...	0.017	1.000	BP

GO:0052042	positive regulation by symbiont of host ...	0.017	1.000	BP
GO:0052330	positive regulation by organism of progr...	0.017	1.000	BP
GO:0052501	positive regulation by organism of apopt...	0.017	1.000	BP
GO:0060313	negative regulation of blood vessel remo...	0.017	1.000	BP
GO:0060448	dichotomous subdivision of terminal unit...	0.017	1.000	BP
GO:0060948	cardiac vascular smooth muscle cell deve...	0.017	1.000	BP
GO:0061789	dense core granule priming	0.017	1.000	BP
GO:0071400	cellular response to oleic acid	0.017	1.000	BP
GO:1903225	negative regulation of endodermal cell d...	0.017	1.000	BP
GO:1904116	response to vasopressin	0.017	1.000	BP
GO:1904117	cellular response to vasopressin	0.017	1.000	BP
GO:0046717	acid secretion	0.017	1.000	BP
GO:0050885	neuromuscular process controlling balanc...	0.017	1.000	BP
GO:0010458	exit from mitosis	0.018	1.000	BP
GO:0051179	localization	0.018	1.000	BP
GO:0032989	cellular component morphogenesis	0.018	1.000	BP
GO:0070126	mitochondrial translational termination	0.018	1.000	BP
GO:0051783	regulation of nuclear division	0.018	1.000	BP
GO:0035306	positive regulation of dephosphorylation	0.018	1.000	BP
GO:0061951	establishment of protein localization to...	0.018	1.000	BP
GO:0002031	G protein-coupled receptor internalizati...	0.019	1.000	BP
GO:0035360	positive regulation of peroxisome prolif...	0.019	1.000	BP
GO:0045039	protein import into mitochondrial inner ...	0.019	1.000	BP
GO:0051639	actin filament network formation	0.019	1.000	BP
GO:0060600	dichotomous subdivision of an epithelial...	0.019	1.000	BP
GO:0097084	vascular smooth muscle cell development	0.019	1.000	BP
GO:0098696	regulation of neurotransmitter receptor ...	0.019	1.000	BP
GO:0048261	negative regulation of receptor-mediated...	0.019	1.000	BP
GO:0055003	cardiac myofibril assembly	0.019	1.000	BP
GO:0071157	negative regulation of cell cycle arrest	0.019	1.000	BP
GO:1903861	positive regulation of dendrite extensio...	0.019	1.000	BP
GO:0007088	regulation of mitotic nuclear division	0.020	1.000	BP
GO:0051726	regulation of cell cycle	0.020	1.000	BP
GO:0043624	cellular protein complex disassembly	0.020	1.000	BP
GO:0021695	cerebellar cortex development	0.020	1.000	BP
GO:0006605	protein targeting	0.020	1.000	BP
GO:0000902	cell morphogenesis	0.020	1.000	BP
GO:0006836	neurotransmitter transport	0.020	1.000	BP
GO:0051640	organelle localization	0.020	1.000	BP
GO:0051784	negative regulation of nuclear division	0.021	1.000	BP
GO:1900006	positive regulation of dendrite developm...	0.021	1.000	BP
GO:0030835	negative regulation of actin filament de...	0.021	1.000	BP
GO:1905819	negative regulation of chromosome separa...	0.021	1.000	BP
GO:2000816	negative regulation of mitotic sister ch...	0.021	1.000	BP
GO:0006810	transport	0.022	1.000	BP
GO:0022037	metencephalon development	0.022	1.000	BP
GO:0050803	regulation of synapse structure or activ...	0.022	1.000	BP
GO:0010921	regulation of phosphatase activity	0.022	1.000	BP
GO:0035556	intracellular signal transduction	0.022	1.000	BP
GO:0060291	long-term synaptic potentiation	0.022	1.000	BP
GO:0032386	regulation of intracellular transport	0.023	1.000	BP
GO:0060998	regulation of dendritic spine developmen...	0.023	1.000	BP

GO:0030194	positive regulation of blood coagulation	0.023	1.000	BP
GO:1900048	positive regulation of hemostasis	0.023	1.000	BP
GO:0006888	ER to Golgi vesicle-mediated transport	0.023	1.000	BP
GO:0043967	histone H4 acetylation	0.023	1.000	BP
GO:0003283	atrial septum development	0.024	1.000	BP
GO:0048791	calcium ion-regulated exocytosis of neur...	0.024	1.000	BP
GO:0099558	maintenance of synapse structure	0.024	1.000	BP
GO:1901032	negative regulation of response to react...	0.024	1.000	BP
GO:1903206	negative regulation of hydrogen peroxide...	0.024	1.000	BP
GO:2000047	regulation of cell-cell adhesion mediate...	0.024	1.000	BP
GO:2001039	negative regulation of cellular response...	0.024	1.000	BP
GO:0016192	vesicle-mediated transport	0.024	1.000	BP
GO:0008306	associative learning	0.025	1.000	BP
GO:0002090	regulation of receptor internalization	0.025	1.000	BP
GO:0030834	regulation of actin filament depolymeriz...	0.025	1.000	BP
GO:0070972	protein localization to endoplasmic reti...	0.026	1.000	BP
GO:0051306	mitotic sister chromatid separation	0.026	1.000	BP
GO:0032102	negative regulation of response to exter...	0.026	1.000	BP
GO:0051712	positive regulation of killing of cells ...	0.027	1.000	BP
GO:0051764	actin crosslink formation	0.027	1.000	BP
GO:0060712	spongiotrophoblast layer development	0.027	1.000	BP
GO:0106049	regulation of cellular response to osmot...	0.027	1.000	BP
GO:0006464	cellular protein modification process	0.027	1.000	BP
GO:0036211	protein modification process	0.027	1.000	BP
GO:0006415	translational termination	0.027	1.000	BP
GO:0034101	erythrocyte homeostasis	0.027	1.000	BP
GO:0050820	positive regulation of coagulation	0.028	1.000	BP
GO:1903859	regulation of dendrite extension	0.028	1.000	BP
GO:0030224	monocyte differentiation	0.028	1.000	BP
GO:1903131	mononuclear cell differentiation	0.028	1.000	BP
GO:0035904	aorta development	0.028	1.000	BP
GO:1902099	regulation of metaphase/anaphase transit...	0.028	1.000	BP
GO:2001243	negative regulation of intrinsic apoptot...	0.028	1.000	BP
GO:1905818	regulation of chromosome separation	0.029	1.000	BP
GO:0007049	cell cycle	0.029	1.000	BP
GO:0017156	calcium ion regulated exocytosis	0.029	1.000	BP
GO:0007266	Rho protein signal transduction	0.029	1.000	BP
GO:0042249	establishment of planar polarity of embr...	0.030	1.000	BP
GO:0043968	histone H2A acetylation	0.030	1.000	BP
GO:0050773	regulation of dendrite development	0.031	1.000	BP
GO:0035307	positive regulation of protein dephospho...	0.031	1.000	BP
GO:0010564	regulation of cell cycle process	0.031	1.000	BP
GO:0043412	macromolecule modification	0.031	1.000	BP
GO:0051983	regulation of chromosome segregation	0.031	1.000	BP
GO:0048675	axon extension	0.032	1.000	BP
GO:0000451	rRNA 2'-O-methylation	0.032	1.000	BP
GO:0003289	atrial septum primum morphogenesis	0.032	1.000	BP
GO:0003357	noradrenergic neuron differentiation	0.032	1.000	BP
GO:0003400	regulation of COPII vesicle coating	0.032	1.000	BP
GO:0032847	regulation of cellular pH reduction	0.032	1.000	BP
GO:0035502	metanephric part of ureteric bud develop...	0.032	1.000	BP
GO:0035910	ascending aorta morphogenesis	0.032	1.000	BP

GO:0042636	negative regulation of hair cycle	0.032	1.000	BP
GO:0045338	farnesyl diphosphate metabolic process	0.032	1.000	BP
GO:0045990	carbon catabolite regulation of transcri...	0.032	1.000	BP
GO:0045991	carbon catabolite activation of transcri...	0.032	1.000	BP
GO:0046015	regulation of transcription by glucose	0.032	1.000	BP
GO:0048104	establishment of body hair or bristle pl...	0.032	1.000	BP
GO:0048105	establishment of body hair planar orient...	0.032	1.000	BP
GO:0051919	positive regulation of fibrinolysis	0.032	1.000	BP
GO:0051971	positive regulation of transmission of n...	0.032	1.000	BP
GO:0071962	mitotic sister chromatid cohesion, centr...	0.032	1.000	BP
GO:0090063	positive regulation of microtubule nucle...	0.032	1.000	BP
GO:0090113	regulation of ER to Golgi vesicle-mediat...	0.032	1.000	BP
GO:0090500	endocardial cushion to mesenchymal trans...	0.032	1.000	BP
GO:0097475	motor neuron migration	0.032	1.000	BP
GO:1902109	negative regulation of mitochondrial mem...	0.032	1.000	BP
GO:1903224	regulation of endodermal cell differenti...	0.032	1.000	BP
GO:1905114	cell surface receptor signaling pathway ...	0.032	1.000	BP
GO:0045930	negative regulation of mitotic cell cycl...	0.032	1.000	BP
GO:0060840	artery development	0.032	1.000	BP
GO:1903035	negative regulation of response to wound...	0.032	1.000	BP
GO:0003205	cardiac chamber development	0.033	1.000	BP
GO:0007094	mitotic spindle assembly checkpoint	0.033	1.000	BP
GO:0031577	spindle checkpoint	0.033	1.000	BP
GO:0071173	spindle assembly checkpoint	0.033	1.000	BP
GO:0071174	mitotic spindle checkpoint	0.033	1.000	BP
GO:0019433	triglyceride catabolic process	0.033	1.000	BP
GO:0030218	erythrocyte differentiation	0.034	1.000	BP
GO:0010660	regulation of muscle cell apoptotic proc...	0.035	1.000	BP
GO:0035308	negative regulation of protein dephospho...	0.035	1.000	BP
GO:0050890	cognition	0.035	1.000	BP
GO:0030042	actin filament depolymerization	0.035	1.000	BP
GO:0002887	negative regulation of myeloid leukocyte...	0.036	1.000	BP
GO:0051709	regulation of killing of cells of other ...	0.036	1.000	BP
GO:0050905	neuromuscular process	0.036	1.000	BP
GO:0022402	cell cycle process	0.036	1.000	BP
GO:0003279	cardiac septum development	0.036	1.000	BP
GO:0098693	regulation of synaptic vesicle cycle	0.036	1.000	BP
GO:0006595	polyamine metabolic process	0.037	1.000	BP
GO:0097107	postsynaptic density assembly	0.037	1.000	BP
GO:0099010	modification of postsynaptic structure	0.037	1.000	BP
GO:1901991	negative regulation of mitotic cell cycl...	0.037	1.000	BP
GO:1903539	protein localization to postsynaptic mem...	0.037	1.000	BP
GO:0051128	regulation of cellular component organiz...	0.037	1.000	BP
GO:1903047	mitotic cell cycle process	0.038	1.000	BP
GO:0048585	negative regulation of response to stimu...	0.038	1.000	BP
GO:0000278	mitotic cell cycle	0.038	1.000	BP
GO:0019083	viral transcription	0.039	1.000	BP
GO:0040020	regulation of meiotic nuclear division	0.039	1.000	BP
GO:1903319	positive regulation of protein maturatio...	0.039	1.000	BP
GO:0007613	memory	0.039	1.000	BP
GO:0044784	metaphase/anaphase transition of cell cy...	0.039	1.000	BP
GO:0010562	positive regulation of phosphorus metabo...	0.039	1.000	BP

GO:0045937	positive regulation of phosphate metabol...	0.039	1.000	BP
GO:0007606	sensory perception of chemical stimulus	0.040	1.000	BP
GO:0000165	MAPK cascade	0.040	1.000	BP
GO:0006414	translational elongation	0.040	1.000	BP
GO:1901988	negative regulation of cell cycle phase ...	0.041	1.000	BP
GO:0000184	nuclear-transcribed mRNA catabolic proce...	0.041	1.000	BP
GO:0051094	positive regulation of developmental pro...	0.041	1.000	BP
GO:1901564	organonitrogen compound metabolic proces...	0.041	1.000	BP
GO:0061097	regulation of protein tyrosine kinase ac...	0.041	1.000	BP
GO:1901987	regulation of cell cycle phase transitio...	0.042	1.000	BP
GO:0008104	protein localization	0.042	1.000	BP
GO:0032594	protein transport within lipid bilayer	0.042	1.000	BP
GO:0045841	negative regulation of mitotic metaphase...	0.042	1.000	BP
GO:0071709	membrane assembly	0.042	1.000	BP
GO:1902100	negative regulation of metaphase/anaphas...	0.042	1.000	BP
GO:0090102	cochlea development	0.044	1.000	BP
GO:0022603	regulation of anatomical structure morph...	0.044	1.000	BP
GO:0006412	translation	0.044	1.000	BP
GO:0002115	store-operated calcium entry	0.044	1.000	BP
GO:0071498	cellular response to fluid shear stress	0.044	1.000	BP
GO:0003230	cardiac atrium development	0.045	1.000	BP
GO:0006851	mitochondrial calcium ion transmembrane ...	0.045	1.000	BP
GO:0070528	protein kinase C signaling	0.045	1.000	BP
GO:0065007	biological regulation	0.046	1.000	BP
GO:0010657	muscle cell apoptotic process	0.046	1.000	BP
GO:1901880	negative regulation of protein depolymer...	0.046	1.000	BP
GO:0007163	establishment or maintenance of cell pol...	0.046	1.000	BP
GO:0048872	homeostasis of number of cells	0.046	1.000	BP
GO:0001765	membrane raft assembly	0.047	1.000	BP
GO:0010663	positive regulation of striated muscle c...	0.047	1.000	BP
GO:0010666	positive regulation of cardiac muscle ce...	0.047	1.000	BP
GO:0038003	opioid receptor signaling pathway	0.047	1.000	BP
GO:0043116	negative regulation of vascular permeabi...	0.047	1.000	BP
GO:0045836	positive regulation of meiotic nuclear d...	0.047	1.000	BP
GO:0047484	regulation of response to osmotic stress	0.047	1.000	BP
GO:0060413	atrial septum morphogenesis	0.047	1.000	BP
GO:0098887	neurotransmitter receptor transport, end...	0.047	1.000	BP
GO:0099151	regulation of postsynaptic density assem...	0.047	1.000	BP
GO:0099638	endosome to plasma membrane protein tran...	0.047	1.000	BP
GO:0099639	neurotransmitter receptor transport, end...	0.047	1.000	BP
GO:0000819	sister chromatid segregation	0.047	1.000	BP
GO:0006120	mitochondrial electron transport, NADH t...	0.048	1.000	BP
GO:0010965	regulation of mitotic sister chromatid s...	0.048	1.000	BP
GO:0048008	platelet-derived growth factor receptor ...	0.048	1.000	BP
GO:0090307	mitotic spindle assembly	0.048	1.000	BP
GO:0090313	regulation of protein targeting to membr...	0.048	1.000	BP
GO:0070301	cellular response to hydrogen peroxide	0.048	1.000	BP
GO:0061640	cytoskeleton-dependent cytokinesis	0.048	1.000	BP
GO:0032984	protein-containing complex disassembly	0.048	1.000	BP
GO:0008542	visual learning	0.049	1.000	BP
GO:0045744	negative regulation of G protein-coupled...	0.049	1.000	BP
GO:0007050	cell cycle arrest	0.049	1.000	BP

GO:0006886	intracellular protein transport	0.049	1.000	BP
GO:0000070	mitotic sister chromatid segregation	0.050	1.000	BP
GO:0003735	structural constituent of ribosome	0.000	0.045	MF
GO:0005516	calmodulin binding	0.000	0.045	MF
GO:0033038	bitter taste receptor activity	0.000	0.345	MF
GO:0005198	structural molecule activity	0.001	0.682	MF
GO:0098918	structural constituent of synapse	0.001	0.682	MF
GO:0004683	calmodulin-dependent protein kinase acti...	0.001	0.682	MF
GO:0008527	taste receptor activity	0.001	0.816	MF
GO:0019208	phosphatase regulator activity	0.003	1.000	MF
GO:0019888	protein phosphatase regulator activity	0.004	1.000	MF
GO:0051020	GTPase binding	0.005	1.000	MF
GO:0019211	phosphatase activator activity	0.006	1.000	MF
GO:0008967	phosphoglycolate phosphatase activity	0.006	1.000	MF
GO:0099510	calcium ion binding involved in regulati...	0.006	1.000	MF
GO:0099534	calcium ion binding involved in regulati...	0.006	1.000	MF
GO:0004860	protein kinase inhibitor activity	0.006	1.000	MF
GO:0035254	glutamate receptor binding	0.006	1.000	MF
GO:0030506	ankyrin binding	0.008	1.000	MF
GO:0005085	guanyl-nucleotide exchange factor activi...	0.008	1.000	MF
GO:0005261	cation channel activity	0.009	1.000	MF
GO:0097110	scaffold protein binding	0.009	1.000	MF
GO:0019210	kinase inhibitor activity	0.010	1.000	MF
GO:0019901	protein kinase binding	0.010	1.000	MF
GO:0004674	protein serine/threonine kinase activity	0.012	1.000	MF
GO:0140103	catalytic activity, acting on a glycopro...	0.012	1.000	MF
GO:0035529	NADH pyrophosphatase activity	0.013	1.000	MF
GO:0140096	catalytic activity, acting on a protein	0.013	1.000	MF
GO:0072542	protein phosphatase activator activity	0.014	1.000	MF
GO:0008194	UDP-glycosyltransferase activity	0.017	1.000	MF
GO:0005544	calcium-dependent phospholipid binding	0.017	1.000	MF
GO:0030306	ADP-ribosylation factor binding	0.019	1.000	MF
GO:0035256	G protein-coupled glutamate receptor bin...	0.019	1.000	MF
GO:0070878	primary miRNA binding	0.019	1.000	MF
GO:0003954	NADH dehydrogenase activity	0.020	1.000	MF
GO:0008137	NADH dehydrogenase (ubiquinone) activity	0.020	1.000	MF
GO:0048156	tau protein binding	0.020	1.000	MF
GO:0050136	NADH dehydrogenase (quinone) activity	0.020	1.000	MF
GO:0098772	molecular function regulator	0.023	1.000	MF
GO:0017136	NAD-dependent histone deacetylase activi...	0.024	1.000	MF
GO:0019900	kinase binding	0.024	1.000	MF
GO:0030332	cyclin binding	0.024	1.000	MF
GO:0004672	protein kinase activity	0.025	1.000	MF
GO:0004690	cyclic nucleotide-dependent protein kina...	0.027	1.000	MF
GO:0004691	cAMP-dependent protein kinase activity	0.027	1.000	MF
GO:0008417	fucosyltransferase activity	0.027	1.000	MF
GO:0005267	potassium channel activity	0.030	1.000	MF
GO:0004551	nucleotide diphosphatase activity	0.030	1.000	MF
GO:0034979	NAD-dependent protein deacetylase activi...	0.030	1.000	MF
GO:0005089	Rho guanyl-nucleotide exchange factor ac...	0.030	1.000	MF
GO:0016758	transferase activity, transferring hexos...	0.031	1.000	MF
GO:0022843	voltage-gated cation channel activity	0.032	1.000	MF

GO:0005080	protein kinase C binding	0.032	1.000	MF
GO:0004096	catalase activity	0.032	1.000	MF
GO:0004849	uridine kinase activity	0.032	1.000	MF
GO:0005314	high-affinity glutamate transmembrane tr...	0.032	1.000	MF
GO:0015501	glutamate:sodium symporter activity	0.032	1.000	MF
GO:0030943	mitochondrion targeting sequence binding	0.032	1.000	MF
GO:0045134	uridine-diphosphatase activity	0.032	1.000	MF
GO:0046912	transferase activity, transferring acyl ...	0.032	1.000	MF
GO:0046920	alpha-(1->3)-fucosyltransferase activity	0.032	1.000	MF
GO:0047696	beta-adrenergic receptor kinase activity	0.032	1.000	MF
GO:0098519	nucleotide phosphatase activity, acting ...	0.032	1.000	MF
GO:0019887	protein kinase regulator activity	0.033	1.000	MF
GO:0019899	enzyme binding	0.036	1.000	MF
GO:0031078	histone deacetylase activity (H3-K14 spe...	0.036	1.000	MF
GO:0032041	NAD-dependent histone deacetylase activi...	0.036	1.000	MF
GO:0043995	histone acetyltransferase activity (H4-K...	0.036	1.000	MF
GO:0043996	histone acetyltransferase activity (H4-K...	0.036	1.000	MF
GO:0046972	histone acetyltransferase activity (H4-K...	0.036	1.000	MF
GO:0099186	structural constituent of postsynapse	0.036	1.000	MF
GO:0017048	Rho GTPase binding	0.037	1.000	MF
GO:0019213	deacetylase activity	0.039	1.000	MF
GO:0042056	chemoattractant activity	0.045	1.000	MF
GO:0051019	mitogen-activated protein kinase binding	0.045	1.000	MF
GO:0001094	TFIID-class transcription factor complex...	0.047	1.000	MF
GO:0015368	calcium:cation antiporter activity	0.047	1.000	MF
GO:0030898	actin-dependent ATPase activity	0.047	1.000	MF
GO:0031386	protein tag	0.047	1.000	MF
GO:0070181	small ribosomal subunit rRNA binding	0.047	1.000	MF
GO:0097458	neuron part	0.000	0.000	CC
GO:0045202	synapse	0.000	0.000	CC
GO:0044456	synapse part	0.000	0.000	CC
GO:0043005	neuron projection	0.000	0.000	CC
GO:0044448	cell cortex part	0.000	0.000	CC
GO:0098978	glutamatergic synapse	0.000	0.000	CC
GO:0098984	neuron to neuron synapse	0.000	0.001	CC
GO:0014069	postsynaptic density	0.000	0.002	CC
GO:0032279	asymmetric synapse	0.000	0.002	CC
GO:0030054	cell junction	0.000	0.002	CC
GO:0036477	somatodendritic compartment	0.000	0.002	CC
GO:0098794	postsynapse	0.000	0.003	CC
GO:0099572	postsynaptic specialization	0.000	0.005	CC
GO:0097060	synaptic membrane	0.000	0.005	CC
GO:0098793	presynapse	0.000	0.016	CC
GO:0098798	mitochondrial protein complex	0.000	0.016	CC
GO:0099738	cell cortex region	0.000	0.016	CC
GO:0043197	dendritic spine	0.000	0.016	CC
GO:0030863	cortical cytoskeleton	0.000	0.016	CC
GO:0016020	membrane	0.000	0.016	CC
GO:0005938	cell cortex	0.000	0.017	CC
GO:0044309	neuron spine	0.000	0.017	CC
GO:0019866	organelle inner membrane	0.000	0.019	CC
GO:0042995	cell projection	0.000	0.022	CC

GO:0097447	dendritic tree	0.000	0.024	CC
GO:0098800	inner mitochondrial membrane protein com...	0.000	0.024	CC
GO:0044391	ribosomal subunit	0.000	0.025	CC
GO:0044463	cell projection part	0.000	0.029	CC
GO:0120038	plasma membrane bounded cell projection ...	0.000	0.029	CC
GO:0030425	dendrite	0.000	0.030	CC
GO:0120025	plasma membrane bounded cell projection	0.001	0.033	CC
GO:0150034	distal axon	0.001	0.040	CC
GO:0099091	postsynaptic specialization, intracellul...	0.001	0.041	CC
GO:0044297	cell body	0.001	0.041	CC
GO:0098688	parallel fiber to Purkinje cell synapse	0.001	0.045	CC
GO:0098831	presynaptic active zone cytoplasmic comp...	0.001	0.045	CC
GO:0033267	axon part	0.001	0.047	CC
GO:0014731	spectrin-associated cytoskeleton	0.001	0.052	CC
GO:0005743	mitochondrial inner membrane	0.001	0.052	CC
GO:0005840	ribosome	0.001	0.052	CC
GO:0099524	postsynaptic cytosol	0.001	0.058	CC
GO:0048786	presynaptic active zone	0.002	0.072	CC
GO:0099092	postsynaptic density, intracellular comp...	0.002	0.077	CC
GO:0072487	MSL complex	0.002	0.077	CC
GO:0030426	growth cone	0.002	0.078	CC
GO:0030424	axon	0.002	0.088	CC
GO:0022626	cytosolic ribosome	0.003	0.108	CC
GO:0030427	site of polarized growth	0.003	0.110	CC
GO:0071944	cell periphery	0.003	0.111	CC
GO:0098685	Schaffer collateral - CA1 synapse	0.003	0.113	CC
GO:0015934	large ribosomal subunit	0.003	0.134	CC
GO:0099568	cytoplasmic region	0.004	0.134	CC
GO:0005886	plasma membrane	0.004	0.141	CC
GO:1902562	H4 histone acetyltransferase complex	0.004	0.144	CC
GO:0033177	proton-transporting two-sector ATPase co...	0.004	0.144	CC
GO:0098590	plasma membrane region	0.005	0.170	CC
GO:0044425	membrane part	0.005	0.175	CC
GO:0043198	dendritic shaft	0.005	0.175	CC
GO:0043025	neuronal cell body	0.005	0.180	CC
GO:0015935	small ribosomal subunit	0.006	0.188	CC
GO:0098999	extrinsic component of postsynaptic endo...	0.006	0.188	CC
GO:0099631	postsynaptic endocytic zone cytoplasmic ...	0.006	0.188	CC
GO:0032420	stereocilium	0.006	0.201	CC
GO:0031594	neuromuscular junction	0.007	0.203	CC
GO:0000276	mitochondrial proton-transporting ATP sy...	0.007	0.224	CC
GO:0044455	mitochondrial membrane part	0.008	0.238	CC
GO:0044305	calyx of Held	0.008	0.238	CC
GO:0099522	region of cytosol	0.008	0.238	CC
GO:0031967	organelle envelope	0.009	0.252	CC
GO:0031975	envelope	0.009	0.252	CC
GO:0005747	mitochondrial respiratory chain complex ...	0.011	0.301	CC
GO:0030964	NADH dehydrogenase complex	0.011	0.301	CC
GO:0032421	stereocilium bundle	0.011	0.301	CC
GO:0045271	respiratory chain complex I	0.011	0.301	CC
GO:0022627	cytosolic small ribosomal subunit	0.011	0.304	CC
GO:0000137	Golgi cis cisterna	0.012	0.321	CC

GO:0008091	spectrin	0.012	0.321	CC
GO:0060076	excitatory synapse	0.013	0.325	CC
GO:0022625	cytosolic large ribosomal subunit	0.014	0.340	CC
GO:0045263	proton-transporting ATP synthase complex...	0.014	0.345	CC
GO:0044459	plasma membrane part	0.014	0.345	CC
GO:0045211	postsynaptic membrane	0.014	0.346	CC
GO:0098803	respiratory chain complex	0.015	0.365	CC
GO:0030672	synaptic vesicle membrane	0.019	0.424	CC
GO:0099501	exocytic vesicle membrane	0.019	0.424	CC
GO:0005844	polysome	0.019	0.424	CC
GO:0000439	transcription factor TFIIH core complex	0.019	0.424	CC
GO:0032437	cuticular plate	0.019	0.424	CC
GO:0005911	cell-cell junction	0.021	0.477	CC
GO:0005746	mitochondrial respiratory chain	0.022	0.483	CC
GO:0031090	organelle membrane	0.022	0.483	CC
GO:0031966	mitochondrial membrane	0.022	0.483	CC
GO:0070469	respiratory chain	0.023	0.490	CC
GO:0098562	cytoplasmic side of membrane	0.023	0.492	CC
GO:0030864	cortical actin cytoskeleton	0.024	0.502	CC
GO:0061617	MICOS complex	0.027	0.550	CC
GO:0044306	neuron projection terminus	0.031	0.631	CC
GO:0098862	cluster of actin-based cell projections	0.031	0.631	CC
GO:0089701	U2AF	0.032	0.631	CC
GO:0098895	postsynaptic endosome membrane	0.032	0.631	CC
GO:0032580	Golgi cisterna membrane	0.032	0.636	CC
GO:0022624	proteasome accessory complex	0.033	0.639	CC
GO:0009898	cytoplasmic side of plasma membrane	0.035	0.677	CC
GO:0044224	juxtaparanode region of axon	0.036	0.685	CC
GO:0034703	cation channel complex	0.037	0.692	CC
GO:0005635	nuclear envelope	0.038	0.715	CC
GO:0032154	cleavage furrow	0.039	0.715	CC
GO:0044445	cytosolic part	0.039	0.715	CC
GO:0008021	synaptic vesicle	0.039	0.715	CC
GO:0000159	protein phosphatase type 2A complex	0.044	0.782	CC
GO:0031234	extrinsic component of cytoplasmic side ...	0.045	0.782	CC
GO:0031985	Golgi cisterna	0.045	0.782	CC
GO:0005795	Golgi stack	0.045	0.782	CC
GO:0005952	cAMP-dependent protein kinase complex	0.047	0.782	CC
GO:0000123	histone acetyltransferase complex	0.048	0.782	CC
GO:0031248	protein acetyltransferase complex	0.048	0.782	CC
GO:1902493	acetyltransferase complex	0.048	0.782	CC
GO:0032153	cell division site	0.050	0.782	CC

vmPFC

GO.ID	Term	FET	BH	Category
GO:1904869	regulation of protein localization to Ca...	0.000	0.085	BP
GO:1904871	positive regulation of protein localizat...	0.000	0.085	BP
GO:1903405	protein localization to nuclear body	0.000	0.085	BP
GO:1904851	positive regulation of establishment of ...	0.000	0.085	BP
GO:1904867	protein localization to Cajal body	0.000	0.085	BP
GO:0070203	regulation of establishment of protein l...	0.000	0.125	BP
GO:0070202	regulation of establishment of protein l...	0.000	0.137	BP
GO:1904816	positive regulation of protein localizat...	0.000	0.137	BP
GO:1990173	protein localization to nucleoplasm	0.000	0.137	BP
GO:1904814	regulation of protein localization to ch...	0.000	0.274	BP
GO:0046425	regulation of JAK-STAT cascade	0.000	0.274	BP
GO:0002250	adaptive immune response	0.000	0.274	BP
GO:0070200	establishment of protein localization to...	0.000	0.302	BP
GO:1904874	positive regulation of telomerase RNA lo...	0.000	0.302	BP
GO:1904892	regulation of STAT cascade	0.000	0.341	BP
GO:0006029	proteoglycan metabolic process	0.000	0.341	BP
GO:0046427	positive regulation of JAK-STAT cascade	0.000	0.341	BP
GO:0036293	response to decreased oxygen levels	0.000	0.348	BP
GO:1904894	positive regulation of STAT cascade	0.000	0.373	BP
GO:0070482	response to oxygen levels	0.001	0.373	BP
GO:0001666	response to hypoxia	0.001	0.373	BP
GO:0006024	glycosaminoglycan biosynthetic process	0.001	0.373	BP
GO:0030203	glycosaminoglycan metabolic process	0.001	0.373	BP
GO:0006023	aminoglycan biosynthetic process	0.001	0.373	BP
GO:0002501	peptide antigen assembly with MHC protei...	0.001	0.373	BP
GO:2000508	regulation of dendritic cell chemotaxis	0.001	0.373	BP
GO:2000510	positive regulation of dendritic cell ch...	0.001	0.373	BP
GO:1904872	regulation of telomerase RNA localizatio...	0.001	0.373	BP
GO:1901998	toxin transport	0.001	0.373	BP
GO:0006457	protein folding	0.001	0.373	BP
GO:0051956	negative regulation of amino acid transp...	0.001	0.373	BP
GO:2000484	positive regulation of interleukin-8 sec...	0.001	0.373	BP
GO:0033044	regulation of chromosome organization	0.001	0.373	BP
GO:0090670	RNA localization to Cajal body	0.001	0.373	BP
GO:0090671	telomerase RNA localization to Cajal bod...	0.001	0.373	BP
GO:0090672	telomerase RNA localization	0.001	0.373	BP
GO:0090685	RNA localization to nucleus	0.001	0.373	BP
GO:0006412	translation	0.001	0.373	BP
GO:0006022	aminoglycan metabolic process	0.001	0.373	BP
GO:0007157	heterophilic cell-cell adhesion via plas...	0.001	0.405	BP
GO:0007566	embryo implantation	0.001	0.405	BP
GO:0098815	modulation of excitatory postsynaptic po...	0.001	0.459	BP
GO:0099068	postsynapse assembly	0.001	0.463	BP
GO:0002396	MHC protein complex assembly	0.001	0.463	BP
GO:1900454	positive regulation of long-term synapti...	0.001	0.463	BP
GO:0007259	JAK-STAT cascade	0.001	0.463	BP
GO:0097106	postsynaptic density organization	0.002	0.533	BP
GO:0006518	peptide metabolic process	0.002	0.540	BP
GO:0043043	peptide biosynthetic process	0.002	0.540	BP
GO:1903510	mucopolysaccharide metabolic process	0.002	0.540	BP

GO:0048852	diencephalon morphogenesis	0.002	0.540	BP
GO:0097696	STAT cascade	0.002	0.614	BP
GO:0032212	positive regulation of telomere maintena...	0.002	0.649	BP
GO:0099084	postsynaptic specialization organization	0.002	0.649	BP
GO:0070199	establishment of protein localization to...	0.002	0.655	BP
GO:1905809	negative regulation of synapse organizat...	0.003	0.780	BP
GO:0015837	amine transport	0.003	0.780	BP
GO:0034975	protein folding in endoplasmic reticulum	0.003	0.780	BP
GO:0002688	regulation of leukocyte chemotaxis	0.003	0.836	BP
GO:0030968	endoplasmic reticulum unfolded protein r...	0.003	0.838	BP
GO:1904861	excitatory synapse assembly	0.003	0.865	BP
GO:0072201	negative regulation of mesenchymal cell ...	0.004	0.865	BP
GO:1901658	glycosyl compound catabolic process	0.004	0.865	BP
GO:1904358	positive regulation of telomere maintena...	0.004	0.865	BP
GO:0032206	positive regulation of telomere maintena...	0.004	0.865	BP
GO:0098742	cell-cell adhesion via plasma-membrane a...	0.004	0.865	BP
GO:0002407	dendritic cell chemotaxis	0.004	0.865	BP
GO:0030595	leukocyte chemotaxis	0.004	0.865	BP
GO:0007218	neuropeptide signaling pathway	0.004	0.865	BP
GO:0019882	antigen processing and presentation	0.004	0.865	BP
GO:0034620	cellular response to unfolded protein	0.004	0.865	BP
GO:0032543	mitochondrial translation	0.004	0.865	BP
GO:0070131	positive regulation of mitochondrial tra...	0.005	0.865	BP
GO:0070198	protein localization to chromosome, telo...	0.005	0.865	BP
GO:0110111	negative regulation of animal organ morp...	0.005	0.865	BP
GO:0051235	maintenance of location	0.005	0.865	BP
GO:0002862	negative regulation of inflammatory resp...	0.005	0.865	BP
GO:0042635	positive regulation of hair cycle	0.005	0.865	BP
GO:0045006	DNA deamination	0.005	0.865	BP
GO:0051798	positive regulation of hair follicle dev...	0.005	0.865	BP
GO:0070391	response to lipoteichoic acid	0.005	0.865	BP
GO:0071223	cellular response to lipoteichoic acid	0.005	0.865	BP
GO:0002399	MHC class II protein complex assembly	0.005	0.865	BP
GO:0002503	peptide antigen assembly with MHC class ...	0.005	0.865	BP
GO:0002767	immune response-inhibiting cell surface ...	0.005	0.865	BP
GO:0002774	Fc receptor mediated inhibitory signalin...	0.005	0.865	BP
GO:0003408	optic cup formation involved in camera-t...	0.005	0.865	BP
GO:0007181	transforming growth factor beta receptor...	0.005	0.865	BP
GO:0038123	toll-like receptor TLR1:TLR2 signaling p...	0.005	0.865	BP
GO:0071725	response to triacyl bacterial lipopeptid...	0.005	0.865	BP
GO:0071727	cellular response to triacyl bacterial l...	0.005	0.865	BP
GO:2000670	positive regulation of dendritic cell ap...	0.005	0.865	BP
GO:0060079	excitatory postsynaptic potential	0.005	0.865	BP
GO:0045471	response to ethanol	0.005	0.873	BP
GO:0048002	antigen processing and presentation of p...	0.006	0.884	BP
GO:0002690	positive regulation of leukocyte chemota...	0.006	0.884	BP
GO:0009164	nucleoside catabolic process	0.006	0.884	BP
GO:2000463	positive regulation of excitatory postsy...	0.006	0.884	BP
GO:0032210	regulation of telomere maintenance via t...	0.006	0.884	BP
GO:0010528	regulation of transposition	0.006	0.884	BP
GO:0010529	negative regulation of transposition	0.006	0.884	BP
GO:0032891	negative regulation of organic acid tran...	0.006	0.884	BP

GO:0036336	dendritic cell migration	0.006	0.884	BP
GO:0046135	pyrimidine nucleoside catabolic process	0.006	0.884	BP
GO:0099175	regulation of postsynapse organization	0.006	0.889	BP
GO:0050680	negative regulation of epithelial cell p...	0.006	0.889	BP
GO:2000573	positive regulation of DNA biosynthetic ...	0.006	0.900	BP
GO:0051952	regulation of amine transport	0.006	0.900	BP
GO:0051955	regulation of amino acid transport	0.007	0.923	BP
GO:0002478	antigen processing and presentation of e...	0.007	0.923	BP
GO:0014009	glial cell proliferation	0.007	0.923	BP
GO:0032655	regulation of interleukin-12 production	0.007	0.923	BP
GO:0071453	cellular response to oxygen levels	0.007	0.923	BP
GO:0002887	negative regulation of myeloid leukocyte...	0.007	0.923	BP
GO:0061450	trophoblast cell migration	0.007	0.923	BP
GO:1901163	regulation of trophoblast cell migration	0.007	0.923	BP
GO:1903789	regulation of amino acid transmembrane t...	0.007	0.923	BP
GO:2000615	regulation of histone H3-K9 acetylation	0.007	0.923	BP
GO:2001198	regulation of dendritic cell differentia...	0.007	0.923	BP
GO:0099173	postsynapse organization	0.007	0.923	BP
GO:0042745	circadian sleep/wake cycle	0.007	0.923	BP
GO:2000482	regulation of interleukin-8 secretion	0.007	0.923	BP
GO:0043604	amide biosynthetic process	0.007	0.933	BP
GO:1901224	positive regulation of NIK/NF-kappaB sig...	0.007	0.933	BP
GO:0002687	positive regulation of leukocyte migrati...	0.007	0.933	BP
GO:0002437	inflammatory response to antigenic stimu...	0.008	0.941	BP
GO:0032259	methylation	0.008	0.941	BP
GO:0032615	interleukin-12 production	0.008	0.941	BP
GO:0036294	cellular response to decreased oxygen le...	0.008	0.941	BP
GO:0019884	antigen processing and presentation of e...	0.008	0.950	BP
GO:0000271	polysaccharide biosynthetic process	0.008	0.965	BP
GO:0140053	mitochondrial gene expression	0.008	0.980	BP
GO:0009100	glycoprotein metabolic process	0.009	0.980	BP
GO:0099565	chemical synaptic transmission, postsyna...	0.009	0.980	BP
GO:0060389	pathway-restricted SMAD protein phosphor...	0.009	0.980	BP
GO:0043414	macromolecule methylation	0.009	0.980	BP
GO:0031061	negative regulation of histone methylati...	0.009	0.980	BP
GO:0098698	postsynaptic specialization assembly	0.009	0.980	BP
GO:1902473	regulation of protein localization to sy...	0.009	0.980	BP
GO:0019538	protein metabolic process	0.009	0.996	BP
GO:0009313	oligosaccharide catabolic process	0.009	1.000	BP
GO:0060394	negative regulation of pathway-restricte...	0.009	1.000	BP
GO:0014074	response to purine-containing compound	0.010	1.000	BP
GO:0031056	regulation of histone modification	0.010	1.000	BP
GO:0043603	cellular amide metabolic process	0.010	1.000	BP
GO:0030167	proteoglycan catabolic process	0.010	1.000	BP
GO:0038124	toll-like receptor TLR6:TLR2 signaling p...	0.010	1.000	BP
GO:0048818	positive regulation of hair follicle mat...	0.010	1.000	BP
GO:0071724	response to diacyl bacterial lipopeptide	0.010	1.000	BP
GO:0071726	cellular response to diacyl bacterial li...	0.010	1.000	BP
GO:0097167	circadian regulation of translation	0.010	1.000	BP
GO:0042339	keratan sulfate metabolic process	0.010	1.000	BP
GO:0071312	cellular response to alkaloid	0.010	1.000	BP
GO:0032196	transposition	0.010	1.000	BP

GO:1904356	regulation of telomere maintenance via t...	0.010	1.000	BP
GO:0030326	embryonic limb morphogenesis	0.010	1.000	BP
GO:0035113	embryonic appendage morphogenesis	0.010	1.000	BP
GO:0043933	protein-containing complex subunit organ...	0.011	1.000	BP
GO:0006986	response to unfolded protein	0.011	1.000	BP
GO:0071456	cellular response to hypoxia	0.011	1.000	BP
GO:0007588	excretion	0.011	1.000	BP
GO:0061351	neural precursor cell proliferation	0.011	1.000	BP
GO:0007292	female gamete generation	0.011	1.000	BP
GO:0019886	antigen processing and presentation of e...	0.011	1.000	BP
GO:0098609	cell-cell adhesion	0.012	1.000	BP
GO:0002685	regulation of leukocyte migration	0.012	1.000	BP
GO:0034622	cellular protein-containing complex asse...	0.012	1.000	BP
GO:0006415	translational termination	0.012	1.000	BP
GO:2000278	regulation of DNA biosynthetic process	0.012	1.000	BP
GO:0007171	activation of transmembrane receptor pro...	0.012	1.000	BP
GO:0043301	negative regulation of leukocyte degranu...	0.012	1.000	BP
GO:0043970	histone H3-K9 acetylation	0.012	1.000	BP
GO:1903894	regulation of IRE1-mediated unfolded pro...	0.012	1.000	BP
GO:0062009	secondary palate development	0.012	1.000	BP
GO:0072606	interleukin-8 secretion	0.012	1.000	BP
GO:0035967	cellular response to topologically incor...	0.013	1.000	BP
GO:0002286	T cell activation involved in immune res...	0.013	1.000	BP
GO:0044728	DNA methylation or demethylation	0.013	1.000	BP
GO:1901566	organonitrogen compound biosynthetic pro...	0.013	1.000	BP
GO:0048534	hematopoietic or lymphoid organ developm...	0.013	1.000	BP
GO:0009059	macromolecule biosynthetic process	0.013	1.000	BP
GO:0002495	antigen processing and presentation of p...	0.014	1.000	BP
GO:0043170	macromolecule metabolic process	0.014	1.000	BP
GO:0048477	oogenesis	0.014	1.000	BP
GO:0046640	regulation of alpha-beta T cell prolifer...	0.014	1.000	BP
GO:0051953	negative regulation of amine transport	0.014	1.000	BP
GO:2000178	negative regulation of neural precursor ...	0.014	1.000	BP
GO:0002088	lens development in camera-type eye	0.015	1.000	BP
GO:0036498	IRE1-mediated unfolded protein response	0.015	1.000	BP
GO:0002504	antigen processing and presentation of p...	0.015	1.000	BP
GO:0032204	regulation of telomere maintenance	0.015	1.000	BP
GO:0008285	negative regulation of cell proliferatio...	0.015	1.000	BP
GO:0007350	blastoderm segmentation	0.015	1.000	BP
GO:0030097	hemopoiesis	0.016	1.000	BP
GO:0009798	axis specification	0.016	1.000	BP
GO:0002765	immune response-inhibiting signal transd...	0.016	1.000	BP
GO:0002865	negative regulation of acute inflammator...	0.016	1.000	BP
GO:0003310	pancreatic A cell differentiation	0.016	1.000	BP
GO:0006689	ganglioside catabolic process	0.016	1.000	BP
GO:0007386	compartment pattern specification	0.016	1.000	BP
GO:0014050	negative regulation of glutamate secreti...	0.016	1.000	BP
GO:0021796	cerebral cortex regionalization	0.016	1.000	BP
GO:0032229	negative regulation of synaptic transmis...	0.016	1.000	BP
GO:0033152	immunoglobulin V(D)J recombination	0.016	1.000	BP
GO:0045627	positive regulation of T-helper 1 cell d...	0.016	1.000	BP
GO:0052150	modulation by symbiont of host apoptotic...	0.016	1.000	BP

GO:0070383	DNA cytosine deamination	0.016	1.000	BP
GO:0071440	regulation of histone H3-K14 acetylation	0.016	1.000	BP
GO:0090009	primitive streak formation	0.016	1.000	BP
GO:1901164	negative regulation of trophoblast cell ...	0.016	1.000	BP
GO:1901552	positive regulation of endothelial cell ...	0.016	1.000	BP
GO:1901628	positive regulation of postsynaptic memb...	0.016	1.000	BP
GO:1903142	positive regulation of establishment of ...	0.016	1.000	BP
GO:0030201	heparan sulfate proteoglycan metabolic p...	0.016	1.000	BP
GO:0007339	binding of sperm to zona pellucida	0.017	1.000	BP
GO:0043372	positive regulation of CD4-positive, alp...	0.017	1.000	BP
GO:0050718	positive regulation of interleukin-1 bet...	0.017	1.000	BP
GO:1903792	negative regulation of anion transport	0.017	1.000	BP
GO:0006954	inflammatory response	0.017	1.000	BP
GO:0055065	metal ion homeostasis	0.018	1.000	BP
GO:0042509	regulation of tyrosine phosphorylation o...	0.018	1.000	BP
GO:0051568	histone H3-K4 methylation	0.018	1.000	BP
GO:0050803	regulation of synapse structure or activ...	0.018	1.000	BP
GO:1903706	regulation of hemopoiesis	0.018	1.000	BP
GO:0055074	calcium ion homeostasis	0.018	1.000	BP
GO:2001252	positive regulation of chromosome organi...	0.018	1.000	BP
GO:0006304	DNA modification	0.018	1.000	BP
GO:1901135	carbohydrate derivative metabolic proces...	0.018	1.000	BP
GO:2001023	regulation of response to drug	0.019	1.000	BP
GO:0043271	negative regulation of ion transport	0.019	1.000	BP
GO:0001911	negative regulation of leukocyte mediate...	0.019	1.000	BP
GO:0002827	positive regulation of T-helper 1 type i...	0.019	1.000	BP
GO:0030213	hyaluronan biosynthetic process	0.019	1.000	BP
GO:0034134	toll-like receptor 2 signaling pathway	0.019	1.000	BP
GO:0046641	positive regulation of alpha-beta T cell...	0.019	1.000	BP
GO:1900452	regulation of long-term synaptic depress...	0.019	1.000	BP
GO:0010575	positive regulation of vascular endothel...	0.019	1.000	BP
GO:0032735	positive regulation of interleukin-12 pr...	0.019	1.000	BP
GO:0040020	regulation of meiotic nuclear division	0.019	1.000	BP
GO:0070129	regulation of mitochondrial translation	0.019	1.000	BP
GO:0003407	neural retina development	0.019	1.000	BP
GO:0034645	cellular macromolecule biosynthetic proc...	0.019	1.000	BP
GO:0042254	ribosome biogenesis	0.020	1.000	BP
GO:0033138	positive regulation of peptidyl-serine p...	0.020	1.000	BP
GO:0034976	response to endoplasmic reticulum stress	0.020	1.000	BP
GO:0016570	histone modification	0.020	1.000	BP
GO:0007004	telomere maintenance via telomerase	0.020	1.000	BP
GO:0032760	positive regulation of tumor necrosis fa...	0.020	1.000	BP
GO:0070231	T cell apoptotic process	0.020	1.000	BP
GO:0007260	tyrosine phosphorylation of STAT protein	0.021	1.000	BP
GO:0071897	DNA biosynthetic process	0.021	1.000	BP
GO:0060291	long-term synaptic potentiation	0.021	1.000	BP
GO:0030166	proteoglycan biosynthetic process	0.022	1.000	BP
GO:0043090	amino acid import	0.022	1.000	BP
GO:0046633	alpha-beta T cell proliferation	0.022	1.000	BP
GO:0050716	positive regulation of interleukin-1 sec...	0.022	1.000	BP
GO:0071560	cellular response to transforming growth...	0.022	1.000	BP
GO:0002520	immune system development	0.022	1.000	BP

GO:0001941	postsynaptic membrane organization	0.023	1.000	BP
GO:0002438	acute inflammatory response to antigenic...	0.023	1.000	BP
GO:0009713	catechol-containing compound biosyntheti...	0.023	1.000	BP
GO:0031342	negative regulation of cell killing	0.023	1.000	BP
GO:0042423	catecholamine biosynthetic process	0.023	1.000	BP
GO:0042749	regulation of circadian sleep/wake cycle	0.023	1.000	BP
GO:0042921	glucocorticoid receptor signaling pathwa...	0.023	1.000	BP
GO:1904469	positive regulation of tumor necrosis fa...	0.023	1.000	BP
GO:1905874	regulation of postsynaptic density organ...	0.023	1.000	BP
GO:2000738	positive regulation of stem cell differe...	0.023	1.000	BP
GO:0060393	regulation of pathway-restricted SMAD pr...	0.023	1.000	BP
GO:0005976	polysaccharide metabolic process	0.023	1.000	BP
GO:0006278	RNA-dependent DNA biosynthetic process	0.024	1.000	BP
GO:0050805	negative regulation of synaptic transmis...	0.024	1.000	BP
GO:1903557	positive regulation of tumor necrosis fa...	0.024	1.000	BP
GO:0002072	optic cup morphogenesis involved in came...	0.024	1.000	BP
GO:0006216	cytidine catabolic process	0.024	1.000	BP
GO:0006685	sphingomyelin catabolic process	0.024	1.000	BP
GO:0009972	cytidine deamination	0.024	1.000	BP
GO:0015015	heparan sulfate proteoglycan biosyntheti...	0.024	1.000	BP
GO:0030311	poly-N-acetyllactosamine biosynthetic pr...	0.024	1.000	BP
GO:0035989	tendon development	0.024	1.000	BP
GO:0044341	sodium-dependent phosphate transport	0.024	1.000	BP
GO:0044531	modulation of programmed cell death in o...	0.024	1.000	BP
GO:0044532	modulation of apoptotic process in other...	0.024	1.000	BP
GO:0046087	cytidine metabolic process	0.024	1.000	BP
GO:0052040	modulation by symbiont of host programme...	0.024	1.000	BP
GO:0052248	modulation of programmed cell death in o...	0.024	1.000	BP
GO:0052433	modulation by organism of apoptotic proc...	0.024	1.000	BP
GO:0070339	response to bacterial lipopeptide	0.024	1.000	BP
GO:0071220	cellular response to bacterial lipoprote...	0.024	1.000	BP
GO:0071221	cellular response to bacterial lipopepti...	0.024	1.000	BP
GO:1903911	positive regulation of receptor clusteri...	0.024	1.000	BP
GO:2001251	negative regulation of chromosome organi...	0.024	1.000	BP
GO:1904029	regulation of cyclin-dependent protein k...	0.025	1.000	BP
GO:0042733	embryonic digit morphogenesis	0.025	1.000	BP
GO:0018146	keratan sulfate biosynthetic process	0.025	1.000	BP
GO:0030149	sphingolipid catabolic process	0.025	1.000	BP
GO:0048247	lymphocyte chemotaxis	0.025	1.000	BP
GO:0070232	regulation of T cell apoptotic process	0.025	1.000	BP
GO:0050807	regulation of synapse organization	0.025	1.000	BP
GO:0002366	leukocyte activation involved in immune ...	0.025	1.000	BP
GO:0051897	positive regulation of protein kinase B ...	0.025	1.000	BP
GO:0045785	positive regulation of cell adhesion	0.026	1.000	BP
GO:1902275	regulation of chromatin organization	0.026	1.000	BP
GO:0046683	response to organophosphorus	0.027	1.000	BP
GO:0002285	lymphocyte activation involved in immune...	0.027	1.000	BP
GO:0071559	response to transforming growth factor b...	0.027	1.000	BP
GO:0070227	lymphocyte apoptotic process	0.027	1.000	BP
GO:1905268	negative regulation of chromatin organiz...	0.027	1.000	BP
GO:0071621	granulocyte chemotaxis	0.027	1.000	BP
GO:0002507	tolerance induction	0.027	1.000	BP

GO:0031000	response to caffeine	0.027	1.000	BP
GO:0031958	corticosteroid receptor signaling pathwa...	0.027	1.000	BP
GO:0034587	piRNA metabolic process	0.027	1.000	BP
GO:0036270	response to diuretic	0.027	1.000	BP
GO:0043046	DNA methylation involved in gamete gener...	0.027	1.000	BP
GO:0051797	regulation of hair follicle development	0.027	1.000	BP
GO:2000736	regulation of stem cell differentiation	0.027	1.000	BP
GO:0031057	negative regulation of histone modificat...	0.028	1.000	BP
GO:0042531	positive regulation of tyrosine phosphor...	0.028	1.000	BP
GO:0099054	presynapse assembly	0.028	1.000	BP
GO:0002263	cell activation involved in immune respo...	0.028	1.000	BP
GO:0016569	covalent chromatin modification	0.028	1.000	BP
GO:0006890	retrograde vesicle-mediated transport, G...	0.028	1.000	BP
GO:1901136	carbohydrate derivative catabolic proces...	0.028	1.000	BP
GO:0072507	divalent inorganic cation homeostasis	0.028	1.000	BP
GO:0010574	regulation of vascular endothelial growt...	0.028	1.000	BP
GO:0010800	positive regulation of peptidyl-threonin...	0.028	1.000	BP
GO:0035966	response to topologically incorrect prot...	0.028	1.000	BP
GO:0030521	androgen receptor signaling pathway	0.029	1.000	BP
GO:0060135	maternal process involved in female preg...	0.029	1.000	BP
GO:0035249	synaptic transmission, glutamatergic	0.029	1.000	BP
GO:0018022	peptidyl-lysine methylation	0.029	1.000	BP
GO:0032680	regulation of tumor necrosis factor prod...	0.029	1.000	BP
GO:0044260	cellular macromolecule metabolic process	0.029	1.000	BP
GO:0051445	regulation of meiotic cell cycle	0.030	1.000	BP
GO:0070228	regulation of lymphocyte apoptotic proce...	0.030	1.000	BP
GO:1900271	regulation of long-term synaptic potenti...	0.030	1.000	BP
GO:0060326	cell chemotaxis	0.031	1.000	BP
GO:0032945	negative regulation of mononuclear cell ...	0.032	1.000	BP
GO:0050672	negative regulation of lymphocyte prolif...	0.032	1.000	BP
GO:0032228	regulation of synaptic transmission, GAB...	0.032	1.000	BP
GO:0032728	positive regulation of interferon-beta p...	0.032	1.000	BP
GO:2000516	positive regulation of CD4-positive, alp...	0.032	1.000	BP
GO:0050727	regulation of inflammatory response	0.032	1.000	BP
GO:0002578	negative regulation of antigen processin...	0.032	1.000	BP
GO:0016554	cytidine to uridine editing	0.032	1.000	BP
GO:0021684	cerebellar granular layer formation	0.032	1.000	BP
GO:0021707	cerebellar granule cell differentiation	0.032	1.000	BP
GO:0030309	poly-N-acetyllactosamine metabolic proce...	0.032	1.000	BP
GO:0032493	response to bacterial lipoprotein	0.032	1.000	BP
GO:0042756	drinking behavior	0.032	1.000	BP
GO:0043305	negative regulation of mast cell degranu...	0.032	1.000	BP
GO:0044154	histone H3-K14 acetylation	0.032	1.000	BP
GO:0051964	negative regulation of synapse assembly	0.032	1.000	BP
GO:0061101	neuroendocrine cell differentiation	0.032	1.000	BP
GO:0097048	dendritic cell apoptotic process	0.032	1.000	BP
GO:0098700	neurotransmitter loading into synaptic v...	0.032	1.000	BP
GO:1902474	positive regulation of protein localizat...	0.032	1.000	BP
GO:2000668	regulation of dendritic cell apoptotic p...	0.032	1.000	BP
GO:0002313	mature B cell differentiation involved i...	0.032	1.000	BP
GO:0002861	regulation of inflammatory response to a...	0.032	1.000	BP
GO:0022410	circadian sleep/wake cycle process	0.032	1.000	BP

GO:0042454	ribonucleoside catabolic process	0.032	1.000	BP
GO:0045063	T-helper 1 cell differentiation	0.032	1.000	BP
GO:0097107	postsynaptic density assembly	0.032	1.000	BP
GO:0034968	histone lysine methylation	0.032	1.000	BP
GO:0009605	response to external stimulus	0.032	1.000	BP
GO:0006888	ER to Golgi vesicle-mediated transport	0.033	1.000	BP
GO:0016575	histone deacetylation	0.033	1.000	BP
GO:0034656	nucleobase-containing small molecule cat...	0.033	1.000	BP
GO:0070126	mitochondrial translational termination	0.033	1.000	BP
GO:0065003	protein-containing complex assembly	0.033	1.000	BP
GO:0032640	tumor necrosis factor production	0.034	1.000	BP
GO:0035107	appendage morphogenesis	0.034	1.000	BP
GO:0035108	limb morphogenesis	0.034	1.000	BP
GO:1903555	regulation of tumor necrosis factor supe...	0.034	1.000	BP
GO:0002252	immune effector process	0.034	1.000	BP
GO:0050877	nervous system process	0.035	1.000	BP
GO:1902533	positive regulation of intracellular sig...	0.035	1.000	BP
GO:0055080	cation homeostasis	0.035	1.000	BP
GO:0010833	telomere maintenance via telomere length...	0.035	1.000	BP
GO:0010573	vascular endothelial growth factor produ...	0.035	1.000	BP
GO:0046466	membrane lipid catabolic process	0.035	1.000	BP
GO:0048246	macrophage chemotaxis	0.035	1.000	BP
GO:0061756	leukocyte adhesion to vascular endotheli...	0.035	1.000	BP
GO:0031328	positive regulation of cellular biosynth...	0.036	1.000	BP
GO:0002448	mast cell mediated immunity	0.036	1.000	BP
GO:0006213	pyrimidine nucleoside metabolic process	0.036	1.000	BP
GO:0099172	presynapse organization	0.036	1.000	BP
GO:0006305	DNA alkylation	0.037	1.000	BP
GO:0006306	DNA methylation	0.037	1.000	BP
GO:0019915	lipid storage	0.037	1.000	BP
GO:0031646	positive regulation of neurological syst...	0.037	1.000	BP
GO:0071216	cellular response to biotic stimulus	0.037	1.000	BP
GO:0032098	regulation of appetite	0.038	1.000	BP
GO:0045624	positive regulation of T-helper cell dif...	0.038	1.000	BP
GO:0045947	negative regulation of translational ini...	0.038	1.000	BP
GO:0048268	clathrin coat assembly	0.038	1.000	BP
GO:0035265	organ growth	0.039	1.000	BP
GO:0002376	immune system process	0.039	1.000	BP
GO:0006418	tRNA aminoacylation for protein translat...	0.039	1.000	BP
GO:0006984	ER-nucleus signaling pathway	0.039	1.000	BP
GO:0061647	histone H3-K9 modification	0.039	1.000	BP
GO:0015012	heparan sulfate proteoglycan biosyntheti...	0.039	1.000	BP
GO:0060292	long-term synaptic depression	0.039	1.000	BP
GO:0097028	dendritic cell differentiation	0.039	1.000	BP
GO:2000780	negative regulation of double-strand bre...	0.039	1.000	BP
GO:0010811	positive regulation of cell-substrate ad...	0.040	1.000	BP
GO:0050921	positive regulation of chemotaxis	0.040	1.000	BP
GO:0050821	protein stabilization	0.040	1.000	BP
GO:0006874	cellular calcium ion homeostasis	0.040	1.000	BP
GO:0050808	synapse organization	0.041	1.000	BP
GO:0017148	negative regulation of translation	0.041	1.000	BP
GO:0071706	tumor necrosis factor superfamily cytoki...	0.041	1.000	BP

GO:0007186	G protein-coupled receptor signaling pat...	0.041	1.000	BP
GO:0098771	inorganic ion homeostasis	0.042	1.000	BP
GO:0001955	blood vessel maturation	0.042	1.000	BP
GO:0002430	complement receptor mediated signaling p...	0.042	1.000	BP
GO:0010958	regulation of amino acid import across p...	0.042	1.000	BP
GO:0030210	heparin biosynthetic process	0.042	1.000	BP
GO:0030854	positive regulation of granulocyte diffe...	0.042	1.000	BP
GO:0044848	biological phase	0.042	1.000	BP
GO:0044851	hair cycle phase	0.042	1.000	BP
GO:0045625	regulation of T-helper 1 cell differenti...	0.042	1.000	BP
GO:0046133	pyrimidine ribonucleoside catabolic proc...	0.042	1.000	BP
GO:0046533	negative regulation of photoreceptor cel...	0.042	1.000	BP
GO:0048819	regulation of hair follicle maturation	0.042	1.000	BP
GO:0061549	sympathetic ganglion development	0.042	1.000	BP
GO:0097113	AMPA glutamate receptor clustering	0.042	1.000	BP
GO:0097688	glutamate receptor clustering	0.042	1.000	BP
GO:1900125	regulation of hyaluronan biosynthetic pr...	0.042	1.000	BP
GO:0001519	peptide amidation	0.042	1.000	BP
GO:0001797	negative regulation of type IIa hypersen...	0.042	1.000	BP
GO:0001811	negative regulation of type I hypersensi...	0.042	1.000	BP
GO:0001814	negative regulation of antibody-dependen...	0.042	1.000	BP
GO:0001827	inner cell mass cell fate commitment	0.042	1.000	BP
GO:0002298	CD4-positive, CD25-positive, alpha-beta ...	0.042	1.000	BP
GO:0002299	alpha-beta intraepithelial T cell differ...	0.042	1.000	BP
GO:0002300	CD8-positive, alpha-beta intraepithelial...	0.042	1.000	BP
GO:0002436	immune complex clearance by monocytes an...	0.042	1.000	BP
GO:0002439	chronic inflammatory response to antigen...	0.042	1.000	BP
GO:0002450	B cell antigen processing and presentati...	0.042	1.000	BP
GO:0002622	regulation of B cell antigen processing ...	0.042	1.000	BP
GO:0002893	negative regulation of type II hypersens...	0.042	1.000	BP
GO:0003322	pancreatic A cell development	0.042	1.000	BP
GO:0003421	growth plate cartilage axis specificatio...	0.042	1.000	BP
GO:0008049	male courtship behavior	0.042	1.000	BP
GO:0009730	detection of carbohydrate stimulus	0.042	1.000	BP
GO:0009732	detection of hexose stimulus	0.042	1.000	BP
GO:0010085	polarity specification of proximal/dista...	0.042	1.000	BP
GO:0010477	response to sulfur dioxide	0.042	1.000	BP
GO:0010879	cholesterol transport involved in choles...	0.042	1.000	BP
GO:0015774	polysaccharide transport	0.042	1.000	BP
GO:0018032	protein amidation	0.042	1.000	BP
GO:0019510	S-adenosylhomocysteine catabolic process	0.042	1.000	BP
GO:0021905	forebrain-midbrain boundary formation	0.042	1.000	BP
GO:0021917	somatic motor neuron fate commitment	0.042	1.000	BP
GO:0021918	regulation of transcription from RNA pol...	0.042	1.000	BP
GO:0030718	germ-line stem cell population maintenan...	0.042	1.000	BP
GO:0032832	regulation of CD4-positive, CD25-positiv...	0.042	1.000	BP
GO:0032834	positive regulation of CD4-positive, CD2...	0.042	1.000	BP
GO:0033316	meiotic spindle assembly checkpoint	0.042	1.000	BP
GO:0033342	negative regulation of collagen binding	0.042	1.000	BP
GO:0033396	beta-alanine biosynthetic process via 3-...	0.042	1.000	BP
GO:0034263	positive regulation of autophagy in resp...	0.042	1.000	BP
GO:0034287	detection of monosaccharide stimulus	0.042	1.000	BP

GO:0036359	renal potassium excretion	0.042	1.000	BP
GO:0036517	chemoattraction of serotonergic neuron a...	0.042	1.000	BP
GO:0042231	interleukin-13 biosynthetic process	0.042	1.000	BP
GO:0042543	protein N-linked glycosylation via argin...	0.042	1.000	BP
GO:0043131	erythrocyte enucleation	0.042	1.000	BP
GO:0043354	enucleate erythrocyte maturation	0.042	1.000	BP
GO:0044722	renal phosphate excretion	0.042	1.000	BP
GO:0044779	meiotic spindle checkpoint	0.042	1.000	BP
GO:0045361	negative regulation of interleukin-1 bio...	0.042	1.000	BP
GO:0046013	regulation of T cell homeostatic prolife...	0.042	1.000	BP
GO:0046680	response to DDT	0.042	1.000	BP
GO:0048321	axial mesodermal cell differentiation	0.042	1.000	BP
GO:0048322	axial mesodermal cell fate commitment	0.042	1.000	BP
GO:0048327	axial mesodermal cell fate specification	0.042	1.000	BP
GO:0048789	cytoskeletal matrix organization at acti...	0.042	1.000	BP
GO:0048850	hypophysis morphogenesis	0.042	1.000	BP
GO:0050724	negative regulation of interleukin-1 bet...	0.042	1.000	BP
GO:0050894	determination of affect	0.042	1.000	BP
GO:0051086	chaperone mediated protein folding indep...	0.042	1.000	BP
GO:0051594	detection of glucose	0.042	1.000	BP
GO:0051615	histamine uptake	0.042	1.000	BP
GO:0052314	phytoalexin metabolic process	0.042	1.000	BP
GO:0060007	linear vestibuloocular reflex	0.042	1.000	BP
GO:0060067	cervix development	0.042	1.000	BP
GO:0060112	generation of ovulation cycle rhythm	0.042	1.000	BP
GO:0060460	left lung morphogenesis	0.042	1.000	BP
GO:0060802	epiblast cell-extraembryonic ectoderm ce...	0.042	1.000	BP
GO:0060809	mesodermal to mesenchymal transition inv...	0.042	1.000	BP
GO:0061347	planar cell polarity pathway involved in...	0.042	1.000	BP
GO:0061348	planar cell polarity pathway involved in...	0.042	1.000	BP
GO:0061349	planar cell polarity pathway involved in...	0.042	1.000	BP
GO:0061350	planar cell polarity pathway involved in...	0.042	1.000	BP
GO:0061354	planar cell polarity pathway involved in...	0.042	1.000	BP
GO:0061395	positive regulation of transcription fro...	0.042	1.000	BP
GO:0061930	regulation of erythrocyte enucleation	0.042	1.000	BP
GO:0061931	positive regulation of erythrocyte enucl...	0.042	1.000	BP
GO:0070105	positive regulation of interleukin-6-med...	0.042	1.000	BP
GO:0070238	activated T cell autonomous cell death	0.042	1.000	BP
GO:0070239	regulation of activated T cell autonomou...	0.042	1.000	BP
GO:0070241	positive regulation of activated T cell ...	0.042	1.000	BP
GO:0071707	immunoglobulin heavy chain V-D-J recombi...	0.042	1.000	BP
GO:0071877	regulation of adenylate cyclase-inhibiti...	0.042	1.000	BP
GO:0072027	connecting tubule development	0.042	1.000	BP
GO:0072165	anterior mesonephric tubule development	0.042	1.000	BP
GO:0072166	posterior mesonephric tubule development	0.042	1.000	BP
GO:0072167	specification of mesonephric tubule iden...	0.042	1.000	BP
GO:0072168	specification of anterior mesonephric tu...	0.042	1.000	BP
GO:0072169	specification of posterior mesonephric t...	0.042	1.000	BP
GO:0072184	renal vesicle progenitor cell differenti...	0.042	1.000	BP
GO:0072258	metanephric interstitial fibroblast diff...	0.042	1.000	BP
GO:0072259	metanephric interstitial fibroblast deve...	0.042	1.000	BP
GO:0072268	pattern specification involved in metane...	0.042	1.000	BP

GO:0072286	metanephric connecting tubule developmen...	0.042	1.000	BP
GO:0086096	adenylate cyclase-inhibiting adrenergic ...	0.042	1.000	BP
GO:0090010	transforming growth factor beta receptor...	0.042	1.000	BP
GO:0090264	regulation of immune complex clearance b...	0.042	1.000	BP
GO:0090601	enucleation	0.042	1.000	BP
GO:0097115	neurexin clustering involved in presynap...	0.042	1.000	BP
GO:0098958	retrograde axonal transport of mitochond...	0.042	1.000	BP
GO:0099627	neurotransmitter receptor cycle	0.042	1.000	BP
GO:0099630	postsynaptic neurotransmitter receptor c...	0.042	1.000	BP
GO:0140193	regulation of adenylate cyclase-inhibiti...	0.042	1.000	BP
GO:0140194	negative regulation of adenylate cyclase...	0.042	1.000	BP
GO:1901994	negative regulation of meiotic cell cycl...	0.042	1.000	BP
GO:1902103	negative regulation of metaphase/anaphas...	0.042	1.000	BP
GO:1902203	negative regulation of hepatocyte growth...	0.042	1.000	BP
GO:1902838	regulation of nuclear migration along mi...	0.042	1.000	BP
GO:1903096	protein localization to meiotic spindle ...	0.042	1.000	BP
GO:1903402	regulation of renal phosphate excretion	0.042	1.000	BP
GO:1903403	negative regulation of renal phosphate e...	0.042	1.000	BP
GO:1903701	substantia propria of cornea development	0.042	1.000	BP
GO:1904118	regulation of otic vesicle morphogenesis	0.042	1.000	BP
GO:1904120	positive regulation of otic vesicle morp...	0.042	1.000	BP
GO:1904373	response to kainic acid	0.042	1.000	BP
GO:1904389	rod bipolar cell differentiation	0.042	1.000	BP
GO:1904391	response to ciliary neurotrophic factor	0.042	1.000	BP
GO:1904585	response to putrescine	0.042	1.000	BP
GO:1904586	cellular response to putrescine	0.042	1.000	BP
GO:1904719	positive regulation of AMPA glutamate re...	0.042	1.000	BP
GO:1904764	chaperone-mediated autophagy translocati...	0.042	1.000	BP
GO:1904933	regulation of cell proliferation in midb...	0.042	1.000	BP
GO:1904934	negative regulation of cell proliferatio...	0.042	1.000	BP
GO:1904955	planar cell polarity pathway involved in...	0.042	1.000	BP
GO:1905133	negative regulation of meiotic chromosom...	0.042	1.000	BP
GO:1905359	protein localization to meiotic spindle	0.042	1.000	BP
GO:1905518	regulation of presynaptic active zone as...	0.042	1.000	BP
GO:1905520	positive regulation of presynaptic activ...	0.042	1.000	BP
GO:1905608	positive regulation of presynapse assemb...	0.042	1.000	BP
GO:1905642	negative regulation of DNA methylation	0.042	1.000	BP
GO:1905875	negative regulation of postsynaptic dens...	0.042	1.000	BP
GO:1990390	protein K33-linked ubiquitination	0.042	1.000	BP
GO:1990828	hepatocyte dedifferentiation	0.042	1.000	BP
GO:1990832	slow axonal transport	0.042	1.000	BP
GO:2000537	regulation of B cell chemotaxis	0.042	1.000	BP
GO:2000538	positive regulation of B cell chemotaxis	0.042	1.000	BP
GO:2000775	histone H3-S10 phosphorylation involved ...	0.042	1.000	BP
GO:2000795	negative regulation of epithelial cell p...	0.042	1.000	BP
GO:2001112	regulation of cellular response to hepat...	0.042	1.000	BP
GO:2001113	negative regulation of cellular response...	0.042	1.000	BP
GO:2001161	negative regulation of histone H3-K79 me...	0.042	1.000	BP
GO:0035418	protein localization to synapse	0.042	1.000	BP
GO:0070664	negative regulation of leukocyte prolife...	0.042	1.000	BP
GO:0046632	alpha-beta T cell differentiation	0.042	1.000	BP
GO:2001021	negative regulation of response to DNA d...	0.042	1.000	BP

GO:0002287	alpha-beta T cell activation involved in...	0.043	1.000	BP
GO:0002293	alpha-beta T cell differentiation involv...	0.043	1.000	BP
GO:0002294	CD4-positive, alpha-beta T cell differen...	0.043	1.000	BP
GO:0016339	calcium-dependent cell-cell adhesion via...	0.043	1.000	BP
GO:0030865	cortical cytoskeleton organization	0.043	1.000	BP
GO:0008356	asymmetric cell division	0.043	1.000	BP
GO:0046339	diacylglycerol metabolic process	0.043	1.000	BP
GO:2000042	negative regulation of double-strand bre...	0.043	1.000	BP
GO:0006968	cellular defense response	0.044	1.000	BP
GO:0007435	salivary gland morphogenesis	0.044	1.000	BP
GO:0033280	response to vitamin D	0.044	1.000	BP
GO:0045684	positive regulation of epidermis develop...	0.044	1.000	BP
GO:0009952	anterior/posterior pattern specification	0.044	1.000	BP
GO:0051896	regulation of protein kinase B signaling	0.045	1.000	BP
GO:0060078	regulation of postsynaptic membrane pote...	0.045	1.000	BP
GO:0015807	L-amino acid transport	0.045	1.000	BP
GO:0033692	cellular polysaccharide biosynthetic pro...	0.045	1.000	BP
GO:0007565	female pregnancy	0.045	1.000	BP
GO:0048736	appendage development	0.045	1.000	BP
GO:0060173	limb development	0.045	1.000	BP
GO:0001906	cell killing	0.046	1.000	BP
GO:0048593	camera-type eye morphogenesis	0.046	1.000	BP
GO:1901222	regulation of NIK/NF-kappaB signaling	0.046	1.000	BP
GO:0009891	positive regulation of biosynthetic proc...	0.046	1.000	BP
GO:0032648	regulation of interferon-beta production	0.046	1.000	BP
GO:0009987	cellular process	0.046	1.000	BP
GO:2000177	regulation of neural precursor cell prol...	0.048	1.000	BP
GO:0080135	regulation of cellular response to stres...	0.048	1.000	BP
GO:0035036	sperm-egg recognition	0.048	1.000	BP
GO:0038083	peptidyl-tyrosine autophosphorylation	0.048	1.000	BP
GO:0045738	negative regulation of DNA repair	0.048	1.000	BP
GO:0050706	regulation of interleukin-1 beta secreti...	0.048	1.000	BP
GO:0072529	pyrimidine-containing compound catabolic...	0.048	1.000	BP
GO:0022407	regulation of cell-cell adhesion	0.048	1.000	BP
GO:0000079	regulation of cyclin-dependent protein s...	0.048	1.000	BP
GO:0008283	cell proliferation	0.049	1.000	BP
GO:0006259	DNA metabolic process	0.049	1.000	BP
GO:0140013	meiotic nuclear division	0.049	1.000	BP
GO:0002825	regulation of T-helper 1 type immune res...	0.049	1.000	BP
GO:0010226	response to lithium ion	0.049	1.000	BP
GO:0048520	positive regulation of behavior	0.049	1.000	BP
GO:0048596	embryonic camera-type eye morphogenesis	0.049	1.000	BP
GO:0051446	positive regulation of meiotic cell cycl...	0.049	1.000	BP
GO:0071305	cellular response to vitamin D	0.049	1.000	BP
GO:0009880	embryonic pattern specification	0.050	1.000	BP
GO:0009988	cell-cell recognition	0.050	1.000	BP
GO:0043039	tRNA aminoacylation	0.050	1.000	BP
GO:0006413	translational initiation	0.050	1.000	BP
GO:0051082	unfolded protein binding	0.001	1.000	MF
GO:0044183	protein binding involved in protein fold...	0.001	1.000	MF
GO:0042277	peptide binding	0.002	1.000	MF
GO:0004920	interleukin-10 receptor activity	0.002	1.000	MF

GO:0023026	MHC class II protein complex binding	0.002	1.000	MF
GO:0023023	MHC protein complex binding	0.002	1.000	MF
GO:0032051	clathrin light chain binding	0.002	1.000	MF
GO:0008757	S-adenosylmethionine-dependent methyltra...	0.004	1.000	MF
GO:0019864	IgG binding	0.005	1.000	MF
GO:0001601	peptide YY receptor activity	0.005	1.000	MF
GO:0004566	beta-glucuronidase activity	0.005	1.000	MF
GO:0005499	vitamin D binding	0.005	1.000	MF
GO:1904399	heparan sulfate binding	0.005	1.000	MF
GO:0004888	transmembrane signaling receptor activit...	0.005	1.000	MF
GO:0048018	receptor ligand activity	0.006	1.000	MF
GO:0004576	oligosaccharyl transferase activity	0.007	1.000	MF
GO:0003723	RNA binding	0.008	1.000	MF
GO:0008146	sulfotransferase activity	0.008	1.000	MF
GO:0016857	racemase and epimerase activity, acting ...	0.009	1.000	MF
GO:0008168	methyltransferase activity	0.012	1.000	MF
GO:0001846	opsonin binding	0.015	1.000	MF
GO:0016251	RNA polymerase II general transcription ...	0.015	1.000	MF
GO:0033218	amide binding	0.015	1.000	MF
GO:0004767	sphingomyelin phosphodiesterase activity	0.016	1.000	MF
GO:0008532	N-acetyllactosaminide beta-1,3-N-acetylg...	0.016	1.000	MF
GO:0019966	interleukin-1 binding	0.016	1.000	MF
GO:0071723	lipopeptide binding	0.016	1.000	MF
GO:0017112	Rab guanyl-nucleotide exchange factor ac...	0.017	1.000	MF
GO:0016741	transferase activity, transferring one-c...	0.018	1.000	MF
GO:0019955	cytokine binding	0.018	1.000	MF
GO:0019865	immunoglobulin binding	0.018	1.000	MF
GO:0030545	receptor regulator activity	0.019	1.000	MF
GO:0038023	signaling receptor activity	0.021	1.000	MF
GO:0015037	peptide disulfide oxidoreductase activit...	0.022	1.000	MF
GO:0042800	histone methyltransferase activity (H3-K...	0.022	1.000	MF
GO:0001849	complement component C1q binding	0.023	1.000	MF
GO:0003680	AT DNA binding	0.023	1.000	MF
GO:0004126	cytidine deaminase activity	0.023	1.000	MF
GO:0030297	transmembrane receptor protein tyrosine ...	0.023	1.000	MF
GO:0032395	MHC class II receptor activity	0.023	1.000	MF
GO:0005102	signaling receptor binding	0.024	1.000	MF
GO:0060089	molecular transducer activity	0.024	1.000	MF
GO:0030020	extracellular matrix structural constitu...	0.026	1.000	MF
GO:0001848	complement binding	0.026	1.000	MF
GO:0003735	structural constituent of ribosome	0.029	1.000	MF
GO:0004715	non-membrane spanning protein tyrosine k...	0.029	1.000	MF
GO:0016854	racemase and epimerase activity	0.031	1.000	MF
GO:0003747	translation release factor activity	0.031	1.000	MF
GO:0005114	type II transforming growth factor beta ...	0.031	1.000	MF
GO:0008079	translation termination factor activity	0.031	1.000	MF
GO:0008312	7S RNA binding	0.031	1.000	MF
GO:0045545	syndecan binding	0.031	1.000	MF
GO:0089720	caspase binding	0.031	1.000	MF
GO:0005125	cytokine activity	0.033	1.000	MF
GO:0005179	hormone activity	0.035	1.000	MF
GO:0016922	nuclear receptor binding	0.036	1.000	MF

GO:0140223	general transcription initiation factor ...	0.036	1.000	MF
GO:0016782	transferase activity, transferring sulfu...	0.037	1.000	MF
GO:0004983	neuropeptide Y receptor activity	0.041	1.000	MF
GO:0043184	vascular endothelial growth factor recep...	0.041	1.000	MF
GO:0070492	oligosaccharide binding	0.041	1.000	MF
GO:0008170	N-methyltransferase activity	0.041	1.000	MF
GO:0001642	group III metabotropic glutamate recepto...	0.041	1.000	MF
GO:0001850	complement component C3a binding	0.041	1.000	MF
GO:0003837	beta-ureidopropionase activity	0.041	1.000	MF
GO:0003948	N4-(beta-N-acetylglucosaminyl)-L-asparag...	0.041	1.000	MF
GO:0004013	adenosylhomocysteinase activity	0.041	1.000	MF
GO:0004058	aromatic-L-amino-acid decarboxylase acti...	0.041	1.000	MF
GO:0004164	diphthine synthase activity	0.041	1.000	MF
GO:0004423	iduronate-2-sulfatase activity	0.041	1.000	MF
GO:0004490	methylglutaconyl-CoA hydratase activity	0.041	1.000	MF
GO:0004504	peptidylglycine monooxygenase activity	0.041	1.000	MF
GO:0004581	dolichyl-phosphate beta-glucosyltransfer...	0.041	1.000	MF
GO:0004598	peptidylamidoglycolate lyase activity	0.041	1.000	MF
GO:0004616	phosphogluconate dehydrogenase (decarbox...	0.041	1.000	MF
GO:0004632	phosphopantothenate--cysteine ligase act...	0.041	1.000	MF
GO:0004925	prolactin receptor activity	0.041	1.000	MF
GO:0005115	receptor tyrosine kinase-like orphan rec...	0.041	1.000	MF
GO:0008068	extracellularly glutamate-gated chloride...	0.041	1.000	MF
GO:0008403	25-hydroxycholecalciferol-24-hydroxylase...	0.041	1.000	MF
GO:0008803	bis(5'-nucleosyl)-tetraphosphatase (symm...	0.041	1.000	MF
GO:0008955	peptidoglycan glycosyltransferase activi...	0.041	1.000	MF
GO:0016232	HNK-1 sulfotransferase activity	0.041	1.000	MF
GO:0016802	trialkylsulfonium hydrolase activity	0.041	1.000	MF
GO:0019770	IgG receptor activity	0.041	1.000	MF
GO:0019772	low-affinity IgG receptor activity	0.041	1.000	MF
GO:0019964	interferon-gamma binding	0.041	1.000	MF
GO:0019969	interleukin-10 binding	0.041	1.000	MF
GO:0030342	1-alpha,25-dihydroxyvitamin D3 24-hydrox...	0.041	1.000	MF
GO:0031877	somatostatin receptor binding	0.041	1.000	MF
GO:0033798	thyroxine 5-deiodinase activity	0.041	1.000	MF
GO:0036468	L-dopa decarboxylase activity	0.041	1.000	MF
GO:0042356	GDP-4-dehydro-D-rhamnose reductase activ...	0.041	1.000	MF
GO:0042497	triacyl lipopeptide binding	0.041	1.000	MF
GO:0042943	D-amino acid transmembrane transporter a...	0.041	1.000	MF
GO:0042945	D-serine transmembrane transporter activ...	0.041	1.000	MF
GO:0043333	2-octaprenyl-6-methoxy-1,4-benzoquinone ...	0.041	1.000	MF
GO:0043423	3-phosphoinositide-dependent protein kin...	0.041	1.000	MF
GO:0043430	2-decaprenyl-6-methoxy-1,4-benzoquinone ...	0.041	1.000	MF
GO:0045515	interleukin-18 receptor binding	0.041	1.000	MF
GO:0047464	heparosan-N-sulfate-glucuronate 5-epimer...	0.041	1.000	MF
GO:0047918	GDP-mannose 3,5-epimerase activity	0.041	1.000	MF
GO:0050011	itaconyl-CoA hydratase activity	0.041	1.000	MF
GO:0050577	GDP-L-fucose synthase activity	0.041	1.000	MF
GO:0061751	neutral sphingomyelin phosphodiesterase ...	0.041	1.000	MF
GO:0070042	rRNA (uridine-N3-)-methyltransferase act...	0.041	1.000	MF
GO:0070123	transforming growth factor beta receptor...	0.041	1.000	MF
GO:0070524	11-beta-hydroxysteroid dehydrogenase (NA...	0.041	1.000	MF

GO:0070611	histone methyltransferase activity (H3-R...	0.041	1.000	MF
GO:0070612	histone methyltransferase activity (H2A-...	0.041	1.000	MF
GO:0099567	calcium ion binding involved in regulati...	0.041	1.000	MF
GO:0102390	mycophenolic acid acyl-glucuronide ester...	0.041	1.000	MF
GO:0106105	Ala-tRNA(Thr) hydrolase activity	0.041	1.000	MF
GO:0120146	sulfatide binding	0.041	1.000	MF
GO:1990188	euchromatin binding	0.041	1.000	MF
GO:1990833	clathrin-uncoating ATPase activity	0.041	1.000	MF
GO:0004553	hydrolase activity, hydrolyzing O-glycos...	0.042	1.000	MF
GO:0042605	peptide antigen binding	0.048	1.000	MF
GO:0005832	chaperonin-containing T-complex	0.000	0.028	CC
GO:0101031	chaperone complex	0.000	0.129	CC
GO:0034663	endoplasmic reticulum chaperone complex	0.000	0.218	CC
GO:0042613	MHC class II protein complex	0.001	0.397	CC
GO:0000812	Swr1 complex	0.001	0.443	CC
GO:0002199	zona pellucida receptor complex	0.001	0.443	CC
GO:0016021	integral component of membrane	0.003	0.648	CC
GO:0043202	lysosomal lumen	0.004	0.648	CC
GO:0005775	vacuolar lumen	0.004	0.648	CC
GO:0060076	excitatory synapse	0.005	0.648	CC
GO:0033018	sarcoplasmic reticulum lumen	0.005	0.648	CC
GO:0000942	condensed nuclear chromosome outer kinet...	0.005	0.648	CC
GO:0005594	collagen type IX trimer	0.005	0.648	CC
GO:1990923	PET complex	0.005	0.648	CC
GO:0042611	MHC protein complex	0.006	0.648	CC
GO:0043186	P granule	0.006	0.648	CC
GO:0045495	pole plasm	0.006	0.648	CC
GO:0060293	germ plasm	0.006	0.648	CC
GO:0031224	intrinsic component of membrane	0.006	0.673	CC
GO:0017146	NMDA selective glutamate receptor comple...	0.007	0.704	CC
GO:0062023	collagen-containing extracellular matrix	0.009	0.872	CC
GO:0032588	trans-Golgi network membrane	0.011	0.946	CC
GO:0005788	endoplasmic reticulum lumen	0.011	0.946	CC
GO:0008250	oligosaccharyltransferase complex	0.012	1.000	CC
GO:0071556	integral component of lumenal side of en...	0.014	1.000	CC
GO:0098553	lumenal side of endoplasmic reticulum me...	0.014	1.000	CC
GO:0031012	extracellular matrix	0.015	1.000	CC
GO:0032580	Golgi cisterna membrane	0.015	1.000	CC
GO:0005794	Golgi apparatus	0.016	1.000	CC
GO:0097346	INO80-type complex	0.019	1.000	CC
GO:0030134	COPII-coated ER to Golgi transport vesic...	0.020	1.000	CC
GO:0098552	side of membrane	0.021	1.000	CC
GO:0044391	ribosomal subunit	0.022	1.000	CC
GO:0005593	FACIT collagen trimer	0.024	1.000	CC
GO:0042824	MHC class I peptide loading complex	0.024	1.000	CC
GO:0044300	cerebellar mossy fiber	0.024	1.000	CC
GO:0071546	pi-body	0.024	1.000	CC
GO:0098985	asymmetric, glutamatergic, excitatory sy...	0.024	1.000	CC
GO:0098992	neuronal dense core vesicle	0.024	1.000	CC
GO:0015935	small ribosomal subunit	0.025	1.000	CC
GO:0031985	Golgi cisterna	0.026	1.000	CC
GO:0005852	eukaryotic translation initiation factor...	0.027	1.000	CC

GO:0022627	cytosolic small ribosomal subunit	0.028	1.000	CC
GO:0044445	cytosolic part	0.028	1.000	CC
GO:0012505	endomembrane system	0.028	1.000	CC
GO:0030176	integral component of endoplasmic reticu...	0.029	1.000	CC
GO:0060205	cytoplasmic vesicle lumen	0.031	1.000	CC
GO:0031983	vesicle lumen	0.032	1.000	CC
GO:0034719	SMN-Sm protein complex	0.032	1.000	CC
GO:0001772	immunological synapse	0.035	1.000	CC
GO:0031045	dense core granule	0.038	1.000	CC
GO:0005581	collagen trimer	0.040	1.000	CC
GO:0044431	Golgi apparatus part	0.041	1.000	CC
GO:0000439	transcription factor TFIIH core complex	0.042	1.000	CC
GO:0099026	anchored component of presynaptic membra...	0.042	1.000	CC
GO:0031227	intrinsic component of endoplasmic retic...	0.042	1.000	CC
GO:0014801	longitudinal sarcoplasmic reticulum	0.042	1.000	CC
GO:0031475	myosin V complex	0.042	1.000	CC
GO:0034592	synaptic vesicle lumen	0.042	1.000	CC
GO:0090658	cone matrix sheath	0.042	1.000	CC
GO:0098898	dense core granule lumen	0.042	1.000	CC
GO:0099013	neuronal dense core vesicle lumen	0.042	1.000	CC
GO:1990674	Golgi cis cisterna membrane	0.042	1.000	CC
GO:0000137	Golgi cis cisterna	0.043	1.000	CC
GO:0000139	Golgi membrane	0.044	1.000	CC
GO:0031228	intrinsic component of Golgi membrane	0.045	1.000	CC
GO:0030662	coated vesicle membrane	0.045	1.000	CC
GO:0032806	carboxy-terminal domain protein kinase c...	0.049	1.000	CC

dlPFC

GO.ID	Term	FET	BH	Category
GO:0006412	translation	3.5E-30	5.48E-26	BP
GO:0043043	peptide biosynthetic process	5.8E-29	4.54E-25	BP
GO:0043604	amide biosynthetic process	1.7E-27	8.87E-24	BP
GO:0006518	peptide metabolic process	3.1E-23	1.21E-19	BP
GO:0016071	mRNA metabolic process	1.3E-22	4.07E-19	BP
GO:0006413	translational initiation	5.1E-21	1.33E-17	BP
GO:0043603	cellular amide metabolic process	3.3E-20	7.38E-17	BP
GO:1901566	organonitrogen compound biosynthetic pro...	3.8E-17	7.44E-14	BP
GO:0022613	ribonucleoprotein complex biogenesis	4.5E-17	7.83E-14	BP
GO:0072599	establishment of protein localization to...	6.9E-17	1.08E-13	BP
GO:0042254	ribosome biogenesis	1.7E-16	2.42E-13	BP
GO:0070972	protein localization to endoplasmic reti...	2.6E-16	3.39E-13	BP
GO:0006402	mRNA catabolic process	4.1E-16	4.94E-13	BP
GO:0045047	protein targeting to ER	5.4E-16	6.04E-13	BP
GO:0070125	mitochondrial translational elongation	9.4E-16	9.81E-13	BP
GO:0019080	viral gene expression	1.5E-15	1.47E-12	BP
GO:0000184	nuclear-transcribed mRNA catabolic proce...	2.7E-15	2.49E-12	BP
GO:0006414	translational elongation	3.1E-15	2.7E-12	BP
GO:0006401	RNA catabolic process	6.9E-15	5.69E-12	BP
GO:0070126	mitochondrial translational termination	1.1E-14	8.54E-12	BP
GO:0019083	viral transcription	1.2E-14	8.54E-12	BP
GO:0032543	mitochondrial translation	1.2E-14	8.54E-12	BP
GO:0043933	protein-containing complex subunit organ...	1.5E-14	1.02E-11	BP
GO:0046907	intracellular transport	2.1E-14	1.32E-11	BP
GO:0006415	translational termination	2.1E-14	1.32E-11	BP
GO:0072594	establishment of protein localization to...	4.3E-14	2.59E-11	BP
GO:0006613	cotranslational protein targeting to mem...	7.5E-14	4.35E-11	BP
GO:0006614	SRP-dependent cotranslational protein ta...	1.3E-13	7.27E-11	BP
GO:0042273	ribosomal large subunit biogenesis	2.1E-13	1.13E-10	BP
GO:0006886	intracellular protein transport	2.6E-13	1.36E-10	BP
GO:0000956	nuclear-transcribed mRNA catabolic proce...	3.1E-13	1.57E-10	BP
GO:0033365	protein localization to organelle	6.6E-13	3.23E-10	BP
GO:0046700	heterocycle catabolic process	2.8E-12	1.29E-09	BP
GO:0140053	mitochondrial gene expression	2.8E-12	1.29E-09	BP
GO:0051649	establishment of localization in cell	2.9E-12	1.3E-09	BP
GO:0006605	protein targeting	6.9E-12	3E-09	BP
GO:0044248	cellular catabolic process	1.1E-11	4.65E-09	BP
GO:0033108	mitochondrial respiratory chain complex ...	1.2E-11	4.94E-09	BP
GO:0044270	cellular nitrogen compound catabolic pro...	1.3E-11	5.22E-09	BP
GO:0034655	nucleobase-containing compound catabolic...	1.4E-11	5.48E-09	BP
GO:0002181	cytoplasmic translation	1.7E-11	6.49E-09	BP
GO:0019439	aromatic compound catabolic process	1.9E-11	7.08E-09	BP
GO:0000375	RNA splicing, via transesterification re...	2.3E-11	8.37E-09	BP
GO:0034622	cellular protein-containing complex asse...	3.6E-11	1.28E-08	BP
GO:0044265	cellular macromolecule catabolic process	3.9E-11	1.36E-08	BP
GO:0000377	RNA splicing, via transesterification re...	5E-11	1.67E-08	BP
GO:0000398	mRNA splicing, via spliceosome	5E-11	1.67E-08	BP
GO:0006119	oxidative phosphorylation	6.2E-11	2.02E-08	BP
GO:0008380	RNA splicing	9E-11	2.88E-08	BP
GO:0007005	mitochondrion organization	1E-10	3.13E-08	BP

GO:0034660	ncRNA metabolic process	1.2E-10	3.61E-08	BP
GO:0006457	protein folding	1.2E-10	3.61E-08	BP
GO:0090150	establishment of protein localization to...	1.3E-10	3.84E-08	BP
GO:0006364	rRNA processing	1.4E-10	4.06E-08	BP
GO:0034641	cellular nitrogen compound metabolic pro...	1.9E-10	5.41E-08	BP
GO:1901361	organic cyclic compound catabolic proces...	2E-10	5.59E-08	BP
GO:0009057	macromolecule catabolic process	2.5E-10	6.87E-08	BP
GO:0006612	protein targeting to membrane	2.9E-10	7.83E-08	BP
GO:0006397	mRNA processing	3.7E-10	9.82E-08	BP
GO:0016072	rRNA metabolic process	4.3E-10	1.12E-07	BP
GO:0015833	peptide transport	4.8E-10	1.23E-07	BP
GO:0044267	cellular protein metabolic process	5.3E-10	1.34E-07	BP
GO:0015031	protein transport	6.8E-10	1.69E-07	BP
GO:0065003	protein-containing complex assembly	8.4E-10	2.05E-07	BP
GO:1901564	organonitrogen compound metabolic proces...	1E-09	2.41E-07	BP
GO:0044237	cellular metabolic process	1.3E-09	3.08E-07	BP
GO:0042886	amide transport	1.4E-09	3.27E-07	BP
GO:0009056	catabolic process	1.6E-09	3.68E-07	BP
GO:0051641	cellular localization	1.7E-09	3.86E-07	BP
GO:0045184	establishment of protein localization	1.9E-09	4.25E-07	BP
GO:0016032	viral process	2E-09	4.41E-07	BP
GO:0044403	symbiont process	2.8E-09	6.09E-07	BP
GO:0042773	ATP synthesis coupled electron transport	3.6E-09	7.72E-07	BP
GO:0061418	regulation of transcription from RNA pol...	4.2E-09	8.89E-07	BP
GO:0034613	cellular protein localization	4.4E-09	9.06E-07	BP
GO:0044419	interspecies interaction between organis...	4.4E-09	9.06E-07	BP
GO:0070727	cellular macromolecule localization	4.5E-09	9.15E-07	BP
GO:0010257	NADH dehydrogenase complex assembly	6.9E-09	1.37E-06	BP
GO:0032981	mitochondrial respiratory chain complex ...	6.9E-09	1.37E-06	BP
GO:0042775	mitochondrial ATP synthesis coupled elec...	7.7E-09	1.51E-06	BP
GO:0022900	electron transport chain	9.2E-09	1.78E-06	BP
GO:0022904	respiratory electron transport chain	1.4E-08	2.67E-06	BP
GO:0007007	inner mitochondrial membrane organizatio...	1.5E-08	2.83E-06	BP
GO:0006396	RNA processing	1.7E-08	3.17E-06	BP
GO:0043624	cellular protein complex disassembly	2E-08	3.68E-06	BP
GO:0032984	protein-containing complex disassembly	2.1E-08	3.82E-06	BP
GO:0045333	cellular respiration	2.5E-08	4.5E-06	BP
GO:0006091	generation of precursor metabolites and ...	3E-08	5.34E-06	BP
GO:0044271	cellular nitrogen compound biosynthetic ...	7.1E-08	1.25E-05	BP
GO:0010608	posttranscriptional regulation of gene e...	8.2E-08	1.43E-05	BP
GO:0071826	ribonucleoprotein complex subunit organi...	1E-07	1.72E-05	BP
GO:0034470	ncRNA processing	1.1E-07	1.87E-05	BP
GO:1902036	regulation of hematopoietic stem cell di...	1.5E-07	2.53E-05	BP
GO:0006521	regulation of cellular amino acid metabo...	1.6E-07	2.67E-05	BP
GO:0044085	cellular component biogenesis	2.1E-07	3.46E-05	BP
GO:0071705	nitrogen compound transport	2.5E-07	4.08E-05	BP
GO:0006120	mitochondrial electron transport, NADH t...	2.7E-07	4.36E-05	BP
GO:0022618	ribonucleoprotein complex assembly	3E-07	4.79E-05	BP
GO:0008104	protein localization	4.3E-07	6.8E-05	BP
GO:0055086	nucleobase-containing small molecule met...	5E-07	7.83E-05	BP
GO:0006753	nucleoside phosphate metabolic process	6.1E-07	9.46E-05	BP
GO:0009117	nucleotide metabolic process	6.8E-07	0.000104	BP

GO:1901575	organic substance catabolic process	7.1E-07	0.000108	BP
GO:0009123	nucleoside monophosphate metabolic proce...	8.6E-07	0.000129	BP
GO:1901576	organic substance biosynthetic process	9.5E-07	0.000142	BP
GO:0042255	ribosome assembly	1.1E-06	0.000162	BP
GO:0006725	cellular aromatic compound metabolic pro...	1.2E-06	0.000171	BP
GO:0033238	regulation of cellular amine metabolic p...	1.2E-06	0.000171	BP
GO:0019538	protein metabolic process	1.2E-06	0.000171	BP
GO:0009141	nucleoside triphosphate metabolic proces...	1.2E-06	0.000171	BP
GO:1990542	mitochondrial transmembrane transport	1.3E-06	0.000183	BP
GO:0044249	cellular biosynthetic process	1.4E-06	0.000191	BP
GO:0031145	anaphase-promoting complex-dependent cat...	1.4E-06	0.000191	BP
GO:0006807	nitrogen compound metabolic process	1.4E-06	0.000191	BP
GO:0009161	ribonucleoside monophosphate metabolic p...	1.4E-06	0.000191	BP
GO:1901360	organic cyclic compound metabolic proces...	1.5E-06	0.000202	BP
GO:0046483	heterocycle metabolic process	1.6E-06	0.000214	BP
GO:0019693	ribose phosphate metabolic process	2.6E-06	0.000345	BP
GO:0006139	nucleobase-containing compound metabolic...	2.7E-06	0.000355	BP
GO:1901532	regulation of hematopoietic progenitor c...	3.1E-06	0.000404	BP
GO:0022411	cellular component disassembly	3.3E-06	0.000427	BP
GO:0009205	purine ribonucleoside triphosphate metab...	3.4E-06	0.000436	BP
GO:0009144	purine nucleoside triphosphate metabolic...	3.6E-06	0.000458	BP
GO:0009058	biosynthetic process	4.2E-06	0.00053	BP
GO:0009167	purine ribonucleoside monophosphate meta...	4.5E-06	0.000564	BP
GO:0009126	purine nucleoside monophosphate metaboli...	5.4E-06	0.000661	BP
GO:0009199	ribonucleoside triphosphate metabolic pr...	5.4E-06	0.000661	BP
GO:0006403	RNA localization	5.4E-06	0.000661	BP
GO:0060218	hematopoietic stem cell differentiation	5.7E-06	0.000692	BP
GO:0046034	ATP metabolic process	6.4E-06	0.000771	BP
GO:0008152	metabolic process	0.000007	0.000837	BP
GO:0044281	small molecule metabolic process	7.7E-06	0.000913	BP
GO:0015980	energy derivation by oxidation of organi...	9.2E-06	0.001083	BP
GO:0042180	cellular ketone metabolic process	0.00001	0.001168	BP
GO:0000470	maturation of LSU-rRNA	0.000011	0.001276	BP
GO:0031146	SCF-dependent proteasomal ubiquitin-depe...	0.000012	0.001371	BP
GO:0009259	ribonucleotide metabolic process	0.000012	0.001371	BP
GO:1904874	positive regulation of telomerase RNA lo...	0.000014	0.001566	BP
GO:0002479	antigen processing and presentation of e...	0.000014	0.001566	BP
GO:0010972	negative regulation of G2/M transition o...	0.000014	0.001566	BP
GO:0009150	purine ribonucleotide metabolic process	0.000016	0.001777	BP
GO:0071704	organic substance metabolic process	0.000017	0.001848	BP
GO:0048193	Golgi vesicle transport	0.000017	0.001848	BP
GO:0042407	cristae formation	0.000017	0.001848	BP
GO:0044238	primary metabolic process	0.000019	0.00201	BP
GO:0043618	regulation of transcription from RNA pol...	0.000019	0.00201	BP
GO:0044106	cellular amine metabolic process	0.000019	0.00201	BP
GO:0072657	protein localization to membrane	0.000019	0.00201	BP
GO:0051169	nuclear transport	0.00002	0.002088	BP
GO:0071840	cellular component organization or bioge...	0.00002	0.002088	BP
GO:0006913	nucleocytoplasmic transport	0.000021	0.002163	BP
GO:0033554	cellular response to stress	0.000021	0.002163	BP
GO:0033036	macromolecule localization	0.000022	0.002251	BP
GO:0006163	purine nucleotide metabolic process	0.000023	0.002338	BP

GO:2000736	regulation of stem cell differentiation	0.000024	0.002424	BP
GO:0051168	nuclear export	0.000025	0.002493	BP
GO:0072521	purine-containing compound metabolic pro...	0.000025	0.002493	BP
GO:0019882	antigen processing and presentation	0.000027	0.002676	BP
GO:0006611	protein export from nucleus	0.000029	0.002856	BP
GO:0016236	macroautophagy	0.00003	0.002917	BP
GO:0042590	antigen processing and presentation of e...	0.00003	0.002917	BP
GO:0070498	interleukin-1-mediated signaling pathway	0.000031	0.002996	BP
GO:0007006	mitochondrial membrane organization	0.000032	0.003055	BP
GO:0002183	cytoplasmic translational initiation	0.000032	0.003055	BP
GO:0019884	antigen processing and presentation of e...	0.000033	0.003131	BP
GO:0002478	antigen processing and presentation of e...	0.000034	0.003207	BP
GO:0019941	modification-dependent protein catabolic...	0.000035	0.003281	BP
GO:0034645	cellular macromolecule biosynthetic proc...	0.000036	0.003355	BP
GO:0043632	modification-dependent macromolecule cat...	0.000037	0.003428	BP
GO:1904872	regulation of telomerase RNA localizatio...	0.000038	0.0035	BP
GO:0006511	ubiquitin-dependent protein catabolic pr...	0.000039	0.003571	BP
GO:0006903	vesicle targeting	0.00004	0.003641	BP
GO:0043620	regulation of DNA-templated transcriptio...	0.000043	0.003869	BP
GO:0009894	regulation of catabolic process	0.000043	0.003869	BP
GO:0006417	regulation of translation	0.000045	0.004003	BP
GO:0006839	mitochondrial transport	0.000045	0.004003	BP
GO:0006996	organelle organization	0.000049	0.004334	BP
GO:0044257	cellular protein catabolic process	0.000059	0.00519	BP
GO:0010467	gene expression	0.000063	0.005511	BP
GO:0009059	macromolecule biosynthetic process	0.000068	0.00585	BP
GO:0006352	DNA-templated transcription, initiation	0.000068	0.00585	BP
GO:0071702	organic substance transport	0.000068	0.00585	BP
GO:0010565	regulation of cellular ketone metabolic ...	0.000072	0.00616	BP
GO:0048002	antigen processing and presentation of p...	0.000073	0.006178	BP
GO:0071806	protein transmembrane transport	0.000073	0.006178	BP
GO:0010629	negative regulation of gene expression	0.000079	0.00665	BP
GO:0051603	proteolysis involved in cellular protein...	0.000081	0.006782	BP
GO:0035722	interleukin-12-mediated signaling pathwa...	0.000091	0.007382	BP
GO:1903573	negative regulation of response to endop...	0.000091	0.007382	BP
GO:0090670	RNA localization to Cajal body	0.000091	0.007382	BP
GO:0090671	telomerase RNA localization to Cajal bod...	0.000091	0.007382	BP
GO:0090672	telomerase RNA localization	0.000091	0.007382	BP
GO:0090685	RNA localization to nucleus	0.000091	0.007382	BP
GO:0061013	regulation of mRNA catabolic process	0.000093	0.007506	BP
GO:0006914	autophagy	0.000099	0.007908	BP
GO:0061919	process utilizing autophagic mechanism	0.000099	0.007908	BP
GO:0042274	ribosomal small subunit biogenesis	0.00011	0.008569	BP
GO:0034976	response to endoplasmic reticulum stress	0.00011	0.008569	BP
GO:0045454	cell redox homeostasis	0.00011	0.008569	BP
GO:1904869	regulation of protein localization to Ca...	0.00011	0.008569	BP
GO:1904871	positive regulation of protein localizat...	0.00011	0.008569	BP
GO:0031329	regulation of cellular catabolic process	0.00012	0.00921	BP
GO:0006353	DNA-templated transcription, termination	0.00012	0.00921	BP
GO:0043488	regulation of mRNA stability	0.00012	0.00921	BP
GO:0009308	amine metabolic process	0.00015	0.011401	BP
GO:0071166	ribonucleoprotein complex localization	0.00015	0.011401	BP

GO:0043161	proteasome-mediated ubiquitin-dependent ...	0.00016	0.012044	BP
GO:0006283	transcription-coupled nucleotide-excisio...	0.00016	0.012044	BP
GO:0006418	tRNA aminoacylation for protein translat...	0.00017	0.012615	BP
GO:0006289	nucleotide-excision repair	0.00017	0.012615	BP
GO:0002474	antigen processing and presentation of p...	0.00017	0.012615	BP
GO:0006810	transport	0.00018	0.013231	BP
GO:0006405	RNA export from nucleus	0.00018	0.013231	BP
GO:0044260	cellular macromolecule metabolic process	0.00019	0.013901	BP
GO:0006367	transcription initiation from RNA polyme...	0.0002	0.014565	BP
GO:1903311	regulation of mRNA metabolic process	0.00022	0.015947	BP
GO:0071349	cellular response to interleukin-12	0.00023	0.016519	BP
GO:0051234	establishment of localization	0.00023	0.016519	BP
GO:0030163	protein catabolic process	0.00024	0.017158	BP
GO:0071426	ribonucleoprotein complex export from nu...	0.00025	0.017792	BP
GO:0061732	mitochondrial acetyl-CoA biosynthetic pr...	0.00026	0.01842	BP
GO:0006446	regulation of translational initiation	0.00029	0.020361	BP
GO:0031124	mRNA 3'-end processing	0.00029	0.020361	BP
GO:0017004	cytochrome complex assembly	0.0003	0.020969	BP
GO:0044743	protein transmembrane import into intrac...	0.00031	0.021288	BP
GO:0042776	mitochondrial ATP synthesis coupled prot...	0.00031	0.021288	BP
GO:0050657	nucleic acid transport	0.00031	0.021288	BP
GO:0050658	RNA transport	0.00031	0.021288	BP
GO:0070671	response to interleukin-12	0.00035	0.02393	BP
GO:0000463	maturation of LSU-rRNA from tricistronic...	0.00037	0.025078	BP
GO:0006122	mitochondrial electron transport, ubiqui...	0.00037	0.025078	BP
GO:0070646	protein modification by small protein re...	0.00039	0.026207	BP
GO:0043487	regulation of RNA stability	0.00039	0.026207	BP
GO:0006888	ER to Golgi vesicle-mediated transport	0.0004	0.026537	BP
GO:0006085	acetyl-CoA biosynthetic process	0.0004	0.026537	BP
GO:0010499	proteasomal ubiquitin-independent protei...	0.0004	0.026537	BP
GO:1904816	positive regulation of protein localizat...	0.00042	0.027514	BP
GO:1990173	protein localization to nucleoplasm	0.00042	0.027514	BP
GO:0010498	proteasomal protein catabolic process	0.00042	0.027514	BP
GO:0016579	protein deubiquitination	0.00044	0.02835	BP
GO:1903405	protein localization to nuclear body	0.00044	0.02835	BP
GO:1904851	positive regulation of establishment of ...	0.00044	0.02835	BP
GO:1904867	protein localization to Cajal body	0.00044	0.02835	BP
GO:0065002	intracellular protein transmembrane tran...	0.00046	0.029517	BP
GO:1902750	negative regulation of cell cycle G2/M p...	0.0005	0.031953	BP
GO:0000045	autophagosome assembly	0.00052	0.032962	BP
GO:0051236	establishment of RNA localization	0.00052	0.032962	BP
GO:2000679	positive regulation of transcription reg...	0.00053	0.033193	BP
GO:0048199	vesicle targeting, to, from or within Go...	0.00053	0.033193	BP
GO:0009060	aerobic respiration	0.00053	0.033193	BP
GO:0009892	negative regulation of metabolic process	0.00055	0.034308	BP
GO:0043039	tRNA aminoacylation	0.00056	0.034793	BP
GO:0042769	DNA damage response, detection of DNA da...	0.00057	0.035275	BP
GO:0022607	cellular component assembly	0.00059	0.036369	BP
GO:1901135	carbohydrate derivative metabolic proces...	0.00061	0.037454	BP
GO:0010605	negative regulation of macromolecule met...	0.00073	0.044647	BP
GO:0000027	ribosomal large subunit assembly	0.00074	0.045082	BP
GO:0006369	termination of RNA polymerase II transcr...	0.00077	0.046728	BP

GO:0043038	amino acid activation	0.0008	0.048361	BP
GO:0055114	oxidation-reduction process	0.00087	0.052391	BP
GO:0018279	protein N-linked glycosylation via aspar...	0.0009	0.05399	BP
GO:2000677	regulation of transcription regulatory r...	0.00095	0.056772	BP
GO:0098781	ncRNA transcription	0.00097	0.057746	BP
GO:0015985	energy coupled proton transport, down el...	0.001	0.059083	BP
GO:0015986	ATP synthesis coupled proton transport	0.001	0.059083	BP
GO:0042276	error-prone translesion synthesis	0.00101	0.05945	BP
GO:1902255	positive regulation of intrinsic apoptot...	0.00102	0.059813	BP
GO:1901293	nucleoside phosphate biosynthetic proces...	0.00103	0.060174	BP
GO:0042026	protein refolding	0.00105	0.060888	BP
GO:2000142	regulation of DNA-templated transcriptio...	0.00105	0.060888	BP
GO:0009165	nucleotide biosynthetic process	0.00109	0.062743	BP
GO:1905037	autophagosome organization	0.00109	0.062743	BP
GO:1901991	negative regulation of mitotic cell cycl...	0.00115	0.065954	BP
GO:0006406	mRNA export from nucleus	0.00117	0.066613	BP
GO:0071427	mRNA-containing ribonucleoprotein comple...	0.00117	0.066613	BP
GO:0070585	protein localization to mitochondrion	0.00119	0.067507	BP
GO:0038095	Fc-epsilon receptor signaling pathway	0.00121	0.068147	BP
GO:0048194	Golgi vesicle budding	0.00121	0.068147	BP
GO:0034620	cellular response to unfolded protein	0.00125	0.069897	BP
GO:0072655	establishment of protein localization to...	0.00125	0.069897	BP
GO:0070203	regulation of establishment of protein l...	0.00126	0.070206	BP
GO:0035567	non-canonical Wnt signaling pathway	0.00131	0.072733	BP
GO:0035967	cellular response to topologically incor...	0.00135	0.074426	BP
GO:0000715	nucleotide-excision repair, DNA damage r...	0.00135	0.074426	BP
GO:0036294	cellular response to decreased oxygen le...	0.00137	0.075263	BP
GO:0071456	cellular response to hypoxia	0.00138	0.075548	BP
GO:0097499	protein localization to non-motile ciliu...	0.00142	0.077467	BP
GO:0018196	peptidyl-asparagine modification	0.00143	0.077741	BP
GO:0007052	mitotic spindle organization	0.00156	0.084515	BP
GO:0034248	regulation of cellular amide metabolic p...	0.00158	0.085304	BP
GO:0042795	snRNA transcription by RNA polymerase II	0.00164	0.088239	BP
GO:0051123	RNA polymerase II preinitiation complex ...	0.00166	0.089009	BP
GO:0006626	protein targeting to mitochondrion	0.00167	0.08924	BP
GO:0071453	cellular response to oxygen levels	0.00172	0.091599	BP
GO:0001732	formation of cytoplasmic translation ini...	0.00176	0.093096	BP
GO:0045324	late endosome to vacuole transport	0.00176	0.093096	BP
GO:0015931	nucleobase-containing compound transport	0.00187	0.098581	BP
GO:0009301	snRNA transcription	0.00213	0.111911	BP
GO:0006901	vesicle coating	0.00216	0.113107	BP
GO:0043486	histone exchange	0.00217	0.113252	BP
GO:0070647	protein modification by small protein co...	0.00218	0.113396	BP
GO:2001242	regulation of intrinsic apoptotic signal...	0.00221	0.114576	BP
GO:0016043	cellular component organization	0.00224	0.115748	BP
GO:0060260	regulation of transcription initiation f...	0.00227	0.11679	BP
GO:0045898	regulation of RNA polymerase II transcri...	0.00229	0.11679	BP
GO:0090151	establishment of protein localization to...	0.00229	0.11679	BP
GO:1904814	regulation of protein localization to ch...	0.00229	0.11679	BP
GO:0016073	snRNA metabolic process	0.00231	0.117428	BP
GO:0006890	retrograde vesicle-mediated transport, G...	0.00239	0.121101	BP
GO:0030150	protein import into mitochondrial matrix	0.00246	0.123846	BP

GO:0033617	mitochondrial respiratory chain complex ...	0.00246	0.123846	BP
GO:0030968	endoplasmic reticulum unfolded protein r...	0.00252	0.12646	BP
GO:0097064	ncRNA export from nucleus	0.00256	0.128057	BP
GO:0016925	protein sumoylation	0.00262	0.130641	BP
GO:1902850	microtubule cytoskeleton organization in...	0.00263	0.130724	BP
GO:0010948	negative regulation of cell cycle proces...	0.00265	0.130887	BP
GO:0019637	organophosphate metabolic process	0.00265	0.130887	BP
GO:0070897	transcription preinitiation complex asse...	0.00269	0.132444	BP
GO:0032446	protein modification by small protein co...	0.00271	0.133011	BP
GO:0051028	mRNA transport	0.00275	0.134552	BP
GO:0043388	positive regulation of DNA binding	0.00286	0.139499	BP
GO:0050821	protein stabilization	0.0029	0.14101	BP
GO:0000387	spliceosomal snRNP assembly	0.00291	0.141058	BP
GO:0070202	regulation of establishment of protein l...	0.00295	0.142556	BP
GO:0043254	regulation of protein complex assembly	0.00315	0.151752	BP
GO:1901565	organonitrogen compound catabolic proces...	0.00324	0.155609	BP
GO:0071985	multivesicular body sorting pathway	0.00327	0.15657	BP
GO:0048863	stem cell differentiation	0.00341	0.162263	BP
GO:0033209	tumor necrosis factor-mediated signaling...	0.00342	0.162263	BP
GO:0002223	stimulatory C-type lectin receptor signa...	0.00342	0.162263	BP
GO:0006084	acetyl-CoA metabolic process	0.00349	0.165084	BP
GO:0017038	protein import	0.00356	0.167888	BP
GO:0019752	carboxylic acid metabolic process	0.00358	0.168325	BP
GO:0031123	RNA 3'-end processing	0.0036	0.168758	BP
GO:0032509	endosome transport via multivesicular bo...	0.00364	0.170124	BP
GO:0036500	ATF6-mediated unfolded protein response	0.00369	0.171947	BP
GO:0003415	chondrocyte hypertrophy	0.00404	0.185496	BP
GO:0015838	amino-acid betaine transport	0.00404	0.185496	BP
GO:0018171	peptidyl-cysteine oxidation	0.00404	0.185496	BP
GO:0045338	farnesyl diphosphate metabolic process	0.00404	0.185496	BP
GO:0051030	snRNA transport	0.00404	0.185496	BP
GO:0071824	protein-DNA complex subunit organization	0.00407	0.186327	BP
GO:0015949	nucleobase-containing small molecule int...	0.0041	0.187154	BP
GO:0002220	innate immune response activating cell s...	0.0042	0.190961	BP
GO:0051656	establishment of organelle localization	0.00422	0.190961	BP
GO:0051170	import into nucleus	0.00422	0.190961	BP
GO:0062012	regulation of small molecule metabolic p...	0.00439	0.196932	BP
GO:0032511	late endosome to vacuole transport via m...	0.00443	0.196932	BP
GO:0032790	ribosome disassembly	0.00443	0.196932	BP
GO:0045039	protein import into mitochondrial inner ...	0.00443	0.196932	BP
GO:0097384	cellular lipid biosynthetic process	0.00443	0.196932	BP
GO:0051101	regulation of DNA binding	0.00444	0.196932	BP
GO:0060071	Wnt signaling pathway, planar cell polar...	0.00444	0.196932	BP
GO:0051186	cofactor metabolic process	0.00447	0.197703	BP
GO:0006101	citrate metabolic process	0.00475	0.209495	BP
GO:0042256	mature ribosome assembly	0.00483	0.212269	BP
GO:0045930	negative regulation of mitotic cell cycl...	0.00484	0.212269	BP
GO:1903047	mitotic cell cycle process	0.00491	0.214575	BP
GO:0043170	macromolecule metabolic process	0.00492	0.214575	BP
GO:0072350	tricarboxylic acid metabolic process	0.00495	0.215284	BP
GO:0008535	respiratory chain complex IV assembly	0.005	0.215661	BP
GO:0071450	cellular response to oxygen radical	0.005	0.215661	BP

GO:0071451	cellular response to superoxide	0.005	0.215661	BP
GO:0050434	positive regulation of viral transcripti...	0.0051	0.21937	BP
GO:0006259	DNA metabolic process	0.00521	0.223305	BP
GO:0030490	maturation of SSU-rRNA	0.00522	0.223305	BP
GO:0090304	nucleic acid metabolic process	0.00532	0.226963	BP
GO:0000086	G2/M transition of mitotic cell cycle	0.00543	0.231026	BP
GO:0000028	ribosomal small subunit assembly	0.00578	0.245251	BP
GO:0006986	response to unfolded protein	0.00608	0.257283	BP
GO:0006294	nucleotide-excision repair, preincision ...	0.00611	0.257855	BP
GO:0036503	ERAD pathway	0.00619	0.260529	BP
GO:0090175	regulation of establishment of planar po...	0.0064	0.268646	BP
GO:1901988	negative regulation of cell cycle phase ...	0.00644	0.269602	BP
GO:0090263	positive regulation of canonical Wnt sig...	0.00649	0.27097	BP
GO:0038061	NIK/NF-kappaB signaling	0.00661	0.275247	BP
GO:0010389	regulation of G2/M transition of mitotic...	0.00686	0.28471	BP
GO:0034508	centromere complex assembly	0.0069	0.28471	BP
GO:1990089	response to nerve growth factor	0.0069	0.28471	BP
GO:0000278	mitotic cell cycle	0.00691	0.28471	BP
GO:1990090	cellular response to nerve growth factor...	0.00697	0.286429	BP
GO:0002244	hematopoietic progenitor cell differenti...	0.007	0.286908	BP
GO:0001736	establishment of planar polarity	0.00728	0.296831	BP
GO:0007164	establishment of tissue polarity	0.00728	0.296831	BP
GO:0035966	response to topologically incorrect prot...	0.00738	0.300126	BP
GO:0006900	vesicle budding from membrane	0.00746	0.302245	BP
GO:0000281	mitotic cytokinesis	0.00749	0.302245	BP
GO:0090114	COPII-coated vesicle budding	0.00749	0.302245	BP
GO:0033866	nucleoside bisphosphate biosynthetic pro...	0.00777	0.311138	BP
GO:0034030	ribonucleoside bisphosphate biosynthetic...	0.00777	0.311138	BP
GO:0034033	purine nucleoside bisphosphate biosynthe...	0.00777	0.311138	BP
GO:0006520	cellular amino acid metabolic process	0.0079	0.314326	BP
GO:0001731	formation of translation preinitiation c...	0.00795	0.314326	BP
GO:0019042	viral latency	0.00795	0.314326	BP
GO:0032700	negative regulation of interleukin-17 pr...	0.00795	0.314326	BP
GO:1900102	negative regulation of endoplasmic retic...	0.00795	0.314326	BP
GO:0006338	chromatin remodeling	0.008	0.314706	BP
GO:0048207	vesicle targeting, rough ER to cis-Golgi	0.00804	0.314706	BP
GO:0048208	COPII vesicle coating	0.00804	0.314706	BP
GO:0048278	vesicle docking	0.00804	0.314706	BP
GO:1905897	regulation of response to endoplasmic re...	0.00819	0.319778	BP
GO:0061640	cytoskeleton-dependent cytokinesis	0.00824	0.32093	BP
GO:0071347	cellular response to interleukin-1	0.00829	0.322076	BP
GO:1902600	proton transmembrane transport	0.00878	0.340268	BP
GO:1903008	organelle disassembly	0.00893	0.345227	BP
GO:0070555	response to interleukin-1	0.00898	0.346305	BP
GO:0006099	tricarboxylic acid cycle	0.00913	0.350364	BP
GO:0032212	positive regulation of telomere maintena...	0.00913	0.350364	BP
GO:0007080	mitotic metaphase plate congression	0.00938	0.357711	BP
GO:0031055	chromatin remodeling at centromere	0.00938	0.357711	BP
GO:0033683	nucleotide-excision repair, DNA incision	0.00939	0.357711	BP
GO:0051291	protein heterooligomerization	0.00955	0.362923	BP
GO:0043687	post-translational protein modification	0.00979	0.371143	BP
GO:0007033	vacuole organization	0.01007	0.380836	BP

GO:0002084	protein depalmitoylation	0.01045	0.393307	BP
GO:0022417	protein maturation by protein folding	0.01045	0.393307	BP
GO:0002758	innate immune response-activating signal...	0.01054	0.395743	BP
GO:0000717	nucleotide-excision repair, DNA duplex u...	0.01065	0.398916	BP
GO:0030178	negative regulation of Wnt signaling pat...	0.01071	0.400206	BP
GO:0065004	protein-DNA complex assembly	0.01079	0.402236	BP
GO:0019081	viral translation	0.01088	0.403669	BP
GO:0046037	GMP metabolic process	0.01088	0.403669	BP
GO:0016999	antibiotic metabolic process	0.0112	0.414559	BP
GO:0051704	multi-organism process	0.01132	0.418012	BP
GO:0000303	response to superoxide	0.0115	0.42069	BP
GO:0008334	histone mRNA metabolic process	0.0115	0.42069	BP
GO:0044766	multi-organism transport	0.0115	0.42069	BP
GO:1902579	multi-organism localization	0.0115	0.42069	BP
GO:0035384	thioester biosynthetic process	0.01157	0.421283	BP
GO:0071616	acyl-CoA biosynthetic process	0.01157	0.421283	BP
GO:0090090	negative regulation of canonical Wnt sig...	0.01169	0.424664	BP
GO:0045786	negative regulation of cell cycle	0.01195	0.433105	BP
GO:0044839	cell cycle G2/M phase transition	0.01235	0.446568	BP
GO:0046390	ribose phosphate biosynthetic process	0.0125	0.449427	BP
GO:0038093	Fc receptor signaling pathway	0.01254	0.449427	BP
GO:0044088	regulation of vacuole organization	0.01256	0.449427	BP
GO:0006409	tRNA export from nucleus	0.0126	0.449427	BP
GO:0071431	tRNA-containing ribonucleoprotein comple...	0.0126	0.449427	BP
GO:0006296	nucleotide-excision repair, DNA incision...	0.01263	0.449427	BP
GO:1901998	toxin transport	0.01263	0.449427	BP
GO:0006399	tRNA metabolic process	0.01278	0.453733	BP
GO:0000723	telomere maintenance	0.01293	0.45802	BP
GO:0036498	IRE1-mediated unfolded protein response	0.01309	0.461599	BP
GO:1902653	secondary alcohol biosynthetic process	0.01309	0.461599	BP
GO:0002218	activation of innate immune response	0.0132	0.464168	BP
GO:0009445	putrescine metabolic process	0.01339	0.464168	BP
GO:0019673	GDP-mannose metabolic process	0.01339	0.464168	BP
GO:0099022	vesicle tethering	0.01339	0.464168	BP
GO:0150093	amyloid-beta clearance by transcytosis	0.01339	0.464168	BP
GO:1903608	protein localization to cytoplasmic stre...	0.01339	0.464168	BP
GO:2000665	regulation of interleukin-13 secretion	0.01339	0.464168	BP
GO:0030433	ubiquitin-dependent ERAD pathway	0.0134	0.464168	BP
GO:0010517	regulation of phospholipase activity	0.01363	0.471093	BP
GO:0016050	vesicle organization	0.0137	0.472469	BP
GO:0016573	histone acetylation	0.01453	0.499992	BP
GO:0043436	oxoacid metabolic process	0.01463	0.501364	BP
GO:0090307	mitotic spindle assembly	0.0147	0.501364	BP
GO:0001956	positive regulation of neurotransmitter ...	0.01473	0.501364	BP
GO:0046823	negative regulation of nucleocytoplasmic...	0.01473	0.501364	BP
GO:0070987	error-free translesion synthesis	0.01473	0.501364	BP
GO:1904293	negative regulation of ERAD pathway	0.01496	0.504712	BP
GO:0010518	positive regulation of phospholipase act...	0.01521	0.504712	BP
GO:0006297	nucleotide-excision repair, DNA gap fill...	0.01583	0.504712	BP
GO:2000144	positive regulation of DNA-templated tra...	0.01583	0.504712	BP
GO:0009408	response to heat	0.01595	0.504712	BP
GO:0051650	establishment of vesicle localization	0.01597	0.504712	BP

GO:0002188	translation reinitiation	0.01604	0.504712	BP
GO:0003025	regulation of systemic arterial blood pr...	0.01604	0.504712	BP
GO:0006404	RNA import into nucleus	0.01604	0.504712	BP
GO:0006990	positive regulation of transcription fro...	0.01604	0.504712	BP
GO:0009149	pyrimidine nucleoside triphosphate catab...	0.01604	0.504712	BP
GO:0009213	pyrimidine deoxyribonucleoside triphosph...	0.01604	0.504712	BP
GO:0009447	putrescine catabolic process	0.01604	0.504712	BP
GO:0015879	carnitine transport	0.01604	0.504712	BP
GO:0016476	regulation of embryonic cell shape	0.01604	0.504712	BP
GO:0045337	farnesyl diphosphate biosynthetic proces...	0.01604	0.504712	BP
GO:0048743	positive regulation of skeletal muscle f...	0.01604	0.504712	BP
GO:0051598	meiotic recombination checkpoint	0.01604	0.504712	BP
GO:0060423	foregut regionalization	0.01604	0.504712	BP
GO:0060424	lung field specification	0.01604	0.504712	BP
GO:0060492	lung induction	0.01604	0.504712	BP
GO:0070084	protein initiator methionine removal	0.01604	0.504712	BP
GO:0097051	establishment of protein localization to...	0.01604	0.504712	BP
GO:1902626	assembly of large subunit precursor of p...	0.01604	0.504712	BP
GO:1903294	regulation of glutamate secretion, neuro...	0.01604	0.504712	BP
GO:1903296	positive regulation of glutamate secreti...	0.01604	0.504712	BP
GO:1903445	protein transport from ciliary membrane ...	0.01604	0.504712	BP
GO:1903895	negative regulation of IRE1-mediated unf...	0.01604	0.504712	BP
GO:1990442	intrinsic apoptotic signaling pathway in...	0.01604	0.504712	BP
GO:2000435	negative regulation of protein neddylati...	0.01604	0.504712	BP
GO:2000664	positive regulation of interleukin-5 sec...	0.01604	0.504712	BP
GO:0008215	spermine metabolic process	0.01615	0.504712	BP
GO:0009438	methylglyoxal metabolic process	0.01615	0.504712	BP
GO:0033299	secretion of lysosomal enzymes	0.01615	0.504712	BP
GO:0061046	regulation of branching involved in lung...	0.01615	0.504712	BP
GO:0072603	interleukin-5 secretion	0.01615	0.504712	BP
GO:1903546	protein localization to photoreceptor ou...	0.01615	0.504712	BP
GO:1904798	positive regulation of core promoter bin...	0.01615	0.504712	BP
GO:1990564	protein polyufmylation	0.01615	0.504712	BP
GO:1990592	protein K69-linked ufmylation	0.01615	0.504712	BP
GO:2000662	regulation of interleukin-5 secretion	0.01615	0.504712	BP
GO:0072522	purine-containing compound biosynthetic ...	0.01634	0.509632	BP
GO:0000305	response to oxygen radical	0.01653	0.513512	BP
GO:0070911	global genome nucleotide-excision repair	0.01653	0.513512	BP
GO:0036258	multivesicular body assembly	0.01692	0.524263	BP
GO:0010506	regulation of autophagy	0.01698	0.524263	BP
GO:0000291	nuclear-transcribed mRNA catabolic proce...	0.01701	0.524263	BP
GO:2000785	regulation of autophagosome assembly	0.01701	0.524263	BP
GO:0017144	drug metabolic process	0.01753	0.539228	BP
GO:0031503	protein-containing complex localization	0.01779	0.546153	BP
GO:0032200	telomere organization	0.01793	0.549374	BP
GO:0034605	cellular response to heat	0.01801	0.549654	BP
GO:0006082	organic acid metabolic process	0.01811	0.549654	BP
GO:0006086	acetyl-CoA biosynthetic process from pyr...	0.01822	0.549654	BP
GO:0015936	coenzyme A metabolic process	0.01822	0.549654	BP
GO:0034975	protein folding in endoplasmic reticulum	0.01822	0.549654	BP
GO:0035067	negative regulation of histone acetylati...	0.01822	0.549654	BP
GO:0045116	protein neddylation	0.01822	0.549654	BP

GO:0070200	establishment of protein localization to...	0.01822	0.549654	BP
GO:0061077	chaperone-mediated protein folding	0.01846	0.555658	BP
GO:0006164	purine nucleotide biosynthetic process	0.01849	0.555658	BP
GO:0051648	vesicle localization	0.01855	0.556393	BP
GO:0016197	endosomal transport	0.01859	0.556527	BP
GO:0009260	ribonucleotide biosynthetic process	0.01951	0.582954	BP
GO:0031647	regulation of protein stability	0.01983	0.591156	BP
GO:0043967	histone H4 acetylation	0.01986	0.591156	BP
GO:0032206	positive regulation of telomere maintena...	0.01997	0.592606	BP
GO:1902749	regulation of cell cycle G2/M phase tran...	0.02	0.592606	BP
GO:0016070	RNA metabolic process	0.02013	0.592606	BP
GO:0009200	deoxyribonucleoside triphosphate metabol...	0.02044	0.592606	BP
GO:0035493	SNARE complex assembly	0.02044	0.592606	BP
GO:0045947	negative regulation of translational ini...	0.02044	0.592606	BP
GO:0006487	protein N-linked glycosylation	0.02058	0.592606	BP
GO:0035589	G protein-coupled purinergic nucleotide ...	0.02059	0.592606	BP
GO:0035646	endosome to melanosome transport	0.02059	0.592606	BP
GO:0043485	endosome to pigment granule transport	0.02059	0.592606	BP
GO:0045899	positive regulation of RNA polymerase II...	0.02059	0.592606	BP
GO:0048757	pigment granule maturation	0.02059	0.592606	BP
GO:0075522	IRES-dependent viral translational initi...	0.02059	0.592606	BP
GO:0098734	macromolecule depalmitoylation	0.02059	0.592606	BP
GO:1901838	positive regulation of transcription of ...	0.02059	0.592606	BP
GO:1903025	regulation of RNA polymerase II regulato...	0.02059	0.592606	BP
GO:1904896	ESCRT complex disassembly	0.02059	0.592606	BP
GO:1904903	ESCRT III complex disassembly	0.02059	0.592606	BP
GO:0050909	sensory perception of taste	0.02069	0.594391	BP
GO:0006695	cholesterol biosynthetic process	0.02086	0.598178	BP
GO:0009124	nucleoside monophosphate biosynthetic pr...	0.02131	0.609965	BP
GO:0019430	removal of superoxide radicals	0.02187	0.623713	BP
GO:1901798	positive regulation of signal transducti...	0.02187	0.623713	BP
GO:0061462	protein localization to lysosome	0.02198	0.625711	BP
GO:0009152	purine ribonucleotide biosynthetic proce...	0.0221	0.627985	BP
GO:0045740	positive regulation of DNA replication	0.0225	0.635889	BP
GO:0051031	tRNA transport	0.0225	0.635889	BP
GO:1904358	positive regulation of telomere maintena...	0.0225	0.635889	BP
GO:0007339	binding of sperm to zona pellucida	0.02269	0.640103	BP
GO:0006370	7-methylguanosine mRNA capping	0.02289	0.642554	BP
GO:0043928	exonucleolytic nuclear-transcribed mRNA ...	0.02289	0.642554	BP
GO:0006278	RNA-dependent DNA biosynthetic process	0.0229	0.642554	BP
GO:0030970	retrograde protein transport, ER to cyto...	0.02306	0.6447	BP
GO:1903513	endoplasmic reticulum to cytosol transpo...	0.02306	0.6447	BP
GO:0036257	multivesicular body organization	0.0231	0.6447	BP
GO:0032210	regulation of telomere maintenance via t...	0.024	0.668626	BP
GO:0016241	regulation of macroautophagy	0.0242	0.672085	BP
GO:0097193	intrinsic apoptotic signaling pathway	0.02421	0.672085	BP
GO:2001243	negative regulation of intrinsic apoptot...	0.02459	0.681426	BP
GO:0051188	cofactor biosynthetic process	0.02485	0.686478	BP
GO:0051640	organelle localization	0.02486	0.686478	BP
GO:0018205	peptidyl-lysine modification	0.02521	0.694917	BP
GO:0006596	polyamine biosynthetic process	0.02544	0.697573	BP
GO:0043248	proteasome assembly	0.02544	0.697573	BP

GO:0072665	protein localization to vacuole	0.02544	0.697573	BP
GO:0007338	single fertilization	0.02656	0.72701	BP
GO:0060193	positive regulation of lipase activity	0.02679	0.732026	BP
GO:0018393	internal peptidyl-lysine acetylation	0.02723	0.740989	BP
GO:0006904	vesicle docking involved in exocytosis	0.02723	0.740989	BP
GO:0046677	response to antibiotic	0.02726	0.740989	BP
GO:0001666	response to hypoxia	0.02742	0.744047	BP
GO:0036293	response to decreased oxygen levels	0.02757	0.746823	BP
GO:1901990	regulation of mitotic cell cycle phase t...	0.02782	0.751483	BP
GO:0051310	metaphase plate congression	0.02819	0.751483	BP
GO:0001976	neurological system process involved in ...	0.02832	0.751483	BP
GO:0007270	neuron-neuron synaptic transmission	0.02832	0.751483	BP
GO:0051342	regulation of cyclic-nucleotide phosphod...	0.02832	0.751483	BP
GO:0072611	interleukin-13 secretion	0.02832	0.751483	BP
GO:0075713	establishment of integrated proviral lat...	0.02832	0.751483	BP
GO:1900364	negative regulation of mRNA polyadenylat...	0.02832	0.751483	BP
GO:1903286	regulation of potassium ion import	0.02832	0.751483	BP
GO:1903715	regulation of aerobic respiration	0.02832	0.751483	BP
GO:1904153	negative regulation of retrograde protei...	0.02832	0.751483	BP
GO:0080135	regulation of cellular response to stres...	0.0285	0.751483	BP
GO:0006071	glycerol metabolic process	0.02851	0.751483	BP
GO:0043968	histone H2A acetylation	0.02851	0.751483	BP
GO:0060261	positive regulation of transcription ini...	0.02851	0.751483	BP
GO:1904706	negative regulation of vascular smooth m...	0.02851	0.751483	BP
GO:0009108	coenzyme biosynthetic process	0.02896	0.760223	BP
GO:0006625	protein targeting to peroxisome	0.0291	0.760223	BP
GO:0007004	telomere maintenance via telomerase	0.0291	0.760223	BP
GO:0072662	protein localization to peroxisome	0.0291	0.760223	BP
GO:0072663	establishment of protein localization to...	0.0291	0.760223	BP
GO:0006732	coenzyme metabolic process	0.02921	0.760223	BP
GO:0010863	positive regulation of phospholipase C a...	0.02923	0.760223	BP
GO:0019068	virion assembly	0.02923	0.760223	BP
GO:0000413	protein peptidyl-prolyl isomerization	0.0301	0.777683	BP
GO:0009262	deoxyribonucleotide metabolic process	0.0301	0.777683	BP
GO:0009452	7-methylguanosine RNA capping	0.0301	0.777683	BP
GO:0036260	RNA capping	0.0301	0.777683	BP
GO:0048524	positive regulation of viral process	0.03075	0.789513	BP
GO:0006360	transcription by RNA polymerase I	0.03075	0.789513	BP
GO:0030049	muscle filament sliding	0.03081	0.789513	BP
GO:0033275	actin-myosin filament sliding	0.03081	0.789513	BP
GO:0071392	cellular response to estradiol stimulus	0.03081	0.789513	BP
GO:0007346	regulation of mitotic cell cycle	0.03098	0.792572	BP
GO:0035666	TRIF-dependent toll-like receptor signal...	0.0313	0.796852	BP
GO:0050686	negative regulation of mRNA processing	0.0313	0.796852	BP
GO:1902253	regulation of intrinsic apoptotic signal...	0.0313	0.796852	BP
GO:0034453	microtubule anchoring	0.03148	0.798837	BP
GO:0070129	regulation of mitochondrial translation	0.03148	0.798837	BP
GO:0045727	positive regulation of translation	0.03185	0.806918	BP
GO:0016567	protein ubiquitination	0.03217	0.813709	BP
GO:0006475	internal protein amino acid acetylation	0.03231	0.815932	BP
GO:0043044	ATP-dependent chromatin remodeling	0.03252	0.819912	BP
GO:0006275	regulation of DNA replication	0.03333	0.838984	BP

GO:0030177	positive regulation of Wnt signaling pat...	0.03379	0.849197	BP
GO:0120163	negative regulation of cold-induced ther...	0.03422	0.858626	BP
GO:0032204	regulation of telomere maintenance	0.03451	0.864517	BP
GO:0016126	sterol biosynthetic process	0.03479	0.868751	BP
GO:0043574	peroxisomal transport	0.03479	0.868751	BP
GO:0035542	regulation of SNARE complex assembly	0.03575	0.884262	BP
GO:0060315	negative regulation of ryanodine-sensiti...	0.03575	0.884262	BP
GO:0071285	cellular response to lithium ion	0.03575	0.884262	BP
GO:0072321	chaperone-mediated protein transport	0.03575	0.884262	BP
GO:1902224	ketone body metabolic process	0.03575	0.884262	BP
GO:1903441	protein localization to ciliary membrane	0.03575	0.884262	BP
GO:1990748	cellular detoxification	0.03687	0.910526	BP
GO:0000819	sister chromatid segregation	0.03714	0.91575	BP
GO:0034080	CENP-A containing nucleosome assembly	0.03733	0.917544	BP
GO:0061641	CENP-A containing chromatin organization	0.03733	0.917544	BP
GO:0060964	regulation of gene silencing by miRNA	0.03764	0.923714	BP
GO:0006368	transcription elongation from RNA polyme...	0.03807	0.925121	BP
GO:0051276	chromosome organization	0.03814	0.925121	BP
GO:0002645	positive regulation of tolerance inducti...	0.03877	0.925121	BP
GO:0008611	ether lipid biosynthetic process	0.03877	0.925121	BP
GO:0016576	histone dephosphorylation	0.03877	0.925121	BP
GO:0030311	poly-N-acetyllactosamine biosynthetic pr...	0.03877	0.925121	BP
GO:0030578	PML body organization	0.03877	0.925121	BP
GO:0035964	COPI-coated vesicle budding	0.03877	0.925121	BP
GO:0036265	RNA (guanine-N7)-methylation	0.03877	0.925121	BP
GO:0046487	glyoxylate metabolic process	0.03877	0.925121	BP
GO:0046504	glycerol ether biosynthetic process	0.03877	0.925121	BP
GO:0046826	negative regulation of protein export fr...	0.03877	0.925121	BP
GO:0048200	Golgi transport vesicle coating	0.03877	0.925121	BP
GO:0048205	COPI coating of Golgi vesicle	0.03877	0.925121	BP
GO:0060729	intestinal epithelial structure maintena...	0.03877	0.925121	BP
GO:0071569	protein ufmylation	0.03877	0.925121	BP
GO:1901503	ether biosynthetic process	0.03877	0.925121	BP
GO:1904796	regulation of core promoter binding	0.03877	0.925121	BP
GO:0000462	maturation of SSU-rRNA from tricistronic...	0.03882	0.925121	BP
GO:0009566	fertilization	0.0389	0.925619	BP
GO:0006220	pyrimidine nucleotide metabolic process	0.03905	0.927778	BP
GO:0006281	DNA repair	0.03998	0.946042	BP
GO:0006244	pyrimidine nucleotide catabolic process	0.04	0.946042	BP
GO:0032656	regulation of interleukin-13 production	0.04	0.946042	BP
GO:0051726	regulation of cell cycle	0.04009	0.946741	BP
GO:2000404	regulation of T cell migration	0.04022	0.948353	BP
GO:0018208	peptidyl-proline modification	0.04029	0.948353	BP
GO:1901137	carbohydrate derivative biosynthetic pro...	0.04034	0.948353	BP
GO:0022402	cell cycle process	0.04112	0.964546	BP
GO:0032392	DNA geometric change	0.04123	0.964546	BP
GO:0046794	transport of virus	0.04137	0.964546	BP
GO:1904356	regulation of telomere maintenance via t...	0.04137	0.964546	BP
GO:1901987	regulation of cell cycle phase transitio...	0.04144	0.964546	BP
GO:0050913	sensory perception of bitter taste	0.04146	0.964546	BP
GO:1902235	regulation of endoplasmic reticulum stre...	0.04146	0.964546	BP
GO:0006595	polyamine metabolic process	0.0422	0.973086	BP

GO:0032660	regulation of interleukin-17 production	0.0422	0.973086	BP
GO:0043923	positive regulation by host of viral tra...	0.0422	0.973086	BP
GO:0045056	transcytosis	0.0422	0.973086	BP
GO:0070131	positive regulation of mitochondrial tra...	0.0422	0.973086	BP
GO:1900363	regulation of mRNA polyadenylation	0.0422	0.973086	BP
GO:0006378	mRNA polyadenylation	0.04244	0.974264	BP
GO:0006851	mitochondrial calcium ion transmembrane ...	0.04245	0.974264	BP
GO:0031365	N-terminal protein amino acid modificati...	0.04245	0.974264	BP
GO:1903706	regulation of hemopoiesis	0.04285	0.974264	BP
GO:0036473	cell death in response to oxidative stre...	0.043	0.974264	BP
GO:0043921	modulation by host of viral transcriptio...	0.04305	0.974264	BP
GO:0052472	modulation by host of symbiont transcrip...	0.04305	0.974264	BP
GO:0070199	establishment of protein localization to...	0.04305	0.974264	BP
GO:0010226	response to lithium ion	0.04306	0.974264	BP
GO:0031645	negative regulation of neurological syst...	0.04306	0.974264	BP
GO:0032620	interleukin-17 production	0.04306	0.974264	BP
GO:0043984	histone H4-K16 acetylation	0.04306	0.974264	BP
GO:1901984	negative regulation of protein acetylati...	0.04306	0.974264	BP
GO:0075733	intracellular transport of virus	0.04381	0.988828	BP
GO:0017148	negative regulation of translation	0.04383	0.988828	BP
GO:0006974	cellular response to DNA damage stimulus	0.04392	0.989432	BP
GO:0045089	positive regulation of innate immune res...	0.04421	0.994534	BP
GO:0072384	organelle transport along microtubule	0.0443	0.995129	BP
GO:0032527	protein exit from endoplasmic reticulum	0.04465	1	BP
GO:0018394	peptidyl-lysine acetylation	0.0447	1	BP
GO:0000910	cytokinesis	0.04521	1	BP
GO:0043462	regulation of ATPase activity	0.04571	1	BP
GO:0001738	morphogenesis of a polarized epithelium	0.04606	1	BP
GO:0034599	cellular response to oxidative stress	0.04621	1	BP
GO:0090317	negative regulation of intracellular pro...	0.04692	1	BP
GO:1900274	regulation of phospholipase C activity	0.04692	1	BP
GO:0009156	ribonucleoside monophosphate biosyntheti...	0.04728	1	BP
GO:0009206	purine ribonucleoside triphosphate biosy...	0.04738	1	BP
GO:0016192	vesicle-mediated transport	0.04814	1	BP
GO:1901989	positive regulation of cell cycle phase ...	0.04816	1	BP
GO:0006733	oxidoreduction coenzyme metabolic proces...	0.04827	1	BP
GO:0032506	cytokinetic process	0.04921	1	BP
GO:0006892	post-Golgi vesicle-mediated transport	0.04931	1	BP
GO:0060147	regulation of posttranscriptional gene s...	0.04931	1	BP
GO:0060966	regulation of gene silencing by RNA	0.04931	1	BP
GO:0030518	intracellular steroid hormone receptor s...	0.04947	1	BP
GO:0032508	DNA duplex unwinding	0.04954	1	BP
GO:0043666	regulation of phosphoprotein phosphatase...	0.04954	1	BP
GO:0005198	structural molecule activity	1.3E-10	4.5E-07	MF
GO:0051082	unfolded protein binding	2E-10	4.5E-07	MF
GO:0003954	NADH dehydrogenase activity	3.6E-08	3.24E-05	MF
GO:0008137	NADH dehydrogenase (ubiquinone) activity	3.6E-08	3.24E-05	MF
GO:0050136	NADH dehydrogenase (quinone) activity	3.6E-08	3.24E-05	MF
GO:0003676	nucleic acid binding	4.8E-08	3.6E-05	MF
GO:0008135	translation factor activity, RNA binding	2.1E-07	0.000135	MF
GO:0005515	protein binding	4E-07	0.000225	MF
GO:0043021	ribonucleoprotein complex binding	9.6E-07	0.00048	MF

GO:0016655	oxidoreductase activity, acting on NAD(P...	1.2E-06	0.00054	MF
GO:0004298	threonine-type endopeptidase activity	4.6E-06	0.001725	MF
GO:0070003	threonine-type peptidase activity	4.6E-06	0.001725	MF
GO:0003743	translation initiation factor activity	6.3E-06	0.00218	MF
GO:1901363	heterocyclic compound binding	9.8E-06	0.003093	MF
GO:0016651	oxidoreductase activity, acting on NAD(P...	0.000011	0.003093	MF
GO:0019843	rRNA binding	0.000011	0.003093	MF
GO:0009055	electron transfer activity	0.000015	0.00397	MF
GO:0097159	organic cyclic compound binding	0.00003	0.007498	MF
GO:0051537	2 iron, 2 sulfur cluster binding	0.000034	0.008051	MF
GO:0015037	peptide disulfide oxidoreductase activit...	0.00013	0.029244	MF
GO:0047485	protein N-terminus binding	0.00019	0.040705	MF
GO:0048027	mRNA 5'-UTR binding	0.0006	0.1227	MF
GO:0016462	pyrophosphatase activity	0.00088	0.144846	MF
GO:0015036	disulfide oxidoreductase activity	0.00092	0.144846	MF
GO:0045296	cadherin binding	0.00094	0.144846	MF
GO:0004812	aminoacyl-tRNA ligase activity	0.00102	0.144846	MF
GO:0016875	ligase activity, forming carbon-oxygen b...	0.00102	0.144846	MF
GO:0004738	pyruvate dehydrogenase activity	0.00104	0.144846	MF
GO:0015288	porin activity	0.00104	0.144846	MF
GO:0034603	pyruvate dehydrogenase [NAD(P)+] activit...	0.00104	0.144846	MF
GO:0034604	pyruvate dehydrogenase (NAD+) activity	0.00104	0.144846	MF
GO:0016817	hydrolase activity, acting on acid anhyd...	0.00109	0.144846	MF
GO:0016818	hydrolase activity, acting on acid anhyd...	0.00109	0.144846	MF
GO:0001883	purine nucleoside binding	0.00117	0.144846	MF
GO:0032550	purine ribonucleoside binding	0.00123	0.144846	MF
GO:0019001	guanyl nucleotide binding	0.00124	0.144846	MF
GO:0032561	guanyl ribonucleotide binding	0.00124	0.144846	MF
GO:0008134	transcription factor binding	0.00126	0.144846	MF
GO:0031386	protein tag	0.00131	0.144846	MF
GO:0032549	ribonucleoside binding	0.00131	0.144846	MF
GO:0000049	tRNA binding	0.00132	0.144846	MF
GO:0008121	ubiquinol-cytochrome-c reductase activit...	0.00146	0.149285	MF
GO:0016681	oxidoreductase activity, acting on diphe...	0.00146	0.149285	MF
GO:0016725	oxidoreductase activity, acting on CH or...	0.00146	0.149285	MF
GO:0005525	GTP binding	0.00171	0.168223	MF
GO:0001882	nucleoside binding	0.00172	0.168223	MF
GO:0046933	proton-transporting ATP synthase activit...	0.00183	0.175174	MF
GO:0044183	protein binding involved in protein fold...	0.00191	0.179023	MF
GO:0017111	nucleoside-triphosphatase activity	0.00198	0.181796	MF
GO:0005048	signal sequence binding	0.00212	0.190758	MF
GO:0043022	ribosome binding	0.00235	0.205051	MF
GO:0004860	protein kinase inhibitor activity	0.00237	0.205051	MF
GO:0140098	catalytic activity, acting on RNA	0.00288	0.244474	MF
GO:0019210	kinase inhibitor activity	0.00307	0.255776	MF
GO:0008139	nuclear localization sequence binding	0.00323	0.264214	MF
GO:0004576	oligosaccharyl transferase activity	0.00381	0.29422	MF
GO:0016679	oxidoreductase activity, acting on diphe...	0.00381	0.29422	MF
GO:0018812	3-hydroxyacyl-CoA dehydratase activity	0.00412	0.29422	MF
GO:0043532	angiostatin binding	0.00412	0.29422	MF
GO:0102158	very-long-chain 3-hydroxyacyl-CoA dehydr...	0.00412	0.29422	MF
GO:0102343	3-hydroxy-arachidoyl-CoA dehydratase act...	0.00412	0.29422	MF

GO:0102344	3-hydroxy-behenoyl-CoA dehydratase activ...	0.00412	0.29422	MF
GO:0102345	3-hydroxy-lignoceroyl-CoA dehydratase ac...	0.00412	0.29422	MF
GO:0016491	oxidoreductase activity	0.00419	0.294544	MF
GO:0008320	protein transmembrane transporter activi...	0.0044	0.304548	MF
GO:0070990	snRNP binding	0.00455	0.310158	MF
GO:0010340	carboxyl-O-methyltransferase activity	0.00494	0.322102	MF
GO:0051998	protein carboxyl O-methyltransferase act...	0.00494	0.322102	MF
GO:0061676	importin-alpha family protein binding	0.00494	0.322102	MF
GO:0022884	macromolecule transmembrane transporter ...	0.00521	0.331405	MF
GO:0003729	mRNA binding	0.00523	0.331405	MF
GO:0016667	oxidoreductase activity, acting on a sul...	0.00547	0.341799	MF
GO:0033038	bitter taste receptor activity	0.006	0.369781	MF
GO:0005523	tropomyosin binding	0.00617	0.375119	MF
GO:0035257	nuclear hormone receptor binding	0.00641	0.384515	MF
GO:0003713	transcription coactivator activity	0.00655	0.387743	MF
GO:0140104	molecular carrier activity	0.0074	0.432371	MF
GO:0003924	GTPase activity	0.0076	0.436403	MF
GO:0035258	steroid hormone receptor binding	0.00771	0.436403	MF
GO:0019887	protein kinase regulator activity	0.00776	0.436403	MF
GO:0140101	catalytic activity, acting on a tRNA	0.00796	0.442124	MF
GO:0140142	nucleocytoplasmic carrier activity	0.00923	0.506412	MF
GO:0016248	channel inhibitor activity	0.00957	0.512565	MF
GO:0044769	ATPase activity, coupled to transmembran...	0.00957	0.512565	MF
GO:0016835	carbon-oxygen lyase activity	0.01036	0.548349	MF
GO:0004579	dolichyl-diphosphooligosaccharide-protei...	0.01072	0.560806	MF
GO:0015078	proton transmembrane transporter activit...	0.01097	0.565443	MF
GO:0008565	protein transporter activity	0.01106	0.565443	MF
GO:0001222	transcription corepressor binding	0.01122	0.567177	MF
GO:0030331	estrogen receptor binding	0.01319	0.659353	MF
GO:0008409	5'-3' exonuclease activity	0.01355	0.668982	MF
GO:0015038	glutathione disulfide oxidoreductase act...	0.01368	0.668982	MF
GO:0016668	oxidoreductase activity, acting on a sul...	0.01537	0.704415	MF
GO:0036402	proteasome-activating ATPase activity	0.01628	0.704415	MF
GO:0098879	structural constituent of postsynaptic s...	0.01628	0.704415	MF
GO:0098919	structural constituent of postsynaptic d...	0.01628	0.704415	MF
GO:0001671	ATPase activator activity	0.01642	0.704415	MF
GO:0008527	taste receptor activity	0.01642	0.704415	MF
GO:0003847	1-alkyl-2-acetylglycerophosphocholine es...	0.01644	0.704415	MF
GO:0004176	ATP-dependent peptidase activity	0.01644	0.704415	MF
GO:0008532	N-acetyllactosaminide beta-1,3-N-acetylg...	0.01644	0.704415	MF
GO:0044388	small protein activating enzyme binding	0.01644	0.704415	MF
GO:0046923	ER retention sequence binding	0.01644	0.704415	MF
GO:0070180	large ribosomal subunit rRNA binding	0.01644	0.704415	MF
GO:1990948	ubiquitin ligase inhibitor activity	0.01644	0.704415	MF
GO:0003712	transcription coregulator activity	0.01752	0.743608	MF
GO:0008200	ion channel inhibitor activity	0.01779	0.748011	MF
GO:0019003	GDP binding	0.01909	0.79524	MF
GO:0051427	hormone receptor binding	0.0209	0.810974	MF
GO:0001608	G protein-coupled nucleotide receptor ac...	0.02108	0.810974	MF
GO:0008474	palmitoyl-(protein) hydrolase activity	0.02108	0.810974	MF
GO:0019869	chloride channel inhibitor activity	0.02108	0.810974	MF
GO:0045028	G protein-coupled purinergic nucleotide ...	0.02108	0.810974	MF

GO:0047429	nucleoside-triphosphate diphosphatase ac...	0.02108	0.810974	MF
GO:0070016	armadillo repeat domain binding	0.02108	0.810974	MF
GO:0098599	palmitoyl hydrolase activity	0.02108	0.810974	MF
GO:0003746	translation elongation factor activity	0.02109	0.810974	MF
GO:0050681	androgen receptor binding	0.02237	0.852904	MF
GO:0008171	O-methyltransferase activity	0.0226	0.854432	MF
GO:0048487	beta-tubulin binding	0.02348	0.880304	MF
GO:0003755	peptidyl-prolyl cis-trans isomerase acti...	0.02384	0.885418	MF
GO:0031369	translation initiation factor binding	0.02401	0.885418	MF
GO:0001846	opsonin binding	0.02611	0.94733	MF
GO:0016251	RNA polymerase II general transcription ...	0.02611	0.94733	MF
GO:0004726	non-membrane spanning protein tyrosine p...	0.0289	1	MF
GO:0008494	translation activator activity	0.0289	1	MF
GO:0015450	P-P-bond-hydrolysis-driven protein trans...	0.0289	1	MF
GO:0001848	complement binding	0.02932	1	MF
GO:0005521	lamin binding	0.02932	1	MF
GO:0061608	nuclear import signal receptor activity	0.03123	1	MF
GO:0035255	ionotropic glutamate receptor binding	0.03199	1	MF
GO:0017025	TBP-class protein binding	0.03222	1	MF
GO:0008097	5S rRNA binding	0.03656	1	MF
GO:0070181	small ribosomal subunit rRNA binding	0.03656	1	MF
GO:0060590	ATPase regulator activity	0.03884	1	MF
GO:0016853	isomerase activity	0.03939	1	MF
GO:0001849	complement component C1q binding	0.03943	1	MF
GO:0019788	NEDD8 transferase activity	0.03943	1	MF
GO:0055105	ubiquitin-protein transferase inhibitor ...	0.03943	1	MF
GO:1990446	U1 snRNP binding	0.03943	1	MF
GO:0016018	cyclosporin A binding	0.041	1	MF
GO:0051536	iron-sulfur cluster binding	0.04103	1	MF
GO:0051540	metal cluster binding	0.04103	1	MF
GO:0004386	helicase activity	0.04109	1	MF
GO:0019207	kinase regulator activity	0.04117	1	MF
GO:0052689	carboxylic ester hydrolase activity	0.04124	1	MF
GO:0005484	SNAP receptor activity	0.04291	1	MF
GO:0004622	lysophospholipase activity	0.04334	1	MF
GO:0005385	zinc ion transmembrane transporter activ...	0.04334	1	MF
GO:0043024	ribosomal small subunit binding	0.04334	1	MF
GO:0051287	NAD binding	0.04588	1	MF
GO:0019903	protein phosphatase binding	0.04968	1	MF
GO:0098798	mitochondrial protein complex	3.3E-27	6.55E-24	CC
GO:0032991	protein-containing complex	4.6E-26	4.57E-23	CC
GO:0044446	intracellular organelle part	3.1E-21	2.05E-18	CC
GO:0044444	cytoplasmic part	8.1E-21	3.31E-18	CC
GO:0005743	mitochondrial inner membrane	8.5E-21	3.31E-18	CC
GO:0005740	mitochondrial envelope	1E-20	3.31E-18	CC
GO:0019866	organelle inner membrane	1.3E-20	3.69E-18	CC
GO:0015934	large ribosomal subunit	2.4E-20	5.96E-18	CC
GO:0044429	mitochondrial part	3.3E-20	7.28E-18	CC
GO:0044422	organelle part	4.2E-20	8.34E-18	CC
GO:0022626	cytosolic ribosome	7.1E-20	1.28E-17	CC
GO:0043231	intracellular membrane-bounded organelle	1.4E-19	2.32E-17	CC
GO:0031966	mitochondrial membrane	2.2E-19	3.26E-17	CC

GO:0005737	cytoplasm	2.3E-19	3.26E-17	CC
GO:0031974	membrane-enclosed lumen	1.4E-18	1.63E-16	CC
GO:0043233	organelle lumen	1.4E-18	1.63E-16	CC
GO:0070013	intracellular organelle lumen	1.4E-18	1.63E-16	CC
GO:0043227	membrane-bounded organelle	5E-18	5.51E-16	CC
GO:0044424	intracellular part	8.8E-18	9.19E-16	CC
GO:0005622	intracellular	1.6E-17	1.47E-15	CC
GO:1902494	catalytic complex	1.6E-17	1.47E-15	CC
GO:0031967	organelle envelope	1.7E-17	1.47E-15	CC
GO:0031975	envelope	1.7E-17	1.47E-15	CC
GO:0044455	mitochondrial membrane part	3.5E-17	2.89E-15	CC
GO:0005739	mitochondrion	1.3E-16	1.03E-14	CC
GO:0098800	inner mitochondrial membrane protein com...	4.6E-16	3.51E-14	CC
GO:0005654	nucleoplasm	4.9E-16	3.6E-14	CC
GO:0044445	cytosolic part	5.3E-16	3.76E-14	CC
GO:0043229	intracellular organelle	9.8E-15	6.71E-13	CC
GO:0015935	small ribosomal subunit	1.3E-14	8.6E-13	CC
GO:0022625	cytosolic large ribosomal subunit	2.1E-14	1.34E-12	CC
GO:0043226	organelle	4.3E-14	2.67E-12	CC
GO:0031981	nuclear lumen	6.1E-14	3.67E-12	CC
GO:0000313	organellar ribosome	8.1E-14	4.59E-12	CC
GO:0005761	mitochondrial ribosome	8.1E-14	4.59E-12	CC
GO:0005829	cytosol	2.5E-13	1.38E-11	CC
GO:0044428	nuclear part	7E-13	3.76E-11	CC
GO:0005681	spliceosomal complex	1.7E-11	8.88E-10	CC
GO:0005759	mitochondrial matrix	1.8E-10	9.16E-09	CC
GO:0005746	mitochondrial respiratory chain	6.6E-10	3.28E-08	CC
GO:0098803	respiratory chain complex	1.1E-09	5.33E-08	CC
GO:0005684	U2-type spliceosomal complex	1.9E-09	8.98E-08	CC
GO:0070469	respiratory chain	2.3E-09	1.06E-07	CC
GO:0005634	nucleus	3E-09	1.35E-07	CC
GO:0022627	cytosolic small ribosomal subunit	8.4E-09	3.71E-07	CC
GO:1990204	oxidoreductase complex	2.1E-08	8.91E-07	CC
GO:0005747	mitochondrial respiratory chain complex ...	2.2E-08	8.91E-07	CC
GO:0030964	NADH dehydrogenase complex	2.2E-08	8.91E-07	CC
GO:0045271	respiratory chain complex I	2.2E-08	8.91E-07	CC
GO:0000315	organellar large ribosomal subunit	3.9E-08	1.52E-06	CC
GO:0005762	mitochondrial large ribosomal subunit	3.9E-08	1.52E-06	CC
GO:0042788	polysomal ribosome	4.1E-08	1.54E-06	CC
GO:1905369	endopeptidase complex	4.1E-08	1.54E-06	CC
GO:1905368	peptidase complex	9E-08	3.28E-06	CC
GO:0005844	polysome	9.1E-08	3.28E-06	CC
GO:0000502	proteasome complex	9.6E-08	3.4E-06	CC
GO:0071013	catalytic step 2 spliceosome	2.2E-07	7.66E-06	CC
GO:0031090	organelle membrane	3E-07	1.03E-05	CC
GO:0000314	organellar small ribosomal subunit	3.8E-07	1.26E-05	CC
GO:0005763	mitochondrial small ribosomal subunit	3.8E-07	1.26E-05	CC
GO:0005689	U12-type spliceosomal complex	4.2E-07	1.37E-05	CC
GO:0071005	U2-type precatalytic spliceosome	8.8E-07	2.82E-05	CC
GO:0070062	extracellular exosome	1.4E-06	4.41E-05	CC
GO:0071011	precatalytic spliceosome	1.5E-06	4.65E-05	CC
GO:1903561	extracellular vesicle	2.1E-06	6.32E-05	CC

GO:0043230	extracellular organelle	2.1E-06	6.32E-05	CC
GO:0043232	intracellular non-membrane-bounded organ...	2.4E-06	7.11E-05	CC
GO:0043228	non-membrane-bounded organelle	2.5E-06	7.3E-05	CC
GO:1990234	transferase complex	3.4E-06	9.78E-05	CC
GO:1904813	ficolin-1-rich granule lumen	0.000011	0.000312	CC
GO:0005753	mitochondrial proton-transporting ATP sy...	0.000012	0.000335	CC
GO:0005686	U2 snRNP	0.000013	0.000358	CC
GO:0030684	preribosome	0.000017	0.000462	CC
GO:0045259	proton-transporting ATP synthase complex	0.000028	0.000751	CC
GO:0005839	proteasome core complex	0.000038	0.001006	CC
GO:0017101	aminoacyl-tRNA synthetase multienzyme co...	0.000041	0.001057	CC
GO:0034709	methylosome	0.000041	0.001057	CC
GO:0071007	U2-type catalytic step 2 spliceosome	0.000058	0.001476	CC
GO:0016272	prefoldin complex	0.000066	0.001658	CC
GO:0031970	organelle envelope lumen	0.000075	0.001861	CC
GO:0005838	proteasome regulatory particle	0.000077	0.001887	CC
GO:0044451	nucleoplasm part	0.0001	0.002421	CC
GO:0034719	SMN-Sm protein complex	0.00011	0.002631	CC
GO:0005832	chaperonin-containing T-complex	0.00012	0.002836	CC
GO:0022624	proteasome accessory complex	0.00013	0.003036	CC
GO:0101002	ficolin-1-rich granule	0.00015	0.003462	CC
GO:0030687	preribosome, large subunit precursor	0.00016	0.003651	CC
GO:0005758	mitochondrial intermembrane space	0.00017	0.003835	CC
GO:0005730	nucleolus	0.00022	0.004852	CC
GO:0044464	cell part	0.00022	0.004852	CC
GO:0034715	pICln-Sm protein complex	0.00026	0.005671	CC
GO:0005623	cell	0.00035	0.007552	CC
GO:0120114	Sm-like protein family complex	0.0004	0.008538	CC
GO:0008540	proteasome regulatory particle, base sub...	0.00043	0.00908	CC
GO:0098796	membrane protein complex	0.00059	0.012328	CC
GO:0046540	U4/U6 x U5 tri-snRNP complex	0.00066	0.013506	CC
GO:0097526	spliceosomal tri-snRNP complex	0.00066	0.013506	CC
GO:0031304	intrinsic component of mitochondrial inn...	0.00072	0.014436	CC
GO:0031305	integral component of mitochondrial inne...	0.00072	0.014436	CC
GO:0016469	proton-transporting two-sector ATPase co...	0.00078	0.015483	CC
GO:0031984	organelle subcompartment	0.00085	0.016705	CC
GO:0033290	eukaryotic 48S preinitiation complex	0.00087	0.016931	CC
GO:0005925	focal adhesion	0.00095	0.018308	CC
GO:0030891	VCB complex	0.00103	0.019472	CC
GO:0101031	chaperone complex	0.00103	0.019472	CC
GO:0005744	TIM23 mitochondrial import inner membran...	0.00107	0.020037	CC
GO:1902554	serine/threonine protein kinase complex	0.00119	0.022076	CC
GO:1902911	protein kinase complex	0.00126	0.023098	CC
GO:0000276	mitochondrial proton-transporting ATP sy...	0.00128	0.023098	CC
GO:0032592	integral component of mitochondrial memb...	0.00128	0.023098	CC
GO:0005924	cell-substrate adherens junction	0.00131	0.023427	CC
GO:0070993	translation preinitiation complex	0.00136	0.024104	CC
GO:0019773	proteasome core complex, alpha-subunit c...	0.00143	0.0249	CC
GO:0000307	cyclin-dependent protein kinase holoenzy...	0.00143	0.0249	CC
GO:0008180	COP9 signalosome	0.00165	0.02848	CC
GO:0098573	intrinsic component of mitochondrial mem...	0.00168	0.028748	CC
GO:0031982	vesicle	0.00172	0.029181	CC

GO:0005852	eukaryotic translation initiation factor...	0.00179	0.030111	CC
GO:0030055	cell-substrate junction	0.00196	0.032694	CC
GO:0016607	nuclear speck	0.00223	0.036888	CC
GO:0000153	cytoplasmic ubiquitin ligase complex	0.00233	0.038224	CC
GO:0030532	small nuclear ribonucleoprotein complex	0.0024	0.039049	CC
GO:0097525	spliceosomal snRNP complex	0.00256	0.041314	CC
GO:0030137	COPI-coated vesicle	0.00272	0.043542	CC
GO:0005687	U4 snRNP	0.00299	0.047481	CC
GO:0042470	melanosome	0.00314	0.049078	CC
GO:0048770	pigment granule	0.00314	0.049078	CC
GO:0016282	eukaryotic 43S preinitiation complex	0.00337	0.052261	CC
GO:0016604	nuclear body	0.00342	0.052626	CC
GO:0005789	endoplasmic reticulum membrane	0.0037	0.056496	CC
GO:0019774	proteasome core complex, beta-subunit co...	0.00374	0.056542	CC
GO:0098827	endoplasmic reticulum subcompartment	0.00376	0.056542	CC
GO:0042175	nuclear outer membrane-endoplasmic retic...	0.0038	0.056714	CC
GO:0030120	vesicle coat	0.00398	0.058687	CC
GO:0005783	endoplasmic reticulum	0.00408	0.058687	CC
GO:0005862	muscle thin filament tropomyosin	0.00408	0.058687	CC
GO:0072487	MSL complex	0.00408	0.058687	CC
GO:0061695	transferase complex, transferring phosph...	0.00408	0.058687	CC
GO:0005682	U5 snRNP	0.0043	0.060968	CC
GO:0030663	COPI-coated vesicle membrane	0.0043	0.060968	CC
GO:0031597	cytosolic proteasome complex	0.00448	0.06307	CC
GO:0000407	phagophore assembly site	0.00456	0.063744	CC
GO:0002199	zona pellucida receptor complex	0.00488	0.066806	CC
GO:0031466	Cul5-RING ubiquitin ligase complex	0.00488	0.066806	CC
GO:0071986	Ragulator complex	0.00488	0.066806	CC
GO:0005865	striated muscle thin filament	0.00509	0.069203	CC
GO:0099738	cell cortex region	0.00522	0.070488	CC
GO:0005847	mRNA cleavage and polyadenylation specif...	0.00587	0.078729	CC
GO:0045263	proton-transporting ATP synthase complex...	0.00605	0.080599	CC
GO:0034708	methyltransferase complex	0.00632	0.083635	CC
GO:0033177	proton-transporting two-sector ATPase co...	0.00698	0.091414	CC
GO:0036464	cytoplasmic ribonucleoprotein granule	0.007	0.091414	CC
GO:0036379	myofilament	0.00786	0.101957	CC
GO:0005741	mitochondrial outer membrane	0.00791	0.101957	CC
GO:0005793	endoplasmic reticulum-Golgi intermediate...	0.00798	0.102195	CC
GO:0031248	protein acetyltransferase complex	0.00852	0.107721	CC
GO:1902493	acetyltransferase complex	0.00852	0.107721	CC
GO:0012505	endomembrane system	0.00948	0.1191	CC
GO:0030117	membrane coat	0.01023	0.126916	CC
GO:0048475	coated membrane	0.01023	0.126916	CC
GO:0034663	endoplasmic reticulum chaperone complex	0.01056	0.129393	CC
GO:0061617	MICOS complex	0.01056	0.129393	CC
GO:0030176	integral component of endoplasmic reticu...	0.01118	0.136149	CC
GO:0030686	90S preribosome	0.01265	0.153111	CC
GO:0005683	U7 snRNP	0.01351	0.159627	CC
GO:0005869	dynactin complex	0.01351	0.159627	CC
GO:0045254	pyruvate dehydrogenase complex	0.01351	0.159627	CC
GO:0071541	eukaryotic translation initiation factor...	0.01351	0.159627	CC
GO:0032806	carboxy-terminal domain protein kinase c...	0.01494	0.171787	CC

GO:0000775	chromosome, centromeric region	0.01497	0.171787	CC
GO:0031968	organelle outer membrane	0.01501	0.171787	CC
GO:0000812	Swr1 complex	0.01513	0.171787	CC
GO:0008250	oligosaccharyltransferase complex	0.01513	0.171787	CC
GO:0019908	nuclear cyclin-dependent protein kinase ...	0.01513	0.171787	CC
GO:0016591	RNA polymerase II, holoenzyme	0.01584	0.171787	CC
GO:0000814	ESCRT II complex	0.01614	0.171787	CC
GO:0016939	kinesin II complex	0.01614	0.171787	CC
GO:0031391	Elg1 RFC-like complex	0.01614	0.171787	CC
GO:0031510	SUMO activating enzyme complex	0.01614	0.171787	CC
GO:0034457	Mpp10 complex	0.01614	0.171787	CC
GO:0097149	centralspindlin complex	0.01614	0.171787	CC
GO:0000275	mitochondrial proton-transporting ATP sy...	0.01627	0.171787	CC
GO:0005787	signal peptidase complex	0.01627	0.171787	CC
GO:0005850	eukaryotic translation initiation factor...	0.01627	0.171787	CC
GO:0042719	mitochondrial intermembrane space protei...	0.01627	0.171787	CC
GO:0045261	proton-transporting ATP synthase complex...	0.01627	0.171787	CC
GO:0097255	R2TP complex	0.01627	0.171787	CC
GO:0098556	cytoplasmic side of rough endoplasmic re...	0.01627	0.171787	CC
GO:0005615	extracellular space	0.01647	0.172979	CC
GO:0044421	extracellular region part	0.01662	0.173246	CC
GO:0035770	ribonucleoprotein granule	0.01667	0.173246	CC
GO:0030126	COPI vesicle coat	0.01844	0.188677	CC
GO:0030479	actin cortical patch	0.01844	0.188677	CC
GO:0061645	endocytic patch	0.01844	0.188677	CC
GO:0019867	outer membrane	0.01881	0.191476	CC
GO:0031227	intrinsic component of endoplasmic retic...	0.02073	0.209944	CC
GO:0055029	nuclear DNA-directed RNA polymerase comp...	0.02094	0.210994	CC
GO:0000776	kinetochore	0.02137	0.21424	CC
GO:1902562	H4 histone acetyltransferase complex	0.02179	0.217353	CC
GO:0005849	mRNA cleavage factor complex	0.02217	0.220037	CC
GO:0000139	Golgi membrane	0.02329	0.229204	CC
GO:0070069	cytochrome complex	0.02342	0.229204	CC
GO:0045171	intercellular bridge	0.02344	0.229204	CC
GO:0000428	DNA-directed RNA polymerase complex	0.02431	0.236546	CC
GO:0098791	Golgi subcompartment	0.02453	0.237522	CC
GO:0005750	mitochondrial respiratory chain complex ...	0.02571	0.245357	CC
GO:0031314	extrinsic component of mitochondrial inn...	0.02571	0.245357	CC
GO:0045275	respiratory chain complex III	0.02571	0.245357	CC
GO:0000123	histone acetyltransferase complex	0.02674	0.253966	CC
GO:0005742	mitochondrial outer membrane translocase...	0.02856	0.266158	CC
GO:0008024	cyclin/CDK positive transcription elonga...	0.02856	0.266158	CC
GO:0031371	ubiquitin conjugating enzyme complex	0.02856	0.266158	CC
GO:0072669	tRNA-splicing ligase complex	0.02856	0.266158	CC
GO:0034045	phagophore assembly site membrane	0.02884	0.266267	CC
GO:0098831	presynaptic active zone cytoplasmic comp...	0.02884	0.266267	CC
GO:0055038	recycling endosome membrane	0.02998	0.275511	CC
GO:0035267	NuA4 histone acetyltransferase complex	0.03069	0.279448	CC
GO:0043189	H4/H2A histone acetyltransferase complex	0.03069	0.279448	CC
GO:0005576	extracellular region	0.03215	0.291344	CC
GO:0030880	RNA polymerase complex	0.03229	0.291344	CC
GO:0005782	peroxisomal matrix	0.03329	0.297661	CC

GO:0031907	microbody lumen	0.03329	0.297661	CC
GO:0098687	chromosomal region	0.03369	0.299886	CC
GO:0005885	Arp2/3 protein complex	0.03608	0.318306	CC
GO:0048188	Set1C/COMPASS complex	0.03608	0.318306	CC
GO:0005635	nuclear envelope	0.03805	0.333865	CC
GO:0034774	secretory granule lumen	0.03818	0.333865	CC
GO:0035748	myelin sheath abaxonal region	0.03905	0.337018	CC
GO:0098837	postsynaptic recycling endosome	0.03905	0.337018	CC
GO:1990130	GATOR1 complex	0.03905	0.337018	CC
GO:0005666	RNA polymerase III complex	0.04267	0.365172	CC
GO:0005643	nuclear pore	0.04268	0.365172	CC
GO:0044432	endoplasmic reticulum part	0.04333	0.365257	CC
GO:0035145	exon-exon junction complex	0.04358	0.365257	CC
GO:0070971	endoplasmic reticulum exit site	0.04358	0.365257	CC
GO:0044322	endoplasmic reticulum quality control co...	0.0436	0.365257	CC
GO:0005798	Golgi-associated vesicle	0.04361	0.365257	CC
GO:0033116	endoplasmic reticulum-Golgi intermediate...	0.04475	0.37323	CC
GO:0031983	vesicle lumen	0.045	0.373745	CC
GO:0005912	adherens junction	0.04569	0.377894	CC

Supplementary Table 9. Descriptive information of discovered modules in MDD group by sex and brain region

Sex	Region	No. module	Min No. genes	Max No. Genes
	aINS	33	77	2441
	OFC	67	50	2357
Male	vmPFC	86	55	2092
MDD	dlPFC	20	122	4395
	NAc	82	62	1619
	vSUB	107	53	3480
	aINS	25	87	4072
	OFC	36	52	7662
Female	vmPFC	78	51	1866
MDD	dlPFC	57	60	2698
	NAc	105	51	1949
	vSub	109	55	2418

Supplemental Table 10_List of modules expressing common symptoms in men and women across six brain region

Module name	Module size	Brain region	Common symptom
mediumpurple3	67	dlPFC	psychomotor agitation/retardation
mediumpurple3	67	dlPFC	low self-esteem
plum1	72	dlPFC	psychomotor agitation/retardation
skyblue2	51	NAc	insomnia/hypersomnia
darkolivegreen	183	NAc	change in appetite/weight
pink	808	NAc	insomnia/hypersomnia
midnightblue	576	NAc	insomnia/hypersomnia
darkgreen	318	NAc	insomnia/hypersomnia
sienna3	177	NAc	insomnia/hypersomnia
maroon	92	NAc	insomnia/hypersomnia
orange	271	NAc	insomnia/hypersomnia
lightcyan1	144	NAc	insomnia/hypersomnia
darkslateblue	112	NAc	insomnia/hypersomnia
black	1519	OFC	insomnia/hypersomnia
orange	183	OFC	difficulty in Conc/indecision
royalblue	275	OFC	insomnia/hypersomnia
saddlebrown	144	OFC	insomnia/hypersomnia
salmon4	52	OFC	insomnia/hypersomnia
lightcyan1	141	vmPFC	insomnia/hypersomnia
lightpink4	68	vmPFC	insomnia/hypersomnia
lightcyan	443	vmPFC	insomnia/hypersomnia
yellow	1615	vmPFC	insomnia/hypersomnia

DEGs Enrichment men	DEGs Enrichment women
No	No
No	No
No	No
No	No
No	No
No	No
No	No
Yes-All-Up	No
No	No
No	No
No	No
Yes-Up	No
No	No
Yes-Down	Yes-All-Down
No	No
Yes-Down	Yes-All-Down
No	Yes-All-Up
No	Yes-All-Down
No	Yes-All-Up
No	No
Yes-Up	Yes-Up
No	Yes-Down

GO Enrichment

cytoskeleton
cytoskeleton
cytoplasm
NS
cilium
mitochondrial matrix
NS
cell periphery
intracellular organelle lumen
dendritic spine
intracellular membrane-bounded organelle
ATP synthesis coupled electron transport
intracellular organelle lumen
meiosis I
synapse
bitter taste receptor activity
kinase activity
COP9 signalosome
synapse
bitter taste receptor activity
cytosolic ribosome
RNA binding

All -> DEGs (upregulated or downregulated)
Up -> DEG (upregulated)
Down -> DEG (Downregulated)
NS -> Not Significant
